# MLL1 and MLL1 fusion proteins have distinct functions in regulating leukemic transcription program

**DOI:** 10.1038/celldisc.2016.8

**Published:** 2016-05-17

**Authors:** Jing Xu, Li Li, Jie Xiong, Aaron denDekker, Andrew Ye, Hacer Karatas, Liu Liu, He Wang, Zhaohui S Qin, Shaomeng Wang, Yali Dou

**Affiliations:** 1 Department of Pathology, University of Michigan, Ann Arbor, MI, USA; 2 Department of Biostatistics and Bioinformatics, Rollins School of Public Health, Emory University, Atlanta, GA, USA; 3 Department of Internal Medicine, University of Michigan, Ann Arbor, MI, USA; 4 Department of Medicinal Chemistry, University of Michigan, Ann Arbor, MI, USA; 5 Department of Pharmacology, University of Michigan, Ann Arbor, MI, USA; 6 China Novartis Institutes for BioMedical Research, Shanghai, China; 7 Department of Biological Chemistry, University of Michigan, Ann Arbor, MI, USA

**Keywords:** acute myeloid leukemia, epigenetic therapeutics, menin, MLL1, MLL fusion proteins

## Abstract

Mixed lineage leukemia protein-1 (MLL1) has a critical role in human MLL1 rearranged leukemia (*MLLr*) and is a validated therapeutic target. However, its role in regulating global gene expression in *MLLr* cells, as well as its interplay with MLL1 fusion proteins remains unclear. Here we show that despite shared DNA-binding and cofactor interacting domains at the N terminus, MLL1 and MLL-AF9 are recruited to distinct chromatin regions and have divergent functions in regulating the leukemic transcription program. We demonstrate that MLL1, probably through C-terminal interaction with WDR5, is recruited to regulatory enhancers that are enriched for binding sites of E-twenty-six (ETS) family transcription factors, whereas MLL-AF9 binds to chromatin regions that have no H3K4me1 enrichment. Transcriptome-wide changes induced by different small molecule inhibitors also highlight the distinct functions of MLL1 and MLL-AF9. Taken together, our studies provide novel insights on how MLL1 and MLL fusion proteins contribute to leukemic gene expression, which have implications for developing effective therapies in the future.

## Introduction

Histone H3 lysine 4 (H3K4) methyltransferase mixed lineage leukemia protein-1 (MLL1, also called MLL, KMT2A, HRX, HTRX and ALL1) is important for epigenetic maintenance of *Hox* gene expression, and is required for normal fetal and adult hematopoiesis [1]. Abnormalities of MLL1 on chromosome 11q23 were originally reported in a group of biphenotypic leukemia, where leukemic blasts express both lymphoid and myeloid surface antigens [2]. Majority of MLL1 abnormalities involve balanced chromosomal translocations that lead to production of over 70 in-frame oncogenic fusion proteins [3]. MLL1 fusion proteins retain the MLL1 N-terminal DNA-binding domains (for example, AT-hook and CxxC) [4–6], as well as the capability to interact with transcription cofactors such as MENIN [7–10] and PAF1C [11, 12]. These interactions have been shown to recruit MLL1 fusion proteins to their target genes. The C terminus of over 90% MLL1 fusion proteins is the transactivation domain from AF9, ENL, ELL, AF10, AF4 or AF6 [3]. Some fusion partner proteins are able to interact with histone H3K79 methyltransferase DOT1L (Dot1-like) [13–15], P-TEFb (positive transcription elongation factor b) [16, 17] or CBX8 (chromobox 8)/TIP60 (Tat-interacting protein 60) [18] to augment expression of *HOXA9* and *MEIS1* for leukemic transformation. Progress in the mechanistic understanding of *MLLr* leukemia has led to significant efforts in the development of targeted therapies in recent years [19–23].

Although MLL1 fusion genes are gain-of-function mutations, recent studies show that wild-type *MLL1* allele is still present in vast majority of *MLLr* leukemia [1]. Genetic deletion of *MLL1* completely blocks *MLLr* leukemia *in vivo* [24]. Targeting the MLL1 complex by small molecule inhibitor MLL1 is also able to inhibit *MLLr* and induce myeloblast differentiation *in vitro* [25]. As wild-type MLL1 and MLL1 fusion proteins share N-terminal DNA-binding domains, it is generally assumed that MLL1 and MLL1 fusion proteins cooperatively regulate a common set of downstream targets [26]. Consistent with this view, direct binding of MLL1 and MLL1 fusion proteins are detected at *Hoxa9* [27]. Recruitment of both proteins has also been described at other MLL1 targets such as *Hoxa7, Hoxa10* and *Meis1* [8, 28]. However, the joint targets of MLL1 and MLL1 fusion proteins has not been extensively characterized in *MLLr* leukemia beyond a handful of genes and it remains unclear how MLL1 and MLL1 fusion proteins contribute to their gene expression.

In this study, we have performed genome-wide analyses on wild-type MLL1 and H3K4me in murine MLL-AF9 leukemia cells. We show that contrary to the prevailing model, wild-type MLL1 binds to chromatin regions distinct from those of MLL1 fusion proteins, despite the shared N-terminal domains. We show that recruitment of wild-type MLL1 is regulated by its interaction with WDR5. Blocking MLL1–WDR5 interaction by small molecule inhibitor MM-401 disrupts MLL1 chromatin association at a significant subset of genes that are important for leukemogenesis. In further support of the MLL1 C-terminal domain in MLL1 recruitment, blocking MENIN interaction with MLL1 and MLL-AF9 has skewed effects on MLL1 fusion protein-mediated transcription. Taken together, our study highlights divergent functions of wild-type MLL1 and MLL1 fusion proteins in *MLLr* leukemia, and provides insights into mechanism-based therapeutic targeting.

## Results

### Wild-type MLL1 protein binds preferentially at gene enhancers in MLL-AF9 leukemia cells

**Figure 1:**
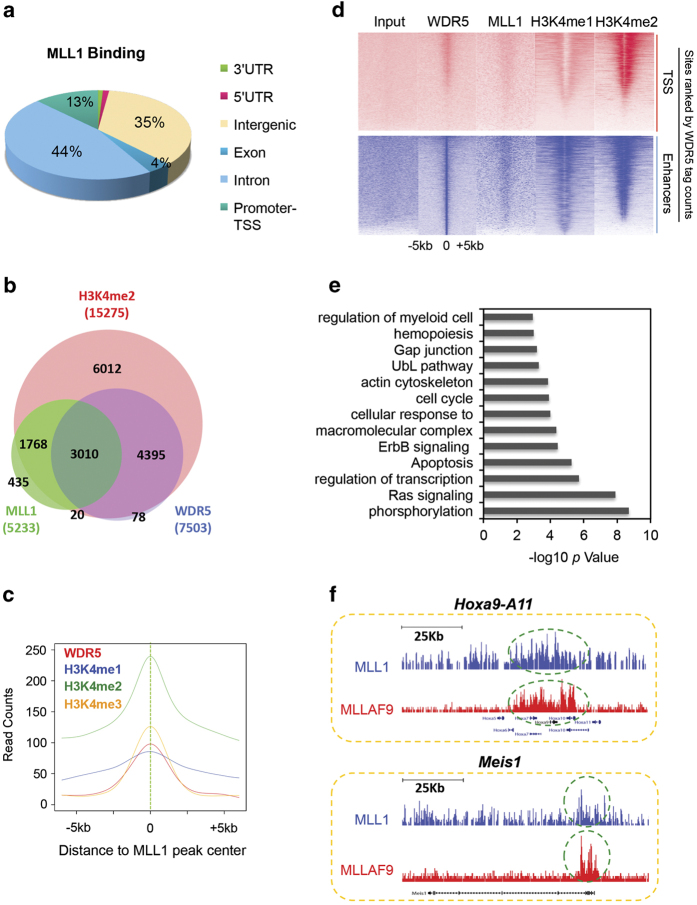
ChIP-seq analyses for the MLL1 complex in the MLL-AF9 cells. (**a**) Genome-wide distribution of MLL1 relative to gene structure. Relative ratio of MLL1 peaks at each defined genomic region versus total peaks was indicated as %. (**b**) Venn diagram of overlap among the annotated targets for MLL1, WDR5 and H3K4me2. (**c**) WDR5, H3K4me1, H3K4me2 and H3K4me3 ChIP-seq meta-profile of MLL1-binding sites. Read counts were normalized to the total number of tags in each sample. (**d**) Heat map representation of ChIP-seq peaks for WDR5, MLL1 and H3K4me2 within ±5 kb of TSS (top) or enhancers (bottom) in MLL-AF9 cells. The rank was ordered from highest to lowest tag counts for WDR5. Red/Blue means enrichment, white means no signal. Total enrichment within ±5 kb of TSS was calculated. (**e**) Gene ontology term analysis of 3 010 direct targets of the MLL1 complex. (**f**) ChIP-seq occupancy profiles of MLL1 (blue) and MLL-AF9 (red) at *Hoxa9-11* and Meis1 loci as indicated on top.

To map the wild-type MLL1 complex in *MLLr* cells, we performed Illumina-based chromatin immunoprecipitation sequencing (ChIP-seq) for MLL1 and WDR5 in primary murine MLL-AF9 cells. The MLL-AF9 cells were derived by transducing bone marrow cells with MLL-AF9 as previously described [21]. Our MLL1 antibody [29] specifically recognized the 180 KDa MLL1 C-terminal fragment and therefore cannot detect the MLL-AF9 protein in leukemia cells (Supplementary Figure S1A). Immunoblot of whole-cell extracts from wild type and *Mll1*^−^^/−^ cells (Supplementary Figure S1A), as well as ChIP experiments at known MLL1-binding sites (Supplementary Figure S1B) confirmed specificity of the MLL1 antibody and its suitability in genome-wide studies. Using this MLL1 antibody in the ChIP-seq experiment, we identified 8 525 MLL1-binding peaks in MLL-AF9 cells using model-based analysis of ChIP-seq (MACS) with significance cutoff of *P*<10^−4^ (Supplementary Table S1). Among them, 13% of MLL1 peaks were located at transcription start sites (TSS) and gene promoters (within 2 kb upstream of TSS), whereas 79% of MLL1 peaks were at intergenic and intronic regions (Figure 1a). In comparison, ChIP-seq for WDR5 identified 13 075 peaks, with 50% of WDR5 binding at intergenic and intronic regions (Supplementary Figure S2A). Consistent with WDR5 as a core component of the MLL family histone methyltransferases [25, 30], ~60% of 5 233 annotated MLL1 targets had WDR5 binding (Figure 1b). In comparison, 40% of WDR5 targets were also bound by MLL1 in MLL-AF9 cells (Figure 1b). WDR5 had much higher promoter occupancy (~37% of total; Supplementary Figure S2A), probably due to its presence in multiple MLL family histone methyltransferases [1].

We next examined the global distribution of mono-, di- and tri-methylated histone H3K4 (H3K4me1, H3K4me2 and H3K4me3) in the MLL-AF9 cells. Significant enrichment of H3K4me1, H3K4me2 or H3K4me3 was found at or near MLL1 peak centers (Figure 1c and Supplementary Figure S2B). Specifically, H3K4me2 was found at majority of the MLL1 direct targets and all MLL1/WDR5 joint targets (3 010; Figure 1b). WDR5, MLL1 and H3K4me1/2 were enriched at promoter (TSS) and enhancer regions (Figure 1d), supporting an important role of the MLL1 complex in transcription regulation. To validate the ChIP-seq results for MLL1 and WDR5, and to establish MLL1-dependent H3K4me in MLL-AF9 cells, we treated the cells with small molecule inhibitor MM-401 that blocks the MLL1–WDR5 interaction [25]. Interestingly, complete or partial loss of H3K4me2 was detected at 10 118 loci, which account for ~60% of total H3K4me2 peaks (Supplementary Figure S2C). Majority of genes that had H3K4me2 change upon MM-401 treatment are MLL1 and WDR5 direct targets (Supplementary Figure S2C), which were defined as targets of the MLL1 complex. Gene ontology analyses showed that they were enriched in gene pathways such as cell signaling, transcription, hypoxia, hematopoiesis and myeloid differentiation (Figure 1e).

### MLL1 and MLL fusion proteins have distinct genome-wide distributions

**Figure 2:**
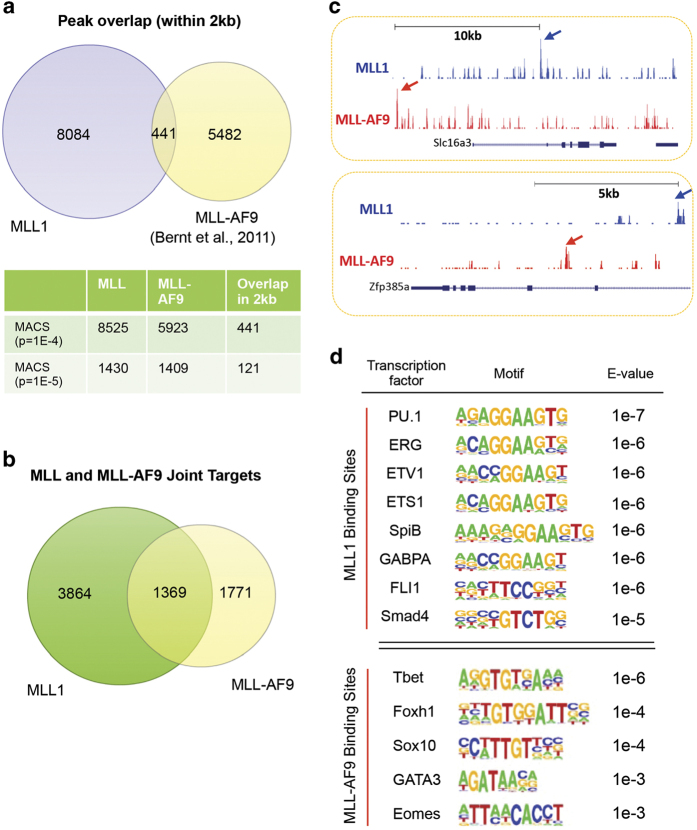
MLL1 and MLL-AF9 bind to distinct chromatin regions. (**a**) Venn diagram of overlap between MLL1 and MLL-AF9 ChIP-seq peaks in the genome. MLL-AF9 ChIP-seq data were previously published (GSE29130) [21]. Bottom, peak numbers for MLL1, MLL-AF9 and their overlaps identified by different significance cutoff using MACS. (**b**) Venn diagram of overlap of the annotated MLL1 and MLL-AF9 direct targets in the MLL-AF9 cells. (**c**) ChIP-seq occupancy profiles of MLL1 (blue) and MLL-AF9 (red) at *Slc16a3* and *Zfp385a* loci as indicated. The arrows indicated the ChIP-seq peaks. (**d**) Motif analyses performed on MLL1 (top) or MLL-AF9 (bottom) occupied sites.

After establishing the direct gene network for the MLL1 complex, we next compared the MLL1-binding sites with previously reported binding sites for the MLL-AF9 fusion protein in the same cells (that is, L-GMPs (Il-7R^−^Lin^−^Sca-1^−^c-Kit^+^CD34^+^FcγRII/III^+^)) [21]. The ChIP-seq results for MLL1 and MLL-AF9 were comparable as shown by similar signal/noise ratio for each data set (Supplementary Figure S1C). To make a direct comparison, we applied the same algorithm and significance cutoff to identify peaks for both MLL1 and MLL-AF9 (see Supplementary Method). Like MLL1, MLL-AF9 preferentially bound at intergenic (46%) and intronic regions (43%; Supplementary Figure S2D). Consistent with previous studies, binding of both MLL1 and MLL-AF9 was identified at 5′ *Hoxa* genes and *Meis1* gene loci (Figure 1f). However, to our surprise, there were minimal overlaps between MLL1 and MLL-AF9 peaks in the genome (Figure 2a). As shown in Figure 2a, we used two different significance cutoffs for peak calling and the results were the same. Specifically, with *P*<10^−5^ as the significance cutoff, a total of 1 409 peaks were identified for MLL-AF9 (Figure 2a), similar to what was previously described [21]. Using the same criterion (that is, *P*<10^−5^), 1 430 MLL1 peaks were identified by MACS (Figure 2a). Only 121 out of 1 430 and 1 409 total peaks for MLL1 and MLL-AF9, respectively, overlapped within a 2 kb region (Figure 2a). Similar results were obtained when we increased the significance cutoff to *P*<10^−4^. In this case, 441 out of 8 525 MLL1 and 5 923 MLL-AF9 peaks overlapped within a 2 kb region (Figure 2a). These results suggested that there was limited physical overlap between MLL1 and MLL-AF9 in MLL-AF9 cells. Consistent with divergent distribution, no significant enrichment of H3K4me1, H3K4me2 or H3K4me3 was found at the MLL-AF9 binding sites (data not shown).

Interestingly, when we compared the annotated targets for MLL1 (and MLL-AF9 (significance cutoff of *P*<10^−4^)), we found that 1 369 genes had both MLL1 and MLL-AF9 binding (for complete list see Supplementary Table S2), which comprise ~26% of total MLL1 (5 233) and 44% of total MLL-AF9 targets (3 140), respectively (Figure 2b). A closer examination showed that with rare exceptions (for example, *Hoxa* and *Meis1*), MLL1 and MLL-AF9 bound to distinct chromatin regions at their joint targets (Figure 2c). Consistent results were also obtained when we used a significance cutoff of *P*<10^−5^ for target identification. In this case, MLL1 and MLL-AF9 shared 418 joint targets, representing 33% of total MLL1 targets (1 504) and 54% of total MLL-AF9 targets (780). The lack of physical overlap between MLL1 and MLL-AF9 peaks (Figure 2a) in the genome, even at their joint targets, suggests that these two proteins are probably recruited by different mechanisms. Indeed, motif analyses showed that MLL1 and MLL-AF9-binding sites were enriched for distinct DNA consensus sequences (Figure 2d). Interestingly, the MLL1-binding sites had modest enrichment for consensus sequences of ETS family transcription factors. Given that aberrant expression of ETS family transcription factors is associated with poor prognosis in acute myeloid leukemia [31–33] and that integrity of some of these factors are important for *MLLr* leukemogenesis [34], it is likely that wild-type MLL1 functions in *MLLr* leukemia, at least in part, by regulating ETS function at active enhancers. In contrast, different consensus sequences were derived from MLL-AF9-binding sites. Moderate enrichment of DNA-binding motifs for embryonic transcription factors (for example, Sox10 and Eomes) and T-cell transcription factors (for example, Tbet and GATA3) were found, consistent with adoption of a more primitive transcription program in *MLLr* leukemia [35, 36].

### Blocking MLL1 or MLL-AF9 has different effects on transcriptome of *MLLr* leukemia cells

**Figure 3:**
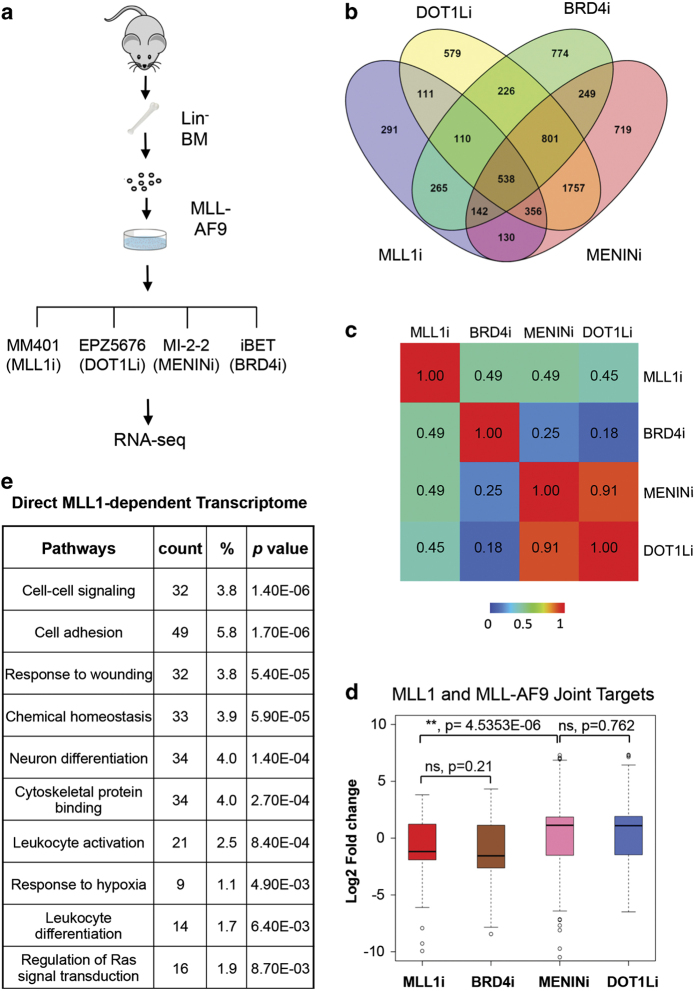
Small molecule inhibitors that target MLL1 or MLL-AF9 show divergent effects on transcription. (**a**) Schematic for RNA-sequencing analyses for primary murine MLL-AF9 cells after inhibitor treatments as indicated. IC_50_ concentration for each inhibitor was used for a 4-day treatment (see Materials and Methods). (**b**) Venn diagram of gene expression changes after different inhibitor treatment as indicated. Genes with RPKM (reads per kilobase per million mapped reads) log_2_ fold change greater than 1 or less than −1 were included. (**c**) Pearson correlation coefficient for pairwise comparison of transcriptome changes after inhibitor treatment. (**d**) The box plots for fold changes in expression after inhibitor treatment. Bottom and top of the boxes correspond to the 25th and 75th percentiles and the internal band is the 50th percentile (median). The plot whiskers extending outside the boxes correspond to the lowest and highest datum within 1.5 interquartile ranges. *P*-values were calculated using non-paired Wilcoxon tests as indicated. Genes with <1 RPKM and abs (log_2_ fold change) <1 were not included in the analyses. NS, not significant. The gene list is shown in Supplementary Table S3. (**e**) Gene pathway analyses for MLL1 direct targets that showed expression changes after MM-401 treatment.

The distinct genome-wide distribution of MLL1 and MLL-AF9 implies that they probably have different functions in regulating transcriptome of MLL-AF9 cells. To study their respective functions on gene expression, we decided to perform Illumina-based RNA-sequencing analyses on MLL-AF9 cells treated with different small molecular inhibitors that target WDR5 (that is, MM-401) [25], DOT1L (that is, EPZ5676) [21, 22], MENIN (that is, MI-2-2) [20] or BRD4 (that is, iBET) [23] (Figure 3a). These inhibitors block *MLLr* leukemia by targeting either transcription cofactors that physically interact with MLL1 (for example, WDR5) or MLL1 fusion proteins (for example, DOT1L and MENIN). The BRD4 inhibitor inhibits *MLLr* leukemia by destabilizing ETS family transcription factors in hematopoietic cells [34] and disrupting pTEFb-dependent gene regulation [37]. As the control, we treated MLL-AF9 cells with 0.01% dimethyl sulfoxide (mock). Respective GI (growth inhibition)_50_ concentration was used for each inhibitor to minimize indirect effects. After 4-day treatment, myeloblast differentiation was obvious for cells that were treated with MM-401 [25], but not other inhibitors (data not shown). As shown in Figure 3b, 1 943 genes showed more than twofold expression changes after MM-401 treatment as compared with mock-treated cells. Similarly, inhibiting BRD4, DOT1L or MENIN also induced significant transcriptome changes (Figure 3b and Supplementary Figure S3A and B). Interestingly, although both MLL1 and BRD4 inhibition led to the downregulation of a majority of genes, MENIN and DOT1L inhibition led to upregulation of most genes in the cells (Supplementary Figure S3A and B). Pairwise comparison of overall transcriptome changes showed that MLL1 inhibition had modest correlation with that of DOT1L, BRD4 or MENIN inhibition with Pearson correlation coefficients (PCC) of ~0.5 in each case (Figure 3c and Supplementary Figure S3B). In contrast, the BRD4 inhibitor had distinct effects on *MLLr* transcriptome from that of the DOT1L inhibitor with PCC of ~0.2. One unexpected result from the experiment is that, despite MENIN interaction with both MLL1 and MLL-AF9, inhibiting MENIN led to almost identical transcriptome changes as the DOT1L inhibitor (PCC=0.91; Figure 3c). This result argues that blocking MENIN has skewed effects on MLL1 fusion proteins, raising questions on the importance of MENIN-MLL1 interaction in genome-wide MLL1 recruitment (see below).

In addition to genome-wide correlation, we examined the transcription outcome of MLL1 and MLL-AF9 joint targets after inhibitor treatments. Inhibiting both MLL1 and BRD4 led to the downregulation of most MLL1 and MLL-AF9 targets with median log2 fold change of −2 (Figure 3d and Supplementary Table S3). In contrast, MENIN and DOT1L inhibition resulted in half of MLL1 and MLL-AF9 joint targets being up- or downregulated with median log2 fold change of +1.5 (Figure 3d and Supplementary Table S3). The difference of MLL1 and DOT1L/MENIN inhibition in gene expression was significant (*P*<4.535E-06, Wilcoxon test, Figure 3d), supporting divergent roles of MLL1 and MLL-AF9 in regulating the leukemic transcription program. Representative genes that were changed by inhibitor treatments are shown in Supplementary Figure S4.

Gene pathway analyses showed that MLL1-regulated genes were enriched for cell–cell signaling, cell adhesion and cell differentiation pathways (Figure 3e). Gene set enrichment analyses showed significant correlation of MLL1 transcriptome with that of *Hoxa9/Meis1* (Supplementary Figure S5A) and *Myc/Mad1* (Supplementary Figure S5B and C), which are established targets for MLL-AF9/DOT1L and BRD4, respectively. This result suggests that MLL1 probably promotes *MLLr* leukemia by targeting both Hoxa9/Meis1-dependent and -independent transcription programs. Enrichment of gene sets such as mTOR antagonist rapamycin and hypoxia were also found (Supplementary Figure S5D and E). Gene ontology analyses showed that DOT1L and MENIN regulated an indistinguishable set of gene pathways (Supplementary Figure S5F), whereas BRD4-dependent pathways were largely different (Supplementary Figure S5G). Interestingly, gene pathways that were upregulated by MENIN/DOT1L inhibitors include a pathway for negative cell proliferation (Supplementary Figure S5H), which may contribute to their potent inhibition of *MLLr* leukemia.

### MLL1 chromatin recruitment depends on its interaction with WDR5

**Figure 4:**
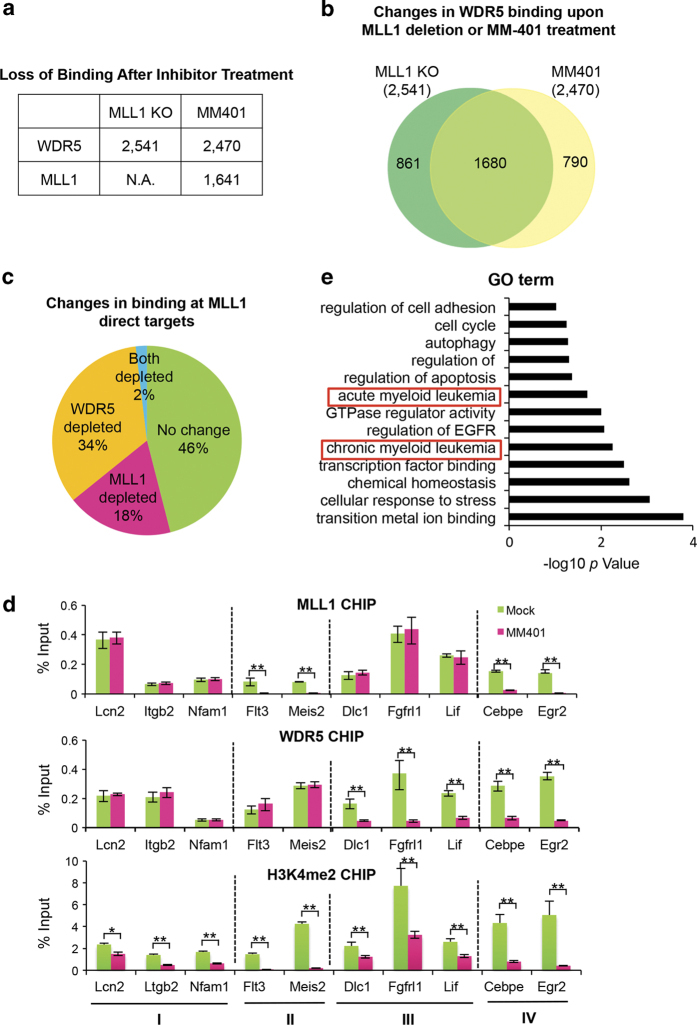
MLL1 interaction with WDR5 is required for MLL1 recruitment at a subset of genes. (**a**) Summary for changes in MLL1 or WDR5 binding after either *Mll1* deletion or MM-401 treatment. (**b**) Venn diagram of overlap of changes in WDR5 binding upon *Mll1* deletion or MM-401 treatment. (**c**) Pie chart of changes in MLL1 or WDR5 binding after MM-401 treatment. Relative ratio of each category versus total peaks was indicated as %. (**d**) ChIP assay for MLL1, WDR5 and H3K4me2 at selected gene loci as indicated on bottom. Signals for each experiment were normalized to 5% input. Means and s.d. (as error bars) from at least three independent experiments were presented. Four groups of genes were selected based on MM-401 induced changes in MLL1 and/or WDR5. (I) No change in MLL1 and WDR5 binding; (II) MLL1 binding is disrupted; (III) WDR5 binding was disrupted; and (IV) both WDR5 and MLL1 binding were disrupted. Student *t*-test were performed for statistical analyses, *P<0.05, **P<0.01. (**e**) Gene ontology term analyses on MLL1 direct targets that have disrupted MLL1 binding after inhibitor treatment.

The distinct genome-wide distribution of MLL1 and MLL-AF9, as well as the skewed effects of MENIN inhibitor on MLL-AF9-dependent transcription suggest that the N-terminal domains of MLL1 probably have a lesser role in targeting the wild-type protein. To test this, we examined genome-wide MLL1-binding after disruption of the MLL1–WDR5 interaction by MM-401. As the control, we had also performed ChIP-seq for WDR5 after MM-401 treatment. As summarized in Figure 4a, MM-401 treatment led to WDR5 dissociation from ~2 500 gene loci. Consistent with this finding, ~70% of these loci had loss of WDR5 binding in *Mll1*^−/−^ cells as well (Figure 4b). MM-401 treatment also led to the loss of MLL1 binding at 1 641 gene loci (Figure 4a), accounting for 32% of total MLL1-binding sites in MLL-AF9 cells. Among the genes that had altered expression as well as reduction of H3K4me upon MM-401 treatment, the MLL1 complex was disrupted at nearly half of these genes with 18 and 34% loss of MLL1 or WDR5 binding, respectively (Figure 4c). Gene ontology term analyses showed that these MLL1 targets were functionally relevant and they were enriched for pathways, such as acute and chronic myeloid leukemia (Figure 4e). ChIP confirmations for MLL1, WDR5 and H3K4me2 at representative genes (for example, Flt3, Meis2, Cebpε and Egr2) are shown in Figure 4d.

To confirm the results in human leukemia cells that carry MLL1 translocation, we performed ChIP experiments for MLL1, WDR5 and H3K4me2, using MOLM13 cells that harbor MLL-AF9 translocation. As shown in Supplementary Figure S6B, dissociation of MLL1 or WDR5 at the same selective MLL1 targets was detected upon MM-401 treatment. Taken together, our results suggest that MLL1 chromatin recruitment partially depends on its C-terminal interaction with WDR5 [30]. It implies that small molecule inhibitor MM-401 inhibits *MLLr* leukemia by two compatible modes of actions: blocking MLL1 methyltransferase activity and disrupting MLL1 complex integrity at a significant subset of MLL1 targets.

## Discussion

Here we show that despite shared DNA-binding as well as cofactor-interacting domains at the N terminus, wild-type MLL1 and MLL-AF9 are recruited to distinct chromatin regions, and have divergent functions in regulating the leukemia transcription program. We further demonstrate that MLL1, partly through its C-terminal interactions with the MLL1 core complex, is mostly recruited to regulatory enhancers that are enriched for H3K4me. Inhibiting MLL1 by small molecule inhibitor MM-401 leads to transcriptome changes that partially overlap with that of MLL fusion proteins in *MLLr* leukemia.

Genome-wide distribution of MLL1 has been previously studied in human leukemia cells. It has been shown that MLL1 is highly enriched at gene promoters and has important roles in transcription initiation [38]. However, MLL1 has also been reported to bind both gene promoters and enhancers in macrophages and functions to define cellular identities together with lineage specific transcription factors [39]. The genome-wide binding of MLL1 by ChIP-seq in murine leukemia cells have not been reported. Our study here shows that MLL1 mostly binds to intergenic and intron regions in murine MLL-AF9 leukemia cells. The MLL1-binding sites are enriched for H3K4me marks as well as consensus sequences of ETS family transcription factors (for example, PU.1 and FLI1 [39, 40]; Figure 1), albeit modestly. Importantly, inhibiting MLL1 by MM-401 led to disruption of the MLL1 complex and reduction of majority of H3K4me2 (60%) in MLL-AF9 cells (Supplementary Figure S2). It is likely that targeting MLL1 blocks *MLLr* leukemogenesis by dysregulating the epigenetic/enhancer landscapes [41], which is distinct from that of MLL1 fusion proteins [21, 42, 43]. Consistent divergent chromosomal localization of MLL1 and another MLL1 fusion protein MLL-AF4 [43] was also observed (Supplementary Figure S6A). We notice that previous studies in human leukemia cells show that both MLL1 and MLL1 fusion gene MLL-AF4 are more enriched at gene promoters [42, 43], instead of intergenic or intronic regions (Figure 1a and Supplementary Figure S2D). The exact reason for this discrepancy is not clear and we cannot completely rule out that they are due to differences in MLL1 antibodies in these studies or the fact that MLL1 fusion genes in human leukemia cells reside at endogenous MLL1 locus, rather than of random integration as the result of retroviral-mediated transduction. Nonetheless, our studies suggest that in the commonly used murine *MLLr* leukemia model, MLL1 and MLL1 fusion proteins are targeted to different regions in the genome despite their shared DNA-binding and cofactor interaction domains at the N terminus.

The distinct distribution of MLL1 and MLL-AF9 in the genome directly challenges the general assumption that MLL1 binding is mainly mediated by its N-terminal DNA-binding domains or by its interaction with transcription cofactors MENIN and LEDGF [26]. Instead, our study suggests a partial reliance of MLL1 C-terminal domains for chromatin association. In support, the MENIN inhibitor MI-2-2 shows a skewed effect on the MLL-AF9-dependent transcriptome in *MLLr* cells (Figure 3). We envision that in addition to MENIN dependent MLL1 recruitment, MLL1 can also be recruited by interacting with transcription (co)-factors such as E2Fs, p53 and c-Myc via WDR5, which in turn, interacts with the histone H3 tail or non-coding RNAs and therefore serves as an anchor to guide the stepwise assembly of the MLL1 complex [1]. In this scenario, disrupting MLL1–WDR5 interaction by MM-401 leads to the disruption of MLL1 chromatin binding, which occurs at 18% of MLL1 targets with transcription changes in MLL-AF9 cells (Figure 4c). Alternatively, the MLL1 SET domain is able to directly interact with transcription factor (for example, RUNX1) [44] or single-strand RNAs [45], which can potentially recruit MLL1 to chromatin. In this scenario, disrupting MLL1–WDR5 interaction by MM-401 will lead to dissociation of WDR5 from the MLL1 targets, which is observed at 34% of MLL1 targets in MLL-AF9 cells that show transcription changes (Figure 4c). In the absence of the MLL1 C-terminal domains, as in the case of MLL1 translocation, MLL1 fusion proteins have to rely more on MENIN/LEDGF interaction for stable chromatin association. Our study is also consistent with a previous report that MENIN is only a sub-stoichiometric component of the MLL1 complex [46]. In light of our finding here, it would be interesting to test whether blocking other MLL1 N-terminal interactions (for example, MLL1 CxxC) also leads to more profound impacts on MLL1 fusion protein function.

The divergent functions of MLL1 and MLL-AF9 in *MLLr* leukemia suggest that small molecule inhibitors targeting these proteins probably inhibit *MLLr* leukemia through distinct mechanisms. Consistent with this view, only modest overlap has been found in the transcriptome-wide changes after MM-401 or MENIN/DOT1L treatment (Figure 3d). One surprising finding of our study is that inhibiting DOT1L and MENIN leads to strikingly similar changes in *MLLr* transcriptome. They go beyond the MLL-AF9 direct targets and result in global activation of majority of non-MLL-AF9 targets (Supplementary Figure S3A). As both MENIN and DOT1L have been reported to have significant roles in non-MLL1-containing protein complexes (for example, KMT2B for MENIN) [1, 3], future delineation of MENIN and DOT1L functions, especially at non-MLL1 targets, is warranted.

## Materials and Methods

### Cell culture conditions

MLL-AF9-transduced mouse bone marrow cells (MAF9), Hoxa9/Meis1-transduced mouse bone marrow cells (HM) and *Mll1*^*Flox+/+*^*;* *ER-Cre*^*+/*−^ mouse bone marrow cell transduced with MLL-AF9 were cultured in Iscove’s modified Dulbecco’s medium with 15% fetal bovine serum and 10 ng ml^−1^ interleukin-3. For obtaining MLL1 knockout cells, *Mll1*^*Flox+/+*^*;* *ER-Cre*^*+/*−^ were treated with 400 nM of 4-Hydroxytamoxifen for 2 days, MLL1 deletion efficiency was determined by genotyping.

### Cell viability assays

MM-401, EPZ5676, MI-2-2 and iBET Inhibitors were diluted by culture media with 0.1% dimethyl sulfoxide. A measure of 2×10^4^ ml^−1^ of MAF9 and HM cells were treated with each inhibitor at different dosage for 4 days. Viability was determined using the CellTiter-Glo Kit (Promega, Madison, WI, USA) according to the manufacturer’s recommendations. Luminescence was monitored on the Molecular Dynamics plate reader (Molecular Devices, Sunnyvale, CA, USA).

### Gene expression analyses

MLL1-AF9 cells were cultured for 4 days in the presence of MM-401, EPZ5676, MI-2-2 and iBET at respective GI_50_ dose. Cells were collected by centrifugation at 300×*g* and washed with 1× phosphate-buffered saline. RNAs from duplicated biological samples were extracted. 1 μg of total RNA was reverse transcribed using Transcriptor First Strand Synthesis kit (Invitrogen, Carlsbad, CA, USA). Real time-PCR was performed on the ABI7300 (Applied Biosystems, Carlsbad, CA, USA) thermo-cycler.

### Chromatin immunoprecipitation experiment

Approximately 1×10^8^ MLL-AF9 cells were treated with 20 μM MM-401 for 2 days. Dimethyl sulfoxide treatment (0.1%) was used as the control. For MLL1 deletion, ~1×10^8^ MLL-AF9 transduced MLL1^*flox/flox*^, ER-Cre^*+/*−^ cells were treated with 400 nM 4-Hydroxytamoxifen for 2 days. In this case, ethanol-treated cells were used as the control. After treatments, cells were cross-linked with 2 mM disuccinimidyl glutarate (Sigma 80424, St Louis, MO, USA) for 30 min at room temperature. Following two washes with 1× phosphate-buffered saline, cells were incubated for 10 min with 1% formaldehyde. Then cells were lysed and the chromatin was sheared for 3× at 20 min. The immunoprecipitation using anti-MLL1, WDR5 or H3K4me2 was performed according to previously published protocol [29].

### ChIP-seq, RNA-sequencing, gene ontology and gene set enrichment analyses

ChIP-seq and RNA-sequencing library preparation and sequencing were performed at University of Michigan DNA Sequencing Facility. For details of data analyses see Supplementary Information.

### Accession numbers

The data have been deposited in NCBI's Gene Expression Omnibus and are accessible through GEO Series accession number GSE68823.

## Notes

Supplementary Table S3

Supplementary Method
