# Supplementary material for: MLL1 and MLL1 fusion proteins have distinct functions in regulating leukemic transcription program
Source: Cell Discov. 2016 May 17;2:16008–. doi: 10.1038/celldisc.2016.8 (PMC4869169; doi:10.1038/celldisc.2016.8)
Supplement: Supplementary Figure S1 [file celldisc20168-s1.pdf]

Supplemental Figure 1

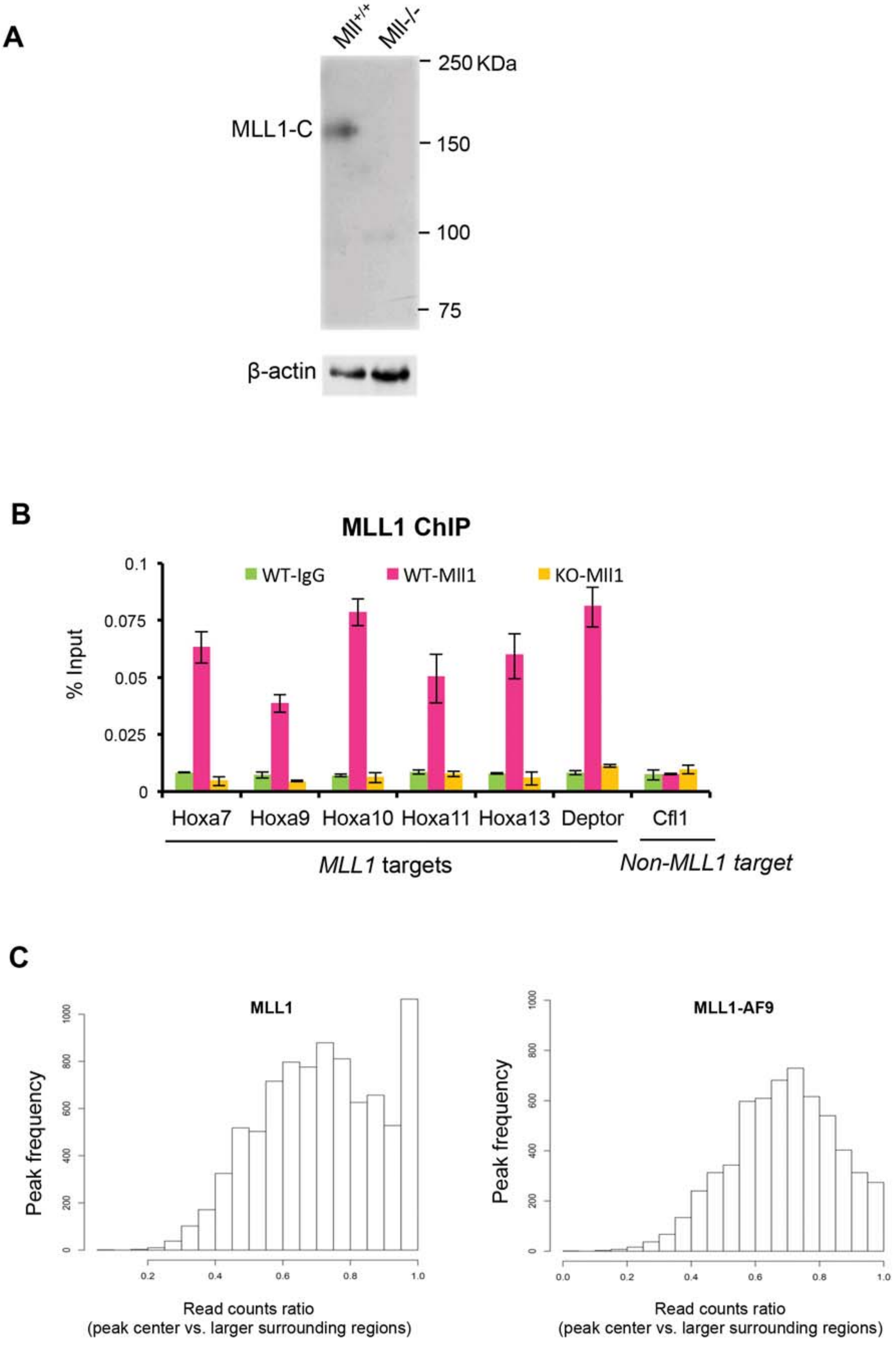

Supplemental Figure 1. The anti-MLL1^{C} antibody is specific and suitable for the ChIP assay. **A.** Immunoblot of whole cell extracts isolated from MLL1 WT and MLL1 knockout cells as indicated on top. Antibodies were indicated on left. **B.** ChIP for MLL1 at selected targets as indicated on bottom. Blue, IgG control; Red, MLL1 ChIP in wild type cells; Green, MLL1 ChIP in knockout cells. ChIP signals were normalized to 5% input. Means and standard deviations (as error bars) from at least three independent experiments were presented. **C.** Signal to noise ratios for the MLL1 or MLL-AF9 ChIP-seq peaks were calculated and presented as read counts ratio. The tag counts within ±200bp of peak center (called by MACS with P-value cutoff 1E-04) were compared to the tag counts within ±500bp of peak center. The ratio reflects signal enrichment over the background, i.e. signal/noise ratio. Left, MLL1 ChIP-Seq data; Right, MLL-AF9 ChIP-seq (GSE29130). Y-axis, peak frequency.
