# Supplementary material for: MLL1 and MLL1 fusion proteins have distinct functions in regulating leukemic transcription program
Source: Cell Discov. 2016 May 17;2:16008–. doi: 10.1038/celldisc.2016.8 (PMC4869169; doi:10.1038/celldisc.2016.8)
Supplement: Supplementary Figure S2 [file celldisc20168-s2.pdf]

Supplemental Figure 2

A

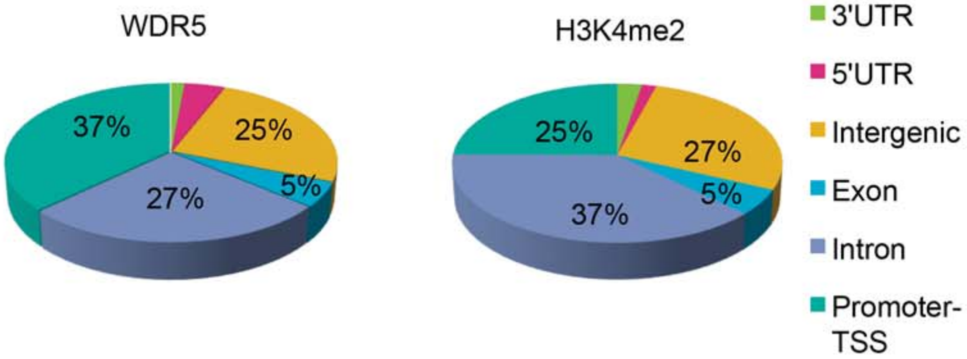

B

	H3K4me1 /MLL1	H3K4me2 /MLL1	H3K4me3 /MLL1
Overlap	2912	3935	2500
P-value	1.82E-36	4.96E-36	1.82E-34

C

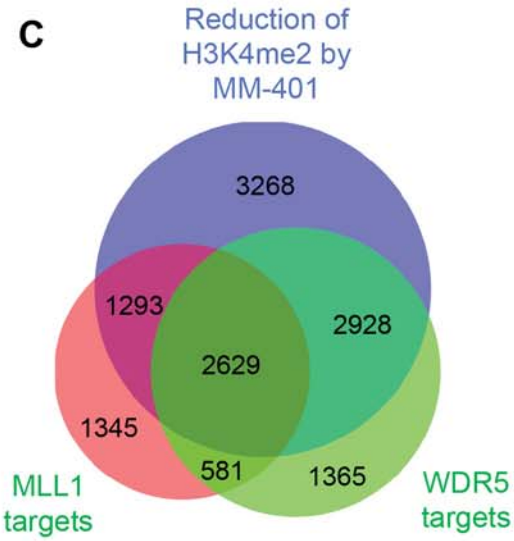

D

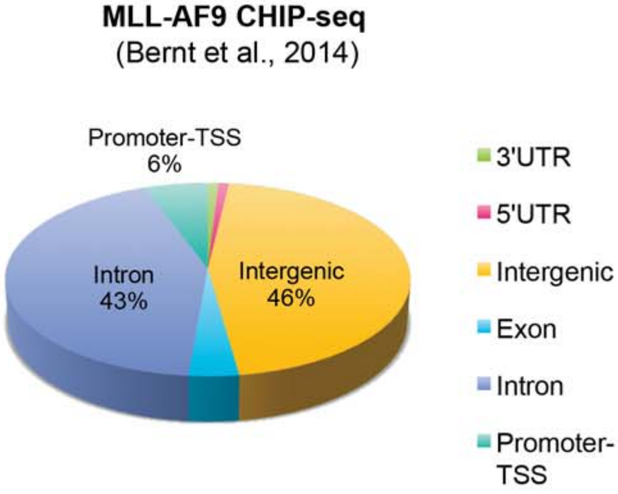

Supplemental Figure 2. ChIP-seq analyses of MLL1 and MLL-AF9 in MLL-AF9 leukemia cells. **A.** Genome-wide distribution of WDR5 and H3K4me2 relative to gene structure. Relative ratio of ChIP-seq peaks at each defined genomic region versus total peaks is indicated as %. **B.** Summary of overlap between MLL1 and H3K4me1 H3K4me2 or H3K4me3 in cells. *t*-test was used for statistic analyses for enrichment. **C.** Venn diagram for overlap among genes that had H3K4me2 alteration after MM-401 treatment and direct binding by MLL1 and/or WDR5. **D.** Genome-wide distribution of MLL-AF9 relative to gene structure. Relative ratio of ChIP-seq peaks at each defined genomic region versus total peaks is indicated as % of total. MLL-AF9 target genes were obtained from previous study GSE29130 [1].
