# Supplementary material for: MLL1 and MLL1 fusion proteins have distinct functions in regulating leukemic transcription program
Source: Cell Discov. 2016 May 17;2:16008–. doi: 10.1038/celldisc.2016.8 (PMC4869169; doi:10.1038/celldisc.2016.8)
Supplement: Supplementary Figure S3 [file celldisc20168-s3.pdf]

Supplemental Figure 3

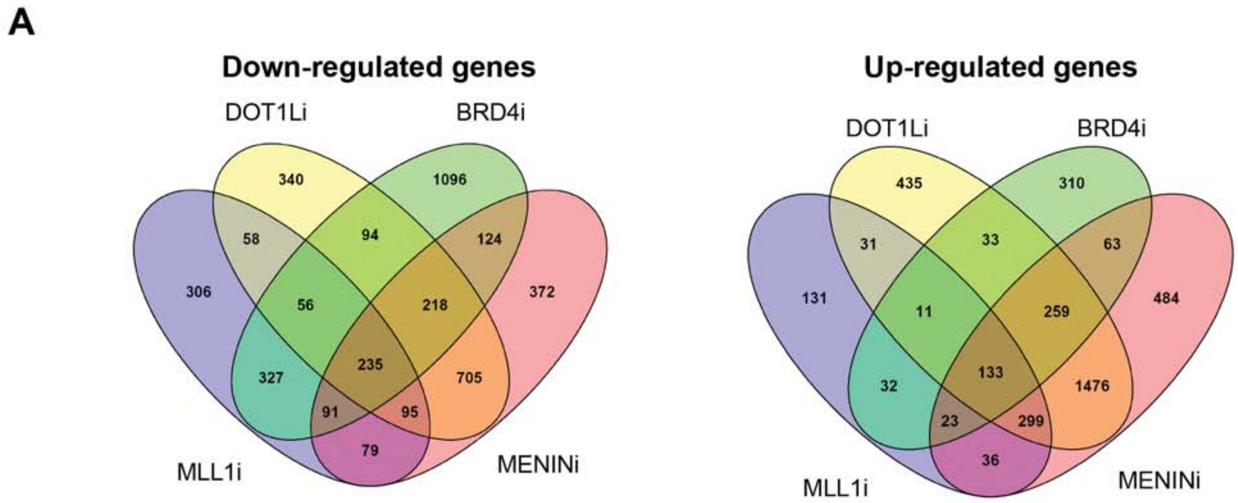

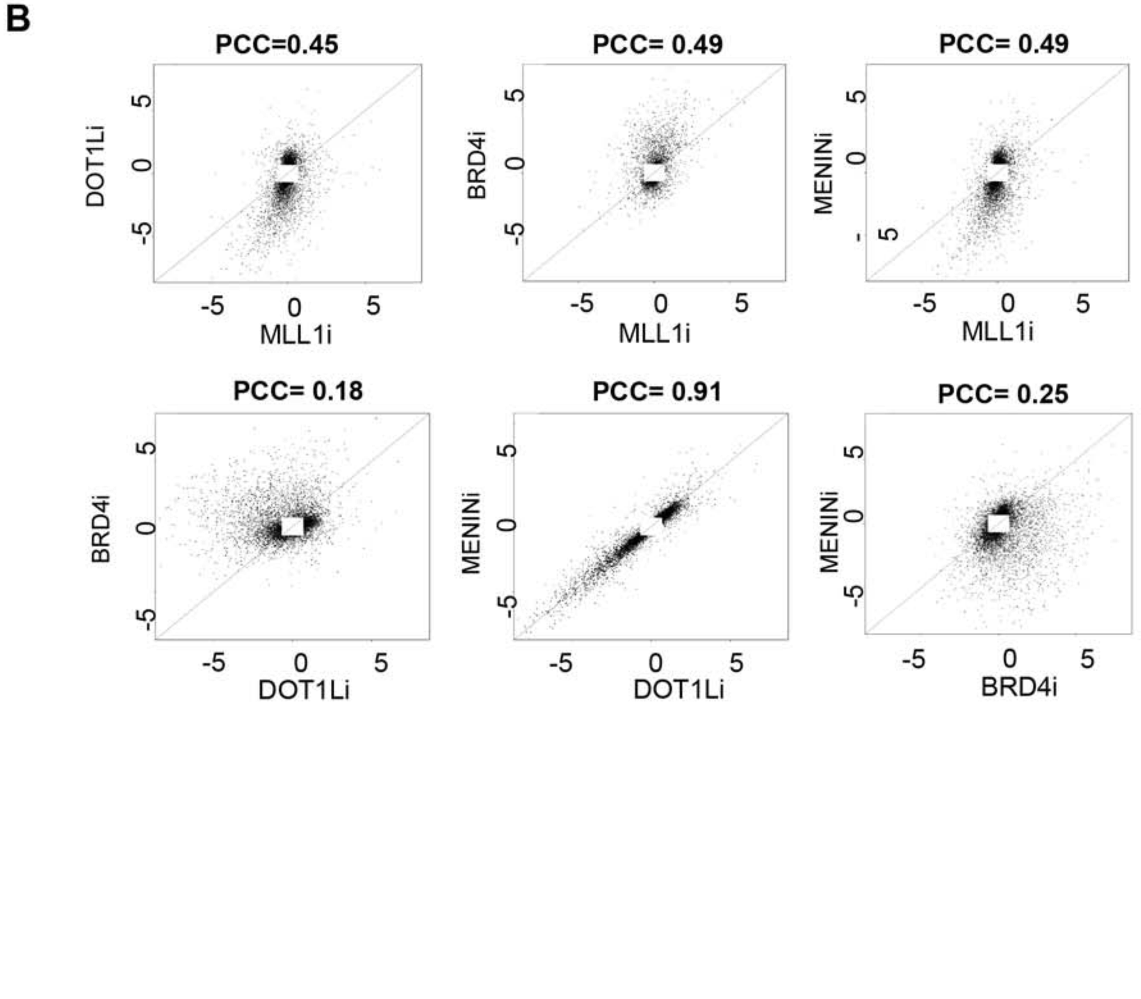

Supplemental Figure 3. Transcriptome analyses for MLL-AF9 cells after small molecule inhibitor treatments. **A.** Venn diagram of down (left) and up (right) regulated genes after inhibitor treatments as indicated. Genes with RPKM Log_2 fold change >1 or <-1 were included. **B.** Scatter plot of pair-wise comparison of gene expression changes after inhibitor treatment. Genes with log2 fold change >1 or < -1 were used.
