# Supplementary material for: MLL1 and MLL1 fusion proteins have distinct functions in regulating leukemic transcription program
Source: Cell Discov. 2016 May 17;2:16008–. doi: 10.1038/celldisc.2016.8 (PMC4869169; doi:10.1038/celldisc.2016.8)
Supplement: Supplementary Figure S4 [file celldisc20168-s4.pdf]

Supplemental Figure 4

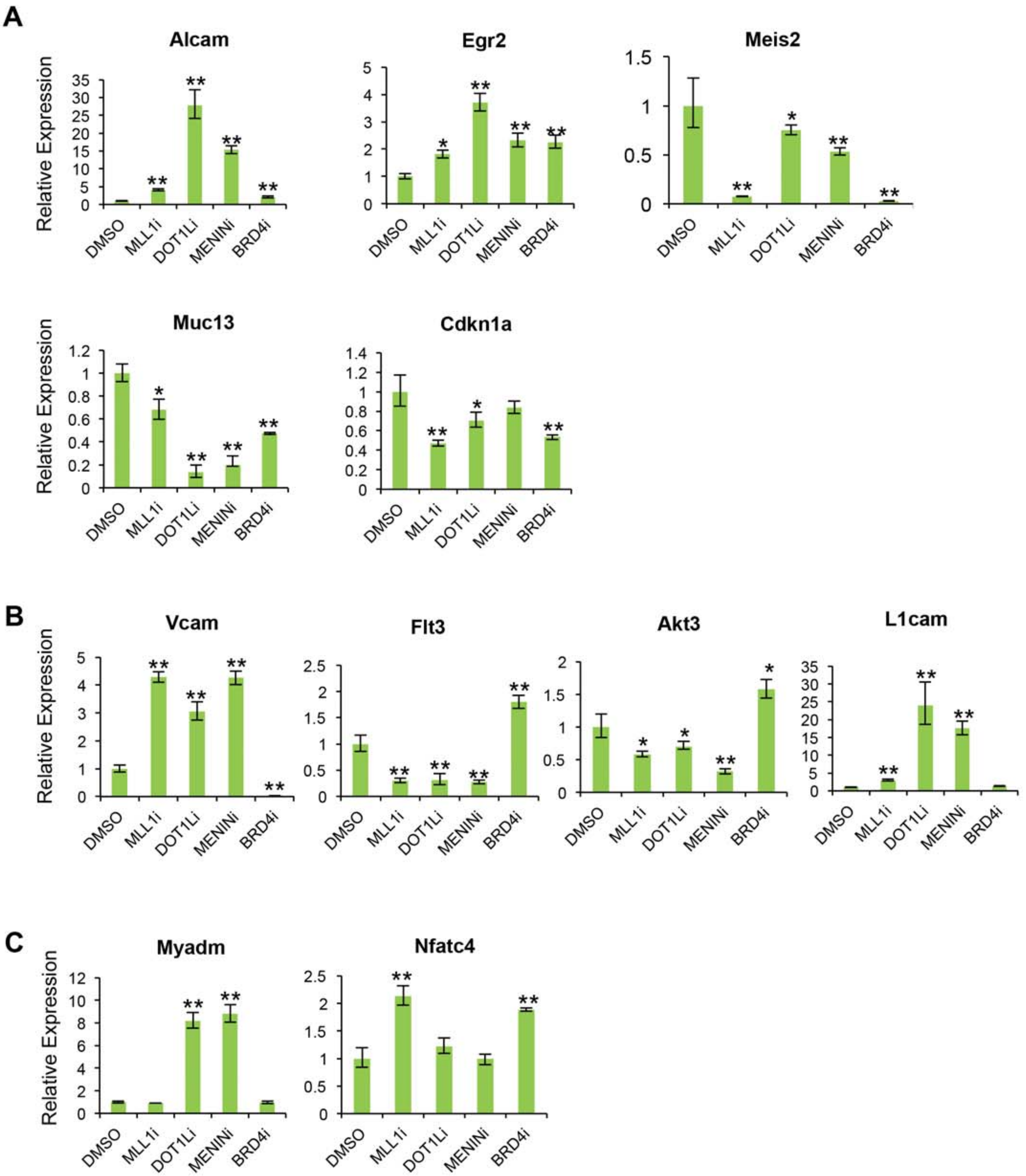

Supplemental Figure 4. PCR confirmation for expression of selected MLL1 target genes. **A-C.** Three groups of genes were selected for real-time PCR confirmation based on similar responses to inhibitors against MLL1, DOT1L, MENIN and BRD4 (A) or against MLL1, DOT1L and MENIN (B) or against DOT1L/MENIN only (C). Gene expression was normalized against *Gapdh* and presented as fold changes. The expression in DMSO control was arbitrarily set as 1. Average results from three independent experiments are shown. Error bars represent standard deviation. Student *t*-test were performed for statistical analyses, * $p<0.05$, ** $p<0.01$.
