# Supplementary material for: MLL1 and MLL1 fusion proteins have distinct functions in regulating leukemic transcription program
Source: Cell Discov. 2016 May 17;2:16008–. doi: 10.1038/celldisc.2016.8 (PMC4869169; doi:10.1038/celldisc.2016.8)
Supplement: Supplementary Figure S5 [file celldisc20168-s5.pdf]

Supplemental Figure 5

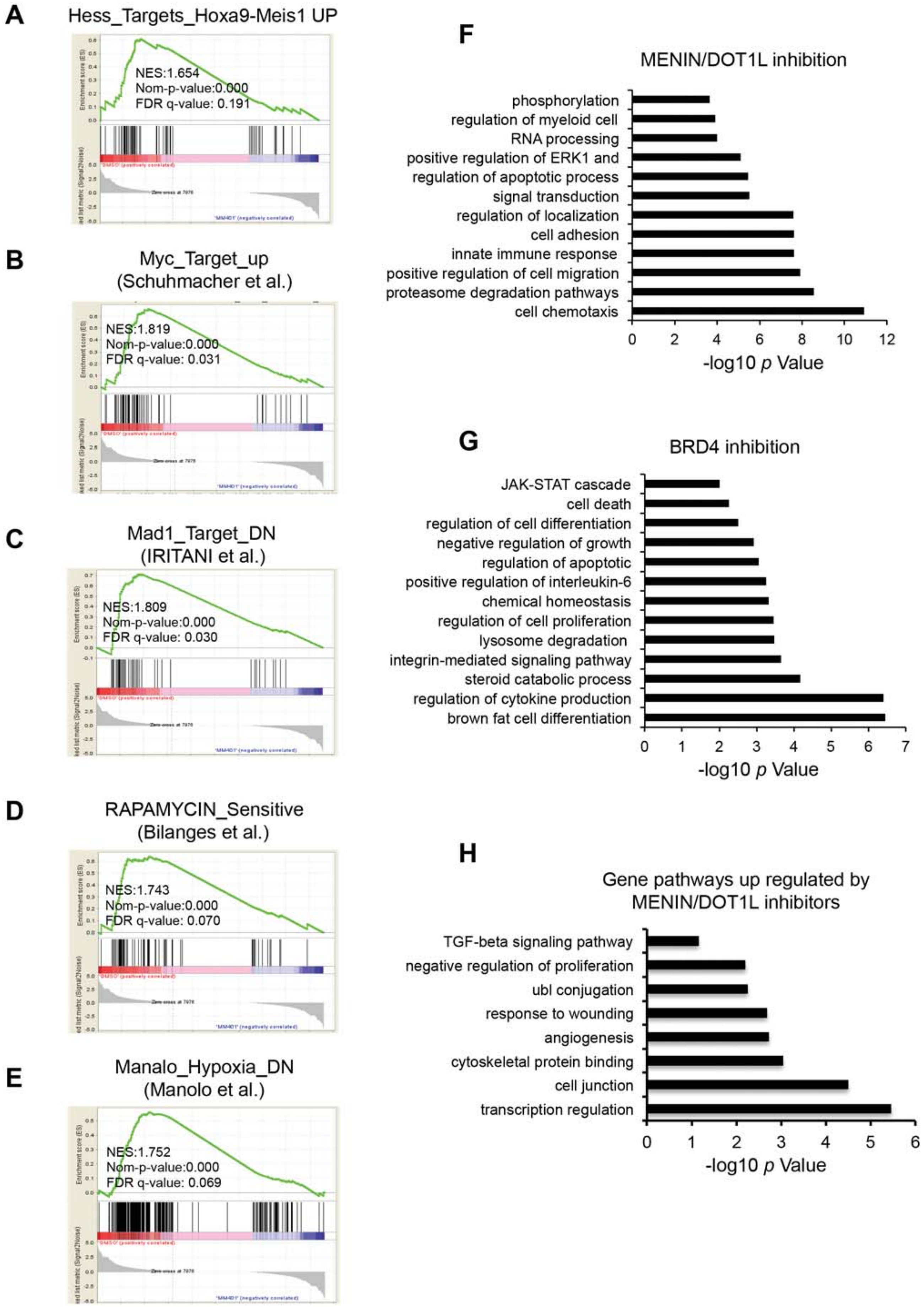

Supplemental Figure 5. Gene pathways that were affected by different small molecule inhibitors for MLL-AF9 leukemia. **A-E.** Gene set enrichment analyses (GSEA) evaluating gene signatures regulated by Hoxa9/Meis1 (A), Myc (B), Mad1 (C), or sensitive to Rapamycin (D) or Hypoxia (E) in the RNA-seq analyses of MM-401 treated MLL-AF9 cells. NES, normalized enrichment score; FDR, false-discovery rate. **F-H**, GO term analysis for genes that were regulated by MENIN/DOT1L (F), BRD4 (G) as well as genes that were up regulated by EPZ-5676 and MI-2-2 (H). Genes with log2 fold change >1 or <-1 were used for analysis.
