# Supplementary material for: MLL1 and MLL1 fusion proteins have distinct functions in regulating leukemic transcription program
Source: Cell Discov. 2016 May 17;2:16008–. doi: 10.1038/celldisc.2016.8 (PMC4869169; doi:10.1038/celldisc.2016.8)
Supplement: Supplementary Figure S6 [file celldisc20168-s6.pdf]

Supplemental Figure 6

A

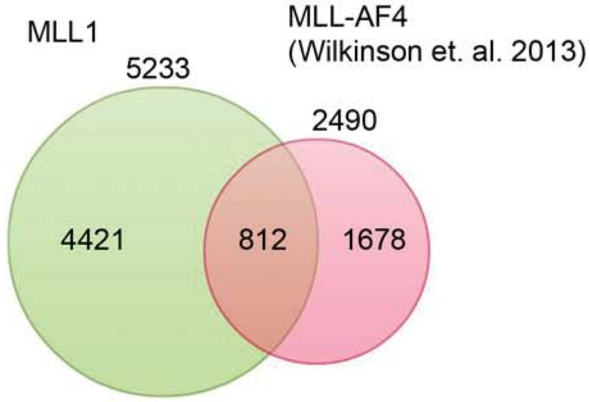


B

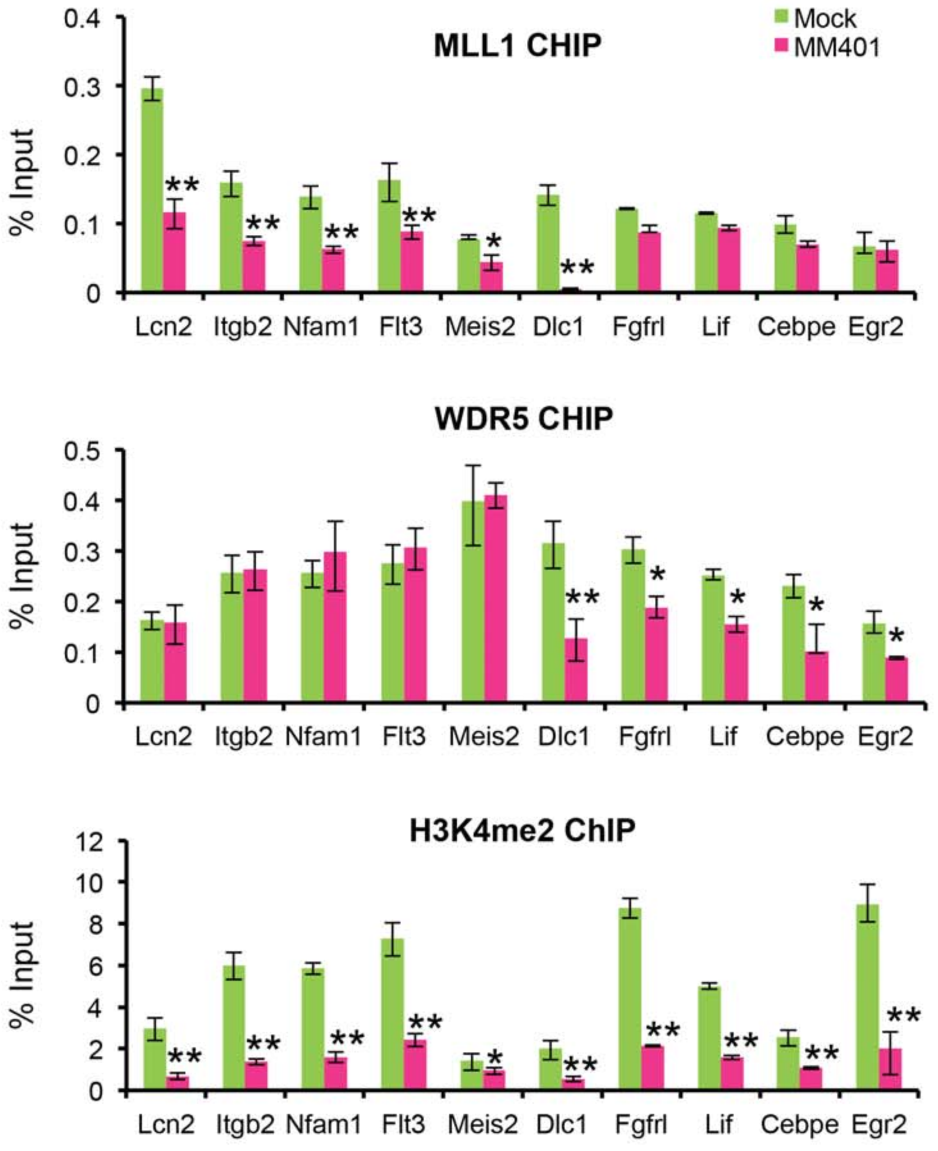

**Supplemental Figure 6** (A) Venn diagram of MLL1 and MLL-AF4 direct targets as indicated. The MLL-AF4 direct targets were previously reported [2]. (B) ChIP assays for MLL1, WDR5 and H3K4me2 in Molm13 cells at selected gene loci as indicated on bottom. Signals for each experiment were normalized to 5% input. Means and standard deviations (as error bars) from at least three independent experiments were presented. Student *t*-test were performed for statistical analyses, * $p<0.05$, ** $p<0.01$.
