# Supplementary material for: MLL1 and MLL1 fusion proteins have distinct functions in regulating leukemic transcription program
Source: Cell Discov. 2016 May 17;2:16008–. doi: 10.1038/celldisc.2016.8 (PMC4869169; doi:10.1038/celldisc.2016.8)
Supplement: Supplementary Table S1 [file celldisc20168-s7.pdf]

Supplemental Table 1 Complete lists for the binding sites for MLL1, WDR5 and H3K4me2 as detected by ChIP-seq.

DMSO_WDR5 bound gene annotation

Chr	Start	End	Annotatior	Detailed Ar	Distance tc	Nearest Pr	Entrez ID	Gene Nam	Gene Alias
chr19	58858502	58858825	Intergenic	MLT1C|LTI	10241	NM_02620	67507	1700019N1	-
chr7	87177377	87177725	intron (NM	intron (NM	6437	NM_17543	209225	Zfp710	5430400N0
chr11	51912927	51913000	intron (NM	CpG	637	NM_01941	19052	Ppp2ca	PP2A|R753
chr10	77888477	77888750	intron (NM	CpG	198	NM_00779	13014	Cstb	AA960480|
chr9	64128877	64128950	promoter-T	promoter-T	-501	NM_02537	66131	Tipin	1110005A0
chr2	1.37E+08	1.37E+08	promoter-T	promoter-T	-157	NM_01382	16449	Jag1	ABE2|Gsfa
chr14	60588627	60588950	intron (NM	intron (NM	116883	NM_01580	50769	Atp8a2	AI415030|/
chr2	90757502	90758275	exon (NM_	exon (NM_	319	NM_02557	66461	Ptpmt1	1110001D1
chr13	91600677	91600825	promoter-T	promoter-T	49	NM_02418	66970	Ssbp2	1500004K0
chr6	51420727	51421125	intron (NM	CpG-12065	311	NM_00762	12417	Cbx3	HP1g|M32
chr13	37491152	37491550	intron (NM	intron (NM	54137	NM_01074	17084	Ly86	MD-1|MD1
chr7	1.07E+08	1.07E+08	intron (NM	L1MC4a|LI	10248	NM_00116	446101	Xrra1	AI449753
chr9	50411652	50411725	promoter-T	promoter-T	266	NM_02584	66925	Sdhd	3110001M
chr1	66747902	66748450	intron (NM	intron (NM	709	NM_02568	66646	Rpe	2810429B0
chr9	43708327	43708625	Intergenic	Intergenic	-142991	NM_00938	21838	Thy1	CD90|T25|
chr3	1.21E+08	1.21E+08	Intergenic	Intergenic	-1592	NM_01017	14066	F3	AA409063|
chr1	26334527	26334650	Intergenic	RMER15|L1	409717	NM_00103	210940	4931408C2	-
chr1	1.64E+08	1.64E+08	Intergenic	Intergenic	-6300	NM_01017	14103	Fasl	APT1LG1|(
chr6	1.23E+08	1.23E+08	Intergenic	B1F2|SINE	-23925	NM_01140	20527	Slc2a3	AA408729|
chr4	1.19E+08	1.19E+08	promoter-T	promoter-T	-303	NM_00129	230700	Foxj3	C330039G0
chr10	7310002	7310100	exon (NM_	exon (NM_	453	NM_17278	237253	Lrp11	1700034J1
chr1	95376152	95376250	promoter-T	promoter-T	563	NM_00115	18000	2-Sep	AW208991
chr16	56029977	56030225	promoter-T	promoter-T	-271	NM_00102	76302	Pcnp	1110018D0
chr10	20859552	20859675	intron (NM	intron (NM	21177	NM_00119	17863	Myb	AI550390|
chr2	1.64E+08	1.64E+08	intron (NM	intron (NM	9040	NM_01110	18769	Pkig	PKIgamma
chr6	1.35E+08	1.35E+08	intron (NM	intron (NM	833	NM_13044	70686	Dusp16	3830417M
chr16	30604402	30604500	Intergenic	Intergenic	4642	NM_17763	224093	Fam43a	Tuf1
chr11	28265652	28265800	Intergenic	Intergenic	218598	NM_00116	216613	Ccdc85a	-
chr9	1.19E+08	1.19E+08	3' UTR (NM	3' UTR (NM	-10043	NM_00739	11481	Acvr2b	ActRIIB
chr6	1.21E+08	1.21E+08	intron (NM	intron (NM	1962	NM_00754	12122	Bid	2700049M
chr11	1.03E+08	1.03E+08	intron (NM	intron (NM	3599	NM_01967	57778	Fmnl1	8030453N1
chr1	95698577	95698650	promoter-T	promoter-T	-102	NM_00110	21915	Dtymk	AU044245
chr10	79578802	79578900	promoter-T	promoter-T	-430	NM_01149	20869	Stk11	AA408040|
chr8	1.26E+08	1.26E+08	intron (NM	intron (NM	26831	NM_02049	57247	Zfp276	AW048709
chr2	1.45E+08	1.45E+08	Intergenic	Intergenic	-15759	NM_05319	94249	Slc24a3	NCKX3
chr16	22060402	22060525	3' UTR (NM	3' UTR (NM	50906	NR_02748	75826	Senp2	2310007L0
chr4	1.01E+08	1.01E+08	intron (NM	Lx8|LINE|L	24814	NM_00116	72685	Dnajc6	2810027M
chr9	32346827	32347125	intron (NM	intron (NM	2061	NM_00802	14247	Fli1	EWSR2|Fli-
chr1	99569502	99569600	Intergenic	Intergenic	-10956	NM_13382	52392	D1Ertd622	AI987691|/
chr1	21551802	21552000	intron (NM	intron (NM	178264	NM_00111	98582	Khdc1b	AU014713
chr15	58836002	58836075	intron (NM	intron (NM	70673	NM_02317	66218	Ndufb9	1190008J1
chr1	97210177	97210625	exon (NM_	exon (NM_	196	NM_02632	67698	Fam174a	2310044D2
chr11	1.2E+08	1.2E+08	promoter-T	promoter-T	378	NM_01156	21681	Alyref	ALY|REF1|
chr9	64677852	64678050	intron (NM	intron (NM	19133	NM_00116	102442	Dennd4a	AI115600|/
chr7	52114677	52114825	intron (NM	intron (NM	1978	NM_00129	59047	Pnkp	1810009G0

chr4	11249102	11249400	promoter-T	promoter-T	27	NM_00108	381510	Dpy19l4	Gm1023|N
chr17	78761677	78761925	intron (NM	intron (NM	55691	NM_00128	225020	Fez2	9030616F1
chr13	61666327	61666575	intron (NM	intron (NM	5036	NM_02690	117066	Cts3	1600000I2
chr12	31786252	31786325	Intergenic	Intergenic	27455	NM_02782	104943	Fam110c	9030611O1
chr2	1.8E+08	1.8E+08	promoter-T	promoter-T	-883	NM_02558	66481	Rps21	1810049N1
chr2	6632077	6632175	intron (NM	intron (NM	173916	NM_01016	14007	Celf2	B230218O
chr15	12307577	12307800	intron (NM	MIRb|SINE	56437	NM_02567	66629	Golph3	4733401N
chr2	1.02E+08	1.02E+08	intron (NM	intron (NM	41954	NM_17888	241576	Ldlrad3	6430500P
chr17	72507652	72507800	intron (NM	MIRc|SINE	376439	NM_03017	78785	Clip4	1700024K1
chr16	32159102	32159275	intron (NM	intron (NM	6374	NM_14606	224109	Nrros	E430025L0
chr1	33776777	33777075	promoter-T	promoter-T	185	NM_00899	19335	Rab23	AW545388
chr11	21302952	21303200	Intergenic	Intergenic	-31806	NM_00129	216558	Ugp2	UDPGP|UG
chr3	19091052	19091275	intron (NR_	RLTR11B|L	2942	NR_04557	67472	Mtfr1	1300002C
chr11	68938652	68938875	Intergenic	Intergenic	-1116	NM_01178	23801	Aloxe3	e-LOX-3|eL
chr11	54734852	54735100	intron (NM	intron (NM	18620	NM_00816	14778	Gpx3	AA960521|
chr1	75371702	75372150	promoter-T	promoter-T	54	NM_00746	11790	Speg	AW125581
chr9	42104627	42104850	Intergenic	Intergenic	-32355	NM_17276	235293	Sc5d	A830037K
chr7	1.17E+08	1.17E+08	Intergenic	Intergenic	42090	NM_00951	22390	Wee1	Wee1A
chr6	1.19E+08	1.19E+08	exon (NM_	exon (NM_	1140	NM_13394	101358	Fbxl14	AW322056
chr5	23670952	23671075	exon (NM_	exon (NM_	232	NM_15309	231042	Nupl2	AV116043|
chr12	21423277	21423350	promoter-T	promoter-T	-16	NM_01173	22630	Ywhaq	2700028P
chr4	1.07E+08	1.07E+08	exon (NM_	exon (NM_	345	NM_02550	56280	Mrpl37	2300004O1
chr17	74738227	74738625	promoter-T	promoter-T	99	NM_01696	50850	Spast	Spg4|mKIA
chr8	47223577	47223750	Intergenic	Intergenic	-14163	NM_20721	102141	Snx25	AI661919|
chr4	1.07E+08	1.07E+08	Intergenic	Intergenic	47966	NM_01987	56296	Dmrtb1	Dmrt6|Prp
chr3	1.33E+08	1.33E+08	Intergenic	Intergenic	-3648	NM_02995	77669	Arhgef38	9130221D2
chr15	36782177	36782375	Intergenic	Intergenic	-55592	NM_00125	22631	Ywhaz	1110013I1
chr6	1.21E+08	1.21E+08	TTS (NM_0	TTS (NM_0	15488	NM_01737	53857	Tuba8	-
chr17	27954077	27954400	intron (NM	intron (NM	3249	NM_00103	224647	D17Wsu92	AU020189
chr18	23159577	23159825	intron (NM	intron (NM	37463	NM_00116	319211	Nol4	1700013J1
chr1	1.62E+08	1.62E+08	intron (NM	CpG	297	NM_00978	12301	Cacybp	SIP
chr12	1.12E+08	1.12E+08	Intergenic	MIRm|SINE	-6193	NM_00128	22031	Traf3	AI528849|
chr11	1.21E+08	1.21E+08	exon (NM_	exon (NM_	219	NM_02642	67880	Dcxr	0610038K
chr11	51420152	51420425	promoter-T	promoter-T	95	NM_00104	15384	Hnrnpab	3010025C1
chr6	35489402	35489625	intron (NM	CpG	375	NM_00809	14489	Mtpn	5033418D1
chr4	1.16E+08	1.16E+08	promoter-T	promoter-T	-269	NM_02986	77110	Gpbp1l1	5330440M
chr13	1.09E+08	1.09E+08	intron (NM	intron (NM	-39618	NM_17868	218581	Depdc1b	9830132O
chr13	1.02E+08	1.02E+08	Intergenic	Intergenic	-32931	NM_02288	69048	Slc30a5	1810010K
chr2	1.02E+08	1.02E+08	intron (NM	intron (NM	-20402	NM_00107	20511	Slc1a2	1700091C1
chr2	1.52E+08	1.52E+08	intron (NM	intron (NM	1725	NM_00778	12995	Csnk2a1	Csnk2a1-rs
chr19	53962877	53963225	Intergenic	Intergenic	-3670	NM_01105	18569	Pdcd4	D19Ucla1|
chr1	1.23E+08	1.23E+08	intron (NM	intron (NM	492	NM_00127	72999	Insig2	2900053I1
chr17	6106927	6107075	intron (NM	CpG	171	NM_00110	68842	Tulp4	1110057P
chr11	59652952	59653075	intron (NM	CpG	256	NM_01199	26572	Cops3	Csn3|Sgn3
chr9	75944777	75945100	intron (NM	intron (NM	82154	NM_17373	208982	Hmgcll1	BC037381
chr16	32644077	32644300	promoter-T	promoter-T	-541	NM_00111	51789	Tnk2	Ack|Ack-1|
chr2	37278077	37278400	intron (NM	CpG	185	NM_00110	319817	Rc3h2	2900024N

chr2	1.53E+08	1.53E+08	Intergenic	MTD|LTR|I	-32456	NM_00113	329540	Nol4l	4931410H1
chr19	46072827	46073025	promoter-T	promoter-T	52	NM_19829	71617	9130011E1	AI431055|/
chr17	17511352	17511425	promoter-T	promoter-T	92	NM_02593	67045	Riok2	2010110K2
chr16	54115652	54115850	Intergenic	L1Md_F2|L	1167599	NM_17872	239852	Zpld1	9430016A2
chr10	35764927	35765600	Intergenic	RLTR13D3|	-461350	NM_00125	319415	Hs3st5	D930005L0
chr6	59112977	59113150	Intergenic	Intergenic	-45801	NM_00108	68140	Tigd2	3632410O1
chr1	97553927	97554325	intron (NM	intron (NM	10045	NM_00918	20452	St8sia4	PST|PST-1|
chr1	64784802	64784975	promoter-T	promoter-T	-564	NM_02272	14367	Fzd5	5330434N0
chr4	1.32E+08	1.32E+08	5' UTR (NM	5' UTR (NM	115	NM_02792	71787	Trnau1ap	1110007F0
chr19	38171452	38171675	promoter-T	promoter-T	-6	NM_18174	107221	Ffar4	AI552415|(
chr3	94690977	94691075	promoter-T	promoter-T	-146	NM_00894	19172	Psmb4	Pros-27
chr1	1.35E+08	1.35E+08	intron (NM	intron (NM	12837	NM_00857	17248	Mdm4	4933417N0
chr7	28233627	28233700	intron (NM	MIR|SINE|	666	NM_14492	233016	Blvrb	-
chr2	1.65E+08	1.65E+08	Intergenic	Intergenic	-1688	NM_19902	329559	Zfp335	1810045J0
chr8	1.08E+08	1.08E+08	promoter-T	promoter-T	-160	NM_02888	74356	4931428F0	AI426165|I
chr2	32306602	32306775	exon (NM_	exon (NM_	302	NM_00116	227731	Slc25a25	1110030N1
chr11	49832302	49832400	Intergenic	Intergenic	-6482	NM_02154	59044	Rnf130	G1RZFP|G1
chr15	63872402	63872550	intron (NM	intron (NM	19534	NM_14484	223601	Fam49b	0910001A0
chr6	1.47E+08	1.47E+08	promoter-T	promoter-T	-138	NM_02531	108098	Med21	0610007L0
chr1	88421327	88422025	Intergenic	CpG	-1635	NM_00897	19231	Ptma	Thym
chr8	72315552	72315650	intron (NM	CpG	539	NM_13322	170759	Atp13a1	Atp13a|Cg
chr5	1.22E+08	1.22E+08	exon (NM_	exon (NM_	1266	NM_01129	19988	Rpl6	Taxreb107
chr4	1.34E+08	1.34E+08	intron (NM	intron (NM	382	NM_00128	71904	Paqr7	2310021M
chr3	95951877	95951950	intron (NM	intron (NM	-13980	NM_18140	194126	Mtmr11	AI035306|I
chr5	73243227	73243325	intron (NM	L2|LINE|L2	16411	NM_00111	21682	Tec	-
chr4	1.55E+08	1.55E+08	5' UTR (NM	5' UTR (NM	235	NM_02533	66077	Aurkaip1	0610033H0
chr9	1.11E+08	1.11E+08	TTS (NM_1	TTS (NM_1	-2872	NM_18310	74306	Prss46	1700112C1
chr2	1.25E+08	1.25E+08	intron (NM	intron (NM	113462	NM_02359	110074	Dut	5031412I0
chr12	29579602	29579850	Intergenic	Intergenic	143033	NM_20135	380752	Tssc1	D12Ertd60
chr6	1.25E+08	1.25E+08	intron (NM	ORR1A2-in	6176	NM_17555	269800	Zfp384	BB163993|
chr12	1.01E+08	1.01E+08	intron (NM	intron (NM	93989	NM_03017	78767	Efcab11	2610021K2
chr3	90051877	90052075	promoter-T	promoter-T	-138	NM_01390	30791	Slc39a1	Zip1|Zirtl
chr2	29988602	29988825	promoter-T	promoter-T	-678	NM_14625	70296	Tbc1d13	2600014A0
chr3	1.01E+08	1.01E+08	intron (NM	intron (NM	42340	NM_20720	78908	Igsf3	1700016K1
chr18	70849177	70849600	Intergenic	Intergenic	121442	NM_01077	17191	Mbd2	MBD2a
chr7	1.08E+08	1.08E+08	promoter-T	promoter-T	-474	NM_02428	19346	Rab6a	2610028L1
chr14	55502402	55502550	intron (NM	intron (NM	214	NM_00753	12050	Bcl2l2	AW048834
chr10	1.27E+08	1.27E+08	intron (NM	intron (NM	16403	NM_00851	16971	Lrp1	A2mr|AI31
chr2	1.67E+08	1.67E+08	Intergenic	Intergenic	-16331	NM_17778	277360	Prex1	G630042G0
chr3	1.29E+08	1.29E+08	intron (NM	intron (NM	88634	NM_13045	170439	Elovl6	C77826|FA
chr1	1.31E+08	1.31E+08	Intergenic	Charlie25|I	-34137	NM_00991	12767	Cxcr4	CD184|Cm
chr2	1.52E+08	1.52E+08	intron (NM	CpG	378	NM_21244	228769	Psmf1	AW048666
chr2	1.2E+08	1.2E+08	exon (NM_	exon (NM_	178	NM_14612	228550	Itpka	-
chr1	52198552	52198650	intron (NM	intron (NM	22135	NM_00120	20846	Stat1	2010005J0
chr7	51655277	51655400	TTS (NM_0	TTS (NM_0	15842	NM_01666	20981	Syt3	AI385753|:
chr6	53457552	53457725	Intergenic	Intergenic	-65730	NM_17272	231991	Creb5	Crebpa|D4
chr14	56724352	56724450	Intergenic	Intergenic	-2990	NM_00780	13035	Ctsg	-

chr10	19467577	19467750	intron (NM	intron (NM	35270	NM_17278	237313	Il20ra	E230031K1
chr1	1.46E+08	1.46E+08	Intergenic	L3|LINE|CF	57166	NM_00906	19735	Rgs2	GOS8
chr6	32730652	32730775	Intergenic	Intergenic	-192521	NM_17575	243743	Plxna4	9330117B1
chr18	67550977	67551400	intron (NM	CpG-7057	803	NM_02647	67951	Tubb6	2310057H1
chr7	1.13E+08	1.13E+08	promoter-T	promoter-T	154	NM_02980	76932	Arfip2	2310002N0
chr13	4608027	4608950	promoter-T	promoter-T	-78	NM_01885	56043	Akr1e1	1810061I1(
chr5	42235377	42235625	promoter-T	promoter-T	53	NM_00108	665775	Bod1l	A230054D0
chr15	81559677	81560000	intron (NM	CpG	511	NM_01124	19387	Rangap1	C79654|Fu
chr12	56567277	56567425	Intergenic	Intergenic	26283	NM_01090	18035	Nfkbia	AI462015|I
chr5	1.46E+08	1.46E+08	intron (NM	CpG	301	NM_02314	11867	Arpc1b	41kDa|AA4
chr2	26662527	26662625	Intergenic	RMER19B|	57939	NM_03071	80908	Abo	NAGAT
chr15	38465227	38465425	Intergenic	Intergenic	-16305	NM_01874	54375	Azin1	1700085L0
chr10	23507127	23507375	promoter-T	promoter-T	-236	NM_01129	20042	Rps12	-
chr8	1.08E+08	1.08E+08	intron (NM	CpG	177	NM_17343	244631	Pskh1	AW539964
chr14	59819652	59820325	promoter-T	promoter-T	-77	NM_02776	71330	Rcbtb1	5430409I1
chr10	11407327	11407525	Intergenic	IAP-d-int|L	344183	NM_01014	13853	Epm2a	TG-B|Tg(Tc
chr2	68698752	68699150	promoter-T	promoter-T	-663	NM_17285	241447	Cers6	4732462C0
chr8	64849927	64850125	Intergenic	Intergenic	-248036	NM_01192	26359	Anxa10	-
chr1	34858727	34859075	intron (NM	CpG	334	NM_18301	226970	Arhgef4	9330140K1
chr19	16207752	16207875	5' UTR (NM	5' UTR (NM	492	NM_00813	14682	Gnaq	1110005L0
chr14	1.04E+08	1.04E+08	promoter-T	promoter-T	4	NM_20721	105689	Mycbp2	AU023734
chr11	1.16E+08	1.16E+08	intron (NM	intron (NM	2494	NM_02417	60441	Mrpl38	1110036N2
chr11	94232302	94232600	intron (NM	intron (NM	21839	NM_02960	76408	Abcc3	1700019L0
chr7	86524827	86524925	exon (NM_	exon (NM_	7037	NM_02059	19771	Rlbp1	3110056M
chr17	31433227	31433325	5' UTR (NM	5' UTR (NM	107	NM_00124	224674	Slc37a1	G3PP
chr18	46901527	46901750	promoter-T	promoter-T	67	NM_00968	11777	Ap3s1	[s]3A
chr12	96692452	96692550	Intergenic	Intergenic	-237935	NM_20151	399558	Flrt2	-
chr13	1.14E+08	1.14E+08	Intergenic	Intergenic	57721	NM_02361	71690	Esm1	0610042H2
chr6	22301677	22301825	intron (NM	intron (NM	4330	NM_13858	27999	Fam3c	D6Wsu176
chr3	75758077	75758375	intron (NM	intron (NM	2645	NM_17519	73124	Golim4	3110027H2
chr7	38716802	38717075	Intergenic	Intergenic	87633	NM_01127	19777	Uri1	C80913|NN
chr4	1.55E+08	1.55E+08	exon (NM_	exon (NM_	462	NM_17869	230991	B930041F1	MNCb-396
chr4	1.38E+08	1.38E+08	intron (NM	intron (NM	330	NM_17270	230861	Eif4g3	1500002J2
chr17	5189952	5190100	intron (NM	CpG	194952	NM_00108	239985	Arid1b	8030481M
chr11	45679502	45679575	intron (NM	Lx9|LINE|L	14072	NM_00104	216705	Clint1	AI642036|/
chr1	1.27E+08	1.27E+08	intron (NM	intron (NM	19516	NM_02373	74117	Actr3	1200003A0
chr6	28373727	28373925	intron (NM	intron (NM	186	NM_00748	11844	Arf5	-
chr5	32714777	32714925	Intergenic	MLT1D|LTI	-46492	NM_17270	19046	Ppp1cb	1200010B1
chr13	98772277	98772400	intron (NM	intron (NM	203782	NM_01202	110596	Arhgef28	9230110L0
chr16	24090177	24090775	Intergenic	Intergenic	-101778	NM_00974	12053	Bcl6	Bcl5
chrX	1.39E+08	1.39E+08	intron (NM	intron (NM	1469	NM_00119	209497	Tmem164	AI316850|/
chr11	98662702	98663000	intron (NM	intron (NM	5768	NM_02872	74026	Msl1	2810017F1
chr5	1.39E+08	1.39E+08	Intergenic	Intergenic	-71572	NM_03056	80752	Fam20c	C76981|DN
chrX	13421902	13422075	intron (NM	intron (NM	1694	NM_00128	12361	Cask	DXPri1|DXI
chr3	21835277	21835350	Intergenic	Intergenic	-140261	NM_03073	81004	Tbl1xr1	8030499H0
chr1	1.17E+08	1.17E+08	Intergenic	RMER17B|	-974276	NM_00107	636808	Cntnap5a	Caspr5-1|E
chr2	32815902	32816100	intron (NM	MIRb|SINE	774	NM_19930	227738	Lrsam1	-

chr18	32513002	32513175	Intergenic	MTC|LTR|I	-23783	NM_00108	30948	Bin1	ALP-1|Amp
chr9	54798277	54798425	promoter-T	promoter-T	-315	NM_01196	26441	Psma4	C9
chr17	24343627	24343800	exon (NM_	exon (NM_	-1206	NM_00116	224617	Tbc1d24	9630033P1
chr4	1.53E+08	1.53E+08	promoter-T	promoter-T	-233	NM_17528	97159	A430005L1	C79672
chr16	32113852	32114050	Intergenic	Intergenic	14063	NM_02589	66994	Cep19	1500031L0
chr1	1.93E+08	1.93E+08	intron (NM	intron (NM	1836	NM_17877	320119	Rps6kc1	AA682037|
chr17	27760202	27760400	promoter-T	promoter-T	96	NM_01983	56409	Nudt3	1110011B0
chr5	1.15E+08	1.15E+08	Intergenic	Intergenic	-13480	NM_00932	21405	Hnf1a	AI323641|
chr3	1.09E+08	1.09E+08	intron (NM	intron (NM	-61962	NM_14613	57257	Vav3	A530094I0
chr1	1.58E+08	1.58E+08	promoter-T	promoter-T	-368	NM_17284	240832	Tor1aip2	1110020D1
chr11	1.03E+08	1.03E+08	Intergenic	Intergenic	-23573	NM_18303	353047	Plekhm1	AP162|B2|
chr11	1.16E+08	1.16E+08	promoter-T	promoter-T	-215	NM_02955	76265	Tsen54	0610034P0
chr1	30929952	30930025	Intergenic	CpG	-9887	NM_00108	213109	Phf3	2310061N1
chr2	52826352	52826500	intron (NM	intron (NM	109524	NM_17240	71409	Fmnl2	5430425K0
chr14	1.06E+08	1.06E+08	Intergenic	Intergenic	-61739	NM_19860	212085	Trim52	4921513B0
chr1	9418052	9418175	Intergenic	RMER16-in	-117376	NM_02151	59014	Rrs1	5730466A0
chrX	1.48E+08	1.48E+08	intron (NM	CpG	198	NM_00116	69499	Tsr2	2310007F1
chr3	61168502	61168750	5' UTR (NM	5' UTR (NM	197	NM_02871	74012	Rap2b	4021402C1
chr10	13194102	13194175	promoter-T	promoter-T	64	NM_00119	215789	Phactr2	AV158170|
chr14	52724302	52724425	promoter-T	promoter-T	-660	NM_00117	15381	Hnrnpc	AL022939|
chr3	83663577	83663800	Intergenic	Intergenic	-18158	NM_01190	24088	Tlr2	Ly105
chr4	62186077	62186200	promoter-T	promoter-T	-90	NM_02149	59001	Pole3	1810034K1
chr9	21640202	21640350	intron (NM	intron (NM	322	NM_00108	624219	Gm6484	Angptl8|EC
chr11	1.04E+08	1.04E+08	intron (NM	intron (NM	567	NM_00108	76719	Kansl1	1700081L1
chr19	57836977	57837350	intron (NM	intron (NM	151639	NM_18141	226255	Atrnl1	AI504415|
chr6	1.2E+08	1.2E+08	promoter-T	promoter-T	-854	NM_14599	214899	Kdm5a	AA409370|
chr1	1.33E+08	1.33E+08	promoter-T	promoter-T	-640	NM_00882	18640	Pfkfb2	4930568D0
chr15	77054102	77054350	intron (NM	intron (NM	-69984	NM_00128	93686	Rbfox2	Fbm2|Fxh|
chr3	95685902	95687000	promoter-T	promoter-T	-300	NM_00103	229599	Ciart	Chrono|Gr
chr5	1.4E+08	1.4E+08	promoter-T	promoter-T	72	NM_00125	77053	Sun1	4632417G1
chr2	69661002	69661200	intron (NM	CpG	674	NM_17778	277396	Klhl23	C130068N1
chr10	80765327	80765700	intron (NM	intron (NM	9805	NM_00114	18717	Pip5k1c	AI115456|
chr8	23988852	23988925	intron (NM	intron (NM	18877	NM_00108	244349	Kat6a	1500036M
chr19	21686577	21686825	intron (NM	MER20|DN	40580	NM_02542	66206	1110059E2	-
chr9	1.1E+08	1.1E+08	5' UTR (NM	5' UTR (NM	430	NM_01875	54369	Nme6	nm23-M6
chr6	91657352	91657425	intron (NM	intron (NM	23327	NM_00932	21366	Slc6a6	AA589629|
chr11	76015527	76015700	promoter-T	promoter-T	55	NM_02777	116972	Fam57a	2310047D1
chrY	2870052	2870225	Intergenic	Intergenic	482880	NM_00127	1E+08	Gm3376	Rbmy1b
chr15	58247127	58247300	5' UTR (NM	5' UTR (NM	190	NM_14595	210998	D15Ertd62	AV220772|
chr13	22036977	22037425	intron (NM	CpG	238	NM_18301	193452	Zfp184	4930500C1
chr8	1.22E+08	1.22E+08	Intergenic	Intergenic	-13461	NM_02795	71839	Osgin1	1700012B1
chr9	74940127	74940200	intron (NM	PB1|SINE|	21150	NM_01086	17918	Myo5a	9630007J1
chr18	34490727	34491050	promoter-T	promoter-T	89	NM_02552	66384	Srp19	2310020D2
chr8	72735802	72736100	intron (NM	CpG	229	NM_00100	73095	Slc25a42	2900084M
chr2	34625627	34625775	Intergenic	MT2B|LTR	-1909	NM_00116	14828	Hspa5	AL022860|
chr10	31259152	31259450	intron (NM	L1MA8|LIN	70230	NM_00114	268291	Rnf217	AU016819
chr18	36675327	36675500	promoter-T	promoter-T	46	NM_01041	15200	Hbegf	AW047313

chr15	34167977	34168125	5' UTR (NM	5' UTR (NM	270	NM_03352	114128	Laptm4b	C330023P1
chr16	23162852	23162950	Intergenic	L1_Mur3|L	16292	NM_00960	11450	Adipoq	30kDa|APN
chr10	3134402	3134500	5' UTR (NM	5' UTR (NM	147	NM_17254	215748	Cnksr3	6820402C0
chr7	19766302	19766375	promoter-T	promoter-T	-181	NM_00116	72205	Eml2	1600029N0
chr13	64255277	64255425	promoter-T	promoter-T	-844	NM_17549	238673	Zfp367	8030486J2
chr2	5623452	5623700	intron (NM	GA-rich|Lo	12232	NM_00129	227541	Camk1d	A630059D1
chr11	1.2E+08	1.2E+08	intron (NM	intron (NM	6819	NM_00119	11302	Aatk	AATYK|aat
chr5	1.46E+08	1.46E+08	promoter-T	promoter-T	32	NM_00103	231887	Pdap1	HASPP28|P
chr3	1.26E+08	1.26E+08	Intergenic	Intergenic	99431	NM_17345	271970	Arsj	9330196J0
chr3	88358102	88358200	intron (NM	CpG	513	NM_03352	94232	Ubqln4	A1Up|A1u
chr16	45025602	45025725	Intergenic	Intergenic	-68503	NM_02643	67896	Ccdc80	2610001E1
chr12	39505102	39505200	Intergenic	Intergenic	-1694	NM_00796	14009	Etv1	ER81|Etsrp
chr11	1.07E+08	1.07E+08	Intergenic	Intergenic	-12787	NM_01065	16647	Kpna2	2410044B1
chr7	31609252	31609325	promoter-T	promoter-T	-494	NM_14618	233079	Ffar2	GPCR43|G
chr7	1.28E+08	1.28E+08	intron (NM	CpG	318	NM_00116	26939	Polr3e	RPC5|Sin
chr7	1.49E+08	1.49E+08	intron (NM	CpG	176	NM_02830	101513	Mob2	1110017M
chr15	73562302	73562475	intron (NM	intron (NM	7028	NM_00116	19245	Ptp4a3	AV088979|
chr1	1.79E+08	1.79E+08	intron (NM	intron (NM	58210	NM_01178	23797	Akt3	AI851531|
chr12	88747852	88747950	Intergenic	B2_Mm2|S	-18721	NM_01147	20773	Sptlc2	AI173915|
chr10	59027227	59027750	intron (NM	MIR|SINE|	51952	NM_00103	215999	Mcu	2010012O1
chr2	1.27E+08	1.27E+08	intron (NM	intron (NM	275	NM_00111	72180	Zfp661	2810405K0
chr2	35359227	35359350	Intergenic	Intergenic	-42343	NM_00114	14594	Ggta1	AW108479
chr15	91021752	91021875	exon (NM_	exon (NM_	425	NM_01199	26874	Abcd2	ABC39|ALD
chr13	52710552	52710750	intron (NM	intron (NM	18400	NM_01151	20963	Syk	Sykb
chr1	1.4E+08	1.4E+08	Intergenic	ID_B1|SINE	154172	NM_02160	59125	Nek7	2810460C1
chr5	1.24E+08	1.24E+08	intron (NM	intron (NM	5518	NM_17787	330192	Vps37b	2300007F2
chr6	1.21E+08	1.21E+08	promoter-T	promoter-T	-135	NM_15351	94044	Bcl2l13	BCL-RAMB(
chr5	46248502	46248650	promoter-T	promoter-T	203	NM_17814	209707	Lcorl	Mlr1
chr6	67008977	67009150	intron (NR_	intron (NR_	-21662	NM_00783	13197	Gadd45a	AA545191|
chr5	1.3E+08	1.3E+08	intron (NM	intron (NM	7978	NM_13390	100678	Psph	AI480570|
chr3	14886527	14886600	5' UTR (NM	5' UTR (NM	137	NM_00980	12349	Car2	AI131712|
chr17	24941077	24941175	promoter-T	promoter-T	-201	NM_02919	75178	Meiob	4930528F2
chr14	80883977	80884250	Intergenic	Intergenic	484004	NM_00103	380924	Olfm4	GC1|GW11
chr17	15244352	15244650	Intergenic	Intergenic	54478	NM_00103	381062	Ermard	2210404J1
chr5	1.08E+08	1.08E+08	intron (NM	intron (NM	-51101	NM_00126	14581	Gfi1	AW495828
chr3	1.33E+08	1.33E+08	Intergenic	Intergenic	62159	NM_02792	71793	Ints12	1110020M
chr2	32549602	32549700	promoter-T	promoter-T	44	NM_01023	14287	Fpgs	AA408187
chr8	1.14E+08	1.14E+08	promoter-T	promoter-T	-804	NM_14621	234736	Rfwd3	BC027246
chr7	1.4E+08	1.4E+08	Intergenic	Intergenic	115943	NM_01697	18242	Oat	AI194874
chr4	7013377	7013500	Intergenic	IAPEY3-int|	-95568	NM_14571	252838	Tox	1700007F0
chr2	1.73E+08	1.73E+08	Intergenic	Intergenic	-101329	NM_02299	65112	Pmepa1	2210418I0
chr9	60642552	60642650	intron (NM	CpG	246	NM_02828	72565	Uaca	2700059D0
chr11	77795677	77795750	promoter-T	promoter-T	-605	NM_17485	268448	Phf12	2410142K1
chr9	1.01E+08	1.01E+08	exon (NM_	exon (NM_	24465	NM_00110	77853	Msl2	E130103E0
chr16	11173752	11174075	intron (NR_	intron (NR_	2573	NR_02750	106205	Zc3h7a	A430104C1
chr6	1E+08	1E+08	Intergenic	Intergenic	-24086	NM_01974	56353	Rybp	2410018J2
chr1	1.66E+08	1.66E+08	promoter-T	promoter-T	-31	NM_01134	20343	Sell	AI528707|

chr10	33670477	33670775	intron (NM	intron (NM	392	NM_02828	72580	Zufsp	2700019D(
chr7	52074352	52074500	intron (NM	intron (NM	-2398	NR_03313(	107503	Atf5	AFTA|Atf7
chr13	89879027	89879175	intron (NM	CpG	3016	NM_00108	13003	Vcan	5430420N(
chr16	31233902	31233975	intron (NR_	intron (NR_	-32614	NM_03013	78618	Acap2	4832442G1
chr5	77739527	77739725	promoter-T	promoter-T	117	NM_15379	231329	Polr2b	Pol2rb|Rpb
chr6	1.18E+08	1.18E+08	Intergenic	Intergenic	-52135	NM_00117	108017	Fxyd4	0610008I0
chr5	21241152	21241350	Intergenic	L1M2|LINE	-1727	NM_02843	73078	Pmpcb	3110004O1
chr18	39669502	39669600	Intergenic	L1ME1|LIN	-22652	NM_00817	14815	Nr3c1	GR|Grl-1|(
chr17	46142577	46142725	Intergenic	Intergenic	19461	NM_00111	22339	Vegfa	Vegf|Vpf
chr4	1.3E+08	1.3E+08	Intergenic	Intergenic	-9445	NM_19895	230777	Hcrtr1	Ox1r
chr6	1.25E+08	1.25E+08	intron (NM	intron (NM	13160	NM_00765	12527	Cd9	Tspan29
chr4	1.34E+08	1.34E+08	promoter-T	promoter-T	-320	NM_01695	15331	Hmgn2	HMG-17|H
chr9	1.03E+08	1.03E+08	5' UTR (NM	5' UTR (NM	458	NM_00113	321022	Cdv3	2510010F1
chr8	18948677	18948825	intron (NM	intron (NM	2120	NM_00128	319581	Xkr5	5430438H(
chr6	1E+08	1E+08	promoter-T	promoter-T	-436	NM_01974	56353	Rybp	2410018J2
chr1	1.33E+08	1.33E+08	intron (NM	intron (NM	45754	NM_01875	54354	Rassf5	1300019G2
chr1	94829702	94829950	TTS (NR_01	TTS (NR_01	-1159	NM_01179	23830	Capn10	AW049679
chr11	53321677	53321750	Intergenic	Intergenic	-11525	NM_03314	20362	8-Sep	AW046166
chr17	35338077	35338250	intron (NM	intron (NM	789	NM_01369	21926	Tnf	DIF|TNF-a|
chr4	59795777	59796000	intron (NM	B4A|SINE|	839	NM_00101	66209	Inip	1110054O(
chr1	89657452	89657675	intron (NM	intron (NM	4977	NM_00120	77040	Atg16l1	1500009K(
chr14	64950452	64950625	intron (NM	intron (NM	85255	NM_00125	110265	Msra	2310045J2
chr5	65252877	65253000	Intergenic	Intergenic	58176	NM_00845	16599	Klf3	9930027G(
chr2	1.47E+08	1.47E+08	promoter-T	promoter-T	-383	NM_01191	24128	Xrn2	-
chr1	1.3E+08	1.3E+08	Intergenic	L1MC3|LIN	78975	NM_00991	12767	Cxcr4	CD184|Cm
chr10	1.2E+08	1.2E+08	promoter-T	promoter-T	-683	NM_02867	73914	Irak3	4833428C1
chr3	86562702	86562975	intron (NM	intron (NM	161968	NM_00119	70762	Dclk2	6330415M
chr7	19680102	19680300	exon (NM_	exon (NM_	308	NM_01138	20475	Six5	Dmahp|MI
chr16	22265527	22265600	intron (NM	CpG	439	NM_00918	20462	Tra2b	5730405G2
chr3	97736002	97736150	intron (NM	intron (NM	1980	NM_00103	433632	Gm5544	EG433632
chr15	97679577	97680200	Intergenic	Intergenic	-4955	NM_00120	56233	Hdac7	5830434K(
chr4	46761877	46762050	intron (NM	intron (NM	-98892	NM_19866	381605	Tbc1d2	A630005A(
chr6	1.38E+08	1.38E+08	5' UTR (NM	5' UTR (NM	198	NM_01149	20901	Strap	AW557906
chr4	1.28E+08	1.28E+08	promoter-T	promoter-T	-227	NM_00119	54383	Phc2	A3galt2|A/
chr7	1.35E+08	1.35E+08	promoter-T	promoter-T	401	NM_17228	233900	Rnf40	AI848422|
chr7	1.09E+08	1.09E+08	intron (NM	intron (NM	6893	NM_00104	69710	Arap1	2410002L1
chr7	1.14E+08	1.14E+08	Intergenic	Intergenic	-7392	NM_00127	434223	Gm1966	-
chrX	1.03E+08	1.03E+08	Intergenic	MLT2F|LTF	-8276	NM_20582	279572	Tlr13	AI666735|
chr10	20710277	20710775	intron (NM	intron (NM	38173	NM_02620	52906	Ahi1	1700015F0
chr6	29559802	29559875	promoter-T	promoter-T	-231	NM_17729	320938	Tnpo3	5730544L1
chr3	1.06E+08	1.06E+08	intron (NM	intron (NM	10655	NM_02811	72121	Dennd2d	2010308M
chr2	1.81E+08	1.81E+08	intron (NR_	CpG	204	NM_00129	66314	Tpd52l2	2810411G2
chr1	1.57E+08	1.57E+08	intron (NM	intron (NM	48037	NM_00978	12290	Cacna1e	A430040I1
chr9	1.23E+08	1.23E+08	promoter-T	promoter-T	-18	NM_02533	66079	Tmem42	0610027O1
chr4	1.29E+08	1.29E+08	exon (NM_	exon (NM_	146	NM_01131	20218	Khdrbs1	Sam68|p62
chr14	46366352	46366425	Intergenic	Intergenic	-88570	NM_00104	114874	Ddhd1	PA-PLA1
chr9	44142802	44142950	promoter-T	promoter-T	78	NM_01043	15270	H2afx	AW228881

chr4	56960102	56960300	promoter-T	promoter-T	100	NM_17551	242474	Tmem245	A630051L1
chr8	34776802	34777000	intron (NM	intron (NM	13191	NM_01034	14782	Gsr	AI325518|
chr1	44175452	44175525	5' UTR (NM	5' UTR (NM	130	NM_02364	72050	Kdelc1	1810049A1
chr8	74846352	74846500	TTS (NM_0	TTS (NM_0	3465	NM_00845	16598	Klf2	Lklf
chr17	56483052	56483175	intron (NM	intron (NM	17640	NM_17213	193796	Kdm4b	4732474L0
chr10	1.19E+08	1.19E+08	promoter-T	promoter-T	-365	NM_02799	71902	Cand1	2310038O0
chr13	99717702	99717800	Intergenic	Intergenic	-21412	NM_17871	238799	Tnpo1	AU021749
chr10	20294802	20295125	intron (NM	intron (NM	149911	NM_01387	29863	Pde7b	-
chr7	86903177	86903375	intron (NM	intron (NM	15227	NM_00103	626359	Wdr93	EG626359
chr3	60300002	60300375	intron (NM	intron (NM	-4986	NM_00125	56758	Mbnl1	Mbnl|mKIA
chr15	55697927	55698100	intron (NM	intron (NM	40491	NM_01666	20649	Sntb1	-
chr3	5860452	5860850	Intergenic	LSU-rRNA_	-284403	NM_00116	19302	Pex2	D3Ertd138
chrX	98352827	98353100	intron (NM	L2|LINE|L2	64939	NM_00111	55988	Snx12	2610001F0
chr11	1.15E+08	1.15E+08	Intergenic	MLT1D|LTI	-4992	NM_00108	237988	Cdr2l	D030068L2
chr6	1.14E+08	1.14E+08	intron (NM	intron (NM	15802	NM_00111	108960	Irak2	6330415L0
chr16	17201377	17201475	intron (NM	CpG	159	NM_00945	22195	Ube2l3	C79827|Ub
chr14	46286902	46287125	Intergenic	Intergenic	-9195	NM_00104	114874	Ddhd1	PA-PLA1
chr14	32482977	32483175	Intergenic	Intergenic	28833	NM_02529	26363	Btd	-
chr11	1.18E+08	1.18E+08	Intergenic	Intergenic	-19355	NM_00116	69926	Dnah17	2810003K2
chr4	34497927	34498000	promoter-T	promoter-T	99	NM_00100	433693	Akirin2	2700059D2
chr6	7783702	7783900	Intergenic	MLT1F1|LT	-11423	NM_05299	94192	C1galt1	2210410E0
chr5	1.3E+08	1.3E+08	intron (NM	intron (NM	3134	NM_00128	13852	Stx2	AW538950
chr15	6361602	6361675	intron (NM	intron (NM	24890	NM_00110	13132	Dab2	5730435J1
chr8	69384852	69385150	intron (NM	CpG	885	NM_00116	234344	Naf1	Gm174
chr13	1.11E+08	1.11E+08	Intergenic	Intergenic	-6758	NM_17771	238875	Gapt	9830130M
chr1	1.09E+08	1.09E+08	Intergenic	MIRc|SINE	-1649	NM_00117	18788	Serpinb2	PAI-2|Plan
chr1	94079802	94080125	Intergenic	Intergenic	-34993	NM_20722	208727	Hdac4	4932408F1
chr1	58770002	58770200	promoter-T	promoter-T	-29	NM_00980	12633	Cflar	2310024N1
chr15	63932202	63932375	intron (NM	intron (NM	-40278	NM_14484	223601	Fam49b	0910001A0
chr3	1.46E+08	1.46E+08	intron (NM	intron (NM	2338	NM_01031	14707	Gng5	G(y)5
chr2	1.05E+08	1.05E+08	intron (NM	CpG	493	NM_17719	320554	Tcp11l1	C130096D0
chr8	4323902	4324125	intron (NM	MER20|DN	1087	NM_01048	15568	Elavl1	2410055N0
chr13	1.11E+08	1.11E+08	intron (NM	intron (NM	60888	NM_02385	67295	Rab3c	2700062I0
chr19	6276177	6276650	promoter-T	promoter-T	-483	NM_01011	13660	Ehd1	AA409636|
chr5	1.36E+08	1.36E+08	promoter-T	promoter-T	333	NM_00128	76571	Styxl1	1700011C1
chr15	57907602	57907675	intron (NM	CpG	425	NM_00104	22770	Zhx1	-
chr17	79162627	79162825	intron (NM	intron (NM	-26826	NM_01150	268980	Strn	AU022939
chr2	1.54E+08	1.54E+08	intron (NM	intron (NM	1551	NM_02936	75608	Chmp4b	2010012F0
chr2	1.26E+08	1.26E+08	promoter-T	promoter-T	89	NM_02945	75823	Fam227b	4930525F2
chr16	4984977	4985050	Intergenic	Intergenic	12932	NM_02766	71089	12-Sep	1700028G0
chr2	32464977	32465100	intron (NM	intron (NM	2533	NR_11034	50935	St6galnac6	ST6GalNAc
chrX	35995552	35995625	Intergenic	Intergenic	-7268	NM_02155	59048	C1galt1c1	1500002I1
chr9	57493252	57493475	promoter-T	promoter-T	-376	NM_00778	12988	Csk	AW212630
chr8	1.24E+08	1.24E+08	3' UTR (NM	3' UTR (NM	33081	NM_14560	234825	Klhdc4	AA408426|
chr18	65047752	65047975	intron (NM	CpG	453	NM_00111	83814	Nedd4l	1300012C0
chr10	1.17E+08	1.17E+08	intron (NM	CpG	278	NM_00101	432508	Cpsf6	4733401N1
chr19	5877777	5878075	promoter-T	promoter-T	-540	NM_13415	107375	Slc25a45	AW491445

chr1	1.79E+08	1.79E+08	Intergenic	Intergenic	49224	NM_01391	30928	Zbtb18	RP58|Zfp2
chr10	79391177	79391500	intron (NM	intron (NM	1252	NM_00128	13496	Arid3a	Bright|Dri1
chr4	1.27E+08	1.27E+08	exon (NM_	exon (NM_	731	NM_02360	71514	Sfpq	1110004P2
chr8	11550752	11550900	promoter-T	promoter-T	-55	NM_02424	71941	Cars2	2310051N1
chr13	36041902	36042025	Intergenic	Charlie1a|	-17812	NM_02962	76487	Ppp1r3g	1600032L1
chr17	56357977	56358300	intron (NM	(CGCGG)n|	174	NM_17262	224897	Dpp9	6430584G1
chr11	26287077	26287325	non-coding	non-coding	117	NR_10238	67030	Fancl	2010322C1
chr14	32968002	32968350	intron (NM	intron (NM	-4897	NM_00103	27057	Ncoa4	ARA70|Rfg
chr2	1.28E+08	1.28E+08	intron (NM	CpG	527	NM_00975	12125	Bcl2l11	1500006F2
chrX	1.37E+08	1.37E+08	intron (NM	intron (NM	1396	NM_02146	19139	Prps1	2310010D1
chr8	33039602	33039800	Intergenic	Intergenic	-11026	NM_17859	211323	Nrg1	6030402G
chr4	53790577	53790775	Intergenic	Intergenic	-1901	NM_00931	21350	Tal2	bHLHa19
chr7	1.47E+08	1.47E+08	intron (NM	intron (NM	659	NM_05311	93747	Echs1	C80529
chr1	92499827	92500150	promoter-T	promoter-T	-12	NM_13380	108679	Cops8	9430009J0
chr13	3536927	3537075	promoter-T	promoter-T	-320	NM_00811	14569	Gdi2	GDI-B|GDI
chr16	49836877	49837250	Intergenic	Intergenic	-18704	NM_01058	16423	Cd47	9130415E2
chr1	36603602	36603675	intron (NM	intron (NM	408	NM_02624	109346	Ankrd39	9130416N
chr3	1.21E+08	1.21E+08	Intergenic	Intergenic	-15964	NM_14539	213603	Slc44a3	BC010552
chr8	74117152	74117475	intron (NM	ID_B1|SINI	1230	NM_00129	66171	Pgls	1110030K0
chr8	4253152	4253300	exon (NM_	exon (NM_	124	NM_13396	102209	Snapc2	0610007H1
chr2	34912877	34912950	exon (NM_	exon (NM_	4048	NM_01040	15139	Hc	C5|C5a|He
chr15	91009627	91009700	exon (NM_	exon (NM_	12575	NM_01199	26874	Abcd2	ABC39|ALD
chr1	1.83E+08	1.83E+08	Intergenic	Intergenic	-20442	NM_14551	226757	Wdr26	1600024A0
chr8	98430577	98430775	Intergenic	Intergenic	-18411	NM_01032	14719	Got2	AL022787|
chr5	1.44E+08	1.44E+08	intron (NM	CpG	273	NM_00900	19353	Rac1	AL023026|
chr12	1.1E+08	1.1E+08	promoter-T	promoter-T	83	NM_00116	22375	Wars	TrpRS|WRS
chr1	1.34E+08	1.34E+08	Intergenic	Intergenic	18435	NM_01192	26361	Avpr1b	AVPR3|V3/
chr8	1.26E+08	1.26E+08	intron (NM	intron (NM	-10473	NM_17228	234865	Nup133	mermaid
chr1	1.65E+08	1.65E+08	intron (NM	intron (NM	6617	NM_01888	55990	Fmo2	2310008D0
chr16	23127627	23127725	intron (NM	intron (NM	127	NM_14548	106344	Rfc4	A1|AI8941
chr2	1.53E+08	1.53E+08	intron (NM	B1_Mus1|	-15674	NM_13384	99237	Tm9sf4	AA986553|
chr9	65732802	65733025	promoter-T	promoter-T	67	NM_00117	56404	Trip4	4930558E0
chr14	45938202	45938400	promoter-T	promoter-T	-54	NM_01577	50527	Ero1l	ERO1-L
chr14	32409552	32409625	Intergenic	Intergenic	-19019	NM_00993	382864	Colq	A130034K2
chr16	88480552	88480925	Intergenic	Intergenic	26485	NM_18149	239931	Cldn17	-
chr7	19460052	19460200	Intergenic	Intergenic	-9913	NM_00940	21946	Pglyrp1	PGRP|PGR
chr2	1.01E+08	1.01E+08	Intergenic	MLT1A0|L	-808767	NM_00902	19374	Rag2	Rag-2
chr14	33583902	33584000	Intergenic	Intergenic	-15198	NM_00116	69069	1810011H1	4733401I0
chr4	74224827	74225025	Intergenic	Intergenic	327143	NM_14478	76804	Kdm4c	2410141F1
chr13	53072077	53072425	intron (NM	intron (NM	4157	NM_01737	18030	Nfil3	AV225605|
chr14	62047002	62047125	intron (NM	RSINE1|SIN	11721	NM_00846	16648	Kpna3	IPOA4
chr10	21714102	21714300	promoter-T	promoter-T	-271	NM_01136	20393	Sgk1	Sgk
chr6	1.49E+08	1.49E+08	intron (NM	CpG	1341	NM_01964	56306	Fam60a	Ppcs1|Pptc
chr19	53393552	53393700	intron (NM	intron (NM	8630	NM_00100	17859	Mxi1	ENSMUSG
chr11	1.03E+08	1.03E+08	intron (NM	CpG-2675	12935	NM_00111	11488	Adam11	AW060611
chr13	45422052	45422150	Intergenic	Intergenic	-63010	NM_15378	218203	Mylip	9430057C2
chr3	1.33E+08	1.33E+08	Intergenic	Intergenic	-82197	NM_00104	214133	Tet2	Ayu17-449

chr16	32395927	32396075	Intergenic	Intergenic	-4591	NM_00116	277203	Tm4sf19	EG277203
chr13	41568852	41569200	intron (NM	intron (NM	13703	NM_00111	18003	Nedd9	Cas-L|CasL
chr6	1.4E+08	1.4E+08	intron (NM	intron (NM	90881	NM_14492	109135	Plekha5	2810431N2
chr4	32855202	32855375	intron (NM	intron (NM	-31946	NM_17536	108755	Lyrm2	2610208E0
chr8	1.07E+08	1.07E+08	promoter-T	promoter-T	349	NM_14493	234664	Nae1	59kDa|App
chr7	17497702	17498125	Intergenic	Intergenic	6074	NM_00896	19222	Ptgir	IP|PGI2
chr5	74036527	74036600	intron (NM	intron (NM	2407	NM_01189	24051	Sgcb	43kDa|AI7
chr6	88784977	88785150	TTS (NM_0	TTS (NM_0	6866	NM_03025	80283	Abtb1	AI847549|
chr5	1.38E+08	1.38E+08	Intergenic	(CGG)n|Sir	-7844	NM_02875	74097	Pop7	0610037N1
chr7	1.1E+08	1.1E+08	intron (NM	intron (NM	258	NM_00108	20821	Trim21	Ro52|Ssa1
chr14	73522252	73522500	Intergenic	Intergenic	-19941	NM_00117	105670	Rcbtb2	2610028E0
chr1	1.07E+08	1.07E+08	Intergenic	Intergenic	186711	NM_17877	320311	Rnf152	A930029B0
chr1	93356252	93356550	promoter-T	promoter-T	-496	NM_01106	18627	Per2	mKIAA0347
chr7	1.16E+08	1.16E+08	exon (NM_	exon (NM_	247	NM_02534	66085	Eif3f	0610037M
chr17	33821477	33821550	intron (NM	intron (NM	890	NM_00110	76936	Hnrnpm	2610023M
chr5	1.06E+08	1.06E+08	Intergenic	Intergenic	-45575	NM_00112	231549	Lrrc8d	2810473G0
chr10	55147002	55147175	Intergenic	Intergenic	-679635	NM_00116	73390	Msl3l2	1700060H1
chr9	50165352	50165625	Intergenic	Intergenic	-137142	NM_02963	76509	Plet1	0610037B2
chr2	75521377	75521625	intron (NM	intron (NM	21197	NM_01090	18024	Nfe2l2	AI194320|
chr1	1.45E+08	1.45E+08	Intergenic	Intergenic	-303576	NM_02002	26878	B3galt2	-
chr19	25035952	25036050	exon (NM_	exon (NM_	105	NR_03374	226043	Cbwd1	AV349248|
chr8	13758202	13758275	intron (NM	intron (NM	548	NM_02727	69957	Cdc16	2700071J1
chr13	37148602	37148825	Intergenic	Intergenic	-6600	NM_00116	74145	F13a1	1200014I0
chr17	48061202	48061275	5' UTR (NM	5' UTR (NM	343	NM_02876	74123	Foxp4	1200010K0
chr14	73181227	73181425	Intergenic	Intergenic	-71516	NM_20763	319448	Fndc3a	1700094E1
chr4	1.55E+08	1.55E+08	Intergenic	CpG	64027	NM_00108	76866	Morn1	2900057D2
chr1	1.75E+08	1.75E+08	Intergenic	Lx8|LINE|L	43664	NM_00876	18313	Olfr16	MOR23|M
chr1	65223602	65223800	intron (NM	intron (NM	2015	NM_01049	15926	Idh1	AI314845|
chr2	32612977	32613075	exon (NM_	exon (NM_	272	NM_15280	30933	Tor2a	Prosalusin
chr13	1.03E+08	1.03E+08	Intergenic	RMER30|D	-27250	NM_00853	17079	Cd180	F630107B1
chr1	1.78E+08	1.78E+08	intron (NM	CpG	217	NM_01201	26909	Exo1	5730442G0
chr19	8741502	8741700	intron (NM	intron (NM	3106	NM_00111	12669	Chrm1	Chrm-1|M
chr13	31097127	31097200	Intergenic	MTD|LTR|	-31247	NM_02558	66482	Exoc2	2410030I2
chr16	10768777	10768875	Intergenic	GA-rich|Lo	16803	NM_00989	12703	Socs1	Cish1|Cish
chr3	97705477	97705650	intron (NM	intron (NM	413	NM_01134	20333	Sec22b	4930564D1
chr10	85420302	85420450	exon (NM_	exon (NM_	162	NM_14542	28088	Rtcb	AI255213|
chr12	53107252	53107425	intron (NM	CpG	150	NM_02954	328092	Dtd2	4930578F0
chr5	1.15E+08	1.15E+08	intron (NM	intron (NM	26428	NM_00100	433940	Fam222a	-
chr1	1.66E+08	1.66E+08	intron (NM	intron (NM	17523	NM_00972	11931	Atp1b1	Atp4b|Atp
chr12	16627152	16627400	Intergenic	Intergenic	-30700	NM_17295	14245	Lpin1	4631420P0
chr5	77380027	77380225	promoter-T	promoter-T	-310	NM_02593	67054	Paics	2610511I0
chr14	65568452	65568625	promoter-T	promoter-T	146	NM_17733	219150	Hmbox1	AI451877|
chr12	80747952	80748050	intron (NM	intron (NM	349663	NM_00125	19363	Rad51b	AI553500|
chr1	1.48E+08	1.48E+08	Intergenic	MLT2C1|LT	-158470	NM_15353	215378	Brinp3	B830045N1
chr1	94694402	94694525	Intergenic	Intergenic	-33800	NM_01669	14733	Gpc1	AI462976
chr9	1.06E+08	1.06E+08	intron (NM	intron (NM	331	NM_00908	19944	Rpl29	Rpl43
chr11	59928377	59928450	intron (NM	intron (NM	9898	NM_00902	19377	Rai1	Gt1

chr1	1.94E+08	1.94E+08	intron (NM	intron (NM	124929	NM_01060	16510	Kcnh1	EAG1|Kv1C
chr4	1.55E+08	1.55E+08	promoter-T	promoter-T	-87	NM_00119	67513	2610002J0	Faap20
chr19	3540402	3540550	intron (NM	intron (NM	35273	NM_00116	52036	Ppp6r3	4930528G0
chr11	67399802	67400175	promoter-T	promoter-T	-14	NM_00110	14457	Gas7	AW124766
chr9	70153377	70153500	intron (NM	intron (NM	98281	NM_18107	71602	Myo1e	2310020N2
chr7	87520727	87521075	Intergenic	Intergenic	-4640	NM_17290	140481	Man2a2	1700052O2
chr6	54077527	54077650	intron (NM	intron (NM	87662	NM_00116	69993	Chn2	1700026N2
chr5	91310752	91310925	Intergenic	Intergenic	-9433	NM_00817	14825	Cxcl1	Fsp|Gro1|I
chr11	1.14E+08	1.14E+08	Intergenic	Intergenic	-12384	NM_00116	56699	Cdc42ep4	1500041M
chr9	20953952	20954100	intron (NM	B4A|SINE|	324	NM_01674	12539	Cdc37	p50|p50Cc
chr14	51515852	51516175	Intergenic	ID4_|SINE|	-3083	NM_00116	546611	Klhl33	EG546611
chr17	46439027	46439275	intron (NM	MIRb|SINE	4274	NM_00128	106565	Dlk2	AI413481|I
chr8	97190877	97191025	promoter-T	promoter-T	-40	NM_02426	107566	Arl2bp	1700010P1
chr4	1.34E+08	1.34E+08	intron (NM	CpG	391	NM_01964	16765	Stmn1	19k|Lag|La
chr4	45354877	45355125	promoter-T	promoter-T	28	NM_15316	242418	Dcaf10	AA959934|
chr13	6635777	6636075	intron (NM	intron (NM	12091	NM_01970	56421	Pfkp	1200015H2
chr8	88005727	88005875	Intergenic	Intergenic	-10715	NM_17386	108682	Gpt2	4631422C0
chr12	73637102	73637850	5' UTR (NM	5' UTR (NM	132	NM_02632	67708	Pcnxl4	1810048J1
chr11	1.01E+08	1.01E+08	promoter-T	promoter-T	71	NM_14482	69684	Aarsd1	1110069E2
chr19	44000752	44001000	intron (NM	ID_B1|SINE	13812	NM_02802	71972	Dnmbp	2410003L0
chr5	1.04E+08	1.04E+08	Intergenic	Intergenic	-25437	NM_00116	74218	1700016H1	-
chr1	36766502	36766625	intron (NM	CpG	207	NM_14610	226977	Actr1b	2310066K2
chr1	1.46E+08	1.46E+08	promoter-T	promoter-T	-416	NM_00103	69367	Glrx2	1700010P2
chr15	74759477	74759550	Intergenic	Intergenic	-25968	NM_00116	17069	Ly6e	9804|Ly67
chr7	29123352	29123450	exon (NM_	exon (NM_	337	NM_01367	20924	Supt5	AL033283|
chr7	1.34E+08	1.34E+08	Intergenic	Intergenic	-4795	NM_13368	67375	Qprt	2410027J0
chr3	51149552	51149675	Intergenic	(GAA)n|Sir	-5047	NM_02350	69257	Elf2	2610036A2
chr3	40820802	40820975	Intergenic	L1MEf|LIN	66080	NM_02755	70804	Pgrmc2	4631434O1
chr1	40691502	40691775	Intergenic	Intergenic	-46919	NM_00103	226999	Slc9a2	2210416H1
chr18	75057052	75057550	Intergenic	Intergenic	63616	NM_01072	16891	Lipg	3110013K0
chr14	66915627	66915950	promoter-T	promoter-T	-74	NM_02997	66854	Trim35	0710005M
chr14	27783777	27783900	promoter-T	promoter-T	-101	NM_14522	72993	Appl1	2900057D2
chr3	9250327	9250425	promoter-T	promoter-T	-191	NM_17766	229055	Zbtb10	4832414A1
chr3	90391177	90391325	Intergenic	Intergenic	-2918	NM_00119	628324	S100a2	CaN19|EG6
chr3	85794052	85794125	Intergenic	Intergenic	12325	NM_00100	368202	Prss48	Esspl|Gm1
chr15	96486177	96486350	Intergenic	ORR1D2|L1	-12919	NM_00116	105727	Slc38a1	AA408026|
chr14	8913252	8913550	intron (NM	intron (NM	574	NM_02593	67053	Rpp14	2610511E0
chr12	83626002	83626425	Intergenic	Intergenic	-91812	NM_01581	50779	Rgs6	-
chr10	62383877	62384075	intron (NM	intron (NM	595	NM_17519	73132	Slc25a16	3110021G1
chr14	51563752	51563950	promoter-T	promoter-T	-127	NM_01363	18950	Pnp	AL024301|
chr8	3646902	3647075	Intergenic	Intergenic	-8782	NM_00120	57264	Retn	ADSF|Fizz3
chr11	53520602	53520850	promoter-T	promoter-T	95	NM_00901	19360	Rad50	Mrell|Rad5
chr7	1.51E+08	1.51E+08	promoter-T	promoter-T	41	NM_00876	18400	Slc22a18	AW260131
chr3	1.02E+08	1.02E+08	Intergenic	ORR1E|LTF	-17987	NM_00103	242126	Slc22a15	2610034P2
chr17	74268152	74268275	intron (NM	intron (NM	31323	NM_01172	22436	Xdh	XO|Xor|Xo
chr1	84719477	84719650	3' UTR (NM	3' UTR (NM	-26767	NM_15291	227325	Dner	A930026D1
chr14	48013152	48013550	intron (NM	intron (NM	19816	NM_00114	16854	Lgals3	GBP|L-34|

chr13	54605177	54605675	intron (NM	CpG	260	NM_17698	319719	Simc1	4732471D1
chr19	6140527	6140775	intron (NM	CpG	486	NM_01972	56327	Arl2	2610009M
chr17	35258452	35258875	promoter-T	promoter-T	222	NM_00112	81845	Gpank1	Bat-4|Bat4
chr8	1.29E+08	1.29E+08	Intergenic	Intergenic	-47261	NM_00115	212728	Gm17296	Gm179|Ta
chr9	1.08E+08	1.08E+08	TTS (NM_0	TTS (NM_0	238	NM_03139	83669	Wdr6	mWDR6
chr9	1.14E+08	1.14E+08	promoter-T	promoter-T	-195	NR_10810	12091	Glb1	AW125515
chr10	60837727	60837825	intron (NM	intron (NM	8495	NM_01375	27355	Pald1	MMPAL|Pa
chr19	37030902	37031125	exon (NM_	exon (NM_	30444	NM_00108	107182	Btaf1	AI414500|/
chr1	42922952	42923125	intron (NM	RLTR9A|LT	14960	NM_02351	69527	Mrps9	2310002AC
chr8	89411402	89411500	Intergenic	B1_Mur2|S	-2294	NM_03056	80750	N4bp1	AI481586|
chr1	1.68E+08	1.68E+08	Intergenic	Intergenic	-10041	NM_00108	68481	Mpzl1	1110007A1
chr10	69375327	69375400	intron (NM	intron (NM	-12917	NM_17068	11735	Ank3	2900054DC
chr9	1.08E+08	1.08E+08	promoter-T	promoter-T	-216	NM_00824	15235	Mst1	D3F15S2h|
chr8	13026702	13026875	intron (NM	intron (NM	754	NM_01017	14068	F7	AI132620|
chr7	71215927	71216250	Intergenic	L1_Mur3|L	-82633	NM_00103	17364	Trpm1	4732499L0
chr2	35083027	35083200	Intergenic	Intergenic	-26473	NM_02669	68365	Rab14	0610030G2
chr18	67930027	67930125	Intergenic	Intergenic	-4454	NM_02811	72124	Seh1l	2610007A1
chr13	67369827	67370050	promoter-T	promoter-T	66	NM_00100	238690	Zfp458	BC062958|
chr1	87488852	87489075	intron (NM	Lx7|LINE|L	6422	NM_03019	109032	Sp110	5031415CC
chr19	34708102	34708225	intron (NM	intron (NM	7070	NM_05321	112419	2010002M	AW412491
chr11	4342352	4342425	Intergenic	MTD|LTR|	-1303	NM_02945	75828	Hormad2	4930529M
chr10	1.16E+08	1.16E+08	intron (NR_	RLTR11B|L	19141	NM_00103	72068	Cnot2	2600016M
chr1	33586577	33586925	intron (NM	intron (NM	139888	NM_00892	19076	Prim2	AI323589
chr2	1.79E+08	1.79E+08	intron (NM	L1MB7|LIN	144755	NM_00986	12561	Cdh4	AW120700
chr14	73924777	73925000	exon (NM_	exon (NM_	15032	NM_02611	67381	Med4	2410046H1
chr2	1.63E+08	1.63E+08	promoter-T	promoter-T	-381	NM_02649	67996	Srsf6	1210001E1
chr16	16685502	16685675	Intergenic	L1_Mur3|L	-11516	NM_14633	18316	Olfr19	M12|MOR
chr11	96835452	96835575	intron (NM	intron (NM	3489	NM_03022	78912	Sp2	4930480I1
chr3	89223152	89223275	promoter-T	promoter-T	740	NM_01136	20416	Shc1	Shc|ShcA|
chr1	65136777	65137325	Intergenic	MER31B|L	-8187	NM_14476	12965	Crygb	Cryg-3|DG
chr17	80461127	80461450	exon (NM_	exon (NM_	320	NM_14480	72692	Hnrnpll	2510028H0
chr8	1.14E+08	1.14E+08	intron (NM	CpG	272	NM_01180	23837	Cfdp1	AA408409|
chr2	84722652	84723000	intron (NM	intron (NM	4023	NM_19922	269295	Rtn4rl2	Ngr2|Ngrh
chr6	49164127	49164375	intron (NM	CpG	702	NM_02367	140488	Igf2bp3	2610101N1
chr4	1.5E+08	1.5E+08	promoter-T	promoter-T	51	NM_13375	74155	Errfi1	1300002F1
chr17	32421427	32421600	promoter-T	promoter-T	-435	NM_02050	57261	Brd4	Brd5|HUNI
chr16	44172602	44172700	promoter-T	promoter-T	-859	NM_00102	207806	Gm608	5530400K2
chr12	70329377	70329500	promoter-T	promoter-T	-261	NM_01113	18974	Pole2	-
chr13	41048577	41048875	intron (NM	intron (NM	35723	NM_13321	14538	Gcnt2	5330430K1
chr9	1.1E+08	1.1E+08	TTS (NM_0	TTS (NM_0	-6725	NM_00108	235626	Setd2	4921524K1
chr11	58136502	58136675	promoter-T	promoter-T	27	NM_00125	319475	Zfp672	4930488PC
chr8	26708002	26708175	promoter-T	promoter-T	-129	NM_17301	270035	Letm2	6030453H1
chr1	54307777	54307850	intron (NM	intron (NM	286	NM_03002	78016	Ccdc150	4930511H1
chr11	96515277	96515375	intron (NM	intron (NM	123543	NM_02896	74479	Snx11	4933439F1
chr3	94217027	94217450	promoter-T	promoter-T	-2	NM_02830	72634	Tdrkh	2700091C2
chr7	1.21E+08	1.21E+08	intron (NM	intron (NM	96944	NM_02584	66922	Rras2	2610016H2
chr9	1.03E+08	1.03E+08	exon (NM_	exon (NM_	4416	NM_00108	28135	Cep63	4921501M

chr15	79571877	79572125	intron (NM	intron (NM	965	NM_19434	223697	Sun2	B230369L0
chr8	73988377	73988550	promoter-T	promoter-T	-362	NM_05316	94065	Mrpl34	0610007O1
chr10	79793202	79793300	intron (NM	intron (NM	361	NM_13929	70335	Reep6	0610011M
chr15	12034802	12034875	Intergenic	Intergenic	-12768	NM_01176	22763	Zfr	C920030H0
chr4	1.33E+08	1.33E+08	promoter-T	promoter-T	-83	NR_110978	52174	Tmem222	5730406H1
chr3	1.27E+08	1.27E+08	intron (NM	intron (NM	1057	NM_14513	211550	Tifa	T2bp
chr13	41169252	41169600	intron (NM	intron (NM	-3592	NM_00854	17152	Mak	A930010O0
chr17	49568402	49568700	intron (NM	intron (NM	862	NM_02004	56738	Mocs1	3110045D1
chr12	80294252	80294525	Intergenic	MURVY-int	-1582	NM_02155	17252	Rdh11	2610319N2
chr11	98769127	98769275	promoter-T	promoter-T	-2	NM_00102	23834	Cdc6	CDC18L
chr18	77304252	77304550	promoter-T	promoter-T	-18	NM_00116	17344	Pias2	6330408K1
chr11	87532927	87533300	intron (NM	B1_Mur1|S	-17954	NM_00929	20922	Supt4a	AL022777|
chr8	88468652	88469050	intron (NM	intron (NM	103950	NM_19944	102093	Phkb	AI463271
chr8	88131652	88131950	Intergenic	RMER2|LTI	-52631	NM_01979	56445	Dnaja2	1500017M
chr1	1.07E+08	1.07E+08	Intergenic	MT2A|LTR	185811	NM_17877	320311	Rnf152	A930029B0
chr7	38985227	38985500	Intergenic	Intergenic	16557	NM_02816	72244	1600014C1	AI428873
chr5	66466202	66466725	intron (NM	B3|SINE|B.	22955	NM_13906	245945	Rbm47	9530077J1
chr8	36439052	36439125	intron (NM	intron (NM	293	NM_17774	244416	Ppp1r3b	6430576E2
chr4	1.23E+08	1.23E+08	promoter-T	promoter-T	-7	NM_02966	76574	Mfsd2a	1700018O1
chr2	1.68E+08	1.68E+08	Intergenic	Intergenic	-98539	NM_01120	19246	Ptpn1	PTP-1B|PT
chr2	1.14E+08	1.14E+08	promoter-T	promoter-T	-45	NM_17546	228491	Zfp770	6430601A2
chr6	85865452	85865725	promoter-T	promoter-T	83	NM_02316	66116	Cml1	1110002I1
chr16	75855602	75856125	Intergenic	Intergenic	53648	NM_02338	67742	Samsn1	4930571B1
chr15	83425177	83425375	intron (NM	CpG	311	NM_18301	223723	Ttll12	BC055368|
chr9	15162352	15162500	promoter-T	promoter-T	-194	NM_17697	319675	5830418K0	5832426L2
chr13	93961952	93962175	5' UTR (NM	5' UTR (NM	175	NM_13390	100715	Papd4	8030446C2
chr5	31350677	31350875	promoter-T	promoter-T	159	NM_00117	330064	Slc5a6	E430023I2
chr6	6528777	6528850	promoter-T	promoter-T	-155	NM_00916	20422	Shfm1	DSS1|Shfd
chr5	68215002	68215400	intron (NM	intron (NM	23469	NM_00128	11980	Atp8a1	AI481521|
chr3	31324302	31324425	Intergenic	Intergenic	-115122	NM_17286	241919	Slc7a14	A930013N0
chr5	92706802	92707000	intron (NM	CpG	306	NM_00116	67111	Naaa	2210023K2
chr4	1.41E+08	1.41E+08	promoter-T	promoter-T	50	NM_00954	22642	Zbtb17	AA589413|
chr1	1.74E+08	1.74E+08	Intergenic	Intergenic	-7335	NM_02797	71870	Ccdc19	1700028D0
chr3	1.43E+08	1.43E+08	intron (NM	intron (NM	1080	NM_17865	109333	Pkn2	6030436C2
chr2	1.2E+08	1.2E+08	intron (NM	intron (NM	-12878	NM_00117	12335	Capn3	AI323605|
chr7	1.44E+08	1.44E+08	promoter-T	promoter-T	-368	NM_00859	17314	Mgmt	AGT|AI267
chr6	1.34E+08	1.34E+08	5' UTR (NM	5' UTR (NM	326	NM_00796	14011	Etv6	AW123102
chr2	37298102	37298175	promoter-T	promoter-T	503	NM_19902	320633	Zbtb26	A630026F2
chr12	79963127	79963425	promoter-T	promoter-T	217	NM_02611	13665	Eif2s1	0910001O2
chr2	80457152	80457225	promoter-T	promoter-T	-183	NM_02443	68082	Dusp19	5930436K2
chr19	8963327	8963450	promoter-T	promoter-T	15	NR_104416	66276	1810009A1	-
chr7	1.34E+08	1.34E+08	promoter-T	promoter-T	-423	NM_14620	233890	Zfp768	BC026432|
chr7	87679502	87679675	5' UTR (NM	5' UTR (NM	296	NM_00755	12144	Blm	-
chr11	22756727	22756875	intron (NM	intron (NM	2934	NM_01688	53625	B3gnt2	AA408337|
chr3	1.42E+08	1.42E+08	intron (NM	intron (NM	226	NM_14554	229900	Gbp7	9830147J2
chr10	29909902	29909975	Intergenic	Intergenic	10408	NM_00110	66311	Cenpw	2610036L1
chr1	1.73E+08	1.73E+08	Intergenic	Intergenic	-1124	NM_01018	14130	Fcgr2b	AI528646|

chr16	78579202	78579525	Intergenic	L1M5|LINE	-2430	NM_02596	67102	D16Ertd47	1700010I1
chr15	80873877	80874075	intron (NM	intron (NM	61501	NM_15304	223701	Mkl1	AMKL|Bsa
chr19	44371927	44372150	intron (NM	intron (NM	3872	NM_00912	20250	Scd2	Scd-2|swty
chr12	70464602	70464875	Intergenic	Intergenic	-6575	NM_02544	66244	Nemf	1500011I1
chr3	1.38E+08	1.38E+08	Intergenic	RSINE1|SIN	-1100	NM_01675	51788	H2afz	H2A.Z|H2a
chr9	1.16E+08	1.16E+08	intron (NM	intron (NM	83643	NM_02957	21813	Tgfbr2	1110020H1
chr3	50247402	50247700	promoter-T	promoter-T	-16	NM_01199	26570	Slc7a11	9930009M
chr16	20359827	20360000	intron (NM	intron (NM	-34436	NM_18315	224044	Cyp2ab1	EG224044|
chr1	1.79E+08	1.79E+08	promoter-T	promoter-T	-153	NM_02975	76816	Sdccag8	2700048G2
chr1	59970802	59971100	intron (NM	intron (NM	1101	NM_00103	72750	Fam117b	2810425F2
chr8	1.09E+08	1.09E+08	intron (NM	CpG	963	NM_00922	20650	Sntb2	Snt2
chr7	74873177	74873275	Intergenic	ORR1E|LTF	31402	NM_18331	233335	Synm	4930412K2
chr7	95459252	95459475	TTS (NM_0	TTS (NM_0	32760	NM_00998	13032	Ctsc	AI047818|
chr19	55213727	55214000	Intergenic	Intergenic	-39926	NM_00814	14732	Gpam	GPAT|GPA
chr11	88959877	88959950	Intergenic	Intergenic	24758	NM_00103	217071	Gm525	-
chr13	49292177	49292325	TTS (NM_0	TTS (NM_0	9335	NM_01361	18081	Ninj1	AU024536
chr8	60723752	60723825	intron (NM	intron (NM	455805	NM_00117	1E+08	Gm15881	OTTMUSG0
chr13	23802227	23802550	intron (NM	intron (NM	292	NM_01042	15216	Hfe	MR2
chr1	1.38E+08	1.38E+08	promoter-T	promoter-T	-332	NM_00108	67886	Camsap2	1600013L1
chr19	53969302	53969600	intron (NM	intron (NM	2730	NM_01105	18569	Pdcd4	D19Ucla1|
chr11	97691502	97691700	intron (NM	intron (NM	-10493	NM_00108	68127	B230217C1	AI840637
chr10	1.11E+08	1.11E+08	Intergenic	Intergenic	33521	NM_00934	21664	Phlda1	DT1P1B11|
chr9	26917327	26917500	intron (NM	intron (NM	45593	NM_02327	83964	Jam3	1110002N2
chr3	50652827	50652950	Intergenic	Intergenic	-375481	NM_00983	12457	Ccrn4l	AU043840
chr1	72301327	72301475	Intergenic	CpG	29487	NM_02352	111175	Pecr	2400003B1
chr10	5959127	5959250	intron (NM	CpG	755	NM_00103	381990	Zbtb2	Gm1103
chr4	1.34E+08	1.34E+08	Intergenic	Intergenic	-2939	NM_02366	27981	Rsrp1	2700043I2
chr18	65048002	65048125	intron (NM	CpG	653	NM_00111	83814	Nedd4l	1300012C0
chr16	17147077	17147350	exon (NM_	exon (NM_	153	NM_02694	69101	Ydjc	1810015A1
chr4	13054227	13054400	Intergenic	Intergenic	220329	NM_00117	208820	Triqk	C130086A1
chr1	1.73E+08	1.73E+08	intron (NM	intron (NM	79963	NM_17706	320078	Olfml2b	1110018N0
chr9	64642252	64642325	Intergenic	Intergenic	-16530	NM_00116	102442	Dennd4a	AI115600|
chr5	1.48E+08	1.48E+08	Intergenic	Intergenic	91044	NM_01166	22217	Usp12	Ubh1
chr11	87977952	87978100	intron (NM	intron (NM	-5041	NM_00117	103841	Cuedc1	AI841487|
chr7	16899027	16899200	exon (NM_	exon (NM_	4181	NM_13323	170770	Bbc3	PUMA|PU
chr11	95895602	95896250	promoter-T	promoter-T	-305	NM_03356	27681	Snf8	D11Moh34
chr13	43620252	43620500	Intergenic	Intergenic	35184	NM_00108	76137	Mcur1	6230416A0
chr5	1.23E+08	1.23E+08	intron (NM	intron (NM	2789	NM_00128	330188	Ccdc63	4921511C1
chr9	1.04E+08	1.04E+08	intron (NM	B1F|SINE|	488	NM_02569	66663	Uba5	5730525G1
chr5	76572702	76572850	intron (NM	B3|SINE|B	3478	NM_02061	57357	Srd5a3	1110025P1
chr5	1.36E+08	1.36E+08	exon (NM_	exon (NM_	165	NM_02145	58805	Mlxipl	ChREBP|W
chr3	1.33E+08	1.33E+08	promoter-T	promoter-T	503	NM_02623	67553	Gstcd	4933434L1
chr8	1.13E+08	1.13E+08	intron (NM	MER3|DNA	1263	NM_14621	234729	Vac14	AA959718|
chr16	45231602	45231700	intron (NM	intron (NM	7201	NM_17758	208154	Btla	A630002H2
chr2	1.57E+08	1.57E+08	promoter-T	promoter-T	100	NM_00104	228836	Dlgap4	AI225853|
chr13	21291777	21291975	Intergenic	Intergenic	20076	NM_00905	19720	Trim27	AW538890
chr3	51548802	51549350	intron (NM	intron (NM	83961	NM_17499	211666	Mgst2	GST2|MGS

chr13	46428727	46428975	Intergenic	Intergenic	59761	NM_00100	380842	Stmnd1	Gm1574
chr10	57909627	57909950	exon (NM_	exon (NM_	188	NM_01124	19386	Ranbp2	A430087B0
chr3	53667377	53667700	exon (NM_	exon (NM_	191	NM_02643	67890	Ufm1	1810045K1
chr2	18860577	18860875	intron (NM	intron (NM	59022	NM_00884	18718	Pip4k2a	AW742916
chr17	13200677	13200975	5' UTR (NM	5' UTR (NM	121	NM_01367	20656	Sod2	MnSOD|Sc
chr16	57167102	57167275	intron (NM	CpG	257	NM_02317	52633	Nit2	1190017B1
chr8	87417352	87417425	intron (NM	intron (NM	432	NM_00104	270076	Gcdh	9030411L1
chr7	1.19E+08	1.19E+08	intron (NM	intron (NM	171878	NM_17373	233733	Galnt18	2900011G2
chrX	75989702	75989775	Intergenic	RMER16-in	6636	NM_00116	70864	Fam47c	4921509A1
chr9	37020852	37021225	intron (NM	L1M5|LINE	5120	NM_02546	66279	Tmem218	1810021J1
chr15	75911077	75911200	intron (NM	CpG	162	NM_00116	67959	Puf60	2410104I1
chr3	65331827	65331975	promoter-T	promoter-T	-468	NM_17889	99929	Tiparp	ARTD14|A\
chr2	1.57E+08	1.57E+08	intron (NM	CT-rich|Lo	-21118	NM_01885	56045	Samhd1	E330031J0
chr11	76758877	76759075	promoter-T	promoter-T	-182	NM_17864	104184	Blmh	AI035728|
chr19	4755977	4756125	promoter-T	promoter-T	-474	NM_02571	66704	Rbm4b	4921506I2
chr12	1.04E+08	1.04E+08	intron (NM	intron (NM	72575	NM_00116	217835	Rin3	6430500K0
chr8	96738552	96738875	intron (NM	CpG	212	NM_17241	71805	Nup93	2410008G0
chr12	1.14E+08	1.14E+08	promoter-T	promoter-T	65	NM_14545	217887	BC022687	AW050009
chr7	1.01E+08	1.01E+08	Intergenic	Intergenic	536974	NM_02142	58238	Fam181b	A830059I2
chr6	92051127	92051275	intron (NM	intron (NM	9789	NM_01163	22026	Nr2c2	TAK1|Tr4|
chr5	1.45E+08	1.45E+08	intron (NM	intron (NM	769	NM_01877	55950	Bri3	I3
chr2	92274652	92274775	promoter-T	promoter-T	-487	NM_00996	12953	Cry2	AV006279|
chr3	1.08E+08	1.08E+08	exon (NM_	exon (NM_	182	NM_01030	14679	Gnai3	AI158965|
chr12	86337677	86337775	exon (NM_	exon (NM_	455	NM_17836	56531	Ylpm1	A930013E1
chr9	69560852	69561250	Intergenic	Intergenic	47696	NM_02237	64290	Foxb1	C43|Fkh5|
chr4	9037477	9037875	Intergenic	L1MB2|LIN	-158788	NM_02894	74438	Clvs1	4933402J2
chr2	61133752	61134050	Intergenic	Intergenic	-282742	NM_01152	21353	Tank	C86182|E4
chr11	66866077	66866225	promoter-T	promoter-T	-21	NM_00104	52892	Sco1	2610001C0
chr1	97425377	97425550	Intergenic	Intergenic	138708	NM_00918	20452	St8sia4	PST|PST-1|
chr9	91482202	91482400	Intergenic	Intergenic	218491	NM_00957	22774	Zic4	-
chr3	88913852	88914100	Intergenic	RLTR24|LT	-8087	NM_00125	110196	Fdps	6030492I1
chr14	56353752	56354000	Intergenic	MIRb|SINE	10645	NM_02681	52585	Dhrs1	1110029G0
chr2	1.48E+08	1.48E+08	Intergenic	RCHARR1|	-48642	NM_01074	17064	Cd93	6030404G0
chr12	86830252	86830475	Intergenic	Intergenic	15512	NM_01023	14281	Fos	D12Rfj1|c-
chr5	3355702	3355900	intron (NM	intron (NM	11489	NM_00987	12571	Cdk6	5830411I2
chr5	1.05E+08	1.05E+08	5' UTR (NM	5' UTR (NM	175	NM_00886	18764	Pkd2	C030034P1
chr13	20886052	20886250	5' UTR (NM	5' UTR (NM	169	NM_01205	27052	Aoah	4930433E1
chr2	64939427	64939950	intron (NM	intron (NM	-78865	NM_01671	50915	Grb14	AI505286
chr3	94641177	94641600	promoter-T	promoter-T	-101	NM_00116	229584	Pogz	9530006B0
chrX	1.1E+08	1.1E+08	intron (NM	Lx6|LINE|L	42597	NM_18157	69693	Pof1b	2310066B1
chr18	5334402	5334675	promoter-T	promoter-T	-101	NM_17872	240186	Zfp438	9430031G0
chr9	95998477	95998575	Intergenic	RMER16-in	-21322	NM_17735	235533	Gk5	AV095337|
chrX	83042802	83043125	Intergenic	Intergenic	-20805	NM_00129	14933	Gyk	D930012N1
chr7	28834877	28835025	intron (NM	intron (NM	160	NM_01187	23996	Psmc4	CIP21|MIP
chr15	82183927	82184050	intron (NM	intron (NM	733	NM_02598	67130	Ndufa6	14kDa|270
chr18	47457602	47457775	intron (NM	intron (NM	70834	NM_01874	20358	Sema6a	9330158E0
chr11	69499052	69499250	promoter-T	promoter-T	-95	NM_02351	69583	Tnfsf13	2310026N0

chr10	44421927	44422200	Intergenic	Intergenic	-243570	NM_00754	12142	Prdm1	Blimp-1|Bl
chr19	56609552	56609900	intron (NM	L1MB7|LIN	12986	NM_01883	55947	Dclre1a	2810043H1
chr18	31769052	31769450	exon (NM_	exon (NM_	305	NM_02616	67453	Slc25a46	1200007B0
chr18	55233202	55233350	Intergenic	Intergenic	-83442	NM_17575	269023	Zfp608	4932417D1
chr4	98860727	98861000	promoter-T	promoter-T	25	NM_00114	242557	Atg4c	Apg4-C|Ap
chr11	1.21E+08	1.21E+08	intron (NM	CpG	132	NM_00103	80879	Slc16a3	Mct3|Mct4
chr4	1.47E+08	1.47E+08	promoter-T	promoter-T	-229	NM_19861	242747	Zfp933	2810408P1
chr3	33722252	33722350	intron (NM	MIRb|SINE	20931	NM_02622	51938	Ccdc39	4921507O1
chr2	27370252	27370600	promoter-T	promoter-T	-241	NM_08084	140858	Wdr5	2410008O0
chr17	25970327	25970425	promoter-T	promoter-T	-70	NM_01971	56424	Stub1	0610033N2
chr4	1.02E+08	1.02E+08	Intergenic	Intergenic	-77927	NM_00128	73094	Sgip1	3110007P0
chr1	37917402	37917500	intron (NM	ORR1B2|LT	4476	NM_20722	211484	Tsga10	4933432N2
chr7	1.26E+08	1.26E+08	intron (NM	intron (NM	2564	NM_01958	56209	Gde1	1200003M
chr7	52253277	52253525	promoter-T	promoter-T	383	NM_01684	54131	Irf3	C920001K0
chr19	40969402	40969475	TTS (NR_10	TTS (NR_10	243	NM_02831	72672	Zfp518a	2810401C2
chr6	90964377	90964550	exon (NM_	exon (NM_	102357	NM_01881	54563	Nup210	9830001L1
chr6	23161752	23161850	Intergenic	Intergenic	36463	NM_02846	73191	Fezf1	3110069A1
chr19	29085927	29086125	intron (NM	intron (NM	21042	NM_02595	67072	Cdc37l1	2700033A1
chr2	1.58E+08	1.58E+08	intron (NM	intron (NM	424	NM_17785	329547	Bpi	9230105K1
chr8	1.23E+08	1.23E+08	intron (NM	L2b|LINE|L	53218	NM_03020	78892	Crispld2	1810049K2
chr15	93106102	93106350	promoter-T	promoter-T	-711	NM_00103	223827	Gxylt1	Glt8d3|Gm
chr17	12962652	12962775	promoter-T	promoter-T	-141	NM_01051	16004	Igf2r	AI661837|
chr12	1.01E+08	1.01E+08	Intergenic	Intergenic	-8963	NM_18315	217827	Nrde2	6720454P0
chr1	37618777	37618975	Intergenic	Intergenic	-21015	NM_17387	269181	Mgat4a	9530018I0
chr13	47109777	47110100	exon (NM_	exon (NM_	281	NM_17534	105193	Nhlrc1	AI505271|
chr1	1.69E+08	1.69E+08	promoter-T	promoter-T	-330	NM_03072	80914	Uck2	AA407809|
chr9	71981052	71981125	Intergenic	CpG	-21462	NM_00125	21406	Tcf12	A130037E0
chr6	86354952	86355100	intron (NM	intron (NM	813	NM_00116	21841	Tia1	2310050N0
chr3	35831877	35832350	promoter-T	promoter-T	-225	NM_00120	114893	Dcun1d1	Rp42|SCCR
chr6	57484552	57484700	intron (NM	intron (NM	794	NM_17552	243382	Ppm1k	2900063A1
chr1	1.2E+08	1.2E+08	Intergenic	Intergenic	-8672	NM_00129	76707	Clasp1	1700030C2
chr11	33062502	33062600	intron (NM	CpG	655	NM_00125	18148	Npm1	B23|NO38
chr8	90886277	90886575	promoter-T	promoter-T	-336	NM_01204	26992	Brd7	BP75|CELT
chr8	85297877	85297975	promoter-T	promoter-T	99	NM_14560	30932	Zfp330	BC008086|
chr18	34919827	34919925	intron (NM	intron (NM	1316	NM_17510	66306	Fam53c	2810012G0
chr15	99217527	99217625	Intergenic	ORR1E-int|	-5802	NM_02666	110213	Tmbim6	5031406P0
chr14	18712252	18712350	intron (NM	intron (NM	-101857	NM_00938	21834	Thrb	Nr1a2|T3R
chr1	1.72E+08	1.72E+08	Intergenic	RMER17C|	-22179	NM_01200	26904	Sh2d1b1	EAT-2|EAT
chr15	78707702	78708175	5' UTR (NM	5' UTR (NM	318	NM_14592	106039	Gga1	4930406E1
chr2	68220852	68221000	intron (NM	intron (NM	89112	NM_01686	53416	Stk39	AW227544
chr4	1.05E+08	1.05E+08	Intergenic	Intergenic	548936	NM_08055	67916	Ppap2b	1110003O2
chr2	1.66E+08	1.66E+08	Intergenic	Intergenic	-15925	NM_00125	228880	Zmynd8	1110013E2
chr1	1.57E+08	1.57E+08	intron (NM	intron (NM	32061	NM_01127	19775	Xpr1	Rmc-1|Rm
chr3	15332102	15332500	promoter-T	promoter-T	1	NM_00107	751864	Gm9733	-
chr19	44220402	44220775	intron (NM	intron (NM	348	NM_02860	73689	Bloc1s2	2410089B1
chr3	95181177	95181300	Intergenic	RMER16-in	-20114	NM_00116	84505	Setdb1	AU022152
chr18	67438477	67438550	Intergenic	Intergenic	-10364	NM_05326	114663	Impa2	2210415D2

chr7	16632552	16632650	Intergenic	Intergenic	-24690	NM_00109	666528	Zfp541	EG666528|
chr8	48799102	48799175	5' UTR (NM	5' UTR (NM	147	NM_17240	70925	Cdkn2aip	4921511I1(
chr16	20717402	20717575	promoter-T	promoter-T	-411	NM_14563	245841	Polr2h	-
chr10	82447802	82447900	promoter-T	promoter-T	-391	NM_02143	58250	Chst11	1110020PC
chr15	31286752	31286975	intron (NM	intron (NM	10651	NM_00116	67434	Ankrd33b	0610012AC
chr15	76437577	76437675	intron (NM	intron (NM	395	NM_05319	94230	Cpsf1	-
chr3	1.16E+08	1.16E+08	promoter-T	promoter-T	200	NM_02719	69740	Dph5	2410012M
chr2	1.28E+08	1.28E+08	Intergenic	Intergenic	121740	NM_00128	12125	Bcl2l11	1500006F2
chr5	65492977	65493100	Intergenic	Intergenic	-29432	NM_17517	71778	Klhl5	1300013C1
chr5	1.19E+08	1.19E+08	intron (NM	intron (NM	14977	NM_00108	76792	2410131K1	-
chr1	1.73E+08	1.73E+08	intron (NM	ID_B1|SINE	296	NM_01018	14131	Fcgr3	CD16
chr8	1.17E+08	1.17E+08	intron (NM	intron (NM	642	NM_00100	403395	Clec3a	1110019O1
chr5	5420077	5420250	Intergenic	Intergenic	-39912	NM_01107	18647	Cdk14	Pftk1|mKIA
chr9	72878302	72878550	intron (NM	ID_B1|SINE	9080	NM_01888	55981	Pigb	Pig-b
chr4	71710652	71710750	Intergenic	Intergenic	151226	NM_01159	21885	Tle1	C230057CC
chr7	1.47E+08	1.47E+08	Intergenic	Intergenic	-6005	NR_027857	14912	Nkx6-2	Gtx|Nkx6.2
chr6	30459977	30460100	promoter-T	promoter-T	189	NM_02707	75647	Ssmem1	1700016KC
chr1	88600127	88600850	intron (NM	CpG	109	NM_00117	208718	Dis3l2	4930429A2
chr19	37624927	37625075	promoter-T	promoter-T	93	NM_17535	107371	Exoc6	4833405E0
chr17	64958427	64958575	intron (NM	intron (NM	7512	NM_00854	17158	Man2a1	Mana-2|M
chr9	16732427	16732800	Intergenic	Intergenic	-549938	NM_00108	270120	Fat3	9430076AC
chr9	86916752	86917075	promoter-T	promoter-T	49	NM_02419	266690	Cyb5r4	2810034J1
chr6	1.19E+08	1.19E+08	intron (NM	intron (NM	21388	NM_19798	68465	Adipor2	1110001I1
chr12	60167927	60168125	exon (NM_	exon (NM_	120	NM_00889	18949	Pnn	AU045199
chr11	29593502	29593725	promoter-T	promoter-T	-161	NM_19405	68585	Rtn4	1110020G1
chr5	92471852	92471925	promoter-T	promoter-T	-166	NM_00127	53886	Cdkl2	5330436L2
chr12	1.04E+08	1.04E+08	Intergenic	MER58A|D	11697	NM_00103	75483	Cox8c	1700007F2
chr5	3905327	3905475	Intergenic	Intergenic	-11468	NM_17213	545725	Mterf1a	4931431L1
chr18	24070577	24070775	Intergenic	Intergenic	-42513	NM_02700	69256	Zfp397	2810411K1
chr5	34125227	34125300	promoter-T	promoter-T	90	NM_01969	56384	Letm1	-
chr3	79372102	79372350	promoter-T	promoter-T	-625	NM_00116	329679	Fnip2	D630023B1
chr7	73985177	73985425	exon (NM_	exon (NM_	680	NM_00103	233332	Adamts17	AU023434
chr7	35019877	35019950	Intergenic	URR1B|DN	-4558	NM_00815	14751	Gpi1	Amf|Gpi|G
chr5	1.31E+08	1.31E+08	5' UTR (NM	5' UTR (NM	248	NM_00113	22021	Tpst1	R75054|Ta
chr5	1.24E+08	1.24E+08	5' UTR (NM	5' UTR (NM	181	NM_02985	77045	Bcl7a	4432415N(
chr4	1.32E+08	1.32E+08	promoter-T	promoter-T	-240	NM_13388	100226	Stx12	AI850350|
chr1	1.81E+08	1.81E+08	intron (NM	intron (NM	112057	NM_00116	269152	Kif26b	4832420M
chr4	1.49E+08	1.49E+08	Intergenic	LTR79|LTR	-7022	NM_00108	110208	Pgd	0610042AC
chr4	1.33E+08	1.33E+08	Intergenic	Intergenic	-3587	NM_00108	93760	Arid1a	1110030E0
chr7	1.09E+08	1.09E+08	promoter-T	promoter-T	-431	NM_01956	56212	Rhog	2810426GC
chr13	42149627	42149900	intron (NM	intron (NM	2373	NM_00777	110521	Hivep1	Cryabp1
chr11	76272402	76272550	intron (NM	intron (NM	9491	NM_00129	109934	Abr	-
chr8	1.11E+08	1.11E+08	Intergenic	Intergenic	-51743	NM_00749	11906	Zfhx3	A230102L0
chr8	1.08E+08	1.08E+08	promoter-T	promoter-T	205	NM_19829	102124	Enkd1	AI606951|
chr14	31271152	31271250	intron (NM	L2a|LINE|L	-21550	NM_13376	75901	Dcp1a	1110066A2
chr3	1.3E+08	1.3E+08	5' UTR (NM	5' UTR (NM	171	NM_00119	619547	Rpl34-ps1	EG619547|
chr9	99469477	99469600	promoter-T	promoter-T	-220	NM_02876	74125	Armc8	1200015K2

chr11	1.07E+08	1.07E+08	intron (NM	intron (NM	1871	NM_14582	71795	Pitpnc1	1110020BC
chr14	35487152	35487375	5' UTR (NM	5' UTR (NM	149	NM_00130	218914	Wapal	A530089A2
chr7	1.13E+08	1.13E+08	intron (NM	CpG	288	NM_02530	27397	Mrpl17	MRP-L26|F
chr1	36996102	36996250	exon (NM_	exon (NM_	196	NM_01887	56030	Tmem131	2610524EC
chr17	34973502	34973575	promoter-T	promoter-T	310	NM_01944	54402	Stk19	G11|RP1
chr5	1.23E+08	1.23E+08	intron (NM	B1F2|SINE	1943	NM_17542	109305	Orai1	D730049H(
chr1	55188302	55188375	intron (NM	CpG	249	NM_02528	19070	Mob4	2610109B1
chr7	1.49E+08	1.49E+08	intron (NM	intron (NM	964	NM_00116	66853	Pnpla2	0610039C2
chr15	77799927	77801875	intron (NM	CpG	353	NM_01874	55944	Eif3d	66/67kDa|
chr11	9091727	9091975	promoter-T	promoter-T	-94	NM_17825	268379	Abca13	9830132L2
chr4	1.09E+08	1.09E+08	promoter-T	promoter-T	-474	NM_02745	70533	Btf3l4	4632412EC
chr8	18842577	18842775	Intergenic	Charlie1|D	-3603	NM_02679	52123	Agpat5	1110013AC
chr3	51322552	51322825	3' UTR (NM	3' UTR (NM	34800	NM_01685	19338	Rab33b	-
chr14	69605677	69605950	Intergenic	Intergenic	-41533	NM_00928	20855	Stc1	Stc
chr4	66482902	66483225	Intergenic	Intergenic	-5782	NM_02129	21898	Tlr4	Lps|Ly87|F
chr2	1.19E+08	1.19E+08	promoter-T	promoter-T	-161	NM_02657	68142	Ino80	2310079N1
chr17	71094052	71094150	intron (NM	intron (NM	102016	NM_00116	21815	Tgif1	AA959811|
chr9	1.14E+08	1.14E+08	intron (NM	intron (NM	-35889	NM_00108	22221	Ubp1	Cp2b|LBP-
chr15	1E+08	1E+08	Intergenic	Lx8|LINE|L	-7577	NM_13409	68614	Letmd1	1110019O1
chr11	83727677	83727775	Intergenic	Intergenic	-27866	NM_03009	78394	Ddx52	2700029CC
chr5	1.23E+08	1.23E+08	non-coding	non-coding	121	NM_00111	11938	Atp2a2	9530097L1
chr12	98395302	98395450	Intergenic	Intergenic	1102171	NM_00807	14420	Galc	2310068BC
chr5	97347002	97347125	Intergenic	Intergenic	-79645	NM_08070	140780	Bmp2k	4933417M
chr15	79239627	79239850	Intergenic	MLT1K|LTF	-6005	NM_17260	223693	Tmem184b	2610507A1
chr13	97754027	97754175	Intergenic	Intergenic	50216	NM_02558	66479	1700029F1	-
chr10	80783927	80784225	exon (NM_	exon (NM_	228	NM_02738	70312	Cactin	2510012J0
chr16	11254077	11254300	TTS (NR_03	TTS (NR_03	230	NM_00113	14852	Gspt1	AI314175|/
chr16	4880627	4880700	promoter-T	promoter-T	-812	NM_14535	207740	Ubald1	1500031H(
chr19	33297002	33297200	intron (NM	intron (NM	169684	NM_00116	67795	Rnls	6530404N2
chr11	1E+08	1E+08	exon (NM_	exon (NM_	631	NM_01979	56354	Dnajc7	2010003F2
chr1	1.36E+08	1.36E+08	Intergenic	CpG	-3604	NM_15289	75605	Kdm5b	2010009J1
chr1	1.58E+08	1.58E+08	intron (NM	intron (NM	1108	NM_00113	11352	Abl2	AA536808|
chr9	50287252	50287350	Intergenic	Intergenic	-15329	NM_02963	76509	Plet1	0610037B2
chr7	30857352	30857500	intron (NM	intron (NM	416	NM_17788	330502	Zfp82	A030010D(
chr1	1.58E+08	1.58E+08	intron (NM	intron (NM	16941	NM_02326	104009	Qsox1	1300003H(
chr6	1.38E+08	1.38E+08	promoter-T	promoter-T	-420	NM_01994	56615	Mgst1	1500002K1
chr5	53845552	53845700	Intergenic	Intergenic	-101392	NM_00108	19664	Rbpj	AI843960|(
chr8	87514577	87514650	exon (NM_	exon (NM_	119	NM_00116	170833	Hook2	A630054I0
chr1	1.88E+08	1.88E+08	intron (NM	CpG	176	NM_14610	226791	Lyplal1	BC027340|
chr2	1.64E+08	1.64E+08	5' UTR (NM	5' UTR (NM	105	NM_01875	54401	Ywhab	1300003C1
chr9	1.22E+08	1.22E+08	Intergenic	Lx8|LINE|L	-9065	NM_01001	13124	Cyp8b1	-
chr2	1.27E+08	1.27E+08	intron (NM	intron (NM	720	NM_02322	66552	Sppl2a	2010106G(
chr11	97677052	97677225	intron (NM	intron (NM	16152	NM_01068	16796	Lasp1	AA408629|
chr16	38433102	38433325	promoter-T	promoter-T	12	NM_13410	85031	Pla1a	AA986889|
chr10	75700777	75700975	promoter-T	promoter-T	-266	NM_13318	15468	Prmt2	AI504737|I
chr9	59135952	59136075	Intergenic	Intergenic	-3366	NM_02812	72141	Adpgk	2610017G(
chr4	1.09E+08	1.09E+08	promoter-T	promoter-T	485	NM_00130	12580	Cdkn2c	C77269|IN

chr11	82896602	82896975	Intergenic	Intergenic	18174	NM_01140	20556	Slfn2	Shlf2
chr2	35780477	35780675	intron (NM	intron (NM	54568	NM_02977	74410	Ttll11	4932702F0
chr1	39635252	39635650	Intergenic	Intergenic	-1259	NM_00103	67702	Rnf149	1600023E1
chr3	1.58E+08	1.58E+08	promoter-T	promoter-T	-417	NM_00101	433667	Ankrd13c	AI505652|/
chr1	1.87E+08	1.87E+08	promoter-T	promoter-T	15	NM_14551	226778	Mark1	AW491150
chr16	25805052	25805175	intron (NM	intron (NM	3111	NM_00112	22061	Trp63	AI462811|
chr2	1.22E+08	1.22E+08	exon (NM_	exon (NM_	140	NM_00103	214616	Spata5l1	AV141009|
chr1	80753727	80753800	intron (NM	intron (NM	1365	NM_17529	210293	Dock10	9330153B1
chr3	67371127	67371200	Intergenic	Intergenic	-15527	NM_02581	66868	Mfsd1	1200003O(
chr13	17787202	17787275	promoter-T	promoter-T	-9	NM_02547	66308	Mplkip	2810021B0
chr11	7662627	7662825	Intergenic	MTEa-int|L	-548800	NM_00834	16009	Igfbp3	AI649005|
chr1	72264852	72265175	Intergenic	Intergenic	-6132	NM_00100	381269	Mreg	Gm974|W
chr16	18655227	18655900	Intergenic	Intergenic	-25532	NM_21361	18951	5-Sep	Cdcrel-1|C
chr8	82848702	82848800	Intergenic	Intergenic	-169193	NM_01036	14934	Gypa	AI853584|
chr11	1.19E+08	1.19E+08	Intergenic	Intergenic	-1225	NM_00762	12418	Cbx4	MPc2|PC2
chr15	27911927	27912075	intron (NM	intron (NM	43602	NM_00108	223435	Trio	6720464I0
chr14	65999727	66000050	intron (NM	intron (NM	22375	NM_19902	380912	Zfp395	BC053701|
chr6	1.16E+08	1.16E+08	intron (NM	intron (NM	33155	NM_03117	81896	Ift122	C86139|W
chr3	8619452	8619575	Intergenic	Intergenic	47525	NM_01042	15213	Hey1	AI316788|/
chr12	60141977	60142075	Intergenic	Intergenic	20422	NM_03005	78232	Trappc6b	5830498C1
chr12	56060602	56060900	intron (NM	L1MB4|LIN	26572	NM_01381	217578	Baz1a	Acf1|B930
chr11	51775352	51775475	Intergenic	Intergenic	-5720	NM_02997	52626	Cdkn2aipnl	A430101B0
chr13	95066152	95066225	Intergenic	Intergenic	-10952	NM_02915	107767	Scamp1	4930505M
chr3	87862352	87862675	promoter-T	promoter-T	-96	NM_14489	246703	Apoa1bp	AA087124|
chr8	11276327	11276450	intron (NM	intron (NM	36438	NM_00993	12826	Col4a1	Bru|Col4a-
chr8	74066902	74067050	intron (NM	CpG	147	NM_02861	73711	Mvb12a	1110012M
chr2	31829977	31830225	exon (NM_	exon (NM_	131	NM_17226	227720	Nup214	BC039282|
chr1	1.55E+08	1.55E+08	Intergenic	Intergenic	20654	NM_02636	67771	Arpc5	5830443F1
chr4	97855802	97855950	Intergenic	Intergenic	194080	NM_05315	94043	Tm2d1	2310026L1
chr6	1.17E+08	1.17E+08	Intergenic	Intergenic	7169	NM_02605	67255	Zfp422	2900028O2
chr6	17644627	17644825	intron (NM	CpG	732	NM_00128	64213	St7	9430001H(
chr2	68817052	68817275	intron (NM	intron (NM	117549	NM_17285	241447	Cers6	4732462C0
chr1	1.72E+08	1.72E+08	Intergenic	Intergenic	-51143	NM_02328	66977	Nuf2	2410003C0
chr15	50790702	50790950	Intergenic	MLT1G3|LT	-69239	NM_03200	83925	Trps1	AI115454|/
chr5	1.15E+08	1.15E+08	intron (NM	intron (NM	3602	NM_02671	68420	Ankrd13a	1100001D1
chr3	1.44E+08	1.44E+08	intron (NM	intron (NM	1897	NM_05310	93684	15-Sep	9430015P0
chr18	55569027	55569375	Intergenic	Intergenic	-419367	NM_17575	269023	Zfp608	4932417D1
chr6	47870777	47871000	intron (NM	CpG	321	NM_00114	232784	Zfp212	Znf212|mK
chr19	46432477	46432625	intron (NM	intron (NM	1181	NM_02918	75146	Tmem180	4930449A0
chr9	63225602	63225700	promoter-T	promoter-T	8	NM_01184	23938	Map2k5	AI324775|/
chrX	67718852	67718950	intron (NM	intron (NM	11317	NM_00103	236848	BC023829	Tmem185a
chr1	1.66E+08	1.66E+08	intron (NM	intron (NM	4398	NM_00972	11931	Atp1b1	Atp4b|Atp
chr13	41664377	41664725	Intergenic	Intergenic	-37034	NM_00116	621976	Tmem170b	EG621976
chr4	19841052	19841600	intron (NM	intron (NM	8387	NM_17540	242341	Atp6v0d2	1620401A0
chr6	4928402	4928600	intron (NM	intron (NM	75181	NM_18159	243725	Ppp1r9a	2810430P2
chr1	1.88E+08	1.88E+08	Intergenic	Intergenic	208208	NM_00936	21808	Tgfb2	BB105277|
chr1	6959977	6960275	Intergenic	B4A|SINE|	-118875	NM_18302	319263	Pcmtd1	8430411F1

chr7	75294627	75294725	intron (NM	intron (NM	114443	NM_03010	78444	Pgpep1l	C330024D1
chr11	95274877	95275275	promoter-T	promoter-T	-321	NM_02528	20747	Spop	AI315626|I
chr2	51893402	51893750	promoter-T	promoter-T	-57	NM_00939	21930	Tnfaip6	TSG-6|Tnfi
chr19	41421552	41421650	intron (NM	MIRb|SINE	37959	NM_03137	83490	Pik3ap1	1810044J0
chr12	1.04E+08	1.04E+08	promoter-T	promoter-T	632	NM_00114	1.01E+08	Gm20604	AK010878-
chr4	86336127	86336500	Intergenic	Intergenic	-20350	NM_00740	11520	Plin2	AA407157|
chr1	1.54E+08	1.54E+08	exon (NM_	exon (NM_	211	NM_02567	66637	Tsen15	5730449L1
chr15	19787402	19787475	Intergenic	Intergenic	809067	NM_02281	64833	Acot10	Acate3|MT
chr14	21731052	21731225	Intergenic	RMER15|LT	-17517	NM_00950	22330	Vcl	9430097D2
chr8	1.25E+08	1.25E+08	5' UTR (NM	5' UTR (NM	147	NM_00102	76014	Zc3h18	1190001B2
chr7	77789177	77789300	Intergenic	Intergenic	-277606	NM_18326	11819	Nr2f2	2700033K0
chr11	86359152	86359250	promoter-T	promoter-T	708	NM_00119	56427	Tubd1	4930550G1
chr2	60722252	60722400	intron (NM	MLT1M|LT	-2831	NM_00114	56878	Rbms1	2600014B1
chr12	21423702	21423850	promoter-T	promoter-T	-479	NM_01173	22630	Ywhaq	2700028P0
chr9	96096927	96097125	intron (NM	CpG	332	NM_00118	211586	Tfdp2	1110029I05
chr5	1E+08	1E+08	intron (NM	CpG	540	NM_01669	50926	Hnrnpdl	AA407431|
chr12	71815127	71815250	Intergenic	Intergenic	-111313	NM_02812	319710	Frmd6	2610019M
chr11	60624552	60624625	5' UTR (NM	5' UTR (NM	179	NM_00917	20425	Shmt1	AI324848|A
chr4	1.29E+08	1.29E+08	intron (NM	CpG	701	NM_01080	17357	Marcksl1	AL022768|
chr5	73578402	73578500	intron (NM	intron (NM	69406	NM_02819	72313	Fryl	2010313D2
chr4	1.35E+08	1.35E+08	Intergenic	Intergenic	68937	NM_01388	29876	Clic4	D0Jmb3|TU
chr11	82577852	82578025	promoter-T	promoter-T	91	NM_02588	66983	Zfp830	2410003C2
chr17	34324752	34324925	promoter-T	promoter-T	337	NM_01368	21354	Tap1	ABC17|APT
chr6	51738227	51738550	Intergenic	Intergenic	224160	NM_01877	54353	Skap2	2610021A1
chr16	18877927	18878000	intron (NM	intron (NM	1120	NM_01043	15260	Hira	AA138857|
chr15	25870202	25870450	promoter-T	promoter-T	-131	NM_00127	66270	Fam134b	1810015C0
chr4	1.26E+08	1.26E+08	promoter-T	promoter-T	-11	NM_01197	26445	Psmb2	AU045357
chr1	1.35E+08	1.35E+08	promoter-T	promoter-T	87	NM_00857	17248	Mdm4	4933417N0
chr10	95964777	95965100	Intergenic	RMER15|LT	-114697	NM_00756	12226	Btg1	AI426953|A
chr12	86679952	86680025	exon (NM_	exon (NM_	324	NM_14513	217718	Nek9	C130021H0
chr9	96918827	96919225	promoter-T	promoter-T	-252	NM_14513	211949	Spsb4	D030068E1
chr2	48392527	48392750	Intergenic	MLT1J|LTR	-276991	NM_00739	11480	Acvr2a	ActrIIa|Acv
chr17	43976077	43976200	intron (NM	intron (NM	35186	NM_00128	53901	Rcan2	Csp2|Dscr1
chr8	73132977	73133350	promoter-T	promoter-T	-950	NM_13377	76900	Ssbp4	1210002E1
chr4	1.29E+08	1.29E+08	Intergenic	L3|LINE|CR	-22989	NM_02921	75234	Rnf19b	4930534K1
chr4	1.52E+08	1.52E+08	Intergenic	CpG	-1585	NM_20768	56226	Espn	je
chr7	79372427	79372500	intron (NM	intron (NM	79018	NM_00102	244049	Mctp2	Gm489
chr18	62340177	62340275	promoter-T	promoter-T	-591	NM_00742	11555	Adrb2	Adrb-2|Bad
chr1	1.84E+08	1.84E+08	Intergenic	Intergenic	-36392	NM_01205	27058	Srp9	9kDa
chr12	1.19E+08	1.19E+08	Intergenic	MIRm|SINE	-85439	NM_17593	217944	Rapgef5	4932413M
chr13	52768702	52768850	Intergenic	Intergenic	76525	NM_01151	20963	Syk	Sykb
chr4	41221802	41222050	intron (NM	intron (NM	242	NM_02687	68926	Ubap2	1190005K0
chrX	45780252	45780325	intron (NM	intron (NM	36021	NM_01968	56501	Elf4	AV314029|
chr2	1.57E+08	1.57E+08	exon (NM_	exon (NM_	172	NM_00103	629499	Mroh8	4922505G1
chr17	46692752	46693050	5' UTR (NM	5' UTR (NM	210	NM_02049	20807	Srf	AW049942
chr15	78992852	78993050	intron (NM	intron (NM	1969	NM_01143	20665	Sox10	Dom|Sox21
chr14	64162527	64162725	5' UTR (NM	5' UTR (NM	164	NM_17759	210376	Mtmr9	9430075G1

chr11	72990177	72990425	promoter-T	promoter-T	209	NM_00116	66048	Emc6	0610009E2
chr10	1.27E+08	1.27E+08	5' UTR (NM	5' UTR (NM	121	NM_00928	20852	Stat6	-
chrX	10816227	10816325	Intergenic	MIR|SINE|	-60107	NM_00111	1E+08	Gm14483	OTTMUSG(
chr7	91033052	91033300	promoter-T	promoter-T	-325	NM_03070	80889	Mesdc1	AW061151
chr5	1.21E+08	1.21E+08	intron (NM	intron (NM	29696	NM_14585	252972	Tpcn1	5730403B0
chr3	1.04E+08	1.04E+08	promoter-T	promoter-T	43	NM_00116	18685	Phtf1	AU041898
chr19	43598577	43598700	intron (NM	MIRc|SINE	457	NM_01032	14718	Got1	AI789014|(
chr18	9734877	9735050	intron (NM	intron (NM	27317	NM_13044	140792	Colec12	CL-P1|SRCI
chr13	1.05E+08	1.05E+08	Intergenic	Intergenic	-1347	NM_00100	59079	Erbb2ip	1700028E0
chr8	1.25E+08	1.25E+08	intron (NM	intron (NM	-16456	NM_00969	11821	Aprt	C85684
chr15	75812377	75812525	exon (NM_	exon (NM_	264	NM_00116	223648	Ccdc166	2410075B1
chr3	1.29E+08	1.29E+08	exon (NM_	exon (NM_	56413	NM_00128	18741	Pitx2	9430085M
chr9	85656002	85656150	Intergenic	L1MD3|LIN	-13135	NM_00108	108837	Ibtk	5430411K1
chr4	8490427	8490550	intron (NM	Lx8|LINE|L	27697	NM_02151	59021	Rab2a	9330148M
chr8	23377302	23377550	Intergenic	Intergenic	-16487	NM_19925	234129	Tpte	Pten2
chr13	19287427	19287575	Intergenic	Intergenic	200120	NM_02427	76205	Stard3nl	0610035N(
chr3	19926502	19926925	intron (NM	intron (NM	8597	NM_00114	12807	Hps3	coa
chr8	73131077	73131150	intron (NM	intron (NM	1100	NM_13377	76900	Ssbp4	1210002E1
chr5	66115952	66116100	Intergenic	Intergenic	-26931	NM_00108	71521	Pds5a	9030416H1
chr19	4192202	4192375	intron (NM	CpG	114	NM_03186	19045	Ppp1ca	Ppp1c|disr
chr10	82683802	82683875	Intergenic	RMER30|D	116724	NM_17738	338365	Slc41a2	A230035L0
chr11	82876702	82876975	Intergenic	Intergenic	-1776	NM_01140	20556	Slfn2	Shlf2
chr5	1.24E+08	1.24E+08	intron (NM	intron (NM	386	NM_02992	77573	Vps33a	3830421M
chr19	10916302	10916625	promoter-T	promoter-T	-571	NM_02304	65221	Slc15a3	Ci1|cI-1
chr5	20208277	20208425	3' UTR (NM	3' UTR (NM	179591	NM_17299	68770	Phtf2	1110054G2
chr1	89694902	89695075	Intergenic	Intergenic	-5267	NM_00911	20215	Sag	A930001K1
chr2	10059002	10059275	Intergenic	Lx9|LINE|L	-6828	NM_01058	16425	Itih2	AI747202|I
chr9	1.21E+08	1.21E+08	intron (NM	intron (NM	15862	NM_17511	67095	Trak1	2310001H1
chr7	80676427	80676500	intron (NM	intron (NM	10169	NM_00108	244059	Chd2	2810013C0
chr2	33308252	33308350	3' UTR (NM	3' UTR (NM	15751	NM_02794	71834	Zbtb43	1700010E0
chrX	1.03E+08	1.03E+08	promoter-T	promoter-T	-126	NM_00882	18655	Pgk1	Pgk-1
chr11	61981977	61982300	intron (NM	intron (NM	-80348	NM_00741	11541	Adora2b	A2BAR|A2I
chr11	6191177	6191425	promoter-T	promoter-T	-299	NM_00125	18293	Ogdh	2210403E0
chr4	28124152	28124300	Intergenic	Intergenic	-616055	NM_00129	13841	Epha7	Cek11|Ebk
chr10	62114227	62114350	promoter-T	promoter-T	-342	NM_05318	94213	Ddx50	4933429B0
chr4	21639377	21639525	Intergenic	RMER21A|	-15397	NM_00129	51813	Ccnc	AI451004|/
chr5	65745277	65745750	intron (NM	MER20|DN	5863	NM_03118	83379	Klb	AV071179|
chr4	10845927	10846150	Intergenic	Intergenic	44393	NM_02600	67157	2610301B2	AI428449
chr16	87440827	87440925	promoter-T	promoter-T	-39	NM_01692	53858	Rwdd2b	ORF5
chr15	83970152	83970450	Intergenic	Intergenic	-16696	NM_02942	75772	Pnpla5	4833426H1
chr2	30785227	30785575	Intergenic	Intergenic	-23120	NM_13367	30934	Tor1b	2610016F0
chr11	76670277	76670400	Intergenic	Intergenic	-9828	NM_00775	12874	Cpd	AA960140|
chr1	9803127	9803275	intron (NM	intron (NM	14990	NM_17754	170755	Sgk3	2510015P2
chr11	6315827	6316450	intron (NM	CpG	265	NM_00890	268373	Ppia	2700098C0
chr7	1.34E+08	1.34E+08	Intergenic	Intergenic	-6627	NM_01195	26417	Mapk3	Erk-1|Erk1
chr7	50785727	50785850	intron (NM	intron (NM	2753	NM_00111	12489	Cd33	Siglec-3|gp
chr4	1.19E+08	1.19E+08	Intergenic	Intergenic	-75907	NM_01065	16656	Hivep3	2900056N(

chr1	1.89E+08	1.89E+08	Intergenic	Intergenic	-14276	NM_02604	67223	Rrp15	2810430M
chr16	36382977	36383275	Intergenic	Intergenic	-15470	NM_00108	1E+08	BC100530	-
chr11	97171227	97171825	Intergenic	Intergenic	-5504	NM_02592	67036	Mrpl45	2600005P0
chr7	53423802	53424075	intron (NM	L1MC4a|LI	11465	NM_01151	20927	Abcc8	D930031B2
chr11	1.11E+08	1.11E+08	Intergenic	Intergenic	377410	NM_00842	16518	Kcnj2	IRK1|Kcnf1
chr2	85799927	85800075	Intergenic	RMER16-in	8779	NM_00101	257936	Olfr1028	MOR198-3
chr15	26508277	26508700	intron (NM	intron (NM	269661	NM_17759	211147	11-Mar	9630025C2
chr14	99498577	99499050	exon (NM_	exon (NM_	170	NM_02932	52023	Pibf1	-
chr7	26181252	26181600	promoter-T	promoter-T	-420	NM_01071	16890	Lipe	4933403G1
chr3	89578302	89578525	intron (NM	intron (NM	882	NM_02731	70093	Ube2q1	1110002C0
chr3	68598377	68598550	Intergenic	Intergenic	-75045	NM_00116	68725	1110032F0	AI115547
chr1	1.93E+08	1.93E+08	intron (NM	CpG	894	NM_14488	226849	Ppp2r5a	PR61alpha
chr1	55144177	55144650	promoter-T	promoter-T	363	NM_01047	15510	Hspd1	60kDa|Hsp
chr1	1.34E+08	1.34E+08	intron (NM	intron (NM	2894	NM_00792	13714	Elk4	2310011G1
chr11	1.21E+08	1.21E+08	promoter-T	promoter-T	-76	NM_02540	66179	Ogfod3	1110031I0
chr2	25463102	25463175	intron (NM	intron (NM	828	NM_00102	227624	Rabl6	B230208H1
chr1	1.84E+08	1.84E+08	Intergenic	Intergenic	47427	NM_00785	13244	Degs1	AA536663|
chr9	56842752	56842825	promoter-T	promoter-T	13	NM_01965	56294	Ptpn9	MEG2
chr7	20216327	20216650	TTS (NM_0	TTS (NM_0	-1701	NM_00904	19698	Relb	shep
chr16	43889427	43890050	promoter-T	promoter-T	51	NM_02912	106248	Qtrtd1	3110012M
chr5	72614202	72614350	intron (NR_	intron (NR_	19694	NM_15338	231287	Atp10d	9830145H1
chr17	66133052	66133200	intron (NM	CpG-6548	431	NR_10446	70351	Ppp4r1	3110001J1
chr9	86358427	86358600	promoter-T	promoter-T	10	NM_02739	70348	Ube2cbp	2610018I0
chr10	75227777	75227900	promoter-T	promoter-T	-736	NM_17254	104248	Cabin1	A330070M
chr8	14039752	14040150	intron (NM	intron (NM	50376	NM_00103	234086	Erich1	-
chr11	79396927	79397200	Intergenic	Intergenic	-7651	NM_17554	268451	Rab11fip4	A730072L0
chr8	1.27E+08	1.27E+08	promoter-T	promoter-T	554	NM_19810	102058	Exoc8	AI414418|
chr15	81278027	81278275	intron (NM	intron (NM	-18595	NM_01971	56438	Rbx1	1500002P1
chr11	62352927	62353050	promoter-T	promoter-T	-225	NM_02844	73139	Cenpv	3110013H0
chr1	10027827	10027925	promoter-T	promoter-T	364	NR_10228	26754	Cops5	AI303502|
chr16	49774727	49775050	Intergenic	Intergenic	75481	NM_02868	73916	Ift57	4833420A1
chr19	11733752	11733950	intron (NM	intron (NM	1198	NM_00103	225922	Oosp2	Gm99|Plac
chr1	1.62E+08	1.62E+08	Intergenic	Intergenic	-54648	NR_02811	67647	4930523C0	-
chr15	72897377	72897450	Intergenic	Intergenic	-5779	NM_00116	76510	Trappc9	1810044A2
chr2	1.29E+08	1.29E+08	promoter-T	promoter-T	-587	NM_00129	19261	Sirpa	AI835480|
chr16	20641827	20641975	intron (NM	intron (NM	-9824	NR_02748	21762	Psmd2	9430095H0
chr14	31683177	31683400	intron (NM	intron (NM	-9155	NM_18139	66175	Mustn1	1110028G0
chr10	85379777	85380000	promoter-T	promoter-T	-198	NM_18165	72843	Prdm4	1700031E1
chr2	39081627	39081975	promoter-T	promoter-T	57	NM_02420	67857	Ppp6c	2310003C1
chr8	41393727	41393800	promoter-T	promoter-T	358	NM_03011	78506	Micu3	2900075B1
chr13	74631452	74631600	promoter-T	promoter-T	-128	NM_14623	235956	Zfp825	-
chr11	87236427	87236675	intron (NM	intron (NM	-4542	NM_00119	83560	Tex14	C85585
chr8	73284352	73284575	intron (NM	intron (NM	357	NM_18317	234384	Mpv17l2	Fksg24
chr7	26840127	26840525	Intergenic	Intergenic	-6188	NM_00781	13089	Cyp2b13	-
chr1	52078802	52079200	intron (NM	intron (NM	13913	NM_01148	20849	Stat4	-
chr1	1.53E+08	1.53E+08	intron (NM	L1MD|LINE	54085	NM_02201	63913	Fam129a	AI256368|
chr6	1.43E+08	1.43E+08	promoter-T	promoter-T	59	NM_00128	74741	C2cd5	5730419I0

chr15	59248002	59248375	intron (NM	intron (NM	42435	NM_00116	68501	Nsmce2	1110014D1
chr2	1.64E+08	1.64E+08	intron (NM	intron (NM	15928	NM_00111	381404	Pabpc1l	1810053B0
chr11	1.14E+08	1.14E+08	intron (NM	intron (NM	3998	NM_02000	56699	Cdc42ep4	1500041M
chr6	1.09E+08	1.09E+08	exon (NM_	exon (NM_	153	NM_13867	192193	Edem1	A130059K2
chr8	72876802	72877075	5' UTR (NM	5' UTR (NM	234	NM_03068	19704	Upf1	B430202H1
chr3	1.2E+08	1.2E+08	Intergenic	Intergenic	-217157	NM_01955	56195	Ptbp2	Ptb2|brPTE
chr3	83959402	83959550	intron (NM	CpG	232	NM_02979	76915	Mnd1	2610034E1
chr8	1.29E+08	1.29E+08	Intergenic	Intergenic	112995	NM_02421	67952	Tomm20	1810060K0
chr3	41966552	41966650	Intergenic	Intergenic	420061	NM_02727	73852	D3Ertd751	2810009O1
chr14	21748952	21749150	intron (NM	MIR|SINE|	396	NM_00950	22330	Vcl	9430097D2
chr8	92320577	92320775	Intergenic	Intergenic	551475	NM_17291	244579	Tox3	500-9|BC0
chr3	1.04E+08	1.04E+08	intron (NR_	intron (NR_	3897	NM_00130	15257	Hipk1	1110062K0
chr1	6204627	6204850	promoter-T	promoter-T	-5	NM_00982	12421	Rb1cc1	2900055E0
chr10	19886627	19886975	intron (NM	B1F|SINE|/	18075	NM_00119	17761	Map7	E-MAP-115
chr12	1.19E+08	1.19E+08	intron (NM	CpG	17486	NM_17593	217944	Rapgef5	4932413M
chr9	72487627	72488200	Intergenic	Intergenic	-22241	NM_01089	17999	Nedd4	AA959633|
chr6	40275427	40275675	promoter-T	promoter-T	74	NM_02353	69923	Agk	2610037M
chr2	1.34E+08	1.34E+08	Intergenic	Intergenic	-8819	NM_02914	52837	Tmx4	2810417D0
chr9	50582702	50582875	promoter-T	promoter-T	-542	NM_13398	102580	Alg9	8230402H1
chr4	1.09E+08	1.09E+08	promoter-T	promoter-T	-929	NM_15339	230603	Ttc39a	4922503N0
chr2	1.46E+08	1.46E+08	promoter-T	promoter-T	-276	NM_02642	67877	Naa20	1500004D1
chr14	9097002	9097275	Intergenic	L1M4c|LIN	-5605	NM_05311	93732	Acox2	THCCox
chr11	3446052	3446200	Intergenic	Intergenic	-6831	NM_00128	29856	Smtn	smsmo
chr15	80573527	80573800	intron (NM	intron (NM	31920	NM_14481	213988	Tnrc6b	2700090M
chr6	17236602	17236825	intron (NM	intron (NM	5528	NM_01690	12390	Cav2	AI447843
chr13	16021752	16022225	Intergenic	Intergenic	-84320	NM_00838	16323	Inhba	-
chr7	20148177	20148275	promoter-T	promoter-T	-479	NM_19914	232947	Ppp1r37	Gm158|Lrr
chr12	1.06E+08	1.06E+08	Intergenic	ETnERV2-ir	-25429	NM_00110	238395	Serpina3j	AI506554|
chr5	1.06E+08	1.06E+08	intron (NR_	CpG	376	NM_01875	54367	Zfp326	5730470H1
chr3	27836477	27836575	Intergenic	Intergenic	-1076	NM_00116	18805	Pld1	AA536939|
chr6	99093777	99093975	intron (NM	intron (NM	-115584	NM_00119	108655	Foxp1	3110052D1
chr16	33830127	33830275	intron (NM	CpG	450	NM_00114	16419	Itgb5	AA475909|
chr2	1.5E+08	1.5E+08	promoter-T	promoter-T	50	NM_08057	68738	Acss1	1110032O1
chr17	66425902	66426050	intron (NM	CpG	410	NM_00102	106585	Ankrd12	2900001A1
chr2	72034627	72034975	intron (NM	ID_B1|SINI	-88893	NM_02305	65964	Zak	AV006891|
chr6	1.14E+08	1.14E+08	exon (NM_	exon (NM_	298	NM_00950	22346	Vhl	Vhlh
chr19	34323152	34323250	intron (NM	intron (NM	6662	NM_00739	11475	Acta2	0610041G0
chr1	87584377	87584450	intron (NM	B4A|SINE|	37788	NM_01367	20684	Sp100	A430075G1
chr14	1.03E+08	1.03E+08	intron (NR_	intron (NR_	-25297	NM_17771	239217	Kctd12	AU046135
chr2	1.68E+08	1.68E+08	Intergenic	Intergenic	-7226	NM_01120	19246	Ptpn1	PTP-1B|PT
chr10	41355177	41355350	Intergenic	Intergenic	-47704	NM_00117	75973	Ccdc162	5033413D2
chr6	1.25E+08	1.25E+08	Intergenic	Intergenic	-26665	NM_01170	22371	Vwf	6820430P0
chr2	91570077	91570750	5' UTR (NM	5' UTR (NM	118	NM_00108	228361	Ambra1	2310079H0
chr7	90807677	90807775	intron (NM	intron (NM	27199	NM_02337	170460	Stard5	2310058G2
chr1	1.53E+08	1.53E+08	Intergenic	Intergenic	-56815	NM_00103	117198	Ivns1abp	1190004M
chr14	78936352	78937075	promoter-T	promoter-T	-46	NM_00116	219181	Akap11	6330501D1
chr3	5430627	5430925	Intergenic	Intergenic	145472	NM_00116	19302	Pex2	D3Ertd138

chr15	59538627	59538900	Intergenic	Intergenic	58554	NM_14454	211770	Trib1	A530090O
chr5	65356827	65356925	Intergenic	Intergenic	-4438	NM_02666	68303	Fam114a1	1190001N(
chr13	49969752	49970025	Intergenic	ETnERV3-ir	192389	NM_17201	105148	Iars	2510016L1
chr17	15577352	15577625	Intergenic	Intergenic	44278	NM_02420	67544	Fam120b	4932442K(
chr2	1.58E+08	1.58E+08	exon (NM_	exon (NM_	4219	NM_00950	22348	Slc32a1	R75019|V(
chr1	1.27E+08	1.27E+08	5' UTR (NM	5' UTR (NM	141	NM_02373	74117	Actr3	1200003A(
chr3	30891202	30891300	Intergenic	Intergenic	-3442	NM_00885	18759	Prkci	2310021H1
chr2	1.81E+08	1.81E+08	intron (NM	CpG	248	NM_00932	21400	Tcea2	AI326274|
chr2	6134077	6134375	promoter-T	promoter-T	-186	NM_02420	67856	Echdc3	2310005D1
chr9	72297302	72297375	exon (NM_	exon (NM_	11002	NM_00861	17427	Mns1	AW546487
chr1	1.21E+08	1.21E+08	exon (NM_	exon (NM_	370	NM_14610	226351	Tmem185b	2500001K1
chr11	20991902	20992000	intron (NM	CpG	624	NM_02332	67245	Peli1	2810468L0
chr11	80197252	80197500	intron (NR_	intron (NR_	595	NM_00113	22680	Zfp207	8430401D1
chr15	9438102	9438400	intron (NM	intron (NM	21380	NM_00837	16197	Il7r	CD127|IL-7
chr11	68873277	68873350	promoter-T	promoter-T	36	NM_02800	71923	2310047M	-
chr13	80642152	80642600	Intergenic	Lx9|LINE|L	-380307	NM_00104	105171	Arrdc3	AI450344|
chr1	1.8E+08	1.8E+08	exon (NM_	exon (NM_	352	NM_01680	51810	Hnrnpu	AA408410|
chr1	1.66E+08	1.66E+08	intron (NM	intron (NM	13239	NM_00116	66352	Blzf1	1700030G(
chr6	1.13E+08	1.13E+08	exon (NM_	exon (NM_	43318	NM_02838	72895	Setd5	2900045N(
chr3	1.21E+08	1.21E+08	Intergenic	Intergenic	-39389	NM_14539	213603	Slc44a3	BC010552
chr12	76201502	76202025	intron (NM	MER110A|	76556	NM_17280	238271	Kcnh5	Eag2
chrX	34666752	34666950	promoter-T	promoter-T	93	NM_02593	67050	Nkap	2610020O(
chr16	44138452	44138650	intron (NM	intron (NM	581	NM_00750	11964	Atp6v1a	AI647066|
chr1	1.46E+08	1.46E+08	Intergenic	ID_B1|SINE	-5824	NM_14599	214498	Cdc73	8430414L1
chr10	1.16E+08	1.16E+08	promoter-T	promoter-T	160	NM_00100	216363	Rab3ip	B230311A(
chr14	99678377	99678675	Intergenic	Intergenic	-19384	NM_00976	12224	Klf5	4930520J0
chr2	1.3E+08	1.3E+08	promoter-T	promoter-T	373	NM_17876	319513	Pced1a	A930025D(
chrX	50342052	50342150	intron (NM	intron (NM	846	NM_01355	15452	Hprt	C81579|HF
chr14	48187802	48188025	exon (NM_	exon (NM_	196	NM_17259	1.01E+08	Atg14	4832427M
chr6	87445877	87445975	exon (NM_	exon (NM_	395	NR_10448	232201	Arhgap25	A130039I2
chr5	1.41E+08	1.41E+08	intron (NM	intron (NM	3443	NM_00849	16848	Lfng	AW061165
chr17	51243777	51243850	intron (NM	intron (NM	54890	NM_00128	72238	Tbc1d5	1600014N(
chr16	16983627	16983750	5' UTR (NM	5' UTR (NM	213	NM_00103	26413	Mapk1	9030612K1
chr11	44163177	44163425	Intergenic	Intergenic	-50264	NM_00835	16160	Il12b	Il-12b|Il-12
chr8	13685702	13685925	Intergenic	Intergenic	-8226	NM_00902	19414	Rasa3	AI326412|
chr8	1.27E+08	1.27E+08	intron (NM	intron (NM	19794	NM_13927	108148	Galnt2	AI480629
chr1	1.59E+08	1.59E+08	promoter-T	promoter-T	100	NM_17764	226525	Rasal2	6530401P1
chr18	34963902	34963975	intron (NM	intron (NM	27276	NM_00108	277250	Kdm3b	5830462I2
chr6	49023902	49023975	exon (NM_	exon (NM_	144	NM_02935	75593	Malsu1	2410003K1
chr6	87885127	87885300	intron (NM	intron (NM	21243	NM_17373	232210	Hmces	8430410A1
chr11	1.06E+08	1.06E+08	promoter-T	promoter-T	-607	NM_19829	21763	Tex2	4930568E0
chr1	1.66E+08	1.66E+08	Intergenic	Intergenic	25194	NM_00116	20343	Sell	AI528707|
chr2	1.67E+08	1.67E+08	intron (NM	intron (NM	940	NM_01983	56336	B4galt5	9430078I0
chr6	38303127	38303200	intron (NM	intron (NM	1440	NM_02886	78781	Zc3hav1	1200014N1
chr11	98011277	98011375	promoter-T	promoter-T	-396	NM_02814	72194	Fbxl20	2610511F2
chr1	1.68E+08	1.68E+08	Intergenic	Intergenic	-7329	NM_00108	68481	Mpzl1	1110007A1
chr9	1.24E+08	1.24E+08	Intergenic	Intergenic	8741	NM_00987	12581	Cdkn2d	INK4d|p19

chr7	1.18E+08	1.18E+08	TTS (NM_0	TTS (NM_0	2768	NM_00962	11535	Adm	AM
chr5	1.38E+08	1.38E+08	promoter-T	promoter-T	71	NM_00100	381678	Zcwpw1	Gm1053
chr4	1.03E+08	1.03E+08	intron (NM	intron (NM	-75983	NM_00116	67344	Tctex1d1	1700055O1
chr1	1.52E+08	1.52E+08	intron (NM	MIRm|SINI	1351	NM_00886	18783	Pla2g4a	Pla2g4|cPL
chr19	21940352	21940500	Intergenic	Intergenic	87596	NM_03199	83921	Tmem2	-
chr5	1.39E+08	1.39E+08	promoter-T	promoter-T	439	NM_02139	11781	Ap4m1	4930443L0
chr19	29302652	29302775	Intergenic	Intergenic	-23580	NM_00104	16452	Jak2	Fd17
chr3	1.31E+08	1.31E+08	Intergenic	Intergenic	-89747	NM_02894	74442	Sgms2	4933405A1
chr1	63159802	63160000	intron (NM	intron (NM	940	NM_00108	227195	Ino80d	7330405I1
chr19	25415502	25415600	intron (NM	intron (NM	103859	NM_18140	107351	Kank1	A930031B0
chr7	1.28E+08	1.28E+08	5' UTR (NM	5' UTR (NM	242	NM_02155	59052	Mettl9	0610012D0
chr16	10411977	10412200	promoter-T	promoter-T	57	NM_01195	26425	Nubp1	-
chr9	21920952	21921125	Intergenic	(TC)n|Simp	-2425	NM_17077	66126	Elof1	1110011K1
chr13	59105252	59105325	intron (NM	intron (NM	196230	NM_00102	18212	Ntrk2	GP145-TrkI
chr2	1.19E+08	1.19E+08	promoter-T	promoter-T	-704	NM_02961	76464	Casc5	2310043D0
chr13	76438177	76438375	Intergenic	Intergenic	-84133	NM_03017	78771	Mctp1	2810465F1
chr16	6715527	6715600	intron (NM	intron (NM	-354365	NM_18318	268859	Rbfox1	A2bp|A2bp
chr9	1.15E+08	1.15E+08	exon (NM_	exon (NM_	235	NM_13397	102545	Cmtm7	AI481279|
chr1	1.84E+08	1.84E+08	Intergenic	Intergenic	-15517	NM_01205	27058	Srp9	9kDa
chr11	83285802	83285875	promoter-T	promoter-T	-772	NM_02742	70439	Taf15	2610111C2
chr5	91119077	91119250	Intergenic	Intergenic	-49650	NM_02847	73246	Rassf6	1600016B1
chr17	28993077	28993275	Intergenic	Intergenic	-2180	NM_00103	433091	Pnpla1	-
chr12	1.14E+08	1.14E+08	promoter-T	promoter-T	-44	NM_19841	70435	Inf2	2610204M
chr4	44025202	44025375	promoter-T	promoter-T	-227	NM_01676	12757	Clta	AV026556|
chr1	16090252	16090325	Intergenic	B3A|SINE|	4226	NM_01129	19989	Rpl7	Rpl7a|Surf
chr12	85230027	85230425	intron (NR_	intron (NR_	-12464	NM_00127	18222	Numb	Nb
chr5	86905677	86905775	Intergenic	Intergenic	-7711	NM_00103	194597	Tmprss11a	Gm7
chr4	1.33E+08	1.33E+08	Intergenic	Intergenic	-23622	NM_00116	230789	Fam76a	-
chr2	25110952	25111175	promoter-T	promoter-T	104	NM_17734	227615	Tmem203	C730025P1
chr1	51594902	51595050	Intergenic	Intergenic	-59733	NM_02869	109019	Nabp1	4930434H0
chr4	46402027	46402375	promoter-T	promoter-T	94	NM_02908	74753	5830415F0	AV014846|
chr2	65076827	65076900	promoter-T	promoter-T	-180	NM_02722	319876	Cobll1	1810047P1
chr4	1.09E+08	1.09E+08	Intergenic	CpG	8001	NM_00767	12580	Cdkn2c	C77269|IN
chr2	1.81E+08	1.81E+08	promoter-T	promoter-T	33	NM_19816	229004	Gmeb2	AI839884
chr2	59617477	59617800	intron (NM	intron (NM	103025	NM_00115	72137	Wdsub1	1700048E1
chr16	21955527	21955950	3' UTR (NM	3' UTR (NM	-8113	NR_03777	66664	Tmem41a	2900010K0
chr17	12708027	12708300	Intergenic	Intergenic	-7593	NM_01139	20519	Slc22a3	EMT|Oct3|
chr8	89148152	89148350	exon (NM_	exon (NM_	309	NM_02582	66887	Lonp2	1300002A0
chr8	88079302	88079600	promoter-T	promoter-T	-281	NM_01979	56445	Dnaja2	1500017M
chr7	25187102	25187250	intron (NM	MER58A|D	2529	NM_02804	71997	Smg9	1500002O2
chr1	1.73E+08	1.73E+08	intron (NM	intron (NM	1147	NM_00100	226652	Arhgap30	6030405P0
chr7	1.06E+08	1.06E+08	intron (NM	CpG	591	NM_17228	233545	2210018M	2310016L0
chr1	87690202	87690550	5' UTR (NM	5' UTR (NM	354	NM_13378	12283	Cab39	AA408805|
chr10	98400402	98400550	intron (NM	intron (NM	22690	NM_02648	67972	Atp2b1	2810442I2
chr3	1.3E+08	1.3E+08	intron (NM	intron (NM	1309	NM_00119	619547	Rpl34-ps1	EG619547|
chr2	1.68E+08	1.68E+08	non-coding	non-coding	127	NM_00129	18019	Nfatc2	AI607462|
chr15	54567902	54568025	Intergenic	Lx9|LINE|L	-9520	NM_01093	18133	Nov	C130088N2

chr11	79805827	79806200	promoter-T	promoter-T	-595	NM_00116	52615	Suz12	2610028O1
chr5	1.45E+08	1.45E+08	intron (NM	intron (NM	159	NM_02741	70381	Tecpr1	2210010N0
chr2	27947577	27948075	Intergenic	Intergenic	-7428	NM_01019	14134	Fcnb	Fcn2
chr8	80073427	80073600	exon (NM_	exon (NM_	217	NM_00108	102182	Prmt10	AI931714|
chr14	55495677	55496000	exon (NM_	exon (NM_	537	NM_00116	105651	Ppp1r3e	A630071A1
chr1	1.83E+08	1.83E+08	intron (NM	intron (NM	1002	NM_14479	208795	Tmem63a	BC014795|
chrX	66608852	66609125	Intergenic	Intergenic	-4518	NM_00803	14266	Aff2	FMR2P|Fm
chr6	85401002	85401300	promoter-T	promoter-T	145	NM_00116	73327	Pradc1	1700040I0
chr4	40670127	40670275	promoter-T	promoter-T	238	NM_00116	15502	Dnaja1	Hsj2|Nedd
chr11	45665902	45665975	intron (NM	CpG	472	NM_00104	216705	Clint1	AI642036|
chr13	47028202	47028325	Intergenic	Intergenic	-3176	NM_01061	16553	Kif13a	4930505I0
chr1	1.31E+08	1.31E+08	Intergenic	Intergenic	-21650	NM_00991	12767	Cxcr4	CD184|Cm
chr2	24043127	24043275	intron (NM	intron (NM	1205	NM_15351	215257	Il1f9	Il36g
chr13	1.14E+08	1.14E+08	promoter-T	promoter-T	162	NM_02815	72198	Skiv2l2	2610528A1
chr7	7230852	7230950	promoter-T	promoter-T	99	NR_00355	76229	Vmn2r29	6430701C0
chr3	65222052	65222300	Intergenic	Intergenic	-25701	NM_02615	67437	Ssr3	0610038P0
chr12	58603377	58603500	Intergenic	Lx8|LINE|L	43670	NM_00825	15375	Foxa1	Hnf-3a|Hn
chr11	1.15E+08	1.15E+08	intron (NM	CpG	483	NM_00825	15374	Hn1	-
chr17	35804327	35804550	promoter-T	promoter-T	99	NM_17513	68915	Vars2	1190004I2
chr13	62960652	62960800	promoter-T	promoter-T	-996	NM_00799	14120	Fbp2	Fbp-1|Fbp
chr11	98821152	98821375	promoter-T	promoter-T	-522	NM_00117	19401	Rara	Nr1b1|RAF
chr14	19071352	19071425	5' UTR (NM	5' UTR (NM	232	NM_01158	353187	Nr1d2	RVR|Rev-e
chr4	4017102	4017400	Intergenic	MTB|LTR|I	-12590	NM_00108	242286	Sdr16c6	4833413O1
chr6	83780952	83781650	5' UTR (NM	5' UTR (NM	434	NM_14616	232164	Paip2b	-
chr1	1.29E+08	1.29E+08	intron (NM	intron (NM	86800	NM_14512	107895	Mgat5	4930471A2
chr5	1.06E+08	1.06E+08	Intergenic	Intergenic	-7735	NM_00103	634650	Gbp11	EG634650|
chr2	72280877	72281150	TTS (NM_0	TTS (NM_0	-33263	NM_02586	66953	Cdca7	2310021G0
chr2	30297077	30297200	intron (NM	intron (NM	25568	NM_13874	110854	Ppp2r4	2610042B2
chr12	35633927	35634000	Intergenic	Intergenic	-32196	NM_00127	79221	Hdac9	AV022454|
chr11	58120602	58120925	intron (NM	CpG	192	NM_00104	103836	Zfp692	AI746306|
chr5	1.36E+08	1.36E+08	promoter-T	promoter-T	-229	NM_00974	12054	Bcl7b	-
chr7	88049777	88049850	exon (NM_	exon (NM_	135	NM_02652	68039	Nmb	3110023K1
chrX	91479977	91480050	promoter-T	promoter-T	-256	NM_00103	236904	Klhl15	6330500C1
chr12	33639227	33639350	promoter-T	promoter-T	237	NM_19871	19027	Sypl	AI314763|
chr7	3663952	3664250	TTS (NM_0	TTS (NM_0	7883	NM_01109	18733	Pirb	Gp91|LIR-3
chr1	1.36E+08	1.36E+08	promoter-T	promoter-T	-336	NM_14551	98710	Rabif	AI842864|
chr1	62750477	62750575	5' UTR (NM	5' UTR (NM	635	NM_00107	18187	Nrp2	1110048P0
chr1	4847527	4848350	promoter-T	promoter-T	163	NM_01154	21399	Tcea1	S-II
chr9	66891452	66891525	exon (NM_	exon (NM_	190	NM_00116	22003	Tpm1	AA986836|
chr3	88330402	88330525	Intergenic	Intergenic	-5854	NM_00102	72640	Mex3a	2700083E1
chr15	88693352	88693475	intron (NM	CpG-5224	789	NM_14547	223775	Pim3	BC026639|
chr1	53841727	53841975	intron (NM	CpG	208	NM_13381	98267	Stk17b	3110009A0
chr6	1.27E+08	1.27E+08	promoter-T	promoter-T	-240	NM_18140	101187	Parp11	5330431N2
chr9	1.22E+08	1.22E+08	intron (NM	intron (NM	5579	NM_02617	67469	Abhd5	1300003D0
chr11	96912702	96912800	intron (NM	(CA)n|Simp	617	NM_00108	71240	Osbpl7	4933437E1
chr9	88146352	88146575	Intergenic	L1_Mm|LIN	-75984	NM_01185	23959	Nt5e	2210401F0
chr11	83778177	83778250	intron (NM	CpG	280	NM_19434	217030	Synrg	Ap1gbp1|S

chr5	12885002	12885150	Intergenic	Intergenic	501910	NM_02888	108151	Sema3d	4631426B1
chr4	1.17E+08	1.17E+08	intron (NM	CpG-10216	36881	NM_02958	68777	Tmem53	1110038M
chr5	68236752	68236950	intron (NM	intron (NM	1819	NM_00128	11980	Atp8a1	AI481521|,
chr10	59791727	59792125	intron (NR_	intron (NR_	-17673	NM_00115	74048	4632428N(	Dies1|PD-1
chr7	49753677	49754000	Intergenic	Intergenic	-50388	NM_02770	71162	4933421I0	-
chr8	1.23E+08	1.23E+08	intron (NM	intron (NM	4775	NM_00832	15900	Irf8	AI893568|
chr1	1.09E+08	1.09E+08	promoter-T	promoter-T	-384	NM_17741	12043	Bcl2	AW986256
chr11	46127977	46128325	Intergenic	MIR3|SINE	-1790	NM_00125	76884	Cyfip2	1500004I0
chr10	42580002	42580125	promoter-T	promoter-T	-255	NM_17293	268297	Scml4	9330161D1
chr11	49472402	49472500	Intergenic	Intergenic	-4646	NM_05403	68662	Scgb3a1	HIN-1|LuLe
chr12	16904577	16904725	intron (NM	intron (NM	2867	NM_00907	19878	Rock2	B230113H1
chr8	13105077	13105150	promoter-T	promoter-T	230	NM_17870	234069	Pcid2	A730042J0
chr1	1.95E+08	1.95E+08	intron (NM	intron (NM	3488	NM_14481	215303	Camk1g	CLICK-III|Ca
chr2	72878327	72878450	Intergenic	Intergenic	-59885	NM_00101	20687	Sp3	D130027J0
chr11	50191927	50192125	intron (NM	CpG-1896	805	NM_02151	59013	Hnrnph1	AI642080|
chr13	74147002	74147150	intron (NM	CpG	209	NM_18283	72948	Tppp	2900041A(
chr7	1.37E+08	1.37E+08	promoter-T	promoter-T	-1	NM_17225	207425	Wdr11	2900055P1
chr16	18498827	18499050	promoter-T	promoter-T	-26	NM_00128	13972	Gnb1l	ESTM55|G
chr1	1.73E+08	1.73E+08	intron (NM	MIR|SINE|	1192	NM_01018	14127	Fcer1g	AI573376|
chr8	13676377	13676475	intron (NM	intron (NM	1161	NM_00902	19414	Rasa3	AI326412|
chr5	98459352	98459525	exon (NM_	exon (NM_	543	NM_13373	71914	Antxr2	2310046B1
chr2	72808402	72808775	exon (NM_	exon (NM_	8750	NM_00109	20687	Sp3	D130027J0
chr6	1.37E+08	1.37E+08	intron (NM	intron (NM	5397	NM_00130	11857	Arhgdib	D4|Gdid4|
chr7	1.18E+08	1.18E+08	intron (NM	Charlie7|D	6162	NM_19446	17540	Mrvi1	BB115629|
chr14	22838602	22838775	promoter-T	promoter-T	-246	NM_02827	76633	1700112E(	2700009F1
chr6	82747327	82747625	Intergenic	Intergenic	-23028	NM_01382	15277	Hk2	AI642394|
chr6	4561927	4562175	intron (NM	intron (NM	10985	NM_14539	213819	Casd1	Cas1|Cast1
chr2	1.8E+08	1.8E+08	promoter-T	promoter-T	488	NM_17875	269397	Ss18l1	A230053O
chr6	75746427	75746525	Intergenic	Intergenic	-1446235	NM_02888	74342	Lrrtm1	4632401D(
chr14	69929777	69929925	Intergenic	Intergenic	-17495	NM_17342	239167	Synb	D930020E(
chr9	21091052	21091275	intron (NM	intron (NM	1250	NM_14541	215194	Kri1	-
chr7	65914052	65914225	exon (NM_	exon (NM_	566	NM_00972	11982	Atp10a	Atp10c|pfa
chr13	16042852	16043250	Intergenic	Intergenic	-63257	NM_00838	16323	Inhba	-
chr12	88688902	88689075	intron (NM	L1MC4a|LI	40192	NM_01147	20773	Sptlc2	AI173915|
chr14	76813652	76814025	Intergenic	Intergenic	-1790	NM_20765	21807	Tsc22d1	AA589566|
chr15	31531202	31531425	promoter-T	promoter-T	246	NM_00763	12465	Cct5	Ccte|TCPE
chr9	48303577	48303975	promoter-T	promoter-T	328	NM_00100	434402	Gm5617	EG434402
chr6	82932552	82932800	intron (NM	intron (NM	35760	NM_03307	110958	M1ap	D6Mm5e
chr8	1.24E+08	1.24E+08	Intergenic	Intergenic	-21469	NM_14560	234825	Klhdc4	AA408426|
chr12	74817102	74817225	intron (NM	intron (NM	131135	NM_00885	18755	Prkch	Pkch
chr5	1.08E+08	1.08E+08	intron (NM	intron (NM	22404	NM_01698	1.01E+08	Rpl5	U21RNA
chr17	28354677	28354875	intron (NM	intron (NM	10053	NM_02718	23853	Def6	2410003F0
chr9	1.07E+08	1.07E+08	promoter-T	promoter-T	68	NM_03073	81000	Rad54l2	Arip4|D13(
chr15	78135852	78135925	intron (NM	intron (NM	263	NM_00778	12984	Csf2rb2	AIC2A|Beta
chrX	1.5E+08	1.5E+08	intron (NM	intron (NM	1316	NM_02732	70123	2210013O	AI181827|
chr4	1.26E+08	1.26E+08	Intergenic	tRNA-Ala-G	-1748	NM_01371	27096	Trappc3	1110058K1
chr7	88358302	88358450	promoter-T	promoter-T	-314	NM_00880	18584	Pde8a	AI551852|

chr11	97296077	97296225	Intergenic	Intergenic	-15323	NM_02149	58996	Arhgap23	A330041B1
chr8	1.11E+08	1.11E+08	Intergenic	Intergenic	-10393	NM_00749	11906	Zfhx3	A230102L0
chr5	1.08E+08	1.08E+08	intron (NM	L1ME2z|LII	70188	NM_01157	21814	Tgfbr3	1110036H2
chr13	38384152	38384300	Intergenic	Intergenic	-53359	NM_00755	12161	Bmp6	D13Wsu11
chr12	35731602	35731725	promoter-T	promoter-T	-198	NM_00101	217463	Snx13	Rgs-px1|m
chr7	54270552	54270625	Intergenic	Intergenic	-6804	NM_17531	101685	Spty2d1	5830435K1
chr4	1.2E+08	1.2E+08	Intergenic	Intergenic	-5955	NM_01956	56222	Cited4	MRG-2|Mr
chr2	1.56E+08	1.56E+08	3' UTR (NM	3' UTR (NM	3232	NM_02594	67067	Romo1	2010100O1
chr5	96432377	96432575	Intergenic	Intergenic	46518	NM_01886	55985	Cxcl13	4631412M
chr2	1.58E+08	1.58E+08	intron (NM	intron (NM	2344	NM_00950	22348	Slc32a1	R75019|VG
chr14	26667802	26667925	intron (NM	intron (NM	6222	NM_01346	11744	Anxa11	A830099O1
chr1	1.74E+08	1.74E+08	intron (NM	intron (NM	5385	NM_01348	12523	Cd84	A130013D2
chr8	1.2E+08	1.2E+08	Intergenic	L1MB8|LIN	-12536	NM_02872	74032	Sdr42e1	4632417N0
chr15	75672852	75673000	promoter-T	promoter-T	-588	NM_17212	223642	Zc3h3	BC049953|
chr12	4234102	4234225	exon (NM_	exon (NM_	131	NM_13404	52504	Cenpo	2810429O0
chr7	86772327	86772575	Intergenic	Intergenic	-9908	NM_01979	56315	Rhcg	BB065800
chr1	1.84E+08	1.84E+08	intron (NM	CpG	333	NM_01205	27058	Srp9	9kDa
chr8	1.23E+08	1.23E+08	intron (NM	intron (NM	2086	NM_00116	234797	6430548M	AW049007
chr5	1.09E+08	1.09E+08	5' UTR (NM	5' UTR (NM	172	NM_02610	13486	Dr1	1700121L0
chr2	1.67E+08	1.67E+08	intron (NM	intron (NM	4318	NM_14553	407243	Tmem189	AI840826|I
chr11	11586227	11586475	5' UTR (NM	5' UTR (NM	135	NM_00102	22778	Ikzf1	5832432G1
chr10	80040602	80040675	promoter-T	promoter-T	-597	NM_07847	118445	Klf16	AI843742|I
chr8	1.25E+08	1.25E+08	promoter-T	promoter-T	71	NM_14493	257633	Acsf3	BB101783
chr16	24199977	24200125	Intergenic	Intergenic	-193385	NM_17866	210126	Lpp	9430020K1
chr8	87187402	87187475	promoter-T	promoter-T	-687	NM_01049	15936	Ier2	AI317238|(
chr8	35884427	35884525	Intergenic	MER5B|DN	13812	NM_17693	319520	Dusp4	2700078F2
chr16	45029202	45030000	Intergenic	Intergenic	-64565	NM_02643	67896	Ccdc80	2610001E1
chr16	13902477	13902725	promoter-T	promoter-T	637	NM_00103	94184	Pdxdc1	2210010A1
chr13	99696002	99696225	intron (NM	CpG	226	NM_17871	238799	Tnpo1	AU021749
chr10	80894077	80894225	Intergenic	Intergenic	-4233	NM_02675	18029	Nfic	1110019L2
chr3	1.44E+08	1.44E+08	Intergenic	Intergenic	160358	NM_00116	16911	Lmo4	A730077C1
chr7	5013777	5014100	exon (NM_	exon (NM_	193	NM_00120	22185	U2af2	65kDa
chr12	1.05E+08	1.05E+08	intron (NM	intron (NM	14327	NM_14483	217847	Serpina10	PZI|ZPI
chr4	1.33E+08	1.33E+08	Intergenic	PB1D10|SI	26968	NM_01695	15331	Hmgn2	HMG-17|H
chr15	78236552	78236725	promoter-T	promoter-T	-349	NM_00943	22117	Tst	Rhodanese
chr15	11646152	11646550	Intergenic	Intergenic	189078	NM_00872	18162	Npr3	ANP-C|ANI
chr1	80022377	80023075	Intergenic	Intergenic	-167486	NM_00925	20720	Serpine2	B230326M
chr9	1.1E+08	1.1E+08	Intergenic	Intergenic	-4436	NM_00869	18054	Ngp	bectenecin
chr2	1.37E+08	1.37E+08	intron (NM	intron (NM	89126	NM_00103	74243	Slx4ip	2210009G2
chr1	1.84E+08	1.84E+08	intron (NM	CpG	181	NM_13381	98386	Lbr	AI505894|i
chr7	1.38E+08	1.38E+08	Intergenic	Intergenic	-18423	NM_13394	101476	Plekha1	AA960558|
chr7	1.24E+08	1.24E+08	Intergenic	Intergenic	-9620	NM_00113	53322	Nucb2	AI607786|(
chr4	1.34E+08	1.34E+08	intron (NM	CpG	220	NM_00125	76824	Mtfr1l	2410166I05
chrX	68538727	68538900	intron (NM	intron (NM	70556	NM_01992	17772	Mtm1	AF073996|
chr13	43493727	43493900	promoter-T	promoter-T	68	NM_02355	70078	Nol7	2210008F1
chr10	83996477	83996575	exon (NM_	exon (NM_	107	NM_17545	216197	Ckap4	5630400A0
chr18	36440352	36440425	promoter-T	promoter-T	-428	NM_00898	19290	Pura	CAGER-1|P

chr15	99602452	99602625	intron (NM	CpG	408	NM_02801	71949	Cers5	2310081H1
chr2	1.73E+08	1.73E+08	intron (NR_	CpG	-8071	NM_02730	70065	Ankrd60	1700019A2
chr12	87583077	87583150	intron (NM	CpG	285	NM_02740	70373	Gpatch2l	1700020O0
chr6	52108052	52108200	exon (NM_	exon (NM_	190	NM_01044	15394	Hoxa1	ERA1|Hox-
chr18	35930877	35930975	promoter-T	promoter-T	-287	NM_01991	56550	Ube2d2a	1500034D0
chr12	71320202	71320500	intron (NM	intron (NM	8319	NM_13319	110095	Pygl	-
chr4	1.32E+08	1.32E+08	intron (NM	CpG-10420	433	NM_14539	213541	Ythdf2	9430020E0
chr17	37139777	37139900	promoter-T	promoter-T	368	NM_00101	22715	Zfp57	G19|Zfp-57
chr6	1.29E+08	1.29E+08	intron (NM	RMER16-in	9101	NM_00115	17057	Klrb1a	Ly55a|NKR
chr2	57765452	57765600	Intergenic	Intergenic	-85040	NM_17285	241391	Galnt5	4832424J2
chr1	57463252	57463925	promoter-T	promoter-T	-70	NM_00103	68736	Tyw5	1110034B0
chr3	1.58E+08	1.58E+08	Intergenic	Intergenic	82064	NM_14595	107869	Cth	0610010I1
chr17	27993677	27993875	intron (NM	CpG	324	NM_00108	224648	Uhrf1bp1	1110020K1
chr7	59237602	59237750	intron (NM	intron (NM	23408	NR_02770	75744	Svip	1700006C1
chr19	5964377	5964600	promoter-T	promoter-T	-282	NM_00889	18969	Pola2	AI573378
chr17	32042352	32042500	Intergenic	MTB|LTR|I	-49689	NM_01083	17691	Sik1	Msk|Sik|Sn
chr2	1.1E+08	1.1E+08	intron (NM	intron (NM	41847	NM_17267	107515	Lgr4	9130225G0
chr13	41555327	41555475	intron (NM	intron (NM	27328	NM_00111	18003	Nedd9	Cas-L|CasL
chr10	39966727	39967050	promoter-T	promoter-T	-43	NM_02332	67239	Rpf2	2810470K2
chr2	1.19E+08	1.19E+08	5' UTR (NM	5' UTR (NM	361	NM_00108	383787	Ankrd63	Gm1337
chr1	93146952	93147125	intron (NM	CpG	142	NM_02645	67921	Ube2f	2510010F1
chr10	1.21E+08	1.21E+08	intron (NM	CpG	455	NM_13895	192678	Rassf3	AW212023
chr8	1.22E+08	1.22E+08	promoter-T	promoter-T	3	NM_02807	72042	Cotl1	1810074P2
chr13	37712152	37712300	Intergenic	Intergenic	-205681	NM_02683	68750	Rreb1	1110037N0
chr4	1.53E+08	1.53E+08	promoter-T	promoter-T	26	NM_20122	72946	Lrrc47	2900010D0
chr4	1.34E+08	1.34E+08	intron (NM	CpG	242	NM_00116	230809	Pdik1l	BC027088|
chr13	59749027	59749200	intron (NM	intron (NM	28032	NM_00103	105348	Golm1	2310001L0
chr3	1.16E+08	1.16E+08	promoter-T	promoter-T	-303	NM_14490	229782	Slc35a3	2310050P1
chr14	32001902	32002150	intron (NM	intron (NM	17986	NM_02265	64652	Nisch	1200007D0
chr3	1.46E+08	1.46E+08	Intergenic	Lx2B|LINE|	-19489	NM_02737	70285	Rpf1	2210420E2
chr1	93958377	93958475	intron (NM	intron (NM	86544	NM_20722	208727	Hdac4	4932408F1
chr12	86523552	86523750	Intergenic	Intergenic	-5388	NM_00882	18654	Pgf	AI854365|
chr10	43198727	43198875	promoter-T	promoter-T	-145	NM_19902	331623	Bend3	mKIAA155
chr9	1.06E+08	1.06E+08	Intergenic	Intergenic	-9114	NM_00116	665033	Col6a5	Col29a1|E(
chr5	1.14E+08	1.14E+08	Intergenic	Intergenic	9623	NM_14616	231633	Tmem119	AW208946
chr1	1.83E+08	1.83E+08	exon (NM_	exon (NM_	583	NM_14551	226757	Wdr26	1600024A0
chr17	29628152	29628225	intron (NM	CpG	198	NM_00884	18712	Pim1	Pim-1
chr2	26215252	26215325	promoter-T	promoter-T	-221	NM_00103	332579	Card9	Gm782
chr1	88295202	88295375	Intergenic	Intergenic	-10572	NM_01034	14767	Nmur1	FM-3|Gpr6
chr1	75176527	75176725	TTS (NM_0	TTS (NM_0	231	NM_02373	74104	Abcb6	1200005B1
chr4	1.47E+08	1.47E+08	intron (NM	intron (NM	572	NM_00964	11610	Agtrap	3300002E1
chr7	29926452	29926575	intron (NR_	intron (NR_	6561	NR_04567	233046	Rasgrp4	-
chr14	58617877	58618425	promoter-T	promoter-T	-52	NM_02864	68514	Micu2	1110008L2
chr1	1.73E+08	1.73E+08	Intergenic	MurERV4-i	-14625	NM_02538	66155	Ufc1	1110021H0
chr13	19974777	19975000	Intergenic	Intergenic	-58762	NM_18175	353346	Gpr141	PGR13
chr5	1.01E+08	1.01E+08	intron (NM	Lx8|LINE|L	4911	NM_13919	231507	Plac8	C15|D5Ws
chr17	47825877	47825975	promoter-T	promoter-T	-591	NM_00116	66119	Tomm6	1110002E2

chr9	43840077	43840325	Intergenic	Intergenic	-11266	NM_00938	21838	Thy1	CD90|T25|
chr5	52217477	52217775	Intergenic	B4|SINE|B	-272466	NM_00890	19017	Ppargc1a	A830037N(
chr3	96532127	96532325	promoter-T	promoter-T	692	NM_02640	67845	Rnf115	2610028E0
chr13	1.01E+08	1.01E+08	promoter-T	promoter-T	-459	NM_01142	20595	Smn1	AI849087|
chr5	28643177	28643375	promoter-T	promoter-T	-453	NM_02823	381626	Rbm33	3200001K1
chr7	71110777	71111000	Intergenic	Intergenic	-27087	NM_02136	50794	Klf13	0610043C1
chr10	80380877	80381050	promoter-T	promoter-T	27	NM_01072	16907	Lmnb2	-
chr5	1.51E+08	1.51E+08	intron (NM	intron (NM	10562	NM_13389	100637	N4bp2l1	2410024N1
chr1	1.73E+08	1.73E+08	Intergenic	Intergenic	-1419	NM_14455	246256	Fcgr4	4833442P2
chr13	41823227	41823400	Intergenic	Intergenic	77182	NM_00114	328231	Gm5082	A030008J0
chr15	8394127	8394300	5' UTR (NM	5' UTR (NM	250	NM_02770	71175	Nipbl	Idn3
chr7	26730227	26730625	Intergenic	Intergenic	47749	NM_00999	13088	Cyp2b10	Cyp2b|Cyp
chr13	50052577	50052675	Intergenic	Intergenic	275127	NM_17201	105148	Iars	2510016L1
chr1	16095402	16095575	promoter-T	promoter-T	-475	NM_13383	98711	Rdh10	3110069K0
chr13	91641627	91641725	intron (NM	MTC|LTR|I	40974	NM_02418	66970	Ssbp2	1500004K0
chr7	1.3E+08	1.3E+08	intron (NM	intron (NM	75866	NM_00112	70497	Arhgap17	5730403H1
chr2	1.8E+08	1.8E+08	intron (NM	intron (NM	1369	NM_18316	228993	Slc17a9	1700019H0
chr1	1.37E+08	1.37E+08	intron (NM	intron (NM	1335	NM_14541	215615	Rnpep	-
chr7	1.35E+08	1.35E+08	Intergenic	Intergenic	-46593	NM_14559	233913	BC017158	-
chr13	83089502	83089750	Intergenic	Lx8|LINE|L	-553407	NM_00117	17260	Mef2c	5430401D1
chr9	1.09E+08	1.09E+08	intron (NM	intron (NM	18702	NM_00892	19087	Prkar2a	1110061A2
chr2	49236852	49236950	Intergenic	Intergenic	-70105	NM_17266	227867	Epc2	5830499L1
chr12	4234927	4235000	promoter-T	promoter-T	-669	NM_13404	52504	Cenpo	2810429O(
chr1	83073902	83073975	Intergenic	Intergenic	-38915	NM_03055	80721	Slc19a3	A230084E2
chr10	67223302	67223400	Intergenic	Intergenic	152059	NM_17867	216049	Zfp365	AI839779|
chr9	1.23E+08	1.23E+08	intron (NM	CpG	111	NM_17336	235682	Zfp445	AW610627
chr8	27585002	27585200	Intergenic	L3|LINE|CF	316460	NM_19904	73754	Thap1	4833431A0
chr10	76999502	76999650	intron (NM	MTD|LTR|I	6483	NM_00840	16414	Itgb2	2E6|AI528
chr2	84781527	84781900	intron (NM	intron (NM	4910	NM_02139	58207	Slc43a3	Eeg1|SEEE
chr12	92881502	92881575	intron (NM	intron (NM	-52611	NM_17533	83602	Gtf2a1	6330549H0
chr3	95720777	95720975	Intergenic	Intergenic	-12304	NM_00125	66471	Anp32e	2810018A1
chr9	63247477	63247550	promoter-T	promoter-T	-462	NM_02709	69478	2300009A(	-
chr1	33870777	33871575	intron (NM	CpG	264	NM_13381	98403	Zfp451	4930515K2
chr1	1.34E+08	1.34E+08	promoter-T	promoter-T	81	NM_00792	13714	Elk4	2310011G1
chr6	1.19E+08	1.19E+08	intron (NM	intron (NM	-29450	NM_00125	12288	Cacna1c	Cav1.2|Cch
chr2	13496227	13496400	exon (NM_	exon (NM_	375	NM_01170	22352	Vim	-
chr12	1.03E+08	1.03E+08	intron (NM	intron (NM	18792	NM_01685	51786	Cpsf2	100kDa|26
chr5	91218902	91219100	Intergenic	Intergenic	3874	NM_20332	330122	Cxcl3	Dcip1|Gm1
chr2	26453327	26453600	intron (NM	L1MB3|LIN	6474	NM_02621	67512	Agpat2	2510002J0
chr4	1.49E+08	1.49E+08	intron (NM	B3|SINE|B	2294	NM_17337	100198	H6pd	AI785303|
chr1	10304927	10305125	Intergenic	Intergenic	-82275	NM_00110	211673	Arfgef1	ARFGEP1|E
chr10	1.17E+08	1.17E+08	Intergenic	Intergenic	-36922	NM_00101	432508	Cpsf6	4733401N1
chr5	1.36E+08	1.36E+08	intron (NM	B1_Mur4|S	22142	NM_00889	18984	Por	4933424M
chr8	1.1E+08	1.1E+08	promoter-T	promoter-T	-473	NM_02555	66427	Cyb5b	1810044O2
chr3	1.54E+08	1.54E+08	intron (NM	intron (NM	30666	NM_17517	209601	Erich3	1700013E1
chr1	80610002	80610225	intron (NM	intron (NM	51814	NM_00128	210293	Dock10	9330153B1
chr12	66273627	66273875	promoter-T	promoter-T	-184	NM_17257	217653	Mis18bp1	6720403H1

chr11	79092202	79092300	Intergenic	Intergenic	-24054	NM_01965	78889	Wsb1	1110056B1
chr1	1.84E+08	1.84E+08	Intergenic	Intergenic	-28235	NM_17337	209456	Trp53bp2	53BP2|AI7
chr15	63486277	63486550	Intergenic	Intergenic	153881	NM_03137	83492	Gsdmc	Gsdmc1|M
chr9	34931402	34931775	TTS (NM_0	TTS (NM_0	-7193	NM_00917	20443	St3gal4	Siat4c
chr13	55423027	55423375	promoter-T	promoter-T	140	NM_00900	19336	Rab24	6530406O0
chr1	1.3E+08	1.3E+08	promoter-T	promoter-T	605	NM_18175	226412	R3hdm1	R3hdm
chr17	9175852	9176000	3' UTR (NM	3' UTR (NM	-5272	NM_02585	66931	1700010I1	-
chr9	20766177	20766350	Intergenic	Intergenic	-1931	NM_00119	13433	Dnmt1	Cxxc9|Dnm
chr3	96000777	96001450	exon (NM_	exon (NM_	396	NM_02697	69168	Bola1	1810037G0
chr1	74600277	74600425	intron (NM	(GAA)n|Sir	9417	NM_14893	18802	Plcd4	4921507K2
chr1	1.33E+08	1.33E+08	intron (NM	MER58B|D	33892	NM_01070	16865	Eif2d	D1Ertd5e|I
chr5	1.37E+08	1.37E+08	Intergenic	CpG-11659	-1041	NM_00129	13047	Cux1	CDP|Cutl1
chr12	88162302	88162425	Intergenic	Intergenic	63401	NM_14583	238330	Irf2bpl	6430527G1
chr9	90008777	90008925	intron (NM	CpG	807	NM_00103	21761	Morf4l1	MORFRG15
chr7	1.24E+08	1.24E+08	intron (NM	CpG	171	NM_01108	18704	Pik3c2a	Cpk-m|PI3
chr1	79311277	79311575	Intergenic	Intergenic	125239	NM_00912	20254	Scg2	Chgc|SgII
chr10	79167252	79167950	intron (NM	intron (NM	498	NM_00976	12215	Bsg	AI115436|A
chr7	71041677	71042000	intron (NM	CpG	-19905	NM_00125	381994	E030018B1	Gm1697
chr3	53266977	53267200	promoter-T	promoter-T	92	NM_17250	212114	Nhlrc3	4833441N1
chr19	7048952	7049125	promoter-T	promoter-T	-500	NM_00888	18938	Ppp1r14b	AOM172|P
chr12	92629727	92629925	intron (NR_	L1MEc|LIN	-6977	NM_18181	75216	Cep128	4930534B0
chr10	1.28E+08	1.28E+08	intron (NM	CpG	264	NM_01111	18813	Pa2g4	38kDa|AA6
chr9	1.05E+08	1.05E+08	intron (NM	intron (NM	12786	NM_00125	235574	Atp2c1	1700121J1
chr7	38804002	38804200	intron (NM	CpG	470	NM_01127	19777	Uri1	C80913|NN
chr9	66019452	66019550	intron (NM	intron (NM	13468	NM_01001	13143	Dapk2	-
chr7	35006752	35006875	intron (NM	intron (NM	8542	NM_00815	14751	Gpi1	Amf|Gpi|G
chr3	1.16E+08	1.16E+08	Intergenic	Intergenic	-12863	NM_00117	229776	Cdc14a	A830059A1
chr13	28509527	28509650	Intergenic	Intergenic	275235	NM_02374	28078	Prl5a1	1600013P0
chr19	42007402	42007575	promoter-T	promoter-T	316	NM_00116	66583	Exosc1	2610035C1
chr1	54867027	54867125	intron (NM	intron (NM	116155	NM_00108	329154	Ankrd44	4930444A1
chr9	44321352	44321425	exon (NM_	exon (NM_	13116	NM_00755	12145	Cxcr5	Blr1|CXC-R
chr1	16655152	16655475	promoter-T	promoter-T	41	NM_02639	70397	Tmem70	1110020A0
chr16	84836152	84836300	promoter-T	promoter-T	-407	NM_01675	11957	Atp5j	-
chr1	72759452	72759550	intron (NM	intron (NM	1667	NM_00908	19981	Rpl37a	-
chr2	81893502	81893625	promoter-T	promoter-T	-252	NM_17551	241514	Zfp804a	C630007C1
chr7	75130052	75130500	intron (NM	intron (NM	33133	NM_01051	16001	Igf1r	A330103N2
chr14	28669327	28669500	intron (NM	intron (NM	233785	NM_17781	238988	Erc2	6430531D0
chr7	1.34E+08	1.34E+08	promoter-T	promoter-T	57	NM_00116	233870	Tufm	2300002G0
chr1	1.01E+08	1.01E+08	Intergenic	Intergenic	300062	NM_02758	70866	Slco6d1	4921511I0
chr16	16565977	16566150	Intergenic	MTD|LTR|I	-5774	NM_13923	224014	Fgd4	9030023J0
chr13	56236177	56236275	intron (NM	CpG	685	NM_01201	26914	H2afy	H2AF12M|
chr12	1.07E+08	1.07E+08	Intergenic	Intergenic	-23456	NM_00974	12062	Bdkrb2	B(2)|B2|B2
chr8	58550052	58550150	intron (NM	intron (NM	130479	NM_08043	110304	Glra3	-
chr4	1.17E+08	1.17E+08	intron (NM	intron (NM	22250	NM_00111	108067	Eif2b3	1190002P1
chr16	38679177	38679250	intron (NM	intron (NM	33935	NM_02026	12549	Arhgap31	5830477L0
chr15	97173377	97173625	intron (NM	MTD|LTR|I	-95783	NM_00116	105827	Amigo2	AI415330|A
chr14	73657152	73657275	intron (NM	intron (NM	19515	NM_17511	67168	Lpar6	2610302I0

chr17	71335277	71335350	intron (NM	B1_Mur3|S	4543	NM_02340	67938	Myl12b	1500001M
chr8	1.29E+08	1.29E+08	Intergenic	Intergenic	93733	NM_02421	67952	Tomm20	1810060K0
chr10	61912352	61912625	promoter-T	promoter-T	-47	NM_18142	338359	Supv3l1	6330443E1
chr12	81891577	81891725	5' UTR (NM	5' UTR (NM	132	NM_00124	217684	4933426M	mKIAA0247
chr1	72466902	72467150	Intergenic	MLT1K|LTR	113031	NM_00953	22596	Xrcc5	AI314015|I
chr1	10251477	10251925	Intergenic	Intergenic	-28950	NM_00110	211673	Arfgef1	ARFGEP1|E
chr10	19524502	19524775	Intergenic	Intergenic	46627	NM_02952	76157	Slc35d3	6230421J1
chr1	1.36E+08	1.36E+08	promoter-T	promoter-T	-387	NM_02058	57439	Tmem183a	1300007B1
chr2	34999377	34999475	intron (NM	intron (NM	11672	NM_00129	26920	Cntrl	6720467O0
chr1	1.54E+08	1.54E+08	intron (NM	L1M3|LINE	131737	NM_19799	69399	1700025G0	2610510E0
chr11	1.14E+08	1.14E+08	intron (NM	CpG	558	NM_01358	16834	Cog1	6030445I1
chr3	1.08E+08	1.08E+08	intron (NM	intron (NM	3503	NM_01035	14862	Gstm1	Gstb-1|Gst
chr9	44607177	44607450	promoter-T	promoter-T	-14	NM_02786	71687	Tmem25	0610039J0
chr14	45775352	45775700	intron (NM	MT2A|LTR	63543	NM_17259	70561	Txndc16	5730420B2
chr9	68891702	68892075	intron (NM	intron (NM	-245760	NM_00128	19883	Rora	9530021D1
chr11	1.17E+08	1.17E+08	intron (NM	ID_B1|SINE	-15884	NM_00111	53860	9-Sep	MSF1|Msf
chr1	39811327	39811525	Intergenic	Intergenic	-33592	NM_00114	619289	Rfx8	4933400N1
chr10	90923402	90923575	Intergenic	Intergenic	-289124	NM_00108	21917	Tmpo	5630400D2
chr7	29527127	29527200	promoter-T	promoter-T	176	NM_02363	71984	Sars2	2410015F0
chr11	1.2E+08	1.2E+08	intron (NM	RLTR16|LT	-17078	NM_02798	71885	2310003H0	Faap100
chr18	78033927	78034000	intron (NM	intron (NM	674	NM_01383	19201	Pstpip2	MAYP|cmc
chr17	35137077	35137200	promoter-T	promoter-T	-714	NM_01169	22321	Vars	Bat6|D17H
chr8	1.23E+08	1.23E+08	Intergenic	Intergenic	17906	NM_00129	12857	Cox4i1	AL024441|
chr10	36694302	36694600	5' UTR (NM	5' UTR (NM	101	NM_00822	15182	Hdac2	D10Wsu17
chr4	1.41E+08	1.41E+08	exon (NM_	exon (NM_	346	NM_00101	68817	Ddi2	1110056G1
chr17	57096852	57096950	intron (NM	intron (NM	-22090	NM_02232	64144	Mllt1	AA407901|
chr10	87970652	87971025	Intergenic	Intergenic	-4123	NM_00114	212862	Chpt1	-
chr9	61210927	61211300	Intergenic	L1MB2|LIN	-9060	NM_00108	21887	Tle3	2610103N0
chr4	95007627	95007975	Intergenic	Intergenic	-216397	NM_00111	75578	Fggy	2310009E0
chr1	1.59E+08	1.59E+08	intron (NM	intron (NM	17542	NM_00115	78255	Ralgps2	1810020P1
chr8	1.28E+08	1.28E+08	intron (NM	intron (NM	42785	NM_02563	66566	Ntpcr	2310079N0
chr7	35826352	35826575	intron (NM	intron (NM	15122	NM_00988	12611	Cebpg	C/EBP[g]|C
chr17	34067902	34068000	intron (NM	intron (NM	1112	NM_00905	19732	Rgl2	KE1.5|Rab2
chr1	1.09E+08	1.09E+08	Intergenic	Intergenic	-9137	NM_00117	18788	Serpinb2	PAI-2|Plan
chr14	57173527	57173850	intron (NM	PB1D7|SIN	16995	NM_00101	219103	Cenpj	4932437H0
chr7	4676177	4676275	intron (NM	intron (NM	458	NM_00114	330460	Tmem150b	A630041N1
chr5	1.37E+08	1.37E+08	Intergenic	Intergenic	-50753	NM_02161	59310	Myl10	1700027I0
chr2	29744802	29745025	promoter-T	promoter-T	-327	NM_00117	18286	Odf2	AI848335|I
chr1	1.93E+08	1.93E+08	intron (NM	intron (NM	136411	NM_17877	320119	Rps6kc1	AA682037|
chr1	66888502	66888725	intron (NM	intron (NM	21270	NM_00738	11363	Acadl	AA960361|
chr14	55331827	55332000	promoter-T	promoter-T	-902	NM_20713	110794	Cebpe	C/EBPe|CR
chr2	3458652	3458875	Intergenic	MIR|SINE|	28426	NM_17764	227526	Cdnf	9330140G2
chr1	95374677	95374825	promoter-T	promoter-T	743	NM_13380	110611	Hdlbp	1110005P1
chr14	27773752	27773975	intron (NM	intron (NM	9874	NM_14522	72993	Appl1	2900057D2
chr5	1.44E+08	1.44E+08	intron (NM	CpG	339	NM_02584	66913	Kdelr2	1110007A1
chr7	19894677	19894800	exon (NM_	exon (NM_	656	NM_00803	14282	Fosb	-
chr15	1.02E+08	1.02E+08	intron (NM	intron (NM	628	NM_00931	21357	Tarbp2	Prbp|TRBP

chr1	1.58E+08	1.58E+08	intron (NM	intron (NM	3058	NM_00923	20652	Soat1	8430426K1
chr3	88139702	88139875	promoter-T	promoter-T	-394	NM_17824	229512	Smg5	BC024683|
chr1	59741852	59742050	5' UTR (NM	5' UTR (NM	101	NM_01886	55989	Nop58	MSSP|Nol5
chr4	46384277	46384350	Intergenic	Intergenic	17982	NM_02908	74753	5830415F0	AV014846|
chr19	10630552	10630925	promoter-T	promoter-T	-10	NM_02679	68642	Tmem216	1110017C2
chr1	1.82E+08	1.82E+08	intron (NM	intron (NM	720	NM_00741	11545	Parp1	5830444G2
chr14	21474177	21474350	intron (NM	intron (NM	1499	NM_02150	59011	Myoz1	2310001N1
chr9	32327727	32328150	intron (NM	intron (NM	21099	NM_00802	14247	Fli1	EWSR2|Fli-
chr17	8109952	8110225	Intergenic	MLT1H|LTI	-8777	NM_14596	72536	Tagap	2610315E1
chr13	9912827	9913200	intron (NM	intron (NM	-148453	NM_14451	66505	Zmynd11	2210402G2
chr2	21435427	21435725	intron (NM	intron (NM	146382	NM_00100	241263	Gpr158	5330427M
chr18	46905652	46905725	intron (NM	intron (NM	4117	NM_00968	11777	Ap3s1	[s]3A
chr10	93296627	93296875	intron (NM	CpG	2451	NM_00120	327799	Usp44	E430004F1
chr11	72420952	72421025	5' UTR (NM	5' UTR (NM	225	NM_02598	67128	Ube2g1	2700059C1
chr12	73769002	73769175	Intergenic	Intergenic	-3273	NM_02552	66375	Dhrs7	2310016E2
chr11	1.18E+08	1.18E+08	Intergenic	Intergenic	5592	NM_00770	12702	Socs3	Cis3|Cish3
chr1	37401177	37401750	intron (NM	intron (NM	-24599	NM_00129	269180	Inpp4a	107kDa|96
chr11	53243702	53243875	intron (NM	intron (NM	545	NM_02535	22272	Uqcrq	1100001F0
chr4	1.41E+08	1.41E+08	intron (NM	MTEa|LTR|	5583	NM_00101	68817	Ddi2	1110056G1
chr11	11630352	11630625	intron (NM	intron (NM	44272	NM_00102	22778	Ikzf1	5832432G1
chr5	16156077	16156375	Intergenic	Intergenic	96507	NM_00128	15234	Hgf	C230052L0
chr3	95477777	95477850	Intergenic	Intergenic	13968	NM_14489	229595	Adamtsl4	Tsrc1
chr15	94373502	94373750	promoter-T	promoter-T	312	NM_17243	78895	Pus7l	3000003F0
chr7	1.21E+08	1.21E+08	intron (NM	intron (NM	90189	NM_14558	233744	Spon1	AI666765|/
chr15	59479977	59480050	promoter-T	promoter-T	-196	NM_14454	211770	Trib1	A530090O1
chr1	63223277	63223675	promoter-T	promoter-T	71	NM_01879	55949	Eef1b2	2810017J0
chr13	37748852	37748975	Intergenic	MER58B|D	-168994	NM_02683	68750	Rreb1	1110037N0
chr11	1.01E+08	1.01E+08	intron (NM	intron (NM	1149	NM_21366	20848	Stat3	1110034C0
chr11	77305252	77305350	Intergenic	CpG	-1613	NM_00100	216963	Git1	Cat-1|p95C
chr10	95097477	95097600	Intergenic	Intergenic	-70737	NM_02772	71207	Nudt4	4933436C1
chr7	1.1E+08	1.1E+08	5' UTR (NM	5' UTR (NM	192	NM_00910	20133	Rrm1	RnrM1
chr15	73491452	73491650	Intergenic	Intergenic	-36377	NM_00103	106068	Slc45a4	9330175B0
chr1	43870952	43871275	intron (NM	intron (NM	13440	NM_02643	67883	Uxs1	1600025I1
chr10	87819852	87819975	promoter-T	promoter-T	-93	NM_02787	71712	Dram1	1200002N1
chr10	40602127	40602275	intron (NM	intron (NM	748	NM_02787	71713	Cdc40	1200003H2
chr11	78959502	78959625	exon (NM_	exon (NM_	293	NM_01357	16706	Ksr1	AW492498
chr1	66749327	66749425	intron (NM	intron (NM	1909	NM_02568	66646	Rpe	2810429B0
chr5	1.4E+08	1.4E+08	intron (NM	CpG	211	NM_01075	17135	Mafk	AW061068
chr18	35091952	35092025	promoter-T	promoter-T	-331	NM_14486	225363	Etf1	AI463371|I
chr19	12519477	12519875	Intergenic	Lx9|LINE|L	-15593	NM_01082	17476	Mpeg1	MPS1|Mpg
chr11	61307177	61307350	exon (NM_	exon (NM_	505	NM_00129	23939	Mapk7	BMK-1|BM
chr1	1.73E+08	1.73E+08	promoter-T	promoter-T	-696	NM_01948	56009	Alyref2	C130042O1
chr18	55006077	55006500	Intergenic	Intergenic	143546	NM_17575	269023	Zfp608	4932417D1
chr15	76734127	76735900	exon (NM_	exon (NM_	512	NM_01205	26961	Rpl8	-
chr8	1.14E+08	1.14E+08	promoter-T	promoter-T	94	NM_17228	234733	Ddx19b	2810457M
chr8	1.13E+08	1.13E+08	Intergenic	RMER19B|	-8539	NM_00917	20444	St3gal2	AI429591|/
chr15	99090052	99090275	intron (NR_	intron (NR_	-7771	NM_01676	51812	Mcrs1	C78274|ICI

chr2	1.74E+08	1.74E+08	intron (NM	intron (NM	2755	NM_02232	64138	Ctsz	AI787083|/
chr2	1.53E+08	1.53E+08	Intergenic	Intergenic	7168	NM_01022	14239	Foxs1	FREAC10|F
chr2	1.35E+08	1.35E+08	Intergenic	B3|SINE|B.	-48169	NM_02914	52837	Tmx4	2810417D0
chr3	1.44E+08	1.44E+08	Intergenic	Intergenic	63395	NM_00116	16911	Lmo4	A730077C1
chr6	1.07E+08	1.07E+08	promoter-T	promoter-T	31	NM_00124	70047	Trnt1	2410043H2
chr2	1.7E+08	1.7E+08	Intergenic	MLT2B3|LT	-21576	NM_00115	228913	Zfp217	4933431C0
chr2	1.7E+08	1.7E+08	Intergenic	Intergenic	-76576	NM_00115	228913	Zfp217	4933431C0
chr17	23729752	23729850	intron (NM	CpG	6653	NM_01174	22654	Zfp13	4933429B2
chr2	33726552	33727075	intron (NM	intron (NM	16653	NM_17518	72543	Mvb12b	2610200O1
chr5	1.18E+08	1.18E+08	intron (NM	intron (NM	15519	NM_02812	72151	Rfc5	2610020K0
chr14	31237777	31237850	intron (NM	intron (NM	-54938	NM_13376	75901	Dcp1a	1110066A2
chr13	3895052	3895250	intron (NM	intron (NM	-2324	NM_00104	56349	Net1	Net1a|mN
chr7	1.06E+08	1.06E+08	Intergenic	CpG	-19759	NM_00128	22411	Wnt11	-
chr1	13363102	13363325	intron (NM	CpG	951	NM_00107	17978	Ncoa2	9530095N1
chr14	79850377	79850450	Intergenic	RMER12|LT	-1229	NM_00103	432879	Zbtbd6	EG432879|
chr7	1.35E+08	1.35E+08	promoter-T	promoter-T	-51	NM_14625	244219	Zfp668	BC030314|
chr5	53060577	53060825	intron (NM	CpG	239	NM_17249	211006	Sepsecs	9130208G1
chr16	36183952	36184200	promoter-T	promoter-T	-222	NM_00108	433016	Gm5483	EG433016
chrX	1.31E+08	1.31E+08	intron (NM	intron (NM	19428	NM_01348	12229	Btk	AI528679|:
chr17	47398027	47398275	intron (NM	intron (NM	30972	NM_17262	224829	Trerf1	9430096I18
chr17	57419852	57419925	intron (NM	intron (NM	1365	NM_00116	22324	Vav1	Vav|vav-T
chr9	90128377	90128500	intron (NM	intron (NM	37169	NM_19433	67016	Tbc1d2b	1810061M
chr8	1.25E+08	1.25E+08	Intergenic	Intergenic	-2759	NM_01391	30927	Snai3	AI643946|!
chr5	64525227	64525425	Intergenic	Intergenic	-26173	NM_01963	57915	Tbc1d1	1110062G0
chr12	58604077	58604375	Intergenic	L1_Rod|LIN	42882	NM_00825	15375	Foxa1	Hnf-3a|Hn
chr6	50495352	50495900	Intergenic	Intergenic	20847	NM_00780	13063	Cycs	-
chr5	44537752	44537925	Intergenic	L1MB5|LIN	-44863	NM_00116	19126	Prom1	4932416E1
chrX	33575627	33575750	intron (NM	L1MB7|LIN	-76446	NM_13399	16164	Il13ra1	AI882074|(
chr17	47731352	47731425	intron (NM	intron (NM	972	NM_00763	12445	Ccnd3	9230106B0
chr14	51708827	51708900	Intergenic	Intergenic	-1889	NM_00116	11727	Ang	AI385586|/
chr13	56804527	56804875	promoter-T	promoter-T	288	NM_00116	17129	Smad5	1110051M
chr12	41750777	41750975	intron (NM	CpG	199	NM_05312	93757	Immp2l	AI853880|I
chr11	1.15E+08	1.15E+08	promoter-T	promoter-T	223	NM_02530	50529	Mrps7	MRP-S7|Rp
chr7	52972902	52974825	promoter-T	promoter-T	422	NM_00907	19899	Rpl18	L18|Rpl18a
chr19	45005677	45005875	5' UTR (NN	5' UTR (NN	167	NM_00108	226151	Fam178a	3632432H1
chr17	36033752	36034100	TTS (NM_0	TTS (NM_0	397	NM_00103	69662	2310061I04	-
chr7	14606602	14606850	Intergenic	RLTR45|LT	-31789	NM_00110	434121	Sult2a4	EG434121|
chr9	1.07E+08	1.07E+08	5' UTR (NN	5' UTR (NN	525	NM_01970	56395	Tmem115	C78915|Pl(
chr6	1.3E+08	1.3E+08	Intergenic	Intergenic	-48171	NM_00101	232415	Gm156	Klrh1
chr11	1.11E+08	1.11E+08	Intergenic	Intergenic	-265759	NM_01060	16517	Kcnj16	6430410F1
chr11	1.07E+08	1.07E+08	intron (NM	intron (NM	29223	NM_17685	207165	Bptf	9430093H1
chr4	49689727	49689925	intron (NM	intron (NM	5029	NM_00100	19059	Ppp3r2	CaNB2
chr11	84693677	84693850	promoter-T	promoter-T	24	NM_00107	70325	Pigw	2610044A1
chr8	93594002	93594125	5' UTR (NN	5' UTR (NN	107	NM_00128	19651	Rbl2	PRB2|RBR-
chr7	1.33E+08	1.33E+08	promoter-T	promoter-T	426	NM_20723	233863	Gtf3c1	-
chr6	90272127	90272225	intron (NM	intron (NM	3003	NM_02792	71797	Chst13	1110067M
chr2	1.44E+08	1.44E+08	intron (NM	L1MB7|LIN	21171	NM_01977	56431	Dstn	2610043P1

chr2	1.3E+08	1.3E+08	non-coding	non-coding	360	NM_00103	67333	Stk35	1700054C1
chr1	1.72E+08	1.72E+08	intron (NM	intron (NM	45024	NM_02256	18214	Ddr2	AW495251
chr6	52692327	52692500	intron (NM	intron (NM	28690	NM_02581	52440	Tax1bp1	1200003J1
chr3	87733877	87734000	promoter-T	promoter-T	179	NM_15356	229503	Rrnad1	-
chr8	1.09E+08	1.09E+08	intron (NM	intron (NM	-9590	NM_01090	18021	Nfatc3	C80703|D8
chr4	40103202	40103575	intron (NM	intron (NM	13090	NM_00738	11428	Aco1	Aco-1|Ireb
chr1	1.35E+08	1.35E+08	Intergenic	CpG	32858	NM_13381	108954	Ppp1r15b	1810033K1
chr9	1.15E+08	1.15E+08	Intergenic	RCHARR1|I	-6862	NM_02422	68292	Stt3b	1300006C1
chr11	57332052	57332125	promoter-T	promoter-T	58	NM_00116	67726	Fam114a2	1810073G1
chr1	1.58E+08	1.58E+08	5' UTR (NM	5' UTR (NM	197	NM_14479	208263	Tor1aip1	LAP1|Lap1
chr4	43455752	43456175	5' UTR (NM	5' UTR (NM	814	NM_01157	21754	Tesk1	AI326901
chr3	35645452	35645700	Intergenic	Intergenic	-7484	NM_02957	76295	Atp11b	1110019I1
chr19	34979952	34980025	Intergenic	Intergenic	-16860	NM_18304	240641	Kif20b	33cex|B13
chr10	87799227	87799375	intron (NM	MER31A|L	20519	NM_02787	71712	Dram1	1200002N1
chr13	76718627	76718900	intron (NM	intron (NM	196354	NM_03017	78771	Mctp1	2810465F1
chr4	1.35E+08	1.35E+08	intron (NM	intron (NM	14386	NM_03018	78806	Stpg1	4930403G1
chr7	1.09E+08	1.09E+08	intron (NM	intron (NM	431	NM_01053	16068	Il18bp	IL-18BP|Igi
chr8	1.29E+08	1.29E+08	Intergenic	Intergenic	-67877	NM_00116	270110	Irf2bp2	E130305N2
chr4	8839927	8840000	Intergenic	Intergenic	222410	NM_00127	320790	Chd7	A730019I0
chr1	1.93E+08	1.93E+08	Intergenic	CpG	27545	NM_03006	381319	Batf3	9130211I0
chr18	61336052	61336250	intron (NM	intron (NM	552	NM_17827	106894	Hmgxb3	2510002C1
chr11	94580027	94580100	Intergenic	Intergenic	-14250	NM_00100	432589	Gm11541	OTTMUSG0
chr10	84379677	84379800	promoter-T	promoter-T	-620	NM_00101	237422	Ric8b	BC051080|
chr3	40548827	40548925	promoter-T	promoter-T	-659	NM_01102	18415	Hspa4l	94kDa|AI4
chr7	1.48E+08	1.48E+08	Intergenic	MTC|LTR|I	-5447	NM_00103	213002	Ifitm6	A330075D0
chr19	47845027	47845125	intron (NM	intron (NM	38830	NM_02637	67788	Sfr1	6330577E1
chr17	36006952	36007025	intron (NM	intron (NM	3428	NM_17524	76448	Ppp1r18	2310014H0
chr1	93294602	93294825	intron (NM	CpG	647	NM_02334	67444	Ilkap	0710007A1
chr6	1.49E+08	1.49E+08	5' UTR (NM	5' UTR (NM	265	NM_00128	67246	2810474O1	6720435I2
chr13	98913052	98913200	intron (NM	intron (NM	62994	NM_01202	110596	Arhgef28	9230110L0
chr19	10364702	10364850	intron (NM	intron (NM	14591	NM_19811	269060	Dagla	Nsddr
chr4	82504927	82505000	intron (NM	intron (NM	602	NM_02664	68268	Zdhhc21	9130404H1
chr2	1.21E+08	1.21E+08	promoter-T	promoter-T	-323	NM_02689	68968	Cdan1	1500015A0
chr1	39559627	39559750	Intergenic	Intergenic	-24096	NM_01877	54610	Tbc1d8	AD3|HBLP1
chr3	51013477	51014000	Intergenic	L1MD1|LIN	-14631	NM_00983	12457	Ccrn4l	AU043840
chr14	49066102	49066300	exon (NM_	exon (NM_	174	NM_17260	218989	Tmem260	5930435P1
chr9	1.09E+08	1.09E+08	Intergenic	L1MB4|LIN	-10573	NM_02540	22273	Uqcrc1	1110032G1
chr5	1.46E+08	1.46E+08	Intergenic	Intergenic	-2361	NM_00108	666311	Zscan25	EG666311|
chr13	59330477	59330675	Intergenic	MLT1A1|LT	315236	NM_00128	67269	Agtpbp1	1700020N1
chr4	1.5E+08	1.5E+08	intron (NM	intron (NM	13435	NM_01974	56485	Slc2a5	AI526984|
chr12	1.19E+08	1.19E+08	promoter-T	promoter-T	-371	NM_14604	217946	Cdca7l	BC006933|
chr6	1E+08	1E+08	Intergenic	Intergenic	-54361	NM_01974	56353	Rybp	2410018J2
chr14	32497427	32497575	Intergenic	Intergenic	43258	NM_02529	26363	Btd	-
chr2	1.03E+08	1.03E+08	5' UTR (NM	5' UTR (NM	321	NM_17889	99382	Abtb2	AW539457
chr11	1.07E+08	1.07E+08	intron (NM	CpG	620	NM_00784	13207	Ddx5	2600009A0
chr1	91350852	91350950	promoter-T	promoter-T	-485	NM_17811	347722	Agap1	Centg2|Gg
chr4	1.51E+08	1.51E+08	intron (NM	CpG	565	NM_02872	74035	Nol9	4632412I2

chr12	66067527	66067625	exon (NM_	exon (NM_	847	NM_17780	328108	Fam179b	A430041B(
chr2	1.21E+08	1.21E+08	intron (NM	L1MD3|LIN	3069	NM_02879	74176	Tgm5	2310007C0
chr4	1.18E+08	1.18E+08	promoter-T	promoter-T	-265	NM_02322	107995	Cdc20	2310042N(
chr15	66934677	66934875	3' UTR (NM	3' UTR (NM	73668	NM_00917	20442	St3gal1	5330418N2
chr6	1.32E+08	1.32E+08	promoter-T	promoter-T	-382	NM_02050	57252	Tas2r105	T2R05|T2R
chr4	1.3E+08	1.3E+08	intron (NM	CpG	227	NM_00115	80912	Pum1	AA517475|
chr4	47961677	47961975	Intergenic	Intergenic	-102294	NM_01574	18124	Nr4a3	AI573420|(
chr18	4375577	4375950	exon (NM_	exon (NM_	173	NM_02615	67440	Mtpap	0610027A1
chr13	97717652	97717775	3' UTR (NM	3' UTR (NM	23069	NM_00116	218476	Gcnt4	C2gnt3|Gn
chr6	67483252	67483325	TTS (NM_0	TTS (NM_0	2528	NM_02004	56753	Tacstd2	C80403|EG
chr5	51812202	51812350	Intergenic	Intergenic	132884	NR_02771(	19017	Ppargc1a	A830037N(
chr15	36324777	36325000	Intergenic	Intergenic	101658	NM_17513	68839	Ankrd46	1110054N(
chr5	67795752	67796200	intron (NM	intron (NM	23062	NM_00116	666938	Bend4	D330027G2
chr11	20149527	20149725	promoter-T	promoter-T	-199	NM_17226	216543	Cep68	6030463E1
chr16	33966352	33966575	intron (NM	intron (NM	626	NM_00947	22247	Umps	1700095D2
chr3	86816552	86816650	Intergenic	RLTR13C1|	-13339	NM_00763	12479	Cd1d1	AI747460|(
chr10	21096452	21096575	promoter-T	promoter-T	-593	NM_17871	237320	Aldh8a1	Raldh4
chr9	1.22E+08	1.22E+08	Intergenic	B1F|SINE|/	-2066	NM_17867	215474	Sec22c	4932412K2
chr10	57961027	57961250	intron (NM	intron (NM	453	NM_00116	76138	Ccdc138	6230424H(
chr9	1.1E+08	1.1E+08	promoter-T	promoter-T	-143	NM_00992	12796	Camp	CAP18|Cnl
chr3	14138477	14138550	intron (NM	intron (NM	192141	NM_00116	76897	Ralyl	0710005M
chr7	1.03E+08	1.03E+08	intron (NM	intron (NM	2379	NM_01185	23966	Tenm4	Doc4|ELM2
chr10	53141477	53141600	Intergenic	Intergenic	-41881	NM_00120	1E+08	Cep85l	ENSMUSG(
chr15	79914077	79914150	promoter-T	promoter-T	-277	NM_01376	27367	Rpl3	F2|J1
chr6	1.36E+08	1.36E+08	Intergenic	MLT1L|LTF	-43484	NM_01942	54343	Atf7ip	2610204M
chr5	92934302	92934375	5' UTR (NM	5' UTR (NM	296	NM_00764	12492	Scarb2	9330185J1
chr16	16303477	16303575	non-coding	non-coding	468	NM_19824	70120	Yars2	2210023C1
chr14	69902177	69902300	intron (NM	intron (NM	922	NM_02633	67712	Slc25a37	1700020E2
chr13	88599127	88599500	Intergenic	Zaphod|DN	-361764	NM_01010	13612	Edil3	Del-1|Del1
chr14	56386402	56386525	exon (NM_	exon (NM_	1664	NM_00851	16995	Ltb4r1	BLT1|BLTR
chr3	85945377	85947025	intron (NM	CpG	389	NM_01695	20091	Rps3a1	Rps3a
chr9	44795127	44795200	TTS (NM_0	TTS (NM_0	5294	NM_01348	12500	Cd3d	T3d
chr6	1.37E+08	1.37E+08	3' UTR (NM	3' UTR (NM	1032	NM_17768	232440	H2afj	E130307C1
chr3	1E+08	1E+08	intron (NM	intron (NM	66808	NM_01076	17156	Man1a2	AI428775|/
chr6	1.18E+08	1.18E+08	Intergenic	Intergenic	-17982	NM_00116	98758	Hnrnpf	4833420I2(
chr14	55195852	55195925	exon (NM_	exon (NM_	610	NM_01059	16475	Ajuba	Jub
chr11	69572927	69573025	promoter-T	promoter-T	-841	NM_00129	20020	Polr2a	220kDa|Rp
chr6	23673902	23674025	intron (NM	intron (NM	-68827	NM_02775	71300	Rnf148	4933432M
chr5	1.22E+08	1.22E+08	Intergenic	Intergenic	-20530	NM_00912	20239	Atxn2	9630045M
chr7	53171877	53172000	exon (NM_	exon (NM_	4281	NM_00114	13732	Emp3	H-4|H4|HN
chr1	1.97E+08	1.97E+08	promoter-T	promoter-T	51	NM_01349	12946	Cr1l	Crry|Mcp|
chr12	81164827	81165075	Intergenic	Intergenic	49049	NM_00756	12192	Zfp36l1	AW742437
chr1	94033752	94034025	intron (NM	intron (NM	11082	NM_20722	208727	Hdac4	4932408F1
chr8	1.14E+08	1.14E+08	promoter-T	promoter-T	-314	NM_00116	170737	Znrf1	B830022L2
chr7	52008152	52008300	intron (NM	intron (NM	4227	NM_13394	101568	Vrk3	AI428238
chr12	74386552	74386625	intron (NM	intron (NM	1110	NM_02958	76357	Trmt5	2610027O1
chr4	1.06E+08	1.06E+08	promoter-T	promoter-T	-92	NM_18322	329908	Usp24	2700066K(

chrX	1.21E+08	1.21E+08	Intergenic	Intergenic	468943	NM_00110	1E+08	Vmn2r121	EG625699
chr1	36366577	36366675	intron (NM	intron (NM	2048	NM_00117	214855	Arid5a	D430024K2
chr6	1.25E+08	1.25E+08	promoter-T	promoter-T	-273	NM_01370	22225	Usp5	AA407472|
chr3	60300952	60301025	intron (NM	intron (NM	-4186	NM_00125	56758	Mbnl1	Mbnl|mKIA
chr2	92886127	92886550	promoter-T	promoter-T	-37	NM_00117	1E+08	Prdm11	8030443D0
chr13	5860127	5860200	promoter-T	promoter-T	-572	NM_01180	23849	Klf6	AI448727|
chr2	1.31E+08	1.31E+08	exon (NM_	exon (NM_	315	NM_15350	74450	Pank2	4933409I19
chr7	1.33E+08	1.33E+08	intron (NM	intron (NM	27651	NM_20723	233863	Gtf3c1	-
chr4	1.34E+08	1.34E+08	Intergenic	Intergenic	-3538	NM_01370	23833	Cd52	AI463198|
chr5	1.24E+08	1.24E+08	Intergenic	Intergenic	-17607	NM_17787	330192	Vps37b	2300007F2
chr3	88355752	88355900	intron (NM	B1_Mus1|S	1023	NM_03124	83409	Lamtor2	2010111E0
chr11	1.01E+08	1.01E+08	intron (NM	intron (NM	2934	NM_00115	21428	Mlx	Tcfl4|bHLH
chr15	73563027	73563225	intron (NM	intron (NM	7766	NM_00116	19245	Ptp4a3	AV088979|
chr11	31269452	31269575	intron (NM	CpG	548	NM_01149	20856	Stc2	AW125853
chr4	1.36E+08	1.36E+08	5' UTR (NM	5' UTR (NM	129	NM_17773	242705	E2f2	9230110J1
chr10	1.27E+08	1.27E+08	promoter-T	promoter-T	67	NM_00892	19075	Prim1	AI324982
chr1	1.3E+08	1.3E+08	Intergenic	Intergenic	-45083	NM_14550	226414	Dars	5730439G1
chr17	48388402	48388475	intron (NM	intron (NM	1542	NM_02140	58218	Trem3	BB134760
chr11	78901652	78901900	intron (NM	intron (NM	58080	NM_01357	16706	Ksr1	AW492498
chr1	1.33E+08	1.33E+08	Intergenic	Intergenic	-1058	NM_01875	54354	Rassf5	1300019G2
chr11	89933152	89933325	Intergenic	Intergenic	41576	NM_02643	67888	Tmem100	1810057C1
chr9	65430902	65431050	Intergenic	Intergenic	-3082	NM_15311	102595	Plekho2	AI840980|
chr16	17759377	17760475	intron (NM	CpG	212	NM_14547	224023	Klhl22	2610318I18
chr11	1.15E+08	1.15E+08	intron (NM	intron (NM	1512	NM_00103	320534	Tmem104	C630005D0
chr12	1.1E+08	1.1E+08	exon (NM_	exon (NM_	655	NM_00953	22632	Yy1	AW488674
chr17	56442777	56443450	promoter-T	promoter-T	353	NM_00111	18140	Uhrf1	AL022808|
chrX	96332252	96332325	exon (NM_	exon (NM_	819	NM_01011	13641	Efnb1	Cek5-L|EFL
chr17	81020077	81020525	intron (NM	(TG)n|Simp	-57127	NM_00103	381113	Cdkl4	AU067824
chr4	1.32E+08	1.32E+08	Intergenic	Intergenic	27456	NM_00108	19204	Ptafr	PAFR
chr9	64188302	64189025	promoter-T	promoter-T	-83	NM_00117	213550	Dis3l	AV340375
chr1	99745152	99745325	intron (NM	intron (NM	78482	NM_02625	252876	Gin1	4930429M
chr6	1.14E+08	1.14E+08	exon (NM_	exon (NM_	-6592	NM_00103	381801	Tatdn2	AI646012|
chr4	57968127	57968275	intron (NM	intron (NM	1082	NM_01166	22166	Txn1	ADF|AW55
chr7	1.05E+08	1.05E+08	promoter-T	promoter-T	-666	NM_02367	12729	Clns1a	2610036D0
chr9	71761027	71761200	intron (NM	intron (NM	-17210	NM_00125	21406	Tcf12	A130037E0
chr4	1.33E+08	1.33E+08	exon (NM_	exon (NM_	675	NM_00108	93760	Arid1a	1110030E0
chr6	97098652	97098950	promoter-T	promoter-T	76	NM_17531	101351	Eogt	A130022J1
chr15	81789252	81789525	intron (NM	intron (NM	1972	NM_00128	29858	Pmm1	C77612
chr9	46080952	46081050	promoter-T	promoter-T	-146	NM_01175	22687	Zpr1	AI303781|Z
chr7	1.06E+08	1.06E+08	intron (NM	intron (NM	38561	NM_00982	12406	Serpinh1	BERF-1|Cb
chr3	1.33E+08	1.33E+08	Intergenic	Intergenic	-39434	NM_00104	214133	Tet2	Ayu17-449
chr10	1.15E+08	1.15E+08	promoter-T	promoter-T	273	NM_00103	216345	Zfc3h1	BC033596|
chr13	45125302	45125400	Intergenic	L1M4|LINE	-27886	NM_02577	94245	Dtnbp1	5430437B1
chr16	58670402	58670550	5' UTR (NM	5' UTR (NM	155	NM_00775	12892	Cpox	CPX|Cpo|H
chr3	95085852	95086000	promoter-T	promoter-T	72	NM_17334	229589	Prune	9230112O0
chr16	49840102	49840400	Intergenic	Intergenic	-15516	NM_01058	16423	Cd47	9130415E2
chr8	1.26E+08	1.26E+08	promoter-T	promoter-T	93	NM_02049	57247	Zfp276	AW048709

chr19	6363952	6364125	non-coding	non-coding	348	NR_122118	22668	Sf1	BBP|MZFM
chr11	77306477	77306550	promoter-T	promoter-T	-401	NM_00100	216963	Git1	Cat-1|p95C
chr8	1.25E+08	1.25E+08	intron (NM	intron (NM	2227	NM_00130	13057	Cyba	b558|nmf3
chrX	74754677	74754850	Intergenic	Intergenic	-1803	NM_02060	21372	Tbl1x	5330429M
chr3	1.18E+08	1.18E+08	intron (NM	intron (NM	641	NM_00119	76561	Snx7	2510028H0
chr6	1.25E+08	1.25E+08	intron (NM	L1M4|LINE	-7378	NM_01160	21937	Tnfrsf1a	CD120a|FF
chr1	1.79E+08	1.79E+08	promoter-T	promoter-T	-629	NM_00101	30928	Zbtb18	RP58|Zfp2
chr1	84932352	84932675	promoter-T	promoter-T	-855	NM_02792	71781	Slc16a14	1110004H1
chr1	88339227	88339400	Intergenic	CpG	16409	NM_02796	71863	1700019O1	-
chr14	31757552	31757800	Intergenic	Intergenic	-1201	NM_00840	16424	Itih1	Intin1|Itih-
chr5	1.4E+08	1.4E+08	intron (NM	intron (NM	1260	NM_17700	319772	C130050O1	-
chr10	80847202	80847350	5' UTR (NM	5' UTR (NM	103	NM_13396	102115	Dohh	1110033C1
chr6	53639677	53640025	intron (NM	intron (NM	116483	NM_17272	231991	Creb5	Crebpa|D4
chr9	1.19E+08	1.19E+08	Intergenic	Intergenic	-13228	NM_13371	69274	Ctdspl	2810418J2
chr9	72937402	72937750	intron (NM	intron (NM	-12629	NM_02589	66991	Khdc3	2410004A2
chr6	64993477	64993625	intron (NM	intron (NM	351	NM_00125	13990	Smarcad1	AV081750|
chr1	26209027	26209250	Intergenic	L1_Mus2|L	-322586	NM_17564	210933	Bai3	A830096D1
chr13	1.14E+08	1.14E+08	Intergenic	Intergenic	28846	NM_13079	170625	Snx18	Snag1
chr13	3609127	3609350	intron (NM	intron (NM	1116	NM_13406	105203	Fam208b	AI645998|
chr12	1.02E+08	1.02E+08	intron (NM	intron (NM	19356	NM_00103	104718	Ttc7b	AA408451|
chrX	99383652	99385125	promoter-T	promoter-T	-678	NM_00909	20102	Rps4x	Rps4|Rps4
chr11	17853552	17853725	exon (NM_	exon (NM_	240	NM_02657	68145	Etaa1	5730466H2
chr6	39675327	39675425	promoter-T	promoter-T	86	NM_13929	109880	Braf	9930012E1
chr2	23427377	23427475	5' UTR (NM	5' UTR (NM	198	NM_02977	76857	Spopl	4921517N0
chr5	20917627	20917775	intron (NM	Lx3_Mus|L	12794	NM_02919	75172	Ccdc146	4930528G0
chr15	59484352	59484725	intron (NM	intron (NM	4329	NM_14454	211770	Trib1	A530090O1
chr1	1.93E+08	1.93E+08	intron (NM	CpG	386	NM_00749	11910	Atf3	LRG-21
chr3	97798602	97799025	Intergenic	L2|LINE|L2	-18648	NM_01092	18129	Notch2	AI853703|
chr1	1.66E+08	1.66E+08	promoter-T	promoter-T	87	NM_02727	69962	Mettl18	2810422O2
chr8	74882477	74882575	intron (NM	intron (NM	39565	NM_00845	16598	Klf2	Lklf
chr8	28088827	28088925	intron (NM	CpG-13927	1068	NM_00111	353310	Zfp703	1110032O1
chr3	41545977	41546075	promoter-T	promoter-T	410	NM_00108	67161	Sclt1	2610207F2
chr17	79290302	79290500	intron (NM	intron (NM	-8489	NM_01116	19106	Eif2ak2	2310047A0
chr1	92949227	92949325	promoter-T	promoter-T	-745	NM_00111	16978	Lrrfip1	AU024550
chr13	22134077	22134150	promoter-T	promoter-T	705	NM_17818	319167	Hist1h2ag	-
chr10	85379302	85379400	5' UTR (NM	5' UTR (NM	339	NM_18165	72843	Prdm4	1700031E1
chr12	1.04E+08	1.04E+08	intron (NM	CpG	328	NM_17280	238386	Btbd7	5730507E0
chr5	53915477	53915600	Intergenic	Intergenic	-31480	NM_00108	19664	Rbpj	AI843960|
chr15	57967027	57967125	promoter-T	promoter-T	-439	NM_02743	70472	Atad2	2610509G1
chr14	47441602	47441825	intron (NM	CpG	204	NM_02202	63985	Gmfb	3110001H2
chr7	1.51E+08	1.51E+08	Intergenic	Intergenic	9335	NM_20349	381974	Mrgprg	Gm1098|M
chr6	72390002	72390200	promoter-T	promoter-T	-549	NM_14556	232087	Mat2a	D630045P1
chr17	80606277	80606700	intron (NM	CpG	157	NM_00119	225027	Srsf7	35kDa|943
chr15	91209477	91209600	intron (NM	intron (NM	-187300	NM_01199	26874	Abcd2	ABC39|ALD
chr7	30638552	30638625	promoter-T	promoter-T	79	NM_17373	233057	Zfp940	-
chr6	30276127	30276225	Intergenic	(A)n|Simpl	-21637	NM_00116	22214	Ube2h	1500009C2
chr1	1.12E+08	1.12E+08	Intergenic	MT2A|LTR	167187	NM_17285	241201	Cdh7	9330156F0

chr15	44451577	44451750	intron (NM	intron (NM	476	NM_01948	55960	Ebag9	AI835379|I
chr8	1.09E+08	1.09E+08	5' UTR (NM	5' UTR (NM	186	NM_17683	77411	Esrp2	9530027K2
chr14	37123552	37123700	Intergenic	L1M5|LINE	215475	NM_17500	218921	4930474N(	-
chr18	39650927	39651050	Intergenic	CpG	-4089	NM_00817	14815	Nr3c1	GR|Grl-1|G
chr1	26562702	26562850	Intergenic	RMER19C|	181529	NM_00103	210940	4931408C2	-
chr4	1.49E+08	1.49E+08	intron (NM	intron (NM	-50796	NM_00129	16561	Kif1b	A530096N(
chr8	4613552	4613700	intron (NM	intron (NM	456	NM_14559	233987	Zfp958	-
chr1	72204977	72205100	TTS (NM_0	TTS (NM_0	53843	NM_00100	381269	Mreg	Gm974|W
chr1	1.41E+08	1.41E+08	intron (NM	CpG	210	NM_02160	59125	Nek7	2810460C1
chr14	34187327	34187550	Intergenic	Intergenic	72906	NM_01670	26419	Mapk8	AI849689|J
chr11	76057277	76057400	promoter-T	promoter-T	100	NM_18326	67390	Rnmtl1	4833420N(
chr9	1.14E+08	1.14E+08	exon (NM_	exon (NM_	239	NM_00116	18571	Pdcd6ip	AI480591|A
chr2	5765552	5765725	promoter-T	promoter-T	368	NM_13383	98828	Cdc123	AA959893|
chrX	7418452	7418675	promoter-T	promoter-T	-394	NM_00129	54644	Otud5	AA407879|
chrX	10271102	10271200	Intergenic	Lx8|LINE|L	-23340	NM_02652	68041	Mid1ip1	3110038L0
chr13	47288927	47289000	promoter-T	promoter-T	-409	NM_00117	218215	Rnf144b	BC025007|
chr19	37568227	37568350	Intergenic	Intergenic	-56620	NM_17535	107371	Exoc6	4833405E0
chr6	1.2E+08	1.2E+08	promoter-T	promoter-T	-201	NR_04555{	67200	Ccdc77	2400002C2
chr2	1.49E+08	1.49E+08	Intergenic	Intergenic	-91897	NM_02140	58214	Cst10	DD72
chr5	1.49E+08	1.49E+08	Intergenic	Intergenic	-1199	NM_01022	14254	Flt1	AI323757|I
chr1	1.46E+08	1.46E+08	intron (NM	intron (NM	980	NM_01956	56207	Uchl5	5830413B1
chr15	98371602	98371800	Intergenic	Intergenic	-7001	NM_00128	69612	Kansl2	2310037I2
chr2	1.65E+08	1.65E+08	intron (NM	CpG	203	NM_14489	228869	Ncoa5	CIA
chr10	18773302	18773575	Intergenic	L1_Mur3|L	-38222	NM_00939	21929	Tnfaip3	A20|Tnfip3
chr1	92131277	92131475	Intergenic	Intergenic	30821	NM_00772	12778	Ackr3	AW541270
chr1	97236502	97236675	Intergenic	RMER19B|	26383	NM_02632	67698	Fam174a	2310044D2
chr17	74889152	74889350	intron (NM	CpG	418	NM_02641	67864	Yipf4	2310034L0
chr14	58374927	58375175	Intergenic	MER58B|D	-10091	NM_01577	50523	Lats2	4932411G(
chr16	45742902	45743050	promoter-T	promoter-T	92	NM_00127	213012	Abhd10	-
chr17	56272102	56272225	exon (NM_	exon (NM_	1112	NM_01366	20359	Sema6b	Sema|Sem
chr10	67012177	67012300	promoter-T	promoter-T	-535	NM_00100	211488	Ado	Gm237
chr17	35036252	35036325	intron (NM	intron (NM	369	NM_14583	110147	Ehmt2	Bat8|D17E
chr2	1.28E+08	1.28E+08	intron (NM	intron (NM	-68023	NM_20768	12125	Bcl2l11	1500006F2
chr12	32867502	32867775	intron (NM	MIR|SINE|	25565	NM_00114	30955	Pik3cg	5830428L0
chr14	35489202	35489325	intron (NM	intron (NM	2149	NM_00130	218914	Wapal	A530089A2
chr15	78078252	78078350	intron (NM	intron (NM	3060	NM_00867	17972	Ncf4	AI451400|p
chr13	1.13E+08	1.13E+08	intron (NM	LTR40b|LT	10298	NM_01056	16195	Il6st	5133400A(
chr4	1.16E+08	1.16E+08	intron (NM	intron (NM	19201	NM_02665	68276	Toe1	4930584N2
chr8	1.07E+08	1.07E+08	exon (NM_	exon (NM_	5032	NM_18199	1.01E+08	Cmtm1	CHLFH1a|C
chr3	1.21E+08	1.21E+08	intron (NM	intron (NM	3536	NM_14539	213603	Slc44a3	BC010552
chr14	66852802	66852925	intron (NM	intron (NM	-20474	NM_00116	19229	Ptk2b	CADTK|CAI
chr4	1.49E+08	1.49E+08	5' UTR (NM	5' UTR (NM	156	NM_17337	100198	H6pd	AI785303|(
chr4	1.2E+08	1.2E+08	intron (NM	CpG	340	NM_01388	29871	Scmh1	AI315320|A
chr10	1.28E+08	1.28E+08	promoter-T	promoter-T	-384	NM_03010	78428	Wibg	A030010B(
chr7	13595152	13595400	promoter-T	promoter-T	-127	NM_00102	232879	Zbtb45	BB161562|
chr4	1.27E+08	1.27E+08	3' UTR (NM	3' UTR (NM	-7052	NM_03025	80284	Smim12	BC003266
chr5	31190902	31191100	promoter-T	promoter-T	-224	NM_17484	231093	Agbl5	4930455N(

chr9	64130052	64130175	intron (NM	intron (NM	699	NM_02537	66131	Tipin	1110005A(
chr8	86239227	86239450	intron (NM	CpG	262	NM_19798	68278	Ddx39	2610307C2
chr4	1.37E+08	1.37E+08	promoter-T	promoter-T	-316	NM_13087	170707	Usp48	2810449C1
chr18	46763952	46764200	intron (NM	intron (NM	6718	NM_01012	13664	Eif1a	C76390|Ef:
chr14	79643277	79643375	intron (NM	RCHARR1|I	3848	NM_00103	105590	Zfp957	AU017455
chr11	1.21E+08	1.21E+08	5' UTR (NM	5' UTR (NM	158	NM_14479	209027	Pycr1	-
chr8	61437127	61437475	Intergenic	MTD-int|LT	46749	NM_17341	234290	BC030500	-
chr4	1.07E+08	1.07E+08	intron (NM	intron (NM	1063	NM_02367	72475	Ssbp3	2610021L1
chr2	1.8E+08	1.8E+08	intron (NM	intron (NM	14780	NM_14450	228983	Osbpl2	C130070J1
chr1	1.35E+08	1.35E+08	Intergenic	Intergenic	-25902	NM_00116	269116	Nfasc	AA387016|
chr6	1.35E+08	1.35E+08	intron (NM	intron (NM	60030	NM_02637	67774	Loh12cr1	5830457J2
chr2	1.72E+08	1.72E+08	Intergenic	Intergenic	97085	NM_00115	21420	Tfap2c	AA409384|
chr1	1.31E+08	1.31E+08	Intergenic	Intergenic	-194700	NM_00991	12767	Cxcr4	CD184|Cm
chr17	27770777	27771025	intron (NM	ID_B1|SINI	1286	NM_02596	67097	Rps10	2210402A(
chr7	1.33E+08	1.33E+08	5' UTR (NM	5' UTR (NM	309	NM_02881	74204	Xpo6	2610005L1
chr7	1.31E+08	1.31E+08	intron (NM	intron (NM	56218	NM_00125	628779	Hs3st4	EG628779|
chr12	72237677	72238075	promoter-T	promoter-T	41	NM_00116	76967	2700049A(	Talpid3|mI
chr1	1.72E+08	1.72E+08	intron (NM	intron (NM	30006	NM_00906	19737	Rgs5	1110070A(
chr1	1.09E+08	1.09E+08	intron (NM	RLTR19-int	10231	NM_02753	70750	Kdsr	6330410P1
chr10	20443452	20443600	intron (NM	intron (NM	1348	NM_01387	29863	Pde7b	-
chr7	29573877	29574150	promoter-T	promoter-T	25	NM_17715	320435	Rinl	5830482F2
chr12	31597027	31597125	promoter-T	promoter-T	542	NM_01370	24057	Sh3yl1	AI314953|I
chr1	1.84E+08	1.84E+08	Intergenic	Intergenic	-15705	NM_01205	27058	Srp9	9kDa
chr6	1.21E+08	1.21E+08	intron (NM	CpG	21139	NM_00127	194401	Mical3	C130040D1
chr12	52966802	52966950	Intergenic	Intergenic	-36353	NM_14478	207304	Hectd1	A630086P(
chr2	1.4E+08	1.4E+08	promoter-T	promoter-T	106	NM_02709	69487	Ndufaf5	2310003L2
chr11	1.16E+08	1.16E+08	5' UTR (NM	5' UTR (NM	117	NM_00821	15081	H3f3b	9430068D(
chr9	71507877	71508075	intron (NM	B1_Mur1|S	-67809	NM_00103	102371	Myzap	AA407270|
chr4	8839002	8839100	Intergenic	Intergenic	221498	NM_00127	320790	Chd7	A730019I0
chr17	34335127	34335375	5' UTR (NM	5' UTR (NM	111	NM_01072	16913	Psmb8	Lmp-7|Lmp
chr17	26056477	26056550	intron (NM	CpG-6110	146	NM_13915	224624	Rab40c	RAR3
chr17	26777127	26777550	intron (NM	intron (NM	-36003	NM_02527	11974	Atp6v0e	Atp6k|Atp(
chr19	39005802	39005950	intron (NM	CpG	396	NM_00823	15201	Hells	AI323785|I
chr3	84472877	84473100	intron (NM	intron (NM	2874	NM_17726	320782	Tmem154	9930117H(
chr2	11627327	11627500	promoter-T	promoter-T	74	NM_00127	16169	Il15ra	AA690181|
chr10	1.21E+08	1.21E+08	intron (NM	intron (NM	24231	NM_17733	216393	D930020B1	1500017I1
chr6	84519002	84519325	Intergenic	Intergenic	24641	NM_17547	232174	Cyp26b1	CP26|P450
chr17	73268427	73268550	intron (NM	intron (NM	843	NM_02999	77889	Lbh	1810009F1
chr1	88383977	88384400	Intergenic	CpG	-39123	NM_00897	19231	Ptma	Thym
chr9	1.19E+08	1.19E+08	promoter-T	promoter-T	-168	NM_01085	17874	Myd88	-
chr12	16532927	16533225	Intergenic	Intergenic	63500	NM_01576	14245	Lpin1	4631420P(
chr19	5972377	5972600	intron (NM	intron (NM	-8282	NM_00889	18969	Pola2	AI573378
chr1	95650602	95650850	promoter-T	promoter-T	689	NM_02592	67026	Thap4	2010320B(
chr1	1.94E+08	1.94E+08	non-coding	non-coding	-11397	NM_00957	22782	Slc30a1	AI839647|(
chr15	96874452	96874600	intron (NM	intron (NM	11861	NM_02705	69354	Slc38a4	1110012E1
chr7	35912202	35912350	Intergenic	Intergenic	7964	NM_00767	12606	Cebpa	C/ebpalpha
chr15	78859127	78859325	exon (NM_	exon (NM_	584	NM_00819	14958	H1f0	D130017D(

chr11	1.09E+08	1.09E+08	promoter-T	promoter-T	-432	NM_01030	14674	Gna13	AU024132
chr2	1.64E+08	1.64E+08	intron (NM	CpG	155	NM_02557	66460	Sys1	2610042O1
chr1	1.83E+08	1.83E+08	Intergenic	ORR1D1|LT	-24733	NM_02840	72978	Cnih3	2900075G0
chr3	88981727	88981850	TTS (NM_0	TTS (NM_0	381	NM_01188	24045	Scamp3	Sc3|TU52
chr18	66162027	66162175	exon (NM_	exon (NM_	188	NM_02740	70361	Lman1	2610020P1
chr12	87046077	87046250	intron (NM	intron (NM	18493	NM_01676	53314	Batf	B-ATF|SFA-
chr6	35127502	35128100	intron (NM	CpG	185	NM_02751	70699	Nup205	3830404O0
chr15	85062502	85062950	intron (NM	intron (NM	26288	NM_01018	14114	Fbln1	-
chr8	1.08E+08	1.08E+08	exon (NM_	exon (NM_	113	NM_14895	104394	E2f4	2010111M
chr2	1.48E+08	1.48E+08	Intergenic	Intergenic	-78805	NM_01074	17064	Cd93	6030404G0
chr6	86959902	86960000	exon (NM_	exon (NM_	121	NM_02004	56748	Nfu1	0610006G1
chr3	75360802	75360950	promoter-T	promoter-T	-102	NM_01974	56426	Pdcd10	2410003B1
chr1	38160827	38160900	intron (NM	MTD|LTR|I	25644	NM_01957	56210	Rev1	1110027I2
chr12	73750027	73750350	Intergenic	Intergenic	15627	NM_02552	66375	Dhrs7	2310016E2
chr12	21292877	21292975	promoter-T	promoter-T	768	NM_01881	54451	Cpsf3	-
chr12	8507627	8507750	promoter-T	promoter-T	-897	NM_00748	11852	Rhob	AA017882|
chr9	44152252	44152350	promoter-T	promoter-T	10	NM_01355	15288	Hmbs	PBGD|T25
chr5	75028452	75028875	intron (NM	intron (NM	45234	NM_01072	16924	Lnx1	Lnx
chr15	9695477	9695550	Intergenic	Intergenic	-16952	NM_17712	320277	Spef2	C230086A0
chr1	1.94E+08	1.94E+08	Intergenic	Intergenic	-30327	NM_17226	226856	Lpgat1	AI649174|
chr11	1.04E+08	1.04E+08	intron (NM	intron (NM	58301	NM_00128	17762	Mapt	AI413597|
chr15	96567377	96567500	Intergenic	ORR1D2|LT	-37309	NM_17512	67760	Slc38a2	5033402L1
chr7	31004852	31004950	Intergenic	Intergenic	12147	NM_02554	66411	Tbcb	2410007D1
chr11	95936002	95936075	intron (NM	MER5B|DN	907	NM_00116	11951	Atp5g1	-
chr10	98632452	98632600	intron (NM	intron (NM	61757	NM_01573	14426	Galnt4	AV011803
chr5	1.15E+08	1.15E+08	intron (NM	intron (NM	5664	NM_02671	68420	Ankrd13a	1100001D1
chr16	32595402	32595475	Intergenic	Intergenic	-13544	NM_01163	22042	Tfrc	2610028K1
chr3	9890877	9891025	Intergenic	Lx2|LINE|L	-57272	NM_00119	94212	Pag1	Cbp|F7300
chr14	55635102	55635200	intron (NM	intron (NM	860	NM_02689	68966	Ngdn	1500001L1
chr4	32702002	32702150	promoter-T	promoter-T	-372	NM_00112	26885	Casp8ap2	AA387232|
chr3	19219902	19220100	Intergenic	Intergenic	-8679	NM_00112	18583	Pde7a	AU015378
chr17	4908277	4908475	Intergenic	CpG	-86698	NM_00108	239985	Arid1b	8030481M
chr3	1.08E+08	1.08E+08	intron (NM	CpG	442	NM_18140	99512	Wdr47	1810073M
chr10	1.28E+08	1.28E+08	intron (NM	CpG	133	NM_17279	237615	Ankrd52	6430544C0
chr8	85297177	85297250	intron (NM	intron (NM	812	NM_14560	30932	Zfp330	BC008086|
chr2	90894252	90894400	5' UTR (NM	5' UTR (NM	153	NM_00894	19182	Psmc3	TBP-1
chr7	87160402	87160575	Intergenic	Intergenic	-9212	NM_00114	209225	Zfp710	5430400N0
chr1	1.23E+08	1.23E+08	intron (NM	CpG	231	NM_02586	66942	Ddx18	2310005B1
chr16	32099227	32099325	promoter-T	promoter-T	537	NM_00111	72084	Pigx	2010319C1
chr7	51654252	51654350	intron (NM	intron (NM	14805	NM_01666	20981	Syt3	AI385753|
chr4	1.16E+08	1.16E+08	exon (NM_	exon (NM_	273	NM_00104	17776	Mast2	MAST205|
chr2	29979702	29979875	intron (NM	CpG	343	NM_17869	227693	Zer1	C230075L1
chr16	23983027	23983200	intron (NM	intron (NM	5585	NM_00974	12053	Bcl6	Bcl5
chr6	17644102	17644175	5' UTR (NM	5' UTR (NM	144	NM_00128	64213	St7	9430001H0
chr8	74684477	74684850	promoter-T	promoter-T	-436	NM_02312	17274	Rab8a	AA409338|
chr8	34838577	34838700	Intergenic	MER89|LTI	-3748	NM_00116	68153	Gtf2e2	34kDa|AI4
chr8	96536427	96536500	promoter-T	promoter-T	77	NM_01178	23802	Amfr	gp78

chr2	18514027	18514175	Intergenic	Intergenic	-79988	NM_14777	12238	Commd3	AW550818
chr2	1.44E+08	1.44E+08	promoter-T	promoter-T	48	NM_00128	74528	Mgme1	8430406I0
chr9	1.03E+08	1.03E+08	promoter-T	promoter-T	-535	NM_13397	22041	Trf	AI266983|
chr14	56387752	56387875	TTS (NM_0	TTS (NM_0	3014	NM_00851	16995	Ltb4r1	BLT1|BLTR
chr5	1.2E+08	1.2E+08	Intergenic	Intergenic	-98209	NM_02876	74111	Rbm19	1200009A0
chr2	1.79E+08	1.79E+08	intron (NM	intron (NM	213055	NM_00986	12561	Cdh4	AW120700
chr12	25785777	25786000	Intergenic	CpG	-4931	NM_01049	15902	Id2	AI255428|
chr1	52289277	52289350	intron (NM	CpG	763	NM_00108	14660	Gls	6330442B1
chr13	12130977	12131300	intron (NM	intron (NM	68074	NM_02386	20191	Ryr2	9330127I2
chr5	23957327	23957625	promoter-T	promoter-T	-519	NM_13915	213990	Agap3	AGAP-3|A\
chr2	38339102	38339400	Intergenic	(CA)n|Simp	-27966	NM_02160	59126	Nek6	1300007C0
chr4	55363027	55363350	5' UTR (NM	5' UTR (NM	274	NM_00901	19359	Rad23b	0610007D1
chr1	6896027	6896150	Intergenic	Intergenic	175956	NR_04518	240690	St18	AV348974|
chr12	29319402	29321300	exon (NM_	exon (NM_	467	NM_01130	20115	Rps7	Mtu|Rps7A
chr9	44903677	44903975	intron (NM	intron (NM	16560	NM_00100	270152	Amica1	AMICA|Cre
chr7	36984152	36984300	Intergenic	Intergenic	-396218	NM_17773	668501	Zfp507	1810022O1
chr18	23833377	23833450	Intergenic	Intergenic	-77421	NM_00116	212307	Mapre2	AI314113|
chr17	5493077	5493175	5' UTR (NM	5' UTR (NM	526	NM_14607	224454	Zdhhc14	B530001K0
chr11	95611952	95612175	intron (NM	intron (NM	1682	NM_20160	268469	Zfp652	9530033F2
chr17	25965952	25966075	promoter-T	promoter-T	25	NM_02810	72106	Jmjd8	2610003J0
chr11	1.18E+08	1.18E+08	intron (NM	intron (NM	122	NM_18132	217353	Tmc6	D11Ertd20
chr17	27759877	27760000	promoter-T	promoter-T	-29	NM_00129	56409	Nudt3	1110011B0
chr10	1.27E+08	1.27E+08	intron (NM	intron (NM	374	NM_00125	108037	Shmt2	2700043D0
chr7	1.43E+08	1.43E+08	intron (NM	intron (NM	8161	NM_17866	212070	Clrn3	AI649392|
chr15	38612252	38612800	intron (NM	intron (NM	20867	NM_02549	66335	Atp6v1c1	1700025B1
chr9	64879752	64879850	promoter-T	promoter-T	-464	NM_02890	74388	Dpp8	2310004I0
chr6	1.29E+08	1.29E+08	intron (NM	intron (NM	6464	NM_17768	232413	Clec12a	CLL-1|D23
chr1	88383702	88383925	Intergenic	CpG	-39498	NM_00897	19231	Ptma	Thym
chr10	1.28E+08	1.28E+08	promoter-T	promoter-T	-473	NM_03125	83430	Il23a	IL-23|p19
chr13	85430377	85430450	Intergenic	Intergenic	-1322	NM_14545	218397	Rasa1	Gap|RasGA
chr12	1.13E+08	1.13E+08	promoter-T	promoter-T	-224	NM_17804	217869	Eif5	2810011H2
chr5	1.26E+08	1.26E+08	Intergenic	Intergenic	-3814	NM_01963	22190	Ubc	2700054O0
chr5	1.22E+08	1.22E+08	Intergenic	L1MA6|LIN	-1040	NM_17272	231713	Naa25	4833422K1
chr2	1.52E+08	1.52E+08	promoter-T	promoter-T	-52	NM_00108	24105	Rbck1	AL033326|
chr10	22451327	22451725	promoter-T	promoter-T	-273	NM_01160	237336	Tbpl1	4732475G0
chr9	1.07E+08	1.07E+08	Intergenic	Intergenic	6606	NM_00989	12700	Cish	AI385595|
chr12	16049102	16049425	Intergenic	Intergenic	-225672	NM_14455	217410	Trib2	AW319517
chr5	1.37E+08	1.37E+08	exon (NM_	exon (NM_	123	NM_02577	66801	Prkrip1	8430424D2
chr1	1.57E+08	1.57E+08	promoter-T	promoter-T	-49	NM_00114	72482	Acbd6	0610010G0
chr18	52997352	52997525	intron (NM	intron (NM	69640	NM_00119	67847	Sncaip	2810407O1
chr17	79134952	79135075	intron (NM	intron (NM	887	NM_01150	268980	Strn	AU022939
chr16	32277352	32277575	promoter-T	promoter-T	-84	NM_02735	70238	Rnf168	3110001H1
chr4	1.55E+08	1.55E+08	intron (NM	intron (NM	5270	NM_00103	433813	Pusl1	2810021I1
chr2	69885877	69886025	intron (NM	MLT1H2|L	8768	NM_17737	329421	Myo3b	A430065P1
chr17	25152277	25152750	promoter-T	promoter-T	-338	NM_02060	57354	Cramp1l	5830477H0
chr7	30066727	30067000	5' UTR (NM	5' UTR (NM	133	NM_00108	20733	Spint2	AL024025|
chr6	53420102	53420200	Intergenic	Intergenic	-103217	NM_17272	231991	Creb5	Crebpa|D4

chr1	1.3E+08	1.3E+08	exon (NM_	exon (NM_	157	NM_00856	17219	Mcm6	ASP-I1|D1\
chr11	57917052	57917550	promoter-T	promoter-T	-354	NM_02694	69125	Cnot8	1500015I0
chr7	86419802	86419875	intron (NM	MIR|SINE|	1686	NM_01881	54608	Abhd2	2210009N1
chr4	35115752	35115825	Intergenic	MTD|LTR|	-11055	NM_17806	214944	Mob3b	8430436F2
chr7	1.4E+08	1.4E+08	exon (NM_	exon (NM_	129	NM_02809	72096	Mettl10	2010208K1
chr7	16642052	16642175	Intergenic	B3|SINE|B	-15178	NM_00109	666528	Zfp541	EG666528|
chr2	1.51E+08	1.51E+08	5' UTR (NM	5' UTR (NM	103	NM_00801	14225	Fkbp1a	12kDa|FKB
chr15	80875902	80876025	intron (NM	intron (NM	59514	NM_15304	223701	Mkl1	AMKL|Bsac
chr15	76180902	76181275	promoter-T	promoter-T	452	NM_02534	106025	Sharpin	0610041B2
chr13	67406977	67407575	promoter-T	promoter-T	72	NM_00100	431706	Zfp457	Rslcan-6
chr5	1.4E+08	1.4E+08	Intergenic	Intergenic	-3353	NM_00125	77053	Sun1	4632417G1
chr1	1.58E+08	1.58E+08	Intergenic	CpG	53596	NM_00923	20652	Soat1	8430426K1
chr15	59312302	59312500	intron (NM	intron (NM	106648	NM_00116	68501	Nsmce2	1110014D1
chr18	3337802	3338100	promoter-T	promoter-T	-364	NM_00111	12916	Crem	ICER|ICERI
chr2	1.67E+08	1.67E+08	promoter-T	promoter-T	-179	NM_02381	76367	Trp53rk	2810408M
chr4	62180802	62181275	promoter-T	promoter-T	59	NM_00852	17025	Alad	ALADH|Lv
chr6	47916902	47917025	Intergenic	Intergenic	13574	NM_17889	101197	Zfp956	AI894139
chr5	1.46E+08	1.46E+08	intron (NM	CpG	160	NM_02829	72611	Zfp655	2700038I1
chr5	1.43E+08	1.43E+08	intron (NM	intron (NM	750	NM_19906	17425	Foxk1	A630048H0
chr18	58038152	58038525	promoter-T	promoter-T	6	NM_00919	20496	Slc12a2	9330166H0
chr1	21934977	21935150	intron (NM	intron (NM	16960	NM_00116	226922	Kcnq5	7730402H1
chr11	21139352	21139525	intron (NM	CpG	403	NM_00129	245944	Vps54	Hcc8|Vps5
chr16	33251577	33251750	exon (NM_	exon (NM_	121	NM_08055	69150	Snx4	1810036H1
chr1	1.27E+08	1.27E+08	intron (NM	RMER1A|C	4641	NM_02373	74117	Actr3	1200003A0
chr19	3881677	3881825	intron (NR_	intron (NR_	23479	NM_01692	27060	Tcirg1	ATP6N1C|
chr2	19273652	19273725	Intergenic	Intergenic	-19575	NM_02961	76467	Msrb2	2310050L0
chr11	68934827	68934950	intron (NM	intron (NM	933	NM_03304	84653	Hes7	bHLHb37
chr5	42006477	42006675	Intergenic	Intergenic	92818	NM_02729	100972	Rab28	2700023P0
chr4	88783077	88783150	promoter-T	promoter-T	-161	NM_02443	66902	Mtap	1300019I2
chr2	75541152	75541375	intron (NM	intron (NM	1435	NM_01090	18024	Nfe2l2	AI194320|
chr19	57527352	57527450	promoter-T	promoter-T	5	NM_02811	72133	Trub1	2610009I0
chr12	56527452	56527550	Intergenic	L1MC2|LIN	27689	NM_01196	26443	Psma6	IOTA
chr16	35983502	35983625	5' UTR (NM	5' UTR (NM	114	NM_00846	16646	Kpna1	AW494490
chr13	1.09E+08	1.09E+08	promoter-T	promoter-T	65	NM_02900	74559	Elovl7	9130013K2
chr13	23618427	23618575	Intergenic	Intergenic	-4412	NM_15317	69386	Hist1h4h	1700024H0
chr7	1.08E+08	1.08E+08	3' UTR (NM	3' UTR (NM	2474	NM_18327	68185	Coa4	5330414O0
chr12	40574827	40574900	Intergenic	Intergenic	189100	NM_00748	11861	Arl4a	AI467555|
chr7	1.21E+08	1.21E+08	intron (NR_	intron (NR_	-44833	NM_01196	26440	Psma1	C2|HC2|Pr
chr1	82792627	82793025	Intergenic	Intergenic	-27795	NM_02545	66261	Tm4sf20	1810018L0
chr1	34516627	34516975	intron (NM	CpG	210	NM_01120	19253	Ptpn18	FLP1|HSCF
chr15	73015102	73015300	exon (NM_	exon (NM_	176	NM_15317	239528	Ago2	1110029L1
chr4	1.25E+08	1.25E+08	Intergenic	Intergenic	-17388	NM_15315	230738	Zc3h12a	BC036563|
chr8	28370902	28371075	exon (NM_	exon (NM_	189	NM_00791	13685	Eif4ebp1	4e-bp1|AA
chr13	1.13E+08	1.13E+08	intron (NM	intron (NM	3540	NM_01194	26401	Map3k1	MAPKKK1|
chr8	13213577	13213650	Intergenic	Intergenic	-12989	NM_02576	66790	Grtp1	5430401C0
chrX	1.05E+08	1.05E+08	Intergenic	RLTR10-int	9524	NM_00103	213450	Gm732	-
chr16	44199427	44199675	intron (NM	intron (NM	26041	NM_00102	207806	Gm608	5530400K2

chr12	79326652	79326900	promoter-T	promoter-T	-866	NM_17295	268566	Gphn	5730552E0
chr2	1.45E+08	1.45E+08	intron (NM	L1MB8|LIN	-93676	NM_02549	66328	Scp2d1	1700010M
chr16	31150452	31150700	intron (NM	HAL1-2a_M	50748	NM_03013	78618	Acap2	4832442G1
chr15	1E+08	1E+08	Intergenic	CpG	-13255	NM_01012	19009	Pou6f1	2310038G1
chr6	39512202	39512325	Intergenic	B4A|SINE|	-4430	NM_17247	209773	Dennd2a	B930096L0
chr16	24651827	24652075	intron (NM	L1M4|LINE	-69990	NM_00114	210126	Lpp	9430020K1
chr7	31741302	31741400	intron (NM	CpG-12993	471	NM_01168	22282	Usf2	Usf-2|bHLH
chr10	1.21E+08	1.21E+08	intron (NM	intron (NM	3771	NM_00108	73192	Xpot	1110004L0
chr6	1.23E+08	1.23E+08	promoter-T	promoter-T	-89	NM_00113	11810	Apobec1	Cdar1
chr8	26773752	26773925	intron (NM	intron (NM	23082	NM_00108	234135	Whsc1l1	6720429E0
chr3	89219377	89219450	TTS (NM_0	TTS (NM_0	2800	NM_01690	54124	Cks1b	2410005G1
chr15	8410102	8410175	Intergenic	Intergenic	-15675	NM_20123	71175	Nipbl	Idn3
chr2	52784052	52784475	intron (NM	intron (NM	67361	NM_17240	71409	Fmnl2	5430425K0
chr3	89154252	89154450	promoter-T	promoter-T	-419	NM_00961	11490	Adam15	MDC15|me
chr14	48147277	48147500	intron (NR_	intron (NR_	40721	NM_17259	1.01E+08	Atg14	4832427M
chr1	54580602	54580775	intron (NM	Helitron3N	33840	NM_00116	241062	Pgap1	5033403E1
chr17	45756802	45757100	Intergenic	Intergenic	-17900	NM_00117	653016	Gm7325	EG653016|
chr7	1.14E+08	1.14E+08	Intergenic	Intergenic	93183	NM_00127	434223	Gm1966	-
chr7	78675852	78676100	Intergenic	Intergenic	775505	NM_00102	244049	Mctp2	Gm489
chr16	20517152	20517350	5' UTR (NM	5' UTR (NM	114	NM_00788	13544	Dvl3	-
chr7	1.28E+08	1.28E+08	intron (NM	intron (NM	723	NM_00126	13631	Eef2k	C86191|eE
chr19	46866477	46866600	intron (NM	MT2B|LTR	30439	NM_00110	94219	Cnnm2	AU015877
chr16	30064027	30064325	Intergenic	CpG	-1267	NM_00823	15205	Hes1	Hry|bHLHb
chr5	1.48E+08	1.48E+08	intron (NM	L1ME2|LIN	11364	NM_01022	14255	Flt3	B230315G0
chr3	54415452	54415600	Intergenic	Intergenic	-81501	NM_01999	56790	Supt20	AA667204|
chr4	45932852	45933025	intron (NM	L1MA4|LIN	29763	NM_19866	381522	E230008N1	Gm1025
chr14	21867127	21867450	intron (NM	PB1|SINE|A	4376	NM_01882	55946	Ap3m1	1200013D0
chr18	75159577	75161250	promoter-T	promoter-T	282	NM_00100	319195	Rpl17	-
chr17	71614202	71614375	intron (NM	intron (NM	46017	NM_14515	246707	Emilin2	FOAP-10
chr10	62873377	62873550	Intergenic	Intergenic	-19383	NM_17761	216033	Ctnna3	4930429L0
chr6	1.24E+08	1.24E+08	intron (NM	(CACAC)n|S	7518	NM_00127	19305	Pex5	AW212715
chr4	1.36E+08	1.36E+08	Intergenic	Intergenic	-6687	NM_01373	27224	Tceb3	110kDa|AA
chr6	38900427	38900525	intron (NM	intron (NM	31491	NM_01153	21391	Tbxas1	CYP5|CYP5
chr8	1.08E+08	1.08E+08	5' UTR (NM	5' UTR (NM	341	NM_01364	19171	Psmb10	Mecl-1|Me
chr18	24179427	24179600	promoter-T	promoter-T	-241	NM_02155	59057	Zfp191	3526401F1
chr7	1.47E+08	1.47E+08	promoter-T	promoter-T	63	NM_19930	212508	Mtg1	Gm169|Gt
chr3	65518327	65518675	intron (NM	intron (NM	48351	NM_00116	624866	Lekr1	EG546798|
chr5	37139527	37140100	exon (NM_	exon (NM_	105	NM_13372	117197	Bloc1s4	2610101N0
chr9	63868302	63868375	exon (NM_	exon (NM_	1528	NM_00854	17130	Smad6	Madh6|b2
chr6	91066802	91067025	promoter-T	promoter-T	-93	NM_01881	54563	Nup210	9830001L1
chr13	51504427	51504575	intron (NM	intron (NM	514	NM_01010	13610	S1pr3	AI132464|
chr6	1.19E+08	1.19E+08	Intergenic	Intergenic	-1375	NM_19798	68465	Adipor2	1110001I1
chr2	61431277	61431850	intron (NM	CpG	409	NM_00116	21353	Tank	C86182|E4
chr6	83065577	83065650	exon (NM_	exon (NM_	113	NM_02061	57377	Mogs	1810017N0
chr3	51505477	51505650	intron (NM	intron (NM	40448	NM_17499	211666	Mgst2	GST2|MGS
chr19	27291502	27291625	promoter-T	promoter-T	53	NM_00116	22359	Vldlr	AA408956|
chr18	25674427	25674550	intron (NM	intron (NM	237996	NM_13319	108013	Celf4	A230070D1

chr13	57147652	57147750	Intergenic	Lx8|LINE|L	-150752	NM_01203	26946	Trpc7	TRP-7|TRP
chr7	1.19E+08	1.19E+08	intron (NM	CpG	531	NM_17724	74996	Usp47	4930502N(
chr13	48965852	48966025	intron (NM	GC_rich|Lc	316	NM_01107	18676	Phf2	GRC5
chr18	32227502	32227675	5' UTR (NM	5' UTR (NM	200	NM_17344	73473	Iws1	1700069O
chr12	39592927	39593075	3' UTR (NM	3' UTR (NM	82703	NM_00116	14009	Etv1	ER81|Etsrp
chr6	37890827	37891150	intron (NM	Lx9|LINE|L	40501	NM_00127	21848	Trim24	A130082H
chr17	46210902	46211100	Intergenic	Intergenic	-36952	NM_02676	68565	Mrps18a	1110004O
chr1	1.72E+08	1.72E+08	Intergenic	RMER5|LTI	34751	NM_13380	107652	Uap1	AA420407|
chr8	1.31E+08	1.31E+08	promoter-T	promoter-T	-278	NM_01057	16412	Itgb1	4633401G
chr5	97340477	97340825	Intergenic	Intergenic	-86057	NM_08070	140780	Bmp2k	4933417M
chr17	57170552	57170650	exon (NM_	exon (NM_	329	NM_01061	16549	Khsrp	6330409F2
chr4	1.38E+08	1.38E+08	intron (NM	intron (NM	7849	NM_02845	73162	Otud3	3110030K1
chr19	54085227	54085375	intron (NM	intron (NM	-34371	NM_00741	11551	Adra2a	AW122659
chr2	1.66E+08	1.66E+08	Intergenic	Intergenic	-9130	NM_02807	72043	Sulf2	2010004N
chr18	49914952	49915075	exon (NM_	exon (NM_	242	NM_02685	68857	Dtwd2	1190002H(
chr2	1.04E+08	1.04E+08	Intergenic	Intergenic	-51201	NM_00114	16909	Lmo2	Rbtn-2|Rbt
chr12	56593677	56593800	promoter-T	promoter-T	-104	NM_01090	18035	Nfkbia	AI462015|
chr19	3376802	3377375	intron (NM	intron (NM	-11769	NM_00103	17771	Mtl5	tesmin
chr4	1.48E+08	1.48E+08	intron (NM	intron (NM	137037	NM_02719	69743	Casz1	2410019P(
chr2	35300152	35300325	intron (NM	intron (NM	16707	NM_01028	14594	Ggta1	AW108479
chr12	86814277	86814400	promoter-T	promoter-T	-513	NM_01023	14281	Fos	D12Rfj1|c-
chr10	1.11E+08	1.11E+08	exon (NM_	exon (NM_	234	NM_00934	21664	Phlda1	DT1P1B11|
chr1	74478752	74478850	intron (NM	intron (NM	22843	NM_00950	22349	Vil1	Vil
chr14	79851477	79851800	promoter-T	promoter-T	-4	NM_00103	432879	Zbtbd6	EG432879|
chr11	1.03E+08	1.03E+08	promoter-T	promoter-T	-938	NM_13875	192231	Hexim1	7330426E1
chr15	93349577	93349650	intron (NM	CpG	76709	NM_00103	106042	Prickle1	1110058P2
chr18	70708477	70708700	Intergenic	MIR3|SINE	-18613	NM_00128	26447	Poli	Rad30b
chr13	52634027	52634275	Intergenic	Intergenic	-7946	NM_00102	68203	Diras2	2900052J1
chr8	47637477	47637625	intron (NM	intron (NM	128	NM_02797	71876	Cenpu	1700029A
chr14	1.06E+08	1.06E+08	Intergenic	Intergenic	-24602	NM_01189	24064	Spry2	sprouty2
chr1	36748777	36748875	intron (NM	intron (NM	494	NM_00994	12859	Cox5b	-
chr5	76733202	76733375	intron (NM	CpG	285	NM_00771	12753	Clock	5330400M
chr18	25645202	25645500	exon (NM_	exon (NM_	267133	NM_13319	108013	Celf4	A230070D
chr7	28589502	28589725	Intergenic	Intergenic	-33990	NM_01176	22717	Zfp59	Mfg-2|Mfg
chr15	99229927	99230275	5' UTR (NM	5' UTR (NM	6451	NM_00117	110213	Tmbim6	5031406P(
chrX	1.47E+08	1.47E+08	5' UTR (NM	5' UTR (NM	130	NM_00116	382245	Tmem29	2700081K(
chr8	89412352	89412675	Intergenic	Intergenic	-3356	NM_03056	80750	N4bp1	AI481586|
chr4	1.18E+08	1.18E+08	Intergenic	MT2B2|LTI	-13923	NM_14631	18363	Olfr62	H12|IH12|
chr6	1.15E+08	1.15E+08	Intergenic	Intergenic	-5846	NM_19903	381802	Tsen2	AU067695
chr10	80957477	80957675	intron (NM	intron (NM	1532	NM_13400	103425	Ncln	3100002P1
chr3	1.31E+08	1.31E+08	Intergenic	Intergenic	-81585	NM_02894	74442	Sgms2	4933405A1
chr13	43601227	43601450	Intergenic	Intergenic	-24996	NM_01993	56705	Ranbp9	IBAP-1|Iba
chr9	37296527	37297000	promoter-T	promoter-T	-143	NM_14622	235184	Msantd2	2810450G1
chr8	1.23E+08	1.23E+08	promoter-T	promoter-T	-79	NM_13396	102193	Zdhhc7	AL024087|
chr4	71861452	71861825	promoter-T	promoter-T	289	NM_01159	21885	Tle1	C230057C(
chr5	1.41E+08	1.41E+08	intron (NM	intron (NM	3313	NM_02152	59031	Chst12	AI595374|
chr10	67587802	67587975	intron (NM	intron (NM	145542	NM_00108	170799	Rtkn2	B130039D

chrX	1.06E+08	1.06E+08	intron (NM	PB1D10|SI	47755	NM_01998	56726	Sh3bgrl	1190008F1
chr9	1.04E+08	1.04E+08	exon (NM_	exon (NM_	-66040	NM_14570	252837	Ackr4	A630091E1
chr9	1.04E+08	1.04E+08	Intergenic	Intergenic	-18211	NM_20766	56318	Acpp	5'-NT|A03(
chr14	35123002	35123800	promoter-T	promoter-T	288	NM_02938	75698	Fam35a	3110001K2
chr11	79119377	79119575	Intergenic	Intergenic	-33918	NM_01089	18015	Nf1	AW494271
chr11	16215327	16215550	Intergenic	Intergenic	57711	NM_14596	211739	Vstm2a	Vstm2
chr19	3323802	3323950	intron (NM	CpG	575	NM_01349	12894	Cpt1a	C730027G(
chr13	21494602	21494925	promoter-T	promoter-T	-139	NM_02368	72739	Zkscan3	2810435N(
chr1	1.93E+08	1.93E+08	intron (NM	intron (NM	5096	NM_02542	66208	Nenf	1110060M
chr1	1.22E+08	1.22E+08	intron (NM	intron (NM	28869	NM_01943	170706	Tmem37	AI173373|/
chr4	1.45E+08	1.45E+08	Intergenic	Intergenic	57011	NM_01130	20148	Dhrs3	Rsdr1|retS
chr11	1.2E+08	1.2E+08	promoter-T	promoter-T	-129	NM_13379	192662	Arhgdia	5330430M
chr3	94846777	94846925	promoter-T	promoter-T	-315	NM_00895	19185	Psmd4	Af1|Mcb1|
chr4	9601777	9601975	Intergenic	Intergenic	-5385	NM_00129	65973	Asph	2310005F1
chrX	39421552	39421650	5' UTR (NM	5' UTR (NM	588	NM_00968	11798	Xiap	1110015C(
chr14	45945202	45945500	Intergenic	MLT1F1|LT	-4148	NM_02595	67089	Psmc6	2300001E(
chr12	99346927	99347000	Intergenic	Intergenic	150584	NM_00807	14420	Galc	2310068B(
chr14	52739002	52739250	intron (NM	intron (NM	8548	NM_02387	77945	Rpgrip1	0610005A(
chr5	1.16E+08	1.16E+08	exon (NM_	exon (NM_	140	NM_02650	52064	Coq5	1810014G(
chr1	1.57E+08	1.57E+08	intron (NM	MLT1K|LTF	6593	NM_02143	58244	Stx6	2310039E(
chr9	1.06E+08	1.06E+08	Intergenic	Intergenic	-13228	NM_02055	11655	Alas1	ALAS|ALAS
chr2	76326902	76326975	intron (NM	RMER2|LTI	-10125	NM_00129	99031	Osbpl6	1110062M
chr3	65772777	65772925	Intergenic	Intergenic	-10704	NM_01993	56706	Ccnl1	2610030E2
chr19	47929477	47929850	intron (NM	CpG	184	NM_01036	14873	Gsto1	AA407097|
chrX	1.02E+08	1.02E+08	promoter-T	promoter-T	-116	NM_00959	11306	Abcb7	AA517758|
chr9	19426552	19426650	promoter-T	promoter-T	66	NM_17291	244713	Zfp317	4932416G(
chr4	1.38E+08	1.38E+08	exon (NM_	exon (NM_	248	NM_02688	68943	Pink1	1190006F0
chr4	1.47E+08	1.47E+08	promoter-T	promoter-T	80	NM_00128	170731	Mfn2	D630023P1
chr2	27448827	27449350	Intergenic	Intergenic	78421	NM_08084	140858	Wdr5	2410008O(
chr1	1.54E+08	1.54E+08	Intergenic	Intergenic	-9126	NM_19799	69399	1700025G(	2610510E(
chr2	24167177	24167400	Intergenic	Intergenic	20572	NM_15307	215274	Il1f10	-
chr10	89245327	89245400	exon (NM_	exon (NM_	37627	NM_02916	75089	Uhrf1bp1l	2010319N2
chr7	1.26E+08	1.26E+08	promoter-T	promoter-T	-26	NM_02319	66356	Knop1	2310008H(
chr12	1.12E+08	1.12E+08	intron (NM	CpG	242	NM_01048	15519	Hsp90aa1	86kDa|89k
chr6	92164552	92164950	promoter-T	promoter-T	54	NM_03008	78287	Zfyve20	5330426D1
chr1	1.84E+08	1.84E+08	intron (NM	intron (NM	-74881	NM_13381	98386	Lbr	AI505894|i
chr6	86388477	86388600	promoter-T	promoter-T	-102	NM_00124	232196	C87436	-
chr1	1.83E+08	1.83E+08	Intergenic	Intergenic	41833	NM_00821	15078	H3f3a	H3.3A
chr1	1.72E+08	1.72E+08	intron (NM	intron (NM	21978	NM_02752	70729	Nos1ap	6330408P1
chr3	1.08E+08	1.08E+08	5' UTR (NM	5' UTR (NM	381	NM_00128	229715	Amigo1	Amigo|ali2
chr8	47399052	47399475	Intergenic	Intergenic	-19238	NM_17378	234219	Helt	A830086M
chr6	1.03E+08	1.03E+08	Intergenic	Intergenic	-265144	NM_00769	12661	Chl1	A530023M
chr4	1.26E+08	1.26E+08	promoter-T	promoter-T	79	NM_02554	66407	Mrps15	1500003E2
chr1	1.81E+08	1.81E+08	intron (NM	intron (NM	471533	NM_02718	69726	Smyd3	2410008A1
chr13	74397302	74397425	intron (NM	intron (NM	32394	NM_00964	11624	Ahrr	mKIAA1234
chr16	70310977	70311175	Intergenic	RMER15-in	-3118	NM_02880	74185	Gbe1	2310045H1
chr1	1.83E+08	1.83E+08	intron (NM	(TC)n|Simp	1896	NM_14551	226757	Wdr26	1600024A(

chr15	1E+08	1E+08	promoter-T	promoter-T	323	NM_00114	18174	Slc11a2	DCT1|DMT
chr15	36385202	36385675	Intergenic	Intergenic	41108	NM_17513	68839	Ankrd46	1110054N0
chr6	91634952	91635025	intron (NM	intron (NM	927	NM_00932	21366	Slc6a6	AA589629|
chr12	1.02E+08	1.02E+08	non-coding	non-coding	174	NM_21135	68734	Smek1	1110034C0
chr4	1.32E+08	1.32E+08	intron (NM	intron (NM	533	NM_00129	100336	Ppp1r8	6330548N2
chr10	1.14E+08	1.14E+08	Intergenic	Intergenic	689488	NM_14624	237553	Trhde	9330155P2
chr5	21301677	21301900	intron (NM	intron (NM	10687	NM_01118	19181	Psmc2	-
chr1	54899302	54899425	intron (NM	intron (NM	83868	NM_00108	329154	Ankrd44	4930444A1
chrX	33623252	33623375	Intergenic	Intergenic	-28821	NM_13399	16164	Il13ra1	AI882074|
chr8	1.17E+08	1.17E+08	intron (NM	intron (NM	155299	NM_01957	80707	Wwox	5330426P0
chr11	1.16E+08	1.16E+08	Intergenic	Intergenic	-10120	NM_17690	319370	Ubald2	1110014K0
chr3	1.08E+08	1.08E+08	Intergenic	Intergenic	-2747	NM_01035	14862	Gstm1	Gstb-1|Gst
chr5	1.23E+08	1.23E+08	promoter-T	promoter-T	-607	NM_17542	109305	Orai1	D730049H0
chr17	34258527	34258625	5' UTR (NM	5' UTR (NM	116	NM_01023	14312	Brd2	AW228947
chr15	76368752	76369025	promoter-T	promoter-T	288	NM_01390	30840	Fbxl6	AU021795
chr5	24347727	24347875	intron (NM	CpG	378	NM_05307	19744	Rheb	-
chr7	74517977	74518225	promoter-T	promoter-T	-357	NM_00129	17258	Mef2a	A430079H0
chr9	1.06E+08	1.06E+08	5' UTR (NM	5' UTR (NM	150	NM_15345	235584	Dusp7	AU015694
chr8	1.08E+08	1.08E+08	promoter-T	promoter-T	212	NM_14582	74334	Ranbp10	4432417N0
chr19	21345827	21345925	promoter-T	promoter-T	-892	NM_00955	22682	Zfand5	2310057A0
chr16	25425402	25425750	Intergenic	Intergenic	138673	NM_17516	71338	Tprg	5430420C1
chr18	66637802	66638000	Intergenic	Intergenic	19643	NM_02145	58801	Pmaip1	Noxa
chr8	91162902	91163025	Intergenic	Intergenic	-2936	NM_02784	71607	Snx20	9130017C1
chr15	89724152	89724275	Intergenic	Intergenic	-51922	NM_01880	54526	Syt10	-
chr5	1.24E+08	1.24E+08	intron (NM	intron (NM	6800	NM_17509	23912	Rhof	AI845056|
chr5	1.23E+08	1.23E+08	promoter-T	promoter-T	-227	NM_01980	56317	Anapc7	APC7|AW5
chr16	57122552	57122750	intron (NM	intron (NM	824	NM_13859	28185	Tomm70a	2610044B2
chr19	4878102	4878450	promoter-T	promoter-T	-367	NM_01345	11474	Actn3	-
chr14	22650877	22651125	intron (NM	CpG	218	NM_01169	22334	Vdac2	Vdac6|mV
chr3	68422352	68422500	intron (NM	intron (NM	46136	NM_01392	30953	Schip1	Nf2ip|Schi
chr1	88484027	88484175	intron (NM	intron (NM	426	NM_17297	26895	Cops7b	D1Wsu66e
chr19	43991602	43991850	intron (NM	intron (NM	22962	NM_02802	71972	Dnmbp	2410003L0
chr7	1.34E+08	1.34E+08	exon (NM_	exon (NM_	1055	NM_01077	17188	Maz	PUR1|Pur-
chr8	35172377	35172625	promoter-T	promoter-T	-735	NM_01172	22428	Dctn6	AU044699
chr12	1.1E+08	1.1E+08	intron (NM	MIR|SINE|	12225	NM_00116	14026	Evl	AI528774
chr8	59967002	59967125	promoter-T	promoter-T	-406	NM_02178	60406	Sap30	30kDa
chr7	31936127	31936750	promoter-T	promoter-T	-369	NM_02789	52857	Gramd1a	1300003M
chr15	78729502	78729600	promoter-T	promoter-T	-665	NM_00916	20401	Sh3bp1	3BP-1
chr1	1.35E+08	1.35E+08	Intergenic	Intergenic	-4517	NM_13381	108954	Ppp1r15b	1810033K1
chr11	59761777	59762075	promoter-T	promoter-T	210	NM_13867	192191	Med9	BC019367|
chr7	88092852	88093025	promoter-T	promoter-T	-502	NM_01995	56529	Sec11a	1810012E0
chr2	1.26E+08	1.26E+08	intron (NM	intron (NM	16063	NM_00823	15186	Hdc	AW108189
chr18	23892377	23892600	Intergenic	Intergenic	-18346	NM_00116	212307	Mapre2	AI314113|
chr6	82954902	82954975	intron (NM	intron (NM	28527	NM_01007	13448	Dok1	AW557123
chr7	4740502	4740725	3' UTR (NM	3' UTR (NM	549	NM_02938	664968	Tmem238	2210411K1
chr1	66947552	66947900	Intergenic	L1MD2|LIN	34214	NM_00111	17901	Myl1	AI325107|
chr11	1.2E+08	1.2E+08	intron (NM	intron (NM	797	NM_02798	71885	2310003H0	Faap100

chr11	54787177	54787250	Intergenic	Intergenic	-10771	NM_00127	57783	Tnip1	ABIN|ABIN
chr4	65957552	65957800	intron (NM	intron (NM	107841	NM_01951	56079	Astn2	1d8|Astnl|
chr13	42787727	42787825	intron (NM	intron (NM	11784	NM_19841	218194	Phactr1	9630030F1
chr2	68634077	68634675	Intergenic	Intergenic	-65238	NM_17285	241447	Cers6	4732462C0
chr7	1.23E+08	1.23E+08	intron (NM	MER119|D	27737	NM_01144	20679	Sox6	AI987981|
chr7	1.17E+08	1.17E+08	intron (NM	B1_Mur1|	49021	NM_00930	20947	Swap70	70kDa|AV2
chr3	1.44E+08	1.44E+08	promoter-T	promoter-T	504	NM_01182	23908	Hs2st1	AW214369
chr17	24607527	24607950	exon (NM_	exon (NM_	320	NM_02595	67078	Pgp	1700012G1
chr6	1.43E+08	1.43E+08	promoter-T	promoter-T	-427	NM_00114	330440	Gm766	-
chr5	35003002	35003200	promoter-T	promoter-T	73	NM_01949	14772	Grk4	A830025H0
chr5	4344452	4344750	Intergenic	L1MA9|LIN	152234	NM_00104	208595	Mterf1b	ENSMUSG0
chr19	41337527	41337700	intron (NM	intron (NM	881	NM_13335	107358	Tm9sf3	1810073M
chr18	32512427	32512525	Intergenic	Lx9|LINE|L	-24395	NM_00108	30948	Bin1	ALP-1|Amp
chr1	60955752	60955950	Intergenic	Intergenic	-10018	NM_00984	12477	Ctla4	Cd152|Ctla
chr8	11575527	11575725	Intergenic	Intergenic	19560	NM_01191	26356	Ing1	2610028J2
chr16	23107002	23108200	promoter-T	promoter-T	60	NR_11033	13682	Eif4a2	4833432N0
chr10	79371477	79371600	intron (NM	CpG	145	NM_00116	216154	Med16	95kDa|A63
chr3	1.03E+08	1.03E+08	exon (NM_	exon (NM_	697	NM_00107	94093	Trim33	8030451N0
chr1	16508927	16509075	intron (NM	C-rich|Low	382	NM_02530	29819	Stau2	-
chr11	51912377	51912500	5' UTR (NM	5' UTR (NM	112	NM_01941	19052	Ppp2ca	PP2A|R753
chr9	59446427	59446800	intron (NM	(TTC)n|Sim	-18478	NR_03807	67287	Parp6	1700119G1
chr6	49167677	49167850	Intergenic	CpG	-2810	NM_02367	140488	Igf2bp3	2610101N1
chr13	91062952	91063150	intron (NM	CpG	324	NM_02417	66475	Rps23	2410044J1
chr6	1.29E+08	1.29E+08	Intergenic	Intergenic	-7879	NM_00115	17059	Klrb1c	AI462337|
chr3	1.23E+08	1.23E+08	promoter-T	promoter-T	-286	NM_13385	99526	Usp53	AA939927|
chr9	1.2E+08	1.2E+08	Intergenic	Intergenic	-8862	NM_15328	215418	Csrnp1	4931429D1
chr13	41701302	41701375	promoter-T	promoter-T	-247	NM_00116	621976	Tmem170b	EG621976
chr10	60837977	60838075	intron (NM	intron (NM	8245	NM_01375	27355	Pald1	MMPAL|Pa
chr7	71144477	71144725	Intergenic	Intergenic	-60800	NM_02136	50794	Klf13	0610043C1
chr8	73819827	73820150	intron (NM	ID_B1|SINE	23375	NM_00114	17925	Myo9b	-
chr13	96548802	96548875	intron (NM	intron (NM	81963	NM_01017	14064	F2rl2	F730031A0
chr9	51771552	51771675	promoter-T	promoter-T	25	NM_00799	14148	Fdx1	-
chr12	85758152	85758425	exon (NM_	exon (NM_	138	NM_02552	66381	Rnf113a2	2310020H1
chr14	28168377	28168450	intron (NM	L1MC4a|LI	-6162	NM_00128	71704	Arhgef3	1200004I2
chr5	1.25E+08	1.25E+08	intron (NM	intron (NM	938	NM_01977	56334	Tmed2	1110032D1
chr4	45025902	45025975	promoter-T	promoter-T	-654	NM_17339	230119	Zbtb5	5930421I1
chr8	1.09E+08	1.09E+08	intron (NM	intron (NM	78895	NM_17303	272538	Tango6	AW413431
chr8	1.22E+08	1.22E+08	intron (NM	CpG	386	NM_00946	22224	Usp10	2610014N0
chr9	1.14E+08	1.14E+08	Intergenic	MTD|LTR|	-4359	NM_00110	382111	Susd5	Gm1126
chr9	66530702	66530775	Intergenic	ORR1D2|L	-30755	NM_17839	76459	Car12	2310047E0
chr2	78708627	78708750	promoter-T	promoter-T	-516	NM_00945	22193	Ube2e3	Ubce4|ubc
chr2	1.32E+08	1.32E+08	intron (NM	intron (NM	10786	NM_17544	215653	Rassf2	3830431H0
chr15	59541652	59541725	Intergenic	Intergenic	61479	NM_14454	211770	Trib1	A530090O
chr4	1.33E+08	1.33E+08	Intergenic	RMER19B|	24968	NM_01695	15331	Hmgn2	HMG-17|H
chr1	1.68E+08	1.68E+08	Intergenic	CpG	-7866	NM_00108	68481	Mpzl1	1110007A1
chr13	1.02E+08	1.02E+08	Intergenic	L1MB2|LIN	82609	NM_00102	18708	Pik3r1	PI3K|p50al
chr2	28915877	28916075	intron (NM	CpG	193	NM_00944	22130	Ttf1	AV245725|

chr16	17276527	17276825	intron (NM	CpG	283	NM_17747	224019	Tmem191c	4933405M
chr11	9026477	9026650	intron (NM	intron (NM	8053	NM_00115	22271	Upp1	AI325217|
chr5	1.14E+08	1.14E+08	intron (NM	CpG	203	NM_19810	231637	Ssh1	AW551225
chr3	1.33E+08	1.33E+08	intron (NM	CpG	452	NM_14614	74776	Ppa2	1110013G1
chr7	1.35E+08	1.35E+08	exon (NM_	exon (NM_	309	NM_19801	101604	E430018J2	AI480612
chr17	32500977	32501450	intron (NR_	(CGG)n|Sir	-13691	NM_01747	54194	Akap8l	HAP95|Nal
chr12	35703802	35703875	Intergenic	MIRb|SINE	-28023	NM_00101	217463	Snx13	Rgs-px1|m
chr7	38971927	38972025	intron (NM	intron (NM	3170	NM_02816	72244	1600014C1	AI428873
chr13	8870252	8870325	promoter-T	promoter-T	2	NM_00103	207615	Wdr37	-
chr7	1.07E+08	1.07E+08	Intergenic	MTC|LTR|I	-1970	NM_17538	108937	Rnf169	2900057K0
chr10	81090177	81090300	promoter-T	promoter-T	115	NM_18158	50721	Sirt6	2810449N1
chr9	69837477	69837700	intron (NM	CpG	315	NM_00100	12175	Bnip2	5730523P1
chr9	57488077	57488225	intron (NM	intron (NM	4836	NM_00778	12988	Csk	AW212630
chr13	37212152	37212350	Intergenic	Intergenic	-70138	NM_00116	74145	F13a1	1200014I0
chr12	1.02E+08	1.02E+08	intron (NM	intron (NM	-16705	NM_00117	238377	Gpr68	BB131428|
chr4	53799102	53799325	3' UTR (NM	3' UTR (NM	6636	NM_00931	21350	Tal2	bHLHa19
chr2	1.19E+08	1.19E+08	promoter-T	promoter-T	-67	NM_02717	69702	Ndufaf1	2410001M
chr16	11176027	11176250	intron (NR_	CpG	348	NR_02750	106205	Zc3h7a	A430104C1
chr11	99002927	99003275	Intergenic	MIR|SINE|	13290	NM_00771	12775	Ccr7	CD197|Cdv
chr18	38456377	38456525	promoter-T	promoter-T	-8	NM_00116	56736	Rnf14	2310075C0
chr11	93979827	93980175	intron (NM	ORR1B2|LT	74482	NM_00102	70834	Spag9	3110018C0
chr8	67356402	67356525	intron (NM	B4A|SINE|	17359	NM_17863	77113	Klhl2	6030411N2
chr2	25050077	25050300	exon (NM_	exon (NM_	2145	NM_14611	227612	Tor4a	A830007P1
chr17	12367177	12367525	intron (NM	intron (NM	55201	NM_02664	68262	Agpat4	1500003P2
chr7	52650077	52650175	exon (NM_	exon (NM_	891	NM_00922	20637	Snrnp70	2700022N2
chr9	20877577	20877700	TTS (NM_0	TTS (NM_0	320	NM_00103	68165	Fdx1l	B230118G1
chr7	1.36E+08	1.36E+08	intron (NM	CpG	716	NM_01386	29810	Bag3	AA407278|
chr8	72425977	72426250	promoter-T	promoter-T	-1	NM_03200	83984	Tssk6	Sstk
chr11	87666752	87666925	intron (NM	CpG	111	NM_00103	380718	Mks1	AK190930|
chr4	98244027	98244225	intron (NM	intron (NM	30801	NM_00100	12695	Inadl	Cipp|Patj
chr11	58012752	58013025	promoter-T	promoter-T	-170	NM_01873	16145	Igtp	AW558444
chr11	58681177	58681275	intron (NM	RMER15|LT	484	NM_17240	69944	2810021J2	AI449137
chr11	50138877	50139075	intron (NM	CpG	199	NM_00111	12330	Canx	1110069N1
chr3	85623402	85623500	Intergenic	Intergenic	67989	NM_17713	320302	Glt28d2	4732486J0
chr2	89511327	89511575	Intergenic	Intergenic	-3410	NM_00101	259145	Olfr1251	GA_x5J8B7
chr10	77707327	77707900	promoter-T	promoter-T	-226	NM_00108	216131	Trappc10	B230307C2
chr2	1.56E+08	1.56E+08	intron (NR_	intron (NR_	-44161	NM_02778	71405	Fam83c	5530400B0
chr1	1.77E+08	1.77E+08	intron (NM	intron (NM	-212501	NM_01182	23893	Grem2	Gremlin2|I
chr13	60609577	60609750	Intergenic	Intergenic	-93645	NM_00128	69635	Dapk1	D13Ucla1|
chr6	88704627	88705000	intron (NM	intron (NM	30115	NM_00116	23945	Mgll	AA589436|
chr7	89561752	89561925	intron (NM	intron (NM	77634	NM_00119	269959	Adamtsl3	9230119C1
chr3	35646027	35646250	Intergenic	Intergenic	-6922	NM_02957	76295	Atp11b	1110019I1
chr5	1.09E+08	1.09E+08	promoter-T	promoter-T	9	NM_00750	11958	Atp5k	2610008D2
chr5	1.03E+08	1.03E+08	intron (NM	intron (NM	56503	NM_02927	231532	Arhgap24	0610025G2
chr7	1.35E+08	1.35E+08	promoter-T	promoter-T	60	NM_17802	233904	Setd1a	BC010250|
chr3	51162952	51163125	Intergenic	CpG	-18472	NM_02350	69257	Elf2	2610036A2
chr10	11000427	11000650	promoter-T	promoter-T	-590	NM_02796	71865	Fbxo30	1700026A1

chr1	1.33E+08	1.33E+08	intron (NM	intron (NM	3364	NM_01977	56489	Ikbke	AW558201
chr15	96540902	96540975	Intergenic	Intergenic	-10809	NM_17512	67760	Slc38a2	5033402L1
chr12	1.06E+08	1.06E+08	Intergenic	Intergenic	-3282	NM_17250	212073	Syne3	nesprin-3
chr9	42072377	42072500	promoter-T	promoter-T	-55	NM_17276	235293	Sc5d	A830037K0
chr8	86671777	86672200	promoter-T	promoter-T	51	NM_02904	74666	4930432K2	-
chr7	1.03E+08	1.03E+08	Intergenic	Intergenic	-39621	NM_01185	23966	Tenm4	Doc4|ELM2
chr1	1.41E+08	1.41E+08	Intergenic	Intergenic	-8847	NM_17264	226470	Zbtb41	8430415N2
chr15	66645602	66645800	intron (NM	intron (NM	17690	NM_00102	20491	Sla	Slap|Slap-1
chr5	44173902	44174050	promoter-T	promoter-T	-588	NM_17872	242960	Fbxl5	Fbl4|Fir4
chr1	51535627	51535750	promoter-T	promoter-T	-445	NM_02869	109019	Nabp1	4930434H0
chr11	86348027	86348350	intron (NM	intron (NM	10121	NM_02825	72508	Rps6kb1	2610318I15
chr1	1.52E+08	1.52E+08	intron (NM	intron (NM	14551	NM_00886	18783	Pla2g4a	Pla2g4|cPL
chr11	67613727	67614000	intron (NM	URR1B|DN	2090	NM_00101	68460	Dhrs7c	1110001P1
chr19	32978102	32978175	Intergenic	Intergenic	146071	NM_00896	19211	Pten	2310035O0
chr15	6658177	6658300	promoter-T	promoter-T	-143	NM_03016	78757	Rictor	4921505C1
chr15	11925452	11925675	intron (NM	CpG	199	NM_01129	20024	Sub1	AI842364|
chr7	17611552	17611700	intron (NM	L2b|LINE|L	1637	NM_01115	19060	Ppp5c	AU020526
chr5	1.22E+08	1.22E+08	promoter-T	promoter-T	13	NM_01076	17165	Mapkapk5	MK5|PRAK
chr2	24442627	24442725	Intergenic	LTRIS5|LTR	-111557	NM_01104	18510	Pax8	Pax-8
chr12	80640102	80640350	intron (NM	(TC)n|Simp	241888	NM_00125	19363	Rad51b	AI553500|
chr14	65276552	65276775	intron (NM	intron (NM	5295	NM_00108	16554	Kif13b	5330429L1
chr12	73186002	73186125	promoter-T	promoter-T	237	NM_02603	67217	L3hypdh	2810055F1
chr5	1.38E+08	1.38E+08	intron (NM	intron (NM	873	NM_00125	68929	Mospd3	1190005J1
chr18	14841577	14841875	promoter-T	promoter-T	-303	NM_00116	268996	Ss18	D130059H1
chr13	94200627	94200700	3' UTR (NM	3' UTR (NM	69100	NM_02131	57748	Jmy	-
chr6	72908102	72908275	promoter-T	promoter-T	38	NM_00103	19240	Tmsb10	Ptmb10|Tb
chr2	71112277	71112550	TTS (NM_1	TTS (NM_1	62410	NM_01006	13427	Dync1i2	3110079H0
chr4	41081877	41081975	Intergenic	Intergenic	-1128	NM_02627	67615	Ube2r2	1200003M
chr9	40622177	40622350	Intergenic	Intergenic	12907	NM_03116	15481	Hspa8	2410008N1
chr17	35263352	35263475	promoter-T	promoter-T	66	NM_03347	114585	D17H6S53E	G4|NG34
chr2	1.7E+08	1.7E+08	Intergenic	Intergenic	-21481	NM_02981	76960	Bcas1	2210416M
chr8	13785277	13785425	promoter-T	promoter-T	-264	NM_02592	67031	Upf3a	2600001C0
chr1	65232702	65232975	promoter-T	promoter-T	215	NM_00111	15926	Idh1	AI314845|
chr10	43621202	43621625	promoter-T	promoter-T	129	NM_00108	76563	Qrsl1	2700038P1
chr8	3288602	3288950	Intergenic	Lx9|LINE|L	-9159	NM_01056	16337	Insr	4932439J0
chrX	13806277	13806450	Intergenic	MLT1G1|LT	18089	NM_00103	385328	Gm5382	EG385328
chr12	70397452	70397850	promoter-T	promoter-T	-17	NM_02711	69554	Klhdc2	2310022K1
chr15	74762077	74762150	Intergenic	Intergenic	-23368	NM_00116	17069	Ly6e	9804|Ly67
chr4	1.52E+08	1.52E+08	intron (NM	intron (NM	2663	NM_00125	16498	Kcnab2	F5|I2rf5|K
chr19	8954777	8954975	3' UTR (NM	3' UTR (NM	547	NM_00116	107197	Uqcc3	AI462493|
chr3	1.02E+08	1.02E+08	Intergenic	B3|SINE|B	-20850	NM_00103	242126	Slc22a15	2610034P2
chr19	3575527	3575650	intron (NM	CpG	161	NM_00116	52036	Ppp6r3	4930528G0
chr14	75585652	75585800	intron (NM	intron (NM	49494	NM_00887	18826	Lcp1	AW536232
chr10	31328977	31329050	5' UTR (NM	5' UTR (NM	518	NM_00114	268291	Rnf217	AU016819
chr2	34580927	34581075	exon (NM_	exon (NM_	29751	NM_02570	66691	Gapvd1	2010005B0
chr8	3232627	3232875	intron (NM	intron (NM	46866	NM_01056	16337	Insr	4932439J0
chr1	43552952	43553050	intron (NM	MER20|DN	50405	NM_01087	17974	Nck2	4833426I1

chr11	98779902	98780125	intron (NM	intron (NM	10548	NM_01179	23834	Cdc6	CDC18L
chr9	1.08E+08	1.08E+08	exon (NM_	exon (NM_	-10377	NM_14622	235606	Apeh	-
chr2	35293327	35293700	intron (NM	MIRb|SINE	23432	NM_01028	14594	Ggta1	AW108479
chr3	95976727	95976825	intron (NM	intron (NM	303	NM_15305	107701	Sf3b4	49kDa|SF3
chr6	1.33E+08	1.33E+08	exon (NM_	exon (NM_	269	NR_07336	381820	2700089E2	AI662692
chr1	1.07E+08	1.07E+08	intron (NM	intron (NM	71711	NM_17877	320311	Rnf152	A930029B0
chr19	18653127	18653275	Intergenic	Intergenic	53102	NM_01737	20409	Ostf1	C78236|SH
chr11	72224727	72224800	exon (NM_	exon (NM_	452	NM_17777	276829	Smtnl2	D130058I2
chr10	62064877	62065175	promoter-T	promoter-T	20	NM_01955	56200	Ddx21	AI255159|
chr2	1.58E+08	1.58E+08	intron (NM	intron (NM	784	NM_00848	16803	Lbp	Bpifd2|Ly8
chr8	89312352	89312450	Intergenic	Intergenic	-42496	NM_00917	20437	Siah1a	AA982064|
chr19	42222427	42222500	exon (NM_	exon (NM_	584	NM_18319	277010	Marveld1	AI504298|
chr9	14849977	14850100	promoter-T	promoter-T	-116	NM_01948	55991	Panx1	AI847747
chr6	8666527	8666775	intron (NM	intron (NM	41807	NM_00125	15893	Ica1	69kDa|ICA
chr3	1.52E+08	1.52E+08	promoter-T	promoter-T	-210	NM_00128	108946	Zzz3	3110065C2
chr5	1.08E+08	1.08E+08	promoter-T	promoter-T	-255	NM_02606	67266	Fam69a	2900024C2
chr13	54794452	54794700	intron (NR_	intron (NR_	745	NR_02739	105239	Rnf44	AI854545|
chr10	83904177	83904250	promoter-T	promoter-T	-997	NM_00100	77976	Nuak1	AU014801
chr12	83495027	83495200	intron (NM	intron (NM	82777	NM_17257	217692	Sipa1l1	4931426N1
chr17	31795452	31795675	intron (NM	CpG	136	NM_02418	108121	U2af1	2010107D1
chr14	8826952	8827075	promoter-T	promoter-T	-317	NM_00787	13421	Dnase1l3	DNasegam
chr6	1.25E+08	1.25E+08	Intergenic	Intergenic	49147	NM_00765	12527	Cd9	Tspan29
chr11	88725477	88725950	5' UTR (NM	5' UTR (NM	187	NM_00104	11640	Akap1	AKAP121|
chr19	21738402	21738925	intron (NM	intron (NM	10864	NM_14609	226016	Abhd17b	5730446C1
chr13	41096327	41096450	promoter-T	promoter-T	9	NM_02655	68083	Pak1ip1	5830431I1
chr17	47874327	47874425	promoter-T	promoter-T	390	NM_00116	21425	Tfeb	Tcfeb|bHL
chr2	1.12E+08	1.12E+08	promoter-T	promoter-T	-204	NM_02651	68032	Emc4	2610318K0
chr1	1.89E+08	1.89E+08	5' UTR (NM	5' UTR (NM	758	NM_00936	21808	Tgfb2	BB105277|
chr17	15838552	15838675	Intergenic	Intergenic	-3318	NM_00769	12648	Chd1	4930525N2
chr11	96778152	96778225	promoter-T	promoter-T	-393	NM_03024	80280	Cdk5rap3	1810007E2
chr3	40754527	40754725	promoter-T	promoter-T	73	NM_00104	214048	Larp1b	1700108L2
chr12	1.17E+08	1.17E+08	Intergenic	Intergenic	-135447	NM_00100	56220	Zfp386	Kzf1|mKIA
chr5	89149552	89149750	promoter-T	promoter-T	-245	NM_02673	68473	Mob1b	1110003E0
chr11	29147002	29147125	exon (NM_	exon (NM_	209	NM_02574	216618	Ccdc104	2300003H1
chr3	1.42E+08	1.42E+08	promoter-T	promoter-T	-486	NM_17376	229905	Ccbl2	KATIII|Kat3
chr12	76834702	76835125	intron (NM	intron (NM	1803	NM_03075	81535	Sgpp1	AI463453|
chr9	15110752	15110900	promoter-T	promoter-T	66	NM_00119	70984	4931406C0	-
chr4	11117602	11117750	promoter-T	promoter-T	-822	NM_00103	12448	Ccne2	-
chr12	77994102	77994200	intron (NM	intron (NM	55697	NM_14592	110606	Fntb	2010013E1
chr3	90463527	90463900	Intergenic	Intergenic	5489	NM_19942	381493	S100a7a	AY465109|
chr16	44724427	44724750	intron (NM	CpG	174	NM_14597	212547	BC027231	Nepro
chr5	32913802	32913975	5' UTR (NM	5' UTR (NM	344	NM_00953	22612	Yes1	Yes|p61-Ye
chr4	1.5E+08	1.5E+08	Intergenic	Intergenic	-3921	NM_02056	57320	Park7	DJ-1|Dj1
chr1	1.95E+08	1.95E+08	intron (NM	intron (NM	637	NM_14488	226861	Hhat	2810432O2
chr10	88194677	88194775	promoter-T	promoter-T	567	NM_02585	104303	Arl1	2310008D2
chr7	85927852	85927925	promoter-T	promoter-T	87	NM_02333	67308	Mrpl46	3110052F1
chr13	1.01E+08	1.01E+08	promoter-T	promoter-T	140	NM_00104	19356	Rad17	MmRad24

chr13	55979002	55979475	Intergenic	Intergenic	-46452	NM_01109	18740	Pitx1	Bft|P-OTX|
chr15	99872052	99872125	intron (NM	intron (NM	2993	NM_00115	239667	Dip2b	4932422C2
chr11	98544252	98544500	intron (NM	intron (NM	508	NM_00943	22123	Psmd3	AI255837|
chr4	59016127	59016350	exon (NM_	exon (NM_	173	NM_00103	72429	Dnajc25	2010109C0
chr7	1.09E+08	1.09E+08	5' UTR (NM	5' UTR (NM	925	NM_00112	16332	Inppl1	51C|SHIP2
chr1	33553077	33553150	intron (NM	intron (NM	173526	NM_00892	19076	Prim2	AI323589
chr15	79518977	79519350	promoter-T	promoter-T	-861	NM_02879	74158	Josd1	1300006C0
chr8	1.09E+08	1.09E+08	intron (NM	intron (NM	23901	NM_00922	20650	Sntb2	Snt2
chr6	99473602	99473725	Intergenic	Intergenic	-88324	NM_05320	108655	Foxp1	3110052D1
chr4	45544402	45544475	promoter-T	promoter-T	-738	NM_00103	230126	Shb	BC028832
chr17	66356752	66357100	intron (NM	intron (NM	-56399	NM_02305	65960	Twsg1	1810013J1
chr4	1.41E+08	1.41E+08	intron (NM	CpG	248	NR_10234	213491	Szrd1	1110022I0
chr4	1.55E+08	1.55E+08	intron (NM	intron (NM	59981	NM_01138	20481	Ski	2310012I0
chr14	1.06E+08	1.06E+08	Intergenic	Intergenic	-26115	NM_01189	24064	Spry2	sprouty2
chr5	65299552	65299675	Intergenic	Intergenic	24325	NM_03068	21897	Tlr1	-
chr4	1.33E+08	1.33E+08	Intergenic	Intergenic	-14333	NM_19930	230796	Wdtc1	Gm695|ad
chr15	83385952	83386025	exon (NM_	exon (NM_	153	NM_00103	223722	Mcat	AI225907|
chr18	68016852	68017025	intron (NM	intron (NM	57177	NM_02755	70799	Cep192	4631422C1
chr13	64375952	64376250	5' UTR (NM	5' UTR (NM	195	NM_17258	218294	Cdc14b	2810432N1
chr16	57083827	57083950	Intergenic	Lx8|LINE|L	-12429	NM_18328	69457	2310005G1	-
chr9	21421002	21421150	intron (NM	CpG	463	NM_00117	20586	Smarca4	Brg1|HP1-I
chr7	63274502	63274575	intron (NM	intron (NM	405	NM_15357	233280	Nipa1	1110027G0
chr1	1.73E+08	1.73E+08	Intergenic	Intergenic	-20569	NM_14455	246256	Fcgr4	4833442P2
chr12	17551152	17551350	promoter-T	promoter-T	-428	NM_01361	18263	Odc1	ODC
chr12	8320327	8320450	intron (NM	intron (NM	149	NM_02142	58240	Hs1bp3	-
chr13	1.05E+08	1.05E+08	intron (NM	intron (NM	2590	NM_00128	59079	Erbb2ip	1700028E0
chr3	1.42E+08	1.42E+08	Intergenic	Intergenic	-18460	NM_15356	229898	Gbp5	5330409J0
chr15	84145602	84146275	3' UTR (NM	3' UTR (NM	-9212	NM_02232	64099	Parvg	AI413459
chr6	1.46E+08	1.46E+08	intron (NM	MER58A|D	277171	NM_01065	16651	Sspn	Krag
chr9	39999627	39999825	promoter-T	promoter-T	-175	NM_03071	80902	Zfp202	C130037E2
chr10	20031377	20031450	promoter-T	promoter-T	-862	NM_00102	72567	Bclaf1	2610102K2
chr4	94055102	94055325	Intergenic	Intergenic	168274	NM_02636	67770	Caap1	5830433M
chr11	3166127	3166325	intron (NM	CpG	163	NM_00787	13494	Drg1	AA408859|
chr17	29301677	29301800	intron (NM	intron (NM	29737	NM_00100	442827	Rab44	9830134C1
chr1	94807152	94807325	promoter-T	promoter-T	-164	NM_18140	108657	Rnpepl1	1110014H1
chr7	1.5E+08	1.5E+08	Intergenic	Intergenic	-16194	NM_00128	55925	Syt8	-
chr5	1.45E+08	1.45E+08	intron (NM	intron (NM	25621	NM_00108	231876	Lmtk2	2900041G1
chr16	10835077	10835350	promoter-T	promoter-T	61	NM_00116	223970	Rmi2	A630055G0
chr13	75936252	75936375	Intergenic	Intergenic	-41021	NM_05310	93692	Glrx	C86710|D1
chr6	5510652	5510875	Intergenic	Intergenic	-64485	NM_01374	27273	Pdk4	AV005916
chr11	72028952	72029150	promoter-T	promoter-T	43	NM_02606	67279	Med31	3110004H1
chr8	26713102	26713300	5' UTR (NM	5' UTR (NM	423	NM_00100	234135	Whsc1l1	6720429E0
chr10	39978202	39978400	promoter-T	promoter-T	-830	NM_02611	67371	Gtf3c6	2410016F1
chr16	23106302	23106525	Intergenic	Intergenic	-1128	NM_00112	13682	Eif4a2	4833432N0
chr10	80500152	80500275	Intergenic	Intergenic	-3556	NM_13413	106947	Slc39a3	AI845814|
chr2	1.46E+08	1.46E+08	Intergenic	Intergenic	-5048	NM_00103	241694	Ralgapa2	A230067G2
chr2	3554252	3554525	Intergenic	Intergenic	-76342	NM_02562	66540	Fam107b	3110001A1

chr18	38532377	38532450	Intergenic	ORR1E|LTF	-33766	NM_01193	26384	Gnpda1	GNPDA|Gr
chr3	1.32E+08	1.32E+08	intron (NM	intron (NM	31045	NM_02026	56811	Dkk2	-
chr6	98676902	98677125	Intergenic	URR1A|DN	301279	NM_00119	108655	Foxp1	3110052D1
chr5	1.48E+08	1.48E+08	5' UTR (NM	5' UTR (NM	247	NM_01964	19933	Rpl21	8430440E0
chr4	1.03E+08	1.03E+08	3' UTR (NM	3' UTR (NM	44443	NM_00103	71148	Mier1	4933425I2
chr15	96890952	96891075	Intergenic	Intergenic	-4626	NM_02705	69354	Slc38a4	1110012E1
chr4	95828452	95828650	Intergenic	Intergenic	-20708	NM_00110	242546	Cyp2j12	BB197342|
chr13	14705577	14705825	promoter-T	promoter-T	192	NM_00894	19166	Psma2	Lmpc3
chr8	1.25E+08	1.25E+08	intron (NM	intron (NM	27270	NM_15377	66965	Ctu2	2310061F2
chr7	56653077	56653225	intron (NM	intron (NM	151592	NM_17527	78286	Nav2	5330421F0
chr6	1.37E+08	1.37E+08	Intergenic	Intergenic	34382	NM_02389	78600	Pde6h	A930033D1
chr1	1.63E+08	1.63E+08	Intergenic	Intergenic	17446	NM_00745	11758	Prdx6	1-cysPrx|9
chr6	28536827	28537075	intron (NM	intron (NM	106603	NM_01977	56463	Snd1	AL033314|
chr2	5887102	5887400	intron (NM	intron (NM	14736	NM_00108	326622	Upf2	-
chr11	79068252	79068450	promoter-T	promoter-T	-154	NM_01965	78889	Wsb1	1110056B1
chr2	24284977	24285075	intron (NM	intron (NM	44109	NM_17761	215632	Psd4	BC046518|
chrX	93152177	93152350	promoter-T	promoter-T	50	NM_15282	76130	Las1l	1810030A0
chr9	88201902	88202300	Intergenic	Intergenic	-20346	NM_01185	23959	Nt5e	2210401F0
chr6	1.03E+08	1.03E+08	Intergenic	Intergenic	-188282	NM_00769	12661	Chl1	A530023M
chr2	1.65E+08	1.65E+08	intron (NM	intron (NM	1474	NM_17837	67538	Zswim3	4921517A0
chr8	1.29E+08	1.29E+08	Intergenic	Kanga2_a|	108570	NM_02421	67952	Tomm20	1810060K0
chr11	5345377	5345500	promoter-T	promoter-T	-588	NM_00108	407821	Znrf3	Gm1167
chr10	1.08E+08	1.08E+08	Intergenic	Intergenic	100093	NM_05405	114774	Pawr	2310001G0
chr11	1.16E+08	1.16E+08	intron (NM	CpG	455	NM_14603	217337	Srp68	2610024I0
chr11	1.01E+08	1.01E+08	intron (NM	RLTR11A2|	22874	NM_21366	20848	Stat3	1110034C0
chr1	79773502	79773875	intron (NM	intron (NM	1095	NM_00108	69163	Mrpl44	1810030E1
chr13	94924477	94924625	intron (NM	intron (NM	96800	NM_17258	218454	Lhfpl2	6030465B1
chr17	34066202	34066725	promoter-T	promoter-T	-376	NM_00905	19732	Rgl2	KE1.5|Rab
chr2	1.58E+08	1.58E+08	exon (NM_	exon (NM_	489	NM_17541	109275	Actr5	AA545173|
chr7	5031602	5031900	promoter-T	promoter-T	-86	NM_01014	13854	Epn1	AA269831|
chr6	1.27E+08	1.27E+08	intron (NM	intron (NM	447	NM_18140	101187	Parp11	5330431N2
chr4	43706927	43707050	TTS (NM_0	TTS (NM_0	6691	NM_01948	56014	Olfr70	MOR262-1
chr10	79256077	79256150	promoter-T	promoter-T	-204	NM_02312	18483	Palm	-
chr1	87791077	87791150	promoter-T	promoter-T	28	NM_02241	64294	Itm2c	3110038L0
chr1	72309802	72309975	intron (NM	RMER12|L	21000	NM_02352	111175	Pecr	2400003B1
chr19	4037602	4037950	intron (NM	CpG	136	NM_01354	14870	Gstp1	GstpiB
chr4	1.09E+08	1.09E+08	promoter-T	promoter-T	-329	NM_15339	230603	Ttc39a	4922503N0
chr17	59900477	59900550	Intergenic	ORR1D2|L	-747768	NM_02649	67993	Nudt12	0610016O1
chr18	40378702	40378875	intron (NM	CpG	265	NM_02331	67180	Yipf5	2610311I1
chr14	95655277	95655450	Intergenic	Intergenic	-625122	NM_02573	66732	4921530L2	-
chr19	32711677	32712125	intron (NM	intron (NM	17406	NM_01186	23972	Papss2	1810018P1
chr9	1.2E+08	1.2E+08	intron (NM	CpG	300	NM_02623	67561	Wdr48	8430408H1
chr6	40526377	40526625	3' UTR (NM	3' UTR (NM	5114	NM_14638	258381	Olfr460	MOR120-2
chr13	13616552	13616650	Intergenic	Intergenic	-66075	NM_01074	17101	Lyst	D13Sfk13|
chr6	1.23E+08	1.23E+08	exon (NM_	exon (NM_	5013	NM_01081	17474	Clec4d	Clecsf8|M
chr15	1.02E+08	1.02E+08	promoter-T	promoter-T	-292	NM_00103	223918	Spryd3	BC008150
chr19	30104352	30104875	promoter-T	promoter-T	-390	NM_14487	109113	Uhrf2	2310065A2

chr16	4560302	4560600	promoter-T	promoter-T	-731	NM_03118	83383	Tfap4	AI642933|A
chr12	4483652	4483875	5' UTR (NM	5' UTR (NM	297	NM_01088	17977	Ncoa1	KAT13A|SR
chr1	1.69E+08	1.69E+08	intron (NM	CpG	466	NM_01999	56752	Aldh9a1	AA139417|
chrX	34731277	34731350	promoter-T	promoter-T	-25	NM_15350	69942	Rnf113a1	2810428C2
chr6	86992427	86992575	promoter-T	promoter-T	-339	NM_01352	14583	Gfpt1	2810423A1
chr1	1.33E+08	1.33E+08	promoter-T	promoter-T	-52	NM_14550	226419	Dyrk3	BC006704
chr3	97836277	97836350	intron (NM	intron (NM	18852	NM_01092	18129	Notch2	AI853703|
chr2	1.67E+08	1.67E+08	intron (NM	intron (NM	-2558	NM_00108	68949	1500012F0	Zfas1
chr16	5203502	5203650	exon (NM_	exon (NM_	529	NM_01379	27426	Nagpa	AI596180|
chr14	21298302	21298400	intron (NM	intron (NM	1004	NM_00111	11750	Anxa7	AI265384|A
chr18	38129702	38129900	promoter-T	promoter-T	-416	NM_17568	319262	Fchsd1	A030002D0
chr5	88898377	88898475	TTS (NM_0	TTS (NM_0	13390	NM_00966	11698	Ambn	-
chr9	1.14E+08	1.14E+08	intron (NM	intron (NM	22786	NM_00104	636931	Trim71	2610206G2
chr2	1.36E+08	1.36E+08	intron (NM	intron (NM	79047	NM_01382	18798	Plcb4	A930039J0
chr9	24928527	24928750	intron (NM	MTA_Mm|	27642	NM_02058	80517	Herpud2	5031400M
chr2	51790302	51790775	promoter-T	promoter-T	-9	NM_00114	71684	Rbm43	0610033I0
chr14	15803477	15803775	intron (NM	intron (NM	150297	NM_00119	674895	Nek10	Gm282
chr2	71916077	71916550	intron (NM	intron (NM	23639	NM_00120	56508	Rapgef4	1300003D1
chr12	77468177	77468625	intron (NM	intron (NM	2050	NM_00117	109929	Zbtb25	2810462M
chr2	91812477	91812675	Intergenic	MER58B|D	-8856	NM_13830	104418	Dgkz	E130307B0
chr8	1.27E+08	1.27E+08	intron (NM	CpG	221	NM_02974	76332	Cog2	1190002B0
chr6	87864127	87864275	TTS (NM_0	TTS (NM_0	231	NM_17373	232210	Hmces	8430410A1
chr3	1.04E+08	1.04E+08	TTS (NM_0	TTS (NM_0	-1467	NM_17268	229675	Rsbn1	C230004D0
chr16	16896802	16896875	intron (NM	intron (NM	276	NM_17683	68606	Ppm1f	1110021B1
chr12	33063827	33063925	exon (NM_	exon (NM_	222	NM_00116	72123	Ccdc71l	2010109K1
chr11	95214752	95214975	intron (NM	intron (NM	16531	NM_17254	215512	Fam117a	5730593F1
chr5	1.39E+08	1.39E+08	intron (NM	intron (NM	226	NM_13390	74570	Zkscan1	5930429A0
chr11	4233152	4233275	Intergenic	Intergenic	66414	NM_00103	16878	Lif	-
chr11	1.06E+08	1.06E+08	Intergenic	Intergenic	-2832	NM_19829	21763	Tex2	4930568E0
chr19	5460452	5460650	promoter-T	promoter-T	-56	NM_00125	58249	Fibp	2010004G0
chr3	1.16E+08	1.16E+08	intron (NM	intron (NM	-18546	NM_03001	229780	Trmt13	4631408H1
chr2	1.54E+08	1.54E+08	intron (NM	CpG	372	NM_19930	228807	Zfp341	Znf341
chr2	1.51E+08	1.51E+08	promoter-T	promoter-T	56	NM_15378	110078	Pygb	-
chr3	1.3E+08	1.3E+08	intron (NM	MIRb|SINE	4108	NM_02790	71760	Etnppl	1300019H0
chrX	50622702	50622800	5' UTR (NM	5' UTR (NM	231	NM_03016	78755	Fam122b	4632404H2
chr1	64164652	64165050	intron (NM	intron (NM	3112	NM_03356	93691	Klf7	9830124P0
chr13	41923927	41924175	intron (NM	intron (NM	18806	NM_17541	109254	Adtrp	9530008L1
chr4	40836327	40836425	Intergenic	Intergenic	-30713	NM_01146	20731	Spink4	MPGC60
chr5	1.06E+08	1.06E+08	promoter-T	promoter-T	-200	NM_00112	231549	Lrrc8d	2810473G0
chr1	1.21E+08	1.21E+08	intron (NM	intron (NM	29871	NM_02232	64143	Ralb	5730472O1
chr17	13407202	13407600	Intergenic	Intergenic	-6653	NM_01374	27263	Smok2a	Smok2
chr5	66329227	66329325	Intergenic	Intergenic	-29087	NM_00108	231252	Chrna9	2410015I0
chr13	51739752	51739900	promoter-T	promoter-T	-775	NM_02541	66197	Cks2	1110038L1
chr5	91089702	91089975	Intergenic	Intergenic	-20325	NM_02847	73246	Rassf6	1600016B1
chr3	1.04E+08	1.04E+08	promoter-T	promoter-T	-342	NM_17268	229675	Rsbn1	C230004D0
chr6	39155302	39155775	intron (NM	CpG	1234	NM_00103	338523	Kdm7a	A630082K2
chr2	1.68E+08	1.68E+08	Intergenic	Intergenic	116527	NM_00129	18019	Nfatc2	AI607462|

chr18	15367627	15367825	Intergenic	Intergenic	-57771	NM_13411	106931	Kctd1	4933402K1
chr7	51175452	51175575	Intergenic	Intergenic	-13628	NM_17486	317652	Klk15	-
chr6	41029077	41029175	Intergenic	Intergenic	-43618	NM_02333	67373	2210010CC	AV072249
chr5	1.25E+08	1.25E+08	intron (NM	intron (NM	566	NM_02567	66627	Ogfod2	1300006G1
chr7	1.3E+08	1.3E+08	5' UTR (NM	5' UTR (NM	102	NM_14492	233833	Tnrc6a	2010321I0!
chr2	1.3E+08	1.3E+08	intron (NM	intron (NM	22837	NM_00116	19262	Ptpra	Ptpa|Ptpal
chr2	1.7E+08	1.7E+08	Intergenic	Intergenic	-42938	NM_00115	228913	Zfp217	4933431CC
chr13	44680652	44680825	Intergenic	MER20|DN	-145405	NM_02187	16468	Jarid2	Jmj|jumon
chr11	75300552	75300625	5' UTR (NM	5' UTR (NM	309	NM_13865	192159	Prpf8	AU019467
chr1	1.64E+08	1.64E+08	promoter-T	promoter-T	25	NM_14487	71449	Mettl13	5630401D2
chr2	72076777	72077050	intron (NM	intron (NM	-46781	NM_02305	65964	Zak	AV006891|
chr16	22615902	22616125	intron (NM	intron (NM	41291	NM_13865	110197	Dgkg	2900055E1
chr4	1.18E+08	1.18E+08	intron (NM	intron (NM	17189	NM_01121	19268	Ptprf	AA591035|
chr8	1.29E+08	1.29E+08	Intergenic	Intergenic	113758	NM_02421	67952	Tomm20	1810060KC
chr9	1.1E+08	1.1E+08	intron (NM	intron (NM	2351	NM_00116	29873	Cspg5	Caleb|Ngc
chrX	1.39E+08	1.39E+08	intron (NM	intron (NM	43053	NM_00100	245650	Gucy2f	A930002I0
chr13	19914852	19914975	intron (NM	intron (NM	1213	NM_18175	353346	Gpr141	PGR13
chr10	82161627	82161700	promoter-T	promoter-T	-89	NM_00108	67933	Hcfc2	1700129L1
chr2	1.29E+08	1.29E+08	Intergenic	MIR|SINE|	-22125	NM_17765	228592	F830045P1	Sirpb3
chr14	1.02E+08	1.02E+08	intron (NM	intron (NM	151518	NM_20152	380928	Lmo7	C78582|FB
chr12	4233677	4233825	promoter-T	promoter-T	-276	NM_00120	69709	Ptrhd1	2410017PC
chr9	77952827	77953050	intron (NM	intron (NM	3525	NM_02360	71538	Fbxo9	9030401P1
chr5	1.3E+08	1.3E+08	Intergenic	Intergenic	16466	NM_02691	69034	Nupr1l	1810010EC
chr7	1.35E+08	1.35E+08	5' UTR (NM	5' UTR (NM	536	NM_14625	244219	Zfp668	BC030314|
chr3	1.07E+08	1.07E+08	intron (NM	intron (NM	2104	NM_00765	12508	Cd53	AI323659|
chr12	83429202	83429325	intron (NM	intron (NM	16927	NM_17257	217692	Sipa1l1	4931426N1
chr7	52383952	52384200	promoter-T	promoter-T	39	NM_00943	22121	Rpl13a	1810026N2
chr8	1.23E+08	1.23E+08	promoter-T	promoter-T	599	NM_01092	18117	Emc8	Cox4nb|Fa
chr4	1.29E+08	1.29E+08	Intergenic	Intergenic	-14214	NM_17287	242669	Azin2	4933429I2
chr12	33730577	33730750	intron (NM	intron (NM	47077	NM_00102	68764	Cdhr3	1110049BC
chr4	1.08E+08	1.08E+08	promoter-T	promoter-T	-435	NM_17704	319965	Cc2d1b	A830039BC
chr15	55592352	55592625	intron (NM	MLT1N2|LT	146016	NM_01666	20649	Sntb1	-
chr10	79625627	79625825	Intergenic	Intergenic	-4860	NM_00770	12696	Cirbp	Cirp|R7494
chr1	1.73E+08	1.73E+08	intron (NM	intron (NM	9019	NM_00862	17528	Mpz	Mpp|P-zer
chr12	1.04E+08	1.04E+08	Intergenic	Intergenic	-8659	NM_17258	217837	Itpk1	BC031182
chr9	72784227	72784375	intron (NM	RMER21B|	10844	NM_02811	72135	Pygo1	2600014C2
chr19	25212752	25213025	intron (NM	intron (NM	-98804	NM_18140	107351	Kank1	A930031BC
chr1	1.36E+08	1.36E+08	intron (NM	intron (NM	1833	NM_15289	75605	Kdm5b	2010009J1
chr3	1.03E+08	1.03E+08	intron (NM	intron (NM	1007	NM_14490	229663	Csde1	AA960392|
chr1	80336477	80336825	intron (NM	GC_rich|Lc	354	NM_01671	26554	Cul3	AI467304|
chr1	15795727	15796050	exon (NM_	exon (NM_	161	NM_00128	21749	Terf1	Pin2|Trbf1
chr6	99471427	99471500	Intergenic	CpG	-86124	NM_05320	108655	Foxp1	3110052D1
chr4	1.55E+08	1.55E+08	exon (NM_	exon (NM_	731	NM_01138	20481	Ski	2310012I0
chr8	24347902	24347975	intron (NM	CpG	202	NM_02424	109145	Gins4	2810037CC
chr5	1.3E+08	1.3E+08	Intergenic	(CACAC)n|	37412	NM_17246	22648	Zfp11	6720465D2
chr9	52054777	52054850	Intergenic	Intergenic	-78597	NM_00116	244871	Zc3h12c	A230108EC
chr5	1.07E+08	1.07E+08	intron (NM	CpG	798	NM_02685	52397	Zfp644	1110068L0

chr5	97428102	97428175	intron (NM	intron (NM	1430	NM_08070	140780	Bmp2k	4933417M
chr11	68912427	68912525	promoter-T	promoter-T	18	NM_01106	18626	Per1	Per|m-rigu
chr7	1.18E+08	1.18E+08	intron (NM	intron (NM	4945	NM_00966	11717	Ampd3	-
chr12	90602802	90603075	intron (NM	intron (NM	411104	NM_17254	18191	Nrxn3	-
chr15	85863227	85863350	exon (NM_	exon (NM_	919	NM_00988	12614	Celsr1	Crsh|Scy|c
chr11	75267977	75268150	5' UTR (NM	5' UTR (NM	156	NM_13895	192652	Wdr81	BC054822|
chr1	1.59E+08	1.59E+08	intron (NM	intron (NM	20872	NM_02833	72713	Angptl1	2810039D0
chr1	1.67E+08	1.67E+08	promoter-T	promoter-T	-237	NM_14551	108735	Sft2d2	2010005O1
chr7	97624852	97624925	promoter-T	promoter-T	-383	NM_02673	68472	Tmem126b	1110001A2
chr12	1.14E+08	1.14E+08	intron (NM	CpG	462	NM_05408	116870	Mta1	-
chr9	82272002	82272300	Intergenic	URR1A|DN	-451262	NM_00116	65099	Irak1bp1	4921528N0
chr5	1.06E+08	1.06E+08	Intergenic	Intergenic	-20381	NM_00103	433926	Lrrc8b	R75581|Ta
chr4	1.17E+08	1.17E+08	promoter-T	promoter-T	29	NM_02317	66233	Dmap1	1500016M
chr11	17157502	17157850	promoter-T	promoter-T	55	NM_02055	57316	C1d	1110036E1
chr8	59990152	59990275	promoter-T	promoter-T	-427	NM_00825	97165	Hmgb2	C80539|HM
chr2	74447552	74447800	Intergenic	ORR1C2|LT	-30671	NM_02713	69605	Lnp	2310011O1
chr18	55012802	55013025	Intergenic	Intergenic	136921	NM_17575	269023	Zfp608	4932417D1
chr10	1.26E+08	1.26E+08	Intergenic	MLT1L|LTR	88564	NM_02685	68876	Xrcc6bp1	1110068E0
chr11	57775902	57776400	Intergenic	Intergenic	-46415	NM_02845	73158	Larp1	1810024J1
chr13	41583827	41583925	Intergenic	RMER15|LT	-1147	NM_00111	18003	Nedd9	Cas-L|CasL
chr11	11464252	11464425	Intergenic	Intergenic	75069	NM_02866	73862	4930415F1	-
chr15	99869227	99869350	exon (NM_	exon (NM_	193	NM_00115	239667	Dip2b	4932422C2
chr11	1.18E+08	1.18E+08	intron (NM	RSINE1|SIN	-21968	NM_00103	72344	Usp36	2700002L0
chr19	47654752	47655075	intron (NM	CpG	404	NM_00116	20874	Slk	9A2|AV021
chr9	66602077	66602250	intron (NM	intron (NM	40670	NM_17839	76459	Car12	2310047E0
chr3	1.21E+08	1.21E+08	Intergenic	Intergenic	22095	NM_00103	619318	4930432M	-
chr11	66885877	66885975	Intergenic	URR1A|DN	-5876	NM_00109	17883	Myh3	MyHC-emb
chr2	34609527	34609775	intron (NM	CpG	1101	NM_02570	66691	Gapvd1	2010005B0
chr8	38057277	38057450	exon (NM_	exon (NM_	734	NM_00103	621080	AI429214	-
chr8	74247377	74247500	intron (NR_	intron (NR_	2142	NR_02826	74015	Fcho1	3322402E1
chr11	33925227	33925350	Intergenic	Intergenic	-21913	NM_01069	16822	Lcp2	AI323664|
chr17	29088752	29088975	Intergenic	Intergenic	-1298	NM_02588	66989	Kctd20	2410004N1
chr1	1.73E+08	1.73E+08	intron (NM	intron (NM	601	NM_00948	22278	Usf1	bHLHb11
chr19	19824652	19825025	Intergenic	Intergenic	-639152	NM_00104	225998	Rorb	Nr1f2|RZR
chr6	1.25E+08	1.25E+08	intron (NM	intron (NM	12647	NM_00765	12527	Cd9	Tspan29
chr13	8994927	8995150	intron (NM	CpG	220	NM_02700	69237	Gtpbp4	2610028C0
chr8	80610227	80610400	Intergenic	Intergenic	-126928	NM_18309	73301	Ttc29	1700031F1
chr12	84958227	84958425	Intergenic	Intergenic	-14858	NM_02734	67039	Rbm25	2600011C0
chr17	23862952	23863075	promoter-T	promoter-T	-290	NM_02305	268930	Pkmyt1	6230424P1
chr5	1.38E+08	1.38E+08	intron (NM	intron (NM	3404	NM_02391	78829	Tsc22d4	0610009M
chr5	98390752	98391050	intron (NM	intron (NM	69080	NM_13373	71914	Antxr2	2310046B1
chr9	98380827	98380950	Intergenic	RMER15|LT	-10068	NM_00903	19660	Rbp2	Crbp-2|Crb
chr15	53177427	53177500	5' UTR (NM	5' UTR (NM	275	NM_01016	14042	Ext1	AA409028
chr2	1.81E+08	1.81E+08	intron (NM	MYSERV6-i	16086	NM_00127	13002	Dnajc5	2610314I24
chr8	1.22E+08	1.22E+08	Intergenic	Intergenic	-10624	NM_02795	71839	Osgin1	1700012B1
chr7	46705052	46705275	intron (NM	intron (NM	275	NM_00124	233147	Zfp939	9430025M
chr16	77014727	77014825	intron (NM	intron (NM	462	NM_01391	30940	Usp25	-

chr12	77938402	77938600	promoter-T	promoter-T	47	NM_14592	110606	Fntb	2010013E1
chr7	25101252	25101400	intron (NM	intron (NM	359	NM_00956	22756	Zfp94	-
chr1	7099627	7099800	intron (NM	L1MA7|LIN	20712	NM_18302	319263	Pcmtd1	8430411F1
chr12	21128752	21128925	intron (NM	L1MB5|LIN	11221	NM_00113	211914	Asap2	6530401G1
chr3	1.45E+08	1.45E+08	Intergenic	CpG	-3577	NM_01051	16007	Cyr61	AI325051|
chr1	64355102	64355250	Intergenic	Intergenic	-187213	NM_03356	93691	Klf7	9830124P0
chr3	1.05E+08	1.05E+08	intron (NM	intron (NM	1026	NM_01739	53975	Ddx20	GEMIN3|d
chr17	8823927	8824025	intron (NM	intron (NM	104310	NM_00129	23984	Pde10a	-
chr8	63110452	63110575	Intergenic	Intergenic	-1109	NM_02775	71306	Mfap3l	4933428A1
chr4	3622627	3622825	intron (NM	intron (NM	17458	NM_01074	17096	Lyn	AA407514|
chr7	3897777	3898000	Intergenic	Intergenic	-30785	NR_02811	18726	Lilra6	7M1|Pira3
chr3	27998152	27998400	intron (NM	intron (NM	115183	NM_00887	18805	Pld1	AA536939|
chr5	1.04E+08	1.04E+08	intron (NM	intron (NM	72485	NM_17874	246293	Klhl8	2310001P0
chr15	88906502	88906625	promoter-T	promoter-T	69	NM_02648	67976	Trabd	5730502D1
chr16	84835702	84835825	promoter-T	promoter-T	56	NM_01675	11957	Atp5j	-
chr18	35758352	35758450	promoter-T	promoter-T	80	NM_02642	67869	Paip2	2310050K1
chr4	62552202	62552475	Intergenic	Intergenic	-74270	NM_02832	72701	Zfp618	2810031P1
chr10	92718177	92718275	intron (NM	intron (NM	55678	NM_01350	13713	Elk3	D430049E2
chr2	1.74E+08	1.74E+08	exon (NM_	exon (NM_	129	NM_01980	56491	Vapb	AI225786|
chr1	1.34E+08	1.34E+08	intron (NM	intron (NM	599	NM_00879	18557	Cdk18	AA682070|
chr9	90169602	90169750	Intergenic	Intergenic	-4069	NM_19433	67016	Tbc1d2b	1810061M
chr6	52172477	52172600	TTS (NM_0	TTS (NM_0	-3966	NM_01045	15404	Hoxa7	AV118143|
chr2	61370377	61370650	Intergenic	Intergenic	-46130	NM_01152	21353	Tank	C86182|E4
chr11	83287902	83287975	intron (NM	intron (NM	1328	NM_02742	70439	Taf15	2610111C2
chr1	60622927	60623050	intron (NM	intron (NM	621	NM_00104	77300	Raph1	9430025M
chr2	35834377	35834925	intron (NM	CpG	493	NM_02977	74410	Ttll11	4932702F0
chr15	31461427	31461675	promoter-T	promoter-T	-759	NM_17260	223455	6-Mar	3830408G0
chr11	3922277	3922425	Intergenic	RLTR45|LT	-9434	NM_14601	103655	Sec14l4	AI256582|
chr1	1.83E+08	1.83E+08	Intergenic	ORR1C2|LT	7671	NM_13370	69051	Pycr2	1810018M
chr9	1.22E+08	1.22E+08	Intergenic	Intergenic	-44171	NM_00116	20623	Snrk	2010012F0
chr5	1.14E+08	1.14E+08	TTS (NM_0	TTS (NM_0	-11216	NM_00915	20345	Selplg	CD162|Psg
chr8	11464302	11464475	intron (NM	intron (NM	14111	NM_01122	19332	Rab20	AA536966|
chr19	33466177	33466425	intron (NM	CpG	484	NM_00116	67795	Rnls	6530404N2
chr4	1.45E+08	1.45E+08	intron (NM	intron (NM	106536	NM_01130	20148	Dhrs3	Rsdr1|retS
chr8	34739652	34739800	exon (NM_	exon (NM_	12722	NM_17864	108159	Ubxn8	D0H8S229
chr7	88377477	88377775	intron (NM	intron (NM	18936	NM_00880	18584	Pde8a	AI551852|
chr17	43713252	43713575	intron (NM	intron (NM	8013	NM_01373	27226	Pla2g7	R75400
chr6	21900102	21900250	intron (NM	CpG-11892	561	NM_02362	71777	Ing3	1300013A0
chr1	23352552	23352725	Intergenic	Intergenic	37376	NM_00108	70155	Ogfrl1	2210417C1
chr7	1.48E+08	1.48E+08	promoter-T	promoter-T	-503	NM_03069	80876	Ifitm2	DSPA2c|Ifi
chr2	1.27E+08	1.27E+08	promoter-T	promoter-T	-104	NM_02145	58800	Trpm7	2310022G1
chr1	1.56E+08	1.56E+08	Intergenic	BGLII_B|LT	28832	NM_01188	24014	Rnasel	E230029I0
chr17	47659352	47659625	intron (NM	intron (NM	17488	NM_00108	12445	Ccnd3	9230106B0
chr16	44136752	44136925	intron (NM	intron (NM	2294	NM_00750	11964	Atp6v1a	AI647066|
chr1	58502052	58502350	5' UTR (NM	5' UTR (NM	129	NM_02737	70225	Ppil3	2310076N2
chr14	67486552	67486625	intron (NM	CpG	849	NM_00995	12934	Dpysl2	AI851130|
chr14	31764152	31764500	promoter-T	promoter-T	-348	NM_01184	23955	Nek4	-

chr5	1.16E+08	1.16E+08	exon (NM_	exon (NM_	313	NM_00774	12861	Cox6a1	VIaL
chr7	1.34E+08	1.34E+08	promoter-T	promoter-T	-35	NM_02735	70233	Cd2bp2	1500011BC
chr7	20092777	20092875	exon (NM_	exon (NM_	854	NM_17769	232946	Bloc1s3	BC043666|
chr17	45745402	45745675	Intergenic	Lx8|LINE|L	-6487	NM_00117	653016	Gm7325	EG653016|
chr11	70189652	70189825	Intergenic	Intergenic	-24205	NM_00966	11687	Alox15	12-LO|12/1
chr2	31427752	31427950	promoter-T	promoter-T	-320	NM_00103	320267	Fubp3	A330051M
chr17	36407952	36408050	intron (NM	CpG	377	NM_17828	79263	Trim39	1100001D1
chr13	17887777	17887900	intron (NM	intron (NM	9093	NM_00108	69562	Cdk13	2310015O1
chr3	65470052	65470125	promoter-T	promoter-T	-62	NM_00103	624866	Lekr1	EG546798|
chr5	1.35E+08	1.35E+08	TTS (NM_0	TTS (NM_0	-7243	NR_10438	17969	Ncf1	NCF-47K|N
chr14	22524777	22524975	intron (NM	RSINE1|SIN	8922	NM_00101	435391	Dupd1	EG435391
chr11	83084727	83084900	Intergenic	Intergenic	15415	NM_00116	237890	Slfn14	Gm20|Slfn
chr1	1.66E+08	1.66E+08	Intergenic	Intergenic	-85327	NM_00972	11931	Atp1b1	Atp4b|Atp
chr10	26837102	26837275	intron (NM	intron (NM	344870	NM_17683	73910	Arhgap18	4833419J0
chr5	1.24E+08	1.24E+08	Intergenic	Intergenic	-4998	NM_02600	67151	Psmd9	1500011J2
chr5	1.15E+08	1.15E+08	intron (NM	intron (NM	7129	NM_02201	63873	Trpv4	0610033BC
chr8	87549602	87549725	promoter-T	promoter-T	-486	NM_01965	56495	Asna1	1810048H2
chr13	95071452	95071575	Intergenic	Intergenic	-16277	NM_02915	107767	Scamp1	4930505M
chr1	1.45E+08	1.45E+08	intron (NM	intron (NM	-17964	NM_02002	26878	B3galt2	-
chr8	1.27E+08	1.27E+08	promoter-T	promoter-T	252	NM_00111	244666	Sprtn	Gm505
chr1	55267177	55267250	intron (NM	MT2A|LTR	16413	NM_02871	74013	Rftn2	2700010EC
chr12	42825577	42826050	intron (NM	intron (NM	-613169	NM_01073	16981	Lrrn3	NLRR-3
chr11	6460652	6460975	intron (NM	intron (NM	627	NM_00119	216527	Ccm2	BC029157
chr2	13996477	13996625	promoter-T	promoter-T	812	NM_01148	20844	Stam	STAM1
chr15	1.01E+08	1.01E+08	intron (NM	intron (NM	-9182	NM_00127	668218	Bin2	-
chr16	8830702	8830775	exon (NM_	exon (NM_	545	NM_00108	69053	1810013L2	1110017PC
chr12	1.2E+08	1.2E+08	intron (NM	intron (NM	42986	NM_17729	320910	Itgb8	4832412OC
chrX	45695352	45695525	intron (NM	CpG	903	NM_17878	320376	Bcorl1	6720425J0
chr15	96473427	96473600	promoter-T	promoter-T	-169	NM_00116	105727	Slc38a1	AA408026|
chr2	69252902	69253050	Intergenic	MIRb|SINE	34457	NM_17551	241452	Dhrs9	C730025I0
chr19	46945652	46945800	intron (NM	intron (NM	91115	NM_02981	76952	Nt5c2	2010002I2
chr5	7980652	7980975	3' UTR (NM	3' UTR (NM	20341	NM_05409	117167	Steap4	1110021O1
chr4	43970727	43970875	intron (NM	CpG	227	NM_02745	384009	Glipr2	5730414AC
chr15	86058802	86058925	intron (NM	Tigger5|DN	13974	NM_14547	223754	Tbc1d22a	BC023106|
chr4	1.29E+08	1.29E+08	promoter-T	promoter-T	293	NM_02597	67106	Zbtb8os	2010001HC
chr1	9981827	9982100	intron (NR_	intron (NR_	17254	NR_11097	69312	Ppp1r42	1700011J1
chr11	83286577	83286750	promoter-T	promoter-T	53	NM_02742	70439	Taf15	2610111C2
chr2	1.04E+08	1.04E+08	Intergenic	Intergenic	-12189	NM_00114	16909	Lmo2	Rbtn-2|Rbt
chr4	1.41E+08	1.41E+08	Intergenic	B3|SINE|B	-6221	NM_00103	69582	Plekhm2	2310034J1
chr10	79861352	79861625	intron (NM	CpG	736	NM_01359	17192	Mbd3	AI181826|A
chr9	57932952	57933175	intron (NM	intron (NM	16969	NM_02921	382077	Ccdc33	4930535E2
chr3	87791452	87791625	TTS (NM_0	TTS (NM_0	12740	NM_00752	12032	Bcan	Cspg7
chr7	1.27E+08	1.27E+08	promoter-T	promoter-T	-5	NM_02961	73919	Lyrm1	1110065L1
chr17	46848402	46848575	promoter-T	promoter-T	76	NM_14548	224824	Pex6	AI132582|
chr14	79790402	79790525	promoter-T	promoter-T	12	NM_02583	66897	Naa16	1300019CC
chr5	1.26E+08	1.26E+08	intron (NM	L2b|LINE|L	49256	NM_02844	73121	Fam101a	3110032G1
chr11	51567127	51567225	intron (NM	intron (NM	3160	NM_00129	77371	Sec24a	-

chr10	19311827	19312025	exon (NM_	exon (NM_	162	NM_01051	15979	Ifngr1	CD119|IFN
chr5	20621527	20621725	Intergenic	Intergenic	-60011	NM_01120	19248	Ptpn12	P19-PTP|P
chr6	5183852	5184025	intron (NM	intron (NM	22295	NM_17300	269823	Pon3	2810004E2
chr17	29699652	29699800	intron (NM	RSINE1|SIN	12979	NM_19864	381085	Tbc1d22b	BC045600
chr10	1.26E+08	1.26E+08	intron (NM	CpG	1140	NM_00111	52468	Ctdsp2	AI586070|
chr5	37221552	37221750	intron (NM	intron (NM	237	NM_00855	17160	Man2b2	mKIAA093
chr1	59536752	59536875	Intergenic	Intergenic	-2178	NM_00805	14369	Fzd7	Fz7
chr12	73862477	73862575	5' UTR (NM	5' UTR (NM	328	NM_00891	19042	Ppm1a	2310003C2
chr8	73051077	73051325	promoter-T	promoter-T	-441	NM_00111	14232	Fkbp8	38kDa|FKB
chr8	87396077	87396325	promoter-T	promoter-T	44	NM_00116	71846	Syce2	1700013H1
chr6	93577177	93577450	Intergenic	Intergenic	285644	NR_104594	14924	Magi1	AIP3|BAP1
chr7	1.34E+08	1.34E+08	intron (NM	intron (NM	554	NM_02682	68742	Tmem219	1110032O1
chr4	1.01E+08	1.01E+08	Intergenic	Intergenic	-27289	NM_14614	16451	Jak1	AA960307|
chr14	21613577	21614000	promoter-T	promoter-T	-478	NM_17859	12325	Camk2g	Camkg
chr7	26429977	26430225	intron (NM	intron (NM	13679	NM_00753	12039	Bckdha	-
chr14	75983502	75983625	exon (NM_	exon (NM_	8229	NM_02645	67926	Spert	1700086N0
chr13	52577177	52577325	Intergenic	Intergenic	48954	NM_00102	68203	Diras2	2900052J1
chr11	1.02E+08	1.02E+08	promoter-T	promoter-T	33	NM_02964	76547	Tmem101	2610511E2
chr4	1.34E+08	1.34E+08	Intergenic	Intergenic	-11469	NM_14555	100017	Ldlrap1	AA691260|
chr4	94123552	94123700	Intergenic	Intergenic	99861	NM_02636	67770	Caap1	5830433M
chr1	60155252	60155400	promoter-T	promoter-T	18	NM_00119	57750	Wdr12	4933402C2
chr6	66904102	66904575	intron (NM	intron (NM	57453	NM_00117	14701	Gng12	2010305F1
chr5	1.4E+08	1.4E+08	Intergenic	MTC-int|LT	31136	NM_17485	231830	Micall2	A930021H1
chr3	1.22E+08	1.22E+08	intron (NM	CpG	306	NM_15380	99480	Dnttip2	4930588M
chr6	1.17E+08	1.17E+08	Intergenic	Intergenic	225173	NM_02170	20315	Cxcl12	Pbsf|Scyb1
chr5	1.23E+08	1.23E+08	promoter-T	promoter-T	128	NM_14491	231724	Rad9b	A630082N1
chr5	5559302	5559425	intron (NM	CpG	138	NM_15311	207704	Gtpbp10	4930545J2
chr8	1.13E+08	1.13E+08	intron (NM	intron (NM	3961	NM_00917	20444	St3gal2	AI429591|
chr10	1.27E+08	1.27E+08	Intergenic	Intergenic	-2879	NM_00928	20852	Stat6	-
chr1	1.82E+08	1.82E+08	intron (NM	intron (NM	526	NM_17865	109232	Sccpdh	AW214504
chr3	89576702	89576900	promoter-T	promoter-T	-730	NM_02731	70093	Ube2q1	1110002C0
chr12	1.18E+08	1.18E+08	exon (NM_	exon (NM_	37651	NM_13376	76044	Ncapg2	5830426I0
chr13	37575002	37575100	Intergenic	Intergenic	137837	NM_01074	17084	Ly86	MD-1|MD1
chr8	72826752	72826875	promoter-T	promoter-T	129	NM_02153	59042	Cope	1110005D1
chr3	68360752	68360975	intron (NM	L2|LINE|L2	-15427	NM_01392	30953	Schip1	Nf2ip|Schi
chr11	60643952	60644300	promoter-T	promoter-T	-7	NM_00117	216820	Dhrs7b	BC003479|
chr11	76576652	76577125	intron (NM	intron (NM	169	NM_01681	53334	Gosr1	AI414660|
chr6	1.16E+08	1.16E+08	intron (NM	MIRb|SINE	43545	NM_00966	11689	Alox5	5-LO|5-LO
chr8	81032252	81033025	promoter-T	promoter-T	189	NM_00125	19656	Rbmxl1	Hnrpg|Rbn
chr7	75338827	75339025	intron (NM	intron (NM	70193	NM_03010	78444	Pgpep1l	C330024D1
chr19	5572052	5572225	TTS (NM_0	TTS (NM_0	4064	NM_00103	381201	Ap5b1	Gm962
chr11	8726652	8726975	Intergenic	Intergenic	-162275	NM_00108	319939	Tns3	BC023928|
chr2	48622152	48622275	Intergenic	B4A|SINE|	-47416	NM_00739	11480	Acvr2a	ActrIIa|Acv
chr12	72070002	72070225	Intergenic	tRNA-Lys-A	-5497	NM_01118	19167	Psma3	Lmpc8
chr10	75384077	75384150	exon (NM_	exon (NM_	246	NM_01141	20587	Smarcb1	AU020204
chr1	1.63E+08	1.63E+08	Intergenic	Intergenic	-17104	NM_00745	11758	Prdx6	1-cysPrx|9
chr5	88983377	88983650	promoter-T	promoter-T	5	NM_02305	65961	Utp3	2400011K0

chr2	1.03E+08	1.03E+08	3' UTR (NM	3' UTR (NM	25863	NM_01012	13711	Elf5	ESE-2|ESE-
chr2	30930777	30930950	intron (NM	intron (NM	21157	NM_00117	14269	Fnbp1	1110057E0
chr19	44461877	44461975	Intergenic	Intergenic	20273	NM_00912	20249	Scd1	AA589638|
chr6	1.45E+08	1.45E+08	intron (NM	MTEa|LTR|	23464	NM_17722	320662	Casc1	A230084G1
chr15	27713102	27713200	intron (NM	intron (NM	-101854	NM_00124	223433	Fam105a	9830126M
chr13	94070902	94071150	Intergenic	Intergenic	-3424	NM_14717	26556	Homer1	PSD-Zip45|
chr4	19969552	19969625	intron (NM	intron (NM	389	NM_01028	14590	Ggh	gamma-GH
chr3	32635277	32635375	promoter-T	promoter-T	351	NM_02901	74600	Mrpl47	4833424P1
chr16	26369202	26369275	intron (NM	intron (NM	2687	NM_01667	12737	Cldn1	AI596271
chr15	78893002	78893150	exon (NM_	exon (NM_	213	NM_14484	223690	Ankrd54	C730048E1
chr1	1.3E+08	1.3E+08	Intergenic	Intergenic	-43158	NM_14550	226414	Dars	5730439G1
chr16	36455602	36455875	promoter-T	promoter-T	-260	NM_02528	20863	Stfa3	Stf3
chr19	5731202	5731275	intron (NM	intron (NM	494	NM_02049	56390	Sssca1	1500016H1
chr17	35337327	35337475	exon (NM_	exon (NM_	1551	NM_01369	21926	Tnf	DIF|TNF-a|
chr5	91212677	91212800	Intergenic	Intergenic	-2389	NM_20332	330122	Cxcl3	Dcip1|Gm1
chr13	55999852	55999950	Intergenic	MIRb|SINE	-67115	NM_01109	18740	Pitx1	Bft|P-OTX|
chr11	62462477	62462550	promoter-T	promoter-T	347	NM_17500	216829	Mmgt2	AI852046
chr9	63563977	63564125	intron (NM	intron (NM	41750	NM_01676	17127	Smad3	AU022421
chr7	66485052	66485150	intron (NM	intron (NM	979	NM_01166	22215	Ube3a	4732496B0
chrX	11403227	11403300	Intergenic	L1MC4|LIN	254416	NM_17504	71458	Bcor	5830466J1
chr11	1.18E+08	1.18E+08	Intergenic	B4|SINE|B4	-1306	NM_00126	76025	Cant1	5830420C2
chr5	65906227	65906375	Intergenic	Intergenic	-22199	NM_01678	53323	Ube2k	AW492011
chr7	1.48E+08	1.48E+08	promoter-T	promoter-T	-479	NM_15377	70552	Lrrc56	5730427C2
chr4	1.26E+08	1.26E+08	promoter-T	promoter-T	-677	NM_15340	214150	Ago3	AW048688
chr6	1.16E+08	1.16E+08	intron (NM	intron (NM	2308	NM_00966	11689	Alox5	5-LO|5-LOX
chr2	1.29E+08	1.29E+08	intron (NM	CpG	430	NM_02059	57432	Zc3h8	AU020882
chr2	38859302	38861325	exon (NM_	exon (NM_	338	NM_02559	66489	Rpl35	2410039E0
chr5	65781252	65783175	promoter-T	promoter-T	457	NM_01129	20005	Rpl9	-
chr3	1.29E+08	1.29E+08	Intergenic	Intergenic	-86470	NM_00128	18741	Pitx2	9430085M
chr9	50333127	50333300	intron (NM	intron (NM	3533	NM_01122	19286	Pts	PTPS
chr17	34258652	34258725	promoter-T	promoter-T	4	NM_01023	14312	Brd2	AW228947
chr6	1.25E+08	1.25E+08	intron (NM	intron (NM	-4012	NM_00941	21991	Tpi1	AI255506|T
chr7	87469027	87469350	TTS (NM_1	TTS (NM_1	152	NM_17344	269955	Rccd1	5830436H0
chr7	24992377	24993075	intron (NM	intron (NM	442	NM_01994	56707	Zfp111	-
chr9	59386752	59386850	promoter-T	promoter-T	-673	NM_01042	15211	Hexa	Hex-1
chr3	1.16E+08	1.16E+08	exon (NM_	exon (NM_	5193	NM_01169	22329	Vcam1	CD106|Vca
chr7	1.17E+08	1.17E+08	promoter-T	promoter-T	-344	NM_15352	233724	Tmem41b	1500015G0
chr7	52828677	52828850	intron (NM	intron (NM	523	NM_00124	12036	Bcat2	Bcat-2|Eca
chr16	49800502	49800675	Intergenic	Intergenic	-55179	NM_01058	16423	Cd47	9130415E2
chr10	74674752	74675050	promoter-T	promoter-T	83	NM_15340	74392	Specc1l	4930470P1
chr7	26004277	26004350	intron (NM	CpG	565	NM_20767	67379	Dedd2	2410050E1
chr12	1.04E+08	1.04E+08	promoter-T	promoter-T	-583	NM_17762	217835	Rin3	6430500K0
chr8	1.11E+08	1.11E+08	intron (NM	intron (NM	34269	NM_00749	11906	Zfhx3	A230102L0
chr9	55955902	55956400	intron (NM	intron (NM	18368	NM_01119	19200	Pstpip1	CD2BP1|de
chr1	1.68E+08	1.68E+08	intron (NM	CpG	346	NM_00100	68481	Mpzl1	1110007A1
chr9	86438252	86438425	intron (NM	intron (NM	27111	NM_00116	109785	Pgm3	2810473H0
chr7	25292002	25292150	promoter-T	promoter-T	-30	NM_00129	232969	Zfp428	2410005H0

chr2	28695577	28695725	promoter-T	promoter-T	229	NM_17297	269252	Gtf3c4	5330400C0
chr7	20250902	20251325	promoter-T	promoter-T	-734	NM_01964	56457	Clptm1	HS9|N14
chr4	1.07E+08	1.07E+08	Intergenic	CpG	-39740	NM_02559	329910	Acot11	1110020M
chr7	1.45E+08	1.45E+08	intron (NM	ORR1E|LTF	173250	NM_18328	70571	Tcerg1l	5730476P1
chr12	1.14E+08	1.14E+08	promoter-T	promoter-T	-472	NM_02539	66174	Nudt14	1110030M
chr10	3592777	3593000	Intergenic	RCHARR1|I	-34948	NM_00103	18390	Oprm1	M-OR-1|M
chr4	32051127	32051250	5' UTR (NM	5' UTR (NM	106	NM_17268	26409	Map3k7	B430101B0
chr2	1.33E+08	1.33E+08	promoter-T	promoter-T	24	NM_00102	66586	Crls1	0610009I2
chr13	9158427	9158825	intron (NM	intron (NM	65475	NM_17258	217980	Larp4b	A630096F1
chr1	1.93E+08	1.93E+08	Intergenic	Intergenic	-24368	NM_14488	226849	Ppp2r5a	PR61alpha
chr3	1.08E+08	1.08E+08	Intergenic	Intergenic	-26651	NM_00111	12977	Csf1	C87615|Cs
chr13	69080152	69080325	intron (NM	intron (NM	58181	NM_15353	210044	Adcy2	mKIAA106(
chr9	26915052	26915200	intron (NM	Lx8|LINE|L	47880	NM_02327	83964	Jam3	1110002N2
chr11	99092077	99092200	5' UTR (NM	5' UTR (NM	193	NM_02061	57376	Smarce1	2810417B2
chr19	46586152	46586275	intron (NM	intron (NM	10075	NM_05310	93679	Trim8	AA408830|
chr6	1.35E+08	1.35E+08	intron (NM	GC_rich|Lc	470	NM_13044	70686	Dusp16	3830417M
chr4	1.34E+08	1.34E+08	promoter-T	promoter-T	-88	NM_00128	71904	Paqr7	2310021M
chr16	37874602	37874900	intron (NM	Tigger7|DN	6265	NM_17709	320184	Lrrc58	1810012N1
chr9	41758977	41759150	Intergenic	Intergenic	173309	NM_01143	20660	Sorl1	2900010L1
chr1	90173052	90174525	intron (NM	CpG	344	NM_17250	212427	A730008H2	mFleg1
chr1	1.54E+08	1.54E+08	promoter-T	promoter-T	-991	NM_00103	66967	Edem3	2310050N1
chr2	17985677	17985800	promoter-T	promoter-T	-474	NM_00125	17354	Mllt10	Af10|B130
chr3	83880502	83880725	Intergenic	MIRb|SINE	-36530	NM_17268	229473	D930015E0	Kiaa0922|r
chr19	46471327	46471675	5' UTR (NM	5' UTR (NM	115	NM_01575	24069	Sufu	Su(fu)
chr6	1.15E+08	1.15E+08	intron (NM	intron (NM	44132	NM_00125	74244	Atg7	1810013K2
chr15	25902677	25902800	3' UTR (NM	3' UTR (NM	-11383	NM_14452	52521	Zfp622	1110033B0
chr12	1.06E+08	1.06E+08	promoter-T	promoter-T	164	NM_02841	73046	Glrx5	2310004O1
chr15	42460227	42460400	intron (NM	intron (NM	48210	NM_00128	11600	Angpt1	1110046O2
chr1	10028977	10029150	promoter-T	promoter-T	764	NM_02649	211660	Cspp1	2310020J1
chr1	1.74E+08	1.74E+08	intron (NM	intron (NM	488	NM_08041	140559	Igsf8	AA033172|
chr2	48669002	48669150	promoter-T	promoter-T	-553	NM_00739	11480	Acvr2a	ActrIIa|Acv
chr1	52176027	52176675	promoter-T	promoter-T	69	NM_00120	20846	Stat1	2010005J0
chr2	72314627	72314850	intron (NM	CpG	462	NM_02586	66953	Cdca7	2310021G0
chr1	66729577	66729650	intron (NM	intron (NM	-17854	NM_02568	66646	Rpe	2810429B0
chr8	73283877	73283975	intron (NM	B1_Mur1|S	894	NM_18317	234384	Mpv17l2	Fksg24
chr7	4744352	4744875	promoter-T	promoter-T	46	NM_00908	19943	Rpl28	D7Wsu21e
chr2	81842352	81842625	Intergenic	Intergenic	-51327	NM_17551	241514	Zfp804a	C630007C1
chrY	2879052	2879125	Intergenic	L1_Mm|LIN	491830	NM_00127	1E+08	Gm3376	Rbmy1b
chr15	80049877	80050575	Intergenic	Intergenic	-14284	NM_17871	239555	Mief1	A230016E2
chr1	1.37E+08	1.37E+08	promoter-T	promoter-T	-212	NM_01159	21854	Timm17a	17kDa|mTi
chr5	1.13E+08	1.13E+08	intron (NM	MIRc|SINE	-10139	NM_00941	22022	Tpst2	AI448750|I
chr16	38550177	38550375	promoter-T	promoter-T	62	NM_17238	224143	Poglut1	9630046K2
chr9	20702077	20702150	intron (NM	intron (NM	921	NM_01687	53356	Eif3g	44kDa|D0J
chr11	1.16E+08	1.16E+08	Intergenic	Intergenic	-4165	NM_00816	14784	Grb2	AA408164|
chr1	1.67E+08	1.67E+08	promoter-T	promoter-T	-799	NM_14551	108735	Sft2d2	2010005O1
chr8	23587302	23587525	promoter-T	promoter-T	-62	NM_00114	102032	Smim19	AI316807|I
chr9	77503227	77503350	exon (NM_	exon (NM_	18749	NM_17292	244923	Klhl31	9830147P1

chr7	20462252	20462425	3' UTR (NM	3' UTR (NM	10976	NM_17703	319930	Ceacam19	C130022P0
chrX	96133252	96133425	5' UTR (NM	5' UTR (NM	218	NM_20763	77929	Yipf6	A430107J0
chr14	70945277	70945450	5' UTR (NM	5' UTR (NM	305	NM_18058	72549	Reep4	2700029E1
chr8	1.19E+08	1.19E+08	intron (NM	MIR|SINE|	105090	NM_02944	75796	Cdyl2	1700029M
chr3	87689952	87690025	promoter-T	promoter-T	-504	NM_03357	94315	Prcc	-
chr5	1.24E+08	1.24E+08	intron (NM	CpG	781	NM_02985	77045	Bcl7a	4432415N0
chr19	41922727	41922800	promoter-T	promoter-T	-141	NM_17760	212398	Frat2	-
chr5	64551452	64551625	promoter-T	promoter-T	39	NM_01963	57915	Tbc1d1	1110062G0
chr2	1.52E+08	1.52E+08	intron (NM	CpG	237	NM_00778	12995	Csnk2a1	Csnk2a1-rs
chr2	25066677	25067075	5' UTR (NM	5' UTR (NM	133	NM_02139	58202	Nelfb	A730008L0
chr15	75687777	75687900	Intergenic	Intergenic	-4931	NM_02696	69146	Gsdmd	1810036L0
chr1	95341752	95342150	intron (NM	CpG	327	NM_00130	110611	Hdlbp	1110005P1
chr18	13107777	13107950	Intergenic	Intergenic	-7513	NM_20753	64291	Osbpl1a	G430090F1
chr10	1.28E+08	1.28E+08	Intergenic	Intergenic	-6385	NM_17225	216459	Myl6b	5730437E0
chr9	1.24E+08	1.24E+08	Intergenic	MLT1A|LTF	-3222	NM_00987	12581	Cdkn2d	INK4d|p19
chr1	67441227	67441425	Intergenic	Intergenic	271725	NM_00108	227231	Cps1	4732433M
chr11	93769252	93769425	intron (NM	intron (NM	21805	NM_13401	103537	Mbtd1	AA408199|
chr1	1.83E+08	1.83E+08	Intergenic	Intergenic	31764	NM_13322	170760	Acbd3	60kDa|843
chr4	1.48E+08	1.48E+08	Intergenic	Intergenic	77934	NM_02787	71707	Ubiad1	1200002M
chr11	62063027	62063150	intron (NM	intron (NM	602	NM_00741	11541	Adora2b	A2BAR|A2I
chr3	5404527	5404875	intron (NM	intron (NM	171547	NM_00116	19302	Pex2	D3Ertd138
chr7	26063527	26063650	Intergenic	CpG	-3610	NM_00111	71722	Cic	1200010B1
chr14	57504352	57504550	Intergenic	CpG	-2181	NM_02949	76007	Zmym2	5830413P0
chr5	1.44E+08	1.44E+08	promoter-T	promoter-T	16	NM_02837	72881	Zdhhc4	1810021D0
chr9	32711327	32711475	Intergenic	MIR|SINE|	207774	NM_00103	23871	Ets1	AI196000|
chr9	1.15E+08	1.15E+08	intron (NM	intron (NM	842	NM_17538	333433	Gpd1l	2210409H2
chr9	50606527	50606850	intron (NR_	intron (NR_	23358	NR_04560	102580	Alg9	8230402H1
chr2	1.81E+08	1.81E+08	intron (NM	intron (NM	653	NM_17376	228994	Ythdf1	2210410K2
chr8	23916577	23916775	promoter-T	promoter-T	-550	NM_00112	64933	Ap3m2	5830445E1
chr2	1.58E+08	1.58E+08	intron (NM	intron (NM	-73251	NM_19862	277432	Vstm2l	Gm691
chr5	35917427	35917600	exon (NM_	exon (NM_	206	NM_03020	78890	Trmt44	2310079F2
chr12	82695952	82696125	promoter-T	promoter-T	-93	NM_02721	69792	Med6	1500012F1
chr1	1.83E+08	1.83E+08	intron (NR_	CpG	295	NR_12049	72568	Lin9	2700022J2
chr2	1.3E+08	1.3E+08	Intergenic	Intergenic	-69313	NM_00937	21818	Tgm3	AI893889|
chr2	1.04E+08	1.04E+08	Intergenic	Intergenic	-11289	NM_00114	16909	Lmo2	Rbtn-2|Rbt
chr2	1.57E+08	1.57E+08	promoter-T	promoter-T	-227	NM_02585	66934	Dsn1	1700022L0
chr2	44941252	44941350	intron (NM	intron (NM	24496	NM_00128	24136	Zeb2	9130203F0
chr4	1.55E+08	1.55E+08	promoter-T	promoter-T	-76	NM_02557	66448	Mrpl20	2610008D0
chr10	12895602	12895925	intron (NM	intron (NM	2066	NM_02917	75122	Zc2hc1b	4930519B0
chr3	1.35E+08	1.35E+08	intron (NM	intron (NM	13965	NM_02535	66105	Ube2d3	1100001F1
chr2	84198902	84199100	intron (NM	intron (NM	66422	NM_01878	54598	Calcrl	AV071593|
chr16	38710202	38710275	intron (NM	intron (NM	2910	NM_02026	12549	Arhgap31	5830477L0
chr19	46402977	46403275	intron (NM	CpG	452	NM_13369	68431	Fbxl15	0710008C1
chr18	77168802	77168925	promoter-T	promoter-T	97	NM_02540	66191	Ier3ip1	1110057H1
chr9	13631102	13631175	non-coding	non-coding	359	NR_03762	74360	Cep57	3110002L1
chr2	1.04E+08	1.04E+08	intron (NM	intron (NM	67440	NM_00103	241589	D430041D0	9630056G0
chr12	83240902	83241050	Intergenic	Intergenic	-30027	NM_00116	217692	Sipa1l1	4931426N1

chr10	17270502	17270750	Intergenic	L4|LINE|RT	-172408	NM_01082	17684	Cited2	AI835299|
chr9	22029727	22030025	promoter-T	promoter-T	-271	NM_17276	235047	Zfp809	BB114266|
chr3	1.22E+08	1.22E+08	intron (NM	intron (NM	32294	NM_00111	214459	Fnbp1l	2610318I0
chr7	1.43E+08	1.43E+08	Intergenic	Intergenic	-19639	NM_00108	17345	Mki67	D630048A1
chr11	48684752	48685050	promoter-T	promoter-T	-53	NM_00832	15944	Irgm1	Ifggd3|Ifi1
chr3	95033202	95033325	promoter-T	promoter-T	398	NM_00103	57912	Cdc42se1	1300002M
chr3	63469177	63469275	Intergenic	Intergenic	185687	NM_00117	269437	Plch1	BC042549|
chr11	29926002	29926125	promoter-T	promoter-T	-30	NM_14601	237711	Eml6	2900083P1
chr6	90270577	90270750	intron (NM	intron (NM	4516	NM_02792	71797	Chst13	1110067M
chr7	35903552	35903650	promoter-T	promoter-T	-711	NM_00128	12606	Cebpa	C/ebpalpha
chr10	80023752	80024275	exon (NM_	exon (NM_	292	NM_02585	66932	Rexo1	1700021P1
chr7	78817102	78817250	Intergenic	B1F|SINE|/	634305	NM_00102	244049	Mctp2	Gm489
chr5	1.22E+08	1.22E+08	Intergenic	Intergenic	-1828	NM_00850	16923	Sh2b3	AI429800|
chr12	76393052	76393225	Intergenic	RMER19B|	-16162	NM_02327	80837	Rhoj	1110005O1
chr9	1.06E+08	1.06E+08	promoter-T	promoter-T	88	NM_00108	75669	Pik3r4	2210010O1
chr10	94764877	94765025	intron (NM	intron (NM	21780	NM_00995	12905	Cradd	RAIDD
chr14	45657777	45658050	Intergenic	L1MB5|LIN	50127	NM_00896	19217	Ptger2	EP2|Ptgere
chr9	69287677	69287825	Intergenic	Intergenic	-13739	NM_00758	12306	Anxa2	AW215814
chr14	79248777	79249225	promoter-T	promoter-T	16	NM_17375	219189	Vwa8	1300010F0
chr15	57738277	57738550	Intergenic	Intergenic	-5341	NM_00108	210544	Tbc1d31	4B3|D3300
chr10	1.21E+08	1.21E+08	promoter-T	promoter-T	-416	NM_00108	73192	Xpot	1110004L0
chr8	1.29E+08	1.29E+08	Intergenic	Intergenic	-2772	NM_17276	52202	Rbm34	4930547K0
chr6	86535352	86535475	Intergenic	Intergenic	-42755	NM_02641	67855	Asprv1	2300003P2
chr19	7115127	7115325	promoter-T	promoter-T	-710	NM_01673	20867	Stip1	Hop|Sti1|p
chr13	56280077	56280225	promoter-T	promoter-T	95	NM_14597	212937	Tifab	-
chr12	93038327	93038500	Intergenic	RMER30|D	-13537	NM_17536	108800	Ston2	4933401N2
chr9	61271652	61272000	Intergenic	Intergenic	51653	NM_00108	21887	Tle3	2610103N0
chr1	17598477	17598850	intron (NM	L2|LINE|L2	6681	NM_05319	94227	Pi15	P24TI|P25T
chr15	98592877	98592950	intron (NM	CpG	636	NM_00747	11842	Arf3	5430400P1
chr7	14702077	14702375	intron (NM	intron (NM	6116	NM_00110	629203	Sult2a3	EG629203|
chr6	1.03E+08	1.03E+08	intron (NM	intron (NM	18343	NM_00769	12661	Chl1	A530023M
chr2	25477027	25477125	intron (NM	intron (NM	446	NM_00104	51875	Tmem141	1110065P1
chr4	1.21E+08	1.21E+08	Intergenic	Intergenic	-8587	NM_00108	109263	Rlf	9230110M
chr11	1.11E+08	1.11E+08	Intergenic	Intergenic	35723	NM_00842	16518	Kcnj2	IRK1|Kcnf1
chr7	1.04E+08	1.04E+08	intron (NM	intron (NM	114761	NM_00117	244144	Usp35	Gm1088|G
chr17	31099527	31099725	intron (NM	intron (NM	7998	NM_17746	52020	Umodl1	D17Ertd48
chr5	96639852	96639925	intron (NM	intron (NM	754	NM_05315	94061	Mrpl1	2410002L0
chr5	92555902	92556025	Intergenic	Intergenic	-11001	NM_01949	56041	Uso1	115kDa|TA
chr17	34743327	34743600	intron (NM	intron (NM	657	NM_00116	55979	Agpat1	1-AGP|1-A
chr6	55940302	55940400	Intergenic	RLTR45|LT	-27158	NM_01115	19051	Ppp1r17	Gsbs|Ppp1
chr1	39526002	39526100	intron (NM	intron (NM	9541	NM_01877	54610	Tbc1d8	AD3|HBLP1
chr4	1.39E+08	1.39E+08	intron (NM	CpG	626	NR_03374	433771	Minos1	2310028O1
chr4	59470827	59470925	Intergenic	Zaphod|DN	-19371	NM_00116	634731	Susd1	A530080P1
chr2	1.53E+08	1.53E+08	Intergenic	Intergenic	10664	NM_19861	241732	Tspyl3	AW212607
chr10	41023052	41023200	promoter-T	promoter-T	-79	NM_13399	103199	Fig4	A530089I1
chr3	68960352	68960425	intron (NM	intron (NM	25233	NM_00124	381452	Gm1647	-
chr8	1.26E+08	1.26E+08	promoter-T	promoter-T	-128	NM_19444	234854	Cdk10	BC017131

chr1	1.84E+08	1.84E+08	exon (NM_	exon (NM_	211	NM_17512	67948	Fbxo28	4833428J1
chr16	18426177	18426625	promoter-T	promoter-T	-109	NM_01371	26462	Txnrd2	AA118373|
chr1	54894677	54894975	intron (NM	intron (NM	88405	NM_00108	329154	Ankrd44	4930444A1
chrX	1.48E+08	1.48E+08	promoter-T	promoter-T	-513	NM_01676	15108	Hsd17b10	17bHSD10
chr7	27980327	27980500	promoter-T	promoter-T	366	NM_00104	53607	Snrpa	C430021M
chr11	72775077	72775175	intron (NM	intron (NM	455	NM_00116	53313	Atp2a3	SERCA3b|S
chr3	1.04E+08	1.04E+08	exon (NM_	exon (NM_	191	NM_00102	269473	Lrig2	4632419I1
chr10	6547702	6547850	intron (NM	intron (NM	58388	NM_00115	213783	Plekhg1	D10Ertd73
chr6	29195752	29195825	Intergenic	Intergenic	-26700	NM_00119	69573	Hilpda	2310016C0
chr2	22908302	22908450	Intergenic	Intergenic	-12615	NM_14599	11308	Abi1	E3B1|NAP1
chr7	75276477	75276575	intron (NM	intron (NM	132593	NM_03010	78444	Pgpep1l	C330024D1
chr11	1.06E+08	1.06E+08	promoter-T	promoter-T	-170	NM_02600	67163	Ccdc47	2610204L2
chr18	74938827	74938975	promoter-T	promoter-T	35	NM_17747	52538	Acaa2	0610011L0
chr4	1.25E+08	1.25E+08	intron (NM	ID_B1|SINE	11402	NM_13868	192199	Rspo1	R-spondin|
chr10	96118827	96119025	Intergenic	Intergenic	39291	NM_00756	12226	Btg1	AI426953|A
chr10	87880302	87880400	intron (NM	intron (NM	38194	NM_00100	432486	Gnptab	EG432486|
chr2	1.74E+08	1.74E+08	intron (NR_	CpG-8954	1073	NR_003258	14683	Gnas	5530400H2
chr18	42974527	42974600	intron (NM	MTB|LTR|I	84597	NR_073583	72930	Ppp2r2b	2900026H0
chr8	36438577	36438650	promoter-T	promoter-T	-182	NM_17774	244416	Ppp1r3b	6430576E2
chr11	1.15E+08	1.15E+08	intron (NM	intron (NM	18993	NM_00116	58222	Rab37	B230331O0
chr9	61223827	61224000	intron (NM	intron (NM	3740	NM_00108	21887	Tle3	2610103N0
chr5	1.07E+08	1.07E+08	intron (NM	CpG	473	NM_02685	52397	Zfp644	1110068L0
chr3	1.09E+08	1.09E+08	Intergenic	RCHARR1|I	-13488	NM_00116	329739	Fam102b	1600010D1
chr6	47404377	47404450	promoter-T	promoter-T	90	NM_01204	26965	Cul1	-
chr2	1.44E+08	1.44E+08	intron (NM	intron (NM	971	NM_01977	56431	Dstn	2610043P1
chr10	42682527	42682625	Intergenic	Intergenic	102258	NM_17293	268297	Scml4	9330161D1
chr10	43297552	43297625	Intergenic	Intergenic	-1387	NM_00984	12484	Cd24a	Cd24|HSA|
chr17	57997602	57997700	intron (NM	intron (NM	88658	NM_00108	620292	Cntnap5c	Caspr5-3|E
chr17	31353102	31353250	intron (NM	MLT1C|LTR	8165	NM_17782	328795	Ubash3a	5830413C0
chr8	1.23E+08	1.23E+08	Intergenic	Intergenic	-119978	NM_19867	382034	Gse1	2210013I1
chr8	34762852	34763100	promoter-T	promoter-T	-734	NM_01034	14782	Gsr	AI325518|I
chr4	1.41E+08	1.41E+08	intron (NM	(TA)n|Simp	771	NM_00101	68817	Ddi2	1110056G1
chr15	36936677	36936775	intron (NM	CpG	431	NM_02652	68036	Zfp706	3110006P0
chr1	1.93E+08	1.93E+08	intron (NM	B3|SINE|B	16575	NM_17863	77065	Ints7	5930412E2
chr15	98863652	98863775	intron (NM	intron (NM	3391	NM_00944	22146	Tuba1c	M[a]6|Tub
chr13	43345927	43346000	intron (NM	intron (NM	53578	NM_00103	328232	Gfod1	9630032O1
chr1	36353977	36354100	Intergenic	Intergenic	-10540	NM_00129	214855	Arid5a	D430024K2
chr11	23206927	23207150	intron (NM	CpG-1773	143	NM_00119	17847	Usp34	A530081C0
chr4	9609852	9610200	Intergenic	MTEb|LTR	-13535	NM_00129	65973	Asph	2310005F1
chr15	78551002	78551275	Intergenic	Intergenic	-2595	NM_18314	207393	Elfn2	6330514E1
chr1	75518127	75518250	promoter-T	promoter-T	84	NM_02788	71728	Stk11ip	1200014D2
chr1	1.69E+08	1.69E+08	intron (NM	intron (NM	540	NM_02556	66447	Mgst3	2010012L1
chr13	98862702	98862850	intron (NM	intron (NM	113344	NM_01202	110596	Arhgef28	9230110L0
chr12	35063502	35063750	intron (NM	intron (NM	150128	NM_02412	79221	Hdac9	AV022454|
chr7	1.29E+08	1.29E+08	intron (NM	intron (NM	-18819	NM_00103	233824	Cog7	5630400E2
chr19	45734352	45734650	exon (NM_	exon (NM_	182	NM_01390	30838	Fbxw4	Dac|Fbw4|
chr8	1.2E+08	1.2E+08	intron (NM	intron (NM	2569	NM_00116	74440	Cmip	4933407C0

chr10	93605327	93605525	3' UTR (NM	3' UTR (NM	-5250	NM_01162	22025	Nr2c1	4831444H0
chr5	53174277	53174425	promoter-T	promoter-T	45	NM_03018	78796	Zcchc4	4930449I2
chr5	33695127	33695350	intron (NM	L1MB7|LIN	17017	NM_02150	59003	Maea	1110030D1
chr5	1.23E+08	1.23E+08	intron (NM	intron (NM	463	NM_00128	59008	Anapc5	2510006G1
chr5	73304952	73305100	promoter-T	promoter-T	-575	NM_00111	75991	Slain2	5033405K1
chr16	45148977	45149075	intron (NM	intron (NM	9760	NM_02875	74102	Slc35a5	1010001J0
chr13	77274452	77274625	promoter-T	promoter-T	191	NM_13407	105377	Ankrd32	2700017A0
chr6	90687027	90687100	intron (NM	CpG	-20540	NM_00113	232227	Iqsec1	AW561907
chr5	1.18E+08	1.18E+08	intron (NM	CpG	309	NM_14556	231670	Fbxo21	2810425J2
chr2	1.07E+08	1.07E+08	intron (NM	intron (NM	1241	NM_00102	212772	Arl14ep	2700007P2
chr6	1.15E+08	1.15E+08	Intergenic	Intergenic	-25196	NM_19903	381802	Tsen2	AU067695
chr3	54611877	54612475	promoter-T	promoter-T	-463	NM_13323	170767	Rfxap	5730495K2
chr14	75579752	75579825	intron (NM	intron (NM	43556	NM_00887	18826	Lcp1	AW536232
chr15	63831877	63832000	intron (NM	(TG)n|Simp	60072	NM_14484	223601	Fam49b	0910001A0
chr5	44617627	44617700	exon (NM_	exon (NM_	182	NM_17376	231225	Tapt1	4932414K1
chr11	80606952	80607250	Intergenic	MER20|DN	-13574	NM_17739	338367	Myo1d	9930104H0
chr10	58122452	58122525	intron (NM	intron (NM	15956	NM_01010	13608	Edar	ED1R|ED3|
chr6	50256652	50257000	intron (NM	intron (NM	-45058	NM_01876	54722	Dfna5	2310037D0
chr11	78159227	78159325	intron (NM	intron (NM	2252	NM_01167	22248	Unc119	D11Bhm52
chr13	37749177	37749400	Intergenic	Intergenic	-168619	NM_02683	68750	Rreb1	1110037N0
chr13	1.01E+08	1.01E+08	promoter-T	promoter-T	-122	NM_01142	20595	Smn1	AI849087|
chr12	1.04E+08	1.04E+08	intron (NM	intron (NM	344	NM_01117	19141	Lgmn	AEP|AI746
chr3	97419827	97420150	Intergenic	Intergenic	-5875	NM_02653	68058	Chd1l	4432404A2
chr9	1.08E+08	1.08E+08	intron (NM	CpG	168	NM_03254	84585	Rnf123	BC003945|
chr3	95622402	95622625	exon (NM_	exon (NM_	363	NM_00108	75137	Rprd2	2810036A1
chrX	1.31E+08	1.31E+08	intron (NM	B3|SINE|B	-45281	NM_01007	13497	Drp2	AW495265
chr7	1.48E+08	1.48E+08	Intergenic	Intergenic	12717	NM_02537	66141	Ifitm3	1110004C0
chr4	53133102	53133325	intron (NM	intron (NM	39554	NM_01345	11303	Abca1	ABC-1|Abc
chr5	1.11E+08	1.11E+08	intron (NM	intron (NM	25548	NM_02933	75560	Ep400	1700020J0
chr17	56092002	56092175	promoter-T	promoter-T	42	NM_01576	50498	Ebi3	EBI-3|IL-27
chr16	48709952	48710125	Intergenic	Intergenic	62031	NM_19829	77647	Trat1	C030046M
chr8	97541477	97542325	intron (NM	intron (NM	309	NM_17303	54672	Gpr97	A030001G2
chr12	73805052	73805375	Intergenic	Intergenic	-39398	NM_02552	66375	Dhrs7	2310016E2
chr1	53831002	53831100	intron (NM	intron (NM	11008	NM_13381	98267	Stk17b	3110009A0
chr2	72887152	72887650	Intergenic	Intergenic	-68898	NM_00101	20687	Sp3	D130027J0
chr4	49251027	49251250	intron (NM	intron (NM	169885	NM_14536	209186	Acnat2	C730036D1
chr11	1.18E+08	1.18E+08	Intergenic	(CACCAT)n	-30273	NM_02791	71776	Tha1	1300017K0
chr11	77845377	77846025	promoter-T	promoter-T	-114	NM_18328	70451	Dhrs13	2610209N1
chr4	11413077	11413300	promoter-T	promoter-T	83	NM_00108	66185	1110037F0	4930422M
chr12	1.14E+08	1.14E+08	intron (NM	CpG	374	NM_00116	11651	Akt1	Akt|PKB|P
chr8	54690502	54690625	intron (NM	intron (NM	33856	NM_14620	234258	Neil3	AI449477|
chr16	30595777	30595875	Intergenic	Intergenic	-3983	NM_17763	224093	Fam43a	Tuf1
chr12	59826977	59827400	Intergenic	Lx8|LINE|L	285816	NM_00914	20334	Sec23a	Msec23|Se
chr11	61562802	61562950	intron (NM	intron (NM	12714	NM_14480	212627	Prpsap2	A230054F2
chr5	44190802	44191100	Intergenic	Intergenic	-17563	NM_17872	242960	Fbxl5	Fbl4|Fir4
chr16	38616752	38616850	intron (NM	intron (NM	53874	NM_02640	67846	Tmem39a	2610033C0
chr10	87694227	87694425	intron (NM	intron (NM	30484	NM_02748	67282	Ccdc53	2900091E1

chr4	80198802	80198875	Intergenic	Intergenic	-281279	NM_03120	22178	Tyrp1	Oca3|TRP-
chr19	23210552	23210975	Intergenic	CpG	-4953	NM_01063	16601	Klf9	2310051E1
chr18	78035102	78035325	intron (NM	intron (NM	1924	NM_01383	19201	Pstpip2	MAYP|cmc
chr11	3068027	3068150	intron (NM	RLTR21|LT	25378	NM_03020	78887	Sfi1	-
chr10	84121252	84121400	intron (NM	intron (NM	36144	NM_02742	70428	Polr3b	2700078H0
chr1	89110277	89110775	promoter-T	promoter-T	12	NM_00103	26987	Eif4e2	2700069E0
chr1	37486927	37487250	promoter-T	promoter-T	71	NM_02612	67387	Unc50	1110002A2
chr5	1.29E+08	1.29E+08	intron (NM	intron (NM	16871	NM_00128	13852	Stx2	AW538950
chr1	72372652	72373050	exon (NM_	exon (NM_	18856	NM_00953	22596	Xrcc5	AI314015|
chr1	1.35E+08	1.35E+08	intron (NM	intron (NM	22377	NM_17544	214253	Etnk2	4933417N2
chr12	3427202	3427325	promoter-T	promoter-T	406	NM_17242	75302	Asxl2	4930556B1
chr4	9037027	9037125	Intergenic	MER53|DN	-159388	NM_02894	74438	Clvs1	4933402J2
chr9	95412102	95412350	intron (NM	CpG	189	NM_02647	67958	U2surp	2610101N1
chr7	53098427	53098550	intron (NM	RMER15-in	-12329	NM_15341	101612	Grwd1	A301|AI50
chr5	1.39E+08	1.39E+08	promoter-T	promoter-T	-297	NM_03056	80752	Fam20c	C76981|DN
chr18	60933727	60935575	intron (NM	CpG	401	NM_02060	20044	Rps14	2600014J0
chr1	1.01E+08	1.01E+08	Intergenic	Intergenic	303525	NM_02758	70866	Slco6d1	4921511I0
chr9	89937702	89938050	Intergenic	MLT1E2|LT	-11229	NM_00780	13036	Ctsh	AL022844
chr1	1.35E+08	1.35E+08	Intergenic	Intergenic	-1855	NM_13381	108954	Ppp1r15b	1810033K1
chr8	1.1E+08	1.1E+08	promoter-T	promoter-T	90	NM_02555	66427	Cyb5b	1810044O2
chr1	10029952	10030100	intron (NM	CpG	1727	NM_02649	211660	Cspp1	2310020J1
chr10	1.17E+08	1.17E+08	promoter-T	promoter-T	-358	NM_17779	327826	Frs2	4732458E1
chr10	90645227	90645525	Intergenic	Intergenic	-11012	NM_00108	21917	Tmpo	5630400D2
chr4	43058227	43058325	intron (NR_	intron (NR_	816	NR_04556	230088	Fam214b	B230312A2
chr4	11893552	11893750	promoter-T	promoter-T	-54	NM_00103	381511	Pdp1	Gm1024|P
chrX	1.52E+08	1.52E+08	Intergenic	L1_Mus3|L	-356818	NM_00109	211612	Ptchd1	9630036J2
chr1	1.22E+08	1.22E+08	intron (NM	MER58B|D	-18099	NM_14550	226352	Epb4.1l5	1700030C1
chr16	4790002	4790075	promoter-T	promoter-T	-103	NM_02567	66626	Cdip1	2700048O1
chr7	87976352	87976575	Intergenic	RLTR19-int	-28246	NM_01672	29875	Iqgap1	AA682088|
chr15	64553327	64553475	intron (NM	intron (NM	200457	NM_00129	11514	Adcy8	AC8|AW06
chr17	47314577	47314675	intron (NM	intron (NM	36735	NM_00109	224829	Trerf1	9430096I1
chr7	1.09E+08	1.09E+08	promoter-T	promoter-T	-166	NR_11193	233575	Pgap2	1810006G2
chr6	72222377	72222500	Intergenic	Intergenic	-32166	NM_00128	20388	Sftpb	AI562151|
chr2	28554252	28554400	intron (NM	MIR|SINE|	845	NM_02728	69987	1700026L0	MAST
chr7	1.18E+08	1.18E+08	intron (NM	intron (NM	1118	NM_00104	13690	Eif4g2	AA589388|
chr7	1.25E+08	1.25E+08	Intergenic	Lx8|LINE|L	316920	NM_17564	233781	Xylt1	8030490L1
chr11	1.06E+08	1.06E+08	intron (NM	intron (NM	25043	NM_17239	67803	Limd2	0610025L0
chr10	84048202	84048325	intron (NM	intron (NM	8571	NM_14600	216198	Tcp11l2	E430026E1
chr5	1.4E+08	1.4E+08	intron (NM	intron (NM	-1215	NM_17700	319772	C130050O1	-
chr4	1.15E+08	1.15E+08	intron (NM	RSINE1|SIN	807	NM_02564	66588	Cmpk1	0610011D0
chr7	1.24E+08	1.24E+08	intron (NM	Kanga1|DN	11230	NM_00113	53322	Nucb2	AI607786|
chr4	91031177	91031475	intron (NM	intron (NM	7420	NM_01048	15569	Elavl2	Hub|mel-N
chr2	84224127	84224225	intron (NM	Lx8|LINE|L	41247	NM_01878	54598	Calcrl	AV071593|
chr1	1.74E+08	1.74E+08	Intergenic	Intergenic	-11597	NM_00853	17085	Ly9	AI893573|
chr7	53229552	53229650	Intergenic	L2|LINE|L2	45833	NM_00103	211535	Ccdc114	-
chr3	1.27E+08	1.27E+08	intron (NM	intron (NM	11157	NM_02780	71481	Alpk1	8430410J1
chr1	52687777	52687850	intron (NR_	intron (NR_	264	NM_00114	227094	Tmem194b	5330401P0

chr10	76978727	76978925	promoter-T	promoter-T	-268	NM_13399	108707	Fam207a	1810008A1
chr7	73279102	73279550	intron (NM	intron (NM	24925	NM_00108	269941	Chsy1	mKIAA099(
chr5	1.41E+08	1.41E+08	Intergenic	Intergenic	-4662	NM_02152	59031	Chst12	AI595374|(
chr1	89753327	89753400	intron (NM	ID_B1|SINE	3501	NM_17764	227333	Dgkd	AI841987|I
chr16	13672052	13672450	intron (NM	CpG	138	NM_02565	66598	3110001I2:	-
chr6	82691502	82691650	intron (NM	intron (NM	32872	NM_01382	15277	Hk2	AI642394|I
chr9	7593502	7593575	Intergenic	Intergenic	22080	NM_00103	234911	Mmp27	Gm180
chr12	74880327	74880575	Intergenic	Intergenic	-128403	NM_01043	15251	Hif1a	AA959795|
chr5	43508227	43508475	Intergenic	Intergenic	-116351	NM_17593	231207	Cpeb2	A630055H1
chr9	1.07E+08	1.07E+08	intron (NM	intron (NM	-17057	NM_02910	74840	Manf	3230402M
chr2	89474452	89474525	Intergenic	Intergenic	-3435	NM_00101	257984	Olfr1249	MOR231-1
chr12	1.05E+08	1.05E+08	intron (NM	intron (NM	3822	NM_00924	20702	Serpina1c	Pi3|Pi6|Ska
chr7	1.35E+08	1.35E+08	intron (NM	intron (NM	4256	NM_02133	16411	Itgax	AI449405|(
chr6	1.35E+08	1.35E+08	Intergenic	MTD|LTR|I	-1143	NM_01354	15199	Hebp1	Hebp
chr2	73322927	73323075	intron (NM	intron (NM	809	NM_00128	215280	Wipf1	AI115543|I
chr8	1.24E+08	1.24E+08	promoter-T	promoter-T	-144	NM_14560	234825	Klhdc4	AA408426|
chr14	22818927	22819200	Intergenic	MLT1J|LTR	-10240	NM_14545	218820	Zfp503	AI181838|I
chr13	16335002	16335525	Intergenic	Intergenic	228955	NM_00838	16323	Inhba	-
chr6	1.13E+08	1.13E+08	5' UTR (NM	5' UTR (NM	120	NM_00128	78783	Brpf1	4833438B1
chr10	1.12E+08	1.12E+08	promoter-T	promoter-T	-231	NM_00103	382423	Atxn7l3b	4921506J0
chr1	1.73E+08	1.73E+08	exon (NM_	exon (NM_	4029	NM_02057	57370	B4galt3	9530061M
chr16	24363652	24363725	Intergenic	L3|LINE|CF	-29748	NM_17866	210126	Lpp	9430020K1
chr12	32745777	32745950	exon (NM_	exon (NM_	281	NM_01115	19088	Prkar2b	AI451071|A
chr1	1.73E+08	1.73E+08	5' UTR (NM	5' UTR (NM	337	NM_01161	21945	Dedd	CASP8IP1|I
chr1	1.73E+08	1.73E+08	Intergenic	CpG	19219	NM_14455	246256	Fcgr4	4833442P2
chr15	63479727	63479875	Intergenic	Intergenic	160493	NM_03137	83492	Gsdmc	Gsdmc1|M
chr1	1.27E+08	1.27E+08	promoter-T	promoter-T	-730	NM_02878	74150	Slc35f5	1300003P1
chr4	1.55E+08	1.55E+08	promoter-T	promoter-T	467	NM_20722	140500	Acap3	Centb5|Kia
chr1	36565752	36566100	TTS (NM_0	TTS (NM_0	-2795	NM_00103	94218	Cnnm3	Acdp3|Clp:
chr8	1.23E+08	1.23E+08	Intergenic	URR1B|DN	-15163	NM_17885	272551	Gins2	2210013I1:
chr1	82793727	82793875	Intergenic	MLT1A1|LT	-28770	NM_02545	66261	Tm4sf20	1810018L0
chr2	26459227	26459625	intron (NM	CpG	511	NM_02621	67512	Agpat2	2510002J0
chr11	12758152	12758500	Intergenic	Intergenic	-393363	NM_00128	12808	Cobl	-
chr5	1.01E+08	1.01E+08	promoter-T	promoter-T	91	NM_17240	70681	Fam175a	3830405G(
chr4	6284477	6284650	Intergenic	Intergenic	-8264	NM_01680	53378	Sdcbp	MDA-9|Syc
chr6	86354427	86354625	intron (NM	CpG	313	NM_00116	21841	Tia1	2310050N(
chr6	38249102	38249175	exon (NM_	exon (NM_	121	NM_17246	209032	Zc3hav1l	B130055L0
chr7	54311552	54311900	Intergenic	Intergenic	5716	NM_02643	67893	Tmem86a	1810054O1
chr17	24327927	24328150	intron (NM	PB1D10|SI	8864	NM_00116	224617	Tbc1d24	9630033P1
chr5	1.34E+08	1.34E+08	Intergenic	Intergenic	-569825	NM_17704	319974	Auts2	2700063G(
chr12	13605327	13605800	Intergenic	Intergenic	329630	NM_02770	71169	Nbas	4933425L0
chr2	26788152	26788275	intron (NM	intron (NM	818	NM_01151	20932	Surf4	AL033340|
chr10	29081577	29081675	intron (NM	CpG	622	NM_02651	68031	Rnf146	2610509H2
chr5	28118427	28118550	promoter-T	promoter-T	-398	NM_01887	55982	Paxip1	D5Ertd149
chr8	27126052	27126400	promoter-T	promoter-T	-153	NM_00803	14272	Fnta	FTA
chr5	1.35E+08	1.35E+08	intron (NM	intron (NM	208	NM_02447	79565	Wbscr27	AW492986
chr4	1.16E+08	1.16E+08	promoter-T	promoter-T	-43	NM_00112	19366	Rad54l	RAD54

chr17	35394827	35395000	Intergenic	RMER15|L1	-5126	NM_01038	14964	H2-D1	H2-D|H2-D	
chr7	86611452	86611775	promoter-T	promoter-T	-454	NM_01746	18975	Polg	AA409516|	
chr14	21522002	21522350	promoter-T	promoter-T	-73	NM_02536	66121	Chchd1	1110001O1	
chr4	1.34E+08	1.34E+08	intron (NM	intron (NM	49754	NM_20723	230815	Man1c1	AI593348	
chr11	97141427	97141650	exon (NM_	exon (NM_	352	NM_00894	19155	Npepps	AAP-S|MP1	
chr1	58851877	58852025	promoter-T	promoter-T	-267	NM_00981	12370	Casp8	CASP-8|FLI	
chr2	72875952	72876050	Intergenic	Intergenic	-57498	NM_00101	20687	Sp3	D130027J0	
chrX	69061502	69061575	promoter-T	promoter-T	119	NM_00129	67048	Vma21	2610030H0	
chr2	25121602	25121750	intron (NM	intron (NM	3558	NM_17528	97031	Tprn	C430004E1	
chr15	76173752	76173925	promoter-T	promoter-T	-115	NM_02556	66445	Cyc1	2610002H1	
chr15	9968827	9968925	Intergenic	Intergenic	-138117	NM_01116	19116	Prlr	AI987712|	
chr10	59740277	59740400	promoter-T	promoter-T	-38	NM_00114	19156	Psap	AI037048|	
chr10	85334102	85334450	promoter-T	promoter-T	-300	NM_13399	103136	Pwp1	2310058A1	
chr9	21042977	21043150	5' UTR (NM	5' UTR (NM	713	NM_01667	50868	Keap1	INRF2|mKI	
chr7	64593552	64593850	intron (NM	L1M4|LINE	48540	NM_00807	14407	Gabrg3	B230362M	
chr11	29030452	29030675	promoter-T	promoter-T	-188	NM_02786	71701	Pnpt1	1200003F1	
chrX	1.31E+08	1.31E+08	Intergenic	ID_B1|SINE	-1030	NM_01986	19982	Rpl36a	L44L|Rpl44	
chr5	93634227	93634600	intron (NM	intron (NM	1108	NM_01736	12453	Ccni	-	
chr18	6490852	6491225	promoter-T	promoter-T	-183	NM_02749	13831	Epc1	2400007E1	
chr11	1.18E+08	1.18E+08	intron (NM	intron (NM	52145	NM_00116	69926	Dnah17	2810003K2	
chr9	1.19E+08	1.19E+08	intron (NM	intron (NM	20305	NM_17711	320256	Dlec1	D630005C0	
chr3	93248252	93248575	exon (NM_	exon (NM_	2161	NM_00116	99681	Tchh	AHF|AI597	
chr13	59776952	59777125	intron (NM	G-rich|Low	107	NM_00103	105348	Golm1	2310001L0	
chr16	70326877	70327000	intron (NM	intron (NM	12744	NM_02880	74185	Gbe1	2310045H1	
chr14	26292177	26292400	intron (NM	MIRb|SINE	13617	NM_18320	328365	Zmiz1	BC065120|	
chr14	27399152	27399325	exon (NM_	exon (NM_	202	NM_14596	211922	Dennd6a	Fam116a	
chr10	1.28E+08	1.28E+08	promoter-T	promoter-T	103	NM_00125	78428	Wibg	A030010B0	
chr5	92799502	92799625	intron (NM	B3|SINE|B	-7241	NR_038116	56066	Cxcl11	Cxc11|H17	
chr12	52792327	52792575	exon (NM_	exon (NM_	450	NM_00117	94186	Strn3	Gs2na|SG2	
chr4	1.54E+08	1.54E+08	Intergenic	L1MB7|LIN	-9865	NM_17893	230979	Tnfrsf14	Atar|HveA	
chr10	1.28E+08	1.28E+08	promoter-T	promoter-T	50	NM_01677	11947	Atp5b	-	
chr8	13158777	13158950	promoter-T	promoter-T	-272	NM_01068	16783	Lamp1	AI196048|	
chr11	60231052	60231200	promoter-T	promoter-T	479	NM_02575	66771	Gid4	4933439F1	
chr8	33051427	33051550	Intergenic	Intergenic	-22813	NM_17859	211323	Nrg1	6030402G2	
chr2	1.73E+08	1.73E+08	Intergenic	Intergenic	24898	NM_01954	56190	Rbm38	Rnpc1|Seb	
chr11	97589852	97590075	intron (NM	intron (NM	16055	NM_05405	108083	Pip4k2b	AI848124|	
chr6	47545102	47545350	promoter-T	promoter-T	-197	NM_00114	14056	Ezh2	Enx-1|Enx1	
chr5	1.51E+08	1.51E+08	Intergenic	Intergenic	-83429	NM_17288	320365	Fry	13CDNA73	
chr1	1.87E+08	1.87E+08	intron (NM	intron (NM	3653	NM_00129	66112	1-Mar	1300013F1	
chr7	1.38E+08	1.38E+08	intron (NM	intron (NM	7234	NM_01956	56213	Htra1	AI429470|	
chr3	1.36E+08	1.36E+08	intron (NM	intron (NM	22121	NM_00129	19055	Ppp3ca	2900074D1	
chr1	1.8E+08	1.8E+08	Intergenic	ORR1E|LTR	-37230	NM_00116	269152	Kif26b	4832420M	
chr9	31097877	31098175	Intergenic	Intergenic	19040	NM_00110	638580	Gm7244	EG638580	
chr12	87165677	87165775	promoter-T	promoter-T	-174	NM_00108	320244	Ttll5	1700048H1	
chr7	54758527	54758650	Intergenic	Intergenic	-13636	NM_15310	235712	Mrgpra2b	MrgA2|Mr	
chr2	3200927	3201025	promoter-T	promoter-T	-508	NM_00870	18108	Nmt2	A930001K0	
chr2	1.18E+08	1.18E+08	promoter-T	promoter-T	-487	NM_13831	171543	Bmf	AW260063	

chr14	32137252	32137850	promoter-T	promoter-T	-469	NM_00103	110084	Dnah1	B230373P0
chr19	10969577	10969875	promoter-T	promoter-T	5	NM_00125	28000	Prpf19	AA617263|
chr18	11742252	11742375	Intergenic	Intergenic	-49472	NM_00125	225182	Rbbp8	9930104E2
chr8	47702327	47702425	promoter-T	promoter-T	178	NM_00100	408022	Primpol	BC065112|
chr10	80396527	80396825	Intergenic	Intergenic	3840	NM_00865	17873	Gadd45b	AI323528|
chr8	11497402	11497725	promoter-T	promoter-T	-5	NM_02699	69225	Carkd	0710008K0
chr2	1.1E+08	1.1E+08	intron (NM	CpG	116	NM_01169	22343	Lin7c	9130007B1
chr2	71591152	71591325	Intergenic	Intergenic	-33758	NM_00839	16403	Itga6	5033401O0
chr8	11636202	11636325	promoter-T	promoter-T	-506	NM_00128	102334	Ankrd10	4833425P1
chr2	11816527	11816650	Intergenic	Intergenic	117208	NM_17726	320816	Ankrd16	2810455F0
chr4	1.28E+08	1.28E+08	intron (NM	intron (NM	405	NM_00108	230761	Zfp362	-
chr10	41529027	41529100	intron (NM	intron (NM	406	NM_00124	103268	Cep57l1	2410017P0
chr2	1.26E+08	1.26E+08	intron (NM	intron (NM	17501	NM_00108	241633	Atp8b4	A530043E1
chr17	27340702	27340900	exon (NM_	exon (NM_	582	NM_14607	224640	Lemd2	BC026588|
chr6	71781552	71781625	intron (NM	CpG	274	NM_00125	76614	Immt	1700082C1
chr4	1.47E+08	1.47E+08	promoter-T	promoter-T	-253	NM_00130	11610	Agtrap	3300002E1
chr1	1.84E+08	1.84E+08	Intergenic	Intergenic	-23531	NM_13381	98386	Lbr	AI505894|
chr19	5660952	5661175	5' UTR (NM	5' UTR (NM	2644	NM_00116	20469	Sipa1	Spa1
chr16	87354102	87354625	promoter-T	promoter-T	-67	NM_00115	67768	N6amt1	5830445C0
chr1	93049977	93050075	exon (NM_	exon (NM_	8346	NM_00103	329207	Rbm44	Gm817
chr11	1.19E+08	1.19E+08	promoter-T	promoter-T	115	NM_00116	338371	Endov	A730011L0
chrX	12455252	12455375	Intergenic	Intergenic	-58686	NM_00127	245350	AA414768	OTTMUSG0
chr9	1.03E+08	1.03E+08	promoter-T	promoter-T	425	NM_18139	69010	Anapc13	1810004D0
chr2	1.2E+08	1.2E+08	Intergenic	CpG	-24213	NM_00116	29808	Mga	AV312082|
chr6	4551452	4551750	intron (NM	intron (NM	535	NM_14539	213819	Casd1	Cas1|Cast1
chr10	68675627	68675700	promoter-T	promoter-T	258	NM_00108	69288	Rhobtb1	1700008H1
chr11	97883677	97883825	5' UTR (NM	5' UTR (NM	190	NM_00128	12295	Cacnb1	CAB1|Cchb
chr15	57812327	57812750	Intergenic	Intergenic	-4920	NM_17386	239463	Fam83a	-
chr18	36177602	36177725	exon (NM_	exon (NM_	53179	NR_11034	74002	Psd2	6330404E2
chr13	99673002	99673150	intron (NM	intron (NM	-12079	NM_00104	238799	Tnpo1	AU021749
chr8	37330352	37330675	Intergenic	MTEb|LTR	-17943	NM_00108	244421	Lonrf1	-
chr12	86452152	86452250	intron (NM	CpG	418	NM_03022	78920	Dlst	1600017E0
chr19	28086177	28086600	promoter-T	promoter-T	-732	NM_01126	19726	Rfx3	C230093O1
chr4	1.43E+08	1.43E+08	intron (NM	CpG	824	NM_00108	110593	Prdm2	4833427P1
chr13	1.13E+08	1.13E+08	Intergenic	ORR1E|LTF	-226458	NM_00116	77318	Ankrd55	C030011J0
chr2	1.48E+08	1.48E+08	Intergenic	Intergenic	-2812	NM_00921	20608	Sstr4	Smstr4|sst
chr1	84836652	84836750	promoter-T	promoter-T	285	NM_02538	66153	Fbxo36	0610008D1
chr1	1.8E+08	1.8E+08	intron (NM	intron (NM	5214	NM_01680	51810	Hnrnpu	AA408410|
chr7	1.4E+08	1.4E+08	non-coding	non-coding	537	NM_02993	77590	Chst15	4631426J0
chr13	81921752	81922175	promoter-T	promoter-T	-328	NM_00768	12626	Cetn3	MmCEN3
chr10	1.17E+08	1.17E+08	Intergenic	Intergenic	-60522	NM_00101	432508	Cpsf6	4733401N1
chr2	1.8E+08	1.8E+08	promoter-T	promoter-T	-106	NM_17555	23856	Dido1	6720461J1
chr19	36296702	36296825	Intergenic	Intergenic	-102429	NM_01346	107765	Ankrd1	Alrp|CARP
chr17	29521852	29522050	Intergenic	Intergenic	24092	NM_00115	26382	Fgd2	Tcd-2|Tcd2
chr13	1.09E+08	1.09E+08	intron (NM	intron (NM	20919	NM_02804	71991	Ercc8	2410022P0
chr5	1.44E+08	1.44E+08	intron (NM	CpG	340	NM_00739	11461	Actb	Actx|E430
chr5	32155177	32155475	intron (NM	intron (NM	154903	NM_18128	107976	Bre	6030405P1

chr7	71081227	71081425	intron (NM	intron (NM	2475	NM_02136	50794	Klf13	0610043C1
chr3	1.57E+08	1.57E+08	intron (NM	intron (NM	-261785	NM_01738	53861	Zranb2	AI227013|2
chr3	1.04E+08	1.04E+08	5' UTR (NM	5' UTR (NM	235	NM_00130	15257	Hipk1	1110062K0
chr1	34658927	34659325	Intergenic	CT-rich|Lov	22588	NM_21372	211383	Amer3	9430069J0
chr14	70553402	70553475	TTS (NM_0	TTS (NM_0	160	NM_14605	219158	Ccar2	2610301G1
chr10	80598077	80598175	promoter-T	promoter-T	-890	NM_01073	16969	Zbtb7a	9030619K0
chr8	41596577	41597275	promoter-T	promoter-T	182	NM_00127	18983	Cnot7	AU022737
chr6	82863427	82863575	exon (NM_	exon (NM_	26243	NM_01135	20355	Sema4f	-
chr7	26687202	26687425	intron (NM	intron (NM	4636	NM_00999	13088	Cyp2b10	Cyp2b|Cyp
chr7	75028027	75028100	Intergenic	Intergenic	-69080	NM_01051	16001	Igf1r	A330103N2
chr18	77820852	77821225	Intergenic	Intergenic	-17163	NM_00116	225743	Rnf165	2900024M
chr6	1.23E+08	1.23E+08	Intergenic	CpG	-8914	NM_02189	60611	Foxj2	Fhx
chr3	10273727	10273925	intron (NM	Lx8|LINE|L	27357	NM_02931	75497	Fabp12	1700008G0
chr18	67459302	67459600	intron (NM	intron (NM	10574	NM_05326	114663	Impa2	2210415D2
chr16	37868052	37868375	promoter-T	promoter-T	-273	NM_17709	320184	Lrrc58	1810012N1
chr1	1.56E+08	1.56E+08	promoter-T	promoter-T	-593	NM_01188	24014	Rnasel	E230029I04
chr14	51711052	51711300	intron (NM	intron (NM	424	NM_02147	58809	Rnase4	C730049F2
chr3	96439452	96439725	promoter-T	promoter-T	-182	NM_00116	18632	Pex11b	PEX11beta
chr2	1.19E+08	1.19E+08	promoter-T	promoter-T	-390	NM_02692	69065	Chac1	1810008K0
chr18	7868877	7869000	promoter-T	promoter-T	108	NM_00128	225131	Wac	1110067P0
chr16	90283127	90283275	intron (NM	intron (NM	1469	NM_17892	224432	Scaf4	AA517739|
chr1	1.36E+08	1.36E+08	intron (NM	intron (NM	13045	NM_03118	17928	Myog	MYF4|bHLI
chr5	1.11E+08	1.11E+08	exon (NM_	exon (NM_	115	NM_14514	107999	Gtpbp6	AV119224|
chr15	66301177	66301350	intron (NM	intron (NM	31209	NM_01945	54562	Lrrc6	LRTP
chr8	1.24E+08	1.24E+08	intron (NM	intron (NM	1280	NM_00103	407789	BC048644	-
chr9	54433902	54433975	promoter-T	promoter-T	-380	NM_02957	67834	Idh3a	1110003P1
chr1	58026327	58027100	promoter-T	promoter-T	215	NM_00115	51960	Kctd18	4932411A2
chr2	1.44E+08	1.44E+08	intron (NM	MER5B|DN	1180	NM_18141	228714	Csrp2bp	2510008M
chr4	63215027	63215175	Intergenic	Intergenic	-6293	NM_14490	230279	6330416G1	AW492431
chrX	13077502	13077575	Intergenic	L1MD|LINE	32740	NM_17341	236690	Nyx	CLNP|CSNE
chr11	1.09E+08	1.09E+08	5' UTR (NM	5' UTR (NM	-11916	NM_00102	104681	Slc16a6	AW743111
chr11	90120502	90120600	intron (NM	intron (NM	9761	NM_02617	67468	Mmd	1200017E0
chr7	29623427	29623675	intron (NM	intron (NM	4350	NM_01070	16857	Lgals6	galectin-6
chr6	86616577	86616675	intron (NM	intron (NM	2527	NM_01075	17119	Mxd1	AW122478
chr2	84510702	84510950	promoter-T	promoter-T	39	NM_00103	72657	2700094K1	Selh
chr10	60938602	60938750	promoter-T	promoter-T	95	NM_15354	216011	Lrrc20	BC036304
chr8	97543927	97544050	intron (NM	exon (NM_	2396	NM_17303	54672	Gpr97	A030001G2
chr5	31797327	31797500	intron (NM	intron (NM	279	NM_13375	74254	Gpn1	2410004J0
chr14	51628002	51628325	intron (NM	intron (NM	559	NM_02914	75019	Rnase10	4930474F2
chr9	1.08E+08	1.08E+08	promoter-T	promoter-T	-355	NM_00111	69232	Qrich1	2610028H0
chr4	59452902	59453200	Intergenic	Intergenic	-1546	NM_00116	634731	Susd1	A530080P1
chr13	53080702	53081475	Intergenic	Intergenic	-4680	NM_01737	18030	Nfil3	AV225605|
chr8	13869677	13869875	5' UTR (NM	5' UTR (NM	135	NM_18185	101994	Champ1	AA675043|
chr8	1.13E+08	1.13E+08	Intergenic	PB1D7|SIN	-7552	NM_00917	20444	St3gal2	AI429591|A
chr5	1.49E+08	1.49E+08	Intergenic	Intergenic	-20916	NM_02562	66537	Pomp	2510048O0
chr2	71712027	71712150	intron (NM	intron (NM	807	NM_17266	228026	Pdk1	B830012B0
chr17	46783202	46783300	promoter-T	promoter-T	54	NM_02530	27398	Mrpl2	CGI-22|MF

chr13	37222602	37222725	Intergenic	Intergenic	-80550	NM_00116	74145	F13a1	1200014I0
chr9	1.24E+08	1.24E+08	3' UTR (NM	3' UTR (NM	4869	NM_00991	12774	Ccr5	AM4-7|CD
chr19	56896302	56896450	Intergenic	CpG	-4323	NM_00100	83561	Tdrd1	MTR-1
chr5	76126802	76127375	Intergenic	Intergenic	156072	NM_02109	16590	Kit	Bs|CD117|
chr19	8787802	8787950	exon (NM_	exon (NM_	511	NM_00857	17254	Slc3a2	4F2|4F2HC
chr6	42303377	42303475	intron (NM	intron (NM	3599	NM_00128	22793	Zyx	9530098H0
chr8	1.23E+08	1.23E+08	intron (NM	intron (NM	48731	NM_03020	78892	Crispld2	1810049K2
chr4	1.47E+08	1.47E+08	Intergenic	Intergenic	-1703	NM_01112	18822	Plod1	2410042F0
chr3	95032077	95032200	promoter-T	promoter-T	-208	NM_17239	57912	Cdc42se1	1300002M
chr19	44210227	44210450	promoter-T	promoter-T	28	NM_00108	72502	Cwf19l1	2610528C0
chr10	92884702	92884950	Intergenic	L1MA8|LIN	-31315	NM_00851	16993	Lta4h	-
chr12	1.07E+08	1.07E+08	intron (NM	intron (NM	20428	NM_00102	22367	Vrk1	51PK
chr12	8666502	8666750	Intergenic	L2b|LINE|L	-14314	NM_00116	80913	Pum2	5730503J2
chr2	83484377	83484575	promoter-T	promoter-T	-259	NM_02693	69082	Zc3h15	1700006A1
chr13	1.13E+08	1.13E+08	Intergenic	Intergenic	-119871	NM_00116	77318	Ankrd55	C030011J0
chr6	93720902	93721050	intron (NM	intron (NM	141981	NR_10459	14924	Magi1	AIP3|BAP1
chr17	56754652	56754775	exon (NM_	exon (NM_	1895	NM_01873	54217	Rpl36	-
chr18	39153002	39153175	5' UTR (NM	5' UTR (NM	289	NM_17516	71302	Arhgap26	1810044B2
chr1	40689602	40689675	Intergenic	Intergenic	-48919	NM_00103	226999	Slc9a2	2210416H1
chr4	1.25E+08	1.25E+08	intron (NM	intron (NM	5437	NM_15315	230738	Zc3h12a	BC036563|
chr8	37158052	37158200	intron (NM	CpG	244	NM_17291	244418	D8Ertd82e	9830148H2
chr11	1.1E+08	1.1E+08	Intergenic	Lx9|LINE|L	-36493	NM_15378	208659	Fam20a	AI606893
chr10	57206552	57206775	promoter-T	promoter-T	472	NM_00829	15500	Hsf2	AI661205
chr6	88674377	88674525	promoter-T	promoter-T	45	NM_01184	23945	Mgll	AA589436|
chr17	34168752	34169475	promoter-T	promoter-T	-182	NM_00120	20182	Rxrb	AL023085|
chr3	36240252	36240500	Intergenic	MTE-int|LT	-119179	NM_19819	229214	Qrfpr	AQ27|Gpr1
chr2	1.26E+08	1.26E+08	intron (NM	intron (NM	16851	NM_00108	241633	Atp8b4	A530043E1
chr10	1.08E+08	1.08E+08	Intergenic	Intergenic	-22330	NM_02789	17931	Ppp1r12a	1200015F0
chr6	70737652	70737825	intron (NM	intron (NM	4431	NM_00907	19895	Rpia	RPI
chr3	1.52E+08	1.52E+08	Intergenic	Intergenic	135188	NM_01686	54120	Gipc2	2200002N0
chr2	78889077	78889175	Intergenic	Intergenic	179922	NM_00945	22193	Ube2e3	Ubce4|ubc
chr4	45421827	45422025	promoter-T	promoter-T	-288	NM_00100	230125	Slc25a51	9130208E0
chr2	1.55E+08	1.55E+08	intron (NM	intron (NM	28051	NM_02936	75608	Chmp4b	2010012F0
chr12	78323727	78323800	Intergenic	Intergenic	-15328	NM_00125	53618	Fut8	-
chr1	1.36E+08	1.36E+08	5' UTR (NM	5' UTR (NM	108	NM_15289	75605	Kdm5b	2010009J1
chr9	40595452	40595700	Intergenic	(GGAGAA)n	-13780	NM_03116	15481	Hspa8	2410008N1
chr11	1.21E+08	1.21E+08	promoter-T	promoter-T	-613	NM_00108	68837	Foxk2	1110054H0
chr9	1.2E+08	1.2E+08	Intergenic	Intergenic	-29690	NM_14455	245049	Myrip	A230081N1
chr14	65962877	65963025	Intergenic	CpG	-14562	NM_19902	380912	Zfp395	BC053701|
chr1	1.68E+08	1.68E+08	promoter-T	promoter-T	-176	NM_00103	226594	Rcsd1	A430105K1
chr8	67191352	67191475	Intergenic	Intergenic	-19576	NM_01349	12876	Cpe	CPH|Cph-1
chr1	1.83E+08	1.83E+08	Intergenic	Intergenic	-2231	NM_14594	208768	Sde2	-
chr17	3557152	3557375	promoter-T	promoter-T	450	NM_14607	224481	Tfb1m	AI429207
chr4	53999852	54000075	Intergenic	Intergenic	161046	NM_02805	52076	Tmem38b	1600017F2
chr13	55993677	55993825	Intergenic	Intergenic	-60965	NM_01109	18740	Pitx1	Bft|P-OTX|
chr12	72932452	72932550	intron (NM	CpG	446	NM_17246	208846	Daam1	-
chr4	1.28E+08	1.28E+08	intron (NM	intron (NM	-109799	NM_02703	69317	Hmgb4	1700001F2

chr2	1.81E+08	1.81E+08	TTS (NM_0	TTS (NM_0	627	NM_02676	68556	Uckl1	1110007H1
chr1	65274502	65274575	intron (NM	intron (NM	41279	NM_01108	18711	Pikfyve	5230400C1
chr4	54528877	54528950	Intergenic	Intergenic	-431904	NM_17286	242466	Zfp462	6030417H(
chr11	50099952	50100100	intron (NM	intron (NM	5812	NM_17533	103806	Maml1	AI644666|/
chr12	76697627	76697875	promoter-T	promoter-T	-564	NM_01202	26932	Ppp2r5e	4633401M
chr11	97172602	97172900	Intergenic	Intergenic	-4279	NM_02592	67036	Mrpl45	2600005P(
chr5	1.51E+08	1.51E+08	exon (NM_	exon (NM_	124	NM_13389	100637	N4bp2l1	2410024N1
chr5	1.22E+08	1.22E+08	intron (NM	intron (NM	1007	NM_02612	67397	Erp29	1200015M
chr11	1.15E+08	1.15E+08	promoter-T	promoter-T	56	NM_00104	67671	Rpl38	0610025G1
chr17	37407627	37407975	intron (NM	intron (NM	622	NM_01381	14991	H2-M3	H-2M3|Hm
chr12	1.13E+08	1.13E+08	intron (NM	intron (NM	1429	NM_00939	21928	Tnfaip2	B94|Exoc3
chr9	5298327	5298425	promoter-T	promoter-T	-141	NM_00980	12362	Casp1	ICE|Il1bc
chr8	67469327	67469525	Intergenic	Intergenic	-1658	NM_00116	73067	Tmem192	3110005G2
chr7	1.35E+08	1.35E+08	5' UTR (NM	5' UTR (NM	110	NM_14620	233893	Zfp764	8030466O1
chr10	1.28E+08	1.28E+08	intron (NM	intron (NM	-1432	NM_01086	17904	Myl6	ESMLC|LC1
chr8	34496702	34496800	promoter-T	promoter-T	-46	NM_15282	75029	Purg	4930486B1
chr10	1.21E+08	1.21E+08	exon (NM_	exon (NM_	180	NM_02936	75612	Gns	2610016K1
chr8	96697402	96697550	TTS (NM_0	TTS (NM_0	958	NM_00863	17750	Mt2	AA409533|
chr16	57121502	57122250	promoter-T	promoter-T	49	NM_13859	28185	Tomm70a	2610044B2
chr9	43917927	43918150	TTS (NM_1	TTS (NM_1	2646	NM_00119	235312	C1qtnf5	Ctrp5
chr5	69983502	69983650	promoter-T	promoter-T	-52	NM_00103	67980	Gnpda2	4921523I1
chr5	30771377	30771500	intron (NM	(ATG)n|Sin	2988	NM_02928	75434	1700001C0	1700047H1
chr1	1.85E+08	1.85E+08	Intergenic	Intergenic	-9093	NM_01692	53791	Tlr5	-
chr1	65154302	65154575	3' UTR (NM	3' UTR (NM	-4501	NM_00777	12964	Cryga	Cryg-4|DG
chr3	95799302	95799425	intron (NM	intron (NM	399	NM_02332	67220	Plekho1	2810052M
chr15	76026302	76026600	promoter-T	promoter-T	-311	NM_20139	18810	Plec	AA591047|
chr5	65884527	65884825	promoter-T	promoter-T	-602	NM_13369	68552	Smim14	1110003E0
chr7	1.09E+08	1.09E+08	promoter-T	promoter-T	-392	NM_00116	69358	Lrrc51	1700008D(
chr1	94803627	94803850	exon (NM_	exon (NM_	172	NM_17511	67446	Dusp28	0710001B2
chr11	74773552	74773675	intron (NM	intron (NM	34239	NM_00100	103677	Smg6	AI317223|/
chr12	70432202	70432425	intron (NM	B3|SINE|B	25850	NM_02544	66244	Nemf	1500011I1
chr9	1.08E+08	1.08E+08	Intergenic	Intergenic	3702	NM_00816	14775	Gpx1	AI195024|/
chr1	1.76E+08	1.76E+08	Intergenic	Lx8|LINE|L	-42313	NM_00832	15951	Ifi204	Ifi16|p204
chr1	1.83E+08	1.83E+08	promoter-T	promoter-T	25	NM_02617	67459	Nvl	1200009I2
chr1	46471727	46472000	Intergenic	RLTR45|LT	348280	NM_00116	227058	Dnah7b	Dnahc7b|G
chr1	51704952	51705025	Intergenic	Intergenic	-169745	NM_02869	109019	Nabp1	4930434H(
chr18	38551277	38551525	Intergenic	MIRb|SINE	-27228	NM_02299	65113	Ndfip1	0610010M
chr5	32440802	32441225	intron (NM	intron (NM	2168	NM_00803	14284	Fosl2	Fra-2
chr2	77783927	77784200	intron (NM	CpG	350	NM_00129	80744	Cwc22	AA684037|
chr15	58715452	58716000	Intergenic	RCHARR1|	-5058	NM_17522	75841	Rnf139	4930555P1
chr10	62801777	62802100	promoter-T	promoter-T	-155	NM_00115	93759	Sirt1	AA673258|
chr6	1.29E+08	1.29E+08	Intergenic	CpG	-16793	NM_03059	80782	Klrb1b	Klrb1d|Ly5
chr2	1.27E+08	1.27E+08	Intergenic	MTD|LTR|	-2096	NR_11098	56461	Kcnip3	4933407H1
chr7	1.21E+08	1.21E+08	Intergenic	Intergenic	-55076	NM_02062	19226	Pth	Pthp
chr2	1.22E+08	1.22E+08	exon (NM_	exon (NM_	615	NM_00103	214616	Spata5l1	AV141009|
chr13	22153352	22153425	Intergenic	Intergenic	18289	NM_17819	319183	Hist1h2bj	-
chr16	10927152	10927425	Intergenic	Intergenic	65926	NM_01998	56722	Litaf	3222402J1

chr5	1.22E+08	1.22E+08	Intergenic	B2_Mm2|S	-21317	NM_00912	20239	Atxn2	9630045M
chr5	31495752	31495950	promoter-T	promoter-T	174	NM_15368	266781	Snx17	5830447M
chr3	90482677	90482850	Intergenic	Intergenic	9770	NM_01365	20201	S100a8	60B8Ag|AI
chr7	4745027	4745200	exon (NM_	exon (NM_	546	NM_00908	19943	Rpl28	D7Wsu21e
chr17	47903752	47904050	intron (NM	intron (NM	-18792	NM_01154	21425	Tfeb	Tcfeb|bHLI
chr5	36807877	36808075	intron (NM	CpG	142	NM_02447	17713	Grpel1	AA408748|
chr13	73741402	73741475	promoter-T	promoter-T	-12	NM_14604	218335	Clptm1l	C130052I1
chr4	3731427	3731525	Intergenic	Intergenic	31271	NM_02614	67427	Rps20	4632426K0
chr2	1.27E+08	1.27E+08	intron (NM	intron (NM	1220	NM_02322	66552	Sppl2a	2010106G0
chr16	24238752	24238825	Intergenic	Intergenic	-154648	NM_17866	210126	Lpp	9430020K1
chr9	72961177	72961400	promoter-T	promoter-T	12	NM_19860	225215	Rsl24d1	2410159K2
chr13	5874727	5875125	Intergenic	Intergenic	14191	NM_01180	23849	Klf6	AI448727|
chr10	11021927	11022025	Intergenic	Intergenic	20591	NM_00116	71865	Fbxo30	1700026A1
chr11	96691902	96692100	promoter-T	promoter-T	-719	NM_00868	18023	Nfe2l1	AA408798|
chr5	1.03E+08	1.03E+08	Intergenic	MTE-int|LT	-56609	NM_14616	231532	Arhgap24	0610025G2
chr1	1.36E+08	1.36E+08	Intergenic	Intergenic	20599	NM_05407	269120	Optc	-
chr14	70451452	70451550	intron (NM	intron (NM	-25751	NM_01878	13655	Egr3	Pilot
chr7	57033852	57034225	exon (NM_	exon (NM_	310	NM_13374	71974	Prmt3	2010005E2
chr15	74358677	74358775	intron (NM	MIR|SINE|	12100	NM_17499	107831	Bai1	B830018M
chr3	9609327	9609550	exon (NM_	exon (NM_	647	NM_13321	170753	Zfp704	C030026M
chr13	65341927	65342000	Intergenic	Intergenic	-34939	NM_17529	97895	Nlrp4f	C330026N0
chr13	1.02E+08	1.02E+08	intron (NM	intron (NM	-26403	NM_00102	18708	Pik3r1	PI3K|p50al
chr1	18542277	18542725	Intergenic	LTR33C|LT	-287282	NM_00104	654464	Gm15386	BD-17|Def
chr2	18615752	18616325	Intergenic	CpG	-4611	NM_00100	381350	BC061194	-
chrX	74776427	74776550	intron (NM	intron (NM	19922	NM_02060	21372	Tbl1x	5330429M
chr17	48386702	48386775	promoter-T	promoter-T	-158	NM_02140	58218	Trem3	BB134760
chr1	1.08E+08	1.08E+08	promoter-T	promoter-T	238	NM_17318	227446	2310035C2	6430401N1
chr11	94189177	94189575	promoter-T	promoter-T	61	NM_14602	71452	Ankrd40	1110011C0
chr8	86424127	86424200	5' UTR (NM	5' UTR (NM	166	NM_18103	330814	Lphn1	2900070I0
chr15	98702002	98702200	promoter-T	promoter-T	-465	NM_00103	381022	Kmt2d	ALR|BC032
chr1	99558077	99558350	intron (NM	CpG	382	NM_13382	52392	D1Ertd622	AI987691|
chr1	1.56E+08	1.56E+08	intron (NM	intron (NM	3357	NM_01188	24014	Rnasel	E230029I0
chr15	1E+08	1E+08	promoter-T	promoter-T	-318	NM_15340	207785	Csrnp2	CSRNP-2|C
chr12	81111902	81112300	Intergenic	Intergenic	101899	NM_00756	12192	Zfp36l1	AW742437
chr19	3401627	3401850	intron (NM	intron (NM	12360	NM_00128	17771	Mtl5	tesmin
chr9	13767652	13767800	Intergenic	Intergenic	135555	NM_17683	72826	Fam76b	2810485I0
chr4	59442402	59442525	intron (NM	intron (NM	9042	NM_00116	634731	Susd1	A530080P1
chr11	1.07E+08	1.07E+08	intron (NM	intron (NM	51233	NM_14582	71795	Pitpnc1	1110020B0
chr8	3672277	3672500	Intergenic	Intergenic	-4089	NM_02570	66682	Trappc5	4021401A1
chr8	1.17E+08	1.17E+08	Intergenic	Intergenic	-27296	NM_00100	403395	Clec3a	1110019O1
chr1	1.83E+08	1.83E+08	intron (NM	MTB|LTR|	7669	NM_14594	208768	Sde2	-
chr4	97800927	97801000	Intergenic	Intergenic	248993	NM_05315	94043	Tm2d1	2310026L1
chr12	4600627	4600775	intron (NM	intron (NM	887	NM_00119	20403	Itsn2	AI327390|
chr8	28089127	28089200	exon (NM_	exon (NM_	1355	NM_00111	353310	Zfp703	1110032O1
chr7	31017352	31017450	promoter-T	promoter-T	308	NM_02725	69920	Polr2i	2810002B1
chr9	1.04E+08	1.04E+08	intron (NM	intron (NM	28922	NM_00116	235567	Dnajc13	D030002L1
chr15	5193977	5194100	5' UTR (NM	5' UTR (NM	149	NM_00113	19219	Ptger4	EP4|Ptgere

chr10	92605852	92605975	Intergenic	(CA)n|Simp	-17708	NM_14623	237459	Cdk17	6430598J1
chr8	71614952	71615175	intron (NM	intron (NM	-1942	NM_15305	110877	Slc18a1	4832416I1
chr5	1.23E+08	1.23E+08	intron (NM	intron (NM	12089	NM_00987	12589	Ift81	AW060663
chr1	1.46E+08	1.46E+08	Intergenic	Lx9|LINE|L	43678	NM_00906	19735	Rgs2	GOS8
chr2	79538152	79538250	Intergenic	Intergenic	-9736	NM_17242	75276	Ppp1r1c	4930565M
chr2	1.58E+08	1.58E+08	intron (NM	intron (NM	6884	NM_00848	16803	Lbp	Bpifd2|Ly8
chr9	92116952	92117125	Intergenic	Lx9|LINE|L	-27994	NM_01163	22038	Plscr1	MmTRA1a
chr9	71521402	71521500	intron (NM	ORR1D2|LT	-81284	NM_00103	102371	Myzap	AA407270|
chr8	47848927	47849125	intron (NM	intron (NM	23927	NM_00839	16363	Irf2	9830146E2
chr11	69446677	69446775	5' UTR (NM	5' UTR (NM	253	NM_01181	23879	Fxr2	Fxr2h
chr13	21545302	21545425	intron (NM	intron (NM	233	NM_00101	432731	Zscan26	BC068174|
chr2	34683802	34683900	Intergenic	Intergenic	-2096	NM_00116	30050	Fbxw2	2700071L0
chr12	80453227	80453425	intron (NM	intron (NM	54988	NM_00125	19363	Rad51b	AI553500|
chr1	1.35E+08	1.35E+08	intron (NM	intron (NM	31124	NM_00109	240752	Pik3c2b	C330011J1
chr1	38053527	38054100	intron (NM	CpG	240	NM_17205	98258	Txndc9	AI098020|
chr15	66920202	66920350	Intergenic	Intergenic	88168	NM_00917	20442	St3gal1	5330418N2
chr19	3696177	3696325	Intergenic	Intergenic	-9687	NM_00851	16973	Lrp5	BMND1|HI
chr7	1.5E+08	1.5E+08	intron (NR_	intron (NR_	17962	NM_00112	27027	Tspan32	AW208513
chr4	83132102	83132300	5' UTR (NM	5' UTR (NM	151	NM_00129	101739	Psip1	AA408851|
chr16	44458052	44458200	intron (NM	Lx8|LINE|L	63214	NM_00103	212517	Cfap44	6330444M
chr5	1.45E+08	1.45E+08	intron (NM	intron (NM	-12179	NM_17768	231874	Ccz1	AU022870
chr7	1.28E+08	1.28E+08	intron (NM	intron (NM	3031	NM_03069	80719	Igsf6	-
chr16	78296152	78296250	Intergenic	Intergenic	-5715	NM_00998	13052	Cxadr	2610206D0
chr5	33678577	33678800	intron (NM	CpG	467	NM_02150	59003	Maea	1110030D1
chr6	1.34E+08	1.34E+08	intron (NM	intron (NM	1288	NM_00796	14011	Etv6	AW123102
chr18	15221577	15221825	exon (NM_	exon (NM_	390	NM_00114	106931	Kctd1	4933402K1
chr17	24563652	24563725	promoter-T	promoter-T	60	NM_01002	13177	Eci1	Dci|eci
chr13	46822752	46822950	5' UTR (NM	5' UTR (NM	367	NM_17574	218210	Nup153	B130015D1
chr8	73429927	73430175	intron (NM	CpG	178	NM_17301	270058	Map1s	6430517J1
chr5	37201802	37202175	intron (NM	intron (NM	-13995	NM_02624	67568	Mrfap1	9130413I2
chr1	59013252	59013475	intron (NM	intron (NM	16963	NM_17240	70827	Trak2	2900022D0
chr1	1.37E+08	1.37E+08	promoter-T	promoter-T	-295	NM_15377	226432	Ipo9	0710008K0
chr19	47939977	47940125	promoter-T	promoter-T	16	NM_03005	68214	Gsto2	1700020F0
chr5	1.38E+08	1.38E+08	intron (NM	CpG-11668	351	NM_03140	83701	Srrt	2810019G0
chr5	67655277	67655550	intron (NM	intron (NM	3609	NM_03010	67878	Tmem33	1110006G0
chr11	1.16E+08	1.16E+08	Intergenic	Intergenic	-13174	NM_00117	20698	Sphk1	1110006G2
chr5	1.37E+08	1.37E+08	Intergenic	RMER3D-in	-12919	NM_00116	66437	Fis1	2010003O1
chr6	90666452	90666575	promoter-T	promoter-T	10	NM_00113	232227	Iqsec1	AW561907
chr2	58176752	58176950	intron (NM	intron (NM	367	NM_00103	269275	Acvr1c	ACVRLK7|A
chr12	99613477	99613800	Intergenic	Lx8|LINE|L	106793	NM_00815	14744	Gpr65	Dig1|Gpcr2
chr6	83005852	83005950	exon (NM_	exon (NM_	1254	NM_00751	11993	Aup1	AA589454
chr2	1.18E+08	1.18E+08	Intergenic	Intergenic	-134720	NM_01158	21825	Thbs1	TSP-1|TSP1
chr4	88510652	88510850	Intergenic	Intergenic	14760	NM_01050	15962	Ifna1	Ifa1
chr2	1.2E+08	1.2E+08	intron (NM	intron (NM	16253	NM_01194	26390	Mapkbp1	2810483F2
chr12	86940452	86940725	promoter-T	promoter-T	222	NM_03088	81703	Jdp2	Jundm2|Ju
chr2	1.03E+08	1.03E+08	Intergenic	Intergenic	-65879	NM_00103	12505	Cd44	AU023126
chr12	60320552	60321025	promoter-T	promoter-T	-318	NM_00103	70611	Fbxo33	5730501N2

chr5	50008852	50009050	Intergenic	Intergenic	-332053	NM_00119	80334	Kcnip4	AV032399|
chr16	16073377	16073775	intron (NM	intron (NM	73368	NM_14606	224008	Spidr	2310008H(
chr10	90671952	90672275	Intergenic	Intergenic	-37749	NM_00108	21917	Tmpo	5630400D2
chr17	26708352	26708700	intron (NM	intron (NM	10069	NM_02617	67458	Ergic1	1200007D1
chr2	1.67E+08	1.67E+08	intron (NM	CpG	217	NM_17837	77031	Slc9a8	1200006P1
chr15	75706552	75706750	Intergenic	Intergenic	12502	NM_00128	223645	Mroh6	Gm19570
chr6	1.31E+08	1.31E+08	promoter-T	promoter-T	86	NM_02556	66441	Magohb	2010012C1
chr12	80273427	80273600	promoter-T	promoter-T	-68	NM_01680	53612	Vti1b	AU015348
chr8	87185552	87185750	3' UTR (NM	3' UTR (NM	1100	NM_01049	15936	Ier2	AI317238|(
chr19	45332527	45332600	Intergenic	Intergenic	-22837	NM_01069	16814	Lbx1	Lbx1h
chr12	1.12E+08	1.12E+08	Intergenic	Intergenic	-10108	NM_01048	15519	Hsp90aa1	86kDa|89k
chr14	76552577	76552725	Intergenic	tRNA-Glu-(	41953	NM_01374	27275	Nufip1	Nufip
chr12	86114427	86114550	promoter-T	promoter-T	268	NM_02886	74316	Isca2	0710001C0
chr7	1.26E+08	1.26E+08	Intergenic	ORR1D2|LT	-8374	NM_00103	319622	Itpripl2	C130081G2
chr11	1.21E+08	1.21E+08	intron (NM	CpG	223	NM_02579	66840	Wdr45b	0610008N2
chr4	1.29E+08	1.29E+08	Intergenic	(TG)n|Simp	-12017	NM_01689	11637	Ak2	Ak-2|D4Ert
chr15	63828202	63828350	intron (NM	intron (NM	63734	NM_14484	223601	Fam49b	0910001A0
chr14	55172977	55173150	5' UTR (NM	5' UTR (NM	135	NM_14546	219072	Haus4	9430093H(
chr11	1.19E+08	1.19E+08	Intergenic	Intergenic	-15649	NM_01392	30951	Cbx8	Pc3
chr3	88489777	88489925	intron (NM	CpG	135	NM_02881	74200	2810403A(	A430106P1
chr3	1.08E+08	1.08E+08	intron (NM	intron (NM	494	NM_00128	72522	Atxn7l2	2610528J1
chr11	87860902	87861125	5' UTR (NM	5' UTR (NM	138	NM_17337	110809	Srsf1	1110054N1
chr11	4603302	4603475	intron (NM	B1_Mur4|S	959	NM_19797	66152	Uqcr10	1110020P1
chr8	90722852	90722925	promoter-T	promoter-T	-224	NM_00116	214627	Papd5	5730445M
chr17	6406177	6406325	Intergenic	Intergenic	-23009	NM_00934	21648	Dynlt1b	AGS2|Dynl
chr6	72849277	72849350	intron (NM	CpG	660	NM_01971	74287	Kcmf1	1700094M
chr10	40021452	40021550	intron (NM	CpG	491	NM_00744	1E+08	Amd2	AdoMetDC
chr7	1.46E+08	1.46E+08	promoter-T	promoter-T	32	NM_02639	52432	Ppp2r2d	1300017E1
chr9	1.14E+08	1.14E+08	intron (NM	PB1D10|SI	2731	NM_17862	72179	Fbxl2	2810423A2
chr10	59406452	59406625	Intergenic	Intergenic	7980	NM_02908	74747	Ddit4	5830413E0
chr7	66042927	66043150	intron (NM	intron (NM	129466	NM_00972	11982	Atp10a	Atp10c|pfa
chr5	33616852	33616950	intron (NM	CpG	752	NM_00119	13016	Ctbp1	BARS|CtBP
chr11	79788377	79788650	Intergenic	HAL1|LINE	-12624	NM_14482	216987	Utp6	4732497O(
chr10	41777402	41777550	Intergenic	Intergenic	-39288	NM_00103	213402	Armc2	2610018I0
chr15	99803077	99803200	promoter-T	promoter-T	-73	NM_00102	207214	Larp4	DXErtd793
chr5	1.22E+08	1.22E+08	intron (NM	B3A|SINE|	12883	NM_00912	20239	Atxn2	9630045M
chr3	51220027	51220175	5' UTR (NM	5' UTR (NM	163	NM_05308	74838	Naa15	5730450D1
chr1	1.93E+08	1.93E+08	Intergenic	Intergenic	47946	NM_02542	66208	Nenf	1110060M
chr10	53608327	53608500	Intergenic	LTR33A|LT	-137698	NM_00108	75906	Fam184a	3110012E0
chr2	27370827	27371100	intron (NM	CpG	296	NM_08084	140858	Wdr5	2410008O(
chr12	81537727	81537800	promoter-T	promoter-T	-175	NM_17726	320808	Dcaf5	9430020B0
chr3	40599252	40599400	Intergenic	Intergenic	-4547	NM_17316	20873	Plk4	1700028H2
chr15	8363352	8363425	intron (NM	MTE2b|LTI	31075	NM_02770	71175	Nipbl	Idn3
chr3	95622877	95623125	promoter-T	promoter-T	-125	NM_00108	75137	Rprd2	2810036A1
chr10	77478127	77478250	Intergenic	Intergenic	-5647	NM_00882	18641	Pfkl	AA407869|
chr14	75469052	75469350	Intergenic	Intergenic	53367	NM_19864	271221	5031414D1	Gm744|Gn
chr3	51100827	51100975	intron (NM	intron (NM	-19465	NM_00129	69257	Elf2	2610036A2

chr11	67340152	67340400	Intergenic	Intergenic	-6224	NM_00808	14457	Gas7	AW124766
chr8	96982477	96982675	Intergenic	Intergenic	-14087	NM_00103	434341	Nlrc5	AI451557|/
chr19	27161952	27162125	Intergenic	Intergenic	-129472	NM_01370	22359	Vldlr	AA408956|
chr10	1.11E+08	1.11E+08	intron (NM	intron (NM	10512	NM_17861	52705	Krr1	2610511F0
chr4	1.39E+08	1.39E+08	promoter-T	promoter-T	6	NM_02985	77056	Tmco4	4632413C1
chr9	4308952	4309200	promoter-T	promoter-T	418	NM_02627	67618	Aasdhppt	2010309J2
chr1	1.73E+08	1.73E+08	intron (NM	B1_Mus2|S	-7965	NM_00100	226652	Arhgap30	6030405P0
chr3	1.03E+08	1.03E+08	Intergenic	Charlie12|	85410	NM_00107	94093	Trim33	8030451N0
chr9	36504002	36504200	intron (NM	intron (NM	3296	NM_00739	11451	Acrv1	Msa63|SP-
chr9	31088477	31088725	Intergenic	CpG	9615	NM_00110	638580	Gm7244	EG638580
chr4	1.33E+08	1.33E+08	Intergenic	MER53|DN	-18644	NM_00101	503610	Zdhhc18	-
chr15	76307927	76308075	promoter-T	promoter-T	126	NM_00829	15499	Hsf1	AA960185|
chr3	1.1E+08	1.1E+08	intron (NM	intron (NM	182427	NM_00116	80883	Ntng1	A930010C0
chr13	36180152	36180325	intron (NM	intron (NM	28988	NM_20135	380840	Lyrm4	BC034664|
chr6	54987602	54987775	promoter-T	promoter-T	-307	NM_18067	353172	Gars	GENA202|
chr16	21857777	21858000	Intergenic	Intergenic	-34154	NM_17282	71751	Map3k13	C130026N1
chr11	29274102	29274175	promoter-T	promoter-T	-34	NM_17684	108686	Ccdc88a	A430106J1
chr3	94820327	94820525	Intergenic	Intergenic	-1266	NM_03007	78266	Zfp687	4931408L0
chr4	12016952	12017300	intron (NM	CpG-9742	609	NM_19895	77604	Rbm12b2	AV299215|
chr19	33001177	33001375	Intergenic	Intergenic	169209	NM_00896	19211	Pten	2310035O0
chr17	31345177	31345350	intron (NM	intron (NM	252	NM_17782	328795	Ubash3a	5830413C0
chr7	1.46E+08	1.46E+08	Intergenic	Intergenic	-1424	NM_00976	12176	Bnip3	Nip3
chr5	73211427	73211625	intron (NM	intron (NM	48161	NM_00111	21682	Tec	-
chr11	54347477	54347550	intron (NM	RLTR14|LT	11166	NM_00125	192786	Rapgef6	A530068K0
chr1	74331027	74331100	promoter-T	promoter-T	249	NM_14611	227290	Aamp	AU040907
chr5	43517077	43517300	Intergenic	Intergenic	-107514	NM_17593	231207	Cpeb2	A630055H1
chr3	51504577	51504750	intron (NM	intron (NM	39548	NM_17499	211666	Mgst2	GST2|MGS
chr19	6061327	6061425	exon (NM_	exon (NM_	169	NM_01385	29805	Znhit2	C11orf5|Fo
chr4	1.45E+08	1.45E+08	intron (NM	B1_Mur1|S	6372	NM_01161	21938	Tnfrsf1b	CD120b|TN
chr19	41338077	41338175	intron (NM	CpG	368	NM_13335	107358	Tm9sf3	1810073M
chr8	80299552	80299650	Intergenic	L1MB5|LIN	-51250	NM_01033	13617	Ednra	ET-AR|ETa
chr4	1.48E+08	1.48E+08	intron (NM	intron (NM	44120	NM_01978	56273	Pex14	Pex14p|R7
chr4	1.55E+08	1.55E+08	intron (NM	intron (NM	5231	NM_01138	20481	Ski	2310012I0
chr4	4007377	4007675	Intergenic	Intergenic	-2865	NM_00108	242286	Sdr16c6	4833413O1
chr4	34423377	34423575	Intergenic	Intergenic	-74388	NM_00100	433693	Akirin2	2700059D2
chr16	36071202	36071475	promoter-T	promoter-T	263	NM_02734	70186	Fam162a	2310056P0
chr9	50812277	50812450	intron (NM	intron (NM	4815	NM_17871	235344	Sik2	G630080D2
chr4	98787427	98787525	5' UTR (NM	5' UTR (NM	130	NM_00129	67299	Dock7	3110056M
chr9	78073127	78073325	Intergenic	Intergenic	-5250	NM_00818	14857	Gsta1	Gst2-1|OT
chr14	55725002	55725300	TTS (NM_1	TTS (NM_1	279	NM_00745	11766	Ap1g2	Adtg2|G2a
chr7	34951952	34952025	intron (NM	RSINE1|SIN	1560	NM_01668	50995	Uba2	AA986091|
chr4	53163427	53163575	intron (NM	intron (NM	9266	NM_01345	11303	Abca1	ABC-1|Abc
chr8	1.12E+08	1.12E+08	promoter-T	promoter-T	-19	NM_02801	71955	Ist1	2400003C1
chr9	66766777	66766950	intron (NM	intron (NM	649	NM_17341	235442	Rab8b	5930437D1
chr15	76701552	76701750	5' UTR (NM	5' UTR (NM	214	NM_00100	71591	Zfp251	9130001M
chr1	1.73E+08	1.73E+08	intron (NM	intron (NM	2589	NM_01872	18106	Cd244	2B4|C9.1|
chr4	1.03E+08	1.03E+08	promoter-T	promoter-T	-24	NM_17773	242585	Slc35d1	AI834976|

chr10	1.2E+08	1.2E+08	TTS (NM_0	TTS (NM_0	-28317	NM_08044	117599	Helb	AI447783|
chr2	1.53E+08	1.53E+08	intron (NM	intron (NM	9629	NM_02554	66405	Mcts2	2400002F1
chr12	33725702	33725825	intron (NM	intron (NM	51977	NM_00102	68764	Cdhr3	1110049B0
chr11	88962552	88962675	Intergenic	Intergenic	27458	NM_00103	217071	Gm525	-
chr11	69664627	69664750	non-coding	non-coding	4542	NR_03313	70310	Plscr3	2210403O2
chr4	74121827	74122025	Intergenic	Intergenic	224143	NM_14478	76804	Kdm4c	2410141F1
chr17	46889077	46889275	promoter-T	promoter-T	-15	NM_02806	72029	Cnpy3	1600025D1
chr5	1.49E+08	1.49E+08	Intergenic	Intergenic	-22299	NM_01022	14254	Flt1	AI323757|
chr2	32463002	32463175	intron (NM	intron (NM	583	NR_11034	50935	St6galnac6	ST6GalNAc
chr3	57267427	57267525	exon (NM_	exon (NM_	38144	NM_14553	229302	Tm4sf4	Iltmp
chr17	32457327	32457525	TTS (NM_0	TTS (NM_0	672	NM_01977	56399	Akap8	1200016A0
chr9	1.08E+08	1.08E+08	Intergenic	Intergenic	-3154	NM_00129	20347	Sema3b	SemA|Sem
chr16	32550227	32550350	Intergenic	Intergenic	53921	NM_19930	245308	Zdhhc19	Gm1744|G
chr13	51805977	51806075	intron (NM	intron (NM	58943	NM_02927	75420	Secisbp2	2210413N0
chr14	65256277	65256475	Intergenic	Intergenic	-14992	NM_00108	16554	Kif13b	5330429L1
chr11	1.2E+08	1.2E+08	intron (NM	intron (NM	1208	NM_13379	192662	Arhgdia	5330430M
chr8	1.25E+08	1.25E+08	Intergenic	Intergenic	-3171	NM_01391	30927	Snai3	AI643946|
chr19	46975727	46976275	intron (NM	intron (NM	60840	NM_02981	76952	Nt5c2	2010002I2
chr17	50514927	50515050	Intergenic	MER20|DN	-82043	NM_00127	13164	Dazl	Daz-like|Da
chr9	15514127	15514375	promoter-T	promoter-T	38	NM_17228	234967	Slc36a4	6330573I1
chr6	52916202	52916275	intron (NM	intron (NM	-56624	NM_00116	231986	Jazf1	AI591476|
chr3	88230127	88230325	Intergenic	MTD|LTR|	-1163	NM_00114	229517	Slc25a44	6720482A1
chr15	84753752	84753975	promoter-T	promoter-T	5	NM_01671	18141	Nup50	1700030K0
chr9	1.15E+08	1.15E+08	intron (NM	CpG	367	NM_17538	333433	Gpd1l	2210409H2
chr12	1.02E+08	1.02E+08	promoter-T	promoter-T	-563	NM_15358	73086	Rps6ka5	3110005L1
chr6	1.37E+08	1.37E+08	exon (NM_	exon (NM_	239	NM_17565	320332	Hist4h4	B130044J0
chr9	65062402	65062650	promoter-T	promoter-T	29	NM_17746	214424	Parp16	ARTD15|B0
chr2	59924352	59924600	intron (NM	intron (NM	39321	NM_00100	407823	Baz2b	5830435C1
chr1	1.81E+08	1.81E+08	promoter-T	promoter-T	535	NM_00824	15278	Tfb2m	Hkp1
chr13	16027602	16028175	Intergenic	Intergenic	-78420	NM_00838	16323	Inhba	-
chr6	99162452	99162625	intron (NM	intron (NM	53971	NM_00119	108655	Foxp1	3110052D1
chr12	77938627	77938725	intron (NM	CpG	222	NM_14592	110606	Fntb	2010013E1
chr1	72335152	72335350	intron (NM	B4|SINE|B4	4304	NM_17556	271711	Tmem169	A830020B0
chr8	1.18E+08	1.18E+08	Intergenic	Intergenic	-11894	NM_00102	17132	Maf	2810401A2
chr1	5073227	5073525	intron (NM	CpG	122	NM_13382	108664	Atp6v1h	0710001F1
chr3	54916127	54916300	5' UTR (NM	5' UTR (NM	125	NM_00114	229285	Spg20	AI840044|
chr2	75776627	75776900	promoter-T	promoter-T	-244	NM_02823	72421	Ttc30b	2510042P0
chr5	32424377	32424575	Intergenic	Intergenic	-14369	NM_00803	14284	Fosl2	Fra-2
chr1	1.64E+08	1.64E+08	Intergenic	Intergenic	-23684	NM_17264	226551	Suco	4732491B1
chr3	65470202	65470425	intron (NM	CpG	163	NM_00116	624866	Lekr1	EG546798|
chr6	72340102	72340475	intron (NM	intron (NM	373	NM_01679	22320	Vamp8	AU041171
chr1	88467977	88468325	intron (NM	intron (NM	10925	NM_00880	18582	Pde6d	AI841218|
chr5	23032702	23032925	intron (NM	intron (NM	89576	NM_00927	20817	Srpk2	AW226533
chr5	1.39E+08	1.39E+08	Intergenic	RMER15|LT	-47359	NM_03056	80752	Fam20c	C76981|DN
chr1	22744202	22744800	intron (NM	intron (NM	68062	NM_00101	116837	Rims1	C030033M
chr12	3441302	3441575	intron (NM	intron (NM	14581	NM_17242	75302	Asxl2	4930556B1
chr6	29297877	29297950	promoter-T	promoter-T	-193	NM_00759	12321	Calu	9530075H2

chr18	66600927	66601025	Intergenic	URR1B|DN	-17282	NM_02145	58801	Pmaip1	Noxa
chr14	98504002	98504175	intron (NM	intron (NM	64896	NM_00103	13134	Dach1	Dac|Dach
chr13	96669852	96670025	Intergenic	Intergenic	-8061	NM_02771	544963	Iqgap2	4933417J2
chr8	1.05E+08	1.05E+08	Intergenic	Intergenic	266460	NM_00986	12552	Cdh11	Cad11
chr18	47019177	47019400	intron (NM	intron (NM	9595	NM_02900	74574	4833403I1!	Aqpep|Lvr
chr19	5366427	5366875	promoter-T	promoter-T	-162	NM_02723	69860	Eif1ad	2010003J0
chr2	1.52E+08	1.52E+08	promoter-T	promoter-T	-725	NM_00778	12995	Csnk2a1	Csnk2a1-rs
chr4	1.26E+08	1.26E+08	Intergenic	Intergenic	-6802	NR_04556	12986	Csf3r	Cd114|Csf
chr9	42679827	42680050	intron (NM	intron (NM	72516	NM_17548	110637	Grik4	6330551K0
chr2	22957902	22958050	intron (NM	Lx8|LINE|L	33384	NM_00110	74159	Acbd5	1300014E1
chr17	3115127	3115400	5' UTR (NM	5' UTR (NM	291	NM_13412	106583	Scaf8	A630086M
chr11	52174052	52174125	promoter-T	promoter-T	-529	NM_01169	22333	Vdac1	AL033343|
chr10	6648652	6648925	Intergenic	Intergenic	-42624	NM_00115	213783	Plekhg1	D10Ertd73
chr17	26122277	26122350	Intergenic	CpG	-19863	NM_01583	50817	Capn15	Solh
chr10	13702902	13703025	intron (NM	intron (NM	16778	NM_01043	15273	Hivep2	MIBP1|Sch
chr9	1.06E+08	1.06E+08	intron (NM	intron (NM	1507	NM_19893	67905	Ppm1m	2810423O1
chr9	24307452	24307525	promoter-T	promoter-T	96	NM_17292	244745	Dpy19l1	1100001I1!
chr2	1.58E+08	1.58E+08	Intergenic	Intergenic	-16998	NM_00937	21817	Tgm2	G[a]h|TG2
chr4	1.16E+08	1.16E+08	promoter-T	promoter-T	-444	NM_02986	77110	Gpbp1l1	5330440M
chr13	45446877	45447075	Intergenic	Intergenic	-38135	NM_15378	218203	Mylip	9430057C2
chr5	1.51E+08	1.51E+08	intron (NM	CpG	149	NM_17531	100710	Pds5b	AI646570|
chr13	76155777	76155850	5' UTR (NM	5' UTR (NM	197	NM_17891	218341	Rfesd	AI256775|
chr10	79834577	79835175	Intergenic	Intergenic	15520	NM_19861	237400	Mex3d	BC059858|
chr8	1.08E+08	1.08E+08	5' UTR (NM	5' UTR (NM	210	NM_02151	59016	Thap11	2810036E2
chr12	1.09E+08	1.09E+08	promoter-T	promoter-T	15	NM_00983	12454	Ccnk	AW123198
chr5	1.26E+08	1.26E+08	intron (NM	CpG	399	NM_01963	22190	Ubc	2700054O0
chr2	38341477	38341650	Intergenic	Intergenic	-25654	NM_02160	59126	Nek6	1300007C0
chr5	1.43E+08	1.43E+08	Intergenic	CpG	-33838	NM_19906	17425	Foxk1	A630048H0
chr11	81820377	81820525	Intergenic	Intergenic	-28628	NM_01133	20296	Ccl2	AI323594|
chr1	75208527	75208625	exon (NM_	exon (NM_	1172	NM_01149	20872	Stk16	EDPK|Krct|
chr19	5024202	5024275	5' UTR (NM	5' UTR (NM	232	NM_00785	13340	Slc29a2	Der12|Ent
chr15	74786377	74786525	intron (NM	intron (NM	915	NM_00116	17069	Ly6e	9804|Ly67
chr11	68912277	68912400	promoter-T	promoter-T	-120	NM_01106	18626	Per1	Per|m-rigu
chr2	52717227	52717325	exon (NM_	exon (NM_	374	NM_17240	71409	Fmnl2	5430425K0
chr9	32464477	32464550	Intergenic	Intergenic	-39114	NM_01180	23871	Ets1	AI196000|
chr4	1.4E+08	1.4E+08	intron (NM	CpG	563	NM_17386	108911	Rcc2	2610510H0
chr3	30992502	30993050	Intergenic	CpG	-1204	NM_01138	20482	Skil	Skir|SnoN|
chr17	28622527	28622750	intron (NM	CpG	456	NM_01022	14229	Fkbp5	D17Ertd59
chr7	1.34E+08	1.34E+08	promoter-T	promoter-T	-403	NM_00926	20768	Sephs2	Sps2|Ysg3
chr6	38303627	38303900	intron (NM	URR1B|DN	840	NM_02886	78781	Zc3hav1	1200014N1
chr2	1.57E+08	1.57E+08	Intergenic	Intergenic	-37699	NM_02998	77799	Sla2	A930009E2
chr3	1.16E+08	1.16E+08	intron (NM	intron (NM	25799	NM_00108	229776	Cdc14a	A830059A1
chr15	99328652	99328775	3' UTR (NM	3' UTR (NM	-23552	NM_02923	75284	Bcdin3d	4930556P0
chr1	1.61E+08	1.61E+08	intron (NM	intron (NM	1319	NM_01193	26374	Rfwd2	AI316802|
chr4	1.55E+08	1.55E+08	Intergenic	Intergenic	-1144	NM_01138	20481	Ski	2310012I0
chr11	4943052	4943200	TTS (NM_0	TTS (NM_0	-15005	NM_14521	75668	Rasl10a	2210403B1
chrX	10294052	10294150	promoter-T	promoter-T	-390	NM_02652	68041	Mid1ip1	3110038L0

chr6	1.15E+08	1.15E+08	Intergenic	Intergenic	-21346	NM_19903	381802	Tsen2	AU067695
chr6	5149877	5150100	Intergenic	Intergenic	-6042	NM_01113	18979	Pon1	Pon
chr2	1.57E+08	1.57E+08	Intergenic	RMER15|L	-1468	NM_00113	19650	Rbl1	AW547426
chr16	57606902	57607150	promoter-T	promoter-T	-46	NM_02559	66497	Cmss1	1110001AC
chr12	81564077	81564375	5' UTR (NM	5' UTR (NM	144	NM_13379	97827	Exd2	4930539P1
chr10	86242252	86242400	intron (NM	intron (NM	576	NM_17533	103466	Nt5dc3	AU040402
chr2	24817902	24818025	promoter-T	promoter-T	21	NM_02604	67228	Dph7	2810443J1
chr2	61383577	61383875	Intergenic	Intergenic	-32917	NM_01152	21353	Tank	C86182|E4
chr2	1.46E+08	1.46E+08	intron (NM	intron (NM	35899	NM_02872	74030	Rin2	2010003K1
chr7	1.52E+08	1.52E+08	exon (NM_	exon (NM_	153	NM_01017	14082	Fadd	Mort1/FAD
chr15	93228752	93229000	promoter-T	promoter-T	-19	NM_00128	223828	Pphln1	CR|HSPC20
chr13	43071577	43071775	intron (NM	MIR|SINE|	-146532	NM_00100	218194	Phactr1	9630030F1
chr4	1.06E+08	1.06E+08	exon (NM_	exon (NM_	345	NM_18322	329908	Usp24	2700066KC
chr16	23927802	23927975	intron (NM	intron (NM	2992	NM_00100	224055	Rtp2	Gm605
chrX	9040002	9040075	intron (NM	intron (NM	6412	NM_00780	13058	Cybb	C88302|CG
chr2	19310127	19310200	intron (NM	intron (NM	16900	NM_02961	76467	Msrb2	2310050L0
chr18	50139927	50140325	intron (NM	intron (NM	1045	NM_00117	106869	Tnfaip8	AA987150|
chr14	1.06E+08	1.06E+08	Intergenic	Intergenic	-1215	NM_01189	24064	Spry2	sprouty2
chr8	48619052	48619250	promoter-T	promoter-T	152	NM_20350	192174	Rwdd4a	BC016198|
chr11	71854952	71855100	promoter-T	promoter-T	-978	NM_14452	109212	Fam64a	2610008F0
chr10	17205977	17206125	Intergenic	MIRc|SINE	-236983	NM_01082	17684	Cited2	AI835299|
chr2	90418102	90418275	intron (NM	intron (NM	2616	NM_00898	19271	Ptprj	AI450271|
chr11	9600727	9601050	Intergenic	Intergenic	508943	NM_17825	268379	Abca13	9830132L2
chr17	34254127	34257000	exon (NM_	exon (NM_	1282	NR_03797(	14312	Brd2	AW228947
chr4	48565677	48565850	intron (NM	intron (NM	12392	NM_02813	66665	Msantd3	2410046L1
chr13	99714827	99714975	Intergenic	Intergenic	-18562	NM_17871	238799	Tnpo1	AU021749
chr7	17004077	17004650	intron (NM	intron (NM	17818	NM_19863	330474	Zc3h4	Bwq1|Gm7
chr5	1.24E+08	1.24E+08	intron (NM	L2a|LINE|L	6575	NM_17509	23912	Rhof	AI845056|
chr4	1.32E+08	1.32E+08	intron (NM	intron (NM	428	NM_00103	230787	Themis2	ICB-1
chr10	67491102	67491325	intron (NM	intron (NM	48867	NM_00108	170799	Rtkn2	B130039D2
chr9	72297402	72297500	intron (NM	intron (NM	11115	NM_00861	17427	Mns1	AW546487
chr17	35074927	35075150	TTS (NM_0	TTS (NM_0	6840	NM_01089	18010	Neu1	AA407268|
chr14	31364277	31364350	intron (NM	intron (NM	1996	NM_00938	21881	Tkt	p68
chr6	16915302	16915575	Intergenic	Intergenic	-66997	NM_03119	21426	Tfec	BB107417|
chr6	30643327	30643725	intron (NM	CpG	156	NM_03199	83922	Cep41	1700017E1
chr15	36264602	36264700	Intergenic	Intergenic	-51749	NM_01392	30945	Rnf19a	AA032313|
chr2	29980052	29980300	promoter-T	promoter-T	-45	NM_00129	227693	Zer1	C230075L1
chr14	31832702	31832775	promoter-T	promoter-T	414	NM_00108	66923	Pbrm1	2610016F0
chr16	33921652	33921950	intron (NM	7SK|RNA|F	45288	NM_00947	22247	Umps	1700095D2
chr7	1.07E+08	1.07E+08	intron (NM	L3b|LINE|(	9204	NM_20135	233552	Gdpd5	BC024955|
chr16	87440702	87440800	promoter-T	promoter-T	86	NM_01692	53858	Rwdd2b	ORF5
chr8	1.09E+08	1.09E+08	promoter-T	promoter-T	295	NM_00100	66432	Slc7a6os	2010007L1
chr6	92041552	92042025	intron (NM	CpG	376	NM_01163	22026	Nr2c2	TAK1|Tr4|
chr13	75916702	75916775	Intergenic	Intergenic	-60596	NM_05310	93692	Glrx	C86710|D1
chr10	19242202	19242400	Intergenic	Intergenic	-69463	NM_01051	15979	Ifngr1	CD119|IFN
chr19	42254477	42254550	intron (NM	intron (NM	9456	NM_00116	319740	Zfyve27	2210011NC
chr4	1.55E+08	1.55E+08	intron (NM	CpG	752	NM_02689	68991	Ssu72	1190002E2

chr13	1.12E+08	1.12E+08	promoter-T	promoter-T	-198	NM_17259	218613	Mier3	5730509D1
chr12	1.03E+08	1.03E+08	Intergenic	MER2|DNA	-44816	NM_17215	238384	Slc24a4	A930002M
chr10	69346852	69347300	intron (NM	intron (NM	-41204	NM_17068	11735	Ank3	2900054D0
chr17	74301477	74301775	Intergenic	Intergenic	-2090	NM_01172	22436	Xdh	XO|Xor|Xo
chr6	48551527	48551775	Intergenic	Intergenic	7769	NM_00107	58887	Repin1	AI425994|A
chr5	1.4E+08	1.4E+08	Intergenic	Intergenic	-2245	NM_17272	231821	Adap1	4930431P1
chr1	1.64E+08	1.64E+08	Intergenic	Intergenic	-1034	NM_17264	226551	Suco	4732491B1
chr1	1.23E+08	1.23E+08	promoter-T	promoter-T	-62	NM_13374	72999	Insig2	2900053I1
chr7	1.06E+08	1.06E+08	intron (NM	intron (NM	710	NM_02638	67800	Dgat2	0610010B0
chr2	1.8E+08	1.8E+08	promoter-T	promoter-T	-126	NM_13384	99296	Hrh3	AW049250
chr11	21123627	21123925	Intergenic	Intergenic	-15259	NM_13906	245944	Vps54	Hcc8|Vps5
chr8	1.26E+08	1.26E+08	intron (NM	intron (NM	1122	NM_13395	69581	Rhou	2310026M
chr17	25716077	25716150	promoter-T	promoter-T	-6	NM_02962	76483	Lmf1	2400010G1
chr16	33169702	33170050	Intergenic	RMER19B|	-15281	NM_17684	106326	Osbpl11	9430097N0
chr6	1.47E+08	1.47E+08	Intergenic	Intergenic	-52169	NM_00897	19227	Pthlh	PTH-like|Pt
chr17	73457352	73457450	promoter-T	promoter-T	76	NM_00117	225010	Lclat1	AI181996|A
chr5	23293552	23293800	exon (NM_	exon (NM_	114	NM_17732	72772	Rint1	1500019C0
chr1	1.82E+08	1.82E+08	intron (NM	CpG	983	NM_02637	226747	Ahctf1	6230412P2
chr1	88050877	88051050	promoter-T	promoter-T	-392	NM_03018	78795	Armc9	3830422A1
chr1	23930852	23931100	Intergenic	Intergenic	-1756	NM_02853	98366	Smap1	1700056O1
chr17	24210252	24210525	promoter-T	promoter-T	64	NM_02700	69259	Kctd5	2610030N0
chr17	18008677	18008925	Intergenic	Intergenic	12102	NM_01352	14293	Fpr1	FPR|LXA4R
chr9	1.07E+08	1.07E+08	Intergenic	L1MC|LINE	22967	NM_17548	235599	6430571L1	-
chr12	1.21E+08	1.21E+08	Intergenic	Intergenic	-26509	NM_00129	217951	Tmem196	Gm528
chr11	5441927	5442100	exon (NM_	exon (NM_	207	NM_13403	104479	Ccdc117	1110004K0
chr8	72240452	72240575	promoter-T	promoter-T	-128	NM_18127	66869	Zfp869	1200003I0
chr2	32243377	32243525	promoter-T	promoter-T	-192	NM_00849	16819	Lcn2	24p3|AW2
chr13	99695777	99695850	intron (NM	CpG	526	NM_17871	238799	Tnpo1	AU021749
chr19	32334452	32334600	5' UTR (NM	5' UTR (NM	-49023	NM_00116	208449	Sgms1	9530058O1
chr17	46993552	46993650	Intergenic	CpG	-25239	NM_00110	210982	Gltscr1l	mKIAA0240
chr4	1.5E+08	1.5E+08	intron (NM	CpG	82470	NM_17377	242773	Slc45a1	C230078B2
chr18	35658127	35658250	intron (NM	intron (NM	391	NM_03074	81500	Sil1	1810057E0
chr12	1.1E+08	1.1E+08	intron (NM	intron (NM	77551	NM_00116	380785	Begain	BM948371
chr11	54680252	54680350	intron (NM	CpG	361	NM_00824	15254	Hint1	AA673479|
chr4	1.07E+08	1.07E+08	promoter-T	promoter-T	503	NM_02956	56374	Tmem59	1110001M
chr16	48788802	48788875	Intergenic	Intergenic	-16769	NM_19829	77647	Trat1	C030046M
chr14	84853877	84854200	intron (NM	intron (NM	10668	NM_00101	219228	Pcdh17	C030033F1
chr10	18734752	18734925	intron (NM	intron (NM	378	NM_00939	21929	Tnfaip3	A20|Tnfip3
chr16	55974102	55974325	non-coding	non-coding	296	NM_17302	271377	Zbtb11	9230110G0
chr11	98448627	98448900	promoter-T	promoter-T	-204	NM_02566	66612	Ormdl3	2810011N1
chr8	27350027	27350275	Intergenic	Intergenic	81510	NM_19904	73754	Thap1	4833431A0
chr4	15941202	15941325	promoter-T	promoter-T	-239	NM_14595	209212	Osgin2	BC010311|
chr4	1.06E+08	1.06E+08	Intergenic	Intergenic	-4877	NM_02559	329910	Acot11	1110020M
chr8	6191402	6191600	Intergenic	RMER19A|	-1086269	NM_01138	20494	Slc10a2	ASBT|ISBT
chr1	1.33E+08	1.33E+08	3' UTR (NM	3' UTR (NM	-6836	NM_01977	56489	Ikbke	AW558201
chr10	1.27E+08	1.27E+08	exon (NM_	exon (NM_	204	NM_00100	216443	Mars	Metrs|Mtr
chr11	95007277	95007625	promoter-T	promoter-T	-336	NM_00786	13394	Dlx4	Dlx-4|Dlx7

chr15	78871527	78871600	promoter-T	promoter-T	-752	NM_01573	14429	Galr3	Galnr3
chr7	1.07E+08	1.07E+08	promoter-T	promoter-T	293	NM_01205	27050	Rps3	D7Ertd795
chr11	22273277	22273425	Intergenic	Intergenic	-86553	NM_00125	216565	Ehbp1	AF424697|
chr3	90280102	90280725	intron (NM	CpG	290	NM_02637	67781	Ilf2	6230405A1
chrX	1.01E+08	1.01E+08	Intergenic	RSINE1|SIN	-42932	NM_00107	245555	C77370	A230051P1
chr16	17210202	17210300	exon (NM_	exon (NM_	2023	NM_00103	239731	Rimbp3	Gm1759|G
chr3	1.33E+08	1.33E+08	intron (NM	RMER15|LT	11971	NM_02792	71793	Ints12	1110020M
chr6	1.31E+08	1.31E+08	promoter-T	promoter-T	-83	NM_00846	16633	Klra2	Klra30|Ly4
chr3	1.21E+08	1.21E+08	intron (NM	intron (NM	11845	NM_00103	619318	4930432M	-
chr17	29636352	29636450	Intergenic	Intergenic	8411	NM_00884	18712	Pim1	Pim-1
chr17	35096452	35096625	promoter-T	promoter-T	-355	NM_01047	15511	Hspa1b	Hsp70|Hsp
chr5	81487202	81487425	intron (NM	intron (NM	36695	NM_19870	319387	Lphn3	5430402I2
chr4	1.38E+08	1.38E+08	intron (NM	intron (NM	1362	NM_00109	230863	Sh2d5	BC036961
chr4	98674877	98675200	intron (NM	intron (NM	-22608	NM_01391	30924	Angptl3	hypl
chr13	43589152	43589325	Intergenic	Intergenic	-12896	NM_01993	56705	Ranbp9	IBAP-1|Iba
chr1	88400127	88400375	Intergenic	Intergenic	-23060	NM_00897	19231	Ptma	Thym
chr1	1.72E+08	1.72E+08	intron (NM	RSINE1|SIN	11665	NM_02752	70729	Nos1ap	6330408P1
chr15	96485477	96485550	Intergenic	ORR1E|LTR	-12169	NM_00116	105727	Slc38a1	AA408026|
chr8	28088602	28088775	intron (NM	CpG	880	NM_00111	353310	Zfp703	1110032O1
chr16	4790152	4790225	promoter-T	promoter-T	-253	NM_02567	66626	Cdip1	2700048O1
chr13	23960477	23960950	promoter-T	promoter-T	-174	NR_121611	20504	Slc17a1	NAPI-1|Na
chr1	90172627	90172875	intron (NM	intron (NM	1381	NM_17250	212427	A730008H2	mFleg1
chr1	95021652	95021950	Intergenic	Intergenic	-10016	NM_01670	11611	Agxt	AGT|Agt1|
chr3	69526077	69526425	intron (NM	CpG	274	NM_13378	97112	Nmd3	C87860
chr19	37517302	37517600	Intergenic	Intergenic	8120	NM_00824	15242	Hhex	Hex|Hex1|
chr11	1.09E+08	1.09E+08	promoter-T	promoter-T	-816	NM_00102	104681	Slc16a6	AW743111
chr14	66598777	66598850	intron (NM	intron (NM	11493	NM_01349	12759	Clu	AI893575|A
chr17	21653852	21653950	Intergenic	Intergenic	-18602	NM_14451	22710	Zfp52	KRAB11|Zf
chr1	1.01E+08	1.01E+08	Intergenic	Intergenic	292800	NM_02758	70866	Slco6d1	4921511I05
chr11	23155127	23155475	promoter-T	promoter-T	-740	NM_00103	103573	Xpo1	AA420417|
chr10	79502727	79502800	promoter-T	promoter-T	-359	NM_02555	66420	Polr2e	2410021N1
chr19	5567402	5567475	promoter-T	promoter-T	-636	NM_00103	381201	Ap5b1	Gm962
chr15	96587852	96587950	Intergenic	PB1D10|SI	-57772	NM_17512	67760	Slc38a2	5033402L1
chr1	1.34E+08	1.34E+08	promoter-T	promoter-T	-243	NM_17251	213464	Rbbp5	4933411J24
chr16	31664227	31664350	non-coding	non-coding	267	NM_00125	13383	Dlg1	B130052P0
chr5	1.45E+08	1.45E+08	promoter-T	promoter-T	156	NM_00888	18861	Pms2	AW555130
chr14	37141527	37141825	Intergenic	Intergenic	233525	NM_17500	218921	4930474N0	-
chr7	87260152	87260575	promoter-T	promoter-T	-127	NM_17301	269951	Idh2	E430004F2
chr13	76193252	76193775	exon (NM_	exon (NM_	931	NM_17549	238725	Gpr150	C030001A1
chr11	1.19E+08	1.19E+08	intron (NM	CpG	216	NM_00806	14387	Gaa	E430018M0
chr9	1.1E+08	1.1E+08	promoter-T	promoter-T	-199	NM_00869	18054	Ngp	bectenecin
chr15	77685477	77685600	Intergenic	ID_B1|SINE	-12933	NM_02241	17886	Myh9	Fltn|Myhn
chr5	1.39E+08	1.39E+08	promoter-T	promoter-T	-204	NM_17787	330216	Mblac1	-
chr8	42502252	42502400	5' UTR (NM	5' UTR (NM	146	NM_01352	14300	Frg1	-
chr16	36874652	36874800	5' UTR (NM	5' UTR (NM	186	NM_13411	106389	Eaf2	AW048865
chr2	11489527	11489950	Intergenic	L1M2|LINE	-14182	NM_00117	170768	Pfkfb3	E330010H2
chr6	1.29E+08	1.29E+08	intron (NM	intron (NM	1243	NM_05310	93694	Clec2d	Clr-b|Clrb|

chr6	17335502	17335950	Intergenic	Intergenic	78086	NM_00124	12389	Cav1	Cav|Cav-1
chr13	1.02E+08	1.02E+08	promoter-T	promoter-T	-104	NM_00987	12572	Cdk7	AI323415|/
chr1	26743927	26744050	intron (NM	RMER4B|L	317	NM_00103	210940	4931408C2	-
chr17	73267777	73267925	5' UTR (NM	5' UTR (NM	206	NM_02999	77889	Lbh	1810009F1
chr17	26078652	26078725	TTS (NM_0	TTS (NM_0	246	NM_00129	14755	Pigq	Gpi1|Gpi1l
chr17	34341702	34341925	intron (NM	CpG	389	NM_01153	21355	Tap2	ABC18|AI4
chr9	63450027	63450325	promoter-T	promoter-T	79	NM_03006	78250	Iqch	4921504K0
chr8	72426402	72426525	promoter-T	promoter-T	-6	NM_02331	67184	Ndufa13	2700054G1
chr15	77074677	77074750	intron (NM	MIR|SINE|	62770	NM_00111	93686	Rbfox2	Fbm2|Fxh|
chr6	1.29E+08	1.29E+08	intron (NM	intron (NM	17567	NM_17552	243653	Clec1a	5930406N1
chr6	1.23E+08	1.23E+08	Intergenic	Intergenic	-9099	NM_00116	17474	Clec4d	Clecsf8|Mp
chr5	1.23E+08	1.23E+08	TTS (NM_0	TTS (NM_0	21718	NR_104441	654470	Tctn1	G730031O
chr3	94142552	94142775	Intergenic	HAL1|LINE	-3358	NM_02541	66198	Them5	1110007B0
chr9	1.11E+08	1.11E+08	intron (NM	intron (NM	28186	NM_00116	71268	Lrrfip2	5133400F2
chr6	1.25E+08	1.25E+08	Intergenic	Intergenic	-30679	NM_19860	213522	Plekhg6	BC026778|
chr5	1.19E+08	1.19E+08	Intergenic	Intergenic	-84002	NM_17242	76199	Med13l	2210413I1
chr10	20561252	20561525	Intergenic	Lx9|LINE|L	-110965	NM_02620	52906	Ahi1	1700015F0
chr5	38611802	38611925	promoter-T	promoter-T	142	NM_02528	17089	Lyar	MLZ-264
chr19	20460902	20461175	intron (NM	intron (NM	4123	NM_01073	16952	Anxa1	Anx-1|Anx-
chr2	94277727	94277850	intron (NM	CpG	516	NM_00746	11800	Api5	AAC-11|AI
chr13	1.09E+08	1.09E+08	promoter-T	promoter-T	244	NM_02804	71991	Ercc8	2410022P0
chr2	91366952	91367175	Intergenic	CpG	-19416	NM_00116	75786	Ckap5	3110043H2
chr11	70796202	70796450	exon (NM_	exon (NM_	202	NM_00757	12261	C1qbp	AA407365|
chr9	44144127	44144300	TTS (NM_0	TTS (NM_0	1415	NM_01043	15270	H2afx	AW228881
chr4	1.35E+08	1.35E+08	promoter-T	promoter-T	-535	NM_00128	14105	Srsf10	FUSIP2|Fu
chr5	63068727	63068825	intron (NM	intron (NM	88640	NM_17840	212285	Arap2	Centd1|Gn
chr1	97319002	97319075	Intergenic	Intergenic	108833	NM_02632	67698	Fam174a	2310044D2
chr3	1.06E+08	1.06E+08	intron (NM	intron (NM	156	NM_00103	433638	I830077J02	-
chr10	9705427	9705650	Intergenic	Intergenic	-84700	NM_00108	78808	Stxbp5	0710001E2
chr9	13632202	13632375	promoter-T	promoter-T	117	NM_17683	72826	Fam76b	2810485I0
chr3	58273327	58273975	Intergenic	Intergenic	54040	NM_00108	72033	Tsc22d2	1810043J1
chr8	83267327	83267400	Intergenic	Lx7|LINE|L	-4005	NM_05312	93762	Smarca5	4933427E2
chr2	1.63E+08	1.63E+08	Intergenic	Intergenic	-3663	NM_00108	241764	L3mbtl1	C630004G0
chr3	21860252	21860325	Intergenic	Intergenic	-115286	NM_03073	81004	Tbl1xr1	8030499H0
chr12	1.13E+08	1.13E+08	Intergenic	Intergenic	-5759	NM_00939	21928	Tnfaip2	B94|Exoc3
chr5	1.43E+08	1.43E+08	intron (NM	intron (NM	1153	NM_17272	231855	Ap5z1	C330006K0
chr3	1.23E+08	1.24E+08	Intergenic	MLT2D|LTI	-106356	NR_121616	83398	Ndst3	4921531K0
chr13	58486252	58486650	exon (NM_	exon (NM_	135	NM_02733	70153	2210016F1	-
chr12	1.01E+08	1.01E+08	Intergenic	Intergenic	-11742	NM_18318	71375	Foxn3	5430426H2
chr11	49527027	49527150	promoter-T	promoter-T	-864	NM_21248	104625	Cnot6	CCR4
chr3	94690527	94690600	exon (NM_	exon (NM_	317	NM_00894	19172	Psmb4	Pros-27
chr5	1.37E+08	1.37E+08	5' UTR (NM	5' UTR (NM	135	NM_01882	23921	Sh2b2	Aps
chr3	60332252	60332400	intron (NM	intron (NM	27152	NM_00125	56758	Mbnl1	Mbnl|mKIA
chr9	30837552	30838650	promoter-T	promoter-T	-128	NM_00111	235132	Zbtb44	6030404E1
chr6	1.21E+08	1.21E+08	intron (NM	intron (NM	14825	NM_00754	12122	Bid	2700049M
chr10	4829577	4829725	intron (NM	intron (NM	33802	NM_15339	64009	Syne1	8B|A33004
chr5	1.35E+08	1.35E+08	promoter-T	promoter-T	-146	NM_00119	68758	Abhd11	1110054D1

chr5	8051702	8051775	intron (NM	intron (NM	-4804	NM_00108	109552	Sri	2210417O(
chr10	1.28E+08	1.28E+08	intron (NM	PB1D10|SI	3347	NM_00125	103135	Pan2	1200014O2
chr5	1.22E+08	1.22E+08	intron (NM	intron (NM	2272	NM_17272	231713	Naa25	4833422K1
chr2	10002327	10002425	promoter-1	promoter-1	137	NM_02528	16588	Kin	Kin17
chr3	1.2E+08	1.2E+08	Intergenic	Intergenic	-217432	NM_01955	56195	Ptbp2	Ptb2|brPTI
chr4	1.19E+08	1.19E+08	Intergenic	Intergenic	-36063	NM_00819	14916	Guca2b	AV066530|
chr7	52917227	52917300	intron (NM	intron (NM	4501	NM_01887	14344	Fut2	-
chr6	1.18E+08	1.18E+08	intron (NM	intron (NM	559	NM_19433	213895	Bms1	AA408648|
chr2	1.43E+08	1.43E+08	intron (NM	CpG	199	NM_00108	16558	Kif16b	8430434E1
chr17	31702377	31702575	intron (NM	CpG	758	NM_01667	18771	Pknox1	D17Wsu76
chr14	56407302	56407400	intron (NM	intron (NM	343	NM_00116	56532	Ripk3	2610528K(
chr4	45542652	45542725	exon (NM_	exon (NM_	1012	NM_00103	230126	Shb	BC028832
chr6	23996902	23997250	Intergenic	Intergenic	-77016	NM_20702	387347	Tas2r118	Gm465|T2
chr12	1.12E+08	1.12E+08	Intergenic	Intergenic	-10376	NM_00113	26931	Ppp2r5c	2610043M
chr8	1.11E+08	1.11E+08	intron (NM	intron (NM	50357	NM_00749	11906	Zfhx3	A230102L(
chr6	29072852	29073275	TTS (NM_1	TTS (NM_1	41661	NM_13392	68272	Rbm28	2810480G1
chr1	1.29E+08	1.29E+08	intron (NM	intron (NM	3375	NM_14512	107895	Mgat5	4930471A2
chr3	96067302	96067600	promoter-1	promoter-1	-211	NM_03359	97122	Hist2h4	H4|X04652
chr11	1.21E+08	1.21E+08	intron (NM	CpG	223	NM_02774	71276	Ccdc57	4933434G(
chr6	34731002	34731075	5' UTR (NM	5' UTR (NM	606	NM_00128	76223	Agbl3	2900053G1
chr4	1.38E+08	1.38E+08	intron (NM	MT2B|LTR	1742	NM_17270	230861	Eif4g3	1500002J2
chr9	1.1E+08	1.1E+08	intron (NM	B3A|SINE|	79503	NM_00125	72831	Dhx30	2810477H(
chr7	53179777	53179850	Intergenic	CpG	-3017	NM_01012	13732	Emp3	H-4|H4|HN
chr6	86682777	86682925	intron (NM	CpG	521	NM_01181	23885	Gmcl1	2810049L1
chr19	37385227	37385525	intron (NM	B1F|SINE|/	19727	NM_03115	15925	Ide	1300012G(
chr1	1.27E+08	1.27E+08	Intergenic	MER5A1|D	92441	NM_02373	74117	Actr3	1200003A(
chr10	20905077	20905150	Intergenic	Intergenic	-24323	NM_01084	17863	Myb	AI550390|
chr1	1.3E+08	1.3E+08	Intergenic	Intergenic	5300	NM_00991	12767	Cxcr4	CD184|Cm
chr2	54410652	54411025	intron (NM	intron (NM	122040	NM_17303	271786	Galnt13	A230002A1
chr17	29528452	29528575	Intergenic	Intergenic	30654	NM_00115	26382	Fgd2	Tcd-2|Tcd2
chr8	1.08E+08	1.08E+08	Intergenic	ORR1D2|L1	-3118	NM_17516	70796	Zdhhc1	4432412D(
chr4	62020902	62021050	promoter-1	promoter-1	606	NM_00104	338355	Fkbp15	BB131447|
chr1	30930727	30930875	Intergenic	CpG	-10700	NM_00108	213109	Phf3	2310061N1
chr6	53395752	53396000	Intergenic	Intergenic	-127492	NM_17272	231991	Creb5	Crebpa|D4
chr15	51524277	51524475	Intergenic	Intergenic	172631	NM_08063	68135	Eif3h	1110008A1
chr1	1.87E+08	1.87E+08	5' UTR (NM	5' UTR (NM	346	NM_00825	15284	Hlx	Hlx1
chr1	1.52E+08	1.52E+08	Intergenic	Lx8|LINE|L	-32306	NM_00111	96875	Prg4	CACP|DOL!
chr18	7869802	7869975	intron (NM	CpG	693	NM_15308	225131	Wac	1110067P(
chr17	45935602	45935850	Intergenic	Intergenic	-64933	NM_02816	72240	1600014C2	-
chr8	75015977	75016100	intron (NM	CpG	475	NM_17776	270066	Slc35e1	6030458H(
chr7	1.33E+08	1.33E+08	Intergenic	L1M4b|LIN	-6655	NM_02188	60504	Il21r	NILR
chr1	1.3E+08	1.3E+08	intron (NM	CpG	505	NM_17744	226414	Dars	5730439G1
chr1	1.74E+08	1.74E+08	Intergenic	RMER16-in	-42475	NM_01373	27218	Slamf1	4933415F1
chr3	89855777	89856100	promoter-1	promoter-1	380	NM_00128	99650	4933434E2	5730552F2
chr9	45792727	45792875	5' UTR (NM	5' UTR (NM	153	NM_00877	18475	Pafah1b2	AI747451|/
chr3	75761252	75761325	promoter-1	promoter-1	-417	NM_00129	73124	Golim4	3110027H2
chr18	20904252	20904350	5' UTR (NM	5' UTR (NM	604	NM_01973	56386	B4galt6	AA536803|

chr4	1.32E+08	1.32E+08	exon (NM_	exon (NM_	383	NM_14490	230784	Sesn2	HI95|SEST2
chr6	29997677	29997875	promoter-T	promoter-T	-212	NM_01093	18181	Nrf1	C87038|D6
chr19	3809027	3809175	TTS (NM_0	TTS (NM_0	41680	NM_00116	225888	Suv420h1	AA117471|
chr10	85333927	85334075	promoter-T	promoter-T	-575	NM_13399	103136	Pwp1	2310058A1
chr3	83843377	83843750	intron (NM	CpG	520	NM_17268	229473	D930015E0	Kiaa0922|r
chr16	64987477	64987825	intron (NM	intron (NM	117959	NM_00831	15557	Htr1f	Htr1eb
chr12	13276127	13276200	intron (NM	intron (NM	230	NM_02770	71169	Nbas	4933425L0
chr1	1.68E+08	1.68E+08	Intergenic	Intergenic	-1335	NM_03024	27878	Tada1	2900026B1
chr10	42619652	42619900	intron (NM	RLTR11A2|	39458	NM_17293	268297	Scml4	9330161D1
chr14	55321552	55321700	Intergenic	Intergenic	9385	NM_20713	110794	Cebpe	C/EBPe|CR
chr9	45651552	45651850	intron (NM	intron (NM	5089	NM_01179	23821	Bace1	C76936
chr4	1.49E+08	1.49E+08	intron (NM	intron (NM	1666	NM_00116	18707	Pik3cd	2410099E0
chr19	5649552	5649675	Intergenic	L2|LINE|L2	12123	NM_00904	19697	Rela	p65
chr9	44892627	44892900	intron (NM	intron (NM	5497	NM_00100	270152	Amica1	AMICA|Cre
chr16	35022852	35023075	intron (NM	CpG	456	NM_02358	70757	Ptplb	6330408J2
chr17	57171627	57171850	promoter-T	promoter-T	-808	NM_01061	16549	Khsrp	6330409F2
chr12	17355052	17355250	promoter-T	promoter-T	-148	NM_00100	217431	Nol10	Gm67
chr5	1.3E+08	1.3E+08	Intergenic	Intergenic	-12041	NM_02712	69568	Vkorc1l1	2310024K0
chr11	1.01E+08	1.01E+08	promoter-T	promoter-T	24	NM_02661	52469	Coa3	1810033A1
chr13	23677202	23677325	TTS (NM_0	TTS (NM_0	576	NM_00129	319179	Hist1h2be	-
chr14	79881802	79881900	promoter-T	promoter-T	-776	NM_01876	22380	Wbp4	AW545037
chr3	41368902	41369000	intron (NM	CpG	149	NM_00113	269424	Jade1	AU041499
chr14	21167052	21167275	promoter-T	promoter-T	180	NM_02747	70601	Ecd	5730461K0
chr10	80986277	80986425	intron (NM	intron (NM	619	NM_01030	14676	Gna15	G[a]15|Gal
chr17	35368002	35368125	intron (NM	ID_B1|SINE	4697	NM_01090	18038	Nfkbil1	Def-7|IKBL
chr7	1.51E+08	1.51E+08	intron (NM	LTR31|LTR	4004	NM_00785	13360	Dhcr7	AA409147
chr12	54009377	54009525	intron (NM	intron (NM	209081	NM_19811	238161	Akap6	AI482140|A
chr10	79754577	79756275	intron (NM	CpG	250	NM_00909	20054	Rps15	rig
chr2	1.56E+08	1.56E+08	promoter-T	promoter-T	-24	NM_02666	68295	Aar2	0610011L1
chr8	67226177	67226425	intron (NM	intron (NM	-13926	NM_02543	66234	Msmo1	1500001G1
chr11	44317527	44317950	Intergenic	Lx8|LINE|L	-14728	NM_02886	74315	Rnf145	3732413I1
chr11	31761502	31761850	Intergenic	MIRb|SINE	-9264	NM_00129	67579	Cpeb4	Cpe-bp4|C
chr8	36028277	36028575	exon (NM_	exon (NM_	318	NM_17509	21951	Tnks	4930554K1
chr4	1.35E+08	1.35E+08	intron (NM	CpG	32053	NM_01973	12399	Runx3	AML2|Cbfa
chr5	1.51E+08	1.51E+08	Intergenic	Intergenic	-3229	NM_17288	320365	Fry	13CDNA73
chr6	1.19E+08	1.19E+08	exon (NM_	exon (NM_	273	NM_00108	232339	Ankrd26	5730521P1
chr15	90879552	90879725	intron (NM	CpG	744	NM_00110	16564	Kif21a	-
chr6	1.27E+08	1.27E+08	Intergenic	Intergenic	-52705	NM_00103	381812	Cracr2a	Efcab4b|Gr
chr5	1.44E+08	1.44E+08	intron (NM	ID_B1|SINE	10740	NM_14491	231871	Daglb	E330036I1
chr3	1.33E+08	1.33E+08	promoter-T	promoter-T	-884	NM_00104	214133	Tet2	Ayu17-449
chr4	48137727	48137900	promoter-T	promoter-T	22	NM_02634	67727	Stx17	4833418L0
chr8	1.09E+08	1.09E+08	intron (NM	intron (NM	-20865	NM_01090	18021	Nfatc3	C80703|D8
chr2	1.63E+08	1.63E+08	intron (NM	CpG	368	NM_02569	66680	Oser1	3230401D1
chr2	1.64E+08	1.64E+08	Intergenic	Intergenic	-3638	NM_00127	11486	Ada	-
chr6	47404077	47404150	promoter-T	promoter-T	-210	NM_01204	26965	Cul1	-
chr9	62971927	62972025	Intergenic	Intergenic	22811	NM_17244	207667	Skor1	AV273001|
chr4	1.19E+08	1.19E+08	intron (NM	intron (NM	3663	NM_01140	20525	Slc2a1	Glut-1|Glu

chrY	2787227	2787375	Intergenic	AT_rich|Lo	400043	NM_00127	1E+08	Gm3376	Rbmy1b
chr17	5287302	5287375	intron (NM	intron (NM	131429	NM_17534	106557	Ldhal6b	4933402O1
chr19	21470902	21471175	intron (NM	intron (NM	76113	NM_01026	14544	Gda	AU015411
chr9	1.09E+08	1.09E+08	intron (NM	MLT1-int|L	-15155	NM_02585	66940	Shisa5	2310008D1
chr8	24622302	24622525	Intergenic	Intergenic	100439	NM_01383	20377	Sfrp1	2210415K0
chr6	1.42E+08	1.42E+08	promoter-T	promoter-T	-16	NM_18316	232491	Pyroxd1	-
chr1	1.56E+08	1.56E+08	promoter-T	promoter-T	-230	NM_01188	24014	Rnasel	E230029I04
chr2	52279927	52280125	intron (NM	CpG	368	NM_18299	75423	Arl5a	2410015N2
chr2	1.54E+08	1.54E+08	Intergenic	Intergenic	-4485	NM_00789	13555	E2f1	E2F-1|mKIA
chr3	8667727	8667975	promoter-T	promoter-T	-813	NM_01042	15213	Hey1	AI316788|A
chr17	33700702	33700950	intron (NM	intron (NM	8174	NM_05321	17916	Myo1f	C330006B1
chr11	1.16E+08	1.16E+08	exon (NM_	exon (NM_	379	NM_17880	338364	Trim65	4732463G1
chr2	1.04E+08	1.04E+08	promoter-T	promoter-T	159	NM_00111	53872	Caprin1	AL022980|
chr16	18101802	18102125	Intergenic	RMER19A|	-12680	NM_01117	19125	Prodh	Pro-1|Pro1
chr7	19893577	19893800	intron (NM	intron (NM	1706	NM_00803	14282	Fosb	-
chr14	69906777	69907075	Intergenic	Intergenic	-3766	NM_02633	67712	Slc25a37	1700020E2
chr11	1.03E+08	1.03E+08	Intergenic	B3|SINE|B	-1726	NM_13875	192231	Hexim1	7330426E1
chr1	1.22E+08	1.22E+08	intron (NM	intron (NM	16731	NM_01943	170706	Tmem37	AI173373|A
chr1	30930052	30930625	Intergenic	CpG	-10237	NM_00108	213109	Phf3	2310061N1
chr13	47139727	47139850	intron (NM	intron (NM	920	NM_17226	218214	Kdm1b	4632428N0
chr6	52196027	52196250	promoter-T	promoter-T	-329	NM_01045	15396	Hoxa11	Hox-1.9|Ho
chr2	1.65E+08	1.65E+08	intron (NM	intron (NM	-4020	NM_20770	140579	Elmo2	1190002F2
chr13	34967302	34967475	promoter-T	promoter-T	25	NM_01383	19134	Prpf4b	2610037H0
chr8	90129702	90130000	Intergenic	Intergenic	42045	NM_17790	330820	4933402J0	-
chrX	1.17E+08	1.17E+08	Intergenic	LTRIS5|LTR	132221	NM_05311	93728	Pabpc5	C820015E1
chr10	66902077	66902450	Intergenic	Intergenic	-98354	NM_01011	13654	Egr2	Egr-2|Krox
chr5	1.21E+08	1.21E+08	intron (NM	(TGAA)n|Si	9652	NM_00805	14357	Dtx1	Fxit1|mKIA
chr7	1.26E+08	1.26E+08	Intergenic	Intergenic	21501	NM_00103	319622	Itpripl2	C130081G2
chr12	1.19E+08	1.19E+08	intron (NM	intron (NM	1715	NM_01006	13411	Dnah11	Dnahc11|b
chr5	1.48E+08	1.48E+08	Intergenic	Lx8|LINE|L	-10090	NM_00125	76366	Mtif3	2810012L1
chr16	20097802	20098000	intron (NM	CpG	274	NM_02943	75785	Klhl24	1110046J1
chr1	1.63E+08	1.63E+08	intron (NM	intron (NM	122	NM_00103	226541	Klhl20	AI504637|I
chr12	74388727	74388800	intron (NM	intron (NM	928	NM_00103	625098	Slc38a6	AW322671
chr3	88100127	88100200	promoter-T	promoter-T	597	NM_02980	76927	Tsacc	1700021C1
chr10	82417977	82418100	Intergenic	SSU-rRNA_	-30204	NM_02143	58250	Chst11	1110020P0
chr13	1.06E+08	1.06E+08	promoter-T	promoter-T	-5	NM_02607	67285	Cwc27	3110009E1
chr6	64378402	64378500	intron (NM	intron (NM	-300689	NM_00750	11921	Atoh1	Hath1|MA
chr11	53163927	53164100	promoter-T	promoter-T	-256	NM_03356	93736	Aff4	AF5Q31|Al
chr1	1.2E+08	1.2E+08	exon (NM_	exon (NM_	131	NM_02647	67949	Nifk	AI852665|
chr15	43309002	43309150	promoter-T	promoter-T	301	NM_02573	66736	Emc2	4921531G1
chr9	71333152	71333975	promoter-T	promoter-T	227	NM_17860	28015	Polr2m	AA407243|
chr9	44066952	44067375	intron (NM	intron (NM	-7616	NM_13322	170761	Pdzd3	NaPi-Cap2|
chr4	1.52E+08	1.52E+08	intron (NM	intron (NM	23791	NM_00108	269610	Chd5	4930532L2
chr8	1.22E+08	1.22E+08	intron (NM	intron (NM	189	NM_02795	71839	Osgin1	1700012B1
chr6	1.46E+08	1.46E+08	intron (NM	CpG	507	NM_01058	16439	Itpr2	AI649341|I
chr6	1.14E+08	1.14E+08	intron (NM	MIRc|SINE	-54043	NM_00103	11941	Atp2b2	D6Abb2e|
chr1	1.84E+08	1.84E+08	promoter-T	promoter-T	-685	NM_17337	209456	Trp53bp2	53BP2|AI7

chr12	1.14E+08	1.14E+08	Intergenic	Intergenic	14525	NM_00110	382639	Zbtb42	EG382639|
chr2	1.81E+08	1.81E+08	promoter-T	promoter-T	59	NM_00100	269400	Rtel1	AI451565|A
chr2	1.31E+08	1.31E+08	promoter-T	promoter-T	-8	NM_00129	69596	Ap5s1	0610038L1
chrX	1.36E+08	1.36E+08	intron (NM	intron (NM	-12691	NM_00125	66889	Rnf128	1300002C1
chr13	63533177	63533250	promoter-T	promoter-T	-160	NM_00128	14088	Fancc	Facc
chr3	10466002	10466250	Intergenic	Intergenic	-25996	NM_00112	74718	Snx16	4930522N2
chr11	1.16E+08	1.16E+08	intron (NM	intron (NM	-9946	NM_02527	14027	Evpl	-
chr5	3502252	3502425	intron (NM	intron (NM	-41495	NM_00104	68152	Fam133b	2900022K0
chr1	64135427	64135650	intron (NM	intron (NM	32425	NM_03356	93691	Klf7	9830124P0
chr2	1.12E+08	1.12E+08	exon (NM_	exon (NM_	405	NM_14688	258889	Olfr1301	MOR248-5
chr11	1.01E+08	1.01E+08	5' UTR (NM	5' UTR (NM	187	NM_02789	71743	Coasy	1300003G0
chr15	76491077	76491150	promoter-T	promoter-T	42	NM_01063	16581	Kifc2	-
chr10	59853777	59853850	intron (NR_	intron (NR_	-8661	NM_00116	1E+08	Gm17455	-
chr19	6364527	6364625	intron (NR_	CpG	886	NR_12211	22668	Sf1	BBP|MZFM
chr3	1.22E+08	1.22E+08	Intergenic	Intergenic	-49937	NM_00812	14630	Gclm	AI649393|
chr14	47614452	47614750	intron (NM	intron (NM	-6414	NM_00116	74480	Samd4	1700024G0
chr8	98377102	98377175	intron (NM	intron (NM	253	NM_17275	234595	Slc38a7	D430050E1
chr4	55972927	55973300	Intergenic	Intergenic	-427766	NM_01063	16600	Klf4	EZF|Gklf|Z
chr15	81524627	81524850	exon (NM_	exon (NM_	2979	NM_00116	214685	Chadl	AY100452|
chr14	32594577	32594850	intron (NM	intron (NM	48888	NM_00102	105522	Ankrd28	AI465466|A
chr1	94639027	94639175	Intergenic	Intergenic	-89162	NM_01669	14733	Gpc1	AI462976
chr7	1.07E+08	1.07E+08	exon (NM_	exon (NM_	2787	NM_02814	72184	Klhl35	2810406K1
chr7	1.17E+08	1.17E+08	intron (NM	intron (NM	104109	NM_00930	20947	Swap70	70kDa|AV2
chr6	71391077	71391400	promoter-T	promoter-T	-607	NM_02428	68477	Rmnd5a	1110007A0
chr8	1.27E+08	1.27E+08	intron (NM	CpG	333	NM_13396	102162	Taf5l	1110005N0
chr1	95448127	95448300	intron (NM	intron (NM	39532	NM_14551	227377	Farp2	AI465173|
chr1	1.3E+08	1.3E+08	promoter-T	promoter-T	218	NM_18175	226412	R3hdm1	R3hdm
chr7	51969302	51969425	intron (NM	ORR1C1|LT	5250	NM_02931	75510	Izumo2	1700023D1
chr8	1.07E+08	1.07E+08	promoter-T	promoter-T	151	NM_00130	12354	Car7	AV343731|
chr1	82836102	82836425	5' UTR (NM	5' UTR (NM	205	NM_01047	15463	Agfg1	AU045498
chr12	1.13E+08	1.13E+08	promoter-T	promoter-T	191	NM_02740	70369	Bag5	1600025G0
chr19	37776902	37777325	Intergenic	Intergenic	4815	NM_00781	13082	Cyp26a1	Cyp26|P45
chr5	1.37E+08	1.37E+08	promoter-T	promoter-T	-341	NM_00129	13047	Cux1	CDP|Cutl1
chr18	3042277	3042350	Intergenic	RMER16-in	81099	NM_00116	1E+08	Vmn1r238	-
chr14	79690427	79690550	intron (NM	intron (NM	10954	NM_02542	66214	Rgcc	1190002H2
chr15	1.03E+08	1.03E+08	TTS (NR_03	TTS (NR_03	165	NM_02646	67942	Atp5g2	1810041M
chr5	96650902	96651100	intron (NM	MLT1H|LTI	11867	NM_05315	94061	Mrpl1	2410002L0
chr1	64564902	64565525	Intergenic	MLT1I|LTR	-14165	NM_13382	12912	Creb1	2310001E1
chr6	1.13E+08	1.13E+08	promoter-T	promoter-T	-925	NM_13393	171508	Creld1	AI843811
chr7	1.07E+08	1.07E+08	intron (NM	CpG	209	NM_13369	67967	Pold3	2410142G1
chr6	1.21E+08	1.21E+08	Intergenic	Intergenic	-10187	NM_01737	53857	Tuba8	-
chr4	1.34E+08	1.34E+08	intron (NR_	CpG	140	NM_02625	67586	Ubxn11	4930506L0
chr2	1.55E+08	1.55E+08	promoter-T	promoter-T	-466	NM_01981	60525	Acss2	1110017C1
chr19	53269352	53269525	intron (NM	intron (NM	50951	NM_01375	27360	Add3	AI463285|
chr19	5295027	5295200	intron (NM	intron (NM	342	NM_03010	319322	Sf3b2	145kDa|26
chr14	61368727	61368925	exon (NM_	exon (NM_	-18145	NM_18317	239126	C1qtnf9	9130217G2
chr12	1.12E+08	1.12E+08	intron (NM	CpG	343	NM_17520	74251	Ankrd9	2500003O2

chr5	1.24E+08	1.24E+08	promoter-T	promoter-T	-260	NM_00113	208908	Ccdc62	AI661708|(
chr15	91002627	91002900	intron (NM	intron (NM	19475	NM_01199	26874	Abcd2	ABC39|ALD
chr15	91463502	91463950	Intergenic	Intergenic	-39929	NM_02573	66725	Lrrk2	4921513O2
chr2	1.28E+08	1.28E+08	promoter-T	promoter-T	-224	NM_00129	53885	Nphp1	-
chr3	1.09E+08	1.09E+08	intron (NM	intron (NM	30409	NM_17268	229731	Slc25a24	2610016M
chr18	24811727	24812100	promoter-T	promoter-T	-279	NM_02677	68591	Mocos	1110018O1
chrX	1.04E+08	1.04E+08	intron (NM	intron (NM	1284	NM_17544	213438	A630033H2	AI662791|I
chr10	34958902	34959050	Intergenic	Intergenic	755770	NM_01023	14302	Frk	BSK|BSK/IY
chr7	52112702	52112800	promoter-T	promoter-T	-22	NM_00129	59047	Pnkp	1810009G0
chr6	1.29E+08	1.29E+08	Intergenic	LTRIS4|LTR	19770	NM_15350	232409	Clec2e	Clra|clr-a
chr11	1.03E+08	1.03E+08	intron (NM	intron (NM	830	NM_18328	544817	Arhgap27	2310069I04
chr3	95303677	95303850	intron (NM	intron (NM	631	NM_00780	13038	Ctsk	AI323530|I
chr18	74938552	74938675	promoter-T	promoter-T	-253	NM_17747	52538	Acaa2	0610011L0
chr11	1.04E+08	1.04E+08	intron (NM	intron (NM	85738	NM_00128	17762	Mapt	AI413597|A
chr17	74926977	74927250	promoter-T	promoter-T	-522	NM_00756	12211	Birc6	A430032G0
chr3	79395327	79395475	promoter-T	promoter-T	90	NM_02635	67738	Ppid	4930564J0
chr13	3729402	3729725	Intergenic	Intergenic	74001	NM_02741	70405	Calml3	2310068O2
chr12	16129727	16129800	Intergenic	Intergenic	-306172	NM_14455	217410	Trib2	AW319517
chr6	91567652	91567925	Intergenic	Intergenic	-66273	NM_00932	21366	Slc6a6	AA589629|
chr15	57721127	57721200	intron (NM	intron (NM	2810	NM_02420	67819	Derl1	1110021N0
chr7	26473052	26473150	exon (NM_	exon (NM_	1080	NM_01157	21803	Tgfb1	TGF-beta1|
chr1	1.41E+08	1.41E+08	Intergenic	Intergenic	-51298	NM_00116	329260	Dennd1b	4632404N1
chr13	90028752	90029025	intron (NM	intron (NM	-146771	NM_00113	13003	Vcan	5430420N0
chr1	1.84E+08	1.84E+08	Intergenic	Intergenic	-16155	NM_01205	27058	Srp9	9kDa
chr2	1.73E+08	1.73E+08	TTS (NM_0	TTS (NM_0	-5078	NM_00113	58203	Zbp1	2010010H0
chr2	1.8E+08	1.8E+08	promoter-T	promoter-T	76	NM_03137	72075	Ogfr	2010013E1
chr7	88138527	88138725	promoter-T	promoter-T	56	NM_17870	233410	Zfp592	8430405N2
chr15	78744252	78744575	promoter-T	promoter-T	64	NM_02027	57028	Pdxp	1600027H0
chr5	23958527	23958600	intron (NM	intron (NM	568	NM_00125	213990	Agap3	AGAP-3|AV
chr2	1.56E+08	1.56E+08	promoter-T	promoter-T	-393	NM_00112	16328	Cep250	AW490617
chr12	74788527	74788625	intron (NM	intron (NM	102548	NM_00885	18755	Prkch	Pkch
chr11	5409952	5410100	Intergenic	Intergenic	-10618	NM_01384	22433	Xbp1	D11Ertd39
chr7	1.17E+08	1.17E+08	promoter-T	promoter-T	-61	NM_02005	56786	Tmem9b	2310004K0
chr1	59819727	59820150	Intergenic	CpG	-1185	NM_00756	12168	Bmpr2	2610024H2
chr5	43434302	43434500	Intergenic	MIR|SINE|	-190301	NM_17593	231207	Cpeb2	A630055H1
chr14	70612402	70612500	Intergenic	Intergenic	-4973	NM_01136	20410	Sorbs3	SCAM-1|SH
chr7	1.08E+08	1.08E+08	TTS (NM_0	TTS (NM_0	-5499	NM_17707	320100	Relt	E430021K2
chr9	1.07E+08	1.07E+08	Intergenic	Intergenic	11368	NM_00989	12700	Cish	AI385595|(
chr1	36621102	36621250	exon (NM_	exon (NM_	-5950	NM_00112	20353	Sema4c	AI426163|S
chr1	75138027	75138350	intron (NM	CpG	754	NM_02697	69171	Cnppd1	1810031K1
chr2	73052077	73052300	intron (NM	CpG	316	NM_03009	67059	Ola1	2510025G0
chr7	1.09E+08	1.09E+08	promoter-T	promoter-T	-256	NM_01956	56212	Rhog	2810426G0
chr4	46466627	46466750	intron (NM	intron (NM	2699	NM_13088	67628	Anp32b	2410015B1
chr15	38008302	38008375	5' UTR (NM	5' UTR (NM	271	NM_00111	70790	Ubr5	Edd|Edd1
chr7	1.35E+08	1.35E+08	Intergenic	Intergenic	21159	NM_00107	434246	Trim72	BC067209|
chr17	28656252	28656425	Intergenic	Intergenic	-11468	NM_02629	67645	Armc12	4930511I1
chr7	88491577	88491725	TTS (NM_0	TTS (NM_0	-1531	NM_00909	20068	Rps17	-

chr10	1.16E+08	1.16E+08	intron (NM	Lx8|LINE|L	-7653	NM_00116	619332	4933416C0	-
chr19	43785602	43786025	intron (NM	intron (NM	21634	NM_05310	93685	Entpd7	1810012B1
chr12	55797702	55797800	promoter-T	promoter-T	-899	NM_02545	66266	Eapp	1810011O1
chr7	54263277	54263900	5' UTR (NM	5' UTR (NM	196	NM_17531	101685	Spty2d1	5830435K1
chr4	1.34E+08	1.34E+08	intron (NM	intron (NM	7631	NM_14555	100017	Ldlrap1	AA691260|
chr10	98695152	98695400	Intergenic	Intergenic	-30589	NM_02626	67603	Dusp6	1300019I0
chr6	7643102	7643200	promoter-T	promoter-T	31	NM_01205	27053	Asns	-
chr2	1.25E+08	1.25E+08	promoter-T	promoter-T	-17	NM_01085	17876	Myef2	9430071B0
chr14	77434477	77434575	intron (NM	intron (NM	1898	NM_17248	210808	Lacc1	9030625A0
chr13	75090077	75090250	Intergenic	Lx8|LINE|L	-137272	NM_01362	18548	Pcsk1	Nec-1|Nec
chr2	1.53E+08	1.53E+08	Intergenic	Lx8|LINE|L	-7351	NM_00844	16569	Kif3b	AI854312|
chr2	26300352	26300575	intron (NM	CpG	273	NM_15312	227648	Sec16a	AU024582
chr13	24927177	24927375	intron (NM	intron (NM	3748	NM_01955	56196	Tdp2	D13Ertd65
chr7	1.48E+08	1.48E+08	TTS (NM_0	TTS (NM_0	2919	NM_01874	54399	Bet1l	2610021K2
chr11	58717552	58717625	intron (NM	CpG	139	NM_01175	22698	Zfp39	CTfin33|Zf
chr12	1.12E+08	1.12E+08	intron (NM	Lx9|LINE|L	26545	NM_01163	22031	Traf3	AI528849|
chr14	76510377	76510550	promoter-T	promoter-T	159	NM_02617	67467	Gpalpp1	1200011I1
chr1	1.64E+08	1.64E+08	Intergenic	Intergenic	-5671	NM_01679	53330	Vamp4	D1Ertd147
chr10	83875177	83875375	intron (NM	MIRb|SINE	27940	NM_00100	77976	Nuak1	AU014801
chr7	4817952	4818225	promoter-T	promoter-T	-307	NM_02615	67441	Isoc2b	0610042E0
chr14	51875802	51876025	promoter-T	promoter-T	-126	NM_05311	93726	Rnase2a	Ear11
chr13	9763077	9763150	intron (NM	CpG	1447	NM_00119	66505	Zmynd11	2210402G2
chr10	1.28E+08	1.28E+08	intron (NM	CpG	4312	NM_05407	116848	Baz2a	AA415431|
chr15	98783177	98783300	intron (NM	intron (NM	694	NM_01165	22142	Tuba1a	Tuba-1|Tu
chr1	1.33E+08	1.33E+08	promoter-T	promoter-T	-118	NM_00855	17164	Mapkapk2	AA960234|
chr13	94766252	94766625	Intergenic	Intergenic	-61313	NM_17258	218454	Lhfpl2	6030465B1
chr17	31194852	31195000	intron (NM	CpG	287	NM_00959	11307	Abcg1	AW413978
chr10	62040952	62041175	exon (NM_	exon (NM_	142	NM_02819	72320	2510003E0	0710007C1
chrY	1769277	1769350	Intergenic	Intergenic	-85696	NM_00111	1E+08	Gm16501	ENSMUSG0
chr6	3515052	3515225	intron (NM	intron (NM	66745	NM_00116	73288	Ccdc132	1700034M
chr13	49437302	49437500	intron (NM	intron (NM	483	NM_00103	76895	Bicd2	-
chr10	79861902	79862150	5' UTR (NM	5' UTR (NM	198	NM_01359	17192	Mbd3	AI181826|
chr16	84795102	84795250	intron (NM	MTB_Mm|	20808	NM_02384	67374	Jam2	1110002N2
chr13	41596127	41596425	Intergenic	Intergenic	-13547	NM_00111	18003	Nedd9	Cas-L|CasL
chr5	1.41E+08	1.41E+08	exon (NM_	exon (NM_	117	NM_13391	27979	Eif3b	AL033316|
chr7	31016977	31017325	promoter-T	promoter-T	58	NM_02725	69920	Polr2i	2810002B1
chr11	59039677	59039800	intron (NM	(A)n|Simpl	1926	NM_00113	11840	Arf1	-
chr5	1.37E+08	1.37E+08	Intergenic	Intergenic	-1012	NM_17875	269717	Orai2	A730041O1
chr11	1.05E+08	1.05E+08	exon (NM_	exon (NM_	149	NM_17256	52686	Mettl2	2810438F0
chr9	57148702	57148900	Intergenic	Intergenic	-38395	NM_02882	74211	1700017B0	AA682102|
chr1	1.82E+08	1.82E+08	promoter-T	promoter-T	-61	NM_17865	109232	Sccpdh	AW214504
chr2	1.05E+08	1.05E+08	intron (NM	CpG	133	NM_14538	98221	Eif3m	Ga17|Pcid
chr9	44869752	44869850	intron (NM	intron (NM	6537	NM_00109	319742	Mpzl3	5430427F1
chr7	20093677	20093800	promoter-T	promoter-T	-58	NM_17769	232946	Bloc1s3	BC043666|
chr5	1.09E+08	1.09E+08	promoter-T	promoter-T	533	NM_15356	231580	Gak	D130045N
chr13	81850027	81850375	promoter-T	promoter-T	-189	NM_00108	67486	Polr3g	2310047G2
chr11	75040877	75041100	intron (NM	intron (NM	33493	NM_17770	237847	Rtn4rl1	Ngr3|Ngrh

chr18	77713977	77714075	intron (NM	intron (NM	89849	NM_00116	225743	Rnf165	2900024M
chr9	89599377	89599825	exon (NM_	exon (NM_	280	NM_02536	66111	Tmed3	1200002G1
chr5	1.36E+08	1.36E+08	promoter-T	promoter-T	-160	NM_14541	100609	Nsun5	9830109N1
chr7	59384652	59384725	Intergenic	RMER16-in	-113602	NM_00119	1.01E+08	1700015G1	Ccdc179
chr6	1.01E+08	1.01E+08	intron (NM	intron (NM	26275	NM_18159	72171	Shq1	2810403P1
chr11	17759477	17759600	Intergenic	Intergenic	94340	NM_02657	68145	Etaa1	5730466H2
chr9	1.06E+08	1.06E+08	intron (NM	intron (NM	7627	NM_17292	245026	Col6a6	E330019B1
chr8	1.19E+08	1.19E+08	promoter-T	promoter-T	-622	NM_02944	75796	Cdyl2	1700029M
chr6	1.08E+08	1.08E+08	intron (NM	intron (NM	811	NM_01058	16438	Itpr1	D6Pas2|EN
chr3	95558677	95559000	promoter-T	promoter-T	62	NM_02793	71807	Tars2	2610024N0
chr1	1.46E+08	1.46E+08	promoter-T	promoter-T	155	NM_01956	56207	Uchl5	5830413B1
chr3	1.35E+08	1.35E+08	intron (NM	intron (NM	88848	NM_00868	18033	Nfkb1	NF-KB1|NF
chr7	1.36E+08	1.36E+08	intron (NM	intron (NM	25910	NM_02641	67865	Rgs10	2310010N1
chr12	1.05E+08	1.05E+08	intron (NM	intron (NM	752	NM_19931	380780	Serpina11	Gm895
chr7	1.34E+08	1.34E+08	Intergenic	(TG)n|Simp	-1477	NM_14563	246779	Il27	IL-27|IL-27
chr15	8461977	8462125	Intergenic	ORR1E|LTF	-67588	NM_20123	71175	Nipbl	Idn3
chr13	1.05E+08	1.05E+08	Intergenic	CpG	-9926	NM_17259	218543	Srek1	8430401B0
chr11	97564652	97564900	promoter-T	promoter-T	28	NM_01197	26446	Psmb3	AL033320|
chr11	1.11E+08	1.11E+08	Intergenic	Intergenic	-270859	NM_01060	16517	Kcnj16	6430410F1
chr1	93310727	93310850	promoter-T	promoter-T	-989	NM_01947	55927	Hes6	AI326893|I
chr7	55987952	55988075	Intergenic	L1_Mur2|L	-56360	NM_02803	243983	Zdhhc13	2410004E0
chr12	1.13E+08	1.13E+08	5' UTR (NM	5' UTR (NM	140	NM_01162	21981	Ppp1r13b	AI449786|A
chr5	1.31E+08	1.31E+08	promoter-T	promoter-T	319	NM_02324	66711	Sbds	4733401P1
chr13	29947302	29947575	promoter-T	promoter-T	19	NM_14453	68916	Cdkal1	1190005B0
chr10	59715402	59715675	Intergenic	Intergenic	-24838	NM_00114	19156	Psap	AI037048|S
chr5	1.07E+08	1.07E+08	5' UTR (NM	5' UTR (NM	246	NM_00127	12545	Cdc7	AI597260|C
chr4	1.41E+08	1.41E+08	3' UTR (NM	3' UTR (NM	-12352	NM_00103	76701	Ctrc	1810044E1
chr4	1.41E+08	1.41E+08	promoter-T	promoter-T	-415	NM_14598	213989	Tmem82	-
chr5	1.01E+08	1.01E+08	exon (NM_	exon (NM_	174	NM_02797	71883	Coq2	2310002F1
chr17	34973702	34973850	promoter-T	promoter-T	72	NM_01944	54402	Stk19	G11|RP1
chr12	56488502	56488650	Intergenic	URR1A|DN	-11236	NM_01196	26443	Psma6	IOTA
chr4	1.11E+08	1.11E+08	intron (NM	intron (NM	-412598	NR_03379	67621	Bend5	2310026E2
chr8	75247427	75247575	intron (NM	CpG	332	NM_00918	20467	Sin3b	2810430C1
chr10	80260802	80260975	promoter-T	promoter-T	483	NM_02390	78670	Plekhj1	9530063M
chr6	83059052	83059250	promoter-T	promoter-T	49	NM_02674	68499	Mrpl53	1110007K1
chr6	1.46E+08	1.46E+08	intron (NM	intron (NM	236496	NM_01065	16651	Sspn	Krag
chr6	86475777	86475850	exon (NM_	exon (NM_	346	NM_01186	23983	Pcbp1	WBP17|[a]
chr11	1.18E+08	1.18E+08	intron (NM	CpG	480	NM_13375	74451	Pgs1	2610019F1
chr8	37382627	37382750	Intergenic	RMER4B|LT	-70118	NM_00108	244421	Lonrf1	-
chr8	47710677	47710825	intron (NM	L1MC1|LIN	8106	NM_00981	12367	Casp3	A830040C1
chr5	1.05E+08	1.05E+08	Intergenic	Intergenic	-27067	NM_00120	20750	Spp1	2AR|Apl-1
chr12	82961277	82961350	5' UTR (NM	5' UTR (NM	296	NM_01881	54604	Pcnx	2900024E2
chr10	19661052	19661250	intron (NM	intron (NM	6819	NM_00858	26408	Map3k5	7420452D2
chr3	1.08E+08	1.08E+08	5' UTR (NM	5' UTR (NM	386	NM_00111	12977	Csf1	C87615|Cs
chr7	73357752	73357925	Intergenic	Intergenic	103437	NM_00108	269941	Chsy1	mKIAA0990
chr19	7131827	7131925	intron (NM	intron (NM	618	NM_13414	107227	Macrod1	AI604841|A
chr2	1.19E+08	1.19E+08	Intergenic	L1MA9|LIN	34043	NM_02811	72136	Chst14	2600016L0

chr14	67583002	67583450	Intergenic	Intergenic	44488	NM_00976	12177	Bnip3l	C86132|D1
chr6	1.34E+08	1.34E+08	intron (NM	intron (NM	18391	NM_02577	66813	Bcl2l14	4930452K2
chr1	79249502	79249725	Intergenic	Intergenic	187052	NM_00912	20254	Scg2	Chgc|SgII
chr10	80568377	80568600	promoter-T	promoter-T	-204	NM_02313	26396	Map2k2	AA589381|
chr7	35925002	35925125	Intergenic	Intergenic	20751	NM_00767	12606	Cebpa	C/ebpalpha
chr12	17516777	17517050	Intergenic	Intergenic	-34766	NM_01361	18263	Odc1	ODC
chr1	79736302	79736525	intron (NM	intron (NM	21931	NM_00111	69368	Wdfy1	1700013B0
chr2	1.02E+08	1.02E+08	Intergenic	Intergenic	-17393	NM_02963	76501	Commd9	1810029F0
chr18	7631577	7632000	Intergenic	Intergenic	-4927	NM_00116	75739	Mpp7	1110068J0
chr12	1.06E+08	1.06E+08	intron (NM	CpG	286	NM_14894	192119	Dicer1	1110006F0
chr19	6400302	6400375	promoter-T	promoter-T	-245	NM_01124	19395	Rasgrp2	CDC25L|Ca
chr3	51916077	51916150	Intergenic	B4A|SINE|	-7185	NM_00100	433586	Maml3	AV234550|
chr15	89204127	89204325	promoter-T	promoter-T	23	NM_00111	1E+08	Sco2	-
chr4	1.43E+08	1.43E+08	Intergenic	Intergenic	-26464	NM_00125	110593	Prdm2	4833427P1
chr5	77615102	77615400	Intergenic	ID2|SINE|I	24979	NM_00128	69982	Spink2	1700007F2
chr11	1.09E+08	1.09E+08	Intergenic	Intergenic	-5685	NM_14594	52639	Wipi1	4930533H0
chr7	1.03E+08	1.03E+08	Intergenic	Intergenic	-39159	NM_01185	23966	Tenm4	Doc4|ELM2
chr5	1.25E+08	1.25E+08	intron (NM	CpG	338	NM_01977	56334	Tmed2	1110032D1
chr1	1.4E+08	1.4E+08	intron (NM	intron (NM	38665	NM_00111	19264	Ptprc	B220|CD45
chr15	36297027	36297300	Intergenic	ORR1E|LTR	-84261	NM_01392	30945	Rnf19a	AA032313|
chr1	93262977	93263300	exon (NM_	exon (NM_	131	NM_17339	227358	Fam132b	4832406C2
chr7	1.36E+08	1.36E+08	promoter-T	promoter-T	-499	NM_00938	21843	Tial1	5330433G1
chr10	68674252	68674425	intron (NM	intron (NM	-1067	NM_00108	69288	Rhobtb1	1700008H1
chrX	1.4E+08	1.4E+08	Intergenic	Intergenic	-33089	NM_00111	13193	Dcx	Dbct
chr6	58590777	58590875	intron (NM	intron (NM	44160	NM_01192	26357	Abcg2	ABC15|AB0
chr9	1.1E+08	1.1E+08	intron (NM	intron (NM	2376	NM_00869	18054	Ngp	bectenecin
chr7	1.34E+08	1.34E+08	non-coding	non-coding	510	NM_02371	73658	Spns1	2210013K0
chr11	86571427	86571550	promoter-T	promoter-T	-494	NM_00100	67300	Cltc	3110065L2
chr8	11016152	11016425	intron (NR_	intron (NR_	-7858	NM_00108	384783	Irs2	Irs-2
chr11	1.01E+08	1.01E+08	exon (NM_	exon (NM_	130	NM_01379	27419	Naglu	-
chr9	1.03E+08	1.03E+08	intron (NM	CpG	154	NM_01976	56332	Amotl2	AW549739
chr14	32248552	32248800	intron (NM	CpG	543	NM_01189	24056	Sh3bp5	AI606498|S
chr7	1.25E+08	1.25E+08	intron (NM	intron (NM	1673	NM_17066	267019	Rps15a	A630031B1
chr5	1.18E+08	1.18E+08	5' UTR (NM	5' UTR (NM	113	NM_00119	330177	Taok3	2900006A0
chr2	28257252	28257500	Intergenic	Intergenic	-46085	NM_00100	241289	Ppp1r26	Gm347|Kia
chr14	51667602	51667725	Intergenic	MTE2a|LTR	2175	NM_00101	497113	Rnase11	Raj1
chr5	77563902	77563975	Intergenic	Intergenic	-19790	NM_17560	74318	Hopx	1110018K1
chr4	1.26E+08	1.26E+08	intron (NM	intron (NM	9079	NM_17270	230751	Oscp1	1810007P1
chr1	1.72E+08	1.72E+08	intron (NM	intron (NM	27794	NM_00906	19737	Rgs5	1110070A0
chr12	1.04E+08	1.04E+08	intron (NM	intron (NM	712	NM_00116	217835	Rin3	6430500K0
chr10	84959802	84960000	intron (NM	MIR|SINE|	-101255	NM_00101	74007	Btbd11	6330404E1
chr5	29761202	29761650	exon (NM_	exon (NM_	219	NM_00103	433864	Nom1	D5Kng1|Gr
chr13	63978552	63978675	intron (NM	intron (NM	61971	NM_02350	76251	Ercc6l2	0610007P0
chr11	58064027	58064275	Intergenic	L1MA5A|LI	35672	NM_01944	54396	Irgm2	AI481100|
chr3	1.17E+08	1.17E+08	Intergenic	Intergenic	-21154	NM_00108	77559	Agl	1110061O1
chr19	36807752	36807975	3' UTR (NM	3' UTR (NM	3231	NM_01685	53412	Ppp1r3c	PTG|Ppp1r
chr1	16560902	16561200	non-coding	non-coding	48368	NM_02577	66799	Ube2w	6130401J0

chr6	1.25E+08	1.25E+08	intron (NM	intron (NM	427	NM_01370	22225	Usp5	AA407472|
chr7	88357502	88357650	Intergenic	MIRc|SINE	-1114	NM_00880	18584	Pde8a	AI551852|
chr1	79855352	79855525	promoter-T	promoter-T	-198	NM_00925	20720	Serpine2	B230326M
chr9	75190202	75190625	intron (NR_	intron (NR_	-5152	NM_01347	12049	Bcl2l10	AA420380|
chr3	90165452	90165550	Intergenic	(A)n|Simpl	19925	NM_13930	229542	Gatad2b	AL118180|
chr9	1.11E+08	1.11E+08	Intergenic	CpG	-16501	NM_15309	235628	Prss42	Tessp2
chrX	53851127	53851325	promoter-T	promoter-T	-130	NM_14623	236792	Mmgt1	9630048L0
chr4	35104302	35104400	intron (NM	CpG	382	NM_17806	214944	Mob3b	8430436F2
chr13	1.05E+08	1.05E+08	promoter-T	promoter-T	8	NM_02179	60411	Cenpk	B130045K2
chr11	72838202	72838375	intron (NM	intron (NM	5778	NM_01888	55984	Camkk1	AI846603|
chr1	1.69E+08	1.69E+08	intron (NM	intron (NM	36420	NM_03072	80914	Uck2	AA407809|
chr5	1.15E+08	1.15E+08	exon (NM_	exon (NM_	286	NM_01167	22256	Ung	UNG1|UNG
chr1	40087227	40087375	Intergenic	Intergenic	-54312	NM_01055	16178	Il1r2	CD121b|Il1
chr5	92938027	92938175	Intergenic	Intergenic	-3467	NM_00764	12492	Scarb2	9330185J1
chr1	92951202	92951300	intron (NM	intron (NM	1230	NM_00851	16978	Lrrfip1	AU024550
chr18	36178227	36178525	intron (NM	intron (NM	53892	NR_11034	74002	Psd2	6330404E2
chr4	1.55E+08	1.55E+08	promoter-T	promoter-T	661	NM_08044	117592	B3galt6	BB129894|
chr10	12695877	12696125	Intergenic	Intergenic	-11936	NM_00116	74732	Stx11	5830405C0
chr7	75081852	75081975	Intergenic	Tigger9a|D	-15230	NM_01051	16001	Igf1r	A330103N
chr5	1.01E+08	1.01E+08	Intergenic	Intergenic	-2839	NM_13919	231507	Plac8	C15|D5Ws
chr10	38550152	38550275	Intergenic	RMER16-in	-8628	NM_00117	215919	Rfpl4b	100040915
chr7	1.38E+08	1.38E+08	Intergenic	Intergenic	57905	NM_00841	16433	Cuzd1	Erg-1|Itma
chr1	1.95E+08	1.95E+08	5' UTR (NM	5' UTR (NM	189	NM_15313	215243	Traf3ip3	6030423D0
chr15	80086077	80086825	intron (NM	CpG	837	NM_00971	11911	Atf4	Atf-4|C/AT
chr7	86040877	86041050	intron (NM	CpG	150	NM_00116	68048	Aen	2700083B0
chr7	1.5E+08	1.5E+08	Intergenic	Intergenic	-14994	NM_00128	55925	Syt8	-
chr19	4200752	4201750	intron (NM	intron (NM	352	NM_01123	19367	Rad9a	Rad9
chr15	9607902	9607975	intron (NM	intron (NM	70623	NM_17712	320277	Spef2	C230086A0
chr1	1.8E+08	1.8E+08	Intergenic	Intergenic	-36743	NM_00116	269152	Kif26b	4832420M
chr11	99005002	99005350	TTS (NM_0	TTS (NM_0	11215	NM_00771	12775	Ccr7	CD197|Cdw
chr3	1.38E+08	1.38E+08	promoter-T	promoter-T	149	NM_17538	108943	Trmt10a	3110023L0
chr7	1.18E+08	1.18E+08	intron (NM	CpG	631	NM_00104	13690	Eif4g2	AA589388|
chr1	1.45E+08	1.45E+08	Intergenic	Intergenic	-925851	NM_02002	26878	B3galt2	-
chr1	97559727	97559825	intron (NM	intron (NM	4395	NM_00918	20452	St8sia4	PST|PST-1|
chr2	1.49E+08	1.49E+08	exon (NM_	exon (NM_	3670	NM_02898	74533	Gzf1	8430437G0
chr4	1.55E+08	1.55E+08	3' UTR (NM	3' UTR (NM	4394	NM_14777	246228	Vwa1	4932416A1
chr1	1.74E+08	1.74E+08	intron (NM	intron (NM	1330	NM_15355	98193	Dcaf8	AA408877|
chr15	66581677	66581900	intron (NM	intron (NM	79456	NM_00937	21819	Tg	Tgn|cog
chr4	1.16E+08	1.16E+08	promoter-T	promoter-T	-956	NM_18158	18710	Pik3r3	AA414954|
chr5	1.14E+08	1.14E+08	intron (NM	intron (NM	747	NM_00101	231630	Ficd	D5Ertd40e
chr1	37356927	37357025	intron (NM	CpG	293	NM_00129	269180	Inpp4a	107kDa|96
chr17	35186802	35186975	promoter-T	promoter-T	-300	NM_03344	114584	Clic1	Clcp|G6
chr16	33751877	33752175	intron (NM	intron (NM	-42097	NM_01073	17063	Muc13	114/A10|1
chr1	1.62E+08	1.62E+08	intron (NM	CpG	110	NM_02547	64659	Mrps14	1810032L2
chr8	93432927	93433100	intron (NM	CpG	80279	NM_17722	109151	Chd9	1810014J1
chr1	40285902	40286150	intron (NM	intron (NM	4101	NM_00836	16177	Il1r1	CD121a|CD
chr15	66932852	66932975	Intergenic	Intergenic	75531	NM_00917	20442	St3gal1	5330418N2

chr3	94386077	94386175	intron (NM	CpG	512	NM_00108	76742	Snx27	5730552M
chr2	72313677	72313775	promoter-T	promoter-T	-550	NM_02586	66953	Cdca7	2310021G(
chr7	27038477	27038725	Intergenic	Intergenic	-13249	NM_00116	1E+08	Vmn1r184	-
chr4	91038827	91038950	promoter-T	promoter-T	-142	NM_20768	15569	Elavl2	Hub|mel-N
chr1	1.83E+08	1.83E+08	Intergenic	Intergenic	36096	NM_00821	15078	H3f3a	H3.3A
chr14	21665052	21665300	Intergenic	Intergenic	9292	NM_00887	18792	Plau	u-PA|uPA
chr5	35445902	35446125	intron (NM	intron (NM	2333	NM_01358	16976	Lrpap1	AA617339|
chr2	45134927	45135250	Intergenic	Intergenic	-166289	NM_01575	24136	Zeb2	9130203F0
chr12	1.13E+08	1.13E+08	intron (NM	CpG-3534	826	NM_17336	217869	Eif5	2810011H2
chr9	26730327	26731175	promoter-T	promoter-T	-85	NM_00128	382064	Gm1110	-
chrX	1.03E+08	1.03E+08	intron (NM	CpG	348	NM_00953	22589	Atrx	4833408C1
chr6	1.37E+08	1.37E+08	Intergenic	Intergenic	-13299	NM_02580	66857	Plbd1	1100001H2
chr1	1.55E+08	1.55E+08	promoter-T	promoter-T	-101	NM_00103	71836	Shcbp1l	1700012A1
chr1	66910377	66910875	promoter-T	promoter-T	-743	NM_00738	11363	Acadl	AA960361|
chr2	1.05E+08	1.05E+08	intron (NM	intron (NM	1381	NM_17719	320554	Tcp11l1	C130096D(
chr2	1.44E+08	1.44E+08	promoter-T	promoter-T	270	NM_02422	69178	Snx5	0910001N(
chr2	56967552	56967700	promoter-T	promoter-T	-177	NM_01361	18227	Nr4a2	HZF-3|NOT
chr4	1.09E+08	1.09E+08	promoter-T	promoter-T	-47	NM_14615	230598	Nrd1	2600011I0(
chr7	1.07E+08	1.07E+08	Intergenic	L1MA4|LIN	15214	NM_14635	258353	Olfr521	MOR101-2
chr19	21105652	21105800	Intergenic	Intergenic	-77034	NM_02895	13409	Tmc1	4933416G(
chr14	58018302	58018425	Intergenic	Intergenic	-1043	NM_03000	68631	Cryl1	1110025H(
chr3	1.58E+08	1.58E+08	intron (NM	intron (NM	27251	NM_14595	107869	Cth	0610010I1:
chr1	1.9E+08	1.9E+08	intron (NM	intron (NM	-92818	NM_01193	26381	Esrrg	ERR3|Errg|
chr1	92938252	92938800	intron (NM	intron (NM	-11495	NM_00111	16978	Lrrfip1	AU024550
chr16	38678977	38679075	intron (NM	RCHARR1|I	34122	NM_02026	12549	Arhgap31	5830477L0
chr16	4726727	4726900	intron (NM	CpG	452	NM_00113	15369	Hmox2	HO-2|HO2
chr5	1.23E+08	1.23E+08	intron (NM	CpG	891	NM_00100	30841	Kdm2b	Cxxc2|E43(
chr12	70683377	70683475	intron (NM	intron (NM	589	NM_00103	207965	Vcpkmt	Gm71|Met
chr2	1.18E+08	1.18E+08	Intergenic	Intergenic	-97632	NM_01158	21825	Thbs1	TSP-1|TSP1
chr17	25270302	25270650	exon (NM_	exon (NM_	137	NM_01193	26373	Clcn7	AA409691|
chr9	44615727	44616500	exon (NM_	exon (NM_	-5079	NM_13895	192653	Ttc36	-
chr2	22666327	22666650	intron (NM	intron (NM	36641	NM_01945	54519	Apbb1ip	9930118P(
chr2	1.04E+08	1.04E+08	intron (NM	intron (NM	770	NM_21243	57443	Fbxo3	1200002G(
chr19	46379527	46379675	promoter-T	promoter-T	374	NM_00117	18034	Nfkb2	NF-kappaB
chr14	1.04E+08	1.04E+08	Intergenic	Intergenic	-75920	NM_00113	13618	Ednrb	ET-B|ET-BF
chr12	1.01E+08	1.01E+08	promoter-T	promoter-T	-113	NM_00979	12313	Calm1	AI256814|/
chr8	1.26E+08	1.26E+08	promoter-T	promoter-T	-557	NM_15317	234847	Spg7	AI452278|/
chr8	95430552	95430700	intron (NM	intron (NM	5332	NM_02711	69543	Capns2	2310005G(
chr18	24688202	24688325	intron (NM	CpG	442	NM_14486	225283	Rprd1a	C77387|mI
chr10	79389777	79390400	promoter-T	promoter-T	2	NM_00128	13496	Arid3a	Bright|Dri1
chr3	1.31E+08	1.31E+08	Intergenic	(TG)n|Simp	-7372	NM_02894	74442	Sgms2	4933405A1
chr11	79038277	79038375	Intergenic	Intergenic	18200	NM_00104	78889	Wsb1	1110056B1
chr1	1.38E+08	1.38E+08	Intergenic	Intergenic	-32106	NM_17343	215690	Nav1	9530089B1
chr12	71203427	71203600	intron (NM	intron (NM	328	NM_00108	18080	Nin	3110068G2
chr3	79090727	79090900	Intergenic	CpG	-58485	NM_00114	1E+08	Gm17359	-
chr18	77952552	77952875	promoter-T	promoter-T	36	NM_17867	212163	8030462N1	Akd2|Ark2
chr17	71202252	71202550	intron (NM	CpG	471	NM_00116	21815	Tgif1	AA959811|

chr11	97887902	97888000	promoter-T	promoter-T	-73	NM_00115	19921	Rpl19	-
chr1	9763177	9763350	Intergenic	Intergenic	-24800	NM_17344	70675	Vcpip1	4932442A0
chr9	65756852	65756975	promoter-T	promoter-T	96	NM_17318	214897	Csnk1g1	9130020E2
chr1	95288277	95288450	intron (NM	intron (NM	17890	NM_00127	404545	Ano7	Ngep|Ngep
chr10	42940802	42940900	promoter-T	promoter-T	-441	NM_02777	71365	Pdss2	5430420P0
chr3	95455602	95455825	Intergenic	PB1|SINE|/	-6930	NM_00856	17210	Mcl1	AW556805
chr11	77978452	77978525	intron (NM	CpG	564	NM_00942	22032	Traf4	A530032M
chr10	87865777	87866025	intron (NM	URR1A|DN	23744	NM_00100	432486	Gnptab	EG432486|
chr11	62352102	62352550	intron (NM	CpG	437	NM_02844	73139	Cenpv	3110013H0
chr3	82162277	82162375	intron (NM	CpG	332	NM_00108	213582	Map9	5033421J1
chr6	48036102	48036375	intron (NM	CpG	354	NM_00116	69228	Zfp746	2810407L0
chr2	57418477	57418800	Intergenic	Intergenic	327845	NM_01027	14571	Gpd2	AA408484|
chr14	31362427	31362825	intron (NM	CpG	309	NM_00938	21881	Tkt	p68
chr5	64731027	64731100	intron (NM	intron (NM	109467	NM_00128	57915	Tbc1d1	1110062G0
chr15	51823027	51823100	intron (NM	CpG	243	NM_00900	19357	Rad21	SCC1|mKIA
chr11	48669252	48669475	Intergenic	Intergenic	15485	NM_00832	15944	Irgm1	Ifggd3|Ifi1
chr6	1.42E+08	1.42E+08	promoter-T	promoter-T	-913	NM_00849	16832	Ldhb	AI790582|
chr2	1.29E+08	1.29E+08	Intergenic	Intergenic	40083	NM_00129	19261	Sirpa	AI835480|
chr4	86220777	86220850	promoter-T	promoter-T	-764	NM_17837	68441	Rraga	1300010C1
chr10	61300752	61301025	TTS (NM_0	TTS (NM_0	-54276	NM_20700	404634	H2afy2	macroH2A2
chr4	1.4E+08	1.4E+08	Intergenic	Intergenic	-1825	NM_17386	108911	Rcc2	2610510H0
chr8	64225952	64226125	intron (NM	intron (NM	12857	NM_00129	72333	Palld	2410003B1
chr4	1.48E+08	1.48E+08	intron (NM	CpG	327	NM_01669	50912	Exosc10	PM-Scl|PM
chr1	39538927	39539150	Intergenic	MLT1B|LTF	-3446	NM_01877	54610	Tbc1d8	AD3|HBLP1
chr18	75063627	75063750	Intergenic	RLTR24|LT	57229	NM_01072	16891	Lipg	3110013K0
chr17	43393727	43394000	Intergenic	ORR1E|LTF	-13433	NM_13377	77596	Gpr110	5031409J1
chr10	94848427	94848650	Intergenic	Intergenic	29379	NM_00116	216233	Socs2	8030460M
chr9	44688677	44688975	intron (NM	intron (NM	531	NM_00108	214162	Kmt2a	6430520K0
chr8	1.29E+08	1.29E+08	Intergenic	Intergenic	-2102	NM_00116	270110	Irf2bp2	E130305N2
chr8	1.12E+08	1.12E+08	promoter-T	promoter-T	-29	NM_01737	15439	Hp	HP-1|preH
chr10	79711727	79712175	promoter-T	promoter-T	-246	NM_02927	75406	Ndufs7	1010001M
chr2	35056952	35057025	promoter-T	promoter-T	-348	NM_02669	68365	Rab14	0610030G2
chr8	41947727	41947925	5' UTR (NM	5' UTR (NM	105	NM_00104	11988	Slc7a2	20.5|AI158
chr2	1.65E+08	1.65E+08	exon (NM_	exon (NM_	113	NM_00100	381406	2810408M	-
chr6	1.24E+08	1.24E+08	intron (NM	intron (NM	13864	NM_15350	232370	Clstn3	CSTN3|Cs3
chr17	15080727	15080850	promoter-T	promoter-T	599	NM_02645	67912	1600012H0	-
chr2	1.3E+08	1.3E+08	promoter-T	promoter-T	303	NM_02419	67134	Nop56	2310044F1
chr2	1.05E+08	1.05E+08	intron (NM	intron (NM	10370	NM_14480	211896	Depdc7	AV216087
chr4	1.33E+08	1.33E+08	Intergenic	Intergenic	-2325	NM_00108	93760	Arid1a	1110030E0
chr11	1.2E+08	1.2E+08	intron (NM	intron (NM	48702	NM_19842	268515	Bahcc1	B930044J0
chr11	1.01E+08	1.01E+08	promoter-T	promoter-T	-419	NM_02445	19345	Rab5c	AI326010|
chr4	10845602	10845900	Intergenic	Intergenic	44106	NM_02600	67157	2610301B2	AI428449
chr1	58859077	58859925	5' UTR (NM	5' UTR (NM	155	NM_00108	12370	Casp8	CASP-8|FLI
chr2	1.63E+08	1.63E+08	intron (NM	intron (NM	468	NM_17215	245866	Ift52	BC037708|
chr12	86452052	86452125	intron (NM	CpG	305	NM_03022	78920	Dlst	1600017E0
chr3	60806777	60806925	5' UTR (NM	5' UTR (NM	144	NM_00128	18441	P2ry1	P2Y1
chr9	1.1E+08	1.1E+08	exon (NM_	exon (NM_	135	NM_00921	20588	Smarcc1	AI115498|

chr5	1.19E+08	1.19E+08	exon (NM_	exon (NM_	104	NM_17272	231672	Fbxw8	4930438M
chr1	1.35E+08	1.35E+08	promoter-T	promoter-T	-113	NM_00109	240752	Pik3c2b	C330011J1
chr12	77478577	77478700	intron (NM	URR1A|DN	7385	NM_17874	268564	Zbtb1	9430077A1
chr11	31571627	31571875	5' UTR (NM	5' UTR (NM	111	NM_00102	69556	Bod1	2310022M
chr17	56812802	56813000	intron (NR_	CpG	253	NM_02793	71810	Ranbp3	2610024N2
chr1	43991277	43991425	intron (NM	CpG	499	NM_00941	22019	Tpp2	TPP-2|Tpp
chr17	32176702	32177100	intron (NM	intron (NM	3794	NM_02824	72462	Rrp1b	2600005C2
chr6	1.47E+08	1.47E+08	3' UTR (NM	3' UTR (NM	-28764	NM_17230	272322	Arntl2	4632430A0
chr5	20694427	20694500	intron (NM	intron (NM	2378	NM_17543	212167	Gsap	A530088I0
chr3	66789752	66789875	intron (NM	CpG	219	NM_02582	66880	Rsrc1	1200013F2
chr8	13159277	13159375	5' UTR (NM	5' UTR (NM	191	NM_01068	16783	Lamp1	AI196048|
chr7	13608502	13608875	promoter-T	promoter-T	-813	NM_01158	21849	Trim28	AA408787|
chr4	1.37E+08	1.37E+08	promoter-T	promoter-T	22	NM_19824	230848	Zbtb40	BC059177|
chr4	6379677	6379875	intron (NM	intron (NM	1642	NM_01094	18201	Nsmaf	AA959567|
chr17	34088102	34088225	TTS (NM_0	TTS (NM_0	270	NM_01942	54218	B3galt4	Gal-T2|Gal
chr5	1.37E+08	1.37E+08	Intergenic	Lx6|LINE|L	-8613	NM_01196	26433	Plod3	AI414586|
chr13	56354802	56354950	Intergenic	Intergenic	-1352	NM_01089	18014	Neurog1	AKA|Math
chr10	61968702	61968775	intron (NM	intron (NM	1765	NM_01115	19073	Srgn	Prg|Prg1|S
chr9	63585777	63586025	intron (NM	MTEa|LTR|	19900	NM_01676	17127	Smad3	AU022421
chr8	1.24E+08	1.24E+08	intron (NM	intron (NM	8305	NM_13376	76454	Fbxo31	1110003O0
chr15	73939977	73940175	Intergenic	Intergenic	-269975	NM_00103	268816	Mroh5	Gm628
chr8	1.21E+08	1.21E+08	intron (NM	intron (NM	345683	NM_01970	12554	Cdh13	4932416G0
chr5	1.23E+08	1.23E+08	intron (NM	intron (NM	8643	NM_00119	207565	Camkk2	6330570N1
chr1	74213677	74213925	Intergenic	Intergenic	13233	NM_00990	12765	Cxcr2	CD128|CD
chr3	1.23E+08	1.23E+08	promoter-T	promoter-T	45	NM_20163	210529	Mettl14	G430022H
chr14	45523802	45524250	Intergenic	Intergenic	-44976	NM_00896	19214	Ptgdr	DP|PGD
chr11	60231227	60231325	promoter-T	promoter-T	629	NM_02575	66771	Gid4	4933439F1
chr4	1.16E+08	1.16E+08	promoter-T	promoter-T	543	NM_15352	230654	Lrrc41	AA409966|
chr2	1.17E+08	1.17E+08	Intergenic	Intergenic	-7762	NM_02662	68215	Fam98b	2610510H0
chrX	34624227	34625725	intron (NM	intron (NM	203	NM_02605	67248	Rpl39	2810465O1
chr8	1.1E+08	1.1E+08	promoter-T	promoter-T	-560	NM_02583	66894	Wwp2	1300010O0
chr16	76527427	76527625	Intergenic	Intergenic	-154232	NM_17344	268903	Nrip1	6030458L2
chr12	78153102	78153175	Intergenic	Intergenic	-89903	NM_00114	17187	Max	AA960152|
chr7	87571102	87571475	Intergenic	(TCC)n|Sim	-20971	NM_00108	18550	Furin	9130404I0
chr2	26768827	26769075	TTS (NR_07	TTS (NR_07	2624	NM_01372	27176	Rpl7a	Surf3
chr13	58229952	58230200	promoter-T	promoter-T	-159	NM_02987	77134	Hnrnpa0	1110055B0
chr13	43344977	43345125	intron (NM	B1_Mur1|S	54490	NM_00103	328232	Gfod1	9630032O1
chr12	1.04E+08	1.04E+08	promoter-T	promoter-T	3	NM_02566	66622	Ubr7	5730410I1
chr4	1.4E+08	1.4E+08	intron (NM	intron (NM	106920	NM_00111	72754	Arhgef10l	2810441C0
chr11	45740127	45740550	Intergenic	Intergenic	18099	NM_02818	72290	Lsm11	2210404M
chr9	1.23E+08	1.23E+08	intron (NM	L4|LINE|RT	19122	NM_02691	69035	Zdhhc3	1110020O2
chr3	84469877	84470200	promoter-T	promoter-T	-76	NM_17726	320782	Tmem154	9930117H0
chr10	1.17E+08	1.17E+08	Intergenic	Intergenic	-27033	NM_02445	215449	Rap1b	2810443E1
chr8	1.23E+08	1.23E+08	intron (NM	CpG-14580	735	NM_19867	382034	Gse1	2210013I1
chr1	1.59E+08	1.59E+08	intron (NM	CpG	178	NM_14541	215015	Fam20b	C530043G
chr3	68428802	68428975	intron (NM	intron (NM	52598	NM_01392	30953	Schip1	Nf2ip|Schi
chr4	86394777	86395000	intron (NM	CpG	429	NM_18408	329877	Dennd4c	1700065A0

chr8	1.09E+08	1.09E+08	intron (NM	intron (NM	86295	NM_17303	272538	Tango6	AW413431
chr2	34227977	34228175	promoter-T	promoter-T	-511	NM_01676	18516	Pbx3	-
chr9	83795827	83796075	Intergenic	Intergenic	-46437	NM_19919	12040	Bckdhb	-
chr9	1E+08	1E+08	Intergenic	Intergenic	40079	NM_00103	213208	Il20rb	AV228068|
chr17	23966127	23966300	promoter-T	promoter-T	-137	NM_02630	67673	Tceb2	0610040H1
chr6	47763752	47763900	promoter-T	promoter-T	-315	NM_00978	12304	Pdia4	AI987846|
chr2	1.7E+08	1.7E+08	Intergenic	Intergenic	-11051	NM_00115	228913	Zfp217	4933431C0
chr10	1.27E+08	1.27E+08	promoter-T	promoter-T	-624	NM_03307	110962	Mbd6	D10Wsu93
chr1	84936877	84937225	Intergenic	Intergenic	-5393	NM_02792	71781	Slc16a14	1110004H1
chr10	1.17E+08	1.17E+08	promoter-T	promoter-T	54	NM_17779	327826	Frs2	4732458E1
chr18	12812502	12812575	intron (NM	intron (NM	10496	NM_02834	72747	Ttc39c	1700008N0
chr13	55629127	55629275	intron (NM	intron (NM	698	NM_01373	27261	Dok3	AI450713|
chr4	1.39E+08	1.39E+08	promoter-T	promoter-T	449	NM_00103	12345	Capzb	1700120C0
chr10	7401077	7401225	promoter-T	promoter-T	144	NM_01069	16798	Lats1	AW208599
chr4	41222527	41222875	promoter-T	promoter-T	-533	NM_02687	68926	Ubap2	1190005K0
chrX	39561452	39561650	intron (NM	intron (NM	6519	NM_00129	20843	Stag2	9230105L2
chr6	50327727	50327800	intron (NM	intron (NM	5073	NM_02788	71720	Osbpl3	1200014M
chr2	94278002	94278325	5' UTR (NM	5' UTR (NM	141	NM_00746	11800	Api5	AAC-11|AI
chr3	1.29E+08	1.29E+08	Intergenic	ID_B1|SINE	-38066	NM_13045	170439	Elovl6	C77826|FA
chr11	60988327	60988450	5' UTR (NM	5' UTR (NM	173	NM_00100	216825	Usp22	AI427806
chr4	1.18E+08	1.18E+08	Intergenic	Intergenic	-15311	NM_14631	18363	Olfr62	H12|IH12|
chr2	24200527	24200950	promoter-T	promoter-T	-73	NM_00115	16181	Il1rn	F630041P1
chr9	1.24E+08	1.24E+08	promoter-T	promoter-T	-13	NM_03332	93730	Lztfl1	5530402H0
chr3	88268627	88268850	Intergenic	Intergenic	-3634	NM_00116	20351	Sema4a	AI132332|
chr4	1.49E+08	1.49E+08	promoter-T	promoter-T	-4	NM_02696	69151	Lzic	1810030J0
chr13	55313627	55314050	intron (NM	CpG	2695	NM_00873	18193	Nsd1	AI528500|
chr11	1.16E+08	1.16E+08	promoter-T	promoter-T	-333	NM_00824	15223	Foxj1	FKHL-13|H
chr4	1.55E+08	1.55E+08	5' UTR (NM	5' UTR (NM	337	NM_00125	76580	Mib2	2210008I1
chr15	36537477	36538200	intron (NM	CpG	890	NM_00877	18458	Pabpc1	PABP|Pabp
chr15	97611002	97611275	Intergenic	Intergenic	-3658	NM_02635	67739	Slc48a1	4930570C0
chr18	15764352	15764475	intron (NM	intron (NM	112142	NM_19905	71367	Chst9	5430438D0
chr12	11272927	11273075	promoter-T	promoter-T	309	NM_02569	67241	Smc6	2810489L2
chr4	1.2E+08	1.2E+08	promoter-T	promoter-T	20	NM_00127	18046	Nfyc	-
chr15	78156277	78156500	promoter-T	promoter-T	-32	NM_00778	12983	Csf2rb	AI848964|
chr5	53658777	53658850	intron (NM	intron (NM	468	NM_00114	66278	Smim20	1110067B1
chr18	61716152	61716400	intron (NM	intron (NM	1040	NM_14608	93687	Csnk1a1	2610208K1
chr5	24348702	24349125	promoter-T	promoter-T	-734	NM_05307	19744	Rheb	-
chr2	72901952	72902225	Intergenic	MIRb|SINE	-83585	NM_00101	20687	Sp3	D130027J0
chr16	22350102	22350250	Intergenic	Intergenic	-84174	NM_00918	20462	Tra2b	5730405G2
chr17	56146327	56146425	Intergenic	Intergenic	-1347	NR_10231	106766	Stap2	AW049765
chr5	1.08E+08	1.08E+08	intron (NM	intron (NM	19438	NM_00796	14020	Evi5	NB4S
chr6	89545302	89545525	intron (NM	CpG	233	NM_02535	66098	Chchd6	0710001P0
chr14	52816277	52816525	intron (NM	CpG	513	NM_03361	114741	Supt16	Cdc68|Fact
chr15	85161702	85161900	Intergenic	B1F2|SINE	-5010	NM_01684	54138	Atxn10	AI325283|
chr19	45282377	45282650	Intergenic	Intergenic	27213	NM_01069	16814	Lbx1	Lbx1h
chr17	71649752	71650000	intron (NM	intron (NM	10429	NM_14515	246707	Emilin2	FOAP-10
chr10	95236152	95236400	Intergenic	Intergenic	-167021	NM_00100	216238	Eea1	A430109M

chr8	1.31E+08	1.31E+08	Intergenic	Intergenic	-1010	NM_00873	18186	Nrp1	C530029I0
chr16	87700327	87700550	intron (NM	intron (NM	1239	NM_00752	12013	Bach1	6230421P0
chr13	37972277	37972550	intron (NM	intron (NM	-7890	NR_03321	68750	Rreb1	1110037N0
chr16	44746327	44746425	promoter-T	promoter-T	100	NM_00115	66067	Gtpbp8	0610037H2
chr11	69727202	69727400	promoter-T	promoter-T	-393	NM_01972	56310	Gps2	AI505953
chr6	1.01E+08	1.01E+08	intron (NM	ORR1D1|LT	54938	NM_18159	72171	Shq1	2810403P1
chr5	1.24E+08	1.24E+08	intron (NM	intron (NM	3174	NM_00104	208043	Setd1b	AA516740|
chr13	98711902	98712225	intron (NM	ID4_|SINE|	264057	NM_01202	110596	Arhgef28	9230110L0
chr5	1.5E+08	1.5E+08	intron (NM	intron (NM	7742	NM_00966	11690	Alox5ap	Flap
chr5	1.44E+08	1.44E+08	Intergenic	Intergenic	-14085	NM_00739	11461	Actb	Actx|E430(
chr4	1.34E+08	1.34E+08	promoter-T	promoter-T	-772	NM_01964	16765	Stmn1	19k|Lag|La
chr2	1.29E+08	1.29E+08	promoter-T	promoter-T	-57	NM_00836	16176	Il1b	IL-1beta|Il-
chr2	1.65E+08	1.65E+08	promoter-T	promoter-T	-80	NM_00108	415115	Neurl2	Neur2|Ozz
chr9	96537727	96537900	Intergenic	Intergenic	-5891	NM_05326	114713	Rasa2	5430433H2
chr8	83267427	83267550	Intergenic	Lx7|LINE|L	-4130	NM_05312	93762	Smarca5	4933427E2
chr3	1.58E+08	1.58E+08	intron (NM	intron (NM	25630	NM_00128	67144	Lrrc40	2610040E1
chr15	97923827	97923925	5' UTR (NM	5' UTR (NM	124	NM_14485	223870	Senp1	2310046A2
chr3	88927402	88927575	Intergenic	Intergenic	-12576	NM_01363	18770	Pklr	Pk-1|Pk1|F
chr12	1.18E+08	1.18E+08	Intergenic	Intergenic	-16305	NM_01121	19276	Ptprn2	4930425H1
chr7	71537727	71537800	promoter-T	promoter-T	106	NM_02862	73724	Mcee	1110007A0
chr1	1.52E+08	1.52E+08	promoter-T	promoter-T	-99	NM_00886	18783	Pla2g4a	Pla2g4|cPL
chr17	29089577	29089775	promoter-T	promoter-T	-485	NM_02588	66989	Kctd20	2410004N1
chr16	75933552	75933825	Intergenic	Intergenic	-24177	NM_02338	67742	Samsn1	4930571B1
chr4	24423127	24423275	promoter-T	promoter-T	-408	NM_19946	212377	Mms22l	F730047E0
chr7	88027977	88028075	Intergenic	Intergenic	18129	NM_02802	71968	Wdr73	1200011I2
chr2	1.21E+08	1.21E+08	intron (NM	intron (NM	299	NM_14597	269344	Ell3	A930015D2
chr8	89290652	89290850	Intergenic	Intergenic	-20846	NM_00917	20437	Siah1a	AA982064|
chr8	87501502	87501600	exon (NM_	exon (NM_	1096	NM_00841	16477	Junb	-
chr1	1.83E+08	1.83E+08	Intergenic	Intergenic	-4412	NM_17709	320202	Lefty2	6030463A2
chr5	1.16E+08	1.16E+08	promoter-T	promoter-T	119	NM_02964	384281	Gatc	2010003O1
chrX	54306477	54306550	promoter-T	promoter-T	-234	NM_02824	72459	Htatsf1	1600023H1
chr16	14317252	14317375	intron (NM	CpG	112	NM_02534	66086	Fopnl	0610037P0
chr6	1.13E+08	1.13E+08	promoter-T	promoter-T	-149	NM_13393	101206	Tada3	1110004B1
chr6	52167302	52167500	exon (NM_	exon (NM_	1171	NM_01045	15404	Hoxa7	AV118143|
chr17	28466677	28466875	intron (NM	intron (NM	1360	NM_01128	19896	Rpl10a	CsA-19|Ne
chr15	27805477	27805750	intron (NM	intron (NM	149990	NM_00108	223435	Trio	6720464I0
chr8	26864852	26865125	promoter-T	promoter-T	-236	NM_02810	72108	Ddhd2	2010305K1
chr8	93489552	93489625	intron (NM	L1MC4a|LI	-104368	NM_00128	19651	Rbl2	PRB2|RBR-
chr12	1.18E+08	1.18E+08	intron (NM	intron (NM	45068	NM_02873	52635	Esyt2	2410017M
chr1	1.9E+08	1.9E+08	intron (NM	RSINE1|SIN	170328	NM_00124	26381	Esrrg	ERR3|Errg|
chr12	32035877	32036025	intron (NM	CpG	385	NM_00786	13382	Dld	-
chr3	28070277	28070475	Intergenic	MER20|DN	-91760	NM_00116	665113	Tnik	1500031A1
chr8	18846527	18846775	exon (NM_	exon (NM_	372	NM_02679	52123	Agpat5	1110013A0
chr1	1.34E+08	1.34E+08	exon (NM_	exon (NM_	274	NM_00119	74137	Nuak2	1200013B2
chr15	1.01E+08	1.01E+08	Intergenic	B1F|SINE|/	-18680	NM_00739	11479	Acvr1b	6820432J0
chr10	1.15E+08	1.15E+08	5' UTR (NM	5' UTR (NM	221	NM_02445	216344	Rab21	9630024B2
chr14	73809952	73810200	Intergenic	Intergenic	-24998	NM_00841	16432	Itm2b	AI256040|

chr11	1.18E+08	1.18E+08	intron (NM	intron (NM	42673	NM_19802	217351	Tnrc6c	9930033H1
chr15	76181727	76182050	promoter-T	promoter-T	164	NM_02685	68877	Maf1	1110068E1
chr11	98227627	98227875	intron (NM	intron (NM	8069	NM_02154	59045	Stard3	Mln64|es6
chr19	28084877	28085050	intron (NM	intron (NM	693	NM_01126	19726	Rfx3	C230093O1
chr1	1.93E+08	1.93E+08	Intergenic	Intergenic	-17964	NM_00749	11910	Atf3	LRG-21
chr15	86013377	86013500	intron (NM	intron (NM	3133	NM_14547	223753	Cerk	AI848610|
chr18	10029677	10029875	intron (NM	CpG	371	NM_02152	59025	Usp14	2610005K1
chr6	1.16E+08	1.16E+08	intron (NM	intron (NM	9815	NM_02978	110157	Raf1	6430402F1
chr11	65609427	65609600	Intergenic	B4A|SINE|	-7714	NM_00915	26398	Map2k4	JNKK1|MEI
chr5	96592127	96592300	Intergenic	Intergenic	-1204	NM_14491	231464	Cnot6l	4932442K2
chr15	71427552	71427650	intron (NM	ORR1E|LTF	130667	NM_17781	70363	Fam135b	1700010C2
chr2	1.2E+08	1.2E+08	promoter-T	promoter-T	-636	NM_00111	211499	Tmem87a	A930025J1
chr2	1.03E+08	1.03E+08	5' UTR (NM	5' UTR (NM	234	NM_00117	12505	Cd44	AU023126
chr13	94710852	94710975	3' UTR (NM	3' UTR (NM	-116838	NM_17258	218454	Lhfpl2	6030465B1
chr9	59060852	59060950	Intergenic	Lx8|LINE|L	-78478	NM_02812	72141	Adpgk	2610017G0
chr6	99473027	99473400	Intergenic	Intergenic	-87874	NM_05320	108655	Foxp1	3110052D1
chr14	68398452	68398525	intron (NM	intron (NM	35202	NM_00814	14714	Gnrh1	Gnrh|Gnrh
chr11	78272477	78272800	Intergenic	ORR1D2|LT	-6565	NM_02674	52466	Slc46a1	1110002C0
chr12	85313377	85313700	Intergenic	Intergenic	-15273	NM_13418	171210	Acot2	AA571646|
chr1	1.73E+08	1.73E+08	promoter-T	promoter-T	-25	NM_01204	27045	Nit1	AI255805|
chr7	20520727	20521025	Intergenic	RMER19A|	-14384	NM_02751	52118	Pvr	3830421F0
chr16	4963977	4964175	promoter-T	promoter-T	254	NM_02830	72615	Anks3	2700067D0
chr7	1.06E+08	1.06E+08	Intergenic	Intergenic	70494	NM_00111	434215	Lrrc32	AI426318|
chr4	94269402	94269550	intron (NM	CpG	462	NM_17269	18786	Plaa	2410007N0
chr11	84683077	84683275	intron (NM	CpG	1064	NM_15314	217039	Ggnbp2	AI451896|
chr14	65059052	65059300	intron (NM	intron (NM	687	NM_00125	110265	Msra	2310045J2
chr12	85481277	85481425	3' UTR (NM	3' UTR (NM	8088	NM_02743	70481	Pnma1	5730402C1
chr1	8880377	8880575	intron (NM	ETnERV3-ir	409482	NM_00129	71096	Sntg1	4933426D1
chr3	1.33E+08	1.33E+08	promoter-T	promoter-T	27	NM_14614	74776	Ppa2	1110013G1
chr7	1.21E+08	1.21E+08	intron (NM	CpG	218	NM_03337	70349	Copb1	2610019B0
chr5	1.23E+08	1.23E+08	promoter-T	promoter-T	-52	NM_01980	56317	Anapc7	APC7|AW5
chr9	78381052	78381425	Intergenic	Intergenic	-51707	NM_01010	13627	Eef1a1	-
chr5	1.3E+08	1.3E+08	promoter-T	promoter-T	60	NM_02545	66258	Mrps17	1810006P0
chr1	1.33E+08	1.33E+08	intron (NM	intron (NM	13769	NM_00855	17164	Mapkapk2	AA960234|
chr2	35057152	35057275	promoter-T	promoter-T	-573	NM_02669	68365	Rab14	0610030G2
chr2	1.25E+08	1.25E+08	intron (NM	intron (NM	49948	NM_00799	14118	Fbn1	AI536462|
chr4	1.07E+08	1.07E+08	intron (NR_	CpG-10126	1287	NM_00108	16975	Lrp8	4932703M
chr1	1.31E+08	1.31E+08	Intergenic	Intergenic	-50112	NM_00991	12767	Cxcr4	CD184|Cm
chr2	69560877	69561325	promoter-T	promoter-T	-44	NM_00108	228005	Ppig	AU019516
chr19	41529777	41529925	Intergenic	RMER19B|	-70291	NM_03137	83490	Pik3ap1	1810044J0
chr10	98569477	98569900	promoter-T	promoter-T	-117	NM_02774	382406	Poc1b	4933430F1
chr6	1.18E+08	1.18E+08	Intergenic	CpG	16393	NM_00100	22685	Zfp239	Mok-2|Mo
chr6	87450077	87450175	intron (NM	intron (NM	-3805	NM_17547	232201	Arhgap25	A130039I2
chr5	1.4E+08	1.4E+08	intron (NM	intron (NM	2097	NM_17700	319772	C130050O1	-
chr9	69290502	69290875	Intergenic	Intergenic	-10802	NM_00758	12306	Anxa2	AW215814
chr7	89750727	89750850	exon (NM_	exon (NM_	46250	NM_17789	330577	Fam154b	1700129I0
chr19	55328227	55328400	5' UTR (NM	5' UTR (NM	454	NM_02797	433256	Acsl5	1700030F0

chr3	1.26E+08	1.26E+08	intron (NM	Lx6|LINE|L	15994	NM_02381	108058	Camk2d	2810011D2
chr11	72156227	72156475	Intergenic	Intergenic	18447	NM_00128	21689	Tekt1	MT14
chr4	97770827	97771250	intron (NM	intron (NM	278918	NM_05315	94043	Tm2d1	2310026L1
chr16	19963327	19963425	intron (NM	Lx8|LINE|L	19746	NM_18339	239743	Klhl6	-
chr9	20632202	20632475	Intergenic	B3A|SINE|	9390	NM_00103	235033	Rdh8	Gm182|prl
chr17	28168327	28168475	intron (NM	L1ME2z|LII	49128	NM_01368	21463	Tcp11	D17Ken1|T
chr7	82740977	82741200	intron (NM	intron (NM	140668	NM_02933	75547	Akap13	1700026G0
chr2	79094727	79094950	promoter-T	promoter-T	-745	NM_01057	16401	Itga4	CD49D|Itga
chr17	45082327	45082425	intron (NM	intron (NM	-64542	NM_02925	75341	4930564C0	-
chr4	1.05E+08	1.05E+08	Intergenic	Intergenic	-8189	NM_08055	67916	Ppap2b	1110003O2
chr19	47083952	47084100	intron (NM	L1MC4a|LI	-5162	NM_14610	226180	Ina	AV028420|
chr11	51076727	51076950	promoter-T	promoter-T	165	NM_00771	12750	Clk4	AI987988|
chr11	83453677	83454050	Intergenic	Intergenic	9017	NM_01133	20302	Ccl3	AI323804|
chr7	51629652	51630100	5' UTR (NM	5' UTR (NM	524	NM_02704	69349	1700008O0	-
chr19	46723227	46723325	intron (NM	intron (NM	24214	NM_00780	13074	Cyp17a1	Cyp17|p45
chr16	30188177	30188375	Intergenic	Intergenic	79342	NM_02790	71756	Cpn2	1300018K1
chr10	1.28E+08	1.28E+08	intron (NM	intron (NM	711	NM_01681	13139	Dgka	80kDa|AW
chr11	87882602	87882700	intron (NM	CpG	870	NM_01668	22344	Vezf1	AI848691|
chr1	1.66E+08	1.66E+08	Intergenic	RLTR45|LT	-39102	NM_00972	11931	Atp1b1	Atp4b|Atp
chr11	96230627	96230850	Intergenic	Intergenic	3666	NM_00826	15407	Hoxb1	Hox-2.9
chr11	51885352	51885625	intron (NM	intron (NM	-26838	NM_01941	19052	Ppp2ca	PP2A|R753
chr6	18026727	18026875	intron (NM	intron (NM	32260	NM_02372	74068	Asz1	4933400N1
chr1	1.93E+08	1.93E+08	intron (NM	intron (NM	12373	NM_17877	320119	Rps6kc1	AA682037|
chr18	24178727	24178925	intron (NM	CpG	446	NM_02155	59057	Zfp191	3526401F1
chr15	76554627	76554800	promoter-T	promoter-T	-438	NM_17682	223665	C030006K1	1110033I1
chr1	1.57E+08	1.57E+08	Intergenic	Intergenic	15115	NM_01050	15939	Ier5	-
chr2	1.53E+08	1.53E+08	Intergenic	Intergenic	-17641	NM_00117	15162	Hck	AI849071|
chr13	93300252	93301000	intron (NM	CpG	139	NM_17339	218441	Zfyve16	AI035632|
chr11	97223902	97223975	promoter-T	promoter-T	73	NM_13865	192157	Socs7	2310063P0
chr9	77676377	77676675	Intergenic	L1_Rod|LIN	74184	NM_01029	14629	Gclc	D9Wsu168
chr8	73158402	73158500	Intergenic	Intergenic	-2917	NM_01181	23886	Gdf15	MIC-1|NAG
chr1	55470502	55470575	intron (NM	intron (NM	7748	NM_00111	227120	Plcl1	C230017K0
chr18	55013302	55013400	Intergenic	Intergenic	136483	NM_17575	269023	Zfp608	4932417D1
chr1	1.53E+08	1.53E+08	promoter-T	promoter-T	-802	NM_00103	117198	Ivns1abp	1190004M
chr17	37116302	37116400	intron (NM	RSINE1|SIN	1831	NM_02974	76797	2410137M	-
chr16	35932727	35933025	exon (NM_	exon (NM_	-5680	NM_03025	80285	Parp9	ARTD9|AW
chr11	83561327	83561575	intron (NM	intron (NM	1009	NM_18324	66107	Wfdc21	1100001G2
chr10	77559402	77559600	Intergenic	Intergenic	27388	NM_01579	50723	Icosl	AU044799
chr6	1.16E+08	1.16E+08	intron (NM	intron (NM	19800	NM_02658	28006	Fam21	A130095H0
chr3	1.28E+08	1.28E+08	promoter-T	promoter-T	-353	NM_14596	211556	Ap1ar	AA407621|
chr3	1.03E+08	1.03E+08	intron (NM	intron (NM	-16482	NM_01093	18176	Nras	AV095280|
chr3	1.32E+08	1.32E+08	Intergenic	Intergenic	-193193	NM_02026	56811	Dkk2	-
chr11	69494527	69494600	intron (NM	intron (NM	909	NM_03070	80886	Senp3	AA408656|
chr5	1.11E+08	1.11E+08	intron (NM	CpG	373	NM_02933	75560	Ep400	1700020J0
chr5	97352352	97352600	Intergenic	Intergenic	-74232	NM_08070	140780	Bmp2k	4933417M
chr1	1.61E+08	1.61E+08	Intergenic	Intergenic	-45231	NM_01193	26374	Rfwd2	AI316802|
chr15	34425302	34425475	promoter-T	promoter-T	322	NM_15289	67724	Pop1	4932434G0

chr14	70451652	70451725	intron (NM	intron (NM	-25564	NM_01878	13655	Egr3	Pilot
chr2	1.27E+08	1.27E+08	non-coding	non-coding	115	NM_00127	14391	Gabpb1	BABPB2|E4
chr18	57404177	57404525	intron (NM	intron (NM	-110036	NM_02844	73137	Prrc1	1190002C0
chr13	68720652	68720825	promoter-T	promoter-T	260	NM_17248	210009	Mtrr	4732420G0
chr10	1.26E+08	1.26E+08	promoter-T	promoter-T	51	NM_02685	68876	Xrcc6bp1	1110068E0
chr9	1.14E+08	1.14E+08	TTS (NM_0	TTS (NM_0	46299	NM_01369	22221	Ubp1	Cp2b|LBP-
chr16	38415077	38415325	intron (NM	intron (NM	18024	NM_13410	85031	Pla1a	AA986889|
chr19	47644577	47644725	Intergenic	Intergenic	-9858	NM_00928	20874	Slk	9A2|AV021
chr7	1.28E+08	1.28E+08	Intergenic	tRNA-Leu-C	-7332	NM_00790	13631	Eef2k	C86191|eE
chr14	66851402	66851625	intron (NM	intron (NM	-19124	NM_00116	19229	Ptk2b	CADTK|CAI
chr16	36334377	36334800	promoter-T	promoter-T	-170	NM_00100	1E+08	BC117090	Gm1975
chr9	78311302	78311500	intron (NM	intron (NM	15384	NM_02665	68291	Mto1	2310039H0
chr3	1.58E+08	1.58E+08	exon (NM_	exon (NM_	246	NM_00101	433667	Ankrd13c	AI505652|A
chr2	38888102	38888450	intron (NR_	intron (NR_	24617	NM_02880	74192	Arpc5l	2010015J0
chr4	65886377	65886550	intron (NM	intron (NM	179054	NM_01951	56079	Astn2	1d8|Astnl|
chr1	75206327	75206450	intron (NM	intron (NM	965	NM_02901	74577	Glb1l	4833408P1
chr9	50873652	50873775	intron (NM	intron (NM	11486	NM_00103	244864	Layn	E030012M
chr1	1.67E+08	1.67E+08	intron (NM	intron (NM	4112	NM_17302	271639	Adcy10	4930431D0
chr11	1.15E+08	1.15E+08	intron (NM	intron (NM	3959	NM_02141	58222	Rab37	B230331O0
chr15	96115852	96116000	Intergenic	CpG	-2027	NM_17525	77044	Arid2	1700124K1
chr15	39157427	39157675	intron (NM	RMER19B|	127719	NM_05327	116838	Rims2	2810036I1
chr11	82608827	82609200	intron (NM	intron (NM	14402	NM_01071	16882	Lig3	D11Wsu78
chr1	57012377	57012450	intron (NM	intron (NM	15765	NM_13914	212712	Satb2	mKIAA1034
chr15	1E+08	1E+08	promoter-T	promoter-T	70	NR_104338	207818	Smagp	-
chr7	26221727	26222100	intron (NR_	intron (NR_	-24008	NM_15357	232983	Cxcl17	VCC-1|Vcc1
chr11	1.06E+08	1.06E+08	intron (NM	intron (NM	-47233	NM_01049	15896	Icam2	CD102|Ica
chr7	1.48E+08	1.48E+08	promoter-T	promoter-T	64	NM_00108	101471	Phrf1	AA673488
chr10	80630827	80630900	promoter-T	promoter-T	-398	NM_02150	59004	Pias4	PIASY|Pias
chr5	15832852	15832975	intron (NM	intron (NM	-226400	NM_00128	15234	Hgf	C230052L0
chr6	1.25E+08	1.25E+08	intron (NM	CpG	828	NM_00808	14433	Gapdh	Gapd
chr2	69723602	69724000	promoter-T	promoter-T	-129	NR_110977	75422	Mettl5	2810410A0
chr1	1.53E+08	1.53E+08	intron (NM	CpG	660	NM_00103	117198	Ivns1abp	1190004M
chr11	61743302	61743500	intron (NM	CpG	358	NM_01992	56697	Akap10	1500031L1
chr5	1.11E+08	1.11E+08	intron (NM	CpG-11321	690	NM_14514	107999	Gtpbp6	AV119224|
chr17	64198152	64198400	Intergenic	Intergenic	-15054	NM_00103	14158	Fert2	AV082135|
chr8	1.28E+08	1.28E+08	promoter-T	promoter-T	-259	NM_01690	53424	Tsnax	R74833|Tr
chr18	60912752	60912825	Intergenic	(CAGAGA)n	-21462	NM_02060	20044	Rps14	2600014J0
chr7	1.35E+08	1.35E+08	promoter-T	promoter-T	-384	NM_00929	20909	Stx4a	Stx4|Syn-4
chr4	32792827	32793050	intron (NM	intron (NM	48844	NM_00108	100019	Mdn1	4833432B2
chr2	30319777	30319925	Intergenic	Intergenic	9868	NM_03024	72500	Ier5l	2610524G0
chr11	84798977	84799175	3' UTR (NM	3' UTR (NM	27820	NM_00760	12351	Car4	AW456718
chr10	75243827	75243975	5' UTR (NM	5' UTR (NM	258	NM_13399	103140	Gstt3	AI118089
chr1	1.81E+08	1.81E+08	intron (NM	intron (NM	14666	NM_14610	226744	Cnst	9630058J2
chr19	40905502	40905700	promoter-T	promoter-T	-168	NM_17283	240665	Ccnj	D430039C2
chr8	73051702	73051825	promoter-T	promoter-T	18	NM_00119	14232	Fkbp8	38kDa|FKB
chr5	1.14E+08	1.14E+08	intron (NM	intron (NM	455	NM_02552	66383	Iscu	2310020H2
chr3	1.46E+08	1.46E+08	promoter-T	promoter-T	426	NM_01031	14707	Gng5	G(y)5

chr6	1.16E+08	1.16E+08	intron (NM	MIR|SINE|	61416	NM_17741	330401	Tmcc1	3632431M
chr2	1.44E+08	1.44E+08	exon (NM_	exon (NM_	124	NM_02531	66044	Dtd1	0610006H(
chr12	36239102	36239325	Intergenic	Intergenic	-19552	NM_01346	11622	Ahr	Ah|Ahh|Al
chr6	1.15E+08	1.15E+08	intron (NM	intron (NM	27494	NM_17768	232334	Vgll4	BC048841|
chr11	69749077	69749550	promoter-T	promoter-T	-88	NM_01687	53422	Ybx2	Msy2
chr2	91012077	91012325	intron (NM	intron (NM	7277	NM_00117	228355	Madd	9630059K2
chr7	1.4E+08	1.4E+08	promoter-T	promoter-T	-497	NM_21247	77938	Fam53b	A930008G:
chr3	1.3E+08	1.3E+08	intron (NM	intron (NM	33448	NM_02577	66815	Ccdc109b	9030408N:
chr1	82312727	82312875	promoter-T	promoter-T	-353	NM_02977	76867	Rhbdd1	4930418P(
chr3	32409527	32410100	intron (NM	CpG	299	NM_14451	67778	Zfp639	6230400O:
chr7	71135602	71135725	Intergenic	Intergenic	-51862	NM_02136	50794	Klf13	0610043C1
chr1	1.37E+08	1.37E+08	intron (NM	intron (NM	1214	NM_00795	13924	Ptprv	Esp|OST|C
chr2	48861177	48861275	intron (NM	intron (NM	56198	NM_02992	109241	Mbd5	9430004D:
chr6	38483877	38484050	promoter-T	promoter-T	-898	NM_02536	66117	1110001J0	Fmc1
chrX	11583952	11584075	Intergenic	CpG	73666	NM_17504	71458	Bcor	5830466J1
chr4	1.26E+08	1.26E+08	promoter-T	promoter-T	14	NM_17555	269582	Clspn	B130025E(
chr18	10993627	10993925	Intergenic	Intergenic	-58732	NM_01025	14465	Gata6	AA410133|
chr7	52308502	52308600	promoter-T	promoter-T	-300	NM_17502	233210	Prr12	6720469B1
chr1	1.08E+08	1.08E+08	intron (NM	intron (NM	96355	NM_13382	98432	Phlpp1	AI836256|
chr12	85028927	85029300	promoter-T	promoter-T	-400	NM_00894	19164	Psen1	Ad3h|PS-1
chr13	37711627	37711800	Intergenic	Intergenic	-206194	NM_02683	68750	Rreb1	1110037N(
chr4	63071402	63071725	Intergenic	CpG	-7084	NM_00104	100182	Akna	AI597013|
chr16	27389027	27389200	promoter-T	promoter-T	71	NM_00128	67501	Ccdc50	C3orf6
chr15	66392477	66392625	promoter-T	promoter-T	57	NM_17251	213068	Tmem71	AI661017|
chr1	66082677	66082800	Intergenic	(TAA)n|Sim	109642	NM_02701	12969	Crygf	3110001K1
chr12	56921502	56921600	intron (NM	CpG	952	NM_01999	56784	Ralgapa1	2310003F2
chr11	98779077	98779200	intron (NM	intron (NM	9673	NM_01179	23834	Cdc6	CDC18L
chr2	1.34E+08	1.34E+08	intron (NM	intron (NM	556	NM_02914	52837	Tmx4	2810417D(
chr9	66195477	66195550	Intergenic	Intergenic	-2744	NM_14561	235439	Herc1	2810449H1
chr8	1.14E+08	1.14E+08	intron (NM	intron (NM	32922	NM_01180	23837	Cfdp1	AA408409|
chr2	90383002	90383325	intron (NM	L2c|LINE|L	37641	NM_00898	19271	Ptprj	AI450271|
chr12	88495402	88495775	intron (NM	MMERVK1(	-6736	NM_15341	217734	Pomt2	A830009D:
chr14	31508802	31509025	Intergenic	Intergenic	-19122	NM_01946	54650	Sfmbt1	4930442N2
chr8	1.25E+08	1.25E+08	intron (NM	CpG	453	NM_00103	234839	Piezo1	9630020g2
chr13	63916602	63917100	intron (NM	CpG	209	NM_02350	76251	Ercc6l2	0610007P(
chr6	38331202	38331425	promoter-T	promoter-T	-211	NM_15360	264134	Ttc26	9330141E2
chr8	1.22E+08	1.22E+08	intron (NM	intron (NM	9604	NM_02692	69047	Atp2c2	1810010G(
chr4	1.36E+08	1.36E+08	exon (NM_	exon (NM_	554	NM_17773	242705	E2f2	9230110J1
chr10	92951152	92951525	promoter-T	promoter-T	-175	NM_01040	15109	Hal	Hsd|his|his
chr10	80819202	80819350	Intergenic	Intergenic	-1039	NM_02865	73822	Mfsd12	C19orf28|F
chr8	1.08E+08	1.08E+08	intron (NM	intron (NM	-21856	NM_02746	70575	Gfod2	5730466C2
chr2	1.15E+08	1.15E+08	promoter-T	promoter-T	-676	NM_20726	399568	BC052040	-
chr2	6130977	6131275	intron (NM	intron (NM	2914	NM_02420	67856	Echdc3	2310005D1
chr2	29744477	29744775	promoter-T	promoter-T	-614	NM_00117	18286	Odf2	AI848335|
chr8	82785727	82785800	Intergenic	Intergenic	-203856	NM_02025	15245	Hhip	Hhip1
chr7	35098677	35098750	Intergenic	CpG	-28085	NM_17274	233103	4931406P1	AA553327|
chr16	25424252	25424325	Intergenic	Intergenic	137385	NM_17516	71338	Tprg	5430420C1

chr11	3188677	3188775	promoter-T	promoter-T	-408	NR_045108	56218	Patz1	8430401L1
chr2	51002452	51002525	intron (NM	intron (NM	2143	NM_02881	74194	Rnd3	2610017M
chr13	74346102	74346175	promoter-T	promoter-T	10	NM_17733	211446	Exoc3	2810050O0
chr1	95202402	95202650	promoter-T	promoter-T	-79	NM_17805	69821	Mterf4	1810059A2
chr9	21921402	21921800	Intergenic	CpG	-2988	NM_17077	66126	Elof1	1110011K1
chr16	18248752	18249225	promoter-T	promoter-T	12	NM_00108	15547	Trmt2a	Htf9c
chr7	29766777	29766875	promoter-T	promoter-T	81	NM_00128	73830	Eif3k	1200009C2
chr4	1.02E+08	1.02E+08	Intergenic	Intergenic	68903	NM_00117	18578	Pde4b	Dpde4|R74
chr11	97642677	97644175	promoter-T	promoter-T	327	NM_02289	65019	Rpl23	2810009A0
chr2	1.53E+08	1.53E+08	promoter-T	promoter-T	393	NM_01880	54711	Plagl2	AU018672
chr3	27664852	27665275	Intergenic	Intergenic	-55702	NM_17318	72007	Fndc3b	1600019O0
chr3	89383152	89383375	intron (NM	intron (NM	59177	NM_08046	140493	Kcnn3	KCa2.3|SK3
chr7	91031577	91031700	exon (NM_	exon (NM_	1213	NM_03070	80889	Mesdc1	AW061151
chr7	26022427	26022625	exon (NM_	exon (NM_	344	NM_00103	606496	Gsk3a	2700086H0
chr5	65098777	65098925	Intergenic	Intergenic	-95911	NM_00845	16599	Klf3	9930027G0
chr7	25240452	25240650	Intergenic	Intergenic	-6968	NM_01111	18793	Plaur	Cd87|u-PA
chr12	89494552	89494750	intron (NM	ORR1A2-in	18290	NM_00101	435337	Gm5662	EG435337
chr14	15427327	15427450	Intergenic	Intergenic	-108151	NM_00103	218756	Slc4a7	E430014N1
chr18	76414477	76414675	intron (NM	intron (NM	-6199	NM_00125	17126	Smad2	7120426M
chr4	43572052	43572375	intron (NM	intron (NM	3242	NM_01160	21894	Tln1	Tln
chr2	83564327	83564425	promoter-T	promoter-T	-178	NM_00840	16410	Itgav	1110004F1
chr3	51458377	51458450	Intergenic	Intergenic	-6702	NM_17499	211666	Mgst2	GST2|MGS
chr14	98466777	98467075	intron (NM	intron (NM	102058	NM_00103	13134	Dach1	Dac|Dach
chr6	94450302	94450450	promoter-T	promoter-T	68	NM_02625	67582	Slc25a26	4930433D1
chr8	86780927	86781350	promoter-T	promoter-T	85	NM_02642	67873	Mri1	2410018C2
chr6	1.47E+08	1.47E+08	intron (NM	intron (NM	588	NM_02628	67623	Tm7sf3	2010003B1
chr9	57109927	57110175	intron (NM	CpG	355	NM_02882	74211	1700017B0	AA682102|
chr4	1.41E+08	1.41E+08	intron (NM	intron (NM	1323	NR_102344	213491	Szrd1	1110022I0
chr9	57335702	57336000	Intergenic	Intergenic	-16058	NM_13398	102614	Rpp25	AI851155
chr6	1.14E+08	1.14E+08	intron (NM	intron (NM	74594	NM_00103	11941	Atp2b2	D6Abb2e|(
chr10	94372102	94372225	intron (NM	intron (NM	35049	NM_01879	54712	Plxnc1	2510048K1
chr4	1.51E+08	1.51E+08	promoter-T	promoter-T	-198	NM_00115	74035	Nol9	4632412I24
chr9	55889777	55890325	intron (NM	CpG	399	NM_01199	26611	Rcn2	AA408742|
chr15	59384077	59384250	intron (NM	intron (NM	-96046	NM_14454	211770	Trib1	A530090O1
chr3	1.05E+08	1.05E+08	Intergenic	Intergenic	190751	NM_02922	75269	4930564D0	-
chr2	32092002	32092300	promoter-T	promoter-T	-52	NM_14514	99011	Pomt1	AI505244
chr5	92512427	92512750	promoter-T	promoter-T	-5	NM_00108	23881	G3bp2	AA409541|
chr1	1.73E+08	1.73E+08	Intergenic	Intergenic	-18741	NM_00108	75472	1700009P1	AV282292|
chr3	30730852	30731500	Intergenic	RLTR16|LT	-23696	NM_00113	71862	Gpr160	1700025D1
chr7	1.05E+08	1.05E+08	intron (NM	intron (NM	427	NM_01103	18479	Pak1	AW045634
chr7	16829477	16829750	promoter-T	promoter-T	-110	NM_17691	319430	C5ar2	C5L2|E030
chr3	1.05E+08	1.05E+08	exon (NM_	exon (NM_	197	NM_00861	17454	Mov10	C77703|M
chrX	83073252	83073650	Intergenic	Intergenic	-42161	NM_00116	71398	5430427O1	-
chr6	1.45E+08	1.45E+08	intron (NM	ID_B1|SINI	46344	NM_00102	12035	Bcat1	BCATc|Eca
chr15	55424952	55425075	intron (NM	intron (NM	36050	NM_00116	105837	Mtbp	AI429604|
chr10	96224277	96224475	Intergenic	Intergenic	144741	NM_00756	12226	Btg1	AI426953|/
chr2	24043777	24043925	intron (NM	intron (NM	1855	NM_15351	215257	Il1f9	Il36g

chr2	1.26E+08	1.26E+08	promoter-T	promoter-T	180	NM_00993	12848	Cops2	AI315723|(
chr11	8902477	8902575	intron (NM	intron (NM	8614	NM_00831	15574	Hus1	mHus1
chr1	88338427	88339050	Intergenic	CpG	15834	NM_02796	71863	1700019O1	-
chr2	24287677	24287850	intron (NM	LTR81B|LT	43356	NM_01104	18510	Pax8	Pax-8
chr18	56229777	56230050	Intergenic	Intergenic	-361873	NM_02624	107022	Gramd3	9030613F0
chr12	74145152	74145300	intron (NM	intron (NM	2473	NM_00918	20471	Six1	BB138287
chr12	34114527	34114750	exon (NM_	exon (NM_	149	NM_17225	28071	Twistnb	2410173G1
chr13	40413152	40413400	Intergenic	Intergenic	-29896	NM_17214	218165	Ofcc1	opo
chr4	1.35E+08	1.35E+08	Intergenic	Intergenic	-1109	NM_00113	51796	Srrm1	AA407769|
chr19	5693052	5693125	intron (NM	intron (NM	3957	NM_02201	26403	Map3k11	2610017K1
chr11	1.06E+08	1.06E+08	5' UTR (NM	5' UTR (NM	148	NM_00113	83796	Smarcd2	AW322457
chr4	1.32E+08	1.32E+08	promoter-T	promoter-T	-460	NM_00116	230789	Fam76a	-
chr6	66486552	66486700	intron (NM	B1_Mur2|S	1164	NM_01949	56150	Mad2l1	AA673185|
chr10	79723477	79723600	exon (NM_	exon (NM_	175	NM_01025	14431	Gamt	AA571402|
chr12	88142177	88142300	Intergenic	Intergenic	66919	NM_00119	627607	Lrrc74	EG627607|
chr2	25316077	25316450	intron (NM	CpG-7838	351	NM_15355	227622	BC029214	D930050G1
chr2	1.49E+08	1.49E+08	Intergenic	Intergenic	-85797	NM_02140	58214	Cst10	DD72
chr14	71165377	71165550	intron (NM	intron (NM	972	NM_02304	65246	Xpo7	4930506C0
chr5	90923577	90923650	intron (NM	Lx8|LINE|L	3873	NM_00742	11576	Afp	-
chr14	58416652	58416725	promoter-T	promoter-T	-338	NM_00911	20220	Sap18	C530046K0
chr4	1.34E+08	1.34E+08	intron (NM	intron (NM	998	NM_02366	27981	Rsrp1	2700043I2
chr1	39560127	39560375	Intergenic	Intergenic	-24659	NM_01877	54610	Tbc1d8	AD3|HBLP1
chr9	13553352	13553525	promoter-T	promoter-T	-187	NM_02385	77116	Mtmr2	6030445P1
chr6	1.23E+08	1.23E+08	promoter-T	promoter-T	1	NM_01081	17474	Clec4d	Clecsf8|Mp
chr1	1.88E+08	1.88E+08	Intergenic	Intergenic	197420	NM_00936	21808	Tgfb2	BB105277|
chr9	59334477	59334625	promoter-T	promoter-T	-370	NM_01992	23806	Arih1	AU021774
chr5	89081777	89081975	Intergenic	MTC|LTR|I	22729	NM_00109	231413	Grsf1	BB232551|
chr2	62484077	62484400	promoter-T	promoter-T	74	NM_02783	71586	Ifih1	9130009C2
chr4	1.21E+08	1.21E+08	Intergenic	Intergenic	-6774	NM_13371	69780	Smap2	1810031K0
chrX	1.54E+08	1.54E+08	exon (NM_	exon (NM_	196	NM_17230	270669	Mbtps2	9630032G2
chr11	63322227	63322400	Intergenic	LTR33A|LT	377301	NM_00888	18858	Pmp22	22kDa|Gas
chr11	1.18E+08	1.18E+08	intron (NM	CpG	898	NM_19802	217351	Tnrc6c	9930033H1
chr15	1.02E+08	1.02E+08	intron (NM	CpG	372	NM_14562	75705	Eif4b	2310046H1
chr6	49168102	49168200	Intergenic	CpG	-3198	NM_02367	140488	Igf2bp3	2610101N1
chr2	3574302	3574400	Intergenic	ORR1E|LTF	-56379	NM_02562	66540	Fam107b	3110001A1
chr2	1.12E+08	1.12E+08	Intergenic	Intergenic	-6144	NM_13374	73024	Emc7	2900064A1
chr1	14586252	14586375	Intergenic	Lx6|LINE|L	159734	NM_01082	17681	Msc	MyoR|bHL
chr17	70883527	70884200	intron (NM	L1M2|LINE	12413	NM_00112	224997	Dlgap1	4933422O1
chr6	28065527	28065825	intron (NM	intron (NM	18693	NM_00817	14823	Grm8	A230002O0
chr3	51684627	51684950	intron (NM	intron (NM	219673	NM_17499	211666	Mgst2	GST2|MGS
chr13	13621327	13621775	Intergenic	Intergenic	-61125	NM_01074	17101	Lyst	D13Sfk13|I
chr2	62544377	62544700	intron (NM	intron (NM	42154	NM_14552	227960	Gca	5133401E0
chr5	73965127	73965300	Intergenic	LTR40c|LTI	58447	NM_15356	231296	Lrrc66	-
chr13	59368302	59368475	Intergenic	Intergenic	277424	NM_00128	67269	Agtpbp1	1700020N1
chr7	97591652	97591750	non-coding	non-coding	410	NR_07343	233490	Crebzf	1110034C1
chr2	92886002	92886075	intron (NM	CpG	263	NM_00117	1E+08	Prdm11	8030443D0
chr10	1.18E+08	1.18E+08	Intergenic	Intergenic	-56492	NM_00116	17245	Mdm1	Arrd2|Mdr

chr2	3391652	3391900	intron (NM	CpG	482	NM_02272	64707	Suv39h2	4930507K2
chr8	1.27E+08	1.27E+08	intron (NM	intron (NM	42177	NM_02370	73647	Capn9	2200003B1
chr15	73253552	73253825	promoter-T	promoter-T	-67	NM_00113	14083	Ptk2	FAK|FRNK|
chr9	36950952	36951025	intron (NM	intron (NM	3919	NM_14895	208076	Pknox2	D230005H2
chr1	60399952	60400300	promoter-T	promoter-T	-90	NM_03001	77951	Cyp20a1	A930011N1
chr11	74584652	74584975	intron (NM	intron (NM	448	NM_02619	67493	Mettl16	2610100D0
chr7	25669527	25669800	promoter-T	promoter-T	-70	NM_02313	20085	Rps19	Dsk3
chrX	1.48E+08	1.48E+08	intron (NM	intron (NM	1237	NM_01676	15108	Hsd17b10	17bHSD10
chr15	52544077	52544350	exon (NM_	exon (NM_	213	NM_02721	69790	Med30	1810038N0
chr5	1.44E+08	1.44E+08	intron (NM	CpG	588	NM_17272	231868	E130309D0	A630028N2
chr7	50801252	50801700	Intergenic	L1MA9|LIN	-12935	NM_02129	12489	Cd33	Siglec-3|gp
chr4	1.32E+08	1.32E+08	Intergenic	Intergenic	-3567	NM_14490	230784	Sesn2	HI95|SEST2
chr18	37966252	37966375	exon (NM_	exon (NM_	249	NM_03358	93706	Pcdhgc3	PC43|Pcdh
chr11	59041352	59041600	5' UTR (NM	5' UTR (NM	188	NM_00113	11840	Arf1	-
chr19	32943702	32943850	Intergenic	Intergenic	111709	NM_00896	19211	Pten	2310035O0
chr8	26126727	26126950	promoter-T	promoter-T	556	NM_00127	11502	Adam9	AU020942
chr6	1.17E+08	1.17E+08	Intergenic	Intergenic	-8112	NM_17876	319776	Tmem72	C230095G0
chr16	33951002	33951375	Intergenic	Intergenic	15901	NM_00947	22247	Umps	1700095D2
chr10	1.21E+08	1.21E+08	intron (NM	intron (NM	9705	NM_02936	75612	Gns	2610016K1
chr2	1.19E+08	1.19E+08	exon (NM_	exon (NM_	335	NM_17226	228545	Vps18	9930024E1
chr11	1.18E+08	1.18E+08	intron (NM	intron (NM	532	NM_00103	72344	Usp36	2700002L0
chr7	13535227	13535325	Intergenic	Intergenic	-15937	NM_17873	243834	Zfp324	A930002M
chr5	1E+08	1E+08	promoter-T	promoter-T	103	NM_01669	50926	Hnrnpdl	AA407431|
chr17	28828227	28828325	promoter-T	promoter-T	-11	NM_00116	26416	Mapk14	CSBP2|Crk
chr9	88332052	88332150	intron (NM	B2_Mm2|S	1688	NM_17292	244962	Snx14	B830022K1
chr4	3866102	3866500	promoter-T	promoter-T	266	NM_00128	66433	Chchd7	1810049H2
chr2	60748752	60749100	intron (NM	intron (NM	-29431	NM_00114	56878	Rbms1	2600014B1
chr4	4720252	4720450	5' UTR (NM	5' UTR (NM	102	NM_17773	242291	Impad1	1110001C2
chr12	39506002	39506100	promoter-T	promoter-T	-794	NM_00796	14009	Etv1	ER81|Etsrp
chr2	91486302	91486575	exon (NM_	exon (NM_	3510	NM_00103	381410	Zfp408	Gm1011
chr5	1.39E+08	1.39E+08	promoter-T	promoter-T	-47	NM_00108	66096	Lamtor4	0910001L0
chr4	44313902	44313975	5' UTR (NM	5' UTR (NM	149	NM_01079	17279	Melk	AI327312|
chr8	60280052	60280300	intron (NM	intron (NM	-148324	NM_14473	108150	Galnt7	AI225872
chr4	1.29E+08	1.29E+08	promoter-T	promoter-T	-140	NM_00846	16650	Kpna6	IPOA7|Kpn
chr8	74222302	74222650	intron (NM	intron (NM	3223	NM_02818	72297	B3gnt3	2210008L1
chr7	30092452	30092675	intron (NM	intron (NM	3539	NM_01387	29861	Dpf1	Neud4
chr6	1.25E+08	1.25E+08	Intergenic	Intergenic	-48679	NM_19860	213522	Plekhg6	BC026778|
chr11	1.06E+08	1.06E+08	intron (NM	intron (NM	705	NM_02600	67163	Ccdc47	2610204L2
chr8	74739727	74739875	Intergenic	Intergenic	-3065	NM_00108	234421	Cib3	C730014M
chr6	1.38E+08	1.38E+08	intron (NM	RMER15|LT	4024	NM_17778	277898	Slc15a5	9830102E0
chr1	1.71E+08	1.71E+08	Intergenic	Intergenic	365332	NM_02328	66977	Nuf2	2410003C0
chr3	9020152	9020500	Intergenic	Intergenic	-15811	NM_00102	21985	Tpd52	AI043038|
chr19	14671602	14671875	intron (NM	CpG	735	NM_01160	21888	Tle4	5730411M
chr9	44773927	44774075	promoter-T	promoter-T	-318	NM_14540	140630	Ube4a	4732444G1
chr1	1.66E+08	1.66E+08	intron (NM	intron (NM	-5927	NM_00797	14067	F5	AI173222|
chr11	23987127	23987375	intron (NM	intron (NM	6556	NM_00115	14025	Bcl11a	2810047E1
chr17	44914477	44914600	intron (NM	CpG	418	NM_17865	109115	Supt3	2310066G2

chr11	95524202	95524375	Intergenic	MTEb|LTR	-3983	NM_00883	18673	Phb	Bap32
chr11	76759102	76759175	promoter-T	promoter-T	-20	NM_17864	104184	Blmh	AI035728|
chr18	44539552	44540025	promoter-T	promoter-T	-366	NM_02749	70640	Dcp2	2410015D2
chr1	87632027	87632100	intron (NM	intron (NM	1118	NM_17705	319997	A630001G2	-
chr3	9516702	9516775	intron (NM	intron (NM	93347	NM_13321	170753	Zfp704	C030026M
chr7	87706852	87707100	Intergenic	MER20|DN	-26971	NM_00104	12144	Blm	-
chr2	1.04E+08	1.04E+08	Intergenic	Intergenic	-23501	NM_00114	16909	Lmo2	Rbtn-2|Rbt
chr1	59539502	59539950	5' UTR (NM	5' UTR (NM	735	NM_00805	14369	Fzd7	Fz7
chr11	9090752	9091100	Intergenic	Intergenic	-1019	NM_17825	268379	Abca13	9830132L2
chr16	5029752	5029875	intron (NM	intron (NM	-16167	NM_13318	66049	Rogdi	0610011C1
chr3	37318977	37319575	promoter-T	promoter-T	74	NM_02134	57815	Spata5	2510048F2
chr8	1.29E+08	1.29E+08	Intergenic	Intergenic	166933	NM_02421	67952	Tomm20	1810060K0
chr4	1.45E+08	1.45E+08	Intergenic	Intergenic	-14740	NM_01161	21938	Tnfrsf1b	CD120b|TN
chr1	1.74E+08	1.74E+08	intron (NM	intron (NM	1636	NM_17859	21346	Tagln2	2700094C1
chr17	53828902	53829100	promoter-T	promoter-T	-361	NM_02823	72415	Sgol1	3300001M
chr10	80986902	80987025	promoter-T	promoter-T	7	NM_01030	14676	Gna15	G[a]15|Gal
chr2	1.68E+08	1.68E+08	promoter-T	promoter-T	491	NM_01007	13480	Dpm1	AI118379|A
chr9	1.07E+08	1.07E+08	5' UTR (NM	5' UTR (NM	412	NM_01970	56395	Tmem115	C78915|Pl6
chr7	29171127	29171275	3' UTR (NM	3' UTR (NM	6508	NM_02604	67224	Med29	2810405O2
chr1	1.93E+08	1.93E+08	promoter-T	promoter-T	337	NM_02976	76843	Dtl	2810047L0
chr9	55945527	55945625	intron (NM	intron (NM	7793	NM_01119	19200	Pstpip1	CD2BP1|de
chr7	3616777	3616950	TTS (NM_1	TTS (NM_1	579	NM_02720	69757	Leng1	1500001K1
chr2	92215652	92215775	5' UTR (NM	5' UTR (NM	317	NM_14512	18633	Pex16	-
chr19	46414227	46414600	Intergenic	Intergenic	-1263	NM_00116	67116	Cuedc2	3010002G0
chr16	22398877	22399075	intron (NM	intron (NM	40667	NM_02379	104156	Etv5	1110005E0
chr14	52639077	52639275	5' UTR (NM	5' UTR (NM	212	NM_00125	69890	Zfp219	2010302A1
chr1	1.46E+08	1.46E+08	promoter-T	promoter-T	63	NM_14599	214498	Cdc73	8430414L1
chr19	34262352	34262750	Intergenic	Intergenic	-4209	NM_02968	76630	Stambpl1	1700095N2
chr2	1.67E+08	1.67E+08	Intergenic	Intergenic	-18948	NM_01983	56336	B4galt5	9430078I07
chr2	72059552	72060000	intron (NM	intron (NM	-63918	NM_02305	65964	Zak	AV006891|
chr14	48750927	48751525	intron (NM	intron (NM	10682	NM_03360	93834	Peli2	AW047589
chr8	1.25E+08	1.25E+08	intron (NM	intron (NM	-5186	NM_00780	13057	Cyba	b558|nmf3
chr6	1.49E+08	1.49E+08	intron (NM	intron (NM	246	NM_00111	232566	Amn1	5830467E0
chr6	40841102	40841225	Intergenic	Intergenic	-3670	NM_13929	194357	Moxd2	Dbhl1
chr5	1.39E+08	1.39E+08	promoter-T	promoter-T	664	NM_00856	17220	Mcm7	AI747533|
chr11	1.2E+08	1.2E+08	Intergenic	Intergenic	11039	NM_00108	208092	Chmp6	2400004G0
chr12	82147952	82148075	intron (NM	intron (NM	20218	NM_02231	64075	Smoc1	2600002F2
chr1	10222277	10222525	intron (NM	CpG	350	NM_00110	211673	Arfgef1	ARFGEP1|E
chr1	52290252	52290425	promoter-T	promoter-T	-262	NM_00111	14660	Gls	6330442B1
chr6	39728527	39728825	Intergenic	Intergenic	32259	NM_01027	14548	Mrps33	AI841153|
chr1	1.3E+08	1.3E+08	intron (NM	L1M4|LINE	4469	NM_00108	226413	Lct	Gm100|LA
chr9	1.23E+08	1.23E+08	intron (NM	intron (NM	-17212	NM_03069	83493	Sacm1l	SAC1|Sac1
chr17	34091152	34093150	promoter-T	promoter-T	435	NM_01129	20084	Rps18	H-2Ke3|H2
chr2	1.67E+08	1.67E+08	promoter-T	promoter-T	96	NM_02381	76367	Trp53rk	2810408M
chr2	1.68E+08	1.68E+08	Intergenic	Intergenic	-6751	NM_01120	19246	Ptpn1	PTP-1B|PT
chr15	68547952	68548025	Intergenic	Intergenic	-210862	NM_01015	13992	Khdrbs3	Etle|SLM-2
chr5	1.3E+08	1.3E+08	promoter-T	promoter-T	-415	NM_01036	110006	Gusb	AI747421|

chr17	48463652	48463775	3' UTR (NM	3' UTR (NM	8226	NM_17708	320148	B430306N(	Treml6
chr11	1.15E+08	1.15E+08	Intergenic	Intergenic	17758	NM_17075	217303	Cd300a	B230315M
chr6	90704977	90705150	intron (NM	intron (NM	-38540	NM_00113	232227	Iqsec1	AW561907
chr15	76731202	76731300	exon (NM_	exon (NM_	880	NM_02553	66398	Commd5	2310065H(
chr12	8674502	8674600	Intergenic	Intergenic	-6389	NM_00116	80913	Pum2	5730503J2
chr9	85269027	85269100	Intergenic	Intergenic	-48306	NM_00116	212943	Fam46a	D930050G(
chr5	1.37E+08	1.37E+08	Intergenic	Intergenic	-9013	NM_01196	26433	Plod3	AI414586|
chr7	1.49E+08	1.49E+08	promoter-T	promoter-T	-249	NM_00874	18218	Dusp8	5530400B(
chr6	39155202	39155275	intron (NM	intron (NM	1534	NM_00103	338523	Kdm7a	A630082K2
chr12	86831127	86831550	Intergenic	Intergenic	16487	NM_01023	14281	Fos	D12Rfj1|c-
chr19	41344577	41344700	Intergenic	ID2|SINE|I	-6144	NM_13335	107358	Tm9sf3	1810073M
chr3	1.08E+08	1.08E+08	intron (NM	intron (NM	14419	NM_01997	20661	Sort1	2900053A1
chr1	1.93E+08	1.93E+08	Intergenic	Intergenic	-42051	NM_00749	11910	Atf3	LRG-21
chr4	1.36E+08	1.36E+08	promoter-T	promoter-T	44	NM_00127	74326	Hnrnpr	2610003J0
chr4	59818752	59818850	5' UTR (NM	5' UTR (NM	279	NM_17246	209131	Snx30	4732481H1
chr1	63527027	63527100	intron (NM	intron (NM	34585	NM_01178	23792	Adam23	AW046396
chr14	79132127	79132250	Intergenic	CpG	-7292	NM_00108	380921	Dgkh	5930402B(
chr2	35098502	35098825	Intergenic	RMER4B|L	-13216	NM_00120	227753	Gsn	ADF
chr5	1.46E+08	1.46E+08	intron (NM	intron (NM	21261	NM_02943	75788	Smurf1	4930431E1
chr1	74708377	74708550	5' UTR (NM	5' UTR (NM	135	NM_00101	67534	Ttll4	4632407P(
chr16	18643702	18643775	Intergenic	Intergenic	-13707	NM_21361	18951	5-Sep	Cdcrel-1|C
chr8	1.12E+08	1.12E+08	intron (NM	CpG	201	NM_00108	52335	Atxn1l	AW558552
chr6	1.19E+08	1.19E+08	intron (NM	CpG	28488	NM_19798	68465	Adipor2	1110001I1
chr11	68780877	68781050	promoter-T	promoter-T	-670	NM_02804	71998	Slc25a35	1810012H1
chr15	3587102	3587275	Intergenic	Intergenic	-53836	NM_01028	14600	Ghr	GHBP|GHR
chr4	34833952	34834175	Intergenic	CpG	-3866	NM_01388	30046	Zfp292	5730450D(
chr11	1.16E+08	1.16E+08	intron (NM	intron (NM	6878	NM_00116	217344	Rhbdf2	4732465I1
chr10	10072027	10072325	intron (NM	intron (NM	119936	NM_00112	215772	Adgb	9130014G2
chr19	5511152	5511275	promoter-T	promoter-T	-724	NM_00102	225861	Snx32	B930037P1
chr15	77662552	77662625	intron (NM	intron (NM	10017	NM_02241	17886	Myh9	Fltn|Myhn
chr14	75347502	75347575	5' UTR (NM	5' UTR (NM	146	NM_00125	380916	Lrch1	4832412D1
chr5	91360427	91360550	intron (NM	GC_rich|Lo	266	NM_02678	665563	Mthfd2l	1110019K2
chr13	42148502	42148600	intron (NM	intron (NM	1161	NM_00777	110521	Hivep1	Cryabp1
chr18	52946052	52946225	intron (NM	intron (NM	18340	NM_00119	67847	Sncaip	2810407O1
chr3	1.54E+08	1.54E+08	promoter-T	promoter-T	-196	NR_02809	19352	Rabggtb	-
chr4	93002277	93002625	promoter-T	promoter-T	-249	NM_02695	69136	Tusc1	2200001D1
chr11	70469202	70469500	Intergenic	CpG-2168	-1199	NM_01107	18643	Pfn1	Pfn
chr3	60408227	60408300	intron (NM	intron (NM	103089	NM_00125	56758	Mbnl1	Mbnl|mKIA
chr7	1.26E+08	1.26E+08	intron (NM	MER3|DNA	-24349	NM_00994	12850	Coq7	clk-1
chr1	88424627	88424950	intron (NM	CpG-365	1477	NM_00897	19231	Ptma	Thym
chr6	83003527	83003725	exon (NM_	exon (NM_	939	NM_01975	64704	Htra2	AI481710|
chr5	1.36E+08	1.36E+08	exon (NM_	exon (NM_	152	NM_14600	215160	Rhbdd2	0610011L1
chr1	78484977	78485050	intron (NM	intron (NM	459	NR_10275	23874	Farsb	C76708|Fa
chr4	1.32E+08	1.32E+08	intron (NM	intron (NM	1816	NM_00103	230787	Themis2	ICB-1
chr1	1.73E+08	1.73E+08	promoter-T	promoter-T	39	NM_00948	22278	Usf1	bHLHb11
chr12	70285602	70285800	promoter-T	promoter-T	556	NM_14603	217664	Mgat2	AA407964|
chr2	1.67E+08	1.67E+08	intron (NM	intron (NM	-11377	NM_17075	263876	Spata2	AI504642|

chr3	1.27E+08	1.27E+08	promoter-T	promoter-T	129	NM_13859	28036	Larp7	C330027G(
chr11	4847202	4847400	promoter-T	promoter-T	-223	NM_00124	11764	Ap1b1	Adtb1|b2b
chr5	3802452	3802700	promoter-T	promoter-T	548	NM_00128	70797	Ankib1	2310061P2
chr15	76755902	76755975	promoter-T	promoter-T	-60	NM_00116	239546	Zfp647	6030449J2
chr14	62207177	62207275	Intergenic	Lx7|LINE|L	-9837	NM_00116	66597	Trim13	3110001L1
chr3	1.43E+08	1.43E+08	5' UTR (NM	5' UTR (NM	205	NM_17865	109333	Pkn2	6030436C2
chr17	64581002	64581250	Intergenic	Intergenic	100097	NM_14485	224938	Pja2	AI447901|/
chr13	23623277	23623450	TTS (NM_1	TTS (NM_1	450	NM_15317	69386	Hist1h4h	1700024H(
chr13	21253402	21253600	Intergenic	Intergenic	-18299	NM_00905	19720	Trim27	AW538890
chr9	16390302	16390375	Intergenic	Intergenic	-207663	NM_00108	270120	Fat3	9430076A(
chr5	1.35E+08	1.35E+08	intron (NM	intron (NM	22994	NM_00108	57080	Gtf2ird1	1700012P1
chr10	62706577	62706775	5' UTR (NM	5' UTR (NM	131	NM_02610	67345	Herc4	1700056O1
chr18	65590077	65590150	promoter-T	promoter-T	-538	NM_17283	240354	Malt1	A630046N1
chr6	1.21E+08	1.21E+08	intron (NM	intron (NM	1165	NM_15351	94044	Bcl2l13	BCL-RAMB(
chr13	40818627	40818850	intron (NM	CpG-3771	7074	NM_00112	21418	Tfap2a	AP-2|AP2a
chr2	27569777	27569975	intron (NM	intron (NM	3773	NM_00129	20181	Rxra	9530071D1
chr9	42122477	42122775	Intergenic	L1ME3B|LI	-50243	NM_17276	235293	Sc5d	A830037K(
chr6	49333427	49333650	intron (NM	intron (NM	-12065	NM_02991	77485	Stk31	C330007K2
chr5	1.36E+08	1.36E+08	intron (NM	intron (NM	684	NM_00974	12054	Bcl7b	-
chr9	79640902	79641050	promoter-T	promoter-T	261	NM_13371	69981	Tmem30a	2010200I2
chr8	19784627	19785000	Intergenic	CpG	55237	NM_00103	626415	4930467E2	ENSMUSG(
chr1	1.33E+08	1.33E+08	exon (NM_	exon (NM_	372	NM_17869	226418	Yod1	9930028C2
chr9	70390727	70390850	exon (NM_	exon (NM_	111	NM_02569	66660	Sltm	5730455C(
chr12	83028802	83029000	intron (NM	intron (NM	67884	NM_01881	54604	Pcnx	2900024E2
chr2	1.04E+08	1.04E+08	intron (NM	intron (NM	456	NM_15312	98956	Nat10	AI429152
chr16	20756202	20756350	Intergenic	RCHARR1|I	-21692	NM_00937	21832	Thpo	Mgdf|Ml|N
chr4	57968377	57969775	exon (NM_	exon (NM_	207	NM_01166	22166	Txn1	ADF|AW55
chr13	36748527	36748675	Intergenic	Intergenic	77745	NM_15352	68404	Nrn1	0710008J2
chr16	22857927	22858075	promoter-T	promoter-T	83	NM_00119	67838	Dnajb11	1810031F2
chr10	1.12E+08	1.12E+08	Intergenic	Intergenic	-25706	NM_17827	353025	Caps2	D630005B(
chr11	1.2E+08	1.2E+08	promoter-T	promoter-T	-122	NM_00103	192173	Fam195b	-
chr7	28115552	28115675	intron (NM	intron (NM	3054	NM_00111	108075	Ltbp4	2310046A1
chr15	93171027	93171550	Intergenic	Intergenic	-3922	NM_02418	67057	Yaf2	2810021M
chr15	31297077	31297150	exon (NM_	exon (NM_	401	NM_00116	67434	Ankrd33b	0610012A(
chr13	51836152	51836550	intron (NM	intron (NM	52765	NM_00128	20354	Sema4d	CD100|Ser
chr12	96695677	96696100	Intergenic	Intergenic	-234548	NM_20151	399558	Flrt2	-
chr11	72020627	72020825	promoter-T	promoter-T	-330	NM_02655	52700	Txndc17	4831443O2
chr17	47733802	47734075	intron (NM	intron (NM	3522	NM_00763	12445	Ccnd3	9230106B(
chr1	1.93E+08	1.93E+08	intron (NR_	CpG	392	NR_03757	52477	Angel2	2610307I2
chr4	1.19E+08	1.19E+08	promoter-T	promoter-T	-295	NM_01173	22608	Ybx1	1700102N1
chr12	42290777	42291000	intron (NM	intron (NM	-78244	NM_01073	16981	Lrrn3	NLRR-3
chr11	34596677	34596825	intron (NM	intron (NM	656	NM_03337	94176	Dock2	AI662014|/
chr11	52065877	52066075	TTS (NM_0	TTS (NM_0	20479	NM_01154	21402	Skp1a	15kDa|261
chr11	32442677	32442800	intron (NM	intron (NM	9472	NM_00928	20868	Stk10	Gek1|Lok|
chr1	1.8E+08	1.8E+08	exon (NM_	exon (NM_	177	NM_00742	11566	Adss	AI314886|/
chr7	34981277	34981625	exon (NM_	exon (NM_	215	NM_02654	68079	Pdcd2l	6030457N1
chr10	41991202	41991375	intron (NM	intron (NM	5260	NM_01974	56484	Foxo3	1110048B1

chr16	20672527	20673425	intron (NM	CpG	154	NM_00100	208643	Eif4g1	E030015G2
chr4	53726677	53727175	promoter-T	promoter-T	-128	NM_13930	246179	Fktn	D830030O1
chr2	66018052	66018175	Intergenic	Intergenic	-55263	NM_01573	14425	Galnt3	-
chr11	83942802	83943025	intron (NM	intron (NM	157	NM_17256	217031	Tada2a	AV319371|
chr13	46792352	46792425	intron (NM	RMER20B|	27497	NM_00103	97863	C78339	-
chr4	33118552	33118700	promoter-T	promoter-T	226	NM_01958	56228	Ube2j1	0710008M
chrX	1.34E+08	1.34E+08	intron (NM	CpG	153	NM_00108	67062	Slc25a53	2310046F1
chr16	16573627	16573700	Intergenic	L1_Mus3|L	-13374	NM_13923	224014	Fgd4	9030023J0
chr5	24728677	24728925	promoter-T	promoter-T	90	NM_14490	231050	Galnt11	A430075I0
chr4	1.56E+08	1.56E+08	Intergenic	CpG	-1622	NM_19830	231003	Klhl17	AL022703|
chr19	4120977	4121500	intron (NM	intron (NM	337	NM_00127	11632	Aip	AA408703|
chr10	1.26E+08	1.26E+08	Intergenic	B1_Mur1|S	73451	NM_02685	68876	Xrcc6bp1	1110068E0
chr6	1.29E+08	1.29E+08	Intergenic	Intergenic	-19295	NM_05310	93694	Clec2d	Clr-b|Clrb|
chr1	1.73E+08	1.73E+08	intron (NM	intron (NM	21150	NM_17706	320078	Olfml2b	1110018N0
chr8	82163877	82164175	intron (NM	CpG	451	NM_00108	73945	Otud4	4930431L1
chr4	1.18E+08	1.18E+08	exon (NM_	exon (NM_	-9475	NM_00116	20441	St3gal3	ST3GalIII|S
chr5	1.11E+08	1.11E+08	promoter-T	promoter-T	-47	NM_00125	71782	Ankle2	1110001J1
chr14	22003202	22003275	intron (NM	Lx6|LINE|L	107864	NM_00124	11534	Adk	2310026J0
chr3	42212902	42213175	Intergenic	Intergenic	666498	NM_02727	73852	D3Ertd751e	2810009O1
chr18	10646652	10646825	intron (NM	intron (NM	28944	NM_00922	20641	Snrpd1	AA407109|
chr8	27099502	27099600	intron (NM	intron (NM	5042	NM_02903	74653	Pomk	4930444A0
chr15	99494802	99495225	Intergenic	Intergenic	-6136	NM_00959	11419	Asic1	AI843610|A
chr10	67268577	67268850	Intergenic	Intergenic	106697	NM_17867	216049	Zfp365	AI839779|A
chr5	1.16E+08	1.16E+08	intron (NM	intron (NM	8723	NM_00770	12704	Cit	C030025P1
chr19	32560327	32560525	5' UTR (NM	5' UTR (NM	167	NM_01079	17330	Minpp1	AA408516
chr10	57252027	57252275	intron (NM	CpG	184	NM_01976	56442	Serinc1	1500011D1
chr10	13273052	13273125	promoter-T	promoter-T	-140	NM_00116	56535	Pex3	1700014F1
chr2	30107102	30107275	intron (NM	intron (NM	13899	NM_17772	241296	Lrrc8a	Lrrc8|mKIA
chr4	1.49E+08	1.49E+08	intron (NM	intron (NM	10940	NM_00108	110208	Pgd	0610042A0
chr18	75229752	75229975	intron (NM	intron (NM	51437	NM_02772	69190	Dym	1810041M
chr16	23675902	23676050	Intergenic	Intergenic	65971	NM_02338	67775	Rtp4	5830458K1
chr17	3114227	3114350	promoter-T	promoter-T	-684	NM_13412	106583	Scaf8	A630086M
chr6	53017852	53018000	intron (NM	GC_rich|Lo	692	NM_17340	231986	Jazf1	AI591476|(
chr2	1.3E+08	1.3E+08	promoter-T	promoter-T	-338	NM_00898	19262	Ptpra	Ptpa|Ptpal
chr17	26078777	26078850	TTS (NM_0	TTS (NM_0	121	NM_00129	14755	Pigq	Gpi1|Gpi1h
chr10	81022202	81022325	promoter-T	promoter-T	74	NM_00127	14797	Aes	AL024115|
chr9	57612527	57612925	intron (NM	CpG	941	NM_00771	102414	Clk3	AI256811
chr6	38303227	38303325	intron (NM	intron (NM	1327	NM_02886	78781	Zc3hav1	1200014N1
chr15	76161652	76161825	promoter-T	promoter-T	14	NM_01033	14731	Gpaa1	C80044|m(
chr7	1.34E+08	1.34E+08	promoter-T	promoter-T	23	NM_17736	244216	Zfp771	G630024C0
chr12	85616127	85616550	Intergenic	RMER19B|	-9908	NM_02988	77219	Ptgr2	1810016I24
chr1	1.82E+08	1.82E+08	Intergenic	Intergenic	-32795	NM_00112	19165	Psen2	ALG-3|Ad4
chr13	97441427	97441625	promoter-T	promoter-T	-635	NM_00825	15357	Hmgcr	HMG-CoAR
chr3	1.38E+08	1.38E+08	intron (NM	GC_rich|Lo	412	NM_00103	70604	Dnajb14	5730496F1
chr8	90885552	90885850	intron (NM	CpG	389	NM_01204	26992	Brd7	BP75|CELT
chrX	10659327	10659450	Intergenic	MIRb|SINE	-216995	NM_00111	1E+08	Gm14483	OTTMUSG0
chr3	1E+08	1E+08	Intergenic	Intergenic	-13573	NM_00114	74645	Fam46c	4930431B0

chr5	1.23E+08	1.23E+08	Intergenic	Intergenic	-45329	NR_02783{	11938	Atp2a2	9530097L1
chr2	70542727	70542875	Intergenic	Intergenic	43235	NM_02735	70231	Gorasp2	0610011A(
chr17	46419327	46419400	5' UTR (NN	5' UTR (NN	612	NM_00125	74094	Tjap1	0610041D1
chr11	9569552	9569675	intron (NM	intron (NM	477668	NM_17825	268379	Abca13	9830132L2
chr10	1.17E+08	1.17E+08	5' UTR (NN	5' UTR (NN	187	NM_02657	64050	Yeats4	4930573H1
chr6	16837702	16837850	intron (NM	intron (NM	10665	NM_03119	21426	Tfec	BB107417|
chr7	54065802	54065925	intron (NM	Lx8|LINE|L	13822	NM_00129	14884	Gtf2h1	62kDa|AW
chr1	1.84E+08	1.84E+08	5' UTR (NN	5' UTR (NN	203	NM_17337	209456	Trp53bp2	53BP2|AI7
chr10	1.11E+08	1.11E+08	Intergenic	Intergenic	33996	NM_00934	21664	Phlda1	DT1P1B11|
chr19	5388902	5389075	promoter-T	promoter-T	-285	NM_01688	20227	Sart1	U5-110K
chr18	33459277	33459475	Intergenic	Intergenic	-85906	NM_13377	170459	Stard4	4632419C1
chr17	28572102	28572175	intron (NM	intron (NM	50956	NM_01022	14229	Fkbp5	D17Ertd59
chr1	55145152	55145425	promoter-T	promoter-T	296	NM_00830	15528	Hspe1	10kDa|Hsp
chr14	1.06E+08	1.06E+08	intron (NM	intron (NM	42048	NM_00119	76273	Ndfip2	0710001O2
chr1	71460077	71460175	intron (NM	intron (NM	1358	NM_17521	74591	Abca12	4832428G1
chr4	1.25E+08	1.25E+08	Intergenic	Intergenic	-114813	NM_15315	230738	Zc3h12a	BC036563|
chr12	55292227	55292450	intron (NM	intron (NM	12523	NM_02813	112407	Egln3	2610021G(
chr1	1.09E+08	1.09E+08	promoter-T	promoter-T	-24	NM_00919	20479	Vps4b	8030489C1
chr1	87960677	87961150	promoter-T	promoter-T	-281	NM_02735	70247	Psmd1	2410026J1
chr13	75962702	75963050	Intergenic	Intergenic	-14458	NM_05310	93692	Glrx	C86710|D1
chr12	86939802	86940050	promoter-T	promoter-T	-129	NM_00120	81703	Jdp2	Jundm2|Ju
chr3	1.16E+08	1.16E+08	Intergenic	Intergenic	-11801	NM_00117	229776	Cdc14a	A830059A1
chr3	95117352	95117525	Intergenic	Intergenic	-1736	NM_02978	76893	Cers2	0610013I1
chr5	1.51E+08	1.51E+08	promoter-T	promoter-T	-963	NM_13389	100637	N4bp2l1	2410024N1
chr16	57391577	57391750	intron (NM	LSU-rRNA_	38273	NM_00104	78749	Filip1l	4631422O(
chr12	81744602	81744700	promoter-T	promoter-T	197	NM_00795	13877	Erh	Mer|Prei1
chr5	73648652	73648725	promoter-T	promoter-T	-831	NM_02819	72313	Fryl	2010313D2
chr2	24790302	24790475	intron (NR_	CpG	413	NR_02787(	215705	Arrdc1	AI957342|
chr2	1.3E+08	1.3E+08	intron (NM	CpG	112	NM_19817	380601	Fastkd5	C78212|m
chr3	80313327	80313450	Intergenic	Intergenic	293325	NM_01354	14800	Gria2	GluA2|Glu
chr6	1.24E+08	1.24E+08	Intergenic	Intergenic	-13886	NM_15350	232370	Clstn3	CSTN3|Cs3
chr11	23671277	23671375	promoter-T	promoter-T	-356	NM_00904	19696	Rel	c-Rel
chr3	51279702	51279925	TTS (NM_0	TTS (NM_0	-8075	NM_01685	19338	Rab33b	-
chr5	86507752	86507975	intron (NM	MIR|SINE|	7010	NM_01999	56792	Stap1	AI586015|
chr12	1.04E+08	1.04E+08	promoter-T	promoter-T	68	NM_17714	320351	Tmem251	D230037D(
chr6	72850302	72850500	promoter-T	promoter-T	-428	NM_01971	74287	Kcmf1	1700094M
chr3	1.09E+08	1.09E+08	intron (NM	intron (NM	21412	NM_02050	57257	Vav3	A530094I0
chrX	20265452	20265525	promoter-T	promoter-T	-131	NM_01104	18555	Cdk16	Crk5|Pctai
chr17	24488377	24488450	promoter-T	promoter-T	-555	NM_01385	27410	Abca3	1810036E2
chr3	79336727	79336800	intron (NM	intron (NM	34838	NM_00116	329679	Fnip2	D630023B1
chr5	1.41E+08	1.41E+08	intron (NM	intron (NM	25207	NM_17340	231842	Amz1	5330426I0
chr2	64958227	64958450	intron (NM	intron (NM	-97515	NM_01671	50915	Grb14	AI505286
chr19	43599727	43599800	promoter-T	promoter-T	-668	NM_01032	14718	Got1	AI789014|
chr14	68545977	68546250	intron (NM	intron (NM	5516	NM_17778	68813	Dock5	1110060D(
chr5	1.24E+08	1.24E+08	promoter-T	promoter-T	79	NM_00103	330189	Tmem120b	-
chr1	1.82E+08	1.82E+08	intron (NM	CpG	182	NM_00741	11545	Parp1	5830444G2
chr1	36614327	36614575	intron (NM	CpG	775	NM_00112	20353	Sema4c	AI426163|

chr14	28628802	28629075	intron (NM	intron (NM	193310	NM_17781	238988	Erc2	6430531D(
chr11	53704702	53704925	exon (NM_	exon (NM_	392	NM_01139	20520	Slc22a5	Lstpl|Octn:
chr13	1.14E+08	1.14E+08	exon (NM_	exon (NM_	334	NM_13079	170625	Snx18	Snag1
chr6	67133677	67133825	Intergenic	Intergenic	-83222	NM_00111	66870	Serbp1	1200009K1
chr13	42145427	42145500	Intergenic	Intergenic	-1927	NM_00777	110521	Hivep1	Cryabp1
chr5	34915952	34916125	promoter-T	promoter-T	-325	NM_01345	11518	Add1	AI256389
chr2	1.28E+08	1.28E+08	intron (NM	intron (NM	9477	NM_00128	12125	Bcl2l11	1500006F2
chr6	72389027	72389525	non-coding	non-coding	276	NM_14556	232087	Mat2a	D630045P1
chr15	5297027	5297200	Intergenic	Intergenic	-102926	NM_00896	19219	Ptger4	EP4|Ptgere
chr8	1.08E+08	1.08E+08	promoter-T	promoter-T	-708	NM_02653	68051	Nutf2	2700067I0:
chr9	48414427	48414525	Intergenic	Intergenic	-1294	NM_01092	18113	Nnmt	-
chr4	67061277	67061475	Intergenic	Intergenic	572531	NM_02129	21898	Tlr4	Lps|Ly87|F
chr16	48993427	48993700	promoter-T	promoter-T	662	NM_00111	224170	Dzip3	2310047C(
chr2	1.63E+08	1.63E+08	Intergenic	Intergenic	18188	NM_02951	76080	Ttpal	3110080A(
chr7	52147777	52148000	promoter-T	promoter-T	-152	NM_02936	75613	Med25	2610034E1
chr7	26948352	26948450	Intergenic	Intergenic	-10027	NM_01000	13094	Cyp2b9	Cyp2b
chr1	43155152	43155825	promoter-T	promoter-T	-21	NM_00101	73122	Tgfbrap1	3110018K1
chr11	1.1E+08	1.1E+08	promoter-T	promoter-T	-712	NM_02188	19084	Prkar1a	1300018C2
chr7	20058377	20058500	Intergenic	B1F|SINE|/	-14595	NM_17227	232944	Mark4	2410090P2
chr2	90929302	90929475	Intergenic	Intergenic	-7566	NM_01135	20375	Spi1	Dis-1|Dis1
chr2	74663777	74663875	promoter-T	promoter-T	-43	NM_01680	53375	Mtx2	1500012G(
chr10	1.21E+08	1.21E+08	TTS (NM_0	TTS (NM_0	312	NM_01978	56480	Tbk1	1200008B(
chr5	1.31E+08	1.31E+08	intron (NM	intron (NM	14233	NM_02785	71667	Tmem248	0610007L0
chr14	12607802	12607975	intron (NM	intron (NM	221821	NM_00898	19270	Ptprg	5430405N1
chr10	67749577	67749650	Intergenic	Intergenic	-8139	NM_02359	71371	Arid5b	4930580B1
chr12	76882802	76883350	Intergenic	Intergenic	-36229	NM_00100	319565	Syne2	6820443O(
chr9	60207852	60208125	intron (NM	intron (NM	150854	NM_00104	207596	Thsd4	ADAMTSL-(
chr5	44218177	44218675	intron (NM	intron (NM	8294	NM_00976	12182	Bst1	114/A10|A
chrX	72627827	72628000	intron (NM	intron (NM	175	NM_02612	67391	Fundc2	4833415N2
chr1	1.73E+08	1.73E+08	Intergenic	Intergenic	-11030	NM_02317	80915	Dusp12	1190004O1
chr3	1.02E+08	1.02E+08	intron (NM	intron (NM	1163	NM_00103	242126	Slc22a15	2610034P2
chr3	1.03E+08	1.03E+08	Intergenic	CT-rich|Lov	-21464	NM_00116	22094	Tshb	-
chr10	20866577	20866775	intron (NM	(T)n|Simpl	14114	NM_00119	17863	Myb	AI550390|
chr6	43616002	43616400	promoter-T	promoter-T	-27	NM_01386	29807	Tpk1	-
chr9	1.09E+08	1.09E+08	promoter-T	promoter-T	-561	NM_00892	19087	Prkar2a	1110061A2
chr6	1.13E+08	1.13E+08	Intergenic	MIRb|SINE	-85861	NM_00108	18430	Oxtr	OTR
chr7	1.49E+08	1.49E+08	promoter-T	promoter-T	398	NM_01687	54006	Deaf1	AU042387
chr5	1.04E+08	1.04E+08	intron (NM	intron (NM	10859	NM_00108	17355	Aff1	9630032B(
chr2	71174502	71174675	intron (NM	intron (NM	31023	NM_17243	78830	Slc25a12	2610002D(
chr6	88396602	88396750	promoter-T	promoter-T	-143	NM_02306	65967	Eefsec	Selb|sec
chr4	1.47E+08	1.47E+08	promoter-T	promoter-T	27	NM_01084	17769	Mthfr	AI323986
chr10	80760977	80761200	intron (NM	intron (NM	5380	NM_00114	18717	Pip5k1c	AI115456|/
chr4	6381502	6381600	promoter-T	promoter-T	-133	NM_01094	18201	Nsmaf	AA959567|
chr9	70132427	70132575	intron (NM	intron (NM	77344	NM_18107	71602	Myo1e	2310020N2
chr7	1.52E+08	1.52E+08	Intergenic	Intergenic	-89883	NM_00763	12443	Ccnd1	AI327039|(
chr1	82722027	82722150	intron (NM	intron (NM	595	NM_02940	75734	Mff	5230400G2
chr18	39098952	39099250	intron (NM	L1MB3|LIN	-20748	NM_01019	14164	Fgf1	Dffrx|Fam|

chr9	1.19E+08	1.19E+08	intron (NR_	PB1D10|SI	11766	NM_00129	18799	Plcd1	AW212592
chr8	1.14E+08	1.14E+08	intron (NM	intron (NM	485	NM_00791	13680	Ddx19a	DBP5|Ddx1
chr5	1.49E+08	1.49E+08	Intergenic	Intergenic	-24624	NM_01190	24109	Ubl3	AW108023
chr1	1.57E+08	1.57E+08	Intergenic	Intergenic	24590	NM_01050	15939	Ier5	-
chr9	20297952	20298225	promoter-T	promoter-T	-898	NM_00111	235028	Zfp426	2900057C0
chr4	1.33E+08	1.33E+08	intron (NM	intron (NM	2553	NM_01020	14191	Fgr	-
chr17	44834252	44834350	intron (NR_	intron (NR_	38824	NR_073425	12393	Runx2	AML3|Cbf|
chr2	1.19E+08	1.19E+08	Intergenic	Intergenic	-31048	NM_02657	68142	Ino80	2310079N1
chr9	41724952	41725350	Intergenic	Intergenic	207221	NM_01143	20660	Sorl1	2900010L1
chr10	79017627	79017800	intron (NM	CpG	123	NM_02742	70427	Mier2	2700087H1
chr4	1.34E+08	1.34E+08	3' UTR (NM	3' UTR (NM	-1383	NM_08055	73723	Sh3bgrl3	1110004L0
chr1	65159452	65159875	intron (NM	intron (NM	-9726	NM_00777	12964	Cryga	Cryg-4|DG
chr8	12393652	12393725	intron (NR_	intron (NR_	-1831	NM_00923	20664	Sox1	BB176347|
chr2	1.56E+08	1.56E+08	promoter-T	promoter-T	44	NM_02594	67067	Romo1	2010100O1
chr1	13650327	13650450	intron (NM	CpG	202	NM_14538	212442	Lactb2	Cgi-83|E43
chr11	88017352	88017525	promoter-T	promoter-T	-452	NM_02417	64656	Mrps23	D11Bwg11
chr1	52111652	52112000	intron (NM	intron (NM	46738	NM_01148	20849	Stat4	-
chr6	88851927	88852125	promoter-T	promoter-T	-219	NM_01190	24100	Tpra1	40kDa|Gpr
chr3	1.06E+08	1.06E+08	promoter-T	promoter-T	-156	NM_13386	99712	Cept1	9930118K0
chr11	88921552	88921825	promoter-T	promoter-T	374	NM_01950	56077	Dgke	C87606|DA
chr6	86559227	86559450	Intergenic	RSINE1|SIN	-18830	NM_02641	67855	Asprv1	2300003P2
chr2	3341002	3341125	promoter-T	promoter-T	-340	NM_14611	227525	Dclre1c	9930121L0
chr9	40383277	40383400	Intergenic	Intergenic	-110709	NM_13373	71566	Clmp	9030425E1
chr17	27974227	27974475	Intergenic	B1F2|SINE	-2681	NM_01143	20630	Snrpc	Snrp1c|U1
chr6	85873002	85873150	intron (NM	intron (NM	7363	NM_00117	69786	Tprkb	0610033G2
chr6	1.09E+08	1.09E+08	intron (NR_	intron (NR_	-2110	NM_01149	20893	Bhlhe40	Bhlhb2|C1
chr7	26793127	26793450	Intergenic	IAP-d-int|L	-53226	NM_00781	13089	Cyp2b13	-
chr19	3768252	3768400	5' UTR (NM	5' UTR (NM	905	NM_00116	225888	Suv420h1	AA117471|
chr12	72415552	72415750	intron (NM	intron (NM	4780	NM_00119	59036	Dact1	4921528D1
chr7	1.38E+08	1.38E+08	promoter-T	promoter-T	-743	NM_00127	11907	Ate1	AI225793|
chr17	28445427	28445825	Intergenic	Intergenic	-4849	NR_028297	72775	Fance	2810451D0
chr8	17401902	17402100	intron (NM	intron (NM	133384	NM_05317	94109	Csmd1	B930082H0
chr1	1.34E+08	1.34E+08	intron (NM	intron (NM	1086	NM_00119	74137	Nuak2	1200013B2
chr6	85245952	85246150	intron (NM	intron (NM	37365	NM_17863	94282	Sfxn5	C230001H0
chr1	74274027	74274100	Intergenic	Intergenic	-9061	NM_02971	76709	Arpc2	2210023N0
chr1	6205127	6205325	intron (NM	CpG	483	NM_00982	12421	Rb1cc1	2900055E0
chr4	45419652	45419750	intron (NM	intron (NM	1937	NM_00100	230125	Slc25a51	9130208E0
chr2	1.26E+08	1.26E+08	intron (NM	intron (NM	23676	NM_00108	241633	Atp8b4	A530043E1
chr5	1.38E+08	1.38E+08	promoter-T	promoter-T	-724	NM_00110	83701	Srrt	2810019G0
chr13	65342577	65342775	Intergenic	CpG	-35652	NM_17529	97895	Nlrp4f	C330026N0
chr12	1.04E+08	1.04E+08	promoter-T	promoter-T	269	NM_00119	27277	Golga5	Ret-II
chr19	36993552	36993800	exon (NM_	exon (NM_	413	NM_02826	72514	Fgfbp3	2610306H1
chr6	95668302	95668400	intron (NM	CpG	489	NM_01150	20917	Suclg2	AF171077|
chr6	87492052	87492250	Intergenic	Intergenic	-8898	NM_00128	232201	Arhgap25	A130039I2
chr1	45943652	45943800	Intergenic	MIRb|SINE	38713	NM_01691	53945	Slc40a1	Dusg|Fpn1
chr10	10168852	10169325	intron (NM	intron (NM	23024	NM_00112	215772	Adgb	9130014G2
chr8	1.07E+08	1.07E+08	Intergenic	Intergenic	12058	NM_19799	72361	Ces2g	2210023G0

chr7	71039402	71039475	intron (NM	RSINE1|SIN	-22305	NM_00125	381994	E030018B1	Gm1697
chr18	55074402	55074600	intron (NM	MTD|LTR|	75333	NM_17575	269023	Zfp608	4932417D1
chr3	40985102	40985225	Intergenic	Intergenic	-98195	NM_02755	70804	Pgrmc2	4631434O1
chr1	98762577	98763000	intron (NM	intron (NM	5960	NM_17265	227394	Slco4c1	9530051F0
chr5	1.22E+08	1.22E+08	intron (NM	intron (NM	920	NM_00965	11669	Aldh2	Ahd-5|Ahd
chr12	88173027	88173150	Intergenic	Intergenic	52676	NM_14583	238330	Irf2bpl	6430527G1
chr11	87284377	87284550	intron (NM	CpG	43370	NM_00119	83560	Tex14	C85585
chr10	39044127	39044425	Intergenic	Intergenic	-45329	NM_00112	14360	Fyn	AI448320|/
chr13	63052927	63053150	Intergenic	Intergenic	-13215	NM_00128	72061	2010111I0	2300006M
chr8	3660952	3661075	Intergenic	RMER1B|C	-4749	NM_02698	69189	Mcemp1	1810033B1
chr14	51417502	51417600	Intergenic	PB1D9|SIN	-2148	NM_00111	239083	Ccnb1ip1	Gm288|He
chr15	74539727	74539825	promoter-T	promoter-T	-24	NM_00841	16469	Jrk	-
chr7	1.34E+08	1.34E+08	promoter-T	promoter-T	77	NM_14620	233890	Zfp768	BC026432|
chr10	85484802	85485050	intron (NM	CpG	252	NM_15319	69754	Fbxo7	2410015K2
chr1	84403452	84403800	intron (NM	intron (NM	-122406	NM_00100	98496	Pid1	5033414K0
chr13	6633927	6634050	intron (NM	intron (NM	14029	NM_01970	56421	Pfkp	1200015H2
chr8	87211302	87211925	5' UTR (NM	5' UTR (NM	148	NM_02578	66830	Nacc1	2010001H0
chr1	30775302	30775775	Intergenic	Intergenic	144563	NM_00108	213109	Phf3	2310061N1
chr6	1.29E+08	1.29E+08	Intergenic	Intergenic	-17480	NM_03059	80782	Klrb1b	Klrb1d|Ly5
chr2	27330927	27331125	promoter-T	promoter-T	-108	NM_02333	67382	Brd3	2410084F2
chr11	59919252	59919450	intron (NM	CpG	836	NM_00902	19377	Rai1	Gt1
chr5	36068777	36068900	intron (NM	intron (NM	3087	NM_19434	231147	Sh3tc1	4631428G1
chr5	32655427	32655500	promoter-T	promoter-T	-490	NM_17214	665270	Plb1	4632413E2
chr2	1.27E+08	1.27E+08	5' UTR (NM	5' UTR (NM	136	NM_13930	99138	Stard7	AI852671|/
chr11	1.02E+08	1.02E+08	Intergenic	Intergenic	-1516	NM_01155	21429	Ubtf	A930005G0
chr16	65726077	65726325	Intergenic	Lx8|LINE|L	-89677	NM_02857	73569	Vgll3	1700110N1
chr10	62486102	62486250	intron (NM	CpG	421	NM_00107	432467	Hnrnph3	AA693301|
chr7	1.5E+08	1.5E+08	intron (NM	intron (NM	-4735	NM_02028	56844	Tssc4	AA241958|
chr9	1.06E+08	1.06E+08	promoter-T	promoter-T	123	NM_14562	27966	Rrp9	55kDa|D19
chr6	17231202	17231350	promoter-T	promoter-T	91	NM_01690	12390	Cav2	AI447843
chr17	32924702	32924775	intron (NM	intron (NM	494	NM_17245	208292	Zfp871	4732483N1
chr5	1.09E+08	1.09E+08	promoter-T	promoter-T	-340	NM_02610	13486	Dr1	1700121L0
chr2	79972977	79973100	Intergenic	Intergenic	-3469	NM_00115	18573	Pde1a	AI987702|/
chr2	35095602	35095725	Intergenic	Intergenic	-16216	NM_00120	227753	Gsn	ADF
chr7	1.49E+08	1.49E+08	promoter-T	promoter-T	-2	NM_00128	54006	Deaf1	AU042387
chr3	90486252	90486375	Intergenic	Intergenic	13320	NM_01365	20201	S100a8	60B8Ag|AI
chr10	44249302	44249500	Intergenic	CpG	-70908	NM_00754	12142	Prdm1	Blimp-1|Bl
chr14	65881002	65881375	5' UTR (NM	5' UTR (NM	112	NM_02145	14365	Fzd3	AU020229
chr9	1.11E+08	1.11E+08	intron (NM	intron (NM	773	NM_01746	54199	Ccrl2	1810047I0
chr5	1.36E+08	1.36E+08	exon (NM_	exon (NM_	135	NM_02537	66138	Wbscr22	1110003N2
chr2	25356627	25356700	promoter-T	promoter-T	365	NM_01390	30839	Fbxw5	AI159739|
chr17	48007452	48007700	intron (NM	intron (NM	-35936	NM_00110	17240	Mdfi	I-mf|I-mfa
chr10	70812102	70812275	intron (NM	intron (NM	1647	NM_02718	69718	Ipmk	2410017C1
chr7	73692527	73692625	Intergenic	B1_Mur3|S	-120213	NM_05308	56847	Aldh1a3	ALDH6|RA
chr19	9004252	9004400	promoter-T	promoter-T	142	NM_14487	225898	Eml3	BC022146
chr9	15161702	15161825	intron (NM	ID_B1|SINE	469	NM_17697	319675	5830418K0	5832426L2
chr2	31945302	31945375	Intergenic	RMER15|LT	-5833	NM_14552	227721	Ppapdc3	D830019K1

chr3	94386827	94387300	promoter-T	promoter-T	-425	NM_02972	76742	Snx27	5730552M
chr9	72978477	72978550	Intergenic	Intergenic	17237	NM_19860	225215	Rsl24d1	2410159K2
chr1	84937577	84937850	Intergenic	Intergenic	-6055	NM_02792	71781	Slc16a14	1110004H1
chr8	86265127	86265300	promoter-T	promoter-T	-3	NM_00116	26364	Cd97	AA409984|
chr4	1.19E+08	1.19E+08	promoter-T	promoter-T	-570	NM_01173	22608	Ybx1	1700102N1
chr14	70253802	70254100	intron (NM	intron (NM	13737	NM_02852	73523	Pebp4	PEBP-4
chr3	51145402	51145525	promoter-T	promoter-T	-897	NM_02350	69257	Elf2	2610036A2
chr1	36315077	36315175	Intergenic	RMER4B|LT	-13979	NM_19889	320011	Uggt1	0910001L1
chr18	38643627	38643850	Intergenic	MLT1B|LTF	65109	NM_02299	65113	Ndfip1	0610010M
chr15	99200877	99201000	promoter-T	promoter-T	-41	NM_01171	22379	Fmnl3	2700073B0
chr12	57446852	57447000	promoter-T	promoter-T	-45	NM_14544	217588	Mbip	4933408E0
chr12	85534827	85534900	5' UTR (NM	5' UTR (NM	102	NM_00116	238317	Elmsan1	9430029N1
chr9	1.22E+08	1.22E+08	Intergenic	Intergenic	-1608	NM_00116	20623	Snrk	2010012F0
chr2	1.55E+08	1.55E+08	promoter-T	promoter-T	-334	NM_02603	67204	Eif2s2	2810026E1
chr6	82716652	82716775	intron (NM	intron (NM	7735	NM_01382	15277	Hk2	AI642394|
chr4	55343052	55343150	Intergenic	Intergenic	-19813	NM_00901	19359	Rad23b	0610007D1
chr1	1.37E+08	1.37E+08	promoter-T	promoter-T	-241	NM_02602	67196	Ube2t	2700084L2
chr3	20054652	20054800	intron (NM	CpG	289	NM_01375	27357	Gyg	AU017667
chr4	65850202	65850450	intron (NM	intron (NM	215191	NM_01951	56079	Astn2	1d8|Astnl|
chr3	1.08E+08	1.08E+08	intron (NM	MIRb|SINE	4042	NM_00814	14686	Gnat2	AW490837
chr11	1.2E+08	1.2E+08	TTS (NM_0	TTS (NM_0	118	NM_02720	56282	Mrpl12	0610034O1
chr8	26221577	26221775	Intergenic	Intergenic	-9393	NM_03125	83436	Plekha2	6430512N2
chr4	1.41E+08	1.41E+08	promoter-T	promoter-T	-476	NM_01976	56381	Spen	Mint|mKIA
chr9	21807627	21807725	promoter-T	promoter-T	244	NM_00892	19089	Prkcsh	80K-H|PKC
chr15	66871152	66871325	Intergenic	Intergenic	-70035	NM_00868	17988	Ndrg1	CAP43|CM
chr14	47973777	47973925	Intergenic	Intergenic	-19684	NM_01070	16854	Lgals3	GBP|L-34|
chr15	76059752	76059825	exon (NM_	exon (NM_	139	NM_20138	18810	Plec	AA591047|
chr1	1.84E+08	1.84E+08	Intergenic	L1_Mus2|L	-21024	NM_00979	12334	Capn2	AI326419|
chr8	68918152	68918325	intron (NM	intron (NM	8038	NM_00116	72925	1-Mar	2900024D2
chrX	1.37E+08	1.37E+08	Intergenic	Intergenic	-13665	NM_00107	14605	Tsc22d3	DIP|Dsip1|
chr18	67879602	67879975	intron (NM	intron (NM	4487	NM_00112	19255	Ptpn2	AI325124|
chr10	1.07E+08	1.07E+08	Intergenic	Intergenic	-36011	NM_00117	628870	Otogl	EG628870|
chr7	1.28E+08	1.28E+08	promoter-T	promoter-T	-133	NM_00116	233812	BC030336	-
chr7	59117377	59117550	exon (NM_	exon (NM_	174	NM_00111	1E+08	Fancf	A730016A1
chr17	46817152	46817300	promoter-T	promoter-T	-176	NM_00127	17256	Mea1	Mea-1
chr11	97687477	97687600	intron (NM	intron (NM	-14556	NM_00108	68127	B230217C1	AI840637
chr4	1.29E+08	1.29E+08	Intergenic	Intergenic	-11755	NM_01689	11637	Ak2	Ak-2|D4Er
chr1	1.34E+08	1.34E+08	intron (NM	intron (NM	11102	NM_17724	320718	Slc26a9	E030002L0
chr12	36739102	36739225	Intergenic	MYSERV16	19651	NM_01178	23795	Agr2	Agr2h|Gob
chr1	1.68E+08	1.68E+08	intron (NM	intron (NM	7474	NM_17859	226594	Rcsd1	A430105K1
chr1	82836552	82836775	intron (NM	CpG	605	NM_01047	15463	Agfg1	AU045498
chr11	22882027	22882200	exon (NM_	exon (NM_	171	NM_14451	17846	Commd1	AI256843|
chr9	53474877	53474950	intron (NM	CpG	699	NM_00116	75717	Cul5	4921514I2
chr8	74012252	74012350	5' UTR (NM	5' UTR (NM	299	NM_03254	70359	Gtpbp3	2410009F1
chr8	36342002	36342375	Intergenic	Intergenic	-96607	NM_17774	244416	Ppp1r3b	6430576E2
chr11	1.01E+08	1.01E+08	exon (NM_	exon (NM_	487	NM_01203	26949	Vat1	VAT-1
chr1	1.68E+08	1.68E+08	intron (NM	intron (NM	16437	NM_17859	226594	Rcsd1	A430105K1

chr1	1.46E+08	1.46E+08	Intergenic	Intergenic	-81429	NM_01581	50778	Rgs1	BL34
chr9	15513702	15513925	promoter-T	promoter-T	-400	NM_17228	234967	Slc36a4	6330573I1!
chr1	1.36E+08	1.36E+08	intron (NM	intron (NM	531	NM_00757	12227	Btg2	AA959598|
chr10	53794952	53795200	promoter-T	promoter-T	526	NM_00854	17155	Man1a	Man1a1|P(
chr16	17722802	17722950	exon (NM_	exon (NM_	117	NM_00128	94112	Med15	A230074L1
chr1	1.53E+08	1.53E+08	Intergenic	Intergenic	-73865	NM_00103	117198	Ivns1abp	1190004M
chr12	80397152	80397275	promoter-T	promoter-T	56	NM_00100	211978	Zfyve26	4930465A1
chr5	66251927	66252300	Intergenic	Intergenic	-2695	NM_00108	74734	Rhoh	5830400A(
chr19	3423177	3423450	Intergenic	Intergenic	-8856	NM_01025	14419	Gal	Galn
chr13	97760702	97760850	Intergenic	Intergenic	43541	NM_02558	66479	1700029F1	-
chr5	99712802	99712925	Intergenic	Intergenic	-30917	NM_14583	320292	Rasgef1b	4732452O(
chr15	59505552	59505800	Intergenic	Intergenic	25467	NM_14454	211770	Trib1	A530090O1
chr6	1.19E+08	1.19E+08	intron (NM	intron (NM	8466	NM_00127	22419	Wnt5b	AW545702
chr3	1.17E+08	1.17E+08	Intergenic	Intergenic	-14584	NM_00111	20321	Frrs1	AI131732|!
chr4	3782252	3782400	Intergenic	L1MB8|LIN	16926	NM_02002	17451	Mos	c-mos
chr1	1.8E+08	1.8E+08	5' UTR (NM	5' UTR (NM	232	NM_00116	269152	Kif26b	4832420M
chr5	31357027	31357275	promoter-T	promoter-T	-2	NM_00128	69719	Cad	2410008J0
chr11	75993227	75993375	promoter-T	promoter-T	-169	NM_02666	68299	Vps53	2010002A(
chr4	1.55E+08	1.55E+08	promoter-T	promoter-T	-244	NM_00116	14688	Gnb1	AA409223|
chr6	63383627	63383775	intron (NM	intron (NM	176850	NM_00816	14804	Grid2	B230104L0
chr7	36103177	36103300	intron (NM	CpG	221	NM_02618	67471	Gpatch1	1300003A1
chr12	35633802	35633900	Intergenic	Intergenic	-32084	NM_00127	79221	Hdac9	AV022454|
chr16	16035752	16035850	intron (NM	Lx9|LINE|L	111143	NM_14606	224008	Spidr	2310008H(
chr13	52684277	52684675	intron (NM	(ATG)n|Sin	5670	NM_00119	20963	Syk	Sykb
chr1	40115252	40115400	Intergenic	Intergenic	-26287	NM_01055	16178	Il1r2	CD121b|Il1
chr15	81702002	81702175	promoter-T	promoter-T	234	NM_02673	68479	Phf5a	1110007B(
chr15	65934877	65935225	intron (NM	intron (NM	-126685	NM_00114	654498	Hhla1	F930104E1
chr2	1.44E+08	1.44E+08	intron (NM	LTR16E1|L	16071	NM_00975	12075	Bfsp1	CP95
chr6	83299352	83299500	promoter-T	promoter-T	-52	NM_17527	78653	Bola3	1810056O2
chr1	72302452	72302525	Intergenic	Intergenic	28400	NM_02352	111175	Pecr	2400003B1
chr3	1.16E+08	1.16E+08	TTS (NM_0	TTS (NM_0	-27803	NM_14490	229782	Slc35a3	2310050P1
chr10	57814352	57814525	intron (NM	intron (NM	-19764	NM_20124	110829	Lims1	2310016J2
chr1	99615177	99615325	intron (NM	intron (NM	51418	NM_17376	227399	Ppip5k2	AW555814
chr17	31875252	31875375	Intergenic	Intergenic	60437	NM_00127	12954	Cryaa	Acry-1|Cry
chr14	56196652	56197225	promoter-T	promoter-T	-393	NM_01118	19186	Psme1	AW413925
chr4	86257602	86257700	exon (NM_	exon (NM_	275	NM_17340	230376	Haus6	6230416J2
chr11	1.07E+08	1.07E+08	intron (NM	intron (NM	7385	NM_17685	207165	Bptf	9430093H1
chr1	36125127	36125250	promoter-T	promoter-T	-57	NM_01581	50785	Hs6st1	6Ost1
chr13	35656002	35656300	Intergenic	Intergenic	-95580	NM_00988	12593	Cdyl	AI325931
chr17	31418802	31418925	Intergenic	Intergenic	-4562	NM_02529	22092	Rsph1	MCA|Tsga2
chr1	1.66E+08	1.66E+08	promoter-T	promoter-T	92	NM_02877	240880	Scyl3	1200016D2
chr3	94758202	94758425	promoter-T	promoter-T	-624	NM_01739	53970	Rfx5	-
chr6	72325052	72325300	intron (NM	B4A|SINE|	5286	NM_01687	53620	Vamp5	AF119384|
chr1	1.41E+08	1.41E+08	intron (NM	intron (NM	440	NM_00116	329260	Dennd1b	4632404N1
chr15	91009077	91009475	intron (NM	intron (NM	12962	NM_01199	26874	Abcd2	ABC39|ALD
chr4	11029852	11030125	Intergenic	Intergenic	-26636	NM_00108	76947	Ndufaf6	2310030N(
chr14	20901702	20902350	intron (NM	intron (NM	368	NM_02620	67509	Saysd1	1810063B(

chr14	35315177	35315325	intron (NM	CpG	481	NM_00975	12166	Bmpr1a	1110037I2
chr12	53198677	53198775	promoter-T	promoter-T	-7	NM_02976	76826	Nubpl	2410170E0
chr15	90497302	90497750	intron (NM	intron (NM	12293	NM_02581	66871	Cpne8	1200003E1
chr15	35360077	35360350	intron (NM	L1M5|LINE	58912	NM_17715	666173	Vps13b	1810042B0
chr8	64410002	64410125	intron (NM	Lx7|LINE|L	3179	NM_00129	234311	Ddx60	9830118M
chr8	67351427	67351575	intron (NM	intron (NM	22321	NM_17863	77113	Klhl2	6030411N2
chr4	88368052	88368275	5' UTR (NM	5' UTR (NM	249	NM_17287	242521	Klhl9	8030469P0
chr16	85132377	85132525	intron (NM	intron (NM	41501	NM_00119	11820	App	Abeta|Abp
chr11	53113427	53113550	intron (NM	CpG	493	NM_00830	15525	Hspa4	70kDa|AI3
chr2	1.12E+08	1.12E+08	promoter-T	promoter-T	-83	NM_02884	74268	Aven	1700013A0
chr5	1.47E+08	1.47E+08	intron (NM	MMAR1|D	17187	NM_15359	264064	Cdk8	-
chr15	78809227	78809550	intron (NM	intron (NM	-4098	NM_00102	110253	Triobp	AI428493|
chr5	1.26E+08	1.26E+08	promoter-T	promoter-T	-567	NM_01142	20602	Ncor2	N-CoR|SM
chr2	33727452	33727625	intron (NM	intron (NM	15928	NM_17518	72543	Mvb12b	2610200O1
chr3	97413877	97414075	intron (NM	CpG	137	NM_02653	68058	Chd1l	4432404A2
chr15	22991327	22991525	intron (NM	Lx2B|LINE|	25208	NM_00108	320865	Cdh18	B230220E1
chr10	60664327	60664750	intron (NM	intron (NM	26528	NM_02306	65971	Tbata	1700021K0
chr6	99616427	99616575	intron (NM	CpG	264	NM_02582	66892	Eif4e3	1300018P1
chr10	1.27E+08	1.27E+08	intron (NM	CpG	736	NM_00111	17938	Naca	AL022831|
chr11	1.01E+08	1.01E+08	exon (NM_	exon (NM_	280	NM_00103	14534	Kat2a	1110051E1
chr2	4984652	4984800	intron (NM	intron (NM	258	NM_18184	71648	Optn	4930441O0
chr9	1.23E+08	1.23E+08	intron (NM	CpG	186	NM_00101	382117	Tcaim	D9Ertd402
chr10	79784077	79784225	promoter-T	promoter-T	-858	NM_02552	66374	2310011J0	AI452186|
chr8	28133577	28133650	promoter-T	promoter-T	-658	NM_15359	244373	Erlin2	BC036333|
chr10	51953602	51953825	intron (NM	CpG	288	NM_02570	66686	Dcbld1	4631413K1
chr5	1.51E+08	1.51E+08	intron (NM	intron (NM	1399	NM_13389	100637	N4bp2l1	2410024N1
chr17	26389952	26390075	exon (NM_	exon (NM_	158	NR_03790	66978	Luc7l	1810045C0
chr8	96910052	96910325	promoter-T	promoter-T	-212	NM_02233	64209	Herpud1	Herp|Mifl|
chr6	81915652	81915825	exon (NM_	exon (NM_	205	NM_02649	56284	Mrpl19	9030416F1
chr5	1.4E+08	1.4E+08	promoter-T	promoter-T	-16	NM_02560	66506	Psmg3	1810042K0
chr11	35641402	35641475	intron (NM	intron (NM	6592	NM_02593	104458	Rars	2610011N1
chr12	1.19E+08	1.19E+08	intron (NM	(TTC)n|Sim	-96333	NM_14604	217946	Cdca7l	BC006933|
chr10	66550277	66550375	intron (NM	intron (NM	9410	NM_00120	28193	Reep3	D10Ucla1
chr2	1.48E+08	1.48E+08	Intergenic	B3|SINE|B	14983	NM_01074	17064	Cd93	6030404G0
chr15	82840127	82840275	intron (NM	intron (NM	13367	NM_02872	74039	Nfam1	4921501M
chr7	1.16E+08	1.16E+08	promoter-T	promoter-T	-91	NM_02534	66085	Eif3f	0610037M
chr11	1.04E+08	1.04E+08	exon (NM_	exon (NM_	17367	NM_00108	76719	Kansl1	1700081L1
chr5	3463327	3463675	intron (NM	intron (NM	-80332	NM_00104	68152	Fam133b	2900022K0
chr3	86028377	86028575	promoter-T	promoter-T	-136	NM_00107	80877	Lrba	C80285|D3
chr10	20603652	20603750	Intergenic	Intergenic	-68652	NM_02620	52906	Ahi1	1700015F0
chr11	67380477	67380600	intron (NM	MTD|LTR|	-19464	NM_00110	14457	Gas7	AW124766
chr1	1.94E+08	1.94E+08	promoter-T	promoter-T	-659	NM_00957	22782	Slc30a1	AI839647|
chr1	88339752	88340050	Intergenic	Intergenic	16997	NM_02796	71863	1700019O	-
chr17	35873452	35873750	Intergenic	B1_Mur4|	-32517	NM_17296	12305	Ddr1	6030432F1
chr14	64803377	64803600	intron (NM	intron (NM	80328	NM_02852	73382	Prss52	1700049K1
chr4	1.09E+08	1.09E+08	promoter-T	promoter-T	-702	NM_00130	12580	Cdkn2c	C77269|IN
chr16	20724402	20724525	TTS (NM_0	TTS (NM_0	6208	NM_00128	21832	Thpo	Mgdf|Ml|

chr9	44074627	44074725	intron (NM	intron (NM	1594	NM_00116	270151	Nlrx1	BC034204|
chr8	1.14E+08	1.14E+08	exon (NM_	exon (NM_	237	NM_02827	72544	Exosc6	2610510N2
chr7	1.35E+08	1.35E+08	promoter-T	promoter-T	-277	NM_17802	233904	Setd1a	BC010250|
chr18	37803577	37803750	5' UTR (NM	5' UTR (NM	195	NM_17577	24074	Taf7	55kDa|AI6
chr10	21790052	21790275	Intergenic	Intergenic	-2278	NM_05326	114671	4930444G2	-
chr3	1.33E+08	1.33E+08	Intergenic	Intergenic	107716	NM_00104	214133	Tet2	Ayu17-449
chr15	79604702	79604800	intron (NM	CpG	146	NM_01747	54152	Dnal4	D15Ertd424
chr8	98424427	98424550	Intergenic	Lx6|LINE|L	-12223	NM_01032	14719	Got2	AL022787|
chr5	1.39E+08	1.39E+08	5' UTR (NM	5' UTR (NM	228	NM_03056	80752	Fam20c	C76981|DM
chr13	16023377	16023550	Intergenic	Intergenic	-82845	NM_00838	16323	Inhba	-
chr19	7562277	7562450	Intergenic	Intergenic	-4582	NM_00100	20168	Rtn3	RTN3-A1
chr16	64770702	64771075	promoter-T	promoter-T	-123	NM_02627	67609	4930453N2	AI649104
chr6	1.2E+08	1.2E+08	intron (NM	intron (NM	49515	NM_01671	29862	Ninj2	-
chr4	1.52E+08	1.52E+08	intron (NR_	intron (NR_	1478	NR_102291	19934	Rpl22	2700038K1
chr10	92773127	92773200	intron (NM	CpG	741	NM_01350	13713	Elk3	D430049E2
chr17	51061702	51061950	intron (NM	intron (NM	223820	NM_00129	12228	Btg3	ANA|tob5
chr13	1.05E+08	1.05E+08	intron (NM	intron (NM	71118	NM_02944	75805	Nln	4930472G1
chr4	40704727	40704825	intron (NM	CpG	142	NM_02153	74255	Smu1	2600001O(
chr15	79346952	79347025	exon (NM_	exon (NM_	150	NM_13409	105785	Kdelr3	AI173274
chr5	1.48E+08	1.48E+08	intron (NM	CpG-11811	404	NM_00908	20018	Polr1d	1110003G1
chr16	87710302	87710425	intron (NM	L1M5|LINE	11164	NM_00752	12013	Bach1	6230421P0
chr6	99125477	99125575	intron (NM	intron (NM	90983	NM_00119	108655	Foxp1	3110052D1
chr4	1.36E+08	1.36E+08	Intergenic	(ATG)n|Sin	-36826	NM_00128	15552	Htr1d	5-HT-1D|5-
chr1	1.33E+08	1.33E+08	intron (NM	MTE2a|LTR	34442	NM_01070	16865	Eif2d	D1Ertd5e|
chr4	1.17E+08	1.17E+08	promoter-T	promoter-T	-12	NM_17513	108067	Eif2b3	1190002P1
chr6	1.37E+08	1.37E+08	Intergenic	Intergenic	10232	NM_02389	78600	Pde6h	A930033D1
chr1	1.4E+08	1.4E+08	Intergenic	IAPEY3-int|	-45289	NM_17739	338375	Atp6v1g3	-
chr12	66176577	66176650	promoter-T	promoter-T	20	NM_17891	104806	Fancm	AI427100|
chr10	62892552	62892700	promoter-T	promoter-T	-220	NM_17761	216033	Ctnna3	4930429L0
chr7	52111827	52112100	promoter-T	promoter-T	-553	NM_02154	59047	Pnkp	1810009G0
chr8	8751302	8751450	Intergenic	Intergenic	-60839	NM_17684	234023	Arglu1	9430010O(
chr15	74916902	74917550	Intergenic	Intergenic	25153	NM_00109	1E+08	Ly6c2	Ly-6C.2|Ly-
chr8	59911652	59911900	Intergenic	Intergenic	-22944	NM_00913	20284	Scrg1	AW124307
chr8	48760752	48760925	promoter-T	promoter-T	-325	NM_02350	69260	Ing2	2810011M
chr6	1.41E+08	1.41E+08	intron (NM	intron (NM	5382	NM_00963	11569	Aebp2	AU023766
chr11	69223752	69223875	intron (NM	CpG	3364	NM_00101	216850	Kdm6b	1700064E0
chr6	71444477	71444675	5' UTR (NM	5' UTR (NM	688	NM_00954	22644	Rnf103	AW146237
chr2	1.81E+08	1.81E+08	promoter-T	promoter-T	-548	NM_13370	68879	Prpf6	1190003A0
chr16	17928277	17928450	promoter-T	promoter-T	-51	NM_15315	13358	Slc25a1	1300019P0
chr5	52216977	52217100	Intergenic	Intergenic	-271878	NM_00890	19017	Ppargc1a	A830037N(
chr1	92764377	92764475	Intergenic	Intergenic	-23878	NM_00124	12835	Col6a3	AI507288|
chr9	70686952	70687050	intron (NM	intron (NM	95614	NM_00828	15450	Lipc	AI256194|
chr2	6133602	6133925	intron (NM	intron (NM	277	NM_02420	67856	Echdc3	2310005D1
chr4	58343752	58344150	intron (NM	intron (NM	45117	NM_01094	18198	Musk	Mdk4|Mlk
chr3	89050802	89050875	exon (NM_	exon (NM_	478	NM_02532	66059	Krtcap2	0610010I1
chr10	81590752	81590850	promoter-T	promoter-T	43	NM_00116	216177	AU041133	-
chr6	1.27E+08	1.27E+08	Intergenic	Intergenic	-48355	NM_00103	381812	Cracr2a	Efcab4b|G

chr14	69930602	69930675	Intergenic	Intergenic	-18282	NM_17342	239167	Synb	D930020E(
chr2	1.14E+08	1.14E+08	intron (NM	CpG	176	NM_02567	66632	Dph6	5730421E1
chr2	1.52E+08	1.52E+08	promoter-T	promoter-T	-72	NM_21244	228769	Psmf1	AW048666
chr9	78290627	78291100	exon (NM_	exon (NM_	181	NR_12572	214763	Mb21d1	E330016A1
chr4	34634402	34634625	5' UTR (NM	5' UTR (NM	174	NM_01189	24060	Slc35a1	AA408150|
chr4	56770077	56770450	intron (NM	intron (NM	13969	NM_00961	11470	Actl7a	Tact2
chr5	1.25E+08	1.25E+08	intron (NM	CpG	546	NM_14450	65105	Arl6ip4	AA408210|
chr1	39592577	39592850	promoter-T	promoter-T	66	NM_02804	52846	Cnot11	2410015L1
chr12	1.12E+08	1.12E+08	exon (NM_	exon (NM_	204	NM_19802	217864	Rcor1	5730409O1
chr11	1.15E+08	1.15E+08	Intergenic	Intergenic	7495	NM_02786	71679	Atp5h	0610009D1
chr2	91297827	91298100	intron (NM	CpG	275	NM_00114	228357	Lrp4	6430526J1
chr14	33437352	33437800	intron (NM	intron (NM	24463	NM_00100	107751	Prrxl1	Drg11|Drg
chr14	55331577	55331725	promoter-T	promoter-T	-640	NM_20713	110794	Cebpe	C/EBPe|CR
chr7	19589252	19589475	promoter-T	promoter-T	-51	NM_17875	272359	Irf2bp1	6330414O(
chr18	73732527	73732825	5' UTR (NM	5' UTR (NM	317	NM_00103	240396	Mex3c	A130001D1
chr1	1.35E+08	1.35E+08	Intergenic	tRNA-Lys-A	-8676	NM_00857	17248	Mdm4	4933417N(
chr12	1.13E+08	1.13E+08	promoter-T	promoter-T	-90	NM_00109	328162	Trmt61a	6720458F0
chr10	26525977	26526100	intron (NM	intron (NM	33720	NM_17683	73910	Arhgap18	4833419J0
chr1	1.09E+08	1.09E+08	Intergenic	Intergenic	-30345	NM_01145	20725	Serpinb8	CAP-2|CAP
chr13	52941077	52941225	intron (NM	intron (NM	83895	NM_01670	11992	Auh	C77140|W
chr18	3004627	3004800	Intergenic	(TTTC)n|Si	118699	NM_00116	1E+08	Vmn1r238	-
chr1	1.66E+08	1.66E+08	Intergenic	MIR|SINE|	21256	NM_00116	20343	Sell	AI528707|
chr5	1.44E+08	1.44E+08	intron (NM	intron (NM	1240	NM_14491	231871	Daglb	E330036I1
chr3	97887177	97887300	intron (NM	intron (NM	69777	NM_01092	18129	Notch2	AI853703|
chr8	1.27E+08	1.27E+08	Intergenic	L1M5|LINE	-30818	NM_13927	108148	Galnt2	AI480629
chr1	89516552	89516675	promoter-T	promoter-T	-274	NM_00111	16331	Inpp5d	SHIP|SHIP-
chr16	48818777	48819050	TTS (NM_0	TTS (NM_0	1944	NM_02388	57263	Retnlb	9030012B2
chr15	57525477	57525700	promoter-T	promoter-T	-634	NM_19944	387609	Zhx2	Afr-1|Afr1
chr3	60241652	60241900	Intergenic	Intergenic	-34976	NM_00125	56758	Mbnl1	Mbnl|mKIA
chr17	24687102	24687200	5' UTR (NM	5' UTR (NM	256	NM_01363	18763	Pkd1	PC1|mFLJ0
chr9	65187102	65187175	Intergenic	Intergenic	-6737	NM_01668	50996	Pdcd7	C80112|ES
chr2	4802652	4803325	intron (NM	CpG	378	NM_17540	109079	Sephs1	1110046B2
chr8	42129152	42129450	intron (NM	intron (NM	-2016	NM_00100	102103	Mtus1	AI481402|
chr11	44333127	44333275	intron (NM	intron (NM	322	NM_00116	74315	Rnf145	3732413I1
chr11	11487427	11487725	Intergenic	Intergenic	98307	NM_02866	73862	4930415F1	-
chr9	43855502	43855600	3' UTR (NM	3' UTR (NM	4084	NM_00938	21838	Thy1	CD90|T25|
chr6	24596852	24597100	intron (NM	intron (NM	18019	NM_00116	73178	Wasl	2900021I1
chr13	43494052	43494125	intron (NM	CpG	343	NM_02355	70078	Nol7	2210008F1
chr5	1.44E+08	1.44E+08	promoter-T	promoter-T	-327	NM_02974	76800	Usp42	2410140K0
chr2	1.78E+08	1.78E+08	Intergenic	Intergenic	-1588	NM_17719	320558	Sycp2	3830402K2
chr6	1.25E+08	1.25E+08	Intergenic	Intergenic	-2671	NM_01353	14794	Spsb2	AI461677|
chr6	1.46E+08	1.46E+08	intron (NM	intron (NM	103132	NM_01058	16439	Itpr2	AI649341|
chr16	34753227	34753350	Intergenic	Intergenic	-31748	NM_13930	107589	Mylk	9530072E1
chr17	79754377	79754575	promoter-T	promoter-T	-45	NM_02651	260409	Cdc42ep3	3200001F0
chr6	34664527	34664875	intron (NM	intron (NM	5257	NM_14557	109624	Cald1	4833423D1
chr7	1.07E+08	1.07E+08	Intergenic	Intergenic	-19579	NM_02762	70974	Pgm2l1	4931406N1
chr8	47511727	47511925	Intergenic	Intergenic	-44570	NM_00798	14081	Acsl1	Acas|Acas1

chr1	87527152	87527275	intron (NM	intron (NM	-19412	NM_01367	20684	Sp100	A430075G1
chr12	16059852	16060075	Intergenic	Intergenic	-236372	NM_14455	217410	Trib2	AW319517
chr5	21291077	21291225	promoter-T	promoter-T	50	NM_01118	19181	Psmc2	-
chr5	1.3E+08	1.3E+08	promoter-T	promoter-T	-593	NM_00939	19384	Ran	-
chr3	63768327	63768925	promoter-T	promoter-T	29	NM_00127	11416	Slc33a1	AI315656|/
chr1	94369852	94370525	intron (NM	CpG	147	NM_02419	67273	Ndufa10	2900053E1
chr6	1.29E+08	1.29E+08	intron (NM	RMER12B|	-25043	NM_03059	80782	Klrb1b	Klrb1d|Ly5
chr6	51831077	51831150	intron (NM	intron (NM	131435	NM_01877	54353	Skap2	2610021A1
chrX	9028777	9028950	intron (NM	intron (NM	17587	NM_00780	13058	Cybb	C88302|CG
chr17	56395002	56395150	Intergenic	AT_rich|Lo	-1140	NM_01019	14154	Fem1a	AW611390
chr2	1.02E+08	1.02E+08	intron (NM	intron (NM	16263	NM_17518	72446	Prr5l	2600010E0
chr11	32242477	32242975	Intergenic	(TG)n|Simp	-5085	NM_17736	268396	Sh3pxd2b	G431001E0
chr15	75899827	75899975	TTS (NM_0	TTS (NM_0	259	NM_13408	105782	Scrib	AI118201|(
chr9	55004202	55004275	intron (NM	intron (NM	7062	NM_18060	109161	Ube2q2	3010021M
chr1	36931777	36932100	intron (NM	ORR1E|LTF	64434	NM_01887	56030	Tmem131	2610524E0
chr2	1.7E+08	1.7E+08	Intergenic	Intergenic	-12651	NM_00115	228913	Zfp217	4933431C0
chr13	99584502	99584775	intron (NM	CpG	766	NM_17259	218503	Fcho2	5832424M
chr19	37657127	37657350	intron (NM	L1M4c|LIN	32330	NM_17535	107371	Exoc6	4833405E0
chr10	17667452	17667725	exon (NM_	exon (NM_	285	NM_00103	380629	Heca	Gm869|HD
chr9	64585227	64585300	intron (NM	CpG	300	NM_01738	53869	Rab11a	-
chr8	98157277	98157675	promoter-T	promoter-T	17	NM_03019	78833	Gins3	2700085M
chr11	1.03E+08	1.03E+08	intron (NM	L1MB7|LIN	12490	NM_18303	353047	Plekhm1	AP162|B2|
chr2	34681702	34681800	promoter-T	promoter-T	4	NM_00116	30050	Fbxw2	2700071L0
chr11	22186702	22186800	promoter-T	promoter-T	47	NM_15307	216565	Ehbp1	AF424697|
chr14	32890427	32890825	Intergenic	Intergenic	7846	NR_10430!	105638	Dph3	DELGIP1|D
chr1	1.83E+08	1.83E+08	Intergenic	Intergenic	-41419	NM_20728	403180	Ccdc121	6530421E2
chr1	88420652	88420875	Intergenic	Intergenic	-2548	NM_00897	19231	Ptma	Thym
chr5	1.45E+08	1.45E+08	intron (NM	intron (NM	652	NM_01355	15467	Eif2ak1	HCR|Hri
chr2	29922327	29922550	intron (NM	CpG	444	NM_00120	56086	Set	2610030F1
chr12	86984602	86984800	Intergenic	Intergenic	-42969	NM_01676	53314	Batf	B-ATF|SFA-
chr2	1.03E+08	1.03E+08	intron (NM	(CAAAA)n|	38009	NM_00117	12505	Cd44	AU023126
chr19	37508352	37508550	promoter-T	promoter-T	-880	NM_00824	15242	Hhex	Hex|Hex1|
chr8	1.08E+08	1.08E+08	promoter-T	promoter-T	590	NM_01582	50788	Fbxl8	FBL8
chr1	1.9E+08	1.9E+08	intron (NM	intron (NM	-164668	NM_01193	26381	Esrrg	ERR3|Errg|
chr15	49185977	49186275	Intergenic	Intergenic	-562591	NM_00108	239420	Csmd3	4930500N1
chr12	25183077	25183650	promoter-T	promoter-T	-83	NM_02061	21340	Taf1b	4930408G0
chr1	34536027	34536125	Intergenic	Intergenic	-19199	NM_00935	21755	Prss39	Tesp1
chr16	8471002	8471100	intron (NM	MIRc|SINE	145	NM_14624	239706	Mettl22	-
chr7	1.06E+08	1.06E+08	Intergenic	Intergenic	-9705	NM_17744	233549	Mogat2	DGAT2L5|I
chr9	1E+08	1E+08	Intergenic	Intergenic	66879	NM_00103	213208	Il20rb	AV228068|
chr11	51688777	51688850	Intergenic	Intergenic	-17830	NM_19929	76901	Jade2	1200017K0
chr5	76565227	76565725	Intergenic	RLTR11A2|	-3822	NM_02061	57357	Srd5a3	1110025P1
chr6	1.23E+08	1.23E+08	Intergenic	ID_B1|SINE	-9714	NM_02189	60611	Foxj2	Fhx
chr5	1.4E+08	1.4E+08	intron (NM	intron (NM	33587	NM_00116	67604	Get4	1110007L1
chr13	1.02E+08	1.02E+08	5' UTR (NM	5' UTR (NM	169	NM_02288	69048	Slc30a5	1810010K0
chr18	74376052	74376125	5' UTR (NM	5' UTR (NM	222	NM_02886	74322	Cxxc1	2410002I1(
chr2	29820477	29820575	promoter-T	promoter-T	-554	NM_00117	20740	Sptan1	2610027H0

chr12	1.07E+08	1.07E+08	Intergenic	CpG	-47072	NM_01170	22367	Vrk1	51PK
chr19	59017927	59018000	promoter-T	promoter-T	48	NM_17868	226265	Eno4	6430537H(
chr8	87823777	87823950	promoter-T	promoter-T	332	NR_02826	56452	Orc6	6720420I1(
chr13	30637102	30637400	promoter-T	promoter-T	-66	NM_02571	66694	Uqcrfs1	4430402G1
chr8	77518152	77518250	intron (NM	CpG	599	NM_17801	70823	Hmgxb4	4733401K0
chr6	1.2E+08	1.2E+08	intron (NM	intron (NM	10461	NM_00835	16172	Il17ra	AW538159
chr16	30967952	30968175	intron (NM	intron (NM	113455	NM_19862	268880	Xxylt1	AI480653
chr9	1.1E+08	1.1E+08	exon (NM_	exon (NM_	105	NM_00108	72341	Elp6	2610001P1
chr11	49922602	49922750	intron (NM	intron (NM	-22186	NM_00129	76795	Tbc1d9b	-
chr3	90016902	90017025	promoter-T	promoter-T	606	NM_00119	1E+08	Rps27rt	ENSMUSG(
chr1	1.83E+08	1.83E+08	Intergenic	Intergenic	-45104	NM_14551	226757	Wdr26	1600024A(
chr10	74598127	74598250	intron (NM	intron (NM	74547	NM_00108	110279	Bcr	5133400C0
chr13	1.12E+08	1.12E+08	Intergenic	Intergenic	-45698	NM_17259	218613	Mier3	5730509D1
chr7	28474177	28474250	promoter-T	promoter-T	-146	NM_00111	112415	C030039L0	-
chr2	53050877	53050975	promoter-T	promoter-T	-192	NM_02298	65103	Arl6ip6	2310057C0
chr14	56279127	56279200	intron (NM	CpG	182	NM_02339	67881	Mdp1	1810034K2
chr17	46918602	46920150	intron (NM	intron (NM	229	NM_02543	66229	Rpl7l1	1500016H1
chr1	1.34E+08	1.34E+08	exon (NM_	exon (NM_	158	NM_17251	213452	Dstyk	A930019K2
chr17	27341252	27341325	promoter-T	promoter-T	95	NM_14607	224640	Lemd2	BC026588|
chr5	74368602	74368875	Intergenic	Intergenic	95698	NM_17756	69727	Usp46	1190009E2
chr12	33420077	33420300	Intergenic	Intergenic	-85012	NM_02152	59027	Nampt	1110035O1
chr3	50239752	50239925	intron (NM	intron (NM	7697	NM_01199	26570	Slc7a11	9930009M
chr15	81230352	81230550	promoter-T	promoter-T	-167	NM_17731	321003	Xpnpep3	APP3|E43C
chr14	31005577	31005650	intron (NM	L1MEc|LIN	161059	NM_00108	12289	Cacna1d	8430418G1
chr4	1.06E+08	1.06E+08	promoter-T	promoter-T	-130	NM_05327	74754	Dhcr24	2310076D1
chr11	1.03E+08	1.03E+08	intron (NM	intron (NM	4864	NM_01689	53859	Map3k14	Nik|aly
chr1	1.82E+08	1.82E+08	Intergenic	Intergenic	-22380	NM_02637	226747	Ahctf1	6230412P2
chr12	16818352	16818575	intron (NM	intron (NM	692	NM_03327	50496	E2f6	AI462434|
chr19	5609777	5609875	promoter-T	promoter-T	-119	NR_03760	81601	Kat5	AI839539|
chr6	1.16E+08	1.16E+08	intron (NM	intron (NM	-23119	NM_14538	212541	Rho	Noerg1|Op
chr5	96638977	96639300	promoter-T	promoter-T	4	NM_05315	94061	Mrpl1	2410002L0
chr4	33064052	33064175	Intergenic	Intergenic	-5860	NM_02749	52187	Rragd	5730543C0
chr4	61944152	61944525	Intergenic	Intergenic	-3125	NM_02528	20530	Slc31a2	AI604396|
chr17	21082202	21082500	promoter-T	promoter-T	-67	NM_01689	51792	Ppp2r1a	6330556D2
chr14	60216552	60216850	promoter-T	promoter-T	95	NM_00116	71891	Cdadc1	2310010M
chr14	66424577	66424850	promoter-T	promoter-T	-35	NM_02320	52033	Pbk	2810434B1
chr1	55109152	55109400	promoter-T	promoter-T	-338	NM_02642	67876	Coq10b	1500041J0
chr17	74242677	74242900	intron (NM	intron (NM	56748	NM_01172	22436	Xdh	XO|Xor|Xo
chr9	24999877	25000200	Intergenic	RLTR12B|L	-43758	NM_02058	80517	Herpud2	5031400M
chr15	58849602	58849675	intron (NM	intron (NM	63943	NM_00114	211401	Mtss1	2310003N1
chr16	49770427	49770775	Intergenic	Intergenic	71194	NM_02868	73916	Ift57	4833420A1
chr13	16990027	16990200	intron (NM	intron (NM	796486	NM_13865	192136	Sugct	5033411D1
chr7	29157177	29157300	5' UTR (NM	5' UTR (NM	443	NM_13875	101497	Plekhg2	AI194308|
chr4	8574352	8574625	Intergenic	CpG	-43065	NM_00127	320790	Chd7	A730019I0
chr10	64438727	64438850	intron (NM	intron (NM	-885785	NM_17867	216028	Lrrtm3	9630044H(
chr7	38892352	38892425	5' UTR (NM	5' UTR (NM	121	NM_00763	12447	Ccne1	AW538188
chr6	72339952	72340025	intron (NM	intron (NM	673	NM_01679	22320	Vamp8	AU041171

chr1	1.27E+08	1.27E+08	Intergenic	Intergenic	89953	NM_02373	74117	Actr3	1200003A(
chr11	1.05E+08	1.05E+08	intron (NM	intron (NM	19178	NM_00862	17534	Mrc2	Endo180|n
chr8	1.26E+08	1.26E+08	intron (NM	intron (NM	6827	NM_00128	66855	Tcf25	1100001J1
chr10	1.11E+08	1.11E+08	Intergenic	MIR|SINE|	-37232	NM_00100	237542	Osbpl8	AA536976|
chr6	1.21E+08	1.21E+08	Intergenic	Intergenic	-3102	NM_13366	14411	Slc6a12	BGT1|GAT:
chr5	1.4E+08	1.4E+08	intron (NM	intron (NM	797	NM_17700	319772	C130050O:	-
chr5	52581302	52581675	promoter-T	promoter-T	297	NM_00104	13204	Dhx15	DBP1|DEAI
chr1	39636127	39636225	Intergenic	Intergenic	-1984	NM_00103	67702	Rnf149	1600023E1
chr11	1.16E+08	1.16E+08	promoter-T	promoter-T	-229	NM_01572	11430	Acox1	AOX|Acox|
chr6	52752127	52752350	intron (NM	intron (NM	88515	NM_02581	52440	Tax1bp1	1200003J1
chrX	7667677	7667850	promoter-T	promoter-T	-146	NM_00951	22376	Was	U42471|W
chr1	1.63E+08	1.63E+08	Intergenic	Intergenic	-12287	NM_18339	240873	Tnfsf18	Gitrl
chr16	56075752	56075875	intron (NM	CpG	291	NM_00100	66315	Senp7	2410152H1
chr6	54485502	54485675	intron (NM	intron (NM	30788	NM_02726	69938	Scrn1	2810019K2
chr3	10278152	10278300	intron (NM	intron (NM	22957	NM_02931	75497	Fabp12	1700008G(
chr11	1.21E+08	1.21E+08	promoter-T	promoter-T	159	NM_14537	209318	Gps1	Cops1|Csn
chr7	1.4E+08	1.4E+08	promoter-T	promoter-T	-797	NM_00998	13017	Ctbp2	AA407280|
chr6	52167202	52167275	exon (NM_	exon (NM_	1334	NM_01045	15404	Hoxa7	AV118143|
chr16	10607827	10608000	intron (NM	intron (NM	62481	NM_00120	74374	Clec16a	4932416N1
chr3	21890877	21890975	Intergenic	Intergenic	-84648	NM_03073	81004	Tbl1xr1	8030499H(
chr16	32177102	32177300	Intergenic	Intergenic	-2685	NM_00103	328660	Bex6	B020003O(
chr6	71221927	71222125	promoter-T	promoter-T	13	NM_14556	57896	Krcc1	AA792894
chr5	1.43E+08	1.43E+08	exon (NM_	exon (NM_	238	NM_17839	74781	Wipi2	1110018O(
chr15	59464752	59464900	Intergenic	B1F1|SINE	-15383	NM_14454	211770	Trib1	A530090O:
chr5	20637177	20637375	Intergenic	Intergenic	-54809	NM_17543	212167	Gsap	A530088I0
chr4	1.47E+08	1.47E+08	intron (NM	intron (NM	-121248	NM_00101	433804	Gm13154	OTTMUSG(
chr2	30093302	30093400	promoter-T	promoter-T	62	NM_17772	241296	Lrrc8a	Lrrc8|mKIA
chr1	23853627	23853775	3' UTR (NM	3' UTR (NM	62874	NR_11097:	98366	Smap1	1700056O:
chr9	63868127	63868275	exon (NM_	exon (NM_	1665	NM_00854	17130	Smad6	Madh6|b2
chr13	48721277	48721375	promoter-T	promoter-T	-384	NM_20723	218232	Ptpdc1	AI843923|/
chr4	1.36E+08	1.36E+08	intron (NM	intron (NM	5592	NM_17773	242705	E2f2	9230110J1
chr16	32501577	32501900	intron (NM	L1ME3|LIN	5371	NM_19930	245308	Zdhhc19	Gm1744|G
chr6	30499652	30499850	intron (NM	intron (NM	8109	NM_00102	232680	Cpa2	-
chr1	97371427	97371750	Intergenic	Lx9|LINE|L	161383	NM_02632	67698	Fam174a	2310044D2
chr7	1.29E+08	1.29E+08	promoter-T	promoter-T	46	NM_02160	59288	Dctn5	4930427E1
chr1	1.01E+08	1.01E+08	Intergenic	Intergenic	372362	NM_02758	70866	Slco6d1	4921511I0!
chr14	70553552	70553725	promoter-T	promoter-T	-40	NM_14605	219158	Ccar2	2610301G1
chr6	91666452	91666700	intron (NM	RSINE1|SIN	32515	NM_00932	21366	Slc6a6	AA589629|
chr8	41473802	41474050	Intergenic	Intergenic	-35243	NM_17839	70546	Zdhhc2	5730415P0
chr1	20810052	20810325	intron (NM	CpG	106	NM_00856	17215	Mcm3	AL033361|
chr15	97142402	97142600	intron (NM	intron (NM	-64783	NM_00116	105827	Amigo2	AI415330|/
chr1	97469427	97469500	Intergenic	ORR1D2|LT	94708	NM_00918	20452	St8sia4	PST|PST-1|
chr4	1.35E+08	1.35E+08	Intergenic	Lx5|LINE|L	-3396	NM_17485	242700	Ifnlr1	CRF2-12|Il:
chr2	1.8E+08	1.8E+08	promoter-T	promoter-T	78	NM_00108	52856	Mtg2	1810011P1
chr13	23852027	23852175	TTS (NM_1	TTS (NM_1	1017	NM_17819	326619	Hist1h4a	-
chr15	77631777	77632000	intron (NM	intron (NM	40717	NM_02241	17886	Myh9	Fltn|Myhn
chr4	1.51E+08	1.51E+08	intron (NM	intron (NM	47876	NM_00108	100072	Camta1	1810059M

chr9	64021027	64021150	promoter-T	promoter-T	-106	NM_02421	67891	Rpl4	2010004J2
chr19	43748702	43748850	promoter-T	promoter-T	595	NM_14515	246696	Slc25a28	2210403D1
chr2	11466427	11466575	intron (NM	intron (NM	9055	NM_00117	170768	Pfkfb3	E330010H2
chr2	90585977	90586175	intron (NM	CpG	549	NM_01882	55935	Fnbp4	FBP30|Fnb
chr17	30141527	30141600	promoter-T	promoter-T	-469	NM_14892	21769	Zfand3	AW539211
chr7	31347977	31348125	promoter-T	promoter-T	152	NM_02549	66340	Psenen	1700023M
chr11	79072202	79072425	Intergenic	Intergenic	-4116	NM_01965	78889	Wsb1	1110056B1
chr14	31638802	31638975	5' UTR (NM	5' UTR (NM	108	NM_02883	69179	Tmem110	1810038N0
chr17	26011952	26012075	promoter-T	promoter-T	-432	NM_02668	68347	0610011F0	-
chr1	16655502	16655775	intron (NM	CpG	366	NM_02639	70397	Tmem70	1110020A0
chr18	21118952	21119175	intron (NM	B1_Mus1|S	15937	NM_02630	67664	Rnf125	4930553F0
chr12	13103877	13104100	Intergenic	Intergenic	151992	NM_13404	104721	Ddx1	AA409185|
chr15	58247452	58247700	intron (NM	intron (NM	553	NM_14595	210998	D15Ertd62	AV220772|
chr13	1.05E+08	1.05E+08	Intergenic	Intergenic	-1790	NM_00108	108154	Adamts6	5031426K1
chr11	1.03E+08	1.03E+08	intron (NM	intron (NM	4011	NM_01967	57778	Fmnl1	8030453N1
chr19	57857277	57857450	intron (NM	intron (NM	171839	NM_18141	226255	Atrnl1	AI504415|
chr12	76072252	76072425	intron (NM	L1M2|LINE	205981	NM_17280	238271	Kcnh5	Eag2
chr12	92648727	92649025	intron (NM	intron (NM	9443	NM_01164	22095	Tshr	AI481368|
chr1	60860877	60861050	Intergenic	URR1A|DN	57731	NM_00764	12487	Cd28	-
chr16	24193527	24194125	Intergenic	Intergenic	-199610	NM_17866	210126	Lpp	9430020K1
chr9	44226252	44226375	promoter-T	promoter-T	-223	NM_20137	382073	Ccdc84	D630044F2
chr6	1.35E+08	1.35E+08	intron (NM	intron (NM	1045	NM_01354	15199	Hebp1	Hebp
chr1	1.59E+08	1.59E+08	Intergenic	B1F|SINE|	-56806	NM_02388	78255	Ralgps2	1810020P1
chr11	54698202	54698600	Intergenic	Intergenic	-17955	NM_00816	14778	Gpx3	AA960521|
chr11	1.02E+08	1.02E+08	intron (NM	intron (NM	25598	NM_00881	18612	Etv4	AW414408
chr5	64265902	64266050	intron (NM	intron (NM	62242	NM_02955	76261	0610040J0	AI662686
chr18	60661027	60661350	5' UTR (NM	5' UTR (NM	449	NM_13413	106878	Smim3	2010002N0
chr6	1.34E+08	1.34E+08	intron (NM	intron (NM	136313	NM_00796	14011	Etv6	AW123102
chr5	1.15E+08	1.15E+08	promoter-T	promoter-T	-295	NM_02901	74585	Sppl3	4833416I0
chr9	1.02E+08	1.02E+08	intron (NM	intron (NM	29633	NM_02429	16716	Ky	CD1|D9Mg
chr8	83262452	83262725	intron (NM	CpG	770	NM_05312	93762	Smarca5	4933427E2
chr19	25041552	25041925	Intergenic	Intergenic	-5632	NM_14609	226043	Cbwd1	AV349248|
chr17	46010277	46010700	Intergenic	Intergenic	-139695	NM_02816	72240	1600014C2	-
chr10	36930127	36930275	Intergenic	MTC|LTR|	-71469	NM_00853	17118	Marcks	Macs|PKCS
chr1	1.83E+08	1.83E+08	Intergenic	Intergenic	-45654	NM_14551	226757	Wdr26	1600024A0
chr4	90971177	90971400	intron (NM	intron (NM	67458	NM_01048	15569	Elavl2	Hub|mel-N
chr10	1.28E+08	1.28E+08	exon (NM_	exon (NM_	1892	NM_00108	210582	Coq10a	Gm1
chr17	29401802	29401900	promoter-T	promoter-T	-935	NM_02684	68816	Ppil1	1110060O1
chr11	6100277	6100400	5' UTR (NM	5' UTR (NM	116	NM_17374	209586	Nudcd3	AI427847|
chr13	1.1E+08	1.1E+08	intron (NM	intron (NM	111967	NM_01105	238871	Pde4d	9630011N2
chr2	1.28E+08	1.28E+08	intron (NM	intron (NM	28701	NM_01690	53885	Nphp1	-
chr19	53675027	53675300	intron (NM	CpG	277	NM_00779	13006	Smc3	Bamacan|C
chr19	37449977	37450100	promoter-T	promoter-T	-855	NM_01061	16551	Kif11	Eg5|Kif8|K
chr16	44794627	44794725	3' UTR (NM	3' UTR (NM	-26165	NM_20724	239849	Cd200r4	F630107N0
chr15	81353802	81353950	Intergenic	Intergenic	57130	NM_01971	56438	Rbx1	1500002P1
chr9	65194627	65194775	exon (NM_	exon (NM_	826	NM_01668	50996	Pdcd7	C80112|ES
chr16	52295752	52296000	intron (NM	intron (NM	157234	NM_00965	11658	Alcam	AI853494|

chr16	49859127	49859350	intron (NM	intron (NM	3471	NM_01058	16423	Cd47	9130415E2
chr16	30587902	30588275	promoter-T	promoter-T	-413	NM_17806	224092	Lsg1	5830465I2
chr2	38499077	38499300	intron (NM	intron (NM	238	NM_01118	19177	Psmb7	AU020723
chr15	81641427	81641625	promoter-T	promoter-T	-318	NM_01737	21685	Tef	2310028D2
chr16	75293377	75293525	Intergenic	Intergenic	292855	NM_00125	320355	Lipi	D930038D(
chr13	1.09E+08	1.09E+08	intron (NM	intron (NM	58582	NM_14545	67263	Zswim6	2900036G(
chr11	1.02E+08	1.02E+08	intron (NM	CpG	184	NM_00115	237943	Gpatch8	5430405G2
chr7	65942427	65942500	intron (NM	intron (NM	28891	NM_00972	11982	Atp10a	Atp10c|pfa
chr1	66748852	66748975	intron (NM	intron (NM	1446	NM_02568	66646	Rpe	2810429B(
chr16	35900702	35900825	Intergenic	Intergenic	-29295	NM_00103	547253	Parp14	1600029O1
chr16	32608802	32608875	promoter-T	promoter-T	-144	NM_01163	22042	Tfrc	2610028K1
chr14	99498352	99498525	promoter-T	promoter-T	-205	NM_02932	52023	Pibf1	-
chr17	35300352	35300450	intron (NM	intron (NM	1421	NM_00119	53761	Prrc2a	3110039B(
chr14	45661002	45661425	Intergenic	MLT1D|LTI	53427	NM_00896	19217	Ptger2	EP2|Ptgere
chr11	22890102	22891875	intron (NM	CpG	395	NM_00983	12464	Cct4	2610204B2
chr16	64873477	64873800	Intergenic	Intergenic	21727	NM_17864	106143	Cggbp1	AA960172|
chr7	76244752	76244925	Intergenic	Intergenic	-350714	NM_02554	66412	Arrdc4	2410003C(
chr1	1.58E+08	1.58E+08	TTS (NM_0	TTS (NM_0	-31447	NM_00102	104009	Qsox1	1300003H(
chr1	1.35E+08	1.35E+08	5' UTR (NM	5' UTR (NM	187	NM_00109	240752	Pik3c2b	C330011J1
chr3	58380677	58380900	exon (NM_	exon (NM_	208	NM_00104	69227	Selt	2810407C(
chr5	12639652	12639825	Intergenic	L1MA8|LIN	256572	NM_02888	108151	Sema3d	4631426B1
chr18	39829302	39829475	Intergenic	Intergenic	-103763	NM_01103	18459	Pabpc2	Pabp|Pabp
chr18	9731977	9732175	intron (NM	intron (NM	24430	NM_13044	140792	Colec12	CL-P1|SRCI
chr5	23606427	23606525	promoter-T	promoter-T	68	NM_02644	52323	Klhl7	2700038B(
chr10	23869177	23869475	5' UTR (NM	5' UTR (NM	203	NM_01679	53331	Stx7	AI315064|,
chr9	62211627	62211800	intron (NM	CpG	22563	NM_00967	11737	Anp32a	Anp32|I1P
chr16	52454027	52454100	promoter-T	promoter-T	-953	NM_00965	11658	Alcam	AI853494|
chr11	98657227	98657350	exon (NM_	exon (NM_	205	NM_02872	74026	Msl1	2810017F1
chr9	45763277	45763425	promoter-T	promoter-T	-19	NM_00128	214597	Sidt2	B930096O1
chr15	76531952	76532225	5' UTR (NM	5' UTR (NM	116	NM_02712	69572	Mfsd3	2310010G1
chr6	21975552	21975650	intron (NM	intron (NM	39691	NM_00108	214642	Cped1	6720481P(
chr6	16680877	16680975	Intergenic	Intergenic	167515	NM_03119	21426	Tfec	BB107417|
chr12	71086702	71086775	intron (NM	MIRb|SINE	1251	NM_02202	64010	Sav1	1700040G(
chr12	71814377	71814650	Intergenic	Intergenic	-111988	NM_02812	319710	Frmd6	2610019M
chr7	1.33E+08	1.33E+08	intron (NM	intron (NM	1192	NM_02984	77035	Kdm8	3110005O2
chr10	87967227	87967600	promoter-T	promoter-T	-698	NM_00114	212862	Chpt1	-
chr12	1.07E+08	1.07E+08	promoter-T	promoter-T	375	NM_02965	76559	Atg2b	2410024A2
chr9	74867152	74867225	intron (NM	intron (NM	-18233	NM_00114	59046	Arpp19	19kDa|270
chr9	26918602	26918700	intron (NM	intron (NM	44355	NM_02327	83964	Jam3	1110002N2
chr2	1.64E+08	1.64E+08	Intergenic	MER21B|L'	24538	NM_13377	78928	Pigt	2510012P1
chr15	63832277	63832375	intron (NM	intron (NM	59684	NM_14484	223601	Fam49b	0910001A(
chr16	55966052	55967725	intron (NM	intron (NM	500	NM_02421	68193	Rpl24	0610008L0
chr15	77705452	77705575	Intergenic	Intergenic	-32908	NM_02241	17886	Myh9	Fltn|Myhn-
chr10	42622727	42622975	intron (NM	intron (NM	42533	NM_17293	268297	Scml4	9330161D1
chr4	84191602	84191875	intron (NM	intron (NM	129252	NM_17287	242509	Bnc2	5031434M
chr14	87590602	87590750	Intergenic	Intergenic	-49755	NM_01967	56419	Diap3	4930417P1
chr14	9004477	9004675	intron (NM	intron (NM	930	NM_02422	68263	Pdhb	2610103L0

chr17	8755627	8755750	intron (NM	intron (NM	36022	NM_00129	23984	Pde10a	-
chr7	1.49E+08	1.49E+08	promoter-T	promoter-T	-91	NM_00125	64540	Tspan4	AI325509|/
chr19	5575627	5575900	Intergenic	ETnERV-int	7689	NM_00103	381201	Ap5b1	Gm962
chr2	25118877	25118975	exon (NM_	exon (NM_	808	NM_17528	97031	Tprn	C430004E1
chr10	1.11E+08	1.11E+08	intron (NM	intron (NM	569	NM_02860	73690	Glipr1	2410114O1
chr17	46908902	46909000	promoter-T	promoter-T	-633	NM_02596	67101	2310039H(	-
chr11	69392952	69393200	promoter-T	promoter-T	-250	NM_14482	216853	Wrap53	BC021790|
chr5	34979227	34979325	exon (NM_	exon (NM_	487	NM_02666	68294	Mfsd10	0610009O(
chr2	32426452	32426550	intron (NM	intron (NM	123	NM_01007	13481	Dpm2	AW557993
chr1	59727002	59727150	intron (NM	intron (NM	602	NM_00946	22218	Sumo1	GMP1|PIC1
chr5	1.23E+08	1.23E+08	intron (NM	CpG	483	NM_01391	30841	Kdm2b	Cxxc2|E43(
chr8	1.26E+08	1.26E+08	promoter-T	promoter-T	618	NM_02049	57247	Zfp276	AW048709
chr17	9049252	9049400	intron (NM	intron (NM	54716	NM_01186	23984	Pde10a	-
chr16	4674002	4674150	intron (NM	intron (NM	5644	NM_03020	78885	Coro7	0610011B1
chr9	22271902	22272000	intron (NM	(CA)n|Simp	849	NM_01873	55934	Rp9	PAP-1|Rp9
chr4	93001702	93002125	exon (NM_	exon (NM_	289	NM_02695	69136	Tusc1	2200001D1
chr2	75569177	75569450	Intergenic	Intergenic	-26615	NM_01090	18024	Nfe2l2	AI194320|I
chr14	78304302	78304400	exon (NM_	exon (NM_	305	NM_02949	108670	Epsti1	2310046K1
chr8	1.1E+08	1.1E+08	Intergenic	ORR1E|LTR	-4298	NM_02555	66427	Cyb5b	1810044O2
chr17	56148152	56148375	promoter-T	promoter-T	-361	NM_02653	68047	Mpnd	E130307M(
chr2	76485827	76485975	exon (NM_	exon (NM_	150	NM_01187	23992	Prkra	AV120107|
chr12	87027627	87027700	promoter-T	promoter-T	-7	NM_01676	53314	Batf	B-ATF|SFA-
chr1	13583427	13583600	Intergenic	ORR1D2|LT	-3568	NM_02817	72265	Tram1	1810049E0
chr3	67177902	67178125	promoter-T	promoter-T	-6	NM_01080	17349	Mlf1	HLS7
chr3	96808502	96808725	Intergenic	Intergenic	-27712	NM_00127	14613	Gja5	5730555N1
chr8	91218277	91218375	Intergenic	Intergenic	-2601	NM_00112	74256	Cyld	2010013M
chr19	46297752	46297900	intron (NM	intron (NM	70778	NM_17893	107338	Gbf1	1700083E0
chr18	77985852	77986025	Intergenic	MuRRS4-in	20581	NM_14608	225745	Haus1	BC024400|
chr2	1.56E+08	1.56E+08	promoter-T	promoter-T	89	NM_02025	19018	Scand1	2310003H2
chr5	53843202	53843500	Intergenic	RSINE1|SIN	-103667	NM_00108	19664	Rbpj	AI843960|(
chr4	19496602	19496675	intron (NM	intron (NM	613	NM_02776	70568	Cpne3	5430428M
chr4	1.25E+08	1.25E+08	promoter-T	promoter-T	-249	NM_17524	76793	Snip1	2410133M
chr4	1.52E+08	1.52E+08	Intergenic	Intergenic	-1935	NM_20768	56226	Espn	je
chr10	5733652	5733875	intron (NM	intron (NM	732	NM_00795	13982	Esr1	AA420328|
chr4	46151677	46151850	promoter-T	promoter-T	380	NM_00103	433702	Ncbp1	AU014645
chr6	1.29E+08	1.29E+08	intron (NM	intron (NM	2726	NM_00108	330428	Tmem52b	D630042F2
chr9	1.05E+08	1.05E+08	intron (NM	intron (NM	36075	NM_17502	235574	Atp2c1	1700121J1
chr16	36210077	36210400	promoter-T	promoter-T	-251	NM_00108	408196	Gm5416	EG408196|
chr11	4968477	4968675	Intergenic	PB1D9|SIN	-3246	NM_00119	78926	Gas2l1	4930500E2
chr8	95478027	95478200	Intergenic	Intergenic	-6833	NM_00920	20538	Slc6a2	NE-T|NET|
chr2	35991952	35992050	promoter-T	promoter-T	83	NM_02642	67871	Mrrf	2400002D0
chr1	88295802	88296150	Intergenic	(CA)n|Simp	-11260	NM_01034	14767	Nmur1	FM-3|Gpr6
chr13	95558927	95559000	promoter-T	promoter-T	65	NM_00932	21371	Tbca	Tbca13
chr2	22662902	22663025	intron (NM	intron (NM	33116	NM_01945	54519	Apbb1ip	9930118P0
chr1	72560452	72560600	intron (NM	intron (NM	22612	NM_00104	381270	4-Mar	BC056494|
chrX	70473402	70473550	promoter-T	promoter-T	-169	NM_00797	14070	F8a	AI852759|I
chr13	41528652	41528875	intron (NM	ORR1C1|LT	53966	NM_00111	18003	Nedd9	Cas-L|CasL

chr7	4453427	4453575	promoter-T	promoter-T	-219	NM_02983	232807	Ppp1r12c	2410197A1
chr6	30497952	30498100	intron (NM	intron (NM	6384	NM_00102	232680	Cpa2	-
chr7	1.39E+08	1.39E+08	promoter-T	promoter-T	-251	NM_02665	68277	2310057M	3110040E1
chr6	49299852	49300250	Intergenic	MLT1E1|LT	-17687	NM_00117	231946	Fam221a	D330028D1
chr1	1.45E+08	1.45E+08	Intergenic	Lx8|LINE|L	-589964	NM_02002	26878	B3galt2	-
chr1	1.58E+08	1.58E+08	intron (NM	intron (NM	185	NM_02232	240832	Tor1aip2	1110020D1
chr4	1.17E+08	1.17E+08	promoter-T	promoter-T	-6	NM_00102	74464	Zswim5	4933426E2
chr6	1.23E+08	1.23E+08	intron (NM	MLT1H|LTI	3142	NM_00117	26888	Clec4a2	Clec4a|Cle
chr4	1.48E+08	1.48E+08	intron (NM	CpG	692	NR_02786	230908	Tardbp	1190002A2
chr2	1.7E+08	1.7E+08	Intergenic	Intergenic	-52913	NM_00115	228913	Zfp217	4933431C0
chr11	77990577	77990725	promoter-T	promoter-T	-483	NM_08084	140859	Nek8	4632401F2
chr17	27165252	27165400	promoter-T	promoter-T	245	NM_00752	12018	Bak1	Bak|N-BAK
chr4	1.44E+08	1.44E+08	promoter-T	promoter-T	-117	NM_00117	20148	Dhrs3	Rsdr1|retS
chr2	1.82E+08	1.82E+08	promoter-T	promoter-T	-71	NM_15359	245867	Pcmtd2	5330414D1
chr7	1.24E+08	1.24E+08	Intergenic	Intergenic	-27845	NM_00113	53322	Nucb2	AI607786|
chr7	1.09E+08	1.09E+08	Intergenic	L1MB2|LIN	-7900	NM_00928	20866	Stim1	SIM
chr13	1.01E+08	1.01E+08	Intergenic	Intergenic	135725	NM_01373	27220	Cartpt	Cart
chr11	48630577	48630750	5' UTR (NM	5' UTR (NM	230	NM_14537	211007	Trim41	AW552703
chr2	1.78E+08	1.78E+08	Intergenic	RLTR45|LT	-9738	NM_00117	626848	Etohi1	-
chr11	76211252	76211475	intron (NM	intron (NM	1280	NM_00875	18230	Nxn	l11Jus13
chr2	1.66E+08	1.66E+08	promoter-T	promoter-T	-260	NM_00128	228880	Zmynd8	1110013E2
chr1	1.38E+08	1.38E+08	intron (NM	intron (NM	3160	NM_00103	16565	Kif21b	2610511N2
chrX	82786627	82786700	Intergenic	Intergenic	-32698	NM_02572	66724	Tab3	4921526G0
chr13	55507027	55507125	intron (NM	intron (NM	6067	NM_01139	20505	Slc34a1	NaPi-IIa|Np
chr17	56465477	56465750	5' UTR (NM	5' UTR (NM	140	NM_17213	193796	Kdm4b	4732474L0
chr5	1.11E+08	1.11E+08	promoter-T	promoter-T	188	NM_01113	18973	Pole	-
chr12	1.06E+08	1.06E+08	Intergenic	ETnERV2-ir	25381	NM_00119	628900	Serpina3i	2B2|EG628
chr5	1.26E+08	1.26E+08	intron (NM	CpG	155	NM_20331	208144	Dhx37	Gm1050|G
chr18	20901177	20901275	intron (NM	B1_Mur4|S	3679	NM_01973	56386	B4galt6	AA536803|
chr17	71528127	71528275	Intergenic	Intergenic	-5117	NM_02288	64898	Lpin2	2610511G0
chr4	1.41E+08	1.41E+08	intron (NM	CpG	159	NM_02538	66147	Necap2	1110005F0
chr1	1.83E+08	1.83E+08	promoter-T	promoter-T	-692	NM_00821	15078	H3f3a	H3.3A
chr1	92895602	92895875	intron (NM	intron (NM	434	NM_00111	16978	Lrrfip1	AU024550
chr2	12656352	12656650	Intergenic	Intergenic	-189167	NM_00896	19212	Pter	AI790318|
chr17	56754352	56754450	TTS (NM_0	TTS (NM_0	1583	NM_01873	54217	Rpl36	-
chr10	90634102	90634300	promoter-T	promoter-T	163	NM_00108	21917	Tmpo	5630400D2
chr1	91810277	91810475	Intergenic	Intergenic	17375	NM_01026	14472	Gbx2	D130058E0
chr7	1.48E+08	1.48E+08	promoter-T	promoter-T	377	NM_00113	15461	Hras	H-ras|Ha-r
chr3	1.31E+08	1.31E+08	intron (NM	intron (NM	63318	NM_00821	15107	Hadh	AA409008|
chr17	71835477	71835550	Intergenic	Intergenic	-10830	NM_02888	74355	Smchd1	4931400A1
chr14	33015202	33015350	promoter-T	promoter-T	119	NM_01196	26430	Parg	AI413217
chr19	55390427	55391125	promoter-T	promoter-T	229	NM_00129	53611	Vti1a	1110014F1
chr18	64929252	64929400	Intergenic	Intergenic	-108672	NM_00100	54670	Atp8b1	AI451886|
chr11	49789927	49790150	intron (NM	B1F|SINE|/	-48795	NM_02154	59044	Rnf130	G1RZFP|G1
chr2	1.29E+08	1.29E+08	promoter-T	promoter-T	-421	NM_01574	20515	Slc20a1	AI607883|
chr3	1.26E+08	1.26E+08	promoter-T	promoter-T	69	NM_02381	108058	Camk2d	2810011D2
chr6	1.08E+08	1.08E+08	intron (NM	intron (NM	386	NM_01058	16438	Itpr1	D6Pas2|EN

chr5	1.09E+08	1.09E+08	exon (NM_	exon (NM_	112	NM_19901	110524	Dgkq	110kDa|DA
chr2	1.04E+08	1.04E+08	intron (NM	intron (NM	-2601	NM_00114	16909	Lmo2	Rbtn-2|Rbt
chr5	31904702	31905025	Intergenic	Intergenic	-11461	NM_02579	66845	Mrpl33	0610009M
chr17	71199827	71200350	promoter-T	promoter-T	462	NM_00937	21815	Tgif1	AA959811|
chr16	25483627	25483700	Intergenic	Intergenic	196760	NM_17516	71338	Tprg	5430420C1
chr4	1.39E+08	1.39E+08	Intergenic	Intergenic	15778	NM_00129	69902	Mrto4	2610012O2
chr5	1.36E+08	1.36E+08	Intergenic	B2_Mm2|S	-22233	NM_00889	18984	Por	4933424M
chr1	1.55E+08	1.55E+08	intron (NM	RLTR40|LT	18462	NM_00103	71836	Shcbp1l	1700012A1
chr3	89380252	89380675	intron (NM	intron (NM	56377	NM_08046	140493	Kcnn3	KCa2.3|SK3
chr11	1.17E+08	1.17E+08	intron (NM	intron (NM	-13903	NM_01738	53860	9-Sep	MSF1|Msf
chr5	1.38E+08	1.38E+08	promoter-T	promoter-T	-124	NM_20137	384309	Trim56	A130009K1
chr1	1.82E+08	1.82E+08	promoter-T	promoter-T	-507	NM_00103	226751	Cdc42bpa	A930014J1
chr18	61383852	61384000	intron (NM	intron (NM	3773	NM_14608	225600	Pde6a	Pdea|nmf2
chr2	1.35E+08	1.35E+08	Intergenic	Intergenic	-34019	NM_02914	52837	Tmx4	2810417D0
chr14	56223652	56223775	intron (NM	intron (NM	297	NM_00115	16391	Irf9	Irf-9|Isgf3g
chr6	90555002	90555075	intron (NM	CpG	164	NM_02786	71699	Slc41a3	1010001P0
chr13	59924252	59924400	intron (NM	CpG	182	NM_15353	214290	Zcchc6	6030448M
chr8	85966702	85966850	promoter-T	promoter-T	-215	NM_01970	56367	Scoc	0610011C0
chr1	74197702	74197975	Intergenic	MIR|SINE|	-2730	NM_00990	12765	Cxcr2	CD128|CDw
chr7	29062627	29062775	promoter-T	promoter-T	-24	NM_00117	434156	Eid2b	3010005C0
chr2	26462727	26462875	Intergenic	Intergenic	-2864	NM_02621	67512	Agpat2	2510002J0
chr13	38102602	38102800	intron (NM	intron (NM	-16642	NM_02596	107513	Ssr1	2510001K0
chr16	22282602	22282750	Intergenic	RLTR45|LT	-16674	NM_00918	20462	Tra2b	5730405G2
chr10	43160027	43160100	TTS (NM_0	TTS (NM_0	-38883	NM_19902	331623	Bend3	mKIAA1553
chr2	33742677	33742750	intron (NM	CpG	753	NM_17518	72543	Mvb12b	2610200O1
chr19	6118227	6118475	exon (NM_	exon (NM_	235	NM_13367	66406	Sac3d1	2410004C2
chr12	58331452	58331700	5' UTR (NM	5' UTR (NM	164	NM_00116	73490	Mipol1	1700081O0
chr11	60351077	60351550	5' UTR (NM	5' UTR (NM	128	NM_17294	268420	Alkbh5	AW050020
chr5	83647427	83647600	Intergenic	Lx2B|LINE|	136707	NM_15380	243078	Tecrl	D330017N1
chr1	1.35E+08	1.35E+08	exon (NM_	exon (NM_	1720	NM_13381	108954	Ppp1r15b	1810033K1
chr16	44988852	44989425	Intergenic	Intergenic	45347	NM_00112	74603	Cd200r3	4733401I18
chr1	39426077	39426225	intron (NM	intron (NM	1455	NM_00125	114641	Rpl31	-
chr3	60177602	60177900	Intergenic	L1M3|LINE	-99001	NM_00125	56758	Mbnl1	Mbnl|mKIA
chr2	1.8E+08	1.8E+08	promoter-T	promoter-T	293	NM_00128	26444	Psma7	C6-I
chr8	97631327	97631550	intron (NM	intron (NM	5804	NM_00114	16582	Kifc3	AI325457|
chr6	22355127	22355225	Intergenic	PB1|SINE|A	-49095	NM_13858	27999	Fam3c	D6Wsu176
chr4	87094377	87094525	Intergenic	Intergenic	-218007	NM_17242	76376	Slc24a2	2810021B1
chr9	53910777	53910875	intron (NM	intron (NM	5392	NM_00103	244882	Tnfaip8l3	9930029P0
chr6	50493602	50493700	Intergenic	L1MC5|LIN	22822	NM_00780	13063	Cycs	-
chr10	95403752	95403850	intron (NM	CpG	504	NM_00100	216238	Eea1	A430109M
chr6	82772977	82773200	Intergenic	(CATG)n|Si	-48640	NM_01382	15277	Hk2	AI642394|
chr19	7280427	7280500	intron (NM	GC_rich|Lo	311	NM_13415	107260	Otub1	AI850305
chr12	10397702	10397900	TTS (NM_0	TTS (NM_0	215	NM_02369	105014	Rdh14	3110030G1
chr2	28001577	28001775	Intergenic	RLTR16|LT	-46937	NM_00103	56177	Olfm1	AMY|AW74
chr8	1.2E+08	1.2E+08	Intergenic	Intergenic	-9658	NM_00829	15486	Hsd17b2	AI194836|A
chr10	7512552	7512825	promoter-T	promoter-T	-4	NM_02614	67418	Ppil4	3732410E1
chr2	1.53E+08	1.53E+08	promoter-T	promoter-T	-211	NM_13384	99237	Tm9sf4	AA986553|

chr10	92865802	92865975	Intergenic	Intergenic	-50253	NM_00851	16993	Lta4h	-
chr15	88961852	88962050	intron (NM	intron (NM	-2821	NR_02844	170787	Hdac10	AW548891
chr1	1.3E+08	1.3E+08	promoter-T	promoter-T	247	NM_02839	72949	Ccnt2	2900041I1
chr3	1.31E+08	1.31E+08	Intergenic	Intergenic	-85681	NM_00128	23971	Papss1	AI325286|/
chr9	1.23E+08	1.23E+08	intron (NM	intron (NM	26624	NM_01160	21922	Clec3b	Tna
chr6	1.15E+08	1.15E+08	promoter-T	promoter-T	-258	NM_01114	19016	Pparg	Nr1c3|PPA
chr1	43973852	43974050	Intergenic	Intergenic	-16901	NM_00941	22019	Tpp2	TPP-2|Tpp
chr18	36868527	36868800	Intergenic	MER44D|D	-13084	NM_17283	240216	E230025N2	EG240216
chr2	75445502	75445675	Intergenic	(TG)n|Simp	-51728	NM_14613	229279	Hnrnpa3	2410013L1
chr1	94935602	94935750	exon (NM_	exon (NM_	32765	NM_00115	208760	Aqp12	AB084104|
chr15	88649552	88649625	promoter-T	promoter-T	160	NM_00114	223774	Alg12	ECM39
chr9	65974652	65974725	exon (NM_	exon (NM_	270	NM_02663	68250	Fam96a	5730536A0
chr14	75684227	75684325	promoter-T	promoter-T	96	NM_02608	67302	Zc3h13	2600010B1
chr19	5875427	5875500	promoter-T	promoter-T	-255	NM_02616	67457	Frmd8	1200004M
chr8	23303277	23303600	exon (NM_	exon (NM_	106	NM_02733	70160	Vps36	1700010A2
chr5	1.44E+08	1.44E+08	intron (NM	CpG	173	NM_08056	108086	Rnf216	2810055G2
chr13	4152277	4152500	Intergenic	L1M4|LINE	-2511	NM_13406	105349	Akr1c18	AW146047
chr15	98702577	98702750	Intergenic	CpG	-1027	NM_00103	381022	Kmt2d	ALR|BC032
chr9	55174402	55174475	promoter-T	promoter-T	-318	NM_17292	244886	AI118078	9630029F1
chr2	1.81E+08	1.81E+08	intron (NM	intron (NM	292	NM_19931	332713	BC051628	1700026M
chr10	95079552	95079650	Intergenic	Intergenic	-52800	NM_02772	71207	Nudt4	4933436C1
chr4	11118477	11118600	promoter-T	promoter-T	40	NM_00103	12448	Ccne2	-
chrX	71696827	71697125	3' UTR (NM	3' UTR (NM	21956	NM_01054	16151	Ikbkg	1110037D2
chr17	47653927	47654000	intron (NM	intron (NM	11963	NM_00108	12445	Ccnd3	9230106B0
chr9	1.07E+08	1.07E+08	intron (NM	CpG	119	NM_01048	15587	Hyal2	AI256841|/
chr18	44988102	44988475	promoter-T	promoter-T	-31	NM_00116	240255	Ythdc2	3010002F0
chr2	81893977	81894075	exon (NM_	exon (NM_	211	NM_17551	241514	Zfp804a	C630007C1
chr15	5161002	5161350	intron (NM	intron (NM	25616	NM_02621	67515	Ttc33	2410099M
chr10	41997952	41998125	Intergenic	CpG-998	-1490	NM_01974	56484	Foxo3	1110048B1
chr3	97798052	97798325	Intergenic	Intergenic	-19273	NM_01092	18129	Notch2	AI853703|
chr2	44972027	44972225	Intergenic	Intergenic	-3327	NM_01575	24136	Zeb2	9130203F0
chr9	44371177	44371250	Intergenic	LTR33C|LT	-36709	NM_00755	12145	Cxcr5	Blr1|CXC-R
chr10	80156277	80156425	intron (NM	intron (NM	8214	NM_17245	208228	Mob3a	5330417K0
chr8	1.18E+08	1.18E+08	intron (NM	intron (NM	472631	NM_00102	17132	Maf	2810401A2
chr17	27692727	27692900	promoter-T	promoter-T	-706	NM_00116	15361	Hmga1	AL023995|
chr14	58144227	58144400	exon (NM_	exon (NM_	647	NM_14583	239114	Il17d	AI462269|
chr7	36539402	36539850	promoter-T	promoter-T	-153	NM_17870	233115	Dpy19l3	6030410G0
chr4	1.55E+08	1.55E+08	Intergenic	Intergenic	-12919	NM_01138	20481	Ski	2310012I0
chr10	24848227	24848450	Intergenic	RMER15|LT	170631	NM_01874	432442	Akap7	6430401D0
chr10	1.17E+08	1.17E+08	intron (NM	CpG	294	NM_00763	12461	Cct2	Cctb
chr11	77991927	77992100	promoter-T	promoter-T	-147	NM_00129	68385	Tlcd1	-
chr19	45132552	45132725	Intergenic	Intergenic	10572	NM_00117	94280	Sfxn3	-
chr1	93308852	93309675	intron (NM	CpG	536	NM_01947	55927	Hes6	AI326893|
chr13	55201252	55201425	intron (NM	CpG	318	NM_01130	20184	Uimc1	9430016E0
chr2	24030852	24031175	Intergenic	Intergenic	-10983	NM_15351	215257	Il1f9	Il36g
chr2	24241827	24242500	intron (NM	intron (NM	1246	NM_17761	215632	Psd4	BC046518|
chr4	99495902	99495975	promoter-T	promoter-T	-129	NM_02634	67733	Itgb3bp	4930471O1

chr19	53259752	53259825	intron (NM	intron (NM	41301	NM_01375	27360	Add3	AI463285|I
chr14	41826877	41827050	intron (NM	CpG	101	NM_02746	70564	Fam213a	5730469M
chr12	32089627	32089825	Intergenic	Intergenic	-33358	NM_02135	13487	Slc26a3	9030623B1
chr2	74657327	74657750	Intergenic	Intergenic	-6331	NM_01680	53375	Mtx2	1500012G(
chr16	38756252	38756400	intron (NM	intron (NM	13954	NM_00128	56375	B4galt4	9130402O(
chr16	16865427	16865725	Intergenic	Intergenic	-1498	NM_00119	16136	Igll1	BB139905|
chr14	55082302	55082500	promoter-T	promoter-T	-583	NM_02299	65107	Lrp10	Lrp9
chr3	1.44E+08	1.44E+08	5' UTR (NM	5' UTR (NM	131	NM_01072	16911	Lmo4	A730077C1
chr10	7676427	7676550	promoter-T	promoter-T	-567	NM_13866	68652	Tab2	1110030N(
chr2	1.57E+08	1.57E+08	intron (NM	CpG	432	NM_01124	19650	Rbl1	AW547426
chr7	1.18E+08	1.18E+08	Intergenic	Intergenic	-4392	NM_17732	319934	Sbf2	4833411B(
chr16	87611827	87612175	Intergenic	Intergenic	58426	NM_14485	224419	Map3k7cl	C21orf7|O
chr5	1.08E+08	1.08E+08	intron (NM	intron (NM	5250	NM_00796	14020	Evi5	NB4S
chr12	35670602	35670900	3' UTR (NM	3' UTR (NM	1318	NM_02929	75456	Prps1l1	1700011K1
chr17	35363352	35363550	intron (NM	intron (NM	9309	NM_01090	18038	Nfkbil1	Def-7|IKBL
chr2	1.57E+08	1.57E+08	promoter-T	promoter-T	230	NM_02612	67388	1110008F1	AI316789
chr12	32730927	32731050	intron (NM	intron (NM	15156	NM_01115	19088	Prkar2b	AI451071|/
chr13	29883377	29883650	intron (NM	intron (NM	63944	NM_14453	68916	Cdkal1	1190005B(
chr8	1.23E+08	1.23E+08	intron (NM	CpG	506	NM_00114	382034	Gse1	2210013I1
chr7	26436352	26436450	intron (NM	intron (NM	7379	NM_00753	12039	Bckdha	-
chr2	14525477	14526275	promoter-T	promoter-T	-57	NM_02311	12296	Cacnb2	AW060387
chrX	68469927	68470050	intron (NM	intron (NM	1731	NM_01992	17772	Mtm1	AF073996|
chr16	22618577	22618850	intron (NM	B1F|SINE|/	38591	NM_13865	110197	Dgkg	2900055E1
chr5	1.44E+08	1.44E+08	intron (NM	ID_B1|SINI	1052	NM_14491	231871	Daglb	E330036I1
chr13	99085052	99085125	intron (NM	intron (NM	1834	NM_14545	218490	Btf3	1700054E1
chr13	25072877	25073300	intron (NM	intron (NM	38067	NM_00815	14756	Gpld1	6330541J1
chr5	37188427	37188850	promoter-T	promoter-T	-645	NM_02624	67568	Mrfap1	9130413I2
chr2	1.74E+08	1.74E+08	intron (NM	CpG-8954	423	NM_00107	14683	Gnas	5530400H2
chr1	1.93E+08	1.93E+08	Intergenic	Intergenic	-25843	NM_14488	226849	Ppp2r5a	PR61alpha
chr3	54433427	54433650	Intergenic	Intergenic	-63489	NM_01999	56790	Supt20	AA667204|
chr1	94728027	94728525	promoter-T	promoter-T	13	NM_01669	14733	Gpc1	AI462976
chr5	1.07E+08	1.07E+08	Intergenic	Intergenic	26708	NM_00127	12545	Cdc7	AI597260|(
chr11	1.18E+08	1.18E+08	intron (NM	intron (NM	1230	NM_00111	19157	Cyth1	CLM1|CTH
chr15	25733152	25733250	intron (NM	intron (NM	-39852	NM_00103	66270	Fam134b	1810015C(
chr10	80318052	80318200	promoter-T	promoter-T	106	NM_17519	73218	Sppl2b	3110056O(
chr15	81756827	81756900	promoter-T	promoter-T	-220	NM_03022	78929	Polr3h	5031409G2
chr12	16701727	16701900	intron (NM	MTE2a|LTI	41537	NM_00874	18217	Ntsr2	NT2R|NTRI
chr2	26358202	26358375	intron (NM	CpG	1054	NM_00871	18128	Notch1	9930111A1
chr7	30907527	30907625	intron (NM	intron (NM	609	NM_00108	233060	Zfp382	5930415A(
chr18	46756927	46757100	promoter-T	promoter-T	176	NM_02569	66676	Tmed7	3930401E1
chr17	32574127	32574275	Intergenic	ID_B1|SINI	-13089	NM_00127	57757	Pglyrp2	C730002N(
chr17	32052677	32052950	Intergenic	Intergenic	-60076	NM_01083	17691	Sik1	Msk|Sik|Sr
chr13	74944427	74944650	intron (NM	intron (NM	870	NM_00981	12380	Cast	-
chr7	71286177	71286250	Intergenic	MER2|DN/	-12508	NM_00103	17364	Trpm1	4732499L0
chr6	87800302	87800825	intron (NM	CpG	537	NM_00110	12785	Cnbp	AA408710|
chr4	1.55E+08	1.55E+08	Intergenic	Intergenic	6087	NM_02198	21936	Tnfrsf18	AITR|Gitr
chr4	1.33E+08	1.33E+08	Intergenic	(TG)n|Simp	31620	NM_00128	20111	Rps6ka1	Mapkapk-1

chr10	1.28E+08	1.28E+08	TTS (NM_0	TTS (NM_0	307	NM_00113	21853	Timeless	C77407|De
chr4	1.24E+08	1.24E+08	intron (NM	CpG	157	NM_01966	56309	Mycbp	5730488M
chr7	1.14E+08	1.14E+08	Intergenic	L1MC3|LIN	67629	NM_02966	76572	Rbmxl2	1700012H(
chr3	1.44E+08	1.44E+08	Intergenic	Intergenic	91508	NM_00116	16911	Lmo4	A730077C1
chr2	1.25E+08	1.25E+08	5' UTR (NM	5' UTR (NM	212	NM_02359	110074	Dut	5031412I0
chr15	65955777	65956025	intron (NM	L1MB5|LIN	-147535	NM_00114	654498	Hhla1	F930104E1
chr1	1.8E+08	1.8E+08	Intergenic	Intergenic	-40080	NM_00116	269152	Kif26b	4832420M
chr11	94410377	94410550	promoter-T	promoter-T	58	NM_00101	237926	Rsad1	B430319G2
chr10	61980127	61980500	Intergenic	Intergenic	-9810	NM_01115	19073	Srgn	Prg|Prg1|S
chr2	70498652	70498750	promoter-T	promoter-T	-865	NR_02734	70231	Gorasp2	0610011A0
chr9	45009402	45009650	intron (NM	intron (NM	2632	NM_14540	214523	Tmprss4	mCAP2
chr8	1.09E+08	1.09E+08	promoter-T	promoter-T	255	NM_14541	214987	Chtf8	5830457O1
chr3	1.44E+08	1.44E+08	promoter-T	promoter-T	-532	NM_01072	16911	Lmo4	A730077C1
chr3	96304802	96305050	Intergenic	Intergenic	-24182	NM_02712	69585	Hfe2	2310035L1
chr7	1.35E+08	1.35E+08	intron (NM	intron (NM	545	NM_02637	67773	Kat8	2010203C0
chr18	7177027	7177300	intron (NM	Lx8|LINE|L	120736	NM_00108	74934	Armc4	4930463I2
chr7	54306352	54306450	intron (NM	intron (NM	391	NM_02643	67893	Tmem86a	1810054O1
chr6	86476202	86476775	promoter-T	promoter-T	-329	NM_01186	23983	Pcbp1	WBP17|[a]
chr14	21665477	21665550	Intergenic	Intergenic	9629	NM_00887	18792	Plau	u-PA|uPA
chr3	1.16E+08	1.16E+08	intron (NM	intron (NM	505	NM_02551	66368	Rtca	2310009A1
chr4	1.47E+08	1.47E+08	promoter-T	promoter-T	152	NM_01084	17769	Mthfr	AI323986
chr14	46278427	46278600	promoter-T	promoter-T	-695	NM_00104	114874	Ddhd1	PA-PLA1
chr1	52289527	52289625	exon (NM_	exon (NM_	500	NM_00108	14660	Gls	6330442B1
chr1	36300802	36300925	intron (NM	CpG	284	NM_19889	320011	Uggt1	0910001L1
chr7	1.28E+08	1.28E+08	intron (NM	intron (NM	9267	NM_02155	59052	Mettl9	0610012D0
chr19	34579902	34580050	intron (NM	intron (NM	21988	NM_00111	16889	Lipa	AA960673|
chr12	56094852	56094950	Intergenic	Intergenic	-7578	NM_01381	217578	Baz1a	Acf1|B930
chr4	1.43E+08	1.43E+08	intron (NM	intron (NM	10666	NM_01032	14726	Pdpn	Gp38|OTS-
chr19	8787477	8787575	intron (NM	intron (NM	861	NM_00857	17254	Slc3a2	4F2|4F2HC
chr13	23618152	23618300	Intergenic	Intergenic	-4687	NM_15317	69386	Hist1h4h	1700024H0
chr11	1.1E+08	1.1E+08	intron (NM	intron (NM	28090	NM_01194	26399	Map2k6	MEK6|MKK
chr8	13036402	13036725	promoter-T	promoter-T	-745	NM_00797	14058	F10	Cf10|fX
chr2	1.7E+08	1.7E+08	Intergenic	Intergenic	-81388	NM_00115	228913	Zfp217	4933431C0
chr18	21121752	21121925	intron (NM	intron (NM	18712	NM_02630	67664	Rnf125	4930553F0
chr15	8683702	8683775	Intergenic	Intergenic	-22931	NM_14893	20512	Slc1a3	AI504299|
chr7	1.35E+08	1.35E+08	Intergenic	RMER19B|	-46381	NM_14559	233913	BC017158	-
chr14	51674252	51674425	Intergenic	Lx8|LINE|L	2579	NM_00101	497106	Rnase12	Rai1
chr10	59811827	59812000	intron (NM	intron (NM	2314	NM_02873	74048	4632428N(	Dies1|PD-1
chr4	70070777	70071025	intron (NM	intron (NM	500	NM_14599	214444	Cdk5rap2	2900018K0
chr11	53520952	53521025	promoter-T	promoter-T	-167	NM_00901	19360	Rad50	Mrell|Rad5
chr1	1.08E+08	1.08E+08	Intergenic	Intergenic	-9157	NM_00112	227449	Zcchc2	9930114B2
chr1	1.84E+08	1.84E+08	Intergenic	Intergenic	-39106	NM_13381	98386	Lbr	AI505894|i
chr8	1.24E+08	1.24E+08	Intergenic	Intergenic	-13555	NM_08085	142682	Zcchc14	AA792890|
chr11	51450702	51450850	Intergenic	PB1D10|SI	-1378	NM_02534	66089	Rmnd5b	0610039K2
chr2	1.07E+08	1.07E+08	promoter-T	promoter-T	-59	NM_00102	212772	Arl14ep	2700007P2
chr13	78097252	78097450	intron (NR_	intron (NR_	-66812	NM_02931	75507	Pou5f2	1700013G1
chr17	13185452	13185550	promoter-T	promoter-T	-96	NM_17539	60532	Wtap	2810408K0

chr19	4294652	4294825	intron (NM	intron (NM	11484	NM_13086	110355	Adrbk1	Adrbk-1|Ba
chr4	6118177	6118400	promoter-T	promoter-T	36	NM_02653	68053	Ubxn2b	3110003A2
chr5	1.44E+08	1.44E+08	Intergenic	CpG	-22510	NM_00739	11461	Actb	Actx|E430(
chr11	1.2E+08	1.2E+08	3' UTR (NM	3' UTR (NM	-4047	NM_15305	209011	Sirt7	-
chr2	35054077	35054225	intron (NM	intron (NM	2489	NM_02669	68365	Rab14	0610030G2
chr4	1.53E+08	1.53E+08	promoter-T	promoter-T	64	NM_00785	13368	Dffb	40kDa|573
chr5	1.41E+08	1.41E+08	intron (NM	intron (NM	400	NM_17227	231834	Snx8	B130023O1
chr5	34512652	34512800	promoter-T	promoter-T	-551	NM_00128	272158	Poln	POL4P
chr3	1.28E+08	1.28E+08	promoter-T	promoter-T	9	NM_14596	211556	Ap1ar	AA407621|
chr3	97695277	97695500	Intergenic	Intergenic	-2758	NM_00128	83679	Pde4dip	4732458A0
chr4	1.1E+08	1.1E+08	intron (NM	MIRb|SINE	-24242	NM_17229	242620	Dmrta2	Dmrt5
chr11	17892327	17892400	Intergenic	Intergenic	-38485	NM_02657	68145	Etaa1	5730466H2
chr7	13619777	13619925	TTS (NM_0	TTS (NM_0	275	NM_02688	68953	Chmp2a	1500016L1
chr1	53246502	53246800	intron (NM	intron (NM	-2190	NM_02707	69397	1700019A0	-
chr14	62781952	62782350	Intergenic	Intergenic	129665	NM_17341	239133	Dleu7	BC038059
chr5	1.15E+08	1.15E+08	promoter-T	promoter-T	360	NM_05409	117146	Ube3b	AI449831|
chr14	21427152	21427350	intron (NM	intron (NM	10325	NM_03018	78787	Usp54	4930429G1
chr14	8926227	8926650	Intergenic	Intergenic	-4289	NM_14545	218699	Pxk	C230080L1
chr13	46664827	46665275	intron (NM	intron (NM	67779	NM_02605	67252	Cap2	2810452G0
chr7	1.4E+08	1.4E+08	intron (NM	intron (NM	7653	NM_00998	13017	Ctbp2	AA407280|
chr13	81003602	81003800	Intergenic	Intergenic	-18982	NM_00104	105171	Arrdc3	AI450344|
chr4	1.55E+08	1.55E+08	exon (NM_	exon (NM_	1386	NM_03187	83771	Tas1r3	Sac|T1r3
chr16	3723427	3723600	Intergenic	Intergenic	-5389	NM_00116	54483	Mefv	FMF|TRIM
chr1	34495902	34496000	promoter-T	promoter-T	566	NM_02715	69668	Ccdc115	2310061I0
chr8	28285477	28285700	promoter-T	promoter-T	-470	NM_00108	75767	Rab11fip1	2010200K2
chr11	58792727	58793025	intron (NM	intron (NM	1281	NM_05316	94091	Trim11	-
chr10	1.28E+08	1.28E+08	promoter-T	promoter-T	-452	NM_17373	211389	Suox	SO
chr2	84556202	84556350	promoter-T	promoter-T	-955	NM_14488	228136	Zdhhc5	1110032A1
chr11	79100977	79101325	Intergenic	Intergenic	-32954	NM_01965	78889	Wsb1	1110056B1
chr4	10801177	10801525	promoter-T	promoter-T	-294	NM_02600	67157	2610301B2	AI428449
chr17	6987077	6987375	promoter-T	promoter-T	-97	NM_00951	22350	Ezr	AW146364
chr8	1.25E+08	1.25E+08	exon (NM_	exon (NM_	50239	NM_00766	12555	Cdh15	AI323380|
chr9	50254477	50254675	Intergenic	Intergenic	-48054	NM_02963	76509	Plet1	0610037B2
chr5	1.23E+08	1.23E+08	intron (NM	intron (NM	9243	NM_00119	207565	Camkk2	6330570N1
chr10	19824477	19824600	intron (NM	intron (NM	-44188	NM_00863	17761	Map7	E-MAP-115
chr19	9040852	9042975	intron (NM	intron (NM	382	NM_02600	67160	Eef1g	2610301D0
chr1	1.29E+08	1.29E+08	intron (NM	B4A|SINE|	86413	NM_14512	107895	Mgat5	4930471A2
chr7	1.25E+08	1.25E+08	intron (NM	intron (NM	-55174	NM_01941	54208	Arl6ip1	AIP-6|AL02
chr19	6996077	6996175	TTS (NM_0	TTS (NM_0	172	NM_00795	26379	Esrra	ERRalpha|
chr7	99985727	99985850	TTS (NM_0	TTS (NM_0	36954	NM_00108	74041	Ddias	4632434I1
chr14	45838777	45838975	promoter-T	promoter-T	193	NM_17259	70561	Txndc16	5730420B2
chr11	1.06E+08	1.06E+08	intron (NM	CpG	118	NM_19829	21763	Tex2	4930568E0
chr7	1.5E+08	1.5E+08	exon (NM_	exon (NM_	968	NM_00855	17173	Ascl2	2410083I1
chr1	14208727	14209025	intron (NM	intron (NM	91404	NM_00125	14048	Eya1	bor
chr2	1.19E+08	1.19E+08	promoter-T	promoter-T	-64	NM_00129	18796	Plcb2	AI550384|
chr11	3135477	3135550	intron (NM	intron (NM	30876	NM_00787	13494	Drg1	AA408859|
chr1	63160277	63161000	promoter-T	promoter-T	203	NM_00108	227195	Ino80d	7330405I1

chr10	79727827	79728000	exon (NM_	exon (NM_	177	NM_13318	70248	Dazap1	2410042M
chr1	89652377	89653100	5' UTR (NM	5' UTR (NM	152	NM_00120	77040	Atg16l1	1500009K0
chr19	10663752	10663850	3' UTR (NM	3' UTR (NM	12154	NM_00128	225912	Cyb561a3	2310004G0
chr10	77013677	77013925	exon (NM_	exon (NM_	20708	NM_00840	16414	Itgb2	2E6|AI528
chr11	1.18E+08	1.18E+08	Intergenic	Intergenic	-3971	NM_00770	12702	Socs3	Cis3|Cish3
chr1	10319527	10319625	exon (NM_	exon (NM_	-96825	NM_00110	211673	Arfgef1	ARFGEP1|B
chr15	3904927	3905225	Intergenic	Intergenic	24497	NM_13409	106052	Fbxo4	1700096C1
chr8	59029627	59030150	promoter-T	promoter-T	-43	NM_01579	50753	Fbxo8	Fbx8
chr1	1.69E+08	1.69E+08	Intergenic	Intergenic	-136287	NM_17284	240894	Fmo9	4831428F0
chr3	1.38E+08	1.38E+08	intron (NM	intron (NM	21556	NM_00741	11532	Adh5	Adh-5|Adh
chr11	1.01E+08	1.01E+08	Intergenic	Intergenic	-12676	NM_01148	20848	Stat3	1110034C0
chr18	42749152	42749275	Intergenic	Intergenic	-9907	NM_18154	240239	Gpr151	C130082O0
chr17	37181677	37182050	Intergenic	MER20B|D	-1048	NM_01943	54393	Gabbr1	GABAB1|G
chr6	91649527	91649600	intron (NM	intron (NM	15502	NM_00932	21366	Slc6a6	AA589629|
chr15	99705527	99705675	intron (NM	intron (NM	286	NM_00111	65970	Lima1	Eplin
chr3	1.38E+08	1.38E+08	intron (NM	intron (NM	20487	NM_00103	70604	Dnajb14	5730496F1
chr9	1.19E+08	1.19E+08	promoter-T	promoter-T	-211	NM_13086	113868	Acaa1a	Acaa|Acaa
chr9	1.16E+08	1.16E+08	Intergenic	B4|SINE|B	-159807	NM_00937	21813	Tgfbr2	1110020H1
chr10	82085677	82085875	5' UTR (NM	5' UTR (NM	197	NM_00113	544717	1190007I0	-
chr18	62337002	62337150	TTS (NM_0	TTS (NM_0	2559	NM_00742	11555	Adrb2	Adrb-2|Ba
chr8	1.29E+08	1.29E+08	Intergenic	Intergenic	97645	NM_02421	67952	Tomm20	1810060K0
chr1	40539377	40539550	intron (NM	intron (NM	16612	NM_00836	16182	Il18r1	Il18ralpha|
chr1	39044652	39044850	promoter-T	promoter-T	92	NM_02685	68833	Pdcl3	1110061A1
chr4	1.02E+08	1.02E+08	intron (NM	intron (NM	-3389	NM_00117	18578	Pde4b	Dpde4|R74
chr14	55324627	55324850	Intergenic	Intergenic	6273	NM_20713	110794	Cebpe	C/EBPe|CR
chr12	1.14E+08	1.14E+08	Intergenic	Intergenic	-7544	NM_19841	70435	Inf2	2610204M
chr5	77334077	77334475	intron (NM	intron (NM	263	NM_17376	231326	Aasdh	A230062G0
chr2	52600852	52601000	intron (NM	CpG	257	NM_01966	56324	Stam2	1200004O1
chr8	97190452	97190850	promoter-T	promoter-T	151	NM_02419	107566	Arl2bp	1700010P1
chr18	36419702	36419975	Intergenic	Intergenic	-20978	NM_00898	19290	Pura	CAGER-1|P
chr19	21515377	21515675	intron (NM	intron (NM	31625	NM_01026	14544	Gda	AU015411
chr9	96835952	96836225	Intergenic	L1M2|LINE	-46195	NM_00128	235534	Pxylp1	9430094M
chr13	52719577	52719825	intron (NM	intron (NM	27450	NM_01151	20963	Syk	Sykb
chr9	75256802	75256950	intron (NM	intron (NM	290	NM_02741	50772	Mapk6	2610021I2
chr3	1.42E+08	1.42E+08	intron (NM	intron (NM	-90171	NM_00127	12167	Bmpr1b	AI385617|
chr5	1.01E+08	1.01E+08	promoter-T	promoter-T	-252	NM_01200	26891	Cops4	AW208976
chr17	34076002	34076075	exon (NM_	exon (NM_	1237	NM_00118	14976	Pfdn6	H-2Ke2|H2
chr8	36892002	36892100	Intergenic	L1MB1|LIN	-2438	NM_02799	71908	Cldn23	2310014B0
chr7	53247952	53248075	intron (NM	intron (NM	37643	NM_01879	27421	Abcc6	Abcc1b|DC
chr12	88141802	88142000	Intergenic	Intergenic	66582	NM_00119	627607	Lrrc74	EG627607|
chr1	1.94E+08	1.94E+08	promoter-T	promoter-T	-408	NM_01163	22033	Traf5	-
chr16	14021602	14021800	intron (NM	intron (NM	-34741	NM_00127	74482	Ifitm7	4933438K1
chr12	76696902	76697200	5' UTR (NM	5' UTR (NM	136	NM_01202	26932	Ppp2r5e	4633401M
chr2	27281752	27281950	exon (NM_	exon (NM_	494	NM_00950	22325	Vav2	2810040F1
chr7	1.34E+08	1.34E+08	promoter-T	promoter-T	40	NM_02933	75565	Ccdc101	1700023O1
chr11	77328127	77328325	promoter-T	promoter-T	-393	NM_02618	67477	Abhd15	1300007F0
chr1	16151652	16151900	Intergenic	Intergenic	55813	NM_13383	98711	Rdh10	3110069K0

chr18	36319777	36319975	intron (NM	intron (NM	36938	NM_00116	1E+08	Nrg2	Don1|NTA
chr2	1.17E+08	1.17E+08	Intergenic	Intergenic	-27781	NM_00127	114715	Spred1	5730461F1
chr5	65484477	65484600	Intergenic	MTEb|LTR	-37932	NM_17517	71778	Klhl5	1300013C1
chr1	1.73E+08	1.73E+08	promoter-T	promoter-T	-531	NM_00103	641376	Tomm40l	-
chr16	29579452	29579725	5' UTR (NM	5' UTR (NM	168	NM_00119	74143	Opa1	1200011N2
chr5	1.07E+08	1.07E+08	intron (NM	CpG	586	NM_02685	52397	Zfp644	1110068L0
chr7	20295002	20295275	intron (NM	ORR1D2|LT	5640	NM_00110	53333	Tomm40	AW539759
chr1	93154877	93155075	intron (NM	intron (NM	8080	NM_02645	67921	Ube2f	2510010F1
chr3	93310677	93310775	Intergenic	(TG)n|Simp	-13692	NM_01674	20195	S100a11	EMAPI|Em
chr19	57441627	57442050	intron (NM	intron (NM	6339	NM_14550	226252	Fam160b1	AI450540|
chr11	1.06E+08	1.06E+08	Intergenic	Intergenic	-5308	NM_01049	15896	Icam2	CD102|Ica
chr8	1.2E+08	1.2E+08	exon (NM_	exon (NM_	232	NM_00116	74440	Cmip	4933407C0
chr5	29616602	29617075	intron (NM	intron (NM	88092	NM_02029	56873	Lmbr1	1110048D1
chr19	44652727	44652850	Intergenic	MIR|SINE|	15444	NM_17695	319594	Hif1an	2310046M
chr10	61971977	61972150	Intergenic	MTE2a|LTF	-1560	NM_01115	19073	Srgn	Prg|Prg1|S
chr5	92782652	92783050	intron (NM	intron (NM	-4936	NM_02127	15945	Cxcl10	C7|CRG-2|
chr1	37374902	37374975	intron (NM	intron (NM	18255	NM_00129	269180	Inpp4a	107kDa|96
chr7	87531527	87531750	intron (NM	intron (NM	1194	NM_01019	14159	Fes	AI586313|
chr6	57531077	57531175	exon (NM_	exon (NM_	140	NM_02599	67138	Herc6	1700121D1
chr2	28554927	28555050	promoter-T	promoter-T	183	NM_02728	69987	1700026L0	MAST
chr10	33739402	33739550	promoter-T	promoter-T	-54	NM_02561	66521	Rwdd1	0710001K0
chr4	63061527	63061600	intron (NM	intron (NM	2916	NM_00104	100182	Akna	AI597013|
chr3	57026602	57026825	Intergenic	Intergenic	79128	NM_00853	17112	Tm4sf1	L6|M3s1
chr11	86900227	86900300	promoter-T	promoter-T	16	NM_02426	74133	Smg8	1200011M
chr14	68333452	68333525	promoter-T	promoter-T	179	NM_00111	108912	Cdca2	2610311M
chr7	1.4E+08	1.4E+08	Intergenic	Intergenic	110380	NM_01697	18242	Oat	AI194874
chr7	74799502	74799675	intron (NM	intron (NM	7292	NM_02590	67009	Ttc23	1600012K1
chr1	1.95E+08	1.95E+08	promoter-T	promoter-T	-97	NM_00104	15483	Hsd11b1	-
chr11	1.13E+08	1.13E+08	intron (NM	intron (NM	2853	NM_00116	69806	Slc39a11	1810074D2
chr10	62255527	62255750	promoter-T	promoter-T	-522	NM_02620	67500	Ccar1	2610511G1
chr1	1.83E+08	1.83E+08	Intergenic	Intergenic	-19921	NM_02840	72978	Cnih3	2900075G0
chr4	1.34E+08	1.34E+08	Intergenic	Intergenic	-30521	NM_20723	230815	Man1c1	AI593348
chr13	1.12E+08	1.12E+08	Intergenic	Intergenic	393074	NM_15280	20620	Plk2	Snk
chr9	1.2E+08	1.2E+08	promoter-T	promoter-T	79	NM_02597	67115	Rpl14	3100001N1
chr5	92863777	92864200	intron (NM	CpG	237	NM_18339	269113	Nup54	3110079L0
chr1	1.68E+08	1.68E+08	intron (NM	B4|SINE|B4	1340	NM_01180	433375	Creg1	AA755314|
chr9	25378052	25378150	intron (NM	intron (NM	88919	NM_02618	67484	Eepd1	2310005P0
chr16	18108302	18108375	Intergenic	Intergenic	-19055	NM_01117	19125	Prodh	Pro-1|Pro1
chr1	57462852	57463050	promoter-T	promoter-T	-441	NM_00108	68115	9430016H0	AA409999|
chr12	1.12E+08	1.12E+08	Intergenic	(GA)n|Simp	-5171	NM_01048	15519	Hsp90aa1	86kDa|89k
chr2	1.72E+08	1.72E+08	Intergenic	Intergenic	-8956	NM_00108	320664	Cass4	F730031O2
chr7	1.3E+08	1.3E+08	intron (NM	intron (NM	168087	NM_00885	18751	Prkcb	A130082F0
chr11	1.17E+08	1.17E+08	promoter-T	promoter-T	-155	NM_01135	20382	Srsf2	D11Wsu17
chr11	93746777	93746850	promoter-T	promoter-T	267	NM_00101	217109	Utp18	6230425C2
chr2	1.3E+08	1.3E+08	promoter-T	promoter-T	-610	NM_02419	67134	Nop56	2310044F1
chr4	8715927	8716050	intron (NM	intron (NM	98435	NM_00127	320790	Chd7	A730019I0
chr6	17516027	17516225	intron (NM	L1ME1|LIN	-70972	NM_00760	12343	Capza2	1110053K0

chr2	1.63E+08	1.63E+08	promoter-T	promoter-T	-931	NM_02649	67996	Srsf6	1210001E1
chr13	64253927	64254100	exon (NM_	exon (NM_	494	NM_17549	238673	Zfp367	8030486J2
chr1	1.65E+08	1.65E+08	promoter-T	promoter-T	-121	NM_01679	53330	Vamp4	D1Ertd147
chr3	1.29E+08	1.29E+08	intron (NM	intron (NM	48729	NM_00793	13809	Enpep	6030431M
chr5	1.31E+08	1.31E+08	promoter-T	promoter-T	43	NM_17250	212919	Kctd7	4932409E1
chr11	80303327	80303600	intron (NM	intron (NM	12915	NM_00987	12569	Cdk5r1	Cdk5r|D11
chr15	81576177	81576250	intron (NM	CpG	935	NM_00108	20286	Zc3h7b	Gm627|Scr
chr4	1.33E+08	1.33E+08	promoter-T	promoter-T	-132	NM_01669	53608	Map3k6	Ask2|MAP
chr3	30494302	30494375	Intergenic	CpG	4454	NM_02969	76652	Actrt3	1700119I2
chr11	51869802	51869950	intron (NM	RMER19B|	-42450	NM_01941	19052	Ppp2ca	PP2A|R753
chr11	97627677	97627925	5' UTR (NM	5' UTR (NM	126	NM_02618	67480	Cwc25	1300013D0
chr11	18921227	18921400	Intergenic	Intergenic	-2341	NM_01078	17268	Meis1	C530044H1
chr12	83631652	83631900	Intergenic	Intergenic	-86249	NM_01581	50779	Rgs6	-
chr5	90653327	90653450	promoter-T	promoter-T	-367	NM_00116	231430	Cox18	BC038311
chr5	65197452	65197775	intron (NM	intron (NM	2851	NM_00845	16599	Klf3	9930027G0
chr3	1.18E+08	1.18E+08	intron (NM	Lx8|LINE|L	191642	NM_17077	99586	Dpyd	AI315208|
chr19	29537052	29537350	intron (NM	ORR1E|LTR	51792	NM_02139	58205	Pdcd1lg2	B7-DC|Btd
chr11	75846877	75847600	Intergenic	ID_B1|SINE	-5954	NM_02965	76566	Fam101b	1500005K1
chr4	44025552	44025825	exon (NM_	exon (NM_	166	NM_00129	12757	Clta	AV026556|
chr10	41193552	41193650	Intergenic	Intergenic	-2519	NM_00116	171580	Mical1	MICAL|MI
chr12	33500877	33500975	Intergenic	MTE-int|LT	-4274	NM_02152	59027	Nampt	1110035O1
chr15	55031752	55031975	intron (NM	intron (NM	66872	NM_00103	97998	Deptor	Depdc6
chr15	80429827	80429925	intron (NM	intron (NM	-24037	NM_01081	17444	Grap2	GRAP-2|GF
chr13	95055827	95056000	promoter-T	promoter-T	-677	NM_02915	107767	Scamp1	4930505M
chr4	1.29E+08	1.29E+08	promoter-T	promoter-T	-855	NM_01689	11637	Ak2	Ak-2|D4Er
chr17	36408352	36409150	promoter-T	promoter-T	198	NM_02446	79263	Trim39	1100001D1
chr8	1.29E+08	1.29E+08	Intergenic	MIRb|SINE	-114290	NM_00116	270110	Irf2bp2	E130305N2
chr13	41758952	41759250	Intergenic	URR1B|DN	12970	NM_00114	328231	Gm5082	A030008J0
chr8	73047102	73047275	promoter-T	promoter-T	-109	NM_02936	75620	Kxd1	0610030B0
chr3	1.04E+08	1.04E+08	Intergenic	Intergenic	-44909	NM_00102	269473	Lrig2	4632419I1
chr8	9934927	9935125	Intergenic	Intergenic	41297	NM_17695	319583	Lig4	5830471N1
chr15	85166977	85167100	intron (NM	CpG	227	NM_01684	54138	Atxn10	AI325283|
chr2	72039627	72039700	intron (NM	intron (NM	-84031	NM_02305	65964	Zak	AV006891|
chr17	66868427	66868700	intron (NM	CpG	447	NM_02444	19328	Rab12	2900054P1
chr5	1.4E+08	1.4E+08	promoter-T	promoter-T	36	NM_00108	433956	Heatr2	BC053401|
chr2	1.54E+08	1.54E+08	TTS (NM_0	TTS (NM_0	-3308	NM_02587	66971	Cdk5rap1	2310066P1
chr5	13527252	13527450	intron (NM	intron (NM	128042	NM_00915	20346	Sema3a	Hsema-I|SI
chr4	43012777	43012950	intron (NM	CpG	516	NM_00950	269523	Vcp	3110001E0
chr4	94828277	94828350	Intergenic	Intergenic	-109400	NM_01059	16476	Jun	AP-1|Junc|
chr11	68821852	68821975	promoter-T	promoter-T	49	NM_00115	237823	Pfas	4432409B1
chr3	27690527	27690725	Intergenic	Intergenic	-81265	NM_17318	72007	Fndc3b	1600019O0
chr19	53260052	53260200	intron (NM	intron (NM	41639	NM_01375	27360	Add3	AI463285|
chr6	1.25E+08	1.25E+08	intron (NM	CpG	152	NM_01200	26894	Cops7a	D6Ertd35e
chr15	98557827	98557950	intron (NM	intron (NM	741	NM_02416	66120	Fkbp11	1110002O2
chr8	92204052	92204450	Intergenic	Intergenic	-636190	NM_02139	58198	Sall1	Msal-3
chr8	73421027	73421300	intron (NM	CpG	179	NM_02975	76808	Rpl18a	2510019J0
chr15	1.02E+08	1.02E+08	intron (NM	intron (NM	212	NM_02538	66151	Prr13	1110020C1

chr1	58449252	58449975	promoter-T	promoter-T	-367	NM_02582	66882	Bzw1	1200015E1
chr12	88235502	88235650	Intergenic	Intergenic	-9812	NM_14583	238330	Irf2bpl	6430527G1
chr12	86734827	86735075	Intergenic	Intergenic	-19284	NM_02677	68581	Tmed10	1110014C0
chr12	45370077	45370200	promoter-T	promoter-T	-3	NM_02616	67452	Pnpla8	1200006O1
chr8	74842602	74842675	promoter-T	promoter-T	-323	NM_00845	16598	Klf2	Lklf
chr11	59352702	59352850	Intergenic	(ATG)n|Sin	-3412	NM_14582	216799	Nlrp3	AGTAVPRL
chr10	1.02E+08	1.02E+08	Intergenic	Intergenic	220432	NM_14624	237504	Rassf9	AW322107
chr6	1.25E+08	1.25E+08	Intergenic	RMER15|LT	-12358	NM_01353	14794	Spsb2	AI461677|
chr7	1.34E+08	1.34E+08	Intergenic	MYSERV6-i	-10673	NM_14620	233890	Zfp768	BC026432|
chr1	87497502	87497725	5' UTR (NM	5' UTR (NM	335	NM_00101	434484	Sp140	-
chr13	99799102	99799250	Intergenic	Tigger7|DN	-44656	NM_02706	69382	1700024PC	H2bl1|Sub
chr5	75551852	75552100	promoter-T	promoter-T	-215	NM_00108	18595	Pdgfra	AI115593|
chr1	1.73E+08	1.73E+08	intron (NM	intron (NM	1996	NM_01018	14131	Fcgr3	CD16
chr12	1.07E+08	1.07E+08	intron (NM	B3|SINE|B	3364	NM_17861	66787	Gskip	4933433P1
chr3	27589752	27589875	intron (NM	ID_B1|SINE	19548	NM_17318	72007	Fndc3b	1600019O0
chr3	65733777	65733900	Intergenic	Intergenic	28309	NM_01993	56706	Ccnl1	2610030E2
chr1	59108177	59108475	intron (NM	intron (NM	43418	NM_17520	73463	Als2cr11	1700052H2
chr4	44085427	44085725	promoter-T	promoter-T	-31	NM_00119	50798	Gne	2310066H0
chr3	30855227	30855550	intron (NM	Lx6|LINE|L	12949	NM_00116	241915	Phc3	E030046K0
chr9	20455977	20456050	promoter-T	promoter-T	-561	NM_02337	23988	Pin1	0610025L0
chr7	1.38E+08	1.38E+08	intron (NM	MIRb|SINE	-56044	NM_02131	57752	Tacc2	mKIAA418
chr3	1.1E+08	1.1E+08	exon (NM_	exon (NM_	291	NM_17889	99890	Prmt6	AW124876
chr11	83275952	83276025	intron (NM	intron (NM	475	NM_17279	118453	Mmp28	D130023P1
chr18	4252102	4252325	Intergenic	Intergenic	86383	NM_02609	67328	Lyzl1	1700038F0
chr3	1.01E+08	1.01E+08	intron (NM	intron (NM	-24352	NM_00101	74044	Ttf2	4632434F2
chr1	59649927	59650100	intron (NM	intron (NM	76905	NM_00101	381260	Gm973	AW554518
chr3	41365802	41365875	intron (NM	B3|SINE|B	-1467	NM_17230	269424	Jade1	AU041499
chr6	1.01E+08	1.01E+08	intron (NM	RMER30|D	158728	NM_01888	55983	Pdzrn3	1110020C0
chr14	14953277	14953500	promoter-T	promoter-T	30	NM_02555	66413	Psmd6	2400006A1
chrX	1.49E+08	1.49E+08	intron (NM	RSINE1|SIN	32918	NM_01366	20591	Kdm5c	D930009K1
chr8	1.14E+08	1.14E+08	promoter-T	promoter-T	-128	NM_00791	13680	Ddx19a	DBP5|Ddx1
chr4	1.09E+08	1.09E+08	intron (NM	intron (NM	-18485	NM_00113	100273	Osbpl9	2600011I0
chr5	1.06E+08	1.06E+08	intron (NM	intron (NM	1259	NM_17870	231549	Lrrc8d	2810473G0
chr15	1.02E+08	1.02E+08	TTS (NM_0	TTS (NM_0	173	NM_02171	60315	Myg1	0610023A0
chr12	35020527	35020675	intron (NM	URR1B|DN	193153	NM_02412	79221	Hdac9	AV022454|
chr8	13337202	13337550	Intergenic	Intergenic	-1375	NM_00129	21781	Tfdp1	Dp1|Drtf1
chr6	88017827	88017950	Intergenic	Intergenic	-16579	NM_13393	103963	Rpn1	AU018702
chr19	5771152	5771350	exon (NM_	exon (NM_	150	NM_02391	78891	Scyl1	2810011O1
chr8	4238327	4238625	promoter-T	promoter-T	-264	NM_01194	26400	Map2k7	5930412N1
chr15	81169677	81169775	intron (NM	intron (NM	21469	NM_01139	20524	Slc25a17	34kDa|47k
chr3	1.16E+08	1.16E+08	promoter-T	promoter-T	-89	NM_00116	58193	Extl2	3000001D0
chr12	77635802	77636175	intron (NM	intron (NM	1441	NM_15380	263406	Plekhg3	BC030417
chr11	52174302	52174400	promoter-T	promoter-T	-266	NM_01169	22333	Vdac1	AL033343|
chr5	1.25E+08	1.25E+08	intron (NM	Tigger7|DN	10293	NM_03025	80291	Rilpl2	-
chr1	1.57E+08	1.57E+08	Intergenic	Intergenic	113453	NM_01050	15939	Ier5	-
chr11	1.17E+08	1.17E+08	intron (NM	intron (NM	-5928	NM_01738	53860	9-Sep	MSF1|Msf
chrY	2858227	2858325	Intergenic	Intergenic	471018	NM_00127	1E+08	Gm3376	Rbmy1b

chr6	83864527	83864950	intron (NM	CpG	391	NM_00871	18139	Zfml	AI323587|l
chr5	54438527	54438875	intron (NM	ORR1E|LTF	48939	NM_00108	116873	Stim2	-
chr2	3581402	3581775	Intergenic	Intergenic	-49142	NM_02562	66540	Fam107b	3110001A1
chr4	1.09E+08	1.09E+08	Intergenic	MER20|DN	55389	NM_00767	12580	Cdkn2c	C77269|IN
chr5	1.35E+08	1.35E+08	intron (NM	CpG	185	NM_03356	22384	Eif4h	AU018978
chr8	1.22E+08	1.22E+08	intron (NM	intron (NM	27516	NM_02807	72042	Cotl1	1810074P2
chr1	1.2E+08	1.2E+08	promoter-T	promoter-T	78	NM_00129	76707	Clasp1	1700030C2
chr19	46764352	46764525	promoter-T	promoter-T	42	NM_02556	66439	2010012O(	4930569L1
chr19	29458177	29458425	intron (NM	intron (NM	16373	NM_02189	60533	Cd274	A530045L1
chr13	13617952	13618175	Intergenic	Intergenic	-64613	NM_01074	17101	Lyst	D13Sfk13|l
chr6	1.4E+08	1.4E+08	intron (NM	intron (NM	43885	NM_01108	18705	Pik3c2g	C80387|PI3
chr11	69572127	69572250	promoter-T	promoter-T	-53	NM_00129	20020	Polr2a	220kDa|Rp
chr6	1.47E+08	1.47E+08	intron (NM	Lx8|LINE|L	9096	NM_14557	232536	Mrps35	MDSO23|M
chr5	1.01E+08	1.01E+08	Intergenic	Intergenic	70378	NM_17271	231510	Agpat9	1-AGPAT|1
chr4	8463102	8463425	intron (NM	CpG	472	NM_02151	59021	Rab2a	9330148M
chr4	1.28E+08	1.28E+08	exon (NM_	exon (NM_	-15364	NM_00119	54383	Phc2	A3galt2|AA
chr2	1.51E+08	1.51E+08	intron (NM	CpG	233	NM_19832	386649	Nsfl1c	Munc-18c|
chr12	71554577	71554725	intron (NM	intron (NM	510	NM_02833	72736	Tmx1	2810425A(
chrX	56466102	56466325	intron (NM	intron (NM	-46948	NM_00129	14168	Fgf13	Fhf2
chr10	98409927	98410075	intron (NM	intron (NM	32215	NM_02648	67972	Atp2b1	2810442I2
chr12	1.09E+08	1.09E+08	promoter-T	promoter-T	243	NM_02826	52690	Setd3	2610102I0
chr1	54982777	54982850	intron (NM	CpG	418	NM_00108	329154	Ankrd44	4930444A1
chr6	8159452	8159700	intron (NM	CpG	349	NM_14537	252875	Mios	C81488
chr5	90652752	90652925	exon (NM_	exon (NM_	183	NR_02808	231430	Cox18	BC038311
chrX	1.37E+08	1.37E+08	Intergenic	Intergenic	34034	NM_02146	19139	Prps1	2310010D1
chr5	1.38E+08	1.38E+08	Intergenic	Intergenic	10477	NM_01347	12007	Azgp1	Zag
chr11	78161027	78161275	intron (NM	intron (NM	4127	NM_01167	22248	Unc119	D11Bhm52
chr2	72124852	72125025	intron (NM	intron (NM	1180	NM_17808	65964	Zak	AV006891|
chr8	87230902	87231450	3' UTR (NM	3' UTR (NM	5820	NM_00853	17095	Lyl1	Lyl-1|bHLH
chr2	1.68E+08	1.68E+08	Intergenic	MIRc|SINE	-88726	NM_01120	19246	Ptpn1	PTP-1B|PT
chr19	55877777	55877900	intron (NM	intron (NM	61538	NM_00114	21416	Tcf7l2	TCF4B|TCF
chr12	32808377	32808500	Intergenic	(CAAA)n|Si	-62294	NM_01115	19088	Prkar2b	AI451071|A
chr10	94380702	94380775	intron (NM	intron (NM	26474	NM_01879	54712	Plxnc1	2510048K1
chr5	1.46E+08	1.46E+08	promoter-T	promoter-T	-112	NM_01976	56443	Arpc1a	0610010H(
chr5	1.05E+08	1.05E+08	exon (NM_	exon (NM_	475	NM_00886	18764	Pkd2	C030034P1
chr12	78058702	78059225	intron (NM	intron (NM	4272	NM_00855	17187	Max	AA960152|
chr1	1.35E+08	1.35E+08	promoter-T	promoter-T	-217	NM_13381	108954	Ppp1r15b	1810033K1
chr18	35016202	35016275	Intergenic	Intergenic	-4623	NM_00791	13653	Egr1	A530045N1
chr16	4969577	4969900	intron (NM	intron (NM	5356	NR_07337(	74684	4930451G(	-
chr2	91536452	91536725	intron (NM	B4A|SINE|	14161	NM_14552	51897	Atg13	1110053A2
chr12	86451602	86451800	promoter-T	promoter-T	-82	NM_03022	78920	Dlst	1600017E0
chr7	82508452	82508525	Intergenic	B3A|SINE|	-54340	NM_15357	64176	Sv2b	A830038F0
chr4	1.23E+08	1.23E+08	exon (NM_	exon (NM_	10403	NM_00116	442834	D830031N(	Kiaa0754|r
chr11	1.01E+08	1.01E+08	intron (NM	CpG	161	NM_00894	19183	Psmc3ip	C79099|GT
chr4	22284627	22285000	non-coding	non-coding	123	NR_10438(	269514	Fbxl4	AI836810|l
chr1	64783152	64783500	5' UTR (NM	5' UTR (NM	998	NM_00104	14367	Fzd5	5330434N(
chr10	79349052	79349150	promoter-T	promoter-T	44	NM_01577	50701	Elane	Ela2|F430(

chr19	4397777	4398025	promoter-T	promoter-T	-824	NM_00100	225876	Kdm2a	100043628
chr5	1E+08	1E+08	Intergenic	RMER15-in	96344	NM_00107	11991	Hnrnpd	Auf1|Hnrp
chr1	1.08E+08	1.08E+08	intron (NM	intron (NM	3268	NM_00112	227449	Zcchc2	9930114B2
chr8	82235202	82235650	promoter-T	promoter-T	213	NM_01575	24015	Abce1	C79080|Oa
chr6	1.38E+08	1.38E+08	promoter-T	promoter-T	90	NM_17273	232449	Dera	2010002D2
chr18	20797802	20797975	Intergenic	Intergenic	-25863	NM_01369	22139	Ttr	AA408768|
chr4	1.29E+08	1.29E+08	intron (NM	CpG	363	NM_00903	19646	Rbbp4	mRbAp48
chr3	51534627	51534725	intron (NM	intron (NM	69561	NM_17499	211666	Mgst2	GST2|MGS
chr7	7252402	7252500	promoter-T	promoter-T	-229	NM_01133	12727	Clcn4-2	Clc4-2|Clcr
chr16	36477102	36477250	Intergenic	Intergenic	-21698	NM_02528	20863	Stfa3	Stf3
chr7	3645402	3645600	promoter-T	promoter-T	289	NM_00116	66078	Tsen34	0610027F0
chr10	79786277	79786375	exon (NM_	exon (NM_	-3033	NM_02552	66374	2310011J0	AI452186|A
chr4	1.49E+08	1.49E+08	intron (NM	CpG	425	NM_02746	70556	Slc25a33	5730438N1
chr1	75138927	75139250	promoter-T	promoter-T	-146	NM_02697	69171	Cnppd1	1810031K1
chr4	41083277	41083500	intron (NM	intron (NM	334	NM_02627	67615	Ube2r2	1200003M
chr9	1.23E+08	1.23E+08	5' UTR (NM	5' UTR (NM	194	NM_00100	72309	Tmem158	2310037P2
chr1	1.46E+08	1.46E+08	Intergenic	Intergenic	102866	NM_00906	19735	Rgs2	GOS8
chr17	28906877	28907175	exon (NM_	exon (NM_	764	NM_01195	26415	Mapk13	SAPK4|Ser
chr3	1.57E+08	1.57E+08	intron (NM	intron (NM	9345	NM_01119	19218	Ptger3	EP3|Pgere
chr1	1.54E+08	1.54E+08	promoter-T	promoter-T	-1	NM_19799	69399	1700025G0	2610510E0
chr7	25689202	25689275	promoter-T	promoter-T	-127	NM_00113	16801	Arhgef1	Lbcl2|Lsc
chr8	11249877	11250050	intron (NM	intron (NM	62863	NM_00993	12826	Col4a1	Bru|Col4a-
chr4	1.03E+08	1.03E+08	intron (NM	intron (NM	-13679	NM_00129	23919	Insl5	RIF2
chr10	58725877	58726050	Intergenic	Intergenic	41293	NM_17293	268301	Sowahc	4921515A0
chr13	98011227	98011650	5' UTR (NM	5' UTR (NM	378	NM_00793	13803	Enc1	Nrpb|PIG1
chr7	25227302	25227550	intron (NM	intron (NM	3275	NM_19901	210145	Irgc1	F630044M
chr8	37514552	37514825	Intergenic	Intergenic	-37557	NM_17695	319582	6430573F1	Kiaa1456
chr19	15999527	15999750	promoter-T	promoter-T	-89	NM_17742	107272	Psat1	D8Ertd814
chr6	1.48E+08	1.48E+08	intron (NM	intron (NM	58600	NM_17879	330450	Far2	A230046P1
chr2	1.7E+08	1.7E+08	intron (NM	intron (NM	3369	NM_00103	228913	Zfp217	4933431C0
chr5	1.31E+08	1.31E+08	intron (NM	CpG	112	NM_00776	12909	Crcp	AL022669
chr9	88453777	88453925	intron (NM	intron (NM	10993	NM_00116	71640	Zfp949	4930422I0
chr6	47350002	47350150	Intergenic	Intergenic	-54247	NM_01204	26965	Cul1	-
chr5	68434677	68434775	intron (NM	intron (NM	11652	NM_00101	433899	Grxcr1	Tg(Eno2-Ga
chr2	1.67E+08	1.67E+08	Intergenic	Intergenic	-2385	NM_01983	56336	B4galt5	9430078I0
chr10	1.15E+08	1.15E+08	Intergenic	Lx6|LINE|L	-5879	NM_02445	216344	Rab21	9630024B2
chr13	43304427	43304650	intron (NM	MLT1E2|LT	-37668	NM_00125	67046	Tbc1d7	2610009C0
chr11	74462927	74463200	promoter-T	promoter-T	66	NM_00108	74148	Cluh	1300001I0
chr6	38869727	38869900	intron (NM	intron (NM	828	NM_01153	21391	Tbxas1	CYP5|CYP5
chr10	79447077	79447425	promoter-T	promoter-T	-176	NM_00100	216157	Tmem259	AC087114.
chr7	17401802	17402075	exon (NM_	exon (NM_	143	NM_17343	243853	Fkrp	A830029B1
chr18	56732652	56732725	promoter-T	promoter-T	-95	NM_00112	110695	Aldh7a1	Atq1|D18V
chr2	58999202	58999325	promoter-T	promoter-T	356	NM_17546	227937	Pkp4	5031422I0
chr4	1.18E+08	1.18E+08	promoter-T	promoter-T	134	NM_02693	69072	Ebna1bp2	1810014B1
chr5	15952852	15953025	Intergenic	Intergenic	-106375	NM_00128	15234	Hgf	C230052L0
chr2	62435502	62435625	3' UTR (NM	3' UTR (NM	-23485	NM_00798	14089	Fap	-
chr11	55282652	55282850	promoter-T	promoter-T	-503	NM_01371	27041	G3bp1	AI849976|

chr3	1.22E+08	1.22E+08	promoter-T	promoter-T	-130	NM_00899	19299	Abcd3	AI313901|/
chrX	91358602	91358825	intron (NM	L1M5|LINE	10033	NM_01176	22764	Zfx	Zfx5|Zfx5,6
chr10	93352602	93352925	5' UTR (NM	5' UTR (NM	1184	NM_01964	56307	Metap2	4930584B2
chr18	5123502	5123575	Intergenic	Intergenic	76951	NM_15315	225115	Svil	AU024053
chr1	95132402	95132900	exon (NM_	exon (NM_	177	NM_17246	208777	Sned1	6720455I24
chr1	58656252	58656475	Intergenic	L1M5|LINE	12920	NM_02559	66495	Ndufb3	2700033I1
chrX	1.36E+08	1.36E+08	intron (NM	intron (NM	4304	NM_02327	66889	Rnf128	1300002C1
chr2	24173927	24174100	Intergenic	Intergenic	-18367	NM_03116	16181	Il1rn	F630041P1
chr9	1.22E+08	1.22E+08	promoter-T	promoter-T	-206	NM_02679	26901	Deb1	1110020I0
chr4	1.27E+08	1.27E+08	promoter-T	promoter-T	-71	NM_00111	67785	Zmym4	6330503C1
chr3	1.44E+08	1.44E+08	intron (NM	intron (NM	18736	NM_00128	54673	Sh3glb1	AA409932|
chr11	84792927	84793250	Intergenic	Intergenic	21832	NM_00760	12351	Car4	AW456718
chr10	26096377	26096525	Intergenic	Intergenic	-1460	NM_17278	237339	L3mbtl3	AI481284|
chrX	55315877	55316200	Intergenic	Intergenic	32233	NM_00957	22773	Zic3	Bn|Ka
chr2	1.36E+08	1.36E+08	intron (NM	intron (NM	111372	NM_01382	18798	Plcb4	A930039J0
chr11	84336127	84336500	intron (NM	CpG	2723	NM_00849	16869	Lhx1	Lim1
chr19	24601302	24601575	intron (NM	intron (NM	28879	NM_00884	18719	Pip5k1b	PI4P5K-I[b]
chr17	28152402	28152475	intron (NM	intron (NM	65091	NM_01368	21463	Tcp11	D17Ken1|T
chr8	12983952	12984325	intron (NM	intron (NM	34720	NM_00115	17207	Mcf2l	C130040G2
chr13	67444152	67444625	exon (NM_	exon (NM_	-10892	NM_17762	218314	Zfp595	A230042K1
chr1	1.55E+08	1.55E+08	intron (NM	CpG	188	NM_00116	226517	Smg7	9430023P1
chr3	84139402	84139675	Intergenic	Intergenic	-30841	NM_00127	80890	Trim2	mKIAA051
chr15	31279577	31279675	intron (NM	intron (NM	17888	NM_00116	67434	Ankrd33b	0610012A0
chr14	66860627	66860800	intron (NM	intron (NM	-28324	NM_00116	19229	Ptk2b	CADTK|CA
chr11	72254977	72255125	exon (NM_	exon (NM_	171	NM_01677	18432	Mybbp1a	AL024407|
chr18	10532527	10532600	intron (NM	intron (NM	77787	NM_00108	77805	Esco1	A930014I1
chr4	94717702	94717800	exon (NM_	exon (NM_	1162	NM_01059	16476	Jun	AP-1|Junc|
chr1	1.86E+08	1.86E+08	intron (NM	intron (NM	18811	NM_02201	63953	Dusp10	2610306G1
chr15	63497052	63497275	Intergenic	BGLII_Mus	143131	NM_03137	83492	Gsdmc	Gsdmc1|M
chr4	1.34E+08	1.34E+08	promoter-T	promoter-T	-260	NM_00128	100163	Pafah2	2310074E2
chr8	3240752	3241025	intron (NM	L1MB3|LIN	38729	NM_01056	16337	Insr	4932439J0
chr7	50800227	50800425	Intergenic	L1MA9|LIN	-11785	NM_02129	12489	Cd33	Siglec-3|gp
chr7	86536552	86536625	promoter-T	promoter-T	-636	NM_14594	208836	Fanci	-
chr11	1.2E+08	1.2E+08	5' UTR (NM	5' UTR (NM	252	NM_00116	72055	Slc38a10	1810073N0
chr9	50270552	50270950	Intergenic	MTEb|LTR	-31879	NM_02963	76509	Plet1	0610037B2
chr1	1.93E+08	1.93E+08	promoter-T	promoter-T	-521	NR_03757	52477	Angel2	2610307I2
chr13	55701127	55701325	promoter-T	promoter-T	-246	NM_14604	218271	B4galt7	-
chr10	98728077	98728150	intron (NM	intron (NM	2248	NM_02626	67603	Dusp6	1300019I0
chr15	1.01E+08	1.01E+08	intron (NM	intron (NM	48660	NM_02153	59033	Slc4a8	AW493845
chr14	76245227	76245425	promoter-T	promoter-T	263	NM_00942	22070	Tpt1	TCTP|Trt|p
chr3	21834702	21834775	Intergenic	Intergenic	-140836	NM_03073	81004	Tbl1xr1	8030499H0
chr9	89545802	89546050	Intergenic	Intergenic	-28102	NM_15350	209743	AF529169	DD1|Kiaa1
chr1	1.33E+08	1.33E+08	intron (NM	intron (NM	5157	NM_00855	17164	Mapkapk2	AA960234|
chr5	1.36E+08	1.36E+08	exon (NM_	exon (NM_	154	NM_01024	14371	Fzd9	mfz9
chr15	78199402	78199775	Intergenic	MER45A|D	19611	NM_00116	73376	Tex33	1700061J0
chr1	1.73E+08	1.73E+08	intron (NM	intron (NM	264	NM_00108	226641	Atf6	9130025P1
chr17	15841302	15841900	promoter-T	promoter-T	-330	NM_00769	12648	Chd1	4930525N2

chr9	1.06E+08	1.06E+08	Intergenic	CpG	-1869	NM_17484	235582	Glyctk	6230410P1
chr15	38174752	38175000	Intergenic	MLT1J1|LT	25918	NM_00875	18285	Odf1	-
chr13	55141502	55141725	intron (NM	intron (NM	-18867	NM_00103	212032	Hk3	HK III|HK-II
chr12	1.17E+08	1.17E+08	exon (NM_	exon (NM_	155	NM_00951	22355	Vipr2	VPAC2|VP/
chr12	59837477	59837850	Intergenic	RMER19B|	275341	NM_00914	20334	Sec23a	Msec23|Se
chr14	61003102	61003225	Intergenic	Intergenic	6040	NM_00116	72125	Amer2	2600011E0
chr9	59135327	59135625	Intergenic	Intergenic	-3903	NM_02812	72141	Adpgk	2610017G0
chr6	88852652	88852825	intron (NM	CpG	493	NM_01190	24100	Tpra1	40kDa|Gpr
chr11	97563702	97563950	promoter-T	promoter-T	-922	NM_01197	26446	Psmb3	AL033320|
chr5	1.35E+08	1.35E+08	promoter-T	promoter-T	-57	NM_05326	114674	Gtf2ird2	1700012P1
chr1	1.66E+08	1.66E+08	Intergenic	Intergenic	25756	NM_00116	20343	Sell	AI528707|
chr1	75212027	75212350	TTS (NM_0	TTS (NM_0	3640	NM_00944	22145	Tuba4a	M[a]4|Tub
chr16	20621727	20621950	promoter-T	promoter-T	-487	NM_02842	73047	Camk2n2	2900075A1
chrX	1.39E+08	1.39E+08	intron (NM	intron (NM	2057	NM_00119	209497	Tmem164	AI316850|
chr10	98410752	98411075	intron (NM	intron (NM	33127	NM_02648	67972	Atp2b1	2810442I2
chr9	1.22E+08	1.22E+08	Intergenic	Intergenic	-2208	NM_02617	67469	Abhd5	1300003D0
chr6	1.37E+08	1.37E+08	Intergenic	Intergenic	-22637	NM_00859	17313	Mgp	Mglap
chr14	22319277	22319425	intron (NM	CpG	344	NM_01747	54169	Kat6b	AI507552|
chr6	1.25E+08	1.25E+08	promoter-T	promoter-T	87	NM_13874	110109	Nop2	120kDa|A5
chr13	1.01E+08	1.01E+08	Intergenic	MT2A|LTR	9225	NM_01373	27220	Cartpt	Cart
chr2	1.53E+08	1.53E+08	intron (NM	intron (NM	4588	NM_00103	228790	Asxl1	mKIAA0978
chr6	18467727	18467825	Intergenic	Intergenic	-2951	NM_08028	30785	Cttnbp2	3010022N2
chr19	8896877	8897025	intron (NM	intron (NM	3060	NM_00108	68693	Hnrnpul2	1110031M
chr8	87432127	87432275	promoter-T	promoter-T	-322	NM_01006	13423	Dnase2a	Dnase2
chr2	30916752	30916850	intron (NM	intron (NM	35219	NM_00117	14269	Fnbp1	1110057E0
chr17	75110252	75110625	Intergenic	Intergenic	-6652	NM_15281	74196	Ttc27	2610511O1
chr11	4194552	4194775	Intergenic	MIR|SINE|	27864	NM_00103	16878	Lif	-
chr6	84873852	84873925	intron (NM	intron (NM	145619	NM_17707	75914	Exoc6b	4930569O1
chr2	1.44E+08	1.44E+08	intron (NM	PB1D10|SI	1148	NM_00125	27054	Sec23b	-
chr13	20878902	20878975	Intergenic	Intergenic	-7044	NM_00128	27052	Aoah	4930433E1
chr4	1.23E+08	1.23E+08	Intergenic	Intergenic	17584	NM_02203	64059	Oxct2a	Oxct|Oxct2
chr10	1.1E+08	1.1E+08	intron (NM	CpG	259	NM_17255	320150	Zdhhc17	A230053P1
chr11	64792127	64792200	promoter-T	promoter-T	-374	NM_02347	68626	Elac2	1110017O0
chr10	1.23E+08	1.23E+08	intron (NM	CpG	203	NM_02760	14479	Usp15	4921514G1
chr15	34168602	34168675	intron (NM	intron (NM	857	NM_03352	114128	Laptm4b	C330023P1
chr1	51620802	51620950	Intergenic	Intergenic	-85633	NM_02869	109019	Nabp1	4930434H0
chr1	9993152	9993225	intron (NR_	intron (NR_	6029	NR_11097	69312	Ppp1r42	1700011J1
chr5	1.23E+08	1.23E+08	promoter-T	promoter-T	-249	NM_01982	56378	Arpc3	1110006A0
chrX	39689927	39690025	Intergenic	Intergenic	134944	NM_00129	20843	Stag2	9230105L2
chr1	1.31E+08	1.31E+08	Intergenic	Intergenic	-22337	NM_00991	12767	Cxcr4	CD184|Cm
chr3	95325052	95325450	Intergenic	Intergenic	-5457	NM_02128	13040	Ctss	-
chr13	74074877	74075175	promoter-T	promoter-T	189	NM_02718	69716	Trip13	2410002G2
chr9	1.14E+08	1.14E+08	promoter-T	promoter-T	-215	NM_00128	76499	Clasp2	1500004F1
chr8	87509227	87509975	Intergenic	Intergenic	-4893	NM_13325	170833	Hook2	A630054I0
chr1	64347652	64347775	Intergenic	Intergenic	-179750	NM_03356	93691	Klf7	9830124P0
chr3	68496652	68496825	intron (NM	intron (NM	1392	NM_00835	16159	Il12a	IL-12p35|Il
chr19	46184727	46185125	Intergenic	Intergenic	-21463	NM_00770	12686	Elovl3	CIN-2|Cig3

chr16	44604302	44604450	Intergenic	Intergenic	-45393	NM_17250	117606	Boc	4732455C1
chr7	82844877	82845000	intron (NM	intron (NM	-148286	NM_00112	207952	Klhl25	2810402K1
chr2	1.27E+08	1.27E+08	Intergenic	B2_Mm1t|	-12396	NM_17555	108011	Ap4e1	2310033A2
chr16	19976052	19976275	intron (NM	RMER5|LTI	6959	NM_18339	239743	Klhl6	-
chr14	1.04E+08	1.04E+08	5' UTR (NM	5' UTR (NM	254	NM_20721	105689	Mycbp2	AU023734
chr4	1.2E+08	1.2E+08	intron (NM	intron (NM	808	NM_00127	18046	Nfyc	-
chr13	93544827	93545300	intron (NM	intron (NM	19710	NM_01158	21828	Thbs4	TSP4
chr9	7184552	7184775	promoter-T	promoter-T	97	NM_02977	76863	Dcun1d5	3110001A1
chr3	68848827	68849000	promoter-T	promoter-T	-249	NM_02586	66949	Trim59	2310035M
chr16	10756327	10756525	Intergenic	Intergenic	29203	NM_00989	12703	Socs1	Cish1|Cish
chr13	1.03E+08	1.03E+08	Intergenic	Intergenic	-31266	NM_00107	18708	Pik3r1	PI3K|p50al
chr10	61158252	61158425	promoter-T	promoter-T	4	NR_073587	71767	Tysnd1	1300019N1
chr11	98927327	98927525	3' UTR (NM	3' UTR (NM	23194	NM_17256	217169	Tns4	9930017A0
chr12	4776002	4776125	promoter-T	promoter-T	10	NM_17543	207921	Fam228b	A830093I2
chr4	1.01E+08	1.01E+08	intron (NM	L2a|LINE|L	4514	NM_01070	16847	Lepr	LEPROT|Le
chr17	44915152	44915225	intron (NM	intron (NM	1068	NM_17865	109115	Supt3	2310066G2
chr2	50151902	50152250	promoter-T	promoter-T	121	NM_13383	109129	Mmadhc	2010311D0
chr17	15097152	15097225	intron (NM	intron (NM	1049	NM_02425	72057	Phf10	1810055P0
chr19	41463402	41463600	Intergenic	Intergenic	-3941	NM_03137	83490	Pik3ap1	1810044J0
chr9	66440352	66440850	exon (NM_	exon (NM_	186	NM_14493	235441	Usp3	AA409661|
chr1	1.87E+08	1.87E+08	Intergenic	Charlie14a	-28185	NM_14551	226778	Mark1	AW491150
chr13	32924377	32924600	Intergenic	Intergenic	18566	NM_02542	66222	Serpinb1a	1190005M
chr19	40734402	40734575	intron (NM	intron (NM	204	NM_00984	12495	Entpd1	2610206B0
chr6	1.16E+08	1.16E+08	intron (NM	intron (NM	21572	NM_02637	67784	Plxnd1	6230425C2
chr11	54601002	54601075	intron (NM	CpG	167	NM_17862	72729	Cdc42se2	2810404F1
chr10	79537777	79538050	intron (NM	intron (NM	21149	NM_00103	625249	Gpx4	GPx-4|GSH
chr6	96615827	96616175	Intergenic	Intergenic	394404	NM_17723	320701	Fam19a4	C130034I1
chr10	85472902	85473050	intron (NM	intron (NM	1629	NM_17777	270757	Bpifc	4732454E2
chr7	20162727	20163125	exon (NM_	exon (NM_	290	NM_02240	63872	Zfp296	2210018A1
chr9	56996077	56996150	Intergenic	Intergenic	-1272	NM_02834	72774	Neil1	2810450N1
chr9	1.23E+08	1.23E+08	Intergenic	RMER15-in	-11876	NM_00119	671232	Topaz1	Gm9524
chr1	1.37E+08	1.37E+08	exon (NM_	exon (NM_	1136	NM_17708	320139	Ptpn7	BPTP-4|C9
chr13	83213202	83213450	Intergenic	Intergenic	-429707	NM_00117	17260	Mef2c	5430401D1
chr8	73153702	73154225	exon (NM_	exon (NM_	1571	NM_01181	23886	Gdf15	MIC-1|NAG
chr7	16895377	16895500	intron (NM	CpG	506	NM_13323	170770	Bbc3	PUMA|PUI
chr18	21160452	21160700	exon (NM_	exon (NM_	734	NM_20762	56515	Rnf138	2410015A1
chr7	1.34E+08	1.34E+08	5' UTR (NM	5' UTR (NM	268	NM_00128	20399	Sh2b1	AI425885|
chr6	94650077	94650200	promoter-T	promoter-T	1	NM_00837	16206	Lrig1	D6Bwg078
chr5	1.11E+08	1.11E+08	promoter-T	promoter-T	74	NM_00946	22241	Ulk1	AU041434
chr5	68106277	68106350	intron (NM	Tigger1|DN	107191	NM_00103	330096	Shisa3	D830007B1
chr4	1.35E+08	1.35E+08	promoter-T	promoter-T	-43	NM_00992	12802	Cnr2	CB-2|CB2|
chr1	1.84E+08	1.84E+08	intron (NM	CpG	477	NM_00785	13244	Degs1	AA536663|
chr4	24825552	24825650	intron (NM	intron (NM	371	NM_02674	68493	Ndufaf4	1110007M
chr1	1.93E+08	1.93E+08	intron (NM	CpG	244	NM_02586	66950	Tmem206	2310028N0
chr3	9249677	9249825	promoter-T	promoter-T	-816	NM_17766	229055	Zbtb10	4832414A1
chr7	20147077	20148050	5' UTR (NM	5' UTR (NM	184	NM_19914	232947	Ppp1r37	Gm158|Lrr
chr4	89157802	89157900	Intergenic	Intergenic	-197038	NM_17564	242523	Dmrta1	Dmrt4

chr9	72822427	72822800	Intergenic	Intergenic	10570	NM_00119	546143	Ccpg1os	EG546143|
chr17	37095027	37095150	promoter-T	promoter-T	285	NM_02316	66136	Znrd1	1110014N(
chr9	85390602	85390750	Intergenic	Intergenic	-169919	NM_00116	212943	Fam46a	D930050G(
chr13	67562652	67562775	Intergenic	Lx4A|LINE|	-10153	NM_17771	238692	Zfp874a	C330011K1
chr16	38460252	38460400	intron (NM	intron (NM	1313	NM_00985	12519	Cd80	B71|Cd28l
chr13	58259577	58259725	intron (NM	intron (NM	641	NM_19800	75731	Idnk	5133401N(
chr5	44173402	44173575	promoter-T	promoter-T	-100	NM_17872	242960	Fbxl5	Fbl4|Fir4
chr16	49699902	49700050	intron (NM	intron (NM	569	NM_02868	73916	Ift57	4833420A1
chr6	47785902	47786000	intron (NM	CpG	291	NM_02747	272347	Zfp398	5730513I2
chr12	85202752	85203100	intron (NR_	intron (NR_	14836	NM_00127	18222	Numb	Nb
chr1	82721402	82721675	promoter-T	promoter-T	45	NM_02940	75734	Mff	5230400G2
chr2	32987052	32987200	Intergenic	Intergenic	-44402	NM_17888	99326	Garnl3	AW120551
chr1	63840977	63841275	Intergenic	Intergenic	-25285	NM_00125	74426	4933402D2	-
chr15	27594052	27594225	exon (NM_	exon (NM_	17159	NM_00124	223433	Fam105a	9830126M
chr5	1.24E+08	1.24E+08	Intergenic	Intergenic	-4614	NM_00104	208043	Setd1b	AA516740|
chr5	1.18E+08	1.18E+08	promoter-T	promoter-T	-128	NM_01885	23980	Pebp1	HCNP|Pbp
chr2	30683952	30684150	promoter-T	promoter-T	-231	NM_13334	72323	Asb6	2510004M
chr13	74729227	74729300	Intergenic	Intergenic	-48057	NM_03071	80898	Erap1	Arts1|ERA/
chr9	91746802	91747000	Intergenic	Intergenic	-340873	NM_00119	1.01E+08	Plscr5	331000
chr12	56093877	56094050	Intergenic	MLT1E2|LT	-6640	NM_01381	217578	Baz1a	Acf1|B930(
chr6	28212027	28212500	promoter-T	promoter-T	-662	NM_00108	627049	Zfp800	AA407452|
chr3	1.04E+08	1.04E+08	intron (NR_	intron (NR_	301	NM_00897	19260	Ptpn22	70zpep|PE
chr3	1.46E+08	1.46E+08	intron (NM	intron (NM	257	NM_00129	74245	Ctbs	2210401K1
chr13	95064977	95065225	Intergenic	MTC|LTR|I	-9865	NM_02915	107767	Scamp1	4930505M
chr3	83704752	83704925	intron (NM	intron (NM	-59308	NM_01190	24088	Tlr2	Ly105
chr3	1.46E+08	1.46E+08	promoter-T	promoter-T	-203	NM_01110	18749	Prkacb	Pkacb
chr2	49306627	49306825	promoter-T	promoter-T	-280	NM_17266	227867	Epc2	5830499L1
chr10	84647327	84647500	5' UTR (NM	5' UTR (NM	386	NM_00777	12952	Cry1	AU020726
chr2	1.29E+08	1.29E+08	Intergenic	Intergenic	-5893	NM_01055	16175	Il1a	Il-1a
chr3	51815202	51815300	intron (NM	intron (NM	93677	NM_00100	433586	Maml3	AV234550|
chr2	1.44E+08	1.44E+08	intron (NM	intron (NM	1623	NM_13362	81910	Rrbp1	1700087N(
chr5	66154727	66155000	intron (NM	CpG	103	NM_00102	333789	N4bp2	B3bp|E43(
chr1	1.53E+08	1.53E+08	intron (NM	CpG	427	NM_01127	19821	Rnf2	AI326319|/
chr4	1.07E+08	1.07E+08	Intergenic	Intergenic	-36951	NR_03349(	16975	Lrp8	4932703M
chr2	91252427	91252700	intron (NM	intron (NM	32236	NM_17512	228356	1110051M	AI586322
chr15	97351027	97351375	Intergenic	ORR1E-int|	185052	NM_02800	71919	Rpap3	2310042P2
chr8	1.25E+08	1.25E+08	Intergenic	MTE2a|LTF	-3464	NM_02601	67177	Cdt1	2610318F1
chr11	86940827	86941075	5' UTR (NM	5' UTR (NM	374	NM_19798	68729	Trim37	1110032A1
chr8	1.14E+08	1.14E+08	intron (NM	intron (NM	24133	NM_00116	170737	Znrf1	B830022L2
chr6	53473952	53474075	Intergenic	Intergenic	-49355	NM_17272	231991	Creb5	Crebpa|D4
chr2	72284327	72284675	Intergenic	Intergenic	-29775	NM_02586	66953	Cdca7	2310021G(
chr11	49057002	49057250	promoter-T	promoter-T	-567	NM_01079	17308	Mgat1	Mgat-1
chr9	96299952	96300100	Intergenic	Intergenic	-35308	NM_00750	11933	Atp1b3	AA409958|
chr1	90551202	90551350	Intergenic	Intergenic	47490	NM_17730	320982	Arl4c	A630084M
chr2	1.65E+08	1.65E+08	Intergenic	Intergenic	-5231	NM_01359	17395	Mmp9	AW743869
chr10	1.27E+08	1.27E+08	Intergenic	Charlie1a|I	-17497	NM_00117	216440	Os9	4632413K1
chr7	26191527	26191750	intron (NM	L2c|LINE|L	6267	NM_15357	232983	Cxcl17	VCC-1|Vcc

chr7	20531477	20531625	intron (NM	intron (NM	4541	NM_02730	70080	Igsf23	2210010C1
chr10	1.21E+08	1.21E+08	intron (NM	intron (NM	56270	NM_17302	270802	BC048403	E030027L1
chr9	90140052	90140375	intron (NM	intron (NM	25394	NM_19433	67016	Tbc1d2b	1810061M
chr8	87609202	87609275	intron (NM	intron (NM	2070	NM_01076	17159	Man2b1	AW107687
chr11	82721627	82721825	exon (NM_	exon (NM_	171	NM_14543	217011	Nle1	AL022765|
chr11	69888177	69888425	Intergenic	Intergenic	-17845	NM_00749	11890	Asgr2	ASGPR2|As
chr7	73205652	73205725	intron (NM	CpG	466	NM_02133	68981	Snrpa1	1500015N(
chr6	1.17E+08	1.17E+08	intron (NM	L1_Mur2|L	7616	NM_17804	213391	Rassf4	3830411C1
chr1	1.64E+08	1.64E+08	Intergenic	MIRc|SINE	11888	NM_00120	14103	Fasl	APT1LG1|(
chr5	1.14E+08	1.14E+08	promoter-T	promoter-T	-355	NM_01692	53890	Sart3	AU045857
chr2	1.67E+08	1.67E+08	promoter-T	promoter-T	42	NM_02323	66589	Ube2v1	0610011J0
chr10	74980702	74980775	promoter-T	promoter-T	-49	NM_02609	67332	Snrpd3	1700043E1
chr7	36039202	36039450	Intergenic	L1MB4|LIN	-38962	NM_00102	435965	Lrp3	-
chr1	53353527	53353775	promoter-T	promoter-T	189	NM_15355	227099	Pms1	-
chr3	32020452	32020600	intron (NM	B3|SINE|B	218901	NM_02823	72413	Kcnmb2	2700049B1
chr1	89905152	89905325	promoter-T	promoter-T	-112	NM_00103	227334	Usp40	B230215L0
chr6	4562952	4563100	intron (NM	L1M5|LINE	11960	NM_14539	213819	Casd1	Cas1|Cast1
chr5	1.13E+08	1.13E+08	promoter-T	promoter-T	248	NM_13864	192232	Hps4	2010205O(
chr3	1.02E+08	1.02E+08	Intergenic	Intergenic	-19212	NM_00103	242126	Slc22a15	2610034P2
chr3	1.52E+08	1.52E+08	intron (NM	ID_B1|SINE	2134	NM_00107	170822	Usp33	9830169D1
chr13	20185502	20185825	intron (NM	intron (NM	3287	NM_08028	140580	Elmo1	6330578D2
chr8	1.27E+08	1.27E+08	promoter-T	promoter-T	230	NM_01032	14712	Gnpat	AU019525
chr8	81407052	81407150	Intergenic	Intergenic	-62050	NM_03014	78651	Lsm6	1500031N1
chr6	1.09E+08	1.09E+08	Intergenic	Intergenic	-29390	NM_02601	67166	Arl8b	2610313E0
chr3	1.31E+08	1.31E+08	promoter-T	promoter-T	-381	NM_00128	23971	Papss1	AI325286|
chr1	1.67E+08	1.67E+08	intron (NM	L4|LINE|RT	3549	NM_02743	70456	Mpc2	0610006C0
chr2	1.32E+08	1.32E+08	Intergenic	Intergenic	-6007	NM_01882	54338	Slc23a2	AI844736|
chr4	35172577	35172725	intron (NM	CpG	478	NM_00108	73205	3110043O2	AI840585
chr8	1.28E+08	1.28E+08	intron (NM	intron (NM	173118	NM_17485	244667	Disc1	-
chr17	35118827	35119050	promoter-T	promoter-T	-137	NM_00120	27756	Lsm2	D17H6S56E
chr13	51196552	51196750	promoter-T	promoter-T	-265	NM_00128	20729	Spin1	Spin
chr5	91311002	91311150	Intergenic	Intergenic	-9195	NM_00817	14825	Cxcl1	Fsp|Gro1|
chr3	84129027	84129125	Intergenic	ORR1E|LTF	-20379	NM_00127	80890	Trim2	mKIAA051
chr8	80775002	80775150	intron (NM	intron (NM	37835	NM_18309	73301	Ttc29	1700031F1
chr11	76659377	76659600	intron (NM	intron (NM	1022	NM_00775	12874	Cpd	AA960140|
chr15	80874227	80874325	intron (NM	intron (NM	61201	NM_15304	223701	Mkl1	AMKL|Bsac
chr8	72757902	72757975	promoter-T	promoter-T	-187	NM_00116	234373	Sugp2	Sfrs14|Srsf
chr17	28068852	28069000	intron (NM	intron (NM	22675	NM_18141	224650	Anks1	Anks1a|mK
chr17	28938177	28938250	5' UTR (NM	5' UTR (NM	142	NM_00108	268936	Brpf3	AI413466|
chr4	10879652	10879850	Intergenic	L1_Mur3|L	55015	NM_17517	71801	Plekhf2	1110070J0
chr6	1.16E+08	1.16E+08	promoter-T	promoter-T	60	NM_02792	71779	8-Mar	1300017E0
chr7	20408927	20409100	promoter-T	promoter-T	-909	NM_03360	12051	Bcl3	AI528691|
chr11	99091477	99091775	intron (NM	CpG	705	NM_02061	57376	Smarce1	2810417B2
chr4	25208827	25208900	exon (NM_	exon (NM_	105	NM_02619	67490	Ufl1	1810074P2
chr19	4200477	4200650	exon (NM_	exon (NM_	1040	NM_01123	19367	Rad9a	Rad9
chr4	1.29E+08	1.29E+08	intron (NM	B1F|SINE|	2070	NM_00103	11637	Ak2	Ak-2|D4Ert
chr18	11815677	11815825	promoter-T	promoter-T	-107	NR_04552	225182	Rbbp8	9930104E2

chr14	48182202	48182450	intron (NM	intron (NM	5783	NM_17259	1.01E+08	Atg14	4832427M
chr10	62226552	62226650	intron (NM	intron (NM	28515	NM_02620	67500	Ccar1	2610511G1
chr12	72069652	72069725	Intergenic	Intergenic	-5922	NM_01118	19167	Psma3	Lmpc8
chr3	33919377	33919500	intron (NM	CpG	437	NM_00111	14359	Fxr1	1110050J0
chr10	98344402	98344475	Intergenic	Intergenic	-33348	NM_02648	67972	Atp2b1	2810442I2
chr7	24974652	24974800	Intergenic	Intergenic	14662	NM_00102	232966	Zfp114	Gm1957|Z
chr10	81591502	81591600	intron (NM	intron (NM	793	NM_00116	216177	AU041133	-
chr10	1.22E+08	1.22E+08	intron (NM	CpG	345	NM_15305	216395	Tmem5	6330415D2
chr1	1.66E+08	1.66E+08	Intergenic	Intergenic	21456	NM_00116	20343	Sell	AI528707|
chr11	87181752	87181875	Intergenic	Intergenic	-9317	NM_17716	320472	Ppm1e	AW049266
chr1	1.34E+08	1.34E+08	intron (NM	intron (NM	20295	NM_17887	68875	Tmcc2	1110063G1
chr17	34740327	34740525	promoter-T	promoter-T	80	NM_01940	54197	Rnf5	2410131O0
chr6	89371552	89371700	intron (NM	intron (NM	-59019	NM_00888	18844	Plxna1	2600013D0
chr5	18815402	18816225	intron (NM	Lx9|LINE|L	82949	NM_00117	50791	Magi2	AIP-1|Acvr
chr6	1.17E+08	1.17E+08	Intergenic	Lx5|LINE|L	7607	NM_02605	67255	Zfp422	2900028O2
chr16	35046527	35046675	intron (NM	intron (NM	24094	NM_02358	70757	Ptplb	6330408J2
chrX	1.05E+08	1.05E+08	Intergenic	Intergenic	45949	NM_00103	213450	Gm732	-
chr9	1.15E+08	1.15E+08	Intergenic	URR1B|DN	-5427	NM_00125	102545	Cmtm7	AI481279|
chr5	1.23E+08	1.23E+08	promoter-T	promoter-T	34	NM_02688	68948	Fam216a	1500011H2
chr9	1.1E+08	1.1E+08	intron (NM	intron (NM	-13974	NM_01388	29873	Cspg5	Caleb|Ngc
chr11	6314927	6315025	promoter-T	promoter-T	-897	NM_00890	268373	Ppia	2700098C0
chr6	1.43E+08	1.43E+08	5' UTR (NM	5' UTR (NM	176	NM_02925	75320	Etnk1	1110061E1
chrX	11241477	11241650	Intergenic	Intergenic	336615	NM_00111	236663	Gm4906	EG236663
chr13	41930177	41930350	intron (NM	Lx8|LINE|L	12594	NM_17541	109254	Adtrp	9530008L1
chr4	8463952	8464250	intron (NM	intron (NM	1310	NM_02151	59021	Rab2a	9330148M
chr7	79410177	79410375	intron (NM	intron (NM	41205	NM_00102	244049	Mctp2	Gm489
chr2	1.29E+08	1.29E+08	promoter-T	promoter-T	-87	NM_17840	78751	Zc3h6	4631426G0
chr8	1.31E+08	1.31E+08	Intergenic	Intergenic	-126266	NM_01057	16412	Itgb1	4633401G2
chr7	20086452	20086775	Intergenic	Intergenic	7067	NM_17769	232946	Bloc1s3	BC043666|
chr8	1.27E+08	1.27E+08	promoter-T	promoter-T	817	NM_19810	102058	Exoc8	AI414418|
chr15	75771127	75771775	promoter-T	promoter-T	69	NM_03019	78834	Zfp623	2610029D0
chr1	1.52E+08	1.52E+08	3' UTR (NM	3' UTR (NM	6672	NM_01119	19225	Ptgs2	COX2|Cox-
chr19	54278252	54278350	Intergenic	Intergenic	158629	NM_00741	11551	Adra2a	AW122659
chr3	16083302	16083475	non-coding	non-coding	205	NM_00114	229096	Ythdf3	9130022A1
chr11	5344652	5344825	5' UTR (NM	5' UTR (NM	112	NM_00108	407821	Znrf3	Gm1167
chr4	21654752	21654925	promoter-T	promoter-T	-10	NM_00129	51813	Ccnc	AI451004|
chr17	36160627	36160725	intron (NR_	intron (NR_	-3171	NM_00820	15042	H2-T24	H-2T24
chr8	87396452	87396575	5' UTR (NM	5' UTR (NM	356	NM_00116	71846	Syce2	1700013H1
chr11	6315227	6315575	promoter-T	promoter-T	-472	NM_00890	268373	Ppia	2700098C0
chr1	1.86E+08	1.86E+08	promoter-T	promoter-T	189	NM_02851	73363	1700056E2	-
chr5	1.21E+08	1.21E+08	intron (NM	intron (NM	800	NM_13322	170756	Slc8b1	AF261233|
chr5	1.38E+08	1.38E+08	exon (NM_	exon (NM_	1841	NM_02391	78829	Tsc22d4	0610009M
chr1	1.67E+08	1.67E+08	promoter-T	promoter-T	-612	NM_14551	108735	Sft2d2	2010005O1
chr2	86015702	86015875	Intergenic	RLTR45|LT	-3816	NM_14701	259013	Olfr1044	MOR185-4
chr9	50489952	50490125	intron (NM	intron (NM	-22153	NM_14561	235339	Dlat	6332404G0
chrX	50673827	50673975	Intergenic	PB1D10|SI	47291	NM_02867	73866	Fam122c	4930432H1
chr3	1.29E+08	1.29E+08	intron (NM	intron (NM	53154	NM_00793	13809	Enpep	6030431M

chr14	21748327	21748500	promoter-T	promoter-T	-242	NM_00950	22330	Vcl	9430097D2
chr11	17111852	17112025	promoter-T	promoter-T	42	NM_17890	103784	Wdr92	AI553587|I
chr3	1.46E+08	1.46E+08	exon (NM_	exon (NM_	225	NM_00974	12042	Bcl10	AI132454|I
chr5	1.44E+08	1.44E+08	intron (NM	B1_Mm|SI	886	NM_08056	108086	Rnf216	2810055G2
chr7	25292227	25292750	intron (NR_	CpG	382	NR_11095	232969	Zfp428	2410005H0
chr7	50235027	50235100	Intergenic	IAPLTR3-in	-39105	NM_00110	668612	Gm9268	EG668612|
chr12	1.04E+08	1.04E+08	intron (NM	L1MB8|LIN	64975	NM_00116	217835	Rin3	6430500K0
chr10	80214752	80214850	Intergenic	Intergenic	-3150	NM_19932	208266	Dot1l	A630076O0
chr1	1.68E+08	1.68E+08	promoter-T	promoter-T	96	NM_17517	71592	Pogk	9130401E2
chr4	1.09E+08	1.09E+08	5' UTR (NM	5' UTR (NM	214	NM_00767	12580	Cdkn2c	C77269|IN
chr19	56466052	56466175	Intergenic	Charlie8|D	-1585	NM_00128	18175	Nrap	-
chr19	47911727	47911825	promoter-T	promoter-T	75	NM_02755	1E+08	Cfap43	4632415N1
chr9	85643227	85643375	promoter-T	promoter-T	-360	NM_00108	108837	Ibtk	5430411K1
chr11	61961877	61962325	intron (NM	intron (NM	71575	NM_00102	432572	Specc1	2810012G0
chr9	99039727	99039975	intron (NM	intron (NM	779	NM_02909	74769	Pik3cb	1110001J0
chr7	1.47E+08	1.47E+08	Intergenic	Intergenic	-21257	NM_18314	212111	Inpp5a	-
chr8	23521327	23521450	promoter-T	promoter-T	-424	NM_02056	57312	Mrps31	1500002D0
chr10	79537502	79537600	intron (NM	intron (NM	20787	NM_00103	625249	Gpx4	GPx-4|GSH
chr7	28393152	28393350	intron (NM	intron (NM	2372	NM_00743	11652	Akt2	2410016A1
chr15	26406677	26406775	Intergenic	Intergenic	167899	NM_17759	211147	11-Mar	9630025C2
chr1	1.54E+08	1.54E+08	intron (NM	intron (NM	-65216	NM_00103	66967	Edem3	2310050N1
chr12	1.14E+08	1.14E+08	5' UTR (NM	5' UTR (NM	194	NM_02819	72308	Brf1	2510002F2
chr1	1.93E+08	1.93E+08	Intergenic	Intergenic	-23631	NM_14488	226849	Ppp2r5a	PR61alpha
chr17	37407252	37407525	intron (NM	intron (NM	209	NM_01381	14991	H2-M3	H-2M3|Hm
chr5	1.24E+08	1.24E+08	intron (NM	intron (NM	1715	NM_02749	70650	Zcchc8	5730565F0
chr10	1.27E+08	1.27E+08	promoter-T	promoter-T	-320	NM_17373	210035	Tmem194	Tmem194a
chr3	84698477	84698650	intron (NM	intron (NM	30496	NM_00117	50754	Fbxw7	1110001A1
chr10	84381177	84381250	intron (NM	MIRc|SINE	855	NM_18317	237422	Ric8b	BC051080|
chr3	67386777	67386850	exon (NM_	exon (NM_	123	NM_02581	66868	Mfsd1	1200003O0
chr10	95928702	95928800	Intergenic	L2a|LINE|L	-150884	NM_00756	12226	Btg1	AI426953|A
chr4	1.32E+08	1.32E+08	intron (NM	B3A|SINE|	-10645	NM_00112	269587	Epb4.1	4.1R|AI415
chr7	26796427	26796525	Intergenic	IAP-d-int|L	-50038	NM_00781	13089	Cyp2b13	-
chr8	1.27E+08	1.27E+08	intron (NM	CpG	282	NM_13927	108148	Galnt2	AI480629
chr5	1.48E+08	1.48E+08	promoter-T	promoter-T	-728	NM_01964	19933	Rpl21	8430440E0
chr11	79391802	79392050	3' UTR (NM	3' UTR (NM	-12788	NM_17554	268451	Rab11fip4	A730072L0
chr1	1.58E+08	1.58E+08	promoter-T	promoter-T	-278	NM_00116	208263	Tor1aip1	LAP1|Lap1
chr18	58663752	58663900	Intergenic	Lx7|LINE|L	-52068	NM_00108	225579	Slc27a6	4732438L2
chr2	1.29E+08	1.29E+08	promoter-T	promoter-T	-4	NM_00129	19261	Sirpa	AI835480|I
chr15	79963402	79963625	promoter-T	promoter-T	-71	NM_02560	66513	Tab1	2310012M
chr13	99086252	99086600	intron (NM	intron (NM	496	NM_14545	218490	Btf3	1700054E1
chr12	88155552	88155950	Intergenic	Intergenic	70013	NM_14583	238330	Irf2bpl	6430527G1
chr11	1.15E+08	1.15E+08	promoter-T	promoter-T	-395	NM_00100	445007	Nup85	Pcnt1|frou
chr5	1.24E+08	1.24E+08	exon (NM_	exon (NM_	164	NM_02600	67151	Psmd9	1500011J2
chr1	64511502	64511650	Intergenic	Intergenic	-67802	NM_13382	12912	Creb1	2310001E1
chr3	89017602	89017825	intron (NM	CpG	544	NM_00116	17827	Mtx1	Gcap6|Mtx
chr7	1.49E+08	1.49E+08	promoter-T	promoter-T	-499	NM_02580	66853	Pnpla2	0610039C2
chr7	87542952	87543050	exon (NM_	exon (NM_	4647	NM_01104	18550	Furin	9130404I0

chr15	76531202	76531275	promoter-T	promoter-T	-734	NM_02712	69572	Mfsd3	2310010G1
chr16	49797327	49797475	Intergenic	LTR16B1|L	-58366	NM_01058	16423	Cd47	9130415E2
chr10	85391277	85391475	exon (NM_	exon (NM_	140	NM_00116	67341	Ascl4	1700054F1
chr1	1.68E+08	1.68E+08	promoter-T	promoter-T	-229	NM_00108	68481	Mpzl1	1110007A1
chr1	1.55E+08	1.55E+08	intron (NM	B3|SINE|B	2643	NM_01087	17970	Ncf2	NOXA2|Nc
chr11	1E+08	1E+08	intron (NM	CpG	894	NM_01979	56354	Dnajc7	2010003F2
chr14	22576852	22577000	intron (NM	intron (NM	7173	NM_02628	67630	Samd8	1110053F0
chr2	50943702	50943775	Intergenic	Intergenic	60893	NM_02881	74194	Rnd3	2610017M
chr11	55283802	55283875	intron (NM	intron (NM	584	NM_01371	27041	G3bp1	AI849976|
chr15	1E+08	1E+08	promoter-T	promoter-T	308	NM_01187	23994	Dazap2	AI314727|
chr8	73219452	73219600	Intergenic	Intergenic	-2112	NM_01059	16478	Jund	Jund1
chr4	1.29E+08	1.29E+08	promoter-T	promoter-T	166	NM_01879	54709	Eif3i	36kDa|D4E
chr11	97745227	97745400	5' UTR (NM	5' UTR (NM	155	NM_00108	72973	Fbxo47	2900052P0
chr5	1.16E+08	1.16E+08	Intergenic	U4|snRNA	-5045	NM_13376	75387	Sirt4	4930596O1
chr9	59333752	59333825	exon (NM_	exon (NM_	393	NM_01992	23806	Arih1	AU021774
chr4	43978727	43978825	intron (NM	intron (NM	8202	NM_02745	384009	Glipr2	5730414A0
chr13	14732302	14732675	Intergenic	Intergenic	9963	NM_13406	105351	AW209491	-
chr4	1.34E+08	1.34E+08	Intergenic	B1_Mus1|S	-30983	NM_20723	230815	Man1c1	AI593348
chr7	3656902	3657050	intron (NM	intron (NM	1333	NM_02976	76846	Rps9	3010033P0
chr1	60656352	60656475	Intergenic	Intergenic	-32804	NM_00104	77300	Raph1	9430025M
chr2	25430852	25431400	promoter-T	promoter-T	-735	NM_02929	75454	Phpt1	1700008C2
chr15	1.01E+08	1.01E+08	TTS (NM_0	TTS (NM_0	6342	NM_00120	1E+08	5430421N2	-
chr11	82938402	82938700	Intergenic	Intergenic	8204	NM_01140	20555	Slfn1	AV316259
chr15	38231877	38231975	Intergenic	Intergenic	-1460	NM_00128	21847	Klf10	AI115143|
chr14	19102127	19103950	promoter-T	promoter-T	462	NM_02558	66480	Rpl15	2510008H0
chr7	1.13E+08	1.13E+08	TTS (NM_0	TTS (NM_0	639	NM_02002	24075	Taf10	30kDa|AU0
chr19	46431702	46431800	intron (NM	intron (NM	381	NM_02918	75146	Tmem180	4930449A0
chr15	50733377	50733650	Intergenic	Intergenic	-11926	NM_03200	83925	Trps1	AI115454|A
chr11	49016877	49016950	promoter-T	promoter-T	-89	NM_00102	22720	Zfp62	-
chr12	77295627	77295725	Intergenic	RMER12B|	-17430	NR_104386	13983	Esr2	ER[b]|ERbe
chr3	1.58E+08	1.58E+08	promoter-T	promoter-T	-592	NM_00101	433667	Ankrd13c	AI505652|A
chr10	1.08E+08	1.08E+08	intron (NM	intron (NM	17330	NM_00930	20979	Syt1	AW124717
chr1	1.3E+08	1.3E+08	Intergenic	Intergenic	-4987	NM_00991	12767	Cxcr4	CD184|Cm
chr1	59970502	59970700	intron (NM	CpG	751	NM_00103	72750	Fam117b	2810425F2
chr2	28695977	28696300	promoter-T	promoter-T	212	NM_00103	227674	Ddx31	5830444G1
chr4	45653502	45653725	Intergenic	MER117|D	-42848	NM_00103	329839	Gm829	-
chr12	25167952	25168275	Intergenic	Intergenic	-15333	NM_02061	21340	Taf1b	4930408G0
chr8	1.29E+08	1.29E+08	Intergenic	Intergenic	-109152	NM_00116	270110	Irf2bp2	E130305N2
chr14	68397302	68397425	intron (NM	intron (NM	34077	NM_00814	14714	Gnrh1	Gnrh|Gnrh
chr6	52648102	52648350	Intergenic	Intergenic	-15497	NM_02581	52440	Tax1bp1	1200003J1
chr4	1.34E+08	1.34E+08	Intergenic	Intergenic	-3264	NM_02366	27981	Rsrp1	2700043I2
chr1	7078477	7078625	promoter-T	promoter-T	-450	NM_18302	319263	Pcmtd1	8430411F1
chr2	91232502	91232600	intron (NM	URR1B|DN	52248	NM_17512	228356	1110051M	AI586322
chr6	17697702	17698075	intron (NM	intron (NM	-1282	NM_00108	64213	St7	9430001H0
chr12	60162152	60162325	5' UTR (NM	5' UTR (NM	210	NM_03005	78232	Trappc6b	5830498C1
chr11	6526177	6526250	promoter-T	promoter-T	-143	NM_13401	21379	Tbrg4	2310042P2
chr4	1.29E+08	1.29E+08	Intergenic	Intergenic	-10877	NM_18278	230766	Fam167b	SEC

chr6	1.23E+08	1.23E+08	Intergenic	ID_B1|SINI	-6901	NM_00977	12267	C3ar1	AZ3B|C3AI
chr7	1.06E+08	1.06E+08	promoter-T	promoter-T	65	NM_02841	72981	Prkrir	2900052B1
chr12	55921702	55922000	Intergenic	Intergenic	-25202	NM_02699	72183	Snx6	2010006G2
chr2	35790677	35790825	intron (NM	L1MC4a|LI	44393	NM_02977	74410	Ttll11	4932702F0
chr2	1.58E+08	1.58E+08	promoter-T	promoter-T	-14	NM_17785	329547	Bpi	9230105K1
chr3	87847002	87847400	5' UTR (NM	5' UTR (NM	173	NM_02566	66614	Gpatch4	2610029K2
chr7	90780602	90780675	exon (NM_	exon (NM_	111	NM_02337	170460	Stard5	2310058G2
chr3	35653227	35653350	5' UTR (NM	5' UTR (NM	228	NM_02957	76295	Atp11b	1110019I1
chr7	75293277	75293525	intron (NM	intron (NM	115718	NM_03010	78444	Pgpep1l	C330024D1
chr1	1.29E+08	1.29E+08	promoter-T	promoter-T	71	NM_00108	210356	Nckap5	8430408F2
chr11	82891477	82891800	Intergenic	Intergenic	13024	NM_01140	20556	Slfn2	Shlf2
chr11	76693177	76693650	Intergenic	ID4|SINE|I	-24634	NM_02565	66601	Tmigd1	2010002A2
chr11	95246052	95246150	promoter-T	promoter-T	-135	NM_01675	110172	Slc35b1	Ugalt2
chr4	1.15E+08	1.15E+08	intron (NM	intron (NM	12594	NM_00100	81906	Cyp4x1	A230025G2
chr13	1.01E+08	1.01E+08	Intergenic	Intergenic	96950	NM_01373	27220	Cartpt	Cart
chrX	1.39E+08	1.39E+08	intron (NM	MTD|LTR|I	13090	NM_00103	50790	Acsl4	9430020A0
chr4	86365002	86365400	Intergenic	MIRb|SINE	-29258	NM_18408	329877	Dennd4c	1700065A0
chr1	1.66E+08	1.66E+08	intron (NM	intron (NM	16948	NM_00972	11931	Atp1b1	Atp4b|Atp
chr8	89123302	89123450	Intergenic	Intergenic	-24566	NM_02582	66887	Lonp2	1300002A0
chrX	45776652	45776775	intron (NM	intron (NM	39596	NM_01968	56501	Elf4	AV314029|
chr13	70776427	70776650	promoter-T	promoter-T	-26	NM_14483	218333	Ice1	C77245|mI
chr1	1.83E+08	1.83E+08	promoter-T	promoter-T	-123	NM_14479	208795	Tmem63a	BC014795|
chr4	1.09E+08	1.09E+08	promoter-T	promoter-T	-161	NM_02957	100087	Kti12	1110001A1
chr1	1.69E+08	1.69E+08	intron (NM	CpG	545	NM_03072	80914	Uck2	AA407809|
chr5	91125377	91125500	Intergenic	Intergenic	-55925	NM_02847	73246	Rassf6	1600016B1
chrX	54206427	54206525	Intergenic	Intergenic	-89775	NM_00976	12209	Brs3	BRS-3
chr2	1.1E+08	1.1E+08	exon (NM_	exon (NM_	-21198	NM_02661	68201	Ccdc34	2810027O1
chr4	19935777	19936000	intron (NM	CpG	313	NM_01576	50500	Ttpa	alpha-TTP
chr12	43141327	43141550	Intergenic	Intergenic	-928794	NM_01073	16981	Lrrn3	NLRR-3
chr8	73389477	73389625	Intergenic	CpG	7838	NM_02696	234388	Ccdc124	1810023B2
chr9	56920427	56920575	promoter-T	promoter-T	654	NM_00111	20466	Sin3a	AW553200
chr11	98590402	98590550	intron (NM	intron (NM	273	NM_01186	23989	Med24	911GSE|AU
chr9	1.22E+08	1.22E+08	intron (NM	CpG-15525	852	NM_01091	18087	Nktr	5330401F1
chr6	1.16E+08	1.16E+08	5' UTR (NM	5' UTR (NM	152	NM_02978	110157	Raf1	6430402F1
chr7	1.34E+08	1.34E+08	intron (NM	CpG	447	NM_01967	56420	Ppp4c	1110002D0
chr18	21126827	21126950	intron (NM	intron (NM	23762	NM_02630	67664	Rnf125	4930553F0
chr13	95071177	95071250	Intergenic	Intergenic	-15977	NM_02915	107767	Scamp1	4930505M
chr5	1.26E+08	1.26E+08	intron (NM	CpG	258	NM_00125	20602	Ncor2	N-CoR|SMI
chr4	1.41E+08	1.41E+08	intron (NM	CpG	521	NM_00101	68817	Ddi2	1110056G1
chr2	60645677	60646000	intron (NM	intron (NM	73657	NM_00114	56878	Rbms1	2600014B1
chr4	94073227	94073400	Intergenic	Intergenic	150174	NM_02636	67770	Caap1	5830433M
chr5	73306927	73307025	intron (NM	intron (NM	1375	NM_15356	75991	Slain2	5033405K1
chr1	1.54E+08	1.54E+08	intron (NM	intron (NM	25678	NM_01684	19731	Rgl1	Rgl|mKIAA
chr19	41459502	41459575	promoter-T	promoter-T	22	NM_03137	83490	Pik3ap1	1810044J0
chr11	72363602	72363825	promoter-T	promoter-T	35	NM_02993	77577	Spns3	9830002I1
chr9	1.06E+08	1.06E+08	exon (NM_	exon (NM_	488	NM_15345	235584	Dusp7	AU015694
chr3	51287652	51287800	promoter-T	promoter-T	-162	NM_01685	19338	Rab33b	-

chr19	33167052	33167400	Intergenic	Intergenic	299559	NM_00116	67795	Rnls	6530404N2
chr15	31268677	31268950	intron (NM	L1MB4|LIN	28701	NM_00116	67434	Ankrd33b	0610012A0
chr9	52055002	52055150	Intergenic	Intergenic	-78860	NM_00116	244871	Zc3h12c	A230108E0
chr15	34026202	34026375	intron (NM	PB1D9|SIN	13814	NM_02600	67154	Mtdh	2610103J2
chr2	59720252	59720425	intron (NM	CpG	325	NM_00115	72137	Wdsub1	1700048E1
chr4	1.18E+08	1.18E+08	intron (NM	intron (NM	17501	NM_01121	19268	Ptprf	AA591035|
chr2	1.29E+08	1.29E+08	intron (NM	intron (NM	10550	NM_17765	228592	F830045P1	Sirpb3
chr13	32084727	32085075	intron (NM	intron (NM	186386	NM_00859	17300	Foxc1	FREAC3|Fk
chr9	21920677	21920750	Intergenic	Intergenic	-2100	NM_17077	66126	Elof1	1110011K1
chr13	20164877	20165075	Intergenic	Intergenic	-17400	NM_08028	140580	Elmo1	6330578D2
chr4	4017002	4017075	Intergenic	Lx6|LINE|L	-12377	NM_00108	242286	Sdr16c6	4833413O1
chr7	1.25E+08	1.25E+08	promoter-T	promoter-T	450	NM_00103	233789	Smg1	2610207I0
chr11	72951352	72951600	exon (NM_	exon (NM_	320	NM_01035	14841	Gsg2	Haspin
chr19	12575877	12576125	5' UTR (NM	5' UTR (NM	485	NM_17244	207521	Dtx4	AI449438|
chr1	55144877	55144950	promoter-T	promoter-T	-79	NM_00830	15528	Hspe1	10kDa|Hsp
chr1	1.65E+08	1.65E+08	intron (NM	ETnERV3-ir	12259	NM_00803	14262	Fmo3	AW111792
chr3	89225877	89225950	exon (NM_	exon (NM_	370	NM_00111	20416	Shc1	Shc|ShcA|
chr11	34678752	34678950	Intergenic	Intergenic	-31708	NM_02741	70385	Spdl1	1700018I0
chr17	56855827	56855925	intron (NM	ID_B1|SINI	468	NM_17892	106639	Vmac	AI662250
chr7	77791777	77792025	Intergenic	Intergenic	-280269	NM_18326	11819	Nr2f2	2700033K0
chr2	1.68E+08	1.68E+08	promoter-T	promoter-T	-264	NM_00962	11538	Adnp	AA589558|
chr10	87875152	87875300	intron (NM	intron (NM	33069	NM_00100	432486	Gnptab	EG432486|
chr6	1.15E+08	1.15E+08	Intergenic	Intergenic	-33071	NM_19903	381802	Tsen2	AU067695
chr17	35325327	35325400	promoter-T	promoter-T	22	NM_01073	16988	Lst1	B144
chr19	5769077	5769175	intron (NM	MER2|DNA	2275	NM_02391	78891	Scyl1	2810011O1
chr19	29441402	29442075	promoter-T	promoter-T	-190	NM_02189	60533	Cd274	A530045L1
chr13	76620552	76620775	intron (NM	intron (NM	98254	NM_03017	78771	Mctp1	2810465F1
chr1	1.89E+08	1.89E+08	intron (NM	intron (NM	13661	NM_02604	67223	Rrp15	2810430M
chr3	86351177	86351250	exon (NM_	exon (NM_	992	NM_01183	23937	Mab21l2	-
chr11	83285977	83286200	promoter-T	promoter-T	-522	NM_02742	70439	Taf15	2610111C2
chr5	1.04E+08	1.04E+08	intron (NM	intron (NM	7884	NM_00108	17355	Aff1	9630032B0
chr8	88083252	88083375	Intergenic	RSINE1|SIN	-4143	NM_01979	56445	Dnaja2	1500017M
chr2	1.53E+08	1.53E+08	Intergenic	Intergenic	7556	NM_01022	14239	Foxs1	FREAC10|F
chr17	34088252	34088600	promoter-T	promoter-T	7	NM_01942	54218	B3galt4	Gal-T2|Gal
chr4	1.26E+08	1.26E+08	intron (NM	CpG	266	NM_14615	230753	Thrap3	9330151F0
chr12	76392677	76392875	Intergenic	RMER19B|	-16524	NM_02327	80837	Rhoj	1110005O1
chr18	77304827	77304900	intron (NM	intron (NM	444	NM_00116	17344	Pias2	6330408K1
chr11	4346602	4346700	Intergenic	Intergenic	-5566	NM_02945	75828	Hormad2	4930529M
chr10	98409802	98409875	intron (NM	intron (NM	32052	NM_02648	67972	Atp2b1	2810442I2
chr16	5233702	5233900	promoter-T	promoter-T	87	NM_14536	208211	Alg1	HMAT1|HN
chr1	16092977	16094975	intron (NM	intron (NM	538	NM_01129	19989	Rpl7	Rpl7a|Surf
chr2	68746102	68746475	intron (NM	RMER15|L	46674	NM_17285	241447	Cers6	4732462C0
chr7	3249577	3249750	promoter-T	promoter-T	78	NM_00103	378425	Nlrp12	Nalp12|PY
chr5	68219777	68220000	intron (NM	MLT1A|LTI	18782	NM_00128	11980	Atp8a1	AI481521|
chr2	1.68E+08	1.68E+08	intron (NM	intron (NM	452	NM_00129	18019	Nfatc2	AI607462|
chr6	1.25E+08	1.25E+08	Intergenic	CpG	-3271	NM_01353	14794	Spsb2	AI461677|
chr5	1.39E+08	1.39E+08	promoter-T	promoter-T	-87	NM_00931	21343	Taf6	80kDa|AW

chr13	37098652	37098825	intron (NM	L1MC4|LIN	42344	NM_02878	74145	F13a1	1200014I0
chr12	73591052	73591300	intron (NM	RLTR19|LT	-46168	NM_02632	67708	Pcnxl4	1810048J1
chr5	1.15E+08	1.15E+08	5' UTR (NM	5' UTR (NM	175	NM_02999	77832	Tchp	A930031F1
chr17	33821602	33822250	TTS (NM_1	TTS (NM_1	477	NM_00110	76936	Hnrnpm	2610023M
chr5	99766277	99766550	intron (NR_	intron (NR_	-84467	NM_14583	320292	Rasgef1b	4732452O0
chr16	25784377	25784500	intron (NM	intron (NM	-17564	NM_00112	22061	Trp63	AI462811|
chr4	3501702	3501925	promoter-T	promoter-T	102	NM_02809	72098	Tmem68	2010300G1
chr7	54050752	54051625	promoter-T	promoter-T	63	NM_00100	246694	Hps5	AI646796|A
chr18	4288027	4288100	Intergenic	L1MB2|LIN	64888	NM_00774	26410	Map3k8	Cot|Cot/Tp
chr13	28509052	28509200	Intergenic	Intergenic	274773	NM_02374	28078	Prl5a1	1600013P0
chr15	72920827	72921100	exon (NM_	exon (NM_	121	NM_05306	93696	Chrac1	2410152E0
chr12	9036152	9037200	promoter-T	promoter-T	-127	NM_02932	75516	Ttc32	1700013G2
chr10	79265252	79265375	intron (NM	MIR|SINE|	8996	NM_00116	18483	Palm	-
chr7	25102027	25102150	promoter-T	promoter-T	-403	NM_00119	22756	Zfp94	-
chr15	96890452	96890825	Intergenic	Intergenic	-4251	NM_02705	69354	Slc38a4	1110012E1
chrX	1.29E+08	1.29E+08	Intergenic	Lx9|LINE|L	1443594	NM_00110	279653	Pcdh19	B530002L0
chr9	79831502	79831625	Intergenic	Intergenic	-5874	NM_00108	70598	Filip1	5730485H2
chr17	35653327	35653775	promoter-T	promoter-T	218	NM_00116	106795	Tcf19	5730403J1
chr15	54403977	54404125	intron (NM	intron (NM	1130	NM_17892	105853	Mal2	AI461653
chr11	58120952	58121175	5' UTR (NM	5' UTR (NM	492	NM_00104	103836	Zfp692	AI746306|A
chr19	3562777	3562900	intron (NM	L1ME3B|LI	12911	NM_00116	52036	Ppp6r3	4930528G0
chr3	27555802	27555900	intron (NM	intron (NM	53510	NM_17318	72007	Fndc3b	1600019O0
chr8	80327327	80327475	Intergenic	Intergenic	-79050	NM_01033	13617	Ednra	ET-AR|ETa
chr9	20264127	20264225	intron (NM	CpG	428	NM_01175	22688	Zfp26	5033428C0
chrX	55530602	55530850	Intergenic	Intergenic	246921	NM_00957	22773	Zic3	Bn|Ka
chr3	1.19E+08	1.19E+08	intron (NM	CpG	668	NM_01955	56195	Ptbp2	Ptb2|brPTB
chr12	4483177	4483325	intron (NM	intron (NM	809	NM_01088	17977	Ncoa1	KAT13A|SR
chr9	20684402	20684475	promoter-T	promoter-T	-284	NM_14515	70726	Angptl6	6330404E1
chr10	1.25E+08	1.25E+08	exon (NM_	exon (NM_	401	NM_17715	320398	Lrig3	9030421L1
chr11	1.18E+08	1.18E+08	intron (NM	intron (NM	23597	NM_02144	58251	BC100451	Ddc8|FS39
chr8	1.1E+08	1.1E+08	promoter-T	promoter-T	-210	NM_02555	66427	Cyb5b	1810044O2
chr1	1.36E+08	1.36E+08	Intergenic	RSINE1|SIN	15714	NM_14551	98710	Rabif	AI842864|A
chr1	1.8E+08	1.8E+08	Intergenic	L1M5|LINE	-15719	NM_02707	69428	1700016C1	-
chr16	18628652	18628800	intron (NM	intron (NM	1305	NM_21361	18951	5-Sep	Cdcrel-1|C
chr1	1.37E+08	1.37E+08	intron (NM	intron (NM	163	NM_01159	21854	Timm17a	17kDa|mTi
chr19	21848127	21848250	Intergenic	Intergenic	-4642	NM_00103	83921	Tmem2	-
chr3	87946427	87946825	intron (NM	CpG	309	NM_13366	17261	Mef2d	C80750
chr9	67272077	67272175	intron (NM	intron (NM	135384	NM_00108	70549	Tln2	5730421P0
chr8	1.24E+08	1.24E+08	intron (NM	intron (NM	746	NM_00128	53325	Banp	AA408158|
chr17	28345727	28345850	intron (NM	intron (NM	1065	NM_02718	23853	Def6	2410003F0
chr3	64991877	64992075	promoter-T	promoter-T	-49	NM_00128	16497	Kcnab1	Akr8a8|Kvb
chr10	67264952	67265225	Intergenic	Intergenic	110322	NM_17867	216049	Zfp365	AI839779|A
chr6	39228377	39228700	Intergenic	Intergenic	-71766	NM_00103	338523	Kdm7a	A630082K2
chr2	72389302	72389650	Intergenic	MER46C|D	75200	NM_02586	66953	Cdca7	2310021G0
chr9	1.24E+08	1.24E+08	Intergenic	Intergenic	-3909	NM_00987	12581	Cdkn2d	INK4d|p19
chr1	93945027	93945200	intron (NM	intron (NM	99857	NM_20722	208727	Hdac4	4932408F1
chr18	55008152	55008300	Intergenic	Intergenic	141608	NM_17575	269023	Zfp608	4932417D1

chr13	93614552	93614975	promoter-T	promoter-T	21	NM_00116	382793	Mtx3	4930470O1
chr11	96912202	96912400	intron (NM	intron (NM	167	NM_00108	71240	Osbpl7	4933437E1
chr17	15567777	15567875	intron (NR_	intron (NR_	34616	NM_02420	67544	Fam120b	4932442K0
chr16	11322702	11322800	promoter-T	promoter-T	-246	NM_02896	74478	Snx29	4933437K1
chrX	92639802	92639875	intron (NM	CpG	341	NM_17517	72345	Amer1	2810002O0
chr13	29883952	29884075	intron (NM	ORR1D2|LT	63444	NM_14453	68916	Cdkal1	1190005B0
chr9	90134927	90135075	intron (NM	L1MB8|LIN	30606	NM_19433	67016	Tbc1d2b	1810061M
chr4	1.23E+08	1.23E+08	intron (NM	intron (NM	1073	NM_02587	66966	Trit1	2310075G1
chr4	1.49E+08	1.49E+08	intron (NM	intron (NM	1907	NM_00129	100198	H6pd	AI785303|
chr17	46909552	46909800	promoter-T	promoter-T	92	NM_02596	67101	2310039H0	-
chr19	57447777	57447850	exon (NM_	exon (NM_	12314	NM_14550	226252	Fam160b1	AI450540|
chr2	1.19E+08	1.19E+08	promoter-T	promoter-T	-970	NM_13913	69408	Dnajc17	1700025B1
chrX	1.33E+08	1.33E+08	intron (NR_	intron (NR_	-5001	NM_00116	56397	Morf4l2	2410017O1
chr10	41315402	41315700	Intergenic	Intergenic	-7992	NM_00117	75973	Ccdc162	5033413D2
chr2	5765752	5766375	promoter-T	promoter-T	-17	NM_01691	53893	Nudt5	-
chr6	98980077	98980275	intron (NM	intron (NM	-1884	NM_00119	108655	Foxp1	3110052D1
chr12	81955727	81955950	intron (NM	intron (NM	64319	NM_00124	217684	4933426M	mKIAA024
chr17	47660252	47660375	intron (NM	intron (NM	18313	NM_00108	12445	Ccnd3	9230106B0
chr2	1.46E+08	1.46E+08	5' UTR (NM	5' UTR (NM	255	NM_01688	53626	Insm1	IA-1
chr6	1.37E+08	1.37E+08	Intergenic	Intergenic	39257	NM_02389	78600	Pde6h	A930033D1
chr5	74121052	74121175	Intergenic	Intergenic	78494	NM_17838	73472	Spata18	1700067I0
chr7	1.09E+08	1.09E+08	promoter-T	promoter-T	167	NM_00124	207728	Pde2a	CGS-PDE|c
chr19	5572727	5572975	Intergenic	Intergenic	4777	NM_00103	381201	Ap5b1	Gm962
chr15	1.02E+08	1.02E+08	intron (NM	intron (NM	2955	NM_00104	19411	Rarg	Nr1b3|RAF
chr8	92204577	92205150	Intergenic	Intergenic	-636802	NM_02139	58198	Sall1	Msal-3
chr8	1.29E+08	1.29E+08	Intergenic	Intergenic	113470	NM_02421	67952	Tomm20	1810060K0
chr1	58480427	58480775	intron (NR_	CpG	331	NR_027853	12747	Clk1	STY
chr9	1.2E+08	1.2E+08	intron (NM	intron (NM	6077	NM_02873	74052	Ttc21a	4921538N1
chr13	41563727	41563800	intron (NM	intron (NM	18966	NM_00111	18003	Nedd9	Cas-L|CasL
chr1	1.84E+08	1.84E+08	intron (NM	intron (NM	7649	NM_17512	67948	Fbxo28	4833428J1
chr5	98393477	98393725	intron (NM	intron (NM	66380	NM_13373	71914	Antxr2	2310046B1
chr2	72204602	72204875	intron (NM	intron (NM	80980	NM_17808	65964	Zak	AV006891|
chr6	1.25E+08	1.25E+08	intron (NM	CpG	397	NM_00765	12527	Cd9	Tspan29
chr2	72038552	72038775	intron (NM	intron (NM	-85031	NM_02305	65964	Zak	AV006891|
chr11	18667602	18667725	Intergenic	Intergenic	251309	NM_00119	17268	Meis1	C530044H1
chr11	3830077	3830275	intron (NM	intron (NM	1788	NM_00113	21452	Tcn2	AW208754
chr5	1.06E+08	1.06E+08	intron (NM	MIRb|SINE	29897	NM_17870	231549	Lrrc8d	2810473G0
chr6	87995102	87995250	promoter-T	promoter-T	88	NM_00129	19349	Rab7	Rab7a
chr15	80623027	80623375	intron (NM	Lx8|LINE|L	-5839	NM_17712	213988	Tnrc6b	2700090M
chr8	82849277	82849375	Intergenic	Intergenic	-168618	NM_01036	14934	Gypa	AI853584|
chr3	1.36E+08	1.37E+08	intron (NM	AT_rich|Lo	166808	NM_00129	19055	Ppp3ca	2900074D1
chr3	27836077	27836450	Intergenic	Intergenic	-1339	NM_00116	18805	Pld1	AA536939|
chr4	1.09E+08	1.09E+08	promoter-T	promoter-T	-617	NM_15339	230603	Ttc39a	4922503N0
chr10	19843227	19843375	intron (NM	intron (NM	-25425	NM_00863	17761	Map7	E-MAP-115
chr2	13466152	13466300	promoter-T	promoter-T	65	NM_01006	13434	Trdmt1	Dnmt2|Rn
chr7	91927877	91928050	Intergenic	Intergenic	-74118	NM_14641	258410	Olfr291	MOR254-2
chr12	1.19E+08	1.19E+08	intron (NM	intron (NM	711	NM_17593	217944	Rapgef5	4932413M

chr5	1.5E+08	1.5E+08	Intergenic	Intergenic	-18821	NM_00966	11690	Alox5ap	Flap
chr13	17787327	17787425	promoter-T	promoter-T	129	NM_02547	66308	Mplkip	2810021BC
chr2	91806502	91806675	Intergenic	L2a|LINE|L	-2868	NM_13830	104418	Dgkz	E130307BC
chr2	1.5E+08	1.5E+08	promoter-T	promoter-T	-100	NM_08057	68738	Acss1	1110032O1
chr6	87799977	87800125	intron (NM	intron (NM	1049	NM_00110	12785	Cnbp	AA408710|
chr8	74116227	74116750	5' UTR (NM	5' UTR (NM	405	NM_00129	66171	Pgls	1110030KC
chr17	66449552	66449725	intron (NM	intron (NM	1261	NM_00127	72900	Ndufv2	2900010C2
chr9	1.07E+08	1.07E+08	TTS (NM_1	TTS (NM_1	252	NM_02590	15983	Ifrd2	1810034A2
chr9	11103552	11103650	Intergenic	ETnERV2-ir	-198826	NM_00117	244682	Cntn5	6720426O1
chr9	72471402	72471875	TTS (NM_0	TTS (NM_0	-38516	NM_01089	17999	Nedd4	AA959633|
chr1	39634177	39634600	promoter-T	promoter-T	-196	NM_00103	67702	Rnf149	1600023E1
chr17	71966077	71966275	intron (NM	CpG	621	NM_17563	72515	Wdr43	2610318GC
chr1	59727377	59727850	promoter-T	promoter-T	65	NM_00946	22218	Sumo1	GMP1|PIC1
chr13	19885677	19886125	intron (NM	intron (NM	30225	NM_18175	353346	Gpr141	PGR13
chr18	34936727	34937025	exon (NM_	exon (NM_	214	NM_00108	277250	Kdm3b	5830462I2
chr1	1.67E+08	1.67E+08	intron (NM	CpG	368	NM_02875	74106	Dcaf6	1200006M
chr10	79614502	79614600	intron (NM	intron (NM	3516	NM_02156	59090	Midn	3000003C1
chr7	1.17E+08	1.17E+08	promoter-T	promoter-T	-175	NM_02061	57373	Akip1	BCA3|C11c
chr15	1.02E+08	1.02E+08	intron (NM	CpG	275	NM_00117	18521	Pcbp2	AW412548
chr14	66861852	66861950	intron (NM	intron (NM	-29512	NM_00116	19229	Ptk2b	CADTK|CAI
chr7	71197427	71197625	Intergenic	MLT1H2|LT	-101195	NM_00103	17364	Trpm1	4732499L0
chr17	51318202	51318275	intron (NM	CpG	439	NM_02816	72238	Tbc1d5	1600014NC
chr15	85801152	85801250	intron (NM	URR1A|DN	63006	NM_00988	12614	Celsr1	Crsh|Scy|c
chr2	1.35E+08	1.35E+08	Intergenic	L1MB7|LIN	-29974	NM_01967	18795	Plcb1	3110043I2
chr10	1.16E+08	1.16E+08	intron (NM	intron (NM	3919	NM_00116	19279	Ptprr	Gmcp1|PT
chr15	76369977	76370050	promoter-T	promoter-T	640	NM_02964	52710	Slc52a2	2010003PC
chr11	51502202	51502650	promoter-T	promoter-T	-290	NM_02531	66050	0610009B2	-
chr8	1.27E+08	1.27E+08	intron (NM	intron (NM	24327	NM_00111	244666	Sprtn	Gm505
chr4	1.54E+08	1.54E+08	Intergenic	Intergenic	-12324	NM_02558	66469	Fam213b	2810405KC
chr14	75045002	75045125	intron (NM	intron (NM	4416	NM_17281	15558	Htr2a	E030013E0
chr16	18812452	18812550	promoter-T	promoter-T	114	NM_01167	22230	Ufd1l	Ufd1
chr5	1.25E+08	1.25E+08	TTS (NM_0	TTS (NM_0	284	NM_14450	65105	Arl6ip4	AA408210|
chr9	40965627	40965825	promoter-T	promoter-T	-149	NM_17686	72828	Ubash3b	2810457I0
chr16	17891227	17891300	intron (NM	CpG	558	NM_01004	13356	Dgcr2	9930034OC
chr6	49024077	49024150	intron (NM	CpG	319	NM_02935	75593	Malsu1	2410003K1
chr8	1.29E+08	1.29E+08	TTS (NM_0	TTS (NM_0	5210	NM_00116	270110	Irf2bp2	E130305N2
chr1	95298277	95298525	exon (NM_	exon (NM_	27928	NM_00127	404545	Ano7	Ngep|Ngep
chr18	20870327	20870450	intron (NM	intron (NM	34517	NM_01973	56386	B4galt6	AA536803|
chr6	1.13E+08	1.13E+08	intron (NM	intron (NM	655	NM_02838	72895	Setd5	2900045NC
chr1	1.46E+08	1.46E+08	promoter-T	promoter-T	-203	NM_00103	69367	Glrx2	1700010P2
chr6	87801177	87801375	promoter-T	promoter-T	-176	NM_00110	12785	Cnbp	AA408710|
chr1	64151302	64151450	intron (NM	Lx8|LINE|L	16587	NM_03356	93691	Klf7	9830124PC
chr1	1.82E+08	1.82E+08	intron (NM	intron (NM	413	NM_02334	67426	Adck3	4632432J1
chr7	1.3E+08	1.3E+08	intron (NM	CpG	564	NM_14492	233833	Tnrc6a	2010321I0
chrX	50265377	50265500	promoter-T	promoter-T	47	NM_00129	70998	Phf6	2700007B1
chr2	51928502	51928725	intron (NM	CpG	256	NM_17523	51869	Rif1	5730435J0
chr1	1.73E+08	1.73E+08	3' UTR (NM	3' UTR (NM	25934	NM_17264	16456	F11r	9130004G2

chr14	77719327	77719400	intron (NM	intron (NM	162793	NM_17281	239188	Enox1	B230207J0
chr12	86680152	86680500	promoter-T	promoter-T	-14	NM_14513	217718	Nek9	C130021H(
chr2	1.53E+08	1.53E+08	intron (NM	L1ME1|LIN	9251	NM_13384	99237	Tm9sf4	AA986553|
chr9	24955752	24956125	5' UTR (NM	5' UTR (NM	342	NM_02058	80517	Herpud2	5031400M
chr11	97888327	97888600	intron (NM	CpG	225	NM_00907	19921	Rpl19	-
chr11	1.02E+08	1.02E+08	intron (NM	CpG-2642	398	NM_00881	18612	Etv4	AW414408
chr6	1.4E+08	1.4E+08	intron (NM	intron (NM	20272	NM_01108	18705	Pik3c2g	C80387|PI:
chr9	54997477	54997625	exon (NM_	exon (NM_	375	NM_18060	109161	Ube2q2	3010021M
chr2	1.56E+08	1.56E+08	promoter-T	promoter-T	-527	NM_13324	170791	Rbm39	1500012C1
chr13	1.05E+08	1.05E+08	intron (NR_	intron (NR_	458	NM_02587	66975	Trappc13	2410002O2
chr11	75547927	75548000	intron (NM	intron (NM	1574	NM_00953	22627	Ywhae	AU019196
chr2	6243352	6243750	promoter-T	promoter-T	-252	NM_00108	98910	Usp6nl	AI316785|
chr10	1.26E+08	1.26E+08	promoter-T	promoter-T	350	NM_01079	17299	Mettl1	2810012D(
chr6	87445777	87445850	intron (NM	intron (NM	508	NR_10448:	232201	Arhgap25	A130039I2
chr8	87548802	87548975	intron (NM	intron (NM	289	NM_01965	56495	Asna1	1810048H2
chr11	1.06E+08	1.06E+08	Intergenic	MT2A|LTR	-1919	NM_19829	21763	Tex2	4930568E0
chr7	25791402	25792075	promoter-T	promoter-T	-782	NM_00129	232975	Atp1a3	Atpa-2
chr11	17922227	17922325	Intergenic	Intergenic	-68398	NM_02657	68145	Etaa1	5730466H2
chr2	1.67E+08	1.67E+08	intron (NM	intron (NM	12106	NM_14553	407243	Tmem189	AI840826|
chr3	1.16E+08	1.16E+08	promoter-T	promoter-T	97	NM_14490	229782	Slc35a3	2310050P1
chr5	35995977	35996050	intron (NM	intron (NM	26416	NM_00104	78558	Htra3	2210021K2
chr18	36434902	36434975	Intergenic	B4|SINE|B4	-5878	NM_00898	19290	Pura	CAGER-1|P
chr10	1.28E+08	1.28E+08	promoter-T	promoter-T	-619	NM_02887	74330	Dnajc14	5730551F1
chr2	71652152	71652400	intron (NM	intron (NM	27280	NM_00127	16403	Itga6	5033401O(
chr10	39977702	39977875	promoter-T	promoter-T	-317	NM_02611	67371	Gtf3c6	2410016F1
chr2	25228102	25228175	exon (NM_	exon (NM_	583	NM_00108	72080	Sapcd2	2010317E2
chr1	94121852	94122250	Intergenic	Intergenic	-77081	NM_20722	208727	Hdac4	4932408F1
chr4	1.47E+08	1.47E+08	intron (NM	CpG	160	NM_00964	11610	Agtrap	3300002E1
chr13	37711827	37711900	Intergenic	Intergenic	-206044	NM_02683	68750	Rreb1	1110037N(
chr1	10315977	10316100	intron (NM	intron (NM	-93287	NM_00110	211673	Arfgef1	ARFGEP1|E
chrX	12857352	12857500	promoter-T	promoter-T	-722	NM_01002	13205	Ddx3x	D1Pas1-rs2
chr14	21493127	21493375	promoter-T	promoter-T	-292	NM_17259	218811	Sec24c	2610204K(
chr12	16702952	16703200	exon (NM_	exon (NM_	42800	NM_00874	18217	Ntsr2	NT2R|NTRI
chr1	75165502	75165650	intron (NM	intron (NM	267	NM_02684	68818	Zfand2b	1110060O1
chr13	59686952	59687125	promoter-T	promoter-T	344	NM_03015	78689	Naa35	A330021G1
chr11	51428752	51428850	Intergenic	Intergenic	-4474	NM_02663	52530	Nhp2	2410130M
chr6	1.49E+08	1.49E+08	Intergenic	Intergenic	-48323	NM_00128	67246	2810474O1	6720435I2
chr1	93103927	93104375	TTS (NM_0	TTS (NM_0	27752	NM_00116	51801	Ramp1	9130218E1
chr1	1.93E+08	1.93E+08	Intergenic	Intergenic	-56915	NM_17226	226856	Lpgat1	AI649174|A
chr11	70240227	70240325	Intergenic	Intergenic	-5806	NM_00127	216869	Arrb2	AI326910|A
chr9	65975277	65975425	intron (NM	intron (NM	933	NM_02663	68250	Fam96a	5730536A(
chr19	5963677	5963800	intron (NM	intron (NM	468	NM_00116	18969	Pola2	AI573378
chr18	36675027	36675200	intron (NM	CpG	346	NM_01041	15200	Hbegf	AW047313
chr12	87376002	87376100	intron (NM	L1MB8|LIN	43940	NM_00936	21809	Tgfb3	Tgfb-3
chr1	1.22E+08	1.22E+08	promoter-T	promoter-T	349	NM_00117	73103	3110009E1	-
chr4	1.25E+08	1.25E+08	promoter-T	promoter-T	148	NM_15356	230734	Yrdc	AV303379|
chr4	1.55E+08	1.55E+08	promoter-T	promoter-T	145	NM_00103	433813	Pusl1	2810021I1

chr4	58925527	58925700	promoter-T	promoter-T	-16	NM_17238	230249	AI314180	AW558785
chr7	1.09E+08	1.09E+08	TTS (NM_0	TTS (NM_0	-1482	NM_01053	16068	Il18bp	IL-18BP|Igi
chr13	37337727	37337875	Intergenic	Intergenic	-99413	NM_01074	17084	Ly86	MD-1|MD1
chr19	45734727	45734850	promoter-T	promoter-T	-105	NM_01390	30838	Fbxw4	Dac|Fbw4|
chr11	1.18E+08	1.18E+08	Intergenic	Intergenic	-19118	NM_00116	69926	Dnah17	2810003K2
chr2	1.27E+08	1.27E+08	Intergenic	Intergenic	25115	NM_19863	329509	1810024B0	-
chr7	75037702	75038325	Intergenic	Intergenic	-59130	NM_01051	16001	Igf1r	A330103N2
chr9	43839452	43839525	Intergenic	MER74A|L	-11979	NM_00938	21838	Thy1	CD90|T25|
chr6	1.05E+08	1.05E+08	Intergenic	RLTR11B|L	479313	NM_01738	53870	Cntn6	NB-3
chr1	52186727	52186900	intron (NM	intron (NM	10347	NM_00120	20846	Stat1	2010005J0
chr17	27270352	27270725	intron (NM	intron (NM	298	NM_02606	67267	Uqcc2	2900010M
chr12	1.02E+08	1.02E+08	promoter-T	promoter-T	-258	NM_02668	68339	Ccdc88c	0610010D2
chr12	86611327	86611425	intron (NM	CpG	138	NM_17533	217716	Mlh3	AV125803|
chr8	77563852	77563950	intron (NM	intron (NM	6316	NM_01162	21968	Tom1	-
chr7	86805702	86805825	exon (NM_	exon (NM_	681	NM_02983	77011	Ticrr	5730590G1
chr9	65635802	65636025	intron (NM	intron (NM	39458	NM_17253	214812	Zfp609	9830165N2
chr15	99649177	99649275	intron (NM	intron (NM	1354	NM_02306	65970	Lima1	Eplin
chr1	45852277	45852925	intron (NM	CpG	255	NM_02859	73674	Wdr75	1300003A1
chr1	36328852	36328975	intron (NM	intron (NM	1357	NM_15340	214854	Neurl3	2010300P0
chr1	1.37E+08	1.37E+08	5' UTR (NM	5' UTR (NM	171	NM_00127	67196	Ube2t	2700084L2
chr1	65277102	65277300	intron (NM	intron (NM	43942	NM_01108	18711	Pikfyve	5230400C1
chr10	1.21E+08	1.21E+08	Intergenic	Intergenic	-12595	NM_13895	192678	Rassf3	AW212023
chr8	34784277	34784425	intron (NM	intron (NM	20641	NM_01034	14782	Gsr	AI325518|
chr5	1.07E+08	1.07E+08	Intergenic	Intergenic	27133	NM_00127	12545	Cdc7	AI597260|
chr8	41690302	41690550	intron (NM	intron (NM	29720	NM_00104	54384	Mtmr7	-
chr1	1.74E+08	1.74E+08	intron (NM	intron (NM	-12635	NM_00115	19298	Pex19	Pxf
chr1	1.8E+08	1.8E+08	intron (NM	CpG	1089	NM_01680	51810	Hnrnpu	AA408410|
chr1	1.83E+08	1.83E+08	promoter-T	promoter-T	-142	NM_14551	226757	Wdr26	1600024A0
chr14	58618677	58618775	promoter-T	promoter-T	-627	NM_02864	68514	Micu2	1110008L2
chr10	1.21E+08	1.21E+08	3' UTR (NM	3' UTR (NM	47044	NM_02905	74694	Tbc1d30	4930505D0
chr6	67482577	67482975	Intergenic	Intergenic	3040	NM_02004	56753	Tacstd2	C80403|EG
chr1	1.94E+08	1.94E+08	promoter-T	promoter-T	-179	NM_00129	214742	Rcor3	4921514E2
chr4	1.07E+08	1.07E+08	Intergenic	Intergenic	-36121	NM_01987	56296	Dmrtb1	Dmrt6|Prp
chr12	1.02E+08	1.02E+08	Intergenic	B1_Mur2|S	-4475	NM_15358	73086	Rps6ka5	3110005L1
chr1	16095927	16096000	promoter-T	promoter-T	0	NM_13383	98711	Rdh10	3110069K0
chr1	1.73E+08	1.73E+08	promoter-T	promoter-T	-550	NM_00112	21945	Dedd	CASP8IP1|
chr10	12728302	12728450	5' UTR (NM	5' UTR (NM	120	NM_17510	66125	Sf3b5	10kDa|111
chr8	1.17E+08	1.17E+08	intron (NM	intron (NM	392924	NM_01957	80707	Wwox	5330426P0
chr2	24041652	24041850	promoter-T	promoter-T	-245	NM_15351	215257	Il1f9	Il36g
chr1	72242752	72242975	intron (NM	intron (NM	16018	NM_00100	381269	Mreg	Gm974|W
chr1	52857102	52857550	intron (NM	intron (NM	17206	NM_00838	16329	Inpp1	2300002C0
chr3	60211302	60211800	Intergenic	Intergenic	-65201	NM_00125	56758	Mbnl1	Mbnl|mKIA
chr10	80783802	80783900	promoter-T	promoter-T	3	NM_02738	70312	Cactin	2510012J0
chr2	1.8E+08	1.8E+08	intron (NM	intron (NM	12732	NM_18316	228993	Slc17a9	1700019H0
chr5	1.07E+08	1.07E+08	5' UTR (NM	5' UTR (NM	111	NM_02685	52397	Zfp644	1110068L0
chr5	28596227	28596450	Intergenic	ORR1E|LTR	-31925	NM_17565	269637	Cnpy1	1500012D2
chrX	1.37E+08	1.37E+08	intron (NM	CpG	1073	NM_00107	14605	Tsc22d3	DIP|Dsip1|

chr1	1.08E+08	1.08E+08	intron (NM	intron (NM	29030	NM_00112	227449	Zcchc2	9930114B2
chr7	26687752	26687875	intron (NM	intron (NM	5136	NM_00999	13088	Cyp2b10	Cyp2b|Cyp
chr14	31952777	31952950	TTS (NM_0	TTS (NM_0	4824	NM_02728	70021	Nt5dc2	2510015F0
chr13	81922327	81922525	intron (NM	CpG	135	NM_00768	12626	Cetn3	MmCEN3
chr7	52281227	52281325	Intergenic	Intergenic	7899	NM_00910	20130	Rras	AI573426
chr9	48288102	48288250	intron (NM	intron (NM	540	NM_02423	104444	Rexo2	1810038D1
chr5	1.01E+08	1.01E+08	intron (NM	intron (NM	14528	NM_17271	231510	Agpat9	1-AGPAT|1
chr5	66116202	66116275	Intergenic	ERVL-B4-in	-27143	NM_00108	71521	Pds5a	9030416H1
chr17	53829452	53829575	promoter-T	promoter-T	-873	NM_02823	72415	Sgol1	3300001M
chr10	80396227	80396500	Intergenic	Intergenic	3527	NM_00865	17873	Gadd45b	AI323528|
chr6	40421227	40421400	promoter-T	promoter-T	-101	NM_21246	381760	Ssbp1	2810480P1
chr7	46911452	46911625	Intergenic	Intergenic	138402	NM_00100	70227	Zfp619	3000002G1
chr19	10970002	10970200	intron (NM	CpG	380	NM_00125	28000	Prpf19	AA617263|
chr1	99986927	99987150	intron (NM	intron (NM	5171	NM_01362	18484	Pam	PHM
chr8	10006577	10006725	promoter-T	promoter-T	18	NM_03362	24099	Tnfsf13b	BAFF|BLyS
chr8	1.24E+08	1.24E+08	Intergenic	Intergenic	-34833	NM_02060	57340	Jph3	JP-3|Jp3
chr5	1.41E+08	1.41E+08	intron (NM	MIRb|SINE	263	NM_02152	59031	Chst12	AI595374|
chr19	4328927	4329000	exon (NM_	exon (NM_	-22741	NM_00129	110355	Adrbk1	Adrbk-1|Ba
chr14	70474952	70475100	Intergenic	CpG	-2226	NM_01878	13655	Egr3	Pilot
chr3	60101577	60101800	Intergenic	RLTR13D3|	-175064	NM_00125	56758	Mbnl1	Mbnl|mKIA
chr9	86962952	86963275	intron (NM	intron (NM	46249	NM_02419	266690	Cyb5r4	2810034J1
chr4	63747052	63747275	Intergenic	Intergenic	-39114	NM_01160	21923	Tnc	AI528729|
chr9	1.09E+08	1.09E+08	exon (NM_	exon (NM_	427	NM_00892	19087	Prkar2a	1110061A2
chr11	23565352	23565725	promoter-T	promoter-T	-136	NM_02895	74467	Pus10	2810013G1
chrX	1.52E+08	1.52E+08	Intergenic	Intergenic	-29596	NM_00108	73347	1700042B1	-
chr17	36973452	36973750	promoter-T	promoter-T	-490	NM_00102	22670	Trim26	AI462198|
chr1	84299402	84299775	Intergenic	Intergenic	-18368	NM_00100	98496	Pid1	5033414K0
chr13	55631152	55631300	TTS (NM_1	TTS (NM_1	-1327	NM_01373	27261	Dok3	AI450713|
chr9	90010077	90010150	promoter-T	promoter-T	-455	NM_02443	21761	Morf4l1	MORFRG15
chr8	27462052	27462450	Intergenic	Intergenic	193610	NM_19904	73754	Thap1	4833431A0
chr2	72877427	72877675	Intergenic	Intergenic	-59048	NM_00101	20687	Sp3	D130027J0
chr7	1.43E+08	1.43E+08	exon (NM_	exon (NM_	11974	NM_00108	17345	Mki67	D630048A1
chr5	1.11E+08	1.11E+08	promoter-T	promoter-T	493	NM_02715	69663	Ddx51	2310061O0
chr7	1.24E+08	1.24E+08	Intergenic	Intergenic	-16495	NM_00113	53322	Nucb2	AI607786|
chr3	95695652	95695875	promoter-T	promoter-T	90	NM_15351	229600	BC028528	L259
chr6	8658502	8658700	intron (NM	intron (NM	49857	NM_00125	15893	Ica1	69kDa|ICA
chr6	1.13E+08	1.13E+08	intron (NM	CpG	350	NM_00124	67767	Jagn1	5830427H1
chr1	1.09E+08	1.09E+08	promoter-T	promoter-T	-21	NM_17741	12043	Bcl2	AW986256
chr2	76448377	76448650	Intergenic	L1MB2|LIN	37538	NM_01187	23992	Prkra	AV120107|
chr12	70732077	70732250	intron (NM	intron (NM	-48148	NM_00103	207965	Vcpkmt	Gm71|Met
chr4	1.17E+08	1.17E+08	intron (NM	ID_B1|SINE	57819	NM_02573	66743	Rnf220	4732477A1
chr17	56698852	56699100	Intergenic	Intergenic	25030	NM_00102	224902	Safb2	AA389433|
chr10	59806877	59807150	intron (NR_	(CA)n|Simp	-2586	NM_00115	74048	4632428N0	Dies1|PD-1
chr8	13027602	13027850	intron (NM	intron (NM	1692	NM_01017	14068	F7	AI132620|
chr12	76394052	76394300	Intergenic	Intergenic	-15124	NM_02327	80837	Rhoj	1110005O1
chr4	1.5E+08	1.5E+08	promoter-T	promoter-T	86	NM_01106	18628	Per3	2810049O0
chr3	1.55E+08	1.55E+08	promoter-T	promoter-T	229	NM_02933	75540	Fpgt	1700016E0

chr19	36422477	36422800	Intergenic	Intergenic	-30919	NM_02950	76073	Pcgf5	0610009F0
chr13	16285002	16285100	Intergenic	Intergenic	178743	NM_00838	16323	Inhba	-
chr11	58793277	58793500	intron (NM	intron (NM	1793	NM_05316	94091	Trim11	-
chr11	1.13E+08	1.13E+08	Intergenic	Intergenic	-3872	NM_02721	69806	Slc39a11	1810074D2
chr15	58046302	58046675	promoter-T	promoter-T	-41	NM_02634	67731	Fbxo32	4833442G1
chr16	90284152	90284300	intron (NM	CpG	444	NM_17892	224432	Scaf4	AA517739|
chr7	31597002	31597200	Intergenic	Intergenic	8895	NM_00116	233079	Ffar2	GPCR43|G
chr9	71388777	71388850	intron (NM	RMER30|D	51354	NM_00103	102371	Myzap	AA407270|
chr2	1.56E+08	1.56E+08	5' UTR (NM	5' UTR (NM	140	NM_00129	170791	Rbm39	1500012C1
chr2	1.58E+08	1.58E+08	exon (NM_	exon (NM_	272	NM_00848	16803	Lbp	Bpifd2|Ly8
chr1	1.73E+08	1.73E+08	Intergenic	L2a|LINE|L	14381	NM_14455	246256	Fcgr4	4833442P2
chr3	1.22E+08	1.22E+08	intron (NM	MIR|SINE|	13378	NM_01386	29815	Bcar3	AI131758|
chr4	91038052	91038150	intron (NM	CpG	645	NM_01048	15569	Elavl2	Hub|mel-N
chr10	84379952	84380175	promoter-T	promoter-T	-295	NM_00101	237422	Ric8b	BC051080|
chr2	1.19E+08	1.19E+08	promoter-T	promoter-T	-348	NM_02657	68142	Ino80	2310079N1
chr2	26405377	26405800	Intergenic	Intergenic	-30988	NM_17844	353156	Egfl7	VE-statin|Z
chr4	1.29E+08	1.29E+08	promoter-T	promoter-T	-117	NM_01689	11637	Ak2	Ak-2|D4Ert
chr5	65252302	65252425	Intergenic	Intergenic	57601	NM_00845	16599	Klf3	9930027G0
chr6	75830177	75830400	Intergenic	L1Md_F2|L	-1362423	NM_02888	74342	Lrrtm1	4632401D0
chr12	35732702	35732775	intron (NM	intron (NM	877	NM_00101	217463	Snx13	Rgs-px1|m
chr7	65913552	65913775	promoter-T	promoter-T	91	NM_00972	11982	Atp10a	Atp10c|pfa
chr18	43226202	43226500	Intergenic	Intergenic	-7226	NM_02839	72930	Ppp2r2b	2900026H0
chr12	74856727	74857050	intron (NM	intron (NM	-151966	NM_01043	15251	Hif1a	AA959795|
chr11	78932577	78932900	intron (NM	intron (NM	27118	NM_01357	16706	Ksr1	AW492498
chr3	35630927	35631075	Intergenic	RCHARR1|	-22059	NM_02957	76295	Atp11b	1110019I1
chr3	36512302	36512400	promoter-T	promoter-T	-40	NM_02781	71492	Bbs7	8430406N1
chr6	4562552	4562675	intron (NM	L1M5|LINE	11547	NM_14539	213819	Casd1	Cas1|Cast1
chr4	1.33E+08	1.33E+08	promoter-T	promoter-T	-992	NM_00128	20111	Rps6ka1	Mapkapk-1
chr8	1.13E+08	1.13E+08	promoter-T	promoter-T	627	NM_13397	102339	Cog4	AW554810
chr12	16901127	16901250	promoter-T	promoter-T	-596	NM_00907	19878	Rock2	B230113H1
chr1	1.73E+08	1.73E+08	promoter-T	promoter-T	-558	NM_15306	226646	Ndufs2	AL033311
chr10	1.28E+08	1.28E+08	promoter-T	promoter-T	-701	NM_05407	116848	Baz2a	AA415431|
chrX	68474077	68474300	intron (NM	MER5A|DN	5931	NM_01992	17772	Mtm1	AF073996|
chr19	3709202	3709375	intron (NM	intron (NM	955	NM_02807	72056	1810055G0	AI256204
chr15	66810827	66811075	Intergenic	Intergenic	-9748	NM_00868	17988	Ndrg1	CAP43|CM
chr11	47113327	47113600	intron (NM	MTE2b|LTR	79341	NM_01189	24052	Sgcd	35kDa|delt
chr1	75209852	75210000	intron (NM	intron (NM	2522	NM_01149	20872	Stk16	EDPK|Krct|
chr2	84222202	84222400	intron (NM	intron (NM	43122	NM_01878	54598	Calcrl	AV071593|
chr2	1.81E+08	1.81E+08	promoter-T	promoter-T	-11	NM_00129	56470	Rgs19	2610042F0
chr1	80336177	80336375	intron (NM	intron (NM	729	NM_01671	26554	Cul3	AI467304|
chr10	1.17E+08	1.17E+08	promoter-T	promoter-T	-990	NM_13401	103468	Nup107	AW541137
chr8	1.14E+08	1.14E+08	intron (NM	CpG	210	NM_00791	13680	Ddx19a	DBP5|Ddx1
chr4	59582527	59582825	Intergenic	L1M4|LINE	-11759	NM_00130	72479	Hsdl2	2610207I1
chr12	1.05E+08	1.05E+08	5' UTR (NM	5' UTR (NM	114	NM_00115	27225	Ddx24	1700055J0
chr7	1.38E+08	1.38E+08	Intergenic	URR1B|DN	-26211	NM_13394	101476	Plekha1	AA960558|
chr15	75644802	75645075	intron (NM	intron (NM	27400	NM_17212	223642	Zc3h3	BC049953|
chr2	1.7E+08	1.7E+08	Intergenic	Intergenic	-15713	NM_00115	228913	Zfp217	4933431C0

chr3	1.04E+08	1.04E+08	Intergenic	Intergenic	69791	NM_00102	269473	Lrig2	4632419I1(
chr14	62078127	62078275	Intergenic	Intergenic	-19417	NM_00846	16648	Kpna3	IPOA4
chr7	29527427	29527525	promoter-T	promoter-T	489	NM_02363	71984	Sars2	2410015F0
chr11	77305902	77306000	promoter-T	promoter-T	-963	NM_00100	216963	Git1	Cat-1|p95(
chr5	53134827	53135025	intron (NM	intron (NM	2113	NM_02595	67073	Pi4k2b	2610042N(
chr8	87189427	87189525	Intergenic	Intergenic	-2725	NM_01049	15936	Ier2	AI317238|(
chr15	76173652	76173725	promoter-T	promoter-T	-265	NM_02556	66445	Cyc1	2610002H1
chr3	30894727	30894875	5' UTR (NM	5' UTR (NM	108	NM_00885	18759	Prkci	2310021H1
chr5	34856252	34856575	exon (NM_	exon (NM_	215	NM_13906	231130	Tnip2	1810020H1
chr1	22917777	22918000	Intergenic	Intergenic	-105325	NM_00101	116837	Rims1	C030033M
chr5	1.08E+08	1.08E+08	promoter-T	promoter-T	-156	NM_00116	170823	Glmn	9330160J1
chr15	38583102	38583275	Intergenic	Intergenic	-8471	NM_02549	66335	Atp6v1c1	1700025B1
chr11	97310052	97310175	Intergenic	Intergenic	-1361	NM_02149	58996	Arhgap23	A330041B1
chr9	1.07E+08	1.07E+08	promoter-T	promoter-T	-481	NM_00101	321006	Vprbp	AI447437|I
chr2	1.21E+08	1.21E+08	intron (NM	intron (NM	105679	NM_00129	241627	Wdr76	5830411K1
chr5	1.09E+08	1.09E+08	promoter-T	promoter-T	-381	NM_00108	433931	Pigg	Gpi7
chr3	1.38E+08	1.38E+08	intron (NR_	intron (NR_	10941	NM_01957	56224	Tspan5	2810455A(
chr11	1.19E+08	1.19E+08	Intergenic	CpG	-17386	NM_01392	30951	Cbx8	Pc3
chr12	73637877	73637950	intron (NM	intron (NM	569	NM_02632	67708	Pcnxl4	1810048J1
chr10	83996352	83996425	exon (NM_	exon (NM_	245	NM_17545	216197	Ckap4	5630400A(
chr9	96799552	96799675	Intergenic	URR1B|DN	-9720	NM_00128	235534	Pxylp1	9430094M
chr2	32816752	32818600	promoter-T	promoter-T	444	NM_00907	269261	Rpl12	E430018F0
chr7	56496802	56496950	intron (NM	intron (NM	-4683	NM_17527	78286	Nav2	5330421F0
chr11	48671602	48671800	Intergenic	Intergenic	13147	NM_00832	15944	Irgm1	Ifggd3|Ifi1
chr4	1.01E+08	1.01E+08	promoter-T	promoter-T	-31	NM_00117	11639	Ak4	AK 4|Ak-3|
chr10	82902002	82902100	Intergenic	RLTR10-int	79085	NM_02657	28109	D10Wsu10	AI747614|(
chr1	1.09E+08	1.09E+08	Intergenic	Intergenic	-14487	NM_00117	18788	Serpinb2	PAI-2|Plan
chr13	51519852	51519925	Intergenic	Intergenic	15901	NM_01010	13610	S1pr3	AI132464|I
chr12	34809477	34809600	intron (NM	intron (NM	167002	NM_01165	22160	Twist1	AA960487|
chr14	26610402	26610675	Intergenic	L1MC3|LIN	-22196	NM_00110	71918	Zcchc24	2310047A(
chr2	1.7E+08	1.7E+08	Intergenic	Intergenic	-77938	NM_00115	228913	Zfp217	4933431C(
chr17	44743402	44743575	non-coding	non-coding	129637	NR_073425	12393	Runx2	AML3|Cbf|
chr1	82761627	82761725	intron (NM	intron (NM	3355	NM_02545	66261	Tm4sf20	1810018L0
chr14	79841277	79842000	Intergenic	Intergenic	-10004	NM_00103	432879	Zbtbd6	EG432879|
chr11	97012152	97012225	Intergenic	CpG	-1162	NM_19810	73174	Tbkbp1	3110043L1
chr1	1.72E+08	1.72E+08	intron (NM	intron (NM	53712	NM_02256	18214	Ddr2	AW495251
chr3	9886727	9887050	Intergenic	MER20|DN	-53209	NM_00119	94212	Pag1	Cbp|F7300
chr1	1.84E+08	1.84E+08	intron (NM	intron (NM	2594	NM_13381	98386	Lbr	AI505894|i
chr15	10436027	10436225	intron (NM	intron (NM	20333	NM_00128	19355	Rad1	mRAD1
chr14	45621952	45622150	3' UTR (NM	3' UTR (NM	14265	NM_00896	19217	Ptger2	EP2|Ptgere
chr1	1.8E+08	1.8E+08	promoter-T	promoter-T	-118	NM_00116	269152	Kif26b	4832420M
chr19	10600002	10600250	promoter-T	promoter-T	392	NM_17230	269061	Cpsf7	5730453I1(
chr1	80633852	80633925	intron (NM	intron (NM	28039	NM_00128	210293	Dock10	9330153B1
chr10	95096952	95097150	Intergenic	MLT1B|LTF	-70250	NM_02772	71207	Nudt4	4933436C1
chr4	62390252	62390400	Intergenic	Intergenic	109620	NM_13425	50780	Rgs3	4930506N(
chr6	4550752	4551175	promoter-T	promoter-T	-103	NM_14539	213819	Casd1	Cas1|Cast1
chr16	13780827	13781100	intron (NM	CpG	171	NM_00103	106298	Rrn3	AL023001|

chr11	61282877	61283125	Intergenic	Intergenic	-15507	NM_00129	22671	Rnf112	ZNF179|Zf
chr10	29274177	29274350	Intergenic	Intergenic	-18590	NM_02835	72780	Rspo3	2810459H(
chr1	95375552	95375775	promoter-T	promoter-T	25	NM_00115	18000	2-Sep	AW208991
chr14	98486752	98486925	intron (NM	intron (NM	82146	NM_00103	13134	Dach1	Dac|Dach
chr1	79861677	79861950	Intergenic	Intergenic	-6573	NM_00925	20720	Serpine2	B230326M
chr5	24191752	24192200	intron (NM	G-rich|Low	343	NM_01673	53312	Nub1	4931404D2
chr5	1.4E+08	1.4E+08	promoter-T	promoter-T	31	NM_02674	68510	Ints1	1110015K0
chr7	5012352	5012600	TTS (NM_1	TTS (NM_1	-1269	NM_13367	22185	U2af2	65kDa
chr7	1.05E+08	1.05E+08	promoter-T	promoter-T	-75	NM_02422	68197	Ndufc2	1810004I0
chr8	1.26E+08	1.26E+08	promoter-T	promoter-T	912	NM_02820	72325	Vps9d1	1300018I1
chr3	40491502	40491825	intron (NM	intron (NM	-21130	NM_17838	73333	Slc25a31	1700034J0
chr18	39720402	39720725	Intergenic	Intergenic	-73664	NM_00817	14815	Nr3c1	GR|Grl-1|(
chr8	1.29E+08	1.29E+08	Intergenic	Intergenic	95720	NM_02421	67952	Tomm20	1810060K0
chr17	31432827	31433050	promoter-T	promoter-T	-231	NM_00124	224674	Slc37a1	G3PP
chr18	21159752	21159950	promoter-T	promoter-T	9	NM_20762	56515	Rnf138	2410015A1
chr11	1.18E+08	1.18E+08	Intergenic	Cheshire|D	33454	NM_00770	12702	Socs3	Cis3|Cish3
chr3	1.44E+08	1.44E+08	Intergenic	Intergenic	64733	NM_00116	16911	Lmo4	A730077C1
chr5	1.39E+08	1.39E+08	promoter-T	promoter-T	-99	NM_01696	50878	Stag3	SA-2
chr5	92556827	92557000	Intergenic	Intergenic	-10051	NM_01949	56041	Uso1	115kDa|TA
chr4	1.55E+08	1.55E+08	promoter-T	promoter-T	123	NM_00766	12537	Cdk11b	AA989746|
chr12	78063377	78063600	promoter-T	promoter-T	-253	NM_00114	17187	Max	AA960152|
chr6	1.47E+08	1.47E+08	intron (NM	CpG	171	NM_14557	232536	Mrps35	MDSO23|N
chr17	29797502	29798575	intron (NM	intron (NM	492	NM_02879	74157	Cmtr1	1300018I0
chr3	97432002	97432275	promoter-T	promoter-T	-589	NM_00116	14263	Fmo5	5033418D1
chr19	25063677	25063850	Intergenic	LTR89|LTR	-10256	NM_02878	76088	Dock8	1200017A2
chr8	1.2E+08	1.2E+08	Intergenic	Intergenic	-13048	NM_02872	74032	Sdr42e1	4632417N0
chr1	20880902	20881125	promoter-T	promoter-T	310	NM_02882	74229	Paqr8	1700019B1
chr17	47432102	47432200	intron (NM	intron (NM	64972	NM_17262	224829	Trerf1	9430096I1
chr1	1.73E+08	1.73E+08	Intergenic	L1MC|LINE	-32671	NM_01948	56009	Alyref2	C130042O1
chr19	12514602	12514875	Intergenic	Intergenic	12343	NM_01954	56093	Pfpl	Epcs5|Epcs
chr3	88100502	88100725	promoter-T	promoter-T	147	NM_02980	76927	Tsacc	1700021C1
chr9	32309952	32310175	intron (NM	intron (NM	38974	NM_00802	14247	Fli1	EWSR2|Fli-
chr18	46905227	46905625	intron (NM	intron (NM	3855	NM_00968	11777	Ap3s1	[s]3A
chr17	6270527	6270850	intron (NM	CpG	218	NM_00103	77106	Tmem181a	5930418K1
chr11	78114402	78114500	promoter-T	promoter-T	-642	NM_01740	54141	Spag5	AI874642|
chr11	20012502	20012575	intron (NM	CpG	416	NM_14624	66713	Actr2	4921510D2
chr4	1.39E+08	1.39E+08	Intergenic	Intergenic	-1263	NM_00979	12345	Capzb	1700120C0
chr1	43557077	43557150	intron (NM	intron (NM	54517	NM_01087	17974	Nck2	4833426I1
chr8	80069477	80069800	Intergenic	BGLII_B|LT	-3658	NM_00108	102182	Prmt10	AI931714|
chr5	1.5E+08	1.5E+08	intron (NM	intron (NM	5735	NM_15357	231912	Katnal1	-
chr10	5075252	5075450	Intergenic	Intergenic	-76483	NM_00107	64009	Syne1	8B|A33004
chr12	73765777	73766050	promoter-T	promoter-T	-98	NM_02552	66375	Dhrs7	2310016E2
chr5	1.26E+08	1.26E+08	Intergenic	Intergenic	-3476	NM_01963	22190	Ubc	2700054O0
chr4	1.25E+08	1.25E+08	promoter-T	promoter-T	-290	NM_02731	70088	Meaf6	2310005N0
chr14	62637727	62637950	Intergenic	Intergenic	273978	NM_17341	239133	Dleu7	BC038059
chr12	98395102	98395175	Intergenic	Intergenic	1102409	NM_00807	14420	Galc	2310068B0
chr4	1.41E+08	1.41E+08	intron (NM	intron (NM	10083	NM_00101	68817	Ddi2	1110056G1

chr5	1.18E+08	1.18E+08	intron (NM	intron (NM	512	NM_02153	59043	Wsb2	AA673511|
chr3	95497977	95498200	Intergenic	Intergenic	-6307	NM_14489	229595	Adamtsl4	Tsrc1
chr17	23813127	23813350	TTS (NM_1	TTS (NM_1	1178	NM_00116	27279	Tnfrsf12a	AI255180|
chr6	47903627	47903750	non-coding	non-coding	299	NM_17889	101197	Zfp956	AI894139
chr6	1.27E+08	1.27E+08	promoter-T	promoter-T	-453	NM_18140	101187	Parp11	5330431N2
chr14	55633702	55633875	promoter-T	promoter-T	-503	NM_02689	68966	Ngdn	1500001L1
chr1	13362752	13362850	intron (NM	intron (NM	1363	NM_00107	17978	Ncoa2	9530095N1
chr5	1.08E+08	1.08E+08	intron (NM	intron (NM	25570	NM_02606	67266	Fam69a	2900024C2
chr15	99163277	99163450	intron (NM	intron (NM	37523	NM_01878	54614	Prpf40b	2610317D2
chr1	1.59E+08	1.59E+08	intron (NM	intron (NM	544	NM_02314	30935	Tor3a	Adir
chr5	89196077	89196225	intron (NM	intron (NM	2113	NM_00783	13178	Dck	-
chr11	79997477	79997550	exon (NM_	exon (NM_	139	NM_02801	71956	Rnf135	0610037N0
chr5	1.44E+08	1.44E+08	promoter-T	promoter-T	-73	NM_14491	231871	Daglb	E330036I1
chr4	40089652	40089875	promoter-T	promoter-T	-535	NM_00738	11428	Aco1	Aco-1|Ireb
chr18	15765602	15765675	intron (NM	Lx8|LINE|L	110917	NM_19905	71367	Chst9	5430438D0
chr3	1.46E+08	1.46E+08	Intergenic	Intergenic	-47270	NM_02914	75015	4930503B2	1700013A0
chr6	90271202	90271350	intron (NM	MTD|LTR|I	3903	NM_02792	71797	Chst13	1110067M
chr13	76759877	76760100	intron (NM	intron (NM	237579	NM_03017	78771	Mctp1	2810465F1
chr1	1.35E+08	1.35E+08	Intergenic	Intergenic	80306	NM_00922	20643	Snrpe	AL022645|
chr17	57414802	57414925	Intergenic	CpG	-3660	NM_00116	22324	Vav1	Vav|vav-T
chr15	3929127	3929300	intron (NM	CpG	360	NM_13409	106052	Fbxo4	1700096C1
chr1	1.34E+08	1.34E+08	intron (NM	B3|SINE|B	13451	NM_17251	213006	Mfsd4	A230072B0
chr1	58784002	58784125	intron (NM	intron (NM	-3333	NM_00129	12633	Cflar	2310024N1
chr12	59011077	59011275	Intergenic	Intergenic	-301615	NM_00921	20605	Sstr1	SRIF-2|SS-1
chr10	87783727	87783875	Intergenic	Intergenic	36019	NM_02787	71712	Dram1	1200002N1
chr19	23210177	23210250	Intergenic	CpG	-5503	NM_01063	16601	Klf9	2310051E1
chr18	35113627	35113975	intron (NM	CpG	204	NM_01048	15526	Hspa9	74kDa|Csa
chr2	1.44E+08	1.44E+08	intron (NM	intron (NM	3496	NM_01977	56431	Dstn	2610043P1
chr4	11250527	11250750	Intergenic	Intergenic	-1360	NM_00108	381510	Dpy19l4	Gm1023|N
chr11	1.11E+08	1.11E+08	Intergenic	MIRc|SINE	378810	NM_00842	16518	Kcnj2	IRK1|Kcnf1
chr1	1.35E+08	1.35E+08	exon (NM_	exon (NM_	2133	NM_13381	108954	Ppp1r15b	1810033K1
chr1	1.73E+08	1.73E+08	intron (NM	intron (NM	15571	NM_17264	16456	F11r	9130004G2
chr5	1.23E+08	1.23E+08	promoter-T	promoter-T	-53	NM_01102	18438	P2rx4	AI504491|
chr16	77039627	77039925	intron (NM	intron (NM	25462	NM_01391	30940	Usp25	-
chr16	36656052	36656350	intron (NM	intron (NM	9962	NM_01938	12524	Cd86	B7|B7-2|B
chr16	37868527	37868650	exon (NM_	exon (NM_	102	NM_17709	320184	Lrrc58	1810012N1
chr5	96589852	96590175	intron (NM	CpG	553	NM_00128	231464	Cnot6l	4932442K2
chr7	14682902	14683200	intron (NM	Lx8|LINE|L	25291	NM_00110	629203	Sult2a3	EG629203|
chr3	1.29E+08	1.29E+08	intron (NM	intron (NM	90472	NM_13045	170439	Elovl6	C77826|FA
chr13	44769052	44769150	Intergenic	Intergenic	-57042	NM_02187	16468	Jarid2	Jmj|jumon
chr13	3741377	3741625	Intergenic	Intergenic	62063	NM_02741	70405	Calml3	2310068O2
chr2	1.2E+08	1.2E+08	promoter-T	promoter-T	-178	NM_00116	68925	Rpap1	1190005L0
chr3	87709827	87710825	promoter-T	promoter-T	83	NM_00823	15191	Hdgf	AI118077|
chr9	56843277	56843500	5' UTR (NM	5' UTR (NM	613	NM_01965	56294	Ptpn9	MEG2
chr4	43071902	43072025	promoter-T	promoter-T	18	NM_02146	22249	Unc13b	Munc13-1|
chr11	84729852	84730000	promoter-T	promoter-T	-68	NM_00100	448850	Znhit3	Myohd1|Tr
chr14	68334452	68334750	promoter-T	promoter-T	446	NM_00128	105440	Kctd9	-

chr11	94853127	94853275	exon (NM_	exon (NM_	675	NM_17226	217124	Ppp1r9b	SPL|Spn
chr3	1.57E+08	1.57E+08	promoter-T	promoter-T	40	NM_01738	53861	Zranb2	AI227013|2
chr11	79067577	79067750	intron (NM	CpG	534	NM_01965	78889	Wsb1	1110056B1
chr13	98883202	98883300	intron (NM	intron (NM	92869	NM_01202	110596	Arhgef28	9230110L0
chr11	1.2E+08	1.2E+08	intron (NM	CpG	313	NM_00119	217365	Nploc4	AK129375|
chr10	53102627	53102725	Intergenic	Intergenic	-3019	NM_00120	1E+08	Cep85l	ENSMUSG0
chr2	11661277	11661450	Intergenic	Intergenic	34024	NM_00127	16169	Il15ra	AA690181|
chr9	21125777	21125875	intron (NM	intron (NM	663	NM_00119	68682	Slc44a2	1110028E1
chr5	53900827	53900900	Intergenic	Intergenic	-46155	NM_00108	19664	Rbpj	AI843960|
chr2	1.04E+08	1.04E+08	5' UTR (NM	5' UTR (NM	145	NM_01043	15259	Hipk3	DYRK6|FIST
chr1	1.83E+08	1.83E+08	exon (NM_	exon (NM_	202	NM_13322	170760	Acbd3	60kDa|843
chr2	28438402	28438650	5' UTR (NM	5' UTR (NM	273	NM_14892	70239	Gtf3c5	2700084A0
chr6	44419152	44419850	Intergenic	(CA)n|Simp	-590559	NM_00100	66797	Cntnap2	5430425M
chr5	1.11E+08	1.11E+08	promoter-T	promoter-T	143	NM_00814	269682	Golga3	5330413L0
chr1	93263427	93263550	intron (NM	intron (NM	481	NM_17339	227358	Fam132b	4832406C2
chr3	1.04E+08	1.04E+08	Intergenic	Intergenic	-5015	NM_00130	15257	Hipk1	1110062K0
chr18	61371202	61371600	promoter-T	promoter-T	-151	NM_00788	13521	Slc26a2	Dtd|ST-OB
chr7	52961027	52961175	exon (NM_	exon (NM_	484	NM_01697	13170	Dbp	-
chr11	1.22E+08	1.22E+08	intron (NM	intron (NM	4360	NM_14479	210029	Metrnl	9430048M
chr3	1.08E+08	1.08E+08	Intergenic	Intergenic	-6496	NM_00818	14867	Gstm6	-
chr4	43043502	43044500	intron (NM	CpG-9863	255	NM_02323	66592	Stoml2	0610038F0
chr8	13104852	13105050	promoter-T	promoter-T	392	NM_17870	234069	Pcid2	A730042J0
chr11	78250827	78250900	Intergenic	Intergenic	-15176	NM_02241	20500	Slc13a2	Nadc1|mN
chr10	1.12E+08	1.12E+08	5' UTR (NM	5' UTR (NM	269	NM_00103	382423	Atxn7l3b	4921506J0
chr10	61912677	61912775	promoter-T	promoter-T	-285	NM_18142	338359	Supv3l1	6330443E1
chr1	74552602	74553100	5' UTR (NM	5' UTR (NM	217	NM_02138	58184	Rqcd1	2610007F2
chr2	1.57E+08	1.57E+08	intron (NM	CpG	284	NM_01386	29812	Ndrg3	4833415O1
chr8	97542677	97542925	intron (NM	intron (NM	1209	NM_17303	54672	Gpr97	A030001G2
chr9	50287652	50287725	Intergenic	Intergenic	-14942	NM_02963	76509	Plet1	0610037B2
chr11	80322052	80322200	intron (NM	intron (NM	31578	NM_00987	12569	Cdk5r1	Cdk5r|D11
chr3	1.07E+08	1.07E+08	intron (NM	intron (NM	703	NM_14554	229709	Ahcyl1	1110034F2
chr1	1.9E+08	1.9E+08	intron (NM	intron (NM	-56555	NM_01193	26381	Esrrg	ERR3|Errg|
chr4	1.33E+08	1.33E+08	Intergenic	Intergenic	-23460	NM_00116	230789	Fam76a	-
chr6	1.48E+08	1.48E+08	intron (NM	intron (NM	61863	NM_17879	330450	Far2	A230046P1
chr11	50229302	50229375	intron (NM	RSINE1|SIN	15275	NM_17255	216724	Rufy1	3000002E0
chr1	1.01E+08	1.01E+08	Intergenic	Intergenic	305675	NM_02758	70866	Slco6d1	4921511I0
chr14	28662852	28663025	intron (NM	intron (NM	227310	NM_17781	238988	Erc2	6430531D0
chr19	42055577	42055675	promoter-T	promoter-T	0	NM_02815	72199	Mms19	2410001K2
chr10	8485827	8486075	intron (NM	intron (NM	119917	NM_17515	70097	Sash1	1100001C1
chr15	8405602	8405825	Intergenic	Intergenic	-11250	NM_20123	71175	Nipbl	Idn3
chr12	92654702	92655100	intron (NM	L2|LINE|L2	15468	NM_01164	22095	Tshr	AI481368|
chr12	32782127	32782325	Intergenic	Intergenic	-36082	NM_01115	19088	Prkar2b	AI451071|
chr7	1.18E+08	1.18E+08	Intergenic	B1_Mur1|S	-4627	NM_00943	22083	Ctr9	AA409336|
chr1	71444652	71444875	intron (NM	intron (NM	16721	NM_17521	74591	Abca12	4832428G1
chr13	74134077	74134200	Intergenic	MIRm|SINI	-12729	NM_18283	72948	Tppp	2900041A0
chr9	1.05E+08	1.05E+08	5' UTR (NM	5' UTR (NM	575	NM_17502	235574	Atp2c1	1700121J1
chr1	54894552	54894650	intron (NM	intron (NM	88630	NM_00108	329154	Ankrd44	4930444A1

chr1	1.55E+08	1.55E+08	promoter-T	promoter-T	-387	NM_00100	226517	Smg7	9430023P1
chr6	1.25E+08	1.25E+08	intron (NM	intron (NM	24095	NM_01353	14791	Emg1	C2f|Grcc2f
chr13	56218677	56218800	intron (NM	B4|SINE|B4	18173	NM_01201	26914	H2afy	H2AF12M|
chr4	6310552	6310725	intron (NM	intron (NM	17811	NM_00109	53378	Sdcbp	MDA-9|Syc
chr5	1.36E+08	1.36E+08	intron (NM	intron (NM	21792	NM_00889	18984	Por	4933424M
chr7	75275077	75275275	intron (NM	intron (NM	133943	NM_03010	78444	Pgpep1l	C330024D1
chr12	74154877	74155150	Intergenic	CpG	-7314	NM_00918	20471	Six1	BB138287
chr16	84884877	84885150	Intergenic	L1MB2|LIN	-49194	NM_01675	11957	Atp5j	-
chr15	58818102	58818250	intron (NM	intron (NM	52811	NM_02317	66218	Ndufb9	1190008J1
chr17	75546702	75546825	intron (NM	intron (NM	-31387	NM_20695	268977	Ltbp1	9430031G1
chr11	13390377	13390525	Intergenic	Intergenic	-1025488	NM_00128	12808	Cobl	-
chr11	3066077	3066150	intron (NM	B1_Mur4|S	27353	NM_03020	78887	Sfi1	-
chr2	31615827	31615900	5' UTR (NM	5' UTR (NM	170	NM_00128	11350	Abl1	AI325092|A
chr8	58564427	58564600	intron (NM	intron (NM	144891	NM_08043	110304	Glra3	-
chr17	34056752	34056975	TTS (NM_0	TTS (NM_0	440	NM_00931	21356	Tapbp	D17Wsu91
chr9	44346052	44346300	Intergenic	Intergenic	-11672	NM_00755	12145	Cxcr5	Blr1|CXC-R
chr17	70961602	70961725	intron (NM	intron (NM	50576	NM_00112	224997	Dlgap1	4933422O1
chr2	34964652	34964850	promoter-T	promoter-T	-261	NM_01201	26920	Cntrl	6720467O0
chr11	1.11E+08	1.11E+08	Intergenic	Intergenic	30473	NM_00842	16518	Kcnj2	IRK1|Kcnf1
chr13	55462502	55462800	intron (NM	intron (NM	1493	NM_02582	66890	Lman2	1110003H0
chr1	39506677	39507300	intron (NM	intron (NM	28604	NM_01877	54610	Tbc1d8	AD3|HBLP1
chr9	44729152	44729300	promoter-T	promoter-T	-401	NM_01379	27425	Atp5l	4933437C0
chr2	21625352	21625675	intron (NM	intron (NM	336319	NM_00100	241263	Gpr158	5330427M
chr1	1.58E+08	1.58E+08	Intergenic	Intergenic	-17204	NM_00923	20652	Soat1	8430426K1
chr1	1.83E+08	1.83E+08	promoter-T	promoter-T	-105	NM_00110	72568	Lin9	2700022J2
chr1	78334952	78335175	intron (NM	L3b|LINE|C	28142	NM_00100	433323	Sgpp2	SPPase2|Sp
chr19	46852877	46852975	intron (NM	L1MB2|LIN	16827	NM_00110	94219	Cnnm2	AU015877
chr7	4634852	4634975	intron (NM	A-rich|Low	1652	NM_02417	66245	Hspbp1	1500019G2
chr17	56428677	56428900	intron (NM	ID_B1|SINE	1146	NM_02583	66905	Plin3	1300012C1
chr3	10246952	10247500	intron (NM	intron (NM	-38650	NM_02440	11770	Fabp4	422/aP2|A
chr13	74527927	74528050	Intergenic	Intergenic	-13974	NM_00108	238722	Zfp72	Zfp74
chr13	39052402	39052750	promoter-T	promoter-T	168	NM_00117	108652	Slc35b3	4921526O0
chr3	93247452	93247900	exon (NM_	exon (NM_	1424	NM_00116	99681	Tchh	AHF|AI597
chr1	1.36E+08	1.36E+08	5' UTR (NM	5' UTR (NM	107	NM_02832	72674	Adipor1	2810031L1
chr4	1.41E+08	1.41E+08	intron (NM	intron (NM	16378	NM_17786	329972	Spata21	4933414G0
chr19	46150027	46150125	promoter-T	promoter-T	-261	NM_00103	70769	Nolc1	NOPP130|I
chr1	1.97E+08	1.97E+08	Intergenic	CpG	-65004	NM_00775	12902	Cr2	C3DR|CD21
chr18	78012727	78013025	intron (NM	CpG	369	NM_00750	11946	Atp5a1	AI035633|A
chr3	54611377	54611650	exon (NM_	exon (NM_	200	NM_13323	170767	Rfxap	5730495K2
chr2	1.35E+08	1.35E+08	Intergenic	RLTR11B|L	-47081	NM_02914	52837	Tmx4	2810417D0
chr9	61220827	61220900	5' UTR (NM	5' UTR (NM	690	NM_00108	21887	Tle3	2610103N0
chr17	71339952	71340200	promoter-T	promoter-T	-220	NM_02340	67938	Myl12b	1500001M
chr2	24285502	24285575	intron (NM	intron (NM	44621	NM_17761	215632	Psd4	BC046518|
chr6	38270652	38270850	intron (NM	RLTR45|LT	-21492	NM_17246	209032	Zc3hav1l	B130055L0
chr14	76154027	76154150	exon (NM_	exon (NM_	212	NM_17738	338337	Cog3	E430004N2
chr2	1.47E+08	1.47E+08	intron (NM	CpG	330	NM_01191	24128	Xrn2	-
chr4	16029802	16030300	Intergenic	Intergenic	60594	NM_13895	192656	Ripk2	2210420D1

chr7	1.05E+08	1.05E+08	Intergenic	Intergenic	-19531	NM_17510	66333	Aqp11	1700015P1
chr15	63489552	63489850	Intergenic	Intergenic	150593	NM_03137	83492	Gsdmc	Gsdmc1|M
chr1	72475052	72475650	exon (NM_	exon (NM_	107787	NM_00104	381270	4-Mar	BC056494|
chr15	36723077	36723150	intron (NM	CpG	1180	NM_01174	22631	Ywhaz	1110013I1
chr11	60637152	60637475	Intergenic	ID_B1|SINI	-6820	NM_00117	216820	Dhrs7b	BC003479|
chr12	88784077	88784800	promoter-T	promoter-T	351	NM_00110	211064	Alkbh1	2700073G1
chr3	88754802	88755425	promoter-T	promoter-T	-909	NM_00116	65111	Dap3	4921514D1
chr1	1.59E+08	1.59E+08	Intergenic	Intergenic	-47044	NM_02388	78255	Ralgps2	1810020P1
chr12	77505377	77505800	exon (NM_	exon (NM_	231	NM_00830	15512	Hspa2	70kDa|HSF
chr15	78411977	78412550	Intergenic	Intergenic	-9050	NM_00900	19354	Rac2	AI323801|/
chr2	1.52E+08	1.52E+08	intron (NM	intron (NM	543	NM_02419	67231	Tbc1d20	1110028I0
chr2	26881127	26881275	intron (NM	L2|LINE|L2	2317	NM_17265	227659	Slc2a6	A330096C2
chr2	1.12E+08	1.12E+08	Intergenic	Intergenic	-6972	NM_20720	99010	Lpcat4	AI505034|/
chr16	4077527	4077875	exon (NM_	exon (NM_	109	NM_02650	68015	Trap1	2410002K2
chr6	54987827	54988225	promoter-T	promoter-T	31	NM_18067	353172	Gars	GENA202|(
chr14	73798202	73798500	Intergenic	Intergenic	-13273	NM_00841	16432	Itm2b	AI256040|I
chr9	65393902	65394000	Intergenic	Intergenic	33943	NM_15311	102595	Plekho2	AI840980|I
chr4	1.2E+08	1.2E+08	intron (NM	CpG	430	NM_01674	51797	Ctps	Ctps1
chr2	1.65E+08	1.65E+08	intron (NM	CpG	133	NM_14612	228866	Pcif1	2310022K1
chr13	9882202	9882400	intron (NM	intron (NM	-117741	NM_14451	66505	Zmynd11	2210402G2
chrX	33561502	33561625	intron (NM	PB1D10|SI	-90571	NM_13399	16164	Il13ra1	AI882074|(
chr17	34764652	34764925	promoter-T	promoter-T	-746	NM_01944	54397	Ppt2	0610007M
chr11	53584127	53584300	intron (NM	intron (NM	238	NM_00115	16362	Irf1	AU020929
chr15	59480152	59480300	promoter-T	promoter-T	17	NM_14454	211770	Trib1	A530090O1
chr6	52141902	52142250	promoter-T	promoter-T	-374	NM_00826	15401	Hoxa4	AV206827|
chr2	35585427	35585500	3' UTR (NM	3' UTR (NM	33211	NM_00129	69601	Dab2ip	2310011D0
chr2	1.51E+08	1.51E+08	intron (NM	intron (NM	-14222	NM_00129	241727	Snph	6430515A0
chr1	1.3E+08	1.3E+08	Intergenic	Intergenic	30763	NM_00991	12767	Cxcr4	CD184|Cm
chr5	1.22E+08	1.22E+08	promoter-T	promoter-T	-82	NM_01120	19247	Ptpn11	2700084A1
chr7	1.21E+08	1.21E+08	intron (NM	intron (NM	1040	NM_02737	67420	Far1	2600011M
chr16	33381352	33381550	promoter-T	promoter-T	590	NM_01174	22661	Zfp148	2210405J0
chr6	1.25E+08	1.25E+08	intron (NM	intron (NM	8651	NM_17555	269800	Zfp384	BB163993|
chr18	70782152	70782375	exon (NM_	exon (NM_	54317	NM_01077	17191	Mbd2	MBD2a
chr2	1.64E+08	1.64E+08	intron (NM	CpG-8831	617	NM_02557	66460	Sys1	2610042O1
chr3	1.29E+08	1.29E+08	intron (NM	intron (NM	80059	NM_13045	170439	Elovl6	C77826|FA
chr6	83081827	83081925	Intergenic	MER63B|D	-3926	NM_00113	20166	Rtkn	-
chr10	1.27E+08	1.27E+08	intron (NM	intron (NM	17366	NM_00851	16971	Lrp1	A2mr|AI31
chr3	1.08E+08	1.08E+08	promoter-T	promoter-T	-204	NM_02619	67495	Tmem167b	2010200O1
chr5	1.22E+08	1.22E+08	exon (NM_	exon (NM_	53736	NM_00116	231712	Trafd1	1110008K0
chr17	16448252	16448350	Intergenic	Intergenic	-484751	NM_17861	68799	Rgmb	1110059F1
chr1	52289102	52289250	intron (NM	CpG	900	NM_00108	14660	Gls	6330442B1
chr16	49800852	49800950	Intergenic	Intergenic	-54866	NM_01058	16423	Cd47	9130415E2
chr1	1.93E+08	1.93E+08	intron (NM	CpG	724	NM_00749	11910	Atf3	LRG-21
chr17	28575727	28575925	intron (NM	intron (NM	47268	NM_01022	14229	Fkbp5	D17Ertd59
chr11	69808927	69809100	promoter-T	promoter-T	-255	NM_00129	78246	Phf23	1-Jun
chr8	64493002	64493275	intron (NM	intron (NM	86254	NM_00129	234311	Ddx60	9830118M
chr5	1.24E+08	1.24E+08	Intergenic	Intergenic	-4200	NM_17509	23912	Rhof	AI845056|/

chr17	24856577	24857900	promoter-T	promoter-T	230	NM_00850	16898	Rps2	Llrep3|S4
chr1	1.84E+08	1.84E+08	Intergenic	Intergenic	-18394	NM_13381	98386	Lbr	AI505894|i
chr18	32664602	32664750	Intergenic	Intergenic	55012	NM_00104	71683	Gypc	0610037F2
chr11	1.02E+08	1.02E+08	promoter-T	promoter-T	-66	NM_00115	237943	Gpatch8	5430405G2
chr4	63005277	63005700	promoter-T	promoter-T	-112	NM_00876	18405	Orm1	Agp-1|Agp
chr15	57847002	57847275	Intergenic	Intergenic	18711	NM_02941	75758	9130401M	AI849328
chr6	71583077	71583900	promoter-T	promoter-T	-577	NM_00103	104263	Kdm3a	1700105C2
chr17	26005127	26005225	intron (NM	intron (NM	507	NM_02663	68241	Fam195a	9530058B0
chr1	1.56E+08	1.56E+08	TTS (NR_1(	TTS (NR_1(	1004	NM_00813	14645	Glul	GS|Glns
chr8	1.23E+08	1.23E+08	intron (NM	intron (NM	7884	NM_00116	234797	6430548M	AW049007
chr12	21422452	21422550	intron (NM	intron (NM	796	NM_01173	22630	Ywhaq	2700028P0
chr10	1.05E+08	1.05E+08	promoter-T	promoter-T	48	NM_20752	216292	Mettl25	-
chr3	1E+08	1E+08	promoter-T	promoter-T	-630	NM_01076	17156	Man1a2	AI428775|/
chr7	86433327	86433425	intron (NM	intron (NM	15224	NM_01881	54608	Abhd2	2210009N1
chr14	1.04E+08	1.04E+08	Intergenic	Intergenic	-62520	NM_02288	64929	Scel	9230114I0
chr13	47509252	47509450	Intergenic	Intergenic	219979	NM_00117	218215	Rnf144b	BC025007|
chr2	1.66E+08	1.66E+08	intron (NM	intron (NM	39059	NM_00125	72043	Sulf2	2010004N2
chr17	4995377	4995525	exon (NM_	exon (NM_	377	NM_00108	239985	Arid1b	8030481M
chr15	95625727	95625900	intron (NM	intron (NM	4539	NM_00125	105722	Ano6	2900059G1
chr9	68882352	68882525	intron (NM	intron (NM	-255210	NM_00128	19883	Rora	9530021D1
chr13	1.09E+08	1.09E+08	intron (NM	CpG	165	NM_02900	74559	Elovl7	9130013K2
chr3	90035552	90035650	promoter-T	promoter-T	82	NM_20692	23922	Jtb	Gm622
chr7	1.08E+08	1.08E+08	intron (NM	intron (NM	7939	NM_17707	320100	Relt	E430021K2
chr8	1.09E+08	1.09E+08	intron (NM	intron (NM	36301	NM_00922	20650	Sntb2	Snt2
chr17	29250877	29250950	Intergenic	Intergenic	20196	NM_00766	12575	Cdkn1a	CAP20|CDI
chr19	16854577	16854675	intron (NM	intron (NM	797	NM_17302	271564	Vps13a	4930425F1
chr1	9535527	9535725	exon (NM_	exon (NM_	137	NM_02151	59014	Rrs1	5730466A0
chr4	1.5E+08	1.5E+08	Intergenic	Intergenic	-4321	NM_02056	57320	Park7	DJ-1|Dj1
chr1	26348577	26348800	Intergenic	Intergenic	395617	NM_00103	210940	4931408C2	-
chr2	26593277	26593525	Intergenic	Intergenic	-66599	NM_01069	16821	Lcn4	A630045M
chr3	1.09E+08	1.09E+08	intron (NM	intron (NM	-95649	NM_14613	57257	Vav3	A530094I0
chr11	4136702	4136900	promoter-T	promoter-T	13	NM_00101	18413	Osm	OncoM
chr9	21397777	21398250	promoter-T	promoter-T	-738	NM_00120	74766	Yipf2	1300010K0
chr17	47469602	47469700	intron (NM	intron (NM	-36185	NM_00114	64657	Mrps10	1110038B1
chr14	73725852	73726000	promoter-T	promoter-T	-328	NM_00902	19645	Rb1	Rb|Rb-1|p
chrX	1.39E+08	1.39E+08	exon (NM_	exon (NM_	1194	NM_00119	209497	Tmem164	AI316850|/
chr4	1.08E+08	1.08E+08	intron (NM	CpG	294	NM_00103	414872	Zyg11b	1110046I0
chr10	9716127	9716325	Intergenic	Intergenic	-95388	NM_00108	78808	Stxbp5	0710001E2
chr5	23606777	23606925	intron (NM	CpG	443	NM_02644	52323	Klhl7	2700038B0
chr15	98513602	98513775	Intergenic	Intergenic	-5796	NM_17261	223881	Rnd1	A830014L0
chr8	30147602	30147750	intron (NM	MIRm|SINI	182432	NM_15313	210801	Unc5d	D930029E1
chr3	89153927	89154050	promoter-T	promoter-T	-56	NM_00961	11490	Adam15	MDC15|me
chr11	98725052	98725275	5' UTR (NM	5' UTR (NM	251	NM_19794	68524	Wipf2	1110014J0
chr1	1.58E+08	1.58E+08	intron (NM	intron (NM	9834	NM_00116	208263	Tor1aip1	LAP1|Lap1
chr11	98543702	98544150	promoter-T	promoter-T	58	NM_00943	22123	Psmd3	AI255837|/
chr11	5906352	5906500	intron (NM	RLTR4_MM	50665	NM_01966	56418	Ykt6	0610042I1!
chr3	83689127	83689625	Intergenic	MIRb|SINE	-43846	NM_01190	24088	Tlr2	Ly105

chr3	89216177	89216275	promoter-T	promoter-T	-441	NM_17704	319945	Flad1	A930017E2
chr12	1.01E+08	1.01E+08	intron (NM	intron (NM	100108	NM_18318	71375	Foxn3	5430426H2
chr12	29435202	29435275	promoter-T	promoter-T	21	NM_00116	217449	Trappc12	CGI-87|D9
chr10	79794452	79794675	intron (NM	intron (NM	1673	NM_13929	70335	Reep6	0610011M
chr5	64465677	64465850	Intergenic	Intergenic	-18426	NM_02570	66681	Pgm1	3230402E0
chr17	24614702	24614875	intron (NM	intron (NM	1235	NM_00125	56716	Mlst8	0610033N1
chr15	91113177	91113250	intron (NM	RMER19B|	-90975	NM_01199	26874	Abcd2	ABC39|ALD
chr4	1.52E+08	1.52E+08	intron (NM	intron (NM	1138	NM_00125	16498	Kcnab2	F5|I2rf5|K
chr3	21852352	21852425	Intergenic	Intergenic	-123186	NM_03073	81004	Tbl1xr1	8030499H0
chrX	7418977	7419075	promoter-T	promoter-T	69	NM_13860	54644	Otud5	AA407879|
chr1	1.27E+08	1.27E+08	intron (NM	intron (NM	15578	NM_02373	74117	Actr3	1200003A0
chr2	52802852	52802950	intron (NM	intron (NM	85999	NM_17240	71409	Fmnl2	5430425K0
chr13	97907852	97908175	promoter-T	promoter-T	121	NM_00127	320806	Gfm2	6530419G1
chr16	32555827	32556000	Intergenic	Intergenic	-53069	NM_01163	22042	Tfrc	2610028K1
chr16	24859752	24859950	intron (NM	intron (NM	137910	NM_00114	210126	Lpp	9430020K1
chr14	48124002	48124225	intron (NM	intron (NM	31877	NM_00114	78938	Fbxo34	2900057B0
chr5	73259477	73259600	intron (NM	intron (NM	149	NM_00111	21682	Tec	-
chr5	1.45E+08	1.45E+08	intron (NM	B4|SINE|B	8971	NM_00108	231876	Lmtk2	2900041G1
chr9	43839077	43839425	Intergenic	B4|SINE|B	-12216	NM_00938	21838	Thy1	CD90|T25|
chr2	1.37E+08	1.37E+08	intron (NM	CpG	1955	NM_01382	16449	Jag1	ABE2|Gsfa
chr9	64129577	64129650	intron (NM	CpG	199	NM_02537	66131	Tipin	1110005A0
chr13	37511102	37511225	TTS (NM_0	TTS (NM_0	73949	NM_01074	17084	Ly86	MD-1|MD1
chr19	7062152	7062225	promoter-T	promoter-T	-47	NM_01169	22340	Vegfb	VEGF-B|Vr
chr12	70782127	70782250	intron (NM	intron (NM	651	NM_00113	20663	Sos2	SOS-2|mSO
chr2	1.65E+08	1.65E+08	promoter-T	promoter-T	74	NM_19902	329559	Zfp335	1810045J0
chr17	73765777	73765975	Intergenic	URR1B|DN	-17240	NM_00103	381122	Capn13	CANP 13|G
chr10	13023877	13023950	intron (NM	intron (NM	20053	NM_00119	215789	Phactr2	AV158170|
chr7	71083127	71083350	exon (NM_	exon (NM_	563	NM_02136	50794	Klf13	0610043C1
chr5	75148152	75148325	Intergenic	B4|SINE|B	-49310	NM_00115	16924	Lnx1	Lnx
chr7	38804352	38804500	5' UTR (NM	5' UTR (NM	145	NM_01127	19777	Uri1	C80913|NM
chr9	50401177	50401300	Intergenic	Intergenic	10716	NM_02584	66925	Sdhd	3110001M
chr10	36693702	36694200	promoter-T	promoter-T	-399	NM_00822	15182	Hdac2	D10Wsu17
chr13	32926427	32926850	Intergenic	Intergenic	16416	NM_02542	66222	Serpinb1a	1190005M
chr5	76208777	76208850	Intergenic	Intergenic	165640	NM_01061	16542	Kdr	6130401C0
chr15	63833077	63833350	intron (NM	intron (NM	58797	NM_14484	223601	Fam49b	0910001A0
chr10	3283152	3283350	Intergenic	Intergenic	-40105	NM_00103	320495	Ipcef1	A130090K0
chr5	67785277	67785475	3' UTR (NM	3' UTR (NM	33662	NM_00116	666938	Bend4	D330027G2
chr1	39544477	39544650	Intergenic	Intergenic	-8971	NM_01877	54610	Tbc1d8	AD3|HBLP1
chr11	60034077	60034150	promoter-T	promoter-T	-7	NM_01148	20787	Srebf1	ADD-1|ADD
chr1	83073802	83073875	Intergenic	Intergenic	-38815	NM_03055	80721	Slc19a3	A230084E2
chr16	16568002	16568175	Intergenic	Intergenic	-7799	NM_13923	224014	Fgd4	9030023J0
chr18	66164877	66165075	Intergenic	L1M5|LINE	-2687	NM_00117	70361	Lman1	2610020P1
chr6	1.23E+08	1.23E+08	Intergenic	Intergenic	17674	NM_00964	11628	Aicda	Aid|Arp2
chr14	61063077	61063325	Intergenic	RLTR40|LT	66078	NM_00116	72125	Amer2	2600011E0
chr17	79102427	79102625	intron (NM	RMER6-int	33374	NM_01150	268980	Strn	AU022939
chr4	1.19E+08	1.19E+08	intron (NM	CpG	115	NM_00111	66101	Ppih	1100001J0
chr13	49311552	49311725	promoter-T	promoter-T	-243	NM_02673	68480	1110007C0	AI851695|

chr11	22764702	22765100	Intergenic	Intergenic	-4565	NM_00116	53625	B3gnt2	AA408337|
chr2	1.21E+08	1.21E+08	intron (NM	intron (NM	1378	NM_17528	96957	Tmem62	B830009D2
chr8	81818352	81818450	promoter-T	promoter-T	454	NM_13382	109136	Mmaa	2810018E0
chr6	1.19E+08	1.19E+08	intron (NM	intron (NM	24538	NM_19798	68465	Adipor2	1110001I1
chr12	52945902	52946075	Intergenic	Intergenic	-15465	NM_14478	207304	Hectd1	A630086P0
chr1	66747552	66747725	intron (NM	CpG	171	NM_02568	66646	Rpe	2810429B0
chr10	1.28E+08	1.28E+08	promoter-T	promoter-T	523	NM_01886	67945	Rpl41	1810055P1
chr1	33814352	33814525	exon (NM_	exon (NM_	157	NM_14539	213539	Bag2	2610042A1
chr5	1.37E+08	1.37E+08	Intergenic	B1_Mus1|S	-61103	NM_00129	13047	Cux1	CDP|Cutl1
chr11	50190477	50190725	promoter-T	promoter-T	-620	NM_02151	59013	Hnrnph1	AI642080|
chr6	1.23E+08	1.23E+08	Intergenic	CpG	-18626	NM_02189	60611	Foxj2	Fhx
chr16	32165627	32165725	promoter-T	promoter-T	-114	NM_14606	224109	Nrros	E430025L0
chr1	1.2E+08	1.2E+08	promoter-T	promoter-T	-42	NM_01165	22099	Tsn	2610034C2
chr1	95715977	95716050	3' UTR (NM	3' UTR (NM	-5804	NM_17888	98314	D2hgdh	AA408776|
chr5	44466627	44467000	intron (NM	intron (NM	19194	NM_00116	19126	Prom1	4932416E1
chr9	75162052	75162125	exon (NM_	exon (NM_	213	NM_13871	14697	Gnb5	GBS|Gbeta
chr1	1.22E+08	1.22E+08	intron (NM	intron (NM	676	NM_00111	226352	Epb4.1l5	1700030C1
chr7	1.35E+08	1.35E+08	intron (NM	CpG	448	NM_14620	233893	Zfp764	8030466O1
chr16	30873002	30873075	Intergenic	(CAGGA)n|	208480	NM_19862	268880	Xxylt1	AI480653
chr10	12589027	12589100	Intergenic	CpG	-7530	NM_01168	22288	Utrn	AA589569|
chr6	1.34E+08	1.34E+08	intron (NM	intron (NM	18254	NM_02577	66813	Bcl2l14	4930452K2
chr2	1.02E+08	1.02E+08	5' UTR (NM	5' UTR (NM	119	NM_02026	80985	Trim44	Dipb|Mc7
chr2	1.8E+08	1.8E+08	Intergenic	L1MA9|LIN	-15157	NM_14893	108115	Slco4a1	OATP-E|Slc
chr10	1.17E+08	1.17E+08	Intergenic	Intergenic	-9102	NM_01359	17110	Lyz1	Lyz|Lzp-s
chr1	72544052	72544475	intron (NM	L1M5|LINE	38875	NM_00104	381270	4-Mar	BC056494|
chr15	57878152	57878600	TTS (NM_0	TTS (NM_0	-12527	NM_02941	75758	9130401M	AI849328
chr11	21271302	21271650	promoter-T	promoter-T	-206	NM_00129	216558	Ugp2	UDPGP|UG
chr3	19126002	19126175	3' UTR (NM	3' UTR (NM	37867	NR_045575	67472	Mtfr1	1300002C0
chr1	88375677	88376000	Intergenic	Intergenic	-47473	NM_00897	19231	Ptma	Thym
chr1	36766102	36766175	intron (NM	intron (NM	632	NM_14610	226977	Actr1b	2310066K2
chr3	9957952	9958275	Intergenic	Intergenic	-54472	NM_00127	16592	Fabp5	E-FABP|Fa
chr7	1.4E+08	1.4E+08	Intergenic	Intergenic	-98863	NM_02993	77590	Chst15	4631426J0
chr7	19894827	19894900	5' UTR (NM	5' UTR (NM	531	NM_00803	14282	Fosb	-
chr17	6079877	6079975	promoter-T	promoter-T	98	NM_18139	66467	Gtf2h5	2700017P0
chr10	79578027	79578125	Intergenic	Intergenic	-1205	NM_01149	20869	Stk11	AA408040|
chr3	79394802	79394875	promoter-T	promoter-T	-473	NM_02635	67738	Ppid	4930564J0
chr5	77738302	77738475	exon (NM_	exon (NM_	723	NM_01983	56412	Noa1	2610024G1
chr1	94831327	94831525	non-coding	non-coding	441	NM_01179	23830	Capn10	AW049679
chr17	74715852	74716125	promoter-T	promoter-T	-215	NM_00114	66310	Dpy30	2810410M
chr11	1.21E+08	1.21E+08	promoter-T	promoter-T	40	NM_00117	209318	Gps1	Cops1|Csn
chr3	94386452	94386675	promoter-T	promoter-T	75	NM_00108	76742	Snx27	5730552M
chr19	37000552	37000725	promoter-T	promoter-T	69	NM_00108	107182	Btaf1	AI414500|
chr17	25929427	25929700	promoter-T	promoter-T	-224	NM_14541	214917	Fam173a	-
chr7	1.17E+08	1.17E+08	promoter-T	promoter-T	-540	NM_00928	20841	Zfp143	AA959806|
chr2	1.56E+08	1.56E+08	intron (NM	intron (NM	-54096	NM_02758	70873	Cnbd2	4921517L1
chr6	1.21E+08	1.21E+08	intron (NM	intron (NM	487	NM_00754	12122	Bid	2700049M
chr4	1.35E+08	1.35E+08	Intergenic	Intergenic	-2840	NM_02638	52830	Pnrc2	0610011E1

chr5	77583802	77584000	Intergenic	Intergenic	-39753	NM_17560	74318	Hopx	1110018K1
chrX	97821227	97821350	promoter-T	promoter-T	-42	NM_02830	72621	Pdzd11	1810012H2
chr7	1.34E+08	1.34E+08	promoter-T	promoter-T	-344	NM_01136	20399	Sh2b1	AI425885|(
chr1	1.35E+08	1.35E+08	Intergenic	Intergenic	-41631	NM_01073	16980	Lrrn2	5730406J0
chr17	8070052	8070175	Intergenic	Intergenic	-48752	NM_14596	72536	Tagap	2610315E1
chr11	51419477	51419675	intron (NM	CpG	807	NM_00104	15384	Hnrnpab	3010025C1
chr19	38129477	38129550	promoter-T	promoter-T	-2	NM_02829	74107	Cep55	1200008O1
chr11	1.01E+08	1.01E+08	exon (NM_	exon (NM_	1381	NM_00772	12777	Ccr10	Cmkbr9|Gp
chr8	1.08E+08	1.08E+08	promoter-T	promoter-T	240	NM_01582	50788	Fbxl8	FBL8
chr4	1.07E+08	1.07E+08	intron (NM	intron (NM	558	NM_02550	56280	Mrpl37	2300004O1
chr1	1.27E+08	1.27E+08	Intergenic	Intergenic	90453	NM_02373	74117	Actr3	1200003A0
chr1	90175627	90176000	Intergenic	Intergenic	-1659	NM_19865	381280	Hjurp	6430706D2
chr1	26340802	26341050	Intergenic	Intergenic	403379	NM_00103	210940	4931408C2	-
chr6	29559452	29559775	promoter-T	promoter-T	-6	NM_17729	320938	Tnpo3	5730544L1
chr14	1.05E+08	1.05E+08	intron (NM	CpG	257	NM_02604	72486	Rnf219	2610206B1
chr9	32334577	32334850	intron (NM	intron (NM	14324	NM_00802	14247	Fli1	EWSR2|Fli-
chr4	88183402	88183600	Intergenic	Intergenic	-14803	NM_01051	15977	Ifnb1	IFN-beta|IF
chr19	45870827	45871025	intron (NM	intron (NM	-13145	NM_02379	76055	Mgea5	2810009A2
chr12	76836852	76837050	promoter-T	promoter-T	-235	NM_03075	81535	Sgpp1	AI463453|S
chr10	39797477	39797675	intron (NM	intron (NM	-52502	NM_14482	215929	AI317395	AI482555|I
chr19	43722977	43723100	Intergenic	Intergenic	26333	NM_14515	246696	Slc25a28	2210403D1
chr12	16539227	16539625	Intergenic	Intergenic	57150	NM_01576	14245	Lpin1	4631420P0
chr13	45350802	45351000	Intergenic	Intergenic	-134210	NM_15378	218203	Mylip	9430057C2
chr1	88294927	88295050	Intergenic	Intergenic	-10272	NM_01034	14767	Nmur1	FM-3|Gpr6
chr18	67696277	67696525	intron (NM	intron (NM	12426	NM_17683	68166	Spire1	6030430B1
chr2	1.51E+08	1.51E+08	promoter-T	promoter-T	-334	NM_00801	14225	Fkbp1a	12kDa|FKB
chr6	32117727	32118000	intron (NM	MER91C|D	117575	NM_02958	76382	1700012A0	AI429084
chr9	65738177	65738350	exon (NM_	exon (NM_	133	NM_02651	68026	2810417H1	AA409629|
chr1	36971227	36971525	intron (NM	intron (NM	24996	NM_01887	56030	Tmem131	2610524E0
chr13	1.01E+08	1.01E+08	Intergenic	(CAT)n|Sim	143662	NM_01373	27220	Cartpt	Cart
chr12	81215302	81215600	Intergenic	CpG	-1451	NM_00756	12192	Zfp36l1	AW742437
chr11	1.16E+08	1.16E+08	promoter-T	promoter-T	4	NM_17880	338364	Trim65	4732463G1
chr15	81529002	81529250	Intergenic	Intergenic	-1409	NM_00116	214685	Chadl	AY100452|
chr14	47407502	47407950	intron (NM	CpG	306	NM_00991	12793	Cnih1	0610007J1
chr3	90313327	90313450	promoter-T	promoter-T	32	NM_00129	66511	Chtop	2500003M
chr8	4276077	4276200	promoter-T	promoter-T	-233	NM_01159	21856	Timm44	0710005E2
chr8	34966177	34966575	intron (NM	L1MB4|LIN	73959	NM_01973	19663	Rbpms	2010300K2
chr6	1.49E+08	1.49E+08	Intergenic	Intergenic	-2722	NM_00125	320204	Mettl20	4833442J1
chr3	60275827	60276200	promoter-T	promoter-T	-739	NM_00125	56758	Mbnl1	Mbnl|mKIA
chr2	30061752	30061950	promoter-T	promoter-T	-632	NM_17240	70266	Ccbl1	2010009K0
chr4	56814877	56815250	promoter-T	promoter-T	-138	NM_00108	230234	Fam206a	-
chr7	16633177	16633400	Intergenic	CpG	-24003	NM_00109	666528	Zfp541	EG666528|
chr1	87397627	87397950	Intergenic	Intergenic	97597	NM_03019	109032	Sp110	5031415C0
chr7	28141827	28141975	promoter-T	promoter-T	-874	NM_13867	192192	Shkbp1	B930062H1
chr9	67692877	67693050	intron (NM	intron (NM	4760	NM_17718	320528	Vps13c	AI552542|A
chr2	38336852	38337025	Intergenic	Intergenic	-30279	NM_02160	59126	Nek6	1300007C0
chr1	64244852	64245150	Intergenic	Intergenic	-77038	NM_03356	93691	Klf7	9830124P0

chr1	30640852	30641000	Intergenic	Intergenic	279175	NM_00108	213109	Phf3	2310061N1
chr8	68982427	68982550	intron (NM	intron (NM	27918	NM_02546	66282	Tma16	1810029B1
chr5	23167052	23167275	Intergenic	Intergenic	-44774	NM_00927	20817	Srpk2	AW226533
chr13	53024727	53025025	exon (NM_	exon (NM_	170	NM_01670	11992	Auh	C77140|W
chr1	34858127	34858400	promoter-T	promoter-T	-304	NM_18301	226970	Arhgef4	9330140K1
chr3	98143277	98143400	intron (NM	CpG	554	NM_01696	236539	Phgdh	3-PGDH|3P
chr4	34554102	34554225	intron (NM	intron (NM	8028	NM_00115	50793	Orc3	Orc3l
chr18	3382902	3382975	promoter-T	promoter-T	-285	NM_02940	71745	Cul2	1300003D1
chr9	54996577	54996650	promoter-T	promoter-T	-563	NM_18060	109161	Ube2q2	3010021M
chr3	1.06E+08	1.06E+08	intron (NM	intron (NM	-3215	NM_00128	67171	Dram2	2010305N1
chr5	1.26E+08	1.26E+08	Intergenic	Intergenic	-15001	NM_01963	22190	Ubc	2700054O(
chr19	32463152	32463225	promoter-T	promoter-T	-244	NM_14479	208449	Sgms1	9530058O1
chr13	43391002	43391250	intron (NM	intron (NM	8415	NM_00103	328232	Gfod1	9630032O1
chr2	1.81E+08	1.81E+08	promoter-T	promoter-T	8	NM_00116	76688	Arfrp1	1500006I0
chr2	32390827	32390975	promoter-T	promoter-T	22	NM_15356	98952	Fam102a	AI426465|(
chr3	96548352	96548500	intron (NM	intron (NM	16892	NM_02640	67845	Rnf115	2610028E0
chr6	35494327	35494550	Intergenic	MLT1I-int|	-4550	NM_00809	14489	Mtpn	5033418D1
chr12	1.1E+08	1.1E+08	intron (NM	intron (NM	3560	NM_18132	214663	Slc25a29	C030003J1
chr10	1.19E+08	1.19E+08	intron (NM	CpG	698	NM_02799	71902	Cand1	2310038O(
chr5	21069802	21070000	intron (NM	intron (NM	20556	NM_02897	74511	Lrrc17	37kDa|483
chr13	1.11E+08	1.11E+08	intron (NM	(CA)n|Simp	60276	NM_02385	67295	Rab3c	2700062I0
chr15	80542227	80542425	intron (NM	CpG	583	NM_14481	213988	Tnrc6b	2700090M
chr9	44067427	44067550	intron (NM	intron (NM	-7941	NM_13322	170761	Pdzd3	NaPi-Cap2|
chr6	72352027	72352200	Intergenic	B3A|SINE|	-11452	NM_01679	22320	Vamp8	AU041171
chr8	67355952	67356175	intron (NM	intron (NM	17759	NM_17863	77113	Klhl2	6030411N2
chr17	81085452	81085750	intron (NM	L1M4|LINE	41764	NM_00129	225028	Map4k3	4833416M
chr1	1.94E+08	1.94E+08	promoter-T	promoter-T	48	NM_17226	226856	Lpgat1	AI649174|/
chr8	32969677	32970025	intron (NM	intron (NM	58824	NM_17859	211323	Nrg1	6030402G2
chr6	91629002	91629225	Intergenic	Intergenic	-4948	NM_00932	21366	Slc6a6	AA589629|
chr8	96535852	96536275	intron (NM	CpG	477	NM_01178	23802	Amfr	gp78
chr5	1.44E+08	1.44E+08	intron (NM	intron (NM	673	NM_00900	19353	Rac1	AL023026|
chr9	22171002	22171400	intron (NM	intron (NM	22449	NM_02839	68743	Anln	1110037A1
chr2	1.13E+08	1.13E+08	intron (NM	intron (NM	-29009	NM_00128	14260	Fmn1	Fmn|ld
chr3	95084677	95084800	intron (NM	intron (NM	1260	NM_17334	229589	Prune	9230112O(
chr5	1.51E+08	1.51E+08	promoter-T	promoter-T	-20	NM_00976	12190	Brca2	Fancd1|RA
chr9	7836902	7836975	Intergenic	CpG	-1683	NM_00746	11797	Birc2	AW146227
chr19	6277027	6277150	exon (NM_	exon (NM_	192	NM_01011	13660	Ehd1	AA409636|
chr5	1.46E+08	1.46E+08	promoter-T	promoter-T	81	NM_00129	54188	Cpsf4	30kDa|C79
chr1	57464027	57464125	promoter-T	promoter-T	-558	NM_00103	68736	Tyw5	1110034B0
chr3	97817002	97817175	promoter-T	promoter-T	-373	NM_01092	18129	Notch2	AI853703|I
chr16	45616227	45616400	intron (NM	intron (NM	5760	NM_00115	14525	Gcsam	Gcet|Gcet2
chr14	32475027	32475175	intron (NM	CpG	20858	NM_02529	26363	Btd	-
chr1	1.33E+08	1.33E+08	intron (NM	CpG	575	NM_00108	14270	Srgap2	9930124L2
chr1	44176127	44176250	promoter-T	promoter-T	-570	NM_02364	72050	Kdelc1	1810049A1
chr17	56359202	56359600	Intergenic	Intergenic	-1089	NM_17262	224897	Dpp9	6430584G1
chr13	3584827	3584900	intron (NM	intron (NM	25491	NM_13406	105203	Fam208b	AI645998|I
chr1	36613602	36613925	intron (NM	CpG	1463	NM_00112	20353	Sema4c	AI426163|S

chr17	79168727	79168875	intron (NM	intron (NM	-32901	NM_01150	268980	Strn	AU022939
chr12	32992227	32992350	Intergenic	RMER15|LT	-71366	NM_00116	72123	Ccdc71l	2010109K1
chr4	1.5E+08	1.5E+08	intron (NM	PB1D10|SI	2593	NM_00102	433182	Eno1b	EG433182|
chr13	58316752	58317300	promoter-T	promoter-T	-12	NM_02684	56085	Ubqln1	1110046H0
chr11	77303027	77303150	promoter-T	promoter-T	92	NM_17294	268445	Ankrd13b	AW124583
chr7	29909927	29910000	intron (NM	intron (NM	207	NM_00910	20190	Ryr1	AI528790|I
chr12	92621627	92621800	intron (NM	intron (NM	1136	NM_18181	75216	Cep128	4930534B0
chr11	98650652	98650825	Intergenic	Intergenic	-6345	NM_02872	74026	Msl1	2810017F1
chr3	1.21E+08	1.21E+08	Intergenic	Intergenic	-38851	NM_14539	213603	Slc44a3	BC010552
chr4	88766377	88766450	Intergenic	L2a|LINE|L	-16861	NM_02443	66902	Mtap	1300019I2
chr16	31200577	31200700	intron (NM	CpG	686	NM_03013	78618	Acap2	4832442G1
chr18	39651077	39651525	Intergenic	CpG	-4402	NM_00817	14815	Nr3c1	GR|Grl-1|G
chr10	78036927	78037100	promoter-T	promoter-T	-232	NM_17375	216136	Ilvbl	5830463I2
chr15	63890477	63890925	intron (NM	intron (NM	1309	NM_14484	223601	Fam49b	0910001A0
chr15	91010327	91010400	intron (NM	intron (NM	11875	NM_01199	26874	Abcd2	ABC39|ALD
chr14	32484802	32485150	Intergenic	Intergenic	30733	NM_02529	26363	Btd	-
chr2	1.55E+08	1.55E+08	promoter-T	promoter-T	-571	NM_02603	67204	Eif2s2	2810026E1
chr9	1.23E+08	1.23E+08	promoter-T	promoter-T	142	NM_02541	66202	1110059G1	-
chr8	55776302	55776400	intron (NM	intron (NM	27680	NM_02822	244484	Wdr17	3010002I1
chr2	1.01E+08	1.01E+08	intron (NM	intron (NM	205	NM_02659	68170	B230118H0	NWC
chr1	91351127	91351275	promoter-T	promoter-T	-185	NM_17811	347722	Agap1	Centg2|Gg
chr1	1.65E+08	1.65E+08	promoter-T	promoter-T	-89	NM_00108	226562	Prrc2c	1810043M
chr3	95105477	95105625	intron (NM	intron (NM	5175	NM_00108	71790	Anxa9	1110003P1
chr14	35582302	35582675	Intergenic	CpG	-50834	NM_00816	14803	Grid1	GluD1|Glu
chr2	1.19E+08	1.19E+08	intron (NM	intron (NM	9119	NM_00114	214230	Pak6	4732456M
chr10	79422852	79423000	promoter-T	promoter-T	29	NM_17545	216156	Wdr18	2310012I1
chr16	62785952	62786025	intron (NM	Lx8|LINE|L	554	NM_17892	106338	Nsun3	6720484A0
chr11	1.01E+08	1.01E+08	exon (NM_	exon (NM_	987	NM_02789	71743	Coasy	1300003G0
chr1	1.8E+08	1.8E+08	Intergenic	Intergenic	-27582	NM_02707	69428	1700016C1	-
chr6	30351427	30351700	promoter-T	promoter-T	-346	NM_02974	76788	Klhdc10	2410127E1
chr7	51812377	51812500	Intergenic	Intergenic	-3103	NM_00128	22260	Nr1h2	AI194859|I
chr13	91680777	91680975	intron (NM	intron (NM	80174	NM_02418	66970	Ssbp2	1500004K0
chr7	1.07E+08	1.07E+08	intron (NM	intron (NM	19892	NM_17538	108937	Rnf169	2900057K0
chr1	65135677	65135825	Intergenic	Intergenic	-6887	NM_14476	12965	Crygb	Cryg-3|DG
chr8	48761202	48761300	promoter-T	promoter-T	-738	NM_02350	69260	Ing2	2810011M
chr13	99726352	99726525	Intergenic	PB1D10|SI	28082	NM_02706	69382	1700024P0	H2bl1|Sub
chr10	20225477	20225725	intron (NM	intron (NM	157976	NM_02793	71804	Mtfr2	2610016C2
chr1	1.73E+08	1.73E+08	Intergenic	RLTR9B|LT	-1129	NM_01018	14131	Fcgr3	CD16
chr8	99019427	99019650	Intergenic	Intergenic	-607273	NM_01032	14719	Got2	AL022787|
chr4	70196252	70196450	promoter-T	promoter-T	-389	NM_17269	230316	Megf9	4933405H1
chr1	92506352	92506450	intron (NM	intron (NM	6401	NM_13380	108679	Cops8	9430009J0
chr7	1.34E+08	1.34E+08	TTS (NM_0	TTS (NM_0	278	NM_03024	67278	Pagr1a	2900092E1
chr1	94106402	94106625	Intergenic	Intergenic	-61543	NM_20722	208727	Hdac4	4932408F1
chr7	1.09E+08	1.09E+08	intron (NM	intron (NM	6580	NM_00104	69710	Arap1	2410002L1
chr2	34681227	34681425	intron (NM	intron (NM	429	NM_00116	30050	Fbxw2	2700071L0
chr8	24318252	24318575	intron (NM	CpG	512	NM_01874	102247	Agpat6	AU041707
chr12	74419052	74419275	intron (NM	intron (NM	31328	NM_00103	625098	Slc38a6	AW322671

chr11	1.16E+08	1.16E+08	exon (NM_	exon (NM_	235	NM_02955	76265	Tsen54	0610034P0
chr17	15533177	15533500	non-coding	non-coding	128	NM_02420	67544	Fam120b	4932442K0
chr10	76982027	76982175	Intergenic	Intergenic	-3543	NM_13399	108707	Fam207a	1810008A1
chr1	1.22E+08	1.22E+08	Intergenic	Intergenic	21420	NM_00103	13167	Dbi	ACBD1|Acb
chr6	1.15E+08	1.15E+08	intron (NM	intron (NM	37704	NM_01114	19016	Pparg	Nr1c3|PPA
chr4	1.32E+08	1.32E+08	intron (NM	intron (NM	565	NM_02792	71787	Trnau1ap	1110007F0
chr3	1.33E+08	1.33E+08	intron (NM	intron (NM	23365	NM_02995	77669	Arhgef38	9130221D2
chr1	1.41E+08	1.41E+08	intron (NM	intron (NM	597	NM_02160	59125	Nek7	2810460C1
chr6	1.18E+08	1.18E+08	Intergenic	ERVL-int|L	-38502	NM_02752	70727	Rasgef1a	6330404M
chr1	63777002	63777400	promoter-T	promoter-T	2	NM_17242	75619	Fastkd2	2810421I2
chr12	60320002	60320325	exon (NM_	exon (NM_	307	NM_00103	70611	Fbxo33	5730501N2
chr2	33725027	33725125	intron (NM	B2_Mm2|S	18390	NM_17518	72543	Mvb12b	2610200O1
chr14	35487477	35487575	5' UTR (NM	5' UTR (NM	412	NM_00130	218914	Wapal	A530089A2
chr10	59080602	59080750	Intergenic	URR1B|DN	-1236	NM_00103	215999	Mcu	2010012O1
chr5	3418352	3418500	intron (NM	intron (NM	74114	NM_00987	12571	Cdk6	5830411I2
chr8	72682452	72682600	intron (NM	CpG	5849	NM_00104	17259	Mef2b	AI451606
chr1	1.09E+08	1.09E+08	intron (NM	intron (NM	10618	NM_02753	70750	Kdsr	6330410P1
chr2	1.26E+08	1.26E+08	exon (NM_	exon (NM_	2574	NM_02698	69185	Dtwd1	1810033A0
chr13	1.14E+08	1.14E+08	3' UTR (NM	3' UTR (NM	2841	NM_00108	218630	Ccno	C86987|Cc
chr2	4985177	4985375	promoter-T	promoter-T	-292	NM_18184	71648	Optn	4930441O0
chr2	32493577	32493725	Intergenic	Intergenic	-8464	NM_00793	13805	Eng	AI528660|A
chr15	73492177	73493175	Intergenic	MIR|SINE|	-37502	NM_00103	106068	Slc45a4	9330175B0
chr3	1.26E+08	1.26E+08	intron (NM	PB1D10|SI	40493	NM_17345	271970	Arsj	9330196J0
chr3	88373102	88373350	3' UTR (NM	3' UTR (NM	-10367	NM_02544	66256	Ssr2	1500032E0
chr19	56471377	56471825	promoter-T	promoter-T	-18	NM_00761	12369	Casp7	AI314680|
chr9	78296002	78296075	promoter-T	promoter-T	21	NM_02665	68291	Mto1	2310039H0
chr4	50971902	50971975	Intergenic	RMER17B|	-257612	NM_00116	74914	Cylc2	4930488P1
chr4	80652552	80652725	Intergenic	Intergenic	96048	NM_02682	52829	Lurap1l	1110029A0
chr12	1.19E+08	1.19E+08	Intergenic	Intergenic	-1258	NM_14604	217946	Cdca7l	BC006933|
chr11	28243452	28243900	Intergenic	Intergenic	240648	NM_00116	216613	Ccdc85a	-
chr1	1.3E+08	1.3E+08	Intergenic	Intergenic	-43558	NM_14550	226414	Dars	5730439G1
chr5	1.3E+08	1.3E+08	promoter-T	promoter-T	203	NM_13390	100678	Psph	AI480570|
chr18	38871402	38871725	Intergenic	MER58A|D	-110641	NM_01189	24066	Spry4	A030006O1
chr5	48763427	48763625	promoter-T	promoter-T	-105	NM_02575	66768	Pacrgl	4933428G0
chr6	23585377	23585575	intron (NM	(TAATG)n|	14829	NM_19825	386611	Rnf133	Greul2
chr13	89515802	89515975	Intergenic	Intergenic	-164353	NM_01350	12950	Hapln1	BB099155|
chr14	65052977	65053225	intron (NM	intron (NM	6762	NM_00125	110265	Msra	2310045J2
chr8	1.14E+08	1.14E+08	intron (NM	ID|SINE|ID	1934	NM_14621	234736	Rfwd3	BC027246
chr3	96067202	96067275	promoter-T	promoter-T	2	NM_03359	97122	Hist2h4	H4|X04652
chr7	52071777	52072100	promoter-T	promoter-T	90	NR_03313	107503	Atf5	AFTA|Atf7
chr17	46094952	46095275	Intergenic	Intergenic	66999	NM_00111	22339	Vegfa	Vegf|Vpf
chr8	96739252	96739425	intron (NM	intron (NM	837	NM_17241	71805	Nup93	2410008G0
chr19	53967577	53967800	intron (NM	intron (NM	967	NM_01105	18569	Pdcd4	D19Ucla1|
chr6	1.45E+08	1.45E+08	Intergenic	Intergenic	117244	NM_00102	12035	Bcat1	BCATc|Eca
chr17	27771127	27772650	intron (NM	CpG	299	NM_02596	67097	Rps10	2210402A0
chr14	62208627	62208975	Intergenic	Intergenic	-8262	NM_00116	66597	Trim13	3110001L1
chr12	1.07E+08	1.07E+08	promoter-T	promoter-T	14	NM_01111	18789	Papola	Pap|PapIII

chr10	57694677	57694825	exon (NM_	exon (NM_	672	NM_00108	664783	Dux	AW822073
chr6	1E+08	1E+08	promoter-T	promoter-T	-699	NM_01974	56353	Rybp	2410018J2
chr15	78859002	78859100	5' UTR (NM	5' UTR (NM	409	NM_00819	14958	H1f0	D130017D
chr5	1.08E+08	1.08E+08	intron (NM	intron (NM	-23876	NM_00126	14581	Gfi1	AW495828
chr1	1.82E+08	1.82E+08	Intergenic	L1MEb|LIN	-7245	NM_00112	19165	Psen2	ALG-3|Ad4
chr3	51505002	51505150	intron (NM	intron (NM	39961	NM_17499	211666	Mgst2	GST2|MGS
chr11	1.03E+08	1.03E+08	Intergenic	Intergenic	-6860	NM_18303	353047	Plekhm1	AP162|B2|
chr10	31328877	31328950	exon (NM_	exon (NM_	618	NM_00114	268291	Rnf217	AU016819
chr13	1.13E+08	1.13E+08	Intergenic	Intergenic	-186433	NM_00116	77318	Ankrd55	C030011J0
chr9	37050427	37050650	intron (NM	intron (NM	12785	NM_02025	56857	Slc37a2	G3PP|Slc3
chr9	70390952	70391200	intron (NM	CpG	399	NM_02569	66660	Sltm	5730455C
chr19	21728127	21728200	promoter-T	promoter-T	364	NM_14609	226016	Abhd17b	5730446C1
chr13	62231302	62231425	5' UTR (NM	5' UTR (NM	113	NM_00103	630579	Zfp808	EG630579|
chr1	58450077	58450350	intron (NM	CpG	233	NM_02582	66882	Bzw1	1200015E1
chr2	1.04E+08	1.04E+08	exon (NM_	exon (NM_	222	NM_00111	53872	Caprin1	AL022980|
chr8	1.08E+08	1.08E+08	intron (NM	intron (NM	1254	NM_01364	19171	Psmb10	Mecl-1|Me
chr9	1.1E+08	1.1E+08	Intergenic	Intergenic	-37078	NM_00101	382106	Fbxw24	EG382106|
chr8	3648227	3648550	Intergenic	Intergenic	-7382	NM_00120	57264	Retn	ADSF|Fizz3
chr5	65255927	65256075	Intergenic	Intergenic	61239	NM_00845	16599	Klf3	9930027G
chr6	16848452	16848825	promoter-T	promoter-T	-197	NM_03119	21426	Tfec	BB107417|
chr14	56381627	56382675	TTS (NM_0	TTS (NM_0	1886	NM_02049	57260	Ltb4r2	5830462O
chr9	20951202	20951350	intron (NM	intron (NM	3074	NM_01674	12539	Cdc37	p50|p50Cc
chr3	88987052	88987200	promoter-T	promoter-T	-21	NM_00101	68521	Fam189b	1110013L0
chr4	46648852	46649125	intron (NM	intron (NM	14083	NM_19866	381605	Tbc1d2	A630005A
chr9	45831727	45832000	intron (NM	L1ME4a|LI	10960	NM_02749	70661	Sik3	5730525O2
chr19	8894752	8894900	exon (NM_	exon (NM_	935	NM_00108	68693	Hnrnpul2	1110031M
chr10	33671152	33671275	promoter-T	promoter-T	-195	NM_02828	72580	Zufsp	2700019D
chr3	86560552	86560775	intron (NM	intron (NM	164143	NM_00119	70762	Dclk2	6330415M
chr8	47296152	47296325	exon (NM_	exon (NM_	125	NM_00745	11739	Slc25a4	AU019225
chr7	1.35E+08	1.35E+08	promoter-T	promoter-T	-386	NM_02688	68961	Phkg2	1500017I0
chr3	87906677	87906750	intron (NM	intron (NM	20740	NM_00103	404710	Iqgap3	AI593484|
chr3	60306977	60307100	intron (NM	intron (NM	1864	NM_00125	56758	Mbnl1	Mbnl|mKIA
chr12	16882027	16882400	Intergenic	Intergenic	-19571	NM_00907	19878	Rock2	B230113H
chr4	1.19E+08	1.19E+08	promoter-T	promoter-T	-395	NM_01173	22608	Ybx1	1700102N
chr1	1.95E+08	1.95E+08	intron (NM	intron (NM	8489	NM_00127	16780	Lamb3	-
chr19	37609827	37609900	Intergenic	Intergenic	-15045	NM_17535	107371	Exoc6	4833405E
chr18	34683852	34684175	Intergenic	Intergenic	-17536	NM_14608	225358	Fam13b	2610024E2
chr11	1.04E+08	1.04E+08	intron (NM	intron (NM	1767	NM_00108	76719	Kansl1	1700081L1
chr7	35903077	35903150	Intergenic	CpG	-1199	NM_00128	12606	Cebpa	C/ebpalpha
chr8	47702102	47702175	promoter-T	promoter-T	416	NM_00100	408022	Primpol	BC065112|
chr8	1.26E+08	1.26E+08	Intergenic	(CAGAGA)r	14734	NM_13395	69581	Rhou	2310026M
chr4	43057552	43057625	intron (NR_	intron (NR_	1504	NR_04556	230088	Fam214b	B230312A2
chr15	38448752	38448875	5' UTR (NM	5' UTR (NM	208	NM_00110	54375	Azin1	1700085L0
chr9	1.03E+08	1.03E+08	exon (NM_	exon (NM_	933	NM_00113	321022	Cdv3	2510010F1
chr4	59796452	59796700	intron (NM	CpG	151	NM_00101	66209	Inip	1110054O
chr4	1.28E+08	1.28E+08	5' UTR (NM	5' UTR (NM	286	NM_00119	54383	Phc2	A3galt2|A
chr12	98396677	98397050	Intergenic	Intergenic	1100684	NM_00807	14420	Galc	2310068B

chr5	1.41E+08	1.41E+08	intron (NM	intron (NM	3581	NM_00849	16848	Lfng	AW061165
chr2	84650777	84650900	TTS (NM_0	TTS (NM_0	11853	NM_01994	56791	Ube2l6	2810489I2
chr18	77182777	77183050	promoter-T	promoter-T	59	NM_00103	76987	Hdhd2	0610039H1
chr18	55293727	55293875	Intergenic	Intergenic	-143967	NM_17575	269023	Zfp608	4932417D1
chr1	1.72E+08	1.72E+08	intron (NM	intron (NM	20590	NM_02752	70729	Nos1ap	6330408P1
chr11	96634577	96634925	intron (NM	intron (NM	4118	NM_02896	74479	Snx11	4933439F1
chr1	71668152	71668325	intron (NM	intron (NM	31570	NM_01023	14268	Fn1	E330027I0
chr17	29751802	29752050	exon (NM_	exon (NM_	150	NM_02141	58230	Rnf8	3830404E2
chr3	1.52E+08	1.52E+08	Intergenic	L2c|LINE|L	-6513	NM_17801	329777	Pigk	3000001O0
chr4	1.5E+08	1.5E+08	intron (NM	URR1B|DN	57645	NM_17377	242773	Slc45a1	C230078B2
chr11	59653252	59653475	promoter-T	promoter-T	-94	NM_01199	26572	Cops3	Csn3|Sgn3
chr9	1.08E+08	1.08E+08	intron (NM	intron (NM	373	NM_14622	235606	Apeh	-
chr11	87452627	87452850	intron (NM	CpG	22072	NM_00104	327992	Hsf5	Gm739|MI
chr9	1.11E+08	1.11E+08	intron (NM	intron (NM	823	NM_00116	71268	Lrrfip2	5133400F2
chr4	1.41E+08	1.41E+08	intron (NM	intron (NM	-31745	NM_00108	76166	Rsg1	6330545A0
chr17	35326127	35326275	promoter-T	promoter-T	-816	NM_01073	16988	Lst1	B144
chr13	23836327	23836525	TTS (NM_1	TTS (NM_1	964	NM_17565	319148	Hist1h3c	H3-143
chr2	26790677	26790800	intron (NM	intron (NM	1149	NM_19862	279029	Stkld1	Gm711|Sg
chr4	1.33E+08	1.33E+08	promoter-T	promoter-T	-496	NR_11097	52174	Tmem222	5730406H1
chr1	33560052	33560125	intron (NM	intron (NM	166551	NM_00892	19076	Prim2	AI323589
chr7	1.49E+08	1.49E+08	Intergenic	Intergenic	-9757	NM_00114	68038	Chid1	3110023E0
chr15	6519152	6519225	Intergenic	Intergenic	-10659	NM_00127	23880	Fyb	ADAP|B63
chr15	79521402	79521675	intron (NM	CpG	212	NM_01381	14904	Gtpbp1	AL022987|
chr4	1.47E+08	1.47E+08	promoter-T	promoter-T	-73	NM_00102	28010	Miip	AA553001|
chr7	19663202	19663300	intron (NM	intron (NM	1702	NM_01005	13401	Dmwd	DMR-N9|D
chr13	41009077	41009250	intron (NM	intron (NM	-3840	NM_13321	14538	Gcnt2	5330430K1
chr2	69723477	69723575	non-coding	non-coding	146	NM_02928	75422	Mettl5	2810410A0
chr3	89577077	89577775	promoter-T	promoter-T	-105	NM_02731	70093	Ube2q1	1110002C0
chr8	37596927	37597175	Intergenic	Intergenic	44806	NM_17695	319582	6430573F1	Kiaa1456
chr14	31248602	31248850	intron (NM	intron (NM	-44025	NM_13376	75901	Dcp1a	1110066A2
chr2	1.31E+08	1.31E+08	intron (NM	intron (NM	23894	NM_00973	11990	Atrn	AW558010
chr3	97723102	97723425	intron (NM	intron (NM	-10833	NM_00103	433632	Gm5544	EG433632
chr14	15893677	15893850	Intergenic	Intergenic	240434	NM_00119	674895	Nek10	Gm282
chr1	88524977	88525075	Intergenic	Intergenic	41351	NM_17297	26895	Cops7b	D1Wsu66e
chr6	85401527	85403075	promoter-T	promoter-T	-337	NR_02808	73327	Pradc1	1700040I0
chr2	66610527	66610875	intron (NM	intron (NM	12266	NM_00913	20272	Scn7a	1110034K0
chr8	89411977	89412050	Intergenic	Intergenic	-2856	NM_03056	80750	N4bp1	AI481586|
chr11	1.01E+08	1.01E+08	intron (NM	intron (NM	512	NM_21366	20848	Stat3	1110034C0
chr5	65726552	65726900	intron (NM	CpG	152	NM_01125	19687	Rfc1	140kDa|Al
chr3	1.27E+08	1.27E+08	intron (NM	intron (NM	682	NM_14513	211550	Tifa	T2bp
chr13	67033777	67033950	promoter-T	promoter-T	97	NM_00895	19210	Ptdss1	AU044268
chr11	77500677	77500775	intron (NM	CpG	1085	NM_00102	68564	Nufip2	1110001M
chr9	1.08E+08	1.08E+08	intron (NM	intron (NM	8243	NM_02894	74443	P4htm	4933406E2
chr4	1.09E+08	1.09E+08	intron (NM	intron (NM	326	NM_02745	70533	Btf3l4	4632412E0
chr2	1.32E+08	1.32E+08	intron (NM	CpG	112	NM_00127	19122	Prnp	AA960666|
chr17	4791977	4792100	Intergenic	Intergenic	-203036	NM_00108	239985	Arid1b	8030481M
chr6	71494177	71494250	intron (NM	CpG	365	NM_02578	66700	Chmp3	25.1|4921

chr2	27342927	27343100	Intergenic	Intergenic	-11820	NM_00111	67382	Brd3	2410084F2
chr14	1.06E+08	1.06E+08	Intergenic	Lx9|LINE|L	-83227	NM_19860	212085	Trim52	4921513BC
chr19	34952277	34952425	promoter-T	promoter-T	56	NM_02379	75735	Pank1	Pank|Pank
chr11	96836102	96836500	intron (NM	intron (NM	2701	NM_03022	78912	Sp2	4930480I1(
chr7	88994152	88994500	Intergenic	Intergenic	-9561	NR_11191(	107769	Tm6sf1	AI428514|
chr2	1.26E+08	1.26E+08	intron (NM	URR1B|DN	3315	NM_00127	14391	Gabpb1	BABPB2|E4
chr9	18096802	18096950	exon (NM_	exon (NM_	165	NM_02584	66917	Chordc1	1110001O(
chr5	66497227	66497425	intron (NM	intron (NM	-7908	NM_13906	245945	Rbm47	9530077J1
chr3	30993402	30993500	promoter-T	promoter-T	-529	NM_01138	20482	Skil	Skir|SnoN|
chr17	15853002	15853200	intron (NM	intron (NM	11170	NM_00769	12648	Chd1	4930525N2
chr12	80292352	80292500	intron (NM	intron (NM	380	NM_02155	17252	Rdh11	2610319N2
chr4	1.23E+08	1.23E+08	promoter-T	promoter-T	-416	NM_00850	16918	Mycl	AW536278
chr5	9161477	9161675	intron (NM	CpG	200	NM_00111	23857	Dmtf1	Dimp|Dmp
chr13	49061627	49061725	intron (NM	intron (NM	1521	NM_00103	218236	Fam120a	C9orf10|O:
chr7	31514052	31514125	TTS (NM_0	TTS (NM_0	465	NM_02721	69804	Tmem147	2010004E1
chr19	4962302	4962625	exon (NM_	exon (NM_	157	NM_02555	66419	Mrpl11	2410001PC
chr17	13202877	13203050	intron (NM	intron (NM	2258	NM_01367	20656	Sod2	MnSOD|Sc
chr11	1.07E+08	1.07E+08	intron (NR_	intron (NR_	7923	NM_00103	380732	Milr1	Allergin-1|
chr2	1.02E+08	1.02E+08	intron (NM	Charlie19a	31566	NM_17888	241576	Ldlrad3	6430500PC
chr1	1.08E+08	1.08E+08	Intergenic	Intergenic	-25095	NM_00112	227449	Zcchc2	9930114B2
chr16	77084377	77084650	intron (NM	intron (NM	70199	NM_01391	30940	Usp25	-
chr16	51967202	51967425	Intergenic	Intergenic	-64349	NM_00103	208650	Cblb	AI429560|/
chr8	26707327	26707450	intron (NM	intron (NM	571	NM_17301	270035	Letm2	6030453H1
chr6	1.34E+08	1.34E+08	intron (NM	intron (NM	576	NM_00796	14011	Etv6	AW123102
chr11	57917902	57917975	intron (NM	CpG	283	NM_02694	69125	Cnot8	1500015I0
chr2	1.68E+08	1.68E+08	Intergenic	Intergenic	-98864	NM_01120	19246	Ptpn1	PTP-1B|PT
chr11	32355477	32355625	intron (NM	CpG	179	NM_17378	327900	Ubtd2	4930571L2
chr16	57196677	57196825	intron (NM	L1_Rod|LII	-29306	NM_02317	52633	Nit2	1190017B1
chr1	1.68E+08	1.68E+08	Intergenic	Intergenic	-26963	NM_00103	226594	Rcsd1	A430105K1
chr18	73862902	73863125	intron (NM	CpG	382	NM_00854	17128	Smad4	AW743858
chr5	38894202	38894500	promoter-T	promoter-T	-960	NM_14555	117591	Slc2a9	Glut9|SLC2
chr9	57491577	57491650	intron (NM	intron (NM	1374	NM_00778	12988	Csk	AW212630
chr9	21216452	21216550	promoter-T	promoter-T	220	NM_02188	60507	Qtrt1	2610028E1
chr8	61184327	61184400	intron (NM	RMER5|LTI	-4770	NM_00117	1E+08	Gm15881	OTTMUSG(
chr9	44903302	44903450	intron (NM	intron (NM	16110	NM_00100	270152	Amica1	AMICA|Cre
chr12	70343277	70343450	intron (NM	intron (NM	544	NM_17825	271005	Klhdc1	-
chr5	1.09E+08	1.09E+08	intron (NM	intron (NM	1609	NM_17288	243197	Mfsd7a	4732482E2
chr13	70780277	70780475	Intergenic	Intergenic	-3864	NM_14483	218333	Ice1	C77245|ml
chr3	1.43E+08	1.43E+08	intron (NM	CpG	492	NM_17865	109333	Pkn2	6030436C2
chr8	86257152	86257275	intron (NM	RLTR40|LT	7997	NM_00116	26364	Cd97	AA409984|
chr3	50247777	50248000	promoter-T	promoter-T	-353	NM_01199	26570	Slc7a11	9930009M
chr8	11556627	11556800	5' UTR (NM	5' UTR (NM	647	NM_01191	26356	Ing1	2610028J2
chr15	80663127	80663800	intron (NM	intron (NM	34423	NM_17712	213988	Tnrc6b	2700090M
chr3	1.02E+08	1.02E+08	Intergenic	Intergenic	-24237	NM_00103	242126	Slc22a15	2610034P2
chr3	1.42E+08	1.42E+08	intron (NM	intron (NM	249	NM_01026	14469	Gbp2	-
chr7	38999902	39000100	Intergenic	Intergenic	13012	NM_02441	72287	Plekhf1	1810013PC
chr9	15104702	15104775	intron (NM	B3|SINE|B.	1313	NM_13373	70984	4931406CC	-

chr3	50652977	50653200	Intergenic	Intergenic	-375281	NM_00983	12457	Ccrn4l	AU043840
chr6	16785652	16785975	intron (NM	intron (NM	62628	NM_03119	21426	Tfec	BB107417|
chr6	86321102	86321400	promoter-T	promoter-T	-283	NM_02650	68011	Snrpg	2810024K1
chr1	94538502	94538575	promoter-T	promoter-T	-13	NR_02810	66915	Myeov2	1110002M
chr5	92747627	92747825	Intergenic	LTR33C|LT	-8676	NM_17271	231452	Sdad1	4931421J1
chr4	1.34E+08	1.34E+08	Intergenic	RMER20B|	-25971	NM_20723	230815	Man1c1	AI593348
chr8	98012377	98012600	5' UTR (NM	5' UTR (NM	232	NM_00997	13000	Csnk2a2	1110035J2
chr13	75962027	75962450	Intergenic	Intergenic	-15096	NM_05310	93692	Glrx	C86710|D1
chr5	1.23E+08	1.23E+08	promoter-T	promoter-T	98	NM_01980	56317	Anapc7	APC7|AW5
chr7	87572727	87572900	Intergenic	Lx7|LINE|L	-22496	NM_00108	18550	Furin	9130404I0
chr1	62770552	62770925	intron (NM	intron (NM	20847	NM_00107	18187	Nrp2	1110048P0
chr18	76404352	76404425	intron (NM	intron (NM	3033	NM_01075	17126	Smad2	7120426M
chr12	70473152	70473375	intron (NM	CpG	126	NM_00748	11845	Arf6	AI788669|
chr6	54668777	54668925	Intergenic	MIRb|SINE	37233	NM_00128	68235	Mturn	2410066E1
chr6	1.2E+08	1.2E+08	promoter-T	promoter-T	86	NR_04555	67200	Ccdc77	2400002C2
chr11	75993602	75993775	promoter-T	promoter-T	-556	NM_02666	68299	Vps53	2010002A0
chr7	1.34E+08	1.34E+08	Intergenic	Intergenic	-12589	NM_01195	26417	Mapk3	Erk-1|Erk1
chr18	46440027	46440350	intron (NM	CpG	316	NM_17262	225467	Pggt1b	2010207C1
chr4	83595652	83595725	Intergenic	MIR|SINE|	424239	NM_00108	320226	Ccdc171	4930418J0
chr1	1.38E+08	1.38E+08	5' UTR (NM	5' UTR (NM	305	NM_00108	67886	Camsap2	1600013L1
chr10	10176777	10177125	intron (NM	intron (NM	15161	NM_00112	215772	Adgb	9130014G2
chr1	1.62E+08	1.62E+08	promoter-T	promoter-T	-228	NM_00978	12301	Cacybp	SIP
chr2	10274702	10274825	Intergenic	Intergenic	-17315	NM_00119	353282	Sfmbt2	D2Wsu23e
chr18	33160752	33160900	intron (NM	intron (NM	62131	NM_00979	12326	Camk4	A430110E2
chr6	88705252	88705325	intron (NM	intron (NM	30590	NM_00116	23945	Mgll	AA589436|
chr16	19203602	19204225	Intergenic	MMERVK10	82921	NM_14645	258443	Olfr164	MOR279-2
chr15	43114002	43114250	intron (NM	CpG	156	NM_00838	16341	Eif3e	48kDa|Eif3
chr1	42908002	42908700	intron (NM	CpG	273	NM_02351	69527	Mrps9	2310002A0
chr6	1.23E+08	1.23E+08	promoter-T	promoter-T	-12	NM_01994	56619	Clec4e	C86253|Cl
chr10	67743027	67743200	Intergenic	Intergenic	-1639	NM_02359	71371	Arid5b	4930580B1
chr2	94017777	94017950	Intergenic	Intergenic	-19797	NM_01965	56348	Hsd17b12	2610510O0
chr5	97224302	97224450	intron (NM	intron (NM	1972	NM_01347	11745	Anxa3	Anx3
chr14	78770152	78770225	Intergenic	L2a|LINE|L	-62338	NM_01161	21943	Tnfsf11	Ly109l|OD
chr7	52151652	52151800	exon (NM_	exon (NM_	277	NM_02737	70300	Fuz	2600013E0
chr14	65634152	65634250	intron (NM	intron (NM	65319	NM_00125	210925	Ints9	BC028953|
chr19	24611477	24611675	intron (NM	intron (NM	18741	NM_00884	18719	Pip5k1b	PI4P5K-I[b]
chr1	1.49E+08	1.49E+08	intron (NM	intron (NM	348321	NM_00114	215378	Brinp3	B830045N1
chr11	49838252	49838450	promoter-T	promoter-T	-482	NM_02154	59044	Rnf130	G1RZFP|G1
chr4	32887127	32887275	promoter-T	promoter-T	-33	NM_17536	108755	Lyrm2	2610208E0
chr19	8756527	8756750	3' UTR (NM	3' UTR (NM	18143	NM_00111	12669	Chrm1	Chrm-1|M1
chr14	55494977	55495100	3' UTR (NM	3' UTR (NM	1337	NM_00116	105651	Ppp1r3e	A630071A1
chr16	10784752	10785025	exon (NM_	exon (NM_	741	NM_00989	12703	Socs1	Cish1|Cish1
chr14	63768977	63769175	intron (NM	MER58A|D	27554	NM_01019	14137	Fdft1	SQS|SS
chr7	74904552	74904650	promoter-T	promoter-T	27	NM_18331	233335	Synm	4930412K2
chr9	91481452	91481700	Intergenic	Intergenic	217766	NM_00957	22774	Zic4	-
chr7	52689727	52690050	promoter-T	promoter-T	-54	NM_01130	20174	Ruvbl2	mp47|p47
chr1	65224477	65224775	intron (NM	intron (NM	1090	NM_01049	15926	Idh1	AI314845|

chr5	37183327	37183450	Intergenic	Intergenic	4605	NM_02624	67568	Mrfap1	9130413I2
chr5	77380377	77381000	promoter-T	promoter-T	-85	NM_17214	231327	Ppat	5730454C1
chr3	96838752	96838825	intron (NM	intron (NM	2463	NM_00812	14613	Gja5	5730555N1
chr10	1.26E+08	1.26E+08	promoter-T	promoter-T	-883	NM_00987	12567	Cdk4	Crk3
chr2	1.32E+08	1.32E+08	intron (NM	intron (NM	10398	NM_17544	215653	Rassf2	3830431H0
chr15	76077527	76077700	intron (NM	CpG	376	NM_02316	66168	Grina	1110025J1
chr8	1.07E+08	1.07E+08	exon (NM_	exon (NM_	219	NM_15358	97487	Cmtm4	Cklfsf4|D1
chr1	99666127	99666200	promoter-T	promoter-T	506	NM_17376	227399	Ppip5k2	AW555814
chr19	53466327	53466550	Intergenic	Intergenic	-1375	NM_17242	76479	Smndc1	2410004J2
chr14	73487077	73487550	Intergenic	Intergenic	-38392	NM_00116	70086	Cysltr2	2300001H0
chr13	35944452	35944550	intron (NM	intron (NM	53715	NM_14593	208366	Rpp40	D8Bwg126
chr15	81689827	81689950	Intergenic	Intergenic	-1132	NM_02050	57259	Tob2	2900090N2
chr19	6128077	6128275	promoter-T	promoter-T	39	NM_02691	69024	Snx15	1500032B0
chr13	41096552	41096800	5' UTR (NM	5' UTR (NM	297	NM_02655	68083	Pak1ip1	5830431I1
chr4	1.36E+08	1.36E+08	Intergenic	Intergenic	-6149	NM_01373	27224	Tceb3	110kDa|A
chr2	33324227	33324375	promoter-T	promoter-T	-249	NM_00102	71834	Zbtb43	1700010E0
chr8	97488827	97489125	Intergenic	Intergenic	-12033	NM_00119	14766	Gpr56	Cyt28|TM7
chr11	67402102	67402250	intron (NM	intron (NM	2174	NM_00110	14457	Gas7	AW124766
chr14	73177652	73178000	Intergenic	MLT1J|LTR	-68016	NM_20763	319448	Fndc3a	1700094E1
chr3	7503477	7503600	intron (NM	CpG	112	NM_17318	67306	Zc2hc1a	3110050N2
chr10	66559552	66559650	5' UTR (NM	5' UTR (NM	135	NM_00120	28193	Reep3	D10Ucla1
chr7	87530777	87530900	intron (NM	MIRm|SINE	1994	NM_01019	14159	Fes	AI586313|
chr18	75054052	75054225	Intergenic	Intergenic	66779	NM_01072	16891	Lipg	3110013K0
chr17	34523752	34523900	TTS (NM_0	TTS (NM_0	9749	NM_00111	1E+08	Btnl1	Btnl3|Gm3
chr5	44179627	44179925	Intergenic	Intergenic	-6388	NM_17872	242960	Fbxl5	Fbl4|Fir4
chr7	17497502	17497625	Intergenic	Intergenic	5724	NM_00896	19222	Ptgir	IP|PGI2
chr16	17209102	17209250	exon (NM_	exon (NM_	948	NM_00103	239731	Rimbp3	Gm1759|G
chr15	76060002	76060325	promoter-T	promoter-T	-236	NM_20138	18810	Plec	AA591047|
chr14	46365977	46366225	Intergenic	Intergenic	-88283	NM_00104	114874	Ddhd1	PA-PLA1
chr13	1.03E+08	1.03E+08	Intergenic	Intergenic	-84904	NM_00107	18708	Pik3r1	PI3K|p50al
chr15	99222702	99222800	promoter-T	promoter-T	-627	NM_02666	110213	Tmbim6	5031406P0
chr1	51562602	51562725	Intergenic	Intergenic	-27420	NM_02869	109019	Nabp1	4930434H0
chr9	72122252	72123000	promoter-T	promoter-T	-80	NM_14622	235469	Zfp280d	A930005F0
chr9	72380252	72380325	promoter-T	promoter-T	241	NM_00103	319758	Rfx7	2510005N2
chr11	1.01E+08	1.01E+08	intron (NM	CpG	429	NM_01128	19942	Rpl27	-
chr19	53112777	53112975	intron (NM	CpG	268	NM_13321	170750	Xpnpep1	D230045I0
chr1	1.66E+08	1.66E+08	promoter-T	promoter-T	-476	NM_05408	116914	Slc19a2	AV276020|
chr7	29135702	29135975	promoter-T	promoter-T	130	NM_01364	20055	Rps16	AA420385|
chr10	60837852	60837925	intron (NM	intron (NM	8383	NM_01375	27355	Pald1	MMPAL|Pa
chr4	45076827	45076925	intron (NM	intron (NM	20600	NM_00102	269529	Fbxo10	FBX10|Gm
chr9	41934852	41934950	Intergenic	Intergenic	-2529	NM_01143	20660	Sorl1	2900010L1
chr10	20866077	20866400	exon (NM_	exon (NM_	14552	NM_00119	17863	Myb	AI550390|
chr1	1.33E+08	1.33E+08	exon (NM_	exon (NM_	25354	NM_01070	16865	Eif2d	D1Ertd5e|
chr1	23407052	23407150	Intergenic	Intergenic	-17087	NM_00108	70155	Ogfrl1	2210417C1
chr13	52633752	52633875	Intergenic	Intergenic	-7608	NM_00102	68203	Diras2	2900052J1
chr13	6888802	6888950	Intergenic	RLTR45|LT	-240859	NM_00129	56421	Pfkp	1200015H2
chr1	1.06E+08	1.06E+08	Intergenic	B4|SINE|B	-1004495	NM_01180	23836	Cdh20	Cdh7

chr9	1.08E+08	1.08E+08	TTS (NM_0	TTS (NM_0	369	NM_02780	71472	Usp19	8430421I0
chr7	1.28E+08	1.28E+08	intron (NM	CpG	160	NM_02589	67003	Uqcrc2	1500004O0
chr9	48075077	48075225	Intergenic	Intergenic	73830	NM_03006	78252	Nxpe2	4432416J0
chr1	84718702	84719050	3' UTR (NM	3' UTR (NM	-26080	NM_15291	227325	Dner	A930026D1
chr10	69558102	69558175	Intergenic	Intergenic	-1731	NM_00111	76551	Ccdc6	2810012H1
chr4	1.55E+08	1.55E+08	intron (NM	intron (NM	40202	NM_00108	76866	Morn1	2900057D2
chr10	20671802	20672125	promoter-T	promoter-T	-390	NM_02620	52906	Ahi1	1700015F0
chr17	32670977	32671150	Intergenic	Intergenic	-2511	NM_02444	70101	Cyp4f16	2310021J0
chr13	47070427	47070500	Intergenic	RLTR12B|L	39756	NM_17534	105193	Nhlrc1	AI505271|
chr17	65958152	65958225	intron (NM	ID_B1|SINE	4707	NM_01393	30960	Vapa	33kDa|VAP
chr3	95021652	95021900	promoter-T	promoter-T	88	NM_17251	213054	Gabpb2	1810015F0
chr11	98885877	98886000	promoter-T	promoter-T	-435	NM_01162	21973	Top2a	Top-2
chr1	93391227	93391350	promoter-T	promoter-T	43	NM_02871	74019	Traf3ip1	3930402D0
chr15	96539202	96540825	Intergenic	CpG	-9884	NM_17512	67760	Slc38a2	5033402L1
chr14	47993777	47993950	intron (NM	CpG	328	NM_00114	16854	Lgals3	GBP|L-34|
chr7	1.16E+08	1.16E+08	Intergenic	Lx8|LINE|L	-13306	NM_02188	22141	Tub	rd5|tub
chr3	94914777	94914975	promoter-T	promoter-T	-88	NM_00933	21427	Vps72	Tcfl1|YL-1
chr1	99134277	99134475	Intergenic	Intergenic	-109496	NM_02894	74441	Slco6c1	4933404A1
chr9	1.18E+08	1.18E+08	promoter-T	promoter-T	-612	NM_02644	67899	Cmc1	2010110K1
chr15	91473277	91473400	Intergenic	Intergenic	-30317	NM_02573	66725	Lrrk2	4921513O2
chr9	1.03E+08	1.03E+08	intron (NM	intron (NM	8266	NM_00108	28135	Cep63	4921501M
chr6	56874227	56874325	promoter-T	promoter-T	-350	NM_02600	107569	Nt5c3	1600024P0
chr8	1.23E+08	1.23E+08	intron (NM	CpG	421	NM_13396	102193	Zdhhc7	AL024087|
chr10	62066127	62066250	Intergenic	URR1A|DN	-1142	NM_01955	56200	Ddx21	AI255159|
chr2	36126727	36127050	Intergenic	Intergenic	40942	NM_00896	19224	Ptgs1	COX1|Cox-
chr14	51550952	51551150	promoter-T	promoter-T	-527	NM_00103	219024	Tmem55b	BC039161
chr1	1.09E+08	1.09E+08	3' UTR (NM	3' UTR (NM	20058	NM_19802	241197	Serpinb10	9830131G0
chr12	56538027	56538375	Intergenic	Intergenic	38389	NM_01196	26443	Psma6	IOTA
chr6	92133602	92133725	intron (NM	CpG	354	NM_02557	64658	Mrps25	2810429N0
chr12	83240427	83240575	Intergenic	Intergenic	-30502	NM_00116	217692	Sipa1l1	4931426N1
chr11	62037527	62037825	Intergenic	MLT1A0|LT	-24810	NM_00741	11541	Adora2b	A2BAR|A2I
chr14	8912852	8913125	5' UTR (NM	5' UTR (NM	161	NM_02593	67053	Rpp14	2610511E0
chr6	1.29E+08	1.29E+08	Intergenic	4.5SRNA|s	27883	NM_00852	17059	Klrb1c	AI462337|
chr5	33994602	33994750	exon (NM_	exon (NM_	547	NM_00128	20492	Slbp	-
chr2	1.53E+08	1.53E+08	intron (NM	CpG	191	NM_00119	99311	Commd7	2310010I2
chr3	1.08E+08	1.08E+08	promoter-T	promoter-T	-224	NM_00116	14865	Gstm4	1110004G1
chr15	13641827	13642075	Intergenic	IAP-d-int|L	-538557	NM_00766	12563	Cdh6	K-cadherin
chr4	1.06E+08	1.06E+08	promoter-T	promoter-T	-505	NM_18322	329908	Usp24	2700066K0
chr8	1.22E+08	1.22E+08	intron (NM	B1F1|SINE	12891	NM_02807	72042	Cotl1	1810074P2
chr9	1.24E+08	1.24E+08	Intergenic	RSINE1|SIN	7841	NM_00987	12581	Cdkn2d	INK4d|p19
chr1	37808502	37808925	Intergenic	Intergenic	-32057	NM_02809	72097	2010300C0	-
chr3	40754777	40754950	intron (NM	CpG-9138	310	NM_00104	214048	Larp1b	1700108L2
chr3	1.16E+08	1.16E+08	Intergenic	Intergenic	-84578	NM_00790	13609	S1pr1	AI849002|
chr5	1.23E+08	1.23E+08	intron (NM	intron (NM	3843	NM_17542	109305	Orai1	D730049H0
chr5	1.19E+08	1.19E+08	Intergenic	Intergenic	131415	NM_00108	76792	2410131K1	-
chr11	33910077	33910375	Intergenic	Intergenic	-17033	NM_00119	70357	Kcnip1	KCHIP1|Kc
chr5	91460677	91460800	intron (NM	intron (NM	4195	NM_05308	71920	Epgn	2310069M

chr16	49854952	49855075	promoter-T	promoter-T	-754	NM_01058	16423	Cd47	9130415E2
chr4	66512952	66513325	Intergenic	Intergenic	24293	NM_02129	21898	Tlr4	Lps|Ly87|F
chr14	69573277	69573550	Intergenic	Intergenic	-73933	NM_00928	20855	Stc1	Stc
chr3	1.31E+08	1.31E+08	Intergenic	Intergenic	-97735	NM_00100	68436	Rpl34	1100001I2
chr10	89185027	89185200	intron (NM	intron (NM	9927	NM_02591	67019	Actr6	2010200J0
chr7	1.46E+08	1.46E+08	intron (NM	intron (NM	-11545	NM_00116	57740	Stk32c	PKE|Pkek|
chr11	69209702	69209925	promoter-T	promoter-T	520	NM_03008	78304	Naa38	1500034E0
chr15	76437702	76438025	exon (NM_	exon (NM_	158	NM_05319	94230	Cpsf1	-
chr5	4530827	4531125	Intergenic	Intergenic	227240	NM_02145	14362	Fzd1	AW227548
chr9	49811452	49811625	Intergenic	Intergenic	-204364	NM_01087	17967	Ncam1	CD56|E-NC
chr14	75595452	75595725	intron (NM	intron (NM	-46506	NM_01977	56373	Cpb2	1110032P0
chr15	78700177	78700275	Intergenic	Intergenic	-7394	NM_14592	106039	Gga1	4930406E1
chr19	44221027	44221125	promoter-T	promoter-T	-140	NM_02860	73689	Bloc1s2	2410089B1
chrX	1.04E+08	1.04E+08	intron (NM	intron (NM	792	NM_00128	58861	Cysltr1	BB147369|
chr9	72907152	72907250	intron (NM	intron (NM	-4754	NM_00130	11891	Rab27a	2210402C0
chr5	1.3E+08	1.3E+08	intron (NM	intron (NM	17773	NM_02882	74222	14-Sep	1700016K1
chr1	1.07E+08	1.07E+08	Intergenic	Intergenic	186311	NM_17877	320311	Rnf152	A930029B0
chr5	1.21E+08	1.21E+08	intron (NM	intron (NM	9334	NM_14585	252972	Tpcn1	5730403B0
chr11	6167852	6168025	promoter-T	promoter-T	-206	NM_02653	52513	Ddx56	2600001H0
chr8	1.29E+08	1.29E+08	Intergenic	Intergenic	138358	NM_02421	67952	Tomm20	1810060K0
chr2	68251152	68251225	intron (NM	L4|LINE|RT	58850	NM_01686	53416	Stk39	AW227544
chr5	3802802	3803025	promoter-T	promoter-T	211	NM_00128	70797	Ankib1	2310061P2
chr4	1.32E+08	1.32E+08	intron (NM	CpG	297	NM_13388	100226	Stx12	AI850350|
chr8	73905577	73905750	5' UTR (NM	5' UTR (NM	188	NM_01015	13864	Nr2f6	AV090102|
chr11	1.21E+08	1.21E+08	exon (NM_	exon (NM_	334	NM_13905	104318	Csnk1d	1200006A0
chr10	55264402	55264675	Intergenic	RMER16-in	-562185	NM_00116	73390	Msl3l2	1700060H1
chr1	1.95E+08	1.95E+08	intron (NM	CpG	294	NM_14541	215193	Diexf	AA408296|
chr15	84921377	84921450	exon (NM_	exon (NM_	40974	NM_08047	140557	Smc1b	SMC-1B|SN
chr1	58776877	58777150	intron (NM	intron (NM	6883	NM_20765	12633	Cflar	2310024N1
chr7	31787552	31787650	Intergenic	Intergenic	20661	NM_00124	76415	Fam187b	1700020B0
chr8	1.17E+08	1.17E+08	intron (NM	intron (NM	152399	NM_01957	80707	Wwox	5330426P0
chr1	72258977	72259075	promoter-T	promoter-T	-145	NM_00100	381269	Mreg	Gm974|W
chr8	1.26E+08	1.26E+08	promoter-T	promoter-T	-562	NM_01692	14087	Fanca	AW208693
chr7	28023727	28023800	intron (NM	intron (NM	5731	NM_13377	76889	Adck4	0610012P1
chr7	16507227	16507350	intron (NM	intron (NM	432	NM_00915	20364	Sepw1	selW
chr12	1.02E+08	1.02E+08	Intergenic	Intergenic	-4100	NM_15358	73086	Rps6ka5	3110005L1
chr4	1.55E+08	1.55E+08	5' UTR (NM	5' UTR (NM	302	NM_02689	68991	Ssu72	1190002E2
chr4	1.49E+08	1.49E+08	promoter-T	promoter-T	69	NM_20768	16561	Kif1b	A530096N0
chr17	66084652	66084725	intron (NM	L1MA6|LIN	37404	NM_13368	106572	Rab31	1700093E0
chr7	1.49E+08	1.49E+08	promoter-T	promoter-T	378	NM_05308	64540	Tspan4	AI325509|
chr16	13109952	13110150	exon (NM_	exon (NM_	222	NM_01576	50505	Ercc4	AI606920|
chr8	74967702	74968025	promoter-T	promoter-T	84	NM_02817	72254	1700030K0	-
chr5	1.15E+08	1.15E+08	promoter-T	promoter-T	-545	NM_02901	74585	Sppl3	4833416I0
chr19	41419927	41420075	intron (NM	intron (NM	39559	NM_03137	83490	Pik3ap1	1810044J0
chrX	42268227	42268400	Intergenic	Intergenic	-124939	NM_17873	245404	Dcaf12l1	9330180J2
chr12	78249177	78249475	Intergenic	MIR3|SINE	-89765	NM_00125	53618	Fut8	-
chr8	1.11E+08	1.11E+08	Intergenic	Intergenic	-20756	NM_00749	11906	Zfhx3	A230102L0

chr17	71901427	71901600	intron (NM	CpG	112	NM_02925	70891	Spdya	4921517J0
chr11	29447577	29447775	promoter-T	promoter-T	-274	NM_02850	73324	1700034F0	Clhc1
chr2	1.65E+08	1.65E+08	Intergenic	MIR|SINE|	-1218	NM_01359	17395	Mmp9	AW743869
chr8	91159852	91160050	promoter-T	promoter-T	76	NM_02784	71607	Snx20	9130017C1
chr3	1.44E+08	1.44E+08	promoter-T	promoter-T	260	NM_05310	93684	15-Sep	9430015P0
chr8	1.18E+08	1.18E+08	intron (NM	intron (NM	431881	NM_00102	17132	Maf	2810401A2
chr7	1.34E+08	1.34E+08	promoter-T	promoter-T	-227	NM_17736	244216	Zfp771	G630024C0
chr14	79915627	79915725	intron (NM	intron (NM	195	NM_00128	13709	Elf1	Elf-1|Sts1|
chr11	3231877	3232325	intron (NM	CpG-1647	1367	NM_17814	216505	Pik3ip1	1500004A0
chr6	1.25E+08	1.25E+08	intron (NM	intron (NM	567	NM_00788	13498	Atn1	Atr1|Drpla
chr17	34136627	34136975	exon (NM_	exon (NM_	477	NM_00100	14972	H2-K1	H-2K|H-2K
chr1	4775552	4775875	promoter-T	promoter-T	94	NR_03353	27395	Mrpl15	HSPC145|M
chr1	1.81E+08	1.81E+08	intron (NM	intron (NM	236695	NM_00116	269152	Kif26b	4832420M
chr15	83957527	83957650	Intergenic	Intergenic	-3983	NM_02942	75772	Pnpla5	4833426H1
chr18	23908277	23908425	Intergenic	Intergenic	-2483	NM_00116	212307	Mapre2	AI314113|
chr1	1.83E+08	1.83E+08	promoter-T	promoter-T	-461	NM_13322	170760	Acbd3	60kDa|843
chr8	1.04E+08	1.04E+08	Intergenic	RMER19C|	1622323	NM_00986	12552	Cdh11	Cad11
chr18	47436477	47436575	intron (NM	intron (NM	91996	NM_01874	20358	Sema6a	9330158E0
chr15	76540952	76541175	promoter-T	promoter-T	-74	NM_05821	79456	Recql4	RecQ4
chr12	31756152	31756250	Intergenic	Intergenic	-2632	NM_02782	104943	Fam110c	9030611O1
chr4	1.37E+08	1.37E+08	intron (NM	intron (NM	-31178	NM_02956	110351	Rap1gap	1300019I1
chr5	1.4E+08	1.4E+08	Intergenic	ID_B1|SINE	-3028	NM_00125	77053	Sun1	4632417G1
chr19	3931852	3931950	Intergenic	Intergenic	-2185	NM_02631	67689	Aldh3b1	1700001N1
chr5	32873752	32873950	Intergenic	PB1D9|SIN	-39693	NM_00120	22612	Yes1	Yes|p61-Ye
chr12	8214827	8215100	promoter-T	promoter-T	30	NM_00116	68832	1110057K0	LDAH
chr5	46266827	46267100	Intergenic	Intergenic	-18184	NM_00116	209707	Lcorl	Mlr1
chr15	1E+08	1E+08	intron (NM	intron (NM	9544	NM_15340	207785	Csrnp2	CSRNP-2|C
chr14	21330477	21330700	intron (NM	intron (NM	-14465	NM_02910	74843	Mss51	4833444M
chr10	62878502	62878950	Intergenic	Intergenic	-14120	NM_17761	216033	Ctnna3	4930429L0
chr19	4625702	4625875	promoter-T	promoter-T	-171	NM_02313	19671	Rce1	D19Ertd28
chr11	95651027	95651325	Intergenic	Intergenic	-34638	NM_15310	237928	Phospho1	D11Moh36
chr1	1.45E+08	1.45E+08	Intergenic	MER89|LTI	-174426	NM_02002	26878	B3galt2	-
chr4	9038277	9038375	Intergenic	Intergenic	-158138	NM_02894	74438	Clvs1	4933402J2
chr8	11403777	11404150	intron (NM	intron (NM	74536	NM_01122	19332	Rab20	AA536966|
chr19	3334127	3334250	intron (NM	intron (NM	10887	NM_01349	12894	Cpt1a	C730027G0
chr14	28164602	28164700	intron (NM	intron (NM	-9924	NM_00128	71704	Arhgef3	1200004I2
chr9	60592202	60592425	Intergenic	RCHARR1|	31385	NM_02623	67557	Larp6	5430431G0
chr14	60378627	60378825	intron (NM	intron (NM	134608	NM_14546	219134	Shisa2	9430059P2
chr9	62981477	62981850	Intergenic	Intergenic	13124	NM_17244	207667	Skor1	AV273001|
chr5	1.47E+08	1.47E+08	Intergenic	L1MB3|LIN	-91436	NM_00101	14738	Gpr12	Gpcr01|Gp
chr13	64376327	64376625	promoter-T	promoter-T	-180	NM_17258	218294	Cdc14b	2810432N1
chr11	83755577	83755800	promoter-T	promoter-T	96	NM_03009	78394	Ddx52	2700029C0
chr7	16931402	16931550	intron (NM	intron (NM	36544	NM_13323	170770	Bbc3	PUMA|PUI
chr2	24774352	24774450	intron (NM	CpG	728	NM_17254	77683	Ehmt1	9230102N1
chr5	1.23E+08	1.23E+08	Intergenic	Intergenic	-7817	NM_18330	330188	Ccdc63	4921511C1
chr2	1.57E+08	1.57E+08	intron (NM	intron (NM	-30315	NM_02585	66934	Dsn1	1700022L0
chr2	1.64E+08	1.64E+08	intron (NM	intron (NM	14130	NM_01875	54401	Ywhab	1300003C1

chr12	80714877	80715025	intron (NM	intron (NM	316613	NM_00125	19363	Rad51b	AI553500|
chr15	74551927	74552100	exon (NM_	exon (NM_	349	NM_19860	223626	Them6	4930572J0
chr4	65592027	65592250	intron (NM	intron (NM	326118	NM_00116	69807	Trim32	1810045E1
chr5	76611377	76611750	Intergenic	Intergenic	-1342	NM_01162	21982	Tmem165	AV026557|
chr13	1.01E+08	1.01E+08	promoter-T	promoter-T	-97	NM_00128	19356	Rad17	MmRad24
chr5	1.37E+08	1.37E+08	intron (NM	L1MA5|LIN	49176	NM_02447	140709	Col26a1	9430032K2
chr13	43621027	43621350	Intergenic	Intergenic	34372	NM_00108	76137	Mcur1	6230416A0
chr4	1.36E+08	1.36E+08	intron (NM	CpG	731	NM_00127	74326	Hnrnpr	2610003J0
chr5	1.36E+08	1.36E+08	promoter-T	promoter-T	-918	NM_01376	27368	Tbl2	C76179|W
chr19	45738702	45738800	Intergenic	Intergenic	-4068	NM_01390	30838	Fbxw4	Dac|Fbw4|
chr2	1.18E+08	1.18E+08	Intergenic	Intergenic	-115032	NM_01158	21825	Thbs1	TSP-1|TSP1
chr11	1.2E+08	1.2E+08	promoter-T	promoter-T	653	NM_01156	21681	Alyref	ALY|REF1|
chrX	35805452	35805650	intron (NM	intron (NM	4086	NM_01068	16784	Lamp2	CD107b|LG
chr16	22144702	22144775	intron (NM	intron (NM	18634	NM_18302	319765	Igf2bp2	C330012H0
chr17	57171327	57171575	promoter-T	promoter-T	-521	NM_01061	16549	Khsrp	6330409F2
chr5	1.46E+08	1.46E+08	promoter-T	promoter-T	-185	NM_00108	100683	Trrap	AI481500
chr15	88692802	88693175	5' UTR (NM	5' UTR (NM	364	NM_14547	223775	Pim3	BC026639|
chrX	83021152	83021850	intron (NM	intron (NM	657	NM_00129	14933	Gyk	D930012N1
chr6	1.43E+08	1.43E+08	intron (NM	intron (NM	27421	NM_00128	74741	C2cd5	5730419I09
chr1	1.68E+08	1.68E+08	Intergenic	Intergenic	-8291	NM_00108	68481	Mpzl1	1110007A1
chr1	1.73E+08	1.73E+08	Intergenic	Intergenic	58663	NM_17706	320078	Olfml2b	1110018N0
chr9	7567377	7567675	exon (NM_	exon (NM_	-3932	NM_00103	234911	Mmp27	Gm180
chr7	1.43E+08	1.43E+08	Intergenic	MLT1H2|LT	-90501	NM_00108	17345	Mki67	D630048A1
chr19	53977177	53977475	promoter-T	promoter-T	-515	NM_00116	18569	Pdcd4	D19Ucla1|
chr8	1.13E+08	1.13E+08	intron (NM	CpG	363	NM_14621	234729	Vac14	AA959718|
chr2	1.57E+08	1.57E+08	promoter-T	promoter-T	-17	NM_00129	228839	Tgif2	4921501K2
chr17	46419677	46420000	intron (NM	intron (NM	137	NM_00125	74094	Tjap1	0610041D1
chr13	31573477	31573575	Intergenic	Intergenic	-76513	NM_00823	15220	Foxq1	HFH-1|Hfh
chr13	21641427	21641725	Intergenic	Lx7|LINE|L	6313	NM_20757	404335	Olfr1535	MOR256-3
chr1	64347902	64348225	Intergenic	Intergenic	-180100	NM_03356	93691	Klf7	9830124P0
chr9	1.04E+08	1.04E+08	intron (NM	intron (NM	6176	NM_01980	56318	Acpp	5'-NT|A030
chr11	1.16E+08	1.16E+08	promoter-T	promoter-T	95	NM_02725	217340	Rnf157	2610036E2
chr11	66860702	66860950	intron (NM	(GA)n|Simp	5294	NM_02551	66358	Adprm	2310004I24
chr5	3380527	3380600	intron (NM	intron (NM	36251	NM_00987	12571	Cdk6	5830411I20
chr7	1.51E+08	1.51E+08	intron (NM	intron (NM	142	NM_02368	79202	Tnfrsf22	2810028K0
chr11	1.14E+08	1.14E+08	Intergenic	Charlie8|D	-14484	NM_00116	56699	Cdc42ep4	1500041M
chr1	1.72E+08	1.72E+08	intron (NM	intron (NM	5208	NM_01200	26904	Sh2d1b1	EAT-2|EAT-
chr4	1.02E+08	1.02E+08	intron (NM	MER5A1|D	26286	NM_00128	73094	Sgip1	3110007P0
chr6	50195027	50195250	intron (NM	intron (NM	16630	NM_01876	54722	Dfna5	2310037D0
chr18	32070677	32070800	exon (NM_	exon (NM_	819	NM_02600	67158	Sft2d3	2610206C2
chr2	24832652	24832800	intron (NM	intron (NM	1173	NM_14625	241274	Pnpla7	BC027342|
chr2	62584352	62584800	intron (NM	intron (NM	82192	NM_14552	227960	Gca	5133401E0
chr4	53840052	53840200	intron (NM	Charlie1b|	1209	NM_02805	52076	Tmem38b	1600017F2
chr9	37464577	37464975	5' UTR (NM	5' UTR (NM	121	NM_02528	21376	Tbrg1	AA408552|
chr1	74472527	74472625	intron (NM	intron (NM	16618	NM_00950	22349	Vil1	Vil
chr4	98049802	98049900	exon (NM_	exon (NM_	105	NM_05315	94043	Tm2d1	2310026L1
chr2	61221852	61222125	Intergenic	Intergenic	-194655	NM_01152	21353	Tank	C86182|E4

chr1	1.73E+08	1.73E+08	Intergenic	Intergenic	22639	NM_00107	14130	Fcgr2b	AI528646|
chrX	1.06E+08	1.06E+08	Intergenic	Intergenic	-11457	NM_00103	622976	Gm6377	EG622976
chr7	28798977	28799400	promoter-T	promoter-T	-54	NM_00125	1E+08	Zfp850	C130069I0
chr15	1.01E+08	1.01E+08	Intergenic	Intergenic	-11440	NM_02153	59033	Slc4a8	AW493845
chr17	12556827	12556900	Intergenic	Intergenic	-14612	NM_00887	18815	Plg	AI649309|
chr1	92748627	92748775	Intergenic	Intergenic	-8153	NM_00124	12835	Col6a3	AI507288|
chr10	90645077	90645150	Intergenic	Intergenic	-10749	NM_00108	21917	Tmpo	5630400D2
chr4	1.45E+08	1.45E+08	Intergenic	Intergenic	67586	NM_01130	20148	Dhrs3	Rsdr1|retS
chr12	80107702	80107875	intron (NM	CpG	476	NM_17874	268567	Tmem229b	6330442E1
chr2	1.56E+08	1.56E+08	promoter-T	promoter-T	-117	NR_024487	56046	Uqcc1	2310079L1
chr4	98797977	98798225	Intergenic	Intergenic	-10495	NM_02608	67299	Dock7	3110056M
chr6	1.25E+08	1.25E+08	promoter-T	promoter-T	-75	NM_01073	17000	Ltbr	AI256028|
chr2	59914677	59915300	intron (NM	intron (NM	48809	NM_00100	407823	Baz2b	5830435C1
chr11	69647552	69647700	exon (NM_	exon (NM_	725	NM_19886	216856	Nlgn2	NL2
chr1	87859452	87859550	Intergenic	MIR|SINE|	-1871	NM_00103	227326	Gpr55	Gm218|Lp
chr11	1.2E+08	1.2E+08	promoter-T	promoter-T	-488	NM_01103	18453	P4hb	ERp59|PDI
chr5	1.38E+08	1.38E+08	Intergenic	Intergenic	-10594	NM_00959	11423	Ache	-
chr2	1.27E+08	1.27E+08	intron (NM	CpG	114	NM_02996	77721	Mrps5	1620401I1
chr15	96682527	96682700	Intergenic	Intergenic	-152484	NM_17512	67760	Slc38a2	5033402L1
chr15	92865052	92865150	Intergenic	Intergenic	240414	NM_00103	223827	Gxylt1	Glt8d3|Gm
chr19	7369652	7369850	5' UTR (NM	5' UTR (NM	186	NM_00108	13728	Mark2	EMK-1|Em
chr15	36371052	36371275	Intergenic	Intergenic	55383	NM_17513	68839	Ankrd46	1110054N0
chr1	37925627	37925825	Intergenic	MTEa|LTR|	-3325	NM_00103	623661	Lipt1	EG623661
chr3	85397502	85397575	intron (NM	intron (NM	19487	NM_14489	229487	Pet112	9430026F0
chr18	67934427	67934900	5' UTR (NM	5' UTR (NM	133	NM_00103	72124	Seh1l	2610007A1
chr14	66211252	66211375	intron (NR_	AT_rich|Lo	636	NM_00125	74195	Elp3	2610507P1
chr2	1.3E+08	1.3E+08	promoter-T	promoter-T	270	NM_20720	99045	Mrps26	AI648866|
chr3	53267602	53267850	promoter-T	promoter-T	-13	NM_17338	212127	Proser1	2810046L0
chr2	35344302	35344425	Intergenic	Intergenic	-27418	NM_00114	14594	Ggta1	AW108479
chr13	1.02E+08	1.02E+08	Intergenic	(GAA)n|Sir	-1619	NM_02288	69048	Slc30a5	1810010K0
chr4	1.55E+08	1.55E+08	Intergenic	Intergenic	-2957	NM_00116	14688	Gnb1	AA409223|
chr10	62348927	62349200	Intergenic	Intergenic	-6301	NM_00125	52463	Tet1	2510010B0
chr8	87230577	87230725	3' UTR (NM	3' UTR (NM	5295	NM_00853	17095	Lyl1	Lyl-1|bHLH
chr16	16576827	16576900	Intergenic	RMER30|D	-16574	NM_13923	224014	Fgd4	9030023J0
chr12	86814552	86814725	promoter-T	promoter-T	-213	NM_01023	14281	Fos	D12Rfj1|c-
chr10	1.11E+08	1.11E+08	Intergenic	Intergenic	23971	NM_00934	21664	Phlda1	DT1P1B11|
chr6	54016952	54017025	intron (NM	intron (NM	27062	NM_00116	69993	Chn2	1700026N2
chr15	79187852	79188250	exon (NM_	exon (NM_	9943	NM_01075	17133	Maff	-
chr3	1.08E+08	1.08E+08	promoter-T	promoter-T	-281	NM_00100	229715	Amigo1	Amigo|ali2
chr6	51479427	51479550	intron (NM	intron (NM	5586	NM_00112	71982	Snx10	2410004M
chr12	85749477	85749600	promoter-T	promoter-T	441	NM_00764	12499	Entpd5	AI196558|
chr5	1.38E+08	1.38E+08	5' UTR (NM	5' UTR (NM	356	NM_02875	74097	Pop7	0610037N1
chr6	91198927	91199025	intron (NM	intron (NM	36218	NM_00799	14115	Fbln2	5730577E1
chr16	36465577	36465975	Intergenic	Intergenic	-10298	NM_02528	20863	Stfa3	Stf3
chr11	44169802	44169925	Intergenic	L1MB2|LIN	-43702	NM_00835	16160	Il12b	Il-12b|Il-12
chr5	1.3E+08	1.3E+08	promoter-T	promoter-T	106	NM_00983	12466	Cct6a	CCT-zeta|C
chr12	73861577	73861925	promoter-T	promoter-T	-447	NM_00891	19042	Ppm1a	2310003C2

chr17	23877002	23877075	5' UTR (NM	5' UTR (NM	259	NM_02382	76498	Paqr4	1500004C1
chr1	60153702	60153875	intron (NM	intron (NM	963	NM_02131	57750	Wdr12	4933402C2
chr16	22618077	22618325	intron (NM	intron (NM	39103	NM_13865	110197	Dgkg	2900055E1
chr18	62348452	62348650	Intergenic	Intergenic	-8916	NM_00742	11555	Adrb2	Adrb-2|Ba
chr10	79514402	79514925	intron (NR_	intron (NR_	-1570	NM_00816	625249	Gpx4	GPx-4|GSH
chr9	1.22E+08	1.22E+08	Intergenic	ORR1D2|L	29809	NM_00116	215474	Sec22c	4932412K2
chr4	44976127	44976575	Intergenic	Intergenic	-17932	NM_08028	76238	Grhpr	1110059D0
chr2	1.68E+08	1.68E+08	promoter-T	promoter-T	629	NM_00116	69372	Mocs3	1700020H1
chr13	52731577	52731725	intron (NM	MamRep60	39400	NM_01151	20963	Syk	Sykb
chr19	28351277	28351425	intron (NM	intron (NM	-265695	NM_01126	19726	Rfx3	C230093O1
chr15	51823127	51823250	5' UTR (NM	5' UTR (NM	118	NM_00900	19357	Rad21	SCC1|mKIA
chr6	1.25E+08	1.25E+08	intron (NM	intron (NM	-3799	NM_00941	21991	Tpi1	AI255506|
chr1	1.64E+08	1.64E+08	promoter-T	promoter-T	-350	NM_14487	71449	Mettl13	5630401D2
chr11	1.19E+08	1.19E+08	intron (NM	intron (NM	2738	NM_00762	12418	Cbx4	MPc2|PC2
chr12	82856602	82857000	intron (NM	intron (NM	25356	NM_17739	338372	Map3k9	E130314H2
chr4	1.55E+08	1.55E+08	Intergenic	CpG	64440	NM_00108	76866	Morn1	2900057D2
chr13	32359227	32359425	intron (NM	intron (NM	71087	NM_14604	218138	Gmds	BC031788|
chr1	39953377	39953650	Intergenic	Intergenic	-4245	NM_00125	26921	Map4k4	9430080K1
chr9	1.24E+08	1.24E+08	intron (NM	CpG	412	NM_13914	102680	Slc6a20a	A730081N2
chr5	1.18E+08	1.18E+08	intron (NM	intron (NM	279	NM_18330	330177	Taok3	2900006A0
chr1	1.73E+08	1.73E+08	Intergenic	tRNA-Val-G	-10166	NM_00108	75472	1700009P1	AV282292|
chr3	1.57E+08	1.57E+08	intron (NM	intron (NM	9845	NM_01119	19218	Ptger3	EP3|Pgere
chr2	34228452	34228700	Intergenic	CpG	-1011	NM_01676	18516	Pbx3	-
chr4	6381077	6381250	exon (NM_	exon (NM_	255	NM_01094	18201	Nsmaf	AA959567|
chr14	31682552	31682625	intron (NM	intron (NM	-9855	NM_18139	66175	Mustn1	1110028G0
chr18	35020827	35021025	promoter-T	promoter-T	65	NM_00791	13653	Egr1	A530045N1
chr15	88958877	88958950	non-coding	non-coding	217	NR_028448	170787	Hdac10	AW548891
chr14	31425652	31425775	intron (NM	intron (NM	13681	NM_01110	18753	Prkcd	AI385711|
chr11	1.02E+08	1.02E+08	intron (NM	intron (NM	432	NM_17294	268490	Lsm12	1110032E1
chr7	1.28E+08	1.28E+08	intron (NM	intron (NM	21428	NM_02895	74466	4933427G1	-
chr4	44769502	44769650	intron (NM	CpG	145	NM_13859	319885	Zcchc7	4930572I0
chr11	1.18E+08	1.18E+08	5' UTR (NM	5' UTR (NM	220	NM_00103	72344	Usp36	2700002L0
chr2	62502452	62502575	exon (NM_	exon (NM_	129	NM_14552	227960	Gca	5133401E0
chr17	15637052	15637225	intron (NM	CpG	286	NM_01368	21374	Tbp	GTF2D1|G
chr10	98360677	98361175	Intergenic	Intergenic	-16860	NM_02648	67972	Atp2b1	2810442I2
chr1	1.33E+08	1.33E+08	intron (NM	intron (NM	2739	NM_01977	56489	Ikbke	AW558201
chr1	88255027	88255100	intron (NM	(A)n|Simpl	967	NM_01088	17975	Ncl	B530004O1
chr13	93962377	93962825	promoter-T	promoter-T	-363	NM_13390	100715	Papd4	8030446C2
chr3	27169202	27169600	Intergenic	Intergenic	-46598	NM_00942	22035	Tnfsf10	A330042I2
chr5	97378952	97379225	Intergenic	Intergenic	-47620	NM_08070	140780	Bmp2k	4933417M
chr10	7445777	7446000	promoter-T	promoter-T	90	NM_01183	23924	Katna1	-
chr12	70992752	70992850	intron (NM	intron (NM	-1291	NM_17862	73991	Atl1	4930435M
chr3	1.03E+08	1.03E+08	intron (NM	CpG	394	NM_14490	229663	Csde1	AA960392|
chr18	34918427	34918725	promoter-T	promoter-T	16	NM_17510	66306	Fam53c	2810012G0
chr2	34581352	34581450	intron (NM	AT_rich|Lo	29351	NM_02570	66691	Gapvd1	2010005B0
chr11	98803202	98803550	intron (NM	intron (NM	2351	NM_00117	19401	Rara	Nr1b1|RAF
chr14	26295777	26295950	intron (NM	intron (NM	17192	NM_18320	328365	Zmiz1	BC065120|

chr7	52823102	52823300	TTS (NM_0	TTS (NM_0	-2322	NM_00973	12036	Bcat2	Bcat-2|Eca
chr16	10838827	10838950	intron (NM	intron (NM	3736	NM_00116	223970	Rmi2	A630055G(
chr1	1.88E+08	1.88E+08	Intergenic	Intergenic	126558	NM_00936	21808	Tgfb2	BB105277|
chr7	26157452	26157800	Intergenic	Intergenic	-2883	NM_02821	72383	Cnfn	2210418J0
chr15	99870002	99870075	intron (NM	intron (NM	943	NM_00115	239667	Dip2b	4932422C2
chr12	91963152	91963350	3' UTR (NM	3' UTR (NM	13627	NM_01005	13371	Dio2	5DII|AI324
chr13	41582677	41582800	promoter-T	promoter-T	-9	NM_00111	18003	Nedd9	Cas-L|CasL
chr2	1.8E+08	1.8E+08	promoter-T	promoter-T	-681	NM_17555	23856	Dido1	6720461J1
chr8	24319077	24319175	promoter-T	promoter-T	-201	NM_01874	102247	Agpat6	AU041707
chr18	67439577	67439675	Intergenic	Intergenic	-9251	NM_05326	114663	Impa2	2210415D2
chr5	6949577	6949675	intron (NM	intron (NM	-354538	NM_02760	70920	4921511H(	-
chr3	1.06E+08	1.06E+08	intron (NM	CpG	386	NM_14554	109905	Rap1a	AI848598|(
chr12	1.14E+08	1.14E+08	promoter-T	promoter-T	-506	NM_00129	217893	Pacs2	6720425G1
chr3	40605027	40605275	intron (NR_	intron (NR_	1278	NR_04557(	20873	Plk4	1700028H2
chr8	74248927	74249075	intron (NR_	intron (NR_	579	NR_02826;	74015	Fcho1	3322402E1
chr2	69660502	69660750	5' UTR (NM	5' UTR (NM	199	NM_17778	277396	Klhl23	C130068N1
chr9	52004127	52004200	Intergenic	Intergenic	-27947	NM_00116	244871	Zc3h12c	A230108E0
chr4	3605552	3605675	intron (NM	CpG	345	NM_01074	17096	Lyn	AA407514|
chr1	7538452	7538600	Intergenic	MIR|SINE|	459525	NM_18302	319263	Pcmtd1	8430411F1
chr11	62871852	62872225	Intergenic	Intergenic	-3123	NM_02766	71062	Tekt3	4933407G0
chr10	82221727	82221925	intron (NM	intron (NM	5060	NM_01091	18045	Nfyb	AA985999|
chr2	72085402	72085550	intron (NM	intron (NM	-38218	NM_02305	65964	Zak	AV006891|
chr5	1.35E+08	1.35E+08	Intergenic	MIRc|SINE	-5368	NR_10438;	17969	Ncf1	NCF-47K|N
chr7	85998952	85999025	Intergenic	Intergenic	-6891	NM_02958	76375	Det1	2610034H2
chr13	18085102	18085350	promoter-T	promoter-T	-41	NM_02590	67008	Yae1d1	1600012F0
chr9	1.01E+08	1.01E+08	intron (NM	Charlie25|I	38290	NM_17214	235542	Ppp2r3a	3222402P1
chr1	16028677	16028750	Intergenic	Intergenic	54409	NM_00124	75799	4930444P1	-
chr7	3849852	3850075	promoter-T	promoter-T	-211	NM_00128	18729	Pira6	12M1|Pira
chr16	38563227	38563425	intron (NM	intron (NM	399	NM_02640	67846	Tmem39a	2610033C0
chr9	26806902	26807100	promoter-T	promoter-T	133	NM_02586	66948	Acad8	2310016C1
chr5	98263677	98264150	Intergenic	Intergenic	196068	NM_13373	71914	Antxr2	2310046B1
chrX	63558527	63558600	Intergenic	Lx8|LINE|L	-343930	NM_00116	245450	Slitrk2	A930040J0
chr16	17233752	17233825	5' UTR (NM	5' UTR (NM	108	NM_17892	58180	Hic2	AA409108|
chr11	75300352	75300425	5' UTR (NM	5' UTR (NM	109	NM_13865	192159	Prpf8	AU019467
chr7	91040752	91040850	exon (NM_	exon (NM_	291	NM_02340	67943	Mesdc2	2210015O1
chr2	1.19E+08	1.19E+08	intron (NM	CpG	407	NM_00103	67809	Rmdn3	1200015F2
chr2	1.53E+08	1.53E+08	Intergenic	Intergenic	-1640	NM_14553	228788	Ccm2l	-
chr8	80073677	80073900	intron (NM	(CAGAGA)r	492	NM_00108	102182	Prmt10	AI931714|I
chr9	66582802	66582950	intron (NM	L1_Mm|LII	21383	NM_17839	76459	Car12	2310047E0
chr1	1.74E+08	1.74E+08	intron (NM	intron (NM	15394	NM_17840	98660	Atp1a2	AW060654
chr17	33774477	33774875	promoter-T	promoter-T	-325	NM_00100	378460	Pram1	AY665714
chrX	71613852	71614125	promoter-T	promoter-T	-101	NM_14540	27643	Ubl4	DXHXS254I
chr8	63369252	63369525	intron (NM	CpG	140	NM_02829	72612	2700029M	C230006B2
chr1	1.73E+08	1.73E+08	5' UTR (NM	5' UTR (NM	225	NM_02538	66155	Ufc1	1110021H0
chr19	53385227	53385375	exon (NM_	exon (NM_	305	NM_00100	17859	Mxi1	ENSMUSG(
chr1	87587102	87587250	intron (NM	intron (NM	40551	NM_01367	20684	Sp100	A430075G1
chr7	50927477	50927550	intron (NM	intron (NM	112	NM_14558	233189	Ctu1	Atpbd3|BC

chr12	4599852	4600325	intron (NM	CpG	274	NM_00119	20403	Itsn2	AI327390|
chr9	96044127	96044400	intron (NM	intron (NM	24415	NM_17735	235533	Gk5	AV095337|
chr2	74444352	74444525	Intergenic	Intergenic	-27433	NM_02713	69605	Lnp	2310011O1
chr6	86977027	86977350	intron (NM	intron (NM	-15652	NM_01352	14583	Gfpt1	2810423A1
chr15	66335427	66335900	Intergenic	Intergenic	-3191	NM_01945	54562	Lrrc6	LRTP
chr2	30142002	30142125	promoter-T	promoter-T	110	NM_19830	227699	Nup188	BC025526|
chr6	1.23E+08	1.23E+08	intron (NM	L1ME3A|LI	4426	NM_01081	17474	Clec4d	Clecsf8|Mp
chr8	23170602	23170800	promoter-T	promoter-T	-155	NM_00751	11979	Atp7b	Atp7a|WN
chr11	1.16E+08	1.16E+08	promoter-T	promoter-T	64	NM_01685	53413	Exoc7	Exo70|sec7
chr5	1.41E+08	1.41E+08	Intergenic	Intergenic	-20225	NM_02152	59031	Chst12	AI595374|
chr16	5202752	5202850	intron (NR_	intron (NR_	1304	NM_01379	27426	Nagpa	AI596180|
chr19	4409027	4409325	Intergenic	Intergenic	-12099	NM_00100	225876	Kdm2a	100043628
chr11	1.06E+08	1.06E+08	promoter-T	promoter-T	-103	NM_01194	26406	Map3k3	AW548911
chr11	9185802	9186075	intron (NM	intron (NM	93993	NM_17825	268379	Abca13	9830132L2
chr9	1.2E+08	1.2E+08	promoter-T	promoter-T	63	NM_02623	67561	Wdr48	8430408H1
chr14	51875402	51875575	exon (NM_	exon (NM_	299	NM_05311	93726	Rnase2a	Ear11
chr15	74761202	74761750	Intergenic	Intergenic	-24005	NM_00116	17069	Ly6e	9804|Ly67
chr6	40538427	40538550	Intergenic	Charlie8|D	-2684	NM_00103	23845	Clec5a	Clecsf5|Ly1
chr19	46433052	46433200	intron (NM	intron (NM	1756	NM_02918	75146	Tmem180	4930449A0
chr19	23215852	23216175	5' UTR (NM	5' UTR (NM	297	NM_01063	16601	Klf9	2310051E1
chr10	19953452	19953625	intron (NM	intron (NM	25293	NM_00110	621697	Gm6251	EG621697
chr9	88168227	88168525	Intergenic	Intergenic	-54071	NM_01185	23959	Nt5e	2210401F0
chr2	1.58E+08	1.58E+08	5' UTR (NM	5' UTR (NM	202	NM_17541	109275	Actr5	AA545173|
chr7	56501077	56501300	promoter-T	promoter-T	-371	NM_17527	78286	Nav2	5330421F0
chrX	71004802	71004925	exon (NM_	exon (NM_	2421	NM_01958	140571	Plxnb3	AI451018|
chr7	1.17E+08	1.17E+08	promoter-T	promoter-T	37	NM_18151	233726	Ipo7	A330055O1
chr13	41695377	41695725	Intergenic	Intergenic	-6034	NM_00116	621976	Tmem170b	EG621976
chr4	97481302	97481600	intron (NM	intron (NM	37134	NM_01090	18027	Nfia	1110047K1
chr13	95055127	95055300	promoter-T	promoter-T	23	NM_02915	107767	Scamp1	4930505M
chr1	1.55E+08	1.55E+08	intron (NM	CpG	427	NM_00784	13211	Dhx9	AI326842|
chr13	94868152	94868500	intron (NM	intron (NM	40575	NM_17258	218454	Lhfpl2	6030465B1
chr13	12501002	12501325	intron (NM	intron (NM	13521	NM_14483	217995	Heatr1	AA517551|
chr1	58502527	58502675	promoter-T	promoter-T	-271	NM_00128	70225	Ppil3	2310076N2
chr5	1.41E+08	1.41E+08	intron (NM	intron (NM	1013	NM_02152	59031	Chst12	AI595374|
chr11	84937077	84937375	intron (NM	intron (NM	16231	NM_00102	237898	Usp32	2900074J0
chr19	32641552	32641825	Intergenic	Intergenic	-28517	NM_00120	23972	Papss2	1810018P1
chr11	1.14E+08	1.14E+08	promoter-T	promoter-T	-772	NM_00116	56699	Cdc42ep4	1500041M
chr1	90174827	90175175	promoter-T	promoter-T	-847	NM_19865	381280	Hjurp	6430706D2
chr2	91895552	91895950	Intergenic	CpG	-31424	NM_01195	26427	Creb3l1	Oasis
chr4	1.09E+08	1.09E+08	Intergenic	Intergenic	-8241	NM_01387	29864	Rnf11	-
chr4	1.33E+08	1.33E+08	intron (NM	intron (NM	44980	NM_14615	230793	Ahdc1	D030015G1
chr7	25251927	25252000	intron (NM	intron (NM	4444	NM_01111	18793	Plaur	Cd87|u-PA
chr16	11252602	11252675	intron (NM	(TTTC)n|Si	1780	NM_00113	14852	Gspt1	AI314175|
chr1	1.33E+08	1.33E+08	Intergenic	Intergenic	-1340	NM_14550	226419	Dyrk3	BC006704
chr4	1.41E+08	1.41E+08	intron (NM	intron (NM	-11400	NM_02909	74772	Atp13a2	1110012E0
chr9	69563877	69564075	Intergenic	Intergenic	44771	NM_02237	64290	Foxb1	C43|Fkh5|
chr19	5727377	5727600	TTS (NM_0	TTS (NM_0	-1171	NM_05325	114601	Ehbp1l1	G430002G1

chr2	34800602	34800725	intron (NM	intron (NM	16629	NM_00942	22029	Traf1	4732496E1
chr2	1.54E+08	1.54E+08	promoter-T	promoter-T	-529	NM_02153	59038	Pxmp4	3010018P0
chr9	20296852	20296925	5' UTR (NM	5' UTR (NM	302	NM_14622	235028	Zfp426	2900057C0
chr9	50399102	50399475	Intergenic	Intergenic	12666	NM_02584	66925	Sdhd	3110001M
chr3	27117702	27117975	intron (NM	intron (NM	35912	NM_17877	320024	Nceh1	Aadacl1|B2
chr12	1.2E+08	1.2E+08	Intergenic	Intergenic	-125989	NM_17708	320145	Sp8	D930049B1
chr10	1.18E+08	1.18E+08	Intergenic	ORR1E|LTF	90158	NM_00101	69181	Dyrk2	1810038L1
chr7	1.06E+08	1.06E+08	promoter-T	promoter-T	-484	NM_17228	233545	2210018M	2310016L0
chr13	33709952	33710425	Intergenic	MYSERV-in	-43086	NM_00107	238568	Serpinb6d	Gm11390|
chr10	93710627	93710700	Intergenic	MTEa|LTR|	48909	NM_02555	66414	Ndufa12	2410011G0
chr8	74061477	74061625	promoter-T	promoter-T	-215	NM_19809	69550	Bst2	2310015I10
chr7	20092902	20093000	5' UTR (NM	5' UTR (NM	729	NM_17769	232946	Bloc1s3	BC043666|
chr3	1.04E+08	1.04E+08	intron (NM	intron (NM	1170	NM_17268	229675	Rsbn1	C230004D0
chr2	1.03E+08	1.03E+08	intron (NM	intron (NM	17121	NM_17889	99382	Abtb2	AW539457
chr3	97571827	97571925	promoter-T	promoter-T	61	NR_11036(	83679	Pde4dip	4732458A0
chr19	29887302	29887500	promoter-T	promoter-T	63	NM_17772	240614	Ranbp6	C630001B1
chr9	72618377	72618525	Intergenic	Intergenic	-36630	NM_17548	235472	Prtg	A230098A1
chr12	78235452	78235675	Intergenic	Intergenic	-103528	NM_00125	53618	Fut8	-
chr2	71995577	71995750	intron (NM	intron (NM	102989	NM_00120	56508	Rapgef4	1300003D1
chr16	17147502	17148025	exon (NM_	exon (NM_	703	NM_02694	69101	Ydjc	1810015A1
chr12	1.04E+08	1.04E+08	intron (NM	intron (NM	43832	NM_00108	217843	Unc79	9030205A0
chr19	29276352	29276475	Intergenic	Intergenic	-49880	NM_00104	16452	Jak2	Fd17
chr8	1.25E+08	1.25E+08	Intergenic	Intergenic	-1040	NM_00956	22761	Zfpm1	FOG|Fog1
chr11	1.07E+08	1.07E+08	intron (NM	intron (NM	16807	NM_00103	18613	Pecam1	C85791|Cc
chr4	1.35E+08	1.35E+08	Intergenic	CpG	66624	NM_01388	29876	Clic4	D0Jmb3|TU
chr5	1E+08	1E+08	5' UTR (NM	5' UTR (NM	219	NM_00107	11991	Hnrnpd	Auf1|Hnrp
chr10	79689677	79689775	intron (NM	intron (NM	383	NM_02343	68114	Mum1	2610019J0
chr11	82606277	82606450	intron (NM	intron (NM	11752	NM_01071	16882	Lig3	D11Wsu78
chr3	1.04E+08	1.04E+08	TTS (NM_0	TTS (NM_0	-1130	NM_17268	229675	Rsbn1	C230004D0
chr2	9893552	9893925	intron (NM	intron (NM	76498	NM_02774	209361	Taf3	140kDa|49
chr2	51828152	51828750	5' UTR (NM	5' UTR (NM	277	NM_00114	64685	Nmi	-
chr12	1.17E+08	1.17E+08	intron (NM	CpG	203	NM_01956	56220	Zfp386	Kzf1|mKIA
chr12	82046777	82046975	promoter-T	promoter-T	385	NM_00915	20384	Srsf5	Sfrs5
chr13	19922502	19922600	Intergenic	Intergenic	-6425	NM_18175	353346	Gpr141	PGR13
chr11	75345802	75345975	5' UTR (NM	5' UTR (NM	274	NM_00119	215113	Slc43a2	7630402D2
chr5	1.48E+08	1.48E+08	intron (NM	intron (NM	1510	NM_01964	19933	Rpl21	8430440E0
chr13	1.05E+08	1.05E+08	Intergenic	Intergenic	-38235	NM_00100	59079	Erbb2ip	1700028E0
chr1	1.63E+08	1.63E+08	exon (NM_	exon (NM_	8094	NM_00119	1.01E+08	4930469G2	-
chr14	61939577	61939750	Intergenic	Intergenic	11073	NM_17733	219144	Arl11	ARLTS1|C7
chr19	5448602	5448825	intron (NM	CpG	1015	NM_01023	14283	Fosl1	AW538199
chr5	1.06E+08	1.06E+08	Intergenic	Intergenic	-3787	NM_00112	231549	Lrrc8d	2810473G0
chr15	1.02E+08	1.02E+08	Intergenic	Intergenic	-2856	NM_15353	209039	Tenc1	C1-ten|nep
chr8	1.27E+08	1.27E+08	intron (NM	intron (NM	32944	NM_13927	108148	Galnt2	AI480629
chr10	75905252	75905375	exon (NM_	exon (NM_	344	NM_00878	18541	Pcnt	AW476095
chr1	95239852	95240000	promoter-T	promoter-T	-296	NM_02320	66385	Ppp1r7	2310014J0
chr6	4923327	4923550	intron (NM	intron (NM	70118	NM_18159	243725	Ppp1r9a	2810430P2
chr1	58852502	58852675	intron (NM	CpG	370	NM_00981	12370	Casp8	CASP-8|FLI

chr6	66816402	66816500	Intergenic	Intergenic	-29940	NM_00117	14701	Gng12	2010305F1
chr13	20441627	20441900	intron (NM	intron (NM	-123096	NM_19809	140580	Elmo1	6330578D2
chr3	1.31E+08	1.31E+08	Intergenic	URR1A|DN	-23897	NM_02894	74442	Sgms2	4933405A1
chr11	4322427	4322500	intron (NM	intron (NM	18622	NM_02945	75828	Hormad2	4930529M
chr1	1.37E+08	1.37E+08	intron (NM	CpG	255	NM_15377	226432	Ipo9	0710008K0
chr2	29745477	29745650	promoter-T	promoter-T	-20	NM_01361	18286	Odf2	AI848335|
chr12	33064527	33064775	3' UTR (NM	3' UTR (NM	997	NM_00116	72123	Ccdc71l	2010109K1
chr6	73178552	73178625	Intergenic	MER5A1|D	-6963	NM_00116	330355	Dnah6	9830168K2
chr14	28192827	28193175	intron (NM	intron (NM	18426	NM_00128	71704	Arhgef3	1200004I2
chr13	69673877	69674050	Intergenic	CpG-3986	-1221	NM_00116	210106	Papd7	LAK-1|POL
chr1	66746877	66747125	promoter-T	promoter-T	-466	NM_02568	66646	Rpe	2810429B0
chr19	5572302	5572700	Intergenic	Intergenic	4427	NM_00103	381201	Ap5b1	Gm962
chr7	1.23E+08	1.23E+08	intron (NM	intron (NM	11997	NM_00127	20679	Sox6	AI987981|
chr2	47271152	47271300	Intergenic	L1_Mus2|L	-1398403	NM_00739	11480	Acvr2a	ActrIIa|Acv
chr5	31543452	31543525	intron (NM	CpG	197	NM_14720	192292	Nrbp1	B230344L1
chr5	1.04E+08	1.04E+08	Intergenic	L1_Mus1|L	-10931	NM_00116	243168	Hsd17b13	AI047820|
chr6	1.16E+08	1.16E+08	Intergenic	Intergenic	-7142	NM_00966	11689	Alox5	5-LO|5-LO
chr4	1.47E+08	1.47E+08	promoter-T	promoter-T	-358	NM_00128	170731	Mfn2	D630023P1
chr8	81033827	81034000	intron (NR_	intron (NR_	686	NM_00128	76775	Slc10a7	2410193C0
chr5	1.14E+08	1.14E+08	intron (NM	intron (NM	3530	NM_02552	66383	Iscu	2310020H2
chr11	70467677	70468000	intron (NM	CpG	314	NM_01107	18643	Pfn1	Pfn
chr10	96091502	96091700	Intergenic	Intergenic	11966	NM_00756	12226	Btg1	AI426953|
chr15	78939502	78939775	5' UTR (NM	5' UTR (NM	225	NM_17746	27008	Micall1	260|AI448
chr19	7568602	7569275	promoter-T	promoter-T	10	NM_14609	109168	Atl3	4633402C0
chr19	42232377	42232500	Intergenic	Intergenic	10559	NM_18319	277010	Marveld1	AI504298|
chr3	66783777	66783850	intron (NM	intron (NM	1880	NM_01366	20429	Shox2	6330543G1
chr9	1.24E+08	1.24E+08	intron (NM	intron (NM	3762	NM_00991	12768	Ccr1	Cmkbr1|M
chr3	1.29E+08	1.29E+08	Intergenic	Intergenic	-38358	NM_00128	18741	Pitx2	9430085M
chr5	1.36E+08	1.36E+08	promoter-T	promoter-T	90	NM_14893	107939	Pom121	2610027A1
chr17	37146527	37146675	exon (NM_	exon (NM_	5990	NR_03313	22715	Zfp57	G19|Zfp-5
chr11	76308477	76308625	intron (NM	CpG	14370	NM_19801	109934	Abr	-
chr2	1.32E+08	1.32E+08	intron (NM	intron (NM	3267	NM_13865	110911	Cds2	5730450N0
chr10	1.27E+08	1.27E+08	promoter-T	promoter-T	-504	NM_00928	20852	Stat6	-
chr7	1.18E+08	1.18E+08	intron (NM	intron (NM	519	NM_02599	67150	Rnf141	2610110L0
chr3	90490652	90491175	Intergenic	Intergenic	8730	NM_00128	20202	S100a9	60B8Ag|A\
chr13	41344677	41345425	promoter-T	promoter-T	-162	NM_00113	108934	Smim13	2900036K0
chr12	85290952	85291050	promoter-T	promoter-T	-557	NM_02363	71952	2410016O0	MAPJD|NC
chr11	95194627	95194750	Intergenic	Intergenic	-3644	NM_17254	215512	Fam117a	5730593F1
chr19	21106377	21106500	Intergenic	Intergenic	-77746	NM_02895	13409	Tmc1	4933416G0
chr1	38187677	38187750	Intergenic	Intergenic	-1206	NM_01957	56210	Rev1	1110027I2
chr5	1.13E+08	1.13E+08	Intergenic	Intergenic	-1738	NM_01878	54723	Tfip11	2810002G0
chr7	87406277	87406450	TTS (NM_1	TTS (NM_1	262	NM_03137	83485	Ngrn	AW552001
chr10	87137877	87138075	Intergenic	Intergenic	153436	NM_00877	18478	Pah	AW106920
chr9	86249477	86249725	intron (NM	URR1B|DN	108922	NM_02739	70348	Ube2cbp	2610018I0
chr4	1.26E+08	1.26E+08	TTS (NM_0	TTS (NM_0	321	NM_13388	100206	Adprhl2	AI836109|
chr4	1.32E+08	1.32E+08	intron (NM	intron (NM	13913	NM_02027	56809	Gmeb1	1110050A0
chr6	29417652	29418075	promoter-T	promoter-T	80	NM_02538	66144	Atp6v1f	1110004G1

chr9	1.1E+08	1.1E+08	promoter-T	promoter-T	-56	NM_00125	72831	Dhx30	2810477H0
chrX	1.39E+08	1.39E+08	Intergenic	L2|LINE|L2	-7242	NM_01949	56068	Ammecr1	6230420G1
chr14	21526977	21527225	exon (NM_	exon (NM_	327	NM_00125	268721	Zswim8	2310021P1
chr5	88994527	88994725	intron (NM	CpG	561	NM_00128	52822	Rufy3	2810428M
chr3	1.2E+08	1.2E+08	Intergenic	Intergenic	-236782	NM_01955	56195	Ptbp2	Ptb2|brPTE
chr2	1.57E+08	1.57E+08	intron (NM	CpG	146	NM_02696	69161	Manbal	1810024K1
chr3	33699077	33699175	promoter-T	promoter-T	21	NM_02597	67120	Ttc14	2700016E0
chr3	68421727	68421925	intron (NM	intron (NM	45536	NM_01392	30953	Schip1	Nf2ip|Schip
chr5	73298752	73298925	Intergenic	Intergenic	-6763	NM_00111	75991	Slain2	5033405K1
chr8	1.16E+08	1.16E+08	promoter-T	promoter-T	27	NM_17301	270096	Mon1b	5031407H1
chr1	1.63E+08	1.63E+08	promoter-T	promoter-T	-379	NM_00745	11758	Prdx6	1-cysPrx|9
chr6	1.35E+08	1.35E+08	Intergenic	Intergenic	-3880	NM_00104	70686	Dusp16	3830417M
chr6	1.15E+08	1.15E+08	intron (NM	intron (NM	46644	NM_17768	232334	Vgll4	BC048841|
chr1	1.69E+08	1.69E+08	promoter-T	promoter-T	12	NM_00103	68944	Tmco1	1190006A0
chr15	59288202	59288350	intron (NM	intron (NM	82523	NM_00116	68501	Nsmce2	1110014D1
chr8	88366102	88366375	intron (NM	intron (NM	1337	NM_19944	102093	Phkb	AI463271
chr10	35764277	35764375	Intergenic	RLTR13D2|	-462287	NM_00125	319415	Hs3st5	D930005L0
chr6	86387877	86387975	promoter-T	promoter-T	-466	NM_14617	232196	C87436	-
chr2	38859077	38859275	intron (NM	intron (NM	1475	NM_02559	66489	Rpl35	2410039E0
chr11	16908602	16908750	promoter-T	promoter-T	45	NM_01954	56193	Plek	2010300B1
chr7	20269152	20269325	Intergenic	L1MB4|LIN	-2429	NM_00738	11425	Apoc4	Acl
chr7	25084327	25084525	5' UTR (NM	5' UTR (NM	142	NM_00956	22719	Zfp61	-
chr19	55324952	55325075	Intergenic	Intergenic	-2846	NM_02797	433256	Acsl5	1700030F0
chr5	91218277	91218550	Intergenic	Intergenic	3286	NM_20332	330122	Cxcl3	Dcip1|Gm1
chr15	33013202	33013350	intron (NM	intron (NM	392	NM_17607	54381	Cpq	1190003P1
chr9	44302952	44303125	Intergenic	Intergenic	-4181	NM_03025	80288	Bcl9l	B9L|BC003
chrX	11582652	11582725	Intergenic	Intergenic	74991	NM_17504	71458	Bcor	5830466J1
chr10	1.26E+08	1.26E+08	Intergenic	ID4_|SINE|	-4274	NM_00115	68876	Xrcc6bp1	1110068E0
chr4	40672702	40672825	intron (NM	intron (NM	2800	NM_00116	15502	Dnaja1	Hsj2|Nedd
chr13	91947052	91947175	intron (NM	CpG	188	NM_14545	69085	Zcchc9	1810019C2
chr1	1.74E+08	1.74E+08	Intergenic	Intergenic	-7910	NM_02797	71870	Ccdc19	1700028D0
chr2	1.64E+08	1.64E+08	Intergenic	RMER15-in	-2159	NM_00111	381404	Pabpc1l	1810053B0
chr8	35182077	35182150	intron (NM	intron (NM	4029	NM_00112	234155	Mboat4	GOAT|Gm1
chr16	36391327	36391400	Intergenic	Intergenic	17086	NM_00108	20862	Stfa2	Stf2
chr11	75003927	75004125	promoter-T	promoter-T	-41	NM_14449	116905	Dph1	2310011M
chr16	76527727	76527975	Intergenic	MLT1C|LTR	-154557	NM_17344	268903	Nrip1	6030458L2
chr6	88116102	88116375	Intergenic	Intergenic	-32420	NM_00809	14461	Gata2	Gata-2
chr10	12695527	12695625	Intergenic	ID_B1|SINE	-11511	NM_00116	74732	Stx11	5830405C0
chr2	1.29E+08	1.29E+08	intron (NM	intron (NM	962	NM_02824	72477	Tmem87b	2610301K1
chr17	28020177	28020350	intron (NM	intron (NM	24406	NM_02683	68776	Taf11	1110038O1
chr9	86360752	86361275	intron (NM	CpG	252	NM_17720	320615	Dopey1	B130005I0
chr9	76255527	76255700	Intergenic	L1MA8|LIN	-84228	NM_19896	387285	Hcrtr2	OX2R|mOX
chr2	1.31E+08	1.31E+08	exon (NM_	exon (NM_	322	NM_00768	12616	Cenpb	-
chr6	1.16E+08	1.16E+08	intron (NM	CpG	137	NM_02658	28006	Fam21	A130095H0
chr7	1.31E+08	1.31E+08	intron (NM	intron (NM	17868	NM_00125	628779	Hs3st4	EG628779|
chr1	88483477	88483825	promoter-T	promoter-T	-24	NM_17297	26895	Cops7b	D1Wsu66e
chr2	84807602	84808100	Intergenic	Intergenic	-12767	NM_00892	19074	Prg2	MBP|mMB

chr5	73204327	73204400	intron (NM	intron (NM	55324	NM_00111	21682	Tec	-
chr1	1.3E+08	1.3E+08	Intergenic	Intergenic	21775	NM_00991	12767	Cxcr4	CD184|Cm
chr10	19282977	19283100	Intergenic	(TAAA)n|Si	-28726	NM_01051	15979	Ifngr1	CD119|IFN
chr18	78034827	78035025	intron (NM	MIR|SINE|	1637	NM_01383	19201	Pstpip2	MAYP|cmc
chr5	1.16E+08	1.16E+08	promoter-T	promoter-T	95	NM_02693	69076	Triap1	1810015M
chr4	63214877	63214975	Intergenic	Intergenic	-6468	NM_14490	230279	6330416G1	AW492431
chr3	83930777	83930950	intron (NM	intron (NM	28845	NM_02979	76915	Mnd1	2610034E1
chr4	20066877	20066975	intron (NM	intron (NM	97727	NM_01028	14590	Ggh	gamma-GH
chr19	46541602	46541750	intron (NM	intron (NM	-34462	NM_05310	93679	Trim8	AA408830|
chr15	83386552	83386650	promoter-T	promoter-T	-460	NM_00103	223722	Mcat	AI225907|
chr13	37553952	37554350	Intergenic	Intergenic	116937	NM_01074	17084	Ly86	MD-1|MD1
chr8	23587877	23588275	promoter-T	promoter-T	-725	NM_00114	102032	Smim19	AI316807|
chr4	28871577	28871775	intron (NM	intron (NM	131381	NM_00112	13841	Epha7	Cek11|Ebk
chr7	6325752	6325900	intron (NM	intron (NM	9811	NM_00111	330463	Zfp78	A330079D1
chr17	36160827	36161050	intron (NR_	intron (NR_	-3433	NM_00820	15042	H2-T24	H-2T24
chr2	84808752	84808825	Intergenic	Intergenic	-11830	NM_00892	19074	Prg2	MBP|mMB
chr8	1.22E+08	1.22E+08	Intergenic	Intergenic	-11424	NM_02795	71839	Osgin1	1700012B1
chr11	83406852	83407200	promoter-T	promoter-T	-437	NM_00913	20305	Ccl6	MRP-1|Scy
chr1	60466452	60466750	5' UTR (NM	5' UTR (NM	138	NM_00119	329165	Abi2	8430425M
chr14	21866902	21867075	intron (NM	intron (NM	4676	NM_01882	55946	Ap3m1	1200013D0
chr6	38925902	38926225	intron (NM	MARNA|DI	57078	NM_01153	21391	Tbxas1	CYP5|CYP5
chr13	51496452	51496550	Intergenic	Intergenic	-7486	NM_01010	13610	S1pr3	AI132464|
chr4	1.15E+08	1.15E+08	intron (NM	intron (NM	16457	NM_02564	66588	Cmpk1	0610011D0
chr1	80965877	80966050	Intergenic	MT2A|LTR	-107929	NM_17284	241134	Nyap2	9430031J1
chr5	34530927	34531125	promoter-T	promoter-T	-667	NM_01075	17122	Mxd4	2810410A0
chr14	66849877	66850150	intron (NM	intron (NM	-17624	NM_00116	19229	Ptk2b	CADTK|CA
chr3	1.32E+08	1.32E+08	promoter-T	promoter-T	-120	NM_00792	13722	Aimp1	9830137A0
chr5	20460252	20460375	non-coding	non-coding	-2673	NM_00108	242860	Rsbn1l	8430412F0
chr10	1.08E+08	1.08E+08	Intergenic	Intergenic	-21905	NM_02789	17931	Ppp1r12a	1200015F0
chr10	67520527	67520875	Intergenic	Charlie1|D	78355	NM_00108	170799	Rtkn2	B130039D2
chr8	1.23E+08	1.23E+08	exon (NM_	exon (NM_	7647	NM_00116	234797	6430548M	AW049007
chr19	34344727	34345025	Intergenic	Intergenic	-15013	NM_00739	11475	Acta2	0610041G0
chr7	1.34E+08	1.34E+08	promoter-T	promoter-T	-15	NM_02663	52858	Cdipt	9530042F1
chr1	1.58E+08	1.58E+08	intron (NM	intron (NM	19531	NM_01071	16872	Lhx4	A330062J1
chr3	8619677	8619925	Intergenic	MTD|LTR|	47237	NM_01042	15213	Hey1	AI316788|
chr3	1.21E+08	1.21E+08	Intergenic	Intergenic	19070	NM_00103	619318	4930432M	-
chr5	1.24E+08	1.24E+08	intron (NM	intron (NM	8824	NM_00104	208043	Setd1b	AA516740|
chr9	63869602	63869725	5' UTR (NM	5' UTR (NM	203	NM_00854	17130	Smad6	Madh6|b2
chr2	1.67E+08	1.67E+08	Intergenic	RSINE1|SIN	-30689	NM_00896	19223	Ptgis	Cyp8|Cyp8
chr7	82732502	82732625	intron (NM	intron (NM	132143	NM_02933	75547	Akap13	1700026G0
chr2	91152327	91152400	intron (NM	intron (NM	47091	NM_02385	77038	Arfgap2	2310032E0
chr2	1.53E+08	1.53E+08	intron (NM	intron (NM	17933	NM_08046	140484	Pofut1	O-FucT-1|r
chr9	96790002	96790225	promoter-T	promoter-T	-220	NM_00128	235534	Pxylp1	9430094M
chr15	96471727	96471800	5' UTR (NM	5' UTR (NM	630	NM_13408	105727	Slc38a1	AA408026|
chr11	79037202	79037350	Intergenic	Intergenic	19250	NM_00104	78889	Wsb1	1110056B1
chr10	61950002	61950075	promoter-T	promoter-T	-485	NM_13367	30930	Vps26a	AA407240|
chr5	1.14E+08	1.14E+08	intron (NM	intron (NM	1223	NM_14616	231633	Tmem119	AW208946

chr11	57614677	57614825	promoter-T	promoter-T	-388	NM_00108	50724	Sap30l	2310079P1
chr1	10028552	10028675	promoter-T	promoter-T	314	NM_02649	211660	Cspp1	2310020J1
chr4	1.18E+08	1.18E+08	intron (NM	CpG	321	NM_03361	114143	Atp6v0b	2310024H1
chr1	1.87E+08	1.87E+08	exon (NM_	exon (NM_	213	NM_13368	67247	2-Mar	2810484M
chr1	1.35E+08	1.35E+08	Intergenic	Intergenic	-11080	NM_13381	108954	Ppp1r15b	1810033K1
chr4	1.34E+08	1.34E+08	intron (NM	intron (NM	3968	NM_14555	100017	Ldlrap1	AA691260|
chr6	17845677	17845950	intron (NM	intron (NM	134632	NM_02365	22413	Wnt2	2610510E1
chr11	1.16E+08	1.16E+08	intron (NM	GC_rich|Lc	298	NM_00816	14784	Grb2	AA408164|
chr1	1.34E+08	1.34E+08	5' UTR (NM	5' UTR (NM	199	NM_00879	18557	Cdk18	AA682070|
chr1	1.68E+08	1.68E+08	intron (NM	intron (NM	7996	NM_00100	68481	Mpzl1	1110007A1
chr19	47805527	47805700	promoter-T	promoter-T	-633	NM_02637	67788	Sfr1	6330577E1
chr4	1.29E+08	1.29E+08	promoter-T	promoter-T	199	NM_13415	107271	Yars	AL024047|
chr7	30769002	30769250	5' UTR (NM	5' UTR (NM	152	NM_00116	243905	Zfp568	C80731|Gr
chr17	47277427	47277550	promoter-T	promoter-T	-403	NM_00109	224829	Trerf1	9430096I1
chr1	1.66E+08	1.66E+08	Intergenic	RMER5|LTI	-68252	NM_00972	11931	Atp1b1	Atp4b|Atp
chr2	1.44E+08	1.44E+08	promoter-T	promoter-T	-17	NM_00116	228714	Csrp2bp	2510008M
chr12	77470477	77470575	promoter-T	promoter-T	21	NM_02835	109929	Zbtb25	2810462M
chr7	90816627	90816875	intron (NM	MTC|LTR|I	36224	NM_02337	170460	Stard5	2310058G2
chr11	79894652	79894850	promoter-T	promoter-T	-258	NM_01877	54394	Crlf3	BB164954|
chr3	30499727	30499950	Intergenic	Intergenic	-1046	NM_02969	76652	Actrt3	1700119I2
chr1	52289427	52289500	exon (NM_	exon (NM_	613	NM_00108	14660	Gls	6330442B1
chr2	1.22E+08	1.22E+08	Intergenic	Intergenic	-8897	NM_00101	435684	Shf	-
chr17	45732577	45732750	promoter-T	promoter-T	-142	NM_00119	63959	Slc29a1	1200014D2
chr17	43710677	43710750	intron (NM	intron (NM	5313	NM_01373	27226	Pla2g7	R75400
chr11	1.19E+08	1.19E+08	intron (NM	intron (NM	36625	NM_17244	207592	Tbc1d16	B930087K0
chr2	1.74E+08	1.74E+08	intron (NM	CpG	411	NM_21373	228961	Npepl1	BC023239|
chr1	60072977	60073100	intron (NM	intron (NM	26893	NM_02740	70375	Ica1l	1700030B1
chr3	87421027	87421150	Intergenic	CpG	-1585	NM_00100	213498	Arhgef11	B930073M
chr8	11089102	11089175	Intergenic	Intergenic	-80708	NM_00108	384783	Irs2	Irs-2
chr2	1.02E+08	1.02E+08	Intergenic	Intergenic	-15259	NM_00129	241576	Ldlrad3	6430500P0
chr10	98360352	98360425	Intergenic	Intergenic	-17398	NM_02648	67972	Atp2b1	2810442I2
chr1	1.93E+08	1.93E+08	promoter-T	promoter-T	-183	NR_03757	52477	Angel2	2610307I2
chr3	69461602	69461775	Intergenic	Intergenic	-58806	NM_02002	26879	B3galnt1	B3galt3|b3
chr16	8784802	8785125	Intergenic	Intergenic	-45230	NM_00108	69053	1810013L2	1110017P0
chr11	83287002	83287150	intron (NM	CpG	466	NM_02742	70439	Taf15	2610111C2
chr9	1.22E+08	1.22E+08	Intergenic	L1M2|LINE	-45257	NM_15354	215494	Pomgnt2	Ago61|C85
chr17	35262177	35262325	TTS (NM_0	TTS (NM_0	-1096	NM_03347	114585	D17H6S53I	G4|NG34
chr12	71241877	71241950	Intergenic	B1_Mm|SI	-13245	NM_00119	1.01E+08	Abhd12b	Gm804
chr2	14150827	14150975	promoter-T	promoter-T	-140	NM_00862	17533	Mrc1	AW259686
chr11	83112102	83112200	intron (NM	intron (NM	328	NM_13402	103737	Pex12	AI451906
chr11	98756702	98756800	intron (NM	B1F|SINE|/	-12452	NM_00102	23834	Cdc6	CDC18L
chr8	87356127	87356300	promoter-T	promoter-T	32	NM_18335	102060	Gadd45gip	2310040G1
chr15	1.01E+08	1.01E+08	intron (NM	intron (NM	8888	NM_03361	109901	Cela1	1810009A1
chr7	78976952	78977400	Intergenic	Intergenic	474305	NM_00102	244049	Mctp2	Gm489
chr5	1.4E+08	1.4E+08	intron (NM	intron (NM	1549	NM_17485	231830	Micall2	A930021H1
chr7	72017502	72017775	exon (NM_	exon (NM_	288	NM_02323	66647	Ndnl2	5730494G1
chr7	20518402	20518575	Intergenic	Intergenic	-11996	NM_02751	52118	Pvr	3830421F0

chr1	1.54E+08	1.54E+08	Intergenic	Intergenic	98836	NM_02567	66637	Tsen15	5730449L1
chr4	1.29E+08	1.29E+08	intron (NM	CpG	784	NM_02860	73680	Zbtb8a	2410081M
chr4	10802477	10802550	intron (NM	intron (NM	868	NM_02600	67157	2610301B2	AI428449
chr8	1.12E+08	1.12E+08	intron (NM	intron (NM	5340	NM_17564	234723	Txnl4b	AI595343|
chr2	1.68E+08	1.68E+08	Intergenic	Intergenic	-1102	NM_00988	12608	Cebpb	C/EBPbeta
chr6	52176952	52177275	5' UTR (NM	5' UTR (NM	256	NM_01045	15405	Hoxa9	D6a9|Hox-
chr19	33297377	33297900	intron (NM	intron (NM	169147	NM_00116	67795	Rnls	6530404N2
chr14	64478852	64479075	promoter-T	promoter-T	-186	NM_02822	72400	Pinx1	2210403I1
chr1	1.83E+08	1.83E+08	5' UTR (NM	5' UTR (NM	171	NM_13370	69051	Pycr2	1810018M
chr14	75197277	75197400	intron (NM	intron (NM	65234	NM_01690	13885	Esd	Es-10|Es10
chr2	35835102	35835275	promoter-T	promoter-T	-44	NM_02977	74410	Ttll11	4932702F0
chr4	94223252	94223325	exon (NM_	exon (NM_	199	NM_02636	67770	Caap1	5830433M
chr1	1.72E+08	1.72E+08	Intergenic	Intergenic	-53256	NM_00906	19737	Rgs5	1110070A0
chr3	87734252	87734425	promoter-T	promoter-T	102	NM_17766	229504	Isg20l2	4930429N2
chr6	29161827	29162050	intron (NM	intron (NM	333	NM_01182	23917	Impdh1	B930086D2
chr4	45962227	45962425	exon (NM_	exon (NM_	-15881	NM_00129	100121	Tdrd7	5730495N1
chr13	52646827	52647175	Intergenic	Intergenic	-20796	NM_00102	68203	Diras2	2900052J1
chr10	1.25E+08	1.25E+08	intron (NM	intron (NM	56865	NM_01139	20503	Slc16a7	4921534N0
chr3	96439752	96439850	promoter-T	promoter-T	31	NM_00116	18632	Pex11b	PEX11beta
chr5	53184402	53184550	intron (NM	B2_Mm2|S	10170	NM_03018	78796	Zcchc4	4930449I2
chr6	38775877	38776150	intron (NM	intron (NM	11297	NM_00113	15258	Hipk2	1110014O2
chr2	32463302	32463575	intron (NM	intron (NM	933	NR_110347	50935	St6galnac6	ST6GalNAc
chr11	1.01E+08	1.01E+08	promoter-T	promoter-T	-177	NM_01119	19192	Psme3	AA410043|
chr13	77274952	77275025	promoter-T	promoter-T	191	NM_00114	72371	2210408I2	-
chr10	43246702	43246800	intron (NM	B1F|SINE|A	14049	NM_02641	67851	1700021F0	-
chr1	88255602	88255875	promoter-T	promoter-T	292	NM_01088	17975	Ncl	B530004O1
chr2	1.1E+08	1.1E+08	3' UTR (NM	3' UTR (NM	8216	NM_01169	22343	Lin7c	9130007B1
chr1	53785277	53785500	Intergenic	Intergenic	-21760	NM_00125	627872	Dnah7a	Dnahc7|Dn
chr11	72503602	72503900	intron (NM	CpG	247	NM_00967	11736	Ankfy1	Ankhzn|ZF
chr11	19819627	19820425	Intergenic	CpG	-4419	NM_03352	114716	Spred2	C79158
chr3	95462677	95462750	promoter-T	promoter-T	70	NM_00856	17210	Mcl1	AW556805
chr5	30481302	30481450	promoter-T	promoter-T	144	NM_17887	97212	Hadha	C77020|M
chr4	40152577	40152950	3' UTR (NM	3' UTR (NM	34095	NM_17268	230073	Ddx58	6430573D2
chr2	57757452	57757750	Intergenic	Intergenic	-92965	NM_17285	241391	Galnt5	4832424J2
chr1	1.73E+08	1.73E+08	promoter-T	promoter-T	-133	NM_01948	56009	Alyref2	C130042O1
chr6	50209502	50209775	intron (NM	MIRb|SINE	2130	NM_01876	54722	Dfna5	2310037D0
chr7	75295202	75295275	intron (NM	intron (NM	113881	NM_03010	78444	Pgpep1l	C330024D1
chr2	11858152	11858250	Intergenic	RMER16-in	158821	NM_17726	320816	Ankrd16	2810455F0
chr5	54200902	54201000	promoter-T	promoter-T	85	NM_14451	67249	Tbc1d19	2810453K0
chr3	68931277	68931400	promoter-T	promoter-T	-324	NM_00846	16649	Kpna4	1110058D0
chr6	47543402	47543500	intron (NM	intron (NM	1578	NM_00797	14056	Ezh2	Enx-1|Enx1
chr6	91422402	91422850	intron (NM	intron (NM	791	NM_13392	72170	Chchd4	2410012P2
chr15	78640327	78640425	Intergenic	Intergenic	-6904	NM_13085	105844	Card10	AI449026|
chr7	14798227	14798375	Intergenic	Lx3C|LINE|	41918	NM_00108	629219	Sult2a6	EG629219|
chr4	19502377	19502450	exon (NM_	exon (NM_	200	NM_02547	66302	Rmdn1	Fam82b|R
chr13	37876202	37876400	Intergenic	Intergenic	-41606	NM_02683	68750	Rreb1	1110037N0
chr13	10263227	10263425	intron (NM	intron (NM	96723	NM_03326	12671	Chrm3	Chrm-3|M

chr18	53320727	53320800	Intergenic	Intergenic	-15256	NM_02638	67804	Snx2	0610030A0
chr8	94370702	94370800	Intergenic	L1MB5|LIN	-45198	NM_00839	16373	Irx3	AI894186
chr3	1.45E+08	1.45E+08	promoter-T	promoter-T	37	NM_02571	52184	Odf2l	4733401D0
chr1	17441552	17441825	Intergenic	MamRep18	-150294	NM_05319	94227	Pi15	P24TI|P25T
chr3	94271352	94271425	intron (NM	intron (NM	6125	NM_02550	66353	Riiad1	2310007A1
chr7	1.48E+08	1.48E+08	promoter-T	promoter-T	79	NM_02588	66985	Rassf7	2400009B1
chr10	1.21E+08	1.21E+08	intron (NM	intron (NM	842	NM_02936	75612	Gns	2610016K1
chr12	1.13E+08	1.13E+08	promoter-T	promoter-T	30	NM_02280	17169	Mark3	1600015G0
chr17	57418627	57418775	exon (NM_	exon (NM_	178	NM_00116	22324	Vav1	Vav|vav-T
chr16	32190127	32190200	Intergenic	ID4|SINE|I	10277	NM_00103	328660	Bex6	B020003O0
chr4	1.41E+08	1.41E+08	intron (NM	intron (NM	1233	NM_00101	68817	Ddi2	1110056G1
chr5	44614802	44615050	intron (NM	intron (NM	2919	NM_17376	231225	Tapt1	4932414K1
chr11	61307552	61307725	5' UTR (NM	5' UTR (NM	130	NM_00129	23939	Mapk7	BMK-1|BM
chr1	1.58E+08	1.58E+08	intron (NM	intron (NM	15533	NM_00923	20652	Soat1	8430426K1
chr4	5133077	5133200	Intergenic	Intergenic	-412685	NM_17773	242291	Impad1	1110001C2
chr7	82732077	82732150	intron (NM	intron (NM	131693	NM_02933	75547	Akap13	1700026G0
chr14	30781202	30781325	promoter-T	promoter-T	-303	NM_01997	80795	Selk	1110001C0
chr9	82869352	82869600	promoter-T	promoter-T	-380	NM_00108	83946	Phip	2810004D2
chr5	1.18E+08	1.18E+08	intron (NM	MTC|LTR|I	3527	NM_00111	333050	Ksr2	-
chr4	1.43E+08	1.43E+08	promoter-T	promoter-T	-14	NM_00125	110593	Prdm2	4833427P1
chr9	50425802	50425875	promoter-T	promoter-T	-269	NM_21244	270156	AU019823	Gm639
chr9	95855052	95855550	exon (NM_	exon (NM_	122	NM_01191	24127	Xrn1	Dhm2|exo
chr4	52929602	52929875	Intergenic	Intergenic	-5074	NM_14683	258836	Olfr272	MOR262-7
chr11	21138577	21138800	promoter-T	promoter-T	-347	NM_13906	245944	Vps54	Hcc8|Vps5
chr19	48504977	48505075	intron (NM	intron (NM	224511	NM_02569	66673	Sorcs3	6330404A1
chr10	67186552	67186675	Intergenic	Intergenic	-174910	NM_00100	211488	Ado	Gm237
chr6	82602827	82602900	promoter-T	promoter-T	-4	NM_02588	66979	Pole4	2400007P0
chr12	73794902	73795125	Intergenic	Intergenic	-29198	NM_02552	66375	Dhrs7	2310016E2
chr1	79708027	79708500	intron (NM	intron (NM	-39716	NM_18302	252903	Ap1s3	1190009B2
chr3	1.08E+08	1.08E+08	Intergenic	Intergenic	-21276	NM_00111	12977	Csf1	C87615|Cs
chr4	23450577	23450675	Intergenic	RMER16-in	-972983	NM_19946	212377	Mms22l	F730047E0
chr2	72314052	72314400	promoter-T	promoter-T	-50	NM_02586	66953	Cdca7	2310021G0
chr11	8608752	8609000	Intergenic	Intergenic	-44338	NM_00108	319939	Tns3	BC023928|
chr6	1.27E+08	1.27E+08	promoter-T	promoter-T	97	NM_18140	101187	Parp11	5330431N2
chr7	87065552	87065775	promoter-T	promoter-T	-137	NM_00968	11778	Ap3s2	[s]3B
chr9	7569827	7569925	Intergenic	Intergenic	-1582	NM_00103	234911	Mmp27	Gm180
chr12	59830102	59830200	Intergenic	Intergenic	282853	NM_00914	20334	Sec23a	Msec23|Se
chr11	77893602	77893875	exon (NM_	exon (NM_	147	NM_02231	57837	Eral1	2610524P0
chr2	72885077	72885375	Intergenic	Intergenic	-66723	NM_00101	20687	Sp3	D130027J0
chr4	8864502	8864575	Intergenic	RMER17D2	246985	NM_00127	320790	Chd7	A730019I0
chr5	1.23E+08	1.23E+08	promoter-T	promoter-T	-100	NM_00128	59008	Anapc5	2510006G1
chr12	1.14E+08	1.14E+08	intron (NM	intron (NM	9443	NM_00742	11565	Adssl1	AI528595|A
chr14	51684627	51684825	Intergenic	Intergenic	6391	NM_20755	404319	Olfr750	GA_x5J8B7
chr11	22714177	22714275	Intergenic	Intergenic	45509	NM_01688	53625	B3gnt2	AA408337|
chr3	1.46E+08	1.46E+08	exon (NM_	exon (NM_	136	NM_02733	70285	Rpf1	2210420E2
chr2	34856177	34856400	intron (NM	intron (NM	-38996	NM_00942	22029	Traf1	4732496E1
chr18	61234377	61234450	intron (NM	intron (NM	29609	NM_00880	18596	Pdgfrb	AI528809|(

chr4	1.45E+08	1.45E+08	promoter-T	promoter-T	45	NM_00127	230895	Vps13d	-
chr17	27193627	27193975	promoter-T	promoter-T	-448	NM_08055	16440	Itpr3	Ip3r3|Itpr-
chr15	8461877	8461950	Intergenic	ORR1E|LTF	-67450	NM_20123	71175	Nipbl	Idn3
chr18	7868752	7868850	promoter-T	promoter-T	-29	NM_00128	225131	Wac	1110067P0
chr15	5065777	5067500	promoter-T	promoter-T	25	NM_02606	67281	Rpl37	3110005M
chr8	1.1E+08	1.1E+08	promoter-T	promoter-T	75	NM_02627	67619	Nob1	1700021I0
chr5	32054502	32054625	intron (NM	intron (NM	54140	NM_18128	107976	Bre	6030405P1
chr3	1.57E+08	1.57E+08	promoter-T	promoter-T	-385	NM_01738	53861	Zranb2	AI227013|
chr12	93017277	93017425	intron (NM	CpG	7525	NM_17536	108800	Ston2	4933401N2
chr15	36936152	36936300	intron (NM	intron (NM	931	NM_02652	68036	Zfp706	3110006P0
chr10	1.11E+08	1.11E+08	Intergenic	PB1|SINE|	-29995	NM_00100	237542	Osbpl8	AA536976|
chr1	1.73E+08	1.73E+08	TTS (NM_0	TTS (NM_0	3625	NM_00124	27045	Nit1	AI255805|
chr5	1.26E+08	1.26E+08	intron (NM	intron (NM	1574	NM_01963	22190	Ubc	2700054O0
chr13	37734502	37734775	Intergenic	LTR16E2|L	-183269	NM_02683	68750	Rreb1	1110037N0
chr5	1.36E+08	1.36E+08	promoter-T	promoter-T	91	NM_17254	215210	Tmem120a	2010310D0
chr7	53085977	53086225	promoter-T	promoter-T	58	NM_15341	101612	Grwd1	A301|AI50
chr16	24393327	24393650	promoter-T	promoter-T	52	NM_17866	210126	Lpp	9430020K1
chr8	1.19E+08	1.19E+08	intron (NM	MIRc|SINE	104228	NM_02944	75796	Cdyl2	1700029M
chr9	44618477	44619050	exon (NM_	exon (NM_	-7729	NM_13895	192653	Ttc36	-
chr12	32036302	32036475	promoter-T	promoter-T	-52	NM_00786	13382	Dld	-
chr6	17643952	17644050	promoter-T	promoter-T	7	NM_00128	64213	St7	9430001H0
chr16	38679277	38679350	intron (NM	intron (NM	33835	NM_02026	12549	Arhgap31	5830477L0
chr10	17333052	17333375	Intergenic	MER63B|D	-109821	NM_01082	17684	Cited2	AI835299|
chr15	63480052	63480225	Intergenic	Intergenic	160156	NM_03137	83492	Gsdmc	Gsdmc1|M
chr2	3249502	3249600	intron (NM	CpG	349	NM_00101	227522	Rpp38	C330006A1
chr15	78135377	78135450	intron (NM	intron (NM	738	NM_00778	12984	Csf2rb2	AIC2A|Beta
chr9	50576252	50576425	promoter-T	promoter-T	-5	NM_19867	382137	Fdxacb1	D630004A1
chr11	1.11E+08	1.11E+08	Intergenic	Intergenic	-77396	NM_01060	16517	Kcnj16	6430410F1
chr4	1.55E+08	1.55E+08	promoter-T	promoter-T	53	NM_20767	56036	Ccnl2	1700010A0
chr3	5413327	5413900	3' UTR (NM	3' UTR (NM	162635	NM_00116	19302	Pex2	D3Ertd138
chr6	90270152	90270275	intron (NM	intron (NM	4966	NM_02792	71797	Chst13	1110067M
chr1	1.55E+08	1.55E+08	Intergenic	Intergenic	-18510	NM_01068	226519	Lamc1	Lamb2
chr13	74227927	74228000	Intergenic	Intergenic	-28230	NM_02895	74470	Cep72	2610029E1
chr10	1.27E+08	1.27E+08	promoter-T	promoter-T	-298	NM_00783	13198	Ddit3	CHOP-10|C
chr17	71334852	71335200	intron (NM	intron (NM	4830	NM_02340	67938	Myl12b	1500001M
chr4	1.2E+08	1.2E+08	Intergenic	ORR1E|LTF	-54810	NM_19406	329934	Foxo6	-
chr11	1.16E+08	1.16E+08	exon (NM_	exon (NM_	424	NM_00100	70450	Unc13d	2610108D0
chr3	1.16E+08	1.16E+08	intron (NM	intron (NM	-84486	NM_02242	64378	Gpr88	AW061286
chr8	1.28E+08	1.28E+08	intron (NM	MLT1E1A|	52954	NM_17556	270109	Pcnxl2	A830048P0
chr1	82795477	82795825	Intergenic	Intergenic	-30620	NM_02545	66261	Tm4sf20	1810018L0
chr11	1.18E+08	1.18E+08	promoter-T	promoter-T	-338	NM_00101	11799	Birc5	AAC-11|Ap
chr4	1.48E+08	1.48E+08	promoter-T	promoter-T	72	NM_02787	71707	Ubiad1	1200002M
chr2	1.53E+08	1.53E+08	intron (NM	intron (NM	2156	NM_01880	54711	Plagl2	AU018672
chr4	1.3E+08	1.3E+08	intron (NM	intron (NM	21934	NM_02564	66585	Snrnp40	0610009C0
chr7	1.48E+08	1.48E+08	intron (NM	intron (NM	387	NM_00117	107702	Rnh1	AW546468
chr1	1.3E+08	1.3E+08	exon (NM_	exon (NM_	318	NM_02639	67812	Ubxn4	1300013G1
chr3	1.22E+08	1.22E+08	promoter-T	promoter-T	-44	NM_15380	99480	Dnttip2	4930588M

chr3	52912602	52912775	intron (NM	intron (NM	67219	NM_17538	108927	Lhfp	2810489O(
chr9	21961827	21961975	intron (NM	intron (NM	611	NM_00108	66268	Pigyl	1810008A1
chr6	1.38E+08	1.38E+08	intron (NM	intron (NM	1055	NM_01994	56615	Mgst1	1500002K1
chr11	1.07E+08	1.07E+08	5' UTR (NM	5' UTR (NM	141	NM_02548	66313	Smurf2	2810411E2
chr1	1.83E+08	1.83E+08	Intergenic	Intergenic	37664	NM_13322	170760	Acbd3	60kDa|843
chr9	1.19E+08	1.19E+08	intron (NM	intron (NM	-8927	NM_00103	382113	Slc22a14	Gm1128
chr2	1.18E+08	1.18E+08	Intergenic	RSINE1|SIN	-23331	NM_00927	20813	Srp14	14kDa|AW
chr4	1.55E+08	1.55E+08	promoter-T	promoter-T	-166	NM_00128	140499	Ube2j2	1200007B1
chr14	60586752	60586875	intron (NM	intron (NM	118858	NM_01580	50769	Atp8a2	AI415030|/
chr8	1.24E+08	1.24E+08	promoter-T	promoter-T	105	NM_00103	407789	BC048644	-
chr6	38384027	38384125	5' UTR (NM	5' UTR (NM	151	NM_17718	320538	Ubn2	2900060J0
chr2	1.57E+08	1.57E+08	intron (NM	intron (NM	11443	NM_02612	67388	1110008F1	AI316789
chr11	30854427	30854675	promoter-T	promoter-T	153	NM_02390	65257	Asb3	2400011J0
chr2	34262577	34262725	5' UTR (NM	5' UTR (NM	280	NM_00129	227743	Mapkap1	AI591529|
chr4	41687827	41687975	intron (NM	CpG-9840	331	NM_00128	18391	Sigmar1	AL024364|
chr9	99082502	99082900	Intergenic	Intergenic	-42071	NM_02909	74769	Pik3cb	1110001J0
chr14	70977702	70977825	exon (NM_	exon (NM_	109	NM_15313	213484	Nudt18	BC036718
chr3	1.35E+08	1.35E+08	promoter-T	promoter-T	-112	NM_02728	110173	Manba	2410030O(
chr8	1.29E+08	1.29E+08	TTS (NM_0	TTS (NM_0	15883	NM_02421	67952	Tomm20	1810060K0
chr16	45030027	45030250	Intergenic	Intergenic	-64028	NM_02643	67896	Ccdc80	2610001E1
chr7	1.04E+08	1.04E+08	intron (NM	CpG	177	NM_01024	14389	Gab2	AI463667|
chr8	1.2E+08	1.2E+08	intron (NM	CpG	260	NM_17228	234779	Plcg2	PLCgamma
chr9	61795577	61795675	Intergenic	Intergenic	-1020	NM_02424	71819	Kif23	3110001D1
chr5	33971552	33971725	intron (NM	intron (NM	646	NM_17839	74504	Fam53a	2410018C1
chr9	69136077	69136175	intron (NM	intron (NM	-1522	NM_00128	19883	Rora	9530021D1
chrX	46219927	46220075	intron (NM	(CAGG)n|S	10889	NM_00108	209005	Gm595	-
chr1	1.3E+08	1.3E+08	Intergenic	Intergenic	-30208	NM_14550	226414	Dars	5730439G1
chr2	1.73E+08	1.73E+08	Intergenic	Intergenic	40273	NM_01954	56190	Rbm38	Rnpc1|Seb
chr11	1.11E+08	1.11E+08	Intergenic	Intergenic	-11702	NM_00842	16518	Kcnj2	IRK1|Kcnf1
chr11	97849152	97849300	Intergenic	Intergenic	-1466	NM_00116	72324	Plxdc1	2410003I0
chr11	68854427	68854575	Intergenic	tRNA-Asp-(	-4644	NM_01149	20877	Aurkb	AIM-1|AIR
chr1	1.73E+08	1.73E+08	Intergenic	Intergenic	-4078	NM_02789	71740	Pvrl4	1200017F1
chr14	61402102	61402300	Intergenic	Intergenic	-1202	NM_02743	70478	Mipep	5730405E0
chr4	19496952	19497025	intron (NM	CpG	263	NM_02776	70568	Cpne3	5430428M
chr1	39535152	39535425	exon (NM_	exon (NM_	304	NM_01877	54610	Tbc1d8	AD3|HBLP1
chr2	1.04E+08	1.04E+08	intron (NM	intron (NM	-1688	NM_00114	16909	Lmo2	Rbtn-2|Rbt
chr10	80023302	80023575	intron (NM	intron (NM	867	NM_02585	66932	Rexo1	1700021P1
chr2	24291477	24291700	intron (NM	intron (NM	39531	NM_01104	18510	Pax8	Pax-8
chr11	51577502	51577625	intron (NM	intron (NM	398	NM_02553	66397	Sar1b	2310075M
chr12	8162502	8162575	Intergenic	Intergenic	-52375	NM_17240	68832	1110057K0	LDAH
chr4	1.41E+08	1.41E+08	promoter-T	promoter-T	-221	NM_00103	69582	Plekhm2	2310034J1
chr14	79689827	79690125	intron (NM	intron (NM	11466	NM_02542	66214	Rgcc	1190002H2
chr13	56237102	56237250	promoter-T	promoter-T	-265	NM_00115	26914	H2afy	H2AF12M|
chr11	54544477	54544550	intron (NM	intron (NM	56692	NM_17862	72729	Cdc42se2	2810404F1
chr1	1.66E+08	1.66E+08	Intergenic	MTEa|LTR|	7412	NM_02727	69962	Mettl18	2810422O2
chr1	59294352	59294750	promoter-T	promoter-T	-476	NM_14610	74018	Als2	3222402C2
chr14	57395277	57395350	intron (NM	intron (NM	1840	NM_02568	66645	Pspc1	5730470C0

chr9	32864752	32864975	Intergenic	L1_Mur3|L	361236	NM_00103	23871	Ets1	AI196000|/
chr7	87679852	87679950	promoter-T	promoter-T	-17	NM_00755	12144	Blm	-
chr10	1.28E+08	1.28E+08	intron (NM	ID_B1|SINI	-2207	NM_01086	17904	Myl6	ESMLC|LC1
chr2	22923127	22923375	promoter-T	promoter-T	-470	NM_02879	74159	Acbd5	1300014E1
chr4	1.16E+08	1.16E+08	intron (NM	intron (NM	10873	NM_00128	17346	Mknk1	2410048M
chr1	1.36E+08	1.36E+08	exon (NM_	exon (NM_	200	NM_00104	57439	Tmem183a	1300007B1
chr3	1.08E+08	1.08E+08	promoter-T	promoter-T	-7	NM_00117	229725	Clcc1	Mclc
chr19	41975102	41975250	Intergenic	Intergenic	-4533	NM_19944	107094	Rrp12	AA408556|
chr10	40602852	40603000	promoter-T	promoter-T	23	NM_02787	71713	Cdc40	1200003H2
chr10	76978277	76978375	intron (NM	CpG	232	NM_13399	108707	Fam207a	1810008A1
chr3	15331552	15331775	intron (NM	intron (NM	639	NM_00107	751864	Gm9733	-
chr2	1.2E+08	1.2E+08	intron (NM	intron (NM	474	NM_01372	29808	Mga	AV312082|
chr7	27610702	27610800	Intergenic	RLTR45|LT	-9607	NM_00781	13087	Cyp2a5	CYPIIA5|Cc
chr9	61236827	61236900	intron (NM	intron (NM	16690	NM_00108	21887	Tle3	2610103N(
chr9	43921302	43921600	promoter-T	promoter-T	-317	NM_15376	213211	Rnf26	8030450I1
chr11	77940077	77940350	intron (NM	Lx8|LINE|L	32038	NM_14543	216971	Fam222b	9630020I1
chr10	5713102	5713250	intron (NM	intron (NM	21319	NM_00795	13982	Esr1	AA420328|
chr10	1.2E+08	1.2E+08	promoter-T	promoter-T	-520	NM_02867	73914	Irak3	4833428C1
chr9	1.03E+08	1.03E+08	promoter-T	promoter-T	-47	NM_13397	22041	Trf	AI266983|
chr14	26299302	26299450	intron (NM	intron (NM	20705	NM_18320	328365	Zmiz1	BC065120|
chr5	35917827	35918025	promoter-T	promoter-T	-207	NM_03020	78890	Trmt44	2310079F2
chr5	1.44E+08	1.44E+08	promoter-T	promoter-T	-448	NM_02584	66913	Kdelr2	1110007A1
chr11	80290727	80290950	5' UTR (NM	5' UTR (NM	290	NM_00987	12569	Cdk5r1	Cdk5r|D11
chr7	1.09E+08	1.09E+08	intron (NM	intron (NM	45588	NM_00928	20866	Stim1	SIM
chr17	57074877	57075300	promoter-T	promoter-T	-277	NM_02232	64144	Mllt1	AA407901|
chr8	1.23E+08	1.23E+08	promoter-T	promoter-T	386	NM_01092	18117	Emc8	Cox4nb|Fa
chr13	37734252	37734425	Intergenic	MER45B|D	-183569	NM_02683	68750	Rreb1	1110037N(
chr7	16880402	16880600	Intergenic	Intergenic	-8357	NM_00113	243846	Ccdc9	2600011L0
chr7	75278752	75278875	intron (NM	intron (NM	130306	NM_03010	78444	Pgpep1l	C330024D1
chr15	1.02E+08	1.02E+08	promoter-T	promoter-T	-499	NM_14606	223922	Atf7	1110012F1
chr10	6280477	6280650	intron (NM	intron (NM	92903	NM_00117	270685	Mthfd1l	2410004L1
chr4	1.54E+08	1.54E+08	promoter-T	promoter-T	-476	NM_00104	668173	Pex10	AV128229|
chr1	1.92E+08	1.92E+08	exon (NM_	exon (NM_	179	NM_02679	226830	Smyd2	1110020E0
chr5	1.22E+08	1.22E+08	Intergenic	Intergenic	-1553	NM_00850	16923	Sh2b3	AI429800|
chr11	1.19E+08	1.19E+08	5' UTR (NM	5' UTR (NM	192	NM_02889	74370	Rptor	4932417H(
chr4	62185902	62186050	promoter-T	promoter-T	72	NM_02149	59001	Pole3	1810034K1
chr2	26245002	26245175	promoter-T	promoter-T	220	NM_17318	66865	Pmpca	1200002L2
chr1	1.95E+08	1.95E+08	intron (NM	intron (NM	9552	NM_15313	215243	Traf3ip3	6030423D(
chr10	37325477	37325750	Intergenic	Intergenic	-466881	NM_00853	17118	Marcks	Macs|PKCS
chr13	59749402	59749850	intron (NM	intron (NM	27519	NM_00103	105348	Golm1	2310001L0
chr5	25265827	25265925	promoter-T	promoter-T	32	NM_00100	242894	Actr3b	9630005C(
chr10	92546327	92546625	Intergenic	Intergenic	-77145	NM_14623	237459	Cdk17	6430598J1
chr9	64295627	64295725	intron (NM	intron (NM	62243	NM_00113	214058	Megf11	2410080H(
chr7	25630877	25630975	Intergenic	B3|SINE|B	23784	NM_18278	232973	Lypd4	4933400F0
chr1	84931327	84931650	5' UTR (NM	5' UTR (NM	170	NM_02792	71781	Slc16a14	1110004H1
chr11	89993977	89994200	Intergenic	Intergenic	102426	NM_02643	67888	Tmem100	1810057C1
chr1	58052552	58052650	promoter-T	promoter-T	-217	NM_19900	68549	Sgol2	1110007N(

chr11	77327152	77327300	promoter-T	promoter-T	-451	NM_00102	216964	Trp53i13	2410019G0
chr9	57173902	57174175	Intergenic	Intergenic	-63632	NM_02882	74211	1700017B0	AA682102|
chr11	97605352	97605450	promoter-T	promoter-T	617	NM_05405	108083	Pip4k2b	AI848124|
chr2	1.7E+08	1.7E+08	Intergenic	Intergenic	-27738	NM_00115	228913	Zfp217	4933431C0
chr15	98904877	98905050	promoter-T	promoter-T	-441	NM_00116	78733	Troap	AW476063
chr4	1.16E+08	1.16E+08	intron (NM	CpG	1143	NM_15352	230654	Lrrc41	AA409966|
chr2	1.47E+08	1.47E+08	intron (NM	intron (NM	288	NM_00103	228730	Kiz	Gm114|Nc
chr7	1.08E+08	1.08E+08	5' UTR (NM	5' UTR (NM	199	NM_19901	207278	Fchsd2	BC034086|
chr1	1.93E+08	1.93E+08	promoter-T	promoter-T	88	NM_17863	77065	Ints7	5930412E2
chr19	8873202	8873275	promoter-T	promoter-T	-191	NM_02632	67710	Polr2g	2410046K1
chr1	36364952	36365100	intron (NM	CpG	448	NM_00117	214855	Arid5a	D430024K2
chr17	29084027	29084275	Intergenic	Intergenic	-4944	NM_15339	69307	Pxt1	1700001G1
chr14	32063852	32063975	promoter-T	promoter-T	491	NM_02794	71838	Phf7	1700006H0
chr6	41152402	41152525	Intergenic	Intergenic	-99808	NM_01164	22074	Try4	0910001B1
chr1	1.74E+08	1.74E+08	Intergenic	MIRb|SINE	-2736	NM_01091	18071	Nhlh1	Hen1|Nscl
chr8	37185752	37185950	intron (NM	intron (NM	27969	NM_17291	244418	D8Ertd82e	9830148H2
chr3	1.16E+08	1.16E+08	exon (NM_	exon (NM_	175	NM_00128	72776	Sass6	2810453L1
chr1	75475327	75475775	promoter-T	promoter-T	495	NM_00100	74241	Chpf	1700028N0
chr14	15535777	15535850	intron (NM	CpG	274	NM_00103	218756	Slc4a7	E430014N1
chr6	64992552	64993200	promoter-T	promoter-T	215	NM_00795	13990	Smarcad1	AV081750|
chr2	1.06E+08	1.06E+08	promoter-T	promoter-T	119	NM_02387	77766	Elp4	A330107A1
chr2	25463427	25464200	TTS (NM_0	TTS (NM_0	153	NM_00102	227624	Rabl6	B230208H1
chr12	1.04E+08	1.04E+08	promoter-T	promoter-T	-285	NM_17280	238386	Btbd7	5730507E0
chr7	1.29E+08	1.29E+08	promoter-T	promoter-T	-131	NM_00103	233824	Cog7	5630400E2
chr13	1.03E+08	1.03E+08	intron (NM	intron (NM	1596	NM_00107	18708	Pik3r1	PI3K|p50al
chr9	1.08E+08	1.08E+08	TTS (NM_0	TTS (NM_0	1309	NM_02659	68176	Fam212a	6230427J0
chr18	21160752	21160900	intron (NM	CpG-6775	984	NM_20762	56515	Rnf138	2410015A1
chr16	4420577	4420700	promoter-T	promoter-T	-140	NM_00962	11515	Adcy9	ACtp10|AV
chr9	1.08E+08	1.08E+08	promoter-T	promoter-T	515	NM_01680	11848	Rhoa	Arha|Arha1
chr1	1.94E+08	1.94E+08	intron (NM	intron (NM	492	NM_01163	22033	Traf5	-
chr7	39056202	39056375	promoter-T	promoter-T	79	NM_02539	66161	Pop4	1110023P2
chr8	82814377	82814500	Intergenic	Intergenic	-203506	NM_01036	14934	Gypa	AI853584|
chr12	1.03E+08	1.03E+08	promoter-T	promoter-T	-58	NM_01685	51786	Cpsf2	100kDa|26
chr10	80638877	80640725	intron (NM	intron (NM	425	NM_00790	13629	Eef2	Ef-2
chr12	88540552	88540700	promoter-T	promoter-T	553	NM_00103	382620	Tmed8	643059501
chr1	89008627	89008775	Intergenic	Intergenic	-10520	NM_00108	76768	Alpi	2010001C1
chr16	49839227	49839300	Intergenic	Intergenic	-16504	NM_01058	16423	Cd47	9130415E2
chr1	36748002	36748550	promoter-T	promoter-T	-56	NM_00994	12859	Cox5b	-
chr9	1.07E+08	1.07E+08	promoter-T	promoter-T	-526	NM_01971	56289	Rassf1	123F2|AA5
chr9	64658502	64658600	promoter-T	promoter-T	-267	NM_00116	102442	Dennd4a	AI115600|
chr1	92977002	92977125	intron (NM	intron (NM	27042	NM_00851	16978	Lrrfip1	AU024550
chr6	82889902	82889975	promoter-T	promoter-T	-194	NM_00111	20355	Sema4f	-
chr8	1.25E+08	1.25E+08	promoter-T	promoter-T	-609	NM_01391	30927	Snai3	AI643946|
chr16	24120902	24121225	Intergenic	Charlie1b|	-132365	NM_00974	12053	Bcl6	Bcl5
chr1	9872052	9872300	intron (NM	intron (NM	-26543	NM_17772	240697	Mcmdc2	6030422M
chr17	50649227	50649350	intron (NM	CpG	416	NM_01388	224860	Plcl2	PLC-L2|PRI
chrX	12888802	12888925	Intergenic	Intergenic	30715	NM_01002	13205	Ddx3x	D1Pas1-rs2

chr1	75188802	75188925	promoter-T	promoter-T	129	NR_05199	52231	Ankzf1	1300008P0
chr6	1.09E+08	1.09E+08	intron (NM	intron (NM	7691	NM_13867	192193	Edem1	A130059K2
chr1	66885002	66885175	intron (NM	intron (NM	-20919	NM_00112	68691	Kansl1l	1110028C1
chr3	1.46E+08	1.46E+08	promoter-T	promoter-T	291	NM_02761	70951	Spata1	4921536I2
chr2	1.81E+08	1.81E+08	exon (NM_	exon (NM_	240	NM_17376	228994	Ythdf1	2210410K2
chr4	11486077	11486475	intron (NM	CpG	209	NM_00125	623474	Rad54b	E130016E0
chr5	64550677	64551100	promoter-T	promoter-T	-611	NM_01963	57915	Tbc1d1	1110062G0
chr16	87455027	87455100	promoter-T	promoter-T	-167	NM_02425	74112	Usp16	1200004E0
chr1	75138577	75138750	promoter-T	promoter-T	279	NM_02697	69171	Cnppd1	1810031K1
chr3	95755152	95755525	Intergenic	Intergenic	22158	NM_02321	66471	Anp32e	2810018A1
chr16	37769102	37769250	Intergenic	MLT1K|LTF	-7965	NM_00804	14314	Fstl1	AI316791|A
chr11	62062227	62062300	promoter-T	promoter-T	-223	NM_00741	11541	Adora2b	A2BAR|A2I
chr19	35290127	35290375	Intergenic	Intergenic	293403	NM_18304	240641	Kif20b	33cex|B13
chr6	1.35E+08	1.35E+08	intron (NM	intron (NM	1145	NM_13044	70686	Dusp16	3830417M
chr4	1.33E+08	1.33E+08	Intergenic	Intergenic	-1614	NM_00114	230801	Pigv	B330013B0
chr16	38648877	38649325	intron (NM	intron (NM	64047	NM_02026	12549	Arhgap31	5830477L0
chr16	36156552	36156775	promoter-T	promoter-T	-234	NM_17386	268885	Stfa2l1	-
chr1	26630977	26631175	Intergenic	Lx3_Mus|L	113229	NM_00103	210940	4931408C2	-
chr1	57027877	57028075	promoter-T	promoter-T	202	NM_13914	212712	Satb2	mKIAA1034
chr2	1.18E+08	1.18E+08	intron (NM	intron (NM	2076	NM_13831	171543	Bmf	AW260063
chr10	1.17E+08	1.17E+08	intron (NM	CpG-1519	313	NM_01078	17246	Mdm2	1700007J1
chr10	80444302	80444475	intron (NM	intron (NM	19722	NM_00103	14708	Gng7	AI840417
chr5	66087577	66087700	intron (NM	intron (NM	1457	NM_00108	71521	Pds5a	9030416H1
chr15	76554202	76554350	promoter-T	promoter-T	-1	NM_17682	223665	C030006K1	1110033I1
chr2	26456177	26456625	intron (NM	intron (NM	3536	NM_02621	67512	Agpat2	2510002J0
chr19	10257452	10257625	5' UTR (NM	5' UTR (NM	160	NM_14609	76267	Fads1	0710001O0
chr3	1.23E+08	1.23E+08	Intergenic	Intergenic	-13530	NM_00893	19142	Prss12	Bssp-3|mo
chr14	76571127	76571275	Intergenic	Intergenic	60503	NM_01374	27275	Nufip1	Nufip
chr2	13995877	13996175	promoter-T	promoter-T	287	NM_01148	20844	Stam	STAM1
chr7	1.24E+08	1.24E+08	Intergenic	Intergenic	-7445	NM_00113	53322	Nucb2	AI607786|
chrX	1.54E+08	1.54E+08	intron (NM	CpG	484	NM_17230	270669	Mbtps2	9630032G2
chr18	36488627	36488725	Intergenic	Intergenic	-19602	NM_00108	66060	Cystm1	0610010O1
chr2	27744227	27744550	intron (NM	intron (NM	2443	NM_01573	12831	Col5a1	AI413331
chr1	16646627	16647125	promoter-T	promoter-T	70	NM_02645	67923	Tceb1	2610043E2
chr2	90780702	90780975	intron (NM	CpG	284	NM_01736	13046	Celf1	1600010O0
chr1	97469552	97469900	Intergenic	Intergenic	94445	NM_00918	20452	St8sia4	PST|PST-1|
chr3	88521602	88521725	intron (NR_	intron (NR_	887	NM_00116	19769	Rit1	RIBB|ROC1
chr1	1.36E+08	1.36E+08	Intergenic	B3A|SINE|	27171	NM_00114	71001	Mgat4e	4931440L1
chr7	1.25E+08	1.25E+08	intron (NM	MTC|LTR|I	68845	NM_17564	233781	Xylt1	8030490L1
chr9	63247327	63247400	promoter-T	promoter-T	-312	NM_02709	69478	2300009A0	-
chr5	93697077	93697225	intron (NM	CpG	552	NM_00763	12452	Ccng2	-
chr12	74997502	74997675	Intergenic	Intergenic	-11266	NM_01043	15251	Hif1a	AA959795|
chr10	19653302	19653550	promoter-T	promoter-T	-906	NM_00858	26408	Map3k5	7420452D2
chr1	1.8E+08	1.8E+08	Intergenic	Intergenic	-36330	NM_00116	269152	Kif26b	4832420M
chr2	1.45E+08	1.45E+08	intron (NM	intron (NM	-103039	NM_02549	66328	Scp2d1	1700010M
chr1	52712152	52712350	TTS (NM_1	TTS (NM_1	24702	NM_00114	227094	Tmem194b	5330401P0
chr1	75212727	75212875	TTS (NM_0	TTS (NM_0	3027	NM_00944	22145	Tuba4a	M[a]4|Tub

chr7	1.35E+08	1.35E+08	intron (NM	L1M2|LINE	26697	NM_00840	16409	Itgam	CD11b/CD1
chr6	1.35E+08	1.35E+08	Intergenic	Intergenic	32952	NM_00128	13730	Emp1	I-8-09|TMF
chr1	21764227	21764350	intron (NM	intron (NM	187735	NM_00116	226922	Kcnq5	7730402H1
chr10	21696227	21696400	intron (NM	intron (NM	-2165	NM_00116	20393	Sgk1	Sgk
chr13	16417552	16417850	Intergenic	Intergenic	311393	NM_00838	16323	Inhba	-
chr3	1.27E+08	1.27E+08	5' UTR (NM	5' UTR (NM	232	NM_02780	71481	Alpk1	8430410J1
chr5	1.35E+08	1.35E+08	exon (NM_	exon (NM_	311	NM_03357	94254	Wbscr16	5730496C0
chr5	1.4E+08	1.4E+08	TTS (NR_03	TTS (NR_03	317	NM_03025	80290	Gpr146	BC003323|
chr7	1.4E+08	1.4E+08	intron (NM	intron (NM	65512	NM_02960	76429	Lhpp	2310007H0
chr19	7131252	7131400	promoter-T	promoter-T	68	NM_13414	107227	Macrod1	AI604841|/
chrY	2895127	2895250	Intergenic	L1Md_F2|L	507930	NM_00127	1E+08	Gm3376	Rbmy1b
chr18	44552377	44552450	intron (NM	intron (NM	12259	NM_02749	70640	Dcp2	2410015D2
chr1	10245377	10245625	Intergenic	Intergenic	-22750	NM_00110	211673	Arfgef1	ARFGEP1|E
chr3	30355002	30355100	intron (NM	intron (NM	53358	NM_02144	14013	Mecom	D630039M
chr15	9794577	9794675	Intergenic	Intergenic	-116065	NM_17712	320277	Spef2	C230086A0
chr2	81846102	81846250	Intergenic	MLT1F2|LT	-47639	NM_17551	241514	Zfp804a	C630007C1
chr4	1.28E+08	1.28E+08	promoter-T	promoter-T	-545	NM_00108	230761	Zfp362	-
chr13	81850602	81850800	promoter-T	promoter-T	285	NM_02837	72852	Mblac2	2900024O1
chr2	66094852	66095000	promoter-T	promoter-T	-252	NM_00129	73668	Ttc21b	2410066K1
chr5	1.01E+08	1.01E+08	promoter-T	promoter-T	73	NM_02682	68735	Mrps18c	1110037D1
chr1	1.33E+08	1.33E+08	intron (NM	CpG	282	NM_00855	17164	Mapkapk2	AA960234|
chr1	1.73E+08	1.73E+08	intron (NM	intron (NM	5846	NM_01018	14131	Fcgr3	CD16
chrY	1751752	1751875	Intergenic	Intergenic	-103196	NM_00111	1E+08	Gm16501	ENSMUSG0
chr5	38429902	38430025	promoter-T	promoter-T	-506	NM_00128	71116	Stx18	1810035L2
chr7	86747077	86747175	intron (NM	MTD|LTR|I	15417	NM_01979	56315	Rhcg	BB065800
chr8	1.24E+08	1.24E+08	exon (NM_	exon (NM_	118	NM_14560	234825	Klhdc4	AA408426|
chr15	97923652	97923800	5' UTR (NM	5' UTR (NM	274	NM_14485	223870	Senp1	2310046A2
chr15	1.01E+08	1.01E+08	Intergenic	Intergenic	-19676	NM_01044	15370	Nr4a1	GFRP1|Gfr
chr10	53127252	53127525	Intergenic	Intergenic	-27731	NM_00120	1E+08	Cep85l	ENSMUSG0
chr19	47481052	47481225	intron (NM	ID_B1|SINE	57763	NM_00116	14218	Sh3pxd2a	2310014D1
chr13	1.01E+08	1.01E+08	5' UTR (NM	5' UTR (NM	127	NM_01135	20365	Serf1	4F5|Msma
chr4	1.34E+08	1.34E+08	5' UTR (NM	5' UTR (NM	692	NM_20723	230815	Man1c1	AI593348
chr3	1.36E+08	1.36E+08	intron (NM	intron (NM	34271	NM_00129	19055	Ppp3ca	2900074D1
chr1	1.29E+08	1.29E+08	Intergenic	(TATATG)n	-5604	NM_17695	210356	Nckap5	8430408F2
chr17	35137802	35138400	5' UTR (NM	5' UTR (NM	249	NM_01169	22321	Vars	Bat6|D17H
chr8	1.13E+08	1.13E+08	intron (NM	intron (NM	7062	NM_17228	234730	Fuk	1110046B1
chr16	36071802	36071900	promoter-T	promoter-T	105	NM_00115	381045	Ccdc58	A930007B1
chr3	9828802	9828950	intron (NM	intron (NM	4803	NM_05318	94212	Pag1	Cbp|F7300
chr4	59465502	59465625	Intergenic	RLTR40|LT	-14058	NM_00116	634731	Susd1	A530080P1
chr4	1.07E+08	1.07E+08	Intergenic	Intergenic	-3463	NM_02835	72787	Ndc1	2810475A1
chr11	59016127	59016200	promoter-T	promoter-T	144	NM_02603	67212	Mrpl55	2810038N0
chr2	3459052	3459275	Intergenic	Intergenic	28826	NM_17764	227526	Cdnf	9330140G2
chr2	1.15E+08	1.15E+08	promoter-T	promoter-T	24	NM_00114	399568	BC052040	-
chr4	1.16E+08	1.16E+08	intron (NM	CpG	194	NM_02986	77110	Gpbp1l1	5330440M
chr10	90970352	90970825	Intergenic	MLT1J1|LT	-336224	NM_00108	21917	Tmpo	5630400D2
chr7	1.43E+08	1.43E+08	Intergenic	Intergenic	-59526	NM_00108	17345	Mki67	D630048A1
chr11	1.06E+08	1.06E+08	promoter-T	promoter-T	186	NM_02807	72047	Ddx42	1810047H2

chr16	14162377	14162500	promoter-T	promoter-T	-930	NM_02331	67203	Nde1	2810027M
chr12	66137277	66137800	intron (NM	CpG	217	NM_17780	328110	Prpf39	Srcs1
chr15	98661977	98662200	promoter-T	promoter-T	-149	NM_01678	19082	Prkag1	AA571379|
chr4	1.33E+08	1.33E+08	Intergenic	MIR|SINE|	27330	NM_01695	15331	Hmgn2	HMG-17|H
chr10	59740702	59740775	intron (NM	intron (NM	362	NM_00114	19156	Psap	AI037048|
chr3	1.06E+08	1.06E+08	exon (NM_	exon (NM_	245	NM_00127	72121	Dennd2d	2010308M
chrX	68540602	68540700	intron (NM	intron (NM	72394	NM_01992	17772	Mtm1	AF073996|
chr6	50546427	50546625	promoter-T	promoter-T	63	NM_02756	70821	4921507PC	TISP74
chr9	31194127	31194250	intron (NM	CpG	411	NM_17276	235134	Nfrkb	A530090G1
chr2	72875577	72875925	Intergenic	MIR|SINE|	-57248	NM_00101	20687	Sp3	D130027J0
chr8	1.11E+08	1.11E+08	intron (NM	intron (NM	8394	NM_00749	11906	Zfhx3	A230102L0
chr8	34769977	34770050	intron (NM	PB1D7|SIN	6303	NM_01034	14782	Gsr	AI325518|
chrX	1.31E+08	1.31E+08	intron (NM	CpG	208	NM_01986	19982	Rpl36a	L44L|Rpl44
chr17	24328452	24328775	intron (NM	B3|SINE|B	8289	NM_00116	224617	Tbc1d24	9630033P1
chr16	4886452	4886575	intron (NM	CpG	413	NM_00125	17237	Mgrn1	2610042J2
chr15	38943477	38943600	promoter-T	promoter-T	724	NM_17240	69906	Slc25a32	2610043O1
chr7	1.38E+08	1.38E+08	promoter-T	promoter-T	89	NM_13394	101476	Plekha1	AA960558|
chr5	1.08E+08	1.08E+08	promoter-T	promoter-T	-215	NM_00125	1.01E+08	Btbd8	EG627196|
chr7	63458477	63458600	intron (NM	intron (NM	-36603	NM_02187	18431	Oca2	D7H15S12
chr11	49063627	49063850	promoter-T	promoter-T	-316	NM_00111	17308	Mgat1	Mgat-1
chr11	83567152	83567425	exon (NM_	exon (NM_	149	NM_14543	217026	Heatr6	2700008B1
chr2	25128052	25128275	promoter-T	promoter-T	177	NM_17530	99152	Anapc2	9230107K0
chr15	96474002	96474150	promoter-T	promoter-T	-732	NM_00116	105727	Slc38a1	AA408026|
chr2	26771052	26771225	promoter-T	promoter-T	-803	NM_01367	20931	Surf2	Surf-2
chr5	91118677	91118975	Intergenic	Intergenic	-49313	NM_02847	73246	Rassf6	1600016B1
chr1	1.52E+08	1.52E+08	intron (NM	intron (NM	-1330	NM_13378	108989	Tpr	2610029M
chr10	80041002	80041100	Intergenic	Intergenic	-1010	NM_07847	118445	Klf16	AI843742|
chr13	43345827	43345900	intron (NM	intron (NM	53678	NM_00103	328232	Gfod1	9630032O1
chr11	60692827	60693000	promoter-T	promoter-T	-373	NM_00116	216821	Tmem11	5730466P1
chr5	92797252	92797450	intron (NM	ID4|SINE|I	-5029	NR_03811	56066	Cxcl11	Cxc11|H17
chr7	1.13E+08	1.13E+08	exon (NM_	exon (NM_	239	NM_01142	20597	Smpd1	A-SMase|A
chr1	89724877	89725075	exon (NM_	exon (NM_	24721	NM_00911	20215	Sag	A930001K1
chr18	6753502	6753625	Intergenic	Lx8|LINE|L	-11602	NM_18107	19330	Rab18	AA959686
chr12	1.05E+08	1.05E+08	Intergenic	Intergenic	39591	NM_00125	20700	Serpina1a	Aat-2|Aat2
chr2	83582952	83583075	intron (NM	B3|SINE|B	18459	NM_00840	16410	Itgav	1110004F1
chr14	27350727	27350950	intron (NM	intron (NM	2388	NM_03200	83997	Slmap	D330001L0
chr11	78798802	78798900	promoter-T	promoter-T	-425	NM_01070	16859	Lgals9	AA407335|
chr7	54968552	54968750	Intergenic	RMER5|LTI	-111909	NM_15306	233222	Mrgpra3	Mrga3
chr12	8928927	8929175	exon (NM_	exon (NM_	938	NM_00864	17775	Laptm4a	AA286466|
chr9	1.07E+08	1.07E+08	intron (NM	intron (NM	6832	NM_17890	102626	Mapkapk3	3PK|AI874
chr10	29033077	29033225	promoter-T	promoter-T	-155	NM_02585	52665	Echdc1	1700028A2
chr3	1.42E+08	1.42E+08	intron (NM	CpG	622	NM_00119	56376	Pdlim5	1110001A0
chr1	14299552	14299675	intron (NM	intron (NM	667	NM_00125	14048	Eya1	bor
chr2	1.27E+08	1.27E+08	promoter-T	promoter-T	-105	NM_02322	66552	Sppl2a	2010106G0
chr3	1.18E+08	1.18E+08	exon (NM_	exon (NM_	128	NM_00119	76561	Snx7	2510028H0
chr1	79772452	79772925	promoter-T	promoter-T	95	NM_00108	69163	Mrpl44	1810030E1
chr2	73374202	73374550	Intergenic	MTEa-int|L	-6832	NM_15313	215280	Wipf1	AI115543|

chr9	31088852	31089150	Intergenic	Intergenic	10015	NM_00110	638580	Gm7244	EG638580
chr15	57907702	57907800	5' UTR (NM	5' UTR (NM	312	NM_00104	22770	Zhx1	-
chr1	34542327	34542975	Intergenic	Intergenic	-12624	NM_00935	21755	Prss39	Tesp1
chr2	1.81E+08	1.81E+08	non-coding	non-coding	111	NM_00127	13002	Dnajc5	2610314I2
chr4	57598277	57598500	intron (NM	intron (NM	17268	NM_17286	242481	Palm2	53-H12|B1
chr7	16973127	16973375	promoter-T	promoter-T	-6	NM_01974	56459	Sae1	2400010M
chr12	52930052	52930450	5' UTR (NM	5' UTR (NM	272	NM_14478	207304	Hectd1	A630086P0
chr14	55713852	55714075	5' UTR (NM	5' UTR (NM	342	NM_15308	105663	Thtpa	AW122966
chr2	3563102	3563225	Intergenic	Intergenic	-67567	NM_02562	66540	Fam107b	3110001A1
chr2	98779302	98779425	Intergenic	Intergenic	1471549	NM_17872	241568	Lrrc4c	6430556C1
chr7	51655527	51655875	TTS (NM_0	TTS (NM_0	16205	NM_01666	20981	Syt3	AI385753|
chr13	13651052	13651525	Intergenic	Intergenic	-31388	NM_01074	17101	Lyst	D13Sfk13|
chr10	31329327	31329550	promoter-T	promoter-T	93	NM_00114	268291	Rnf217	AU016819
chrX	1.03E+08	1.03E+08	intron (NM	intron (NM	6312	NM_20582	279572	Tlr13	AI666735|
chr18	67529677	67529950	Intergenic	Intergenic	-20572	NM_02647	67951	Tubb6	2310057H1
chr6	1.29E+08	1.29E+08	Intergenic	CpG	-10579	NM_00115	17059	Klrb1c	AI462337|
chr3	95086227	95086475	promoter-T	promoter-T	-353	NM_17334	229589	Prune	9230112O0
chr6	1.49E+08	1.49E+08	promoter-T	promoter-T	-222	NM_00125	320204	Mettl20	4833442J1
chr19	6364227	6364425	intron (NR_	CpG	636	NR_12211	22668	Sf1	BBP|MZFM
chr11	90102052	90102225	Intergenic	Intergenic	-8652	NM_02617	67468	Mmd	1200017E0
chr9	1.22E+08	1.22E+08	promoter-T	promoter-T	-541	NM_17867	215474	Sec22c	4932412K2
chr1	1.07E+08	1.07E+08	5' UTR (NM	5' UTR (NM	71861	NM_17877	320311	Rnf152	A930029B0
chr2	71337977	71338250	intron (NM	intron (NM	-29389	NM_01005	13390	Dlx1	Dlx|Dlx-1
chr7	1.34E+08	1.34E+08	intron (NM	B1_Mus1|	7230	NM_02320	66422	Dctpp1	2410015N1
chr1	30929727	30929925	Intergenic	Intergenic	-9725	NM_00108	213109	Phf3	2310061N1
chr12	87046702	87046875	intron (NM	intron (NM	19118	NM_01676	53314	Batf	B-ATF|SFA
chr10	40244227	40244475	Intergenic	Intergenic	-45817	NM_02757	70840	Slc22a16	4921504E1
chr17	79365577	79365925	exon (NM_	exon (NM_	29276	NM_02861	73694	Ndufaf7	2410091C1
chr3	1.06E+08	1.06E+08	intron (NM	intron (NM	10068	NM_02811	72121	Dennd2d	2010308M
chr11	79997977	79998175	intron (NM	intron (NM	702	NM_02801	71956	Rnf135	0610037N0
chr3	1.16E+08	1.16E+08	promoter-T	promoter-T	211	NM_02321	66500	Slc30a7	1810059J1
chr19	53406602	53406875	intron (NM	intron (NM	1802	NM_00100	17859	Mxi1	ENSMUSG0
chr4	62069877	62069950	promoter-T	promoter-T	96	NM_02729	70052	Prpf4	1600015H1
chr16	17181477	17181725	intron (NM	L3|LINE|CF	19984	NM_00945	22195	Ube2l3	C79827|Ub
chr6	39542352	39542475	promoter-T	promoter-T	-169	NM_02661	68198	Ndufb2	1810011O0
chr1	1.3E+08	1.3E+08	intron (NM	intron (NM	-26930	NM_00856	17219	Mcm6	ASP-l1|D1V
chr3	1.33E+08	1.33E+08	Intergenic	Intergenic	-79009	NM_00104	214133	Tet2	Ayu17-449
chr7	26434902	26435100	intron (NM	intron (NM	8779	NM_00753	12039	Bckdha	-
chr7	1.38E+08	1.38E+08	exon (NM_	exon (NM_	344	NM_00116	67872	Nsmce4a	2410003A1
chr3	94820677	94820775	Intergenic	Intergenic	-1566	NM_03007	78266	Zfp687	4931408L0
chr7	1.07E+08	1.07E+08	Intergenic	Intergenic	-9158	NM_17538	108937	Rnf169	2900057K0
chr8	1.03E+08	1.03E+08	Intergenic	L1M4c|LIN	-850342	NM_00103	12564	Cdh8	AI851472|
chr16	59472802	59472950	intron (NM	intron (NM	1275	NM_02591	67014	Mina	1810047J0
chr8	47619302	47619650	intron (NM	intron (NM	-17947	NM_02797	71876	Cenpu	1700029A2
chr3	95119902	95120050	intron (NM	intron (NM	802	NM_02978	76893	Cers2	0610013I1
chr17	3104202	3104375	Intergenic	Intergenic	-10684	NM_13412	106583	Scaf8	A630086M
chr16	32130352	32130700	Intergenic	Intergenic	30638	NM_02589	66994	Cep19	1500031L0

chr9	44774177	44774475	promoter-T	promoter-T	-643	NM_14540	140630	Ube4a	4732444G1
chr4	1.29E+08	1.29E+08	Intergenic	CpG	-9560	NM_00897	19244	Ptp4a2	Prl-2
chr4	43617902	43618175	Intergenic	Intergenic	-20672	NM_00109	1E+08	Msmp	OTTMUSG(
chr7	13551627	13551725	intron (NM	intron (NM	463	NM_17873	243834	Zfp324	A930002M
chr9	44075502	44075625	intron (NM	intron (NM	707	NM_00116	270151	Nlrx1	BC034204|
chr17	31648852	31649325	intron (NM	CpG	344	NM_02132	57773	Wdr4	AI415180|/
chr13	1.12E+08	1.12E+08	intron (NM	CpG	386	NM_02848	73274	Gpbp1	1700034P1
chr7	1.41E+08	1.41E+08	intron (NM	intron (NM	47892	NM_00740	11489	Adam12	Mltna|mKI
chr12	1.1E+08	1.1E+08	promoter-T	promoter-T	-895	NM_00953	22632	Yy1	AW488674
chr11	1.07E+08	1.07E+08	intron (NM	intron (NM	42696	NM_14582	71795	Pitpnc1	1110020B(
chr3	41935677	41935800	Intergenic	Intergenic	389198	NM_02727	73852	D3Ertd751	2810009O1
chr8	4324452	4325025	promoter-T	promoter-T	362	NM_01048	15568	Elavl1	2410055N(
chr1	87600302	87600800	intron (NM	intron (NM	32630	NM_17705	319997	A630001G2	-
chr12	1.12E+08	1.12E+08	exon (NM_	exon (NM_	164	NM_01202	26931	Ppp2r5c	2610043M
chr10	85334677	85334800	exon (NM_	exon (NM_	162	NM_13399	103136	Pwp1	2310058A1
chr19	3388827	3389300	promoter-T	promoter-T	206	NM_00103	17771	Mtl5	tesmin
chr6	97155027	97155150	intron (NM	intron (NM	548	NM_00111	22200	Uba3	A830034N(
chr9	55103452	55103775	intron (NM	intron (NM	27819	NM_03200	83961	Nrg4	AI552600
chr2	1.54E+08	1.54E+08	5' UTR (NM	5' UTR (NM	114	NM_02936	75608	Chmp4b	2010012F0
chr3	86812352	86812575	Intergenic	RLTR13D3|	-9201	NM_00763	12479	Cd1d1	AI747460|(
chr7	31702352	31702500	Intergenic	MLT1B|LTF	-2575	NM_01075	17136	Mag	Gma|siglec
chr13	36055602	36055825	Intergenic	MER34-int	-4062	NM_02962	76487	Ppp1r3g	1600032L1
chr6	40968727	40968800	Intergenic	ORR1E-int|	16745	NM_02333	67373	2210010C(	AV072249
chr7	29997177	29997425	intron (NM	intron (NM	1751	NM_00116	320225	Catsperg1	A230107C(
chrX	96331702	96331825	5' UTR (NM	5' UTR (NM	294	NM_01011	13641	Efnb1	Cek5-L|EFL
chr6	83275677	83275925	promoter-T	promoter-T	-232	NM_14557	232157	Mob1a	4022402H(
chr9	1.18E+08	1.18E+08	intron (NM	CpG	523	NM_01372	27215	Azi2	AA410145|
chr6	1.25E+08	1.25E+08	promoter-T	promoter-T	52	NM_00108	22317	Vamp1	Syb-1|Syb1
chr12	31786427	31786975	Intergenic	Lx6|LINE|L	27868	NM_02782	104943	Fam110c	9030611O1
chr8	32278127	32278250	intron (NM	L2|LINE|L2	1008	NM_02645	67920	Mak16	2600016B(
chr17	80482677	80482800	Intergenic	Intergenic	-21130	NM_14480	72692	Hnrnpll	2510028H(
chr4	1.36E+08	1.36E+08	5' UTR (NM	5' UTR (NM	167	NM_00955	22704	Zfp46	AI426962|
chr1	92499477	92499600	promoter-T	promoter-T	-462	NM_13380	108679	Cops8	9430009J0
chr3	90393102	90393300	promoter-T	promoter-T	-968	NM_00119	628324	S100a2	CaN19|EG(
chr4	49251777	49251950	intron (NM	intron (NM	169160	NM_14536	209186	Acnat2	C730036D1
chr14	52639777	52639950	promoter-T	promoter-T	-475	NM_00125	69890	Zfp219	2010302A1
chr10	1.2E+08	1.2E+08	promoter-T	promoter-T	-603	NM_02543	66225	Llph	1190005P1
chr4	1.33E+08	1.33E+08	Intergenic	CpG	-60262	NM_00108	93760	Arid1a	1110030E(
chr6	1.2E+08	1.2E+08	Intergenic	ORR1E|LTF	-8327	NM_00835	16172	Il17ra	AW538159
chr10	67061477	67061650	Intergenic	Intergenic	-49860	NM_00100	211488	Ado	Gm237
chr19	39005327	39005475	promoter-T	promoter-T	-79	NM_00823	15201	Hells	AI323785|
chr1	1.09E+08	1.09E+08	intron (NM	CpG	464	NM_00919	20479	Vps4b	8030489C1
chr5	97212552	97212650	3' UTR (NM	3' UTR (NM	-9803	NM_01347	11745	Anxa3	Anx3
chr19	5118027	5118225	non-coding	non-coding	282	NR_045528	16594	Klc2	8030455F0
chr5	74590902	74591250	promoter-T	promoter-T	-275	NM_02687	68939	Rasl11b	1190017B1
chr16	36985752	36985875	Intergenic	ORR1D1|LT	-6591	NM_00103	207215	Fbxo40	9830003A1
chr1	97236252	97236425	Intergenic	RMER19B|	26133	NM_02632	67698	Fam174a	2310044D2

chr7	30601452	30601550	intron (NM	intron (NM	410	NM_00114	233056	Zfp790	6330581L2
chr10	17241827	17242000	Intergenic	Intergenic	-201121	NM_01082	17684	Cited2	AI835299|I
chr6	53401952	53402050	Intergenic	Intergenic	-121367	NM_17272	231991	Creb5	Crebpa|D4
chr7	1.51E+08	1.51E+08	Intergenic	Intergenic	9772	NM_20349	381974	Mrgprg	Gm1098|M
chr1	72190852	72191375	Intergenic	RLTR19|LT	67768	NM_00100	381269	Mreg	Gm974|W
chr3	1.21E+08	1.21E+08	Intergenic	Intergenic	-15751	NM_14539	213603	Slc44a3	BC010552
chr1	1.84E+08	1.84E+08	Intergenic	Intergenic	-15855	NM_01205	27058	Srp9	9kDa
chr9	54582127	54582225	5' UTR (NM	5' UTR (NM	180	NM_02319	66317	Wdr61	2700038L1
chr15	77786927	77787400	promoter-T	promoter-T	-11	NM_00101	239554	Foxred2	A430097D0
chr10	80598377	80598500	promoter-T	promoter-T	-578	NM_01073	16969	Zbtb7a	9030619K0
chr17	80952527	80952850	intron (NM	intron (NM	10486	NM_00103	381113	Cdkl4	AU067824
chr1	37486452	37486600	promoter-T	promoter-T	422	NM_19800	76178	Coa5	1700001A2
chr9	64584227	64584450	intron (NM	intron (NM	1225	NM_01738	53869	Rab11a	-
chr10	66999002	66999075	Intergenic	CpG	-1579	NM_01011	13654	Egr2	Egr-2|Krox
chr10	20301177	20301325	intron (NM	intron (NM	143623	NM_01387	29863	Pde7b	-
chr1	62726052	62726325	Intergenic	RMER10A|	-23703	NM_00107	18187	Nrp2	1110048P0
chr18	39180252	39180375	intron (NM	intron (NM	27514	NM_17516	71302	Arhgap26	1810044B2
chr1	1.79E+08	1.79E+08	Intergenic	Intergenic	44011	NM_01391	30928	Zbtb18	RP58|Zfp2
chr14	47451827	47452100	promoter-T	promoter-T	44	NM_02683	68755	Cgrrf1	1110038G0
chr13	1.14E+08	1.14E+08	intron (NM	intron (NM	6362	NM_02815	72198	Skiv2l2	2610528A1
chr11	51394902	51395150	3' UTR (NM	3' UTR (NM	-3233	NM_02839	72947	Phykpl	2900006B1
chr3	85375302	85375475	Intergenic	Intergenic	-2663	NM_14489	229487	Pet112	9430026F0
chr8	1.08E+08	1.08E+08	5' UTR (NM	5' UTR (NM	125	NM_18132	13018	Ctcf	AW108038
chr11	1.03E+08	1.03E+08	intron (NM	intron (NM	3411	NM_01967	57778	Fmnl1	8030453N1
chr9	1.14E+08	1.14E+08	intron (NM	intron (NM	7156	NM_17862	72179	Fbxl2	2810423A2
chr2	69699752	69700175	intron (NM	CpG	344	NM_00927	20823	Ssb	SS-B
chr15	76991327	76991625	intron (NM	intron (NM	-7234	NM_00128	93686	Rbfox2	Fbm2|Fxh|
chr13	1.13E+08	1.13E+08	Intergenic	Intergenic	-209722	NM_01194	26401	Map3k1	MAPKKK1|
chr11	78364327	78364725	promoter-T	promoter-T	-289	NM_13370	69071	Tmem97	1810014L1
chr4	82505352	82505525	5' UTR (NM	5' UTR (NM	127	NM_02664	68268	Zdhhc21	9130404H1
chr10	57961352	57961500	intron (NM	intron (NM	741	NM_00116	76138	Ccdc138	6230424H0
chrX	1.37E+08	1.37E+08	intron (NM	CpG	646	NM_02146	19139	Prps1	2310010D1
chr7	87484202	87484350	intron (NM	intron (NM	829	NM_13395	101869	Unc45a	AW538196
chr11	28297377	28297500	intron (NM	intron (NM	186886	NM_00116	216613	Ccdc85a	-
chr12	74685727	74685975	promoter-T	promoter-T	-177	NM_00885	18755	Prkch	Pkch
chr3	96025402	96025550	promoter-T	promoter-T	432	NM_17821	319190	Hist2h2be	AV127319|
chr4	1.18E+08	1.18E+08	intron (NM	intron (NM	460	NM_02322	107995	Cdc20	2310042N0
chr9	34412552	34412850	intron (NM	intron (NM	116385	NM_00119	67703	Kirrel3	1500010O2
chr16	33966852	33967300	promoter-T	promoter-T	13	NM_00947	22247	Umps	1700095D2
chr14	49792052	49792225	intron (NM	G-rich|Low	236	NM_00108	70646	Naa30	4930487N1
chr7	52124777	52124925	exon (NM_	exon (NM_	307	NM_13394	84113	Ptov1	1110030G0
chr10	59342377	59342700	exon (NM_	exon (NM_	199	NM_01996	56709	Dnajb12	Dj10|mDj1
chr15	76779327	76779625	intron (NM	intron (NM	502	NM_05409	117171	1110038F1	-
chr6	67485227	67485350	exon (NM_	exon (NM_	528	NM_02004	56753	Tacstd2	C80403|EG
chr16	31080902	31081150	exon (NM_	exon (NM_	492	NM_19862	268880	Xxylt1	AI480653
chr12	74213702	74213850	exon (NM_	exon (NM_	456	NM_01138	20474	Six4	AI047561|A
chr1	63522202	63522450	intron (NM	intron (NM	29848	NM_01178	23792	Adam23	AW046396

chr2	90910252	90910625	promoter-T	promoter-T	34	NM_02672	68427	Slc39a13	1100001L1
chr2	1.26E+08	1.26E+08	intron (NM	intron (NM	18026	NM_00823	15186	Hdc	AW108189
chr4	46828177	46828425	intron (NM	intron (NM	-165230	NM_19866	381605	Tbc1d2	A630005A(
chr8	1.09E+08	1.09E+08	intron (NM	intron (NM	18420	NM_17303	272538	Tango6	AW413431
chr13	91364102	91364300	promoter-T	promoter-T	-609	NM_02577	66795	Atg10	5330424L2
chr1	1.82E+08	1.82E+08	Intergenic	Intergenic	-33645	NM_00112	19165	Psen2	ALG-3|Ad4
chr6	1.13E+08	1.13E+08	promoter-T	promoter-T	263	NM_13393	101206	Tada3	1110004B1
chr10	53317077	53317350	intron (NM	CpG	446	NM_02554	66403	Asf1a	2310079C1
chr7	19851502	19851575	intron (NM	intron (NM	5665	NM_00128	22323	Vasp	-
chr11	68936077	68936200	exon (NM_	exon (NM_	2183	NM_03304	84653	Hes7	bHLHb37
chr13	59343352	59343650	Intergenic	Intergenic	302311	NM_00128	67269	Agtpbp1	1700020N1
chr15	98314802	98315000	Intergenic	Intergenic	-1787	NM_01067	16770	Lalba	AW208827
chr3	1.23E+08	1.23E+08	intron (NM	CpG	477	NM_13385	99526	Usp53	AA939927|
chr6	1.3E+08	1.3E+08	intron (NM	(TA)n|Simp	-6092	NM_00845	16628	Klra10	Ly49J|Ly49
chr1	1.53E+08	1.53E+08	Intergenic	MTD|LTR|I	-73640	NM_00103	117198	Ivns1abp	1190004M
chr4	1.34E+08	1.34E+08	promoter-T	promoter-T	-695	NM_01695	15331	Hmgn2	HMG-17|H
chr5	1.47E+08	1.47E+08	Intergenic	Intergenic	17301	NM_00125	74132	Rnf6	1200013I0
chr9	1.09E+08	1.09E+08	exon (NM_	exon (NM_	1225	NM_08043	107934	Celsr3	Fmi1|flami
chr7	1.13E+08	1.13E+08	TTS (NM_0	TTS (NM_0	1209	NM_01950	14356	Timm10b	Fxc1|Tim1(
chr5	1.45E+08	1.45E+08	Intergenic	Intergenic	-1129	NM_17768	231874	Ccz1	AU022870
chr11	54785552	54785650	Intergenic	Intergenic	-9159	NM_00127	57783	Tnip1	ABIN|ABIN
chr4	1.36E+08	1.36E+08	5' UTR (NM	5' UTR (NM	367	NM_00955	22704	Zfp46	AI426962|
chr8	13105702	13105850	promoter-T	promoter-T	55	NM_14620	99375	Cul4a	2810470J2
chr16	70314152	70314550	5' UTR (NM	5' UTR (NM	157	NM_02880	74185	Gbe1	2310045H1
chr9	1.1E+08	1.1E+08	intron (NM	intron (NM	305	NM_01875	54369	Nme6	nm23-M6
chr9	1.08E+08	1.08E+08	intron (NM	CpG	187	NM_01125	19654	Rbm6	4930506F1
chr9	1.01E+08	1.01E+08	intron (NM	B3|SINE|B	107193	NM_02583	66904	Pccb	1300012P0
chr9	64880102	64880600	promoter-T	promoter-T	86	NM_02890	74388	Dpp8	2310004I0
chr11	87603377	87603800	Intergenic	RLTR33|LT	-3698	NM_01082	17523	Mpo	mKIAA403
chr11	53317527	53317600	Intergenic	Intergenic	-15675	NM_03314	20362	8-Sep	AW046166
chr10	1.27E+08	1.27E+08	Intergenic	HAL1|LINE	38485	NM_02730	70061	Sdr9c7	1810054F2
chr12	81155427	81155500	Intergenic	L3|LINE|CF	58537	NM_00756	12192	Zfp36l1	AW742437
chr6	1.25E+08	1.25E+08	Intergenic	Intergenic	39885	NM_00765	12527	Cd9	Tspan29
chr15	36211702	36211800	intron (NM	intron (NM	1151	NM_01392	30945	Rnf19a	AA032313|
chr3	88357327	88357400	promoter-T	promoter-T	-275	NM_03352	94232	Ubqln4	A1Up|A1u
chr8	84907327	84907600	intron (NM	intron (NM	19022	NM_00125	16168	Il15	AI503618|
chr8	93372027	93372300	intron (NM	intron (NM	19429	NM_17722	109151	Chd9	1810014J1
chr8	18950052	18950450	5' UTR (NM	5' UTR (NM	620	NM_00128	319581	Xkr5	5430438H(
chr4	76178802	76179075	intron (NM	intron (NM	-1254748	NM_02584	66928	Tmem261	1700027K2
chr12	86470452	86470900	intron (NM	intron (NM	18893	NM_03022	78920	Dlst	1600017E0
chr2	1.19E+08	1.19E+08	promoter-T	promoter-T	-158	NM_13385	108907	Nusap1	2610201A1
chr1	88255152	88255500	promoter-T	promoter-T	704	NM_01088	17975	Ncl	B530004O1
chr1	1.45E+08	1.45E+08	intron (NM	ORR1E|LTF	-7339	NM_02002	26878	B3galt2	-
chr7	52003477	52003600	promoter-T	promoter-T	181	NM_00128	243963	Zfp473	D030014N
chr2	1.21E+08	1.21E+08	TTS (NM_1	TTS (NM_1	157	NM_01076	17151	Ccndbp1	AU022347
chr2	1.48E+08	1.48E+08	Intergenic	ORR1E|LTF	-2100	NM_00921	20608	Sstr4	Smstr4|sst
chr3	89084252	89084425	promoter-T	promoter-T	535	NM_00116	13636	Efna1	AI325262|

chr6	1.47E+08	1.47E+08	Intergenic	Intergenic	-3963	NM_02531	108098	Med21	0610007L0
chr19	21799577	21799975	Intergenic	RCHARR1|I	-53054	NM_00103	83921	Tmem2	-
chr6	1.37E+08	1.37E+08	exon (NM_	exon (NM_	2962	NM_17877	320135	BC049715	-
chr13	22073427	22073625	exon (NM_	exon (NM_	463	NM_00116	195236	Pom121l2	Gm24
chr1	1.33E+08	1.33E+08	promoter-T	promoter-T	-421	NM_01875	54354	Rassf5	1300019G2
chr5	3491877	3492125	intron (NM	intron (NM	-51832	NM_00104	68152	Fam133b	2900022KC
chr17	71606152	71606500	intron (NM	intron (NM	52332	NM_00116	64898	Lpin2	2610511GC
chr16	56037677	56037925	promoter-T	promoter-T	86	NM_02909	52575	Trmt10c	1300018J1
chr1	94077152	94077350	Intergenic	CpG	-32281	NM_20722	208727	Hdac4	4932408F1
chr5	32438477	32438675	promoter-T	promoter-T	-269	NM_00803	14284	Fosl2	Fra-2
chr7	1.26E+08	1.26E+08	exon (NM_	exon (NM_	101	NM_01958	56209	Gde1	1200003M
chr11	1.15E+08	1.15E+08	intron (NM	CpG	171	NM_02672	68572	Ict1	1110001AC
chr10	31177427	31177550	Intergenic	Intergenic	-11761	NM_00941	21987	Tpd52l1	D53
chr2	1.31E+08	1.31E+08	Intergenic	ORR1E|LTF	-67985	NM_00117	228608	Smox	B130066HC
chr17	30985852	30986050	intron (NM	intron (NM	-52861	NM_02133	14652	Glp1r	GLP-1R|GL
chr3	1.26E+08	1.26E+08	promoter-T	promoter-T	-418	NM_00129	108058	Camk2d	2810011D2
chr13	56030327	56030400	Intergenic	Intergenic	-97577	NM_01109	18740	Pitx1	Bft|P-OTX|
chr6	1.38E+08	1.38E+08	promoter-T	promoter-T	-165	NM_01149	20901	Strap	AW557906
chr12	1.19E+08	1.19E+08	intron (NM	intron (NM	429	NM_14604	217946	Cdca7l	BC006933|
chr2	93850627	93851000	promoter-T	promoter-T	74	NM_02694	69113	Alkbh3	1700108HC
chr9	70381152	70381450	Intergenic	L1ME3A|LI	-9284	NM_02633	66660	Sltm	5730455CC
chr4	55362527	55362675	promoter-T	promoter-T	-313	NM_00901	19359	Rad23b	0610007D1
chr6	52590027	52590200	intron (NM	CpG	181	NM_14556	58875	Hibadh	6430402H1
chr7	97631127	97631200	Intergenic	Intergenic	-6658	NM_02673	68472	Tmem126b	1110001A2
chr3	51464852	51465150	promoter-T	promoter-T	-114	NM_17499	211666	Mgst2	GST2|MGS
chr5	1.37E+08	1.37E+08	Intergenic	Intergenic	-51090	NM_02161	59310	Myl10	1700027I0
chr7	1.09E+08	1.09E+08	5' UTR (NM	5' UTR (NM	140	NM_00104	69710	Arap1	2410002L1
chr18	34330227	34330300	Intergenic	RLTR11B|L	-50375	NM_00746	11789	Apc	AI047805|/
chr13	5872927	5873200	Intergenic	Intergenic	12328	NM_01180	23849	Klf6	AI448727|I
chr4	1.32E+08	1.32E+08	intron (NM	intron (NM	711	NM_00751	11983	Atpif1	Atpi|If1
chr11	70920052	70920175	intron (NM	intron (NM	38093	NM_00100	195046	Nlrp1a	CARD7|DE
chr5	1.37E+08	1.37E+08	intron (NM	CpG	177	NM_01129	20022	Polr2j	14.5kDa|P
chr6	72294802	72295375	promoter-T	promoter-T	81	NM_13859	28035	Usp39	AA408960|
chr1	1.35E+08	1.35E+08	Intergenic	Intergenic	-11430	NM_13381	108954	Ppp1r15b	1810033K1
chr6	71858427	71858800	promoter-T	promoter-T	143	NM_02727	69956	Ptcd3	2610034F1
chr15	57808377	57808450	Intergenic	ORR1C2|LT	-9045	NM_17386	239463	Fam83a	-
chr12	1.12E+08	1.12E+08	intron (NM	intron (NM	1320	NM_01163	22031	Traf3	AI528849|
chr16	32541577	32541850	Intergenic	MLT1C|LTF	45346	NM_19930	245308	Zdhhc19	Gm1744|G
chr10	1.28E+08	1.28E+08	promoter-T	promoter-T	-848	NM_03125	83430	Il23a	IL-23|p19
chr10	1.27E+08	1.27E+08	intron (NM	B3|SINE|B	849	NM_00125	108037	Shmt2	2700043DC
chr8	80041402	80041625	non-coding	non-coding	293	NM_03011	78514	Arhgap10	A930033BC
chr17	31176027	31176100	Intergenic	Intergenic	-18576	NM_00959	11307	Abcg1	AW413978
chr6	1.29E+08	1.29E+08	promoter-T	promoter-T	-124	NM_17768	232413	Clec12a	CLL-1|D23C
chr13	67229402	67229475	intron (NM	intron (NM	274	NM_17239	268670	Zfp759	BC028265|
chr7	1.09E+08	1.09E+08	intron (NM	MIRc|SINE	7519	NM_01956	56212	Rhog	2810426GC
chr17	24986702	24986825	promoter-T	promoter-T	484	NM_02348	68636	Fahd1	1110025H1
chr17	17511602	17511725	intron (NM	intron (NM	367	NM_02593	67045	Riok2	2010110K2

chr10	1.17E+08	1.17E+08	Intergenic	MTE2b|LTI	-35847	NM_00101	432508	Cpsf6	4733401N1
chr6	1.08E+08	1.08E+08	intron (NM	intron (NM	113811	NM_01058	16438	Itpr1	D6Pas2|EN
chr11	1.03E+08	1.03E+08	Intergenic	Intergenic	-24073	NM_18303	353047	Plekhm1	AP162|B2|
chr13	85428552	85428650	5' UTR (NM	5' UTR (NM	490	NM_14545	218397	Rasa1	Gap|RasGA
chr5	1.22E+08	1.22E+08	promoter-T	promoter-T	-378	NM_17272	231713	Naa25	4833422K1
chr11	95639302	95639400	Intergenic	MLT1F1|LT	28970	NM_20160	268469	Zfp652	9530033F2
chr1	1.37E+08	1.37E+08	Intergenic	Intergenic	-8631	NM_17343	215690	Nav1	9530089B1
chr4	59451402	59451750	promoter-T	promoter-T	-71	NM_00116	634731	Susd1	A530080P1
chr2	68663602	68663825	Intergenic	Intergenic	-35901	NM_17285	241447	Cers6	4732462C0
chr15	81842527	81842700	intron (NM	intron (NM	3957	NM_13409	28075	Desi1	AI427858|A
chr2	1.78E+08	1.78E+08	5' UTR (NM	5' UTR (NM	2137	NM_00108	71532	Fam217b	9030418K0
chr7	1.06E+08	1.06E+08	intron (NM	intron (NM	40198	NM_00982	12406	Serpinh1	BERF-1|Cb
chr3	94247302	94247375	promoter-T	promoter-T	80	NM_03011	78523	Mrpl9	8030480E2
chr5	23949752	23949875	exon (NM_	exon (NM_	1240	NM_02322	66587	Fastk	0610011K0
chr18	76400752	76401025	promoter-T	promoter-T	-467	NM_01075	17126	Smad2	7120426M
chr12	1.01E+08	1.01E+08	intron (NM	intron (NM	8726	NM_03017	78767	Efcab11	2610021K2
chr12	40878927	40879075	Intergenic	Intergenic	-18186	NM_00114	20259	Scin	AW545522
chr1	1.87E+08	1.87E+08	intron (NM	intron (NM	2491	NM_00129	66112	1-Mar	1300013F1
chr17	5402377	5402925	Intergenic	Intergenic	16116	NM_17534	106557	Ldhal6b	4933402O1
chr1	1.73E+08	1.73E+08	Intergenic	(TA)n|Simp	-1825	NM_17706	320078	Olfml2b	1110018N0
chr17	66425802	66425875	intron (NM	CpG	548	NM_00102	106585	Ankrd12	2900001A1
chr13	95128952	95129175	promoter-T	promoter-T	148	NM_00968	11774	Ap3b1	AP-3|AU01
chr10	80958252	80958500	intron (NM	intron (NM	732	NM_13400	103425	Ncln	3100002P1
chr6	29718552	29718650	exon (NM_	exon (NM_	158	NM_00117	74340	Ahcyl2	4631427C1
chr5	1.37E+08	1.37E+08	Intergenic	L1MB5|LIN	-14964	NM_02577	66801	Prkrip1	8430424D2
chr4	1.35E+08	1.35E+08	promoter-T	promoter-T	-506	NM_02298	53902	Rcan3	AU041093
chr17	29631427	29631575	intron (NM	intron (NM	3511	NM_00884	18712	Pim1	Pim-1
chr13	30076577	30078175	exon (NM_	exon (NM_	556	NM_01009	13557	E2f3	E2F3b|E2f3
chr16	32180027	32180325	intron (NM	B2_Mm2|S	290	NM_00103	328660	Bex6	B020003O0
chr9	1.05E+08	1.05E+08	Intergenic	PB1D10|SI	-26951	NM_00125	235574	Atp2c1	1700121J1
chr7	16705152	16705275	promoter-T	promoter-T	-12	NM_13372	70394	Kptn	2310042D1
chr1	18297427	18297675	Intergenic	Intergenic	-42332	NM_00104	654464	Gm15386	BD-17|Def
chr7	1.08E+08	1.08E+08	intron (NR_	CpG	243	NM_19883	52443	Mrpl48	1810030E2
chr3	90052677	90052750	intron (NM	intron (NM	599	NM_01390	30791	Slc39a1	Zip1|Zirtl
chr13	1.01E+08	1.01E+08	intron (NM	intron (NM	28091	NM_01142	20595	Smn1	AI849087|(
chr5	20917102	20917475	intron (NM	intron (NM	13207	NM_02919	75172	Ccdc146	4930528G0
chr1	1.93E+08	1.93E+08	promoter-T	promoter-T	49	NM_00749	11910	Atf3	LRG-21
chr6	1.47E+08	1.47E+08	promoter-T	promoter-T	45	NM_02591	67015	Ccdc91	1700086G0
chr2	23011827	23012025	promoter-T	promoter-T	-99	NM_01377	27377	Yme1l1	FtsH1|Ftsh
chr1	94830677	94830825	promoter-T	promoter-T	-234	NM_01179	23830	Capn10	AW049679
chr11	1.04E+08	1.04E+08	intron (NM	CpG	159	NM_01171	22412	Wnt9b	Wnt14b|W
chr11	1.04E+08	1.04E+08	promoter-T	promoter-T	-346	NM_00108	76719	Kansl1	1700081L1
chr4	14792077	14792225	intron (NM	intron (NM	785	NM_02826	72519	Tmem55a	2610319K0
chr5	1.51E+08	1.51E+08	intron (NM	CpG	411	NM_17531	100710	Pds5b	AI646570|A
chr11	1.07E+08	1.07E+08	Intergenic	L1MB7|LIN	-27073	NM_19829	78455	Helz	3110078M
chr1	21950402	21950675	intron (NM	intron (NM	1485	NM_00116	226922	Kcnq5	7730402H1
chr9	89971027	89971200	TTS (NM_0	TTS (NM_0	22008	NM_00780	13036	Ctsh	AL022844

chr1	1.22E+08	1.22E+08	promoter-T	promoter-T	-140	NM_01993	19258	Ptpn4	PTPMEG|P
chr2	1.64E+08	1.64E+08	intron (NM	intron (NM	-6693	NM_00903	19668	Rbpjl	RBP-L|Rbp
chr10	12255202	12255425	intron (NM	MIRb|SINE	326220	NM_01168	22288	Utrn	AA589569|
chr19	3882702	3882875	intron (NR_	intron (NR_	22442	NM_01692	27060	Tcirg1	ATP6N1C|
chr4	1.37E+08	1.37E+08	Intergenic	ID_B1|SINE	-14577	NM_00124	12540	Cdc42	AI747189|
chr2	1.67E+08	1.67E+08	Intergenic	Intergenic	-22116	NM_02381	76367	Trp53rk	2810408M
chr7	1.4E+08	1.4E+08	intron (NM	intron (NM	8303	NM_00998	13017	Ctbp2	AA407280|
chr2	1.4E+08	1.4E+08	promoter-T	promoter-T	-135	NR_11035	75812	Tasp1	4930485D0
chr9	1.11E+08	1.11E+08	intron (NM	intron (NM	7650	NM_00114	235633	Als2cl	79H19D|79
chr2	32702402	32702475	intron (NM	intron (NM	314	NM_00929	20910	Stxbp1	AI317162|
chr19	45857577	45857675	5' UTR (NM	5' UTR (NM	155	NM_02379	76055	Mgea5	2810009A2
chr17	29104077	29104300	3' UTR (NM	3' UTR (NM	14027	NM_02588	66989	Kctd20	2410004N1
chr13	1.09E+08	1.09E+08	promoter-T	promoter-T	-297	NM_02900	74559	Elovl7	9130013K2
chr4	84332002	84332125	Intergenic	Intergenic	-11073	NM_17287	242509	Bnc2	5031434M
chr9	22030127	22030350	promoter-T	promoter-T	82	NM_00116	235047	Zfp809	BB114266|
chr2	5693677	5693800	Intergenic	Intergenic	-57930	NM_17734	227541	Camk1d	A630059D1
chr7	1.17E+08	1.17E+08	intron (NM	B1_Mur2|S	2196	NM_00930	20947	Swap70	70kDa|AV2
chr1	1.57E+08	1.57E+08	Intergenic	Intergenic	-58939	NM_01127	19775	Xpr1	Rmc-1|Rm
chr11	1.18E+08	1.18E+08	promoter-T	promoter-T	-591	NM_14543	217353	Tmc6	D11Ertd20
chr2	75523627	75523825	intron (NM	intron (NM	18972	NM_01090	18024	Nfe2l2	AI194320|
chr12	28348102	28348250	Intergenic	Intergenic	-320593	NM_00923	20666	Sox11	1110038H0
chr1	99667652	99667825	intron (NM	intron (NM	982	NM_02625	252876	Gin1	4930429M
chr2	1.45E+08	1.45E+08	Intergenic	Intergenic	193474	NM_02549	66328	Scp2d1	1700010M
chr11	22743377	22743625	intron (NM	intron (NM	16234	NM_01688	53625	B3gnt2	AA408337|
chr4	1.35E+08	1.35E+08	promoter-T	promoter-T	7	NM_02298	53902	Rcan3	AU041093
chr10	18782402	18782600	Intergenic	Intergenic	-47285	NM_00939	21929	Tnfaip3	A20|Tnfip3
chr1	54495727	54495925	promoter-T	promoter-T	-11	NM_00103	98488	Gtf3c3	AL022818|
chr12	25516627	25516725	intron (NM	CpG	212	NM_00108	67216	Mboat2	2810049G0
chr16	57132502	57132750	intron (NM	intron (NM	10799	NM_13859	28185	Tomm70a	2610044B2
chr16	33267752	33267900	intron (NM	MTC|LTR|I	16284	NM_08055	69150	Snx4	1810036H1
chr11	54846727	54846875	intron (NM	CpG	172	NM_00111	11749	Anxa6	AW107198
chr16	16305202	16305275	intron (NR_	intron (NR_	2180	NM_19824	70120	Yars2	2210023C1
chr10	19462627	19463050	exon (NM_	exon (NM_	30445	NM_17278	237313	Il20ra	E230031K1
chr14	50681952	50682275	Intergenic	CpG	22241	NM_14631	258313	Olfr726	MOR246-4
chr2	52777252	52777425	intron (NM	intron (NM	60436	NM_17240	71409	Fmnl2	5430425K0
chr3	1.21E+08	1.21E+08	Intergenic	Intergenic	-1979	NM_01017	14066	F3	AA409063|
chr17	27760477	27760725	promoter-T	promoter-T	-204	NM_01983	56409	Nudt3	1110011B0
chr6	1.22E+08	1.22E+08	Intergenic	Intergenic	-3977	NM_01074	17113	M6pr	CD-MPR|M
chr19	57685477	57685550	promoter-T	promoter-T	-11	NM_18141	226255	Atrnl1	AI504415|
chr13	23685327	23685400	intron (NM	intron (NM	-7524	NM_00129	319179	Hist1h2be	-
chr16	30596402	30596550	Intergenic	Intergenic	-3333	NM_17763	224093	Fam43a	Tuf1
chr3	1.28E+08	1.28E+08	intron (NM	intron (NM	1228	NM_02748	70617	5730508B0	2700063A1
chr19	8971927	8972250	promoter-T	promoter-T	-459	NM_00129	14376	Ganab	AU042638
chr15	59485427	59485550	intron (NM	intron (NM	5279	NM_14454	211770	Trib1	A530090O1
chr14	80384752	80385325	Intergenic	Intergenic	-15071	NM_00103	380924	Olfm4	GC1|GW11
chr3	62291977	62292075	intron (NM	intron (NM	18884	NM_02813	72162	Dhx36	2810407E2
chr1	1.66E+08	1.66E+08	promoter-T	promoter-T	714	NM_00116	66352	Blzf1	1700030G0

chr18	24629377	24629525	promoter-T	promoter-T	79	NM_02652	68046	2700062CC	AI195775|(
chr5	1.24E+08	1.24E+08	intron (NM	URR1B|DN	4131	NM_17787	330192	Vps37b	2300007F2
chr18	13071877	13072125	intron (NM	intron (NM	28349	NM_20753	64291	Osbpl1a	G430090F1
chr4	1.36E+08	1.36E+08	Intergenic	ID4|SINE|I	-7112	NM_01373	27224	Tceb3	110kDa|AA
chr2	1.64E+08	1.64E+08	Intergenic	Intergenic	-2947	NM_00111	381404	Pabpc1l	1810053BC
chr2	1.64E+08	1.64E+08	intron (NM	CpG	262	NM_00739	11486	Ada	-
chr6	1E+08	1E+08	Intergenic	Intergenic	-48186	NM_01974	56353	Rybp	2410018J2
chr8	72346302	72346375	promoter-T	promoter-T	-126	NM_02002	53978	Lpar2	Edg4|IPA2
chr12	31595977	31596100	promoter-T	promoter-T	439	NM_02133	11431	Acp1	4632432E0
chr7	1.33E+08	1.33E+08	intron (NM	intron (NM	-52651	NM_00110	269994	Gsg1l	AI852434|(
chr7	71078377	71078550	intron (NM	intron (NM	5338	NM_02136	50794	Klf13	0610043C1
chr5	67651927	67652025	5' UTR (NM	5' UTR (NM	172	NM_03010	67878	Tmem33	1110006G(
chr16	20357852	20358225	intron (NM	intron (NM	-32561	NM_18315	224044	Cyp2ab1	EG224044|
chr10	4540227	4540300	promoter-T	promoter-T	-813	NM_02599	67141	Fbxo5	2510044I1(
chr9	43172627	43172725	Intergenic	Intergenic	53830	NM_02365	72169	Trim29	1110047J2
chr1	1.34E+08	1.34E+08	exon (NM_	exon (NM_	-8206	NM_02902	74626	Tmem81	4930429O2
chr3	87689227	87689425	5' UTR (NM	5' UTR (NM	158	NM_03357	94315	Prcc	-
chr8	67356552	67356850	intron (NM	intron (NM	17121	NM_17863	77113	Klhl2	6030411N2
chr17	27770477	27770675	intron (NM	intron (NM	1611	NM_02596	67097	Rps10	2210402AC
chr13	75931802	75932000	Intergenic	Intergenic	-45433	NM_05310	93692	Glrx	C86710|D1
chr8	90169652	90169750	Intergenic	Intergenic	81895	NM_17790	330820	4933402J0	-
chr9	43896227	43896325	intron (NM	intron (NM	3273	NM_19809	53376	Usp2	B930035K2
chr9	99464102	99464325	intron (NM	intron (NM	5105	NM_00116	74125	Armc8	1200015K2
chr7	29062977	29063300	exon (NM_	exon (NM_	413	NM_00117	434156	Eid2b	3010005CC
chr7	78608477	78608775	Intergenic	Intergenic	842855	NM_00102	244049	Mctp2	Gm489
chr10	42432877	42432975	Intergenic	RLTR24|LT	34204	NM_17241	14628	Ostm1	1200002H1
chr2	1.02E+08	1.02E+08	promoter-T	promoter-T	-381	NM_02963	76501	Commd9	1810029F0
chr17	71966302	71966500	intron (NM	intron (NM	846	NM_17563	72515	Wdr43	2610318G(
chr8	1.08E+08	1.08E+08	TTS (NM_1	TTS (NM_1	152	NM_00116	74356	4931428F0	AI426165|I
chr9	43999227	43999625	intron (NM	Charlie1a|I	42703	NM_00761	12402	Cbl	4732447J0
chr18	46900627	46900725	promoter-T	promoter-T	557	NM_02621	67526	Atg12	4931423H1
chr17	74843102	74843200	intron (NM	MTA_Mm|	15297	NM_00103	268973	Nlrc4	9530011P1
chr11	1.21E+08	1.21E+08	promoter-T	promoter-T	-16	NM_15354	217366	Lrrc45	BC023296
chr11	48984177	48984325	intron (NM	intron (NM	10733	NM_14627	258273	Olfr1394	MOR280-1
chr5	1.06E+08	1.06E+08	intron (NM	intron (NM	39136	NM_13389	100604	Lrrc8c	AD158|AI3
chr6	1.21E+08	1.21E+08	intron (NM	intron (NM	7778	NM_02873	74043	Pex26	4632428M
chr8	1.09E+08	1.09E+08	promoter-T	promoter-T	63	NM_17879	330836	Slc7a6	AI643885|I
chr12	72236452	72236575	intron (NM	intron (NM	1149	NM_00102	30056	Timm9	2810011L1
chr11	76030802	76030950	promoter-T	promoter-T	198	NM_17736	276919	Gemin4	4932415L0
chr1	26319927	26320075	Intergenic	RLTR6_Mm	424304	NM_00103	210940	4931408C2	-
chr16	64934727	64934850	intron (NM	MLT1A1|LT	82877	NM_17864	106143	Cggbp1	AA960172|
chr5	1.24E+08	1.24E+08	Intergenic	Intergenic	33037	NM_03070	80885	Hcar2	Gpr109a|G
chr13	1.03E+08	1.03E+08	Intergenic	B4A|SINE|	-28050	NM_00853	17079	Cd180	F630107B1
chr5	46235727	46236100	intron (NM	(CA)n|Simp	11878	NM_17215	209707	Lcorl	Mlr1
chr10	84125002	84125200	intron (NM	intron (NM	39919	NM_02742	70428	Polr3b	2700078H(
chr15	96573677	96574075	Intergenic	Intergenic	-43747	NM_17512	67760	Slc38a2	5033402L1
chr1	34521252	34521375	intron (NM	L1MB8|LIN	4722	NM_01120	19253	Ptpn18	FLP1|HSCF

chr4	1.3E+08	1.3E+08	promoter-T	promoter-T	27	NM_01068	16792	Laptm5	E3
chr5	44193002	44193325	Intergenic	Intergenic	-16969	NM_00976	12182	Bst1	114/A10|A
chr4	45543002	45543400	5' UTR (NM	5' UTR (NM	499	NM_00103	230126	Shb	BC028832
chr2	1.8E+08	1.8E+08	intron (NM	intron (NM	557	NM_14585	252966	Cables2	ik3-2
chr17	26839502	26839600	Intergenic	Intergenic	-13044	NM_02987	77128	Crebrf	A930001N(
chr2	1.4E+08	1.4E+08	non-coding	non-coding	303	NM_00115	75812	Tasp1	4930485D(
chr1	53830002	53830125	intron (NM	intron (NM	11996	NM_13381	98267	Stk17b	3110009A(
chr15	41615702	41615925	intron (NM	intron (NM	-4767	NM_00113	170719	Oxr1	2210416C2
chr7	71156777	71157025	Intergenic	Intergenic	-73100	NM_02136	50794	Klf13	0610043C1
chr18	9957977	9958375	promoter-T	promoter-T	-2	NM_15355	225160	Thoc1	3110002N2
chr5	1.15E+08	1.15E+08	intron (NM	intron (NM	9677	NM_02671	68420	Ankrd13a	1100001D1
chr3	14871627	14871775	3' UTR (NM	3' UTR (NM	8163	NM_00760	12350	Car3	BB219044|
chr11	54299677	54299900	intron (NM	intron (NM	-36559	NM_00125	192786	Rapgef6	A530068K(
chr1	9690452	9690550	promoter-T	promoter-T	-211	NM_00865	17864	Mybl1	A-myb|G1-
chr15	37931027	37931100	intron (NM	intron (NM	-40253	NM_19947	382985	Rrm2b	p53R2
chr10	20818277	20818425	Intergenic	MT2B|LTR	57725	NM_00117	52906	Ahi1	1700015F0
chr1	1.62E+08	1.62E+08	intron (NM	intron (NM	622	NM_00978	12301	Cacybp	SIP
chr1	1.93E+08	1.93E+08	Intergenic	Intergenic	13070	NM_03006	381319	Batf3	9130211I0
chr7	1.33E+08	1.33E+08	Intergenic	Intergenic	-35820	NM_14558	104175	Sbk1	Sbk
chr12	76771052	76771175	promoter-T	promoter-T	-589	NM_02820	72338	Wdr89	2600001A1
chr1	1.66E+08	1.66E+08	promoter-T	promoter-T	211	NM_20136	381306	BC055324	-
chr16	70297927	70298050	Intergenic	MTA_Mm|	-16206	NM_02880	74185	Gbe1	2310045H1
chr11	22274377	22274700	Intergenic	Intergenic	-87740	NM_00125	216565	Ehbp1	AF424697|
chr1	1.74E+08	1.74E+08	intron (NM	intron (NM	9960	NM_01348	12523	Cd84	A130013D2
chr19	37282402	37282525	promoter-T	promoter-T	428	NM_00116	69104	5-Mar	1810015H1
chr3	1.52E+08	1.52E+08	intron (NM	Lx8|LINE|L	49249	NM_02566	329777	Pigk	3000001O(
chr11	87861277	87861675	exon (NM_	exon (NM_	601	NM_17337	110809	Srsf1	1110054N1
chr3	19347802	19348100	Intergenic	Intergenic	-60644	NM_00116	66326	Dnajc5b	1700008A(
chr5	1.43E+08	1.43E+08	intron (NM	intron (NM	-21922	NM_17272	231855	Ap5z1	C330006K(
chr6	1.18E+08	1.18E+08	promoter-T	intron (NM	-196	NM_00116	98758	Hnrnpf	4833420I2(
chr3	74708302	74708450	Intergenic	RMER20B|	239269	NM_17251	213234	Zbbx	4931432L2
chr1	95700552	95700775	intron (NM	CpG	121	NM_02545	66262	Ing5	1700001C1
chr8	1.26E+08	1.26E+08	intron (NM	RLTR22_M	26043	NM_02049	57247	Zfp276	AW048709
chr3	69120777	69121150	5' UTR (NM	5' UTR (NM	123	NM_17872	242083	Ppm1l	3222401G1
chr18	65591002	65591200	intron (NM	CpG	450	NM_17283	240354	Malt1	A630046N1
chr7	87163552	87163650	Intergenic	Intergenic	-6099	NM_00114	209225	Zfp710	5430400N(
chr10	20735077	20735375	intron (NM	intron (NM	-25400	NM_00117	52906	Ahi1	1700015F0
chr18	69752252	69752350	intron (NM	intron (NM	246926	NM_00108	21413	Tcf4	5730422P(
chr2	1.72E+08	1.72E+08	Intergenic	Intergenic	34185	NM_00115	21420	Tfap2c	AA409384|
chr14	60870327	60870600	promoter-T	promoter-T	-248	NM_17059	71844	Nupl1	1700017F1
chr3	1.33E+08	1.33E+08	intron (NM	Lx8|LINE|L	19377	NM_02995	77669	Arhgef38	9130221D2
chr6	1.14E+08	1.14E+08	intron (NM	MER2|DNA	-27755	NM_00103	381801	Tatdn2	AI646012|
chr4	1.07E+08	1.07E+08	Intergenic	Intergenic	-48777	NM_02559	329910	Acot11	1110020M
chr8	1.08E+08	1.08E+08	promoter-T	promoter-T	37	NM_02548	66320	Tmem208	1700006C(
chr14	61831802	61831900	intron (NM	intron (NM	45476	NM_01189	24053	Sgcg	35kDa|543
chr1	1.68E+08	1.68E+08	5' UTR (NM	5' UTR (NM	190	NM_03024	27878	Tada1	2900026B1
chr19	43598077	43598200	intron (NM	intron (NM	957	NM_01032	14718	Got1	AI789014|

chr1	1.85E+08	1.85E+08	Intergenic	Intergenic	449957	NM_01692	53791	Tlr5	-
chr2	25051127	25051325	exon (NM_	exon (NM_	1107	NM_14611	227612	Tor4a	A830007P1
chrX	9046452	9046525	promoter-T	promoter-T	-38	NM_00780	13058	Cybb	C88302|CG
chr6	35449977	35450425	Intergenic	L1MB4|LIN	39687	NM_00809	14489	Mtpn	5033418D1
chr12	73037552	73037850	intron (NM	intron (NM	47171	NM_00128	208846	Daam1	-
chr1	1.84E+08	1.84E+08	Intergenic	Intergenic	-15330	NM_01205	27058	Srp9	9kDa
chr14	45511127	45511325	Intergenic	Intergenic	-32176	NM_00896	19214	Ptgdr	DP|PGD
chr16	16896927	16897075	intron (NM	intron (NM	439	NM_17683	68606	Ppm1f	1110021B1
chr13	51747052	51747175	promoter-T	promoter-T	30	NM_02927	75420	Secisbp2	2210413N0
chr4	1.01E+08	1.01E+08	Intergenic	CpG	-27964	NM_14614	16451	Jak1	AA960307|
chr9	65049627	65049750	Intergenic	Intergenic	-12809	NM_17746	214424	Parp16	ARTD15|BC
chr10	90544902	90545025	promoter-T	promoter-T	525	NM_00104	11783	Apaf1	6230400I0
chr19	54120752	54120900	exon (NM_	exon (NM_	1154	NM_00741	11551	Adra2a	AW122659
chr4	66005827	66006025	intron (NM	intron (NM	59591	NM_01951	56079	Astn2	1d8|Astnl|
chr16	25948177	25948550	Intergenic	Intergenic	146361	NM_00112	22061	Trp63	AI462811|
chr9	1.06E+08	1.06E+08	Intergenic	Intergenic	-11078	NM_02055	11655	Alas1	ALAS|ALAS
chr6	86960227	86960325	intron (NM	intron (NM	446	NM_02004	56748	Nfu1	0610006G1
chr8	49195077	49195425	promoter-T	promoter-T	-61	NR_02775	320685	Dctd	6030466N0
chr1	1.09E+08	1.09E+08	Intergenic	Lx8|LINE|L	-7974	NM_00117	18788	Serpinb2	PAI-2|Plan
chr1	65233327	65233425	promoter-T	promoter-T	117	NM_01108	18711	Pikfyve	5230400C1
chr9	71981152	71981475	Intergenic	Intergenic	-21687	NM_00125	21406	Tcf12	A130037E0
chr3	65762177	65762275	promoter-T	promoter-T	-79	NM_01993	56706	Ccnl1	2610030E2
chr15	36722902	36723050	intron (NM	CpG	1317	NM_01174	22631	Ywhaz	1110013I1
chr1	72353777	72354025	promoter-T	promoter-T	-94	NM_00953	22596	Xrcc5	AI314015|
chr7	1.23E+08	1.23E+08	intron (NM	intron (NM	43887	NM_01144	20679	Sox6	AI987981|
chr4	1.38E+08	1.38E+08	3' UTR (NM	3' UTR (NM	-2590	NM_00119	16559	Kif17	5930435E0
chr2	24790977	24791200	promoter-T	promoter-T	-287	NM_17840	215705	Arrdc1	AI957342|
chr14	75580602	75580850	intron (NM	intron (NM	44494	NM_00887	18826	Lcp1	AW536232
chr10	26819752	26820025	intron (NM	Lx|LINE|L1	327570	NM_17683	73910	Arhgap18	4833419J0
chr2	1.19E+08	1.19E+08	intron (NM	CpG	314	NM_02657	68142	Ino80	2310079N1
chr15	59313027	59313425	intron (NM	intron (NM	107473	NM_00116	68501	Nsmce2	1110014D1
chr8	37311102	37311325	intron (NM	CpG	1357	NM_00108	244421	Lonrf1	-
chr17	50432302	50432650	intron (NM	CpG	469	NM_01002	13164	Dazl	Daz-like|Da
chr1	1.83E+08	1.83E+08	intron (NM	intron (NM	10251	NM_03013	98417	Cnih4	AI647760|
chr9	77956152	77956375	promoter-T	promoter-T	200	NM_02360	71538	Fbxo9	9030401P1
chr3	89518927	89519450	intron (NM	CpG	244	NM_00103	56417	Adar	AV242451|
chr4	1.32E+08	1.32E+08	Intergenic	Intergenic	-20084	NM_00103	230787	Themis2	ICB-1
chr9	56053427	56053550	3' UTR (NM	3' UTR (NM	-44611	NM_01979	56434	Tspan3	1700055K0
chr17	31791877	31791950	intron (NM	intron (NM	3786	NM_02418	108121	U2af1	2010107D1
chr5	1.15E+08	1.15E+08	intron (NM	B4|SINE|B4	29466	NM_00100	433940	Fam222a	-
chr4	70956652	70956800	Intergenic	RMER16-in	-760764	NM_17269	230316	Megf9	4933405H1
chr4	1.34E+08	1.34E+08	intron (NM	intron (NM	5243	NM_14555	100017	Ldlrap1	AA691260|
chr8	59989802	59990000	promoter-T	promoter-T	-739	NM_00825	97165	Hmgb2	C80539|HM
chr11	1.21E+08	1.21E+08	intron (NM	CpG	545	NM_00103	80879	Slc16a3	Mct3|Mct4
chr1	1.84E+08	1.84E+08	Intergenic	Intergenic	-39256	NM_13381	98386	Lbr	AI505894|i
chr8	1.23E+08	1.23E+08	Intergenic	Intergenic	-4826	NM_00116	234797	6430548M	AW049007
chr2	76198802	76198925	promoter-T	promoter-T	-353	NM_00998	13067	Cyct	T-Cc

chr7	1.18E+08	1.18E+08	intron (NM	CpG	518	NM_00127	11717	Ampd3	-
chr9	66060727	66060800	intron (NM	intron (NM	54730	NM_01001	13143	Dapk2	-
chr17	35138852	35139050	intron (NM	intron (NM	1099	NM_01169	22321	Vars	Bat6|D17H
chr14	21872077	21872300	promoter-T	promoter-T	392	NM_13407	11534	Adk	2310026J0
chr1	36569352	36569450	exon (NM_	exon (NM_	680	NM_05318	94218	Cnnm3	Acdp3|Clp
chr3	1.3E+08	1.3E+08	intron (NM	CpG	236	NM_02550	66357	Ostc	2310008M
chrX	33747577	33747675	Intergenic	Intergenic	11727	NM_02832	72693	Zcchc12	2810028AC
chr5	1.23E+08	1.23E+08	exon (NM_	exon (NM_	305	NM_17542	109305	Orai1	D730049H(
chr3	1.44E+08	1.44E+08	intron (NM	intron (NM	1379	NM_01182	23908	Hs2st1	AW214369
chr14	22632577	22632750	Intergenic	Intergenic	-18120	NM_01169	22334	Vdac2	Vdac6|mVI
chr4	1.34E+08	1.34E+08	Intergenic	Intergenic	-1775	NM_00128	71904	Paqr7	2310021M
chr19	40969502	40969875	TTS (NR_10	TTS (NR_10	493	NM_02831	72672	Zfp518a	2810401C2
chrX	35995152	35995275	Intergenic	MamGyp-ii	-6893	NM_02155	59048	C1galt1c1	1500002I1
chr1	4839527	4839750	Intergenic	Intergenic	-8137	NM_00115	21399	Tcea1	S-II
chr5	1.16E+08	1.16E+08	intron (NM	intron (NM	5841	NM_01669	50849	Rnf10	AA675014|
chr1	1.39E+08	1.39E+08	intron (NM	(CGGGG)n|	185	NM_00116	226442	Zfp281	Znf281
chr11	11461477	11461650	Intergenic	Intergenic	72294	NM_02866	73862	4930415F1	-
chr7	4744102	4744325	promoter-T	promoter-T	-354	NM_00908	19943	Rpl28	D7Wsu21e
chr15	92206602	92207100	Intergenic	Intergenic	-20390	NM_00116	239618	Pdzrn4	1110017D0
chr12	8352327	8352475	Intergenic	Intergenic	32162	NM_02142	58240	Hs1bp3	-
chr13	49262877	49263000	Intergenic	L1MB7|LIN	-19241	NM_00129	75607	Wnk2	1810073P0
chr5	33196552	33196800	intron (NM	intron (NM	203	NM_19434	215476	Prr14l	6030436E0
chr1	67410352	67410850	Intergenic	MIRb|SINE	241000	NM_00108	227231	Cps1	4732433M
chr6	91422877	91423350	promoter-T	promoter-T	304	NM_13392	72170	Chchd4	2410012P2
chr7	1.34E+08	1.34E+08	exon (NM_	exon (NM_	494	NM_14452	68449	Tbc1d10b	1110003P2
chr13	47025577	47025925	promoter-T	promoter-T	-664	NM_01061	16553	Kif13a	4930505I0
chr17	56396102	56396175	promoter-T	promoter-T	-78	NM_01019	14154	Fem1a	AW611390
chr15	89308752	89308875	promoter-T	promoter-T	-958	NM_00971	11883	Arsa	AS-A|ASA|
chr7	38974677	38974825	intron (NM	intron (NM	5945	NM_02816	72244	1600014C1	AI428873
chr7	73849552	73849750	intron (NR_	intron (NR_	14876	NR_03403(	72635	Lins	2700083B0
chr3	51364602	51364775	promoter-T	promoter-T	57	NM_08079	73251	Setd7	1600028F2
chr7	29592827	29592950	Intergenic	Intergenic	-3021	NM_17730	15388	Hnrnpl	C79783|D8
chr10	88193652	88193975	promoter-T	promoter-T	33	NM_00103	237433	Gm4925	EG237433
chr7	31935802	31935950	intron (NM	CpG	193	NM_02789	52857	Gramd1a	1300003M
chr11	1.01E+08	1.01E+08	exon (NM_	exon (NM_	1376	NM_01047	15485	Hsd17b1	17HSDB1|1
chr4	13364877	13365050	Intergenic	Intergenic	-305486	NM_00111	12395	Runx1t1	Cbfa2t1h|E
chr7	1.13E+08	1.13E+08	Intergenic	Lx6|LINE|L	-33684	NM_02900	74558	Gvin1	9130002C2
chr17	26649452	26649700	Intergenic	Intergenic	-4159	NM_01364	19252	Dusp1	3CH134|M
chr6	6527577	6527700	intron (NM	intron (NM	1020	NM_00916	20422	Shfm1	DSS1|Shfd
chr15	83303727	83303875	Intergenic	MTC|LTR|I	-8765	NM_00115	23970	Pacsin2	AI197433
chr7	1.45E+08	1.45E+08	Intergenic	Intergenic	225275	NM_18328	70571	Tcerg1l	5730476P1
chr2	1.29E+08	1.29E+08	Intergenic	Intergenic	-11626	NM_02824	72477	Tmem87b	2610301K1
chr15	84128027	84128100	intron (NM	URR1A|DN	-27087	NM_02232	64099	Parvg	AI413459
chr11	59778077	59778150	exon (NM_	exon (NM_	331	NM_00902	19416	Rasd1	Dexras1
chr16	78577477	78577775	promoter-T	promoter-T	-693	NM_02596	67102	D16Ertd47	1700010I1
chr6	1.43E+08	1.43E+08	Intergenic	B4A|SINE|	-4330	NM_00990	12764	Cmas	AW208911
chr1	1.15E+08	1.15E+08	Intergenic	Intergenic	-1133093	NM_00108	319901	Dsel	9330132E0

chr1	1.31E+08	1.31E+08	Intergenic	RMER19B|	-155275	NM_00991	12767	Cxcr4	CD184|Cm
chr5	4008352	4008550	exon (NM_	exon (NM_	80265	NM_19446	100986	Akap9	5730481H2
chr1	37258227	37258650	Intergenic	L1MC4|LIN	-16685	NM_00128	12790	Cnga3	CNG3
chr17	24592027	24592150	exon (NM_	exon (NM_	168	NM_00789	13560	E4f1	-
chr13	65223277	65223675	Intergenic	Intergenic	-9186	NM_13368	66631	Hiatl1	5730414C1
chr14	45911477	45911575	intron (NM	intron (NM	26721	NM_01577	50527	Ero1l	ERO1-L
chr1	1.57E+08	1.57E+08	Intergenic	Intergenic	64643	NM_02143	58244	Stx6	2310039E0
chr15	78729402	78729475	promoter-T	promoter-T	-778	NM_00916	20401	Sh3bp1	3BP-1
chr7	35098152	35098600	Intergenic	CpG	-27748	NM_17274	233103	4931406P1	AA553327|
chr6	92503177	92503450	intron (NM	intron (NM	14047	NM_00113	243548	Prickle2	6230400G1
chr19	46943977	46944100	intron (NM	intron (NM	92803	NM_02981	76952	Nt5c2	2010002I2
chr13	21258027	21258100	Intergenic	MT2A|LTR	-13737	NM_00905	19720	Trim27	AW538890
chr3	40482577	40482675	intron (NM	intron (NM	-30167	NM_17838	73333	Slc25a31	1700034J0
chr11	95153377	95153550	intron (NM	intron (NM	18097	NM_00119	217127	Kat7	Hbo1|Hbo
chr9	1.04E+08	1.04E+08	promoter-T	promoter-T	-220	NM_17532	102632	Acad11	5730439E1
chr6	1.19E+08	1.19E+08	intron (NM	Lx5|LINE|L	17963	NM_19798	68465	Adipor2	1110001I1
chr9	41930577	41930850	intron (NM	MIRc|SINE	1659	NM_01143	20660	Sorl1	2900010L1
chr4	9608377	9608650	Intergenic	ORR1E|LTF	-12022	NM_00129	65973	Asph	2310005F1
chr5	1.45E+08	1.45E+08	intron (NM	CpG	569	NM_01877	55950	Bri3	I3
chr17	46150602	46150675	Intergenic	Intergenic	11474	NM_00111	22339	Vegfa	Vegf|Vpf
chr11	1.21E+08	1.21E+08	intron (NM	intron (NM	1068	NM_17876	319530	Zfp750	A030007D2
chr2	61549702	61550075	5' UTR (NM	5' UTR (NM	137	NM_02152	59029	Psmd14	2610312C0
chr6	39370102	39370450	promoter-T	promoter-T	92	NM_01881	54484	Mkrn1	RFP
chr3	83599677	83599750	Intergenic	ORR1D1|LT	29470	NM_00914	20319	Sfrp2	AI851596|
chr10	57814652	57814725	intron (NM	intron (NM	-19514	NM_20124	110829	Lims1	2310016J2
chr4	45806777	45806875	Intergenic	Intergenic	-5068	NM_02827	72535	Aldh1b1	2700007F1
chr6	1.17E+08	1.17E+08	Intergenic	Intergenic	-39737	NM_17876	319776	Tmem72	C230095G0
chr7	30979827	30980075	intron (NM	intron (NM	116	NM_00979	12336	Capns1	Capa-4|Ca
chr19	3282902	3283050	promoter-T	promoter-T	34	NM_00921	20589	Ighmbp2	AEP|Catf1|
chr14	55724702	55724850	TTS (NM_1	TTS (NM_1	654	NM_00745	11766	Ap1g2	Adtg2|G2a
chr14	33597327	33597525	Intergenic	Intergenic	-1723	NM_00116	69069	1810011H1	4733401I0
chr11	72025602	72025800	intron (NM	intron (NM	-2939	NM_00116	75304	4930563E2	-
chr17	34776352	34776500	Intergenic	MTC|LTR|I	-5402	NM_01987	56299	Fkbpl	DIR1|NG7|
chr8	1.13E+08	1.13E+08	promoter-T	promoter-T	340	NM_13395	101943	Sf3b3	1810061H2
chr12	60231227	60231450	intron (NM	intron (NM	605	NM_00116	217615	Ctage5	D12Bwg05
chr19	5041352	5041425	promoter-T	promoter-T	-16	NM_13415	107392	Brms1	AV003220|
chr3	65677052	65677200	Intergenic	L2|LINE|L2	85021	NM_01993	56706	Ccnl1	2610030E2
chr5	1.05E+08	1.05E+08	intron (NM	intron (NM	24248	NM_03023	27405	Abcg3	Abcp2|Mx
chr16	8667427	8667575	intron (NM	L2b|LINE|L	4745	NM_02582	52502	Carhsp1	1200011K0
chr11	60726602	60726775	intron (NM	intron (NM	606	NM_02529	24083	Natd1	AI256713|
chr14	69891677	69892075	intron (NM	intron (NM	11284	NM_02633	67712	Slc25a37	1700020E2
chr8	1.31E+08	1.31E+08	Intergenic	B4A|SINE|	121484	NM_01057	16412	Itgb1	4633401G2
chr1	88388877	88388950	Intergenic	Intergenic	-34398	NM_00897	19231	Ptma	Thym
chr3	54379877	54379950	Intergenic	Intergenic	-117114	NM_01999	56790	Supt20	AA667204|
chr7	1.08E+08	1.08E+08	Intergenic	Intergenic	-8961	NM_00111	73683	Atg16l2	2410118P2
chr4	1.38E+08	1.38E+08	exon (NM_	exon (NM_	174	NM_02845	73162	Otud3	3110030K1
chr5	24347477	24347550	intron (NM	intron (NM	666	NM_05307	19744	Rheb	-

chr1	87749927	87750050	Intergenic	Intergenic	-41097	NM_02241	64294	Itm2c	3110038L0
chr11	95699952	95700025	intron (NM	intron (NM	-3621	NM_02312	14710	Gngt2	AV096488
chr13	51513077	51513150	intron (NM	intron (NM	9126	NM_01010	13610	S1pr3	AI132464|
chr14	73071852	73072050	intron (NM	PB1D10|SI	37859	NM_20763	319448	Fndc3a	1700094E1
chr1	1.21E+08	1.21E+08	promoter-T	promoter-T	-329	NM_02232	64143	Ralb	5730472O1
chr5	76954502	76954575	Intergenic	RLTR28|LT	-3798	NM_00128	69940	Exoc1	2810407P2
chr8	3308277	3308400	Intergenic	RLTR44-int	-28721	NM_01056	16337	Insr	4932439J0
chr17	48371827	48371900	promoter-T	promoter-T	-40	NM_02140	58217	Trem1	-
chr4	98587702	98587925	Intergenic	PB1D10|SI	-2688	NM_14614	230484	Usp1	-
chr6	1.03E+08	1.03E+08	Intergenic	Intergenic	-245982	NM_00769	12661	Chl1	A530023M
chrX	1.05E+08	1.05E+08	Intergenic	Intergenic	-324463	NM_00103	213450	Gm732	-
chr1	74439102	74439175	intron (NM	intron (NM	955	NM_15308	227292	Ctdsp1	GIP|NLIIF|
chr1	1.37E+08	1.37E+08	intron (NM	intron (NM	1564	NM_00795	13924	Ptprv	Esp|OST|C
chr4	1.16E+08	1.16E+08	exon (NM_	exon (NM_	114	NM_00108	50927	Nasp	5033430J0
chr5	77854752	77854950	Intergenic	Intergenic	-17781	NM_00804	29817	Igfbp7	AGM|Fstl2
chr7	1.19E+08	1.19E+08	promoter-T	promoter-T	-44	NM_13375	74996	Usp47	4930502N0
chr19	34141752	34141950	promoter-T	promoter-T	3	NM_02734	70166	Lipn	2210418G0
chr11	93829502	93829625	5' UTR (NM	5' UTR (NM	272	NM_00870	18102	Nme1	AL024257|
chr12	4082052	4082825	promoter-T	promoter-T	-136	NM_15308	217378	Dnajc27	AI639580|
chrX	1.4E+08	1.4E+08	Intergenic	Intergenic	-104451	NM_00111	13193	Dcx	Dbct
chr19	5974627	5974700	intron (NM	MLT1E2|LT	-10457	NM_00889	18969	Pola2	AI573378
chr13	57397127	57397275	Intergenic	Intergenic	-400252	NM_01203	26946	Trpc7	TRP-7|TRP
chr10	80959327	80959475	promoter-T	promoter-T	-293	NM_13400	103425	Ncln	3100002P1
chr10	1.11E+08	1.11E+08	intron (NM	intron (NM	918	NM_17548	237542	Osbpl8	AA536976|
chr4	1.19E+08	1.19E+08	Intergenic	Intergenic	37791	NM_13389	100470	Lao1	AW990848
chr16	22009727	22009850	exon (NM_	exon (NM_	231	NR_02748	75826	Senp2	2310007L0
chr16	23144452	23144750	Intergenic	Intergenic	-2008	NM_00960	11450	Adipoq	30kDa|APN
chr12	86738227	86738600	Intergenic	Intergenic	-22746	NM_02677	68581	Tmed10	1110014C0
chr12	56592627	56592725	exon (NM_	exon (NM_	958	NM_01090	18035	Nfkbia	AI462015|
chr14	31292577	31292850	promoter-T	promoter-T	-38	NM_13376	75901	Dcp1a	1110066A2
chr18	30457827	30457950	intron (NM	intron (NM	25338	NM_18141	225326	Pik3c3	5330434F2
chr12	38719202	38719425	intron (NM	LTR40b|LT	112021	NM_17868	217480	Dgkb	6430574F2
chr3	95976327	95976575	promoter-T	promoter-T	-22	NM_15305	107701	Sf3b4	49kDa|SF3
chr1	6205352	6205525	intron (NM	intron (NM	695	NM_00982	12421	Rb1cc1	2900055E0
chr5	1.49E+08	1.49E+08	intron (NM	intron (NM	24851	NM_01022	14254	Flt1	AI323757|
chr13	1.01E+08	1.01E+08	intron (NM	intron (NM	6602	NM_00116	76041	Ccdc125	5830436D0
chr1	1.93E+08	1.93E+08	Intergenic	B3|SINE|B	22984	NM_02542	66208	Nenf	1110060M
chr2	1.65E+08	1.65E+08	TTS (NM_2	TTS (NM_2	399	NM_00125	228875	Slc35c2	C85957|CG
chr5	5514552	5514625	intron (NM	CpG	201	NM_00119	64945	Cldn12	-
chr2	24530002	24530075	intron (NM	intron (NM	88634	NM_00104	12287	Cacna1b	AW050276
chr11	1.2E+08	1.2E+08	intron (NM	CpG	408	NM_13379	192662	Arhgdia	5330430M
chr1	1.2E+08	1.2E+08	Intergenic	Intergenic	13406	NM_02647	67949	Nifk	AI852665|
chr8	1.14E+08	1.14E+08	promoter-T	promoter-T	-129	NM_14621	234734	Aars	AI316495|
chr9	40605352	40605525	Intergenic	(TTC)n|Sim	-3918	NM_03116	15481	Hspa8	2410008N1
chr15	99556102	99556175	promoter-T	promoter-T	89	NM_18325	66379	Cox14	2310016M
chr6	36087202	36087575	Intergenic	Intergenic	-250696	NM_20349	243764	Chrm2	AChR-M2|
chr16	84835377	84835600	5' UTR (NM	5' UTR (NM	119	NM_00806	14390	Gabpa	GABPalpha

chr1	94696327	94696400	Intergenic	Intergenic	-31900	NM_01669	14733	Gpc1	AI462976
chr19	56622677	56622950	promoter-T	promoter-T	62	NM_02581	66866	Nhlrc2	1200003G(
chr1	37597477	37597575	intron (NM	CpG	335	NM_17387	269181	Mgat4a	9530018I0
chr11	1.16E+08	1.16E+08	exon (NM_	exon (NM_	147	NM_02636	67763	Prpsap1	5730409F2
chr13	1.05E+08	1.05E+08	Intergenic	Intergenic	206270	NM_02607	67285	Cwc27	3110009E1
chr4	1.26E+08	1.26E+08	intron (NM	intron (NM	6842	NM_17270	230751	Oscp1	1810007P1
chr11	16856627	16856725	Intergenic	Intergenic	5263	NM_17698	319701	Fbxo48	A630050E1
chr13	43619877	43620050	Intergenic	Intergenic	35597	NM_00108	76137	Mcur1	6230416A(
chr8	1.17E+08	1.17E+08	intron (NM	intron (NM	176411	NM_01957	80707	Wwox	5330426P(
chr19	47929902	47930200	intron (NM	intron (NM	572	NM_01036	14873	Gsto1	AA407097|
chr5	65748077	65748625	intron (NM	Charlie21a	8701	NM_03118	83379	Klb	AV071179|
chr17	24349327	24349425	Intergenic	Intergenic	-3044	NM_01094	18209	Ntn3	Ntn2l
chr13	98797552	98797800	intron (NM	intron (NM	178444	NM_01202	110596	Arhgef28	9230110L0
chr9	21229577	21229675	exon (NM_	exon (NM_	274	NM_00103	13430	Dnm2	Dyn2|Udn
chr14	1.06E+08	1.06E+08	promoter-T	promoter-T	56	NM_13407	74213	Rbm26	1700009P(
chr15	58810302	58810725	intron (NM	intron (NM	45148	NM_02317	66218	Ndufb9	1190008J1
chr7	29227927	29228100	intron (NM	MIRb|SINE	2057	NM_00103	63986	Gmfg	0610039G1
chr14	99871427	99871675	Intergenic	Intergenic	173641	NM_00976	12224	Klf5	4930520J0
chr10	61245927	61246075	intron (NM	CpG	611	NM_20700	404634	H2afy2	macroH2A
chr1	40842802	40843000	intron (NM	intron (NM	4601	NM_17249	211798	Mfsd9	4931419K(
chr9	1.08E+08	1.08E+08	exon (NM_	exon (NM_	-10965	NM_14622	235606	Apeh	-
chr1	1.73E+08	1.73E+08	Intergenic	L2|LINE|L2	-20086	NM_01018	14130	Fcgr2b	AI528646|
chr18	67412002	67412125	Intergenic	Lx8|LINE|L	-6579	NM_17263	225651	Mppe1	A530095G1
chr5	1.41E+08	1.41E+08	intron (NM	intron (NM	1993	NM_00849	16848	Lfng	AW061165
chr14	31431102	31431225	intron (NM	intron (NM	8231	NM_01110	18753	Prkcd	AI385711|
chr10	42622552	42622675	intron (NM	intron (NM	42295	NM_17293	268297	Scml4	9330161D1
chr2	1.58E+08	1.58E+08	intron (NM	B3A|SINE|	4547	NM_00848	16803	Lbp	Bpifd2|Ly8
chr4	1.08E+08	1.08E+08	Intergenic	ID4|SINE|I	-1356	NM_00103	414872	Zyg11b	1110046I0
chr3	1.42E+08	1.42E+08	promoter-T	promoter-T	-37	NM_00108	229900	Gbp7	9830147J2
chr12	83488052	83488325	intron (NM	intron (NM	75852	NM_17257	217692	Sipa1l1	4931426N1
chrY	2885902	2885975	Intergenic	Intergenic	498680	NM_00127	1E+08	Gm3376	Rbmy1b
chr1	1.81E+08	1.81E+08	intron (NM	intron (NM	346	NM_02718	69726	Smyd3	2410008A1
chr1	43502877	43503125	intron (NM	CpG	405	NM_01087	17974	Nck2	4833426I1
chr2	35194227	35194575	Intergenic	Intergenic	-1872	NM_01351	13830	Stom	Epb7.2
chr8	1.2E+08	1.2E+08	exon (NM_	exon (NM_	31386	NM_02968	76645	Pkd1l2	1700126L0
chr14	65999302	65999450	intron (NM	intron (NM	21863	NM_19902	380912	Zfp395	BC053701|
chr9	20807502	20807575	exon (NM_	exon (NM_	357	NM_02316	66163	Mrpl4	1110017G1
chr19	3686027	3686200	intron (NM	CpG	451	NM_00851	16973	Lrp5	BMND1|HI
chr16	44139102	44140175	promoter-T	promoter-T	-284	NM_02810	72117	Naa50	2600005K2
chr1	1.3E+08	1.3E+08	Intergenic	Intergenic	-51758	NM_14550	226414	Dars	5730439G1
chr4	1.5E+08	1.5E+08	intron (NM	CpG	342	NM_02056	57320	Park7	DJ-1|Dj1
chr17	29639027	29639250	Intergenic	Intergenic	11148	NM_00884	18712	Pim1	Pim-1
chr13	55816052	55816250	exon (NM_	exon (NM_	140	NM_17515	69672	Txndc15	2310047H2
chr2	1.12E+08	1.12E+08	3' UTR (NM	3' UTR (NM	830	NM_02540	66181	Nop10	1110036B1
chr6	13557702	13558050	5' UTR (NM	5' UTR (NM	187	NM_02899	101118	Tmem168	5730526F1
chr9	15084827	15085025	promoter-T	promoter-T	-615	NM_14493	234959	Med17	77kDa|C33
chr15	61815077	61815250	Intergenic	Intergenic	-1733	NM_00117	17869	Myc	AU016757

chr17	46439302	46439375	TTS (NM_1	TTS (NM_1	4461	NM_00128	106565	Dlk2	AI413481|[illegible]
chr6	99342527	99342750	intron (NM	intron (NM	42701	NM_05320	108655	Foxp1	3110052D1
chr7	1.35E+08	1.35E+08	promoter-T	promoter-T	-328	NM_02699	69234	Zfp688	2810407K0
chr1	7727352	7727725	Intergenic	RLTR26|LT	648537	NM_18302	319263	Pcmtd1	8430411F1
chr6	8720952	8721150	intron (NM	intron (NM	7433	NM_01049	15893	Ica1	69kDa|ICA
chr1	1.46E+08	1.46E+08	Intergenic	Intergenic	86678	NM_00906	19735	Rgs2	GOS8
chr5	50332852	50332925	Intergenic	Lx8|LINE|L	117347	NM_13391	70693	Gpr125	3830613O2
chr8	28090352	28090425	exon (NM_	exon (NM_	2580	NM_00111	353310	Zfp703	1110032O1
chr12	77641177	77641425	intron (NM	intron (NM	6754	NM_15380	263406	Plekhg3	BC030417
chr11	1.2E+08	1.2E+08	Intergenic	Intergenic	18837	NM_01377	27376	Slc25a10	Dic
chr6	6528102	6528625	intron (NM	CpG	295	NM_00916	20422	Shfm1	DSS1|Shfd
chr7	1.21E+08	1.21E+08	intron (NM	intron (NM	97182	NM_02584	66922	Rras2	2610016H2
chr9	1.09E+08	1.09E+08	exon (NM_	exon (NM_	152	NM_03072	80987	Nckipsd	AF3P21|DI
chr6	88697852	88697950	intron (NM	intron (NM	23203	NM_00116	23945	Mgll	AA589436|
chr15	91403052	91403250	exon (NM_	exon (NM_	541	NM_00103	239606	Slc2a13	6530403A0
chr12	86940927	86941025	promoter-T	promoter-T	8	NM_00120	81703	Jdp2	Jundm2|Ju
chr15	79513852	79513950	intron (NM	intron (NM	4401	NM_02879	74158	Josd1	1300006C0
chr1	1.64E+08	1.64E+08	intron (NM	intron (NM	991	NM_17264	226551	Suco	4732491B1
chr19	44323477	44323550	Intergenic	Intergenic	-44653	NM_00912	20250	Scd2	Scd-2|swty
chr10	23506877	23506950	intron (NM	CpG-934	102	NM_01129	20042	Rps12	-
chr13	24883277	24883350	Intergenic	Intergenic	-10213	NM_02447	79555	BC005537	8030460C0
chr1	1.72E+08	1.72E+08	5' UTR (NM	5' UTR (NM	111	NM_01063	16589	Uhmk1	4732477C1
chr3	1.01E+08	1.01E+08	exon (NM_	exon (NM_	148	NM_00101	74044	Ttf2	4632434F2
chrX	71499077	71499275	promoter-T	promoter-T	-850	NM_00792	13726	Emd	AW550900
chr4	1.49E+08	1.49E+08	5' UTR (NM	5' UTR (NM	227	NM_02202	63958	Ube4b	4930551I1
chr7	38379527	38379650	intron (NM	intron (NM	175183	NM_17238	243937	Zfp536	9630010P1
chr5	89149402	89149525	promoter-T	promoter-T	-433	NM_02673	68473	Mob1b	1110003E0
chr7	26540202	26540350	promoter-T	promoter-T	-537	NM_14492	232989	Hnrnpul1	E130317O1
chr9	57408727	57408925	promoter-T	promoter-T	75	NM_02281	24044	Scamp2	AI875466|
chr5	1.08E+08	1.08E+08	intron (NM	intron (NM	370	NM_02606	67266	Fam69a	2900024C2
chr17	66300252	66300475	intron (NM	CpG	164	NM_02305	65960	Twsg1	1810013J1
chr1	93352927	93353125	intron (NM	intron (NM	2879	NM_01106	18627	Per2	mKIAA034
chr6	83066102	83066275	exon (NM_	exon (NM_	688	NM_02061	57377	Mogs	1810017N0
chr4	41315202	41315650	intron (NM	intron (NM	19397	NM_02330	67123	Ubap1	2700092A0
chr12	78051327	78051600	intron (NM	intron (NM	11772	NM_00855	17187	Max	AA960152|
chr18	68017177	68017400	intron (NM	intron (NM	57527	NM_02755	70799	Cep192	4631422C1
chr1	1.79E+08	1.79E+08	promoter-T	promoter-T	-990	NM_02975	76816	Sdccag8	2700048G2
chr8	1.07E+08	1.07E+08	intron (NR_	intron (NR_	15470	NR_04564	57813	Tk2	AU024611
chr8	34710752	34710900	intron (NM	intron (NM	733	NM_01737	19053	Ppp2cb	AI115466|
chr13	1.05E+08	1.05E+08	promoter-T	promoter-T	-435	NM_00100	59079	Erbb2ip	1700028E0
chr4	94742377	94742700	Intergenic	Intergenic	-23625	NM_01059	16476	Jun	AP-1|Junc|
chr12	17688902	17689225	Intergenic	Intergenic	-8557	NM_01667	53602	Hpcal1	NVP-3|Nvp
chr7	1.5E+08	1.5E+08	intron (NM	ORR1E|LTR	22726	NM_17726	320802	Ifitm10	6330512M
chr13	73468702	73468900	intron (NM	CpG	344	NM_05316	94066	Mrpl36	AI646041
chr12	79850277	79850350	intron (NM	CpG	379	NM_01957	56217	Mpp5	3830420B0
chr17	35271677	35271800	promoter-T	promoter-T	-385	NM_00125	224727	Bag6	2410045D2
chr2	1.28E+08	1.28E+08	intron (NM	intron (NM	-63036	NM_20768	12125	Bcl2l11	1500006F2

chr3	1.42E+08	1.42E+08	intron (NM	CpG	201	NM_00129	229905	Ccbl2	KATIII|Kat3
chr6	1.34E+08	1.34E+08	Intergenic	Intergenic	-16724	NM_00796	14011	Etv6	AW123102
chr14	73183102	73183250	Intergenic	Intergenic	-73366	NM_20763	319448	Fndc3a	1700094E1
chr1	1.36E+08	1.36E+08	5' UTR (NM	5' UTR (NM	149	NM_00116	667118	Zbed6	Gm8466|M
chr4	58980927	58981075	intron (NM	intron (NM	18949	NM_02596	67103	Ptgr1	2510002C2
chr9	59389052	59389175	intron (NM	intron (NM	1639	NM_01042	15211	Hexa	Hex-1
chr11	98412127	98412300	promoter-T	promoter-T	-198	NM_19941	69376	Zpbp2	1700017D1
chr8	26711827	26711925	promoter-T	promoter-T	-902	NM_00100	234135	Whsc1l1	6720429E0
chr7	82932177	82932425	Intergenic	Intergenic	-60923	NM_00112	207952	Klhl25	2810402K1
chr3	27997977	27998125	intron (NM	B2_Mm1t|	114958	NM_00887	18805	Pld1	AA536939|
chr16	13109502	13109625	promoter-T	promoter-T	-266	NM_01576	50505	Ercc4	AI606920|
chr4	96204027	96204100	intron (NM	intron (NM	16279	NM_01000	13110	Cyp2j6	-
chr4	1.55E+08	1.55E+08	promoter-T	promoter-T	494	NM_17920	108888	Atad3a	2400004H0
chr13	41066677	41066850	Intergenic	Intergenic	-29616	NM_02655	68083	Pak1ip1	5830431I1
chr8	86177202	86177350	intron (NM	intron (NM	699	NM_01877	67903	Gipc1	GIPC|Glut1
chr13	42469577	42469675	Intergenic	Intergenic	72987	NM_01010	13614	Edn1	ET-1|prepr
chr5	12877627	12877800	Intergenic	RLTR45|LT	494547	NM_02888	108151	Sema3d	4631426B1
chr7	1.28E+08	1.28E+08	promoter-T	promoter-T	-520	NM_02529	26939	Polr3e	RPC5|Sin
chr16	23104252	23104325	Intergenic	MTB_Mm|	-3253	NM_00112	13682	Eif4a2	4833432N0
chr4	1.33E+08	1.33E+08	Intergenic	Intergenic	26380	NM_01695	15331	Hmgn2	HMG-17|H
chr19	7921102	7921200	Intergenic	ETnERV3-ir	-43994	NM_14623	236149	Slc22a26	-
chr14	21277627	21277750	intron (NM	intron (NM	-6241	NM_00116	76670	Ttc18	-
chr5	1.23E+08	1.23E+08	intron (NM	intron (NM	3426	NM_00128	330188	Ccdc63	4921511C1
chr18	75178452	75178750	5' UTR (NM	5' UTR (NM	175	NM_02772	69190	Dym	1810041M
chr14	65481577	65481675	intron (NM	URR1A|DN	87058	NM_17733	219150	Hmbox1	AI451877|
chr7	17559402	17559700	Intergenic	Intergenic	14407	NM_00100	71691	Pnmal1	0710005I1
chr13	96482752	96483200	intron (NM	intron (NM	16101	NM_01017	14064	F2rl2	F730031A0
chr2	1.56E+08	1.56E+08	promoter-T	promoter-T	73	NM_00129	170791	Rbm39	1500012C1
chr19	5487777	5487850	exon (NM_	exon (NM_	523	NM_02787	71711	Mus81	1200008A1
chr18	65072977	65073050	intron (NM	intron (NM	25603	NM_00111	83814	Nedd4l	1300012C0
chr13	37196852	37197150	Intergenic	RLTR4_MM	-54888	NM_00116	74145	F13a1	1200014I0
chr10	81106602	81106725	intron (NM	CpG	-5151	NM_00114	1E+08	Gm10778	ENSMUSG0
chr7	89581777	89581900	intron (NM	L1MD1|LIN	97634	NM_00119	269959	Adamtsl3	9230119C1
chr8	1.17E+08	1.17E+08	intron (NM	intron (NM	408174	NM_01957	80707	Wwox	5330426P0
chr10	80462577	80462650	intron (NM	intron (NM	1497	NM_00103	14708	Gng7	AI840417
chr10	60937227	60937475	Intergenic	Intergenic	-1230	NM_15354	216011	Lrrc20	BC036304
chr1	16509427	16509600	promoter-T	promoter-T	-130	NM_00111	29819	Stau2	-
chr13	9102027	9102225	intron (NM	intron (NM	8975	NM_17258	217980	Larp4b	A630096F1
chr1	1.56E+08	1.56E+08	intron (NM	MTE2a|LTF	36970	NM_01188	24014	Rnasel	E230029I0
chr9	69880102	69880325	3' UTR (NM	3' UTR (NM	5682	NM_02808	72077	Gcnt3	2010013H2
chr8	73810202	73810450	intron (NM	intron (NM	13713	NM_00114	17925	Myo9b	-
chr2	1.58E+08	1.58E+08	exon (NM_	exon (NM_	3657	NM_00950	22348	Slc32a1	R75019|VG
chr15	96566252	96566325	Intergenic	Intergenic	-36159	NM_17512	67760	Slc38a2	5033402L1
chr2	1.32E+08	1.32E+08	Intergenic	Intergenic	32165	NM_00127	26434	Prnd	AI450264|
chr13	94944327	94944550	exon (NM_	exon (NM_	110798	NM_02915	107767	Scamp1	4930505M
chr1	99600802	99600975	Intergenic	Lx9|LINE|L	-42293	NM_13382	52392	D1Ertd622	AI987691|
chr3	51162002	51162175	Intergenic	Intergenic	-17522	NM_02350	69257	Elf2	2610036A2

chr5	31522652	31522850	exon (NM_	exon (NM_	167	NM_00801	14208	Ppm1g	AI385652|I
chr12	35682652	35682825	Intergenic	Intergenic	13305	NM_02929	75456	Prps1l1	1700011K1
chr15	75900202	75900275	promoter-T	promoter-T	-78	NM_13408	105782	Scrib	AI118201|(
chr13	1.02E+08	1.02E+08	Intergenic	Intergenic	71834	NM_00102	18708	Pik3r1	PI3K|p50al
chr8	96738277	96738375	promoter-T	promoter-T	-175	NM_17241	71805	Nup93	2410008G(
chr11	33953377	33953725	intron (NM	intron (NM	6350	NM_01069	16822	Lcp2	AI323664|I
chr5	21292652	21292800	intron (NM	CpG-10862	1625	NM_01118	19181	Psmc2	-
chr7	1.09E+08	1.09E+08	intron (NR_	intron (NR_	-1760	NM_00749	11871	Art2a-ps	ARTC2|Art
chr13	97512927	97513100	Intergenic	Intergenic	-72122	NM_00825	15357	Hmgcr	HMG-CoAR
chr18	38465902	38466425	intron (NM	intron (NM	9704	NM_00116	56736	Rnf14	2310075C(
chr2	18688527	18688700	Intergenic	CpG	67964	NM_00100	381350	BC061194	-
chr8	1.23E+08	1.23E+08	intron (NM	intron (NM	22006	NM_03020	78892	Crispld2	1810049K2
chr7	20249727	20249875	intron (NM	MIR3|SINE	578	NM_01964	56457	Clptm1	HS9|N14
chr13	8873002	8873275	Intergenic	Intergenic	-2156	NM_00103	207615	Wdr37	-
chr15	66801127	66801500	promoter-T	promoter-T	-110	NM_00868	17988	Ndrg1	CAP43|CM
chr19	42165177	42165250	exon (NM_	exon (NM_	288	NM_14550	84095	Pi4k2a	Pi4k2
chr17	74297302	74297750	intron (NM	intron (NM	2010	NM_01172	22436	Xdh	XO|Xor|Xo
chr11	51912627	51912700	intron (NM	CpG	337	NM_01941	19052	Ppp2ca	PP2A|R753
chr10	77875302	77875475	intron (NM	CpG	400	NM_01092	18114	Rrp1	AL033305|
chr6	72462527	72462875	Intergenic	Intergenic	-31684	NM_00759	12332	Capg	gCap39|ml
chr8	13757327	13757425	promoter-T	promoter-T	-314	NM_02727	69957	Cdc16	2700071J1
chr5	31939402	31939625	intron (NM	intron (NM	23189	NM_02579	66845	Mrpl33	0610009M
chr9	1.06E+08	1.06E+08	promoter-T	promoter-T	-237	NM_15345	235584	Dusp7	AU015694
chr7	1.36E+08	1.36E+08	5' UTR (NM	5' UTR (NM	130	NM_14595	210711	Mcmbp	1110007A1
chr13	60783402	60783525	intron (NM	intron (NM	79891	NM_13406	69635	Dapk1	D13Ucla1|
chr5	1.3E+08	1.3E+08	promoter-T	promoter-T	20	NM_17227	231769	Sfswap	1190005N2
chr5	1.03E+08	1.03E+08	intron (NM	intron (NM	90241	NM_02927	231532	Arhgap24	0610025G2
chr17	3157177	3157300	intron (NM	RLTR11B|L	42266	NM_13412	106583	Scaf8	A630086M
chr2	1.58E+08	1.58E+08	promoter-T	promoter-T	-403	NM_00848	16803	Lbp	Bpifd2|Ly8
chr7	52459752	52459825	intron (NM	intron (NM	474	NM_20137	384619	Ccdc155	Gm1434|K
chr3	94493052	94493325	Intergenic	(GAA)n|Sir	-4307	NM_01941	20342	Selenbp2	AP56|Lpsb
chr5	1.22E+08	1.22E+08	5' UTR (NM	5' UTR (NM	125	NM_01076	17165	Mapkapk5	MK5|PRAK
chr8	34786577	34786725	intron (NM	intron (NM	22941	NM_01034	14782	Gsr	AI325518|I
chr8	86521227	86521300	promoter-T	promoter-T	-308	NM_00108	666704	Samd1	AA589507
chr18	49846777	49847250	Intergenic	Intergenic	68242	NM_02685	68857	Dtwd2	1190002H(
chr1	36767027	36767100	promoter-T	promoter-T	-293	NM_14610	226977	Actr1b	2310066K2
chr15	11967127	11967250	Intergenic	Intergenic	-41426	NM_01129	20024	Sub1	AI842364|I
chr6	70737027	70737400	intron (NM	intron (NM	4956	NM_00907	19895	Rpia	RPI
chr7	59242227	59242675	intron (NM	intron (NM	18633	NR_02770	75744	Svip	1700006C1
chr13	1.14E+08	1.14E+08	intron (NM	B4A|SINE|	17846	NM_13079	170625	Snx18	Snag1
chr8	89426602	89426800	Intergenic	Intergenic	-17544	NM_03056	80750	N4bp1	AI481586|(
chr17	51015852	51015975	intron (NM	intron (NM	177907	NM_00129	12228	Btg3	ANA|tob5
chr9	96276402	96276525	Intergenic	Intergenic	-11745	NM_00750	11933	Atp1b3	AA409958|
chr19	6235977	6236200	promoter-T	promoter-T	-248	NM_19816	225849	Ppp2r5b	B'beta|BC(
chr11	86014902	86015250	promoter-T	promoter-T	-381	NM_17830	237911	Brip1	3110009N1
chrX	13232477	13232650	intron (NM	intron (NM	-5926	NM_17566	319200	Gpr82	-
chr10	50312227	50312300	promoter-T	promoter-T	-212	NM_19800	77987	Ascc3	ASC1p200|

chr5	36827402	36827550	promoter-T	promoter-T	239	NM_02572	66717	Ccdc96	4921513E0
chr7	52309252	52309450	3' UTR (NM	3' UTR (NM	-1100	NM_17502	233210	Prr12	6720469B1
chr1	1.68E+08	1.68E+08	Intergenic	Intergenic	-23476	NM_00103	226594	Rcsd1	A430105K1
chr2	1.45E+08	1.45E+08	Intergenic	Intergenic	-16334	NM_05319	94249	Slc24a3	NCKX3
chr4	41082852	41083025	promoter-T	promoter-T	-116	NM_02627	67615	Ube2r2	1200003M
chr3	78956827	78957100	Intergenic	Intergenic	-7166	NM_00109	76089	Rapgef2	5830453M
chr5	1.15E+08	1.15E+08	promoter-T	promoter-T	-229	NM_00103	100756	Usp30	6330590F1
chr11	1.02E+08	1.02E+08	intron (NM	CpG	288	NM_00104	21429	Ubtf	A930005G(
chr3	1.33E+08	1.33E+08	promoter-T	promoter-T	366	NM_02623	67553	Gstcd	4933434L1
chr9	44872502	44872725	intron (NM	intron (NM	9349	NM_00109	319742	Mpzl3	5430427F1
chr4	1.19E+08	1.19E+08	5' UTR (NM	5' UTR (NM	122	NM_17269	230700	Foxj3	C330039G(
chr8	63966727	63966900	intron (NM	CpG	282	NM_14559	234309	Cbr4	A730083J1
chr8	70431827	70431900	intron (NM	L1M3|LINE	2982	NM_03026	234353	Psd3	4931420C2
chrX	99239777	99240050	intron (NM	L2a|LINE|L	26733	NM_00127	331474	Rgag4	6430402L0
chr10	80317602	80317675	promoter-T	promoter-T	316	NM_02534	66094	Lsm7	0910001B(
chr7	1.03E+08	1.03E+08	Intergenic	RLTR11A2|	-130696	NM_01185	23966	Tenm4	Doc4|ELM:
chr11	24021302	24021400	intron (NM	intron (NM	40656	NM_00115	14025	Bcl11a	2810047E1
chr11	68685277	68685625	Intergenic	Intergenic	-16604	NM_17706	320040	Rnf222	9930039A1
chr11	57953327	57953425	intron (NM	intron (NM	28665	NM_17255	216766	Gemin5	AA407055|
chr10	10869402	10869550	intron (NM	CpG	248	NM_00128	268281	Shprh	2610103K1
chr2	3430452	3430700	promoter-T	promoter-T	239	NM_17764	227526	Cdnf	9330140G2
chr7	52241377	52241600	promoter-T	promoter-T	302	NM_00125	15469	Prmt1	6720434D(
chr1	1.08E+08	1.08E+08	promoter-T	promoter-T	-83	NM_13382	98432	Phlpp1	AI836256|I
chr18	55643877	55643950	Intergenic	Intergenic	-494079	NM_17575	269023	Zfp608	4932417D1
chr18	13344777	13344975	Intergenic	Intergenic	179377	NM_15308	225192	Hrh4	AXOR35|B(
chr1	46909702	46910075	intron (NM	CpG	466	NM_17265	227059	Slc39a10	2900042E1
chr15	7928777	7928900	intron (NM	intron (NM	120371	NM_00108	545085	Wdr70	4833422F0
chr12	8928427	8928575	5' UTR (NM	5' UTR (NM	388	NM_00864	17775	Laptm4a	AA286466|
chr9	1.14E+08	1.14E+08	exon (NM_	exon (NM_	342	NM_01992	56693	Crtap	5730529N2
chr9	1.15E+08	1.15E+08	intron (NM	intron (NM	25405	NM_15358	78893	Cnot10	2600001P1
chr1	38187027	38187100	promoter-T	promoter-T	-556	NM_01957	56210	Rev1	1110027I2:
chr7	1.27E+08	1.27E+08	intron (NM	intron (NM	14946	NM_17340	233805	Dcun1d3	1700020A1
chr17	43384327	43384425	Intergenic	Intergenic	-22920	NM_13377	77596	Gpr110	5031409J1
chr11	1.06E+08	1.06E+08	promoter-T	promoter-T	-402	NM_00113	83796	Smarcd2	AW322457
chr19	6401352	6401675	intron (NM	CpG	930	NM_01124	19395	Rasgrp2	CDC25L|Ca
chr12	1.06E+08	1.06E+08	Intergenic	Intergenic	-10394	NM_17250	212073	Syne3	nesprin-3
chr6	1.44E+08	1.44E+08	intron (NM	intron (NM	197500	NM_00111	20678	Sox5	A730017D(
chr2	26765977	26767500	promoter-T	promoter-T	411	NM_01372	27176	Rpl7a	Surf3
chr17	51961627	51961750	intron (NM	intron (NM	4225	NM_00116	20230	Satb1	2610306G1
chr14	32150202	32150300	intron (NM	CpG	341	NM_00979	12339	Capn7	AU022319
chr1	1.68E+08	1.68E+08	promoter-T	promoter-T	-160	NM_03024	27878	Tada1	2900026B1
chr8	37330877	37331175	Intergenic	Intergenic	-18456	NM_00108	244421	Lonrf1	-
chr2	1.56E+08	1.56E+08	intron (NM	CpG	107	NM_17721	16328	Cep250	AW490617
chr7	73344077	73344150	Intergenic	Intergenic	89712	NM_00108	269941	Chsy1	mKIAA099(
chr8	47703052	47703125	promoter-T	promoter-T	443	NM_00981	12367	Casp3	A830040C1
chr2	11470077	11470250	intron (NM	intron (NM	5393	NM_00117	170768	Pfkfb3	E330010H2
chr18	35019602	35019700	Intergenic	CpG	-1210	NM_00791	13653	Egr1	A530045N:

chr2	1.49E+08	1.49E+08	intron (NM	intron (NM	4720	NM_02898	74533	Gzf1	8430437G(
chr6	82772002	82772200	Intergenic	Intergenic	-47653	NM_01382	15277	Hk2	AI642394|I
chrX	70960652	70960800	intron (NM	intron (NM	788	NM_01206	27061	Bcap31	Bap31
chr11	1.02E+08	1.02E+08	promoter-T	promoter-T	-302	NM_00128	15184	Hdac5	AI426555|I
chr10	1.15E+08	1.15E+08	promoter-T	promoter-T	-291	NM_02445	216344	Rab21	9630024B2
chr3	1.33E+08	1.33E+08	Intergenic	RMER1C|C	-177359	NM_00104	214133	Tet2	Ayu17-449
chrX	39755202	39755400	Intergenic	Intergenic	-100484	NM_01136	20400	Sh2d1a	Gm686|SA
chr10	19654402	19654500	exon (NM_	exon (NM_	119	NM_00858	26408	Map3k5	7420452D2
chr1	37357452	37357675	intron (NM	intron (NM	880	NM_00129	269180	Inpp4a	107kDa|96
chr11	51355902	51356525	intron (NM	intron (NM	-42046	NM_02839	72947	Phykpl	2900006B1
chr1	39977527	39977675	intron (NM	intron (NM	19843	NM_00869	26921	Map4k4	9430080K1
chr19	37444802	37445250	Intergenic	Intergenic	-5867	NM_01061	16551	Kif11	Eg5|Kif8|K
chr1	93196277	93196800	intron (NM	intron (NM	1623	NM_01671	50880	Scly	9830169H(
chr4	1.33E+08	1.33E+08	Intergenic	Intergenic	58133	NM_00128	20111	Rps6ka1	Mapkapk-1
chr6	1.45E+08	1.45E+08	intron (NM	intron (NM	46606	NM_00102	12035	Bcat1	BCATc|Eca
chr14	79834027	79834200	Intergenic	Intergenic	7795	NM_00102	211255	Kbtbd7	1110008P(
chr7	1.36E+08	1.36E+08	intron (NM	intron (NM	614	NM_00938	21843	Tial1	5330433G1
chr13	49404452	49404725	promoter-T	promoter-T	-11	NM_01575	30938	Fgd3	5830461L0
chr1	37273002	37273175	Intergenic	Intergenic	-2035	NM_00128	12790	Cnga3	CNG3
chr5	1.15E+08	1.15E+08	exon (NM_	exon (NM_	146	NM_00103	100756	Usp30	6330590F1
chr9	54317077	54317500	intron (NM	intron (NM	32145	NM_17277	235380	Dmxl2	6330586A1
chr11	1.06E+08	1.06E+08	promoter-T	promoter-T	15	NM_02946	75870	Tcam1	4930570F0
chr5	1.23E+08	1.23E+08	promoter-T	promoter-T	-179	NM_02688	68948	Fam216a	1500011H2
chr1	79226002	79226200	Intergenic	Intergenic	210564	NM_00912	20254	Scg2	Chgc|SgII
chr2	1.23E+08	1.23E+08	promoter-T	promoter-T	-498	NR_027888	59010	Sqrdl	0610039J1
chr7	13609752	13609950	5' UTR (NM	5' UTR (NM	350	NM_01158	21849	Trim28	AA408787|
chr1	1.8E+08	1.8E+08	Intergenic	CpG	-1030	NM_00116	269152	Kif26b	4832420M
chr10	67984677	67984800	intron (NM	RLTR33|LT	19885	NM_02849	73287	1700040L0	-
chr5	1.23E+08	1.23E+08	intron (NM	B1F2|SINE	16706	NM_00119	207565	Camkk2	6330570N1
chr7	19930377	19930575	promoter-T	promoter-T	56	NM_00794	13870	Ercc1	Ercc-1
chr13	64120202	64120275	Intergenic	Intergenic	70271	NM_00829	15487	Hsd17b3	-
chr13	49062577	49062650	promoter-T	promoter-T	584	NM_00103	218236	Fam120a	C9orf10|O:
chr11	1.07E+08	1.07E+08	intron (NM	intron (NM	1196	NM_02589	66997	Psmd12	1500002F1
chr13	1.09E+08	1.09E+08	promoter-T	promoter-T	94	NM_02804	71991	Ercc8	2410022P(
chr8	1.09E+08	1.09E+08	intron (NM	RSINE1|SIN	88845	NM_17303	272538	Tango6	AW413431
chr11	1.11E+08	1.11E+08	Intergenic	Intergenic	-275046	NM_01060	16517	Kcnj16	6430410F1
chr10	80569402	80569550	intron (NM	intron (NM	784	NM_02313	26396	Map2k2	AA589381|
chr4	1.26E+08	1.26E+08	intron (NM	intron (NM	6775	NM_00114	74178	Stk40	2310004N1
chr11	1.18E+08	1.18E+08	promoter-T	promoter-T	-707	NM_01118	19157	Cyth1	CLM1|CTH
chr10	1.28E+08	1.28E+08	intron (NM	intron (NM	4111	NM_17741	19344	Rab5b	C030027M
chr2	28323677	28323825	intron (NM	CpG	165	NM_00116	118451	Mrps2	1500019M
chr18	38081502	38081650	intron (NM	intron (NM	13489	NM_00785	13367	Diap1	D18Wsu15
chr9	1.03E+08	1.03E+08	promoter-T	promoter-T	-471	NM_01976	56332	Amotl2	AW549739
chr1	1.95E+08	1.95E+08	intron (NM	L1MC4a|LI	12653	NM_00104	15483	Hsd11b1	-
chr15	1.03E+08	1.03E+08	intron (NM	intron (NM	702	NM_02646	67942	Atp5g2	1810041M
chr6	1.34E+08	1.34E+08	intron (NM	intron (NM	99413	NM_00796	14011	Etv6	AW123102
chr3	90269352	90269450	exon (NM_	exon (NM_	387	NM_00872	18160	Npr1	AI893888|(

chr3	1.38E+08	1.38E+08	intron (NM	RLTR19|LT	999	NM_00128	11532	Adh5	Adh-5|Adh
chr10	38928477	38928900	Intergenic	Intergenic	-45088	NM_00112	327743	Wisp3	CCN6|ENSI
chr10	1.28E+08	1.28E+08	intron (NM	intron (NM	1749	NM_05407	116848	Baz2a	AA415431|
chr8	54688527	54688625	intron (NM	intron (NM	35843	NM_14620	234258	Neil3	AI449477|
chr9	44616927	44617500	exon (NM_	exon (NM_	-6179	NM_13895	192653	Ttc36	-
chr2	1.44E+08	1.44E+08	5' UTR (NM	5' UTR (NM	173	NM_13362	81910	Rrbp1	1700087N(
chr7	74872652	74872875	Intergenic	MTE-int|LT	31865	NM_18331	233335	Synm	4930412K2
chr2	32837252	32837475	exon (NM_	exon (NM_	213	NM_01948	56017	Slc2a8	D2Ertd44e
chr3	1.46E+08	1.46E+08	intron (NM	L1M3|LINE	7813	NM_02298	65086	Lpar3	Edg7|lpA3
chr3	1.44E+08	1.44E+08	Intergenic	Intergenic	-176044	NM_01072	16911	Lmo4	A730077C1
chr8	93400227	93400400	intron (NM	intron (NM	47579	NM_17722	109151	Chd9	1810014J1
chr19	36908377	36908550	promoter-T	promoter-T	-259	NM_00116	74493	Tnks2	5430432P1
chr18	23475877	23476225	intron (NM	intron (NM	7530	NM_00128	13527	Dtna	2210407P2
chr19	10763352	10763575	5' UTR (NM	5' UTR (NM	158	NM_18140	107305	Vps37c	5730409F2
chr1	1.3E+08	1.3E+08	intron (NM	intron (NM	1122	NM_02839	72949	Ccnt2	2900041I1
chr2	1.31E+08	1.31E+08	intron (NR_	intron (NR_	567	NR_11099	69596	Ap5s1	0610038L1
chr3	1.17E+08	1.17E+08	intron (NM	intron (NM	333	NM_00108	77559	Agl	1110061O1
chr3	93273602	93273825	intron (NM	intron (NM	1037	NM_02776	71325	Tchhl1	5430400H2
chr3	1.38E+08	1.38E+08	intron (NM	intron (NM	12799	NM_00103	70604	Dnajb14	5730496F1
chr1	92953077	92953200	intron (NM	intron (NM	3117	NM_00851	16978	Lrrfip1	AU024550
chr15	31471327	31471425	Intergenic	Intergenic	-10584	NM_17260	223455	6-Mar	3830408G(
chr13	51476877	51477275	Intergenic	Intergenic	-26911	NM_01010	13610	S1pr3	AI132464|
chr1	1.89E+08	1.89E+08	Intergenic	Intergenic	-53294	NM_03307	110957	D1Pas1	Pl10
chr7	1.38E+08	1.38E+08	intron (NM	intron (NM	55611	NM_00776	12945	Dmbt1	CRP|CRP-[
chr17	63848727	63849425	exon (NM_	exon (NM_	853	NM_01579	50758	Fbxl17	6330576B(
chr1	16608402	16608475	intron (NR_	intron (NR_	981	NM_02577	66799	Ube2w	6130401J0
chr4	63070352	63070475	Intergenic	Intergenic	-5934	NM_00104	100182	Akna	AI597013|
chr7	36019302	36019625	Intergenic	Intergenic	-19099	NM_00102	435965	Lrp3	-
chr1	79858177	79858450	Intergenic	Intergenic	-3073	NM_00925	20720	Serpine2	B230326M
chr1	52556952	52557050	intron (NM	CpG	291	NM_00866	17936	Nab1	-
chr19	11715402	11715525	promoter-T	promoter-T	-122	NM_13324	170813	Ms4a3	HTm4
chr1	63223027	63223225	promoter-T	promoter-T	270	NM_14551	227197	Ndufs1	5830412M
chr18	53405127	53405525	promoter-T	promoter-T	10	NM_02939	69226	Snx24	2810011K1
chr7	35098777	35098850	Intergenic	Intergenic	-28185	NM_17274	233103	4931406P1	AA553327|
chr17	7032627	7032700	Intergenic	CpG	-45534	NM_00951	22350	Ezr	AW146364
chr8	1.24E+08	1.24E+08	intron (NM	intron (NM	12168	NM_00103	407789	BC048644	-
chr11	58073602	58073875	Intergenic	Intergenic	45259	NM_01944	54396	Irgm2	AI481100|
chr2	26264527	26264700	promoter-T	promoter-T	34	NM_00129	64436	Inpp5e	1200002L2
chr8	37091802	37091950	Intergenic	Intergenic	-66006	NM_17291	244418	D8Ertd82e	9830148H2
chr11	61743002	61743100	intron (NM	intron (NM	708	NM_01992	56697	Akap10	1500031L1
chr10	77046577	77046675	intron (NM	intron (NM	2114	NM_14592	108705	Pttg1ip	1810010L2
chr14	25309552	25311075	intron (NM	CpG	410	NM_01129	20088	Rps24	-
chr4	1.55E+08	1.55E+08	promoter-T	promoter-T	-514	NM_02557	66448	Mrpl20	2610008D(
chr5	1.44E+08	1.44E+08	intron (NM	RSINE1|SIN	1465	NM_14491	231871	Daglb	E330036I1
chr6	72222177	72222325	Intergenic	Intergenic	-32353	NM_00128	20388	Sftpb	AI562151|
chr5	1.45E+08	1.45E+08	promoter-T	promoter-T	132	NM_00117	231872	Aimp2	AA407136|
chr5	42236277	42236400	promoter-T	promoter-T	-784	NM_00108	665775	Bod1l	A230054D(

chr11	1.01E+08	1.01E+08	exon (NM_	exon (NM_	392	NM_02677	28084	Vps25	1110020N1
chr7	1.38E+08	1.38E+08	intron (NM	intron (NM	54653	NM_20685	57752	Tacc2	mKIAA418(
chr19	4222227	4222425	exon (NM_	exon (NM_	7934	NM_01995	56708	Clcf1	Bsf3|CLC
chr14	77437952	77438050	intron (NM	intron (NM	1422	NM_17536	108811	Ccdc122	4933415L0
chr5	1.38E+08	1.38E+08	intron (NM	intron (NM	3585	NM_00125	68929	Mospd3	1190005J1
chr2	62483627	62483900	exon (NM_	exon (NM_	549	NM_02783	71586	Ifih1	9130009C2
chr10	83976902	83977100	Intergenic	Intergenic	19632	NM_17545	216197	Ckap4	5630400A(
chr16	33747877	33748075	intron (NM	L1MA6|LIN	-46147	NM_01073	17063	Muc13	114/A10|1
chr12	55757002	55757225	intron (NM	intron (NM	446	NM_13405	104725	Sptssa	1110002B(
chr6	7598427	7598650	Intergenic	ORR1B1-in	44644	NM_01205	27053	Asns	-
chr7	1.08E+08	1.08E+08	intron (NM	intron (NM	7301	NM_17707	320100	Relt	E430021K2
chrX	11665227	11665400	intron (NM	CpG	-7634	NM_02951	71458	Bcor	5830466J1
chr1	1.93E+08	1.93E+08	Intergenic	Intergenic	-83393	NM_14488	226849	Ppp2r5a	PR61alpha
chr3	1.33E+08	1.33E+08	Intergenic	Intergenic	-39309	NM_00104	214133	Tet2	Ayu17-449
chr11	1.16E+08	1.16E+08	intron (NM	PB1D10|SI	35970	NM_02725	217340	Rnf157	2610036E2
chr10	60030052	60030175	intron (NR_	intron (NR_	129125	NM_00125	22295	Cdh23	4930542A(
chr2	1.67E+08	1.67E+08	Intergenic	B1_Mur4|S	-28210	NM_01983	56336	B4galt5	9430078I0
chr13	43306602	43306675	intron (NM	intron (NM	-39768	NM_00125	67046	Tbc1d7	2610009C(
chr12	16084127	16084200	Intergenic	Intergenic	-260572	NM_14455	217410	Trib2	AW319517
chr13	95531577	95531675	Intergenic	Intergenic	-27272	NM_00932	21371	Tbca	Tbca13
chr5	29896227	29896425	intron (NM	CpG	544	NM_13390	100763	Ube3c	AI853514|
chr8	1.2E+08	1.2E+08	intron (NM	CpG	223	NM_02657	68133	Gcsh	1100001L0
chr3	1.29E+08	1.29E+08	intron (NM	CpG	1834	NM_13045	170439	Elovl6	C77826|FA
chr2	70499477	70499600	promoter-T	promoter-T	-28	NR_027343	70231	Gorasp2	0610011A(
chr2	73150677	73150900	promoter-T	promoter-T	79	NM_02902	74616	Scrn3	4833415E2
chr5	1.36E+08	1.36E+08	Intergenic	Intergenic	-13396	NM_01024	14371	Fzd9	mfz9
chr11	4160902	4161000	intron (NM	intron (NM	3380	NM_00850	16878	Lif	-
chr15	57807952	57808025	Intergenic	Intergenic	-9470	NM_17386	239463	Fam83a	-
chr6	52167002	52167175	exon (NM_	exon (NM_	1484	NM_01045	15404	Hoxa7	AV118143|
chr13	74946052	74946300	promoter-T	promoter-T	32	NM_00130	12380	Cast	-
chr6	4538027	4538175	Intergenic	LTR37A|LT	-12965	NM_14539	213819	Casd1	Cas1|Cast1
chr15	98859902	98860325	promoter-T	promoter-T	-209	NM_00944	22146	Tuba1c	M[a]6|Tub
chr1	1.33E+08	1.33E+08	intron (NM	intron (NM	13194	NM_00855	17164	Mapkapk2	AA960234|
chr10	1.27E+08	1.27E+08	intron (NM	intron (NM	839	NM_03307	110962	Mbd6	D10Wsu93
chr5	34333977	34334075	Intergenic	ETnERV2-ir	-4607	NM_00100	269642	Nat8l	1110038O(
chr5	1.33E+08	1.33E+08	intron (NM	intron (NM	75112	NM_17704	319974	Auts2	2700063G(
chr8	13758602	13758675	intron (NM	intron (NM	948	NM_02727	69957	Cdc16	2700071J1
chr14	45945727	45945800	Intergenic	Intergenic	-3736	NM_02595	67089	Psmc6	2300001E(
chr17	29169002	29169200	promoter-T	promoter-T	-504	NM_01366	20383	Srsf3	AL024116|
chr11	58767577	58767825	promoter-T	promoter-T	313	NM_20688	382522	Hist3h2bb-	4930534G1
chr7	1.28E+08	1.28E+08	intron (NM	intron (NM	11779	NM_02155	59052	Mettl9	0610012D(
chr13	81768552	81768850	intron (NM	intron (NM	3442	NM_05405	110789	Gpr98	Frings|Mas
chr8	34751852	34752000	intron (NM	intron (NM	522	NM_17864	108159	Ubxn8	D0H8S229
chr14	51405052	51405225	promoter-T	promoter-T	57	NM_17762	219022	Ttc5	-
chr13	56105302	56105450	Intergenic	Intergenic	131535	NM_01201	26914	H2afy	H2AF12M|
chr3	68590352	68590475	Intergenic	Intergenic	-83095	NM_00116	68725	1110032F0	AI115547
chr1	1.41E+08	1.41E+08	promoter-T	promoter-T	-47	NM_17264	226470	Zbtb41	8430415N2

chr8	89270102	89270250	promoter-T	promoter-T	-271	NM_00917	20437	Siah1a	AA982064|
chr5	1.09E+08	1.09E+08	promoter-T	promoter-T	395	NM_15356	231580	Gak	D130045N
chr5	1.07E+08	1.07E+08	promoter-T	promoter-T	-252	NM_02685	52397	Zfp644	1110068L0
chr2	1.78E+08	1.78E+08	intron (NM	Zaphod|DN	1275	NM_17719	320558	Sycp2	3830402K2
chr2	1.09E+08	1.09E+08	promoter-T	promoter-T	96	NM_02979	76894	Mettl15	0610027B0
chr16	32180452	32180825	intron (NM	intron (NM	752	NM_00103	328660	Bex6	B020003O0
chr2	1.05E+08	1.05E+08	promoter-T	promoter-T	-485	NM_00112	99003	Qser1	4732486I2
chr1	1.84E+08	1.84E+08	Intergenic	RMER1B|C	-34656	NM_13381	98386	Lbr	AI505894|i
chr2	1.37E+08	1.37E+08	intron (NM	intron (NM	80039	NM_00103	74243	Slx4ip	2210009G2
chr6	1.41E+08	1.41E+08	intron (NM	intron (NM	37032	NM_00963	11569	Aebp2	AU023766
chr7	63049627	63049975	intron (NM	intron (NM	283	NM_14619	233276	Tubgcp5	B130010C1
chr14	64086777	64086850	Intergenic	Intergenic	31582	NM_17762	219148	Fam167a	A030013D2
chr12	55263677	55263975	Intergenic	Intergenic	41035	NM_02813	112407	Egln3	2610021G0
chr4	1.08E+08	1.08E+08	Intergenic	Intergenic	10459	NM_02875	74098	0610037L1	1110008H1
chr11	51433327	51433500	exon (NM_	exon (NM_	138	NM_02663	52530	Nhp2	2410130M
chr17	57333477	57333700	promoter-T	promoter-T	16	NM_01941	50930	Tnfsf14	HVEM-L|H
chr17	28669277	28669800	intron (NM	intron (NM	1732	NM_02629	67645	Armc12	4930511I1
chr16	33056052	33057650	promoter-T	promoter-T	251	NM_02133	57808	Rpl35a	2810431L1
chr15	8683327	8683475	Intergenic	Intergenic	-22594	NM_14893	20512	Slc1a3	AI504299|
chr6	83051627	83051700	5' UTR (NM	5' UTR (NM	153	NM_00108	243510	Ccdc142	A230058J2
chr1	36614727	36614950	intron (NM	CpG	388	NM_00112	20353	Sema4c	AI426163|
chr7	56164502	56164625	Intergenic	Intergenic	-28152	NM_00101	108961	E2f8	4432406C0
chr4	1.4E+08	1.4E+08	Intergenic	HAL1-2a_M	-1406	NM_00111	72754	Arhgef10l	2810441C0
chr9	44795627	44795800	TTS (NM_0	TTS (NM_0	5844	NM_01348	12500	Cd3d	T3d
chr2	1.74E+08	1.74E+08	promoter-T	promoter-T	-714	NM_17267	228960	Stx16	4930401D0
chr7	1.35E+08	1.35E+08	intron (NM	intron (NM	7551	NM_13335	76560	Prss8	2410039E1
chr15	73317752	73318225	Intergenic	Intergenic	-25002	NM_00108	105841	Dennd3	AI447457|
chr14	21666452	21666525	Intergenic	B3|SINE|B	10604	NM_00887	18792	Plau	u-PA|uPA
chr5	1.01E+08	1.01E+08	intron (NM	intron (NM	17662	NM_02797	71883	Coq2	2310002F1
chr3	8969602	8969775	intron (NM	intron (NM	-5634	NM_00102	21985	Tpd52	AI043038|
chr5	1.23E+08	1.23E+08	intron (NM	intron (NM	8181	NM_00119	207565	Camkk2	6330570N1
chr16	87612327	87613100	Intergenic	Intergenic	59138	NM_14485	224419	Map3k7cl	C21orf7|O
chr5	1.18E+08	1.18E+08	intron (NM	CpG	280	NM_02134	57816	Tesc	1010001A1
chrX	1.26E+08	1.26E+08	intron (NM	intron (NM	209823	NM_17249	54004	Diap2	Dia3|Diaph
chr14	63380402	63380650	promoter-T	promoter-T	-577	NM_00871	18130	Ints6	2900075H2
chr8	59958577	59958775	Intergenic	Intergenic	7981	NM_02178	60406	Sap30	30kDa
chr11	49624827	49624925	intron (NM	intron (NM	17219	NM_01352	14584	Gfpt2	AI480523|
chr1	54969402	54969475	intron (NM	intron (NM	13793	NM_00108	329154	Ankrd44	4930444A1
chr10	61971327	61971525	promoter-T	promoter-T	-923	NM_01115	19073	Srgn	Prg|Prg1|S
chr19	10006227	10006375	Intergenic	Intergenic	-32278	NM_01669	16319	Incenp	2700067E2
chr8	87246727	87246925	intron (NM	intron (NM	21470	NM_00853	17095	Lyl1	Lyl-1|bHLH
chr1	1.71E+08	1.71E+08	Intergenic	Intergenic	373207	NM_02328	66977	Nuf2	2410003C0
chr3	1.07E+08	1.07E+08	Intergenic	Lx2A1|LINE	-13076	NM_14648	258482	Olfr266	MOR122-2
chr11	63779027	63779250	intron (NM	intron (NM	-43352	NM_01880	54710	Hs3st3b1	3-OST-3B|3
chr2	1.17E+08	1.17E+08	promoter-T	promoter-T	-731	NM_00127	114715	Spred1	5730461F1
chr16	41032377	41032650	Intergenic	B1F1|SINE	-500942	NM_17554	268890	Lsamp	5430428I1
chr4	40917752	40918500	intron (NM	CpG	150	NM_02373	74164	Nfx1	1300017N1

chr1	97296302	97296425	Intergenic	B3A|SINE|	86158	NM_02632	67698	Fam174a	2310044D2
chr14	37948152	37948500	promoter-T	promoter-T	-1	NM_00119	66092	Ghitm	1010001P1
chr2	1.28E+08	1.28E+08	intron (NM	intron (NM	28351	NM_01690	53885	Nphp1	-
chr11	79391477	79391600	intron (NM	intron (NM	-13176	NM_17554	268451	Rab11fip4	A730072L0
chr12	99497202	99497450	intron (NM	CpG	221	NM_00807	14420	Galc	2310068B0
chr9	63606427	63606625	promoter-T	promoter-T	-725	NM_01676	17127	Smad3	AU022421
chr19	45235077	45235200	Intergenic	Intergenic	9933	NM_02190	21908	Tlx1	Hox-11|Ho
chr4	1.16E+08	1.16E+08	intron (NM	intron (NM	293	NM_02814	72181	Nsun4	2310010O1
chr1	1.36E+08	1.36E+08	intron (NM	CpG	256	NM_00922	20643	Snrpe	AL022645|
chr17	47505777	47505850	promoter-T	promoter-T	-23	NM_00114	64657	Mrps10	1110038B1
chr2	28001277	28001550	Intergenic	Intergenic	-47200	NM_00103	56177	Olfm1	AMY|AW74
chr17	32177452	32177550	intron (NM	RSINE1|SIN	4394	NM_02824	72462	Rrp1b	2600005C2
chr10	87866127	87866250	intron (NM	Charlie1a|I	24031	NM_00100	432486	Gnptab	EG432486|
chr8	23235302	23235375	intron (NM	intron (NM	187	NM_17789	330721	Nek5	6530411J2
chr11	60988102	60988175	intron (NM	CpG	423	NM_00100	216825	Usp22	AI427806
chr4	86502227	86503975	intron (NM	CpG	170	NM_00909	20104	Rps6	S6R
chr13	77970027	77970150	intron (NM	Lx8|LINE|L	122137	NM_00116	68675	Fam172a	1110033M
chr8	1.22E+08	1.22E+08	Intergenic	Intergenic	-8854	NM_01996	56690	Mlycd	AI324784|I
chr15	36426327	36426525	intron (NM	CpG	120	NM_17513	68839	Ankrd46	1110054N0
chr16	37011827	37011900	promoter-T	promoter-T	-9	NM_02997	77782	Polq	A430110D1
chr13	1.05E+08	1.05E+08	Intergenic	CpG	-9251	NM_17259	218543	Srek1	8430401B0
chr15	39509802	39509925	intron (NM	MLT1B|LTF	18806	NM_00125	116838	Rims2	2810036I1
chr11	16887827	16887950	intron (NM	intron (NM	20833	NM_01954	56193	Plek	2010300B1
chr4	1.03E+08	1.03E+08	promoter-T	promoter-T	68	NM_00103	71148	Mier1	4933425I2
chr5	1.48E+08	1.48E+08	Intergenic	Intergenic	-10578	NM_00125	76366	Mtif3	2810012L1
chr12	1.04E+08	1.04E+08	exon (NM_	exon (NM_	27516	NM_02566	66622	Ubr7	5730410I1
chr4	1.18E+08	1.18E+08	intron (NM	intron (NM	1122	NM_02693	69072	Ebna1bp2	1810014B1
chr6	1.47E+08	1.47E+08	intron (NM	(ATGGTG)n	2911	NM_00128	272322	Arntl2	4632430A0
chr7	52884052	52884275	exon (NM_	exon (NM_	1256	NM_02854	69903	Rasip1	1110025D0
chr8	1.26E+08	1.26E+08	promoter-T	promoter-T	346	NR_03786	77087	Ankrd11	2410104C1
chr1	1.59E+08	1.59E+08	Intergenic	Intergenic	-1772	NM_14541	215015	Fam20b	C530043G2
chr1	1.29E+08	1.29E+08	Intergenic	Intergenic	-46992	NM_17695	210356	Nckap5	8430408F2
chr3	84470952	84471025	intron (NM	RMER1B|C	874	NM_17726	320782	Tmem154	9930117H0
chr2	1.48E+08	1.48E+08	exon (NM_	exon (NM_	1661	NM_00937	21824	Thbd	AI385582|
chr5	97221452	97221525	promoter-T	promoter-T	-916	NM_01347	11745	Anxa3	Anx3
chr8	1.23E+08	1.23E+08	Intergenic	Intergenic	-43440	NM_19867	382034	Gse1	2210013I1
chr11	1.11E+08	1.11E+08	Intergenic	Intergenic	22698	NM_00842	16518	Kcnj2	IRK1|Kcnf1
chr11	84936952	84937050	intron (NM	intron (NM	16456	NM_00102	237898	Usp32	2900074J0
chr5	1.09E+08	1.09E+08	promoter-T	promoter-T	-247	NM_00116	116701	Fgfrl1	FGFR5|FGF
chr4	11118052	11118275	promoter-T	promoter-T	-335	NM_00103	12448	Ccne2	-
chr9	66124752	66124950	Intergenic	Intergenic	-73406	NM_14561	235439	Herc1	2810449H1
chr8	1.31E+08	1.31E+08	Intergenic	Intergenic	-1310	NM_00873	18186	Nrp1	C530029I0
chr5	1.08E+08	1.08E+08	intron (NM	CpG	5825	NM_00796	14020	Evi5	NB4S
chr3	32607652	32608025	intron (NM	CpG	370	NM_01967	56456	Actl6a	2810432C0
chr14	87816752	87816850	intron (NM	CpG	411	NM_00125	219249	Tdrd3	4732418C0
chr9	77764977	77765050	promoter-T	promoter-T	-159	NM_13425	68801	Elovl5	1110059L2
chr13	9418452	9418725	intron (NM	intron (NM	142817	NM_00108	208440	Dip2c	2900024P2

chr4	1.39E+08	1.39E+08	intron (NM	CpG	162	NM_00103	12345	Capzb	1700120C0
chr9	1.01E+08	1.01E+08	exon (NM_	exon (NM_	552	NM_00110	77853	Msl2	E130103E0
chr4	1.39E+08	1.39E+08	intron (NM	CpG	264	NM_00116	69116	Ubr4	1810009A1
chr11	95153852	95153925	intron (NM	intron (NM	17672	NM_00119	217127	Kat7	Hbo1|Hbo
chr12	35669927	35670050	exon (NM_	exon (NM_	555	NM_02929	75456	Prps1l1	1700011K1
chr10	84084952	84085275	promoter-T	promoter-T	-69	NM_02742	70428	Polr3b	2700078H0
chr13	58229377	58229525	exon (NM_	exon (NM_	466	NM_02987	77134	Hnrnpa0	1110055B0
chr18	12280302	12280475	promoter-T	promoter-T	-342	NM_00128	338363	Tmem241	6030446N2
chr9	1.23E+08	1.23E+08	promoter-T	promoter-T	147	NM_02691	69035	Zdhhc3	1110020O2
chr5	53840952	53841175	Intergenic	Intergenic	-105955	NM_00108	19664	Rbpj	AI843960|
chr5	1.06E+08	1.06E+08	Intergenic	RLTR13D3|	-21868	NM_00103	433926	Lrrc8b	R75581|Ta
chr1	10222027	10222100	intron (NM	intron (NM	688	NM_00110	211673	Arfgef1	ARFGEP1|E
chrY	1790652	1790775	Intergenic	Lx3A|LINE|	-64296	NM_00111	1E+08	Gm16501	ENSMUSG0
chr17	27148877	27149075	Intergenic	MTEa|LTR|	-6004	NM_00125	70772	Ggnbp1	0610031G0
chr15	97616352	97616500	intron (NM	intron (NM	1630	NM_02635	67739	Slc48a1	4930570C0
chr14	22771927	22772150	Intergenic	Intergenic	36785	NM_14545	218820	Zfp503	AI181838|
chr3	1.02E+08	1.02E+08	Intergenic	Intergenic	104584	NM_01360	18049	Ngf	Ngfb
chr19	43827577	43827700	promoter-T	promoter-T	125	NM_02553	66388	Cutc	2310039I1
chr1	72555927	72556050	intron (NM	intron (NM	27150	NM_00104	381270	4-Mar	BC056494|
chr10	79390752	79390925	intron (NM	intron (NM	752	NM_00128	13496	Arid3a	Bright|Dri1
chr17	71824252	71824425	exon (NM_	exon (NM_	345	NM_02888	74355	Smchd1	4931400A1
chr4	1.2E+08	1.2E+08	5' UTR (NM	5' UTR (NM	158	NM_00127	18046	Nfyc	-
chr1	36425327	36425900	intron (NM	CpG	413	NM_17265	226976	Kansl3	4632411B1
chr6	1.27E+08	1.27E+08	Intergenic	L3b|LINE|	-56680	NM_00103	381812	Cracr2a	Efcab4b|G
chr7	30954527	30954600	promoter-T	promoter-T	183	NM_01198	26465	Zfp146	OZF|Znf14
chr8	95007127	95007525	Intergenic	Intergenic	125631	NM_01882	54352	Irx5	-
chr13	55422452	55422600	promoter-T	promoter-T	815	NM_00900	19336	Rab24	6530406O0
chr14	56441652	56442000	Intergenic	Intergenic	-1806	NM_00116	73181	Nfatc4	3110041H0
chr10	95200427	95200600	Intergenic	Intergenic	-173712	NM_02772	71207	Nudt4	4933436C1
chr4	46498752	46498900	Intergenic	Intergenic	-3375	NM_05317	94181	Nans	4632418E0
chr1	43990452	43990925	promoter-T	promoter-T	-164	NM_00941	22019	Tpp2	TPP-2|Tpp
chr8	77517327	77517975	promoter-T	promoter-T	49	NM_17801	70823	Hmgxb4	4733401K0
chr1	1.87E+08	1.87E+08	Intergenic	Intergenic	-2804	NM_00825	15284	Hlx	Hlx1
chr7	53128827	53129150	intron (NM	intron (NM	778	NM_13395	68137	Kdelr1	8030486F0
chr3	1.07E+08	1.07E+08	5' UTR (NM	5' UTR (NM	190	NM_14554	229709	Ahcyl1	1110034F2
chr7	86047627	86047900	intron (NM	intron (NM	6950	NM_00116	68048	Aen	2700083B0
chr5	24606352	24606425	promoter-T	promoter-T	72	NM_14540	108099	Prkag2	2410051C1
chr11	1.03E+08	1.03E+08	intron (NM	intron (NM	643	NM_18328	544817	Arhgap27	2310069I0
chr10	7736577	7736675	Intergenic	Intergenic	-60705	NM_13866	68652	Tab2	1110030N0
chr1	74202127	74202225	intron (NM	intron (NM	1608	NM_00990	12765	Cxcr2	CD128|CD
chr19	29444677	29444900	intron (NM	MER20|DN	2860	NM_02189	60533	Cd274	A530045L1
chr16	14132102	14132250	intron (NM	intron (NM	27191	NM_00108	223989	Marf1	4921513D2
chr10	59723577	59723650	Intergenic	Intergenic	-16763	NM_00114	19156	Psap	AI037048|
chr14	56262002	56262550	5' UTR (NM	5' UTR (NM	367	NM_02878	74140	Tm9sf1	1200014D0
chr18	74518127	74518325	intron (NR_	LTRIS_Mus	75472	NM_02894	74453	Ccdc11	4933415I0
chr14	52877202	52877300	Intergenic	CpG	-20004	NM_20163	67772	Chd8	5830451P1
chr14	31814377	31814575	promoter-T	promoter-T	-103	NM_00116	76485	Glt8d1	2410004H0

chr18	44989052	44989225	intron (NM	intron (NM	819	NM_00116	240255	Ythdc2	3010002F0
chr9	1.22E+08	1.22E+08	promoter-T	promoter-T	-154	NM_17867	215474	Sec22c	4932412K2
chr16	48799652	48799975	Intergenic	Intergenic	-17156	NM_02388	57263	Retnlb	9030012B2
chr4	1.33E+08	1.33E+08	Intergenic	Intergenic	-12610	NM_15342	242687	Wasf2	AW742646
chr7	74280027	74280325	intron (NM	intron (NM	-87254	NM_17521	75099	Lysmd4	4930506D2
chr3	97705002	97705175	promoter-T	promoter-T	-62	NM_01134	20333	Sec22b	4930564D1
chr15	1.01E+08	1.01E+08	Intergenic	Intergenic	-20014	NM_01044	15370	Nr4a1	GFRP1|Gfr
chr2	1.65E+08	1.65E+08	promoter-T	promoter-T	324	NM_17837	67538	Zswim3	4921517A0
chr15	65951927	65952200	intron (NM	intron (NM	-143697	NM_00114	654498	Hhla1	F930104E1
chr12	32319527	32319800	promoter-T	promoter-T	-140	NM_00116	12033	Bcap29	AW208404
chr7	1.07E+08	1.07E+08	Intergenic	Intergenic	14677	NM_14635	258353	Olfr521	MOR101-2
chr4	94881977	94882225	Intergenic	RMER19C|	-163188	NM_01059	16476	Jun	AP-1|Junc|
chr14	52487277	52487450	Intergenic	Intergenic	13690	NM_01738	53873	Ear7	mR7
chr3	1.58E+08	1.58E+08	promoter-T	promoter-T	-754	NM_00101	433667	Ankrd13c	AI505652|
chr8	75071802	75072025	5' UTR (NM	5' UTR (NM	296	NM_02748	70625	Med26	5730493L1
chr16	30171527	30171900	Intergenic	Intergenic	95905	NM_02790	71756	Cpn2	1300018K1
chr17	8828702	8828900	intron (NM	intron (NM	109135	NM_00129	23984	Pde10a	-
chr14	57506227	57506450	promoter-T	promoter-T	-294	NM_02949	76007	Zmym2	5830413P0
chr11	93746627	93746750	promoter-T	promoter-T	392	NM_00101	217109	Utp18	6230425C2
chr7	1.48E+08	1.48E+08	Intergenic	Intergenic	20605	NM_02537	66141	Ifitm3	1110004C0
chr14	1.04E+08	1.04E+08	Intergenic	Intergenic	50030	NM_00790	13618	Ednrb	ET-B|ET-BR
chr1	66910102	66910275	promoter-T	promoter-T	-305	NM_00738	11363	Acadl	AA960361|
chr3	54433252	54433375	Intergenic	B1_Mur1|S	-63714	NM_01999	56790	Supt20	AA667204|
chr17	12700052	12700125	exon (NM_	exon (NM_	482	NM_01139	20519	Slc22a3	EMT|Oct3|
chr4	14753302	14753425	intron (NM	intron (NM	371	NM_15281	72201	Otud6b	2600013N1
chr3	1.3E+08	1.3E+08	Intergenic	Intergenic	-12313	NM_20720	99683	Sec24b	AI605202|S
chr2	56976652	56976775	promoter-T	promoter-T	-299	NM_00113	18227	Nr4a2	HZF-3|NOT
chr9	65738402	65738500	intron (NM	intron (NM	321	NM_02651	68026	2810417H1	AA409629|
chr11	1.01E+08	1.01E+08	intron (NM	intron (NM	7874	NM_21366	20848	Stat3	1110034C0
chr10	80958927	80959000	exon (NM_	exon (NM_	145	NM_13400	103425	Ncln	3100002P1
chr7	20158752	20158875	promoter-T	promoter-T	-121	NM_02718	69731	Gemin7	2400008I04
chr2	57482052	57482175	Intergenic	(TTAGGG)n	-368453	NM_17285	241391	Galnt5	4832424J2
chr1	88337702	88337825	Intergenic	Intergenic	14859	NM_02796	71863	1700019O1	-
chr1	1.83E+08	1.83E+08	intron (NM	CpG	183	NM_00821	15078	H3f3a	H3.3A
chr6	1.03E+08	1.03E+08	Intergenic	Intergenic	-38294	NM_00769	12661	Chl1	A530023M
chr6	1.43E+08	1.43E+08	Intergenic	Intergenic	15225	NM_00842	16523	Kcnj8	AI448900|
chr19	46380577	46380650	intron (NM	intron (NM	193	NM_00117	18034	Nfkb2	NF-kappaB
chr6	1.49E+08	1.49E+08	promoter-T	promoter-T	-34	NM_00125	320204	Mettl20	4833442J1
chr15	57624877	57624975	intron (NM	intron (NM	98704	NM_19944	387609	Zhx2	Afr-1|Afr1
chr3	24462902	24463075	Intergenic	RMER16-in	1589241	NM_00116	192167	Nlgn1	6330415N0
chr3	96801827	96801900	Intergenic	Intergenic	-34462	NM_00127	14613	Gja5	5730555N1
chr5	92804527	92804850	intron (NM	intron (NM	-12366	NR_038116	56066	Cxcl11	Cxc11|H17
chr11	62283977	62284300	intron (NM	L1MB4|LIN	12176	NM_00103	327942	Pigl	Gm737
chr10	1.11E+08	1.11E+08	exon (NM_	exon (NM_	10607	NM_02860	73690	Glipr1	2410114O1
chr5	64689577	64689750	intron (NM	intron (NM	68067	NM_00128	57915	Tbc1d1	1110062G0
chr6	1.47E+08	1.47E+08	Intergenic	Intergenic	-69481	NM_00897	19227	Pthlh	PTH-like|Pt
chr2	21728277	21728500	intron (NM	Lx8|LINE|L	-420742	NM_14841	667663	Myo3a	9030416P0

chr7	1.48E+08	1.48E+08	promoter-T	promoter-T	-47	NM_00103	213002	Ifitm6	A330075D(
chr9	64302127	64302350	intron (NM	intron (NM	68805	NM_00113	214058	Megf11	2410080H(
chr10	75495802	75495925	intron (NM	intron (NM	506	NM_17747	64453	Zfp280b	D10Jhu82e
chr15	90998352	90998500	intron (NM	intron (NM	23812	NM_01199	26874	Abcd2	ABC39|ALD
chr13	1.01E+08	1.01E+08	Intergenic	(TG)n|Simp	140925	NM_01373	27220	Cartpt	Cart
chr10	42741227	42741375	exon (NM_	exon (NM_	153035	NM_17540	109205	Sobp	2900009C1
chr10	1.28E+08	1.28E+08	promoter-T	promoter-T	-127	NM_01995	56530	Cnpy2	5330432A1
chr17	43013302	43013550	promoter-T	promoter-T	-53	NM_00984	12488	Cd2ap	AL024079|
chr13	59788952	59789175	3' UTR (NM	3' UTR (NM	6399	NM_18329	70900	4921517D2	-
chr5	1.41E+08	1.41E+08	promoter-T	promoter-T	-821	NM_13391	27979	Eif3b	AL033316|
chr5	1.11E+08	1.11E+08	promoter-T	promoter-T	-298	NM_00112	231605	Galnt9	GalNAc-T9
chr14	21553502	21553750	TTS (NM_1	TTS (NM_1	158	NM_01081	17423	Ndst2	Mndns|ND
chr3	21861252	21861450	Intergenic	Intergenic	-114223	NM_03073	81004	Tbl1xr1	8030499H(
chr9	57115977	57116150	Intergenic	Intergenic	-5657	NM_02882	74211	1700017B(	AA682102|
chr11	97579677	97579925	3' UTR (NM	3' UTR (NM	15053	NM_01197	26446	Psmb3	AL033320|
chr7	1.38E+08	1.38E+08	intron (NM	intron (NM	-34832	NM_02131	57752	Tacc2	mKIAA418(
chr11	78687052	78687125	Intergenic	L1MC|LINE	46991	NM_00107	66274	Lyrm9	1810012P1
chr6	1.23E+08	1.23E+08	intron (NM	intron (NM	2560	NM_00100	381809	Clec4b2	Aplra1|F83
chr15	76149877	76150025	Intergenic	Intergenic	-7876	NM_17539	109075	Exosc4	1110039I0
chr11	17899427	17899575	Intergenic	Intergenic	-45623	NM_02657	68145	Etaa1	5730466H2
chr3	1.16E+08	1.16E+08	intron (NM	intron (NM	516	NM_01002	13171	Dbt	D3Wsu60e
chr8	98424752	98426450	Intergenic	Intergenic	-13336	NM_01032	14719	Got2	AL022787|
chr2	22750577	22751025	promoter-T	promoter-T	-241	NM_01950	56075	Pdss1	2610203G2
chr1	55272327	55272600	intron (NM	intron (NM	11163	NM_02871	74013	Rftn2	2700010E0
chr4	6119277	6119350	intron (NM	MTC|LTR|I	1061	NM_02653	68053	Ubxn2b	3110003A2
chr9	62191177	62191250	intron (NM	CpG	2063	NM_00967	11737	Anp32a	Anp32|I1P
chr9	1.18E+08	1.18E+08	promoter-T	promoter-T	-24	NM_02644	67899	Cmc1	2010110K1
chr1	1.2E+08	1.2E+08	intron (NM	intron (NM	-60084	NM_02375	81879	Tfcp2l1	1810030F0
chr3	95621902	95622000	intron (NM	intron (NM	925	NM_00108	75137	Rprd2	2810036A1
chr19	37511227	37511500	intron (NM	intron (NM	2032	NM_00824	15242	Hhex	Hex|Hex1|
chr14	37263402	37263675	Intergenic	L1MA8|LIN	355387	NM_17500	218921	4930474N(	-
chr9	58429252	58429375	promoter-T	promoter-T	-734	NM_00129	20320	Nptn	AW554172
chr11	77990352	77990475	promoter-T	promoter-T	-245	NM_08084	140859	Nek8	4632401F2
chr10	5734577	5734750	promoter-T	promoter-T	-168	NM_00795	13982	Esr1	AA420328|
chr14	21871102	21871400	promoter-T	promoter-T	413	NM_01882	55946	Ap3m1	1200013D(
chr14	73562202	73562575	intron (NR_	intron (NR_	19796	NR_03318	105670	Rcbtb2	2610028E0
chr11	97883877	97884150	promoter-T	promoter-T	-72	NM_00128	12295	Cacnb1	CAB1|Cchb
chr12	83348602	83348925	intron (NM	intron (NM	-63573	NM_17257	217692	Sipa1l1	4931426N1
chr4	1.48E+08	1.48E+08	intron (NM	CpG	404	NM_00927	20810	Srm	AA407669|
chr12	71209152	71209275	intron (NM	intron (NM	3403	NR_10439	18080	Nin	3110068G2
chr5	1.24E+08	1.24E+08	intron (NM	intron (NM	1956	NM_02985	77045	Bcl7a	4432415N(
chr11	1.2E+08	1.2E+08	intron (NM	intron (NM	2948	NM_01075	17134	Mafg	AA545192|
chr7	26023902	26024050	Intergenic	Intergenic	-1106	NM_00103	606496	Gsk3a	2700086H(
chr2	1.04E+08	1.04E+08	Intergenic	Intergenic	-10951	NM_00114	16909	Lmo2	Rbtn-2|Rbt
chr4	1.41E+08	1.41E+08	Intergenic	Intergenic	26313	NM_00116	74202	Fblim1	2410043F0
chr4	1.48E+08	1.48E+08	promoter-T	promoter-T	-496	NM_00927	20810	Srm	AA407669|
chr4	1.25E+08	1.25E+08	Intergenic	Intergenic	-14020	NM_14555	230737	Gnl2	BC003262|

chr4	97723177	97723350	intron (NM	intron (NM	278946	NM_01090	18027	Nfia	1110047K1
chr3	52302852	52303025	Intergenic	Intergenic	230679	NM_01973	56458	Foxo1	AI876417|A
chr5	34978902	34979100	intron (NM	intron (NM	762	NM_02666	68294	Mfsd10	0610009O0
chr15	89260827	89260950	promoter-T	promoter-T	-530	NR_03715	12651	Chkb	Chetk|Chkl
chr9	96602402	96602700	intron (NM	intron (NM	29543	NM_17553	245007	Zbtb38	A930014K0
chr1	1.01E+08	1.01E+08	Intergenic	Intergenic	299650	NM_02758	70866	Slco6d1	4921511I0
chr15	99251177	99251250	Intergenic	Intergenic	27563	NM_00117	110213	Tmbim6	5031406P0
chr6	1.24E+08	1.24E+08	promoter-T	promoter-T	-7	NM_00127	19305	Pex5	AW212715
chr9	59336077	59336150	Intergenic	Intergenic	-1932	NM_01992	23806	Arih1	AU021774
chr2	1.65E+08	1.65E+08	intron (NM	intron (NM	4266	NM_08028	140579	Elmo2	1190002F2
chr17	57487152	57487225	Intergenic	RSINE1|SIN	-10921	NM_01013	13733	Emr1	DD7A5-7|E
chr1	39424252	39425900	exon (NM_	exon (NM_	380	NM_00125	114641	Rpl31	-
chr16	4674302	4674550	intron (NM	intron (NM	5294	NM_03020	78885	Coro7	0610011B1
chr7	1.34E+08	1.34E+08	intron (NM	intron (NM	6020	NM_08063	78388	Mvp	2310009M
chr14	70750802	70750875	intron (NM	CpG	393	NM_14480	213053	Slc39a14	FAD-123|Z
chr11	72271777	72272050	intron (NM	intron (NM	16616	NM_00127	216892	Spns2	-
chr1	1.36E+08	1.36E+08	intron (NM	intron (NM	4757	NM_02832	72674	Adipor1	2810031L1
chr14	67486902	67487150	promoter-T	promoter-T	411	NM_00995	12934	Dpysl2	AI851130|
chr11	95120377	95120550	Intergenic	CpG	-2380	NM_05309	93670	Tac4	AW489379
chr2	1.3E+08	1.3E+08	intron (NM	CpG	349	NM_00922	20638	Snrpb	AL024368|
chr7	26954077	26954350	Intergenic	Intergenic	-4215	NM_01000	13094	Cyp2b9	Cyp2b
chr13	94709927	94710025	intron (NM	intron (NM	-117775	NM_17258	218454	Lhfpl2	6030465B1
chr11	80191327	80191775	promoter-T	promoter-T	-34	NM_02747	70591	5730455P1	-
chr18	35758002	35758075	promoter-T	promoter-T	-283	NM_02642	67869	Paip2	2310050K1
chr16	16896227	16896700	promoter-T	promoter-T	-99	NM_17683	68606	Ppm1f	1110021B1
chr3	85376252	85376625	Intergenic	Intergenic	-1613	NM_14489	229487	Pet112	9430026F0
chr11	1.15E+08	1.15E+08	intron (NM	intron (NM	393	NM_00116	246746	Cd300lf	CLIM1|CLM
chr9	1.14E+08	1.14E+08	Intergenic	ORR1B1|LT	-2076	NM_00108	22221	Ubp1	Cp2b|LBP-
chr1	1.56E+08	1.56E+08	TTS (NR_10	TTS (NR_10	204	NM_00813	14645	Glul	GS|Glns
chr2	49665052	49665150	intron (NM	intron (NM	21895	NM_02799	71897	Lypd6b	2310010M
chrX	50264977	50265150	promoter-T	promoter-T	-328	NM_02764	70998	Phf6	2700007B1
chr2	30663602	30663700	intron (NM	CpG	154	NM_17059	66617	Ntmt1	2610205E2
chr3	1.32E+08	1.32E+08	intron (NM	intron (NM	18295	NM_02026	56811	Dkk2	-
chr15	1.02E+08	1.02E+08	Intergenic	Intergenic	-6509	NM_01367	20683	Sp1	1110003E1
chr13	68927827	68928125	intron (NM	ID_B1|SINE	191660	NM_02703	69315	1700001L1	-
chr2	48595252	48595400	Intergenic	Intergenic	-74303	NM_00739	11480	Acvr2a	ActrIIa|Acv
chr6	1.15E+08	1.15E+08	promoter-T	promoter-T	37	NM_00112	19016	Pparg	Nr1c3|PPA
chr16	35982527	35982600	promoter-T	promoter-T	-886	NM_00846	16646	Kpna1	AW494490
chr13	96661327	96661450	intron (NM	intron (NM	489	NM_02771	544963	Iqgap2	4933417J2
chr4	32743802	32743950	promoter-T	promoter-T	-218	NM_00108	100019	Mdn1	4833432B2
chr3	82622377	82622600	Intergenic	Intergenic	57917	NM_00127	633285	Rbm46	EG633285|
chr18	55067702	55068025	intron (NM	intron (NM	81971	NM_17575	269023	Zfp608	4932417D1
chr11	82683752	82684025	intron (NM	intron (NM	358	NM_00116	67338	Rffl	1700051E0
chr8	73159652	73159725	Intergenic	Intergenic	-4154	NM_01181	23886	Gdf15	MIC-1|NAG
chr9	78379652	78380000	Intergenic	Intergenic	-50295	NM_01010	13627	Eef1a1	-
chr7	20108952	20109175	exon (NM_	exon (NM_	4983	NM_02711	69547	Nkpd1	2310015G0
chr11	8728852	8729150	Intergenic	Intergenic	-164463	NM_00108	319939	Tns3	BC023928|

chr4	34833677	34833775	Intergenic	Intergenic	-3529	NM_01388	30046	Zfp292	5730450D(
chr1	1.08E+08	1.08E+08	promoter-1	promoter-1	505	NM_13382	98432	Phlpp1	AI836256|I
chr2	38863602	38863875	promoter-1	promoter-1	79	NM_02880	74192	Arpc5l	2010015J0
chr1	64270027	64270175	Intergenic	Intergenic	-102138	NM_03356	93691	Klf7	9830124P(
chr11	57822902	57823375	intron (NM	CpG	572	NM_02845	73158	Larp1	1810024J1
chr3	1.01E+08	1.01E+08	Intergenic	Intergenic	-2165	NM_00116	229644	Trim45	4921530N(
chr1	66568577	66568850	intron (NM	intron (NM	53692	NM_17551	329178	Unc80	C030018G1
chr5	1.11E+08	1.11E+08	exon (NM_	exon (NM_	462	NM_00946	22241	Ulk1	AU041434
chr16	8671277	8671375	intron (NM	intron (NM	920	NM_02582	52502	Carhsp1	1200011K(
chr19	46692777	46693075	intron (NM	intron (NM	-4965	NM_00117	226178	Wbp1l	D19Wsu16
chr11	4965452	4965525	promoter-1	promoter-1	-158	NM_00119	78926	Gas2l1	4930500E2
chr4	11893052	11893225	promoter-1	promoter-1	-10	NM_00129	381511	Pdp1	Gm1024|P
chr8	73140327	73140500	promoter-1	promoter-1	-330	NM_15307	211228	Lrrc25	Mapa
chr7	30295552	30295650	Intergenic	Intergenic	-5122	NM_00108	74206	Sipa1l3	2610511M
chr3	1.33E+08	1.33E+08	Intergenic	MTD|LTR|I	-20611	NM_00129	74776	Ppa2	1110013G1
chr7	26404527	26404675	promoter-1	promoter-1	89	NM_02550	66349	Atp5sl	2310004L0
chr4	1.36E+08	1.36E+08	intron (NM	CpG	238	NM_01373	27224	Tceb3	110kDa|AA
chr6	52915327	52915625	intron (NM	intron (NM	-55862	NM_00116	231986	Jazf1	AI591476|(
chr1	1.35E+08	1.35E+08	5' UTR (NM	5' UTR (NM	408	NM_13381	108954	Ppp1r15b	1810033K1
chr1	1.67E+08	1.67E+08	promoter-1	promoter-1	-351	NM_17302	271639	Adcy10	4930431D(
chrX	34624127	34624200	intron (NM	intron (NM	1016	NM_02605	67248	Rpl39	2810465O1
chr1	93355552	93355625	intron (NM	CpG	317	NM_01106	18627	Per2	mKIAA0347
chr4	61743827	61744125	TTS (NM_0	TTS (NM_0	4370	NM_00103	17842	Mup3	MUP15|M
chr1	1.53E+08	1.53E+08	promoter-1	promoter-1	623	NM_02687	98685	Trmt1l	1190005F2
chr17	37095527	37095775	promoter-1	promoter-1	114	NM_02960	76416	Znrd1as	1700022C2
chr13	93125052	93125375	promoter-1	promoter-1	-255	NM_01082	17686	Msh3	D13Em1|R
chr10	77517327	77517650	promoter-1	promoter-1	-592	NM_00128	54427	Dnmt3l	D6Ertd14e
chr7	78772452	78772550	Intergenic	ORR1E|LTF	678980	NM_00102	244049	Mctp2	Gm489
chr11	50060552	50060625	Intergenic	B2_Mm2|S	-8615	NM_00852	17001	Ltc4s	-
chr14	98509177	98509500	intron (NM	intron (NM	59646	NM_00103	13134	Dach1	Dac|Dach
chr2	1.03E+08	1.03E+08	intron (NM	L2b|LINE|L	12734	NM_17889	99382	Abtb2	AW539457
chr7	89592827	89593125	intron (NM	intron (NM	108772	NM_00119	269959	Adamtsl3	9230119C1
chr12	25443177	25443400	Intergenic	Intergenic	50169	NM_00910	20135	Rrm2	AA407299|
chr9	77563802	77563900	Intergenic	Intergenic	-38491	NM_01029	14629	Gclc	D9Wsu168
chr11	97274077	97274200	Intergenic	Intergenic	-37336	NM_02149	58996	Arhgap23	A330041B1
chr1	55293902	55294250	promoter-1	promoter-1	55	NM_17543	212679	Mars2	C730026E2
chr11	54599102	54599200	intron (NM	(TC)n|Simp	2054	NM_17862	72729	Cdc42se2	2810404F1
chr4	1.29E+08	1.29E+08	intron (NM	CpG	156	NM_19930	230770	Tmem39b	6330509E(
chr11	55283202	55283475	promoter-1	promoter-1	84	NM_01371	27041	G3bp1	AI849976|I
chr8	1.29E+08	1.29E+08	Intergenic	MTD|LTR|I	-127490	NM_00116	270110	Irf2bp2	E130305N2
chr11	79691752	79691825	Intergenic	Intergenic	84101	NM_14482	216987	Utp6	4732497O(
chr2	49276002	49276175	Intergenic	Intergenic	-30918	NM_17266	227867	Epc2	5830499L1
chr15	59381827	59382025	intron (NM	intron (NM	-98283	NM_14454	211770	Trib1	A530090O1
chr1	1.74E+08	1.74E+08	exon (NM_	exon (NM_	239	NM_02623	67556	Pigm	4933437L0
chr6	1.16E+08	1.16E+08	intron (NM	CpG	254	NM_17741	330401	Tmcc1	3632431M
chr9	72839327	72839525	intron (NM	intron (NM	6115	NM_02818	72278	Ccpg1	1700030B(
chr6	28210777	28210875	5' UTR (NM	5' UTR (NM	775	NM_00108	627049	Zfp800	AA407452|

chr10	86208177	86208400	intron (NM	intron (NM	32778	NM_15359	103220	BC030307	AI449705|A
chr2	91022727	91022900	intron (NM	CpG	391	NM_00117	228355	Madd	9630059K2
chr9	66148627	66148750	Intergenic	ORR1E|LTF	-49569	NM_14561	235439	Herc1	2810449H1
chr18	57614602	57614700	Intergenic	Intergenic	-13489	NM_00113	629147	Ctxn3	ENSMUSG0
chr8	87595777	87595900	exon (NM_	exon (NM_	182	NM_00103	330817	Dhps	Dhs
chr8	1.27E+08	1.27E+08	intron (NM	intron (NM	24928	NM_05320	112405	Egln1	AI503754|(
chr12	88291527	88291750	intron (NM	intron (NM	3345	NM_00128	217732	Cipc	2310044G1
chr6	88028827	88028900	Intergenic	B3|SINE|B	-5604	NM_13393	103963	Rpn1	AU018702
chr18	61918527	61918650	intron (NM	intron (NM	27728	NM_17892	106877	Afap1l1	AI173486
chr14	63741727	63741975	intron (NM	intron (NM	552	NM_00779	13030	Ctsb	CB
chr6	52167527	52167725	5' UTR (NM	5' UTR (NM	946	NM_01045	15404	Hoxa7	AV118143|
chr7	1.34E+08	1.34E+08	Intergenic	Intergenic	-11839	NM_14563	246779	Il27	IL-27|IL-27
chr1	59538152	59538350	promoter-T	promoter-T	-740	NM_00805	14369	Fzd7	Fz7
chr19	34242477	34242700	Intergenic	Intergenic	-2057	NM_02420	52024	Ankrd22	5430429D2
chr5	1.38E+08	1.38E+08	intron (NM	intron (NM	579	NM_02391	78829	Tsc22d4	0610009M
chr8	10277377	10277450	intron (NM	intron (NM	123490	NM_00108	244281	Myo16	C230040D1
chr1	1.53E+08	1.53E+08	intron (NM	CpG	260	NM_00103	117198	Ivns1abp	1190004M
chr16	36367627	36367950	promoter-T	promoter-T	-132	NM_00108	1E+08	BC100530	-
chr11	95316727	95316850	intron (NM	intron (NM	41391	NM_02528	20747	Spop	AI315626|
chr5	1.14E+08	1.14E+08	intron (NM	intron (NM	580	NM_02552	66383	Iscu	2310020H2
chr8	82787477	82787600	Intergenic	Intergenic	-205631	NM_02025	15245	Hhip	Hhip1
chr7	20278702	20279250	promoter-T	promoter-T	-968	NM_00746	11812	Apoc1	Apo-CIB|Ap
chr15	41622302	41622475	intron (NM	MIR3|SINE	1327	NM_13088	170719	Oxr1	2210416C2
chr11	98902777	98902950	exon (NM_	exon (NM_	289	NM_01051	16010	Igfbp4	AI875747|
chr13	41763727	41763900	Intergenic	Intergenic	17682	NM_00114	328231	Gm5082	A030008J0
chr12	84973727	84974025	intron (NM	CpG	692	NM_02734	67039	Rbm25	2600011C0
chr7	25662952	25663100	TTS (NM_0	TTS (NM_0	-6707	NM_02313	20085	Rps19	Dsk3
chr3	19935077	19935225	exon (NM_	exon (NM_	159	NM_00114	12807	Hps3	coa
chr7	1.34E+08	1.34E+08	intron (NR_	intron (NR_	3285	NM_02371	73658	Spns1	2210013K0
chr2	1.68E+08	1.68E+08	Intergenic	Intergenic	-6426	NM_01120	19246	Ptpn1	PTP-1B|PT
chr4	1.29E+08	1.29E+08	intron (NM	intron (NM	444	NM_19930	230770	Tmem39b	6330509E0
chr8	73421652	73421750	promoter-T	promoter-T	-359	NM_02975	76808	Rpl18a	2510019J0
chr8	68866852	68867175	intron (NM	intron (NM	-43187	NM_00116	72925	1-Mar	2900024D2
chr1	1.37E+08	1.37E+08	promoter-T	promoter-T	399	NM_17708	320139	Ptpn7	BPTP-4|C9
chr7	87485302	87485500	promoter-T	promoter-T	-296	NM_13395	101869	Unc45a	AW538196
chr13	37695252	37695450	Intergenic	Intergenic	-222556	NM_02683	68750	Rreb1	1110037N0
chr3	67361127	67361225	Intergenic	Intergenic	-25514	NM_02581	66868	Mfsd1	1200003O0
chr2	90405602	90405700	intron (NM	intron (NM	15153	NM_00898	19271	Ptprj	AI450271|
chr19	40758652	40758825	intron (NM	intron (NM	24454	NM_00984	12495	Entpd1	2610206B0
chr2	1.33E+08	1.33E+08	promoter-T	promoter-T	224	NM_00129	66634	Mcm8	5730432L0
chr5	28117477	28117850	intron (NM	CpG	427	NM_01887	55982	Paxip1	D5Ertd149
chr7	25235452	25235775	Intergenic	Intergenic	-4912	NM_19901	210145	Irgc1	F630044M0
chr1	1.93E+08	1.93E+08	promoter-T	promoter-T	-40	NM_02689	68972	Tatdn3	1500010M
chr8	81344652	81344975	intron (NM	CpG	238	NM_00119	78651	Lsm6	1500031N1
chr4	1.26E+08	1.26E+08	Intergenic	Intergenic	-3449	NM_15317	76850	Ago4	5730550L0
chr2	48666452	48666600	Intergenic	Lx8|LINE|L	-3103	NM_00739	11480	Acvr2a	ActrIIa|Acv
chr1	75165027	75165175	promoter-T	promoter-T	-119	NM_00115	68818	Zfand2b	1110060O1

chr15	37931352	37931525	intron (NM	RLTR16|LT	-40628	NM_19947	382985	Rrm2b	p53R2
chr3	1.44E+08	1.44E+08	exon (NM_	exon (NM_	311	NM_00128	54673	Sh3glb1	AA409932|
chr10	13280252	13280350	intron (NM	intron (NM	-7353	NM_00116	56535	Pex3	1700014F1
chr9	22750202	22750300	Intergenic	Intergenic	-277269	NM_02847	73230	Bmper	3110056H(
chr7	26005127	26005350	promoter-T	promoter-T	-360	NM_20767	67379	Dedd2	2410050E1
chr3	1.52E+08	1.52E+08	promoter-T	promoter-T	-113	NM_17801	329777	Pigk	3000001O(
chr15	98522477	98522650	Intergenic	MIRc|SINE	-14671	NM_17261	223881	Rnd1	A830014L0
chr19	31157127	31157400	promoter-T	promoter-T	-68	NM_03124	83410	Cstf2t	64kDa|C77
chr5	1.4E+08	1.4E+08	intron (NM	intron (NM	11037	NM_02846	73212	3110082I1	AW411904
chr11	76998452	76998550	intron (NM	MER58B|D	3969	NM_17696	319634	Efcab5	4930563A0
chr15	75692602	75692725	promoter-T	promoter-T	-106	NM_02696	69146	Gsdmd	1810036L0
chr1	1.83E+08	1.83E+08	Intergenic	B1_Mur2|S	-6662	NM_17709	320202	Lefty2	6030463A2
chr1	1.57E+08	1.57E+08	intron (NM	CpG	448	NM_01127	19775	Xpr1	Rmc-1|Rm
chr19	23833127	23833275	promoter-T	promoter-T	-165	NM_17703	319924	Apba1	6430513E0
chr16	44725802	44725900	intron (NM	MIR|SINE|	1437	NM_14597	212547	BC027231	Nepro
chr3	88963502	88963775	intron (NM	intron (NM	441	NM_00822	15168	Hcn3	BCNG-4|Bc
chr6	1.01E+08	1.01E+08	intron (NM	ORR1D1|LT	54788	NM_18159	72171	Shq1	2810403P1
chr9	26250677	26250900	Intergenic	Lx5|LINE|L	-308359	NM_02979	76898	B3gat1	0710007K0
chr7	82801127	82801350	intron (NM	intron (NM	-191986	NM_00112	207952	Klhl25	2810402K1
chr8	83538302	83538375	5' UTR (NM	5' UTR (NM	467	NM_02755	74841	Usp38	4631402N1
chr4	1.34E+08	1.34E+08	intron (NM	intron (NM	5056	NM_14555	100017	Ldlrap1	AA691260|
chr6	18204577	18204775	intron (NM	intron (NM	83989	NM_02105	12638	Cftr	AW495489
chr5	1.44E+08	1.44E+08	promoter-T	promoter-T	-410	NM_00739	11461	Actb	Actx|E430(
chr11	16899302	16899400	intron (NM	L1MA4|LIN	9370	NM_01954	56193	Plek	2010300B1
chr14	31430002	31430225	intron (NM	intron (NM	9281	NM_01110	18753	Prkcd	AI385711|
chr4	8748752	8748875	intron (NM	intron (NM	131260	NM_00127	320790	Chd7	A730019I0
chr8	87503627	87503850	Intergenic	Intergenic	-1091	NM_00841	16477	Junb	-
chr16	36041652	36041825	exon (NM_	exon (NM_	462	NM_02711	69544	Wdr5b	2310009C0
chr12	84973052	84973375	promoter-T	promoter-T	29	NM_02734	67039	Rbm25	2600011C0
chr8	13339052	13339150	intron (NM	CpG	297	NM_00936	21781	Tfdp1	Dp1|Drtf1
chr13	1.01E+08	1.01E+08	intron (NM	intron (NM	577	NM_00116	76041	Ccdc125	5830436D(
chr11	21123077	21123250	Intergenic	Intergenic	-15872	NM_13906	245944	Vps54	Hcc8|Vps5
chr18	10052477	10052700	Intergenic	Intergenic	-22441	NM_00103	59025	Usp14	2610005K1
chr6	72849377	72849525	intron (NM	CpG	522	NM_01971	74287	Kcmf1	1700094M
chr8	23816802	23816900	intron (NM	CpG	204	NM_01054	16150	Ikbkb	AI132552|
chr1	59045252	59045400	intron (NM	intron (NM	14911	NM_17265	227154	Stradb	AA792893|
chr8	72736477	72736575	promoter-T	promoter-T	-346	NM_00100	73095	Slc25a42	2900084M
chr17	24551002	24552950	promoter-T	promoter-T	356	NM_00108	19826	Rnps1	-
chr7	1.06E+08	1.06E+08	Intergenic	Intergenic	-24825	NM_00113	14918	Gucy2d	-
chr8	26895602	26895900	promoter-T	promoter-T	-70	NM_02612	67384	Bag4	2410112I1
chr2	25995227	25995800	promoter-T	promoter-T	-450	NM_00125	109299	C330006A1	AI551216
chr7	20525327	20525500	intron (NM	intron (NM	10679	NM_02730	70080	Igsf23	2210010C1
chr18	76401052	76401125	promoter-T	promoter-T	-267	NM_01075	17126	Smad2	7120426M
chr11	83253077	83253450	intron (NM	B1F2|SINE	2067	NM_02549	66330	1700020L2	-
chr9	1.19E+08	1.19E+08	intron (NM	intron (NM	20567	NM_17711	320256	Dlec1	D630005C(
chr8	73046727	73046900	intron (NM	CpG	266	NM_02936	75620	Kxd1	0610030B0
chr5	1.16E+08	1.16E+08	promoter-T	promoter-T	-264	NM_01968	56455	Dynll1	Dlc8|Dnclc

chr3	83788902	83789100	intron (NM	intron (NM	55082	NM_17268	229473	D930015E(	Kiaa0922|r
chr2	1.29E+08	1.29E+08	Intergenic	MIRb|SINE	-4105	NM_01055	16175	Il1a	Il-1a
chr6	1.18E+08	1.18E+08	intron (NM	CpG	216	NM_17768	232337	Zfp637	AI646709|
chr17	35183302	35183425	5' UTR (NM	5' UTR (NM	188	NM_01360	17687	Msh5	G7|Mut5
chr4	94717102	94717675	exon (NM_	exon (NM_	1525	NM_01059	16476	Jun	AP-1|Junc|
chr19	47061752	47061975	intron (NM	intron (NM	-17806	NM_00116	76952	Nt5c2	2010002I2:
chr12	85292002	85292150	exon (NM_	exon (NM_	518	NM_02363	71952	2410016O(	MAPJD|NC
chr9	79607452	79607650	5' UTR (NM	5' UTR (NM	109	NM_00994	12866	Cox7a2	COX7AL|Cc
chr17	15842052	15842350	5' UTR (NM	5' UTR (NM	270	NM_00769	12648	Chd1	4930525N2
chr2	1.64E+08	1.64E+08	Intergenic	Intergenic	-18793	NM_00903	19668	Rbpjl	RBP-L|Rbp
chr2	1.29E+08	1.29E+08	Intergenic	Intergenic	-11513	NM_02824	72477	Tmem87b	2610301K1
chr19	55332252	55332500	intron (NM	intron (NM	4517	NM_02797	433256	Acsl5	1700030F0
chr10	98590527	98590700	intron (NM	L2|LINE|L2	19844	NM_01573	14426	Galnt4	AV011803
chr7	88358627	88359075	exon (NM_	exon (NM_	161	NM_00880	18584	Pde8a	AI551852|
chr10	98704702	98704775	Intergenic	Intergenic	-21127	NM_02626	67603	Dusp6	1300019I0:
chr2	60741627	60741725	intron (NM	intron (NM	-22181	NM_00114	56878	Rbms1	2600014B1
chr17	28152502	28152650	intron (NM	intron (NM	64953	NM_01368	21463	Tcp11	D17Ken1|T
chr11	65601627	65601725	exon (NM_	exon (NM_	123	NM_00915	26398	Map2k4	JNKK1|MEI
chr5	91046977	91047125	intron (NM	intron (NM	22462	NM_02847	73246	Rassf6	1600016B1
chr5	66534902	66535250	intron (NM	intron (NM	7141	NM_00129	245945	Rbm47	9530077J1
chr5	89107902	89108175	Intergenic	Intergenic	-2842	NM_17870	231413	Grsf1	BB232551|
chr1	1.87E+08	1.87E+08	Intergenic	Intergenic	-50435	NM_14551	226778	Mark1	AW491150
chr11	1.18E+08	1.18E+08	intron (NM	CpG	180	NM_13375	74451	Pgs1	2610019F1
chr4	1.4E+08	1.4E+08	Intergenic	Intergenic	-1650	NM_17386	108911	Rcc2	2610510H(
chr5	1.3E+08	1.3E+08	intron (NM	L1M2|LINE	4910	NM_02882	74222	14-Sep	1700016K1
chr8	86117852	86118025	intron (NM	intron (NM	452	NM_02717	106529	Tecr	2410016D2
chr4	1.03E+08	1.03E+08	promoter-T	promoter-T	-149	NM_17773	242585	Slc35d1	AI834976|
chr1	1.52E+08	1.52E+08	Intergenic	Intergenic	-4637	NM_00886	18783	Pla2g4a	Pla2g4|cPL
chr1	9897127	9897525	Intergenic	Intergenic	-1393	NM_17772	240697	Mcmdc2	6030422M
chr5	1.25E+08	1.25E+08	intron (NM	intron (NM	23962	NM_02953	76167	Snrnp35	6330548G2
chr2	11446477	11446550	intron (NM	Lx8|LINE|L	-22796	NM_00117	170768	Pfkfb3	E330010H2
chr1	1.31E+08	1.31E+08	Intergenic	Intergenic	-120662	NM_00991	12767	Cxcr4	CD184|Cm
chr15	76648052	76648350	intron (NM	CpG	399	NM_00116	223666	Arhgap39	9530053N2
chr1	89904752	89904900	intron (NM	CpG	300	NM_00119	227334	Usp40	B230215L0
chr9	21397227	21397425	promoter-T	promoter-T	-51	NM_00120	74766	Yipf2	1300010K(
chr8	34296727	34296925	Intergenic	Intergenic	199173	NM_00112	22427	Wrn	AI846146
chr12	93087302	93087500	exon (NM_	exon (NM_	196	NM_00103	20338	Sel1l	AW493766
chr5	96606177	96606350	Intergenic	Intergenic	-15254	NM_14491	231464	Cnot6l	4932442K2
chr4	1.48E+08	1.48E+08	intron (NM	CpG	145	NM_01978	56273	Pex14	Pex14p|R7
chr5	1.24E+08	1.24E+08	Intergenic	Intergenic	-4239	NM_00104	208043	Setd1b	AA516740|
chr17	45082227	45082300	intron (NM	intron (NM	-64429	NM_02925	75341	4930564C(	-
chr2	1.52E+08	1.52E+08	promoter-T	promoter-T	-42	NM_17509	228775	Trib3	Ifld2|Nipk|
chr11	3075902	3076075	intron (NM	intron (NM	17478	NM_03020	78887	Sfi1	-
chr6	6446352	6447125	Intergenic	Intergenic	81920	NM_00916	20422	Shfm1	DSS1|Shfdg
chr11	83477152	83477300	intron (NM	intron (NM	1140	NM_01365	20303	Ccl4	AT744.1|A
chr2	69554602	69554825	Intergenic	RMER15|LT	-4050	NM_17724	320720	Fastkd1	5330408N(
chr2	1.03E+08	1.03E+08	Intergenic	Intergenic	-42279	NM_00103	12505	Cd44	AU023126

chr1	1.66E+08	1.66E+08	intron (NM	intron (NM	1498	NM_00972	11931	Atp1b1	Atp4b|Atp
chr5	1.44E+08	1.44E+08	intron (NM	CpG	476	NM_17272	231868	E130309D(	A630028N2
chr9	20682877	20683775	intron (NM	intron (NM	828	NM_14515	70726	Angptl6	6330404E1
chr5	73684027	73684325	5' UTR (NM	5' UTR (NM	143	NM_00115	68095	Ociad1	6030432N(
chr8	80263977	80264200	Intergenic	LTR16B2|LT	-15737	NM_01033	13617	Ednra	ET-AR|ETa
chr2	1.19E+08	1.19E+08	Intergenic	Intergenic	5835	NM_02692	69065	Chac1	1810008K(
chr2	92447377	92447700	intron (NM	intron (NM	7674	NM_02385	76969	Chst1	2610008E2
chr6	72494327	72494725	promoter-T	promoter-T	-5	NM_00127	12332	Capg	gCap39|ml
chr8	1.25E+08	1.25E+08	intron (NM	intron (NM	2952	NM_00130	13057	Cyba	b558|nmf3
chr10	79317302	79317500	non-coding	non-coding	224	NM_00128	19205	Ptbp1	AA407203|
chr1	1.46E+08	1.46E+08	intron (NM	intron (NM	776	NM_14599	214498	Cdc73	8430414L1
chr10	62710277	62710600	intron (NM	intron (NM	3893	NM_02610	67345	Herc4	1700056O1
chr5	1.18E+08	1.18E+08	intron (NM	CpG	212	NM_02153	59043	Wsb2	AA673511|
chr16	33937027	33937125	intron (NM	intron (NM	30013	NM_00947	22247	Umps	1700095D2
chr13	13618252	13618400	Intergenic	Intergenic	-64350	NM_01074	17101	Lyst	D13Sfk13|l
chr11	59263752	59263825	promoter-T	promoter-T	241	NM_00120	194952	Jmjd4	6430559I2
chr1	60290752	60290975	intron (NM	L1_Mus2|L	53420	NM_17344	269198	Nbeal1	2310076G1
chr11	69734127	69734350	promoter-T	promoter-T	-106	NM_00116	276770	Eif5a	AA410058|
chr3	68480952	68481125	Intergenic	Intergenic	-13528	NM_00115	16159	Il12a	IL-12p35|Il
chr1	1.57E+08	1.57E+08	Intergenic	Charlie16a	33190	NM_01050	15939	Ier5	-
chr19	7200302	7200600	intron (NM	intron (NM	-20232	NM_20141	396184	Flrt1	AW742165
chr18	7186677	7186900	intron (NM	intron (NM	111111	NM_00108	74934	Armc4	4930463I2
chr6	38842152	38842275	Intergenic	MLT1J2|LT	-16024	NM_00129	15258	Hipk2	1110014O2
chr19	57172352	57172500	intron (NM	intron (NM	21088	NM_00110	226251	Ablim1	2210411C1
chr18	35863052	35863200	promoter-T	promoter-T	-328	NM_02966	76594	Dnajc18	2700075B(
chr6	96627902	96628100	Intergenic	Intergenic	382404	NM_17723	320701	Fam19a4	C130034I1
chr1	58643702	58643825	promoter-T	promoter-T	320	NM_02559	66495	Ndufb3	2700033I1(
chr18	24171802	24171900	3' UTR (NM	3' UTR (NM	7421	NM_02155	59057	Zfp191	3526401F1
chr15	68606902	68607050	Intergenic	MLT1F1|LT	-151874	NM_01015	13992	Khdrbs3	Etle|SLM-2
chr1	52874577	52874700	promoter-T	promoter-T	-106	NM_00838	16329	Inpp1	2300002C(
chr13	64523827	64523900	Intergenic	Intergenic	-9998	NM_05318	105278	Cdk20	4932702G(
chr4	1.09E+08	1.09E+08	Intergenic	Intergenic	-58786	NM_02629	67646	4930522H1	1700007J2
chr4	1.43E+08	1.43E+08	intron (NM	intron (NM	27816	NM_01032	14726	Pdpn	Gp38|OTS-
chr19	5487927	5488125	exon (NM_	exon (NM_	310	NM_02787	71711	Mus81	1200008A1
chr11	1.15E+08	1.15E+08	intron (NM	intron (NM	-8580	NM_00799	14149	Fdxr	-
chr7	1.35E+08	1.35E+08	intron (NM	intron (NM	397	NM_02699	69234	Zfp688	2810407K(
chr8	1.23E+08	1.23E+08	TTS (NM_1	TTS (NM_1	-19076	NM_17885	272551	Gins2	2210013I1
chr17	44846802	44847225	intron (NR_	intron (NR_	26112	NR_07342	12393	Runx2	AML3|Cbf|
chr8	25793677	25793825	intron (NM	intron (NM	-8524	NM_01008	13524	Adam18	Adam27|D
chr6	87452677	87452775	intron (NM	intron (NM	-6405	NM_17547	232201	Arhgap25	A130039I2
chr12	81744077	81744200	intron (NM	intron (NM	710	NM_00795	13877	Erh	Mer|Prei1
chr4	1.3E+08	1.3E+08	intron (NM	intron (NM	2949	NM_00116	19244	Ptp4a2	Prl-2
chr10	58393452	58393750	intron (NM	(CAGAG)n|	117494	NM_17278	237353	Sh3rf3	4831416G1
chr1	1.89E+08	1.89E+08	Intergenic	Intergenic	-36164	NM_02604	67223	Rrp15	2810430M
chr6	53648552	53648725	Intergenic	Intergenic	122181	NM_02581	66873	Tril	1200009O2
chr6	1.38E+08	1.38E+08	intron (NM	intron (NM	82503	NM_17273	232449	Dera	2010002D2
chr15	72398977	72399200	Intergenic	Intergenic	-22379	NM_00103	223604	Kcnk9	Task3

chr12	4775752	4775975	promoter-T	promoter-T	210	NM_17543	207921	Fam228b	A830093I2
chr11	75841277	75841475	promoter-T	promoter-T	-92	NM_02965	76566	Fam101b	1500005K1
chr2	34683427	34683525	Intergenic	Intergenic	-1721	NM_00116	30050	Fbxw2	2700071L0
chr9	1.14E+08	1.14E+08	intron (NR_	CpG	225	NM_00120	1.01E+08	Tmppe	-
chr7	1.4E+08	1.4E+08	Intergenic	Intergenic	-41888	NM_02993	77590	Chst15	4631426J0
chr6	1.03E+08	1.03E+08	intron (NM	intron (NM	23281	NM_00769	12661	Chl1	A530023M
chr3	1.05E+08	1.05E+08	Intergenic	L1MEe|LIN	-88572	NM_00103	56543	Kcnd3	AW045978
chr2	1.19E+08	1.19E+08	Intergenic	Intergenic	-18338	NM_00104	228536	Bahd1	AL022997|
chr2	62568677	62568775	intron (NM	Lx3C|LINE|	66342	NM_14552	227960	Gca	5133401E0
chr2	1.26E+08	1.26E+08	intron (NM	intron (NM	13626	NM_00108	241633	Atp8b4	A530043E1
chr2	1.25E+08	1.25E+08	intron (NM	intron (NM	845	NM_00116	17876	Myef2	9430071B0
chr10	79712427	79712500	intron (NM	intron (NM	266	NM_02927	75406	Ndufs7	1010001M
chr1	1.61E+08	1.61E+08	Intergenic	Intergenic	-9806	NM_01193	26374	Rfwd2	AI316802|
chr12	73862652	73862725	intron (NM	CpG	490	NM_00891	19042	Ppm1a	2310003C2
chr7	1.48E+08	1.48E+08	exon (NM_	exon (NM_	-2765	NM_00125	54123	Irf7	-
chr19	28992402	28992500	Intergenic	Intergenic	-45959	NM_02892	74411	Ppapdc2	4932443D1
chr1	1.66E+08	1.66E+08	intron (NM	intron (NM	-16239	NM_00797	14067	F5	AI173222|
chr14	52560827	52560925	Intergenic	Intergenic	-18428	NM_00101	497071	Rnase13	-
chr9	1.08E+08	1.08E+08	promoter-T	promoter-T	-218	NM_01163	22036	Traip	Trip
chr2	1.81E+08	1.81E+08	promoter-T	promoter-T	21	NM_00117	228998	Arfgap1	AI115377|
chr16	27319352	27319425	intron (NM	intron (NM	11661	NM_19811	239790	Ostn	Ostc
chr11	74538002	74538350	promoter-T	promoter-T	-290	NM_01362	18472	Pafah1b1	LIS-1|Lis1|
chr8	74763952	74764100	promoter-T	promoter-T	-5	NM_00745	11767	Ap1m1	AA408894|
chr5	1E+08	1E+08	promoter-T	promoter-T	-185	NM_01669	50926	Hnrnpdl	AA407431|
chr6	1.25E+08	1.25E+08	promoter-T	promoter-T	70	NM_00125	269800	Zfp384	BB163993|
chr7	29157452	29157525	5' UTR (NM	5' UTR (NM	193	NM_13875	101497	Plekhg2	AI194308|
chr9	22482977	22483275	intron (NM	intron (NM	202967	NM_17841	319845	Bbs9	E130103I1
chr5	1.3E+08	1.3E+08	Intergenic	Intergenic	-30712	NM_00108	243277	Gpr133	E230012M
chr5	1.38E+08	1.38E+08	intron (NM	intron (NM	5568	NM_15351	231805	Pilra	AV021745|
chr4	3802652	3802775	Intergenic	Intergenic	-3461	NM_02002	17451	Mos	c-mos
chr18	77820552	77820650	Intergenic	Tigger16b|	-16726	NM_00116	225743	Rnf165	2900024M
chr2	1.67E+08	1.67E+08	intron (NM	intron (NM	4073	NM_01149	20853	Stau1	5830401L1
chr8	11208677	11208925	intron (NM	URR1A|DN	104025	NM_00993	12826	Col4a1	Bru|Col4a-
chr17	34090427	34090700	intron (NM	intron (NM	2023	NM_01129	20084	Rps18	H-2Ke3|H2
chr2	72307752	72307950	Intergenic	RMER15|L	-6425	NM_02586	66953	Cdca7	2310021G0
chr15	99532727	99532875	promoter-T	promoter-T	83	NM_03184	83797	Smarcd1	AA407987|
chrX	84844452	84844700	intron (NM	intron (NM	516386	NM_00116	331461	Il1rapl1	6330532G1
chr5	1.3E+08	1.3E+08	Intergenic	Intergenic	-1278	NM_02712	69568	Vkorc1l1	2310024K0
chr13	59344027	59344375	Intergenic	MTD|LTR|	301611	NM_00128	67269	Agtpbp1	1700020N1
chr7	3503352	3503700	promoter-T	promoter-T	-93	NM_17736	245126	Tarm1	9930022N0
chr10	94377052	94377150	intron (NM	intron (NM	30111	NM_01879	54712	Plxnc1	2510048K1
chr2	33769927	33770100	Intergenic	B4A|SINE|	-26547	NM_17518	72543	Mvb12b	2610200O1
chr15	74350102	74350225	intron (NM	CpG	3537	NM_17499	107831	Bai1	B830018M
chr1	1.74E+08	1.74E+08	intron (NM	intron (NM	630	NM_15355	98193	Dcaf8	AA408877|
chr12	3427352	3427425	promoter-T	promoter-T	531	NM_17242	75302	Asxl2	4930556B1
chrX	71838652	71838725	promoter-T	promoter-T	-979	NM_14639	258393	Olfr1325	MOR102-1
chr17	56273627	56273725	promoter-T	promoter-T	-401	NM_01366	20359	Sema6b	Sema|Sem

chr15	64172277	64172650	intron (NM	intron (NM	-28228	NM_00127	13196	Asap1	AV239055|
chr6	72898477	72898625	Intergenic	MLT2B3|LT	9675	NM_00103	19240	Tmsb10	Ptmb10|Tb
chr11	75162052	75162125	promoter-T	promoter-T	153	NM_00110	319822	Smyd4	G430029E2
chr5	16084227	16084500	exon (NM_	exon (NM_	24644	NM_00128	15234	Hgf	C230052L0
chr4	21655027	21655300	intron (NM	intron (NM	315	NM_00129	51813	Ccnc	AI451004|A
chr19	14671452	14671525	intron (NM	CpG	985	NM_01160	21888	Tle4	5730411M
chr13	6633302	6633400	intron (NM	intron (NM	14666	NM_01970	56421	Pfkp	1200015H2
chr3	96433827	96434600	promoter-T	promoter-T	362	NM_00110	60365	Rbm8a	2310057C0
chr19	30181952	30182225	intron (NM	intron (NM	67843	NM_13859	104174	Gldc	D030049L1
chr11	59356777	59357200	intron (NM	L1MB2|LIN	800	NM_14582	216799	Nlrp3	AGTAVPRL
chr16	17406177	17406250	5' UTR (NM	5' UTR (NM	120	NM_02334	67474	Snap29	1300018G0
chr12	90529752	90529950	intron (NM	intron (NM	338017	NM_17254	18191	Nrxn3	-
chr11	1.06E+08	1.06E+08	promoter-T	promoter-T	-295	NM_17239	67803	Limd2	0610025L0
chr5	22989702	22989875	intron (NM	B3|SINE|B	49541	NM_02698	69188	Kmt2e	1810033J1
chrX	11584427	11584625	Intergenic	Intergenic	73153	NM_17504	71458	Bcor	5830466J1
chr10	92898202	92898475	Intergenic	Intergenic	-17803	NM_00851	16993	Lta4h	-
chr8	1.27E+08	1.27E+08	promoter-T	promoter-T	149	NM_02685	68865	Arv1	1110067L2
chr3	89397102	89397175	intron (NM	intron (NM	73052	NM_08046	140493	Kcnn3	KCa2.3|SK3
chr4	1.36E+08	1.36E+08	5' UTR (NM	5' UTR (NM	229	NM_17773	242705	E2f2	9230110J1
chr4	1.23E+08	1.23E+08	TTS (NM_0	TTS (NM_0	22993	NM_00891	19063	Ppt1	9530043G0
chr6	83117352	83117500	intron (NM	intron (NM	1508	NM_00119	13191	Dctn1	AL022633|
chr13	56027277	56027400	Intergenic	Intergenic	-94552	NM_01109	18740	Pitx1	Bft|P-OTX|
chr8	34785902	34786100	intron (NM	B3|SINE|B	22291	NM_01034	14782	Gsr	AI325518|
chr18	42421877	42422050	promoter-T	promoter-T	-238	NM_13413	107045	Lars	2310045K2
chr3	65695652	65695750	Intergenic	Intergenic	66446	NM_01993	56706	Ccnl1	2610030E2
chr16	25535927	25536025	Intergenic	Intergenic	-147875	NM_00112	22061	Trp63	AI462811|
chr3	1.16E+08	1.16E+08	intron (NM	intron (NM	25362	NM_00108	229776	Cdc14a	A830059A1
chr6	94442477	94442725	Intergenic	Intergenic	-7707	NM_02625	67582	Slc25a26	4930433D1
chr11	1.17E+08	1.17E+08	3' UTR (NM	3' UTR (NM	67336	NM_17294	268510	Mgat5b	C330018B0
chr1	7696102	7696225	Intergenic	RLTR6_Mm	617162	NM_18302	319263	Pcmtd1	8430411F1
chr15	85163127	85163200	Intergenic	MTC|LTR|I	-3648	NM_01684	54138	Atxn10	AI325283|
chr7	87927252	87927500	intron (NM	intron (NM	20841	NM_01672	29875	Iqgap1	AA682088|
chr8	63462377	63462825	promoter-T	promoter-T	-493	NM_17387	12725	Clcn3	Clc3
chr4	1.18E+08	1.18E+08	intron (NM	intron (NM	10309	NM_02678	68625	Wdr65	1110020C0
chr2	1.7E+08	1.7E+08	intron (NM	intron (NM	-3031	NM_00103	228913	Zfp217	4933431C0
chr15	80332277	80332425	intron (NM	intron (NM	58549	NM_00116	383075	Enthd1	Gm1242|G
chr15	66397327	66397550	Intergenic	Intergenic	-4830	NM_17251	213068	Tmem71	AI661017|
chr13	63916277	63916375	promoter-T	promoter-T	-302	NM_00101	76251	Ercc6l2	0610007P0
chr5	1.14E+08	1.14E+08	exon (NM_	exon (NM_	195	NM_01692	53890	Sart3	AU045857
chr6	67216277	67218150	exon (NM_	exon (NM_	240	NM_02581	66870	Serbp1	1200009K1
chr7	1.3E+08	1.3E+08	intron (NM	intron (NM	170287	NM_00885	18751	Prkcb	A130082F0
chr4	1.51E+08	1.51E+08	promoter-T	promoter-T	127	NM_02872	74035	Nol9	4632412I2
chr4	1.55E+08	1.55E+08	Intergenic	Intergenic	5975	NM_02198	21936	Tnfrsf18	AITR|Gitr
chr12	82990027	82990200	intron (NM	L1MB2|LIN	29096	NM_01881	54604	Pcnx	2900024E2
chr10	79352202	79352375	Intergenic	Intergenic	-1310	NM_01345	11537	Cfd	Adn|DF
chr2	25118177	25118475	exon (NM_	exon (NM_	208	NM_17528	97031	Tprn	C430004E1
chr2	73749227	73749350	promoter-T	promoter-T	63	NM_17501	228033	Atp5g3	6030447M

chr12	36219402	36219675	5' UTR (NM	5' UTR (NM	123	NM_01346	11622	Ahr	Ah|Ahh|Al
chr8	1.22E+08	1.22E+08	intron (NM	intron (NM	8992	NM_02692	69047	Atp2c2	1810010G0
chr3	66949352	66949525	intron (NM	intron (NM	159844	NM_02582	66880	Rsrc1	1200013F2
chr5	1.36E+08	1.36E+08	intron (NM	L1MC4a|LI	13579	NM_00889	18984	Por	4933424M
chr9	50583152	50583375	promoter-T	promoter-T	-67	NM_13398	102580	Alg9	8230402H1
chr13	41535627	41535850	intron (NM	intron (NM	46991	NM_00111	18003	Nedd9	Cas-L|CasL
chr2	51455077	51455225	Intergenic	Intergenic	-27880	NM_19915	387511	Tas2r134	T2R134|Ta
chr1	1.54E+08	1.54E+08	intron (NM	intron (NM	31424	NM_19799	69399	1700025G0	2610510E0
chr11	97660527	97660725	promoter-T	promoter-T	-360	NM_01068	16796	Lasp1	AA408629|
chr2	29627602	29628050	Intergenic	Intergenic	10778	NM_00117	433416	Gm13547	OTTMUSG0
chr6	1.37E+08	1.37E+08	TTS (NM_0	TTS (NM_0	-1137	NM_02698	69187	Erp27	1810033M
chr5	69947927	69948050	promoter-T	promoter-T	-193	NM_17271	231279	Guf1	4631409J1
chr6	17359702	17360000	Intergenic	LTR81A|LT	-54106	NM_00859	17295	Met	AI838057|
chr18	77168502	77168675	promoter-T	promoter-T	-178	NM_02540	66191	Ier3ip1	1110057H1
chr3	1.05E+08	1.05E+08	promoter-T	promoter-T	614	NM_15309	229681	St7l	St7r
chr9	57102977	57103075	intron (NM	intron (NM	7380	NM_02882	74211	1700017B0	AA682102|
chr19	53614502	53614800	intron (NM	intron (NM	10843	NM_00108	240672	Dusp5	Gm337
chr4	1.27E+08	1.27E+08	Intergenic	Intergenic	-149118	NM_01029	14622	Gjb5	Cnx31.1|Cx
chr14	79641027	79641200	intron (NM	intron (NM	6061	NM_00103	105590	Zfp957	AU017455
chr7	19967352	19967500	promoter-T	promoter-T	38	NM_00794	13871	Ercc2	AA407812|
chr16	90239427	90239725	intron (NM	intron (NM	18589	NM_01143	20655	Sod1	B430204E1
chr11	52516877	52517150	Intergenic	Intergenic	-61195	NM_17705	320027	Fstl4	B230374F2
chr1	1.84E+08	1.84E+08	promoter-T	promoter-T	64	NM_00785	13244	Degs1	AA536663|
chr1	1.34E+08	1.34E+08	intron (NM	intron (NM	649	NM_00119	74137	Nuak2	1200013B2
chr2	1.7E+08	1.7E+08	Intergenic	Intergenic	-70226	NM_00115	228913	Zfp217	4933431C0
chr5	1.37E+08	1.37E+08	intron (NM	intron (NM	450	NM_17875	269717	Orai2	A730041O1
chr15	52240377	52240675	Intergenic	Intergenic	113418	NM_17281	239436	Slc30a8	C820002P1
chr15	55388902	55389200	promoter-T	promoter-T	88	NM_00116	105837	Mtbp	AI429604|
chr15	88582552	88582725	intron (NM	CpG	497	NM_18141	223773	Zbed4	1700009F0
chr17	35378102	35379775	promoter-T	promoter-T	247	NM_01969	53817	Ddx39b	0610030D1
chr6	99161777	99162000	intron (NM	intron (NM	54621	NM_00119	108655	Foxp1	3110052D1
chr7	1.35E+08	1.35E+08	exon (NM_	exon (NM_	309	NM_17516	71131	Zfp689	4933416E0
chr14	15178702	15178975	promoter-T	promoter-T	-171	NM_00836	16188	Il3ra	CD123|CDw
chr1	87629327	87629625	intron (NM	Lx8|LINE|L	3705	NM_17705	319997	A630001G2	-
chr6	1.29E+08	1.29E+08	Intergenic	Intergenic	-16480	NM_03059	80782	Klrb1b	Klrb1d|Ly5
chr3	27641227	27641300	Intergenic	Intergenic	-31902	NM_17318	72007	Fndc3b	1600019O0
chr4	46150977	46151175	promoter-T	promoter-T	271	NM_17303	272027	Tstd2	3010020C0
chr17	28819752	28820075	intron (NM	L1MB4|LIN	7019	NM_00129	224661	Slc26a8	-
chr16	45158952	45159025	promoter-T	promoter-T	46	NM_02640	67841	Atg3	2610016C1
chr2	32005827	32007725	5' UTR (NM	5' UTR (NM	108	NM_00115	227723	Prrc2b	5830434P2
chr5	74560677	74561075	Intergenic	Intergenic	-30475	NM_02687	68939	Rasl11b	1190017B1
chr6	1.23E+08	1.23E+08	promoter-T	promoter-T	-212	NM_00116	17474	Clec4d	Clecsf8|Mp
chrY	2789502	2789800	Intergenic	YREP_Mm|	402393	NM_00127	1E+08	Gm3376	Rbmy1b
chr1	39559827	39559925	Intergenic	Intergenic	-24284	NM_01877	54610	Tbc1d8	AD3|HBLP1
chr9	36941052	36941450	intron (NM	intron (NM	13656	NM_14895	208076	Pknox2	D230005H2
chr7	1.24E+08	1.24E+08	promoter-T	promoter-T	-354	NM_01108	18704	Pik3c2a	Cpk-m|PI3
chr8	74999327	74999400	promoter-T	promoter-T	-231	NM_13858	27967	Cherp	5730408I1

chr10	79158602	79158750	TTS (NM_0	TTS (NM_0	6911	NM_00850	16904	Gzmm	Lmet1|MM
chr16	18288927	18289150	intron (NM	CpG	223	NM_03332	94223	Dgcr8	D16H22S1
chr1	75206052	75206225	intron (NM	intron (NM	1215	NM_02901	74577	Glb1l	4833408P1
chr7	51559902	51560125	exon (NM_	exon (NM_	2316	NM_00913	20256	Clec11a	AW457320
chr11	74773127	74773300	intron (NM	intron (NM	33839	NM_00100	103677	Smg6	AI317223|
chr2	1.49E+08	1.49E+08	Intergenic	Intergenic	-70359	NM_02140	58214	Cst10	DD72
chr14	58505427	58505825	intron (NM	intron (NM	3473	NM_02949	75965	Zdhhc20	5033406L1
chr5	1.26E+08	1.26E+08	Intergenic	Intergenic	-16701	NM_01963	22190	Ubc	2700054O
chr17	70860927	70861050	intron (NM	intron (NM	-10462	NM_00112	224997	Dlgap1	4933422O
chr13	52041352	52041750	Intergenic	Intergenic	99507	NM_01181	23882	Gadd45g	AI327420|
chrX	1.49E+08	1.49E+08	5' UTR (NM	5' UTR (NM	131	NM_01366	20591	Kdm5c	D930009K1
chr16	50449302	50449400	Intergenic	B3A|SINE|	-16849	NM_02744	70508	Bbx	5530401J0
chr6	49281952	49282250	intron (NM	RCHARR1|	12749	NM_17509	57895	Ccdc126	6330407D1
chr18	56207152	56207325	Intergenic	Intergenic	-384548	NM_02624	107022	Gramd3	9030613F0
chr11	68781327	68781400	promoter-T	promoter-T	-270	NM_02804	71998	Slc25a35	1810012H1
chr17	51028852	51029175	intron (NM	intron (NM	191007	NM_00129	12228	Btg3	ANA|tob5
chr4	1.02E+08	1.02E+08	Intergenic	Intergenic	34703	NM_00117	18578	Pde4b	Dpde4|R7
chr2	25282252	25282475	TTS (NM_0	TTS (NM_0	-1831	NM_00737	11305	Abca2	AI413825|
chr2	1.51E+08	1.51E+08	promoter-T	promoter-T	-79	NM_02701	69270	Gins1	2810418N
chr5	1.35E+08	1.35E+08	5' UTR (NM	5' UTR (NM	354	NM_00108	14886	Gtf2i	6030441I2
chr12	1.13E+08	1.13E+08	exon (NM_	exon (NM_	610	NM_17737	328162	Trmt61a	6720458F0
chr13	73741727	73741800	exon (NM_	exon (NM_	313	NM_14604	218335	Clptm1l	C130052I1
chr9	15511002	15511425	Intergenic	Intergenic	-3000	NM_17228	234967	Slc36a4	6330573I1
chr10	57251877	57251950	intron (NM	CpG	422	NM_01976	56442	Serinc1	1500011D1
chr5	31504127	31504625	promoter-T	promoter-T	39	NM_00117	101023	Zfp513	AW990386
chr4	59202377	59202625	promoter-T	promoter-T	79	NM_01167	22234	Ugcg	AU043821
chr9	57870927	57871150	intron (NM	intron (NM	8214	NM_01977	13070	Cyp11a1	Cyp11a|Cy
chr13	73605377	73605450	intron (NM	intron (NM	582	NM_14537	210992	Lpcat1	2900035H
chr6	90737227	90737775	intron (NM	ID4|SINE|	22616	NM_00113	232227	Iqsec1	AW561907
chr1	1.81E+08	1.81E+08	promoter-T	promoter-T	160	NM_00824	15278	Tfb2m	Hkp1
chr10	81008252	81008325	promoter-T	promoter-T	-497	NM_01030	14672	Gna11	Dsk7|E430
chr4	1.45E+08	1.45E+08	Intergenic	Intergenic	-5168	NM_00127	230895	Vps13d	-
chr1	1.73E+08	1.73E+08	Intergenic	L1MD3|LIN	45413	NM_17706	320078	Olfml2b	1110018N
chr11	59918802	59919025	intron (NM	CpG	398	NM_00902	19377	Rai1	Gt1
chr1	60706377	60706575	Intergenic	MIRc|SINE	-82867	NM_00104	77300	Raph1	9430025M
chr9	1.01E+08	1.01E+08	intron (NM	CpG	624	NM_00116	235542	Ppp2r3a	3222402P1
chr4	1.27E+08	1.27E+08	exon (NM_	exon (NM_	-7527	NM_03025	80284	Smim12	BC003266
chr7	36841552	36841675	Intergenic	B3A|SINE|	-253605	NM_17773	668501	Zfp507	1810022O
chr4	1.26E+08	1.26E+08	promoter-T	promoter-T	-40	NR_04556	12986	Csf3r	Cd114|Csf
chr19	23761727	23761925	promoter-T	promoter-T	-64	NM_02820	72351	Ptar1	1700084D
chr10	43988177	43988250	promoter-T	promoter-T	49	NM_05306	11793	Atg5	2010107M
chr17	47891202	47891350	intron (NM	intron (NM	16396	NM_00116	21425	Tfeb	Tcfeb|bHL
chr17	32487302	32487425	intron (NM	intron (NM	159	NM_01747	54194	Akap8l	HAP95|Na
chr14	70195277	70195425	intron (NM	intron (NM	10001	NM_15351	246710	Rhobtb2	Dbc2|E130
chr17	29396002	29396200	intron (NM	intron (NM	4815	NM_02684	68816	Ppil1	1110060O
chr13	40124252	40124575	intron (NM	intron (NM	258967	NM_17214	218165	Ofcc1	opo
chr9	88243427	88243650	intron (NM	intron (NM	21091	NM_01185	23959	Nt5e	2210401F0

chr1	34516477	34516575	promoter-T	promoter-T	-65	NM_01120	19253	Ptpn18	FLP1|HSCF
chr16	10977402	10977550	intron (NM	ID_B1|SINE	15738	NM_01998	56722	Litaf	3222402J1
chr1	74350777	74351050	promoter-T	promoter-T	-3	NM_02715	69660	Tmbim1	2310061B0
chr17	50650302	50650425	intron (NM	intron (NM	1491	NM_01388	224860	Plcl2	PLC-L2|PRI
chr5	92514902	92515075	Intergenic	Intergenic	-2227	NM_00108	23881	G3bp2	AA409541|
chr16	24238502	24238700	Intergenic	Intergenic	-154835	NM_17866	210126	Lpp	9430020K1
chr9	72974077	72974250	Intergenic	Intergenic	12887	NM_19860	225215	Rsl24d1	2410159K2
chr4	1.09E+08	1.09E+08	promoter-T	promoter-T	-822	NM_14615	230598	Nrd1	2600011I0
chr1	1.84E+08	1.84E+08	Intergenic	Intergenic	-11812	NM_00114	170725	Capn8	nCL-2|nCL-
chr2	1.26E+08	1.26E+08	intron (NM	intron (NM	9118	NM_17760	70354	Secisbp2l	3110001I2
chr5	1.03E+08	1.03E+08	5' UTR (NM	5' UTR (NM	128	NM_02927	231532	Arhgap24	0610025G2
chr6	1.29E+08	1.29E+08	Intergenic	Intergenic	-17230	NM_03059	80782	Klrb1b	Klrb1d|Ly5
chr10	1.11E+08	1.11E+08	promoter-T	promoter-T	100	NM_17861	52705	Krr1	2610511F0
chr1	72258552	72258875	5' UTR (NM	5' UTR (NM	168	NM_00100	381269	Mreg	Gm974|W
chr1	88445552	88445750	intron (NM	RSINE1|SIN	22340	NM_00897	19231	Ptma	Thym
chr11	94068427	94068525	Intergenic	Intergenic	-4292	NM_00942	22057	Tob1	Tob|Trob
chr1	1.36E+08	1.36E+08	Intergenic	Intergenic	-47739	NM_21361	381290	Atp2b4	Pmca4
chr3	1.1E+08	1.1E+08	intron (NM	intron (NM	200389	NM_00116	80883	Ntng1	A930010C0
chr7	16986552	16986750	exon (NM_	exon (NM_	106	NM_19863	330474	Zc3h4	Bwq1|Gm7
chr15	98699452	98699550	intron (NM	intron (NM	2135	NM_00103	381022	Kmt2d	ALR|BC032
chr13	67370102	67370250	promoter-T	promoter-T	-172	NM_00100	238690	Zfp458	BC062958|
chr12	13255702	13256050	intron (NM	CpG	104	NM_13404	104721	Ddx1	AA409185|
chr1	18781527	18781825	Intergenic	Intergenic	-311427	NM_15315	226896	Tfap2d	Tcfap2d
chr14	66844027	66844100	intron (NM	intron (NM	-11674	NM_00116	19229	Ptk2b	CADTK|CAI
chr13	65342277	65342525	Intergenic	CpG	-35377	NM_17529	97895	Nlrp4f	C330026N0
chr3	1.08E+08	1.08E+08	intron (NM	intron (NM	14656	NM_01997	20661	Sort1	2900053A1
chr2	75543177	75543525	promoter-T	promoter-T	-653	NM_01090	18024	Nfe2l2	AI194320|
chr19	36339752	36340150	Intergenic	MIRb|SINE	-113606	NM_02950	76073	Pcgf5	0610009F0
chr4	1.06E+08	1.06E+08	intron (NM	intron (NM	767	NM_15356	100102	Pcsk9	AI415265|
chr16	57607227	57607475	promoter-T	promoter-T	-371	NM_02559	66497	Cmss1	1110001A0
chr3	51365502	51365575	promoter-T	promoter-T	-793	NM_08079	73251	Setd7	1600028F2
chr10	92574052	92574400	Intergenic	MER94|DN	-49395	NM_14623	237459	Cdk17	6430598J1
chr4	3739702	3740050	Intergenic	RLTR45|LT	22871	NM_02614	67427	Rps20	4632426K0
chr8	1.05E+08	1.05E+08	Intergenic	Intergenic	270498	NM_00986	12552	Cdh11	Cad11
chr11	23976802	23977000	Intergenic	CpG	-1155	NM_01670	14025	Bcl11a	2810047E1
chr9	71508577	71508775	intron (NM	intron (NM	-68509	NM_00103	102371	Myzap	AA407270|
chr9	96805502	96805675	Intergenic	Intergenic	-15695	NM_00128	235534	Pxylp1	9430094M
chr15	5296577	5296700	Intergenic	Intergenic	-102451	NM_00896	19219	Ptger4	EP4|Ptgere
chr19	9016027	9016200	promoter-T	promoter-T	-297	NM_01184	23942	Mta2	AW550797
chr14	35122852	35122925	intron (NM	intron (NM	801	NM_02938	75698	Fam35a	3110001K2
chr10	60149827	60150025	intron (NR_	CpG-1077	9312	NM_00125	22295	Cdh23	4930542A0
chr2	84877502	84877800	promoter-T	promoter-T	43	NM_00113	20833	Ssrp1	C81323|Hr
chr1	37267177	37267300	Intergenic	LTR41B|LT	-7885	NM_00128	12790	Cnga3	CNG3
chr7	29225927	29226500	intron (NM	intron (NM	257	NM_00103	63986	Gmfg	0610039G1
chr2	3388602	3388825	intron (NR_	intron (NR_	3545	NM_02272	64707	Suv39h2	4930507K2
chr1	1.07E+08	1.07E+08	intron (NM	CpG	586	NM_17877	320311	Rnf152	A930029B0
chr1	1.33E+08	1.33E+08	Intergenic	Intergenic	-22196	NM_01054	16153	Il10	CSIF|Il-10

chr3	90472777	90473050	promoter-T	promoter-T	-80	NM_01365	20201	S100a8	60B8Ag|AI
chr4	1.34E+08	1.34E+08	5' UTR (NM	5' UTR (NM	342	NM_20723	230815	Man1c1	AI593348
chr16	23855402	23855700	Intergenic	Intergenic	35379	NM_00921	20604	Sst	SOM|SRIF|
chr13	91062627	91062875	promoter-T	promoter-T	24	NM_02417	66475	Rps23	2410044J1
chr12	1.12E+08	1.12E+08	promoter-T	promoter-T	-483	NM_02714	69641	Wdr20	2310040A1
chr18	65257252	65257375	intron (NM	intron (NM	74132	NM_03188	83814	Nedd4l	1300012C0
chr1	1.35E+08	1.35E+08	intron (NM	CpG	524	NM_00109	240752	Pik3c2b	C330011J1
chr3	1.08E+08	1.08E+08	intron (NM	intron (NM	2031	NM_00128	109674	Ampd2	1200014F0
chr1	1.52E+08	1.52E+08	promoter-T	promoter-T	-5	NM_13378	108989	Tpr	2610029M
chr1	1.46E+08	1.46E+08	Intergenic	Intergenic	-2559	NM_00906	19735	Rgs2	GOS8
chr2	34726552	34727050	promoter-T	promoter-T	-319	NM_08055	66998	Psmd5	1500032A0
chr7	1.5E+08	1.5E+08	5' UTR (NM	5' UTR (NM	158	NM_13365	12520	Cd81	Tapa-1|Tap
chr2	84510402	84510575	TTS (NM_0	TTS (NM_0	377	NM_00103	72657	2700094K1	Selh
chr8	26825752	26826150	3' UTR (NM	3' UTR (NM	-4569	NR_121642	71910	Ppapdc1b	1810019D0
chr9	21043352	21043425	5' UTR (NM	5' UTR (NM	388	NM_01667	50868	Keap1	INRF2|mKI
chr9	1.24E+08	1.24E+08	Intergenic	Intergenic	17487	NM_00991	12768	Ccr1	Cmkbr1|M
chr3	97571177	97571275	exon (NM_	exon (NM_	711	NR_11036(	83679	Pde4dip	4732458A0
chr2	79920327	79920975	intron (NM	intron (NM	48477	NM_01674	18573	Pde1a	AI987702|/
chr5	32441277	32441400	intron (NM	intron (NM	2493	NM_00803	14284	Fosl2	Fra-2
chr11	44203677	44203850	Intergenic	Intergenic	-9802	NM_00835	16160	Il12b	Il-12b|Il-12
chr19	42251427	42251650	intron (NM	intron (NM	6481	NM_00116	319740	Zfyve27	2210011N0
chr1	74634302	74634475	promoter-T	promoter-T	214	NM_02988	77264	Zfp142	9330177B1
chr10	1.17E+08	1.17E+08	intron (NM	CpG	335	NM_13401	103468	Nup107	AW541137
chr7	73253827	73253975	promoter-T	promoter-T	-500	NM_00108	269941	Chsy1	mKIAA099(
chr1	40572127	40572200	promoter-T	promoter-T	-44	NM_01055	16174	Il18rap	AcPL|IL-18
chr18	39829702	39829975	Intergenic	Intergenic	-103313	NM_01103	18459	Pabpc2	Pabp|Pabp
chr6	88415177	88415275	promoter-T	promoter-T	-191	NM_01968	56505	Ruvbl1	2510009G0
chr2	1.2E+08	1.2E+08	exon (NM_	exon (NM_	353	NM_01194	26390	Mapkbp1	2810483F2
chr7	1.49E+08	1.49E+08	Intergenic	Intergenic	-10258	NM_00119	1E+08	Gm4559	-
chr12	80399027	80399100	intron (NM	intron (NM	725	NM_00125	19363	Rad51b	AI553500|
chr7	1.49E+08	1.49E+08	promoter-T	promoter-T	161	NM_01687	54006	Deaf1	AU042387
chr12	66923252	66923625	Intergenic	L1MC2|LIN	461950	NM_00116	238217	Rpl10l	EG238217
chr5	1.49E+08	1.49E+08	intron (NM	CpG	392	NM_00751	11987	Slc7a1	4831426K0
chr7	38891777	38891850	exon (NM_	exon (NM_	696	NM_00763	12447	Ccne1	AW538188
chr5	96638052	96638175	Intergenic	Intergenic	-1021	NM_00103	94061	Mrpl1	2410002L0
chr9	85220102	85220275	exon (NM_	exon (NM_	543	NM_00116	212943	Fam46a	D930050G(
chr12	36710077	36710275	Intergenic	Intergenic	-9336	NM_01178	23795	Agr2	Agr2h|Gob
chr8	49195527	49195675	non-coding	non-coding	234	NM_00116	320685	Dctd	6030466N0
chr1	1.68E+08	1.68E+08	intron (NM	B1_Mus2|S	4074	NM_17859	226594	Rcsd1	A430105K1
chr4	1.47E+08	1.47E+08	exon (NM_	exon (NM_	147	NM_02984	77034	2510039O1	Kiaa2013
chr6	86634252	86634375	intron (NM	intron (NM	172	NM_02566	66618	Snrnp27	2610209M
chr19	56623102	56623250	promoter-T	promoter-T	425	NM_02581	66866	Nhlrc2	1200003G0
chr17	35272177	35272275	promoter-T	promoter-T	103	NM_05717	224727	Bag6	2410045D2
chr10	1.07E+08	1.07E+08	Intergenic	Intergenic	-115536	NM_00117	628870	Otogl	EG628870|
chr2	78900602	78900700	Intergenic	Intergenic	191447	NM_00945	22193	Ube2e3	Ubce4|ubc
chr3	1.16E+08	1.16E+08	intron (NM	(TAGA)n|Si	75774	NM_00108	229776	Cdc14a	A830059A1
chr4	1.32E+08	1.32E+08	intron (NM	intron (NM	1291	NM_00103	230787	Themis2	ICB-1

chr6	1.18E+08	1.18E+08	Intergenic	Tigger6a|D	17568	NM_00100	22685	Zfp239	Mok-2|Mo
chr7	1.09E+08	1.09E+08	promoter-T	promoter-T	31	NM_00129	75430	Anapc15	3200002M
chr2	1.26E+08	1.26E+08	intron (NM	intron (NM	24676	NM_00108	241633	Atp8b4	A530043E1
chr5	48763652	48763775	promoter-T	promoter-T	82	NM_02575	66768	Pacrgl	4933428G(
chr8	72426277	72426350	exon (NM_	exon (NM_	144	NM_02331	67184	Ndufa13	2700054G1
chr13	1.13E+08	1.13E+08	Intergenic	Intergenic	-29783	NM_00116	77318	Ankrd55	C030011J0
chr1	1.83E+08	1.83E+08	Intergenic	Intergenic	-40371	NM_02840	72978	Cnih3	2900075G(
chr8	97561577	97561650	intron (NM	(TG)n|Simp	-17390	NM_00104	330830	Ccdc135	Gm770|SR
chr7	1.34E+08	1.34E+08	Intergenic	Intergenic	3497	NM_00926	20768	Sephs2	Sps2|Ysg3
chr17	34325027	34325200	promoter-T	promoter-T	612	NM_01368	21354	Tap1	ABC17|APT
chr3	51144777	51144950	promoter-T	promoter-T	-297	NM_02350	69257	Elf2	2610036A2
chr18	68092777	68092900	promoter-T	promoter-T	-73	NM_17263	52662	Ldlrad4	8230401C2
chr3	1.27E+08	1.27E+08	5' UTR (NM	5' UTR (NM	113	NM_00971	11924	Neurog2	Atoh4|Mat
chr12	81536027	81536425	intron (NM	CpG	1362	NM_17726	320808	Dcaf5	9430020B(
chr1	45981277	45982125	intron (NM	intron (NM	738	NM_01691	53945	Slc40a1	Dusg|Fpn1
chr7	1.28E+08	1.28E+08	promoter-T	promoter-T	-226	NM_00126	13631	Eef2k	C86191|eE
chr8	1.23E+08	1.23E+08	intron (NM	intron (NM	52606	NM_03020	78892	Crispld2	1810049K2
chr1	1.95E+08	1.95E+08	intron (NM	intron (NM	592	NM_18104	319266	A130010J1	-
chr5	1.24E+08	1.24E+08	intron (NM	intron (NM	217	NM_02660	68184	Denr	1500003K(
chr8	37185477	37185650	intron (NM	intron (NM	27681	NM_17291	244418	D8Ertd82e	9830148H2
chr4	1.36E+08	1.36E+08	intron (NM	CpG	335	NM_02591	67025	Rpl11	2010203J1
chr16	18248452	18248700	promoter-T	promoter-T	211	NM_01123	19385	Ranbp1	Htf9a
chr19	54232527	54232600	Intergenic	Intergenic	112891	NM_00741	11551	Adra2a	AW122659
chr5	1.25E+08	1.25E+08	promoter-T	promoter-T	-209	NM_02567	66627	Ogfod2	1300006G1
chr8	96703627	96703775	intron (NM	intron (NM	712	NM_01360	17748	Mt1	MT-I|Mt-1
chr4	58253377	58253500	Intergenic	Intergenic	-33970	NM_02281	64817	Svep1	1110021D1
chr2	1.79E+08	1.79E+08	intron (NM	RLTR45|LT	172318	NM_00986	12561	Cdh4	AW120700
chr14	65988452	65988650	intron (NM	intron (NM	11038	NM_19902	380912	Zfp395	BC053701|
chr1	1.77E+08	1.77E+08	Intergenic	L1MB8|LIN	-16337	NM_00119	24012	Rgs7	-
chr14	51490077	51490225	promoter-T	promoter-T	78	NM_00935	21745	Tep1	Tp1
chr11	50103777	50103925	intron (NM	intron (NM	1987	NM_17533	103806	Maml1	AI644666|
chr3	1.33E+08	1.33E+08	Intergenic	Intergenic	-21373	NM_02995	77669	Arhgef38	9130221D2
chr7	73532852	73533150	intron (NM	CpG	226	NM_14619	233328	Lrrk1	AW319595
chr14	27456927	27457000	promoter-T	promoter-T	-720	NM_00747	11843	Arf4	AA407803
chr3	95908527	95908700	5' UTR (NM	5' UTR (NM	163	NM_00102	229603	Otud7b	2900060B2
chr2	83484777	83485025	intron (NM	CpG	166	NM_02693	69082	Zc3h15	1700006A1
chr1	82811202	82811450	Intergenic	Intergenic	-24732	NM_01047	15463	Agfg1	AU045498
chr7	1.13E+08	1.13E+08	promoter-T	promoter-T	-13	NM_01056	16202	Ilk	AA511515|
chr4	53843677	53843850	intron (NM	intron (NM	4846	NM_02805	52076	Tmem38b	1600017F2
chr17	28147552	28147650	intron (NM	intron (NM	69928	NM_01368	21463	Tcp11	D17Ken1|T
chr13	51494252	51494400	Intergenic	Intergenic	-9661	NM_01010	13610	S1pr3	AI132464|
chr1	65159077	65159225	intron (NM	intron (NM	-9214	NM_00777	12964	Cryga	Cryg-4|DG
chr5	36243927	36244025	intron (NM	intron (NM	8008	NM_02737	70292	Afap1	2600003E2
chr5	1.22E+08	1.22E+08	intron (NM	intron (NM	2372	NM_17272	231713	Naa25	4833422K1
chr2	75630927	75631050	Intergenic	Intergenic	-39246	NM_17266	228061	Agps	5832437L2
chr5	1.19E+08	1.19E+08	intron (NM	intron (NM	55023	NM_17242	76199	Med13l	2210413I1
chr11	69659677	69659750	promoter-T	promoter-T	-161	NM_00116	70310	Plscr3	2210403O2

chr10	1.28E+08	1.28E+08	Intergenic	B1_Mm|SI	-3120	NM_00108	210582	Coq10a	Gm1
chr8	96590802	96591150	TTS (NM_0	TTS (NM_0	29878	NM_00109	270086	Ogfod1	4930415J2
chr4	53425002	53425150	Intergenic	MTD|LTR|	-28209	NM_00115	100434	Slc44a1	2210409B2
chr9	1.08E+08	1.08E+08	promoter-T	promoter-T	-237	NM_00127	13138	Dag1	D9Wsu13e
chr4	54141852	54142100	Intergenic	Intergenic	303059	NM_02805	52076	Tmem38b	1600017F2
chr13	37213477	37213750	Intergenic	Intergenic	-71500	NM_00116	74145	F13a1	1200014I0
chr7	1.49E+08	1.49E+08	intron (NM	L1MC4a|LI	2117	NM_01152	21351	Taldo1	-
chr2	66041077	66041250	intron (NM	L1MB3|LIN	53511	NM_00104	73668	Ttc21b	2410066K1
chr5	1.22E+08	1.22E+08	promoter-T	promoter-T	-355	NM_00965	11669	Aldh2	Ahd-5|Ahd
chr1	51624752	51625025	Intergenic	Intergenic	-89645	NM_02869	109019	Nabp1	4930434H0
chr11	1.1E+08	1.1E+08	intron (NM	intron (NM	20554	NM_14722	217262	Abca9	D630040K0
chr11	70460152	70460425	promoter-T	promoter-T	253	NM_02421	67863	Slc25a11	2310022P1
chr5	1.16E+08	1.16E+08	intron (NM	intron (NM	21099	NM_03186	19079	Prkab1	1300015D2
chr16	32102402	32102575	intron (NM	intron (NM	2600	NM_02589	66994	Cep19	1500031L0
chr6	1.25E+08	1.25E+08	intron (NM	intron (NM	562	NM_13874	110109	Nop2	120kDa|A5
chr2	44776202	44776375	Intergenic	Intergenic	-59146	NM_17266	227835	Gtdc1	E330008O2
chr12	56920052	56920325	intron (NM	intron (NM	2315	NM_01999	56784	Ralgapa1	2310003F2
chr5	23072302	23072475	intron (NM	intron (NM	50001	NM_00927	20817	Srpk2	AW226533
chr13	53076952	53077150	promoter-T	promoter-T	-643	NM_01737	18030	Nfil3	AV225605|
chr7	1.52E+08	1.52E+08	intron (NM	intron (NM	23614	NM_00780	13043	Cttn	1110020L0
chr16	23988627	23988725	promoter-T	promoter-T	22	NM_00974	12053	Bcl6	Bcl5
chr1	1.64E+08	1.64E+08	Intergenic	Intergenic	-42021	NM_17264	226551	Suco	4732491B1
chr15	99330752	99330850	intron (NM	intron (NM	-25640	NM_02923	75284	Bcdin3d	4930556P0
chr15	84753077	84753250	promoter-T	promoter-T	-695	NM_01671	18141	Nup50	1700030K0
chr14	73109752	73109975	promoter-T	promoter-T	-53	NM_20763	319448	Fndc3a	1700094E1
chr13	1E+08	1E+08	promoter-T	promoter-T	320	NM_02687	68927	Ptcd2	1190005P0
chr2	18508902	18509075	Intergenic	Intergenic	-85101	NM_14777	12238	Commd3	AW550818
chr15	78078027	78078100	intron (NM	MIR|SINE|	2822	NM_00867	17972	Ncf4	AI451400|
chr13	51806152	51806500	intron (NM	intron (NM	59243	NM_02927	75420	Secisbp2	2210413N0
chr11	1.17E+08	1.17E+08	intron (NM	intron (NM	1936	NM_00111	53860	9-Sep	MSF1|Msf
chr5	1.49E+08	1.49E+08	intron (NM	L4|LINE|RT	23363	NM_01022	14254	Flt1	AI323757|
chr11	1.15E+08	1.15E+08	intron (NM	MIRb|SINE	3124	NM_19922	217304	Cd300lb	CLM-7|Clm
chr6	1.15E+08	1.15E+08	Intergenic	L1MB7|LIN	-24296	NM_19903	381802	Tsen2	AU067695
chrX	71157502	71157650	promoter-T	promoter-T	-939	NM_00116	171207	Arhgap4	A130009C1
chr4	98787577	98787650	promoter-T	promoter-T	-7	NM_02608	67299	Dock7	3110056M
chrX	10615952	10616125	Intergenic	MIR|SINE|	-260345	NM_00111	1E+08	Gm14483	OTTMUSG0
chr16	78296477	78296625	Intergenic	Intergenic	-5365	NM_00998	13052	Cxadr	2610206D0
chr2	91489952	91490150	promoter-T	promoter-T	-103	NM_00103	381410	Zfp408	Gm1011
chr4	1.16E+08	1.16E+08	Intergenic	Intergenic	-3063	NM_14625	242642	Hpdl	A830048M
chr16	23103902	23104050	Intergenic	Intergenic	-3565	NM_00112	13682	Eif4a2	4833432N0
chr7	1.22E+08	1.22E+08	promoter-T	promoter-T	208	NM_01105	18576	Pde3b	9830102A0
chr1	21951702	21951775	5' UTR (NM	5' UTR (NM	285	NM_00116	226922	Kcnq5	7730402H1
chr2	1.44E+08	1.44E+08	intron (NM	intron (NM	4111	NM_00128	74528	Mgme1	8430406I0
chr9	15648477	15648900	Intergenic	ETnERV-int	30312	NM_14571	244701	Mtnr1b	Mel-1B-R|
chr18	38578702	38578900	exon (NM_	exon (NM_	172	NM_02299	65113	Ndfip1	0610010M
chr14	61479077	61479150	intron (NM	intron (NM	75710	NR_040642	70478	Mipep	5730405E0
chr6	54884027	54884375	intron (NM	intron (NM	37454	NM_00117	107607	Nod1	C230079P1

chr3	1.52E+08	1.52E+08	Intergenic	Intergenic	-8884	NM_00896	19220	Ptgfr	AI957154|I
chr2	1.03E+08	1.03E+08	intron (NM	intron (NM	39959	NM_17889	99382	Abtb2	AW539457
chr1	99557577	99557875	intron (NM	CpG	869	NM_13382	52392	D1Ertd622	AI987691|/
chr8	1.18E+08	1.18E+08	Intergenic	Intergenic	283606	NM_00102	17132	Maf	2810401A2
chr3	1.03E+08	1.03E+08	Intergenic	Intergenic	100197	NM_00107	94093	Trim33	8030451N0
chr9	78299002	78299175	intron (NM	intron (NM	3071	NM_02665	68291	Mto1	2310039H0
chr10	1.18E+08	1.18E+08	promoter-T	promoter-T	-742	NM_00101	69181	Dyrk2	1810038L1
chr17	3115452	3115725	intron (NM	CpG	616	NM_13412	106583	Scaf8	A630086M
chr11	1.21E+08	1.21E+08	promoter-T	promoter-T	-77	NM_02774	71276	Ccdc57	4933434G0
chr15	76528127	76528325	intron (NM	intron (NM	1032	NM_18280	76282	Gpt	1300007J0
chr6	29297552	29297825	promoter-T	promoter-T	-418	NM_00759	12321	Calu	9530075H2
chr6	99195227	99195350	intron (NM	intron (NM	21221	NM_00119	108655	Foxp1	3110052D1
chr19	45734152	45734325	intron (NM	CpG	445	NM_01390	30838	Fbxw4	Dac|Fbw4|
chr12	86940302	86940375	promoter-T	promoter-T	-28	NM_03088	81703	Jdp2	Jundm2|Ju
chr7	51654552	51654900	intron (NM	GA-rich|Lo	15230	NM_01666	20981	Syt3	AI385753|s
chr3	54828427	54828850	Intergenic	Intergenic	30339	NM_00762	12427	Ccna1	-
chr19	44040177	44040300	intron (NM	intron (NM	20772	NM_03070	93721	Cpn1	0610011F2
chr14	76510652	76511175	promoter-T	promoter-T	215	NM_01374	27275	Nufip1	Nufip
chr11	71064952	71065050	intron (NR_	RMER21A|	-20766	NM_00104	637515	Nlrp1b	ENSMUSG0
chr14	68669477	68669625	Intergenic	Intergenic	-32390	NM_01091	18039	Nefl	AI847934|(
chr11	9600452	9600525	Intergenic	Intergenic	508543	NM_17825	268379	Abca13	9830132L2
chr7	25687527	25687650	promoter-T	promoter-T	-417	NM_00113	16801	Arhgef1	Lbcl2|Lsc
chr3	1.22E+08	1.22E+08	promoter-T	promoter-T	-424	NM_00812	14630	Gclm	AI649393|(
chr15	76338702	76338950	intron (NM	intron (NM	3422	NM_01004	13350	Dgat1	ARAT|C759
chr11	96777452	96777650	intron (NM	CpG-2511	244	NM_03024	80280	Cdk5rap3	1810007E2
chr6	1.19E+08	1.19E+08	exon (NM_	exon (NM_	602	NM_13394	101358	Fbxl14	AW322056
chr12	55302377	55302450	intron (NM	intron (NM	2448	NM_02813	112407	Egln3	2610021G0
chr13	76080702	76080875	exon (NM_	exon (NM_	484	NM_02849	73296	Rhobtb3	1700040C1
chr19	5295402	5295525	promoter-T	promoter-T	-8	NM_03010	319322	Sf3b2	145kDa|26
chr12	82096627	82096725	Intergenic	B3|SINE|B.	-27610	NM_00117	20493	Slc10a1	Ntcp
chr2	24774827	24774975	intron (NM	CpG	228	NM_17254	77683	Ehmt1	9230102N1
chr4	45994027	45994175	intron (NM	intron (NM	8932	NM_14614	100121	Tdrd7	5730495N1
chr4	1.33E+08	1.33E+08	intron (NM	CpG	342	NM_14615	230793	Ahdc1	D030015G1
chr4	48485827	48486325	intron (NM	CpG	218	NM_17230	269536	Tex10	2610206N1
chr7	29655977	29656300	Intergenic	MER58B|D	6733	NM_00849	16858	Lgals7	Galectin-7|
chr7	1.26E+08	1.26E+08	exon (NM_	exon (NM_	863	NM_00103	319622	Itpripl2	C130081G2
chr11	1.2E+08	1.2E+08	promoter-T	promoter-T	52	NM_00116	72055	Slc38a10	1810073N0
chr10	89203802	89204000	Intergenic	Intergenic	-3835	NM_02916	75089	Uhrf1bp1l	2010319N2
chr5	1.3E+08	1.3E+08	intron (NM	CpG	257	NM_00939	19384	Ran	-
chr19	3404427	3404550	intron (NM	(CAG)n|Sin	9969	NM_01025	14419	Gal	Galn
chr11	72074052	72074200	intron (NM	intron (NM	5980	NM_00100	237831	Slc13a5	Indy|NaC2,
chr11	1.07E+08	1.07E+08	exon (NM_	exon (NM_	178	NR_02778	50776	Polg2	-
chr2	60048052	60048150	5' UTR (NM	5' UTR (NM	108	NM_02057	57438	7-Mar	Axo|Axot|(
chr1	1.53E+08	1.53E+08	Intergenic	MLT1H|LTI	-93390	NM_00103	117198	Ivns1abp	1190004M
chr5	1.41E+08	1.41E+08	intron (NM	L1MC5|LIN	1259	NM_01030	14673	Gna12	AI414047|/
chr6	1.24E+08	1.24E+08	Intergenic	MLT1F1|LT	-13511	NM_15350	232370	Clstn3	CSTN3|Cs3
chr14	22123377	22123550	intron (NM	intron (NM	-195529	NM_00120	54169	Kat6b	AI507552|I

chr8	74061252	74061325	promoter-T	promoter-T	48	NM_19809	69550	Bst2	2310015I1(
chr8	82726577	82726675	Intergenic	Intergenic	-144719	NM_02025	15245	Hhip	Hhip1
chr5	1.23E+08	1.23E+08	intron (NM	B1_Mur2|S	4368	NM_17542	109305	Orai1	D730049H(
chr2	1.46E+08	1.46E+08	TTS (NM_0	TTS (NM_0	-14714	NM_02642	67877	Naa20	1500004D1
chr17	34257877	34258075	intron (NM	CpG-6277	716	NM_01023	14312	Brd2	AW228947
chr15	47615077	47615200	intron (NM	intron (NM	1008397	NM_00108	239420	Csmd3	4930500N1
chr12	76853327	76853400	Intergenic	Intergenic	-16647	NM_03075	81535	Sgpp1	AI463453|S
chr3	41368602	41368675	promoter-T	promoter-T	-164	NM_00113	269424	Jade1	AU041499
chr8	1.08E+08	1.08E+08	Intergenic	Intergenic	-1946	NM_02653	68051	Nutf2	2700067I0
chr18	55069927	55070150	intron (NM	intron (NM	79796	NM_17575	269023	Zfp608	4932417D1
chr15	93228352	93228725	promoter-T	promoter-T	183	NM_02602	67197	Zcrb1	2700088M
chr6	38304202	38304425	5' UTR (NM	5' UTR (NM	290	NM_02886	78781	Zc3hav1	1200014N1
chr8	1.25E+08	1.25E+08	Intergenic	Intergenic	-5559	NM_00103	234839	Piezo1	9630020g2
chr14	31363302	31363450	intron (NM	intron (NM	1059	NM_00938	21881	Tkt	p68
chr4	62009527	62009625	intron (NM	intron (NM	12006	NM_00104	338355	Fkbp15	BB131447|
chr5	44247027	44247300	Intergenic	Intergenic	-12903	NM_00764	12494	Cd38	ADPRC 1|C
chr9	64637727	64637800	Intergenic	Lx2|LINE|L	-21055	NM_00116	102442	Dennd4a	AI115600|A
chr9	1.06E+08	1.06E+08	promoter-T	promoter-T	-272	NM_02989	77305	Wdr82	9430077D2
chr1	72423202	72423275	intron (NM	intron (NM	69243	NM_00953	22596	Xrcc5	AI314015|I
chr9	20264277	20264750	promoter-T	promoter-T	91	NM_01175	22688	Zfp26	5033428C0
chr2	35163902	35163975	TTS (NM_0	TTS (NM_0	26038	NM_14612	227753	Gsn	ADF
chr9	32461902	32462300	Intergenic	Intergenic	-41526	NM_01180	23871	Ets1	AI196000|A
chr10	67460752	67460975	intron (NM	intron (NM	18517	NM_00108	170799	Rtkn2	B130039D2
chr2	19579827	19579900	promoter-T	promoter-T	174	NM_02771	71198	Otud1	4933428L1
chr6	42215027	42215200	promoter-T	promoter-T	75	NM_00761	12366	Casp2	Caspase-2|
chr19	8810152	8810400	promoter-T	promoter-T	-53	NM_13413	107071	Wdr74	5730436H2
chr15	59491727	59491800	Intergenic	Intergenic	11554	NM_14454	211770	Trib1	A530090O1
chr2	75656652	75657050	Intergenic	Intergenic	-13383	NM_17266	228061	Agps	5832437L2
chr7	28516377	28516575	promoter-T	promoter-T	48	NM_00956	22718	Zfp60	6330516O1
chr1	1.8E+08	1.8E+08	Intergenic	B1F|SINE|A	-5933	NM_02551	66359	Cox20	2310005N0
chr19	6077027	6077325	promoter-T	promoter-T	11	NM_00108	68505	Vps51	1110014N2
chr2	1.29E+08	1.29E+08	intron (NM	intron (NM	46887	NM_17765	228592	F830045P1	Sirpb3
chr19	5490677	5490950	intron (NM	CpG	358	NM_00768	12631	Cfl1	AA959946|
chr10	93560802	93561225	intron (NM	intron (NM	-49663	NM_01162	22025	Nr2c1	4831444H0
chr10	1.2E+08	1.2E+08	promoter-T	promoter-T	-183	NM_02867	73914	Irak3	4833428C1
chr11	34520977	34521100	intron (NM	intron (NM	76369	NM_03337	94176	Dock2	AI662014|A
chr1	1.93E+08	1.93E+08	Intergenic	Intergenic	63609	NM_02542	66208	Nenf	1110060M
chr5	1.37E+08	1.37E+08	intron (NM	intron (NM	6062	NM_02171	60363	Cldn15	2210009B0
chr8	1.15E+08	1.15E+08	5' UTR (NM	5' UTR (NM	101	NM_01392	30947	Adat1	MMADAT1
chr2	1.67E+08	1.67E+08	5' UTR (NM	5' UTR (NM	111	NM_17075	263876	Spata2	AI504642|r
chr19	33130777	33131075	Intergenic	Intergenic	298859	NM_00896	19211	Pten	2310035O0
chr13	1.14E+08	1.14E+08	promoter-T	promoter-T	71	NM_13079	170625	Snx18	Snag1
chr9	1.04E+08	1.04E+08	intron (NM	intron (NM	30759	NM_00116	235567	Dnajc13	D030002L1
chr10	5160602	5160975	exon (NM_	exon (NM_	8954	NM_00107	64009	Syne1	8B|A33004
chr2	90927927	90928175	Intergenic	Intergenic	-8903	NM_01135	20375	Spi1	Dis-1|Dis1
chr1	88130502	88130725	intron (NM	intron (NM	-69183	NM_14522	227327	B3gnt7	C330001H2
chr11	1.21E+08	1.21E+08	intron (NM	(GA)n|Simp	18150	NM_00108	68837	Foxk2	1110054H0

chr13	43069952	43070075	intron (NM	MTEb|LTR	-148195	NM_00100	218194	Phactr1	9630030F1
chr2	1.18E+08	1.18E+08	Intergenic	Intergenic	-143007	NM_01158	21825	Thbs1	TSP-1|TSP1
chrX	1.26E+08	1.26E+08	intron (NM	intron (NM	1098	NM_17249	54004	Diap2	Dia3|Diaph
chr2	1.53E+08	1.53E+08	Intergenic	Intergenic	5414	NM_01049	15901	Id1	AI323524|
chr11	1.16E+08	1.16E+08	intron (NM	intron (NM	8328	NM_00116	217344	Rhbdf2	4732465I1
chr14	75433827	75433975	intron (NM	intron (NM	18067	NM_19864	271221	5031414D1	Gm744|Gm
chr12	1.1E+08	1.1E+08	intron (NM	B4A|SINE|	43394	NM_17760	212198	Wdr25	B930090D1
chr10	1.25E+08	1.25E+08	Intergenic	RCHARR1|	-42172	NM_01139	20503	Slc16a7	4921534N0
chr11	17080802	17081000	intron (NM	intron (NM	21600	NM_02445	19058	Ppp3r1	CaNB1|Cnb
chr1	77928927	77929025	Intergenic	Intergenic	264735	NM_00878	18505	Pax3	Pax-3|Sp|s
chr7	1.38E+08	1.38E+08	promoter-T	promoter-T	307	NM_00102	11907	Ate1	AI225793|
chr13	30347077	30347175	Intergenic	ID_B1|SINE	-81099	NM_17732	11607	Agtr1a	1810074K2
chr7	66299027	66299200	Intergenic	ETnERV3-ir	-185007	NM_17301	22215	Ube3a	4732496B0
chr17	66408877	66409000	intron (NM	L1_Mus3|L	17448	NM_00102	106585	Ankrd12	2900001A1
chr5	1.51E+08	1.51E+08	intron (NM	CpG	349	NM_20136	381695	N4bp2l2	2700092H0
chr16	55973877	55974000	promoter-T	promoter-T	21	NM_17302	271377	Zbtb11	9230110G0
chr10	61111302	61111625	promoter-T	promoter-T	94	NM_02643	67895	Ppa1	2010317E0
chr3	1.08E+08	1.08E+08	Intergenic	Intergenic	-7556	NM_01196	26442	Psma5	ZETA
chr11	98300102	98300225	intron (NM	CpG	139	NM_02555	103742	Mien1	1810046J1
chr1	1.57E+08	1.57E+08	exon (NM_	exon (NM_	765	NM_01050	15939	Ier5	-
chr9	1.19E+08	1.19E+08	promoter-T	promoter-T	-216	NM_13371	69274	Ctdspl	2810418J2
chr3	1.54E+08	1.54E+08	exon (NM_	exon (NM_	151	NM_00996	12972	Cryz	Sez9
chr12	99972902	99972975	promoter-T	promoter-T	-83	NM_01187	24000	Ptpn21	PTPD1|PTP
chr4	1.43E+08	1.43E+08	promoter-T	promoter-T	84	NM_17287	242736	Pramef8	4732496O0
chr6	83045727	83045825	Intergenic	A-rich|Low	-5734	NM_00108	243510	Ccdc142	A230058J2
chr7	1.06E+08	1.06E+08	intron (NM	intron (NM	116153	NM_17863	78610	Uvrag	9530039D0
chr9	4376027	4376100	Intergenic	Intergenic	-7474	NM_14560	78100	Msantd4	8430410K2
chr13	56804402	56804475	promoter-T	promoter-T	25	NM_00116	17129	Smad5	1110051M
chr13	21622752	21623025	promoter-T	promoter-T	95	NM_13914	93681	Zkscan8	2510038J0
chr5	90794427	90794500	intron (NM	CpG	748	NM_03088	81702	Ankrd17	4933425K2
chr1	1.73E+08	1.73E+08	promoter-T	promoter-T	-265	NM_00100	226652	Arhgap30	6030405P0
chr9	5298552	5298700	5' UTR (NM	5' UTR (NM	109	NM_00980	12362	Casp1	ICE|Il1bc
chr11	87965027	87965200	intron (NM	intron (NM	-17954	NM_00117	103841	Cuedc1	AI841487|
chr5	1.51E+08	1.51E+08	intron (NM	CpG	762	NM_13389	100637	N4bp2l1	2410024N1
chr11	1.03E+08	1.03E+08	Intergenic	Intergenic	-1388	NM_13875	192231	Hexim1	7330426E1
chr2	1.48E+08	1.48E+08	exon (NM_	exon (NM_	598	NM_00937	21824	Thbd	AI385582|
chr16	21955352	21955450	3' UTR (NM	3' UTR (NM	-7776	NR_03777	66664	Tmem41a	2900010K0
chr19	48163502	48163625	Intergenic	MTC|LTR|	-116952	NM_02569	66673	Sorcs3	6330404A1
chr18	3481202	3481275	Intergenic	RLTR41|LT	-26717	NM_02650	68010	Bambi	2610003H0
chr8	35198977	35199075	3' UTR (NM	3' UTR (NM	10767	NM_02660	68192	Leprotl1	1110067H1
chr11	1.18E+08	1.18E+08	intron (NM	CpG	189	NM_17257	217364	Engase	C130099A0
chr7	1.46E+08	1.46E+08	intron (NM	intron (NM	62050	NM_02870	74004	Jakmip3	6330417G0
chr9	50829352	50829600	Intergenic	Intergenic	-12298	NM_17871	235344	Sik2	G630080D2
chr16	20141177	20141450	intron (NM	CpG	177	NM_00114	208146	Yeats2	BC042768|
chr8	67212002	67212350	intron (NM	intron (NM	199	NM_02543	66234	Msmo1	1500001G1
chr15	92832452	92832550	Intergenic	Intergenic	273014	NM_00103	223827	Gxylt1	Glt8d3|Gm
chr11	1.15E+08	1.15E+08	Intergenic	Intergenic	-14358	NM_00111	70355	Gprc5c	1110028I0

chr13	41223652	41223725	Intergenic	Intergenic	-18331	NM_00810	107889	Gcm2	Gcm-rs1|G
chr15	36646052	36646200	Intergenic	Intergenic	74561	NM_00125	22631	Ywhaz	1110013I1
chr6	86388827	86389025	5' UTR (NM	5' UTR (NM	286	NM_00124	232196	C87436	-
chr11	60267852	60268225	promoter-T	promoter-T	-81	NM_02135	13495	Drg2	AI255295|/
chr15	77661652	77662050	intron (NM	intron (NM	10754	NM_02241	17886	Myh9	Fltn|Myhn
chr13	21454752	21455025	intron (NM	CpG	199	NM_01668	22758	Zscan12	2510038J0
chr1	1.83E+08	1.83E+08	Intergenic	Intergenic	-1167	NM_00821	15078	H3f3a	H3.3A
chr4	98680577	98680675	intron (NM	intron (NM	-17020	NM_01391	30924	Angptl3	hypl
chr9	14010102	14010175	Intergenic	Intergenic	-70607	NM_03026	75747	Sesn3	5630400E1
chr3	1.03E+08	1.03E+08	promoter-T	promoter-T	-12	NM_02567	66641	Sike1	2810005O1
chr17	28464752	28466550	intron (NM	CpG	235	NM_01128	19896	Rpl10a	CsA-19|Ne
chr11	67372927	67373200	intron (NM	intron (NM	26563	NM_00808	14457	Gas7	AW124766
chr15	75739527	75739700	promoter-T	promoter-T	161	NM_00128	66656	Eef1d	EF-1-delta
chr7	1.23E+08	1.23E+08	intron (NM	intron (NM	1566	NM_00127	20679	Sox6	AI987981|S
chr5	1.38E+08	1.38E+08	TTS (NM_0	TTS (NM_0	316	NM_02735	70240	Ufsp1	2700038N0
chr15	8344402	8344625	intron (NM	intron (NM	49950	NM_02770	71175	Nipbl	Idn3
chr4	34436727	34436825	Intergenic	L1_Rod|LIN	-61088	NM_00100	433693	Akirin2	2700059D2
chr10	1.21E+08	1.21E+08	intron (NM	intron (NM	1321	NM_00108	73192	Xpot	1110004L0
chr8	1.14E+08	1.14E+08	promoter-T	promoter-T	-161	NM_00914	20340	Glg1	AI593353|/
chr1	1.83E+08	1.83E+08	intron (NM	intron (NM	2339	NM_14479	208795	Tmem63a	BC014795|
chr1	1.79E+08	1.79E+08	intron (NM	MTC|LTR|I	153460	NM_01178	23797	Akt3	AI851531|I
chr7	1.46E+08	1.46E+08	Intergenic	ORR1D2|LT	141912	NM_00104	69546	Mapk1ip1	2310009E0
chr6	1.31E+08	1.31E+08	Intergenic	RMER16-in	42817	NM_00117	16633	Klra2	Klra30|Ly4
chr10	51317052	51317325	Intergenic	Intergenic	-6922	NM_02989	77296	Fam162b	9430073N0
chr11	4491777	4492150	intron (NM	intron (NM	2855	NM_02886	74302	Mtmr3	1700092A2
chr7	1.19E+08	1.19E+08	intron (NM	intron (NM	32531	NM_17724	74996	Usp47	4930502N0
chr11	69495202	69495375	TTS (NM_0	TTS (NM_0	184	NM_03070	80886	Senp3	AA408656|
chr1	1.8E+08	1.8E+08	intron (NM	intron (NM	677	NM_00742	11566	Adss	AI314886|/
chr3	83844227	83844375	promoter-T	promoter-T	-218	NM_17268	229473	D930015E0	Kiaa0922|r
chr13	32304127	32304300	intron (NM	intron (NM	126200	NM_14604	218138	Gmds	BC031788|
chr9	50260002	50260200	Intergenic	Intergenic	-42529	NM_02963	76509	Plet1	0610037B2
chr8	1.12E+08	1.12E+08	Intergenic	Intergenic	-6641	NM_01737	15439	Hp	HP-1|preH
chr13	46822627	46822700	intron (NM	CpG	555	NM_17574	218210	Nup153	B130015D1
chr11	95926252	95926425	exon (NM_	exon (NM_	340	NM_17230	268470	Ube2z	AW049199
chr7	88138052	88138350	promoter-T	promoter-T	-369	NM_17870	233410	Zfp592	8430405N2
chr17	26275177	26275375	promoter-T	promoter-T	-355	NM_00973	12005	Axin1	AI316800|/
chr1	1.41E+08	1.41E+08	promoter-T	promoter-T	-60	NM_00116	329260	Dennd1b	4632404N1
chr3	93316552	93316950	Intergenic	Intergenic	-7667	NM_01674	20195	S100a11	EMAPI|Em
chr5	1.23E+08	1.23E+08	intron (NM	CpG	519	NM_17724	320717	Pptc7	9130017A1
chr15	77630827	77630900	intron (NM	intron (NM	41742	NM_02241	17886	Myh9	Fltn|Myhn
chr5	64203377	64203450	promoter-T	promoter-T	-321	NM_02955	76261	0610040J0	AI662686
chr18	66618627	66618850	intron (NM	intron (NM	480	NM_02145	58801	Pmaip1	Noxa
chr1	1.73E+08	1.73E+08	intron (NM	intron (NM	1592	NM_01018	14127	Fcer1g	AI573376|(
chr5	1.35E+08	1.35E+08	intron (NR_	intron (NR_	1200	NR_03379	68758	Abhd11	1110054D1
chr12	8439602	8440075	Intergenic	L1MB4|LIN	66953	NM_00748	11852	Rhob	AA017882|
chr6	71443902	71444275	5' UTR (NM	5' UTR (NM	200	NM_00954	22644	Rnf103	AW146237
chr8	11012202	11012375	intron (NR_	intron (NR_	-3858	NM_00108	384783	Irs2	Irs-2

chr9	45714277	45714425	promoter-T	promoter-T	-301	NM_00879	18554	Pcsk7	AA959856|
chr1	1.29E+08	1.29E+08	promoter-T	promoter-T	-104	NM_17695	210356	Nckap5	8430408F2
chr9	71010377	71010600	promoter-T	promoter-T	-48	NM_00127	64008	Aqp9	1700020I2
chr16	10545127	10545325	promoter-T	promoter-T	-206	NM_17756	74374	Clec16a	4932416N1
chr13	56284977	56285150	Intergenic	ORR1E|LTF	-4817	NM_00116	212937	Tifab	-
chr16	44173452	44173525	promoter-T	promoter-T	-22	NM_00102	207806	Gm608	5530400K2
chr4	1.36E+08	1.36E+08	intron (NM	MTC|LTR|I	36212	NM_02445	269593	Luzp1	2700072H0
chr3	58312127	58312700	Intergenic	L1M4|LINE	-17330	NM_00100	229317	Eif2a	D030048D2
chr6	1.22E+08	1.22E+08	Intergenic	URR1A|DN	50797	NM_02766	108653	Rimklb	4931417E2
chr7	1.27E+08	1.27E+08	promoter-T	promoter-T	86	NM_14558	233802	Thumpd1	6330575P1
chr5	38651627	38651750	promoter-T	promoter-T	74	NM_02548	66309	Tmem128	2810021O1
chr6	47781827	47781950	Intergenic	RLTR19B|L	-1384	NM_17788	330301	Zfp786	A730012O1
chr6	1.29E+08	1.29E+08	intron (NM	Lx5|LINE|L	6626	NM_17768	232413	Clec12a	CLL-1|D230
chr16	75786527	75786675	Intergenic	Intergenic	-19538	NM_03020	110920	Hspa13	1600002I1
chr11	1.04E+08	1.04E+08	intron (NM	intron (NM	54767	NM_00108	76719	Kansl1	1700081L1
chr19	57511102	57511175	Intergenic	Intergenic	-16258	NM_02983	72133	Trub1	2610009I0
chr15	81642002	81642075	exon (NM_	exon (NM_	194	NM_01737	21685	Tef	2310028D2
chr3	88521327	88521475	intron (NM	intron (NM	625	NM_00116	19769	Rit1	RIBB|ROC1
chr1	1.35E+08	1.35E+08	Intergenic	Intergenic	-8863	NM_00857	17248	Mdm4	4933417N0
chr19	43749002	43749275	promoter-T	promoter-T	233	NM_14515	246696	Slc25a28	2210403D1
chr17	24343177	24343300	promoter-T	promoter-T	-731	NM_00116	224617	Tbc1d24	9630033P1
chr2	1.28E+08	1.28E+08	promoter-T	promoter-T	-76	NM_02876	74121	Acoxl	1200014P0
chr1	1.41E+08	1.41E+08	intron (NM	MusHAL1|	74390	NM_13323	170788	Crb1	7530426H1
chr3	60266227	60266400	Intergenic	Intergenic	-10439	NM_00125	56758	Mbnl1	Mbnl|mKIA
chr7	52926152	52926575	Intergenic	RLTR22_M	-4599	NM_00127	14344	Fut2	-
chr3	1.06E+08	1.06E+08	Intergenic	L1Md_F2|L	-5131	NM_00989	12655	Chil3	AI505981|
chr2	22923677	22924075	promoter-T	promoter-T	155	NM_00110	74159	Acbd5	1300014E1
chr5	8062402	8062675	intron (NM	intron (NM	5996	NM_00108	109552	Sri	2210417O0
chr15	79377252	79377525	promoter-T	promoter-T	-217	NM_19908	67040	Ddx17	2610007K2
chr14	58331777	58331975	intron (NM	intron (NM	21379	NM_15338	50523	Lats2	4932411G0
chr18	60686552	60686700	intron (NM	intron (NM	751	NM_02630	67665	Dctn4	1110001K0
chr5	1.43E+08	1.43E+08	intron (NM	intron (NM	18993	NM_17521	75104	Mmd2	4930518M
chr6	1.21E+08	1.21E+08	intron (NM	L1MB8|LIN	4215	NM_15351	94044	Bcl2l13	BCL-RAMB
chr2	69243777	69244175	Intergenic	Intergenic	25457	NM_17551	241452	Dhrs9	C730025I0
chr1	1.59E+08	1.59E+08	intron (NM	intron (NM	8053	NM_14541	215015	Fam20b	C530043G2
chr15	25760252	25760425	Intergenic	Intergenic	-12715	NM_00103	66270	Fam134b	1810015C0
chr9	30837302	30837375	promoter-T	promoter-T	-891	NM_00111	235132	Zbtb44	6030404E1
chr13	64254202	64254475	5' UTR (NM	5' UTR (NM	169	NM_17549	238673	Zfp367	8030486J2
chr2	18919577	18919800	promoter-T	promoter-T	60	NM_00884	18718	Pip4k2a	AW742916
chr1	94808027	94808250	intron (NM	intron (NM	736	NM_18140	108657	Rnpepl1	1110014H1
chr16	33829377	33829550	promoter-T	promoter-T	-288	NM_01058	16419	Itgb5	AA475909|
chr7	1.08E+08	1.08E+08	intron (NM	intron (NM	-8653	NM_01167	22228	Ucp2	Slc25a8
chr3	9879602	9879800	Intergenic	Intergenic	-46022	NM_00119	94212	Pag1	Cbp|F7300
chr3	60311927	60312200	intron (NM	MIR|SINE|	6889	NM_00125	56758	Mbnl1	Mbnl|mKIA
chr2	38367552	38367700	intron (NM	CpG	230	NM_00115	59126	Nek6	1300007C0
chr17	45879427	45879600	Intergenic	L1MB3|LIN	-8720	NM_02816	72240	1600014C2	-
chr4	1.32E+08	1.32E+08	Intergenic	B1F|SINE|A	-12502	NM_02603	67219	Med18	2810046C0

chr1	1.83E+08	1.83E+08	Intergenic	Intergenic	-41983	NM_02840	72978	Cnih3	2900075G(
chr6	1.16E+08	1.16E+08	intron (NM	intron (NM	23672	NM_02637	67784	Plxnd1	6230425C2
chr16	64852002	64852225	intron (NM	CpG	202	NM_17864	106143	Cggbp1	AA960172|
chr19	3880502	3880625	intron (NR_	intron (NR_	24667	NM_01692	27060	Tcirg1	ATP6N1C|/
chr3	78965102	78965300	Intergenic	Intergenic	-15404	NM_00109	76089	Rapgef2	5830453M
chr18	50068727	50068850	intron (NM	intron (NM	-70293	NM_00117	106869	Tnfaip8	AA987150|
chr15	58586577	58586850	Intergenic	MER91C|D	68269	NM_17521	74868	Tmem65	2610029O1
chr13	48965327	48965500	intron (NM	intron (NM	841	NM_01107	18676	Phf2	GRC5
chr3	1.17E+08	1.17E+08	intron (NM	intron (NM	966	NM_00111	20321	Frrs1	AI131732|!
chr1	1.83E+08	1.83E+08	Intergenic	Intergenic	-39469	NM_20728	403180	Ccdc121	6530421E2
chr4	1.35E+08	1.35E+08	intron (NM	intron (NM	1023	NM_02638	52830	Pnrc2	0610011E1
chr9	96792502	96792750	Intergenic	Intergenic	-2733	NM_00128	235534	Pxylp1	9430094M
chr2	1.65E+08	1.65E+08	Intergenic	Intergenic	-5194	NM_00101	408190	Wfdc13	Wfdc13l1
chr15	77685727	77686125	Intergenic	Intergenic	-13321	NM_02241	17886	Myh9	Fltn|Myhn-
chr2	1.56E+08	1.56E+08	intron (NM	MIRb|SINE	-11373	NM_02025	19018	Scand1	2310003H2
chr1	1.3E+08	1.3E+08	Intergenic	Intergenic	-21370	NM_14550	226414	Dars	5730439G1
chr8	83263877	83264025	promoter-T	promoter-T	-593	NM_05312	93762	Smarca5	4933427E2
chr3	35832602	35832925	promoter-T	promoter-T	-875	NM_00120	114893	Dcun1d1	Rp42|SCCR
chr4	1.32E+08	1.32E+08	promoter-T	promoter-T	-715	NM_20136	381560	Xkr8	4931440N(
chr9	45793352	45793525	promoter-T	promoter-T	-484	NM_00877	18475	Pafah1b2	AI747451|/
chr14	57504977	57505100	Intergenic	CpG	-1594	NM_02949	76007	Zmym2	5830413P(
chr13	90228652	90228825	promoter-T	promoter-T	475	NM_02801	108138	Xrcc4	2310057B2
chr2	60122202	60122425	5' UTR (NM	5' UTR (NM	228	NM_02542	66205	Cd302	1110055L2
chr7	1.17E+08	1.17E+08	intron (NM	intron (NM	92121	NM_00930	20947	Swap70	70kDa|AV2
chr4	1.51E+08	1.51E+08	intron (NM	CpG	234	NM_17270	230935	Dnajc11	E030019A(
chr15	51709052	51709175	exon (NM_	exon (NM_	126	NM_03013	78581	Utp23	1700010I2
chr4	1.25E+08	1.25E+08	intron (NM	intron (NM	3137	NM_15315	230738	Zc3h12a	BC036563|
chrX	13428477	13428725	Intergenic	Intergenic	-4919	NM_00980	12361	Cask	DXPri1|DXI
chr4	91038352	91038475	intron (NM	CpG	333	NM_01048	15569	Elavl2	Hub|mel-N
chr5	54328477	54328750	Intergenic	MLT1H2|LT	-61149	NM_00108	116873	Stim2	-
chr6	1.35E+08	1.35E+08	promoter-T	promoter-T	230	NM_00116	14760	Gpr19	-
chr2	1.55E+08	1.55E+08	intron (NM	MER58B|D	22143	NM_00124	56406	Ncoa6	AIB3|ASC-2
chr17	27760077	27760175	promoter-T	promoter-T	-217	NM_00129	56409	Nudt3	1110011B(
chr16	20426252	20426450	5' UTR (NM	5' UTR (NM	116	NM_01379	27416	Abcc5	2900011L1
chr15	1E+08	1E+08	intron (NM	intron (NM	16763	NM_03347	21422	Tfcp2	CP-2|CP2|
chr1	1.62E+08	1.62E+08	intron (NM	intron (NM	572	NM_02547	64659	Mrps14	1810032L2
chr2	1.8E+08	1.8E+08	intron (NM	intron (NM	7443	NM_14893	108115	Slco4a1	OATP-E|Slc
chr12	78453452	78453575	intron (NR_	intron (NR_	-36338	NM_00125	53618	Fut8	-
chr5	34679202	34679450	intron (NM	CpG	287	NM_01127	19822	Rnf4	AU018689
chr13	49723677	49723800	intron (NM	intron (NM	20298	NM_00876	18295	Ogn	3110079A1
chr11	72812227	72812350	promoter-T	promoter-T	-359	NM_00877	18436	P2rx1	AI323649|
chr13	97753477	97753550	Intergenic	Intergenic	50804	NM_02558	66479	1700029F1	-
chr17	17762327	17762600	Intergenic	CpG-6002	-1010	NM_17282	240028	Lnpep	2010309L0
chr14	21661877	21662250	3' UTR (NM	3' UTR (NM	6179	NM_00887	18792	Plau	u-PA|uPA
chr12	27145102	27145300	Intergenic	Intergenic	-3884	NM_02138	58185	Rsad2	2510004L0
chr8	1.26E+08	1.26E+08	Intergenic	Intergenic	11609	NM_13395	69581	Rhou	2310026M
chr10	18729277	18729350	intron (NM	intron (NM	2456	NM_00116	21929	Tnfaip3	A20|Tnfip3

chr11	54673327	54673425	intron (NM	B1F|SINE|/	717	NM_02932	75530	Lyrm7	1700024C2
chr4	15885227	15885350	intron (NM	CpG	174	NM_01375	27354	Nbn	Nbs1
chr17	47131902	47132250	intron (NM	intron (NM	15405	NM_14607	224826	Ubr2	9930021A0
chr3	51212702	51212800	intron (NM	CpG	126	NM_02552	66377	Ndufc1	2310016K2
chr1	1.34E+08	1.34E+08	intron (NM	intron (NM	11258	NM_17251	213452	Dstyk	A930019K2
chr8	87823977	87824050	promoter-T	promoter-T	482	NR_02826	56452	Orc6	6720420I1
chr9	1.07E+08	1.07E+08	intron (NM	CpG	130	NM_13398	69536	Hemk1	2310008M
chr2	1.1E+08	1.1E+08	promoter-T	promoter-T	-222	NM_01169	22343	Lin7c	9130007B1
chr13	84332352	84332550	Intergenic	Lx8|LINE|L	-29450	NR_02941	72745	Tmem161b	2810446P0
chr12	86159302	86159625	intron (NM	intron (NM	45243	NM_02886	74316	Isca2	0710001C0
chr2	32248627	32248775	Intergenic	Intergenic	-2709	NM_13378	96979	Ptges2	0610038H1
chr15	78758727	78758800	intron (NM	G-rich|Low	1608	NM_00849	16852	Lgals1	AA410090|
chr13	1.13E+08	1.13E+08	Intergenic	PB1D9|SIN	-3860	NM_01194	26401	Map3k1	MAPKKK1|
chr10	19246177	19246450	Intergenic	Intergenic	-65451	NM_01051	15979	Ifngr1	CD119|IFN
chr6	30536827	30537225	intron (NM	intron (NM	18650	NM_02792	71791	Cpa4	1110019K2
chr16	32878077	32878250	intron (NM	CpG	293	NM_00115	69823	Fyttd1	2010005M
chr16	16303302	16303450	non-coding	non-coding	318	NM_19824	70120	Yars2	2210023C1
chr17	21626102	21626200	5' UTR (NM	5' UTR (NM	199	NM_01384	24132	Zfp53	D030067O
chr5	1.4E+08	1.4E+08	promoter-T	promoter-T	-56	NM_19798	68033	Cox19	1810074D0
chr6	70741752	70742400	promoter-T	promoter-T	93	NM_00907	19895	Rpia	RPI
chr17	25252602	25252850	5' UTR (NM	5' UTR (NM	124	NM_02788	71718	Telo2	1200003M
chr14	1.04E+08	1.04E+08	intron (NM	MER20|DN	18255	NM_02288	64929	Scel	9230114I0
chr16	22202052	22202150	Intergenic	Intergenic	-38729	NM_18302	319765	Igf2bp2	C330012H0
chr3	68930277	68930375	intron (NM	CpG	688	NM_00846	16649	Kpna4	1110058D0
chr10	37335777	37336000	Intergenic	Intergenic	-477156	NM_00853	17118	Marcks	Macs|PKCS
chr13	76192777	76192850	3' UTR (NM	3' UTR (NM	1631	NM_17549	238725	Gpr150	C030001A1
chr2	71711627	71711775	intron (NM	CpG	420	NM_17266	228026	Pdk1	B830012B0
chr3	90499527	90499950	promoter-T	promoter-T	-95	NM_00911	20202	S100a9	60B8Ag|A\
chr4	1.1E+08	1.1E+08	Intergenic	MLT1H1|LT	9808	NM_17229	242620	Dmrta2	Dmrt5
chr4	1.06E+08	1.06E+08	Intergenic	ORR1C1|LT	-1902	NM_02559	329910	Acot11	1110020M
chr6	18552027	18552100	Intergenic	Intergenic	-87238	NM_08028	30785	Cttnbp2	3010022N2
chr6	1.47E+08	1.47E+08	Intergenic	Intergenic	-46706	NM_00897	19227	Pthlh	PTH-like|P
chr7	1.51E+08	1.51E+08	promoter-T	promoter-T	54	NM_00785	13360	Dhcr7	AA409147
chr7	1.07E+08	1.07E+08	5' UTR (NM	5' UTR (NM	529	NM_20135	233552	Gdpd5	BC024955|
chr18	35278452	35278675	promoter-T	promoter-T	-3	NM_00981	12385	Ctnna1	2010010M
chr11	51670102	51670225	intron (NM	CpG	820	NM_19929	76901	Jade2	1200017K0
chr2	90420127	90420350	intron (NM	CpG	566	NM_00898	19271	Ptprj	AI450271|
chr17	74299502	74299650	promoter-T	promoter-T	-40	NM_01172	22436	Xdh	XO|Xor|Xo
chr2	29904602	29904900	promoter-T	promoter-T	-352	NM_00100	71820	Wdr34	3200002I0
chr1	95651402	95651725	promoter-T	promoter-T	-47	NM_17487	66615	Atg4b	2510009N0
chr18	73732927	73733100	exon (NM_	exon (NM_	654	NM_00103	240396	Mex3c	A130001D1
chr5	1.22E+08	1.22E+08	Intergenic	Intergenic	67893	NM_00110	19247	Ptpn11	2700084A1
chr5	20694827	20695000	intron (NM	intron (NM	2828	NM_17543	212167	Gsap	A530088I0
chr12	1.02E+08	1.02E+08	intron (NM	intron (NM	-14193	NM_00117	238377	Gpr68	BB131428|
chr12	56100227	56100350	Intergenic	MLT1J2|LT	-12965	NM_01381	217578	Baz1a	Acf1|B930
chr3	19415602	19415800	intron (NM	intron (NM	7106	NM_02548	66326	Dnajc5b	1700008A0
chr17	25929177	25929350	promoter-T	promoter-T	69	NM_00128	214917	Fam173a	-

chr13	24498852	24499000	intron (NM	MER21-int	-8065	NM_00771	12763	Cmah	-
chr2	52601377	52601750	promoter-T	promoter-T	-380	NM_01966	56324	Stam2	1200004O1
chr9	1E+08	1E+08	intron (NM	CpG	584	NM_01087	17973	Nck1	6330586M
chr1	95721852	95722025	intron (NM	intron (NM	121	NM_17888	98314	D2hgdh	AA408776|
chr11	29147252	29147400	promoter-T	promoter-T	-54	NM_02574	216618	Ccdc104	2300003H1
chr4	1.38E+08	1.38E+08	promoter-T	promoter-T	-763	NM_00109	230863	Sh2d5	BC036961
chr1	1.56E+08	1.56E+08	intron (NM	intron (NM	3132	NM_01188	24014	Rnasel	E230029I0
chr1	34529502	34529775	exon (NM_	exon (NM_	13047	NM_01120	19253	Ptpn18	FLP1|HSCF
chrX	74661052	74661175	Intergenic	ID4|SINE|I	-95453	NM_02060	21372	Tbl1x	5330429M
chr15	96588077	96588300	Intergenic	Intergenic	-58059	NM_17512	67760	Slc38a2	5033402L1
chr8	28153152	28153250	intron (NM	CpG	174	NM_05405	114863	Prosc	1700024N2
chr5	46072177	46072250	intron (NM	MTEa|LTR|	11049	NM_01943	54392	Ncapg	5730507H0
chr7	1.06E+08	1.06E+08	Intergenic	Intergenic	-5159	NM_17863	78610	Uvrag	9530039D0
chr17	24005452	24005875	exon (NM_	exon (NM_	624	NM_02048	57256	Prss21	1700023E1
chr8	81331402	81331525	3' UTR (NM	3' UTR (NM	13588	NM_00119	78651	Lsm6	1500031N1
chr15	36264927	36265200	Intergenic	Intergenic	-52161	NM_01392	30945	Rnf19a	AA032313|
chr19	5366927	5367100	promoter-T	promoter-T	-137	NM_01179	23825	Banf1	Baf|Bcrp1|
chr16	18082427	18082525	intron (NM	intron (NM	6807	NM_01117	19125	Prodh	Pro-1|Pro1
chr11	22193952	22194150	Intergenic	Intergenic	-7253	NM_00125	216565	Ehbp1	AF424697|
chrX	1.01E+08	1.01E+08	intron (NM	CpG	522	NM_01127	19820	Rlim	AL022832|
chr1	38160202	38160475	intron (NM	intron (NM	26169	NM_01957	56210	Rev1	1110027I2
chr17	5206777	5207050	intron (NM	intron (NM	211839	NM_00108	239985	Arid1b	8030481M
chr1	94965002	94965075	intron (NM	intron (NM	14019	NM_00129	16560	Kif1a	ATSV|C630
chr4	1.5E+08	1.5E+08	intron (NM	CpG	90051	NM_00108	68703	Rere	1110033A1
chr6	48504677	48504850	promoter-T	promoter-T	-35	NM_00111	243371	Lrrc61	BC027309
chr17	35095927	35096175	5' UTR (NM	5' UTR (NM	132	NM_01047	15511	Hspa1b	Hsp70|Hsp
chr13	96611952	96612025	intron (NM	intron (NM	49889	NM_02771	544963	Iqgap2	4933417J2
chr5	80395427	80395500	Intergenic	Intergenic	-1055155	NM_19870	319387	Lphn3	5430402I2
chr1	1.01E+08	1.01E+08	Intergenic	Intergenic	312575	NM_02758	70866	Slco6d1	4921511I0
chr5	77403627	77403975	promoter-T	promoter-T	75	NM_02569	66661	Srp72	5730576P1
chr6	1.21E+08	1.21E+08	Intergenic	Intergenic	16825	NM_01737	53857	Tuba8	-
chr15	78860852	78861050	promoter-T	promoter-T	-353	NM_01384	26912	Gcat	AI526977|
chr1	1.63E+08	1.63E+08	Intergenic	Intergenic	-17666	NM_00745	11758	Prdx6	1-cysPrx|9
chr1	72301952	72302250	Intergenic	Intergenic	28787	NM_02352	111175	Pecr	2400003B1
chr3	41369427	41369575	intron (NM	CpG	699	NM_00113	269424	Jade1	AU041499
chr6	51419027	51419400	intron (NM	CpG	680	NM_18265	53379	Hnrnpa2b1	9130414A0
chr7	1.33E+08	1.33E+08	Intergenic	Intergenic	-6242	NM_02188	60504	Il21r	NILR
chr10	85514652	85514925	3' UTR (NM	3' UTR (NM	30114	NM_15319	69754	Fbxo7	2410015K2
chr19	5560102	5560175	intron (NM	CpG	437	NM_01993	18426	Ovol1	BB147136|
chr16	32396102	32396175	Intergenic	Intergenic	-4454	NM_00116	277203	Tm4sf19	EG277203
chr5	1.45E+08	1.45E+08	promoter-T	promoter-T	-91	NM_17768	231874	Ccz1	AU022870
chr8	1.09E+08	1.09E+08	promoter-T	promoter-T	-450	NM_02149	58994	Smpd3	4631433G0
chr7	87377177	87377375	promoter-T	promoter-T	415	NM_00129	23991	Cib1	Cibkip|Kip|
chr8	87550927	87551100	exon (NM_	exon (NM_	281	NM_02676	68544	2310036O2	1110002H1
chr1	34541602	34541800	Intergenic	Intergenic	-13574	NM_00935	21755	Prss39	Tesp1
chr6	1.21E+08	1.21E+08	intron (NM	CpG	152	NM_00751	11973	Atp6v1e1	2410029D2
chr16	36935502	36935575	intron (NM	intron (NM	469	NM_00822	15163	Hcls1	AW213261

chr8	41824652	41824875	Intergenic	ETnERV3-ir	-12799	NM_01178	23793	Adam25	-
chr14	21460227	21460450	Intergenic	CpG	15424	NM_02150	59011	Myoz1	2310001N1
chr11	1.01E+08	1.01E+08	intron (NM	intron (NM	1015	NM_00116	20850	Stat5a	AA959963|
chr7	1.4E+08	1.4E+08	intron (NM	intron (NM	7453	NM_00998	13017	Ctbp2	AA407280|
chr11	90044177	90044475	Intergenic	Intergenic	-66464	NM_02617	67468	Mmd	1200017E0
chr15	81354127	81354350	Intergenic	Intergenic	57492	NM_01971	56438	Rbx1	1500002P1
chr14	32977727	32977900	promoter-T	promoter-T	-959	NM_00128	27057	Ncoa4	ARA70|Rfg
chr3	94690677	94690950	promoter-T	promoter-T	67	NM_00894	19172	Psmb4	Pros-27
chr17	24615827	24615975	intron (NM	CpG	122	NM_00125	56716	Mlst8	0610033N1
chr3	63780102	63780525	intron (NM	CpG	248	NM_00103	229363	Gmps	AA591640|
chr2	1.1E+08	1.1E+08	Intergenic	MYSERV16_	-60226	NM_02627	67606	Fibin	1110018M
chr4	1.33E+08	1.33E+08	Intergenic	Intergenic	12536	NM_00114	230801	Pigv	B330013B0
chr14	47808677	47809025	exon (NM_	exon (NM_	226	NM_00810	14528	Gch1	GTP-CH|GT
chr1	3436027	3436125	intron (NM	RLTR45|LT	225503	NM_00101	497097	Xkr4	AY534250|
chr14	45938952	45939025	promoter-T	promoter-T	-741	NM_01577	50527	Ero1l	ERO1-L
chr2	11524602	11524775	intron (NM	CpG	138	NM_15282	76938	Rbm17	2700027J0
chrX	9037802	9037950	intron (NM	intron (NM	8574	NM_00780	13058	Cybb	C88302|CG
chr10	20803477	20803725	Intergenic	Intergenic	42975	NM_00117	52906	Ahi1	1700015F0
chr8	98411777	98411900	intron (NM	intron (NM	427	NM_01032	14719	Got2	AL022787|
chr9	71980452	71980975	Intergenic	CpG	-21087	NM_00125	21406	Tcf12	A130037E0
chr10	17196527	17196725	Intergenic	ORR1E|LTF	-246408	NM_01082	17684	Cited2	AI835299|
chr19	6389952	6390125	intron (NM	intron (NM	5609	NM_01122	19309	Pygm	AI115133|
chr9	1.11E+08	1.11E+08	intron (NM	L1MB4|LIN	6506	NM_02831	72654	Ccdc12	2700094L0
chr2	1.02E+08	1.02E+08	Intergenic	MIR|SINE|	-4306	NM_02963	76501	Commd9	1810029F0
chr4	1.52E+08	1.52E+08	intron (NM	intron (NM	6653	NM_00108	269610	Chd5	4930532L2
chr11	88017977	88018075	non-coding	non-coding	136	NM_00129	64656	Mrps23	D11Bwg11
chr10	26476277	26476350	Intergenic	Intergenic	-16005	NM_17683	73910	Arhgap18	4833419J0
chr12	1.05E+08	1.05E+08	intron (NM	intron (NM	952	NM_19931	380780	Serpina11	Gm895
chr8	98011927	98012150	intron (NM	intron (NM	682	NM_00997	13000	Csnk2a2	1110035J2
chr8	1.22E+08	1.22E+08	intron (NM	intron (NM	7161	NM_02339	67866	Wfdc1	2310058A0
chr1	1.55E+08	1.55E+08	intron (NM	L2|LINE|L2	56057	NM_17546	226518	Nmnat2	AI843915|
chrX	39264727	39265025	intron (NM	CpG	202	NM_00103	331401	Thoc2	6330441O1
chr14	62026077	62026150	intron (NM	B4A|SINE|	32671	NM_00846	16648	Kpna3	IPOA4
chr19	21514002	21514125	intron (NM	intron (NM	33088	NM_01026	14544	Gda	AU015411
chr3	1.23E+08	1.23E+08	Intergenic	Intergenic	-13330	NM_00893	19142	Prss12	Bssp-3|mo
chr11	1.16E+08	1.16E+08	intron (NM	intron (NM	-4267	NM_01572	11430	Acox1	AOX|Acox|
chr6	86800152	86800275	promoter-T	promoter-T	702	NM_00104	269774	Aak1	5530400K1
chr17	80623652	80623775	promoter-T	promoter-T	-116	NM_02605	67242	Gemin6	2610019B1
chr14	48269452	48269650	intron (NM	CpG	565	NM_00129	16709	Ktn1	-
chr10	1.2E+08	1.2E+08	promoter-T	promoter-T	-240	NM_00108	380664	Lemd3	AI316861|
chr4	55636152	55636625	Intergenic	Intergenic	-91041	NM_01063	16600	Klf4	EZF|Gklf|Z
chr17	79754152	79754275	5' UTR (NM	5' UTR (NM	218	NM_02651	260409	Cdc42ep3	3200001F0
chr18	25467077	25467200	intron (NM	Lx9|LINE|L	139617	NM_00103	225289	AW554918	4921538P2
chr14	79699677	79699875	intron (NM	intron (NM	1666	NM_02542	66214	Rgcc	1190002H2
chr12	1.12E+08	1.12E+08	Intergenic	Intergenic	-3839	NM_01197	26448	Mok	RAGE1|Rag
chr1	64579577	64579825	intron (NM	CpG	323	NM_00103	12912	Creb1	2310001E1
chr17	51132902	51133100	intron (NM	RLTR14-int	165702	NM_00128	72238	Tbc1d5	1600014N0

chr8	90724102	90724200	exon (NM_	exon (NM_	1039	NM_00116	214627	Papd5	5730445M
chr8	1.27E+08	1.27E+08	promoter-T	promoter-T	-54	NM_13396	102162	Taf5l	1110005N(
chr1	39582552	39583075	Intergenic	Intergenic	-9834	NM_02804	52846	Cnot11	2410015L1
chr6	1.47E+08	1.47E+08	promoter-T	promoter-T	168	NM_02621	67529	Fgfr1op2	1500031J0
chr7	51846727	51846900	exon (NM_	exon (NM_	557	NM_00129	16504	Kcnc3	KShIIID|Kc
chr8	1.07E+08	1.07E+08	promoter-T	promoter-T	45	NM_00102	382051	Pdp2	4833426J0
chr14	32643152	32643225	intron (NM	CpG	413	NM_00102	105522	Ankrd28	AI465466|
chr6	34827902	34827975	5' UTR (NM	5' UTR (NM	127	NM_19914	78412	3110062M	AL023049|
chr6	24560352	24560675	Intergenic	Intergenic	12742	NM_05309	93677	Lmod2	C-Lmod
chr5	1.48E+08	1.48E+08	promoter-T	promoter-T	93	NM_02565	66596	Gtf3a	2010015D(
chr9	54998127	54998225	intron (NM	intron (NM	1000	NM_18060	109161	Ube2q2	3010021M
chr7	36587752	36587925	non-coding	non-coding	170	NM_17773	668501	Zfp507	1810022O1
chr7	52112402	52112525	promoter-T	promoter-T	-53	NM_02154	59047	Pnkp	1810009G(
chr18	46756627	46756750	promoter-T	promoter-T	501	NM_02569	66676	Tmed7	3930401E1
chr17	74922627	74922825	Intergenic	URR1B|DN	-4909	NM_00756	12211	Birc6	A430032G(
chr4	59453752	59453950	Intergenic	B4A|SINE|	-2346	NM_00116	634731	Susd1	A530080P1
chr6	86475877	86476050	5' UTR (NM	5' UTR (NM	196	NM_01186	23983	Pcbp1	WBP17|[a]
chr2	1.73E+08	1.73E+08	Intergenic	Intergenic	46363	NM_00108	664799	Ctcfl	Boris|OTTM
chr18	3005302	3005900	Intergenic	Intergenic	117811	NM_00116	1E+08	Vmn1r238	-
chr3	1.09E+08	1.09E+08	intron (NM	Charlie25|	31346	NM_17268	229731	Slc25a24	2610016M
chr19	37665002	37665575	intron (NM	intron (NM	40380	NM_17535	107371	Exoc6	4833405E0
chr15	91003477	91003575	intron (NM	intron (NM	18712	NM_01199	26874	Abcd2	ABC39|ALD
chr7	57033652	57033825	promoter-T	promoter-T	10	NM_13374	71974	Prmt3	2010005E2
chr3	69908127	69908425	Intergenic	Intergenic	96741	NM_00101	229389	Otol1	Gm414
chr7	26492577	26492725	Intergenic	Intergenic	11421	NM_02877	52132	Ccdc97	1200014H1
chr6	1.31E+08	1.31E+08	intron (NM	intron (NM	492	NM_13911	56449	Ybx3	Csda|Dpba
chr11	5402352	5402725	Intergenic	MIRc|SINE	-18106	NM_01384	22433	Xbp1	D11Ertd39
chr7	1.07E+08	1.07E+08	promoter-T	promoter-T	64	NM_02601	67164	Lipt2	2610209A2
chr8	80774527	80774975	intron (NM	B1_Mus1|	37510	NM_18309	73301	Ttc29	1700031F1
chr15	63832702	63832800	intron (NM	intron (NM	59259	NM_14484	223601	Fam49b	0910001A(
chr2	4802077	4802425	promoter-T	promoter-T	-359	NM_17540	109079	Sephs1	1110046B2
chr10	40020802	40020875	intron (NM	CpG	1154	NM_00744	1E+08	Amd2	AdoMetDC
chr8	93463902	93464050	intron (NM	intron (NM	111242	NM_17722	109151	Chd9	1810014J1
chr14	35306977	35307175	intron (NM	Lx7|LINE|L	8656	NM_00975	12166	Bmpr1a	1110037I2
chr9	65064852	65065000	intron (NM	intron (NM	2429	NM_17746	214424	Parp16	ARTD15|B(
chr6	67215852	67216025	Intergenic	Intergenic	-1035	NM_00111	66870	Serbp1	1200009K1
chr8	47570352	47570600	intron (NM	L1MB4|LIN	14080	NM_00798	14081	Acsl1	Acas|Acas1
chr6	1.34E+08	1.34E+08	intron (NM	intron (NM	5863	NM_00796	14011	Etv6	AW123102
chr11	1.01E+08	1.01E+08	promoter-T	promoter-T	-928	NM_01692	11975	Atp6v0a1	AA959968|
chr17	79626677	79626825	Intergenic	Intergenic	127680	NM_02651	260409	Cdc42ep3	3200001F0
chr11	1.2E+08	1.2E+08	intron (NM	CpG	335	NM_01075	17134	Mafg	AA545192|
chr16	33185327	33185450	5' UTR (NM	5' UTR (NM	231	NM_17684	106326	Osbpl11	9430097N(
chr1	88406102	88406225	Intergenic	Intergenic	-17148	NM_00897	19231	Ptma	Thym
chr6	92042502	92042600	intron (NM	intron (NM	1139	NM_01163	22026	Nr2c2	TAK1|Tr4|
chr4	1.16E+08	1.16E+08	intron (NM	CpG	216	NM_17530	100465	Mob3c	AW822253
chr7	1.08E+08	1.08E+08	Intergenic	MIR|SINE|	-15296	NM_00877	18442	P2ry2	P2U1|P2Y2
chr11	53164452	53165000	intron (NM	CpG	457	NM_03356	93736	Aff4	AF5Q31|Al

chr4	1.35E+08	1.35E+08	intron (NM	intron (NM	32503	NM_01973	12399	Runx3	AML2|Cbfa
chr5	1.47E+08	1.47E+08	promoter-T	promoter-T	132	NM_02877	74132	Rnf6	1200013I0
chr2	1.25E+08	1.25E+08	intron (NM	CpG	445	NM_00116	17876	Myef2	9430071B0
chr17	31432552	31432625	promoter-T	promoter-T	160	NM_15306	224674	Slc37a1	G3PP
chr3	89074377	89074675	promoter-T	promoter-T	-34	NM_00905	19729	Slc50a1	MmSWEET
chr13	1.08E+08	1.08E+08	intron (NM	CpG	257	NM_02966	76582	Ipo11	1700081H0
chr6	1.13E+08	1.13E+08	intron (NM	intron (NM	1900	NM_00124	67767	Jagn1	5830427H1
chr12	1.1E+08	1.1E+08	Intergenic	Intergenic	-28295	NM_00953	22632	Yy1	AW488674
chr3	94819577	94819800	promoter-T	promoter-T	-528	NM_03007	78266	Zfp687	4931408L0
chr1	1.73E+08	1.73E+08	promoter-T	promoter-T	-396	NM_01948	56009	Alyref2	C130042O1
chr1	1.66E+08	1.66E+08	intron (NM	CpG	592	NM_02877	240880	Scyl3	1200016D2
chr7	1.29E+08	1.29E+08	promoter-T	promoter-T	91	NM_00103	76179	Usp31	6330567E2
chr18	4969677	4970025	Intergenic	Intergenic	-76736	NM_15315	225115	Svil	AU024053
chr1	88159677	88159775	intron (NM	intron (NM	-40070	NM_14522	227327	B3gnt7	C330001H2
chr4	1.07E+08	1.07E+08	intron (NM	intron (NM	38308	NM_02561	66526	Tceanc2	2210010B2
chr6	83745502	83745700	intron (NM	intron (NM	449	NM_00116	56174	Nagk	Gnk
chr15	80085202	80085350	promoter-T	promoter-T	-338	NM_00128	11911	Atf4	Atf-4|C/AT
chr4	1.49E+08	1.49E+08	promoter-T	promoter-T	-925	NM_00102	18707	Pik3cd	2410099E0
chr10	1.27E+08	1.27E+08	promoter-T	promoter-T	-425	NM_01976	56351	Ptges3	5730442A2
chr8	67350877	67351025	intron (NM	URR1A|DN	22871	NM_17863	77113	Klhl2	6030411N2
chr4	89941052	89941150	Intergenic	RMER16-in	55590	NM_15310	236537	Zfp352	2czf48
chr17	35372752	35372900	promoter-T	promoter-T	-66	NM_01090	18038	Nfkbil1	Def-7|IKBL
chr12	1.12E+08	1.12E+08	intron (NM	intron (NM	1695	NM_01163	22031	Traf3	AI528849|
chr3	86029152	86029275	exon (NM_	exon (NM_	601	NM_00107	80877	Lrba	C80285|D3
chr5	1.36E+08	1.36E+08	intron (NM	intron (NM	-68910	NM_14893	107939	Pom121	2610027A1
chr12	1.2E+08	1.2E+08	promoter-T	promoter-T	-400	NM_00116	20688	Sp4	5730497N0
chr1	53798977	53799175	Intergenic	Intergenic	-35448	NM_00125	627872	Dnah7a	Dnahc7|Dn
chr3	97828377	97828575	intron (NM	intron (NM	11015	NM_01092	18129	Notch2	AI853703|
chr3	89716827	89717300	promoter-T	promoter-T	21	NM_01055	16194	Il6ra	CD126|IL-6
chr16	30502627	30502800	Intergenic	(CGAG)n|S	47951	NM_17261	224090	Tmem44	1700007N0
chr1	36697352	36697575	intron (NM	(CA)n|Simp	42565	NM_00112	381337	Fam178b	1700024G1
chr14	49895077	49895250	Intergenic	Intergenic	-30060	NM_02595	67082	1700011H1	-
chr2	1.73E+08	1.73E+08	promoter-T	promoter-T	92	NM_02443	19334	Rab22a	3732413A1
chr14	56178802	56179000	promoter-T	promoter-T	35	NM_13373	28199	Dcaf11	0710008A1
chr12	25339952	25340175	exon (NM_	exon (NM_	3827	NM_17835	194655	Klf11	9830142A1
chr8	74246752	74247200	intron (NR_	intron (NR_	2604	NR_028267	74015	Fcho1	3322402E1
chr16	62786577	62787175	promoter-T	promoter-T	-334	NM_17892	106338	Nsun3	6720484A0
chr9	1.09E+08	1.09E+08	exon (NM_	exon (NM_	5562	NM_08043	107934	Celsr3	Fmi1|flami
chr5	1.3E+08	1.3E+08	Intergenic	Intergenic	-11803	NM_02712	69568	Vkorc1l1	2310024K0
chr7	1.47E+08	1.47E+08	promoter-T	promoter-T	-179	NM_05311	93747	Echs1	C80529
chr17	71644002	71644250	intron (NM	intron (NM	16179	NM_14515	246707	Emilin2	FOAP-10
chr14	55222952	55223075	intron (NM	intron (NM	1732	NM_02889	74359	4931414P1	-
chr3	94247477	94247575	intron (NM	CpG	268	NM_03011	78523	Mrpl9	8030480E2
chr2	1.74E+08	1.74E+08	Intergenic	Intergenic	-4558	NM_02232	64138	Ctsz	AI787083|
chr1	1.22E+08	1.22E+08	intron (NM	intron (NM	12919	NM_00101	319229	Sctr	6530402O0
chr1	1.82E+08	1.82E+08	5' UTR (NM	5' UTR (NM	433	NM_02637	226747	Ahctf1	6230412P2
chr6	1.27E+08	1.27E+08	Intergenic	Lx5|LINE|L	-51880	NM_00103	381812	Cracr2a	Efcab4b|G

chr12	1.07E+08	1.07E+08	intron (NM	intron (NM	21653	NM_00102	22367	Vrk1	51PK
chr11	19825202	19825300	intron (NM	CpG	806	NM_03352	114716	Spred2	C79158
chr12	74387452	74387575	promoter-T	promoter-T	185	NM_02958	76357	Trmt5	2610027O1
chr10	79756352	79756575	exon (NM_	exon (NM_	1287	NM_00909	20054	Rps15	rig
chr12	33452202	33452675	Intergenic	Intergenic	-52762	NM_02152	59027	Nampt	1110035O1
chr2	1.52E+08	1.52E+08	Intergenic	Intergenic	-9575	NM_00778	12995	Csnk2a1	Csnk2a1-rs
chr6	72295477	72295600	promoter-T	promoter-T	-369	NM_13859	28035	Usp39	AA408960|
chr6	57653027	57653175	exon (NM_	exon (NM_	652	NM_13373	71835	Lancl2	1700003F1
chr1	58643102	58643500	promoter-T	promoter-T	-124	NM_17251	213056	Fam126b	C130065N1
chr11	22541427	22541775	Intergenic	Lx8|LINE|L	129315	NM_15359	103765	Tmem17	AI503894|A
chr6	1.19E+08	1.19E+08	intron (NM	intron (NM	-2013	NM_00125	12288	Cacna1c	Cav1.2|Cch
chr6	1.19E+08	1.19E+08	intron (NM	intron (NM	8140	NM_13394	101358	Fbxl14	AW322056
chr17	26388827	26388950	promoter-T	promoter-T	-967	NM_02588	66978	Luc7l	1810045C0
chr9	40449452	40449775	Intergenic	Intergenic	-44434	NM_13373	71566	Clmp	9030425E1
chr2	53051227	53051475	exon (NM_	exon (NM_	233	NM_02298	65103	Arl6ip6	2310057C0
chr5	1.39E+08	1.39E+08	intron (NM	intron (NM	9428	NM_03056	80752	Fam20c	C76981|DN
chr11	1.04E+08	1.04E+08	promoter-T	promoter-T	308	NM_00128	217232	Cdc27	AI452358|A
chr15	79722452	79722725	promoter-T	promoter-T	-250	NM_00116	80287	Apobec3	Arp3|BC00
chr8	25568002	25568350	Intergenic	Intergenic	-18758	NM_02693	69068	1810011O1	1110065B0
chr19	37382302	37382425	intron (NM	B1F|SINE|A	22740	NM_03115	15925	Ide	1300012G0
chr2	1.63E+08	1.63E+08	intron (NM	CpG	130	NM_17215	245866	Ift52	BC037708|
chr5	3420302	3420525	intron (NM	intron (NM	76101	NM_00987	12571	Cdk6	5830411I2
chr12	1.13E+08	1.13E+08	5' UTR (NM	5' UTR (NM	147	NM_00102	16593	Klc1	AI874768|
chr10	1.17E+08	1.17E+08	Intergenic	MTE2b|LTR	13052	NM_01737	17105	Lyz2	AI326280|
chr4	1.48E+08	1.48E+08	intron (NM	intron (NM	136712	NM_02719	69743	Casz1	2410019P0
chr9	99080877	99081000	Intergenic	Intergenic	-40308	NM_02909	74769	Pik3cb	1110001J0
chr6	1.18E+08	1.18E+08	promoter-T	promoter-T	-141	NM_19433	213895	Bms1	AA408648|
chr8	4677502	4677675	Intergenic	CpG	64418	NM_14559	233987	Zfp958	-
chr1	88400502	88401000	Intergenic	CpG	-22560	NM_00897	19231	Ptma	Thym
chr10	1.11E+08	1.11E+08	Intergenic	ID_B1|SINE	-30482	NM_00100	237542	Osbpl8	AA536976|
chr5	23957977	23958100	promoter-T	promoter-T	43	NM_00125	213990	Agap3	AGAP-3|AV
chr14	35124252	35124600	promoter-T	promoter-T	513	NM_00813	14661	Glud1	AI118167|
chr7	1.26E+08	1.26E+08	Intergenic	Intergenic	23826	NM_00103	319622	Itpripl2	C130081G2
chr5	1.22E+08	1.22E+08	intron (NM	CpG	683	NM_00912	20239	Atxn2	9630045M
chr4	71860852	71860925	intron (NM	CpG	1039	NM_01159	21885	Tle1	C230057C0
chr10	82406852	82407150	Intergenic	RMER19B|	-41241	NM_02143	58250	Chst11	1110020P0
chr11	1.01E+08	1.01E+08	promoter-T	promoter-T	-29	NM_02656	68107	Cntd1	1700051C0
chr4	1.19E+08	1.19E+08	intron (NM	CpG	585	NM_17269	230700	Foxj3	C330039G0
chr9	54397252	54397600	intron (NM	intron (NM	10460	NM_01968	56506	Cib2	2810434I2
chr13	58503652	58504475	promoter-T	promoter-T	105	NM_00116	74386	Rmi1	4932432N1
chr13	47139402	47139475	promoter-T	promoter-T	570	NM_17226	218214	Kdm1b	4632428N0
chr12	1.19E+08	1.19E+08	intron (NM	CpG	17924	NM_17593	217944	Rapgef5	4932413M
chr3	96301452	96301525	Intergenic	Intergenic	-27620	NM_02712	69585	Hfe2	2310035L1
chr6	64377377	64377475	intron (NM	L1_Mur3|L	-301714	NM_00750	11921	Atoh1	Hath1|MA
chr11	23184502	23184725	exon (NM_	exon (NM_	-22282	NM_00119	17847	Usp34	A530081C0
chr7	19857252	19857425	promoter-T	promoter-T	-135	NM_00128	22323	Vasp	-
chr6	52196277	52196550	promoter-T	promoter-T	-604	NM_01045	15396	Hoxa11	Hox-1.9|Ho

chr2	76513377	76513675	5' UTR (NM	5' UTR (NM	154	NM_03125	83435	Plekha3	FAPP1
chr10	95280202	95280275	Intergenic	L1MB4|LIN	-123059	NM_00100	216238	Eea1	A430109M
chr14	52534002	52534250	promoter-T	promoter-T	-963	NM_01386	29811	Ndrg2	AI182517|/
chr7	1.25E+08	1.25E+08	intron (NM	CpG	398	NM_17066	267019	Rps15a	A630031B1
chr3	1.44E+08	1.44E+08	promoter-T	promoter-T	-428	NM_00116	16911	Lmo4	A730077C1
chr3	84284352	84284475	promoter-T	promoter-T	-52	NM_00103	229474	Fhdc1	6330505N2
chr2	1.57E+08	1.57E+08	promoter-T	promoter-T	-231	NM_00113	19650	Rbl1	AW547426
chr2	1.28E+08	1.28E+08	intron (NM	intron (NM	-11443	NM_14553	228576	Mall	BC012256|
chr13	55312452	55312600	intron (NM	CpG	1383	NM_00873	18193	Nsd1	AI528500|
chr12	1.14E+08	1.14E+08	promoter-T	promoter-T	-290	NM_17891	104759	Pld4	AI132321|
chr5	17339777	17339900	intron (NM	intron (NM	1876	NM_00115	12491	Cd36	FAT|GPIV|
chr10	87848527	87848800	intron (NM	intron (NM	6506	NM_00100	432486	Gnptab	EG432486|
chr4	1.4E+08	1.4E+08	intron (NM	intron (NM	81845	NM_00111	72754	Arhgef10l	2810441C0
chr17	34088002	34088075	TTS (NM_0	TTS (NM_0	395	NM_01942	54218	B3galt4	Gal-T2|Gal
chr2	1.81E+08	1.81E+08	intron (NM	intron (NM	433	NM_19816	229004	Gmeb2	AI839884
chr8	88078952	88079175	intron (NM	CpG	107	NM_01979	56445	Dnaja2	1500017M
chr19	6077352	6077600	promoter-T	promoter-T	-289	NM_00108	68505	Vps51	1110014N2
chr4	87101927	87102375	Intergenic	Lx7|LINE|L	-225707	NM_17242	76376	Slc24a2	2810021B1
chr5	1.23E+08	1.23E+08	Intergenic	Intergenic	-8442	NM_18330	330188	Ccdc63	4921511C1
chr3	51894202	51894375	intron (NM	intron (NM	14640	NM_00100	433586	Maml3	AV234550|
chr12	76126302	76126500	intron (NM	intron (NM	151918	NM_17280	238271	Kcnh5	Eag2
chr11	1.01E+08	1.01E+08	promoter-T	promoter-T	-363	NM_01148	20848	Stat3	1110034C0
chr6	1.15E+08	1.15E+08	intron (NM	intron (NM	2579	NM_01114	19016	Pparg	Nr1c3|PPA
chr19	41492502	41493025	Intergenic	Intergenic	-33203	NM_03137	83490	Pik3ap1	1810044J0
chr18	61927377	61927450	intron (NM	intron (NM	18903	NM_17892	106877	Afap1l1	AI173486
chr11	95685227	95685350	promoter-T	promoter-T	-526	NM_15310	237928	Phospho1	D11Moh36
chr15	5093252	5093525	promoter-T	promoter-T	-473	NM_00101	105787	Prkaa1	AI194361|/
chr10	1.17E+08	1.17E+08	3' UTR (NM	3' UTR (NM	-8543	NM_00763	12461	Cct2	Cctb
chr10	9620177	9620425	intron (NM	CpG	537	NM_00108	78808	Stxbp5	0710001E2
chr17	12870202	12870350	Intergenic	ORR1E|LTF	-1572	NM_00920	20517	Slc22a1	Lx1|Oct1|(
chr12	86451827	86451950	exon (NM_	exon (NM_	105	NM_03022	78920	Dlst	1600017E0
chr2	83483827	83483950	promoter-T	promoter-T	-847	NM_02693	69082	Zc3h15	1700006A1
chr5	1.26E+08	1.26E+08	Intergenic	Intergenic	67201	NM_01674	20778	Scarb1	AI120173|(
chr5	31828977	31829500	promoter-T	promoter-T	-103	NM_02815	72195	Supt7l	2610524B0
chr12	32339377	32339925	promoter-T	promoter-T	40	NM_02800	71916	Dus4l	2310069P0
chr4	11180777	11180850	intron (NM	intron (NM	593	NM_17811	72656	Ints8	2810013E0
chr15	8684152	8684550	Intergenic	(CAAAA)n|	-23544	NM_14893	20512	Slc1a3	AI504299|
chr17	9167677	9167975	intron (NM	intron (NM	-13372	NM_02585	66931	1700010I1	-
chr14	58042952	58043025	promoter-T	promoter-T	80	NM_00937	21821	Ift88	AW552028
chr12	33986952	33987150	promoter-T	promoter-T	-329	NM_00103	380753	Atxn7l1	2810423G0
chr10	94977802	94978000	intron (NM	CpG	105	NM_08056	93765	Ube2n	1500026J1
chr17	27694127	27694375	intron (NM	CpG-6155	653	NM_00116	111241	Hmga1-rs1	ENSMUSG0
chr16	56075002	56075475	promoter-T	promoter-T	-284	NM_02548	66315	Senp7	2410152H1
chr12	83347402	83347675	intron (NM	L1M4c|LIN	-64798	NM_17257	217692	Sipa1l1	4931426N1
chr3	60206352	60206700	Intergenic	Intergenic	-70226	NM_00125	56758	Mbnl1	Mbnl|mKIA
chr8	1.12E+08	1.12E+08	5' UTR (NM	5' UTR (NM	168	NM_00130	11765	Ap1g1	AA409002|
chr5	1.04E+08	1.04E+08	Intergenic	RMER10B|	-1636	NM_05326	114664	Hsd17b11	Dhrs8|Pan

chr19	25213852	25214100	intron (NM	intron (NM	-97716	NM_18140	107351	Kank1	A930031B(
chr4	1.03E+08	1.03E+08	intron (NM	intron (NM	-51483	NM_00116	67344	Tctex1d1	1700055O:
chr7	1.35E+08	1.35E+08	promoter-T	promoter-T	82	NM_02637	67773	Kat8	2010203C(
chr1	1.22E+08	1.22E+08	Intergenic	Intergenic	20295	NM_00103	13167	Dbi	ACBD1|Acb
chr15	5135502	5136025	5' UTR (NM	5' UTR (NM	203	NM_02621	67515	Ttc33	2410099M
chr1	53841527	53841625	intron (NM	intron (NM	483	NM_13381	98267	Stk17b	3110009A(
chr4	1.39E+08	1.39E+08	promoter-T	promoter-T	167	NM_02533	110198	Akr7a5	0610025K2
chr6	76641802	76641975	Intergenic	MT2A|LTR	-550823	NM_02888	74342	Lrrtm1	4632401D(
chr7	4866752	4866825	promoter-T	promoter-T	-31	NM_17075	232816	Zfp628	Zec|Znf628
chr15	78234627	78234725	intron (NM	intron (NM	1613	NM_00943	22117	Tst	Rhodanese
chr7	1.03E+08	1.03E+08	Intergenic	Intergenic	-25121	NM_01185	23966	Tenm4	Doc4|ELM2
chr7	73375702	73375825	Intergenic	Intergenic	121362	NM_00108	269941	Chsy1	mKIAA099(
chr13	1.09E+08	1.09E+08	intron (NM	intron (NM	7145	NM_17868	218581	Depdc1b	9830132O(
chr11	3377427	3377750	intron (NR_	intron (NR_	-10642	NM_17279	237625	Pla2g3	-
chr9	1.08E+08	1.08E+08	intron (NM	intron (NM	6585	NM_00813	14678	Gnai2	C76432|Ga
chr2	1.59E+08	1.59E+08	intron (NM	CpG	170	NM_00129	71715	Dhx35	1200009D(
chr11	1.13E+08	1.13E+08	intron (NM	intron (NM	166	NM_00116	69806	Slc39a11	1810074D2
chr13	37962452	37962725	intron (NM	intron (NM	-17715	NR_03321	68750	Rreb1	1110037N(
chr12	58545702	58545875	intron (NM	intron (NM	101320	NM_00825	15375	Foxa1	Hnf-3a|Hn
chr18	24147777	24147950	promoter-T	promoter-T	-272	NM_01175	22694	Zfp35	Zfp-35|Znf
chr4	1.33E+08	1.33E+08	promoter-T	promoter-T	-480	NM_00128	20111	Rps6ka1	Mapkapk-1
chr7	1.05E+08	1.05E+08	promoter-T	promoter-T	-79	NM_02367	12729	Clns1a	2610036D(
chr1	1.8E+08	1.8E+08	promoter-T	promoter-T	-436	NM_02662	68226	Efcab2	1700073K(
chr15	97610002	97610100	Intergenic	ORR1B1|LT	-4745	NM_02635	67739	Slc48a1	4930570C(
chr5	92751652	92751900	3' UTR (NM	3' UTR (NM	5329	NM_00859	17329	Cxcl9	BB139920|
chr11	58088177	58088350	Intergenic	Intergenic	-32308	NM_18299	103836	Zfp692	AI746306|
chr2	1.56E+08	1.56E+08	intron (NM	CpG	230	NM_17267	228829	Phf20	6820402O2
chr8	59849777	59849925	Intergenic	Intergenic	50071	NM_01040	15111	Hand2	AI225906|
chr11	90447602	90447725	intron (NM	intron (NM	51759	NM_01150	20913	Stxbp4	6030470M
chr9	20750702	20750925	intron (NM	MLT1E2|LT	6254	NM_00119	13433	Dnmt1	Cxxc9|Dnm
chr3	37352702	37353025	intron (NM	intron (NM	33661	NM_02134	57815	Spata5	2510048F2
chr5	1.08E+08	1.08E+08	intron (NM	CpG	379	NM_01698	1.01E+08	Rpl5	U21RNA
chr17	36007277	36007350	intron (NM	intron (NM	3753	NM_17524	76448	Ppp1r18	2310014H(
chr4	1.55E+08	1.55E+08	promoter-T	promoter-T	523	NM_00766	12537	Cdk11b	AA989746|
chr6	50405602	50405800	intron (NM	CpG	468	NM_00116	71720	Osbpl3	1200014M
chr13	56038302	56038800	Intergenic	Intergenic	-105765	NM_01109	18740	Pitx1	Bft|P-OTX|
chr2	92025752	92026000	intron (NM	intron (NM	236	NM_00110	192285	Phf21a	80kDa|Bhc
chr1	79758327	79758925	promoter-T	promoter-T	-282	NM_02705	69368	Wdfy1	1700013B(
chr8	23969452	23969600	promoter-T	promoter-T	-485	NM_00108	244349	Kat6a	1500036M
chr13	38296827	38296900	promoter-T	promoter-T	55	NM_02638	67797	Snrnp48	1110050F0
chr12	1.07E+08	1.07E+08	Intergenic	Intergenic	-16931	NM_00974	12062	Bdkrb2	B(2)|B2|B2
chr11	1.2E+08	1.2E+08	promoter-T	promoter-T	52	NM_00108	208092	Chmp6	2400004G(
chr16	16870952	16871025	promoter-T	promoter-T	4	NM_01162	21976	Top3b	-
chr3	1.49E+08	1.49E+08	Intergenic	CpG	-33689	NM_00108	99633	Lphn2	AI450192|
chr5	65144327	65144625	Intergenic	Intergenic	-50286	NM_00845	16599	Klf3	9930027G(
chr1	43884377	43884525	intron (NM	CpG	102	NM_02643	67883	Uxs1	1600025I1
chr14	75347152	75347250	exon (NM_	exon (NM_	483	NM_00125	380916	Lrch1	4832412D1

chr1	93294902	93295300	intron (NM	CpG	259	NM_02334	67444	Ilkap	0710007A1
chr10	79253502	79253575	Intergenic	Intergenic	-1808	NM_00104	73106	Prss57	2900092M
chr8	1.23E+08	1.23E+08	intron (NM	intron (NM	972	NM_19867	382034	Gse1	2210013I1
chr19	45088577	45088800	promoter-T	promoter-T	-978	NM_14550	226154	Lzts2	BC014695|
chr6	57641802	57642025	exon (NM_	exon (NM_	159	NM_02557	66459	Pyurf	2610022G0
chr11	61080227	61080650	exon (NM_	exon (NM_	191	NM_00743	11671	Aldh3a2	AI194803|
chr3	96048702	96048775	promoter-T	promoter-T	-642	NM_17821	319192	Hist2h2aa2	H2a-614|H
chr6	34730402	34730775	5' UTR (NM	5' UTR (NM	156	NM_00128	76223	Agbl3	2900053G1
chr17	46972502	46972700	Intergenic	CT-rich|Lov	-4239	NM_00110	210982	Gltscr1l	mKIAA024
chr4	1.01E+08	1.01E+08	intron (NM	intron (NM	109729	NM_19803	320508	Cachd1	1190007F1
chr3	40549802	40549950	intron (NM	intron (NM	341	NM_01102	18415	Hspa4l	94kDa|AI4
chr2	32391402	32391475	5' UTR (NM	5' UTR (NM	559	NM_15356	98952	Fam102a	AI426465|
chr11	77328777	77328975	exon (NM_	exon (NM_	257	NM_02618	67477	Abhd15	1300007F0
chr6	90319977	90320175	exon (NM_	exon (NM_	588	NM_03026	80292	Zxdc	A930012H2
chr4	1.34E+08	1.34E+08	intron (NM	ID_B1|SINE	6306	NM_14555	100017	Ldlrap1	AA691260|
chr1	1.35E+08	1.35E+08	exon (NM_	exon (NM_	783	NM_13381	108954	Ppp1r15b	1810033K1
chr2	69627702	69627975	promoter-T	promoter-T	44	NM_02852	73373	Phospho2	1700048E2
chr14	67689877	67691700	intron (NM	CpG	520	NM_02803	71978	Ppp2r2a	2410004D0
chr12	1.19E+08	1.19E+08	intron (NM	MLT1B|LTR	1086	NM_17593	217944	Rapgef5	4932413M
chr6	91050802	91050900	intron (NM	MER2|DNA	15969	NM_01881	54563	Nup210	9830001L1
chr3	20055452	20055625	promoter-T	promoter-T	-523	NM_01375	27357	Gyg	AU017667
chr3	89882327	89882425	intron (NM	intron (NM	-1067	NM_00125	59069	Tpm3	TM30nm|T
chr5	1.26E+08	1.26E+08	Intergenic	B1_Mur1|S	-12589	NM_01963	22190	Ubc	2700054O0
chr12	87000377	87000600	Intergenic	Intergenic	-27182	NM_01676	53314	Batf	B-ATF|SFA-
chr9	1.05E+08	1.05E+08	promoter-T	promoter-T	53	NM_05315	94062	Mrpl3	2010320L1
chr2	90936652	90936900	promoter-T	promoter-T	-178	NM_01135	20375	Spi1	Dis-1|Dis1
chr16	22656027	22656100	intron (NM	intron (NM	1241	NM_13865	110197	Dgkg	2900055E1
chr15	72897127	72897200	Intergenic	B3A|SINE|	-5529	NM_00116	76510	Trappc9	1810044A2
chr9	1.16E+08	1.16E+08	Intergenic	L3|LINE|CR	90730	NM_02957	21813	Tgfbr2	1110020H1
chr1	72367352	72367750	intron (NM	Lx8|LINE|L	13556	NM_00953	22596	Xrcc5	AI314015|
chr15	66144077	66144275	Intergenic	Intergenic	-26390	NM_15292	110862	Kcnq3	-
chr9	13553777	13554025	intron (NR_	CpG	276	NR_02402	77116	Mtmr2	6030445P1
chr1	92895052	92895125	promoter-T	promoter-T	-216	NM_00111	16978	Lrrfip1	AU024550
chr7	1.48E+08	1.48E+08	Intergenic	B1_Mus1|S	-5138	NM_13394	101613	Nlrp6	AI504961|
chr9	22247652	22247975	intron (NM	RLTR44E|L	24987	NM_01873	55934	Rp9	PAP-1|Rp9
chr14	70575127	70575450	intron (NM	intron (NM	1334	NM_00125	213019	Pdlim2	4732462F1
chr10	80363302	80363375	TTS (NM_0	TTS (NM_0	376	NM_01389	30055	Timm13	D10Ertd37
chr4	91223727	91223950	Intergenic	Intergenic	-157163	NM_20768	15569	Elavl2	Hub|mel-N
chr6	1.01E+08	1.01E+08	promoter-T	promoter-T	-350	NM_18159	72171	Shq1	2810403P1
chr1	13363902	13364025	intron (NM	CpG	201	NM_00107	17978	Ncoa2	9530095N1
chr18	37965627	37965900	promoter-T	promoter-T	-301	NM_03358	93706	Pcdhgc3	PC43|Pcdh
chr17	71890402	71890550	Intergenic	Intergenic	-10925	NM_00114	70891	Spdya	4921517J0
chr8	77559002	77559075	intron (NM	intron (NM	1453	NM_01162	21968	Tom1	-
chr10	6190652	6190950	3' UTR (NM	3' UTR (NM	-110589	NM_03118	83397	Akap12	AI317366|S
chr8	95472927	95473125	Intergenic	MTEa|LTR|	-11920	NM_00920	20538	Slc6a2	NE-T|NET|
chr17	57367502	57367775	promoter-T	promoter-T	-79	NM_00977	12266	C3	AI255234|
chr15	38008727	38008800	promoter-T	promoter-T	-154	NM_00108	70790	Ubr5	Edd|Edd1

chr4	1.25E+08	1.25E+08	intron (NM	CpG	276	NM_17524	76793	Snip1	2410133M
chr3	1.04E+08	1.04E+08	intron (NR_	intron (NR_	1410	NM_00130	15257	Hipk1	1110062KC
chr2	52826577	52826725	intron (NM	intron (NM	109749	NM_17240	71409	Fmnl2	5430425KC
chr12	1.04E+08	1.04E+08	promoter-T	promoter-T	-83	NM_17762	217835	Rin3	6430500KC
chr4	65266277	65266425	intron (NM	intron (NM	331	NM_00116	69807	Trim32	1810045E1
chr7	50800102	50800200	Intergenic	Intergenic	-11610	NM_02129	12489	Cd33	Siglec-3|gp
chr10	21920152	21920300	intron (NM	RLTR45|LT	26518	NM_00901	19370	Raet1c	RAE-1gamr
chr4	46464402	46465475	intron (NM	CpG	949	NM_13088	67628	Anp32b	2410015B1
chr17	71201102	71201175	promoter-T	promoter-T	-64	NM_00116	21815	Tgif1	AA959811|
chr1	37375027	37375150	intron (NM	intron (NM	18405	NM_00129	269180	Inpp4a	107kDa|96
chr1	1.01E+08	1.01E+08	Intergenic	Intergenic	302412	NM_02758	70866	Slco6d1	4921511I0
chr7	1.17E+08	1.17E+08	Intergenic	Intergenic	-7590	NM_02149	19347	Dennd5a	1500012B1
chr17	36116027	36116475	promoter-T	promoter-T	161	NM_14548	75210	Prr3	4930540G(
chr2	1.66E+08	1.66E+08	intron (NM	intron (NM	497	NM_00125	228880	Zmynd8	1110013E2
chr4	1.52E+08	1.52E+08	promoter-T	promoter-T	-22	NM_00127	19934	Rpl22	2700038K1
chr12	71186877	71186975	intron (NM	intron (NM	16915	NM_00108	18080	Nin	3110068G2
chr10	40058102	40058200	Intergenic	RMER15|LT	-10963	NM_00129	78334	Cdk19	2700084L0
chr19	46375827	46376025	Intergenic	MYSERV6-i	-3301	NM_00117	18034	Nfkb2	NF-kappaB
chr19	5601052	5601350	promoter-T	promoter-T	-672	NM_02661	68209	Rnaseh2c	1500026D1
chr12	1.14E+08	1.14E+08	promoter-T	promoter-T	12	NM_02802	71963	Cdca4	2410018CC
chr1	1.82E+08	1.82E+08	Intergenic	Intergenic	-38605	NM_02637	226747	Ahctf1	6230412P2
chr13	17677377	17677650	intron (NM	intron (NM	109086	NM_13865	192136	Sugct	5033411D1
chr1	72531677	72531775	intron (NM	intron (NM	51412	NM_00104	381270	4-Mar	BC056494|
chr4	43575077	43575175	promoter-T	promoter-T	329	NM_01160	21894	Tln1	Tln
chr11	48647802	48648000	intron (NM	intron (NM	8261	NM_05316	94089	Trim7	AI790312
chr6	1.23E+08	1.23E+08	Intergenic	Intergenic	-27020	NM_00117	26888	Clec4a2	Clec4a|Cle
chr8	74999852	74999950	promoter-T	promoter-T	-769	NM_13858	27967	Cherp	5730408I1
chr4	1.55E+08	1.55E+08	Intergenic	Intergenic	-22169	NM_14555	230996	9430015G1	-
chr2	57364252	57364400	Intergenic	Intergenic	273533	NM_01027	14571	Gpd2	AA408484|
chr2	1.04E+08	1.04E+08	intron (NM	CpG	733	NM_01043	15259	Hipk3	DYRK6|FIS
chr3	95498377	95498650	Intergenic	Intergenic	-6732	NM_14489	229595	Adamtsl4	Tsrc1
chr17	47448202	47448375	intron (NM	intron (NM	-57548	NM_00114	64657	Mrps10	1110038B1
chr2	25092902	25093000	promoter-T	promoter-T	-644	NM_00103	329360	Rnf224	Gm757
chr1	1.29E+08	1.29E+08	intron (NM	intron (NM	17463	NM_14512	107895	Mgat5	4930471A2
chr1	1.38E+08	1.38E+08	intron (NM	intron (NM	3773	NM_00103	16565	Kif21b	2610511N2
chr6	1.08E+08	1.08E+08	promoter-T	promoter-T	76	NM_14593	58911	Sumf1	AA543204|
chr16	19973627	19973750	intron (NM	intron (NM	9434	NM_18339	239743	Klhl6	-
chr13	1.01E+08	1.01E+08	Intergenic	LTR33B|LT	117187	NM_01373	27220	Cartpt	Cart
chr10	1.16E+08	1.16E+08	promoter-T	promoter-T	-259	NM_00103	72068	Cnot2	2600016M
chr6	48971102	48971325	Intergenic	Intergenic	-15304	NM_05311	93695	Gpnmb	DC-HIL|Dcl
chr16	4594902	4595050	intron (NM	CpG	263	NM_03118	83396	Glis2	Gli5|Klf16|
chr9	91876627	91877075	Intergenic	Intergenic	-210923	NM_00119	1.01E+08	Plscr5	331000
chr11	76220477	76220875	promoter-T	promoter-T	31	NM_01981	56322	Timm22	Tim22
chr11	4998627	4999000	promoter-T	promoter-T	278	NM_00128	14030	Ewsr1	Ews|Ewsh
chr1	1.35E+08	1.35E+08	promoter-T	promoter-T	-201	NM_00857	17248	Mdm4	4933417N(
chr6	1.29E+08	1.29E+08	promoter-T	promoter-T	-43	NM_13864	108078	Olr1	LOX-1|SR-E
chr3	89382652	89382975	intron (NM	intron (NM	58727	NM_08046	140493	Kcnn3	KCa2.3|SK3

chr15	13695602	13695725	Intergenic	Intergenic	-592269	NM_00766	12563	Cdh6	K-cadherin
chr1	39539352	39539750	Intergenic	Intergenic	-3959	NM_01877	54610	Tbc1d8	AD3|HBLP1
chr7	1.39E+08	1.39E+08	exon (NM_	exon (NM_	186	NM_02665	68277	2310057M	3110040E1
chrX	34730552	34730750	promoter-T	promoter-T	582	NM_01944	54405	Ndufa1	1810049F1
chr17	56751527	56754050	promoter-T	promoter-T	-30	NM_01873	54217	Rpl36	-
chr8	1.1E+08	1.1E+08	promoter-T	promoter-T	186	NM_13922	97484	Cog8	BB235941|
chr8	9460727	9460925	intron (NM	intron (NM	310197	NM_17344	270028	Fam155a	6430500D1
chr1	1.58E+08	1.58E+08	intron (NM	intron (NM	17297	NM_02232	240832	Tor1aip2	1110020D1
chr2	1.29E+08	1.29E+08	intron (NM	intron (NM	783	NM_00129	19261	Sirpa	AI835480|
chr1	78592427	78592650	Intergenic	Intergenic	-61862	NM_00103	74205	Acsl3	2610510B1
chr3	81736452	81736750	promoter-T	promoter-T	63	NM_17766	229445	Ctso	A330105D(
chr1	39485202	39485300	intron (NM	intron (NM	50341	NM_01877	54610	Tbc1d8	AD3|HBLP1
chr2	35992377	35992550	promoter-T	promoter-T	-239	NR_02751	67889	Rbm18	2010004P1
chr7	1.48E+08	1.48E+08	promoter-T	promoter-T	-988	NM_00117	107702	Rnh1	AW546468
chr8	1.1E+08	1.1E+08	intron (NM	intron (NM	606	NM_01081	17463	Psmd7	AW107203
chr11	51451777	51451850	Intergenic	Intergenic	-2415	NM_02534	66089	Rmnd5b	0610039K2
chr1	93334152	93334525	intron (NM	intron (NM	21567	NM_01106	18627	Per2	mKIAA034
chr5	1.37E+08	1.37E+08	Intergenic	Intergenic	-7113	NM_01196	26433	Plod3	AI414586|
chr1	1.1E+08	1.1E+08	Intergenic	Intergenic	67905	NM_00115	20725	Serpinb8	CAP-2|CAP
chr5	1.23E+08	1.23E+08	intron (NM	intron (NM	1266	NM_00100	30841	Kdm2b	Cxxc2|E43(
chr8	1.25E+08	1.25E+08	Intergenic	CpG	22314	NM_00766	12555	Cdh15	AI323380|
chr2	61368802	61368975	Intergenic	Intergenic	-47755	NM_01152	21353	Tank	C86182|E4
chr8	87514402	87514475	promoter-T	promoter-T	-56	NM_13325	170833	Hook2	A630054I0
chr11	1.17E+08	1.17E+08	intron (NM	intron (NM	20641	NM_00111	53860	9-Sep	MSF1|Msf
chr7	16638577	16638875	Intergenic	Intergenic	-18565	NM_00109	666528	Zfp541	EG666528|
chr8	63996477	63996550	intron (NM	intron (NM	29982	NM_14559	234309	Cbr4	A730083J1
chr12	1.03E+08	1.03E+08	promoter-T	promoter-T	-357	NM_02844	109181	Trip11	2610511G2
chr3	1.31E+08	1.31E+08	Intergenic	Intergenic	-94439	NM_01070	16842	Lef1	3000002B(
chr2	1.53E+08	1.53E+08	Intergenic	Intergenic	18197	NM_14553	228788	Ccm2l	-
chr8	97118327	97118525	intron (NM	intron (NM	7200	NM_02822	102122	Fam192a	1700001O1
chr15	89026702	89027050	promoter-T	promoter-T	29	NM_02708	69440	Dennd6b	1700027J0
chr7	4100852	4101225	exon (NM_	exon (NM_	436	NM_17552	243813	Leng9	9530024C2
chr12	1.13E+08	1.13E+08	Intergenic	MTD|LTR|	81344	NM_00109	668303	Kif26a	mKIAA123(
chr11	51694527	51694850	Intergenic	Intergenic	-23705	NM_19929	76901	Jade2	1200017K(
chr4	1.2E+08	1.2E+08	exon (NM_	exon (NM_	533	NM_01956	56222	Cited4	MRG-2|Mr
chr13	55546702	55547100	exon (NM_	exon (NM_	209	NM_00111	26385	Grk6	Gprk6
chr2	1.05E+08	1.05E+08	intron (NM	CpG	215	NM_00112	99003	Qser1	4732486I2
chr8	1.21E+08	1.21E+08	Intergenic	Intergenic	-239509	NM_02675	68533	Mphosph6	1110001M
chr5	1.4E+08	1.4E+08	exon (NM_	exon (NM_	286	NM_17485	231830	Micall2	A930021H1
chr11	4087077	4087150	intron (NM	CpG	277	NM_13402	103724	Tbc1d10a	AI447804|
chr1	1.46E+08	1.46E+08	Intergenic	Intergenic	-7874	NM_14599	214498	Cdc73	8430414L1
chr4	1.21E+08	1.21E+08	Intergenic	Intergenic	-7512	NM_00108	109263	Rlf	9230110M
chr5	77017977	77018200	Intergenic	CpG	-2651	NM_19903	381644	Cep135	BC062951|
chr1	1.45E+08	1.45E+08	Intergenic	Lx5|LINE|L	-590701	NM_02002	26878	B3galt2	-
chr5	1.09E+08	1.09E+08	promoter-T	promoter-T	270	NM_15356	231580	Gak	D130045N1
chr4	1.17E+08	1.17E+08	promoter-T	promoter-T	-252	NM_17524	76608	Hectd3	1700064K(
chr1	1.82E+08	1.82E+08	TTS (NM_0	TTS (NM_0	51284	NM_00108	381310	6330403A(	ENSMUSG(

chr6	91827477	91827575	promoter-T	promoter-T	-521	NM_17273	232236	Ccdc174	C130022K2
chr1	1.55E+08	1.55E+08	intron (NM	intron (NM	449	NM_00103	71836	Shcbp1l	1700012A1
chr2	1.29E+08	1.29E+08	exon (NM_	exon (NM_	230	NM_02719	69737	Ttl	2410003M
chr14	79918852	79919000	intron (NM	intron (NM	3445	NM_00128	13709	Elf1	Elf-1|Sts1|
chr2	1.31E+08	1.31E+08	promoter-T	promoter-T	-265	NM_00768	12616	Cenpb	-
chr2	13459777	13460125	intron (NM	MER3|DNA	6340	NM_01006	13434	Trdmt1	Dnmt2|Rnı
chr8	23372902	23373075	Intergenic	Lx9|LINE|L	-20925	NM_19925	234129	Tpte	Pten2
chr3	1.07E+08	1.07E+08	intron (NM	intron (NM	1040	NM_15356	229707	Strip1	6330569M
chr2	1.56E+08	1.56E+08	intron (NM	CpG	269	NM_01982	56407	Trpc4ap	4833429F0
chr12	76697477	76697575	promoter-T	promoter-T	-339	NM_01202	26932	Ppp2r5e	4633401M
chr1	13152452	13152625	intron (NM	intron (NM	-35294	NM_00108	383491	Prdm14	-
chr7	75056852	75056950	Intergenic	L1MB5|LIN	-40242	NM_01051	16001	Igf1r	A330103N2
chr10	77425702	77425825	intron (NM	L1MDa|LIN	6854	NM_13830	28240	Trpm2	9830168K1
chr11	50244352	50244525	exon (NM_	exon (NM_	175	NM_17255	216724	Rufy1	3000002E0
chr2	27294152	27294225	Intergenic	Intergenic	-11843	NM_00950	22325	Vav2	2810040F1
chr5	77584452	77584875	Intergenic	Intergenic	-40515	NM_17560	74318	Hopx	1110018K1
chr13	1.05E+08	1.05E+08	Intergenic	MTE-int|LT	-28690	NM_00108	108154	Adamts6	5031426K1
chr12	1.06E+08	1.06E+08	promoter-T	promoter-T	-801	NM_00104	212073	Syne3	nesprin-3
chr7	1E+08	1E+08	Intergenic	Intergenic	12062	NM_02142	58238	Fam181b	A830059I2
chr9	1.1E+08	1.1E+08	intron (NM	B1F1|SINE	451	NM_00869	18054	Ngp	bectenecin
chr1	1.68E+08	1.68E+08	Intergenic	Intergenic	-39922	NM_03024	27878	Tada1	2900026B1
chr7	87547302	87547375	intron (NM	CpG	310	NM_01104	18550	Furin	9130404I0
chr9	98197002	98197375	5' UTR (NM	5' UTR (NM	186	NM_14453	74080	Nmnat3	4933408N0
chr2	1.58E+08	1.58E+08	5' UTR (NM	5' UTR (NM	137	NM_00129	228850	Ralgapb	9330195P1
chr1	1.33E+08	1.33E+08	3' UTR (NM	3' UTR (NM	-7448	NM_01977	56489	Ikbke	AW558201
chr6	54631977	54632075	intron (NM	CpG	408	NM_00128	68235	Mturn	2410066E1
chr10	1.17E+08	1.17E+08	Intergenic	Intergenic	-30621	NM_02445	215449	Rap1b	2810443E1
chr16	30388527	30388800	promoter-T	promoter-T	-47	NM_00112	224088	Atp13a3	AU022875
chr12	58545477	58545625	intron (NM	intron (NM	101557	NM_00825	15375	Foxa1	Hnf-3a|Hn
chr16	17209627	17209950	exon (NM_	exon (NM_	1560	NM_00103	239731	Rimbp3	Gm1759|G
chr1	80010177	80010450	Intergenic	B3|SINE|B	-155073	NM_00925	20720	Serpine2	B230326M
chr9	1.16E+08	1.16E+08	promoter-T	promoter-T	-295	NM_00937	21813	Tgfbr2	1110020H1
chr19	10650902	10651050	promoter-T	promoter-T	611	NM_02841	72982	Tmem138	1700113I0
chr11	60597627	60597700	exon (NM_	exon (NM_	6636	NM_17549	237782	Smcr8	2310076G0
chr2	1.53E+08	1.53E+08	intron (NM	intron (NM	6689	NM_13384	99237	Tm9sf4	AA986553|
chr7	1.29E+08	1.29E+08	promoter-T	promoter-T	164	NM_02614	67417	Ears2	3230401I0
chr8	1.29E+08	1.29E+08	Intergenic	Intergenic	-2952	NM_00116	270110	Irf2bp2	E130305N2
chr8	12671827	12671975	intron (NM	CpG	199	NM_19803	259279	Tubgcp3	GCP3|Spc9
chr15	99712277	99712425	Intergenic	RLTR14|LT	-6464	NM_00111	65970	Lima1	Eplin
chr4	1.44E+08	1.44E+08	intron (NM	intron (NM	11023	NM_01130	20148	Dhrs3	Rsdr1|retS
chr5	1.15E+08	1.15E+08	intron (NM	intron (NM	1338	NM_02614	330171	Kctd10	AW536343
chr1	1.62E+08	1.62E+08	intron (NM	intron (NM	-50565	NM_00978	12301	Cacybp	SIP
chr10	77011377	77011575	exon (NM_	exon (NM_	18383	NM_00840	16414	Itgb2	2E6|AI528
chr1	1.08E+08	1.08E+08	intron (NM	CpG	1305	NM_00112	227449	Zcchc2	9930114B2
chr12	87583727	87583800	intron (NM	intron (NM	935	NM_02740	70373	Gpatch2l	1700020O0
chr3	87881477	87881550	intron (NM	B1_Mur2|S	713	NM_17252	214191	Ttc24	A430025D1
chr1	16218452	16218775	TTS (NM_0	TTS (NM_0	122650	NM_13383	98711	Rdh10	3110069K0

chr4	1.45E+08	1.45E+08	intron (NM	MIRb|SINE	1910	NM_01161	21938	Tnfrsf1b	CD120b|TN
chr6	1.34E+08	1.34E+08	Intergenic	Intergenic	-29396	NM_02577	66813	Bcl2l14	4930452K2
chr14	31440777	31441000	Intergenic	Intergenic	-1494	NM_01110	18753	Prkcd	AI385711|
chr3	1.38E+08	1.38E+08	intron (NM	intron (NM	262	NM_01193	26377	Dapp1	Bam32
chr10	12547077	12547325	intron (NM	intron (NM	34332	NM_01168	22288	Utrn	AA589569|
chr15	93425602	93426200	intron (NM	CpG	421	NM_00103	106042	Prickle1	1110058P2
chr11	86621152	86621375	promoter-T	promoter-T	-101	NM_02619	67487	Dhx40	2410016C1
chr2	84990577	84990725	Intergenic	Intergenic	14134	NM_01178	23796	Aplnr	APJ|Agtrl1
chr19	53464702	53465125	5' UTR (NM	5' UTR (NM	150	NM_17242	76479	Smndc1	2410004J2
chr19	53215527	53215600	intron (NM	intron (NM	630	NM_00116	27360	Add3	AI463285|
chr16	36260802	36260975	Intergenic	Intergenic	-16346	NM_00108	20861	Stfa1	Stefin-3|St
chr15	98711552	98711625	exon (NM_	exon (NM_	257	NM_02696	69159	Rhebl1	1810036J2
chr11	8910877	8911350	promoter-T	promoter-T	27	NM_00831	15574	Hus1	mHus1
chr2	1.69E+08	1.69E+08	promoter-T	promoter-T	-114	NM_00956	22722	Zfp64	-
chr16	34755977	34756075	Intergenic	Intergenic	-29010	NM_13930	107589	Mylk	9530072E1
chr1	64664527	64664750	promoter-T	promoter-T	-896	NM_02596	67099	Mettl21a	2310038H1
chr3	1.38E+08	1.38E+08	promoter-T	promoter-T	-84	NR_12119!	56224	Tspan5	2810455A0
chr16	11908727	11909450	intron (NR_	intron (NR_	428	NM_14606	223978	Cpped1	C530044N1
chr8	1.29E+08	1.29E+08	Intergenic	Intergenic	-112927	NM_00116	270110	Irf2bp2	E130305N2
chr18	60720202	60720475	promoter-T	promoter-T	-102	NM_02577	66810	Rbm22	8430430L2
chr16	85152802	85153100	intron (NM	URR1B|DN	21001	NM_00119	11820	App	Abeta|Abp
chr7	1.34E+08	1.34E+08	promoter-T	promoter-T	-168	NM_03056	70314	Rabep2	2610011A0
chr18	36315402	36315475	intron (NM	L2a|LINE|L	41376	NM_00116	1E+08	Nrg2	Don1|NTA
chr17	53709302	53709450	intron (NM	intron (NM	3080	NM_02000	18519	Kat2b	A930006P1
chr10	61978777	61979050	Intergenic	Intergenic	-8410	NM_01115	19073	Srgn	Prg|Prg1|S
chr3	1.04E+08	1.04E+08	intron (NM	intron (NM	2276	NM_00919	20501	Slc16a1	AL022710|
chr1	40102702	40102850	Intergenic	Intergenic	-38837	NM_01055	16178	Il1r2	CD121b|Il1
chr1	1.3E+08	1.3E+08	intron (NM	RLTR12B|L	68447	NM_02813	72160	Tmem163	2610024A0
chr2	23904102	23904175	intron (NM	intron (NM	761	NM_08046	140483	Hnmt	1500031F0
chr7	1.09E+08	1.09E+08	intron (NM	intron (NM	6069	NM_01956	56212	Rhog	2810426G0
chr1	93147252	93147325	intron (NM	CpG	392	NM_02645	67921	Ube2f	2510010F1
chr11	1.08E+08	1.08E+08	intron (NM	intron (NM	120651	NM_01110	18750	Prkca	AI875142|
chr1	1.84E+08	1.84E+08	intron (NM	intron (NM	952	NM_00785	13244	Degs1	AA536663|
chr9	1.07E+08	1.07E+08	5' UTR (NM	5' UTR (NM	459	NM_01157	21767	Tex264	TEG-264
chr2	1.7E+08	1.7E+08	Intergenic	Intergenic	-11726	NM_00115	228913	Zfp217	4933431C0
chr1	40381152	40381300	promoter-T	promoter-T	-246	NM_13319	107527	Il1rl2	AI481289|
chr13	43304202	43304300	intron (NM	intron (NM	-37381	NM_00125	67046	Tbc1d7	2610009C0
chr13	99660852	99660950	promoter-T	promoter-T	96	NM_00104	238799	Tnpo1	AU021749
chr9	1.08E+08	1.08E+08	5' UTR (NM	5' UTR (NM	133	NM_17514	69232	Qrich1	2610028H0
chr8	1.09E+08	1.09E+08	promoter-T	promoter-T	-212	NM_00922	20650	Sntb2	Snt2
chr2	24240902	24241075	promoter-T	promoter-T	71	NM_17761	215632	Psd4	BC046518|
chr13	43493952	43494025	exon (NM_	exon (NM_	243	NM_02355	70078	Nol7	2210008F1
chr8	36457277	36457425	Intergenic	Intergenic	18556	NM_17774	244416	Ppp1r3b	6430576E2
chr8	1.24E+08	1.24E+08	Intergenic	Intergenic	16353	NM_02959	76405	1700018B0	1700084O2
chr5	1.38E+08	1.38E+08	5' UTR (NM	5' UTR (NM	603	NM_14491	231803	Mepce	Bcdin3|D5'
chr18	61714977	61715125	promoter-T	promoter-T	-185	NM_14608	93687	Csnk1a1	2610208K1
chr17	35188302	35188475	intron (NM	intron (NM	1200	NM_03344	114584	Clic1	Clcp|G6

chr2	92305052	92305200	Intergenic	Intergenic	-4451	NM_14583	228368	Slc35c1	E430007K1
chr10	19796952	19797125	intron (NM	(CCA)n|Sim	-71688	NM_00863	17761	Map7	E-MAP-115
chr7	1.5E+08	1.5E+08	intron (NM	intron (NM	216	NM_01128	19935	Mrpl23	L23mrp|Rp
chr14	1.06E+08	1.06E+08	intron (NM	intron (NM	831	NM_13407	74213	Rbm26	1700009P0
chr6	90268802	90269000	intron (NM	intron (NM	6278	NM_02792	71797	Chst13	1110067M
chr10	1.11E+08	1.11E+08	Intergenic	MTD|LTR|	-5068	NM_02860	73690	Glipr1	2410114O1
chr10	84728827	84729225	Intergenic	Intergenic	-81227	NM_00777	12952	Cry1	AU020726
chr13	25034877	25035000	promoter-T	promoter-T	-83	NM_00815	14756	Gpld1	6330541J1
chr1	52289802	52289950	5' UTR (NM	5' UTR (NM	200	NM_00108	14660	Gls	6330442B1
chr3	1.45E+08	1.45E+08	intron (NM	CpG	312	NM_02571	52184	Odf2l	4733401D0
chr2	1.16E+08	1.16E+08	intron (NM	intron (NM	174319	NM_00115	17536	Meis2	A430109D2
chr13	1.02E+08	1.02E+08	intron (NM	CpG	196	NM_00987	12572	Cdk7	AI323415|A
chr1	94728777	94728975	intron (NM	CpG	613	NM_01669	14733	Gpc1	AI462976
chr1	1.08E+08	1.08E+08	Intergenic	Intergenic	-18470	NM_00112	227449	Zcchc2	9930114B2
chr4	1.41E+08	1.41E+08	promoter-T	promoter-T	-254	NM_17233	214063	Dnajc16	2900037O0
chr8	42050352	42050500	intron (NM	intron (NM	38839	NM_02684	68797	Pdgfrl	1110039P1
chr10	1.28E+08	1.28E+08	TTS (NM_0	TTS (NM_0	912	NM_02571	66701	Spryd4	4633402N2
chr7	1.29E+08	1.29E+08	promoter-T	promoter-T	176	NM_13858	28018	Ubfd1	AI467302|
chr18	46757627	46757775	promoter-T	promoter-T	343	NM_01012	13664	Eif1a	C76390|Ef
chr5	1.07E+08	1.07E+08	intron (NM	ZP3AR|Sat	3208	NM_00127	12545	Cdc7	AI597260|
chr13	74777027	74777650	promoter-T	promoter-T	18	NM_03071	80898	Erap1	Arts1|ERA
chr4	1.24E+08	1.24E+08	promoter-T	promoter-T	50	NM_01747	54170	Rragc	AU041672
chr11	1.18E+08	1.18E+08	intron (NM	intron (NM	5030	NM_00111	19157	Cyth1	CLM1|CTH
chr2	1.3E+08	1.3E+08	promoter-T	promoter-T	-347	NM_02419	67134	Nop56	2310044F1
chr10	39679802	39680150	promoter-T	promoter-T	-17	NM_00117	1E+08	G630090E1	Naglt1c
chr3	85914402	85914500	intron (NM	intron (NM	26095	NM_00108	27059	Sh3d19	AW011754
chr13	1.01E+08	1.01E+08	intron (NM	intron (NM	18476	NM_00112	17948	Naip2	Birc1b|Nai
chr7	1.28E+08	1.28E+08	intron (NM	intron (NM	979	NM_02155	59052	Mettl9	0610012D0
chr1	1.66E+08	1.66E+08	intron (NM	intron (NM	827	NM_00116	66352	Blzf1	1700030G0
chr4	58566002	58566125	5' UTR (NM	5' UTR (NM	102	NM_17298	14745	Lpar1	AI326300|
chr10	62006377	62006550	Intergenic	Intergenic	34742	NM_02819	72320	2510003E0	0710007C1
chr1	94831027	94831200	non-coding	non-coding	128	NM_01179	23830	Capn10	AW049679
chr5	1.44E+08	1.44E+08	Intergenic	Intergenic	-14360	NM_00739	11461	Actb	Actx|E4300
chr11	61757852	61758025	Intergenic	Intergenic	-12327	NM_00128	432572	Specc1	2810012G0
chr17	34259752	34259900	promoter-T	promoter-T	49	NM_01038	14998	H2-DMa	H-2Ma|H2-
chr15	98465377	98465575	5' UTR (NM	5' UTR (NM	278	NM_00128	12297	Cacnb3	Beta3|CAB
chr5	77695327	77695400	intron (NM	CpG	844	NM_01126	19712	Rest	2610008J0
chr4	63060377	63060550	intron (NM	intron (NM	4016	NM_00104	100182	Akna	AI597013|
chr1	36290002	36290400	intron (NM	intron (NM	10946	NM_19889	320011	Uggt1	0910001L1
chr6	52108477	52108625	promoter-T	promoter-T	-235	NM_01044	15394	Hoxa1	ERA1|Hox-
chr7	88488752	88490950	exon (NM_	exon (NM_	269	NM_00909	20068	Rps17	-
chr2	84568127	84568200	promoter-T	promoter-T	-738	NM_13384	98985	Clp1	AI462438|
chr2	1.44E+08	1.44E+08	Intergenic	ORR1B1|LT	-15291	NM_01977	56431	Dstn	2610043P1
chr13	41888302	41888500	intron (NM	intron (NM	54456	NM_17541	109254	Adtrp	9530008L1
chr9	21172427	21172525	promoter-T	promoter-T	161	NM_00104	16201	Ilf3	MBII-26|M
chr19	4304552	4304650	intron (NM	intron (NM	1621	NM_13086	110355	Adrbk1	Adrbk-1|Ba
chr3	96305277	96305500	Intergenic	tRNA-Glu-G	-23720	NM_02712	69585	Hfe2	2310035L1

chr2	4803377	4803650	intron (NM	CpG	903	NM_17540	109079	Sephs1	1110046B2
chr15	81756152	81756650	5' UTR (NM	5' UTR (NM	242	NM_03022	78929	Polr3h	5031409G2
chr10	79870277	79870450	promoter-T	promoter-T	-797	NM_02565	66594	Uqcr11	0710008D0
chr18	6765302	6765600	intron (NM	CpG	286	NM_00127	19330	Rab18	AA959686
chr16	84736027	84736125	promoter-T	promoter-T	-529	NM_01740	27393	Mrpl39	C21orf8|M
chr14	46277977	46278150	promoter-T	promoter-T	-245	NM_00104	114874	Ddhd1	PA-PLA1
chr3	1.44E+08	1.44E+08	Intergenic	Intergenic	92458	NM_00116	16911	Lmo4	A730077C1
chr2	1.17E+08	1.17E+08	intron (NM	CpG	238	NM_02662	68215	Fam98b	2610510H0
chr16	3717902	3718200	promoter-T	promoter-T	73	NM_00116	54483	Mefv	FMF|TRIM
chr10	59812552	59812700	intron (NM	intron (NM	3027	NM_02873	74048	4632428N0	Dies1|PD-1
chr11	20712477	20712825	Intergenic	MIRb|SINE	18460	NM_17375	216551	Lgalsl	1110067D2
chr13	80996652	80996750	Intergenic	Cheshire|D	-25982	NM_00104	105171	Arrdc3	AI450344|
chr2	68058052	68058200	intron (NM	intron (NM	115397	NM_02028	26877	B3galt1	beta3Gal-T
chr9	1.06E+08	1.06E+08	intron (NM	intron (NM	5790	NM_17292	245026	Col6a6	E330019B1
chr11	1.07E+08	1.07E+08	intron (NM	intron (NM	14832	NM_00103	18613	Pecam1	C85791|Cc
chr17	12986952	12987050	intron (NR_	intron (NR_	-24429	NM_01051	16004	Igf2r	AI661837|
chr8	1.24E+08	1.24E+08	intron (NM	intron (NM	8770	NM_08085	142682	Zcchc14	AA792890|
chr11	97145877	97146275	Intergenic	Intergenic	-4186	NM_00894	19155	Npepps	AAP-S|MP1
chr2	1.25E+08	1.25E+08	intron (NM	intron (NM	48623	NM_00799	14118	Fbn1	AI536462|
chr16	4969452	4969525	intron (NM	intron (NM	5106	NR_07337(	74684	4930451G0	-
chr3	1.49E+08	1.49E+08	Intergenic	CpG	-34477	NM_00108	99633	Lphn2	AI450192|
chr5	1.01E+08	1.01E+08	promoter-T	promoter-T	-572	NM_17240	70681	Fam175a	3830405G0
chr2	1.19E+08	1.19E+08	intron (NM	MIRb|SINE	-5752	NM_00108	383787	Ankrd63	Gm1337
chr4	1.34E+08	1.34E+08	intron (NM	intron (NM	1355	NM_01695	15331	Hmgn2	HMG-17|H
chr16	87496052	87496325	promoter-T	promoter-T	-74	NM_00984	12469	Cct8	AI132397|
chr14	52504827	52505025	exon (NM_	exon (NM_	409	NM_00102	52535	Mettl17	2310032K1
chr1	52783777	52783900	intron (NM	CpG	324	NM_17808	98682	Mfsd6	2210010L0
chr4	1.07E+08	1.07E+08	intron (NR_	CpG	874	NM_00108	16975	Lrp8	4932703M
chr4	10943702	10943975	Intergenic	Intergenic	-9072	NM_17517	71801	Plekhf2	1110070J0
chr3	1.16E+08	1.16E+08	Intergenic	Intergenic	-25290	NM_14490	229782	Slc35a3	2310050P1
chr9	69301652	69301825	intron (NM	CpG	248	NM_00758	12306	Anxa2	AW215814
chr17	47745077	47745250	intron (NM	intron (NM	3278	NM_01685	53414	Bysl	Bys|Enp1
chr14	76410352	76410875	promoter-T	promoter-T	59	NM_02681	68705	Gtf2f2	1110031C1
chr6	1.08E+08	1.08E+08	intron (NM	intron (NM	94323	NM_01058	16438	Itpr1	D6Pas2|EN
chr9	53909477	53909800	intron (NM	intron (NM	6580	NM_00103	244882	Tnfaip8l3	9930029P0
chr1	1.83E+08	1.83E+08	promoter-T	promoter-T	-369	NM_14594	208768	Sde2	-
chr19	34411252	34411525	Intergenic	L1MB5|LIN	46239	NM_00114	14102	Fas	AI196731|
chr15	78088327	78088500	intron (NM	intron (NM	13172	NM_00867	17972	Ncf4	AI451400|
chr9	41698527	41698850	Intergenic	CpG	233684	NM_01143	20660	Sorl1	2900010L1
chr1	1.33E+08	1.33E+08	Intergenic	Intergenic	-4346	NM_01054	16153	Il10	CSIF|Il-10
chr12	1.05E+08	1.05E+08	intron (NM	intron (NM	3132	NM_17302	271047	Serpina3b	6A1|A0300
chr2	1.07E+08	1.07E+08	intron (NM	intron (NM	216	NM_00102	212772	Arl14ep	2700007P2
chr9	89595827	89596050	intron (NM	intron (NM	3943	NM_02536	66111	Tmed3	1200002G1
chr14	8932377	8932500	intron (NM	intron (NM	1711	NM_17827	218699	Pxk	C230080L1
chr2	35008777	35008900	exon (NM_	exon (NM_	21084	NM_00129	26920	Cntrl	6720467O0
chr15	1.02E+08	1.02E+08	promoter-T	promoter-T	-288	NM_00117	66151	Prr13	1110020C1
chr11	1.1E+08	1.1E+08	intron (NM	intron (NM	4577	NM_01194	26399	Map2k6	MEK6|MKK

chr2	33259052	33259325	Intergenic	RSINE1|SIN	27656	NM_00108	241311	Zbtb34	Gm349|mI
chr5	1.01E+08	1.01E+08	Intergenic	Intergenic	37187	NM_02797	71883	Coq2	2310002F1
chr14	51684452	51684575	Intergenic	tRNA-Pro-C	6604	NM_20755	404319	Olfr750	GA_x5J8B7
chr2	75496427	75496525	promoter-T	promoter-T	-840	NM_14613	229279	Hnrnpa3	2410013L1
chr1	1.27E+08	1.27E+08	Intergenic	Intergenic	-27459	NM_00120	74117	Actr3	1200003AC
chr11	40547527	40547625	promoter-T	promoter-T	413	NM_00129	52653	Nudcd2	-
chr1	1.89E+08	1.89E+08	intron (NM	intron (NM	25886	NM_02636	67769	Gpatch2	5830433G2
chr4	1.52E+08	1.52E+08	intron (NR_	CpG	391	NR_10229:	19934	Rpl22	2700038K1
chr2	68636677	68636800	Intergenic	RLTR17|LT	-62876	NM_17285	241447	Cers6	4732462CC
chr17	46044452	46044575	Intergenic	Intergenic	117599	NM_00111	22339	Vegfa	Vegf|Vpf
chr2	1.82E+08	1.82E+08	Intergenic	RLTR33|LT	-60393	NM_00120	672125	Gm14496	OTTMUSG(
chr13	49393102	49393350	intron (NM	intron (NM	11351	NM_01575	30938	Fgd3	5830461L0
chr5	91182977	91183125	Intergenic	Intergenic	-5273	NM_00914	20311	Cxcl5	AMCF-II|EI
chr2	62510902	62511200	intron (NM	intron (NM	8667	NM_14552	227960	Gca	5133401E0
chr5	1.22E+08	1.22E+08	intron (NM	intron (NM	2607	NM_00965	11669	Aldh2	Ahd-5|Ahd
chr6	47870452	47870700	promoter-T	promoter-T	9	NM_00114	232784	Zfp212	Znf212|mK
chr4	1.35E+08	1.35E+08	intron (NM	CpG	31628	NM_01973	12399	Runx3	AML2|Cbfa
chr15	73448052	73448275	intron (NM	intron (NM	7011	NM_00103	106068	Slc45a4	9330175BC
chr4	8463452	8463875	intron (NM	intron (NM	872	NM_02151	59021	Rab2a	9330148M
chr2	25356277	25356600	promoter-T	promoter-T	140	NM_01390	30839	Fbxw5	AI159739|I
chr2	1.23E+08	1.23E+08	promoter-T	promoter-T	-119	NM_00116	59010	Sqrdl	0610039J1
chr8	1.25E+08	1.25E+08	TTS (NR_02	TTS (NR_02	214	NM_00130	13057	Cyba	b558|nmf3
chr2	30783077	30783150	Intergenic	Intergenic	-24296	NM_02241	64292	Ptges	2410099E2
chr15	34419502	34419775	intron (NM	intron (NM	5363	NM_00828	15473	Hrsp12	HR12|HRP:
chr6	48618177	48618250	Intergenic	Intergenic	-7921	NM_17496	317758	Gimap9	A630002K2
chr1	1.34E+08	1.34E+08	promoter-T	promoter-T	-134	NM_17529	98415	Nucks1	2700010L1
chr13	20384202	20384400	intron (NM	intron (NM	-180558	NM_19809	140580	Elmo1	6330578D2
chr11	98638277	98638350	Intergenic	Intergenic	-1622	NM_14543	217166	Nr1d1	A530070CC
chr5	1.3E+08	1.3E+08	promoter-T	promoter-T	-640	NM_01036	110006	Gusb	AI747421|(
chr14	62950827	62951000	promoter-T	promoter-T	-29	NM_02600	67153	Rnaseh2b	1110019NC
chr4	1.28E+08	1.28E+08	intron (NM	intron (NM	-119287	NM_02703	69317	Hmgb4	1700001F2
chr7	50892602	50892825	intron (NM	MLT1D|LTI	2604	NM_02823	72431	Ceacam18	2010110OC
chr14	65931202	65931525	intron (NM	intron (NM	45825	NM_01579	50759	Fbxo16	4932435C2
chr10	58699277	58699525	Intergenic	Intergenic	14731	NM_17293	268301	Sowahc	4921515AC
chr11	45768777	45768925	promoter-T	promoter-T	154	NM_00108	66628	Thg1l	-
chr8	26178577	26178725	intron (NM	intron (NM	-29217	NM_00108	330723	Htra4	B430206E1
chr12	81745402	81745475	promoter-T	promoter-T	236	NM_02624	328133	Slc39a9	2010002AC
chr12	60317277	60317400	intron (NM	intron (NM	3132	NM_00103	70611	Fbxo33	5730501N2
chr11	1.06E+08	1.06E+08	promoter-T	promoter-T	-232	NM_19829	21763	Tex2	4930568E0
chr7	25292852	25293075	intron (NR_	intron (NR_	857	NR_11095{	232969	Zfp428	2410005HC
chr19	55894852	55895025	intron (NM	intron (NM	-74873	NM_00114	21416	Tcf7l2	TCF4B|TCF
chr5	1.35E+08	1.35E+08	Intergenic	Intergenic	-3131	NR_10438:	17969	Ncf1	NCF-47K|N
chr3	27588477	27588625	intron (NM	GA-rich|Lo	20810	NM_17318	72007	Fndc3b	1600019OC
chr10	79347427	79347525	Intergenic	Intergenic	-1581	NM_01577	50701	Elane	Ela2|F430C
chr4	1.49E+08	1.49E+08	promoter-T	promoter-T	-34	NM_00128	230917	Tmem201	AV028368|
chrX	1.31E+08	1.31E+08	intron (NM	intron (NM	730	NM_01389	30058	Timm8a1	DXHXS127
chr19	15030677	15030775	Intergenic	Intergenic	-358253	NM_01160	21888	Tle4	5730411M

chr19	9016502	9016725	TTS (NM_1	TTS (NM_1	203	NM_01184	23942	Mta2	AW550797
chr1	1.62E+08	1.62E+08	intron (NM	intron (NM	-229960	NR_02811	67647	4930523C0	-
chr4	1.35E+08	1.35E+08	Intergenic	Intergenic	39603	NM_01679	51796	Srrm1	AA407769|
chr1	9788302	9788450	intron (NM	CpG	165	NM_17754	170755	Sgk3	2510015P2
chr12	56089652	56089725	Intergenic	Intergenic	-2365	NM_01381	217578	Baz1a	Acf1|B930
chr6	1.27E+08	1.27E+08	promoter-T	promoter-T	11	NM_02535	66108	Ndufa9	1010001N1
chr10	79446702	79446925	exon (NM_	exon (NM_	262	NM_00100	216157	Tmem259	AC087114.
chr6	1.17E+08	1.17E+08	Intergenic	(TGG)n|Sin	-21512	NM_17876	319776	Tmem72	C230095G0
chr11	89942177	89942450	Intergenic	Intergenic	50651	NM_02643	67888	Tmem100	1810057C1
chr3	1.3E+08	1.3E+08	promoter-T	promoter-T	-237	NM_02657	68147	Gar1	AA409823|
chr6	42662677	42663025	Intergenic	Intergenic	-19793	NM_02993	77574	Fam115a	2810407D0
chr11	75545927	75547400	intron (NM	CpG	274	NM_00953	22627	Ywhae	AU019196
chr9	55938052	55938225	5' UTR (NM	5' UTR (NM	355	NM_01119	19200	Pstpip1	CD2BP1|de
chr6	47762502	47762625	intron (NM	intron (NM	948	NM_00978	12304	Pdia4	AI987846|
chr2	27940427	27940625	promoter-T	promoter-T	-128	NM_01019	14134	Fcnb	Fcn2
chr3	1.21E+08	1.21E+08	Intergenic	Intergenic	-1467	NM_01017	14066	F3	AA409063|
chr7	3644127	3644225	intron (NM	CpG	951	NM_02993	77582	Mboat7	5730589L0
chr19	57435602	57435700	promoter-T	promoter-T	152	NM_14550	226252	Fam160b1	AI450540|
chr17	64213702	64213825	intron (NM	CpG	433	NM_00103	14158	Fert2	AV082135|
chr16	28894927	28895075	intron (NM	intron (NM	34783	NM_17771	239796	Mb21d2	1600021P1
chr6	53573902	53574075	intron (NM	intron (NM	50620	NM_17272	231991	Creb5	Crebpa|D4
chr4	41261377	41261575	intron (NM	CpG	458	NM_02689	68970	Dcaf12	1500001L2
chr10	1.07E+08	1.07E+08	intron (NM	intron (NM	39427	NM_00117	628870	Otogl	EG628870|
chr2	59603802	59604075	intron (NM	Charlie1a|I	116725	NM_00115	72137	Wdsub1	1700048E1
chr8	1.25E+08	1.25E+08	promoter-T	promoter-T	-277	NM_02601	67177	Cdt1	2610318F1
chr2	62236177	62236475	intron (NM	intron (NM	13962	NM_01007	13482	Dpp4	Cd26|Dpp-
chr10	1.28E+08	1.28E+08	Intergenic	Intergenic	-8489	NM_01027	14561	Gdf11	BMP-11|Br
chr16	17619577	17619675	intron (NM	CpG	179	NM_00116	77626	Smpd4	4122402O2
chr1	1.83E+08	1.83E+08	intron (NM	intron (NM	1527	NM_14479	208795	Tmem63a	BC014795|
chr6	1.38E+08	1.38E+08	exon (NM_	exon (NM_	323	NM_01149	20901	Strap	AW557906
chr15	68089877	68090425	intron (NM	CpG	267	NM_00114	380993	Zfat	Gm922|Zfa
chr1	58659977	58660350	Intergenic	Intergenic	16720	NM_02559	66495	Ndufb3	2700033I1
chr8	87230777	87230850	3' UTR (NM	3' UTR (NM	5457	NM_00853	17095	Lyl1	Lyl-1|bHLH
chr2	1.67E+08	1.67E+08	promoter-T	promoter-T	-54	NM_02381	76367	Trp53rk	2810408M
chr1	1.71E+08	1.71E+08	promoter-T	promoter-T	-131	NM_02328	66977	Nuf2	2410003C0
chr8	74000677	74000800	TTS (NM_0	TTS (NM_0	7645	NR_12211	66498	Dda1	1500034J0
chr2	1.19E+08	1.19E+08	exon (NM_	exon (NM_	8248	NM_17226	228545	Vps18	9930024E1
chr1	1.57E+08	1.57E+08	Intergenic	Intergenic	-60213	NM_00978	12290	Cacna1e	A430040I1
chr7	1.4E+08	1.4E+08	Intergenic	Intergenic	-4688	NM_02960	76429	Lhpp	2310007H0
chr10	94662277	94662500	intron (NM	intron (NM	124343	NM_00995	12905	Cradd	RAIDD
chr18	15733427	15733500	intron (NM	intron (NM	143092	NM_19905	71367	Chst9	5430438D0
chr4	1.29E+08	1.29E+08	Intergenic	Intergenic	-9780	NM_01689	11637	Ak2	Ak-2|D4Ert
chr8	1.22E+08	1.22E+08	Intergenic	Intergenic	-11661	NM_02795	71839	Osgin1	1700012B1
chr12	3832902	3832975	intron (NM	intron (NM	25778	NM_00127	13435	Dnmt3a	MmuIIIA
chr9	1.23E+08	1.23E+08	3' UTR (NM	3' UTR (NM	-16768	NM_01386	29806	Limd1	AW822033
chrX	1.38E+08	1.38E+08	TTS (NM_0	TTS (NM_0	108345	NM_17487	666468	Atg4a	AI627006|
chr10	93352502	93352575	5' UTR (NM	5' UTR (NM	1409	NM_01964	56307	Metap2	4930584B2

chr2	1.68E+08	1.68E+08	promoter-T	promoter-T	-47	NM_01007	13480	Dpm1	AI118379|/
chr13	16023577	16023775	Intergenic	Intergenic	-82632	NM_00838	16323	Inhba	-
chr12	4937627	4937925	intron (NM	intron (NM	13617	NM_00109	320817	Atad2b	1110014E1
chr6	87621877	87622000	exon (NM_	exon (NM_	224	NM_02425	72103	Aplf	2010301N0
chr11	74390552	74390900	intron (NM	intron (NM	12934	NM_00101	380711	Rap1gap2	AU067654
chr7	20085752	20085975	Intergenic	Intergenic	7817	NM_17769	232946	Bloc1s3	BC043666|
chrX	91368127	91368950	5' UTR (NM	5' UTR (NM	208	NM_01176	22764	Zfx	Zfx5|Zfx5,6
chr7	88005302	88005525	Intergenic	Intergenic	-1581	NM_00955	22691	Zscan2	Zfp-29|Zfp
chr18	56591677	56591850	promoter-T	promoter-T	-23	NM_02624	107022	Gramd3	9030613F0
chr6	88018827	88018900	Intergenic	L1MB2|LIN	-15604	NM_13393	103963	Rpn1	AU018702
chr16	10758727	10758900	Intergenic	Intergenic	26816	NM_00989	12703	Socs1	Cish1|Cish1
chr1	64348477	64348550	Intergenic	Intergenic	-180550	NM_03356	93691	Klf7	9830124P0
chr5	54500552	54500650	intron (NM	intron (NM	110839	NM_00108	116873	Stim2	-
chr9	1.2E+08	1.2E+08	intron (NM	intron (NM	-76336	NM_02689	68969	Eif1b	1500010M
chr3	67234277	67234425	intron (NM	CpG	314	NM_13859	28030	Gfm1	AW545374
chr3	1.16E+08	1.16E+08	Intergenic	Intergenic	-12763	NM_00117	229776	Cdc14a	A830059A1
chr11	18926702	18926825	Intergenic	Intergenic	-7791	NM_01078	17268	Meis1	C530044H1
chr12	83209177	83209300	Intergenic	Intergenic	-61765	NM_00116	217692	Sipa1l1	4931426N1
chr3	1.52E+08	1.52E+08	Intergenic	Intergenic	-3321	NM_00896	19220	Ptgfr	AI957154|I
chr10	94879202	94879375	promoter-T	promoter-T	203	NM_00770	216233	Socs2	8030460M
chr9	50643202	50643375	intron (NR_	intron (NR_	-21741	NM_00103	73699	Ppp2r1b	2410091N0
chr15	81640977	81641100	promoter-T	promoter-T	-806	NM_01737	21685	Tef	2310028D2
chr4	1.09E+08	1.09E+08	Intergenic	Intergenic	-5386	NM_13388	100273	Osbpl9	2600011I0
chr2	1.67E+08	1.67E+08	Intergenic	RMER5|LTI	-9510	NM_01983	56336	B4galt5	9430078I0
chr7	53423477	53423550	intron (NM	MTEb|LTR	11890	NM_01151	20927	Abcc8	D930031B2
chr9	50287152	50287225	Intergenic	Intergenic	-15442	NM_02963	76509	Plet1	0610037B2
chr5	1.35E+08	1.35E+08	intron (NM	intron (NM	19728	NM_00103	269713	Clip2	B230327O2
chr5	1.51E+08	1.51E+08	Intergenic	Intergenic	98038	NM_08046	140498	Rxfp2	Gpr106|Gr
chr8	1.22E+08	1.22E+08	intron (NM	intron (NM	27766	NM_02807	72042	Cotl1	1810074P2
chr15	54921727	54921950	exon (NM_	exon (NM_	195	NM_18308	72107	Dscc1	2010006I0
chr7	1.46E+08	1.46E+08	Intergenic	Intergenic	35357	NM_00109	101631	Pwwp2b	AI594893|I
chr8	37677352	37677450	promoter-T	promoter-T	-404	NM_01580	50768	Dlc1	A730069N0
chr9	55314602	55314800	intron (NM	intron (NM	45349	NM_14561	110842	Etfa	2010200I2
chr12	78208327	78208525	Intergenic	Intergenic	-130665	NM_00125	53618	Fut8	-
chr5	1.24E+08	1.24E+08	intron (NM	intron (NM	7718	NM_17787	330192	Vps37b	2300007F2
chr6	67019202	67019500	intron (NR_	Lx5|LINE|L	-31950	NM_00783	13197	Gadd45a	AA545191|
chr9	50469602	50469875	Intergenic	Intergenic	-1853	NM_14561	235339	Dlat	6332404G0
chr1	1.84E+08	1.84E+08	promoter-T	promoter-T	-176	NM_17512	67948	Fbxo28	4833428J1
chr5	1.31E+08	1.31E+08	intron (NM	CpG	283	NM_02785	71667	Tmem248	0610007L0
chr5	68237502	68237625	intron (NM	intron (NM	1107	NM_00128	11980	Atp8a1	AI481521|/
chr2	28980152	28980225	promoter-T	promoter-T	-324	NM_19803	269254	Setx	A130090N0
chr8	74195202	74195375	intron (NM	CpG	368	NM_00102	382018	Unc13a	2410078G0
chr9	20532177	20532300	intron (NM	CpG	420	NM_17377	244723	Olfm2	A030009A0
chr15	8747952	8748175	Intergenic	L1MA6|LIN	-87256	NM_14893	20512	Slc1a3	AI504299|I
chr2	72039752	72039850	intron (NM	intron (NM	-83893	NM_02305	65964	Zak	AV006891|
chr5	1.14E+08	1.14E+08	promoter-T	promoter-T	-303	NM_00101	231630	Ficd	D5Ertd40e
chr9	21706027	21706150	Intergenic	LTR33A|LT	16477	NM_03187	19340	Rab3d	C130057E1

chr15	1.01E+08	1.01E+08	intron (NM	intron (NM	50399	NM_01132	20273	Scn8a	AI853486|(
chr10	41170352	41170475	intron (NM	CpG	249	NR_10236(	268294	Zbtb24	BC055367|
chr1	1.65E+08	1.65E+08	intron (NM	CpG	324	NM_00108	226562	Prrc2c	1810043M
chr11	86923427	86923550	intron (NM	intron (NM	725	NM_02537	66140	Ska2	1110001A(
chr1	64173252	64174200	Intergenic	Intergenic	-5763	NM_03356	93691	Klf7	9830124P(
chr1	87637627	87637850	Intergenic	Intergenic	-4557	NM_17705	319997	A630001G:	-
chr6	1.25E+08	1.25E+08	Intergenic	Intergenic	-12508	NM_01353	14794	Spsb2	AI461677|(
chr2	1.65E+08	1.65E+08	intron (NM	intron (NM	1574	NM_17837	67538	Zswim3	4921517A(
chr15	80504302	80504375	intron (NM	intron (NM	2061	NM_14598	213956	Fam83f	AW544981
chr9	96532152	96532375	promoter-T	promoter-T	-341	NM_05326	114713	Rasa2	5430433H2
chr1	4797927	4798175	promoter-T	promoter-T	77	NM_00886	18777	Lypla1	Pla1a
chr1	1.73E+08	1.73E+08	exon (NM_	exon (NM_	195	NM_02317	80915	Dusp12	1190004O1
chr7	35175902	35176050	Intergenic	L2a|LINE|L	-1417	NM_02594	67070	Lsm14a	2700023B1
chr8	12514327	12514475	Intergenic	Intergenic	-58648	NM_02427	78634	Spaca7	1700094C(
chr8	32279377	32279775	promoter-T	promoter-T	-380	NM_02645	67920	Mak16	2600016B(
chr5	98460052	98460150	promoter-T	promoter-T	-120	NM_13373	71914	Antxr2	2310046B1
chr3	1.01E+08	1.01E+08	Intergenic	Intergenic	-1258	NM_00116	630146	Cd101	Gm1016|G
chr4	3948252	3948325	Intergenic	Intergenic	-1478	NM_18198	242285	Sdr16c5	Rdhe2|Scd
chr4	97010152	97010350	Intergenic	Intergenic	-238383	NM_00112	18027	Nfia	1110047K1
chr11	68829602	68829750	intron (NM	intron (NM	263	NM_00101	68964	Ctc1	1500010J0
chr11	77029827	77029900	promoter-T	promoter-T	-64	NM_17771	237860	Ssh2	SSH-2|SSH-
chr3	1.26E+08	1.26E+08	intron (NM	Lx6|LINE|L	15844	NM_02381	108058	Camk2d	2810011D2
chr4	24661102	24661175	intron (NM	intron (NM	117095	NM_00116	212390	Klhl32	6430524H(
chr10	39112377	39112450	intron (NM	intron (NM	22808	NM_00112	14360	Fyn	AI448320|/
chr3	95454852	95454925	Intergenic	Intergenic	-7755	NM_00856	17210	Mcl1	AW556805
chr8	74844152	74844225	intron (NM	PB1D7|SIN	1227	NM_00845	16598	Klf2	Lklf
chr3	1.15E+08	1.15E+08	Intergenic	Intergenic	-45383	NM_15345	229759	Olfm3	B230206G(
chr1	5073102	5073175	promoter-T	promoter-T	-116	NM_13382	108664	Atp6v1h	0710001F1
chr6	86321602	86321675	intron (NM	CpG	104	NM_02650	68011	Snrpg	2810024K1
chr7	28114602	28114700	exon (NM_	exon (NM_	4016	NM_00111	108075	Ltbp4	2310046A1
chr17	34067152	34067275	TTS (NM_0	TTS (NM_0	374	NM_00905	19732	Rgl2	KE1.5|Rab:
chr3	1.16E+08	1.16E+08	intron (NM	intron (NM	5730	NM_01169	22329	Vcam1	CD106|Vca
chr11	78311427	78311500	promoter-T	promoter-T	-207	NM_17279	237868	Sarm1	A830091I1
chr8	87225027	87225175	promoter-T	promoter-T	-255	NM_00853	17095	Lyl1	Lyl-1|bHLH
chr8	92186302	92186600	Intergenic	Intergenic	-618390	NM_02139	58198	Sall1	Msal-3
chr17	44376952	44377025	intron (NM	intron (NM	51467	NM_17262	224796	Clic5	5730531E1
chr13	95055502	95055675	promoter-T	promoter-T	-352	NM_02915	107767	Scamp1	4930505M
chr4	1.09E+08	1.09E+08	Intergenic	Intergenic	8889	NM_00767	12580	Cdkn2c	C77269|IN
chr19	53268577	53269050	intron (NM	intron (NM	50326	NM_01375	27360	Add3	AI463285|I
chr3	27576552	27576750	intron (NM	intron (NM	32710	NM_17318	72007	Fndc3b	1600019O(
chr4	1.41E+08	1.41E+08	intron (NM	CpG	473	NR_10234	213491	Szrd1	1110022I0
chr2	31940127	31940325	Intergenic	CpG	-1001	NM_17551	241303	Fam78a	A130092J0
chr6	47762952	47763100	intron (NM	intron (NM	485	NM_00978	12304	Pdia4	AI987846|(
chr4	1.51E+08	1.51E+08	exon (NM_	exon (NM_	426	NM_00103	242785	Klhl21	1810045K(
chr14	55483327	55483425	promoter-T	promoter-T	-381	NM_00117	239099	Homez	mKIAA144:
chrX	1.49E+08	1.49E+08	Intergenic	Intergenic	-24151	NM_00119	635396	Gm7157	-
chr18	24762027	24762250	promoter-T	promoter-T	180	NM_13914	106957	Slc39a6	Ermelin

chr3	65761077	65761375	intron (NM	intron (NM	921	NM_01993	56706	Ccnl1	2610030E2
chr1	58773127	58773275	intron (NM	intron (NM	3071	NM_20765	12633	Cflar	2310024N1
chr7	20300502	20300650	exon (NM_	exon (NM_	202	NM_00110	53333	Tomm40	AW539759
chr5	1.06E+08	1.06E+08	promoter-T	promoter-T	0	NM_00112	231549	Lrrc8d	2810473G0
chr9	59334202	59334350	promoter-T	promoter-T	-95	NM_01992	23806	Arih1	AU021774
chr15	1.02E+08	1.02E+08	intron (NM	CpG	226	NM_13410	106073	Mfsd5	AA409375|
chr2	69700227	69700300	intron (NM	intron (NM	644	NM_00927	20823	Ssb	SS-B
chr10	79724352	79724575	promoter-T	promoter-T	-750	NM_01025	14431	Gamt	AA571402|
chr2	3575002	3575350	Intergenic	Intergenic	-55554	NM_02562	66540	Fam107b	3110001A1
chr4	46178777	46178875	intron (NM	intron (NM	27443	NM_00103	433702	Ncbp1	AU014645
chr14	14845202	14845650	intron (NM	CpG	421	NM_13922	246103	Atxn7	A430107N1
chr13	99798577	99798725	Intergenic	ORR1D1|LT	-44131	NM_02706	69382	1700024P0	H2bl1|Sub
chr7	1.23E+08	1.23E+08	Intergenic	(CA)n|Simp	-32533	NM_17274	233765	Plekha7	A430081P2
chr15	88692077	88692400	promoter-T	promoter-T	-386	NM_14547	223775	Pim3	BC026639|
chr12	77478277	77478425	intron (NM	intron (NM	7098	NM_17874	268564	Zbtb1	9430077A1
chr4	1.02E+08	1.02E+08	intron (NM	intron (NM	-56964	NM_00117	18578	Pde4b	Dpde4|R74
chr1	62771702	62772050	intron (NM	intron (NM	21985	NM_00107	18187	Nrp2	1110048P0
chr7	1.05E+08	1.05E+08	promoter-T	promoter-T	-489	NM_02540	66190	Acer3	1110057L1
chrX	72759727	72759825	intron (NM	intron (NM	140	NM_01169	22327	Vbp1	-
chr1	59628402	59628650	intron (NM	intron (NM	55418	NM_00101	381260	Gm973	AW554518
chrY	2862577	2862750	Intergenic	Intergenic	475405	NM_00127	1E+08	Gm3376	Rbmy1b
chr4	70207002	70207500	Intergenic	RMER15-in	-11289	NM_17269	230316	Megf9	4933405H1
chr2	24173502	24173725	Intergenic	Intergenic	-18767	NM_03116	16181	Il1rn	F630041P1
chr3	1.31E+08	1.31E+08	5' UTR (NM	5' UTR (NM	119	NM_01186	23971	Papss1	AI325286|A
chr1	1.87E+08	1.87E+08	Intergenic	Intergenic	-50023	NM_14551	226778	Mark1	AW491150
chr11	32442152	32442300	intron (NM	intron (NM	8960	NM_00928	20868	Stk10	Gek1|Lok|
chr7	20189127	20189350	intron (NM	intron (NM	579	NM_01668	53609	Clasrp	Clasp|Sfrs1
chr19	10956152	10956625	promoter-T	promoter-T	-155	NM_13414	68539	Tmem109	1110006I15
chr14	58505852	58505950	intron (NM	ID4_|SINE|	3198	NM_02949	75965	Zdhhc20	5033406L1
chr2	1.29E+08	1.29E+08	intron (NM	CpG	293	NM_00856	17222	Anapc1	2610021O0
chr4	45421227	45421425	intron (NM	CpG	312	NM_00100	230125	Slc25a51	9130208E0
chr8	86266227	86266525	Intergenic	Intergenic	-1166	NM_00116	26364	Cd97	AA409984|
chr11	82804727	82804825	intron (NM	intron (NM	556	NM_17279	237886	Slfn9	9830137M
chr17	8072552	8072775	Intergenic	Intergenic	-46202	NM_14596	72536	Tagap	2610315E1
chr2	72392927	72393075	Intergenic	Intergenic	78725	NM_02586	66953	Cdca7	2310021G0
chr5	1.14E+08	1.14E+08	promoter-T	promoter-T	-283	NM_02552	66383	Iscu	2310020H2
chr12	76919402	76919675	intron (NM	CpG	233	NM_00100	319565	Syne2	6820443O0
chr13	96152502	96152750	Intergenic	Intergenic	-7314	NM_02563	66549	Aggf1	2010009L1
chr13	32925302	32925425	Intergenic	Intergenic	17691	NM_02542	66222	Serpinb1a	1190005M
chr7	90014402	90014525	Intergenic	CpG	-1380	NM_17536	108797	Mex3b	4931439A0
chr9	65908177	65908250	intron (NM	CpG	237	NM_01114	19035	Ppib	AA408962|
chr15	27560227	27560350	exon (NM_	exon (NM_	160	NM_00101	432940	Otulin	C79097|Fa
chr1	82313177	82313625	intron (NM	CpG	247	NM_02977	76867	Rhbdd1	4930418P0
chr18	57195452	57195825	Intergenic	L1MC1|LIN	-60605	NM_02990	77422	C330018D2	AU018666
chr10	75931352	75931950	promoter-T	promoter-T	-65	NM_01943	54387	Mcm3ap	GANP|mKI
chr5	1.44E+08	1.44E+08	promoter-T	promoter-T	178	NM_02132	57782	Rbak	-
chr6	39726252	39726700	Intergenic	L1MA9|LIN	34459	NM_01027	14548	Mrps33	AI841153|(

chr16	23518927	23519200	intron (NM	intron (NM	1600	NM_00855	17174	Masp1	AW048060
chr9	56978252	56978450	promoter-T	promoter-T	-233	NM_02863	73744	Man2c1	1110025H2
chr19	4191477	4191700	promoter-T	promoter-T	-541	NM_17865	108995	Tbc1d10c	1810062O1
chr11	1.19E+08	1.19E+08	intron (NM	CpG	256	NM_13866	192170	Eif4a3	2400003O0
chr13	41562177	41562525	intron (NM	intron (NM	20378	NM_00111	18003	Nedd9	Cas-L|CasL
chr9	1.2E+08	1.2E+08	Intergenic	ORR1E|LTF	-52828	NM_14455	245049	Myrip	A230081N1
chr15	81229752	81229825	promoter-T	promoter-T	336	NM_13372	70356	St13	1110007I0
chr2	30141577	30141675	promoter-T	promoter-T	248	NM_17764	227697	Dolk	BC026973|
chr7	1.06E+08	1.06E+08	Intergenic	Intergenic	-18609	NM_00128	22411	Wnt11	-
chr10	42955702	42955825	intron (NM	intron (NM	14471	NM_00116	71365	Pdss2	5430420P0
chr7	26212177	26212425	intron (NR_	intron (NR_	-14396	NM_15357	232983	Cxcl17	VCC-1|Vcc1
chr1	1.37E+08	1.37E+08	intron (NM	intron (NM	1598	NM_14541	215615	Rnpep	-
chr6	1.16E+08	1.16E+08	Intergenic	ID_B1|SINE	-18765	NM_02637	67784	Plxnd1	6230425C2
chr1	1.53E+08	1.53E+08	intron (NM	CpG	127	NM_01127	19821	Rnf2	AI326319|A
chr4	1.29E+08	1.29E+08	promoter-T	promoter-T	-69	NM_19930	230770	Tmem39b	6330509E0
chr6	53058277	53058475	Intergenic	MurERV4-i	-39758	NM_17340	231986	Jazf1	AI591476|(
chr16	38376402	38376850	intron (NM	intron (NM	14330	NM_02231	64082	Popdc2	AV006127|
chr4	1.23E+08	1.23E+08	intron (NM	intron (NM	-3628	NM_18185	353371	Oxct2b	Scot-t2
chr3	1.58E+08	1.58E+08	exon (NM_	exon (NM_	292	NM_00109	69207	Srsf11	0610009J0
chr9	1.03E+08	1.03E+08	promoter-T	promoter-T	-392	NM_00113	321022	Cdv3	2510010F1
chr11	57917752	57917875	intron (NM	CpG	158	NM_02694	69125	Cnot8	1500015I0
chr19	12085027	12085100	Intergenic	Intergenic	26167	NM_14668	258675	Olfr1423	MOR239-3
chr16	57606702	57606875	exon (NM_	exon (NM_	192	NM_02559	66497	Cmss1	1110001A0
chr4	1.29E+08	1.29E+08	intron (NM	CpG	210	NM_00846	16650	Kpna6	IPOA7|Kpn
chr15	1E+08	1E+08	promoter-T	promoter-T	-622	NM_02733	70152	Mettl7a1	2210414H1
chr16	35932427	35932550	exon (NM_	exon (NM_	-6068	NM_03025	80285	Parp9	ARTD9|AW
chr10	1.27E+08	1.27E+08	Intergenic	MTD|LTR|I	-15322	NM_00117	216440	Os9	4632413K1
chr3	27689977	27690175	Intergenic	Intergenic	-80715	NM_17318	72007	Fndc3b	1600019O0
chr1	63873052	63873200	Intergenic	Intergenic	-57285	NM_00125	74426	4933402D2	-
chr7	1.34E+08	1.34E+08	Intergenic	L3|LINE|CF	-13139	NM_01195	26417	Mapk3	Erk-1|Erk1
chr19	47605777	47605850	intron (NM	intron (NM	5697	NM_17536	108689	Obfc1	0610009H2
chr17	66868877	66869025	promoter-T	promoter-T	59	NM_02444	19328	Rab12	2900054P1
chr1	51641727	51641875	Intergenic	LTR82B|Lo	-106558	NM_02869	109019	Nabp1	4930434H0
chr7	16881902	16882000	Intergenic	Intergenic	-9807	NM_00113	243846	Ccdc9	2600011L0
chr10	79565052	79565175	intron (NM	CpG	334	NM_18342	216161	Sbno2	BC019206|
chr2	1.65E+08	1.65E+08	Intergenic	Intergenic	-4931	NM_01359	17395	Mmp9	AW743869
chr1	53397902	53398100	Intergenic	Intergenic	11595	NM_00129	70396	Asnsd1	2210409M
chr1	1.83E+08	1.83E+08	promoter-T	promoter-T	44	NM_14594	208768	Sde2	-
chr4	1.55E+08	1.55E+08	Intergenic	Intergenic	-12532	NM_01138	20481	Ski	2310012I0
chr13	74660377	74660525	Intergenic	Intergenic	-29053	NM_14623	235956	Zfp825	-
chr11	54615502	54615750	Intergenic	Intergenic	-14421	NM_17862	72729	Cdc42se2	2810404F1
chr6	1.49E+08	1.49E+08	Intergenic	Intergenic	-55629	NM_00111	232566	Amn1	5830467E0
chr1	1.67E+08	1.67E+08	promoter-T	promoter-T	-201	NM_02743	70456	Mpc2	0610006C0
chr5	91326377	91326450	Intergenic	Intergenic	6142	NM_00817	14825	Cxcl1	Fsp|Gro1|I
chr6	1.17E+08	1.17E+08	promoter-T	promoter-T	-68	NM_02605	67255	Zfp422	2900028O2
chr8	1.14E+08	1.14E+08	intron (NM	CpG	208	NM_01174	22640	Zfp1	Fnp-1|Zfp-
chr8	1.27E+08	1.27E+08	intron (NM	intron (NM	33857	NM_19863	330863	Trim67	D130049O1

chr2	1.67E+08	1.67E+08	Intergenic	Intergenic	42754	NM_02323	66589	Ube2v1	0610011J0
chr2	38920777	38921125	TTS (NM_1	TTS (NM_1	110	NR_11096	76899	Golga1	0710001G(
chr9	85368877	85369075	Intergenic	Intergenic	-148219	NM_00116	212943	Fam46a	D930050G(
chr1	1.93E+08	1.93E+08	intron (NM	intron (NM	832	NM_02586	66950	Tmem206	2310028N(
chr7	13420352	13420550	5' UTR (NM	5' UTR (NM	292	NM_02298	65020	Zfp110	2900024E0
chr5	73647427	73647675	intron (NM	CpG	306	NM_02819	72313	Fryl	2010313D2
chr16	5050352	5050525	promoter-T	promoter-T	277	NM_02666	170644	Ubn1	1110029L1
chr17	34574127	34574400	intron (NM	intron (NM	38498	NM_00116	407788	BC051142	NG8|TSBP
chr2	1.31E+08	1.31E+08	intron (NM	intron (NM	26531	NM_00973	11990	Atrn	AW558010
chr2	1.34E+08	1.34E+08	Intergenic	RLTR45|LT	322856	NM_00755	12156	Bmp2	AI467020|
chr12	8680677	8680925	promoter-T	promoter-T	-139	NM_00116	80913	Pum2	5730503J2
chr11	1.09E+08	1.09E+08	intron (NM	intron (NM	-21978	NM_00102	104681	Slc16a6	AW743111
chr12	85189677	85189875	intron (NR_	intron (NR_	-6468	NM_01094	18222	Numb	Nb
chr16	43979777	43980125	promoter-T	promoter-T	-788	NM_00100	332175	Zdhhc23	Gm1751|G
chr10	75322677	75322750	intron (NM	CpG-1163	282	NM_01079	17319	Mif	GIF|Glif
chr6	28347077	28347200	Intergenic	Intergenic	24586	NM_02890	74375	Gcc1	4932417P0
chr8	80327102	80327225	Intergenic	Intergenic	-78812	NM_01033	13617	Ednra	ET-AR|ETa
chr19	40662402	40662500	intron (NM	intron (NM	502	NM_01969	56454	Aldh18a1	2810433K0
chr19	34273352	34273650	intron (NM	intron (NM	6741	NM_02968	76630	Stambpl1	1700095N2
chr2	1.32E+08	1.32E+08	intron (NM	intron (NM	49906	NM_01882	54338	Slc23a2	AI844736|
chr11	1.16E+08	1.16E+08	intron (NM	intron (NM	828	NM_00116	217344	Rhbdf2	4732465I1
chr3	1.29E+08	1.29E+08	intron (NM	intron (NM	36779	NM_00793	13809	Enpep	6030431M
chr2	1.32E+08	1.32E+08	Intergenic	Intergenic	-35694	NM_01882	54338	Slc23a2	AI844736|
chr10	1.21E+08	1.21E+08	Intergenic	L1MB7|LIN	-34105	NM_17302	270802	BC048403	E030027L1
chr7	71134902	71135150	Intergenic	MTC|LTR|	-51225	NM_02136	50794	Klf13	0610043C1
chr4	1.25E+08	1.25E+08	Intergenic	Intergenic	-137926	NM_15315	230738	Zc3h12a	BC036563|
chr2	90938302	90938575	intron (NM	intron (NM	1484	NM_01135	20375	Spi1	Dis-1|Dis1
chr1	1.33E+08	1.33E+08	3' UTR (NM	3' UTR (NM	25192	NM_01070	16865	Eif2d	D1Ertd5e|
chr9	66222777	66222900	intron (NM	intron (NM	24581	NM_14561	235439	Herc1	2810449H1
chr13	1.03E+08	1.03E+08	Intergenic	Lx8|LINE|L	-248516	NM_00107	18708	Pik3r1	PI3K|p50al
chr2	48804377	48804675	promoter-T	promoter-T	261	NM_00117	26428	Orc4	Orc4P|Orc
chr1	90072627	90072875	intron (NM	RMER16-in	10164	NM_20164	394433	Ugt1a5	-
chr6	53427652	53427775	Intergenic	Intergenic	-95655	NM_17272	231991	Creb5	Crebpa|D4
chr6	4852727	4853025	promoter-T	promoter-T	-444	NM_18159	243725	Ppp1r9a	2810430P2
chr1	1.69E+08	1.69E+08	intron (NM	intron (NM	37995	NM_03072	80914	Uck2	AA407809|
chr8	72876577	72876650	intron (NM	intron (NM	559	NM_03068	19704	Upf1	B430202H1
chr1	90551552	90551750	Intergenic	Intergenic	47115	NM_17730	320982	Arl4c	A630084M
chr7	52309027	52309225	promoter-T	promoter-T	-875	NM_17502	233210	Prr12	6720469B1
chr7	24974127	24974275	Intergenic	Intergenic	14137	NM_00102	232966	Zfp114	Gm1957|Z
chr2	91284452	91284600	intron (NM	intron (NM	273	NM_17512	228356	1110051M	AI586322
chr11	1.02E+08	1.02E+08	intron (NM	CpG-2660	409	NM_01155	21429	Ubtf	A930005G(
chr9	25311752	25312000	intron (NM	intron (NM	22694	NM_02618	67484	Eepd1	2310005P0
chr7	71432077	71432450	promoter-T	promoter-T	-293	NM_17274	233315	Mtmr10	BB128963|
chr9	57369277	57369350	intron (NM	CpG	274	NM_00774	12858	Cox5a	AA959768|
chr1	59820402	59821050	promoter-T	promoter-T	-397	NM_00756	12168	Bmpr2	2610024H2
chr2	35360727	35360800	Intergenic	ORR1A1|LT	-43818	NM_00114	14594	Ggta1	AW108479
chr14	73801227	73801400	Intergenic	Intergenic	-16235	NM_00841	16432	Itm2b	AI256040|

chr1	1.58E+08	1.58E+08	promoter-T	promoter-T	-255	NM_00959	11352	Abl2	AA536808|
chr11	76759402	76759550	intron (NM	CpG	318	NM_17864	104184	Blmh	AI035728|
chr6	1.13E+08	1.13E+08	intron (NM	intron (NM	533	NM_00103	101122	Rpusd3	AI527266
chr8	35869702	35870200	promoter-T	promoter-T	-713	NM_17693	319520	Dusp4	2700078F2
chr14	97437502	97437625	Intergenic	Intergenic	-519310	NM_05310	93688	Klhl1	mKIAA149(
chr11	84795727	84795850	Intergenic	Intergenic	24532	NM_00760	12351	Car4	AW456718
chr1	1.58E+08	1.58E+08	promoter-T	promoter-T	44	NM_00116	240832	Tor1aip2	1110020D1
chr4	1.35E+08	1.35E+08	TTS (NM_0	TTS (NM_0	35384	NM_00101	230824	Grhl3	AI561912|
chr10	98191077	98191350	Intergenic	Intergenic	-186573	NM_02648	67972	Atp2b1	2810442I2
chr3	1.58E+08	1.58E+08	intron (NM	intron (NM	165642	NM_00128	67144	Lrrc40	2610040E1
chr15	38447827	38447900	intron (NM	intron (NM	1158	NM_00110	54375	Azin1	1700085L0
chr8	1.24E+08	1.24E+08	intron (NM	CpG	346	NM_00128	53325	Banp	AA408158|
chr5	97360627	97360925	Intergenic	Intergenic	-65932	NM_08070	140780	Bmp2k	4933417M
chr4	1.45E+08	1.45E+08	Intergenic	Intergenic	-20878	NM_01161	21938	Tnfrsf1b	CD120b|TN
chr13	9878177	9878275	exon (NM_	exon (NM_	-113666	NM_14451	66505	Zmynd11	2210402G2
chr10	76722127	76722225	promoter-T	promoter-T	131	NM_03026	80294	Pofut2	2310011G2
chr17	37088577	37088725	promoter-T	promoter-T	86	NM_02963	76497	Ppp1r11	1500041B0
chr17	64212602	64213150	promoter-T	promoter-T	-454	NM_00103	14158	Fert2	AV082135|
chr5	1.21E+08	1.21E+08	promoter-T	promoter-T	37	NM_02804	71990	Ddx54	2410015A1
chrX	55449452	55449675	Intergenic	Intergenic	165758	NM_00957	22773	Zic3	Bn|Ka
chr1	95187652	95187925	intron (NM	intron (NM	14659	NM_17805	69821	Mterf4	1810059A2
chr5	1.39E+08	1.39E+08	TTS (NM_1	TTS (NM_1	471	NM_17787	330216	Mblac1	-
chr6	18537552	18537925	Intergenic	Intergenic	-72913	NM_08028	30785	Cttnbp2	3010022N2
chr1	7766727	7767225	Intergenic	RMER17A-i	687975	NM_18302	319263	Pcmtd1	8430411F1
chr2	1.53E+08	1.53E+08	intron (NM	intron (NM	728	NM_00127	13436	Dnmt3b	MmuIIIB
chr1	1.68E+08	1.68E+08	promoter-T	promoter-T	-686	NM_01113	18986	Pou2f1	2810482H(
chr17	29090977	29091075	intron (NM	intron (NM	865	NM_02588	66989	Kctd20	2410004N1
chr4	94717977	94718225	5' UTR (NM	5' UTR (NM	812	NM_01059	16476	Jun	AP-1|Junc|
chr4	1.55E+08	1.55E+08	intron (NM	CpG	181	NM_17720	230996	9430015G1	-
chr4	1.17E+08	1.17E+08	intron (NM	intron (NM	33146	NM_00114	381544	Gm1661	Gm1662|N
chr12	60141302	60141425	Intergenic	U2|snRNA	21085	NM_03005	78232	Trappc6b	5830498C1
chr4	1.2E+08	1.2E+08	intron (NM	intron (NM	-99541	NM_00790	13615	Edn2	VIC
chr4	1.32E+08	1.32E+08	intron (NM	intron (NM	8535	NM_13388	100340	Smpdl3b	1110054A2
chr1	10222727	10222925	promoter-T	promoter-T	-75	NM_00110	211673	Arfgef1	ARFGEP1|E
chr12	83648127	83648350	Intergenic	L1MD1|LIN	-69787	NM_01581	50779	Rgs6	-
chr17	46739127	46739250	intron (NM	intron (NM	27265	NM_17516	71461	Ptk7	8430404F2
chr10	42135852	42136150	intron (NM	intron (NM	62370	NM_14574	215951	Lace1	CG8520
chr2	1.21E+08	1.21E+08	promoter-T	promoter-T	-220	NM_01373	27223	Trp53bp1	53BP1|Tp5
chr5	13527077	13527200	intron (NM	intron (NM	127829	NM_00915	20346	Sema3a	Hsema-I|SI
chr19	47294727	47295100	intron (NM	(CACAT)n|S	-8420	NM_00116	18011	Neurl1a	2410129E1
chr9	1.24E+08	1.24E+08	Intergenic	Intergenic	7466	NM_00987	12581	Cdkn2d	INK4d|p19
chr7	86610852	86611050	intron (NM	CpG	208	NM_01746	18975	Polg	AA409516|
chrX	1.36E+08	1.36E+08	promoter-T	promoter-T	-671	NM_02327	66889	Rnf128	1300002C1
chr10	98569202	98569425	promoter-T	promoter-T	-492	NM_02774	382406	Poc1b	4933430F1
chr2	1.7E+08	1.7E+08	Intergenic	Intergenic	-21831	NM_02981	76960	Bcas1	2210416M
chr11	97182577	97182675	intron (NM	intron (NM	5596	NM_02592	67036	Mrpl45	2600005P(
chr13	93420952	93421025	intron (NM	MIRb|SINE	39895	NM_17258	218442	Serinc5	A130038L2

chr6	1.25E+08	1.25E+08	intron (NM	intron (NM	618	NM_00116	381810	Lpar5	GPR93|Gm
chr16	49713502	49713700	intron (NM	Lx9|LINE|L	14194	NM_02868	73916	Ift57	4833420A1
chr13	67552802	67552900	promoter-T	promoter-T	-291	NM_17771	238692	Zfp874a	C330011K1
chr1	1.46E+08	1.46E+08	Intergenic	Intergenic	87328	NM_00906	19735	Rgs2	GOS8
chrX	1.39E+08	1.39E+08	intron (NM	intron (NM	84295	NM_01949	56068	Ammecr1	6230420G1
chr13	67034377	67034500	promoter-T	promoter-T	-430	NM_02554	66410	Mterf3	2410017I1
chr9	1.07E+08	1.07E+08	Intergenic	Intergenic	-4123	NM_00117	56808	Cacna2d2	Cacna2d|a
chr15	80660277	80660350	intron (NM	PB1D10|SI	31273	NM_17712	213988	Tnrc6b	2700090M
chr8	87428477	87428575	intron (NM	intron (NM	2699	NM_01063	16596	Klf1	Eklf|Nan
chr4	1.39E+08	1.39E+08	intron (NM	CpG	493	NM_02985	77056	Tmco4	4632413C1
chr13	67912827	67912925	intron (NR_	RLTR26|LT	-1877	NM_19832	212569	Zfp273	6820416H(
chr9	59131952	59132175	Intergenic	Intergenic	-7316	NM_02812	72141	Adpgk	2610017G(
chr11	84011452	84011700	intron (NM	intron (NM	2636	NM_13336	107476	Acaca	A530025K(
chr17	31176577	31176850	Intergenic	Intergenic	-17926	NM_00959	11307	Abcg1	AW413978
chr14	22133902	22134000	intron (NM	intron (NM	-185041	NM_00120	54169	Kat6b	AI507552|
chr19	8915127	8915275	intron (NM	MIRb|SINE	208	NM_00129	14705	Bscl2	2900097C1
chr4	88941127	88941250	promoter-T	promoter-T	-665	NM_00987	12578	Cdkn2a	ARF-INK4a
chr19	4497802	4497875	intron (NM	intron (NM	745	NM_02723	69861	2010003K1	-
chr3	30692227	30692450	intron (NM	CpG	540	NM_02701	69276	Sec62	3100002M
chr12	1.18E+08	1.18E+08	5' UTR (NM	5' UTR (NM	135	NM_14603	217935	Wdr60	D430033N(
chr1	1.73E+08	1.73E+08	promoter-T	promoter-T	-961	NM_00108	226641	Atf6	9130025P1
chr1	1.57E+08	1.57E+08	Intergenic	L2|LINE|L2	-68901	NM_00978	12290	Cacna1e	A430040I1
chr14	21747402	21747625	Intergenic	Intergenic	-1142	NM_00950	22330	Vcl	9430097D2
chr13	30076477	30076550	promoter-T	promoter-T	-253	NM_00128	13557	E2f3	E2F3b|E2f3
chr2	26819952	26820025	promoter-T	promoter-T	-82	NM_20723	227656	Rexo4	Gm111|Xp
chr16	20535702	20535825	intron (NM	CpG	186	NM_00967	11773	Ap2m1	-
chr11	65601377	65601450	intron (NM	CpG	386	NM_00915	26398	Map2k4	JNKK1|MEI
chr18	32717177	32717650	intron (NM	intron (NM	2275	NM_00104	71683	Gypc	0610037F2
chr7	82926352	82926425	Intergenic	Intergenic	-66836	NM_00112	207952	Klhl25	2810402K1
chr7	97591302	97591375	promoter-T	promoter-T	47	NR_073438	233490	Crebzf	1110034C1
chr10	1.1E+08	1.1E+08	intron (NM	CpG	471	NM_17255	320150	Zdhhc17	A230053P1
chr1	1.93E+08	1.93E+08	intron (NM	intron (NM	18948	NM_17877	320119	Rps6kc1	AA682037|
chr15	36217252	36217450	Intergenic	Intergenic	-4449	NM_01392	30945	Rnf19a	AA032313|
chr12	71809052	71809275	Intergenic	L1MB8|LIN	-117338	NM_02812	319710	Frmd6	2610019M
chr10	1.26E+08	1.26E+08	TTS (NM_0	TTS (NM_0	32939	NM_02685	68876	Xrcc6bp1	1110068E(
chr7	1.33E+08	1.33E+08	promoter-T	promoter-T	92	NM_02984	77035	Kdm8	3110005O2
chr1	59176677	59176850	promoter-T	promoter-T	-113	NM_00103	381259	Tmem237	AI853305|/
chr7	54094827	54094900	Intergenic	Intergenic	-6311	NM_01069	16828	Ldha	Ldh1|Ldhm
chr8	27090752	27091050	TTS (NM_0	TTS (NM_0	-3685	NM_02988	52120	Hgsnat	9430010M
chr3	68761377	68761600	intron (NM	Lx8|LINE|L	47004	NM_02664	68259	Ift80	4921524P2
chr16	18809102	18809500	intron (NM	intron (NM	2431	NM_00116	12544	Cdc45	Cdc45l
chr3	30867627	30867925	intron (NM	CpG	561	NM_00116	241915	Phc3	E030046K(
chr7	34953602	34953825	promoter-T	promoter-T	-165	NM_01668	50995	Uba2	AA986091|
chr2	29790677	29790750	promoter-T	promoter-T	-216	NM_02892	74412	Gle1	4933405K2
chr8	74874627	74874750	intron (NM	intron (NM	31727	NM_00845	16598	Klf2	Lklf
chr12	86337152	86337575	promoter-T	promoter-T	92	NM_17836	56531	Ylpm1	A930013E1
chr5	1.16E+08	1.16E+08	Intergenic	Intergenic	-6097	NM_01122	19303	Pxn	AW108311

chr1	33692477	33692650	intron (NM	intron (NM	34076	NM_00892	19076	Prim2	AI323589
chr10	67267402	67267625	Intergenic	Intergenic	107897	NM_17867	216049	Zfp365	AI839779|/
chr18	47118477	47118675	promoter-T	promoter-T	46	NM_17837	69456	Commd10	2310003AC
chr4	93883027	93883300	Intergenic	MER45R|D	340324	NM_02636	67770	Caap1	5830433M
chr4	1.31E+08	1.31E+08	intron (NM	CpG	247	NM_02058	57317	Srsf4	5730499P1
chr4	1.23E+08	1.23E+08	intron (NM	CpG	236	NM_02587	66966	Trit1	2310075G1
chr4	33159827	33160200	intron (NM	intron (NM	9926	NM_00807	14409	Gabrr2	-
chr1	88968502	88968775	Intergenic	Intergenic	17865	NM_00743	11650	Alppl2	Akp5|C772
chr5	1.23E+08	1.23E+08	Intergenic	Intergenic	-20892	NR_027838	11938	Atp2a2	9530097L1
chr14	31366352	31366700	intron (NM	intron (NM	4209	NM_00938	21881	Tkt	p68
chr2	59915527	59915650	intron (NM	intron (NM	48209	NM_00100	407823	Baz2b	5830435C1
chr11	53840777	53840925	intron (NM	intron (NM	741	NM_01968	30805	Slc22a4	Octn1
chr1	1.79E+08	1.79E+08	Intergenic	Intergenic	-3341	NM_00101	30928	Zbtb18	RP58|Zfp2
chr4	1.49E+08	1.49E+08	promoter-T	promoter-T	-60	NM_00108	110208	Pgd	0610042AC
chr1	1.91E+08	1.91E+08	exon (NM_	exon (NM_	243	NM_17265	226823	Kctd3	4930438A2
chr6	1.4E+08	1.4E+08	intron (NM	intron (NM	11285	NM_01108	18705	Pik3c2g	C80387|PI3
chr8	1.08E+08	1.08E+08	promoter-T	promoter-T	-30	NM_00130	234699	Edc4	BC022641
chrX	74755352	74755425	Intergenic	Intergenic	-1178	NM_02060	21372	Tbl1x	5330429M
chr12	88224152	88224350	exon (NM_	exon (NM_	1513	NM_14583	238330	Irf2bpl	6430527G1
chr2	1.18E+08	1.18E+08	Intergenic	Intergenic	-122482	NM_01158	21825	Thbs1	TSP-1|TSP1
chr13	6634152	6634300	intron (NM	B4A|SINE|	13791	NM_01970	56421	Pfkp	1200015H2
chr4	69957877	69957950	intron (NM	intron (NM	113488	NM_14599	214444	Cdk5rap2	2900018KC
chr11	9433227	9433375	intron (NM	intron (NM	341356	NM_17825	268379	Abca13	9830132L2
chr4	1.55E+08	1.55E+08	exon (NM_	exon (NM_	318	NM_01138	20481	Ski	2310012I0
chr4	60020452	60020600	Intergenic	Intergenic	4105	NM_00108	620807	Mup6	Gm12544|
chr5	1.41E+08	1.41E+08	intron (NM	intron (NM	6859	NM_17527	78339	Ttyh3	2900029G1
chr15	76470627	76470750	promoter-T	promoter-T	-329	NM_18309	72749	Tonsl	2810439M
chr10	74518377	74518550	Intergenic	Intergenic	-5178	NM_00108	110279	Bcr	5133400CC
chr8	82163327	82163425	promoter-T	promoter-T	-199	NM_00125	73945	Otud4	4930431L1
chr8	47196777	47196900	intron (NM	intron (NM	12662	NM_20721	102141	Snx25	AI661919|
chr12	85749152	85749275	promoter-T	promoter-T	766	NM_00764	12499	Entpd5	AI196558|/
chr17	46297602	46297775	promoter-T	promoter-T	-293	NM_01958	56055	Gtpbp2	nmf205
chr19	5444552	5444850	Intergenic	L1MB5|LIN	-2997	NM_01023	14283	Fosl1	AW538199
chr15	76157702	76157825	promoter-T	promoter-T	-64	NM_17539	109075	Exosc4	1110039I0
chr17	3114627	3114975	promoter-T	promoter-T	-171	NM_13412	106583	Scaf8	A630086M
chr3	51685852	51686050	intron (NM	intron (NM	220836	NM_17499	211666	Mgst2	GST2|MGS
chr4	1.18E+08	1.18E+08	promoter-T	promoter-T	-179	NM_03361	114143	Atp6v0b	2310024H1
chr12	56937377	56937725	exon (NM_	exon (NM_	198	NM_00103	52592	Brms1l	0710008O1
chr8	86263952	86264150	intron (NM	intron (NM	1159	NM_00116	26364	Cd97	AA409984|
chr19	37643652	37643750	intron (NM	intron (NM	18793	NM_17535	107371	Exoc6	4833405E0
chr9	70132602	70132750	intron (NM	intron (NM	77519	NM_18107	71602	Myo1e	2310020N2
chr11	70795602	70795875	exon (NM_	exon (NM_	790	NM_00757	12261	C1qbp	AA407365|
chr13	75962477	75962550	Intergenic	Intergenic	-14821	NM_05310	93692	Glrx	C86710|D1
chr16	45224127	45224250	promoter-T	promoter-T	-262	NM_00103	208154	Btla	A630002H2
chr13	1.14E+08	1.14E+08	intron (NM	intron (NM	4471	NM_13079	170625	Snx18	Snag1
chr1	71603752	71604050	intron (NM	CpG	171	NM_02619	108147	Atic	2610509C2
chr10	1.27E+08	1.27E+08	promoter-T	promoter-T	101	NM_00124	14421	B4galnt1	4933429D1

chr1	55187777	55188000	promoter-T	promoter-T	-201	NM_02528	19070	Mob4	2610109B1
chr5	3596052	3596450	5' UTR (NM	5' UTR (NM	185	NM_00129	71382	Pex1	5430414H0
chr1	82758252	82758600	intron (NM	intron (NM	6605	NM_02545	66261	Tm4sf20	1810018L0
chrX	23270877	23271050	intron (NM	intron (NM	720	NM_17518	72404	Wdr44	2610034K1
chr17	71443327	71443450	intron (NM	intron (NM	74491	NM_00108	17929	Myom1	D430047A1
chr1	87961277	87961375	5' UTR (NM	5' UTR (NM	132	NM_02735	70247	Psmd1	2410026J1
chr9	1.04E+08	1.04E+08	intron (NM	intron (NM	1872	NM_00116	235567	Dnajc13	D030002L1
chr4	65883727	65884225	intron (NM	MIRm|SINI	181541	NM_01951	56079	Astn2	1d8|Astnl|
chr3	1E+08	1E+08	Intergenic	Intergenic	-6461	NM_00114	74645	Fam46c	4930431B0
chr6	29399477	29399550	intron (NM	intron (NM	16360	NM_00108	68794	Flnc	1110055E1
chr7	65951052	65951350	intron (NM	intron (NM	37629	NM_00972	11982	Atp10a	Atp10c|pfa
chr1	1.09E+08	1.09E+08	Intergenic	MIR|SINE|	27535	NM_02586	66957	Serpinb11	2310046M
chr13	31065552	31066050	intron (NM	CpG	115	NM_02558	66482	Exoc2	2410030I2
chr5	1.3E+08	1.3E+08	promoter-T	promoter-T	-905	NM_00939	19384	Ran	-
chr12	86910652	86910725	Intergenic	Intergenic	-29367	NM_00120	81703	Jdp2	Jundm2|Ju
chr12	55289277	55289475	intron (NM	intron (NM	15485	NM_02813	112407	Egln3	2610021G0
chr3	1.52E+08	1.52E+08	promoter-T	promoter-T	109	NM_02728	67035	Dnajb4	1700029A2
chr15	76028827	76029100	promoter-T	promoter-T	-324	NM_20139	18810	Plec	AA591047|
chr1	36893502	36893650	intron (NM	intron (NM	57747	NM_00128	22637	Zap70	Srk|ZAP-70
chr18	75522902	75523225	Intergenic	Intergenic	-3956	NM_00104	17131	Smad7	Madh7
chr15	99195477	99195550	intron (NM	intron (NM	5384	NM_01171	22379	Fmnl3	2700073B0
chr15	74819402	74819700	Intergenic	Intergenic	-5691	NM_02049	57248	Ly6i	AI789751|
chr6	1.31E+08	1.31E+08	Intergenic	L1_Mus2|L	14924	NM_02556	66441	Magohb	2010012C1
chr6	1.24E+08	1.24E+08	intron (NM	HAL1|LINE	3132	NM_18134	232371	C1rl	C1r-LP|C1r
chr17	35119952	35120050	intron (NM	intron (NM	926	NM_03059	27756	Lsm2	D17H6S56E
chr10	1.18E+08	1.18E+08	Intergenic	Intergenic	-38217	NM_00116	17245	Mdm1	Arrd2|Mdr
chr9	1.24E+08	1.24E+08	intron (NM	L1_Rod|LIN	30870	NM_01173	22599	Slc6a20b	AA986306|
chr8	48619352	48619425	promoter-T	promoter-T	389	NM_20350	192174	Rwdd4a	BC016198|
chr14	72963702	72963975	intron (NM	intron (NM	145972	NM_20763	319448	Fndc3a	1700094E1
chr1	37947102	37947650	promoter-T	promoter-T	-22	NM_02709	107734	Mrpl30	2310001L2
chr7	1.28E+08	1.28E+08	3' UTR (NM	3' UTR (NM	-6670	NM_17769	233813	Vwa3a	E030013G0
chr1	1.59E+08	1.59E+08	Intergenic	Intergenic	-56669	NM_02388	78255	Ralgps2	1810020P1
chr1	1.65E+08	1.65E+08	Intergenic	Intergenic	21146	NM_17888	98376	Gorab	AI467484|
chr18	24762377	24762575	promoter-T	promoter-T	14	NM_02144	58523	Elp2	AU023723
chr9	78328602	78329475	intron (NM	CpG	493	NM_01010	13627	Eef1a1	-
chr3	79371277	79371775	promoter-T	promoter-T	75	NM_00116	329679	Fnip2	D630023B1
chr5	1.11E+08	1.11E+08	intron (NM	CpG	255	NM_00116	72542	Pgam5	2610528A1
chr11	98221452	98221600	intron (NM	intron (NM	1844	NM_02154	59045	Stard3	Mln64|es6
chr16	48994002	48994225	promoter-T	promoter-T	112	NM_00111	224170	Dzip3	2310047C0
chr2	24603727	24603800	intron (NM	Tigger1|DN	14909	NM_00104	12287	Cacna1b	AW050276
chr14	35362052	35362225	intron (NM	CpG	-26151	NM_03006	78243	9230112D1	-
chr13	57067727	57067900	Intergenic	IAP-d-int|L	-70864	NM_01203	26946	Trpc7	TRP-7|TRP
chr2	1.28E+08	1.28E+08	Intergenic	Intergenic	76352	NM_00128	12125	Bcl2l11	1500006F2
chr10	61802002	61802225	intron (NM	intron (NM	1056	NM_00114	15275	Hk1	BB404130|
chr15	43311277	43311450	intron (NM	B4A|SINE|	2588	NM_02573	66736	Emc2	4921531G1
chr11	1.07E+08	1.07E+08	intron (NM	intron (NM	53746	NM_14582	71795	Pitpnc1	1110020B0
chr13	56712602	56712725	intron (NM	intron (NM	1699	NM_00936	21810	Tgfbi	68kDa|AI1

chr9	72313752	72313825	intron (NM	intron (NM	25978	NM_00935	21778	Tex9	Gm19407|
chr15	56868777	56869025	Intergenic	Intergenic	-342800	NM_00821	15117	Has2	-
chr3	95659402	95660500	promoter-T	promoter-T	-275	NM_02754	70767	Prpf3	3632413F1
chr7	1.49E+08	1.49E+08	promoter-T	promoter-T	43	NM_02652	68038	Chid1	3110023E0
chr15	3184127	3184350	Intergenic	RMER10B|	-36529	NM_00104	20363	Sepp1	AU018766
chr18	52668927	52669100	Intergenic	Intergenic	20362	NM_00128	16948	Lox	AI893619|
chr2	1.18E+08	1.18E+08	Intergenic	Intergenic	-127207	NM_01158	21825	Thbs1	TSP-1|TSP1
chr13	43266602	43266850	5' UTR (NM	5' UTR (NM	144	NM_00125	67046	Tbc1d7	2610009C0
chr11	11685702	11685825	Intergenic	Intergenic	23202	NM_02189	60530	Fignl1	-
chr8	1.26E+08	1.26E+08	exon (NM_	exon (NM_	102	NM_17228	234865	Nup133	mermaid
chr11	52173527	52173900	promoter-T	promoter-T	-904	NM_01169	22333	Vdac1	AL033343|
chr11	68912602	68912700	intron (NM	intron (NM	193	NM_01106	18626	Per1	Per|m-rigu
chr8	80042052	80042200	promoter-T	promoter-T	-320	NM_03011	78514	Arhgap10	A930033B0
chr13	64375577	64375850	intron (NM	CpG	583	NM_17258	218294	Cdc14b	2810432N1
chr15	76730302	76730525	promoter-T	promoter-T	42	NM_02553	66398	Commd5	2310065H0
chr7	29797502	29797675	intron (NM	ORR1C2|LT	29715	NM_00827	26411	Map4k1	Hpk1|mHP
chr5	1.11E+08	1.11E+08	intron (NM	CpG	191	NM_00126	209683	Ttc28	2310015L0
chr3	40604377	40604550	intron (NR_	CpG	590	NR_045576	20873	Plk4	1700028H2
chr1	1.83E+08	1.83E+08	Intergenic	Intergenic	-1006	NM_14594	208768	Sde2	-
chr8	13848802	13848975	Intergenic	RMER16-in	-10799	NM_15309	231201	AF366264	Susp4
chr1	1.57E+08	1.57E+08	Intergenic	Intergenic	16528	NM_01050	15939	Ier5	-
chr8	1.12E+08	1.12E+08	promoter-T	promoter-T	-206	NM_02946	75871	Zfp821	4930566A1
chr13	42147652	42147925	intron (NM	CpG	398	NM_00777	110521	Hivep1	Cryabp1
chr4	1.19E+08	1.19E+08	intron (NM	intron (NM	1080	NM_01173	22608	Ybx1	1700102N1
chr14	56340152	56340400	intron (NM	MIR|SINE|	737	NM_01951	56187	Rabggta	gm
chr14	28241602	28243025	exon (NM_	exon (NM_	280	NM_00111	218850	Fam208a	4732418E1
chr13	58230402	58230950	promoter-T	promoter-T	-759	NM_02987	77134	Hnrnpa0	1110055B0
chr11	1.22E+08	1.22E+08	promoter-T	promoter-T	-536	NM_17866	210004	B3gntl1	6030413G2
chr13	32894027	32894625	exon (NM_	exon (NM_	427	NM_03021	78903	Wrnip1	4833444L2
chr11	32167652	32167725	promoter-T	promoter-T	19	NM_18156	17168	Nprl3	Aag|CGTHI
chr1	88423827	88423975	intron (NM	CpG-365	590	NM_00897	19231	Ptma	Thym
chr2	1.73E+08	1.73E+08	intron (NR_	CpG	-7359	NM_02730	70065	Ankrd60	1700019A2
chr1	95239027	95239300	5' UTR (NM	5' UTR (NM	202	NM_08085	269224	Pask	mKIAA0135
chr1	1.22E+08	1.22E+08	intron (NM	intron (NM	18069	NM_01943	170706	Tmem37	AI173373|A
chr18	39151902	39152175	promoter-T	promoter-T	-761	NM_17516	71302	Arhgap26	1810044B2
chr8	1.17E+08	1.17E+08	Intergenic	Intergenic	31327	NM_02443	67528	Nudt7	1300007B2
chr14	79050527	79050825	intron (NM	intron (NM	-6418	NR_104044	380921	Dgkh	5930402B0
chr2	1.55E+08	1.55E+08	intron (NM	CpG	279	NM_02603	67204	Eif2s2	2810026E1
chr6	83004652	83004825	promoter-T	promoter-T	91	NM_00751	11993	Aup1	AA589454
chr4	82919552	82919700	intron (NM	RMER17B|	50467	NM_02723	69863	Ttc39b	1810054D0
chr3	9573777	9573925	intron (NM	intron (NM	36234	NM_13321	170753	Zfp704	C030026M
chr7	1.52E+08	1.52E+08	Intergenic	Intergenic	-30204	NM_00124	101772	Ano1	Tmem16a
chr17	46787302	46787650	TTS (NM_0	TTS (NM_0	189	NM_02561	66515	Cul7	2510004L2
chr12	78279327	78279550	Intergenic	Intergenic	-59653	NM_00125	53618	Fut8	-
chr5	36013602	36013800	exon (NM_	exon (NM_	8728	NM_00104	78558	Htra3	2210021K2
chr3	1.04E+08	1.04E+08	intron (NM	URR1B|DN	36016	NM_13385	99470	Magi3	4732496O1
chr18	4266252	4266425	Intergenic	Intergenic	86613	NM_00774	26410	Map3k8	Cot|Cot/Tp

chr12	66066727	66067000	promoter-T	promoter-T	134	NM_17780	328108	Fam179b	A430041B0
chr7	1.26E+08	1.26E+08	Intergenic	L2b|LINE|L	10701	NM_00994	12850	Coq7	clk-1
chr2	1.53E+08	1.53E+08	intron (NM	intron (NM	30567	NM_05309	84682	Cox4i2	Cox4b|Cox
chr12	80190427	80190525	exon (NM_	exon (NM_	181	NM_02998	110417	Pigh	2210416H0
chr15	63536252	63536600	Intergenic	Intergenic	103868	NM_03137	83492	Gsdmc	Gsdmc1|M
chrX	10721852	10721950	Intergenic	RMER30|D	-154482	NM_00111	1E+08	Gm14483	OTTMUSG0
chr11	4684727	4684850	intron (NM	L2c|LINE|L	-38007	NM_13894	192650	Cabp7	-
chr4	35047652	35047875	intron (NM	intron (NM	-51541	NM_19915	387510	Ifnk	-
chr6	39068027	39068225	promoter-T	promoter-T	222	NM_17289	243771	Parp12	9930021O1
chr4	1.25E+08	1.25E+08	Intergenic	Intergenic	13674	NM_15315	230738	Zc3h12a	BC036563|
chr11	1.15E+08	1.15E+08	Intergenic	B4|SINE|B4	19087	NM_19922	217304	Cd300lb	CLM-7|Clm
chr3	60335152	60335450	intron (NM	intron (NM	30127	NM_00125	56758	Mbnl1	Mbnl|mKIA
chr17	28358402	28358500	intron (NM	intron (NM	-11248	NM_01114	19015	Ppard	NUC-1|NU
chr19	53855527	53855975	intron (NM	intron (NM	103955	NM_00117	73713	Rbm20	1110018J2
chr12	1.01E+08	1.01E+08	Intergenic	L1MC4a|LI	-44765	NM_00894	19179	Psmc1	AI325227|
chr15	31383577	31383700	promoter-T	promoter-T	-194	NM_14585	252967	Ropn1l	ASP|AV047
chr4	1.32E+08	1.32E+08	intron (NM	CpG	552	NM_00119	100088	Rcc1	4931417M
chr13	36200452	36200675	intron (NM	intron (NM	8663	NM_20135	380840	Lyrm4	BC034664|
chr9	7331902	7332000	Intergenic	Intergenic	-15423	NM_00860	17381	Mmp12	AV378681|
chr9	46081152	46081300	promoter-T	promoter-T	79	NM_01175	22687	Zpr1	AI303781|
chr1	1.33E+08	1.33E+08	promoter-T	promoter-T	-478	NM_17869	226418	Yod1	9930028C2
chr15	93150027	93150100	intron (NR_	intron (NR_	17303	NR_02831	67057	Yaf2	2810021M
chr10	62706177	62706325	promoter-T	promoter-T	-294	NM_03011	67345	Herc4	1700056O1
chr13	46665502	46665725	intron (NM	intron (NM	68341	NM_02605	67252	Cap2	2810452G0
chr9	78284552	78284625	intron (NR_	MT2B|LTR	6456	NR_12572	214763	Mb21d1	E330016A1
chr3	88213627	88213875	TTS (NM_0	TTS (NM_0	487	NM_02592	67037	Pmf1	2600009M
chr5	75778152	75778575	Intergenic	Intergenic	-192649	NM_00112	16590	Kit	Bs|CD117|
chr14	69904352	69905025	Intergenic	L1ME4a|LI	-1528	NM_02633	67712	Slc25a37	1700020E2
chr1	1.3E+08	1.3E+08	Intergenic	Intergenic	-46158	NM_14550	226414	Dars	5730439G1
chrX	1.26E+08	1.26E+08	intron (NM	(T)n|Simple	180210	NM_17249	54004	Diap2	Dia3|Diaph
chr8	47896652	47896850	intron (NM	intron (NM	71652	NM_00839	16363	Irf2	9830146E2
chr10	41945002	41945100	intron (NM	intron (NM	51497	NM_01974	56484	Foxo3	1110048B1
chr2	1.56E+08	1.56E+08	exon (NM_	exon (NM_	177	NM_13915	245865	Spag4	1700041K2
chr1	1.52E+08	1.52E+08	promoter-T	promoter-T	-303	NM_14551	226499	BC003331	1810053E1
chr2	35095077	35095350	Intergenic	RMER20B|	-16666	NM_00120	227753	Gsn	ADF
chr8	97056977	97057150	5' UTR (NM	5' UTR (NM	135	NM_15350	234577	Cpne2	3322401K1
chr13	1.03E+08	1.03E+08	Intergenic	Intergenic	-79516	NM_00107	18708	Pik3r1	PI3K|p50al
chr12	71328402	71328525	promoter-T	promoter-T	207	NM_13319	110095	Pygl	-
chr18	60659827	60659950	intron (NM	intron (NM	1749	NM_13413	106878	Smim3	2010002N0
chr10	1.2E+08	1.2E+08	promoter-T	promoter-T	-342	NM_08044	117599	Helb	AI447783|
chr10	1.11E+08	1.11E+08	Intergenic	Intergenic	-5368	NM_02860	73690	Glipr1	2410114O1
chrX	1.2E+08	1.2E+08	Intergenic	Intergenic	-72117	NM_02842	73061	3110007F1	-
chr11	72020227	72020400	promoter-T	promoter-T	-743	NM_02655	52700	Txndc17	4831443O2
chr11	1E+08	1E+08	intron (NM	CpG	478	NM_01150	20918	Eif1	Sui1-rs1
chr2	1.12E+08	1.12E+08	promoter-T	promoter-T	-81	NM_13374	73024	Emc7	2900064A1
chr11	97490502	97490800	5' UTR (NM	5' UTR (NM	379	NM_17533	103551	E130012A1	AA409164|
chr3	89047552	89047700	exon (NM_	exon (NM_	1309	NR_12168	360213	Trim46	TRIFIC

chr6	54093402	54093625	intron (NM	intron (NM	103587	NM_00116	69993	Chn2	1700026N2
chr9	1.22E+08	1.22E+08	intron (NM	intron (NM	21542	NM_13374	20623	Snrk	2010012F0
chr2	79475952	79476125	intron (NM	CpG	456	NM_08055	70599	Ssfa2	5730488C1
chr7	1.51E+08	1.51E+08	promoter-T	promoter-T	89	NM_02429	79201	Tnfrsf23	Dctrailr1|T
chr13	1.14E+08	1.14E+08	Intergenic	Intergenic	17534	NM_02361	71690	Esm1	0610042H2
chr10	13272802	13273025	promoter-T	promoter-T	35	NM_01996	56535	Pex3	1700014F1
chr4	1.16E+08	1.16E+08	intron (NM	CpG	297	NM_00838	16351	Ipp	D4Jhu8|Mi
chr1	94806902	94807100	promoter-T	promoter-T	-401	NM_18140	108657	Rnpepl1	1110014H1
chr5	1.02E+08	1.02E+08	intron (NM	intron (NM	131939	NM_17288	72145	Wdfy3	2610509D0
chr6	83081277	83081375	Intergenic	RSINE1|SIN	-4476	NM_00113	20166	Rtkn	-
chr1	74283427	74283775	intron (NM	CpG	477	NM_02971	76709	Arpc2	2210023N0
chr11	98572127	98572275	intron (NM	intron (NM	9574	NM_00997	12985	Csf3	Csfg|G-CSF
chr1	60623402	60623475	5' UTR (NM	5' UTR (NM	171	NM_00104	77300	Raph1	9430025M
chr2	25430602	25430825	promoter-T	promoter-T	-322	NM_02929	75454	Phpt1	1700008C2
chr16	11144152	11144350	intron (NR_	LSU-rRNA_	-9626	NM_02958	106200	Txndc11	2810408E1
chr16	65197102	65197625	Intergenic	MTA_Mm|	-91753	NM_00831	15557	Htr1f	Htr1eb
chr15	6017377	6017725	Intergenic	Intergenic	-232238	NM_00100	13132	Dab2	5730435J1
chr17	50643552	50643675	Intergenic	Intergenic	-5259	NM_01388	224860	Plcl2	PLC-L2|PRI
chr7	1.21E+08	1.21E+08	intron (NM	CpG	227	NM_13370	68815	Btbd10	1110056N0
chr7	1.34E+08	1.34E+08	5' UTR (NM	5' UTR (NM	189	NM_00125	16408	Itgal	(p180)|Cd1
chr17	47921327	47921550	intron (NM	MIRm|SINE	-1255	NM_01154	21425	Tfeb	Tcfeb|bHLI
chr14	70274002	70274100	intron (NM	intron (NM	33837	NM_02852	73523	Pebp4	PEBP-4
chr8	27585477	27585550	Intergenic	Intergenic	316872	NM_19904	73754	Thap1	4833431A0
chr12	1.01E+08	1.01E+08	intron (NM	intron (NM	125	NM_00129	217827	Nrde2	6720454P0
chr5	32713727	32714000	Intergenic	Intergenic	-47480	NM_17270	19046	Ppp1cb	1200010B1
chr12	1.13E+08	1.13E+08	promoter-T	promoter-T	79	NM_00939	21928	Tnfaip2	B94|Exoc3
chr1	59969902	59970175	exon (NM_	exon (NM_	188	NM_00103	72750	Fam117b	2810425F2
chr5	31495327	31495425	promoter-T	promoter-T	136	NM_00112	13667	Eif2b4	Eif2b
chr11	61818477	61818700	intron (NM	intron (NM	48323	NM_00128	432572	Specc1	2810012G0
chr19	8972702	8972925	TTS (NM_1	TTS (NM_1	266	NM_00806	14376	Ganab	AU042638
chr13	43510652	43510725	intron (NM	intron (NM	16943	NM_02355	70078	Nol7	2210008F1
chr2	32443277	32443550	intron (NM	intron (NM	815	NM_01137	20448	St6galnac4	SIAT7-D|ST
chr5	42098102	42098200	intron (NM	intron (NM	1243	NM_02729	100972	Rab28	2700023P0
chr7	73820677	73821025	intron (NM	intron (NM	13596	NM_08044	117589	Asb7	AI449039|A
chr2	72998727	72999075	intron (NM	RSINE1|SIN	53603	NM_03009	67059	Ola1	2510025G0
chr2	18598952	18599025	5' UTR (NM	5' UTR (NM	343	NM_00755	12151	Bmi1	AW546694
chr13	40548777	40548975	Intergenic	Intergenic	-165496	NM_17214	218165	Ofcc1	opo
chr9	42291927	42292175	Intergenic	L1MEg|LIN	-11742	NM_17303	272589	Tbcel	D330014K2
chr10	62443027	62443225	intron (NM	CpG	155	NM_02742	70432	Rufy2	2610111M
chr2	1.27E+08	1.27E+08	exon (NM_	exon (NM_	206	NM_01009	13537	Dusp2	PAC1
chr1	1.68E+08	1.68E+08	exon (NM_	exon (NM_	302	NM_01180	433375	Creg1	AA755314|
chr1	1.77E+08	1.77E+08	intron (NM	intron (NM	101707	NM_01944	54418	Fmn2	AU024104
chr1	1.08E+08	1.08E+08	Intergenic	L1_Mm|LIN	-54520	NM_00112	227449	Zcchc2	9930114B2
chr4	1.33E+08	1.33E+08	promoter-T	promoter-T	-139	NM_00909	20111	Rps6ka1	Mapkapk-1
chr18	67921227	67921450	Intergenic	B1_Mus1|S	-13192	NM_02811	72124	Seh1l	2610007A1
chr15	1E+08	1E+08	5' UTR (NM	5' UTR (NM	132	NM_15340	207785	Csrnp2	CSRNP-2|C
chr1	30931052	30931275	Intergenic	PB1D9|SIN	-11062	NM_00108	213109	Phf3	2310061N1

chr13	96680402	96680550	Intergenic	Intergenic	-18599	NM_02771	544963	Iqgap2	4933417J2
chr2	79999052	79999150	Intergenic	Intergenic	-29532	NM_00115	18573	Pde1a	AI987702|/
chr3	1.1E+08	1.1E+08	exon (NM_	exon (NM_	529	NM_17889	99890	Prmt6	AW124876
chr17	33102752	33102900	promoter-T	promoter-T	50	NM_15306	224691	Zfp472	KRIM-1|Kri
chr5	73224152	73224500	intron (NM	intron (NM	35361	NM_00111	21682	Tec	-
chr17	26078927	26079025	promoter-T	promoter-T	-42	NM_01182	14755	Pigq	Gpi1|Gpi1l
chr8	35171377	35171550	promoter-T	promoter-T	303	NM_00129	22428	Dctn6	AU044699
chr13	25112002	25112100	exon (NM_	exon (NM_	135	NM_00101	380836	Mrs2	Gm902|HP
chr5	1.19E+08	1.19E+08	Intergenic	Intergenic	-84802	NM_17242	76199	Med13l	2210413I1
chr8	1.22E+08	1.22E+08	promoter-T	promoter-T	-4	NM_01996	56690	Mlycd	AI324784|
chr3	90482877	90482975	Intergenic	Intergenic	9933	NM_01365	20201	S100a8	60B8Ag|AI
chr17	47990702	47990950	Intergenic	Intergenic	-19186	NM_00110	17240	Mdfi	I-mf|I-mfa
chr11	1.2E+08	1.2E+08	intron (NM	intron (NM	746	NM_13379	192662	Arhgdia	5330430M
chr16	75912402	75912500	Intergenic	Intergenic	-2940	NM_02338	67742	Samsn1	4930571B1
chr3	1.06E+08	1.06E+08	promoter-T	promoter-T	-227	NM_14554	109905	Rap1a	AI848598|
chr9	94437777	94438225	exon (NM_	exon (NM_	499	NM_00103	68861	1190002N1	GoPro49|H
chr4	97800752	97800825	Intergenic	Intergenic	249168	NM_05315	94043	Tm2d1	2310026L1
chr17	29853627	29853850	intron (NM	intron (NM	224	NM_02678	68597	Ccdc167	1110021J0
chr15	66879727	66880325	Intergenic	Intergenic	-78823	NM_00868	17988	Ndrg1	CAP43|CM
chr14	73543152	73543500	intron (NR_	CpG	734	NR_033185	105670	Rcbtb2	2610028E0
chr4	3801927	3802150	Intergenic	Intergenic	-2786	NM_02002	17451	Mos	c-mos
chr1	1.53E+08	1.53E+08	Intergenic	Intergenic	-99290	NM_00103	117198	Ivns1abp	1190004M
chr10	92607852	92608175	Intergenic	Charlie24|	-15608	NM_14623	237459	Cdk17	6430598J1
chr18	33496902	33497450	Intergenic	Intergenic	-123706	NM_13377	170459	Stard4	4632419C1
chr7	17200052	17200600	promoter-T	promoter-T	16	NM_17273	232906	Arhgap35	6430596G1
chr7	59116827	59117175	exon (NM_	exon (NM_	636	NM_00111	1E+08	Fancf	A730016A1
chr17	35036702	35036800	exon (NM_	exon (NM_	832	NM_14583	110147	Ehmt2	Bat8|D17E
chr1	1.37E+08	1.37E+08	intron (NM	intron (NM	73005	NM_00930	20980	Syt2	R74640|m
chr19	16196927	16197225	Intergenic	Lx8|LINE|L	-10245	NM_00813	14682	Gnaq	1110005L0
chr3	1.07E+08	1.07E+08	intron (NM	intron (NM	2854	NM_00765	12508	Cd53	AI323659|
chr15	74968277	74968375	Intergenic	Intergenic	-25947	NM_00109	1E+08	Ly6c2	Ly-6C.2|Ly-
chr2	1.22E+08	1.22E+08	intron (NM	intron (NM	-4573	NM_08051	70928	Trim69	4921519C1
chr12	81110927	81111150	Intergenic	MIRc|SINE	102962	NM_00756	12192	Zfp36l1	AW742437
chr3	36183002	36183200	Intergenic	Intergenic	-61904	NM_19819	229214	Qrfpr	AQ27|Gpr1
chr16	23127952	23128075	promoter-T	promoter-T	-210	NM_14548	106344	Rfc4	A1|AI8941
chr3	1.08E+08	1.08E+08	intron (NM	intron (NM	20929	NM_00814	14686	Gnat2	AW490837
chr18	65541577	65541725	intron (NM	intron (NM	11891	NM_00103	225638	Alpk2	Gm549|Ha
chr17	28852827	28853100	intron (NM	intron (NM	23450	NM_00116	26416	Mapk14	CSBP2|Crk
chr9	48792602	48793200	promoter-T	promoter-T	-589	NM_17548	235323	Usp28	9830148O2
chr14	30835902	30836100	5' UTR (NM	5' UTR (NM	182	NM_17534	218865	Chdh	AU041472
chr1	72757352	72759125	intron (NM	CpG	404	NM_00908	19981	Rpl37a	-
chr16	10411677	10411875	promoter-T	promoter-T	-255	NM_01195	26425	Nubp1	-
chr8	3664502	3664750	Intergenic	Intergenic	-1136	NM_02698	69189	Mcemp1	1810033B1
chr9	43874902	43875150	promoter-T	promoter-T	-78	NM_19809	53376	Usp2	B930035K2
chr17	32421627	32421700	promoter-T	promoter-T	-585	NM_02050	57261	Brd4	Brd5|HUN
chr5	31191352	31191500	promoter-T	promoter-T	47	NM_00104	231093	Agbl5	4930455N0
chr9	54996727	54996875	promoter-T	promoter-T	-375	NM_18060	109161	Ube2q2	3010021M

chr19	44096102	44096275	exon (NM_	exon (NM_	7549	NM_00100	226143	Cyp2c44	AW107714
chr7	73205277	73205600	5' UTR (NM	5' UTR (NM	216	NM_02133	68981	Snrpa1	1500015N0
chr9	57571077	57571275	intron (NM	intron (NM	14800	NM_15379	353190	Edc3	AA517853|
chr11	1.01E+08	1.01E+08	intron (NM	intron (NM	6907	NM_14483	217203	Tmem106a	0610008L1
chr1	1.36E+08	1.36E+08	Intergenic	Intergenic	15606	NM_00757	12227	Btg2	AA959598|
chr17	28655027	28655125	Intergenic	CpG	-12730	NM_02629	67645	Armc12	4930511I1
chr1	1.73E+08	1.73E+08	3' UTR (NM	3' UTR (NM	25696	NM_17264	16456	F11r	9130004G2
chr4	1.51E+08	1.51E+08	intron (NM	intron (NM	55726	NM_00108	100072	Camta1	1810059M
chr1	1.36E+08	1.36E+08	promoter-T	promoter-T	69	NM_15312	240756	Klhl12	C3ip1
chr6	1.13E+08	1.13E+08	exon (NM_	exon (NM_	150	NM_00124	67767	Jagn1	5830427H1
chr7	1.17E+08	1.17E+08	promoter-T	promoter-T	279	NM_01197	26451	Rpl27a	L27'|L27A|
chr1	1.82E+08	1.82E+08	intron (NM	intron (NM	40466	NM_14610	226744	Cnst	9630058J2
chr8	37595877	37595950	Intergenic	Intergenic	43668	NM_17695	319582	6430573F1	Kiaa1456
chr1	1.47E+08	1.47E+08	Intergenic	Lx8|LINE|L	-517650	NM_02288	64214	Rgs18	-
chr16	44347452	44347800	intron (NM	CpG	112	NM_14455	212514	Spice1	Ccdc52|D1
chr12	37160452	37160650	intron (NM	intron (NM	51817	NM_00128	75847	Ispd	4930579E1
chr14	65060527	65060750	promoter-T	promoter-T	-761	NM_00125	110265	Msra	2310045J2
chr9	21639677	21640025	promoter-T	promoter-T	-103	NM_00108	624219	Gm6484	Angptl8|EC
chr14	14793502	14793625	intron (NM	CpG	264	NM_00128	66231	Thoc7	Nif3l1bp1
chr17	24388752	24388825	promoter-T	promoter-T	-434	NM_00763	12449	Ccnf	CycF|Fbxo1
chr11	69714702	69714900	promoter-T	promoter-T	-313	NM_02741	70419	2810408A1	1700019L0
chr13	24708427	24708700	intron (NM	intron (NM	2088	NM_17865	193385	Fam65b	-
chr17	28044577	28044825	promoter-T	promoter-T	-32	NM_02683	68776	Taf11	1110038O1
chr5	66156052	66156150	intron (NM	CpG	1341	NM_00102	333789	N4bp2	B3bp|E430
chr5	1.06E+08	1.06E+08	intron (NM	intron (NM	2644	NM_00103	433926	Lrrc8b	R75581|Ta
chr19	41338227	41338500	5' UTR (NM	5' UTR (NM	131	NM_13335	107358	Tm9sf3	1810073M
chr5	1.49E+08	1.49E+08	Intergenic	Intergenic	-49037	NM_01190	24109	Ubl3	AW108023
chr6	51474652	51474900	intron (NM	intron (NM	874	NM_00112	71982	Snx10	2410004M
chr6	1.19E+08	1.19E+08	exon (NM_	exon (NM_	167	NM_00103	319618	Dcp1b	B930050E0
chr2	30990752	30990900	intron (NM	intron (NM	6702	NM_00117	14269	Fnbp1	1110057E0
chr17	32168627	32168875	intron (NM	intron (NM	2702	NM_02890	74377	Hsf2bp	4932437G1
chr7	1.36E+08	1.36E+08	promoter-T	promoter-T	-121	NM_00102	207352	Sec23ip	D7Ertd373
chr11	1.17E+08	1.17E+08	intron (NM	CpG	645	NM_01135	20382	Srsf2	D11Wsu17
chr3	51221652	51221775	intron (NM	intron (NM	1775	NM_05308	74838	Naa15	5730450D1
chr13	98011977	98012100	intron (NM	intron (NM	978	NM_00793	13803	Enc1	Nrpb|PIG1
chr3	1.01E+08	1.01E+08	Intergenic	Intergenic	-12821	NM_14490	11928	Atp1a1	Atpa-1|BC0
chr12	17007027	17007225	promoter-T	promoter-T	-202	NM_00116	217430	Pqlc3	C78076|E0
chr17	34742852	34743225	5' UTR (NM	5' UTR (NM	232	NM_00116	55979	Agpat1	1-AGP|1-A
chr8	1.13E+08	1.13E+08	intron (NM	intron (NM	30685	NM_00791	13680	Ddx19a	DBP5|Ddx1
chr10	60814052	60814200	intron (NM	intron (NM	32145	NM_01375	27355	Pald1	MMPAL|Pa
chr17	44790102	44790300	intron (NM	intron (NM	82924	NR_073425	12393	Runx2	AML3|Cbf|
chr11	23566052	23566600	promoter-T	promoter-T	350	NM_02830	74467	Pus10	2810013G1
chr1	65160027	65160475	intron (NM	intron (NM	9537	NM_17529	98303	D630023F1	AI314969
chr16	18643577	18643675	Intergenic	Intergenic	-13595	NM_21361	18951	5-Sep	Cdcrel-1|C
chr7	1.34E+08	1.34E+08	exon (NM_	exon (NM_	1010	NM_02663	52858	Cdipt	9530042F1
chr8	72149302	72149400	promoter-T	promoter-T	96	NM_17275	234362	Zfp868	AI449175|
chr2	61368402	61368750	Intergenic	Intergenic	-48067	NM_01152	21353	Tank	C86182|E4

chr9	1.09E+08	1.09E+08	promoter-T	promoter-T	-198	NM_00892	19087	Prkar2a	1110061A2
chr12	1.12E+08	1.12E+08	Intergenic	Intergenic	-9621	NM_01048	15519	Hsp90aa1	86kDa|89k
chr5	75817302	75817375	Intergenic	Intergenic	-153674	NM_00112	16590	Kit	Bs|CD117|
chr10	77441427	77441500	promoter-T	promoter-T	68	NM_02643	67884	1810043G(	AV026620|
chr7	1.49E+08	1.49E+08	promoter-T	promoter-T	-237	NM_02830	101513	Mob2	1110017M
chr1	43148827	43148925	intron (NM	intron (NM	6591	NM_00101	73122	Tgfbrap1	3110018K1
chr18	77481077	77481150	intron (NM	intron (NM	-39584	NM_17283	240411	Loxhd1	1700096C2
chr10	44293977	44294125	Intergenic	Intergenic	-115558	NM_00754	12142	Prdm1	Blimp-1|Bl
chr10	79146352	79146550	intron (NM	intron (NM	1511	NM_17761	216150	Cdc34	AI327276|
chr12	8514252	8514450	Intergenic	Intergenic	-7560	NM_00748	11852	Rhob	AA017882|
chr1	23929152	23929425	promoter-T	promoter-T	-68	NM_02853	98366	Smap1	1700056O1
chr8	87185952	87186100	exon (NM_	exon (NM_	725	NM_01049	15936	Ier2	AI317238|
chr9	32363977	32364075	Intergenic	Intergenic	-14989	NM_00802	14247	Fli1	EWSR2|Fli-
chr17	34341477	34341625	5' UTR (NM	5' UTR (NM	127	NM_01153	21355	Tap2	ABC18|AI4
chr4	1.41E+08	1.41E+08	intron (NM	CpG	2224	NM_01976	56381	Spen	Mint|mKIA
chr4	6417227	6417425	Intergenic	Intergenic	-35908	NM_01094	18201	Nsmaf	AA959567|
chr18	75062852	75062925	Intergenic	Intergenic	58029	NM_01072	16891	Lipg	3110013K0
chr9	50575852	50575925	promoter-T	promoter-T	369	NM_02348	68721	1110032A(	4930500N(
chr5	65883552	65883975	intron (NM	CpG	311	NM_13369	68552	Smim14	1110003E0
chr8	37108652	37108750	Intergenic	Intergenic	-49181	NM_17291	244418	D8Ertd82e	9830148H2
chr12	52967427	52967550	Intergenic	Intergenic	-36965	NM_14478	207304	Hectd1	A630086P(
chr17	35300852	35302225	intron (NM	CpG	284	NM_00119	53761	Prrc2a	3110039B0
chr5	1.04E+08	1.04E+08	intron (NM	intron (NM	28846	NM_00108	17355	Aff1	9630032B(
chr10	80856402	80856475	intron (NM	intron (NM	629	NM_00109	72273	Smim24	2210404O(
chr1	1.02E+08	1.02E+08	intron (NM	intron (NM	307096	NM_17285	241175	Cntnap5b	C230078M
chr2	71291452	71291825	intron (NM	intron (NM	243	NM_02563	66559	Metap1d	2310066F2
chr15	75740377	75740450	promoter-T	promoter-T	248	NM_17864	105734	Tigd5	AA409802|
chr19	40906052	40906225	intron (NM	CpG	369	NM_17283	240665	Ccnj	D430039C2
chr12	16817502	16818300	non-coding	non-coding	130	NM_03327	50496	E2f6	AI462434|
chr2	1.81E+08	1.81E+08	promoter-T	promoter-T	-547	NM_00116	269401	Znf512b	Gm632
chr1	45925777	45925950	Intergenic	Intergenic	56576	NM_01691	53945	Slc40a1	Dusg|Fpn1
chr9	50817002	50817075	5' UTR (NM	5' UTR (NM	140	NM_17871	235344	Sik2	G630080D2
chr5	1.09E+08	1.09E+08	promoter-T	promoter-T	497	NM_02887	73130	Tmed5	3110020O1
chr16	18248352	18248425	promoter-T	promoter-T	399	NM_01123	19385	Ranbp1	Htf9a
chr1	1.86E+08	1.86E+08	promoter-T	promoter-T	73	NM_02201	63953	Dusp10	2610306G1
chr6	85911752	85911850	promoter-T	promoter-T	-140	NM_02809	72102	Dusp11	2010300F2
chr1	1.95E+08	1.95E+08	intron (NM	intron (NM	26037	NM_14488	226861	Hhat	2810432O2
chr13	65343202	65343375	Intergenic	CpG	-36264	NM_17529	97895	Nlrp4f	C330026N(
chr2	3336902	3336975	intron (NM	intron (NM	2982	NM_00857	104362	Meig1	MLZ-278|M
chr11	1.21E+08	1.21E+08	exon (NM_	exon (NM_	200	NM_00108	68837	Foxk2	1110054H(
chr2	1.2E+08	1.2E+08	Intergenic	L1MB2|LIN	-3777	NM_13383	98878	Ehd4	2210022F1
chr7	1.09E+08	1.09E+08	promoter-T	promoter-T	56	NM_13394	101706	Numa1	6720401E0
chr4	45419127	45419475	intron (NM	intron (NM	2337	NM_00100	230125	Slc25a51	9130208E0
chr2	65962952	65963300	promoter-T	promoter-T	-276	NM_01573	14425	Galnt3	-
chr7	52074977	52075075	intron (NM	intron (NM	-2998	NR_03313(	107503	Atf5	AFTA|Atf7
chr9	1.08E+08	1.08E+08	promoter-T	promoter-T	-185	NM_00816	14775	Gpx1	AI195024|
chr2	48862627	48862775	intron (NM	intron (NM	57673	NM_02992	109241	Mbd5	9430004D1

chr8	88012577	88012850	Intergenic	Intergenic	-3803	NM_17386	108682	Gpt2	4631422C0
chr10	1.21E+08	1.21E+08	Intergenic	Intergenic	-22507	NM_13895	192678	Rassf3	AW212023
chr6	86589252	86589550	Intergenic	Intergenic	11233	NM_02641	67855	Asprv1	2300003P2
chr8	23970002	23970200	promoter-T	promoter-T	90	NM_00108	244349	Kat6a	1500036M
chr19	47653777	47653950	promoter-T	promoter-T	-646	NM_00928	20874	Slk	9A2|AV021
chr13	53472627	53472850	promoter-T	promoter-T	-8	NM_00926	268656	Sptlc1	AW552086
chr11	1.1E+08	1.1E+08	intron (NM	intron (NM	863	NM_02188	19084	Prkar1a	1300018C2
chr11	51781102	51781525	exon (NM_	exon (NM_	180	NM_02997	52626	Cdkn2aipnl	A430101B0
chr13	1.13E+08	1.13E+08	Intergenic	MLT1A|LTR	-158146	NM_00116	77318	Ankrd55	C030011J0
chr4	55091752	55091975	intron (NM	intron (NM	131046	NM_17286	242466	Zfp462	6030417H0
chr12	36102577	36102850	Intergenic	Intergenic	116948	NM_01346	11622	Ahr	Ah|Ahh|Ah
chr11	29426277	29426625	promoter-T	promoter-T	54	NM_13376	76784	Mtif2	2310038D1
chr5	31556552	31556675	exon (NM_	exon (NM_	2534	NM_02722	69815	Krtcap3	2010001C0
chr6	88320327	88320550	intron (NM	intron (NM	76095	NM_02306	65967	Eefsec	Selb|sec
chr3	40887452	40887550	promoter-T	promoter-T	-533	NM_02755	70804	Pgrmc2	4631434O1
chr4	1.34E+08	1.34E+08	intron (NM	CpG	407	NM_00125	76824	Mtfr1l	2410166I05
chr13	37306702	37307000	Intergenic	Intergenic	-130363	NM_01074	17084	Ly86	MD-1|MD1
chr3	1.01E+08	1.01E+08	5' UTR (NM	5' UTR (NM	167	NM_14490	11928	Atp1a1	Atpa-1|BC0
chr14	51544202	51544275	promoter-T	promoter-T	330	NM_13367	66246	Osgep	1500019L2
chr13	54691877	54692100	promoter-T	promoter-T	396	NM_02593	67044	Higd2a	2010110M
chr10	57511252	57511500	Intergenic	Intergenic	-2974	NM_02056	57319	Smpdl3a	0610010C2
chr12	72293752	72293975	intron (NM	intron (NM	56028	NM_00116	76967	2700049A0	Talpid3|mK
chr6	1.46E+08	1.46E+08	intron (NM	intron (NM	1007	NM_01058	16439	Itpr2	AI649341|I
chr14	45839627	45840000	promoter-T	promoter-T	421	NM_02751	70713	Gpr137c	6330416L1
chr3	86558152	86558275	intron (NM	intron (NM	166593	NM_00119	70762	Dclk2	6330415M
chr13	59329577	59329875	Intergenic	Intergenic	316086	NM_00128	67269	Agtpbp1	1700020N1
chr4	1.05E+08	1.05E+08	Intergenic	MER58B|D	-7776	NM_08055	67916	Ppap2b	1110003O2
chr2	24191252	24191325	Intergenic	Intergenic	-1092	NM_03116	16181	Il1rn	F630041P1
chr8	67353277	67353500	intron (NM	B3|SINE|B.	20434	NM_17863	77113	Klhl2	6030411N2
chr10	82616677	82617050	intron (NM	intron (NM	168621	NM_02143	58250	Chst11	1110020P0
chr14	64806427	64806650	intron (NM	intron (NM	83378	NM_02852	73382	Prss52	1700049K1
chr12	74147977	74148050	promoter-T	promoter-T	-314	NM_00918	20471	Six1	BB138287
chr12	70781702	70781925	intron (NM	GC_rich|Lc	1026	NM_00113	20663	Sos2	SOS-2|mSC
chr12	76554402	76554825	3' UTR (NM	3' UTR (NM	-36845	NM_17564	217674	Gphb5	Zlut1
chr9	44039202	44039300	intron (NM	intron (NM	2878	NM_00761	12402	Cbl	4732447J0
chr19	21535777	21536275	intron (NM	intron (NM	11125	NM_01026	14544	Gda	AU015411
chr12	1.02E+08	1.02E+08	Intergenic	Intergenic	-26619	NM_00103	104718	Ttc7b	AA408451|
chr1	89651827	89651925	promoter-T	promoter-T	-710	NM_02984	77040	Atg16l1	1500009K0
chr19	36484227	36484575	intron (NM	CpG	30844	NM_02950	76073	Pcgf5	0610009F0
chr6	1.37E+08	1.37E+08	promoter-T	promoter-T	-486	NM_17565	320332	Hist4h4	B130044J0
chr14	55325127	55325725	Intergenic	Intergenic	5585	NM_20713	110794	Cebpe	C/EBPe|CR
chr11	1.16E+08	1.16E+08	intron (NM	intron (NM	641	NM_17880	338364	Trim65	4732463G1
chr6	17059302	17059525	Intergenic	Intergenic	44264	NM_20717	21753	Tes	D6Ertd352
chr10	98733052	98733425	Intergenic	Intergenic	7373	NM_02626	67603	Dusp6	1300019I0
chr6	1.25E+08	1.25E+08	promoter-T	promoter-T	-128	NM_01160	21937	Tnfrsf1a	CD120a|FP
chrX	39540102	39540175	intron (NM	intron (NM	-14894	NM_00129	20843	Stag2	9230105L2
chr11	1.21E+08	1.21E+08	promoter-T	promoter-T	-54	NM_02682	68730	Dus1l	1110032N1

chr2	1.1E+08	1.1E+08	promoter-T	promoter-T	52	NM_02661	68201	Ccdc34	2810027O1
chr6	1.41E+08	1.41E+08	promoter-T	promoter-T	-422	NM_00100	11569	Aebp2	AU023766
chr1	1.3E+08	1.3E+08	intron (NM	intron (NM	5044	NM_00108	226413	Lct	Gm100|LA
chr1	1.27E+08	1.27E+08	intron (NM	intron (NM	928	NM_02373	74117	Actr3	1200003AC
chr11	1.05E+08	1.05E+08	5' UTR (NM	5' UTR (NM	113	NM_18107	77097	Tanc2	3526402J0
chr3	69908452	69908650	Intergenic	Intergenic	97016	NM_00101	229389	Otol1	Gm414
chr10	1.19E+08	1.19E+08	promoter-T	promoter-T	-802	NM_02799	71902	Cand1	2310038O0
chr6	1.18E+08	1.18E+08	Intergenic	L1MA7|LIN	-7565	NM_02752	70727	Rasgef1a	6330404M
chr1	75205627	75205800	exon (NM_	exon (NM_	1640	NM_02901	74577	Glb1l	4833408P1
chr2	1.15E+08	1.15E+08	Intergenic	MLT1B|LTR	-231012	NM_02567	66632	Dph6	5730421E1
chr6	1.15E+08	1.15E+08	intron (NM	intron (NM	1682	NM_00125	74244	Atg7	1810013K2
chr17	56615827	56616300	promoter-T	promoter-T	-160	NM_01121	19280	Ptprs	AL022616|
chr1	39633652	39634025	exon (NM_	exon (NM_	354	NM_00103	67702	Rnf149	1600023E1
chr15	78858327	78858450	promoter-T	promoter-T	-254	NM_00819	14958	H1f0	D130017D0
chr11	1.05E+08	1.05E+08	3' UTR (NM	3' UTR (NM	-11372	NM_00862	17534	Mrc2	Endo180|m
chr19	3767952	3768025	intron (NM	CpG	567	NM_00116	225888	Suv420h1	AA117471|
chr17	80882652	80882875	Intergenic	Intergenic	-2970	NM_00923	20662	Sos1	4430401P0
chr12	1.12E+08	1.12E+08	Intergenic	Intergenic	-6245	NM_17520	74251	Ankrd9	2500003O2
chr12	1.1E+08	1.1E+08	intron (NM	intron (NM	31012	NM_00104	68519	Eml1	1110008N2
chr1	94651877	94652250	Intergenic	Intergenic	-76200	NM_01669	14733	Gpc1	AI462976
chr5	1.37E+08	1.37E+08	intron (NM	ID_B1|SINE	47088	NM_00129	13047	Cux1	CDP|Cutl1
chr4	56623177	56623300	Intergenic	Intergenic	131059	NM_02527	11471	Actl7b	Tact1
chr5	1.16E+08	1.16E+08	promoter-T	promoter-T	-528	NM_17540	109154	Mlec	2410014AC
chr15	81344702	81344775	Intergenic	Intergenic	47992	NM_01971	56438	Rbx1	1500002P1
chr13	63215052	63215175	intron (NM	intron (NM	-126225	NR_11052(	72061	2010111I0	2300006M
chr10	20602577	20602850	Intergenic	Intergenic	-69640	NM_02620	52906	Ahi1	1700015F0
chr5	1.44E+08	1.44E+08	promoter-T	promoter-T	-515	NM_00900	19353	Rac1	AL023026|
chr2	1.81E+08	1.81E+08	promoter-T	promoter-T	309	NM_00100	269400	Rtel1	AI451565|
chr2	52457802	52458175	intron (NM	intron (NM	-43857	NM_00128	12298	Cacnb4	3110038O1
chr7	91420827	91420900	intron (NM	intron (NM	-120460	NM_13372	70178	Abhd17c	2210412D0
chr10	63650802	63651150	intron (NM	intron (NM	-97973	NM_17867	216028	Lrrtm3	9630044H0
chr11	79118902	79119050	Intergenic	MLT1F|LTR	-34418	NM_01089	18015	Nf1	AW494271
chr14	73735002	73735150	Intergenic	Intergenic	-9478	NM_00902	19645	Rb1	Rb|Rb-1|p
chr8	42138752	42139025	intron (NM	intron (NM	1262	NM_00100	102103	Mtus1	AI481402|
chr14	32898727	32898825	promoter-T	promoter-T	102	NR_10430	105638	Dph3	DELGIP1|D
chr5	97341477	97341550	Intergenic	CT-rich|Lov	-85195	NM_08070	140780	Bmp2k	4933417M
chr8	1.27E+08	1.27E+08	exon (NM_	exon (NM_	144	NM_13927	108148	Galnt2	AI480629
chr14	33326877	33327075	intron (NM	CpG	269	NM_00108	319955	Ercc6	4732403I0
chr18	73731527	73731725	promoter-T	promoter-T	-733	NM_00103	240396	Mex3c	A130001D1
chr3	97871727	97871900	intron (NM	intron (NM	54352	NM_01092	18129	Notch2	AI853703|
chr1	97458502	97458675	Intergenic	Intergenic	105583	NM_00918	20452	St8sia4	PST|PST-1|
chr2	1.43E+08	1.43E+08	Intergenic	Intergenic	-1801	NM_00108	16558	Kif16b	8430434E1
chr1	87584127	87584350	intron (NM	B4A|SINE|	37613	NM_01367	20684	Sp100	A430075G1
chr8	47461452	47461650	Intergenic	L1MB3|LIN	-81526	NM_17378	234219	Helt	A830086M
chr7	30858027	30858100	promoter-T	promoter-T	-221	NM_00125	330502	Zfp82	A030010D0
chr9	99324227	99324375	intron (NM	intron (NM	12830	NM_00862	17532	Mras	2900078C0
chr1	75207477	75207675	promoter-T	promoter-T	172	NM_01149	20872	Stk16	EDPK|Krct|

chr1	1.74E+08	1.74E+08	promoter-T	promoter-T	54	NM_02160	59287	Ncstn	9430068N1
chr4	1.33E+08	1.33E+08	exon (NM_	exon (NM_	103	NM_17287	242691	Gpatch3	D930035B0
chr7	26465077	26465350	promoter-T	promoter-T	-964	NM_17214	232987	B9d2	stumpy
chr18	3004377	3004525	Intergenic	CT-rich|Lov	118961	NM_00116	1E+08	Vmn1r238	-
chr14	56291052	56291325	promoter-T	promoter-T	116	NM_17799	105446	Gmpr2	1810008P1
chr12	88812827	88813150	intron (NM	CpG	261	NM_02550	66354	Snw1	2310008B0
chr7	1.1E+08	1.1E+08	exon (NM_	exon (NM_	350	NM_14638	258378	Olfr593	MOR24-2
chr14	47984727	47984800	Intergenic	Intergenic	-8772	NM_01070	16854	Lgals3	GBP|L-34|
chr5	1.46E+08	1.46E+08	promoter-T	promoter-T	207	NM_00103	231887	Pdap1	HASPP28|P
chr18	63851427	63851725	intron (NM	CpG	437	NM_01679	53382	Txnl1	32kDa|TRP
chr9	1.1E+08	1.1E+08	intron (NM	CpG	612	NM_00108	235626	Setd2	4921524K1
chrX	1.04E+08	1.04E+08	Intergenic	Intergenic	29522	NM_17544	213438	A630033H2	AI662791|
chr17	74927902	74928025	exon (NM_	exon (NM_	328	NM_00756	12211	Birc6	A430032G0
chr3	65327427	65327650	Intergenic	Intergenic	-4831	NM_17889	99929	Tiparp	ARTD14|A\
chr6	1.28E+08	1.28E+08	intron (NM	intron (NM	4633	NM_00103	381812	Cracr2a	Efcab4b|G
chr3	88420827	88421075	intron (NM	intron (NM	822	NM_00119	16800	Arhgef2	AA408978|
chr16	16571577	16571700	Intergenic	Intergenic	-11349	NM_13923	224014	Fgd4	9030023J0
chr5	1.24E+08	1.24E+08	exon (NM_	exon (NM_	165	NM_00113	208908	Ccdc62	AI661708|
chr12	1.07E+08	1.07E+08	intron (NM	CpG	353	NM_00102	22367	Vrk1	51PK
chr18	39825952	39826375	Intergenic	Intergenic	-106988	NM_01103	18459	Pabpc2	Pabp|Pabp
chr5	21290752	21291025	promoter-T	promoter-T	95	NM_00958	22791	Dnajc2	AU020218
chr5	29704927	29705775	promoter-T	promoter-T	-421	NM_02029	56873	Lmbr1	1110048D1
chr14	61376377	61376475	intron (NM	intron (NM	-10545	NM_18317	239126	C1qtnf9	9130217G2
chr1	1.59E+08	1.59E+08	promoter-T	promoter-T	-43	NM_02314	30935	Tor3a	Adir
chr1	1.66E+08	1.66E+08	intron (NM	intron (NM	4794	NM_00116	20343	Sell	AI528707|
chr6	1.13E+08	1.13E+08	promoter-T	promoter-T	131	NM_17510	66087	Emc3	0610039A1
chr10	17417627	17417925	Intergenic	Intergenic	-25258	NM_01082	17684	Cited2	AI835299|
chr4	58926502	58926775	Intergenic	ORR1D2|LT	-1041	NM_17238	230249	AI314180	AW558785
chr17	56899902	56900025	Intergenic	Intergenic	-3010	NM_00873	18188	Nrtn	NTN
chr13	59660002	59660225	Intergenic	Intergenic	-1405	NM_02332	67269	Agtpbp1	1700020N1
chr10	33739677	33739850	promoter-T	promoter-T	-341	NM_02561	66521	Rwdd1	0710001K0
chr10	24589802	24590000	5' UTR (NM	5' UTR (NM	109	NM_02734	70208	Med23	130kDa|30
chr11	69225002	69225150	intron (NM	CpG	2101	NM_00101	216850	Kdm6b	1700064E0
chr5	1.49E+08	1.49E+08	Intergenic	Intergenic	-16724	NM_01022	14254	Flt1	AI323757|
chr8	36271402	36271500	Intergenic	Intergenic	-167344	NM_17774	244416	Ppp1r3b	6430576E2
chr5	24082802	24083025	intron (NM	CpG	372	NM_00119	27407	Abcf2	0710005O0
chr1	1.33E+08	1.33E+08	intron (NM	intron (NM	45392	NM_01070	16865	Eif2d	D1Ertd5e|
chr2	1.78E+08	1.78E+08	promoter-T	promoter-T	155	NM_00108	228966	Ppp1r3d	2610024M
chr13	98801452	98801625	intron (NM	intron (NM	174582	NM_01202	110596	Arhgef28	9230110L0
chr13	3536527	3536600	promoter-T	promoter-T	-758	NM_00811	14569	Gdi2	GDI-B|GDII
chr11	76296252	76296325	intron (NM	intron (NM	-14321	NM_00129	109934	Abr	-
chr1	1.4E+08	1.4E+08	Intergenic	Intergenic	54023	NM_17739	338375	Atp6v1g3	-
chr16	22315877	22316025	Intergenic	Intergenic	-49949	NM_00918	20462	Tra2b	5730405G2
chr16	8637702	8638100	promoter-T	promoter-T	101	NM_01688	54128	Pmm2	AI585868|
chrY	2788752	2788950	Intergenic	Intergenic	401593	NM_00127	1E+08	Gm3376	Rbmy1b
chr3	94385552	94385625	intron (NM	intron (NM	1050	NM_00108	76742	Snx27	5730552M
chr1	16678277	16678450	promoter-T	promoter-T	-174	NM_00115	17087	Ly96	ESOP-1|MI

chr7	36340552	36340850	exon (NM_	exon (NM_	246	NM_03308	110959	Nudt19	D7Rp2|D7I
chr1	44204302	44204525	promoter-T	promoter-T	-176	NM_01172	22592	Ercc5	Xpg
chr15	59510727	59511050	Intergenic	MIR|SINE|	30679	NM_14454	211770	Trib1	A530090O1
chr1	94113352	94113425	Intergenic	ID_B1|SINE	-68418	NM_20722	208727	Hdac4	4932408F1
chr1	1.73E+08	1.73E+08	exon (NM_	exon (NM_	229	NM_01948	56009	Alyref2	C130042O1
chr2	1.5E+08	1.5E+08	Intergenic	Intergenic	127101	NM_02326	104348	Zfp120	AI315103|I
chr4	1.3E+08	1.3E+08	Intergenic	Intergenic	-7151	NM_00129	230775	Bai2	-
chr19	12531977	12532100	Intergenic	Intergenic	-3231	NM_01082	17476	Mpeg1	MPS1|Mpg
chr15	90054327	90054525	promoter-T	promoter-T	-316	NM_00103	380959	Alg10b	AA469671|
chr3	85654027	85654300	Intergenic	MER2|DNA	37277	NM_17713	320302	Glt28d2	4732486J0
chr6	83817502	83817725	Intergenic	Intergenic	-35878	NM_14616	232164	Paip2b	-
chr13	89879402	89879650	intron (NM	intron (NM	2591	NM_00108	13003	Vcan	5430420N0
chr19	37494527	37494800	intron (NM	intron (NM	-14668	NM_00824	15242	Hhex	Hex|Hex1|
chr12	11184427	11184550	Intergenic	Intergenic	-26840	NM_00116	238076	Kcns3	-
chr9	74918877	74919475	5' UTR (NM	5' UTR (NM	163	NM_01086	17918	Myo5a	9630007J1
chr9	1.09E+08	1.09E+08	intron (NM	intron (NM	4491	NM_17301	270198	Pfkfb4	C230090D1
chr12	74816852	74817075	intron (NM	intron (NM	130935	NM_00885	18755	Prkch	Pkch
chr11	1.02E+08	1.02E+08	intron (NM	intron (NM	355	NM_02654	68066	Slc25a39	3010027G1
chr11	34659527	34659850	Intergenic	Intergenic	-12545	NM_02741	70385	Spdl1	1700018I0
chr16	17531477	17531550	promoter-T	promoter-T	-368	NM_02690	69009	Thap7	1810004B0
chr16	32277602	32277700	5' UTR (NM	5' UTR (NM	104	NM_02735	70238	Rnf168	3110001H1
chr13	46072827	46072975	Intergenic	Intergenic	-12541	NM_00912	20238	Atxn1	2900016G2
chr12	80336402	80336750	intron (NM	intron (NM	26675	NM_03001	77974	Rdh12	A930033N0
chr2	32568152	32568350	promoter-T	promoter-T	53	NM_13086	107951	Cdk9	PITALRE
chr14	27783252	27783575	intron (NM	CpG	324	NM_14522	72993	Appl1	2900057D2
chr15	40655852	40656025	intron (NM	intron (NM	169350	NM_01176	22762	Zfpm2	B330005D2
chr15	35085177	35085300	intron (NM	CpG	323	NM_01963	56274	Stk3	0610042I0
chr6	99724677	99724925	Intergenic	Intergenic	-48415	NM_00117	50501	Prok2	Bv8|PK2|P
chr14	60210952	60211225	intron (NM	intron (NM	5708	NM_00116	71891	Cdadc1	2310010M
chr6	60916977	60917275	intron (NM	intron (NM	22815	NM_00116	70945	Mmrn1	4921530G0
chr8	64390177	64390450	Intergenic	Intergenic	-8822	NM_00129	72333	Palld	2410003B1
chr8	1.09E+08	1.09E+08	intron (NM	intron (NM	-21040	NM_01090	18021	Nfatc3	C80703|D8
chr9	37015827	37016075	promoter-T	promoter-T	33	NM_02546	66279	Tmem218	1810021J1
chr9	1.1E+08	1.1E+08	Intergenic	Intergenic	-37378	NM_00101	382106	Fbxw24	EG382106|
chr13	89879302	89879375	intron (NM	CpG	2779	NM_00108	13003	Vcan	5430420N0
chr2	1.64E+08	1.64E+08	Intergenic	Intergenic	-16213	NM_00127	11486	Ada	-
chr9	96853077	96853325	intron (NM	intron (NM	-63308	NM_00128	235534	Pxylp1	9430094M
chr14	66480077	66480225	intron (NM	intron (NM	24012	NM_14594	67179	Ccdc25	2610528H1
chr18	35118227	35118850	Intergenic	Intergenic	-4533	NM_01048	15526	Hspa9	74kDa|Csa
chr17	27956702	27957100	intron (NM	intron (NM	586	NM_00103	224647	D17Wsu92	AU020189
chr12	1.19E+08	1.19E+08	Intergenic	Intergenic	-6358	NM_14604	217946	Cdca7l	BC006933|
chr3	90293677	90293775	intron (NM	intron (NM	1209	NM_13385	20615	Snapin	25kDa|AA4
chr2	76477802	76477875	intron (NM	intron (NM	8213	NM_01187	23992	Prkra	AV120107|
chr7	1.15E+08	1.15E+08	promoter-T	promoter-T	-914	NM_00890	19024	Ppfibp2	Cclp1
chr9	70587852	70588150	intron (NM	intron (NM	61193	NM_00739	11487	Adam10	1700031C1
chr11	99092427	99092575	promoter-T	promoter-T	-170	NM_02061	57376	Smarce1	2810417B2
chr12	74405777	74405975	intron (NM	intron (NM	18041	NM_00103	625098	Slc38a6	AW322671

chr5	1.51E+08	1.51E+08	intron (NM	intron (NM	1762	NM_13389	100637	N4bp2l1	2410024N1
chr5	3388752	3388950	intron (NM	intron (NM	44539	NM_00987	12571	Cdk6	5830411I2
chr2	1.12E+08	1.12E+08	intron (NM	CpG	294	NM_13374	73024	Emc7	2900064A1
chr13	1.02E+08	1.02E+08	intron (NM	CpG	278	NM_02188	26886	Cenph	1700021I1
chr10	1.27E+08	1.27E+08	promoter-T	promoter-T	-332	NM_14601	216445	Arhgap9	AU043488
chr7	1.01E+08	1.01E+08	Intergenic	L1_Mur1|L	537262	NM_02142	58238	Fam181b	A830059I2
chr6	81873727	81873850	exon (NM_	exon (NM_	125	NM_17788	330361	Gcfc2	A130099G
chr8	60723652	60723725	intron (NM	URR1A|DN	455905	NM_00117	1E+08	Gm15881	OTTMUSG(
chr11	89931702	89931800	Intergenic	Lx8|LINE|L	40089	NM_02643	67888	Tmem100	1810057C1
chr8	85875252	85875475	intron (NM	intron (NM	2992	NM_02623	67555	Mgat4d	4933434I2
chr4	1.33E+08	1.33E+08	Intergenic	Intergenic	59945	NM_00128	20111	Rps6ka1	Mapkapk-1
chr10	1.28E+08	1.28E+08	intron (NM	intron (NM	1630	NM_00108	210582	Coq10a	Gm1
chr11	1.03E+08	1.03E+08	intron (NM	CpG-2675	13410	NM_00111	11488	Adam11	AW060611
chr4	53795202	53795300	intron (NM	intron (NM	2674	NM_00931	21350	Tal2	bHLHa19
chr6	95668477	95668700	intron (NM	CpG	252	NM_01150	20917	Suclg2	AF171077|
chr3	58218777	58218900	promoter-T	promoter-T	-773	NM_00108	72033	Tsc22d2	1810043J1
chr18	36919502	36919600	promoter-T	promoter-T	-342	NM_02646	67936	Wdr55	2410080P2
chr8	28238827	28239225	promoter-T	promoter-T	78	NM_02568	66653	Brf2	2700059M
chr9	1.03E+08	1.03E+08	promoter-T	promoter-T	58	NM_00113	321022	Cdv3	2510010F1
chr5	1.38E+08	1.38E+08	promoter-T	promoter-T	-149	NM_00110	83701	Srrt	2810019G(
chr1	1.8E+08	1.8E+08	Intergenic	RLTR25A|L	-18694	NM_02707	69428	1700016C1	-
chr2	1.19E+08	1.19E+08	promoter-T	promoter-T	-323	NM_17715	320415	Gchfr	2010323F1
chr3	58495552	58495725	promoter-T	promoter-T	672	NM_00917	20439	Siah2	AA415433|
chr2	1.43E+08	1.43E+08	promoter-T	promoter-T	33	NM_02133	20639	Snrpb2	2810052G(
chr14	79880677	79880825	promoter-T	promoter-T	-250	NM_00792	13709	Elf1	Elf-1|Sts1|
chr9	32317352	32317775	intron (NM	intron (NM	31474	NM_00802	14247	Fli1	EWSR2|Fli-
chr11	1.14E+08	1.14E+08	Intergenic	(TTC)n|Sim	-99481	NM_02337	67671	Rpl38	0610025G1
chr15	76198877	76199050	promoter-T	promoter-T	-365	NM_02155	59053	Hgh1	Brp16|D15
chr7	51723527	51723650	TTS (NM_0	TTS (NM_0	238	NM_00120	66124	Josd2	1110007C(
chr4	73951477	73951900	intron (NM	intron (NM	53905	NM_14478	76804	Kdm4c	2410141F1
chr2	1.48E+08	1.48E+08	exon (NM_	exon (NM_	1108	NM_01074	17064	Cd93	6030404G(
chr6	52811077	52811675	intron (NM	intron (NM	48238	NM_00116	231986	Jazf1	AI591476|
chr11	53114377	53114450	promoter-T	promoter-T	-432	NM_00830	15525	Hspa4	70kDa|AI3
chr1	34899952	34900125	promoter-T	promoter-T	-143	NM_17499	214469	Fam168b	mKIAA404
chr2	27330627	27330775	5' UTR (NM	5' UTR (NM	217	NM_02333	67382	Brd3	2410084F2
chr10	79785552	79785950	exon (NM_	exon (NM_	-2458	NM_02552	66374	2310011J0	AI452186|
chr1	1.29E+08	1.29E+08	intron (NM	intron (NM	38425	NM_14512	107895	Mgat5	4930471A2
chr14	20570402	20570500	promoter-T	promoter-T	-28	NM_00869	18074	Nid2	AW547149
chr3	87371402	87371900	Intergenic	Intergenic	42151	NM_01205	27049	Etv3	AI414410|
chr5	1.4E+08	1.4E+08	intron (NM	B2_Mm1a|	34454	NM_17552	243312	Elfn1	A930017N(
chr10	79479552	79479700	exon (NM_	exon (NM_	209	NM_00114	70719	Hmha1	6330406L2
chr4	1.35E+08	1.35E+08	promoter-T	promoter-T	-234	NM_17485	242700	Ifnlr1	CRF2-12|Il
chr13	1.05E+08	1.05E+08	Intergenic	L1ME3A|LI	206607	NM_02607	67285	Cwc27	3110009E1
chr6	1.29E+08	1.29E+08	intron (NM	intron (NM	1244	NM_02000	56644	Clec7a	BGR|Clecsf
chr12	75009102	75009200	5' UTR (NM	5' UTR (NM	297	NM_01043	15251	Hif1a	AA959795|
chr19	37999927	38000125	intron (NM	MIRc|SINE	118041	NM_00109	226101	Myof	2310004N1
chr2	90686927	90687000	promoter-T	promoter-T	-349	NM_01975	56428	Mtch2	2310034D2

chr2	1.8E+08	1.8E+08	promoter-T	promoter-T	656	NM_00128	26444	Psma7	C6-I
chr7	1.18E+08	1.18E+08	promoter-T	promoter-T	-417	NM_17732	319934	Sbf2	4833411BC
chr5	77615427	77615525	Intergenic	Intergenic	24754	NM_00128	69982	Spink2	1700007F2
chr2	35056052	35056275	intron (NM	CpG	477	NM_02669	68365	Rab14	0610030G2
chr17	46008977	46009175	Intergenic	Intergenic	-138283	NM_02816	72240	1600014C2	-
chr10	20871552	20871650	intron (NM	intron (NM	9189	NM_00119	17863	Myb	AI550390|
chr9	67626427	67626650	Intergenic	Intergenic	19294	NM_00108	75697	C2cd4b	3300001AC
chr17	24687227	24687325	exon (NM_	exon (NM_	381	NM_01363	18763	Pkd1	PC1|mFLJ0
chr15	82953002	82953075	intron (NM	CpG	193	NM_02910	74778	Rrp7a	1110014J0
chr18	56895602	56895700	intron (NM	intron (NM	28184	NM_01072	16906	Lmnb1	-
chr2	1.65E+08	1.65E+08	intron (NM	CpG	349	NM_19902	329559	Zfp335	1810045J0
chr1	95714827	95715050	3' UTR (NM	3' UTR (NM	-6879	NM_17888	98314	D2hgdh	AA408776|
chr11	1.2E+08	1.2E+08	promoter-T	promoter-T	80	NM_00103	66156	Anapc11	1110011I1
chr1	95645227	95645300	intron (NM	intron (NM	6152	NM_02592	67026	Thap4	2010320BC
chr3	21976127	21976375	intron (NM	intron (NM	677	NM_03073	81004	Tbl1xr1	8030499HC
chr14	31528552	31528700	promoter-T	promoter-T	-175	NM_00116	54650	Sfmbt1	4930442N2
chr7	1.41E+08	1.41E+08	intron (NM	intron (NM	193167	NM_00740	11489	Adam12	Mltna|mKI
chr19	43748327	43748525	intron (NM	intron (NM	945	NM_14515	246696	Slc25a28	2210403D1
chr1	20880727	20880800	promoter-T	promoter-T	60	NM_02882	74229	Paqr8	1700019B1
chr3	1.31E+08	1.31E+08	Intergenic	Intergenic	-88644	NM_00128	23971	Papss1	AI325286|
chr5	1.22E+08	1.22E+08	intron (NM	CpG	135	NM_17272	231713	Naa25	4833422K1
chr16	52371977	52372475	intron (NM	intron (NM	80884	NM_00965	11658	Alcam	AI853494|
chr1	88367002	88367250	Intergenic	Intergenic	44222	NM_02796	71863	1700019O1	-
chr16	44984977	44985250	Intergenic	Intergenic	41322	NM_00112	74603	Cd200r3	4733401I1
chr1	1.33E+08	1.33E+08	3' UTR (NM	3' UTR (NM	-7073	NM_01977	56489	Ikbke	AW558201
chr9	65308802	65308925	5' UTR (NM	5' UTR (NM	119	NM_13858	27965	Spg21	ACP33|BM
chr19	25054252	25054350	Intergenic	MLT1B|LTF	-18195	NM_14609	226043	Cbwd1	AV349248|
chr10	87853177	87853350	intron (NM	intron (NM	11106	NM_00100	432486	Gnptab	EG432486|
chr10	9620802	9620950	promoter-T	promoter-T	-38	NM_00108	78808	Stxbp5	0710001E2
chr2	90929752	90930075	Intergenic	L1MC|LINE	-7041	NM_01135	20375	Spi1	Dis-1|Dis1
chr11	98802877	98803100	intron (NM	intron (NM	1963	NM_00117	19401	Rara	Nr1b1|RAF
chr3	95032327	95032525	promoter-T	promoter-T	80	NM_17239	57912	Cdc42se1	1300002M
chr3	9725352	9725825	intron (NM	intron (NM	108091	NM_05318	94212	Pag1	Cbp|F7300
chr2	1.56E+08	1.56E+08	promoter-T	promoter-T	-283	NM_02758	70873	Cnbd2	4921517L1
chr7	31197952	31198075	promoter-T	promoter-T	-794	NM_01166	22177	Tyrobp	DAP12|KAI
chr6	53075027	53075275	Intergenic	MER63B|D	-56533	NM_17340	231986	Jazf1	AI591476|
chr5	1.46E+08	1.46E+08	promoter-T	promoter-T	-112	NM_02314	11867	Arpc1b	41kDa|AA4
chr1	1.09E+08	1.09E+08	5' UTR (NM	5' UTR (NM	354	NM_00974	12043	Bcl2	AW986256
chr12	1.13E+08	1.13E+08	intron (NM	CpG-3536	248	NM_02127	12709	Ckb	B-CK|Bck|
chr3	89085127	89085525	promoter-T	promoter-T	-453	NM_00116	13636	Efna1	AI325262|
chr2	32115352	32115525	exon (NM_	exon (NM_	187	NM_01167	22245	Uck1	URK1|Ump
chr4	61926577	61926750	Intergenic	Intergenic	-20800	NM_02528	20530	Slc31a2	AI604396|
chr2	1.64E+08	1.64E+08	exon (NM_	exon (NM_	413	NM_13377	78928	Pigt	2510012P1
chr16	16066477	16066625	intron (NM	Lx8|LINE|L	80393	NM_14606	224008	Spidr	2310008HC
chr1	1.62E+08	1.62E+08	intron (NM	ID4|SINE|I	-2699	NM_00103	29809	Rabgap1l	5830411OC
chr2	34682227	34682300	promoter-T	promoter-T	-508	NM_00116	30050	Fbxw2	2700071L0
chr6	1.15E+08	1.15E+08	intron (NM	intron (NM	8587	NM_00112	19016	Pparg	Nr1c3|PPA

chr15	77759177	77759300	intron (NM	intron (NM	186	NM_01991	56551	Txn2	2510006J1
chr1	1.95E+08	1.95E+08	Intergenic	Intergenic	-1281	NM_00805	14373	G0s2	AI255151|/
chr7	88344002	88344175	Intergenic	MLT1L|LTF	-14602	NM_00880	18584	Pde8a	AI551852|I
chr1	1.58E+08	1.58E+08	Intergenic	Intergenic	-19197	NM_00102	104009	Qsox1	1300003H(
chr6	21908952	21909350	intron (NM	Lx9|LINE|L	9536	NM_02362	71777	Ing3	1300013A(
chr7	1.33E+08	1.33E+08	Intergenic	Intergenic	-20570	NM_02633	67711	Nsmce1	2510027N1
chr12	96987377	96987575	intron (NM	intron (NM	57040	NM_20151	399558	Flrt2	-
chr1	1.35E+08	1.35E+08	Intergenic	Intergenic	-7638	NM_00857	17248	Mdm4	4933417N(
chr16	84735277	84735500	intron (NM	intron (NM	159	NM_01740	27393	Mrpl39	C21orf8|M
chr17	65024352	65024575	exon (NM_	exon (NM_	73474	NM_00854	17158	Man2a1	Mana-2|M
chr18	9449677	9449900	5' UTR (NM	5' UTR (NM	360	NM_02648	67974	Ccny	1700025H1
chr2	1.15E+08	1.15E+08	intron (NM	intron (NM	311	NM_00114	399568	BC052040	-
chr2	44972302	44972375	Intergenic	Intergenic	-3539	NM_01575	24136	Zeb2	9130203F0
chr11	22908027	22908250	Intergenic	Intergenic	-5249	NM_02867	73873	Fam161a	4930430E1
chr15	79272052	79272775	promoter-T	promoter-T	74	NM_00128	27373	Csnk1e	AI426939|/
chr1	36125477	36125850	exon (NM_	exon (NM_	418	NM_01581	50785	Hs6st1	6Ost1
chr9	45625777	45625900	intron (NM	B3|SINE|B	10883	NM_00108	214552	Cep164	AI450905|I
chr11	54697752	54697850	Intergenic	B4|SINE|B	17861	NM_00824	15254	Hint1	AA673479|
chr9	16403677	16403950	Intergenic	Intergenic	-221138	NM_00108	270120	Fat3	9430076A(
chr17	25410327	25411475	promoter-T	promoter-T	-42	NM_00117	22196	Ube2i	5830467E0
chr15	79572527	79572675	promoter-T	promoter-T	365	NM_19434	223697	Sun2	B230369L0
chr9	75209977	75210300	Intergenic	Intergenic	14573	NM_01347	12049	Bcl2l10	AA420380|
chr17	74694777	74694875	promoter-T	promoter-T	-623	NM_13377	76890	Memo1	0610016J1
chr2	1.8E+08	1.8E+08	intron (NM	intron (NM	8517	NM_14450	228983	Osbpl2	C130070J1
chr2	38499402	38499475	promoter-T	promoter-T	-12	NM_01118	19177	Psmb7	AU020723
chr8	1.09E+08	1.09E+08	intron (NM	intron (NM	17988	NM_00922	20650	Sntb2	Snt2
chr2	1.21E+08	1.21E+08	intron (NM	CpG	140	NM_02689	68968	Cdan1	1500015A(
chr11	44332002	44332250	promoter-T	promoter-T	-340	NM_02886	74315	Rnf145	3732413I1
chr1	94926077	94926300	intron (NM	intron (NM	23277	NM_00115	208760	Aqp12	AB084104|
chr8	47060377	47060575	promoter-T	promoter-T	130	NM_00103	69479	1700029J0	-
chr14	99447902	99448000	intron (NM	intron (NM	2355	NM_17526	77744	Bora	6720463M
chr15	85188152	85188350	intron (NM	B1_Mur4|S	21440	NM_01684	54138	Atxn10	AI325283|(
chr13	1.12E+08	1.12E+08	promoter-T	promoter-T	-539	NM_00112	73274	Gpbp1	1700034P1
chr1	1.79E+08	1.79E+08	intron (NM	intron (NM	4297	NM_02975	76816	Sdccag8	2700048G2
chr12	1.01E+08	1.01E+08	Intergenic	Intergenic	-39329	NM_18318	71375	Foxn3	5430426H2
chr11	21629027	21629400	intron (NM	intron (NM	156929	NM_14542	216560	Wdpcp	AV249152
chr2	1.8E+08	1.8E+08	Intergenic	Intergenic	-23595	NM_14893	108115	Slco4a1	OATP-E|Slc
chr4	1.34E+08	1.34E+08	intron (NR_	intron (NR_	10680	NM_08055	73723	Sh3bgrl3	1110004L0
chr11	98657577	98658050	exon (NM_	exon (NM_	730	NM_02872	74026	Msl1	2810017F1
chr13	89967577	89967650	Intergenic	Intergenic	-85496	NM_00113	13003	Vcan	5430420N(
chr9	1.24E+08	1.24E+08	Intergenic	Intergenic	34373	NM_00991	12771	Ccr3	CC-CKR3|C
chr14	9005077	9005175	intron (NM	CpG	380	NM_02422	68263	Pdhb	2610103L0
chr11	1.03E+08	1.03E+08	intron (NM	intron (NM	433	NM_17780	328019	Spata32	4933400C(
chr17	70920202	70920475	intron (NM	intron (NM	9251	NM_00112	224997	Dlgap1	4933422O1
chr9	45792102	45792175	intron (NM	intron (NM	816	NM_00877	18475	Pafah1b2	AI747451|/
chr16	55790552	55790650	Intergenic	Intergenic	31650	NM_00115	80859	Nfkbiz	AA408868|
chr4	1.5E+08	1.5E+08	intron (NM	intron (NM	68582	NM_17377	242773	Slc45a1	C230078B2

chr13	1.13E+08	1.13E+08	intron (NM	intron (NM	12365	NM_01194	26401	Map3k1	MAPKKK1|
chr11	1.19E+08	1.19E+08	Intergenic	CpG	-17686	NM_01392	30951	Cbx8	Pc3
chr11	51670802	51671000	promoter-T	promoter-T	82	NM_19929	76901	Jade2	1200017K0
chr2	71625027	71625225	5' UTR (NM	5' UTR (NM	130	NM_00127	16403	Itga6	5033401O0
chr17	56147002	56147075	Intergenic	Intergenic	-1586	NM_02653	68047	Mpnd	E130307M0
chr13	95746252	95746575	5' UTR (NM	5' UTR (NM	114	NM_17259	218460	Wdr41	B830029I0
chr8	72241177	72241425	promoter-T	promoter-T	-500	NM_00103	66869	Zfp869	1200003I0
chr9	64119002	64119150	Intergenic	Intergenic	-10338	NM_02537	66131	Tipin	1110005A0
chr9	1E+08	1E+08	Intergenic	Intergenic	47054	NM_00103	213208	Il20rb	AV228068|
chr5	1.44E+08	1.44E+08	intron (NM	intron (NM	22590	NM_17824	231861	Tnrc18	BC025631|
chr11	5607452	5607625	exon (NM_	exon (NM_	164	NM_02608	64660	Mrps24	3110030K2
chr1	72275627	72275825	Intergenic	Intergenic	-16845	NM_00100	381269	Mreg	Gm974|W
chr7	1.34E+08	1.34E+08	Intergenic	Intergenic	-2174	NM_17890	101602	AI467606	-
chr17	22498577	22498675	promoter-T	promoter-T	206	NM_14548	224598	Zfp758	BC021442
chr2	1.02E+08	1.02E+08	intron (NM	CpG	1088	NM_17518	72446	Prr5l	2600010E0
chr7	76243752	76243875	Intergenic	Intergenic	-349689	NM_02554	66412	Arrdc4	2410003C0
chr2	37286627	37286700	promoter-T	promoter-T	-224	NM_14625	241322	Zbtb6	A830092L0
chr19	58942302	58942425	Intergenic	RMER19A|	-6889	NM_17519	73442	Hspa12a	1700063D1
chr6	1.2E+08	1.2E+08	intron (NM	intron (NM	7711	NM_00835	16172	Il17ra	AW538159
chr4	86221277	86221525	promoter-T	promoter-T	-176	NM_17837	68441	Rraga	1300010C1
chr4	8672252	8672400	intron (NM	intron (NM	54773	NM_00127	320790	Chd7	A730019I0
chr11	48612852	48615025	promoter-T	promoter-T	76	NM_00814	14694	Gnb2l1	AL033335|
chr4	88385052	88385200	Intergenic	MTEa|LTR|	-15646	NM_00108	435791	Gm13271	Ifnz|OTTM
chr12	53415827	53416050	Intergenic	Intergenic	-201126	NM_00970	11855	Arhgap5	p190-B|p1
chr4	1.55E+08	1.55E+08	Intergenic	Intergenic	-2482	NM_00116	14688	Gnb1	AA409223|
chr8	77727377	77727500	Intergenic	Intergenic	-10405	NM_02918	75141	Rasd2	4930526B1
chr17	18027152	18027250	intron (NM	intron (NM	2413	NM_00803	14289	Fpr2	E330010I0
chr15	59479052	59479125	Intergenic	CpG	-1121	NM_14454	211770	Trib1	A530090O
chr13	51739377	51739725	Intergenic	Intergenic	-1050	NM_02541	66197	Cks2	1110038L1
chr5	38430252	38430650	promoter-T	promoter-T	-18	NM_00128	71116	Stx18	1810035L2
chr2	1.64E+08	1.64E+08	Intergenic	Intergenic	-16308	NM_01141	20568	Slpi	-
chr15	25199202	25199350	Intergenic	Intergenic	144243	NM_02739	70350	Basp1	2610024P1
chr8	1.08E+08	1.08E+08	intron (NM	intron (NM	15859	NM_02648	67971	Tppp3	2700055K0
chr3	1.21E+08	1.21E+08	intron (NM	intron (NM	9657	NM_00103	619318	4930432M	-
chr13	91819777	91820200	intron (NM	intron (NM	-61138	NM_02879	74156	Acot12	1300004O0
chr1	1.84E+08	1.84E+08	intron (NM	intron (NM	2819	NM_13381	98386	Lbr	AI505894|
chr7	28459327	28460175	promoter-T	promoter-T	71	NM_19841	112415	C030039L0	-
chr16	35769202	35769300	exon (NM_	exon (NM_	191	NM_15355	224132	Dirc2	RCC4
chr5	1.15E+08	1.15E+08	promoter-T	promoter-T	-334	NM_00100	433940	Fam222a	-
chr11	75980452	75980725	intron (NM	intron (NM	12544	NM_02666	68299	Vps53	2010002A0
chr17	79754852	79755100	promoter-T	promoter-T	-545	NM_02651	260409	Cdc42ep3	3200001F0
chr6	1.21E+08	1.21E+08	intron (NM	CpG	140	NM_15351	94044	Bcl2l13	BCL-RAMB
chr11	79902777	79903225	promoter-T	promoter-T	99	NM_00102	237877	Atad5	C130052G0
chr16	17209977	17210125	exon (NM_	exon (NM_	1823	NM_00103	239731	Rimbp3	Gm1759|G
chr1	26653527	26653800	Intergenic	Intergenic	90642	NM_00103	210940	4931408C2	-
chr13	16908702	16908875	Intergenic	Intergenic	802480	NM_00838	16323	Inhba	-
chr4	1.36E+08	1.36E+08	intron (NM	intron (NM	9306	NM_00127	74326	Hnrnpr	2610003J0

chr5	77524102	77524175	5' UTR (NM	5' UTR (NM	154	NM_00115	74318	Hopx	1110018K1
chr5	20637677	20638050	Intergenic	MIRm|SINI	-54222	NM_17543	212167	Gsap	A530088I0
chr1	55085727	55085825	Intergenic	Intergenic	-1454	NM_03117	81898	Sf3b1	155kDa|28
chr3	1.22E+08	1.22E+08	Intergenic	MT2A|LTR	-15730	NM_00899	19299	Abcd3	AI313901|/
chr16	30969827	30969975	intron (NM	intron (NM	111617	NM_19862	268880	Xxylt1	AI480653
chr13	23852202	23852300	TTS (NM_1	TTS (NM_1	867	NM_17819	326619	Hist1h4a	-
chr3	1.33E+08	1.33E+08	promoter-T	promoter-T	178	NM_02623	67553	Gstcd	4933434L1
chr7	1.29E+08	1.29E+08	Intergenic	Intergenic	-32251	NM_00885	18751	Prkcb	A130082F0
chr15	59205527	59205675	promoter-T	promoter-T	106	NM_15354	223593	E430025E2	AL022848|
chr12	1.13E+08	1.13E+08	Intergenic	Intergenic	-13111	NM_17804	217869	Eif5	2810011H2
chr17	73456702	73456800	promoter-T	promoter-T	-574	NM_00117	225010	Lclat1	AI181996|/
chr9	64644027	64644300	Intergenic	Intergenic	-14655	NM_00116	102442	Dennd4a	AI115600|/
chr10	66998202	66998475	Intergenic	CpG	-2279	NM_01011	13654	Egr2	Egr-2|Krox
chr11	1.01E+08	1.01E+08	promoter-T	promoter-T	-363	NM_02930	75482	Hspb9	1700007H2
chr16	32177752	32177825	Intergenic	Intergenic	-2098	NM_00103	328660	Bex6	B020003O0
chr9	25293977	25294075	intron (NM	intron (NM	4844	NM_02618	67484	Eepd1	2310005P0
chr11	1.16E+08	1.16E+08	promoter-T	promoter-T	-268	NM_02710	69535	Ten1	2310004N2
chr3	60214252	60214400	Intergenic	Intergenic	-62426	NM_00125	56758	Mbnl1	Mbnl|mKIA
chr1	1.27E+08	1.27E+08	Intergenic	Intergenic	212403	NM_02373	74117	Actr3	1200003A0
chr1	88422252	88422850	promoter-T	promoter-T	-760	NM_00897	19231	Ptma	Thym
chr10	12594627	12594825	Intergenic	Intergenic	-13193	NM_01168	22288	Utrn	AA589569|
chr4	1.09E+08	1.09E+08	promoter-T	promoter-T	-181	NM_00798	14084	Faf1	AA408698|
chr2	32236652	32237175	intron (NM	intron (NM	522	NM_19800	73737	1110008P1	C79326
chr8	72426652	72427100	promoter-T	promoter-T	-419	NM_02331	67184	Ndufa13	2700054G1
chr9	65528777	65528975	intron (NM	intron (NM	4521	NM_00130	18247	Oaz2	AZ-2|AZ2|(
chr16	32002952	32003200	intron (NM	CpG	297	NM_17710	320213	Senp5	6230429P1
chr3	10322577	10322825	intron (NM	intron (NM	8738	NM_01886	55980	Impa1	2610002K0
chr8	1.26E+08	1.26E+08	intron (NR_	intron (NR_	389	NR_02826(	114896	Afg3l1	1700047G0
chr15	76199477	76199650	exon (NM_	exon (NM_	235	NM_02155	59053	Hgh1	Brp16|D15
chr5	1.4E+08	1.4E+08	exon (NM_	exon (NM_	130	NM_17272	231821	Adap1	4930431P1
chr7	1.06E+08	1.06E+08	Intergenic	Intergenic	-6128	NM_02638	67800	Dgat2	0610010B0
chr4	1.3E+08	1.3E+08	intron (NM	intron (NM	5063	NM_02644	67898	Pef1	2600002E2
chr9	42079677	42079850	Intergenic	Intergenic	-7380	NM_17276	235293	Sc5d	A830037K0
chr17	26013227	26013325	intron (NM	intron (NM	831	NM_02668	68347	0610011F0	-
chr5	1.22E+08	1.22E+08	intron (NM	CpG	228	NM_01129	19988	Rpl6	Taxreb107
chr3	1.3E+08	1.3E+08	intron (NM	CpG	363	NM_02657	68147	Gar1	AA409823|
chr9	64064427	64064675	intron (NM	intron (NM	-18457	NM_03360	93841	Uchl4	-
chr6	1.2E+08	1.2E+08	intron (NM	intron (NM	79228	NM_01671	29862	Ninj2	-
chr13	23677052	23677125	TTS (NM_0	TTS (NM_0	751	NM_00129	319179	Hist1h2be	-
chr2	1.03E+08	1.03E+08	intron (NM	intron (NM	73968	NM_00107	20511	Slc1a2	1700091C1
chr1	90598802	90598975	promoter-T	promoter-T	-122	NM_17730	320982	Arl4c	A630084M
chr18	10617852	10618125	intron (NM	CpG	194	NM_00922	20641	Snrpd1	AA407109|
chr15	99495427	99495600	Intergenic	Intergenic	-5636	NM_00959	11419	Asic1	AI843610|/
chr6	17355477	17355700	Intergenic	Intergenic	-58369	NM_00859	17295	Met	AI838057|I
chr13	35555152	35555425	Intergenic	Intergenic	-196443	NM_00988	12593	Cdyl	AI325931
chr3	1.33E+08	1.33E+08	promoter-T	promoter-T	-459	NM_00104	214133	Tet2	Ayu17-449
chr6	1.29E+08	1.29E+08	Intergenic	Intergenic	-8720	NM_05310	93694	Clec2d	Clr-b|Clrb|

chr2	30093427	30093500	promoter-T	promoter-T	174	NM_17772	241296	Lrrc8a	Lrrc8|mKIA
chr1	97365427	97365675	Intergenic	Intergenic	155346	NM_02632	67698	Fam174a	2310044D2
chr1	84314352	84314625	Intergenic	Intergenic	-33268	NM_00100	98496	Pid1	5033414K0
chrX	7576102	7576200	Intergenic	Intergenic	19467	NM_02722	69824	Glod5	2010001H1
chr18	21119927	21120000	intron (NM	CpG	16837	NM_02630	67664	Rnf125	4930553F0
chr4	1.36E+08	1.36E+08	intron (NM	intron (NM	7142	NM_17773	242705	E2f2	9230110J1
chr14	55496252	55496400	promoter-T	promoter-T	49	NM_00116	105651	Ppp1r3e	A630071A1
chr16	33836877	33836975	intron (NM	intron (NM	7175	NM_00114	16419	Itgb5	AA475909|
chr5	52747077	52747250	Intergenic	L1MB5|LIN	-7880	NM_01143	20657	Sod3	AI314465|
chr2	1.54E+08	1.54E+08	5' UTR (NM	5' UTR (NM	124	NM_00129	13555	E2f1	E2F-1|mKIA
chr8	1.08E+08	1.08E+08	exon (NM_	exon (NM_	765	NM_00127	97440	B3gnt9	3-Gn-T9|B3

Gene Desc	Gene Type
RIKEN cDN	protein-coding
zinc finger	protein-coding
protein ph	protein-coding
cystatin B	protein-coding
timeless in	protein-coding
jagged 1	protein-coding
ATPase, an	protein-coding
protein tyr	protein-coding
single-stra	protein-coding
chromobo	protein-coding
lymphocyt	protein-coding
X-ray radia	protein-coding
succinate c	protein-coding
ribulose-5-	protein-coding
thymus cel	protein-coding
coagulatio	protein-coding
RIKEN cDN	protein-coding
Fas ligand (	protein-coding
solute carr	protein-coding
forkhead b	protein-coding
low density	protein-coding
septin 2	protein-coding
PEST prote	protein-coding
myeloblast	protein-coding
protein kin	protein-coding
dual specif	protein-coding
family with	protein-coding
coiled-coil	protein-coding
activin rec	protein-coding
BH3 intera	protein-coding
formin-like	protein-coding
deoxythym	protein-coding
serine/thre	protein-coding
zinc finger	protein-coding
solute carr	protein-coding
SUMO/sen	protein-coding
DnaJ (Hsp4	protein-coding
Friend leuk	protein-coding
DNA segm	protein-coding
KH domain	protein-coding
NADH deh	protein-coding
family with	protein-coding
Aly/REF ex	protein-coding
DENN/MA	protein-coding
polynucleo	protein-coding

dpy-19-like	protein-coding
fasciculatic	protein-coding
cathepsin 3	protein-coding
family with	protein-coding
ribosomal	protein-coding
CUGBP, Ela	protein-coding
golgi phosp	protein-coding
low density	protein-coding
CAP-GLY dc	protein-coding
negative re	protein-coding
RAB23, me	protein-coding
UDP-glucos	protein-coding
mitochond	protein-coding
arachidona	protein-coding
glutathione	protein-coding
SPEG comp	protein-coding
sterol-C5-d	protein-coding
WEE 1 hom	protein-coding
F-box and l	protein-coding
nucleopori	protein-coding
tyrosine 3-	protein-coding
mitochond	protein-coding
spastin	protein-coding
sorting nex	protein-coding
DMRT-like	protein-coding
Rho guanin	protein-coding
tyrosine 3-	protein-coding
tubulin, alp	protein-coding
DNA segme	protein-coding
nucleolar p	protein-coding
calcyclin bi	protein-coding
TNF recept	protein-coding
dicarbonyl	protein-coding
heterogene	protein-coding
myotrophi	protein-coding
GC-rich prc	protein-coding
DEP domai	protein-coding
solute carri	protein-coding
solute carri	protein-coding
casein kina	protein-coding
programme	protein-coding
insulin indu	protein-coding
tubby like	protein-coding
COP9 (cons	protein-coding
3-hydroxyr	protein-coding
tyrosine kir	protein-coding
ring finger	protein-coding

nucleolar p	protein-coding
RIKEN cDN.	protein-coding
RIO kinase	protein-coding
zona pelluc	protein-coding
heparan su	protein-coding
tigger trans	protein-coding
ST8 alpha-I	protein-coding
frizzled hoı	protein-coding
tRNA selen	protein-coding
free fatty a	protein-coding
proteasom	protein-coding
transforme	protein-coding
biliverdin r	protein-coding
zinc finger	protein-coding
RIKEN cDN.	protein-coding
solute carri	protein-coding
ring finger	protein-coding
family with	protein-coding
mediator c	protein-coding
prothymos	protein-coding
ATPase typ	protein-coding
ribosomal	protein-coding
progestin a	protein-coding
myotubula	protein-coding
tec protein	protein-coding
aurora kina	protein-coding
protease, s	protein-coding
deoxyuridi	protein-coding
tumor supp	protein-coding
zinc finger	protein-coding
EF-hand ca	protein-coding
solute carri	protein-coding
TBC1 doma	protein-coding
immunoglo	protein-coding
methyl-Cp(	protein-coding
RAB6A, me	protein-coding
BCL2-like 2	protein-coding
low density	protein-coding
phosphatic	protein-coding
ELOVL fam	protein-coding
chemokine	protein-coding
proteasom	protein-coding
inositol 1,4	protein-coding
signal trans	protein-coding
synaptotag	protein-coding
cAMP resp	protein-coding
cathepsin (	protein-coding

interleukin	protein-coding
regulator o	protein-coding
plexin A4	protein-coding
tubulin, be	protein-coding
ADP-ribosy	protein-coding
aldo-keto r	protein-coding
biorientatio	protein-coding
RAN GTPas	protein-coding
nuclear fac	protein-coding
actin relate	protein-coding
ABO blood	protein-coding
antizyme ir	protein-coding
ribosomal	protein-coding
protein ser	protein-coding
regulator o	protein-coding
epilepsy, p	protein-coding
ceramide s	protein-coding
annexin A1	protein-coding
Rho guanin	protein-coding
guanine nu	protein-coding
MYC bindir	protein-coding
mitochond	protein-coding
ATP-bindin	protein-coding
retinaldehy	protein-coding
solute carri	protein-coding
adaptor-re	protein-coding
fibronectin	protein-coding
endothelia	protein-coding
family with	protein-coding
golgi integr	protein-coding
URI1, prefo	protein-coding
RIKEN cDN	protein-coding
eukaryotic	protein-coding
AT rich inte	protein-coding
clathrin int	protein-coding
ARP3 actin	protein-coding
ADP-ribosy	protein-coding
protein pho	protein-coding
Rho guanin	protein-coding
B cell leuke	protein-coding
transmemb	protein-coding
male-speci	protein-coding
family with	protein-coding
calcium/ca	protein-coding
transducin	protein-coding
contactin a	protein-coding
leucine rich	protein-coding

bridging int	protein-coding
proteasom	protein-coding
TBC1 doma	protein-coding
RIKEN cDN.	protein-coding
centrosom	protein-coding
ribosomal p	protein-coding
nudix (nucl	protein-coding
HNF1 hom	protein-coding
vav 3 onco	protein-coding
torsin A int	protein-coding
pleckstrin h	protein-coding
tRNA splici	protein-coding
PHD finger	protein-coding
formin-like	protein-coding
tripartite m	protein-coding
RRS1 ribos	protein-coding
TSR2 20S rI	protein-coding
RAP2B, me	protein-coding
phosphatas	protein-coding
heterogene	protein-coding
toll-like rec	protein-coding
polymerase	protein-coding
predicted g	protein-coding
KAT8 regul	protein-coding
attractin lik	protein-coding
lysine (K)-s	protein-coding
6-phospho	protein-coding
RNA bindin	protein-coding
circadian a	protein-coding
Sad1 and U	protein-coding
kelch-like 2	protein-coding
phosphatic	protein-coding
K(lysine) ac	protein-coding
RIKEN cDN.	protein-coding
NME/NM2	protein-coding
solute carri	protein-coding
family with	protein-coding
predicted g	protein-coding
DNA segme	protein-coding
zinc finger	protein-coding
oxidative st	protein-coding
myosin VA	protein-coding
signal reco	protein-coding
solute carri	protein-coding
heat shock	protein-coding
ring finger	protein-coding
heparin-bir	protein-coding

lysosomal-a	protein-coding
adiponectii	protein-coding
Cnksr famil	protein-coding
echinoderr	protein-coding
zinc finger	protein-coding
calcium/ca	protein-coding
apoptosis-a	protein-coding
PDGFA asso	protein-coding
arylsulfatas	protein-coding
ubiquilin 4	protein-coding
coiled-coil	protein-coding
ets variant	protein-coding
karyopheri	protein-coding
free fatty a	protein-coding
polymerase	protein-coding
MOB kinas	protein-coding
protein tyr	protein-coding
thymoma v	protein-coding
serine palm	protein-coding
mitochond	protein-coding
zinc finger	protein-coding
glycoprotei	protein-coding
ATP-bindin	protein-coding
spleen tyro	protein-coding
NIMA (neve	protein-coding
vacuolar pr	protein-coding
BCL2-like 1	protein-coding
ligand depe	protein-coding
growth arre	protein-coding
phosphose	protein-coding
carbonic ar	protein-coding
meiosis spe	protein-coding
olfactomec	protein-coding
ER membra	protein-coding
growth fac	protein-coding
integrator	protein-coding
folylpolyglu	protein-coding
ring finger	protein-coding
ornithine a	protein-coding
thymocyte	protein-coding
prostate tr	protein-coding
uveal auto	protein-coding
PHD finger	protein-coding
male-speci	protein-coding
zinc finger	protein-coding
RING1 and	protein-coding
selectin, lyr	protein-coding

zinc finger	protein-coding
activating t	protein-coding
versican	protein-coding
ArfGAP wit	protein-coding
polymerase	protein-coding
FXYD doma	protein-coding
peptidase (	protein-coding
nuclear rec	protein-coding
vascular er	protein-coding
hypocretin	protein-coding
CD9 antige	protein-coding
high mobili	protein-coding
carnitine d	protein-coding
X Kell blooc	protein-coding
RING1 and	protein-coding
Ras associa	protein-coding
calpain 10	protein-coding
septin 8	protein-coding
tumor necr	protein-coding
INTS3 and	protein-coding
autophagy	protein-coding
methionine	protein-coding
Kruppel-lik	protein-coding
5'-3' exorib	protein-coding
chemokine	protein-coding
interleukin	protein-coding
doublecort	protein-coding
sine oculis-	protein-coding
transforme	protein-coding
predicted g	protein-coding
histone dea	protein-coding
TBC1 doma	protein-coding
serine/thre	protein-coding
polyhomeo	protein-coding
ring finger	protein-coding
ArfGAP wit	protein-coding
predicted g	protein-coding
toll-like rec	protein-coding
Abelson he	protein-coding
transportin	protein-coding
DENN/MAI	protein-coding
tumor prot	protein-coding
calcium cha	protein-coding
transmemb	protein-coding
KH domain	protein-coding
DDHD dom	protein-coding
H2A histon	protein-coding

transmemb	protein-coding
glutathione	protein-coding
KDEL (Lys-A	protein-coding
Kruppel-lik	protein-coding
lysine (K)-s	protein-coding
cullin assoc	protein-coding
transportin	protein-coding
phosphodie	protein-coding
WD repeat	protein-coding
muscleblin	protein-coding
syntrophin,	protein-coding
peroxisoma	protein-coding
sorting nex	protein-coding
cerebellar (	protein-coding
interleukin	protein-coding
ubiquitin-c	protein-coding
DDHD dom	protein-coding
biotinidase	protein-coding
dynein, axo	protein-coding
akirin 2	protein-coding
core 1 synt	protein-coding
syntaxin 2	protein-coding
disabled 2,	protein-coding
nuclear ass	protein-coding
Grb2-bindi	protein-coding
serine (or c	protein-coding
histone dea	protein-coding
CASP8 and	protein-coding
family with	protein-coding
guanine nu	protein-coding
t-complex 1	protein-coding
ELAV (emb	protein-coding
RAB3C, me	protein-coding
EH-domain	protein-coding
serine/thre	protein-coding
zinc fingers	protein-coding
striatin, cal	protein-coding
charged mu	protein-coding
family with	protein-coding
septin 12	protein-coding
ST6 (alpha-	protein-coding
C1GALT1-s	protein-coding
c-src tyrosi	protein-coding
kelch doma	protein-coding
neural prec	protein-coding
cleavage ar	protein-coding
solute carri	protein-coding

zinc finger	protein-coding
AT rich inte	protein-coding
splicing fac	protein-coding
cysteinyl-tF	protein-coding
protein pho	protein-coding
dipeptidylp	protein-coding
Fanconi an	protein-coding
nuclear rec	protein-coding
BCL2-like 1	protein-coding
phosphorib	protein-coding
neuregulin	protein-coding
T cell acute	protein-coding
enoyl Coen	protein-coding
COP9 (cons	protein-coding
guanosine	protein-coding
CD47 antig	protein-coding
ankyrin rep	protein-coding
solute carri	protein-coding
6-phospho	protein-coding
small nucle	protein-coding
hemolytic c	protein-coding
ATP-bindin	protein-coding
WD repeat	protein-coding
glutamate	protein-coding
RAS-relate	protein-coding
tryptophar	protein-coding
arginine va	protein-coding
nucleopori	protein-coding
flavin conta	protein-coding
replication	protein-coding
transmemb	protein-coding
thyroid hor	protein-coding
ERO1-like (	protein-coding
collagen-lik	protein-coding
claudin 17	protein-coding
peptidogly	protein-coding
recombina	protein-coding
RIKEN cDN	protein-coding
lysine (K)-s	protein-coding
nuclear fac	protein-coding
karyopheri	protein-coding
serum/gluc	protein-coding
family with	protein-coding
Max intera	protein-coding
a disintegri	protein-coding
myosin reg	protein-coding
tet methyl	protein-coding

transmemb	protein-coding
neural prec	protein-coding
pleckstrin h	protein-coding
LYR motif c	protein-coding
NEDD8 acti	protein-coding
prostaglan	protein-coding
sarcoglycar	protein-coding
ankyrin rep	protein-coding
processing	protein-coding
tripartite m	protein-coding
regulator o	protein-coding
ring finger	protein-coding
period circa	protein-coding
eukaryotic	protein-coding
heterogene	protein-coding
leucine rich	protein-coding
male-speci	protein-coding
placenta ex	protein-coding
nuclear fac	protein-coding
UDP-Gal:be	protein-coding
COBW dom	protein-coding
CDC16 cell	protein-coding
coagulation	protein-coding
forkhead b	protein-coding
fibronectin	protein-coding
MORN repe	protein-coding
olfactory re	protein-coding
isocitrate d	protein-coding
torsin fami	protein-coding
CD180 anti	protein-coding
exonucleas	protein-coding
cholinergic	protein-coding
exocyst cor	protein-coding
suppressor	protein-coding
SEC22 vesic	protein-coding
RNA 2',3'-c	protein-coding
D-tyrosyl-tl	protein-coding
family with	protein-coding
ATPase, Na	protein-coding
lipin 1	protein-coding
phosphorib	protein-coding
homeobox	protein-coding
RAD51 hom	protein-coding
bone morp	protein-coding
glypican 1	protein-coding
ribosomal	protein-coding
retinoic aci	protein-coding

potassium	protein-coding
RIKEN cDN.	protein-coding
protein ph	protein-coding
growth arr	protein-coding
myosin IE	protein-coding
mannosida	protein-coding
chimerin 2	protein-coding
chemokine	protein-coding
CDC42 effe	protein-coding
cell divisior	protein-coding
kelch-like 3	protein-coding
delta-like 2	protein-coding
ADP-ribosy	protein-coding
stathmin 1	protein-coding
DDB1 and (	protein-coding
phosphofru	protein-coding
glutamic py	protein-coding
pecanex-lik	protein-coding
alanyl-tRN/	protein-coding
dynamin bi	protein-coding
RIKEN cDN.	protein-coding
ARP1 actin-	protein-coding
glutaredox	protein-coding
lymphocyte	protein-coding
suppressor	protein-coding
quinolinate	protein-coding
E74-like fac	protein-coding
progestero	protein-coding
solute carri	protein-coding
lipase, end	protein-coding
tripartite m	protein-coding
adaptor pr	protein-coding
zinc finger	protein-coding
S100 calciu	protein-coding
protease, s	protein-coding
solute carri	protein-coding
ribonucleas	protein-coding
regulator o	protein-coding
solute carri	protein-coding
purine-nuc	protein-coding
resistin	protein-coding
RAD50 hon	protein-coding
solute carri	protein-coding
solute carri	protein-coding
xanthine de	protein-coding
delta/notcl	protein-coding
lectin, gala	protein-coding

SUMO-inte	protein-coding
ADP-ribosy	protein-coding
G patch do	protein-coding
predicted g	protein-coding
WD repeat	protein-coding
galactosida	protein-coding
phosphata:	protein-coding
BTAF1 RNA	protein-coding
mitochond	protein-coding
NEDD4 bin	protein-coding
myelin pro	protein-coding
ankyrin 3, e	protein-coding
macrophag	protein-coding
coagulatio	protein-coding
transient re	protein-coding
RAB14, me	protein-coding
SEH1-like (!	protein-coding
zinc finger	protein-coding
Sp110 nucl	protein-coding
RIKEN cDN,	protein-coding
HORMA do	protein-coding
CCR4-NOT	protein-coding
DNA prima	protein-coding
cadherin 4	protein-coding
mediator c	protein-coding
serine/argi	protein-coding
olfactory re	protein-coding
Sp2 transcı	protein-coding
src homolo	protein-coding
crystallin, g	protein-coding
heterogene	protein-coding
craniofacia	protein-coding
reticulon 4	protein-coding
insulin-like	protein-coding
ERBB recep	protein-coding
bromodom	protein-coding
predicted g	protein-coding
polymerase	protein-coding
glucosamir	protein-coding
SET domair	protein-coding
zinc finger	protein-coding
leucine zipı	protein-coding
coiled-coil	protein-coding
sorting nex	protein-coding
tudor and I	protein-coding
related RA!	protein-coding
centrosom	protein-coding

Sad1 and U	protein-coding
mitochond	protein-coding
receptor ac	protein-coding
zinc finger	protein-coding
transmemb	protein-coding
TRAF-intera	protein-coding
male germ	protein-coding
molybdenu	protein-coding
retinol deh	protein-coding
cell divisior	protein-coding
protein inh	protein-coding
suppressor	protein-coding
phosphory	protein-coding
DnaJ (Hsp4	protein-coding
ring finger	protein-coding
RIKEN cDN	protein-coding
RNA bindin	protein-coding
protein pho	protein-coding
major facili	protein-coding
protein tyr	protein-coding
zinc finger	protein-coding
camello-lik	protein-coding
SAM doma	protein-coding
tubulin tyr	protein-coding
RIKEN cDN	protein-coding
PAP associa	protein-coding
solute carri	protein-coding
split hand/	protein-coding
ATPase, an	protein-coding
solute carri	protein-coding
N-acyletha	protein-coding
zinc finger	protein-coding
coiled-coil	protein-coding
protein kin	protein-coding
calpain 3	protein-coding
O-6-methy	protein-coding
ets variant	protein-coding
zinc finger	protein-coding
eukaryotic	protein-coding
dual specif	protein-coding
RIKEN cDN	protein-coding
zinc finger	protein-coding
Bloom sync	protein-coding
UDP-GlcNA	protein-coding
guanylate b	protein-coding
centromere	protein-coding
Fc receptor	protein-coding

DNA segme	protein-coding
MKL (mega	protein-coding
stearoyl-Co	protein-coding
nuclear exp	protein-coding
H2A histon	protein-coding
transformi	protein-coding
solute carri	protein-coding
cytochrom	protein-coding
serological	protein-coding
family with	protein-coding
syntrophin	protein-coding
synemin, in	protein-coding
cathepsin (	protein-coding
glycerol-3-p	protein-coding
predicted g	protein-coding
ninjurin 1	protein-coding
predicted g	protein-coding
hemochron	protein-coding
calmodulin	protein-coding
programm	protein-coding
RIKEN cDN	protein-coding
pleckstrin h	protein-coding
junction ad	protein-coding
CCR4 carbo	protein-coding
peroxisoma	protein-coding
zinc finger	protein-coding
arginine/se	protein-coding
neural prec	protein-coding
YdjC homo	protein-coding
triple QxxK	protein-coding
olfactomec	protein-coding
DENN/MAI	protein-coding
ubiquitin sp	protein-coding
CUE domai	protein-coding
BCL2 bindi	protein-coding
SNF8, ESCR	protein-coding
mitochond	protein-coding
coiled-coil	protein-coding
ubiquitin-li	protein-coding
steroid 5 al	protein-coding
MLX intera	protein-coding
glutathione	protein-coding
Vac14 hom	protein-coding
B and T lym	protein-coding
discs, large	protein-coding
tripartite m	protein-coding
microsoma	protein-coding

stathmin d	protein-coding
RAN bindin	protein-coding
ubiquitin-f	protein-coding
phosphatic	protein-coding
superoxide	protein-coding
nitrilase fa	protein-coding
glutaryl-Co	protein-coding
UDP-N-ace	protein-coding
family with	protein-coding
transmeml	protein-coding
poly-U binc	protein-coding
TCDD-indu	protein-coding
SAM doma	protein-coding
bleomycin	protein-coding
RNA bindin	protein-coding
Ras and Ra	protein-coding
nucleopori	protein-coding
cDNA sequ	protein-coding
family with	protein-coding
nuclear rec	protein-coding
brain prote	protein-coding
cryptochro	protein-coding
guanine nu	protein-coding
YLP motif c	protein-coding
forkhead b	protein-coding
clavesin 1	protein-coding
TRAF famil	protein-coding
SCO cytoch	protein-coding
ST8 alpha-I	protein-coding
zinc finger	protein-coding
farnesyl dip	protein-coding
dehydroge	protein-coding
CD93 antig	protein-coding
FBJ osteosa	protein-coding
cyclin-depe	protein-coding
polycystic I	protein-coding
acyloxyacy	protein-coding
growth fac	protein-coding
pogo trans	protein-coding
premature	protein-coding
zinc finger	protein-coding
glycerol kir	protein-coding
glycerol kir	protein-coding
proteasom	protein-coding
NADH dehy	protein-coding
sema doma	protein-coding
tumor necr	protein-coding

PR domain	protein-coding
DNA cross-	protein-coding
solute carri	protein-coding
zinc finger	protein-coding
autophagy	protein-coding
solute carri	protein-coding
zinc finger	protein-coding
coiled-coil	protein-coding
WD repeat	protein-coding
STIP1 hom	protein-coding
SH3-domai	protein-coding
testis speci	protein-coding
glycerophо	protein-coding
interferon	protein-coding
zinc finger	protein-coding
nucleopori	protein-coding
Fez family	protein-coding
cell divisior	protein-coding
bactericida	protein-coding
cysteine-ric	protein-coding
glucoside x	protein-coding
insulin-like	protein-coding
nrde-2 nec	protein-coding
mannoside	protein-coding
NHL repeat	protein-coding
uridine-cyt	protein-coding
transcriptic	protein-coding
cytotoxic g	protein-coding
DCN1, defe	protein-coding
protein pho	protein-coding
CLIP associ	protein-coding
nucleophos	protein-coding
bromodom	protein-coding
zinc finger	protein-coding
family with	protein-coding
transmemk	protein-coding
thyroid hor	protein-coding
SH2 domai	protein-coding
golgi assoc	protein-coding
serine/thre	protein-coding
phosphatic	protein-coding
zinc finger,	protein-coding
xenotropic	protein-coding
predicted g	protein-coding
biogenesis	protein-coding
SET domair	protein-coding
inositol (m	protein-coding

zinc finger	protein-coding
CDKN2A in	protein-coding
polymerase	protein-coding
carbohydra	protein-coding
ankyrin rep	protein-coding
cleavage ar	protein-coding
DPH5 hom	protein-coding
BCL2-like 1	protein-coding
kelch-like 5	protein-coding
RIKEN cDN	protein-coding
Fc receptor	protein-coding
C-type lect	protein-coding
cyclin-depe	protein-coding
phosphatic	protein-coding
transducin-	protein-coding
NK6 home	protein-coding
serine-rich	protein-coding
DIS3 mitoti	protein-coding
exocyst co	protein-coding
mannosida	protein-coding
FAT tumor	protein-coding
cytochrom	protein-coding
adiponecti	protein-coding
pinin	protein-coding
reticulon 4	protein-coding
cyclin-depe	protein-coding
cytochrom	protein-coding
mitochond	protein-coding
zinc finger	protein-coding
leucine zip	protein-coding
folliculin in	protein-coding
a disintegri	protein-coding
glucose ph	protein-coding
protein-tyr	protein-coding
B cell CLL/l	protein-coding
syntaxin 12	protein-coding
kinesin fam	protein-coding
phosphoglu	protein-coding
AT rich inte	protein-coding
ras homolo	protein-coding
human imr	protein-coding
active BCR-	protein-coding
zinc finger	protein-coding
enkurin do	protein-coding
DCP1 deca	protein-coding
ribosomal	protein-coding
armadillo r	protein-coding

phosphatic	protein-coding
wings apar	protein-coding
mitochond	protein-coding
transmeml	protein-coding
serine/thre	protein-coding
ORAI calciu	protein-coding
MOB family	protein-coding
patatin-like	protein-coding
eukaryotic	protein-coding
ATP-bindin	protein-coding
basic trans	protein-coding
1-acylglyce	protein-coding
RAB33B, m	protein-coding
stanniocalc	protein-coding
toll-like rec	protein-coding
INO80 hom	protein-coding
TGFB-induc	protein-coding
upstream k	protein-coding
LETM1 don	protein-coding
DEAD (Asp-	protein-coding
ATPase, Ca	protein-coding
galactosylc	protein-coding
BMP2 indu	protein-coding
transmeml	protein-coding
RIKEN cDN.	protein-coding
cactin, splic	protein-coding
G1 to S pha	protein-coding
UBA-like dc	protein-coding
renalase, F.	protein-coding
DnaJ (Hsp4	protein-coding
lysine (K)-s	protein-coding
v-abl Abels	protein-coding
placenta ex	protein-coding
zinc finger	protein-coding
quiescin Q(	protein-coding
microsoma	protein-coding
recombina	protein-coding
hook homc	protein-coding
lysophosph	protein-coding
tyrosine 3-	protein-coding
cytochrom	protein-coding
signal pept	protein-coding
LIM and SH	protein-coding
phospholip	protein-coding
protein arg	protein-coding
ADP-deper	protein-coding
cyclin-depe	protein-coding

schlafen 2	protein-coding
tubulin tyr	protein-coding
ring finger	protein-coding
ankyrin rep	protein-coding
MAP/micro	protein-coding
transforma	protein-coding
spermatog	protein-coding
dedicator c	protein-coding
major facili	protein-coding
M-phase sp	protein-coding
insulin-like	protein-coding
melanoreg	protein-coding
septin 5	protein-coding
glycophori	protein-coding
chromobox	protein-coding
triple funct	protein-coding
zinc finger	protein-coding
intraflagell	protein-coding
hairy/enha	protein-coding
trafficking	protein-coding
bromodom	protein-coding
CDKN2A in	protein-coding
secretory c	protein-coding
apolipopro	protein-coding
collagen, ty	protein-coding
multivesicu	protein-coding
nucleopori	protein-coding
actin relate	protein-coding
TM2 doma	protein-coding
zinc finger	protein-coding
suppressio	protein-coding
ceramide s	protein-coding
NUF2, NDC	protein-coding
trichorhino	protein-coding
ankyrin rep	protein-coding
selenoprot	protein-coding
zinc finger	protein-coding
Zinc finger	protein-coding
transmemb	protein-coding
mitogen-ac	protein-coding
cDNA sequ	protein-coding
ATPase, Na	protein-coding
transmemb	protein-coding
ATPase, H+	protein-coding
protein ph	protein-coding
transformi	protein-coding
protein-L-is	protein-coding

pyroglutam	protein-coding
speckle-typ	protein-coding
tumor necr	protein-coding
phosphoin	protein-coding
predicted g	protein-coding
perilipin 2	protein-coding
tRNA splici	protein-coding
acyl-CoA th	protein-coding
vinculin	protein-coding
zinc finger	protein-coding
nuclear rec	protein-coding
tubulin, de	protein-coding
RNA bindin	protein-coding
tyrosine 3-	protein-coding
transcriptio	protein-coding
heterogene	protein-coding
FERM dom	protein-coding
serine hydr	protein-coding
MARCKS-lil	protein-coding
furry homo	protein-coding
chloride in	protein-coding
zinc finger	protein-coding
transporte	protein-coding
src family a	protein-coding
histone cel	protein-coding
family with	protein-coding
proteasom	protein-coding
transforme	protein-coding
B cell trans	protein-coding
NIMA (nev	protein-coding
splA/ryano	protein-coding
activin rece	protein-coding
regulator o	protein-coding
single stran	protein-coding
ring finger	protein-coding
espin	protein-coding
multiple C2	protein-coding
adrenergic	protein-coding
signal reco	protein-coding
Rap guanin	protein-coding
spleen tyro	protein-coding
ubiquitin-a	protein-coding
E74-like fac	protein-coding
maestro he	protein-coding
serum resp	protein-coding
SRY (sex de	protein-coding
myotubula	protein-coding

ER membra	protein-coding
signal trans	protein-coding
predicted g	protein-coding
mesoderm	protein-coding
two pore c	protein-coding
putative hc	protein-coding
glutamate	protein-coding
collectin su	protein-coding
Erbb2 inter	protein-coding
adenine ph	protein-coding
coiled-coil	protein-coding
paired-like	protein-coding
inhibitor of	protein-coding
RAB2A, me	protein-coding
transmemk	protein-coding
STARD3 N-	protein-coding
Hermansky	protein-coding
single strar	protein-coding
PDS5, regu	protein-coding
protein pho	protein-coding
solute carri	protein-coding
schlafen 2	protein-coding
vacuolar pr	protein-coding
solute carri	protein-coding
putative hc	protein-coding
S-antigen,	protein-coding
inter-alpha	protein-coding
trafficking	protein-coding
chromodor	protein-coding
zinc finger	protein-coding
phosphogly	protein-coding
adenosine	protein-coding
oxoglutara	protein-coding
Eph recept	protein-coding
DEAD (Asp-	protein-coding
cyclin C	protein-coding
klotho beta	protein-coding
RIKEN cDN.	protein-coding
RWD doma	protein-coding
patatin-like	protein-coding
torsin fami	protein-coding
carboxypep	protein-coding
serum/gluc	protein-coding
peptidylpro	protein-coding
mitogen-ac	protein-coding
CD33 antig	protein-coding
human imr	protein-coding

ribosomal I	protein-coding
cDNA sequ	protein-coding
mitochond	protein-coding
ATP-bindin	protein-coding
potassium	protein-coding
olfactory re	protein-coding
membrane	protein-coding
progestero	protein-coding
lipase, horr	protein-coding
ubiquitin-c	protein-coding
RIKEN cDN.	protein-coding
protein pho	protein-coding
heat shock	protein-coding
ELK4, mem	protein-coding
2-oxoglutai	protein-coding
RAB, meml	protein-coding
degenerati	protein-coding
protein tyr	protein-coding
avian reticu	protein-coding
queuine tR	protein-coding
ATPase, cla	protein-coding
protein pho	protein-coding
ubiquitin-c	protein-coding
calcineurin	protein-coding
glutamate	protein-coding
RAB11 fam	protein-coding
exocyst cor	protein-coding
ring-box 1	protein-coding
centromere	protein-coding
COP9 (cons	protein-coding
intraflagell	protein-coding
oocyte secr	protein-coding
RIKEN cDN.	protein-coding
trafficking	protein-coding
signal-regu	protein-coding
proteasom	protein-coding
musculoske	protein-coding
PR domain	protein-coding
protein pho	protein-coding
mitochond	protein-coding
zinc finger	protein-coding
testis expre	protein-coding
MPV17 mit	protein-coding
cytochrom	protein-coding
signal trans	protein-coding
family with	protein-coding
C2 calcium	protein-coding

non-SMC e	protein-coding
poly(A) bin	protein-coding
CDC42 effe	protein-coding
ER degrada	protein-coding
UPF1 regul	protein-coding
polypyrimi	protein-coding
meiotic nu	protein-coding
translocase	protein-coding
DNA segme	protein-coding
vinculin	protein-coding
TOX high m	protein-coding
homeodon	protein-coding
RB1-induci	protein-coding
microtubul	protein-coding
Rap guanin	protein-coding
neural prec	protein-coding
acylglycero	protein-coding
thioredoxir	protein-coding
asparagine	protein-coding
tetratricop	protein-coding
N(alpha)-a	protein-coding
acyl-Coenz	protein-coding
smoothelir	protein-coding
trinucleotic	protein-coding
caveolin 2	protein-coding
inhibin bet	protein-coding
protein pho	protein-coding
serine (or c	protein-coding
zinc finger	protein-coding
phospholip	protein-coding
forkhead b	protein-coding
integrin be	protein-coding
acyl-CoA sy	protein-coding
ankyrin rep	protein-coding
sterile alph	protein-coding
von Hippel	protein-coding
actin, alpha	protein-coding
nuclear ant	protein-coding
potassium	protein-coding
protein tyr	protein-coding
coiled-coil	protein-coding
Von Willeb	protein-coding
autophagy,	protein-coding
StAR-relate	protein-coding
influenza v	protein-coding
A kinase (P	protein-coding
peroxisoma	protein-coding

tribbles ho	protein-coding
family with	protein-coding
isoleucine-	protein-coding
family with	protein-coding
solute carri	protein-coding
ARP3 actin	protein-coding
protein kin	protein-coding
transcriptic	protein-coding
enoyl Coen	protein-coding
meiosis-spe	protein-coding
transmeml	protein-coding
pellino 1	protein-coding
zinc finger	protein-coding
interleukin	protein-coding
RIKEN cDN.	protein-coding
arrestin do	protein-coding
heterogene	protein-coding
basic leucir	protein-coding
SET domair	protein-coding
solute carri	protein-coding
potassium	protein-coding
NFKB activ	protein-coding
ATPase, H+	protein-coding
cell divisior	protein-coding
RAB3A inte	protein-coding
Kruppel-lik	protein-coding
PC-esterase	protein-coding
hypoxanthi	protein-coding
autophagy	protein-coding
Rho GTPase	protein-coding
LFNG O-fuc	protein-coding
TBC1 doma	protein-coding
mitogen-ac	protein-coding
interleukin	protein-coding
RAS p21 pr	protein-coding
UDP-N-ace	protein-coding
RAS protei	protein-coding
KDM3B lysi	protein-coding
mitochond	protein-coding
5-hydroxyr	protein-coding
testis expre	protein-coding
selectin, lyı	protein-coding
UDP-Gal:be	protein-coding
zinc finger	protein-coding
F-box and l	protein-coding
myelin pro	protein-coding
cyclin-depe	protein-coding

adrenomec	protein-coding
zinc finger,	protein-coding
Tctex1 don	protein-coding
phospholip	protein-coding
transmemk	protein-coding
adaptor-re	protein-coding
Janus kinas	protein-coding
sphingomy	protein-coding
INO80 com	protein-coding
KN motif a	protein-coding
methyltran	protein-coding
nucleotide	protein-coding
elongation	protein-coding
neurotroph	protein-coding
cancer susc	protein-coding
multiple C2	protein-coding
RNA bindin	protein-coding
CKLF-like M	protein-coding
signal reco	protein-coding
TAF15 RNA	protein-coding
Ras associa	protein-coding
patatin-like	protein-coding
inverted fo	protein-coding
clathrin, lig	protein-coding
ribosomal	protein-coding
numb gene	protein-coding
transmemk	protein-coding
family with	protein-coding
transmemk	protein-coding
nucleic acic	protein-coding
RIKEN cDN	protein-coding
Cobl-like 1	protein-coding
cyclin-depe	protein-coding
glucocortic	protein-coding
WD repeat	protein-coding
transmemk	protein-coding
solute carri	protein-coding
lon peptida	protein-coding
DnaJ (Hsp4	protein-coding
smg-9 hom	protein-coding
Rho GTPas	protein-coding
RIKEN cDN	protein-coding
calcium bir	protein-coding
ATPase, Ca	protein-coding
ribosomal	protein-coding
nuclear fac	protein-coding
nephroblas	protein-coding

suppressor	protein-coding
tectonin be	protein-coding
ficolin B	protein-coding
protein arg	protein-coding
protein ph	protein-coding
transmeml	protein-coding
AF4/FMR2	protein-coding
protease-a	protein-coding
DnaJ (Hsp4	protein-coding
clathrin int	protein-coding
kinesin fam	protein-coding
chemokine	protein-coding
interleukin	protein-coding
superkiller	protein-coding
vomeronas	protein-coding
signal sequ	protein-coding
forkhead b	protein-coding
hematolog	protein-coding
valyl-tRNA	protein-coding
fructose bi	protein-coding
retinoic aci	protein-coding
nuclear rec	protein-coding
short chain	protein-coding
poly(A) bin	protein-coding
mannoside	protein-coding
guanylate l	protein-coding
cell divisior	protein-coding
protein ph	protein-coding
histone dea	protein-coding
zinc finger	protein-coding
B cell CLL/l	protein-coding
neuromedi	protein-coding
kelch-like 1	protein-coding
synaptophy	protein-coding
paired Ig-lil	protein-coding
RAB intera	protein-coding
neuropilin	protein-coding
transcriptio	protein-coding
tropomyos	protein-coding
mex3 hom	protein-coding
proviral int	protein-coding
serine/thre	protein-coding
poly (ADP-r	protein-coding
abhydrolas	protein-coding
oxysterol b	protein-coding
5' nucleoti	protein-coding
synergin, g	protein-coding

sema doma	protein-coding
transmemb	protein-coding
ATPase, an	protein-coding
RIKEN cDN.	protein-coding
RIKEN cDN.	protein-coding
interferon	protein-coding
B cell leuke	protein-coding
cytoplasmi	protein-coding
sex comb c	protein-coding
secretoglol	protein-coding
Rho-associ	protein-coding
PCI domair	protein-coding
calcium/ca	protein-coding
trans-actin	protein-coding
heterogene	protein-coding
tubulin pol	protein-coding
WD repeat	protein-coding
guanine nu	protein-coding
Fc receptoi	protein-coding
RAS p21 pr	protein-coding
anthrax to›	protein-coding
trans-actin	protein-coding
Rho, GDP c	protein-coding
MRV integı	protein-coding
RIKEN cDN.	protein-coding
hexokinase	protein-coding
CAS1 doma	protein-coding
synovial sa	protein-coding
leucine rich	protein-coding
syncytin b	protein-coding
KRI1 homo	protein-coding
ATPase, cla	protein-coding
inhibin bet	protein-coding
serine palm	protein-coding
TSC22 dom	protein-coding
chaperonir	protein-coding
predicted g	protein-coding
meiosis 1 a	protein-coding
kelch doma	protein-coding
protein kin	protein-coding
ribosomal	protein-coding
differential	protein-coding
RAD54 like	protein-coding
colony stim	protein-coding
RIKEN cDN.	protein-coding
trafficking	protein-coding
phosphodi	protein-coding

Rho GTPas	protein-coding
zinc finger	protein-coding
transformi	protein-coding
bone morp	protein-coding
sorting nex	protein-coding
SPT2, Supp	protein-coding
Cbp/p300-i	protein-coding
reactive ox	protein-coding
chemokine	protein-coding
solute carri	protein-coding
annexin A1	protein-coding
CD84 antig	protein-coding
short chain	protein-coding
zinc finger	protein-coding
centromer	protein-coding
Rhesus blo	protein-coding
signal reco	protein-coding
RIKEN cDN	protein-coding
down-regu	protein-coding
transmemb	protein-coding
IKAROS fan	protein-coding
Kruppel-lik	protein-coding
acyl-CoA sy	protein-coding
LIM domai	protein-coding
immediate	protein-coding
dual specif	protein-coding
coiled-coil	protein-coding
pyridoxal-c	protein-coding
transportir	protein-coding
nuclear fac	protein-coding
LIM domai	protein-coding
U2 small nu	protein-coding
serine (or c	protein-coding
high mobili	protein-coding
thiosulfate	protein-coding
natriuretic	protein-coding
serine (or c	protein-coding
neutrophili	protein-coding
SLX4 intera	protein-coding
lamin B rec	protein-coding
pleckstrin h	protein-coding
nucleobind	protein-coding
mitochond	protein-coding
X-linked my	protein-coding
nucleolar p	protein-coding
cytoskeleto	protein-coding
purine rich	protein-coding

ceramide s	protein-coding
ankyrin rep	protein-coding
G patch do	protein-coding
homeobox	protein-coding
ubiquitin-c	protein-coding
liver glycog	protein-coding
YTH domai	protein-coding
zinc finger	protein-coding
killer cell le	protein-coding
UDP-N-ace	protein-coding
tRNA-yW s	protein-coding
cystathiona	protein-coding
UHRF1 (ICE	protein-coding
small VCP/	protein-coding
polymerase	protein-coding
salt inducib	protein-coding
leucine-rich	protein-coding
neural prec	protein-coding
ribosome p	protein-coding
ankyrin rep	protein-coding
ubiquitin-c	protein-coding
Ras associa	protein-coding
coactosin-l	protein-coding
ras respons	protein-coding
leucine rich	protein-coding
PDLIM1 int	protein-coding
golgi memb	protein-coding
solute carri	protein-coding
nischarin	protein-coding
ribosome p	protein-coding
histone dea	protein-coding
placental g	protein-coding
BEN domai	protein-coding
collagen, ty	protein-coding
transmemb	protein-coding
WD repeat	protein-coding
proviral int	protein-coding
caspase rec	protein-coding
neuromedi	protein-coding
ATP-bindin	protein-coding
angiotensin	protein-coding
RAS guanyl	protein-coding
mitochond	protein-coding
ubiquitin-fo	protein-coding
G protein-c	protein-coding
placenta-sp	protein-coding
translocase	protein-coding

thymus cel	protein-coding
peroxisom	protein-coding
ring finger	protein-coding
survival mc	protein-coding
RNA bindin	protein-coding
Kruppel-lik	protein-coding
lamin B2	protein-coding
NEDD4 bin	protein-coding
Fc recepto	protein-coding
predicted g	protein-coding
Nipped-B h	protein-coding
cytochrom	protein-coding
isoleucine-	protein-coding
retinol deh	protein-coding
single-stra	protein-coding
Rho GTPas	protein-coding
solute carri	protein-coding
arginyl ami	protein-coding
cDNA sequ	protein-coding
myocyte er	protein-coding
protein kin	protein-coding
enhancer c	protein-coding
centromer	protein-coding
solute carri	protein-coding
zinc finger	protein-coding
zinc finger	protein-coding
THAP dom	protein-coding
integrin be	protein-coding
solute carri	protein-coding
general tra	protein-coding
acidic (leuc	protein-coding
RIKEN cDN	protein-coding
zinc finger	protein-coding
ELK4, mem	protein-coding
calcium ch	protein-coding
vimentin	protein-coding
cleavage ar	protein-coding
chemokine	protein-coding
1-acylglyce	protein-coding
hexose-6-p	protein-coding
ADP-ribosy	protein-coding
cleavage ar	protein-coding
P450 (cyto	protein-coding
cytochrom	protein-coding
glutamate	protein-coding
dedicator c	protein-coding
MIS18 binc	protein-coding

WD repeat	protein-coding
transforma	protein-coding
gasdermin	protein-coding
ST3 beta-ga	protein-coding
RAB24, me	protein-coding
R3H domai	protein-coding
RIKEN cDN.	protein-coding
DNA methy	protein-coding
bolA-like 1	protein-coding
phospholip	protein-coding
eukaryotic	protein-coding
cut-like hoı	protein-coding
interferon	protein-coding
mortality fa	protein-coding
phosphatic	protein-coding
secretograı	protein-coding
basigin	protein-coding
RIKEN cDN.	protein-coding
NHL repeat	protein-coding
protein pho	protein-coding
centrosom	protein-coding
proliferatic	protein-coding
ATPase, Ca	protein-coding
URI1, prefc	protein-coding
death-asso	protein-coding
glucose ph	protein-coding
CDC14 cell	protein-coding
prolactin fa	protein-coding
exosome co	protein-coding
ankyrin rep	protein-coding
chemokine	protein-coding
transmemb	protein-coding
ATP syntha	protein-coding
ribosomal p	protein-coding
zinc finger	protein-coding
insulin-like	protein-coding
ELKS/RAB6	protein-coding
Tu translat	protein-coding
solute carri	protein-coding
FYVE, RhoG	protein-coding
H2A histon	protein-coding
bradykinin	protein-coding
glycine rec	protein-coding
eukaryotic	protein-coding
Rho GTPase	protein-coding
adhesion m	protein-coding
lysophosph	protein-coding

myosin, ligl	protein-coding
translocase	protein-coding
suppressor	protein-coding
RIKEN cDN.	protein-coding
X-ray repai	protein-coding
ADP-ribosy	protein-coding
solute carri	protein-coding
transmemb	protein-coding
centriolin	protein-coding
RIKEN cDN.	protein-coding
componen	protein-coding
glutathione	protein-coding
transmemb	protein-coding
thioredoxir	protein-coding
RAR-relate	protein-coding
septin 9	protein-coding
regulatory	protein-coding
thymopoie	protein-coding
seryl-amin	protein-coding
RIKEN cDN.	protein-coding
proline-ser	protein-coding
valyl-tRNA	protein-coding
cytochrom	protein-coding
histone dea	protein-coding
DNA-dama	protein-coding
myeloid/ly	protein-coding
choline ph	protein-coding
transducin-	protein-coding
FGGY carb	protein-coding
Ral GEF wit	protein-coding
nucleoside	protein-coding
CCAAT/enh	protein-coding
ral guanine	protein-coding
serine (or c	protein-coding
centromer	protein-coding
transmemb	protein-coding
myosin, ligl	protein-coding
outer dens	protein-coding
ribosomal	protein-coding
acyl-Coenz	protein-coding
CCAAT/enh	protein-coding
cerebral dc	protein-coding
high densit	protein-coding
adaptor pr	protein-coding
KDEL (Lys-/	protein-coding
FBJ osteos	protein-coding
TAR (HIV) F	protein-coding

sterol O-ac	protein-coding
Smg-5 hom	protein-coding
NOP58 ribo	protein-coding
RIKEN cDN.	protein-coding
transmemb	protein-coding
poly (ADP-r	protein-coding
myozenin 1	protein-coding
Friend leuk	protein-coding
T cell activa	protein-coding
zinc finger,	protein-coding
G protein-c	protein-coding
adaptor-re	protein-coding
ubiquitin sp	protein-coding
ubiquitin-c	protein-coding
dehydroge	protein-coding
suppressor	protein-coding
inositol pol	protein-coding
ubiquinol-c	protein-coding
DNA-dama	protein-coding
IKAROS fan	protein-coding
hepatocyte	protein-coding
ADAMTS-lil	protein-coding
pseudouric	protein-coding
spondin 1,	protein-coding
tribbles ho	protein-coding
eukaryotic	protein-coding
ras respons	protein-coding
signal trans	protein-coding
G protein-c	protein-coding
nudix (nucl	protein-coding
ribonucleo	protein-coding
solute carri	protein-coding
UDP-glucur	protein-coding
DNA-dama	protein-coding
cell divisior	protein-coding
kinase supp	protein-coding
ribulose-5-	protein-coding
v-maf musc	protein-coding
eukaryotic	protein-coding
macrophag	protein-coding
mitogen-ac	protein-coding
Aly/REF exp	protein-coding
zinc finger	protein-coding
ribosomal p	protein-coding
DEAD (Asp-	protein-coding
ST3 beta-ga	protein-coding
microsphei	protein-coding

cathepsin Z	protein-coding
forkhead b	protein-coding
thioredoxir	protein-coding
LIM domair	protein-coding
tRNA nucle	protein-coding
zinc finger	protein-coding
zinc finger	protein-coding
zinc finger	protein-coding
multivesicu	protein-coding
replication	protein-coding
DCP1 decap	protein-coding
neuroepith	protein-coding
wingless-ty	protein-coding
nuclear rec	protein-coding
kelch repea	protein-coding
zinc finger	protein-coding
Sep (O-pho	protein-coding
predicted g	protein-coding
Bruton aga	protein-coding
transcriptic	protein-coding
vav 1 oncog	protein-coding
TBC1 doma	protein-coding
snail family	protein-coding
TBC1 doma	protein-coding
forkhead b	protein-coding
cytochrom	protein-coding
prominin 1	protein-coding
interleukin	protein-coding
cyclin D3	protein-coding
angiogenin	protein-coding
SMAD fami	protein-coding
IMP2 inner	protein-coding
mitchondri	protein-coding
ribosomal p	protein-coding
family with	protein-coding
RIKEN cDNA	protein-coding
sulfotransf	protein-coding
transmemb	protein-coding
predicted g	protein-coding
potassium	protein-coding
bromodom	protein-coding
protein pho	protein-coding
phosphatic	protein-coding
retinoblast	protein-coding
general tra	protein-coding
carbohydra	protein-coding
destrin	protein-coding

serine/thre	protein-coding
discoidin d	protein-coding
Tax1 (huma	protein-coding
ribosomal I	protein-coding
nuclear fac	protein-coding
aconitase 1	protein-coding
protein pho	protein-coding
STT3, subu	protein-coding
family with	protein-coding
torsin A int	protein-coding
testis speci	protein-coding
ATPase, cla	protein-coding
kinesin fam	protein-coding
DNA-dama	protein-coding
multiple C2	protein-coding
sperm tail I	protein-coding
interleukin	protein-coding
interferon	protein-coding
chromodor	protein-coding
basic leucir	protein-coding
HMG box d	protein-coding
predicted g	protein-coding
resistance	protein-coding
heat shock	protein-coding
interferon	protein-coding
SWI5 depe	protein-coding
protein pho	protein-coding
integrin-lin	protein-coding
RIKEN cDN	protein-coding
Rho guanir	protein-coding
diacylglyce	protein-coding
zinc finger,	protein-coding
congenital	protein-coding
TBC1 doma	protein-coding
CCR4 carbo	protein-coding
transmemb	protein-coding
ubiquinol-c	protein-coding
zinc finger	protein-coding
ATP/GTP b	protein-coding
solute carri	protein-coding
cell divisior	protein-coding
RING1 and	protein-coding
biotinidase	protein-coding
ankyrin rep	protein-coding
DEAD (Asp-	protein-coding
ArfGAP wit	protein-coding
nucleolar p	protein-coding

family with	protein-coding
transglutar	protein-coding
cell divisior	protein-coding
ST3 beta-ga	protein-coding
taste recep	protein-coding
pumilio RN	protein-coding
nuclear rec	protein-coding
mitochond	protein-coding
glucosamir	protein-coding
tumor-assc	protein-coding
peroxisom	protein-coding
ankyrin rep	protein-coding
BEN domai	protein-coding
centrosom	protein-coding
uridine mo	protein-coding
CD1d1 anti	protein-coding
aldehyde d	protein-coding
SEC22 vesi	protein-coding
coiled-coil	protein-coding
cathelicidir	protein-coding
RALY RNA I	protein-coding
teneurin tr	protein-coding
centrosom	protein-coding
ribosomal	protein-coding
activating t	protein-coding
scavenger	protein-coding
tyrosyl-tRN	protein-coding
solute carri	protein-coding
EGF-like re	protein-coding
leukotriene	protein-coding
ribosomal	protein-coding
CD3 antige	protein-coding
H2A histon	protein-coding
mannosida	protein-coding
heterogene	protein-coding
ajuba LIM	protein-coding
polymeras	protein-coding
ring finger	protein-coding
ataxin 2	protein-coding
epithelial n	protein-coding
complemer	protein-coding
zinc finger	protein-coding
histone de	protein-coding
zinc and rir	protein-coding
vaccinia rel	protein-coding
TRM5 tRN	protein-coding
ubiquitin s	protein-coding

vomeronas	protein-coding
AT rich inte	protein-coding
ubiquitin s|	protein-coding
muscleblin	protein-coding
PR domain	protein-coding
Kruppel-lik	protein-coding
pantothen	protein-coding
general tra	protein-coding
CD52 antig	protein-coding
vacuolar pr	protein-coding
late endos	protein-coding
MAX-like p	protein-coding
protein tyr	protein-coding
stanniocal	protein-coding
E2F transcr	protein-coding
DNA prima	protein-coding
aspartyl-tR	protein-coding
triggering r	protein-coding
kinase sup	protein-coding
Ras associa	protein-coding
transmemb	protein-coding
pleckstrin I	protein-coding
kelch-like 2	protein-coding
transmemb	protein-coding
YY1 transcr	protein-coding
ubiquitin-li	protein-coding
ephrin B1	protein-coding
cyclin-depe	protein-coding
platelet-ac	protein-coding
DIS3 mitoti	protein-coding
gypsy retrc	protein-coding
TatD DNas	protein-coding
thioredoxi	protein-coding
chloride ch	protein-coding
transcriptic	protein-coding
AT rich inte	protein-coding
EGF domai	protein-coding
phosphom	protein-coding
ZPR1 zinc f	protein-coding
serine (or c	protein-coding
tet methyl	protein-coding
zinc finger,	protein-coding
dystrobrev	protein-coding
coproporp	protein-coding
prune hom	protein-coding
CD47 antig	protein-coding
zinc finger	protein-coding

splicing fac	protein-coding
G protein-c	protein-coding
cytochrom	protein-coding
transducin	protein-coding
sorting nex	protein-coding
tumor necr	protein-coding
zinc finger	protein-coding
solute carri	protein-coding
RIKEN cDN.	protein-coding
inter-alpha	protein-coding
RIKEN cDN.	protein-coding
deoxyhypu	protein-coding
cAMP resp	protein-coding
CTD (carbo	protein-coding
KH domain	protein-coding
SWI/SNF-re	protein-coding
brain-speci	protein-coding
sorting nex	protein-coding
family with	protein-coding
tetratricop	protein-coding
ribosomal	protein-coding
Ewing tum	protein-coding
Braf transf	protein-coding
speckle-typ	protein-coding
coiled-coil	protein-coding
tribbles ho	protein-coding
activating t	protein-coding
notch 2	protein-coding
methyltran	protein-coding
Kruppel-lik	protein-coding
zinc finger	protein-coding
sodium cha	protein-coding
eukaryotic	protein-coding
leucine rich	protein-coding
histone clu	protein-coding
PR domain	protein-coding
BTB (POZ)	protein-coding
recombina	protein-coding
ATPase fan	protein-coding
glia matura	protein-coding
MAS-relate	protein-coding
methionine	protein-coding
serine/argi	protein-coding
ATP-bindin	protein-coding
zinc finger	protein-coding
ubiquitin-c	protein-coding
cadherin 7,	protein-coding

estrogen re	protein-coding
epithelial s	protein-coding
RIKEN cDN.	protein-coding
nuclear rec	protein-coding
RIKEN cDN.	protein-coding
kinesin fam	protein-coding
zinc finger	protein-coding
melanoreg	protein-coding
NIMA (nev	protein-coding
mitogen-ac	protein-coding
RNA methy	protein-coding
programm	protein-coding
cell divisior	protein-coding
OTU domai	protein-coding
Mid1 intera	protein-coding
ring finger	protein-coding
exocyst cor	protein-coding
coiled-coil	protein-coding
cystatin 10	protein-coding
FMS-like ty	protein-coding
ubiquitin ca	protein-coding
KAT8 regul	protein-coding
nuclear rec	protein-coding
tumor necr	protein-coding
atypical ch	protein-coding
family with	protein-coding
Yip1 domai	protein-coding
large tumo	protein-coding
abhydrolas	protein-coding
sema doma	protein-coding
2-aminoetl	protein-coding
euchromat	protein-coding
BCL2-like 1	protein-coding
phosphoin	protein-coding
wings apar	protein-coding
neutrophil	protein-coding
interleukin	protein-coding
target of E	protein-coding
CKLF-like M	protein-coding
solute carri	protein-coding
PTK2 prote	protein-coding
hexose-6-p	protein-coding
sex comb c	protein-coding
within bgcr	protein-coding
zinc finger	protein-coding
small integ	protein-coding
ATP/GTP b	protein-coding

timeless in	protein-coding
DEAD (Asp-	protein-coding
ubiquitin s|	protein-coding
eukaryotic	protein-coding
zinc finger	protein-coding
pyrroline-5	protein-coding
cDNA sequ	protein-coding
single-strar	protein-coding
oxysterol b	protein-coding
neurofascir	protein-coding
loss of hete	protein-coding
transcriptic	protein-coding
chemokine	protein-coding
ribosomal		protein-coding
exportin 6	protein-coding
heparan su	protein-coding
RIKEN cDN.	protein-coding
regulator o	protein-coding
3-ketodihy	protein-coding
phosphodi	protein-coding
Ras and Ra	protein-coding
Sh3 domair	protein-coding
signal reco	protein-coding
microtubul	protein-coding
HECT doma	protein-coding
NADH dehy	protein-coding
H3 histone	protein-coding
myocardial	protein-coding
chromodor	protein-coding
proteasom	protein-coding
Rab40C, m	protein-coding
ATPase, H+	protein-coding
helicase, ly	protein-coding
transmemb	protein-coding
interleukin	protein-coding
RIKEN cDN.	protein-coding
cytochrom	protein-coding
limb-bud a	protein-coding
prothymos	protein-coding
myeloid dif	protein-coding
lipin 1	protein-coding
polymerase	protein-coding
THAP doma	protein-coding
solute carri	protein-coding
solute carri	protein-coding
CCAAT/enh	protein-coding
H1 histone	protein-coding

guanine nu	protein-coding
SYS1 Golgi-	protein-coding
cornichon l	protein-coding
secretory c	protein-coding
lectin, man	protein-coding
basic leucir	protein-coding
nucleopori	protein-coding
fibulin 1	protein-coding
E2F transcr	protein-coding
CD93 antig	protein-coding
NFU1 iron-	protein-coding
programme	protein-coding
REV1 homo	protein-coding
dehydroge	protein-coding
cleavage ar	protein-coding
ras homolo	protein-coding
hydroxyme	protein-coding
ligand of nu	protein-coding
sperm flage	protein-coding
lysophosph	protein-coding
microtubul	protein-coding
solute carri	protein-coding
tubulin folc	protein-coding
ATP syntha	protein-coding
UDP-N-ace	protein-coding
ankyrin rep	protein-coding
transferrin	protein-coding
phosphopr	protein-coding
neuroguidi	protein-coding
caspase 8 a	protein-coding
phosphodi	protein-coding
AT rich inte	protein-coding
WD repeat	protein-coding
ankyrin rep	protein-coding
zinc finger	protein-coding
proteasom	protein-coding
zinc finger	protein-coding
DEAD (Asp-	protein-coding
phosphatic	protein-coding
synaptotag	protein-coding
microtubul	protein-coding
zyg-11 rela	protein-coding
B cell leuke	protein-coding
suppressio	protein-coding
RAB8A, me	protein-coding
general tra	protein-coding
autocrine r	protein-coding

COMM dor	protein-coding
mitochond	protein-coding
transferrin	protein-coding
leukotriene	protein-coding
RNA bindir	protein-coding
cadherin 4	protein-coding
inhibitor of	protein-coding
glutaminas	protein-coding
ryanodine	protein-coding
ArfGAP wit	protein-coding
NIMA (nev	protein-coding
RAD23b hc	protein-coding
suppressio	protein-coding
ribosomal	protein-coding
adhesion n	protein-coding
zinc finger	protein-coding
microtubul	protein-coding
zinc finger,	protein-coding
zinc finger	protein-coding
jumonji do	protein-coding
transmemb	protein-coding
nudix (nucl	protein-coding
serine hydr	protein-coding
clarin 3	protein-coding
ATPase, H+	protein-coding
dipeptidylp	protein-coding
C-type lect	protein-coding
prothymos	protein-coding
interleukin	protein-coding
RAS p21 pr	protein-coding
eukaryotic	protein-coding
ubiquitin C	protein-coding
N(alpha)-ac	protein-coding
RanBP-type	protein-coding
TATA box b	protein-coding
cytokine in	protein-coding
tribbles ho	protein-coding
Prkr intera	protein-coding
acyl-Coenz	protein-coding
synuclein, a	protein-coding
striatin, cal	protein-coding
ring finger	protein-coding
pseudouric	protein-coding
myosin IIIB	protein-coding
Crm, cramp	protein-coding
serine prot	protein-coding
cAMP resp	protein-coding

minichrom	protein-coding
CCR4-NOT	protein-coding
abhydrolas	protein-coding
MOB kinas	protein-coding
methyltran	protein-coding
zinc finger	protein-coding
FK506 bind	protein-coding
MKL (mega	protein-coding
SHANK-ass	protein-coding
zinc finger	protein-coding
Sad1 and U	protein-coding
sterol O-ac	protein-coding
non-SMC e	protein-coding
cAMP resp	protein-coding
transforma	protein-coding
aminolevul	protein-coding
zinc finger	protein-coding
zinc finger	protein-coding
forkhead b	protein-coding
solute carri	protein-coding
potassium	protein-coding
vacuolar pr	protein-coding
sorting nex	protein-coding
ARP3 actin	protein-coding
T cell, imm	protein-coding
methionine	protein-coding
hairy and e	protein-coding
RAB28, me	protein-coding
methylthio	protein-coding
nuclear fac	protein-coding
TruB pseuc	protein-coding
proteasom	protein-coding
karyopheri	protein-coding
ELOVL fam	protein-coding
histone clu	protein-coding
cytochrom	protein-coding
ADP-ribosy	protein-coding
proteasom	protein-coding
transmemb	protein-coding
protein tyr	protein-coding
argonaute	protein-coding
zinc finger	protein-coding
eukaryotic	protein-coding
mitogen-ac	protein-coding
GH regulat	protein-coding
predicted g	protein-coding
predicted g	protein-coding

gephyrin	protein-coding
SCP2 sterol	protein-coding
ArfGAP wit	protein-coding
POU doma	protein-coding
DENN/MAI	protein-coding
LIM domai	protein-coding
upstream t	protein-coding
exportin, tl	protein-coding
apolipopro	protein-coding
Wolf-Hirscl	protein-coding
CDC28 prot	protein-coding
Nipped-B h	protein-coding
formin-like	protein-coding
a disintegri	protein-coding
autophagy	protein-coding
post-GPI at	protein-coding
predicted g	protein-coding
predicted g	protein-coding
multiple C2	protein-coding
dishevelled	protein-coding
eukaryotic	protein-coding
cyclin M2	protein-coding
hairy and e	protein-coding
FMS-like ty	protein-coding
suppressor	protein-coding
RIKEN cDN.	protein-coding
adaptor-re	protein-coding
ribosomal p	protein-coding
elastin mic	protein-coding
catenin (ca	protein-coding
peroxisoma	protein-coding
transcriptio	protein-coding
thromboxa	protein-coding
proteasom	protein-coding
zinc finger	protein-coding
mitochond	protein-coding
leucine, glu	protein-coding
biogenesis	protein-coding
SMAD fami	protein-coding
nucleopori	protein-coding
sphingosine	protein-coding
adiponectii	protein-coding
TRAF family	protein-coding
mannosyl-(	protein-coding
microsoma	protein-coding
very low de	protein-coding
CUGBP, Ela	protein-coding

transient re	protein-coding
ubiquitin s|	protein-coding
PHD finger	protein-coding
IWS1 homo	protein-coding
ets variant	protein-coding
tripartite m	protein-coding
mitochond	protein-coding
UDP-N-ace	protein-coding
integrin be	protein-coding
BMP2 indu	protein-coding
KH-type sp	protein-coding
OTU domai	protein-coding
adrenergic	protein-coding
sulfatase 2	protein-coding
DTW doma	protein-coding
LIM domai	protein-coding
nuclear fac	protein-coding
metallothic	protein-coding
castor zinc	protein-coding
glycoprotei	protein-coding
FBJ osteosa	protein-coding
pleckstrin h	protein-coding
villin 1	protein-coding
kelch repea	protein-coding
hexamethy	protein-coding
prickle hon	protein-coding
polymerase	protein-coding
DIRAS fami	protein-coding
centromere	protein-coding
sprouty ho	protein-coding
cytochrom	protein-coding
circadian lc	protein-coding
CUGBP, Ela	protein-coding
zinc finger	protein-coding
transmeml	protein-coding
transmeml	protein-coding
NEDD4 bin	protein-coding
olfactory re	protein-coding
tRNA splici	protein-coding
nicalin hon	protein-coding
sphingomy	protein-coding
RAN bindin	protein-coding
Myb/SANT	protein-coding
zinc finger,	protein-coding
transducin-	protein-coding
carbohydra	protein-coding
rhotekin 2	protein-coding

SH3-bindin	protein-coding
atypical ch	protein-coding
acid phosp	protein-coding
family with	protein-coding
neurofibro	protein-coding
V-set and t	protein-coding
carnitine pa	protein-coding
zinc finger	protein-coding
neuron der	protein-coding
transmemb	protein-coding
dehydroge	protein-coding
Rho GDP di	protein-coding
proteasom	protein-coding
aspartate-b	protein-coding
X-linked inl	protein-coding
proteasom	protein-coding
galactosylc	protein-coding
retinitis pig	protein-coding
coenzyme	protein-coding
syntaxin 6	protein-coding
aminolevul	protein-coding
oxysterol b	protein-coding
cyclin L1	protein-coding
glutathione	protein-coding
ATP-bindin	protein-coding
zinc finger	protein-coding
PTEN induc	protein-coding
mitofusin 2	protein-coding
WD repeat	protein-coding
RIKEN cDN	protein-coding
interleukin	protein-coding
UHRF1 (ICE	protein-coding
lysine rich	protein-coding
heat shock	protein-coding
zinc finger,	protein-coding
lamin B rec	protein-coding
expressed	protein-coding
H3 histone	protein-coding
nitric oxide	protein-coding
adhesion m	protein-coding
helt bHLH t	protein-coding
cell adhesi	protein-coding
mitochond	protein-coding
SET and MY	protein-coding
aryl-hydroc	protein-coding
glucan (1,4	protein-coding
WD repeat	protein-coding

solute carri	protein-coding
ankyrin rep	protein-coding
solute carri	protein-coding
SMEK hom	protein-coding
protein pho	protein-coding
TRH-degrad	protein-coding
proteasom	protein-coding
ankyrin rep	protein-coding
interleukin	protein-coding
WW domai	protein-coding
UBA-like do	protein-coding
glutathione	protein-coding
ORAI calciu	protein-coding
bromodom	protein-coding
F-box and l	protein-coding
Ras homolo	protein-coding
myocyte en	protein-coding
dual specif	protein-coding
RAN bindin	protein-coding
zinc finger,	protein-coding
transforma	protein-coding
phorbol-12	protein-coding
sorting nex	protein-coding
synaptotag	protein-coding
ras homolo	protein-coding
anaphase p	protein-coding
translocase	protein-coding
actinin alph	protein-coding
voltage-dep	protein-coding
schwannom	protein-coding
COP9 (cons	protein-coding
dynamin bi	protein-coding
MYC-assoc	protein-coding
dynactin 6	protein-coding
Ena-vasodi	protein-coding
sin3 associ	protein-coding
GRAM dom	protein-coding
SH3-domai	protein-coding
protein pho	protein-coding
mediator c	protein-coding
SEC11 hom	protein-coding
histidine de	protein-coding
microtubul	protein-coding
docking pro	protein-coding
transmemb	protein-coding
myosin, ligh	protein-coding
RIKEN cDN	protein-coding

TNFAIP3 in	protein-coding
astrotactin	protein-coding
phosphata	protein-coding
ceramide s	protein-coding
SRY (sex de	protein-coding
SWA-70 pr	protein-coding
heparan su	protein-coding
phosphogl	protein-coding
predicted g	protein-coding
G protein-c	protein-coding
mitochond	protein-coding
transmemb	protein-coding
bridging in	protein-coding
cytotoxic T	protein-coding
inhibitor of	protein-coding
eukaryotic	protein-coding
mediator c	protein-coding
tripartite m	protein-coding
staufen (RN	protein-coding
protein ph	protein-coding
poly (ADP-	protein-coding
insulin-like	protein-coding
ribosomal	protein-coding
killer cell le	protein-coding
ubiquitin s	protein-coding
cysteine-se	protein-coding
transmemb	protein-coding
phosphata	protein-coding
Kruppel-lik	protein-coding
myosin IXb	protein-coding
coagulatio	protein-coding
ferredoxin	protein-coding
ring finger	protein-coding
Rho guanin	protein-coding
transmemb	protein-coding
zinc finger	protein-coding
transport a	protein-coding
ubiquitin s	protein-coding
sushi doma	protein-coding
carbonic an	protein-coding
ubiquitin-c	protein-coding
Ras associa	protein-coding
tribbles ho	protein-coding
high mobili	protein-coding
myelin pro	protein-coding
phosphatid	protein-coding
transcriptio	protein-coding

transmemb	protein-coding
uridine pho	protein-coding
slingshot h	protein-coding
pyrophosp	protein-coding
RIKEN cDN.	protein-coding
A kinase (P	protein-coding
sorting nex	protein-coding
RIKEN cDN.	protein-coding
WD repeat	protein-coding
ring finger	protein-coding
sirtuin 6	protein-coding
BCL2/aden	protein-coding
c-src tyrosi	protein-coding
coagulatior	protein-coding
G protein-c	protein-coding
T cell acute	protein-coding
NADH dehy	protein-coding
zinc finger	protein-coding
chemokine	protein-coding
ring finger	protein-coding
sperm assc	protein-coding
kelch-like 2	protein-coding
torsin fami	protein-coding
1-acylglyce	protein-coding
small nucle	protein-coding
ferredoxin	protein-coding
BCL2-assoc	protein-coding
testis-speci	protein-coding
Meckel syn	protein-coding
InaD-like (I	protein-coding
interferon	protein-coding
RIKEN cDN.	protein-coding
calnexin	protein-coding
glycosyltra	protein-coding
olfactory re	protein-coding
trafficking	protein-coding
family with	protein-coding
gremlin 2 h	protein-coding
death asso	protein-coding
monoglyce	protein-coding
ADAMTS-lil	protein-coding
ATPase, cla	protein-coding
ATP syntha	protein-coding
Rho GTPas	protein-coding
SET domair	protein-coding
E74-like fac	protein-coding
F-box prote	protein-coding

inhibitor of	protein-coding
solute carri	protein-coding
spectrin re	protein-coding
sterol-C5-d	protein-coding
RIKEN cDN.	protein-coding
teneurin tr	protein-coding
zinc finger	protein-coding
src-like ada	protein-coding
F-box and l	protein-coding
nucleic acid	protein-coding
ribosomal p	protein-coding
phospholip	protein-coding
dehydroge	protein-coding
phosphatas	protein-coding
RPTOR inde	protein-coding
SUB1 homo	protein-coding
protein pho	protein-coding
MAP kinase	protein-coding
paired box	protein-coding
RAD51 hom	protein-coding
kinesin fam	protein-coding
L-3-hydroxy	protein-coding
motile sper	protein-coding
synovial sa	protein-coding
junction-m	protein-coding
thymosin, b	protein-coding
dynein cyto	protein-coding
ubiquitin-c	protein-coding
heat shock	protein-coding
DNA segme	protein-coding
breast carc	protein-coding
UPF3 regul	protein-coding
isocitrate d	protein-coding
glutaminyl-	protein-coding
insulin rece	protein-coding
predicted g	protein-coding
kelch doma	protein-coding
lymphocyte	protein-coding
potassium	protein-coding
ubiquinol-c	protein-coding
solute carri	protein-coding
protein pho	protein-coding
lymphocyte	protein-coding
ring finger	protein-coding
GTPase act	protein-coding
insulin rece	protein-coding
non-catalyt	protein-coding

cell divisior	protein-coding
acylpeptide	protein-coding
glycoprotei	protein-coding
splicing fac	protein-coding
RIKEN cDN.	protein-coding
ring finger	protein-coding
osteoclast :	protein-coding
smoothelir	protein-coding
DEAD (Asp-	protein-coding
lipopolysac	protein-coding
seven in ab	protein-coding
MARVEL (n	protein-coding
pannexin 1	protein-coding
islet cell au	protein-coding
zinc finger,	protein-coding
family with	protein-coding
ring finger	protein-coding
NUAK fami	protein-coding
signal-indu	protein-coding
U2 small nu	protein-coding
deoxyribor	protein-coding
CD9 antige	protein-coding
A kinase (P	protein-coding
abhydrolas	protein-coding
PAK1 intera	protein-coding
transcriptic	protein-coding
ER membra	protein-coding
transformii	protein-coding
chromodor	protein-coding
CDK5 regul	protein-coding
La ribonucl	protein-coding
zinc finger	protein-coding
MOB kinas	protein-coding
coiled-coil	protein-coding
cysteine co	protein-coding
sphingosin	protein-coding
RIKEN cDN.	protein-coding
cyclin E2	protein-coding
farnesyltra	protein-coding
S100 calciu	protein-coding
cDNA sequ	protein-coding
Yamaguchi	protein-coding
Parkinson (	protein-coding
hedgehog a	protein-coding
ADP-ribosy	protein-coding
mitochond	protein-coding
RAD17 hon	protein-coding

paired-like	protein-coding
DIP2 disco-	protein-coding
proteasom	protein-coding
DnaJ (Hsp4	protein-coding
inositol pol	protein-coding
DNA prima	protein-coding
Josephin d	protein-coding
syntrophin	protein-coding
forkhead b	protein-coding
src homolo	protein-coding
twisted gas	protein-coding
SUZ RNA bi	protein-coding
ski sarcom	protein-coding
sprouty ho	protein-coding
toll-like rec	protein-coding
WD and te	protein-coding
malonyl Co	protein-coding
centrosom	protein-coding
CDC14 cell	protein-coding
RIKEN cDN	protein-coding
SWI/SNF re	protein-coding
non imprin	protein-coding
Fc recepto	protein-coding
ornithine d	protein-coding
HCLS1 binc	protein-coding
Erbb2 inter	protein-coding
guanylate l	protein-coding
parvin, gan	protein-coding
sarcospan	protein-coding
zinc finger	protein-coding
BCL2-assoc	protein-coding
caspase act	protein-coding
developme	protein-coding
RAB44, me	protein-coding
arginyl ami	protein-coding
synaptotag	protein-coding
lemur tyros	protein-coding
RMI2, RecC	protein-coding
glutaredox	protein-coding
pyruvate d	protein-coding
mediator c	protein-coding
Wolf-Hirscl	protein-coding
general tra	protein-coding
eukaryotic	protein-coding
solute carri	protein-coding
Ral GTPase	protein-coding
family with	protein-coding

glucosamir	protein-coding
dickkopf hc	protein-coding
forkhead b	protein-coding
ribosomal	protein-coding
mesoderm	protein-coding
solute carri	protein-coding
cytochrom	protein-coding
proteasom	protein-coding
cytosolic th	protein-coding
neuron nav	protein-coding
phosphodi	protein-coding
peroxiredo	protein-coding
staphyloco	protein-coding
UPF2 regul	protein-coding
WD repeat	protein-coding
pleckstrin a	protein-coding
LAS1-like (S	protein-coding
5' nucleotic	protein-coding
cell adhesic	protein-coding
zinc finger	protein-coding
translocase	protein-coding
zinc and rir	protein-coding
PRKC, apop	protein-coding
signal reco	protein-coding
signal trans	protein-coding
mitochond	protein-coding
lipoma HM	protein-coding
ral guanine	protein-coding
ARP5 actin	protein-coding
epsin 1	protein-coding
poly (ADP-r	protein-coding
olfactory re	protein-coding
paralemmi	protein-coding
integral me	protein-coding
peroxisoma	protein-coding
glutathione	protein-coding
tetratricop	protein-coding
nudix (nucl	protein-coding
Yip1 domai	protein-coding
RIKEN cDN	protein-coding
3'-phospho	protein-coding
WD repeat	protein-coding
olfactory re	protein-coding
lysosomal t	protein-coding
C-type lect	protein-coding
SPRY doma	protein-coding
ubiquitin-li	protein-coding

transcriptic	protein-coding
nuclear rec	protein-coding
aldehyde d	protein-coding
ring finger	protein-coding
glutamine	protein-coding
dual-specif	protein-coding
notch 2	protein-coding
RIKEN cDN	protein-coding
N-acetylglu	protein-coding
annexin A7	protein-coding
FCH and dc	protein-coding
ameloblast	protein-coding
tripartite m	protein-coding
phospholip	protein-coding
HERPUD fa	protein-coding
RNA bindin	protein-coding
NIMA (neve	protein-coding
Rap guanin	protein-coding
zinc finger	protein-coding
diacylglyce	protein-coding
componen	protein-coding
5-hydroxyr	protein-coding
rosbin, rou	protein-coding
protein pho	protein-coding
coiled-coil	protein-coding
family with	protein-coding
zinc finger	protein-coding
leukemia ir	protein-coding
testis expre	protein-coding
fibroblast g	protein-coding
tRNA meth	protein-coding
zinc finger	protein-coding
brain glyco	protein-coding
ethanolam	protein-coding
family with	protein-coding
Kruppel-lik	protein-coding
androgen c	protein-coding
serine pept	protein-coding
leucine rich	protein-coding
v-ral simiar	protein-coding
sperm mot	protein-coding
cholinergic	protein-coding
CDC28 prot	protein-coding
Ras associa	protein-coding
rosbin, rou	protein-coding
lysine (K)-s	protein-coding
nuclear fac	protein-coding

potassium	protein-coding
kallikrein re	protein-coding
RIKEN cDN.	protein-coding
2-oxogluta	protein-coding
trinucleotic	protein-coding
protein tyr	protein-coding
zinc finger	protein-coding
jumonji, AT	protein-coding
pre-mRNA	protein-coding
methyltran	protein-coding
sterile alph	protein-coding
diacylglyce	protein-coding
protein tyr	protein-coding
translocase	protein-coding
chondroitir	protein-coding
guanylate c	protein-coding
G protein-c	protein-coding
host cell fa	protein-coding
RIKEN cDN.	protein-coding
LIM domain	protein-coding
peptidyl-tR	protein-coding
f-box prote	protein-coding
nuclear prc	protein-coding
zinc finger	protein-coding
CD53 antig	protein-coding
signal-indu	protein-coding
ribosomal		protein-coding
ER membra	protein-coding
antizyme ir	protein-coding
cadherin-re	protein-coding
coiled-coil	protein-coding
syntrophin	protein-coding
cold induci	protein-coding
myelin pro	protein-coding
inositol 1,3	protein-coding
pygopus 1	protein-coding
KN motif ar	protein-coding
lysine (K)-s	protein-coding
cold shock	protein-coding
cullin 3	protein-coding
telomeric r	protein-coding
forkhead b	protein-coding
ski sarcoma	protein-coding
GINS comp	protein-coding
zinc finger	protein-coding
zinc finger	protein-coding
zinc finger	protein-coding

BMP2 indu	protein-coding
period circ	protein-coding
adenosine	protein-coding
neurexin III	protein-coding
cadherin, E	protein-coding
WD repeat	protein-coding
angiopoieti	protein-coding
SFT2 doma	protein-coding
transmemb	protein-coding
metastasis	protein-coding
interleukin	protein-coding
leucine rich	protein-coding
DNA methy	protein-coding
C1D nuclea	protein-coding
high mobili	protein-coding
limb and n	protein-coding
zinc finger	protein-coding
XRCC6 binc	protein-coding
La ribonucl	protein-coding
neural prec	protein-coding
RIKEN cDN	protein-coding
DIP2 disco-	protein-coding
ubiquitin s	protein-coding
STE20-like	protein-coding
carbonic ar	protein-coding
RIKEN cDN	protein-coding
myosin, he	protein-coding
GTPase act	protein-coding
expressed	protein-coding
FCH domai	protein-coding
lymphocyt	protein-coding
potassium	protein-coding
upstream t	protein-coding
RAR-relate	protein-coding
CD9 antige	protein-coding
GTP bindin	protein-coding
tetratricop	protein-coding
RNA bindin	protein-coding
protein kin	protein-coding
TSC22 dom	protein-coding
anthrax to	protein-coding
retinol binc	protein-coding
exostoses (	protein-coding
DnaJ (Hsp4	protein-coding
oxidative s	protein-coding
zinc finger	protein-coding
ubiquitin s	protein-coding

farnesyltra	protein-coding
zinc finger	protein-coding
protein-L-is	protein-coding
ArfGAP wit	protein-coding
cysteine ric	protein-coding
Kruppel-lik	protein-coding
DEAD (Asp-	protein-coding
phosphodi	protein-coding
microfibrill	protein-coding
Yamaguchi	protein-coding
leukocyte i	protein-coding
phospholip	protein-coding
kelch-like 8	protein-coding
TraB doma	protein-coding
ATP syntha	protein-coding
polyadenyl	protein-coding
zinc finger	protein-coding
ELK3, mem	protein-coding
vesicle-ass	protein-coding
cyclin-depe	protein-coding
TBC1 doma	protein-coding
homeobox	protein-coding
TRAF famil	protein-coding
TAF15 RNA	protein-coding
Ras associa	protein-coding
tubulin tyr	protein-coding
membrane	protein-coding
SEC14-like	protein-coding
pyrroline-5	protein-coding
SNF relatec	protein-coding
selectin, pl	protein-coding
RAB20, me	protein-coding
renalase, F	protein-coding
dehydroge	protein-coding
UBX domai	protein-coding
phosphodi	protein-coding
phospholip	protein-coding
inhibitor of	protein-coding
opioid grov	protein-coding
interferon	protein-coding
transient re	protein-coding
ribonucleas	protein-coding
cyclin D3	protein-coding
ATPase, H+	protein-coding
peptidylpro	protein-coding
dihydropyr	protein-coding
NIMA (neve	protein-coding

cytochrom	protein-coding
CD2 antige	protein-coding
biogenesis	protein-coding
predicted g	protein-coding
arachidona	protein-coding
far upstrea	protein-coding
tripartite m	protein-coding
cyclin-depe	protein-coding
leucine, glu	protein-coding
neutrophil	protein-coding
dual specif	protein-coding
schlafen 14	protein-coding
ATPase, Na	protein-coding
Rho GTPase	protein-coding
proteasom	protein-coding
transient re	protein-coding
arsA arseni	protein-coding
secretory c	protein-coding
UDP-Gal:be	protein-coding
SprT-like N	protein-coding
raftlin fami	protein-coding
leucine rich	protein-coding
cerebral ca	protein-coding
signal trans	protein-coding
bridging int	protein-coding
RIKEN cDNA	protein-coding
integrin be	protein-coding
BCL6 co-re	protein-coding
solute carri	protein-coding
dehydroge	protein-coding
5'-nucleoti	protein-coding
STEAP fami	protein-coding
GLI pathog	protein-coding
TBC1 doma	protein-coding
zinc finger	protein-coding
protein pho	protein-coding
TAF15 RNA	protein-coding
LIM domai	protein-coding
pleckstrin h	protein-coding
methyl-CpG	protein-coding
coiled-coil	protein-coding
brevican	protein-coding
LYR motif c	protein-coding
peroxisoma	protein-coding
N(alpha)-ac	protein-coding
family with	protein-coding
Sec24 relat	protein-coding

interferon ;	protein-coding
protein tyr	protein-coding
paraoxona:	protein-coding
TBC1 doma	protein-coding
CTD (carbo	protein-coding
mannosida	protein-coding
frizzled hor	protein-coding
protein ph	protein-coding
FK506 bind	protein-coding
synaptonei	protein-coding
membrane	protein-coding
transmemb	protein-coding
Janus kinas	protein-coding
calcium/ca	protein-coding
branched c	protein-coding
spermatid	protein-coding
DIRAS fami	protein-coding
transmemb	protein-coding
low density	protein-coding
caspase ac	protein-coding
WD repeat	protein-coding
guanine nu	protein-coding
MICAL-like	protein-coding
deoxynucle	protein-coding
chemokine	protein-coding
RAD9 hom	protein-coding
GTP-bindin	protein-coding
ST3 beta-g	protein-coding
signal trans	protein-coding
saccharopi	protein-coding
ubiquitin-c	protein-coding
non-SMC c	protein-coding
lymphocyt	protein-coding
coatomer p	protein-coding
schwannor	protein-coding
dehydroge	protein-coding
golgi SNAP	protein-coding
arachidona	protein-coding
RNA bindin	protein-coding
pyroglutar	protein-coding
adaptor-re	protein-coding
tensin 3	protein-coding
activin rece	protein-coding
proteasom	protein-coding
SWI/SNF re	protein-coding
peroxiredo	protein-coding
UTP3, smal	protein-coding

E74-like fac	protein-coding
formin binc	protein-coding
stearoyl-Cc	protein-coding
cancer susc	protein-coding
family with	protein-coding
homer hon	protein-coding
gamma-glu	protein-coding
mitochond	protein-coding
claudin 1	protein-coding
ankyrin rep	protein-coding
aspartyl-tR	protein-coding
stefin A3	protein-coding
Sjogren's s	protein-coding
tumor necr	protein-coding
chemokine	protein-coding
paired-like	protein-coding
membrane	protein-coding
SMAD fami	protein-coding
ubiquitin p	protein-coding
BCL6 intera	protein-coding
calcium act	protein-coding
ubiquitin-c	protein-coding
leucine rich	protein-coding
argonaute	protein-coding
arachidona	protein-coding
zinc finger	protein-coding
ribosomal	protein-coding
ribosomal	protein-coding
paired-like	protein-coding
6-pyruvoyl-	protein-coding
bromodom	protein-coding
triosephos	protein-coding
RCC1 doma	protein-coding
zinc finger	protein-coding
hexosamin	protein-coding
vascular ce	protein-coding
transmemb	protein-coding
branched c	protein-coding
CD47 antig	protein-coding
sperm anti	protein-coding
death effec	protein-coding
Ras and Ra	protein-coding
zinc finger	protein-coding
proline-ser	protein-coding
myelin pro	protein-coding
phosphoglu	protein-coding
zinc finger	protein-coding

general tra	protein-coding
cleft lip and	protein-coding
acyl-CoA th	protein-coding
transcriptic	protein-coding
nudix (nucl	protein-coding
opioid rece	protein-coding
mitogen-ac	protein-coding
cardiolipin	protein-coding
La ribonucl	protein-coding
protein pho	protein-coding
colony stim	protein-coding
adenylate c	protein-coding
junction ad	protein-coding
SWI/SNF re	protein-coding
tripartite m	protein-coding
dual specif	protein-coding
progestin a	protein-coding
leucine rich	protein-coding
sortilin-rela	protein-coding
RIKEN cDN.	protein-coding
ER degrada	protein-coding
myeloid/lyr	protein-coding
RIKEN cDN.	protein-coding
suppressor	protein-coding
autophagy	protein-coding
zinc finger	protein-coding
glutaredox	protein-coding
angiopoieti	protein-coding
centrosom	protein-coding
immunoglo	protein-coding
activin rece	protein-coding
signal trans	protein-coding
cell divisior	protein-coding
ribulose-5-	protein-coding
MPV17 mit	protein-coding
ribosomal	protein-coding
zinc finger	protein-coding
predicted g	protein-coding
mitochond	protein-coding
translocase	protein-coding
protein-tyr	protein-coding
protein O-g	protein-coding
eukaryotic	protein-coding
growth fac	protein-coding
SFT2 doma	protein-coding
small integ	protein-coding
kelch-like 3	protein-coding

carcinoeml	protein-coding
Yip1 domai	protein-coding
receptor ac	protein-coding
chromodor	protein-coding
papillary re	protein-coding
B cell CLL/l	protein-coding
frequently	protein-coding
TBC1 doma	protein-coding
casein kina	protein-coding
negative el	protein-coding
gasdermin	protein-coding
high densit	protein-coding
oxysterol b	protein-coding
myosin, ligl	protein-coding
cyclin-depe	protein-coding
carbamoyl-	protein-coding
mbt domai	protein-coding
acyl-Coenz	protein-coding
UbiA preny	protein-coding
adenosine	protein-coding
peroxisoma	protein-coding
capicua ho	protein-coding
zinc finger,	protein-coding
zinc finger,	protein-coding
E26 avian l	protein-coding
glycerol-3-|	protein-coding
asparagine	protein-coding
YTH domai	protein-coding
adaptor-re	protein-coding
V-set and t	protein-coding
tRNA meth	protein-coding
mediator c	protein-coding
lin-9 homo	protein-coding
transglutar	protein-coding
LIM domaiı	protein-coding
DSN1, MIN	protein-coding
zinc finger	protein-coding
mitochond	protein-coding
zinc finger,	protein-coding
ubiquitin-c	protein-coding
calcitonin r	protein-coding
Rho GTPase	protein-coding
F-box and l	protein-coding
immediate	protein-coding
centrosom	protein-coding
RIKEN cDN.	protein-coding
signal-indu	protein-coding

Cbp/p300-i	protein-coding
zinc finger	protein-coding
formin binc	protein-coding
antigen ide	protein-coding
immunity-r	protein-coding
CDC42 sma	protein-coding
phospholip	protein-coding
echinoderr	protein-coding
carbohydra	protein-coding
CCAAT/enh	protein-coding
REX1, RNA	protein-coding
multiple C2	protein-coding
SH2B adap	protein-coding
ras homolc	protein-coding
phosphatic	protein-coding
CASP2 and	protein-coding
prostaglan	protein-coding
annexin A2	protein-coding
von Willebr	protein-coding
TBC1 doma	protein-coding
exportin, tl	protein-coding
RNA bindin	protein-coding
aspartic pe	protein-coding
stress-indu	protein-coding
TRAF-intera	protein-coding
stonin 2	protein-coding
transducin-	protein-coding
peptidase i	protein-coding
ADP-ribosy	protein-coding
sulfotransf	protein-coding
cell adhesi	protein-coding
transmemb	protein-coding
rearranged	protein-coding
potassium	protein-coding
ubiquitin s	protein-coding
uromoduli	protein-coding
mitochond	protein-coding
USO1 vesic	protein-coding
1-acylglyce	protein-coding
protein ph	protein-coding
TBC1 doma	protein-coding
mitochond	protein-coding
sushi doma	protein-coding
TSPY-like 3	protein-coding
FIG4 homo	protein-coding
predicted g	protein-coding
cyclin-depe	protein-coding

F-box prote	protein-coding
thioredoxir	protein-coding
ankyrin rep	protein-coding
hydroxyste	protein-coding
small nucle	protein-coding
ATPase, Ca	protein-coding
leucine-ricl	protein-coding
pleckstrin l	protein-coding
hypoxia inc	protein-coding
abl-interac	protein-coding
pyroglutam	protein-coding
coiled-coil	protein-coding
acetyl-Coel	protein-coding
R-spondin l	protein-coding
B cell trans	protein-coding
N-acetylglu	protein-coding
GNAS (guai	protein-coding
protein phc	protein-coding
protein phc	protein-coding
RAB37, me	protein-coding
transducin-	protein-coding
zinc finger	protein-coding
family with	protein-coding
cullin 1	protein-coding
destrin	protein-coding
sex comb c	protein-coding
CD24a anti	protein-coding
contactin a	protein-coding
ubiquitin a	protein-coding
genetic sup	protein-coding
glutathione	protein-coding
DNA-dama	protein-coding
zinc finger	protein-coding
integrator	protein-coding
tubulin, alp	protein-coding
glucose-fru	protein-coding
AT rich inte	protein-coding
ubiquitin s	protein-coding
aspartate-l	protein-coding
leucine rich	protein-coding
serine/thre	protein-coding
microsoma	protein-coding
Rho guanir	protein-coding
histone dea	protein-coding
componen	protein-coding
F-box and	protein-coding
c-Maf indu	protein-coding

nuclear rec	protein-coding
zinc finger,	protein-coding
macrophag	protein-coding
anaphase-p	protein-coding
SLAIN moti	protein-coding
solute carri	protein-coding
ankyrin rep	protein-coding
IQ motif ar	protein-coding
F-box prote	protein-coding
ADP-ribosy	protein-coding
tRNA splici	protein-coding
regulatory	protein-coding
lymphocyte	protein-coding
family with	protein-coding
transmemb	protein-coding
myosin ID	protein-coding
ectodyspla	protein-coding
deafness, a	protein-coding
unc-119 hc	protein-coding
ras respons	protein-coding
survival mc	protein-coding
legumain	protein-coding
chromodor	protein-coding
ring finger	protein-coding
regulation	protein-coding
dystrophin	protein-coding
interferon	protein-coding
ATP-bindin	protein-coding
E1A binding	protein-coding
Epstein-Bar	protein-coding
T cell recep	protein-coding
G protein-c	protein-coding
dehydroge	protein-coding
serine/thre	protein-coding
trans-actin	protein-coding
acyl-coenzy	protein-coding
threonine a	protein-coding
dehydroge	protein-coding
RIKEN cDN	protein-coding
thymoma v	protein-coding
nei like 3 (E	protein-coding
family with	protein-coding
SEC23A (S.	protein-coding
phosphorib	protein-coding
F-box and l	protein-coding
transmemb	protein-coding
coiled-coil	protein-coding

tyrosinase-	protein-coding
Kruppel-lik	protein-coding
proline-ser	protein-coding
Sfi1 homol	protein-coding
polymerase	protein-coding
eukaryotic	protein-coding
unc-50 hor	protein-coding
syntaxin 2	protein-coding
X-ray repai	protein-coding
ethanolam	protein-coding
additional	protein-coding
clavesin 1	protein-coding
U2 snRNP-a	protein-coding
glutamate-	protein-coding
family with	protein-coding
ribosomal	protein-coding
solute carri	protein-coding
cathepsin H	protein-coding
protein pho	protein-coding
cytochrom	protein-coding
centrosom	protein-coding
fibroblast g	protein-coding
thymopoie	protein-coding
family with	protein-coding
pyruvate d	protein-coding
patched dc	protein-coding
erythrocyte	protein-coding
cell death i	protein-coding
IQ motif co	protein-coding
adenylate c	protein-coding
transcriptic	protein-coding
post-GPI at	protein-coding
surfactant	protein-coding
RIKEN cDN.	protein-coding
eukaryotic	protein-coding
xylosyltran	protein-coding
LIM domai	protein-coding
t-complex	protein-coding
RIKEN cDN.	protein-coding
cytidine mo	protein-coding
nucleobind	protein-coding
ELAV (emb	protein-coding
calcitonin r	protein-coding
lymphocyte	protein-coding
coiled-coil	protein-coding
alpha-kinas	protein-coding
transmemb	protein-coding

family with	protein-coding
chondroitir	protein-coding
carbohydra	protein-coding
diacylglyce	protein-coding
RIKEN cDN.	protein-coding
hexokinase	protein-coding
matrix met	protein-coding
hypoxia inc	protein-coding
cytoplasmi	protein-coding
mesencepl	protein-coding
olfactory re	protein-coding
serine (or c	protein-coding
integrin alp	protein-coding
heme bind	protein-coding
WAS/WASI	protein-coding
kelch doma	protein-coding
zinc finger	protein-coding
inhibin bet	protein-coding
bromodom	protein-coding
ataxin 7-lik	protein-coding
UDP-Gal:be	protein-coding
LIM domair	protein-coding
protein kin	protein-coding
death effec	protein-coding
Fc receptoi	protein-coding
gasdermin	protein-coding
solute carri	protein-coding
ArfGAP wit	protein-coding
cyclin M3	protein-coding
GINS comp	protein-coding
transmeml	protein-coding
1-acylglyce	protein-coding
cordon-ble	protein-coding
family with	protein-coding
syndecan b	protein-coding
cytotoxic g	protein-coding
zinc finger	protein-coding
transmeml	protein-coding
TBC1 doma	protein-coding
autism sus	protein-coding
neuroblast	protein-coding
surfeit gen	protein-coding
ring finger	protein-coding
PAX interac	protein-coding
farnesyltra	protein-coding
Williams Be	protein-coding
RAD54 like	protein-coding

histocompa	protein-coding
polymerase	protein-coding
coiled-coil-	protein-coding
mannosida	protein-coding
aminopept	protein-coding
caspase 8	protein-coding
trans-actin	protein-coding
VMA21 vac	protein-coding
taperin	protein-coding
cytochrom	protein-coding
prolactin re	protein-coding
prosaposin	protein-coding
PWP1 hom	protein-coding
kelch-like E	protein-coding
gamma-am	protein-coding
polyribonu	protein-coding
ribosomal	protein-coding
cyclin I	protein-coding
enhancer c	protein-coding
dynein, axc	protein-coding
deleted in	protein-coding
trichohyali	protein-coding
golgi meml	protein-coding
glucan (1,4	protein-coding
zinc finger,	protein-coding
DENN/MAI	protein-coding
within bgcr	protein-coding
chemokine	protein-coding
striatin, cal	protein-coding
tumor necr	protein-coding
ATP syntha	protein-coding
lysosomal-	protein-coding
GID comple	protein-coding
neuregulin	protein-coding
RNA bindin	protein-coding
phosphatic	protein-coding
enhancer c	protein-coding
furry homc	protein-coding
mitochond	protein-coding
HtrA serine	protein-coding
protein phc	protein-coding
kinesin fam	protein-coding
predicted g	protein-coding
tubulin tyrc	protein-coding
MAS-relate	protein-coding
N-myristoy	protein-coding
BCL2 modi	protein-coding

dynein, axc	protein-coding
PRP19/PSC	protein-coding
retinoblast	protein-coding
primase an	protein-coding
growth arr	protein-coding
carbohydra	protein-coding
lin-7 homo	protein-coding
integrin alp	protein-coding
ankyrin rep	protein-coding
ankyrin rep	protein-coding
zinc finger	protein-coding
centrosom	protein-coding
ATPase, cla	protein-coding
LEM domai	protein-coding
inner mem	protein-coding
angiotensir	protein-coding
lamin B rec	protein-coding
signal-indu	protein-coding
N-6 adenin	protein-coding
RNA bindin	protein-coding
endonuclea	protein-coding
expressed :	protein-coding
anaphase p	protein-coding
MAX gene	protein-coding
CAS1 doma	protein-coding
Rho-relate	protein-coding
calcium cha	protein-coding
family with	protein-coding
pleckstrin a	protein-coding
transportir	protein-coding
LON peptic	protein-coding
dihydrolipo	protein-coding
regulatory	protein-coding
PR domain	protein-coding
ankyrin rep	protein-coding
somatostat	protein-coding
F-box prote	protein-coding
heterogene	protein-coding
carbohydra	protein-coding
centrin 3	protein-coding
cleavage ar	protein-coding
death indu	protein-coding
ankyrin rep	protein-coding
FYVE, RhoC	protein-coding
excision re	protein-coding
actin, beta	protein-coding
brain and r	protein-coding

Kruppel-lik	protein-coding
zinc finger,	protein-coding
homeodom	protein-coding
APC memb	protein-coding
cell cycle a	protein-coding
zinc finger	protein-coding
CCR4-NOT	protein-coding
sema doma	protein-coding
cytochrom	protein-coding
insulin-like	protein-coding
ring finger	protein-coding
forkhead b	protein-coding
fatty acid b	protein-coding
inositol (m	protein-coding
leucine rich	protein-coding
ribonuclea	protein-coding
ribonuclea	protein-coding
peroxisom	protein-coding
ChaC, catio	protein-coding
WW domai	protein-coding
SR-related	protein-coding
myogenin	protein-coding
GTP bindin	protein-coding
leucine rich	protein-coding
cDNA sequ	protein-coding
isocitrate d	protein-coding
potassium	protein-coding
cysteine ar	protein-coding
RIKEN cDN	protein-coding
nyctalopin	protein-coding
solute carri	protein-coding
monocyte	protein-coding
lectin, gala	protein-coding
MAX dimer	protein-coding
RIKEN cDN	protein-coding
leucine rich	protein-coding
G protein-c	protein-coding
GPN-loop (	protein-coding
ribonuclea	protein-coding
glutamine-	protein-coding
sushi doma	protein-coding
nuclear fac	protein-coding
chromosor	protein-coding
ST3 beta-g	protein-coding
proteasom	protein-coding
pyruvate d	protein-coding
mitochond	protein-coding

coagulatior	protein-coding
chemokine	protein-coding
tudor doma	protein-coding
kit oncoger	protein-coding
solute carri	protein-coding
zyxin	protein-coding
cysteine-ric	protein-coding
procollager	protein-coding
CDC42 sma	protein-coding
CWF19-like	protein-coding
leukotriene	protein-coding
vaccinia rel	protein-coding
pumilio RN	protein-coding
zinc finger	protein-coding
ankyrin rep	protein-coding
membrane	protein-coding
ribosomal	protein-coding
Rho GTPase	protein-coding
solute carri	protein-coding
zinc finger	protein-coding
DNA segme	protein-coding
family with	protein-coding
heat shock	protein-coding
monoglyce	protein-coding
retinoid X r	protein-coding
pyroglutam	protein-coding
ATPase, cla	protein-coding
protein pho	protein-coding
ribose 5-ph	protein-coding
GIPC PDZ d	protein-coding
ubiquitin-c	protein-coding
solute carri	protein-coding
charged mu	protein-coding
fucosyltran	protein-coding
lysine (K)-s	protein-coding
heat shock	protein-coding
forkhead b	protein-coding
myosin VIIA	protein-coding
zinc finger	protein-coding
RCSD doma	protein-coding
carboxypep	protein-coding
SDE2 telom	protein-coding
transcriptio	protein-coding
transmemb	protein-coding
paired-like	protein-coding
dishevelled	protein-coding
high-mobil	protein-coding

uridine-cyt	protein-coding
phosphoin	protein-coding
zinc finger	protein-coding
mastermin	protein-coding
protein ph	protein-coding
mitochond	protein-coding
NEDD4 bin	protein-coding
endoplasm	protein-coding
ribosomal	protein-coding
histocompa	protein-coding
tumor necr	protein-coding
caspase 1	protein-coding
transmemb	protein-coding
zinc finger	protein-coding
myosin, lig	protein-coding
purine-rich	protein-coding
glucosamir	protein-coding
metallothic	protein-coding
translocase	protein-coding
C1q and tu	protein-coding
glucosamir	protein-coding
RIKEN cDN	protein-coding
toll-like rec	protein-coding
crystallin, g	protein-coding
pleckstrin h	protein-coding
plectin	protein-coding
small integ	protein-coding
leucine rich	protein-coding
dual specif	protein-coding
Smg-6 hom	protein-coding
nuclear exp	protein-coding
glutathione	protein-coding
interferon	protein-coding
nuclear VC	protein-coding
dynein, axo	protein-coding
nucleic acic	protein-coding
Nedd4 fam	protein-coding
fos-like ant	protein-coding
CWC22 spli	protein-coding
ring finger	protein-coding
sirtuin 1	protein-coding
killer cell le	protein-coding
Kv channel	protein-coding
parathyroid	protein-coding
spermatog	protein-coding
histone clu	protein-coding
LPS-induce	protein-coding

ataxin 2	protein-coding
sorting nex	protein-coding
S100 calciu	protein-coding
ribosomal	protein-coding
transcriptic	protein-coding
GrpE-like 1	protein-coding
CLPTM1-lik	protein-coding
ribosomal	protein-coding
signal pept	protein-coding
LIM domai	protein-coding
ribosomal	protein-coding
Kruppel-lik	protein-coding
F-box prote	protein-coding
nuclear fac	protein-coding
Rho GTPas	protein-coding
opticin	protein-coding
early grow	protein-coding
protein arg	protein-coding
brain-speci	protein-coding
zinc finger	protein-coding
NLR family,	protein-coding
phosphatic	protein-coding
predicted g	protein-coding
cDNA sequ	protein-coding
transducin	protein-coding
triggering r	protein-coding
RIKEN cDN	protein-coding
ankyrin rep	protein-coding
latrophilin	protein-coding
lysine (K)-s	protein-coding
DNA segme	protein-coding
ribonuclea	protein-coding
cysteine-se	protein-coding
zinc finger	protein-coding
metallothic	protein-coding
family with	protein-coding
sushi doma	protein-coding
phosphatic	protein-coding
trafficking	protein-coding
C-type lect	protein-coding
SDE2 telon	protein-coding
TM2 doma	protein-coding
intersectin	protein-coding
zinc finger	protein-coding
polymeras	protein-coding
DnaJ (Hsp4	protein-coding
prostaglan	protein-coding

cyclin-depe	protein-coding
solute carri	protein-coding
intraflagell	protein-coding
regulator o	protein-coding
protein pho	protein-coding
lipopolysac	protein-coding
phospholip	protein-coding
myocardial	protein-coding
interferon	protein-coding
fragile X m	protein-coding
zinc finger	protein-coding
F-box and \	protein-coding
RAD51 hon	protein-coding
phosphoin	protein-coding
thioredoxir	protein-coding
ST3 beta-g	protein-coding
low density	protein-coding
tetraspanir	protein-coding
PC4 and SF	protein-coding
cilia and fla	protein-coding
CCZ1 vacuo	protein-coding
immunoglo	protein-coding
coxsackie v	protein-coding
macrophag	protein-coding
ets variant	protein-coding
potassium	protein-coding
enoyl-Coer	protein-coding
nucleopori	protein-coding
microtubul	protein-coding
Morf4 fam	protein-coding
trafficking	protein-coding
importin 9	protein-coding
glutathione	protein-coding
serrate RN	protein-coding
transmemb	protein-coding
sphingosin	protein-coding
fission 1 (m	protein-coding
IQ motif ar	protein-coding
activin A re	protein-coding
G-protein c	protein-coding
ancient ubi	protein-coding
thrombosp	protein-coding
interferon	protein-coding
mitogen-ac	protein-coding
Jun dimeriz	protein-coding
CD44 antig	protein-coding
F-box prote	protein-coding

Kv channel	protein-coding
scaffolding	protein-coding
thymopoie	protein-coding
endoplasm	protein-coding
solute carri	protein-coding
maestro he	protein-coding
mago-nash	protein-coding
vesicle trar	protein-coding
immediate	protein-coding
ladybird hc	protein-coding
heat shock	protein-coding
nuclear fra	protein-coding
iron-sulfur	protein-coding
inositol 1,4	protein-coding
WD repeat	protein-coding
adenylate l	protein-coding
family with	protein-coding
HAUS augn	protein-coding
chromobox	protein-coding
RIKEN cDN.	protein-coding
ataxin 7-lik	protein-coding
serine/argi	protein-coding
ubiquinol-c	protein-coding
PAP associa	protein-coding
dynein ligh	protein-coding
potassium	protein-coding
S-adenosyl	protein-coding
protein pho	protein-coding
F-box and l	protein-coding
DNA-dama	protein-coding
ATPase, cla	protein-coding
C-terminal	protein-coding
UTP6, smal	protein-coding
armadillo r	protein-coding
La ribonucl	protein-coding
ataxin 2	protein-coding
N(alpha)-ac	protein-coding
neuron der	protein-coding
family with	protein-coding
WD repeat	protein-coding
DDB1 and (	protein-coding
polo-like ki	protein-coding
Nipped-B h	protein-coding
regulation	protein-coding
phosphofru	protein-coding
RIKEN cDN.	protein-coding
E74-like fac	protein-coding

growth arr	protein-coding
NLR family,	protein-coding
very low de	protein-coding
KRR1, smal	protein-coding
transmemb	protein-coding
aminoadipa	protein-coding
Rho GTPase	protein-coding
tripartite m	protein-coding
acrosomal	protein-coding
predicted g	protein-coding
zinc finger,	protein-coding
heat shock	protein-coding
netrin G1	protein-coding
LYR motif c	protein-coding
glycyl-tRNA	protein-coding
mitogen-ac	protein-coding
coiled coil c	protein-coding
zinc finger	protein-coding
RNA bindin	protein-coding
phosphatas	protein-coding
ubiquitin a	protein-coding
BCL2/aden	protein-coding
tec protein	protein-coding
Rap guanin	protein-coding
angio-asso	protein-coding
cytoplasmi	protein-coding
microsoma	protein-coding
zinc finger,	protein-coding
tumor necr	protein-coding
transmemb	protein-coding
endothelin	protein-coding
peroxisoma	protein-coding
ski sarcoma	protein-coding
short chain	protein-coding
akirin 2	protein-coding
family with	protein-coding
salt induci	protein-coding
dedicator c	protein-coding
glutathione	protein-coding
adaptor pr	protein-coding
ubiquitin-li	protein-coding
ATP-bindin	protein-coding
increased s	protein-coding
RAB8B, me	protein-coding
zinc finger	protein-coding
CD244 natu	protein-coding
solute carri	protein-coding

helicase (D	protein-coding
malignant T	protein-coding
cadherin-re	protein-coding
predicted g	protein-coding
phospholip	protein-coding
lysine (K)-s	protein-coding
canopy 3 h	protein-coding
FMS-like ty	protein-coding
ST6 (alpha-	protein-coding
transmemb	protein-coding
A kinase (P	protein-coding
sema doma	protein-coding
zinc finger,	protein-coding
SECIS bindi	protein-coding
kinesin fam	protein-coding
Rho GDP di	protein-coding
snail family	protein-coding
5'-nucleoti	protein-coding
deleted in a	protein-coding
solute carri	protein-coding
JAZF zinc fi	protein-coding
solute carri	protein-coding
nucleopori	protein-coding
glycerol-3-p	protein-coding
ribosomal p	protein-coding
histone clu	protein-coding
poly (ADP-r	protein-coding
bromodom	protein-coding
transcriptic	protein-coding
inhibin bet	protein-coding
forkhead b	protein-coding
farnesyltra	protein-coding
transmemb	protein-coding
avian musc	protein-coding
ATPase, H+	protein-coding
spastic par	protein-coding
tetratricop	protein-coding
fos-like ant	protein-coding
SUN domai	protein-coding
leucine, glu	protein-coding
vesicle-ass	protein-coding
phosphodi	protein-coding
serine/argi	protein-coding
family with	protein-coding
regulating s	protein-coding
additional s	protein-coding
calumenin	protein-coding

phorbol-12	protein-coding
dachshund	protein-coding
IQ motif co	protein-coding
cadherin 1:	protein-coding
RIKEN cDN.	protein-coding
eukaryotic	protein-coding
casein kina	protein-coding
colony stin	protein-coding
glutamate	protein-coding
acyl-Coenz	protein-coding
SR-related	protein-coding
voltage-de	protein-coding
pleckstrin l	protein-coding
calpain 15	protein-coding
human imr	protein-coding
protein pho	protein-coding
dpy-19-like	protein-coding
transglutar	protein-coding
GC-rich pro	protein-coding
myosin reg	protein-coding
PDS5, regu	protein-coding
Rieske (Fe-	protein-coding
mex3 hom	protein-coding
THAP dom	protein-coding
cyclin K	protein-coding
ubiquitin C	protein-coding
NIMA (nev	protein-coding
forkhead b	protein-coding
chemokine	protein-coding
serine/thre	protein-coding
solute carri	protein-coding
lymphocyte	protein-coding
period circ	protein-coding
formin-like	protein-coding
E26 avian l	protein-coding
regulator o	protein-coding
SKI-like	protein-coding
FK506 bind	protein-coding
selenophos	protein-coding
zinc finger	protein-coding
Src-like-ad	protein-coding
CDC14 cell	protein-coding
BCDIN3 do	protein-coding
ring finger	protein-coding
ski sarcom	protein-coding
RAS-like, fa	protein-coding
Mid1 inter	protein-coding

tRNA splici	protein-coding
paraoxonas	protein-coding
retinoblast	protein-coding
cms small r	protein-coding
exonucleas	protein-coding
5'-nucleoti	protein-coding
diphthamir	protein-coding
TRAF famil	protein-coding
Ras and Ra	protein-coding
Fas (TNFRS	protein-coding
periphilin 1	protein-coding
phosphatas	protein-coding
ubiquitin sp	protein-coding
receptor tr	protein-coding
cytochrom	protein-coding
methionine	protein-coding
tumor necr	protein-coding
sprouty ho	protein-coding
RWD doma	protein-coding
family with	protein-coding
Cbp/p300-i	protein-coding
protein tyr	protein-coding
ATP-bindin	protein-coding
bromodom	protein-coding
Myb/SANT	protein-coding
transportin	protein-coding
zinc finger	protein-coding
ras homolo	protein-coding
thymocyte	protein-coding
rhotekin 2	protein-coding
meiosis-sp	protein-coding
neuraminic	protein-coding
transketola	protein-coding
transcriptic	protein-coding
centrosom	protein-coding
ring finger	protein-coding
zyg-11 rela	protein-coding
polybromo	protein-coding
uridine mo	protein-coding
glycerophо	protein-coding
RWD doma	protein-coding
solute carri	protein-coding
nuclear rec	protein-coding
glutaredox	protein-coding
interferon	protein-coding
zinc finger,	protein-coding
Ssu72 RNA	protein-coding

mesoderm	protein-coding
solute carri	protein-coding
ankyrin 3, e	protein-coding
xanthine de	protein-coding
replication	protein-coding
ArfGAP wit	protein-coding
SUN domai	protein-coding
insulin indu	protein-coding
diacylglyce	protein-coding
histamine r	protein-coding
vacuolar pr	protein-coding
ras homolo	protein-coding
lipase matu	protein-coding
oxysterol b	protein-coding
parathyroic	protein-coding
lysocardiol	protein-coding
RAD50 inte	protein-coding
AT hook co	protein-coding
armadillo r	protein-coding
small ArfGA	protein-coding
potassium	protein-coding
formyl pep	protein-coding
RIKEN cDNA	protein-coding
transmemb	protein-coding
coiled-coil	protein-coding
zinc finger	protein-coding
lipocalin 2	protein-coding
transportin	protein-coding
sphingomy	protein-coding
GLTSCR1-li	protein-coding
solute carri	protein-coding
endoplasm	protein-coding
brain-enric	protein-coding
histidine tr	protein-coding
transmemb	protein-coding
T cell recep	protein-coding
protocadhe	protein-coding
tumor necr	protein-coding
zinc finger	protein-coding
ORM1-like	protein-coding
THAP doma	protein-coding
oxidative st	protein-coding
acyl-CoA th	protein-coding
solute carri	protein-coding
inhibitor of	protein-coding
methionine	protein-coding
distal-less l	protein-coding

galanin rec	protein-coding
ribosomal	protein-coding
EH domain	protein-coding
interleukin	protein-coding
expressed	protein-coding
RIMS bindi	protein-coding
integrator	protein-coding
killer cell le	protein-coding
RIKEN cDN.	protein-coding
proviral int	protein-coding
heat shock	protein-coding
latrophilin	protein-coding
SH2 domai	protein-coding
angiopoiet	protein-coding
RAN bindin	protein-coding
prothymos	protein-coding
nitric oxide	protein-coding
solute carri	protein-coding
zinc finger	protein-coding
cell death i	protein-coding
solute carri	protein-coding
RIKEN cDN.	protein-coding
alanine-gly	protein-coding
NMD3 hom	protein-coding
hematopoi	protein-coding
solute carri	protein-coding
clusterin	protein-coding
zinc finger	protein-coding
solute carri	protein-coding
exportin 1,	protein-coding
polymeras	protein-coding
adaptor-re	protein-coding
solute carri	protein-coding
retinoblast	protein-coding
discs, large	protein-coding
postmeioti	protein-coding
RIKEN cDN.	protein-coding
isocitrate d	protein-coding
G protein-c	protein-coding
glucosidas	protein-coding
neutrophili	protein-coding
myosin, he	protein-coding
metallo-be	protein-coding
FSHD regio	protein-coding
ELL associa	protein-coding
6-phospho	protein-coding
C-type lect	protein-coding

caveolin 1,	protein-coding
cyclin-depe	protein-coding
RIKEN cDN.	protein-coding
limb-bud a	protein-coding
phosphatic	protein-coding
transporte	protein-coding
IQ motif co	protein-coding
NADH deh	protein-coding
RNA bindin	protein-coding
C-type lect	protein-coding
C-type lect	protein-coding
tectonic fa	protein-coding
thioesteras	protein-coding
leucine rich	protein-coding
pleckstrin h	protein-coding
mediator c	protein-coding
Abelson he	protein-coding
Ly1 antibo	protein-coding
annexin A1	protein-coding
apoptosis i	protein-coding
excision re	protein-coding
cytoskeleto	protein-coding
compleme	protein-coding
H2A histon	protein-coding
serine/argi	protein-coding
ArfGAP wit	protein-coding
family with	protein-coding
RIKEN cDN.	protein-coding
syntaxin bi	protein-coding
family with	protein-coding
TSC22 dom	protein-coding
SWI/SNF re	protein-coding
l(3)mbt-like	protein-coding
transducin	protein-coding
tumor necr	protein-coding
adaptor-re	protein-coding
N-deacetyl	protein-coding
RIKEN cDN.	protein-coding
forkhead b	protein-coding
CCR4-NOT	protein-coding
proteasom	protein-coding
SH2B adap	protein-coding
muscleblin	protein-coding
zinc finger	protein-coding
BH3 intera	protein-coding
spectrin re	protein-coding
abhydrolas	protein-coding

sorcin	protein-coding
PAN2 poly/	protein-coding
N(alpha)-ac	protein-coding
antigenic d	protein-coding
polypyrimi	protein-coding
guanylate c	protein-coding
fucosyltran	protein-coding
BMS1 hom	protein-coding
kinesin fam	protein-coding
Pbx/knotte	protein-coding
receptor-in	protein-coding
src homolo	protein-coding
taste recep	protein-coding
protein pho	protein-coding
zinc finger	protein-coding
RNA bindin	protein-coding
mannoside	protein-coding
histone clu	protein-coding
coiled-coil	protein-coding
ATP/GTP b	protein-coding
eukaryotic	protein-coding
DEAH (Asp-	protein-coding
epithelial n	protein-coding
germ cell-lo	protein-coding
insulin deg	protein-coding
ARP3 actin-	protein-coding
myeloblast	protein-coding
chemokine	protein-coding
UDP-N-ace	protein-coding
FYVE, RhoG	protein-coding
zinc finger,	protein-coding
FK506 bind	protein-coding
PHD finger	protein-coding
cAMP resp	protein-coding
eukaryotic	protein-coding
H2.0-like h	protein-coding
proteoglyca	protein-coding
WW domai	protein-coding
RIKEN cDN	protein-coding
solute carri	protein-coding
interleukin	protein-coding
aspartyl-tR	protein-coding
signaling ly	protein-coding
RIKEN cDN	protein-coding
platelet-ac	protein-coding
golgi integr	protein-coding
UDP-Gal:be	protein-coding

sestrin 2	protein-coding
nuclear res	protein-coding
suppressor	protein-coding
PWP1 hom	protein-coding
RIKEN cDN.	protein-coding
5-hydroxyt	protein-coding
neuroblast	protein-coding
transcriptic	protein-coding
sex comb c	protein-coding
CCAAT/enh	protein-coding
beta-site A	protein-coding
phosphatic	protein-coding
v-rel reticu	protein-coding
adhesion m	protein-coding
protein tyr	protein-coding
KH-type sp	protein-coding
nucleolar p	protein-coding
vitamin K e	protein-coding
cytochrom	protein-coding
histone clu	protein-coding
WW domai	protein-coding
jade family	protein-coding
ecdysonele	protein-coding
guanine nu	protein-coding
nuclear fac	protein-coding
7-dehydroc	protein-coding
A kinase (P	protein-coding
ribosomal	protein-coding
AAR2 splici	protein-coding
methylster	protein-coding
ring finger	protein-coding
cytoplasmi	protein-coding
tankyrase,	protein-coding
runt relate	protein-coding
furry homc	protein-coding
ankyrin rep	protein-coding
kinesin fam	protein-coding
calcium rel	protein-coding
diacylglyce	protein-coding
tet methylc	protein-coding
syntaxin 17	protein-coding
nuclear fac	protein-coding
oxidative s	protein-coding
adenosine	protein-coding
cullin 1	protein-coding
SKI family t	protein-coding
solute carri	protein-coding

predicted g	protein-coding
lactate deh	protein-coding
guanine de	protein-coding
shisa homc	protein-coding
secreted fr	protein-coding
pyridine nu	protein-coding
ribonuclea:	protein-coding
ADP-ribosy	protein-coding
E2F transcr	protein-coding
hairy/enha	protein-coding
myosin IF	protein-coding
tripartite m	protein-coding
cell cycle a:	protein-coding
proline deh	protein-coding
FBJ osteosa	protein-coding
solute carri	protein-coding
hexamethy	protein-coding
transmemb	protein-coding
PHD finger	protein-coding
lysine (K)-s	protein-coding
homeobox	protein-coding
engulfmen	protein-coding
PRP4 pre-n	protein-coding
RIKEN cDN.	protein-coding
poly(A) bin	protein-coding
early growt	protein-coding
deltex 1 ho	protein-coding
inositol 1,4	protein-coding
dynein, axc	protein-coding
mitochond	protein-coding
kelch-like 2	protein-coding
kelch-like 2	protein-coding
solute carri	protein-coding
TSSK6 activ	protein-coding
carbohydra	protein-coding
CWC27 spli	protein-coding
atonal hom	protein-coding
AF4/FMR2	protein-coding
nucleolar p	protein-coding
ER membra	protein-coding
polymerase	protein-coding
PDZ domai	protein-coding
chromodor	protein-coding
oxidative s	protein-coding
inositol 1,4	protein-coding
ATPase, Ca	protein-coding
transforma	protein-coding

zinc finger	protein-coding
regulator o	protein-coding
adaptor-re	protein-coding
ring finger	protein-coding
Fanconi an	protein-coding
sorting nex	protein-coding
envoplakin	protein-coding
family with	protein-coding
Kruppel-lik	protein-coding
olfactory re	protein-coding
Coenzyme	protein-coding
kinesin fan	protein-coding
predicted g	protein-coding
splicing fac	protein-coding
glutamate-	protein-coding
sterile alph	protein-coding
solute carri	protein-coding
Kruppel-lik	protein-coding
chondroad	protein-coding
ankyrin rep	protein-coding
glypican 1	protein-coding
kelch-like 3	protein-coding
SWA-70 pr	protein-coding
required fc	protein-coding
TAF5-like R	protein-coding
FERM, Rho	protein-coding
R3H domai	protein-coding
IZUMO fan	protein-coding
carbonic ar	protein-coding
ArfGAP wit	protein-coding
BCL2-assoc	protein-coding
cytochrom	protein-coding
cut-like hoi	protein-coding
vomeronas	protein-coding
regulator o	protein-coding
ATP syntha	protein-coding
mitochond	protein-coding
cAMP resp	protein-coding
cysteine-ric	protein-coding
polymeras	protein-coding
tubulin, alp	protein-coding
UBX domai	protein-coding
acyl-CoA sy	protein-coding
adducin 3 (	protein-coding
splicing fac	protein-coding
C1q and tu	protein-coding
ankyrin rep	protein-coding

coiled-coil	protein-coding
ATP-bindin	protein-coding
leucine-ricl	protein-coding
nephronop	protein-coding
solute carri	protein-coding
molybdenu	protein-coding
RIKEN cDN.	protein-coding
fyn-related	protein-coding
polynucleo	protein-coding
C-type lect	protein-coding
Rho GTPas	protein-coding
cathepsin k	protein-coding
acetyl-Coe	protein-coding
microtubul	protein-coding
baculoviral	protein-coding
peptidylprc	protein-coding
calmodulin	protein-coding
tribbles ho	protein-coding
solute carri	protein-coding
Der1-like d	protein-coding
transformi	protein-coding
DENN/MAI	protein-coding
versican	protein-coding
signal reco	protein-coding
Z-DNA binc	protein-coding
opioid grov	protein-coding
zinc finger	protein-coding
pyridoxal (|	protein-coding
ArfGAP wit	protein-coding
centrosom	protein-coding
protein kin	protein-coding
X-box bindi	protein-coding
TMEM9 do	protein-coding
bone morp	protein-coding
cytoplasmi	protein-coding
sorbin and	protein-coding
RELT tumo	protein-coding
cytokine in	protein-coding
sema doma	protein-coding
cyclin Pas1	protein-coding
Obg-like A	protein-coding
ras homolc	protein-coding
acidic (leuc	protein-coding
ubiquitin p	protein-coding
tripartite n	protein-coding
armadillo r	protein-coding
ribosomal	protein-coding

RIKEN cDN.	protein-coding
ectonucleo	protein-coding
E2F-associa	protein-coding
SPT2, Supp	protein-coding
low density	protein-coding
dual specif	protein-coding
asparagine	protein-coding
myelin bas	protein-coding
laccase (mu	protein-coding
proprotein	protein-coding
kinesin fam	protein-coding
SEC16 hom	protein-coding
tyrosyl-DN.	protein-coding
blocked ea	protein-coding
zinc finger	protein-coding
TNF recept	protein-coding
GPALPP mo	protein-coding
vesicle-asso	protein-coding
NUAK fami	protein-coding
isochorism	protein-coding
ribonuclea:	protein-coding
zinc finger,	protein-coding
bromodom	protein-coding
tubulin, alp	protein-coding
MAP kinase	protein-coding
lipoma HM	protein-coding
ATP-bindin	protein-coding
RIKEN cDN.	protein-coding
predicted g	protein-coding
coiled-coil	protein-coding
bicaudal D	protein-coding
methyl-CpG	protein-coding
junction ad	protein-coding
neural prec	protein-coding
eukaryotic	protein-coding
polymerase	protein-coding
ADP-ribosy	protein-coding
ORAI calciu	protein-coding
methyltran	protein-coding
RIKEN cDN.	protein-coding
saccharopi	protein-coding
eukaryotic	protein-coding
myelin pro	protein-coding
biogenesis	protein-coding
cyclin G ass	protein-coding
polymerase	protein-coding
reticulon 4	protein-coding

ring finger	protein-coding
transmemk	protein-coding
NOL1/NOP	protein-coding
RIKEN cDN.	protein-coding
SHQ1 hom	protein-coding
Ewing tum	protein-coding
collagen, t	protein-coding
chromodor	protein-coding
inositol 1,4	protein-coding
threonyl-tF	protein-coding
ubiquitin c	protein-coding
nuclear fac	protein-coding
regulator o	protein-coding
serine (or c	protein-coding
interleukin	protein-coding
Nipped-B h	protein-coding
splicing reg	protein-coding
proteasom	protein-coding
potassium	protein-coding
hairy and e	protein-coding
zinc finger,	protein-coding
protein ph	protein-coding
Shwachma	protein-coding
CDK5 regul	protein-coding
prosaposin	protein-coding
cell divisior	protein-coding
chymotryp	protein-coding
transmemk	protein-coding
coenzyme	protein-coding
serine/thre	protein-coding
proteasom	protein-coding
BEN domai	protein-coding
transcriptic	protein-coding
pleckstrin h	protein-coding
mitochond	protein-coding
sarcospan	protein-coding
poly(rC) bir	protein-coding
phosphatic	protein-coding
LON peptic	protein-coding
caspase 3	protein-coding
secreted pl	protein-coding
pecanex hc	protein-coding
mitogen-ac	protein-coding
colony stim	protein-coding
chondroitir	protein-coding
MACRO do	protein-coding
carbohydra	protein-coding

BCL2/aden	protein-coding
BCL2-like 1	protein-coding
secretogra	protein-coding
mitogen-ac	protein-coding
CCAAT/enh	protein-coding
ornithine d	protein-coding
WD repeat	protein-coding
COMM dor	protein-coding
membrane	protein-coding
dicer 1, rib	protein-coding
RAS, guany	protein-coding
mastermin	protein-coding
SCO cytoch	protein-coding
PR domain	protein-coding
serine pept	protein-coding
WD repeat	protein-coding
teneurin tr	protein-coding
transmemb	protein-coding
protein tyr	protein-coding
ring finger	protein-coding
family with	protein-coding
Tia1 cytoto	protein-coding
Rho-relate	protein-coding
doublecort	protein-coding
ATP-bindin	protein-coding
neutrophili	protein-coding
spinster ho	protein-coding
clathrin, he	protein-coding
insulin rece	protein-coding
alpha-N-ac	protein-coding
angiomotir	protein-coding
SH3-domai	protein-coding
ribosomal	protein-coding
TAO kinase	protein-coding
protein pho	protein-coding
ribonuclea	protein-coding
HOP home	protein-coding
organic sol	protein-coding
regulator o	protein-coding
Ras and Ra	protein-coding
BTB (POZ)	protein-coding
nucleolar p	protein-coding
excision re	protein-coding
immunity-r	protein-coding
amylo-1,6-	protein-coding
protein pho	protein-coding
ubiquitin-c	protein-coding

ubiquitin s|	protein-coding
phosphodi	protein-coding
serine (or c	protein-coding
Bcl2-like 1(	protein-coding
GATA zinc f	protein-coding
protease, s	protein-coding
membrane	protein-coding
MOB kinas	protein-coding
centromer	protein-coding
calcium/ca	protein-coding
uridine-cyt	protein-coding
uracil DNA	protein-coding
interleukin	protein-coding
scavenger r	protein-coding
leucine rich	protein-coding
pleckstrin a	protein-coding
UDP-Gal:be	protein-coding
syntaxin 11	protein-coding
insulin-like	protein-coding
placenta-sp	protein-coding
ret finger p	protein-coding
CUB and zo	protein-coding
TRAF3 inte	protein-coding
activating t	protein-coding
apoptosis e	protein-coding
synaptotag	protein-coding
RAD9 hom	protein-coding
sperm flag	protein-coding
kinesin fam	protein-coding
chemokine	protein-coding
tRNA meth	protein-coding
eukaryotic	protein-coding
UDP-Gal:be	protein-coding
ST8 alpha-I	protein-coding
GDNF-indu	protein-coding
von Willebr	protein-coding
DDB1 and (	protein-coding
thyroglobu	protein-coding
phosphatic	protein-coding
FIC domain	protein-coding
inositol pol	protein-coding
chloride int	protein-coding
mucin 13, e	protein-coding
mitochond	protein-coding
chromodor	protein-coding
interleukin	protein-coding
ST3 beta-g	protein-coding

sorting nex	protein-coding
cell divisior	protein-coding
vomeronas	protein-coding
ELAV (emb	protein-coding
H3 histone	protein-coding
plasminoge	protein-coding
low density	protein-coding
zinc finger	protein-coding
eukaryotic	protein-coding
predicted g	protein-coding
alpha thala	protein-coding
phospholip	protein-coding
Shc SH2-dc	protein-coding
acyl-Coenz	protein-coding
t-complex :	protein-coding
sorting nex	protein-coding
nuclear rec	protein-coding
nardilysin,	protein-coding
olfactory re	protein-coding
transmemb	protein-coding
crystallin, l	protein-coding
cystathiona	protein-coding
estrogen-re	protein-coding
leucine rich	protein-coding
Rho GTPase	protein-coding
heme oxyg	protein-coding
lysine (K)-s	protein-coding
valosin con	protein-coding
thrombosp	protein-coding
chloride ch	protein-coding
tetratricop	protein-coding
amyloid be	protein-coding
F-box prote	protein-coding
nuclear fac	protein-coding
endothelin	protein-coding
calmodulin	protein-coding
spastic para	protein-coding
calpain, sm	protein-coding
regulation	protein-coding
AT rich inte	protein-coding
sphingomy	protein-coding
WD repeat	protein-coding
neuron nav	protein-coding
ninein	protein-coding
predicted g	protein-coding
RIKEN cDNA	protein-coding
TGFB-induc	protein-coding

ribosomal (	protein-coding
valosin con	protein-coding
casein kina	protein-coding
anoctamin	protein-coding
prenyl (sola	protein-coding
myeloid ce	protein-coding
TNF recept	protein-coding
N-acetylglu	protein-coding
centromere	protein-coding
microtubul	protein-coding
zinc finger	protein-coding
glycerol ph	protein-coding
transketola	protein-coding
TBC1 doma	protein-coding
RAD21 hon	protein-coding
immunity-r	protein-coding
lactate deh	protein-coding
signal-regu	protein-coding
Ras-relatec	protein-coding
H2A histon	protein-coding
regulator o	protein-coding
palladin, cy	protein-coding
exosome c	protein-coding
TBC1 doma	protein-coding
lipase, end	protein-coding
G protein-c	protein-coding
suppressor	protein-coding
lysine (K)-s	protein-coding
interferon	protein-coding
haptoglobi	protein-coding
NADH dehy	protein-coding
RAB14, me	protein-coding
solute carri	protein-coding
RIKEN cDN,	protein-coding
calsyntenir	protein-coding
RIKEN cDN,	protein-coding
NOP56 ribc	protein-coding
DEP domai	protein-coding
AT rich inte	protein-coding
BAH domai	protein-coding
RAB5C, me	protein-coding
RIKEN cDN,	protein-coding
caspase 8	protein-coding
intraflagell	protein-coding
dihydrolipc	protein-coding
purinergic	protein-coding
SWI/SNF re	protein-coding

F-box and \	protein-coding
phosphoin	protein-coding
zinc finger	protein-coding
biorientati	protein-coding
RAN bindin	protein-coding
tripeptidyl	protein-coding
ribosomal	protein-coding
aryl hydroc	protein-coding
gamma-sec	protein-coding
arginine/se	protein-coding
lysosomal-	protein-coding
tripartite m	protein-coding
zinc finger	protein-coding
neutral sph	protein-coding
UDP-Gal:be	protein-coding
procollager	protein-coding
neurogenir	protein-coding
serglycin	protein-coding
SMAD fami	protein-coding
F-box prote	protein-coding
maestro he	protein-coding
cadherin 1:	protein-coding
calcium/ca	protein-coding
chemokine	protein-coding
methyltran	protein-coding
prostaglan	protein-coding
GID comple	protein-coding
leucine rich	protein-coding
family with	protein-coding
ribosomal	protein-coding
WW domai	protein-coding
nuclear rec	protein-coding
Max protei	protein-coding
furin (paire	protein-coding
ribosomal	protein-coding
heterogene	protein-coding
glucose-fru	protein-coding
ubiquitin p	protein-coding
Rho guanir	protein-coding
U7 snRNP-:	protein-coding
zinc finger,	protein-coding
transmemb	protein-coding
RAS relatec	protein-coding
genetic sur	protein-coding
family with	protein-coding
schwannor	protein-coding
DENN/MAI	protein-coding

transport a	protein-coding
pre B cell le	protein-coding
branched c	protein-coding
interleukin	protein-coding
transcriptio	protein-coding
protein dis	protein-coding
zinc finger	protein-coding
methyl-CpG	protein-coding
solute carri	protein-coding
fibroblast g	protein-coding
tetratricop	protein-coding
docking pro	protein-coding
capping pro	protein-coding
large tumo	protein-coding
ubiquitin-a	protein-coding
stromal an	protein-coding
oxysterol b	protein-coding
apoptosis i	protein-coding
ELOVL fam	protein-coding
ubiquitin sp	protein-coding
olfactory re	protein-coding
interleukin	protein-coding
leucine zipp	protein-coding
sema doma	protein-coding
leucine zipp	protein-coding
nuclear rec	protein-coding
forkhead b	protein-coding
mindbomb	protein-coding
poly(A) bin	protein-coding
solute carri	protein-coding
carbohydra	protein-coding
structural r	protein-coding
nuclear tra	protein-coding
colony stim	protein-coding
small integ	protein-coding
casein kina	protein-coding
Ras homolo	protein-coding
trans-acting	protein-coding
transforme	protein-coding
signal trans	protein-coding
ecotropic v	protein-coding
coiled-coil-	protein-coding
suppressor	protein-coding
ataxin 10	protein-coding
ladybird ho	protein-coding
elastin mic	protein-coding
early endos	protein-coding

neuropilin	protein-coding
BTB and CN	protein-coding
ras respons	protein-coding
GTP-bindin	protein-coding
G protein p	protein-coding
SHQ1 hom	protein-coding
SET domair	protein-coding
Rho guanir	protein-coding
arachidona	protein-coding
actin, beta	protein-coding
stathmin 1	protein-coding
interleukin	protein-coding
neuralized-	protein-coding
RAS p21 pr	protein-coding
SWI/SNF re	protein-coding
leucine rich	protein-coding
SUMO1/se	protein-coding
pyruvate ki	protein-coding
protein tyr	protein-coding
methylmal	protein-coding
phospholip	protein-coding
potassium	protein-coding
SAM doma	protein-coding
MMS22-lik	protein-coding
WD repeat	protein-coding
elongation	protein-coding
seven in ab	protein-coding
jun B proto	protein-coding
left-right d	protein-coding
glutamyl-tF	protein-coding
HIV TAT sp	protein-coding
Fgfr1op N-	protein-coding
transcriptio	protein-coding
homeobox	protein-coding
ribosomal	protein-coding
triple funct	protein-coding
DDHD dom	protein-coding
retinoblast	protein-coding
extended s	protein-coding
estrogen-re	protein-coding
dihydrolipo	protein-coding
TRAF2 and	protein-coding
1-acylglyce	protein-coding
NUAK fami	protein-coding
activin A re	protein-coding
RAB21, me	protein-coding
integral me	protein-coding

trinucleotic	protein-coding
MAF1 hom	protein-coding
START dom	protein-coding
regulatory	protein-coding
activating t	protein-coding
ceramide k	protein-coding
ubiquitin s|	protein-coding
v-raf-leuke	protein-coding
mitogen-ac	protein-coding
CCR4-NOT	protein-coding
family with	protein-coding
transmemb	protein-coding
CD44 antig	protein-coding
lipoma HM	protein-coding
ADP-deper	protein-coding
forkhead b	protein-coding
gonadotro|	protein-coding
solute carri	protein-coding
acyl-CoA th	protein-coding
nitrilase 1	protein-coding
poliovirus r	protein-coding
ankyrin rep	protein-coding
leucine rich	protein-coding
phospholip	protein-coding
gametoger	protein-coding
methionine	protein-coding
paraneopla	protein-coding
syntrophin	protein-coding
pyrophosp	protein-coding
coatomer p	protein-coding
anaphase p	protein-coding
eukaryotic	protein-coding
mitochond	protein-coding
MAP kinase	protein-coding
RAB14, me	protein-coding
fibrillin 1	protein-coding
low density	protein-coding
chemokine	protein-coding
peptidyl-pr	protein-coding
phosphoin	protein-coding
POC1 centr	protein-coding
zinc finger	protein-coding
Rho GTPase	protein-coding
RIKEN cDN.	protein-coding
annexin A2	protein-coding
family with	protein-coding
acyl-CoA sy	protein-coding

calcium/ca	protein-coding
tektin 1	protein-coding
TM2 doma	protein-coding
kelch-like 6	protein-coding
retinol deh	protein-coding
t-complex	protein-coding
A kinase (P	protein-coding
integrin alp	protein-coding
RIKEN cDN	protein-coding
phosphatic	protein-coding
internexin	protein-coding
CDC like kin	protein-coding
chemokine	protein-coding
RIKEN cDN	protein-coding
cytochrom	protein-coding
carboxypep	protein-coding
diacylglyce	protein-coding
vascular en	protein-coding
ATPase, Na	protein-coding
homeobox	protein-coding
protein pho	protein-coding
ankyrin rep	protein-coding
ribosomal	protein-coding
zinc finger	protein-coding
RIKEN cDN	protein-coding
immediate	protein-coding
hemopoiet	protein-coding
zinc finger,	protein-coding
suppressor	protein-coding
glutamate-	protein-coding
growth diff	protein-coding
phospholip	protein-coding
zinc finger	protein-coding
influenza v	protein-coding
RIKEN cDN	protein-coding
poly (ADP-r	protein-coding
WAP four-c	protein-coding
icos ligand	protein-coding
family with	protein-coding
adaptor-re	protein-coding
neuroblast	protein-coding
dickkopf ho	protein-coding
SUMO/sen	protein-coding
E1A bindin	protein-coding
BMP2 indu	protein-coding
ring finger	protein-coding
processing	protein-coding

early growt	protein-coding
GA repeat	protein-coding
proline-rich	protein-coding
5-methylte	protein-coding
XRCC6 binc	protein-coding
upstream b	protein-coding
phospholip	protein-coding
STE20-like	protein-coding
eukaryotic	protein-coding
PTK2 prote	protein-coding
cDNA sequ	protein-coding
mitochond	protein-coding
ankyrin rep	protein-coding
actin relate	protein-coding
astrotactin	protein-coding
galactosida	protein-coding
layilin	protein-coding
adenylate c	protein-coding
RAB37, me	protein-coding
AT rich inte	protein-coding
regulating s	protein-coding
ligase III, D	protein-coding
special AT-	protein-coding
small cell a	protein-coding
chemokine	protein-coding
intercellula	protein-coding
PHD and ri	protein-coding
protein inh	protein-coding
hepatocyte	protein-coding
glyceraldeh	protein-coding
methyltran	protein-coding
influenza v	protein-coding
A kinase (P	protein-coding
GTP bindin	protein-coding
fer (fms/fp	protein-coding
translin-ass	protein-coding
ribosomal p	protein-coding
syntaxin 4A	protein-coding
midasin ho	protein-coding
immediate	protein-coding
carbonic ar	protein-coding
glutathione	protein-coding
consortin, c	protein-coding
cyclin J	protein-coding
FK506 bind	protein-coding
IscU iron-su	protein-coding
guanine nu	protein-coding

transmemb	protein-coding
D-tyrosyl-tl	protein-coding
aryl-hydroc	protein-coding
vestigial lik	protein-coding
Y box prote	protein-coding
MAP-kinase	protein-coding
family with	protein-coding
coiled-coil	protein-coding
rhomboid c	protein-coding
zinc finger	protein-coding
Kruppel-lik	protein-coding
protein tyr	protein-coding
methyl-CpC	protein-coding
RIKEN cDN.	protein-coding
BCL6 intera	protein-coding
claspin	protein-coding
GATA bindi	protein-coding
proline rich	protein-coding
PH domain	protein-coding
presenilin 1	protein-coding
ras respons	protein-coding
AT-hook tra	protein-coding
coiled-coil	protein-coding
transmemb	protein-coding
crystallin, g	protein-coding
Ral GTPase	protein-coding
cell divisior	protein-coding
thioredoxir	protein-coding
hect (homo	protein-coding
craniofacia	protein-coding
protein tyr	protein-coding
protein-O-r	protein-coding
Scm-like wi	protein-coding
piezo-type	protein-coding
excision re	protein-coding
tetratricop	protein-coding
ATPase, Ca	protein-coding
E2F transcr	protein-coding
histidine ar	protein-coding
major facili	protein-coding
glucose-fru	protein-coding
cDNA sequ	protein-coding
enoyl Coen	protein-coding
outer dens	protein-coding
Hedgehog-	protein-coding
RIKEN cDN.	protein-coding
transforma	protein-coding

POZ (BTB) a	protein-coding
Rho family	protein-coding
exocyst co	protein-coding
mitochond	protein-coding
elongation	protein-coding
TRM2 tRNA	protein-coding
eukaryotic	protein-coding
phosphodi	protein-coding
ribosomal	protein-coding
pleiomorpl	protein-coding
fibronectin	protein-coding
potassium	protein-coding
mesoderm	protein-coding
glycogen s	protein-coding
Kruppel-lik	protein-coding
plasminoge	protein-coding
predicted g	protein-coding
solute carri	protein-coding
SMAD fami	protein-coding
talin 1	protein-coding
integrin alp	protein-coding
microsoma	protein-coding
dachshund	protein-coding
solute carri	protein-coding
methylthio	protein-coding
transmemb	protein-coding
RIKEN cDNA	protein-coding
SUZ RNA bi	protein-coding
ribonucleas	protein-coding
ATPase, Ca	protein-coding
plexin C1	protein-coding
nucleolar p	protein-coding
reticulocall	protein-coding
tribbles ho	protein-coding
RIKEN cDNA	protein-coding
protein-O-r	protein-coding
GTPase act	protein-coding
RIKEN cDNA	protein-coding
G protein-c	protein-coding
p21 protein	protein-coding
complemer	protein-coding
Moloney le	protein-coding
RIKEN cDNA	protein-coding
branched c	protein-coding
Mdm2, tran	protein-coding
B cell trans	protein-coding
interleukin	protein-coding

COP9 (cons	protein-coding
Hus1 homo	protein-coding
RIKEN cDN.	protein-coding
paired box	protein-coding
GRAM dom	protein-coding
sine oculis-	protein-coding
TWIST neig	protein-coding
orofacial cl	protein-coding
serine/argi	protein-coding
mitogen-ac	protein-coding
SWI/SNF re	protein-coding
family with	protein-coding
MAD2 mitc	protein-coding
guanidinoa	protein-coding
leucine rich	protein-coding
cDNA sequ	protein-coding
cystatin 10	protein-coding
exportin 7	protein-coding
alpha fetop	protein-coding
Sin3-associ	protein-coding
arginine/se	protein-coding
TBC1 doma	protein-coding
myotubula	protein-coding
C-type lect	protein-coding
transformi	protein-coding
ariadne ub	protein-coding
G-rich RNA	protein-coding
interferon	protein-coding
small ArfG/	protein-coding
membrane	protein-coding
peripheral	protein-coding
trinucleotic	protein-coding
eukaryotic	protein-coding
insulin-like	protein-coding
family with	protein-coding
ER membra	protein-coding
musculin	protein-coding
discs, large	protein-coding
glutamate	protein-coding
microsoma	protein-coding
lysosomal t	protein-coding
grancalcin	protein-coding
leucine rich	protein-coding
ATP/GTP b	protein-coding
CREB/ATF b	protein-coding
PR domain	protein-coding
transforme	protein-coding

suppressor	protein-coding
calpain 9	protein-coding
PTK2 prote	protein-coding
Pbx/knotte	protein-coding
cytochrom	protein-coding
methyltran	protein-coding
ribosomal	protein-coding
hydroxyste	protein-coding
mediator c	protein-coding
RIKEN cDN	protein-coding
CD33 antig	protein-coding
sestrin 2	protein-coding
protocadhe	protein-coding
ADP-ribosy	protein-coding
phosphata	protein-coding
a disintegri	protein-coding
transmemb	protein-coding
uridine mo	protein-coding
glucosamir	protein-coding
vacuolar pr	protein-coding
ubiquitin s	protein-coding
zinc finger	protein-coding
heterogen	protein-coding
mitogen-ac	protein-coding
sorting nex	protein-coding
coiled-coil-	protein-coding
RNA bindin	protein-coding
inositol mo	protein-coding
ets variant	protein-coding
zinc finger	protein-coding
late endos	protein-coding
maternal e	protein-coding
UDP-N-ace	protein-coding
karyopheri	protein-coding
UDP-GlcNA	protein-coding
D4, zinc an	protein-coding
pleckstrin	protein-coding
coiled-coil	protein-coding
calcium an	protein-coding
solute carri	protein-coding
NUF2, NDC	protein-coding
tumor prot	protein-coding
transducin	protein-coding
ubiquitinat	protein-coding
coagulatior	protein-coding
B cell CLL/l	protein-coding
suppressor	protein-coding

prohibitin	protein-coding
bleomycin	protein-coding
DCP2 deca|	protein-coding
RIKEN cDN.	protein-coding
zinc finger	protein-coding
Bloom sync	protein-coding
LIM domai	protein-coding
frizzled hor	protein-coding
ATP-bindin	protein-coding
rogdi homc	protein-coding
spermatog	protein-coding
translocase	protein-coding
tumor necr	protein-coding
transgelin 2	protein-coding
shugoshin-	protein-coding
guanine nu	protein-coding
dolichol-ph	protein-coding
transmemb	protein-coding
mediator c	protein-coding
denticleles	protein-coding
proline-ser	protein-coding
leukocyte r	protein-coding
peroxisoma	protein-coding
CUE domai	protein-coding
ets variant	protein-coding
zinc finger	protein-coding
cell divisior	protein-coding
STAM bind	protein-coding
UDP-Gal:be	protein-coding
sterile alph	protein-coding
pellino 2	protein-coding
cytochrom	protein-coding
antagonist	protein-coding
monooxyge	protein-coding
minichrom	protein-coding
charged mu	protein-coding
SPARC rela	protein-coding
ADP-ribosy	protein-coding
glutaminas	protein-coding
mitochond	protein-coding
lactase	protein-coding
SAC1 (supp	protein-coding
ribosomal		protein-coding
transforma	protein-coding
protein tyr	protein-coding
KH domain	protein-coding
glucuronid	protein-coding

RIKEN cDN.	protein-coding
CD300A an	protein-coding
IQ motif ar	protein-coding
COMM dor	protein-coding
pumilio RN	protein-coding
family with	protein-coding
procollagen	protein-coding
dual specif	protein-coding
lysine (K)-s	protein-coding
FBJ osteosa	protein-coding
transmemb	protein-coding
sortilin 1	protein-coding
activating t	protein-coding
heterogene	protein-coding
sorting nex	protein-coding
a disintegri	protein-coding
diacylglyce	protein-coding
gelsolin	protein-coding
SMAD spec	protein-coding
tubulin tyr	protein-coding
septin 5	protein-coding
ataxin 1-lik	protein-coding
adiponecti	protein-coding
solute carri	protein-coding
growth hor	protein-coding
zinc finger	protein-coding
rhomboid 5	protein-coding
androglobi	protein-coding
sorting nex	protein-coding
myosin, he	protein-coding
leucine-ricl	protein-coding
methylene	protein-coding
human imr	protein-coding
synuclein, a	protein-coding
Rab gerany	protein-coding
tumor supp	protein-coding
profilin 1	protein-coding
muscleblin	protein-coding
demethyl-(	protein-coding
prothymos	protein-coding
HtrA serine	protein-coding
rhomboid (	protein-coding
phenylalan	protein-coding
thymocyte	protein-coding
upstream t	protein-coding
mannoside	protein-coding
spermatog	protein-coding

La ribonucl	protein-coding
adaptor pr	protein-coding
ankyrin rep	protein-coding
zinc finger	protein-coding
tripartite m	protein-coding
protein kin	protein-coding
praja 2, RIN	protein-coding
histone clu	protein-coding
tripartite m	protein-coding
FAT tumor	protein-coding
general tra	protein-coding
hect domai	protein-coding
mucosa ass	protein-coding
BCL2-like 1	protein-coding
transcriptic	protein-coding
retinoid X r	protein-coding
sterol-C5-d	protein-coding
serine thre	protein-coding
B cell CLL/l	protein-coding
transmemb	protein-coding
RIKEN cDN.	protein-coding
YOD1 OTU	protein-coding
SAFB-like, t	protein-coding
pecanex hc	protein-coding
N-acetyltra	protein-coding
thrombopc	protein-coding
thioredoxir	protein-coding
neuritin 1	protein-coding
DnaJ (Hsp4	protein-coding
calcyphosp	protein-coding
family with	protein-coding
latent trans	protein-coding
YY1 associa	protein-coding
ankyrin rep	protein-coding
sema doma	protein-coding
fibronectin	protein-coding
thioredoxir	protein-coding
cyclin D3	protein-coding
angel homc	protein-coding
Y box prote	protein-coding
leucine rich	protein-coding
dedicator c	protein-coding
S-phase kir	protein-coding
serine/thre	protein-coding
adenylosuc	protein-coding
programm	protein-coding
forkhead b	protein-coding

eukaryotic	protein-coding
fukutin	protein-coding
UDP-N-ace	protein-coding
transcriptic	protein-coding
expressed :	protein-coding
ubiquitin-c	protein-coding
solute carri	protein-coding
FYVE, RhoC	protein-coding
UDP-N-ace	protein-coding
kelch-like 1	protein-coding
aryl-hydroc	protein-coding
XRCC6 binc	protein-coding
C-type lect	protein-coding
olfactomec	protein-coding
OTU domai	protein-coding
ST3 beta-ga	protein-coding
ankyrin rep	protein-coding
adenosine	protein-coding
DNA segme	protein-coding
small nucle	protein-coding
protein-O-r	protein-coding
acid-sensin	protein-coding
zinc finger	protein-coding
citron	protein-coding
multiple in	protein-coding
serine inco	protein-coding
peroxisoma	protein-coding
leucine rich	protein-coding
phosphoglu	protein-coding
dymeclin	protein-coding
receptor tr	protein-coding
SR-related	protein-coding
JAZF zinc fi	protein-coding
protein tyr	protein-coding
phosphatic	protein-coding
amino-term	protein-coding
CDC-like ki	protein-coding
zinc finger	protein-coding
GPI anchor	protein-coding
zinc finger	protein-coding
prostaglan	protein-coding
presenilin 2	protein-coding
3-hydroxy-	protein-coding
DnaJ (Hsp4	protein-coding
bromodom	protein-coding
predicted g	protein-coding
family with	protein-coding

ATPase, Ca	protein-coding
golgi reasse	protein-coding
tight juncti	protein-coding
ATP-bindin	protein-coding
YEATS dom	protein-coding
transcriptic	protein-coding
general tra	protein-coding
transforma	protein-coding
pleckstrin h	protein-coding
squamous	protein-coding
StAR-relate	protein-coding
FK506 bind	protein-coding
heat shock	protein-coding
Nedd4 fam	protein-coding
ATP-bindin	protein-coding
zinc finger	protein-coding
egl-9 family	protein-coding
vacuolar pr	protein-coding
proteasom	protein-coding
glutaredox	protein-coding
Jun dimeriz	protein-coding
CDC14 cell	protein-coding
ceramide s	protein-coding
NEDD4 bin	protein-coding
filamin A ir	protein-coding
enhancer c	protein-coding
furry homc	protein-coding
arrestin do	protein-coding
FAST kinas	protein-coding
glutamate	protein-coding
calsyntenir	protein-coding
reticuloenc	protein-coding
RAB33B, m	protein-coding
signal trans	protein-coding
transmemb	protein-coding
potassium	protein-coding
vav 3 onco	protein-coding
cyclin-depe	protein-coding
ATP-bindin	protein-coding
folliculin in	protein-coding
archaelysir	protein-coding
growth fac	protein-coding
glutamate	protein-coding
dedicator c	protein-coding
transmemb	protein-coding
poly (ADP-r	protein-coding
sema doma	protein-coding

ELKS/RAB6	protein-coding
solute carri	protein-coding
sorting nex	protein-coding
serpine1 m	protein-coding
human imr	protein-coding
adducin 1 (	protein-coding
BCL2-like 1	protein-coding
methionine	protein-coding
prostaglan	protein-coding
nuclear tra	protein-coding
nicotinami	protein-coding
toll-like rec	protein-coding
DAZ intera	protein-coding
tocopherol	protein-coding
mediator c	protein-coding
cytochrom	protein-coding
transformi	protein-coding
protein kin	protein-coding
MAP/micro	protein-coding
spleen foci	protein-coding
metaxin 2	protein-coding
TANK-bindi	protein-coding
transmemb	protein-coding
protein tyr	protein-coding
AT rich inte	protein-coding
spectrin re	protein-coding
thrombosp	protein-coding
bone marro	protein-coding
FUN14 don	protein-coding
dual specif	protein-coding
solute carri	protein-coding
thyroid stir	protein-coding
myeloblast	protein-coding
thiamine p	protein-coding
protein kin	protein-coding
oxytocin re	protein-coding
deformed e	protein-coding
AF4/FMR2	protein-coding
solute carri	protein-coding
eukaryotic	protein-coding
5,10-methy	protein-coding
phosphatic	protein-coding
neutral spł	protein-coding
myosin IE	protein-coding
cyclin D1	protein-coding
mitochond	protein-coding
fibroblast g	protein-coding

phospholip	protein-coding
DEAD (Asp-	protein-coding
ubiquitin-li	protein-coding
immediate	protein-coding
zinc finger	protein-coding
Gardner-Ra	protein-coding
runt relate	protein-coding
INO80 hom	protein-coding
sortilin-rela	protein-coding
mesoderm	protein-coding
SH3 domai	protein-coding
crystallin, g	protein-coding
SRY (sex de	protein-coding
reactive ox	protein-coding
lactamase,	protein-coding
mitochond	protein-coding
signal trans	protein-coding
transmemb	protein-coding
choline/eth	protein-coding
diacylglyce	protein-coding
aspartic pe	protein-coding
DNA cross-	protein-coding
CXADR-like	protein-coding
U1 small nu	protein-coding
Tp53rk bin	protein-coding
basic helix-	protein-coding
cytochrom	protein-coding
suppressor	protein-coding
dapper hor	protein-coding
arginyltran	protein-coding
Fanconi an	protein-coding
CUB and Su	protein-coding
NUAK fami	protein-coding
sideroflexin	protein-coding
actin relate	protein-coding
RB1-induci	protein-coding
solute carri	protein-coding
ATPase, cla	protein-coding
serrate RN/	protein-coding
NLR family,	protein-coding
golgi autoa	protein-coding
fibroblast g	protein-coding
succinate-C	protein-coding
Rho GTPase	protein-coding
solute carri	protein-coding
androglobi	protein-coding
carboxylest	protein-coding

RIKEN cDN.	protein-coding
zinc finger	protein-coding
progestero	protein-coding
solute carri	protein-coding
aldehyde d	protein-coding
interferon	protein-coding
testis expre	protein-coding
Fyn proto-c	protein-coding
RIKEN cDN.	protein-coding
mast cell e:	protein-coding
cyclin B1 in	protein-coding
jerky	protein-coding
zinc finger	protein-coding
F-box prote	protein-coding
phosphoty	protein-coding
phosphofru	protein-coding
nucleus acc	protein-coding
PHD finger	protein-coding
killer cell le	protein-coding
bromodom	protein-coding
retinoic aci	protein-coding
SH3 domai	protein-coding
phospholip	protein-coding
START dom	protein-coding
upstream k	protein-coding
vestigial lik	protein-coding
heterogene	protein-coding
tumor-supp	protein-coding
RRP9, smal	protein-coding
caveolin 2	protein-coding
zinc finger	protein-coding
down-regu	protein-coding
phosphodi	protein-coding
gelsolin	protein-coding
deformed e	protein-coding
S100 calciu	protein-coding
PR domain	protein-coding
frizzled hor	protein-coding
chemokine	protein-coding
Williams Be	protein-coding
F-box and \	protein-coding
MyoD fami	protein-coding
inositol pol	protein-coding
aldehyde d	protein-coding
echinoderr	protein-coding
RIKEN cDN.	protein-coding
phosphatic	protein-coding

sorting nex	protein-coding
ribosomal l	protein-coding
solute carri	protein-coding
CD97 antig	protein-coding
Y box prote	protein-coding
phosphatic	protein-coding
E74-like fac	protein-coding
UDP-glucos	protein-coding
Nedd4 fam	protein-coding
formin-like	protein-coding
MAP3K12 l	protein-coding
ELM2 and I	protein-coding
SNF relatec	protein-coding
eukaryotic	protein-coding
hexokinase	protein-coding
RAD23b hc	protein-coding
ubiquitin-c	protein-coding
glycogenin	protein-coding
astrotactin	protein-coding
guanine nu	protein-coding
mitochond	protein-coding
pleckstrin l	protein-coding
SPEN homc	protein-coding
protein kin	protein-coding
N-myc dow	protein-coding
lectin, gala	protein-coding
plectin	protein-coding
calpain 2	protein-coding
membrane	protein-coding
TSC22 dom	protein-coding
protein tyr	protein-coding
otogelin-lik	protein-coding
cDNA sequ	protein-coding
Fanconi an	protein-coding
male enhai	protein-coding
RIKEN cDN	protein-coding
adenylate l	protein-coding
solute carri	protein-coding
anterior gr	protein-coding
RCSD doma	protein-coding
ArfGAP wit	protein-coding
COMM dor	protein-coding
cullin 5	protein-coding
GTP bindin	protein-coding
protein phc	protein-coding
vesicle ami	protein-coding
RCSD doma	protein-coding

regulator o	protein-coding
solute carri	protein-coding
B cell trans	protein-coding
mannosida	protein-coding
mediator c	protein-coding
influenza v	protein-coding
zinc finger,	protein-coding
ras homolo	protein-coding
galanin	protein-coding
RIKEN cDN.	protein-coding
RasGEF doı	protein-coding
tribbles ho	protein-coding
wingless-ty	protein-coding
ferric-chela	protein-coding
Moloney sa	protein-coding
kinesin fam	protein-coding
carbamoyl-	protein-coding
vacuolar pı	protein-coding
guanine nu	protein-coding
glutamate	protein-coding
G patch do	protein-coding
histone dea	protein-coding
scaffolding	protein-coding
spleen tyro	protein-coding
interleukin	protein-coding
PHD finger	protein-coding
HERV-H LTI	protein-coding
beaded fila	protein-coding
bolA-like 3	protein-coding
peroxisoma	protein-coding
solute carri	protein-coding
LIM and se	protein-coding
diphosphoı	protein-coding
crystallin, a	protein-coding
proteasom	protein-coding
HAUS augn	protein-coding
bromodom	protein-coding
heparan su	protein-coding
chromodoı	protein-coding
radial spok	protein-coding
SCY1-like 3	protein-coding
regulatory	protein-coding
vesicle-ass	protein-coding
DENN/MAI	protein-coding
ATP-bindin	protein-coding
NADH dehy	protein-coding
SAYSVFN m	protein-coding

bone morp	protein-coding
nucleotide	protein-coding
copine VIII	protein-coding
vacuolar pr	protein-coding
DEAD (Asp-	protein-coding
kelch-like 2	protein-coding
kelch-like 9	protein-coding
amyloid be	protein-coding
heat shock	protein-coding
apoptosis,	protein-coding
cyclin-depe	protein-coding
TRIO and F	protein-coding
nuclear rec	protein-coding
multivesicu	protein-coding
chromodor	protein-coding
cadherin 1	protein-coding
thymus, br	protein-coding
eukaryotic	protein-coding
nascent po	protein-coding
K(lysine) ac	protein-coding
optineurin	protein-coding
T cell activa	protein-coding
RIKEN cDN	protein-coding
ER lipid raf	protein-coding
discoidin, C	protein-coding
NEDD4 bin	protein-coding
Luc7 homo	protein-coding
homocyste	protein-coding
mitochond	protein-coding
proteasom	protein-coding
arginyl-tRN	protein-coding
cell divisior	protein-coding
receptor ac	protein-coding
CD93 antig	protein-coding
Nfat activa	protein-coding
eukaryotic	protein-coding
KAT8 regul	protein-coding
family with	protein-coding
LPS-respon	protein-coding
Abelson he	protein-coding
growth arr	protein-coding
solute carri	protein-coding
RIKEN cDN	protein-coding
discoidin d	protein-coding
protease, s	protein-coding
cyclin-depe	protein-coding
thrombopc	protein-coding

NLR family	protein-coding
exosome c	protein-coding
SET domai	protein-coding
TAF7 RNA	protein-coding
RIKEN cDN	protein-coding
tet methyl	protein-coding
dynein, axo	protein-coding
glutamate	protein-coding
family with	protein-coding
inhibin bet	protein-coding
reticulon 3	protein-coding
RIKEN cDN	protein-coding
ninjurin 2	protein-coding
ribosomal	protein-coding
ELK3, mem	protein-coding
B cell trans	protein-coding
neurolysin	protein-coding
smu-1 supp	protein-coding
KDEL (Lys-A	protein-coding
polymerase	protein-coding
BTB and CN	protein-coding
forkhead b	protein-coding
5-hydroxyt	protein-coding
eukaryotic	protein-coding
eukaryotic	protein-coding
phosphodie	protein-coding
ATPase, H+	protein-coding
Fanconi an	protein-coding
catenin (ca	protein-coding
polynucleo	protein-coding
arginine an	protein-coding
lymphocyte	protein-coding
scrapie res	protein-coding
inhibitor of	protein-coding
AE binding	protein-coding
KDM1 lysin	protein-coding
ring finger	protein-coding
PRP6 pre-n	protein-coding
solute carri	protein-coding
peroxisome	protein-coding
collagen, ty	protein-coding
lipase, hep	protein-coding
enoyl Coen	protein-coding
muscle, ske	protein-coding
keratinocyt	protein-coding
expressed s	protein-coding
calcium rel	protein-coding

syncytin b	protein-coding
diphthamir	protein-coding
proteasom	protein-coding
Mab-21 do	protein-coding
solute carri	protein-coding
actin-like 7	protein-coding
ADP-ribosy	protein-coding
CCR4-NOT	protein-coding
REST corep	protein-coding
ATP syntha	protein-coding
low density	protein-coding
paired rela	protein-coding
CCAAT/enh	protein-coding
interferon	protein-coding
mex3 hom	protein-coding
transforme	protein-coding
tRNA meth	protein-coding
Rho GTPas	protein-coding
serine (or c	protein-coding
AU RNA bir	protein-coding
vomeronas	protein-coding
selectin, ly	protein-coding
diacylglyce	protein-coding
notch 2	protein-coding
UDP-N-ace	protein-coding
inositol pol	protein-coding
resistin like	protein-coding
zinc fingers	protein-coding
muscleblin	protein-coding
polycystic k	protein-coding
programm	protein-coding
selenophos	protein-coding
mitochond	protein-coding
ring finger	protein-coding
RIKEN cDN	protein-coding
thymus cel	protein-coding
Wiskott-Al	protein-coding
nucleolar p	protein-coding
ubiquitin sp	protein-coding
synaptone	protein-coding
splA/ryano	protein-coding
inositol 1,4	protein-coding
myosin, ligl	protein-coding
CDC42 effe	protein-coding
caldesmon	protein-coding
phosphoglu	protein-coding
acyl-CoA sy	protein-coding

nuclear ant	protein-coding
tribbles ho	protein-coding
proteasom	protein-coding
RAN, meml	protein-coding
solute carri	protein-coding
NADH dehy	protein-coding
killer cell le	protein-coding
src family a	protein-coding
cytochrom	protein-coding
feminizatic	protein-coding
proline rich	protein-coding
SH3 and PX	protein-coding
scribbled h	protein-coding
ubiquitin-c	protein-coding
transmemk	protein-coding
zinc finger	protein-coding
FCH domai	protein-coding
exocyst coi	protein-coding
headcase h	protein-coding
RAB11A, m	protein-coding
GINS comp	protein-coding
pleckstrin I	protein-coding
F-box and \	protein-coding
EH domain	protein-coding
diphthamir	protein-coding
coiled-coil	protein-coding
prothymos	protein-coding
eukaryotic	protein-coding
SET nuclea	protein-coding
basic leucir	protein-coding
CD44 antig	protein-coding
hematopoi	protein-coding
F-box and l	protein-coding
estrogen-re	protein-coding
CUB and Su	protein-coding
TATA box b	protein-coding
protease, s	protein-coding
methyltran	protein-coding
monoacylg	protein-coding
interleukin	protein-coding
jade family	protein-coding
steroid 5 al	protein-coding
forkhead b	protein-coding
golgi to ER	protein-coding
solute carri	protein-coding
CXXC finge	protein-coding
spectrin alp	protein-coding

vaccinia rel	protein-coding
enolase 4	protein-coding
origin reco	protein-coding
ubiquinol-c	protein-coding
HMG box d	protein-coding
interleukin	protein-coding
xyloside xy	protein-coding
elongator a	protein-coding
TBC1 doma	protein-coding
ribosomal p	protein-coding
WD repeat	protein-coding
breakpoint	protein-coding
mesoderm	protein-coding
RIKEN cDN	protein-coding
ADP-ribosy	protein-coding
magnesium	protein-coding
ribosomal p	protein-coding
dual serine	protein-coding
LEM domai	protein-coding
ubiquitin sp	protein-coding
nicotinamid	protein-coding
solute carri	protein-coding
X-prolyl am	protein-coding
calcium cha	protein-coding
24-dehydro	protein-coding
mitogen-ac	protein-coding
AT hook co	protein-coding
E2F transcr	protein-coding
K(lysine) ac	protein-coding
rhodopsin	protein-coding
mitochond	protein-coding
Ras-related	protein-coding
solute carri	protein-coding
protein pho	protein-coding
cytidine an	protein-coding
PDZ bindin	protein-coding
coenzyme	protein-coding
xanthine de	protein-coding
HERPUD fa	protein-coding
metastasis	protein-coding
intraflagell	protein-coding
succinyl-Co	protein-coding
pleckstrin h	protein-coding
chromodom	protein-coding
leucine rich	protein-coding
cyclin E1	protein-coding
vesicle-ass	protein-coding

ARP3 actin	protein-coding
mannose re	protein-coding
transcriptic	protein-coding
oxysterol b	protein-coding
solute carri	protein-coding
RIKEN cDN.	protein-coding
DEAH (Asp-	protein-coding
ring finger	protein-coding
acyl-Coenz	protein-coding
Tax1 (huma	protein-coding
Wiskott-Ald	protein-coding
tumor necr	protein-coding
SUMO1/se	protein-coding
secernin 1	protein-coding
fatty acid b	protein-coding
G protein p	protein-coding
C-terminal	protein-coding
homeobox	protein-coding
C-type lect	protein-coding
transducin	protein-coding
brain expre	protein-coding
lysine-rich	protein-coding
WD repeat	protein-coding
tribbles ho	protein-coding
gamma-sec	protein-coding
predicted g	protein-coding
leucine rich	protein-coding
small ArfGA	protein-coding
SMAD fami	protein-coding
protein tyro	protein-coding
E2F transcr	protein-coding
zinc finger,	protein-coding
carboxypep	protein-coding
family with	protein-coding
dynactin 5	protein-coding
solute carri	protein-coding
cell cycle a	protein-coding
solute carri	protein-coding
zinc finger,	protein-coding
minichrom	protein-coding
adhesion m	protein-coding
ST8 alpha-I	protein-coding
interferon	protein-coding
mitochond	protein-coding
histone clu	protein-coding
myosin, he	protein-coding
calmodulin	protein-coding

ribosomal	protein-coding
solute carri	protein-coding
6-phospho	protein-coding
formin bin	protein-coding
zinc finger,	protein-coding
presenilin	protein-coding
WD repeat	protein-coding
transmemk	protein-coding
RIKEN cDN	protein-coding
transmemk	protein-coding
ring finger	protein-coding
DEAD (Asp	protein-coding
DNA segme	protein-coding
a disintegri	protein-coding
formin-like	protein-coding
attractin lik	protein-coding
potassium	protein-coding
thyroid stir	protein-coding
CD28 antig	protein-coding
LIM domai	protein-coding
coiled-coil	protein-coding
heme bind	protein-coding
Ral GEF wit	protein-coding
glutathione	protein-coding
ets variant	protein-coding
RIKEN cDN	protein-coding
small integ	protein-coding
ets variant	protein-coding
signal pept	protein-coding
kyphoscoli	protein-coding
SWI/SNF re	protein-coding
COBW dom	protein-coding
RIKEN cDN	protein-coding
myristoylat	protein-coding
WD repeat	protein-coding
ELAV (emb	protein-coding
coenzyme	protein-coding
peptidylpro	protein-coding
NudC dom	protein-coding
phosphodi	protein-coding
nephronop	protein-coding
structural	protein-coding
kinesin fam	protein-coding
CD200 rece	protein-coding
ring-box 1	protein-coding
programm	protein-coding
activated l	protein-coding

CD47 antig	protein-coding
large subur	protein-coding
proteasom	protein-coding
thyrotroph	protein-coding
lipase, mer	protein-coding
zinc finger	protein-coding
G patch do	protein-coding
ATPase, cla	protein-coding
ribulose-5-	protein-coding
poly (ADP-ı	protein-coding
transferrin	protein-coding
progestero	protein-coding
proline-rich	protein-coding
prostaglan	protein-coding
chaperonir	protein-coding
CGG triplet	protein-coding
arrestin do	protein-coding
quiescin Q(	protein-coding
phosphoin	protein-coding
selenoprot	protein-coding
sema doma	protein-coding
poly(A) bin	protein-coding
collectin su	protein-coding
kelch-like 7	protein-coding
syntaxin 7	protein-coding
acidic (leuc	protein-coding
activated le	protein-coding
male-speci	protein-coding
SID1 transr	protein-coding
major facili	protein-coding
cadherin-lil	protein-coding
transcriptic	protein-coding
salvador hc	protein-coding
FERM dom	protein-coding
lysine (K)-s	protein-coding
choline phc	protein-coding
autophagy	protein-coding
cAMP-regu	protein-coding
junction ad	protein-coding
phosphatic	protein-coding
family with	protein-coding
ribosomal		protein-coding
myosin, he	protein-coding
sex comb c	protein-coding
basonuclin	protein-coding
diaphanou:	protein-coding
pyruvate d	protein-coding

phosphodi	protein-coding
tetraspanir	protein-coding
adaptor-re	protein-coding
taperin	protein-coding
GLI pathog	protein-coding
RIKEN cDN.	protein-coding
WD repeat	protein-coding
major facili	protein-coding
dolichol-ph	protein-coding
SMT3 supp	protein-coding
lysine (K)-s	protein-coding
zinc finger	protein-coding
phosphodi	protein-coding
coronin 7	protein-coding
retinitis pig	protein-coding
tumor sup	protein-coding
nuclear fac	protein-coding
epithelial s	protein-coding
cytochrom	protein-coding
MPN doma	protein-coding
protein kin	protein-coding
basic leucir	protein-coding
translocati	protein-coding
myeloid leu	protein-coding
gap junctio	protein-coding
cylindroma	protein-coding
golgi-specit	protein-coding
HAUS augn	protein-coding
SCAN dom	protein-coding
recombina	protein-coding
copine III	protein-coding
Smad nucle	protein-coding
espin	protein-coding
estrogen re	protein-coding
nuclear cap	protein-coding
transmemb	protein-coding
ATPase, Ca	protein-coding
predicted g	protein-coding
growth arr	protein-coding
solute carri	protein-coding
mitochond	protein-coding
neuromedi	protein-coding
tubulin cof	protein-coding
amyloid be	protein-coding
membrane	protein-coding
factor 8-as	protein-coding
neural prec	protein-coding

protein pho	protein-coding
carboxypep	protein-coding
RIKEN cDN.	protein-coding
family with	protein-coding
UDP-Gal:be	protein-coding
torsin A int	protein-coding
zinc finger	protein-coding
C-type lect	protein-coding
TAR DNA b	protein-coding
zinc finger	protein-coding
NIMA (neve	protein-coding
BCL2-antag	protein-coding
dehydroge	protein-coding
protein-L-is	protein-coding
nucleobind	protein-coding
stromal int	protein-coding
CART prepr	protein-coding
tripartite m	protein-coding
ethanol inc	protein-coding
nucleoredc	protein-coding
zinc finger,	protein-coding
kinesin fam	protein-coding
TGF-beta a	protein-coding
solute carri	protein-coding
lysine (K)-s	protein-coding
polymerase	protein-coding
serine (or c	protein-coding
DEAH (Asp-	protein-coding
UDP-Gal:be	protein-coding
lipin 2	protein-coding
NECAP end	protein-coding
H3 histone,	protein-coding
leucine rich	protein-coding
phosphotri	protein-coding
ribosomal p	protein-coding
thymopoie	protein-coding
gastrulatio	protein-coding
Harvey rat	protein-coding
hydroxyacy	protein-coding
SMC hinge	protein-coding
poly (ADP-r	protein-coding
vesicle tran	protein-coding
ATPase, cla	protein-coding
ring finger	protein-coding
solute carri	protein-coding
calcium/ca	protein-coding
inositol 1,4	protein-coding

diacylglyce	protein-coding
LIM domai	protein-coding
mitochond	protein-coding
TGFB-induc	protein-coding
transforma	protein-coding
MRT4, mRI	protein-coding
P450 (cyto	protein-coding
Shc SH2-dc	protein-coding
potassium	protein-coding
septin 9	protein-coding
tripartite n	protein-coding
CDC42 binc	protein-coding
phosphodi	protein-coding
thioredoxir	protein-coding
interferon	protein-coding
solute carri	protein-coding
zinc finger,	protein-coding
short coile	protein-coding
chemokine	protein-coding
EP300 inte	protein-coding
1-acylglyce	protein-coding
signal sequ	protein-coding
transforme	protein-coding
BEN domai	protein-coding
multivesicu	protein-coding
SAC3 doma	protein-coding
mirror-ima	protein-coding
alkB, alkyla	protein-coding
trans-2,3-e	protein-coding
protein ph	protein-coding
CD200 rece	protein-coding
ribosomal	protein-coding
muscleblin	protein-coding
proteasom	protein-coding
kinesin fan	protein-coding
family with	protein-coding
solute carri	protein-coding
tumor necr	protein-coding
cytochrom	protein-coding
early endo	protein-coding
hexokinase	protein-coding
OTU doma	protein-coding
retinol deh	protein-coding
olfactomec	protein-coding
hydroxyste	protein-coding
peptidylprc	protein-coding
transmemk	protein-coding

leukotriene	protein-coding
histone dea	protein-coding
cyclin T2	protein-coding
3'-phospho	protein-coding
C-type lect	protein-coding
peroxisome	protein-coding
tripeptidyl	protein-coding
Riken cDNA	protein-coding
heterogene	protein-coding
aquaporin	protein-coding
asparagine	protein-coding
family with	protein-coding
zinc finger	protein-coding
FERM dom	protein-coding
vacuolar pr	protein-coding
ring finger	protein-coding
aldo-keto r	protein-coding
lysine (K)-s	protein-coding
expressed s	protein-coding
cDNA sequ	protein-coding
nudix (nucl	protein-coding
cyclin E2	protein-coding
inhibitor of	protein-coding
cyclin D3	protein-coding
hyaluronog	protein-coding
YTH domai	protein-coding
zinc finger	protein-coding
tetratricop	protein-coding
forkhead b	protein-coding
notch 2	protein-coding
zinc finger	protein-coding
chemokine	protein-coding
MOB kinas	protein-coding
avian musc	protein-coding
high mobili	protein-coding
interleukin	protein-coding
dpy-19-like	protein-coding
ski sarcoma	protein-coding
A kinase (P	protein-coding
chaperonir	protein-coding
TLC domair	protein-coding
sideroflexir	protein-coding
hairy and e	protein-coding
ubiquitin ir	protein-coding
interleukin	protein-coding
pleckstrin a	protein-coding
integrin be	protein-coding

adducin 3 (	protein-coding
family with	protein-coding
solute carri	protein-coding
metaxin 2	protein-coding
UDP-Gal:be	protein-coding
immunoglo	protein-coding
low-density	protein-coding
LIM domai	protein-coding
TGF-beta a	protein-coding
retinoblast	protein-coding
SET binding	protein-coding
Map3k7 C-	protein-coding
ecotropic v	protein-coding
phosphorib	protein-coding
nuclear fac	protein-coding
RIKEN cDN.	protein-coding
protein kin	protein-coding
CDK5 regul	protein-coding
genetic sup	protein-coding
branched c	protein-coding
calcium cha	protein-coding
X-linked my	protein-coding
diacylglyce	protein-coding
diacylglyce	protein-coding
basic transc	protein-coding
glycosylpho	protein-coding
Morf4 fami	protein-coding
GNAS (guan	protein-coding
protein pho	protein-coding
suppressor	protein-coding
glypican 1	protein-coding
cell divisior	protein-coding
cytohesin 1	protein-coding
family with	protein-coding
signal pept	protein-coding
polymerase	protein-coding
neurotensi	protein-coding
notch 1	protein-coding
zinc finger	protein-coding
transmemb	protein-coding
peptidoglyc	protein-coding
salt inducib	protein-coding
calpastatin	protein-coding
transient re	protein-coding
cellular nuc	protein-coding
tumor necr	protein-coding
ribosomal p	protein-coding

timeless ci	protein-coding
c-myc bind	protein-coding
RNA bindin	protein-coding
LIM domai	protein-coding
deoxyuridi	protein-coding
HERV-H LTI	protein-coding
kinesin fam	protein-coding
radical S-ac	protein-coding
serglycin	protein-coding
golgi reass	protein-coding
transmemb	protein-coding
CTF8, chro	protein-coding
LIM domai	protein-coding
hemochro	protein-coding
K(lysine) ac	protein-coding
armadillo r	protein-coding
transmemb	protein-coding
poly(rC) bi	protein-coding
plasminog	protein-coding
RNA 3'-ter	protein-coding
5,10-meth	protein-coding
DDHD dom	protein-coding
glutaminas	protein-coding
UDP-gluco	protein-coding
methyltran	protein-coding
lysosomal	protein-coding
bromodom	protein-coding
podoplani	protein-coding
solute carr	protein-coding
histone clu	protein-coding
mitogen-ac	protein-coding
coagulatio	protein-coding
zinc finger	protein-coding
ring finger	protein-coding
solute carr	protein-coding
cDNA sequ	protein-coding
ribonuclea	protein-coding
RIKEN cDN	protein-coding
CDK5 regul	protein-coding
RAD50 hon	protein-coding
zinc finger,	protein-coding
lamin B rec	protein-coding
zinc finger,	protein-coding
required fc	protein-coding
ADP-ribosy	protein-coding
POU doma	protein-coding
Wilms tum	protein-coding

adrenergic	protein-coding
UBX domai	protein-coding
actin, beta	protein-coding
sirtuin 7	protein-coding
RAB14, me	protein-coding
DNA fragm	protein-coding
sorting nex	protein-coding
DNA polym	protein-coding
adaptor-re	protein-coding
phosphodi	protein-coding
doublesex	protein-coding
Ewing tum	protein-coding
charged m	protein-coding
RIKEN cDN	protein-coding
deleted in	protein-coding
ubiquitin p	protein-coding
ubiquitin s	protein-coding
PX domain	protein-coding
CAP, adeny	protein-coding
C-terminal	protein-coding
arrestin do	protein-coding
taste recep	protein-coding
Mediterrar	protein-coding
coiled-coil	protein-coding
RAB11 fam	protein-coding
tripartite n	protein-coding
sulfite oxid	protein-coding
zinc finger,	protein-coding
WD repeat	protein-coding
RIKEN cDN	protein-coding
ezrin	protein-coding
cadherin 1	protein-coding
placenta e	protein-coding
calcium/ca	protein-coding
microtubul	protein-coding
eukaryotic	protein-coding
mannoside	protein-coding
ADP-ribosy	protein-coding
estrogen re	protein-coding
DNA dama	protein-coding
thioredoxir	protein-coding
testis expre	protein-coding
achaete-sc	protein-coding
eyes absen	protein-coding
phospholip	protein-coding
developme	protein-coding
INO80 com	protein-coding

DAZ associ	protein-coding
autophagy	protein-coding
cytochrom	protein-coding
integrin be	protein-coding
suppressor	protein-coding
ADP-ribosy	protein-coding
F-box prote	protein-coding
F-box prote	protein-coding
flavin cont	protein-coding
alcohol deh	protein-coding
signal trans	protein-coding
G protein-c	protein-coding
gamma-am	protein-coding
solute carri	protein-coding
LIM domai	protein-coding
DnaJ (Hsp4	protein-coding
acetyl-Coe	protein-coding
transformi	protein-coding
RIKEN cDN	protein-coding
adrenergic	protein-coding
translocase	protein-coding
interleukin	protein-coding
phosducin-	protein-coding
phosphodi	protein-coding
CCAAT/enh	protein-coding
inverted fo	protein-coding
aminoadip	protein-coding
signal trans	protein-coding
ADP-ribosy	protein-coding
purine rich	protein-coding
guanine de	protein-coding
2-phospho	protein-coding
spleen tyro	protein-coding
mitogen-ac	protein-coding
bone morp	protein-coding
COP9 (cons	protein-coding
prefoldin s	protein-coding
claudin 23	protein-coding
ATP-bindin	protein-coding
leucine rich	protein-coding
TNF recept	protein-coding
interferon	protein-coding
protein ph	protein-coding
vav 2 onco	protein-coding
coiled-coil	protein-coding
abhydrolas	protein-coding
retinol deh	protein-coding

neuregulin	protein-coding
sprouty pro	protein-coding
kelch-like 5	protein-coding
translocase	protein-coding
optic atrop	protein-coding
zinc finger	protein-coding
translocase	protein-coding
ubiquitin-c	protein-coding
S100 calciu	protein-coding
family with	protein-coding
intercellula	protein-coding
c-Maf indu	protein-coding
limb region	protein-coding
hypoxia-ind	protein-coding
serglycin	protein-coding
chemokine	protein-coding
inositol pol	protein-coding
feline sarco	protein-coding
hect domai	protein-coding
RIKEN cDNA	protein-coding
RWD doma	protein-coding
AT-hook tra	protein-coding
transmemb	protein-coding
smg-8 hom	protein-coding
cell division	protein-coding
ornithine a	protein-coding
tetratricop	protein-coding
hydroxyste	protein-coding
solute carri	protein-coding
cell division	protein-coding
cornichon l	protein-coding
mannosida	protein-coding
polo-like ki	protein-coding
ribosomal p	protein-coding
nucleopori	protein-coding
cellular rep	protein-coding
endonuclea	protein-coding
proline deh	protein-coding
RIKEN cDNA	protein-coding
heat shock	protein-coding
Cas scaffold	protein-coding
protein kin	protein-coding
serine/argi	protein-coding
UTP18, sma	protein-coding
NOP56 ribo	protein-coding
chromodom	protein-coding
capping pro	protein-coding

serine/argi	protein-coding
zinc finger	protein-coding
vesicle-ass	protein-coding
glutamyl ar	protein-coding
potassium	protein-coding
cyclin-depe	protein-coding
zinc finger	protein-coding
mitogen-ac	protein-coding
actin relate	protein-coding
protein pho	protein-coding
CWC25 spli	protein-coding
Meis home	protein-coding
regulator o	protein-coding
cytochrom	protein-coding
Kruppel-lik	protein-coding
dihydropyr	protein-coding
programm	protein-coding
family with	protein-coding
clathrin, lig	protein-coding
microtubul	protein-coding
nicotinami	protein-coding
DEP domai	protein-coding
GRB2-relat	protein-coding
secretory c	protein-coding
adenylate k	protein-coding
tripartite m	protein-coding
interferon	protein-coding
predicted g	protein-coding
KxDL motif	protein-coding
leucine-rich	protein-coding
ligase IV, D	protein-coding
ataxin 10	protein-coding
sterile alph	protein-coding
RAB12, me	protein-coding
HEAT repea	protein-coding
CDK5 regul	protein-coding
sema doma	protein-coding
valosin con	protein-coding
jun proto-c	protein-coding
phosphorib	protein-coding
fibronectin	protein-coding
adducin 3 (	protein-coding
COP9 (cons	protein-coding
FK506 bind	protein-coding
sal-like 1 (D	protein-coding
ribosomal p	protein-coding
proline rich	protein-coding

basic leucir	protein-coding
interferon	protein-coding
transmemk	protein-coding
patatin-like	protein-coding
Kruppel-lik	protein-coding
NLR family,	protein-coding
Ras associa	protein-coding
splA/ryano	protein-coding
zinc finger	protein-coding
Sp140 nucl	protein-coding
RIKEN cDN.	protein-coding
platelet de	protein-coding
Fc receptoi	protein-coding
GSK3B inte	protein-coding
fibronectin	protein-coding
cyclin L1	protein-coding
amyotroph	protein-coding
glucosamir	protein-coding
polyhomec	protein-coding
protein (pe	protein-coding
transformii	protein-coding
protein arg	protein-coding
matrix met	protein-coding
lysozyme-li	protein-coding
transcriptic	protein-coding
predicted g	protein-coding
jade family	protein-coding
PDZ domai	protein-coding
proteasom	protein-coding
lysine (K)-s	protein-coding
DEAD (Asp-	protein-coding
oxysterol b	protein-coding
leucine rich	protein-coding
melanocyte	protein-coding
histone dea	protein-coding
transcriptic	protein-coding
ribophorin	protein-coding
SCY1-like 1	protein-coding
mitogen-ac	protein-coding
solute carri	protein-coding
exostoses (	protein-coding
pleckstrin l	protein-coding
voltage-de|	protein-coding
Rab interac	protein-coding
immediate	protein-coding
septin 9	protein-coding
predicted g	protein-coding

zinc finger,	protein-coding
stromal int	protein-coding
family with	protein-coding
cyclin-depe	protein-coding
eukaryotic	protein-coding
coactosin-l	protein-coding
CLIP associ	protein-coding
RIKEN cDN.	protein-coding
CD274 anti	protein-coding
lysosomal t	protein-coding
phosphatic	protein-coding
polymerase	protein-coding
mitochond	protein-coding
1-acylglyce	protein-coding
RAB2A, me	protein-coding
polyhomec	protein-coding
NSFL1 (p97	protein-coding
thioredoxir	protein-coding
fibroblast g	protein-coding
ATPase, Ca	protein-coding
SET domair	protein-coding
ankyrin rep	protein-coding
missing oo	protein-coding
cytochrom	protein-coding
phosphorib	protein-coding
alpha-2-gly	protein-coding
unc-119 hc	protein-coding
sterile alph	protein-coding
lymphobla:	protein-coding
protein tyr	protein-coding
transcriptic	protein-coding
protein kin	protein-coding
plexin C1	protein-coding
actin relate	protein-coding
polycystic k	protein-coding
Max protei	protein-coding
protein ph	protein-coding
early growt	protein-coding
RIKEN cDN.	protein-coding
autophagy	protein-coding
dihydrolipc	protein-coding
synaptic ve	protein-coding
RIKEN cDN.	protein-coding
proteasom	protein-coding
F-box and l	protein-coding
frizzled hor	protein-coding
elastase, n	protein-coding

lysine (K)-s	protein-coding
heterogene	protein-coding
zinc finger,	protein-coding
ATP-bindin	protein-coding
2-deoxyrib	protein-coding
transthyret	protein-coding
retinoblast	protein-coding
microsoma	protein-coding
chloride ch	protein-coding
stefin A3	protein-coding
tRNA splici	protein-coding
RIKEN cDN.	protein-coding
solute carri	protein-coding
cyclin Pas1	protein-coding
ubiquitin-c	protein-coding
transmemb	protein-coding
regulator o	protein-coding
mitogen-ac	protein-coding
prostagland	protein-coding
RIKEN cDN.	protein-coding
Rho guanin	protein-coding
collagen, ty	protein-coding
insulin-like	protein-coding
sosondowa	protein-coding
ectoderma	protein-coding
immunity-r	protein-coding
RIKEN cDN.	protein-coding
phosphose	protein-coding
fatty acyl C	protein-coding
zinc finger	protein-coding
calcitonin g	protein-coding
zinc finger	protein-coding
cullin 1	protein-coding
glutaredox	protein-coding
UDP-Gal:be	protein-coding
RAB21, me	protein-coding
TBC1 doma	protein-coding
clustered n	protein-coding
thromboxa	protein-coding
transmemb	protein-coding
fukutin rela	protein-coding
aldehyde d	protein-coding
plakophilin	protein-coding
EBNA1 bind	protein-coding
hepatocyte	protein-coding
fibroblast a	protein-coding
GTPase act	protein-coding

ATP-bindin	protein-coding
zinc finger	protein-coding
methionine	protein-coding
supervillin	protein-coding
sushi, nido	protein-coding
NADH dehy	protein-coding
ring finger	protein-coding
interleukin	protein-coding
differential	protein-coding
zinc finger,	protein-coding
SH3-domai	protein-coding
carbonic ar	protein-coding
l(3)mbt-like	protein-coding
zinc finger	protein-coding
phospholip	protein-coding
LIM homec	protein-coding
phosphatic	protein-coding
t-complex	protein-coding
mcf.2 trans	protein-coding
zinc finger	protein-coding
Smg-7 hom	protein-coding
tripartite m	protein-coding
ankyrin rep	protein-coding
PTK2 prote	protein-coding
MYB bindir	protein-coding
establishm	protein-coding
jun proto-c	protein-coding
dual specif	protein-coding
gasdermin	protein-coding
platelet-ac	protein-coding
insulin rece	protein-coding
CD33 antig	protein-coding
Fanconi an	protein-coding
solute carri	protein-coding
placenta ex	protein-coding
angel home	protein-coding
xylosylprot	protein-coding
dual specif	protein-coding
solute carri	protein-coding
tumor prot	protein-coding
transducin	protein-coding
cDNA sequ	protein-coding
MAP kinase	protein-coding
frizzled hor	protein-coding
testis expre	protein-coding
activating t	protein-coding
chromodor	protein-coding

glycerate k	protein-coding
outer dens	protein-coding
hexokinase	protein-coding
vasoactive	protein-coding
SEC23A (S.	protein-coding
APC memb	protein-coding
ADP-deper	protein-coding
transmemb	protein-coding
proteasom	protein-coding
GTF2I repe	protein-coding
selectin, ly	protein-coding
tubulin, alp	protein-coding
calcium/ca	protein-coding
transmemb	protein-coding
ATPase, Ca	protein-coding
abhydrolas	protein-coding
matrix Gla	protein-coding
K(lysine) ac	protein-coding
NOP2 nucl	protein-coding
CART prep	protein-coding
additional	protein-coding
cortactin b	protein-coding
heterogen	protein-coding
deoxyribor	protein-coding
formin bin	protein-coding
tetratricop	protein-coding
leukemia ir	protein-coding
exocyst co	protein-coding
SEC23B (S.	protein-coding
acyloxyacy	protein-coding
3-oxoacid (	protein-coding
zinc finger,	protein-coding
elaC homo	protein-coding
ubiquitin s	protein-coding
lysosomal-	protein-coding
nucleic aci	protein-coding
protein ph	protein-coding
actin relate	protein-coding
stromal an	protein-coding
chemokine	protein-coding
cathepsin S	protein-coding
thyroid ho	protein-coding
CLIP associ	protein-coding
hook homo	protein-coding
Kruppel-lik	protein-coding
interleukin	protein-coding
elongation	protein-coding

biregional (	protein-coding
kelch-like 2	protein-coding
adaptor-re	protein-coding
kelch-like 6	protein-coding
MYC bindir	protein-coding
nuclear tra	protein-coding
thrombosp	protein-coding
DCN1, defe	protein-coding
tripartite m	protein-coding
suppressor	protein-coding
phosphatic	protein-coding
trypsin dor	protein-coding
tensin 4	protein-coding
family with	protein-coding
leptin rece	protein-coding
suppressor	protein-coding
methylmal	protein-coding
PHD finger	protein-coding
phosphoin	protein-coding
ubiquitin s	protein-coding
MAP/micro	protein-coding
serine (or c	protein-coding
ectonucleo	protein-coding
plexin D1	protein-coding
CDC42 sma	protein-coding
glutathione	protein-coding
family with	protein-coding
BPI fold co	protein-coding
zinc finger	protein-coding
nei endonu	protein-coding
testis and c	protein-coding
protein tyr	protein-coding
myocyte er	protein-coding
growth diff	protein-coding
BCL2 bindi	protein-coding
ring finger	protein-coding
SH2B adap	protein-coding
leucine-rich	protein-coding
unc-51 like	protein-coding
shisa homo	protein-coding
cannabinoi	protein-coding
degenerati	protein-coding
NADH dehy	protein-coding
transmemb	protein-coding
zinc finger	protein-coding
protein pho	protein-coding
doublesex	protein-coding

cell cycle p	protein-coding
zinc ribbon	protein-coding
family with	protein-coding
zinc finger	protein-coding
CD80 antig	protein-coding
idnK glucor	protein-coding
F-box and l	protein-coding
intraflagell	protein-coding
zinc finger	protein-coding
numb gene	protein-coding
mitochond	protein-coding
GTPase act	protein-coding
RIKEN cDN.	protein-coding
family with	protein-coding
SET domair	protein-coding
phosphatic	protein-coding
ankyrin rep	protein-coding
endoplasm	protein-coding
phospholip	protein-coding
bromodom	protein-coding
zinc finger	protein-coding
protein tyr	protein-coding
chitobiase,	protein-coding
secretory c	protein-coding
toll-like rec	protein-coding
protein kin	protein-coding
enhancer c	protein-coding
cryptochro	protein-coding
interleukin	protein-coding
mastermin	protein-coding
ribosome b	protein-coding
NEDD4 bin	protein-coding
ring finger	protein-coding
low density	protein-coding
RIKEN cDN.	protein-coding
RNA polym	protein-coding
chromatin	protein-coding
tripartite m	protein-coding
zinc and rir	protein-coding
cAMP resp	protein-coding
cell divisior	protein-coding
mannoside	protein-coding
ATPase, Na	protein-coding
ADP-ribosy	protein-coding
matrix met	protein-coding
amplified ir	protein-coding
chemokine	protein-coding

immunoglo	protein-coding
cDNA sequ	protein-coding
TBC1 doma	protein-coding
mannosida	protein-coding
notchless h	protein-coding
asialoglyco	protein-coding
small nucle	protein-coding
Ras associa	protein-coding
Fas ligand (	protein-coding
squamous	protein-coding
ubiquitin-c	protein-coding
small nucle	protein-coding
low density	protein-coding
postmeioti	protein-coding
potassium	protein-coding
ubiquitin sp	protein-coding
CAS1 doma	protein-coding
Hermansky	protein-coding
solute carri	protein-coding
ubiquitin sp	protein-coding
engulfment	protein-coding
glyceronep	protein-coding
LSM6 homo	protein-coding
ADP-ribosy	protein-coding
3'-phospho	protein-coding
mitochond	protein-coding
solute carri	protein-coding
RIKEN cDNA	protein-coding
disrupted i	protein-coding
LSM2 homo	protein-coding
spindlin 1	protein-coding
chemokine	protein-coding
tripartite m	protein-coding
tetratricop	protein-coding
carboxypep	protein-coding
MKL (mega	protein-coding
SURP and G	protein-coding
ankyrin rep	protein-coding
bromodom	protein-coding
pleckstrin h	protein-coding
membrane	protein-coding
B cell leuke	protein-coding
SWI/SNF re	protein-coding
UFM1 spec	protein-coding
RAD9 homo	protein-coding
adenylate k	protein-coding
retinoblast	protein-coding

autophagy	protein-coding
cell divisior	protein-coding
proteasom	protein-coding
fragile X m	protein-coding
ATPase, Ca	protein-coding
zinc finger	protein-coding
expressed :	protein-coding
transmemb	protein-coding
selectin, ly	protein-coding
protein ph	protein-coding
transmemb	protein-coding
ring finger	protein-coding
plexin A1	protein-coding
membrane	protein-coding
zinc finger	protein-coding
protein tyr	protein-coding
predicted g	protein-coding
CKLF-like M	protein-coding
family with	protein-coding
chondroitir	protein-coding
peptidylpr	protein-coding
ethanolam	protein-coding
predicted g	protein-coding
androgen c	protein-coding
RAB2A, me	protein-coding
multiple C2	protein-coding
zinc finger	protein-coding
integrin be	protein-coding
biogenesis	protein-coding
exocyst cor	protein-coding
zinc finger	protein-coding
prostaglan	protein-coding
adrenergic	protein-coding
YTH domai	protein-coding
zinc and rir	protein-coding
cyclin C	protein-coding
histocompa	protein-coding
synaptone	protein-coding
peptidylpr	protein-coding
RIKEN cDN	protein-coding
solute carri	protein-coding
TSC22 dom	protein-coding
SFT2 doma	protein-coding
olfactory re	protein-coding
dihydrolipc	protein-coding
family with	protein-coding
glutamyl ar	protein-coding

vinculin	protein-coding
WD repeat	protein-coding
B cell leuke	protein-coding
ring finger	protein-coding
zinc finger	protein-coding
predicted g	protein-coding
Ras and Ra	protein-coding
DOT1-like,	protein-coding
pogo trans	protein-coding
cyclin-depe	protein-coding
nebulin-rel	protein-coding
cilia and fla	protein-coding
inhibitor of	protein-coding
sperm anti	protein-coding
phosphatic	protein-coding
inositol pol	protein-coding
mitochond	protein-coding
glutathione	protein-coding
thymoma v	protein-coding
membrane	protein-coding
ER degrada	protein-coding
BRF1 homo	protein-coding
protein pho	protein-coding
histocompa	protein-coding
zinc finger,	protein-coding
transmemb	protein-coding
F-box and V	protein-coding
resistance	protein-coding
major facili	protein-coding
B cell trans	protein-coding
erythrocyte	protein-coding
cytochrom	protein-coding
UDP-N-ace	protein-coding
ribosomal	protein-coding
RAB11 fam	protein-coding
torsin A int	protein-coding
solute carri	protein-coding
signal-regu	protein-coding
TGF-beta a	protein-coding
basic trans	protein-coding
interferon	protein-coding
nucleopori	protein-coding
proteasom	protein-coding
cAMP resp	protein-coding
metaxin 1	protein-coding
patatin-like	protein-coding
furin (paire	protein-coding

major facili	protein-coding
CD47 antig	protein-coding
achaete-sc	protein-coding
myelin pro	protein-coding
neutrophil	protein-coding
DnaJ (Hsp4	protein-coding
sterile alph	protein-coding
Rho family	protein-coding
GTPase act	protein-coding
DAZ associ	protein-coding
jun D proto	protein-coding
eukaryotic	protein-coding
F-box prote	protein-coding
sirtuin 4	protein-coding
ariadne ub	protein-coding
GLI pathog	protein-coding
expressed :	protein-coding
mannosida	protein-coding
ribosomal	protein-coding
Ras associa	protein-coding
phosphohi:	protein-coding
RIKEN cDN.	protein-coding
schlafen 1	protein-coding
Kruppel-lik	protein-coding
ribosomal	protein-coding
TAF10 RNA	protein-coding
transmeml	protein-coding
trichorhinc	protein-coding
zinc finger	protein-coding
estrogen re	protein-coding
ankyrin rep	protein-coding
synaptotag	protein-coding
chemokine	protein-coding
family with	protein-coding
DEAD/H (A	protein-coding
predicted g	protein-coding
TATA box b	protein-coding
interferon	protein-coding
gonadotro	protein-coding
Tax1 (huma	protein-coding
arginine/se	protein-coding
protein-L-is	protein-coding
RIKEN cDN.	protein-coding
suppressio	protein-coding
trafficking	protein-coding
transformi	protein-coding
family with	protein-coding

complemen	protein-coding
protein-kin	protein-coding
sorting nex	protein-coding
tubulin tyr	protein-coding
bactericida	protein-coding
G patch do	protein-coding
StAR-relate	protein-coding
ATPase, cla	protein-coding
pyroglutam	protein-coding
NCK-associ	protein-coding
schlafen 2	protein-coding
transmemb	protein-coding
solute carri	protein-coding
cytochrom	protein-coding
CART prep	protein-coding
acyl-CoA sy	protein-coding
DENN/MAI	protein-coding
ATPase, Na	protein-coding
lon peptida	protein-coding
E74-like fa	protein-coding
interactor	protein-coding
transmemb	protein-coding
KTI12 hom	protein-coding
uridine-cyt	protein-coding
Ras associa	protein-coding
bombesin-l	protein-coding
coiled-coil	protein-coding
tocopherol	protein-coding
leucine rich	protein-coding
coiled-coil	protein-coding
transcriptio	protein-coding
mediator c	protein-coding
natural kill	protein-coding
v-raf-leuke	protein-coding
protein pho	protein-coding
ring finger	protein-coding
secretory c	protein-coding
nuclear rec	protein-coding
DNA-dama	protein-coding
RNA bindin	protein-coding
caspase act	protein-coding
SLAIN moti	protein-coding
ral guanine	protein-coding
phosphoino	protein-coding
spinster ho	protein-coding
dual specif	protein-coding
RAB33B, m	protein-coding

renalase, F.	protein-coding
ankyrin rep	protein-coding
zinc finger	protein-coding
metadherin	protein-coding
WD repeat	protein-coding
protein tyr	protein-coding
RIKEN cDN.	protein-coding
forkhead b	protein-coding
elongation	protein-coding
engulfmen	protein-coding
short chain	protein-coding
SMG1 hom	protein-coding
germ cell-s	protein-coding
deltex 4 ho	protein-coding
heat shock	protein-coding
flavin conta	protein-coding
src homolo	protein-coding
spindle app	protein-coding
vimentin-ty	protein-coding
nuclear rec	protein-coding
activity-dep	protein-coding
N-acetylglu	protein-coding
tRNA splici	protein-coding
leukocyte s	protein-coding
SCY1-like 1	protein-coding
CD274 anti	protein-coding
multiple C2	protein-coding
ribosomal l	protein-coding
mab-21-like	protein-coding
TAF15 RNA	protein-coding
AF4/FMR2	protein-coding
DnaJ (Hsp4	protein-coding
forkhead b	protein-coding
UDP-Gal:be	protein-coding
thyroid hor	protein-coding
ras homolo	protein-coding
protein inh	protein-coding
HORMA do	protein-coding
ATPase, Ca	protein-coding
asparagine	protein-coding
ribosomal p	protein-coding
ceramide s	protein-coding
NLR family,	protein-coding
ATPase, am	protein-coding
nuclear fac	protein-coding
splA/ryano	protein-coding
TAF6 RNA p	protein-coding

coagulatior	protein-coding
pecanex-lik	protein-coding
trichoplein	protein-coding
heterogene	protein-coding
RasGEF doı	protein-coding
transforma	protein-coding
transmemk	protein-coding
Hermansky	protein-coding
mitogen-ac	protein-coding
prolactin fa	protein-coding
chromatin	protein-coding
tetratricop	protein-coding
paralemmi	protein-coding
zinc finger	protein-coding
solute carri	protein-coding
protocadhe	protein-coding
filamin A ir	protein-coding
transcriptic	protein-coding
mal, T cell (	protein-coding
zinc finger	protein-coding
protein pho	protein-coding
fibronectin	protein-coding
endothelin	protein-coding
zinc finger	protein-coding
zinc finger	protein-coding
polypyrimi	protein-coding
nuclear rec	protein-coding
angiopoieti	protein-coding
leucine-ricl	protein-coding
cDNA sequ	protein-coding
cytochrom	protein-coding
RAB intera	protein-coding
RIKEN cDN	protein-coding
septin 5	protein-coding
translocase	protein-coding
transmemk	protein-coding
myocyte er	protein-coding
talin 2	protein-coding
BTG3 assoc	protein-coding
differential	protein-coding
potassium	protein-coding
zinc finger	protein-coding
lysine (K)-s	protein-coding
cell divisior	protein-coding
cyclin-depe	protein-coding
histone dea	protein-coding
zinc finger	protein-coding

metaxin 3	protein-coding
oxysterol b	protein-coding
family with	protein-coding
sorting nex	protein-coding
APC memb	protein-coding
CDK5 regul	protein-coding
TBC1 doma	protein-coding
tRNA isope	protein-coding
hexose-6-p	protein-coding
RIKEN cDN.	protein-coding
family with	protein-coding
DnaJ (Hsp4	protein-coding
mortality fa	protein-coding
coiled-coil	protein-coding
nudix (nucl	protein-coding
forkhead b	protein-coding
RIKEN cDN.	protein-coding
cyclin D3	protein-coding
insulinoma	protein-coding
phosphodi	protein-coding
spermatog	protein-coding
phosphodi	protein-coding
adaptor-re	protein-coding
retinoic aci	protein-coding
sal-like 1 (D	protein-coding
translocase	protein-coding
CDC-like ki	protein-coding
tetratricop	protein-coding
neural prec	protein-coding
F-box prote	protein-coding
anthrax to	protein-coding
sterile alph	protein-coding
CD9 antige	protein-coding
sterile alph	protein-coding
Meis home	protein-coding
transcobal	protein-coding
leucine rich	protein-coding
RAB7, men	protein-coding
trinucleoti	protein-coding
glycophori	protein-coding
protein ph	protein-coding
phospholip	protein-coding
tetratricop	protein-coding
microtubul	protein-coding
tRNA aspar	protein-coding
olfactory re	protein-coding
Rap guanin	protein-coding

arachidona	protein-coding
M-phase sp	protein-coding
diacylglyce	protein-coding
acyl-CoA sy	protein-coding
cellular nuc	protein-coding
6-phospho	protein-coding
NADH dehy	protein-coding
interferon-	protein-coding
contactin 5	protein-coding
neural prec	protein-coding
ring finger	protein-coding
WD repeat	protein-coding
SMT3 supp	protein-coding
G protein-c	protein-coding
KDM3B lysi	protein-coding
DDB1 and (	protein-coding
midnolin	protein-coding
A kinase (P	protein-coding
poly(rC) bir	protein-coding
PTK2 prote	protein-coding
transient re	protein-coding
TBC1 doma	protein-coding
cadherin, E	protein-coding
phospholip	protein-coding
protein tyr	protein-coding
solute carri	protein-coding
RIKEN cDN	protein-coding
SprT-like N	protein-coding
family with	protein-coding
5-hydroxyt	protein-coding
ubiquitin fu	protein-coding
ADP-ribosy	protein-coding
ubiquitin a	protein-coding
DiGeorge s	protein-coding
mitochond	protein-coding
interferon	protein-coding
anoctamin	protein-coding
UDP-Gal:be	protein-coding
SET domair	protein-coding
glutaredox	protein-coding
cellular nuc	protein-coding
Kruppel-lik	protein-coding
aarF domai	protein-coding
trinucleotic	protein-coding
PHD finger	protein-coding
Rap1 intera	protein-coding
F11 recept	protein-coding

ecto-NOX c	protein-coding
NIMA (neve	protein-coding
transmemb	protein-coding
HERPUD fa	protein-coding
ribosomal p	protein-coding
ets variant	protein-coding
phosphatic	protein-coding
ubiquitin-c	protein-coding
RNA bindin	protein-coding
trafficking p	protein-coding
tyrosine 3-	protein-coding
USP6 N-ter	protein-coding
methyltran	protein-coding
Rho GTPas	protein-coding
arsA arseni	protein-coding
testis expre	protein-coding
ATPase, Na	protein-coding
Ewing tum	protein-coding
transmemb	protein-coding
solute carri	protein-coding
HtrA serine	protein-coding
purine rich	protein-coding
DnaJ (Hsp4	protein-coding
integrin alp	protein-coding
general tra	protein-coding
suppressor	protein-coding
histone dea	protein-coding
angiotensir	protein-coding
ras respons	protein-coding
ADP-ribosy	protein-coding
DEAD/H (A	protein-coding
Sec24 relat	protein-coding
neurotensi	protein-coding
zinc finger,	protein-coding
N(alpha)-ac	protein-coding
NHP2 ribor	protein-coding
RIKEN cDN	protein-coding
receptor (c	protein-coding
lysophosph	protein-coding
arrestin, be	protein-coding
family with	protein-coding
polymerase	protein-coding
heparin-bir	protein-coding
transformir	protein-coding
RIKEN cDN	protein-coding
yrdC doma	protein-coding
pseudouric	protein-coding

expressed :	protein-coding
interleukin	protein-coding
lymphocyte	protein-coding
F-box and \	protein-coding
dynein, axc	protein-coding
RIKEN cDN.	protein-coding
insulin-like	protein-coding
thymus cel	protein-coding
contactin 6	protein-coding
signal trans	protein-coding
ubiquinol-c	protein-coding
coiled-coil	protein-coding
mutL homc	protein-coding
target of m	protein-coding
TOPBP1-int	protein-coding
zinc finger	protein-coding
LIM domaiı	protein-coding
WD repeat	protein-coding
neuralized	protein-coding
ubiquitin-c	protein-coding
phosphoin	protein-coding
Ras associa	protein-coding
glutathione	protein-coding
cell divisior	protein-coding
myotubula	protein-coding
peroxisom	protein-coding
heterogene	protein-coding
WD repeat	protein-coding
mitochond	protein-coding
TBC1 doma	protein-coding
tumor-assc	protein-coding
REST corep	protein-coding
DMRT-like	protein-coding
ribosomal	protein-coding
retinol deh	protein-coding
death effec	protein-coding
splicing fac	protein-coding
WW domai	protein-coding
interleukin	protein-coding
melanoreg	protein-coding
inositol pol	protein-coding
muscleblin	protein-coding
cactin, splic	protein-coding
solute carri	protein-coding
zinc finger	protein-coding
canopy 1 h	protein-coding
TSC22 dom	protein-coding

zinc finger,	protein-coding
cytochrom	protein-coding
5'-nucleoti	protein-coding
centrin 3	protein-coding
Harvey rat	protein-coding
REX2, RNA	protein-coding
1-acylglyce	protein-coding
PDS5, regu	protein-coding
shugoshin-	protein-coding
growth arr	protein-coding
single-stran	protein-coding
zinc finger	protein-coding
PRP19/PSC	protein-coding
peptidylgly	protein-coding
tumor necr	protein-coding
junctophili	protein-coding
carbohydra	protein-coding
adrenergic	protein-coding
early growt	protein-coding
muscleblin	protein-coding
cytochrom	protein-coding
tenascin C	protein-coding
protein kin	protein-coding
pseudouric	protein-coding
RIKEN cDN	protein-coding
tripartite m	protein-coding
phosphoty	protein-coding
docking pro	protein-coding
mortality fa	protein-coding
THAP doma	protein-coding
trans-actin	protein-coding
antigen ide	protein-coding
DEAD (Asp-	protein-coding
nucleobind	protein-coding
cDNA sequ	protein-coding
islet cell au	protein-coding
jagunal hor	protein-coding
B cell leuke	protein-coding
protein kin	protein-coding
valosin con	protein-coding
ring finger	protein-coding
scaffold att	protein-coding
RIKEN cDN	protein-coding
coagulatior	protein-coding
ras homolo	protein-coding
period circa	protein-coding
fucose-1-pl	protein-coding

polycomb g	protein-coding
inhibin bet	protein-coding
tripartite m	protein-coding
solute carri	protein-coding
F-box prote	protein-coding
SR-related	protein-coding
free fatty a	protein-coding
myocardial	protein-coding
RNA bindin	protein-coding
lipopolysac	protein-coding
Fc receptor	protein-coding
breast canc	protein-coding
ELAV (emb	protein-coding
resistance	protein-coding
INO80 hom	protein-coding
EGF-like do	protein-coding
adenylate k	protein-coding
Kruppel-lik	protein-coding
leucine rich	protein-coding
sorting nex	protein-coding
ATPase, cla	protein-coding
protein pho	protein-coding
hypoxia inc	protein-coding
kinase supp	protein-coding
ATPase, cla	protein-coding
Bardet-Bie	protein-coding
CAS1 doma	protein-coding
ribosomal p	protein-coding
componen	protein-coding
Rho-associ	protein-coding
NADH dehy	protein-coding
bromodom	protein-coding
X-linked my	protein-coding
RIKEN cDN	protein-coding
N-myc dow	protein-coding
sarcoglycan	protein-coding
serine/thre	protein-coding
calcitonin r	protein-coding
regulator o	protein-coding
cullin 3	protein-coding
nucleopori	protein-coding
DEAD (Asp-	protein-coding
hydroxyste	protein-coding
DEAD (Asp-	protein-coding
pleckstrin h	protein-coding
zinc finger	protein-coding
zinc finger	protein-coding

leucine-ricl	protein-coding
karyopheri	protein-coding
seryl-amin(	protein-coding
G protein-c	protein-coding
phosphatic	protein-coding
immediate	protein-coding
cytochrom	protein-coding
protein kin	protein-coding
TNFAIP3 in	protein-coding
regulating :	protein-coding
glomulin, F	protein-coding
ATPase, H+	protein-coding
Rho GTPas	protein-coding
Vpr (HIV-1)	protein-coding
WD repeat	protein-coding
phosphatic	protein-coding
tetraspanir	protein-coding
chromobo›	protein-coding
pecanex-lik	protein-coding
cytoskeleto	protein-coding
2-phospho:	protein-coding
ribosomal		protein-coding
neuron nav	protein-coding
immunity-r	protein-coding
adenylate l	protein-coding
DNA segme	protein-coding
serine (or c	protein-coding
sphingosin	protein-coding
twist basic	protein-coding
zinc finger,	protein-coding
zinc finger	protein-coding
runt relate	protein-coding
transmemb	protein-coding
kelch repea	protein-coding
TBK1 bindi	protein-coding
discoidin d	protein-coding
phosphopr	protein-coding
lamin B rec	protein-coding
RAD1 hom	protein-coding
prostaglan	protein-coding
kinesin fam	protein-coding
cleavage ar	protein-coding
dedicator c	protein-coding
nudix (nucl	protein-coding
regulator o	protein-coding
CAS1 doma	protein-coding
RRN3 RNA	protein-coding

ring finger	protein-coding
R-spondin :	protein-coding
septin 2	protein-coding
dachshund	protein-coding
serine (or c	protein-coding
negative re	protein-coding
integrator (	protein-coding
U2 small nu	protein-coding
NADH dehy	protein-coding
VPS9 doma	protein-coding
solute carri	protein-coding
nuclear rec	protein-coding
translocase	protein-coding
solute carri	protein-coding
ring finger	protein-coding
suppressor	protein-coding
LIM domai	protein-coding
stromal an	protein-coding
USO1 vesic	protein-coding
cyclin-depe	protein-coding
Max protei	protein-coding
mitochond	protein-coding
cap methyl	protein-coding
flavin conta	protein-coding
dedicator c	protein-coding
short chain	protein-coding
progestin a	protein-coding
transcriptic	protein-coding
Aly/REF ex	protein-coding
pore formi	protein-coding
TSSK6 activ	protein-coding
Friend leuk	protein-coding
adaptor-re	protein-coding
transmemb	protein-coding
sperm asso	protein-coding
ARP2 actin	protein-coding
capping pro	protein-coding
non-catalyt	protein-coding
protein arg	protein-coding
katanin p60	protein-coding
spectrin re	protein-coding
dehydroge	protein-coding
ubiquitin C	protein-coding
MYST/Esa1	protein-coding
deleted in l	protein-coding
galactosylc	protein-coding
DNA-dama	protein-coding

WD repeat	protein-coding
ADAMTS-li	protein-coding
tumor necr	protein-coding
zinc finger	protein-coding
poly (ADP-r	protein-coding
neuroguidi	protein-coding
nuclear rec	protein-coding
family with	protein-coding
PRP40 pre-	protein-coding
torsin fami	protein-coding
deoxycytid	protein-coding
ring finger	protein-coding
diacylglyce	protein-coding
aconitase 1	protein-coding
carbohydra	protein-coding
RIKEN cDN.	protein-coding
carbohydra	protein-coding
multiple C2	protein-coding
small nucle	protein-coding
vav 1 onco	protein-coding
F-box prote	protein-coding
major facili	protein-coding
CASP8 and	protein-coding
somatostat	protein-coding
DNA-dama	protein-coding
Kruppel-lik	protein-coding
heat shock	protein-coding
destrin	protein-coding
dpy-19-like	protein-coding
potassium	protein-coding
protein pho	protein-coding
F11 recept	protein-coding
purinergic	protein-coding
ubiquitin sp	protein-coding
CD86 antig	protein-coding
leucine rich	protein-coding
CCR4-NOT	protein-coding
sulfotransf	protein-coding
ELOVL fam	protein-coding
jumonji, AT	protein-coding
calmodulin	protein-coding
RNA polym	protein-coding
hepatoma-	protein-coding
protein tyr	protein-coding
unc-13 hor	protein-coding
zinc finger,	protein-coding
potassium	protein-coding

protein pho	protein-coding
zinc finger,	protein-coding
WD repeat	protein-coding
Rho guanin	protein-coding
nuclear pro	protein-coding
centrosom	protein-coding
interleukin	protein-coding
solute carri	protein-coding
recombina	protein-coding
homeodom	protein-coding
acyl-Coenz	protein-coding
general tra	protein-coding
contactin a	protein-coding
golgi autoa	protein-coding
family with	protein-coding
homeodom	protein-coding
solute carri	protein-coding
D site albu	protein-coding
meteorin, g	protein-coding
glutathione	protein-coding
stomatin (E	protein-coding
PCI domair	protein-coding
solute carri	protein-coding
ataxin 7-lik	protein-coding
suppressor	protein-coding
rcd1 (requi	protein-coding
N-myc dow	protein-coding
G protein-c	protein-coding
placenta ex	protein-coding
cyclin-depe	protein-coding
S-adenosyl	protein-coding
estrogen-re	protein-coding
family with	protein-coding
fatty acyl C	protein-coding
RUN and FY	protein-coding
solute carri	protein-coding
ELKS/RAB6	protein-coding
MMS19 (M	protein-coding
SAM and SI	protein-coding
Nipped-B h	protein-coding
thyroid stir	protein-coding
protein kin	protein-coding
Ctr9, Paf1/	protein-coding
ATP-bindin	protein-coding
tubulin pol	protein-coding
ATPase, Ca	protein-coding
ankyrin rep	protein-coding

Smg-7 hom	protein-coding
EMG1 nucl	protein-coding
H2A histon	protein-coding
syndecan b	protein-coding
P450 (cytoc	protein-coding
pyroglutam	protein-coding
sine oculis-	protein-coding
ATP syntha	protein-coding
NADH dehy	protein-coding
latent trans	protein-coding
cordon-ble	protein-coding
Sfi1 homol	protein-coding
c-abl oncog	protein-coding
glycine rec	protein-coding
TAP bindin	protein-coding
chemokine	protein-coding
discs, large	protein-coding
centriolin	protein-coding
potassium	protein-coding
lectin, man	protein-coding
TBC1 doma	protein-coding
ATP syntha	protein-coding
G protein-c	protein-coding
sterol O-ac	protein-coding
lin-9 homo	protein-coding
sphingosin	protein-coding
cyclin M2	protein-coding
HSPA (heat	protein-coding
perilipin 3	protein-coding
fatty acid b	protein-coding
zinc finger	protein-coding
solute carri	protein-coding
trichohyali	protein-coding
adiponecti	protein-coding
spermatog	protein-coding
nucleolar a	protein-coding
compleme	protein-coding
ATP syntha	protein-coding
regulatory	protein-coding
thioredoxi	protein-coding
transducin	protein-coding
myosin, ligl	protein-coding
pleckstrin a	protein-coding
zinc finger	protein-coding
componen	protein-coding
5'-3' exorib	protein-coding
receptor (T	protein-coding

aquaporin	protein-coding
gasdermin	protein-coding
membrane	protein-coding
tyrosine 3-	protein-coding
dehydroge	protein-coding
alkB, alkyla	protein-coding
death asso	protein-coding
Ral GEF wit	protein-coding
heat shock	protein-coding
RAS-relate	protein-coding
TBC1 doma	protein-coding
solute carri	protein-coding
lysophosph	protein-coding
TNF recept	protein-coding
glycyl-tRNA	protein-coding
integral me	protein-coding
pleckstrin h	protein-coding
cytidine 5'-	protein-coding
PDX1 C-ter	protein-coding
zinc finger,	protein-coding
interleukin	protein-coding
palmitoyl-p	protein-coding
interferon	protein-coding
tribbles ho	protein-coding
homeobox	protein-coding
disabled 2	protein-coding
syntaphilin	protein-coding
chemokine	protein-coding
protein tyr	protein-coding
fatty acyl C	protein-coding
zinc finger	protein-coding
zinc finger	protein-coding
methyl-CpG	protein-coding
SYS1 Golgi-	protein-coding
ELOVL fam	protein-coding
rhotekin	protein-coding
low density	protein-coding
transmemb	protein-coding
TRAF type	protein-coding
repulsive g	protein-coding
glutaminas	protein-coding
CD47 antig	protein-coding
activating t	protein-coding
FK506 bind	protein-coding
PHD finger	protein-coding
DEAD (Asp-	protein-coding
ras homolo	protein-coding

ribosomal p	protein-coding
lamin B rec	protein-coding
glycophorin	protein-coding
G patch do	protein-coding
orosomucc	protein-coding
RIKEN cDN.	protein-coding
lysine (K)-s	protein-coding
family with	protein-coding
glutamate-	protein-coding
RIKEN cDN.	protein-coding
tyrosine 3-	protein-coding
methyltran	protein-coding
mannosida	protein-coding
abhydrolas	protein-coding
sciellin	protein-coding
ring finger	protein-coding
sulfatase 2	protein-coding
AT rich inte	protein-coding
anoctamin	protein-coding
RAR-relate	protein-coding
ELOVL fam	protein-coding
jumping tra	protein-coding
RELT tumo	protein-coding
syntrophin	protein-coding
cyclin-depe	protein-coding
vacuolar pr	protein-coding
RRS1 ribos	protein-coding
Parkinson d	protein-coding
RIKEN cDN.	protein-coding
lipocalin 4	protein-coding
vav 3 onco	protein-coding
oncostatin	protein-coding
Yip1 domai	protein-coding
mitochond	protein-coding
retinoblast	protein-coding
transmemb	protein-coding
zyg-ll famil	protein-coding
syntaxin bi	protein-coding
kelch-like 7	protein-coding
Rho family	protein-coding
unc-5 hom	protein-coding
a disintegri	protein-coding
WAS/WASI	protein-coding
torsin A int	protein-coding
proteasom	protein-coding
YKT6 homo	protein-coding
toll-like rec	protein-coding

RFad1, flav	protein-coding
forkhead b	protein-coding
trafficking	protein-coding
receptor ac	protein-coding
phosphoglu	protein-coding
MTOR assc	protein-coding
ATP-bindin	protein-coding
potassium	protein-coding
transducin	protein-coding
OTU domai	protein-coding
ARP3 actin	protein-coding
formin-like	protein-coding
G elongatic	protein-coding
transferrin	protein-coding
LIM domain	protein-coding
F-box prote	protein-coding
tec protein	protein-coding
lemur tyros	protein-coding
thymus cel	protein-coding
jagged 1	protein-coding
timeless in	protein-coding
lymphocyte	protein-coding
vascular en	protein-coding
son of seve	protein-coding
zinc finger	protein-coding
calpain 13	protein-coding
phosphatas	protein-coding
Kruppel-lik	protein-coding
ligand of nu	protein-coding
URI1, prefc	protein-coding
succinate c	protein-coding
histone dea	protein-coding
serine (or c	protein-coding
kinase inse	protein-coding
family with	protein-coding
interaction	protein-coding
BEN domai	protein-coding
TBC1 doma	protein-coding
sterol regu	protein-coding
solute carri	protein-coding
FYVE, RhoG	protein-coding
lectin, man	protein-coding
activation-i	protein-coding
APC memb	protein-coding
striatin, cal	protein-coding
peptidyl pr	protein-coding
RIKEN cDN.	protein-coding

UDP-GlcNA	protein-coding
transmemb	protein-coding
methylmal	protein-coding
adiponecti	protein-coding
HECT doma	protein-coding
ribulose-5-	protein-coding
ribosomal	protein-coding
BCL2-assoc	protein-coding
cut-like ho	protein-coding
heterogene	protein-coding
forkhead b	protein-coding
negative re	protein-coding
translin	protein-coding
D-2-hydrox	protein-coding
prominin 1	protein-coding
guanine nu	protein-coding
erythrocyt	protein-coding
zinc finger	protein-coding
xyloside xy	protein-coding
utrophin	protein-coding
BCL2-like 1	protein-coding
tripartite n	protein-coding
solute carr	protein-coding
lysozyme 1	protein-coding
membrane	protein-coding
RIKEN cDN	protein-coding
UDP-gluco	protein-coding
mitochond	protein-coding
prothymos	protein-coding
ARP1 actin	protein-coding
fatty acid b	protein-coding
carbohydra	protein-coding
FBJ osteos	protein-coding
general tra	protein-coding
serine/thre	protein-coding
peptidylpr	protein-coding
nitric oxide	protein-coding
calpain 10	protein-coding
dpy-30 hor	protein-coding
G protein p	protein-coding
sorting nex	protein-coding
BTAF1 RNA	protein-coding
family with	protein-coding
zinc finger	protein-coding
cyclic nucle	protein-coding
BH3 intera	protein-coding
proline-ricl	protein-coding

HOP home	protein-coding
PDZ domai	protein-coding
SH2B adap	protein-coding
leucine rich	protein-coding
T cell activa	protein-coding
heterogene	protein-coding
centrosom	protein-coding
chemokine	protein-coding
F-box and l	protein-coding
mitochond	protein-coding
ARP3 actin	protein-coding
Holliday ju	protein-coding
RIKEN cDN	protein-coding
transportin	protein-coding
ring finger	protein-coding
Friend leuk	protein-coding
interferon	protein-coding
meningiom	protein-coding
sphingosin	protein-coding
expressed	protein-coding
solute carri	protein-coding
lipin 1	protein-coding
myosin reg	protein-coding
neuromedi	protein-coding
spire homo	protein-coding
FK506 bind	protein-coding
RIKEN cDN	protein-coding
RIKEN cDN	protein-coding
transmemb	protein-coding
CART prepr	protein-coding
zinc finger	protein-coding
tripartite m	protein-coding
chondroad	protein-coding
cornichon l	protein-coding
chromatin	protein-coding
translocase	protein-coding
RNA bindin	protein-coding
methyltran	protein-coding
muscleblin	protein-coding
cysteine co	protein-coding
family with	protein-coding
zinc finger	protein-coding
Sp110 nucl	protein-coding
Sh3kbp1 bi	protein-coding
vacuolar pr	protein-coding
NIMA (neve	protein-coding
Kruppel-lik	protein-coding

PHD finger	protein-coding
translation	protein-coding
serine/argi	protein-coding
AU RNA bir	protein-coding
Rho guanir	protein-coding
3-phospho	protein-coding
origin reco	protein-coding
cullin 2	protein-coding
ubiquitin-c	protein-coding
DNA-dama	protein-coding
ubiquitin C	protein-coding
sphingomy	protein-coding
glucose-fru	protein-coding
ADP-ribosy	protein-coding
family with	protein-coding
ring finger	protein-coding
myotrophi	protein-coding
solute carri	protein-coding
cullin assoc	protein-coding
leucine rich	protein-coding
RAB3C, me	protein-coding
trinucleotic	protein-coding
PDZ domai	protein-coding
vesicle-ass	protein-coding
kelch-like 2	protein-coding
mitogen-ac	protein-coding
lysophosph	protein-coding
neuregulin	protein-coding
solute carri	protein-coding
autocrine r	protein-coding
RAS-relate	protein-coding
anillin, acti	protein-coding
formin 1	protein-coding
prune hom	protein-coding
breast canc	protein-coding
baculoviral	protein-coding
EH-domain	protein-coding
cleavage ar	protein-coding
tRNA-yW s	protein-coding
notch 2	protein-coding
germinal ce	protein-coding
biotinidase	protein-coding
SLIT-ROBO	protein-coding
KDEL (Lys-A	protein-coding
dipeptidylp	protein-coding
family with	protein-coding
sema doma	protein-coding

striatin, cal	protein-coding
coiled-coil	protein-coding
enolase 1B	protein-coding
ubiquilin 1	protein-coding
ankyrin rep	protein-coding
ryanodine	protein-coding
centrosom	protein-coding
male-speci	protein-coding
solute carri	protein-coding
methylthio	protein-coding
ArfGAP wit	protein-coding
nuclear rec	protein-coding
ilvB (bacter	protein-coding
family with	protein-coding
ATP-bindin	protein-coding
biotinidase	protein-coding
eukaryotic	protein-coding
RIKEN cDN	protein-coding
WD repeat	protein-coding
RIKEN cDN	protein-coding
ArfGAP wit	protein-coding
proline-rich	protein-coding
annexin A9	protein-coding
glutamate	protein-coding
p21 protein	protein-coding
WD repeat	protein-coding
NOL1/NOP	protein-coding
Coenzyme	protein-coding
RIKEN cDN	protein-coding
kelch doma	protein-coding
nuclear rec	protein-coding
single-stran	protein-coding
ring finger	protein-coding
crystallin, g	protein-coding
inhibitor of	protein-coding
RIKEN cDN	protein-coding
mitochond	protein-coding
Fc receptor	protein-coding
glutamate	protein-coding
multiple EG	protein-coding
COP9 (cons	protein-coding
PAXIP1 ass	protein-coding
histone dea	protein-coding
ArfGAP wit	protein-coding
F-box and W	protein-coding
1-acylglyce	protein-coding
solute carri	protein-coding

tRNA splici	protein-coding
family with	protein-coding
family with	protein-coding
diazepam b	protein-coding
peroxisom	protein-coding
tRNA selen	protein-coding
Rho guanin	protein-coding
NIMA (nev	protein-coding
RasGEF do	protein-coding
FAST kinas	protein-coding
F-box prot	protein-coding
multivesicu	protein-coding
wings apar	protein-coding
mitochond	protein-coding
cyclin-depe	protein-coding
myocyte er	protein-coding
3-ketodihy	protein-coding
DTW doma	protein-coding
cyclin O	protein-coding
optineurin	protein-coding
endoglin	protein-coding
solute carri	protein-coding
arylsulfata	protein-coding
signal sequ	protein-coding
caspase 7	protein-coding
mitochond	protein-coding
cylicin, bas	protein-coding
leucine rich	protein-coding
cell divisio	protein-coding
coiled-coil	protein-coding
aspartyl-tR	protein-coding
phosphose	protein-coding
sprouty ho	protein-coding
PARK2 co-r	protein-coding
ring finger	protein-coding
hyaluronar	protein-coding
methionine	protein-coding
ring finger	protein-coding
histone clu	protein-coding
activating t	protein-coding
vascular er	protein-coding
nucleopori	protein-coding
programm	protein-coding
branched c	protein-coding
ribosomal	protein-coding
tripartite n	protein-coding
poly (A) po	protein-coding

double hor	protein-coding
RING1 and	protein-coding
H1 histone	protein-coding
growth fac	protein-coding
presenilin 2	protein-coding
microsoma	protein-coding
pleckstrin h	protein-coding
ring finger	protein-coding
ankyrin rep	protein-coding
solute carri	protein-coding
SAFB-like, t	protein-coding
abhydrolas	protein-coding
zinc finger	protein-coding
basic leucir	protein-coding
cell cycle a:	protein-coding
proteasom	protein-coding
F-box and V	protein-coding
resistin	protein-coding
Kruppel-lik	protein-coding
transcriptio	protein-coding
leukotriene	protein-coding
cell divisior	protein-coding
family with	protein-coding
TBC1 doma	protein-coding
SIK family k	protein-coding
heterogene	protein-coding
zinc finger	protein-coding
doublecort	protein-coding
solute carri	protein-coding
phosphoryl	protein-coding
IQ motif co	protein-coding
muscleblin	protein-coding
Rho-associa	protein-coding
Y box prote	protein-coding
laminin, be	protein-coding
exocyst cor	protein-coding
family with	protein-coding
KAT8 regul	protein-coding
CCAAT/enh	protein-coding
primase an	protein-coding
ras homolo	protein-coding
family with	protein-coding
antizyme ir	protein-coding
carnitine d	protein-coding
INTS3 and I	protein-coding
polyhomeo	protein-coding
galactosylc	protein-coding

LFNG O-fuc	protein-coding
ubiquitin-c	protein-coding
haloacid de	protein-coding
zinc finger	protein-coding
nitric oxide	protein-coding
sorting nex	protein-coding
fibronectin	protein-coding
ring finger	protein-coding
phosphatic	protein-coding
solute carri	protein-coding
COP9 (cons	protein-coding
acylpeptide	protein-coding
heat shock	protein-coding
leucine rich	protein-coding
REM2 and	protein-coding
leukocyte s	protein-coding
histone clu	protein-coding
serine/thre	protein-coding
transmemb	protein-coding
DNA prima	protein-coding
chitinase d	protein-coding
FYN bindin	protein-coding
GTP bindin	protein-coding
migration a	protein-coding
dystrophia	protein-coding
glucosamir	protein-coding
methyltran	protein-coding
ubiquitin-c	protein-coding
RIKEN cDN	protein-coding
DCP1 decap	protein-coding
attractin	protein-coding
predicted g	protein-coding
NIMA (neve	protein-coding
COP9 (cons	protein-coding
protease-a	protein-coding
sodium cha	protein-coding
NEDD4 bin	protein-coding
signal trans	protein-coding
replication	protein-coding
TRAF-inter	protein-coding
phosphatic	protein-coding
nuclear fra	protein-coding
prolyl 4-hy	protein-coding
basic trans	protein-coding
prion prote	protein-coding
AT rich inte	protein-coding
charged mu	protein-coding

bromodom	protein-coding
tripartite m	protein-coding
pantothena	protein-coding
Sp2 transcr	protein-coding
transmemb	protein-coding
GA repeat	protein-coding
cysteine ar	protein-coding
RNA bindin	protein-coding
SKI-like	protein-coding
chromodor	protein-coding
retinol deh	protein-coding
v-myc mye	protein-coding
cyclin D bir	protein-coding
family with	protein-coding
transmemb	protein-coding
mitochond	protein-coding
superoxide	protein-coding
mast cell ir	protein-coding
low density	protein-coding
zinc finger,	protein-coding
ubiquitin s	protein-coding
Casitas B-li	protein-coding
leucine zip	protein-coding
ets variant	protein-coding
CCR4-NOT	protein-coding
protein tyr	protein-coding
ubiquitin d	protein-coding
nitrilase fa	protein-coding
RCSD doma	protein-coding
SMAD fami	protein-coding
solute carri	protein-coding
c-src tyrosi	protein-coding
queuine tR	protein-coding
predicted g	protein-coding
adhesion m	protein-coding
kelch doma	protein-coding
major facili	protein-coding
interactor c	protein-coding
protein kin	protein-coding
CD97 antig	protein-coding
solute carri	protein-coding
inhibitor of	protein-coding
trinucleotic	protein-coding
solute carri	protein-coding
guanylate b	protein-coding
pleckstrin h	protein-coding
RIKEN cDN.	protein-coding

CCR4 carbo	protein-coding
transcriptio	protein-coding
small nucle	protein-coding
myeloma o	protein-coding
SDA1 doma	protein-coding
mannosida	protein-coding
casein kina	protein-coding
glutaredox	protein-coding
anaphase p	protein-coding
furin (paire	protein-coding
neuropilin	protein-coding
SMAD fami	protein-coding
ADP-ribosy	protein-coding
maturin, ne	protein-coding
coiled-coil	protein-coding
vacuolar pr	protein-coding
mitogen-ac	protein-coding
protein ger	protein-coding
coiled-coil	protein-coding
calmodulin	protein-coding
androglobi	protein-coding
calcyclin bi	protein-coding
Scm-like wi	protein-coding
calcium/ca	protein-coding
monoglyce	protein-coding
olfactory re	protein-coding
eukaryotic	protein-coding
mitochond	protein-coding
C-type lect	protein-coding
AT rich inte	protein-coding
hydroxyste	protein-coding
annexin A3	protein-coding
tumor necr	protein-coding
fuzzy homo	protein-coding
integrator	protein-coding
phosphatic	protein-coding
bone morp	protein-coding
ring finger	protein-coding
LYR motif c	protein-coding
cholinergic	protein-coding
protein pho	protein-coding
suppressor	protein-coding
farnesyl dip	protein-coding
synemin, in	protein-coding
zinc finger	protein-coding
RuvB-like p	protein-coding
isocitrate d	protein-coding

Morf4 fami	protein-coding
phosphorib	protein-coding
gap junctio	protein-coding
cyclin-depe	protein-coding
Ras associa	protein-coding
glutamate	protein-coding
CKLF-like M	protein-coding
diphosphoi	protein-coding
survival mo	protein-coding
cysteinyl le	protein-coding
ribonucleas	protein-coding
transducer	protein-coding
sorting nex	protein-coding
PAK1 intera	protein-coding
transcriptio	protein-coding
zinc finger	protein-coding
G protein-c	protein-coding
growth arre	protein-coding
fibronectin	protein-coding
zinc finger,	protein-coding
receptor ac	protein-coding
feline sarco	protein-coding
lipase, end	protein-coding
butyrophili	protein-coding
F-box and l	protein-coding
prostagland	protein-coding
RIMS bindi	protein-coding
plectin	protein-coding
DDHD dom	protein-coding
phosphatic	protein-coding
transmemb	protein-coding
nucleic acic	protein-coding
zinc finger	protein-coding
regulatory	protein-coding
ribosomal p	protein-coding
X-prolyl am	protein-coding
solute carri	protein-coding
ribosomal p	protein-coding
phosphatas	protein-coding
F-box prote	protein-coding
sortilin-rela	protein-coding
myeloblast	protein-coding
eukaryotic	protein-coding
opioid grov	protein-coding
DIRAS fami	protein-coding
phosphofru	protein-coding
cadherin 2(	protein-coding

ubiquitin s|	protein-coding
ubiquinol c	protein-coding
neurexoph	protein-coding
delta/notcl	protein-coding
coiled-coil	protein-coding
MORN rep	protein-coding
Abelson he	protein-coding
cytochrom	protein-coding
NHL repeat	protein-coding
vesicle-ass	protein-coding
GA repeat	protein-coding
topoisome	protein-coding
TRAF3 inte	protein-coding
solute carri	protein-coding
lectin, gala	protein-coding
tubby cand	protein-coding
vacuolar pr	protein-coding
solute carri	protein-coding
COX assem	protein-coding
leucine-ricl	protein-coding
centrosom	protein-coding
5'-nucleoti	protein-coding
zinc finger,	protein-coding
DEAD (Asp-	protein-coding
prostaglan	protein-coding
transmemb	protein-coding
serine (or c	protein-coding
proteasom	protein-coding
mitochond	protein-coding
signal-indu	protein-coding
adenosine	protein-coding
ribonuclea:	protein-coding
killer cell le	protein-coding
stem-loop	protein-coding
COMM dor	protein-coding
glutathione	protein-coding
cadherin 6	protein-coding
ubiquitin s|	protein-coding
coactosin-l	protein-coding
cyclin-depe	protein-coding
RIKEN cDN.	protein-coding
La ribonucl	protein-coding
sphingosin	protein-coding
ORAI calciu	protein-coding
RIKEN cDN.	protein-coding
Kv channel	protein-coding
epithelial n	protein-coding

CD47 antig	protein-coding
toll-like rec	protein-coding
stanniocalc	protein-coding
ribosomal	protein-coding
ARP6 actin-	protein-coding
serine/thre	protein-coding
N(alpha)-ac	protein-coding
cleavage ar	protein-coding
frizzled hor	protein-coding
neural cell	protein-coding
carboxypep	protein-coding
golgi assoc	protein-coding
biogenesis	protein-coding
cysteinyl le	protein-coding
RAB27A, m	protein-coding
septin 14	protein-coding
ring finger	protein-coding
two pore c	protein-coding
DEAD (Asp-	protein-coding
translocase	protein-coding
serine/thre	protein-coding
ankyrin rep	protein-coding
syntaxin 12	protein-coding
nuclear rec	protein-coding
casein kina	protein-coding
male-speci	protein-coding
digestive o	protein-coding
structural r	protein-coding
CASP8 and	protein-coding
family with	protein-coding
WW domai	protein-coding
melanoreg	protein-coding
Fanconi an	protein-coding
aarF domai	protein-coding
selenoprot	protein-coding
ribosomal	protein-coding
Ssu72 RNA	protein-coding
kinesin fan	protein-coding
RAB31, me	protein-coding
tetraspanir	protein-coding
excision re	protein-coding
RIKEN cDN.	protein-coding
signal pept	protein-coding
phosphoin	protein-coding
DDB1 and (	protein-coding
fucosyltran	protein-coding
zinc finger	protein-coding

speedy hor	protein-coding
RIKEN cDN.	protein-coding
matrix met	protein-coding
sorting nex	protein-coding
selenoprot	protein-coding
avian musc	protein-coding
zinc finger	protein-coding
E74-like fac	protein-coding
phosphoin	protein-coding
atrophin 1	protein-coding
histocompa	protein-coding
mitochond	protein-coding
kinesin fam	protein-coding
patatin-like	protein-coding
microtubul	protein-coding
acyl-Coenz	protein-coding
cadherin 1:	protein-coding
sema doma	protein-coding
RecQ prote	protein-coding
family with	protein-coding
Rap1 GTPa	protein-coding
Sad1 and U	protein-coding
aldehyde d	protein-coding
Yamaguchi	protein-coding
RIKEN cDN.	protein-coding
ligand depe	protein-coding
cysteine-se	protein-coding
MSS51 mit	protein-coding
catenin (ca	protein-coding
RCE1 homc	protein-coding
phosphatas	protein-coding
UDP-Gal:be	protein-coding
clavesin 1	protein-coding
RAB20, me	protein-coding
carnitine p	protein-coding
Rho guanir	protein-coding
La ribonucl	protein-coding
shisa homc	protein-coding
SKI family t	protein-coding
G-protein c	protein-coding
CDC14 cell	protein-coding
DEAD (Asp-	protein-coding
BCL2 bindi	protein-coding
euchromat	protein-coding
coiled-coil	protein-coding
DSN1, MIN	protein-coding
tyrosine 3-	protein-coding

RAD51 hon	protein-coding
thioesteras	protein-coding
tripartite m	protein-coding
transmemb	protein-coding
RAD17 hon	protein-coding
collagen, ty	protein-coding
mitochond	protein-coding
heterogene	protein-coding
transducin	protein-coding
F-box and V	protein-coding
thrombosp	protein-coding
Aly/REF exp	protein-coding
lysosomal-a	protein-coding
insulin-like	protein-coding
KH-type sp	protein-coding
transforma	protein-coding
proviral int	protein-coding
glycerol kir	protein-coding
C2 calcium	protein-coding
myelin pro	protein-coding
olfactomed	protein-coding
matrix met	protein-coding
antigen ide	protein-coding
programme	protein-coding
Vac14 hom	protein-coding
TGFB-induc	protein-coding
tight juncti	protein-coding
forkhead b	protein-coding
olfactory re	protein-coding
Kruppel-lik	protein-coding
acid phosp	protein-coding
ring finger	protein-coding
ADP-ribose	protein-coding
cyclin-depe	protein-coding
tumor necr	protein-coding
CDC42 effe	protein-coding
SH2 domai	protein-coding
SH3-domai	protein-coding
deafness, a	protein-coding
SFT2 doma	protein-coding
patatin-like	protein-coding
grancalcin	protein-coding
transmemb	protein-coding
transformi	protein-coding
villin 1	protein-coding
TM2 doma	protein-coding
TRAF family	protein-coding

Fc receptoı	protein-coding
predicted g	protein-coding
zinc finger	protein-coding
solute carri	protein-coding
plasminoge	protein-coding
collagen, ty	protein-coding
thymopoie	protein-coding
dehydroge	protein-coding
transmemk	protein-coding
ubiquinol-c	protein-coding
dedicator c	protein-coding
lymphotox	protein-coding
bromodom	protein-coding
neuroligin :	protein-coding
G protein-c	protein-coding
prolyl 4-hy	protein-coding
acetylcholi	protein-coding
mitochond	protein-coding
solute carri	protein-coding
glucoside x	protein-coding
MAP/micrc	protein-coding
ankyrin rep	protein-coding
lipoyltransf	protein-coding
PET112 hoı	protein-coding
SEH1-like (!	protein-coding
elongator a	protein-coding
mitochond	protein-coding
proline anc	protein-coding
glycoprotei	protein-coding
solute carri	protein-coding
guanine nu	protein-coding
tet methylc	protein-coding
lymphoblas	protein-coding
FYVE, RhoG	protein-coding
FBJ osteosa	protein-coding
pleckstrin h	protein-coding
chimerin 2	protein-coding
v-maf musc	protein-coding
adhesion m	protein-coding
sorting nex	protein-coding
ectonucleo	protein-coding
processing	protein-coding
fibulin 2	protein-coding
stefin A3	protein-coding
interleukin	protein-coding
chaperonir	protein-coding
protein phc	protein-coding

progestin a	protein-coding
WD repeat	protein-coding
diacylglyce	protein-coding
adrenergic	protein-coding
glutathione	protein-coding
SEC22 vesi	protein-coding
glyoxylate	protein-coding
molybdenu	protein-coding
spleen tyrc	protein-coding
regulatory	protein-coding
RAD21 hon	protein-coding
triosephos	protein-coding
methyltran	protein-coding
chromobox	protein-coding
mitogen-ac	protein-coding
MORN repe	protein-coding
GDP-mann	protein-coding
mitogen-ac	protein-coding
solute carri	protein-coding
TAO kinase	protein-coding
RIKEN cDN	protein-coding
prostaglan	protein-coding
pre B cell le	protein-coding
neutral sph	protein-coding
musculoske	protein-coding
early grow	protein-coding
histone dea	protein-coding
protein kin	protein-coding
LSM12 hon	protein-coding
RIKEN cDN	protein-coding
zinc finger,	protein-coding
ubiquitin s	protein-coding
grancalcin	protein-coding
TATA box b	protein-coding
ATPase, Ca	protein-coding
inhibitor of	protein-coding
nucleolin	protein-coding
PAP associa	protein-coding
tumor necr	protein-coding
BMP2 indu	protein-coding
katanin p6	protein-coding
atlastin GT	protein-coding
cold shock	protein-coding
family with	protein-coding
GTPase act	protein-coding
retinoic aci	protein-coding
zinc finger,	protein-coding

branched c	protein-coding
RMI2, RecC	protein-coding
transformi	protein-coding
cornifelin	protein-coding
DIP2 disco-	protein-coding
deiodinase	protein-coding
neural prec	protein-coding
death indu	protein-coding
1-acylglyce	protein-coding
inositol (m	protein-coding
RIKEN cDN	protein-coding
RAS-relate	protein-coding
phosphofu	protein-coding
polo-like ki	protein-coding
FCH domai	protein-coding
kelch-like 2	protein-coding
zinc finger	protein-coding
Yamaguchi	protein-coding
protein-L-is	protein-coding
tektin 3	protein-coding
nuclear tra	protein-coding
sterile alph	protein-coding
neutrophil	protein-coding
de-etiolate	protein-coding
Yae1 doma	protein-coding
protein pho	protein-coding
RIKEN cDN	protein-coding
paired-Ig-li	protein-coding
transmemb	protein-coding
acyl-Coenz	protein-coding
anthrax tox	protein-coding
SLIT and N	protein-coding
hypermeth	protein-coding
pre-mRNA	protein-coding
mesoderm	protein-coding
regulator o	protein-coding
cerebral ca	protein-coding
protein arg	protein-coding
carbonic ar	protein-coding
ATPase, Na	protein-coding
PML-RAR a	protein-coding
ubiquitin-li	protein-coding
RIKEN cDN	protein-coding
ubiquitin-fo	protein-coding
Max intera	protein-coding
nuclear ant	protein-coding
cytosolic th	protein-coding

intersectin	protein-coding
glycerol kir	protein-coding
limb and n	protein-coding
glutamine	protein-coding
leucine rich	protein-coding
nucleopori	protein-coding
C-type lect	protein-coding
ATPase, Cu	protein-coding
exocyst cor	protein-coding
carbohydra	protein-coding
N-acetylglu	protein-coding
lysine (K)-s	protein-coding
mitogen-ac	protein-coding
ATP-bindin	protein-coding
WD repeat	protein-coding
ribonuclea	protein-coding
lymphocyt	protein-coding
C-type lect	protein-coding
transmemb	protein-coding
Kruppel-lik	protein-coding
predicted g	protein-coding
5' nucleoti	protein-coding
ARP5 actin	protein-coding
neuron nav	protein-coding
plexin B3	protein-coding
importin 7	protein-coding
transmemb	protein-coding
nuclear fac	protein-coding
secretory c	protein-coding
DEAH (Asp	protein-coding
lipoma HM	protein-coding
HEAT repea	protein-coding
peptidylpr	protein-coding
carbohydra	protein-coding
ubiquitin s	protein-coding
3'-phospho	protein-coding
CDC42 effe	protein-coding
Holliday ju	protein-coding
cAMP resp	protein-coding
ring finger	protein-coding
AT hook, D	protein-coding
plasminog	protein-coding
G1 to S pha	protein-coding
dual-specif	protein-coding
ATPase typ	protein-coding
forkhead b	protein-coding
EH domain	protein-coding

TNF recept	protein-coding
peroxisom	protein-coding
zinc finger	protein-coding
succinate d	protein-coding
neutral chc	protein-coding
trans-actin	protein-coding
dual-specif	protein-coding
RIKEN cDN	protein-coding
serine (or c	protein-coding
NADH dehy	protein-coding
bone marr	protein-coding
biogenesis	protein-coding
rosbin, rou	protein-coding
ankyrin rep	protein-coding
phosphodi	protein-coding
RAN bindin	protein-coding
protogenin	protein-coding
fucosyltran	protein-coding
Rap guanin	protein-coding
YdjC homo	protein-coding
unc-79 hor	protein-coding
Janus kinas	protein-coding
zinc finger	protein-coding
platelet/en	protein-coding
chloride int	protein-coding
heterogen	protein-coding
melanoma	protein-coding
ligase III, D	protein-coding
rosbin, rou	protein-coding
TAF3 RNA	protein-coding
N-myc (anc	protein-coding
zinc finger	protein-coding
serine/argi	protein-coding
G protein-c	protein-coding
solute carr	protein-coding
ribosomal	protein-coding
Erbb2 inter	protein-coding
RIKEN cDN	protein-coding
ADP-ribosy	protein-coding
fos-like ant	protein-coding
leucine rich	protein-coding
tensin like	protein-coding
UDP-N-ace	protein-coding
pericentrin	protein-coding
protein ph	protein-coding
protein ph	protein-coding
caspase 8	protein-coding

guanine nu	protein-coding
engulfmen	protein-coding
sphingomy	protein-coding
HORMA do	protein-coding
importin 9	protein-coding
outer dens	protein-coding
coiled-coil	protein-coding
dynein, axo	protein-coding
Rho guanin	protein-coding
PAP associ	protein-coding
ribulose-5-	protein-coding
adaptor-re	protein-coding
SRY (sex de	protein-coding
activin rece	protein-coding
nuclear rec	protein-coding
hydroxyste	protein-coding
arachidona	protein-coding
mitofusin 2	protein-coding
solute carri	protein-coding
IscU iron-su	protein-coding
profilin 1	protein-coding
B cell trans	protein-coding
microtubul	protein-coding
atlastin GT	protein-coding
MARVEL (m	protein-coding
short statu	protein-coding
chemokine	protein-coding
paired-like	protein-coding
nuclear po	protein-coding
zinc finger	protein-coding
active BCR-	protein-coding
CDP-diacylg	protein-coding
signal trans	protein-coding
ring finger	protein-coding
S100 calciu	protein-coding
small integ	protein-coding
RIKEN cDNA	protein-coding
family with	protein-coding
transmemb	protein-coding
REV1 homo	protein-coding
tuftelin inte	protein-coding
neugrin, ne	protein-coding
phenylalan	protein-coding
ubiquitin-c	protein-coding
ADP-ribosy	protein-coding
glucocortic	protein-coding
ATPase, H+	protein-coding

DEAH (Asp	protein-coding
Alport sync	protein-coding
zinc finger	protein-coding
RUN and F	protein-coding
polypyrimi	protein-coding
mannosida	protein-coding
tetratricop	protein-coding
schwannor	protein-coding
SLAIN moti	protein-coding
MON1 hon	protein-coding
peroxiredo	protein-coding
dual specif	protein-coding
vestigial lik	protein-coding
transmemb	protein-coding
non-SMC e	protein-coding
phosphory	protein-coding
heparan su	protein-coding
expressed	protein-coding
ribosomal	protein-coding
pleckstrin	protein-coding
apolipopro	protein-coding
zinc finger	protein-coding
acyl-CoA sy	protein-coding
chemokine	protein-coding
carboxypep	protein-coding
B cell CLL/l	protein-coding
BCL6 intera	protein-coding
XRCC6 binc	protein-coding
DnaJ (Hsp4	protein-coding
zinc finger,	protein-coding
coiled-coil	protein-coding
poly(A) bin	protein-coding
membrane	protein-coding
stefin A2	protein-coding
DPH1 hom	protein-coding
nuclear rec	protein-coding
GATA bindi	protein-coding
syntaxin 11	protein-coding
transmemb	protein-coding
TAF11 RNA	protein-coding
dopey fami	protein-coding
hypocretin	protein-coding
centromer	protein-coding
family with	protein-coding
heparan su	protein-coding
COP9 (cons	protein-coding
proteoglyc	protein-coding

tec protein	protein-coding
chemokine	protein-coding
interferon ;	protein-coding
proline-ser	protein-coding
TP53 regul:	protein-coding
RIKEN cDN.	protein-coding
meiotic nu	protein-coding
gamma-glu	protein-coding
tripartite m	protein-coding
malonyl Co	protein-coding
lymphocyt	protein-coding
small integ	protein-coding
Eph recept	protein-coding
zinc finger	protein-coding
histocomp	protein-coding
proteoglyc	protein-coding
oxidative s	protein-coding
chemokine	protein-coding
abl-interac	protein-coding
adaptor-re	protein-coding
thromboxa	protein-coding
sphingosin	protein-coding
cytidine m	protein-coding
neuronal ty	protein-coding
Max dimer	protein-coding
PTK2 prote	protein-coding
aminoacyl	protein-coding
round sper	protein-coding
protein ph	protein-coding
rhotekin 2	protein-coding
RIKEN cDN.	protein-coding
actin, alph	protein-coding
CDP-diacyl	protein-coding
LIM homec	protein-coding
hairy/enha	protein-coding
RIKEN cDN.	protein-coding
SET domair	protein-coding
SMAD fami	protein-coding
prostaglan	protein-coding
A kinase (P	protein-coding
ADP-ribosy	protein-coding
protein O-f	protein-coding
2-phospho:	protein-coding
solute carri	protein-coding
WD repeat	protein-coding
vacuolar pı	protein-coding
transmemk	protein-coding

SAP30-like	protein-coding
centrosom	protein-coding
ATPase, H+	protein-coding
mitochond	protein-coding
protein pho	protein-coding
low density	protein-coding
wingless-ty	protein-coding
growth fac	protein-coding
cyclin-depe	protein-coding
myelin pro	protein-coding
SWI5 depe	protein-coding
tyrosyl-tRN	protein-coding
zinc finger	protein-coding
transcriptio	protein-coding
ATPase, Na	protein-coding
cysteine ar	protein-coding
zinc finger	protein-coding
StAR-relate	protein-coding
cytokine re	protein-coding
actin relate	protein-coding
glutaminas	protein-coding
Src homolo	protein-coding
solute carri	protein-coding
phospholip	protein-coding
TBC1 doma	protein-coding
aminopept	protein-coding
islet cell au	protein-coding
Rho guanin	protein-coding
insulin rece	protein-coding
low density	protein-coding
ATPase, Ca	protein-coding
angel hom	protein-coding
UDP-GalNA	protein-coding
RIKEN cDN	protein-coding
TAF15 RNA	protein-coding
protein O-l	protein-coding
DNA segme	protein-coding
abhydrolas	protein-coding
mannose r	protein-coding
peroxisoma	protein-coding
cell divisior	protein-coding
growth arr	protein-coding
chymotryp	protein-coding
multiple C2	protein-coding
MICAL-like	protein-coding
necdin-like	protein-coding
poliovirus r	protein-coding

tRNA splici	protein-coding
zinc finger	protein-coding
RIKEN cDN.	protein-coding
thioredoxir	protein-coding
CCAAT/enh	protein-coding
homeobox	protein-coding
renalase, F.	protein-coding
PIN2/TERF:	protein-coding
pyrroline-5	protein-coding
esterase D,	protein-coding
tubulin tyr	protein-coding
caspase ac	protein-coding
regulator o	protein-coding
interferon	protein-coding
inosine 5'-|	protein-coding
tudor dom	protein-coding
DIRAS fami	protein-coding
solute carri	protein-coding
peroxisom	protein-coding
zinc finger,	protein-coding
homeodon	protein-coding
ST6 (alpha-	protein-coding
proteaseor	protein-coding
RIKEN cDN.	protein-coding
RIKEN cDN.	protein-coding
nucleolin	protein-coding
lin-7 homo	protein-coding
dynein, axc	protein-coding
ankyrin rep	protein-coding
sprouty-rel	protein-coding
myeloid ce	protein-coding
hydroxyacy	protein-coding
DEAD (Asp-	protein-coding
UDP-N-ace	protein-coding
Aly/REF ex	protein-coding
deafness, a	protein-coding
pyroglutar	protein-coding
ankyrin rep	protein-coding
TBC1 doma	protein-coding
karyopheri	protein-coding
enhancer c	protein-coding
coiled-coil-	protein-coding
caspase re	protein-coding
sulfotransf	protein-coding
regulator o	protein-coding
ras respons	protein-coding
cholinergic	protein-coding

sorting nex	protein-coding
Iroquois re	protein-coding
outer dens	protein-coding
peptidase i	protein-coding
regulatory	protein-coding
Ras associa	protein-coding
glucosamir	protein-coding
MAP/micrc	protein-coding
vav 1 oncoţ	protein-coding
brain expre	protein-coding
DNA-dama	protein-coding
transmeml	protein-coding
mitogen-ac	protein-coding
sterol O-ac	protein-coding
inositol mc	protein-coding
A kinase (P	protein-coding
selenoprot	protein-coding
pleckstrin I	protein-coding
kinase sup	protein-coding
PR domain	protein-coding
expressed :	protein-coding
5'-3' exorib	protein-coding
olfactory re	protein-coding
vacuolar pı	protein-coding
sortilin-rela	protein-coding
2-aminoetl	protein-coding
polymerase	protein-coding
dehydroge	protein-coding
adaptor-re	protein-coding
colony stin	protein-coding
MMS22-lik	protein-coding
cell divisior	protein-coding
tensin 3	protein-coding
poly (ADP-ı	protein-coding
adaptor-re	protein-coding
matrix met	protein-coding
SEC23A (S.	protein-coding
Era (G-prot	protein-coding
trans-actin	protein-coding
chromodor	protein-coding
anaphase-ı	protein-coding
adenylosuc	protein-coding
olfactory re	protein-coding
UDP-GlcNA	protein-coding
ribosome ţ	protein-coding
TNF recept	protein-coding
platelet de	protein-coding

vacuolar pr	protein-coding
inositol 1,4	protein-coding
Nipped-B h	protein-coding
WW domai	protein-coding
ribosomal p	protein-coding
NIN1/RPN1	protein-coding
brain and r	protein-coding
zinc finger,	protein-coding
stonin 2	protein-coding
zinc finger	protein-coding
oxysterol b	protein-coding
nitrilase 1	protein-coding
ubiquitin C	protein-coding
ras respons	protein-coding
transmemb	protein-coding
glutamate-	protein-coding
LIM domai	protein-coding
chromodon	protein-coding
tetratricop	protein-coding
dihydrolipo	protein-coding
suppressio	protein-coding
Rho GTPase	protein-coding
Cbp/p300-i	protein-coding
gasdermin	protein-coding
ribonucleas	protein-coding
colony stim	protein-coding
ferredoxin-	protein-coding
potassium	protein-coding
cyclin L2	protein-coding
peroxisoma	protein-coding
carbohydra	protein-coding
laminin, ga	protein-coding
centrosom	protein-coding
DNA-dama	protein-coding
myosin, ligl	protein-coding
forkhead b	protein-coding
unc-13 hor	protein-coding
G-protein c	protein-coding
pecanex-lik	protein-coding
transmemb	protein-coding
baculoviral	protein-coding
UbiA preny	protein-coding
pleiomorph	protein-coding
small nucle	protein-coding
ribonucleas	protein-coding
UBX domai	protein-coding
deoxynucle	protein-coding

lipoma HM	protein-coding
phosphatic	protein-coding
microsoma	protein-coding
SMAD spec	protein-coding
acyl-Coenz	protein-coding
solute carri	protein-coding
signal reco	protein-coding
ubiquitin-c	protein-coding
ATPase, am	protein-coding
cDNA sequ	protein-coding
ubinuclein	protein-coding
RIKEN cDN	protein-coding
ankyrin rep	protein-coding
mitogen-ac	protein-coding
sigma non-	protein-coding
phosphatic	protein-coding
nudix (nucl	protein-coding
mannosida	protein-coding
translocase	protein-coding
coiled-coil	protein-coding
growth fac	protein-coding
phospholip	protein-coding
kinesin fam	protein-coding
family with	protein-coding
RAR-relate	protein-coding
predicted g	protein-coding
aspartyl-tR	protein-coding
RNA bindin	protein-coding
potassium	protein-coding
plexin dom	protein-coding
aurora kina	protein-coding
poliovirus r	protein-coding
mitochond	protein-coding
copine III	protein-coding
TBC1 doma	protein-coding
LIM domai	protein-coding
REX1, RNA	protein-coding
paired box	protein-coding
SAR1 gene	protein-coding
RIKEN cDN	protein-coding
pleckstrin h	protein-coding
regulator o	protein-coding
H2A histon	protein-coding
CDC42 sma	protein-coding
methyltran	protein-coding
amyotroph	protein-coding
paraspeckl	protein-coding

E26 avian l	protein-coding
Bloom sync	protein-coding
myosin, ligl	protein-coding
acyl-Coenz	protein-coding
MAP kinase	protein-coding
transmemb	protein-coding
chloride ch	protein-coding
ribosomal I	protein-coding
cell divisior	protein-coding
family with	protein-coding
predicted g	protein-coding
MAX gene	protein-coding
cytochrom	protein-coding
transducin-	protein-coding
ring finger	protein-coding
family with	protein-coding
estrogen re	protein-coding
interleukin	protein-coding
transferrin	protein-coding
zinc finger,	protein-coding
tRNA meth	protein-coding
KDEL (Lys-A	protein-coding
cyclin-depe	protein-coding
stromal int	protein-coding
myeloid/ly	protein-coding
ER membra	protein-coding
ras respons	protein-coding
coiled-coil	protein-coding
pyroglutam	protein-coding
activating t	protein-coding
methylene	protein-coding
peroxisoma	protein-coding
SET and MY	protein-coding
SH2B adap	protein-coding
regulatory	protein-coding
polymerase	protein-coding
peptidase (	protein-coding
TRAF3 inte	protein-coding
myristoylat	protein-coding
golgi meml	protein-coding
ARP3 actin-	protein-coding
cyclin-depe	protein-coding
multiple EC	protein-coding
Ly6/Plaur c	protein-coding
solute carri	protein-coding
transmemb	protein-coding
shugoshin-	protein-coding

transforma	protein-coding
RIKEN cDN.	protein-coding
phosphatic	protein-coding
zinc finger	protein-coding
trophinin a	protein-coding
leucine rich	protein-coding
kizuna cent	protein-coding
FCH and dc	protein-coding
integrator (	protein-coding
polymerase	protein-coding
AT rich inte	protein-coding
peroxisoma	protein-coding
PHD finger	protein-coding
trypsin 4	protein-coding
nescient he	protein-coding
DNA segme	protein-coding
spindle ass	protein-coding
chondroitir	protein-coding
solute carri	protein-coding
SWI/SNF-re	protein-coding
elongation	protein-coding
RAB, meml	protein-coding
BTB (POZ) (	protein-coding
componen	protein-coding
phosphatic	protein-coding
family with	protein-coding
ring finger	protein-coding
adenylate (	protein-coding
ras homolc	protein-coding
TNF recept	protein-coding
processing	protein-coding
glycophorir	protein-coding
cleavage ar	protein-coding
eukaryotic	protein-coding
transmeml	protein-coding
alkaline ph	protein-coding
CD47 antig	protein-coding
cytochrom	protein-coding
Ras associa	protein-coding
DENN/MAI	protein-coding
leucine rich	protein-coding
sema doma	protein-coding
snail family	protein-coding
B cell leuke	protein-coding
minichrom	protein-coding
phospholip	protein-coding
DEAD/H (A	protein-coding

ankyrin rep	protein-coding
ER degrada	protein-coding
KAT8 regul	protein-coding
spermatog	protein-coding
YTH domai	protein-coding
RAD54 hon	protein-coding
TBC1 doma	protein-coding
ubiquitin s	protein-coding
cyclin Pas1	protein-coding
acidic (leuc	protein-coding
follistatin-l	protein-coding
adenosine	protein-coding
kinesin fam	protein-coding
dual specif	protein-coding
phosphatic	protein-coding
Rho GTPas	protein-coding
stefin A2 lil	protein-coding
RIKEN cDN	protein-coding
special AT-	protein-coding
BCL2 modi	protein-coding
transforme	protein-coding
guanine nu	protein-coding
PDS5, regu	protein-coding
RIKEN cDN	protein-coding
1-acylglyce	protein-coding
fatty acid d	protein-coding
protease, s	protein-coding
nuclear fra	protein-coding
signal trans	protein-coding
nucleobind	protein-coding
membrane	protein-coding
cysteine-ri	protein-coding
collagen, t	protein-coding
transcriptic	protein-coding
CUGBP, Ela	protein-coding
ST8 alpha-I	protein-coding
Ras-like wi	protein-coding
MGAT4 far	protein-coding
xylosyltran	protein-coding
RIKEN cDN	protein-coding
cyclin G2	protein-coding
hypoxia inc	protein-coding
mitogen-ac	protein-coding
kinesin fam	protein-coding
SCP2 sterol	protein-coding
transmemb	protein-coding
tubulin, alp	protein-coding

integrin alp	protein-coding
epithelial n	protein-coding
potassium	protein-coding
serum/gluc	protein-coding
inhibin bet	protein-coding
alpha-kinas	protein-coding
Williams-B	protein-coding
G protein-c	protein-coding
phospholys	protein-coding
MACRO do	protein-coding
predicted g	protein-coding
DCP2 decap	protein-coding
ADP-ribosy	protein-coding
MDS1 and	protein-coding
sperm flag	protein-coding
zinc finger	protein-coding
zinc finger	protein-coding
metallo-be	protein-coding
tetratricop	protein-coding
mitochond	protein-coding
MAP kinase	protein-coding
Fc recepto	protein-coding
predicted g	protein-coding
syntaxin 18	protein-coding
Rhesus blo	protein-coding
kelch doma	protein-coding
SUMO1/se	protein-coding
nuclear rec	protein-coding
centrosom	protein-coding
SH3 and PX	protein-coding
small EDRK	protein-coding
mannosida	protein-coding
protein pho	protein-coding
NCK-associ	protein-coding
valyl-tRNA	protein-coding
fucokinase	protein-coding
coiled-coil	protein-coding
phosphopr	protein-coding
sushi doma	protein-coding
NDC1 trans	protein-coding
mitochond	protein-coding
cerebral do	protein-coding
cDNA sequ	protein-coding
GC-rich pro	protein-coding
thymopoie	protein-coding
antigen ide	protein-coding
DEAD (Asp	protein-coding

nuclear dis	protein-coding
PRP39 pre-	protein-coding
protein kin	protein-coding
high mobili	protein-coding
prosaposin	protein-coding
DENN/MAI	protein-coding
X-linked my	protein-coding
RIKEN cDN	protein-coding
nuclear fac	protein-coding
trans-actin	protein-coding
zinc finger	protein-coding
glutathione	protein-coding
ribosomal	protein-coding
TBC1 doma	protein-coding
mahogunir	protein-coding
solute carri	protein-coding
pleckstrin h	protein-coding
BTB (POZ)	protein-coding
oculocutan	protein-coding
mannoside	protein-coding
HEAT repea	protein-coding
anaphase p	protein-coding
solute carri	protein-coding
surfeit gen	protein-coding
Ras associa	protein-coding
translocate	protein-coding
Kruppel-lik	protein-coding
glucose-fru	protein-coding
transmemb	protein-coding
chemokine	protein-coding
sphingomy	protein-coding
S-antigen,	protein-coding
RAB18, me	protein-coding
serine (or c	protein-coding
integrin alp	protein-coding
sarcolemm	protein-coding
lectin, gala	protein-coding
MAS-relate	protein-coding
lysosomal-	protein-coding
mitogen-ac	protein-coding
enoyl Coen	protein-coding
PDZ and LII	protein-coding
eyes absen	protein-coding
signal pept	protein-coding
sorting nex	protein-coding
mitochond	protein-coding
WAS/WASI	protein-coding

predicted g	protein-coding
zinc fingers	protein-coding
protease, s	protein-coding
DnaJ (Hsp4	protein-coding
paralemmi	protein-coding
SUMO1 act	protein-coding
HECT doma	protein-coding
thiamine tr	protein-coding
family with	protein-coding
leucine rich	protein-coding
synaptotag	protein-coding
lysosomal t	protein-coding
ring finger	protein-coding
toll-like rec	protein-coding
tubulin, be	protein-coding
killer cell le	protein-coding
prune hom	protein-coding
methyltran	protein-coding
splicing fac	protein-coding
monocyte t	protein-coding
SEC22 vesi	protein-coding
ring finger	protein-coding
distal-less	protein-coding
dCTP pyrop	protein-coding
PHD finger	protein-coding
basic leucir	protein-coding
solute carri	protein-coding
NADH dehy	protein-coding
DENN/MAI	protein-coding
ring finger	protein-coding
solute carri	protein-coding
Max intera	protein-coding
PRP4 pre-n	protein-coding
ubiquitin-c	protein-coding
NADH dehy	protein-coding
minichrom	protein-coding
tet methylo	protein-coding
branched c	protein-coding
non-SMC e	protein-coding
zinc finger	protein-coding
ring finger	protein-coding
cadherin 8	protein-coding
myc induce	protein-coding
centromere	protein-coding
ceramide s	protein-coding
SR-related	protein-coding
centrosom	protein-coding

ubiquitinat	protein-coding
protein tyr	protein-coding
microsemi	protein-coding
zinc finger	protein-coding
NLR family	protein-coding
WD repeat	protein-coding
GC-rich prc	protein-coding
a disintegri	protein-coding
YY1 transcr	protein-coding
phosphatic	protein-coding
DNA segme	protein-coding
ELAV (emb	protein-coding
RIKEN cDN.	protein-coding
protein ph	protein-coding
PWP1 hom	protein-coding
metallothic	protein-coding
ubiquitin-li	protein-coding
neuregulin	protein-coding
charged m	protein-coding
CD1d1 anti	protein-coding
myelin-ass	protein-coding
protein ph	protein-coding
RIKEN cDN.	protein-coding
catsper cha	protein-coding
ephrin B1	protein-coding
MOB kinas	protein-coding
5-azacytidi	protein-coding
vesicle-ass	protein-coding
family with	protein-coding
MAK16 hor	protein-coding
heterogene	protein-coding
zinc finger	protein-coding
COP9 (cons	protein-coding
S100 calciu	protein-coding
acyl-coenz	protein-coding
zinc finger	protein-coding
LLP homolo	protein-coding
AT rich inte	protein-coding
interleukin	protein-coding
2-aminoeth	protein-coding
helicase, ly	protein-coding
vacuolar pr	protein-coding
annexin A3	protein-coding
kinesin ligh	protein-coding
RAS-like, fa	protein-coding
F-box prote	protein-coding
family with	protein-coding

zinc finger	protein-coding
Cbp/p300-i	protein-coding
cAMP resp	protein-coding
MAS-relate	protein-coding
melanoreg	protein-coding
solute carri	protein-coding
signal reco	protein-coding
WD repeat	protein-coding
FAD-depen	protein-coding
zinc finger	protein-coding
cyclin-depe	protein-coding
cytochrom	protein-coding
RAB11A, m	protein-coding
early growt	protein-coding
phosphodi	protein-coding
neuropilin	protein-coding
Rho GTPas	protein-coding
zinc finger	protein-coding
cell growth	protein-coding
superkiller	protein-coding
5-phospho	protein-coding
PET112 ho	protein-coding
CCCTC-bin	protein-coding
formin-like	protein-coding
F-box and l	protein-coding
Sjogren syr	protein-coding
RNA bindin	protein-coding
mitogen-ac	protein-coding
transmeml	protein-coding
zinc finger,	protein-coding
coiled-coil	protein-coding
phosphorik	protein-coding
unc-45 hor	protein-coding
coiled-coil	protein-coding
protein kin	protein-coding
histone clu	protein-coding
cell divisior	protein-coding
kin of IRRE	protein-coding
uridine mo	protein-coding
N(alpha)-a	protein-coding
prostate tu	protein-coding
DnaJ (Hsp4	protein-coding
RIKEN cDN	protein-coding
tumor-assc	protein-coding
xyloside xy	protein-coding
sine oculis-	protein-coding
a disintegri	protein-coding

solute carri	protein-coding
histidine de	protein-coding
TBC1 doma	protein-coding
transport a	protein-coding
autophagy	protein-coding
presenilin 2	protein-coding
transcriptic	protein-coding
anti-silenci	protein-coding
vasodilator	protein-coding
hairy and e	protein-coding
ATP/GTP b	protein-coding
lactalbumi	protein-coding
ubiquitin sp	protein-coding
killer cell le	protein-coding
influenza v	protein-coding
high mobili	protein-coding
ring finger	protein-coding
cadherin, E	protein-coding
translocase	protein-coding
CCZ1 vacuo	protein-coding
TNFAIP3 in	protein-coding
zinc finger	protein-coding
cullin 4A	protein-coding
glucan (1,4	protein-coding
NME/NM2	protein-coding
RNA bindin	protein-coding
propionyl C	protein-coding
dipeptidylp	protein-coding
myelopero	protein-coding
septin 8	protein-coding
4short chai	protein-coding
zinc finger	protein-coding
CD9 antige	protein-coding
ring finger	protein-coding
ubiquilin 4	protein-coding
interleukin	protein-coding
chromodor	protein-coding
X Kell bloo	protein-coding
transmemb	protein-coding
dihydrolipc	protein-coding
nucleolar a	protein-coding
nucleolin	protein-coding
UDP-Gal:be	protein-coding
zinc finger	protein-coding
cyclin D-typ	protein-coding
somatostat	protein-coding
ephrin A1	protein-coding

mediator c	protein-coding
transmemb	protein-coding
cDNA sequ	protein-coding
POM121 m	protein-coding
Ras associa	protein-coding
family with	protein-coding
lipin 2	protein-coding
tRNA meth	protein-coding
histone de	protein-coding
fos-like ant	protein-coding
glyceropho	protein-coding
immature (	protein-coding
tumor prot	protein-coding
spermine c	protein-coding
glucagon-li	protein-coding
calcium/ca	protein-coding
paired-like	protein-coding
serine/thre	protein-coding
cell divisior	protein-coding
alkB, alkyla	protein-coding
SAFB-like, t	protein-coding
RAD23b hc	protein-coding
3-hydroxyi:	protein-coding
transmemb	protein-coding
microsoma	protein-coding
myosin, ligl	protein-coding
ArfGAP wit	protein-coding
adenomatc	protein-coding
Kruppel-lik	protein-coding
ATPase inh	protein-coding
NLR family,	protein-coding
polymerase	protein-coding
ubiquitin s	protein-coding
protein ph	protein-coding
pentatrico	protein-coding
family with	protein-coding
TNF recept	protein-coding
zinc finger,	protein-coding
interleukin	protein-coding
serine hydr	protein-coding
Rho GTPas	protein-coding
ATP-bindin	protein-coding
C-type lect	protein-coding
zinc finger	protein-coding
ras homolc	protein-coding
fumarylace	protein-coding
RIO kinase	protein-coding

cleavage ar	protein-coding
inositol 1,4	protein-coding
pleckstrin h	protein-coding
RAS p21 pr	protein-coding
N(alpha)-ac	protein-coding
zinc finger	protein-coding
neuron nav	protein-coding
sushi doma	protein-coding
ceramide s	protein-coding
desumoyla	protein-coding
family with	protein-coding
serine (or c	protein-coding
mitochond	protein-coding
Fas-activat	protein-coding
SMAD fami	protein-coding
EF-hand ca	protein-coding
scinderin	protein-coding
mitochond	protein-coding
lactate deh	protein-coding
olfactomed	protein-coding
ankyrin rep	protein-coding
adaptor-re	protein-coding
nicalin hom	protein-coding
S-adenosyl	protein-coding
Prkr interac	protein-coding
regulator o	protein-coding
proviral int	protein-coding
E2F transcr	protein-coding
brain expre	protein-coding
ATPase, Ca	protein-coding
kaptin	protein-coding
predicted g	protein-coding
mitochond	protein-coding
solute carri	protein-coding
survival mo	protein-coding
coiled-coil	protein-coding
activating t	protein-coding
coiled-coil	protein-coding
YME1-like 1	protein-coding
calpain 10	protein-coding
wingless-ty	protein-coding
KAT8 regul	protein-coding
transmemb	protein-coding
PDS5, regu	protein-coding
helicase wi	protein-coding
potassium	protein-coding
cathepsin H	protein-coding

protein tyr	protein-coding
recombina	protein-coding
utrophin	protein-coding
T cell, imm	protein-coding
cell divisior	protein-coding
transforma	protein-coding
C-terminal	protein-coding
taspase, th	protein-coding
ALS2 C-terr	protein-coding
syntaxin bi	protein-coding
meningiom	protein-coding
potassium	protein-coding
ELOVL fam	protein-coding
basonuclin	protein-coding
zinc finger	protein-coding
calcium/ca	protein-coding
SWA-70 pr	protein-coding
xenotropic	protein-coding
transmemb	protein-coding
nuclear fac	protein-coding
SRY (sex de	protein-coding
gypsy retro	protein-coding
SCP2 sterol	protein-coding
UDP-GlcNA	protein-coding
regulator o	protein-coding
tumor necr	protein-coding
general tra	protein-coding
membrane	protein-coding
translocase	protein-coding
sorting nex	protein-coding
annexin A6	protein-coding
tyrosyl-tRN	protein-coding
interleukin	protein-coding
olfactory re	protein-coding
formin-like	protein-coding
coagulatior	protein-coding
nudix (nucl	protein-coding
mannose-6	protein-coding
attractin lik	protein-coding
histone clu	protein-coding
family with	protein-coding
RIKEN cDN	protein-coding
alpha gluco	protein-coding
tribbles ho	protein-coding
olfactomec	protein-coding
DEAH (Asp-	protein-coding
basic leucir	protein-coding

RIKEN cDN.	protein-coding
vacuolar pr	protein-coding
oxysterol b	protein-coding
transcriptic	protein-coding
poly(A) bin	protein-coding
adenosine	protein-coding
RING1 and	protein-coding
lysophosph	protein-coding
acid phosp	protein-coding
GSG1-like	protein-coding
Kruppel-lik	protein-coding
transmemb	protein-coding
cytochrom	protein-coding
F-box prote	protein-coding
tripartite m	protein-coding
transmemb	protein-coding
papillary re	protein-coding
kelch-like 2	protein-coding
ribosomal		protein-coding
glutaredox	protein-coding
RIKEN cDN.	protein-coding
ubiquitin sp	protein-coding
armadillo r	protein-coding
EP300 inter	protein-coding
multiple C2	protein-coding
osteopetro	protein-coding
COMM dor	protein-coding
WD repeat	protein-coding
RIKEN cDN.	protein-coding
Casitas B-li	protein-coding
autophagy	protein-coding
NLR family,	protein-coding
leucine rich	protein-coding
olfactory re	protein-coding
leucine rich	protein-coding
peroxisoma	protein-coding
solute carri	protein-coding
translocase	protein-coding
gem (nucle	protein-coding
RIKEN cDN.	protein-coding
CGG triplet	protein-coding
hydroxycar	protein-coding
CD180 anti	protein-coding
ligand depe	protein-coding
polymerase	protein-coding
solute carri	protein-coding
protein tyr	protein-coding

lysosomal-a	protein-coding
bone marro	protein-coding
src homolo	protein-coding
CDK5 and A	protein-coding
CREB3 regu	protein-coding
taspase, th	protein-coding
serine/thre	protein-coding
oxidation r	protein-coding
Kruppel-lik	protein-coding
THO compl	protein-coding
ankyrin rep	protein-coding
carbonic an	protein-coding
Rap guanin	protein-coding
myeloblast	protein-coding
ribonucleo	protein-coding
Abelson he	protein-coding
calcyclin bi	protein-coding
basic leucir	protein-coding
SH3-bindin	protein-coding
WD repeat	protein-coding
cDNA sequ	protein-coding
glucan (1,4	protein-coding
EH domain	protein-coding
CD84 antig	protein-coding
membrane	protein-coding
phosphatic	protein-coding
serine/argi	protein-coding
DnaJ (Hsp4	protein-coding
adaptor-re	protein-coding
heterogene	protein-coding
zinc finger,	protein-coding
inhibitor of	protein-coding
zinc finger	protein-coding
protein pho	protein-coding
mucosa ass	protein-coding
zinc finger	protein-coding
Abelson he	protein-coding
transcriptic	protein-coding
transcriptic	protein-coding
nucleopori	protein-coding
Rho guanir	protein-coding
TatD DNas	protein-coding
acyl-CoA th	protein-coding
transmemb	protein-coding
sarcoglycar	protein-coding
transcriptic	protein-coding
glutamate	protein-coding

toll-like rec	protein-coding
torsin fami	protein-coding
cytochrom	protein-coding
myotrophi	protein-coding
dishevellec	protein-coding
signal reco	protein-coding
prostaglan	protein-coding
protein ph	protein-coding
SECIS bindi	protein-coding
Janus kinas	protein-coding
poly (ADP-r	protein-coding
apoptotic p	protein-coding
adrenergic	protein-coding
astrotactin	protein-coding
transforma	protein-coding
aminolevul	protein-coding
NFU1 iron-	protein-coding
dCMP dear	protein-coding
serine (or c	protein-coding
phosphoin	protein-coding
transcriptic	protein-coding
cyclin L1	protein-coding
tyrosine 3-	protein-coding
X-ray repai	protein-coding
SRY (sex de	protein-coding
kinesin fan	protein-coding
arrestin do	protein-coding
lymphocyt	protein-coding
Rho GTPas	protein-coding
INO80 hom	protein-coding
non-SMC e	protein-coding
LON peptic	protein-coding
deleted in	protein-coding
cornichon	protein-coding
f-box prote	protein-coding
adenosine	protein-coding
thymocyte	protein-coding
tetraspanir	protein-coding
U2 small nu	protein-coding
family with	protein-coding
multiple EC	protein-coding
low density	protein-coding
high mobili	protein-coding
solute carri	protein-coding
lamin B rec	protein-coding
RIKEN cDN	protein-coding
cytochrom	protein-coding

adenosine	protein-coding
death-asso	protein-coding
valyl-tRNA	protein-coding
adenosine	protein-coding
cyclin M3	protein-coding
oligosacchа	protein-coding
zinc finger,	protein-coding
ORAI calciu	protein-coding
heparan su	protein-coding
voltage-de	protein-coding
progestin a	protein-coding
zinc finger	protein-coding
C1GALT1-s	protein-coding
transcriptic	protein-coding
ring finger	protein-coding
zinc finger	protein-coding
RIKEN cDN	protein-coding
ribosomal	protein-coding
PDZ domai	protein-coding
HCLS1 binc	protein-coding
WNK lysine	protein-coding
proline rich	protein-coding
carbamoyl-	protein-coding
coiled-coil-	protein-coding
TBC1 doma	protein-coding
kinesin fam	protein-coding
feminizatic	protein-coding
arylsulfatas	protein-coding
RIKEN cDN	protein-coding
lines homo	protein-coding
SET domaiı	protein-coding
heterogene	protein-coding
predicted g	protein-coding
GRAM dom	protein-coding
hydroxyste	protein-coding
runt-relate	protein-coding
GTPase, ve	protein-coding
dual specif	protein-coding
split hand/	protein-coding
protein kin	protein-coding
transcriptic	protein-coding
transmemb	protein-coding
parvin, gan	protein-coding
RAS, dexan	protein-coding
DNA segme	protein-coding
cytidine mc	protein-coding
dermatan s	protein-coding

chemokine	protein-coding
A kinase (P	protein-coding
cyclic nucle	protein-coding
E4F transcr	protein-coding
hippocamp	protein-coding
ERO1-like (	protein-coding
syntaxin 6	protein-coding
SH3-domai	protein-coding
RIKEN cDN.	protein-coding
prickle hon	protein-coding
5'-nucleoti	protein-coding
tripartite m	protein-coding
solute carri	protein-coding
K(lysine) ac	protein-coding
acyl-Coenz	protein-coding
adiponecti	protein-coding
sortilin-rela	protein-coding
aspartate-b	protein-coding
brain prote	protein-coding
vascular en	protein-coding
zinc finger	protein-coding
proteasom	protein-coding
makorin, ri	protein-coding
secreted fr	protein-coding
LIM and se	protein-coding
aldehyde d	protein-coding
transmemb	protein-coding
calpain, sm	protein-coding
immunoglo	protein-coding
adaptor pro	protein-coding
RIKEN cDN.	protein-coding
RIKEN cDN.	protein-coding
FK506 bind	protein-coding
splicing fac	protein-coding
CTAGE fam	protein-coding
breast canc	protein-coding
cyclin L1	protein-coding
ATP-bindin	protein-coding
calcium reg	protein-coding
N-acetyltra	protein-coding
solute carri	protein-coding
integrin be	protein-coding
prothymos	protein-coding
suppressor	protein-coding
autophagy	protein-coding
OTU domai	protein-coding
Ras homolo	protein-coding

integral me	protein-coding
guanine nu	protein-coding
sphingosin	protein-coding
fibronectin	protein-coding
v-ral simiar	protein-coding
exocyst cor	protein-coding
insulin rece	protein-coding
triggering r	protein-coding
ubiquitin sp	protein-coding
cell adhesi	protein-coding
predicted g	protein-coding
CTD (carbo	protein-coding
protein tyr	protein-coding
nuclear aut	protein-coding
insulin-like	protein-coding
ubiquitin sp	protein-coding
lipase, fam	protein-coding
NME/NM2	protein-coding
DnaJ (Hsp4	protein-coding
doublecort	protein-coding
polymerase	protein-coding
transient re	protein-coding
nicalin hom	protein-coding
oxysterol b	protein-coding
L-amino ac	protein-coding
SUMO/sen	protein-coding
adiponecti	protein-coding
transmemb	protein-coding
nuclear fac	protein-coding
DCP1 deca	protein-coding
phosphoin	protein-coding
diacylglyce	protein-coding
splicing fac	protein-coding
RB1-induci	protein-coding
FMS-like ty	protein-coding
coiled-coil	protein-coding
neuron der	protein-coding
solute carri	protein-coding
claudin 12	protein-coding
calcium ch	protein-coding
Rho GDP di	protein-coding
nucleolar p	protein-coding
alanyl-tRN	protein-coding
heat shock	protein-coding
cytochrom	protein-coding
cholinergic	protein-coding
GA repeat	protein-coding

glypican 1	protein-coding
NHL repeat	protein-coding
mannoside	protein-coding
phosphorib	protein-coding
CWC27 spli	protein-coding
organic sol	protein-coding
F-box prote	protein-coding
mitochond	protein-coding
WW domai	protein-coding
glutathione	protein-coding
klotho beta	protein-coding
netrin 3	protein-coding
Rho guanin	protein-coding
dynamin 2	protein-coding
RNA bindin	protein-coding
NADH dehy	protein-coding
glia matura	protein-coding
Kruppel-lik	protein-coding
H2A histon	protein-coding
major facili	protein-coding
acylpeptide	protein-coding
Fc receptor	protein-coding
metallopho	protein-coding
LFNG O-fuc	protein-coding
protein kin	protein-coding
sex comb c	protein-coding
lipopolysac	protein-coding
zyg-ll famil	protein-coding
guanylate b	protein-coding
signal-indu	protein-coding
predicted g	protein-coding
SET and MY	protein-coding
non-catalyt	protein-coding
stomatin	protein-coding
polycystic k	protein-coding
zinc finger	protein-coding
mitochond	protein-coding
low density	protein-coding
N(alpha)-ac	protein-coding
aspartyl-tR	protein-coding
Parkinson c	protein-coding
proviral int	protein-coding
thioredoxin	protein-coding
NOP10 ribo	protein-coding
transmemb	protein-coding
mediator c	protein-coding
myelocyton	protein-coding

delta-like 2	protein-coding
forkhead b	protein-coding
zinc finger	protein-coding
protein-L-is	protein-coding
islet cell au	protein-coding
regulator o	protein-coding
G protein-c	protein-coding
zinc finger	protein-coding
pleckstrin h	protein-coding
solute carri	protein-coding
split hand/	protein-coding
related RAS	protein-coding
NCK interac	protein-coding
monoglyce	protein-coding
solute carri	protein-coding
Jun dimeriz	protein-coding
Josephin do	protein-coding
SUN domai	protein-coding
stearoyl-Co	protein-coding
ribosomal p	protein-coding
cDNA sequ	protein-coding
U2AF homo	protein-coding
transcriptio	protein-coding
emerin	protein-coding
ubiquitinat	protein-coding
zinc finger	protein-coding
MOB kinase	protein-coding
heterogene	protein-coding
secretory c	protein-coding
family with	protein-coding
twisted gas	protein-coding
period circa	protein-coding
mannosyl-c	protein-coding
ubiquitin-a	protein-coding
Max protei	protein-coding
centrosom	protein-coding
serological	protein-coding
thymidine k	protein-coding
protein pho	protein-coding
Erbb2 inter	protein-coding
jun proto-o	protein-coding
hippocalcin	protein-coding
interferon	protein-coding
mitochond	protein-coding
membrane	protein-coding
BCL2-assoc	protein-coding
BCL2-like 1	protein-coding

cysteine co	protein-coding
ets variant	protein-coding
fibronectin	protein-coding
zinc finger,	protein-coding
prostaglan	protein-coding
hexosamin	protein-coding
zona pelluc	protein-coding
Wolf-Hirscl	protein-coding
kelch-like 2	protein-coding
phospholip	protein-coding
excision re	protein-coding
cytochrom	protein-coding
ATPase fan	protein-coding
PAK1 inter	protein-coding
GIPC PDZ d	protein-coding
endothelin	protein-coding
sema dom	protein-coding
polymeras	protein-coding
eukaryotic	protein-coding
high mobili	protein-coding
solute carr	protein-coding
tetratricop	protein-coding
coiled-coil	protein-coding
dymeclin	protein-coding
homeobox	protein-coding
PNMA-like	protein-coding
coagulatio	protein-coding
RNA bindin	protein-coding
MUS81 end	protein-coding
neural prec	protein-coding
coagulatio	protein-coding
predicted g	protein-coding
ADAMTS-li	protein-coding
WW doma	protein-coding
guanine nu	protein-coding
leucine rich	protein-coding
staufen (RN	protein-coding
La ribonucl	protein-coding
ribonuclea	protein-coding
glucosamir	protein-coding
myosin IXb	protein-coding
solute carr	protein-coding
solute carr	protein-coding
prion prote	protein-coding
secretory c	protein-coding
DNA segme	protein-coding
E74-like fac	protein-coding

protein pho	protein-coding
phosphorib	protein-coding
scribbled h	protein-coding
phosphatic	protein-coding
nucleopori	protein-coding
lymphocyte	protein-coding
proteasom	protein-coding
ADP-ribosy	protein-coding
3-hydroxy-	protein-coding
ring finger	protein-coding
cDNA sequ	protein-coding
cysteine-ric	protein-coding
cleft lip and	protein-coding
WD repeat	protein-coding
N-myc dow	protein-coding
phosphatic	protein-coding
xanthine de	protein-coding
protein pho	protein-coding
ribosomal	protein-coding
capping pro	protein-coding
CDC16 cell	protein-coding
mitochond	protein-coding
dual specif	protein-coding
MCM (mini	protein-coding
death asso	protein-coding
splicing fac	protein-coding
Rho GTPase	protein-coding
SR-related	protein-coding
lipopolysac	protein-coding
coiled-coil	protein-coding
selenium b	protein-coding
MAP kinase	protein-coding
glutathione	protein-coding
sterile alph	protein-coding
DTW doma	protein-coding
ARP1 actin	protein-coding
SUB1 homo	protein-coding
ribose 5-ph	protein-coding
small VCP/	protein-coding
sorting nex	protein-coding
NEDD4 bin	protein-coding
B cell trans	protein-coding
ATPase, Na	protein-coding
protein pho	protein-coding
BRCA1 inte	protein-coding
G protein-c	protein-coding
activating s	protein-coding

coiled-coil	protein-coding
proline rich	protein-coding
RCSD doma	protein-coding
solute carri	protein-coding
ubiquitin-c	protein-coding
Rap guanin	protein-coding
ubiquitin s	protein-coding
upstream b	protein-coding
glutathione	protein-coding
myelin pro	protein-coding
forkhead b	protein-coding
carbonyl re	protein-coding
pleckstrin a	protein-coding
retrotransp	protein-coding
LSM7 hom	protein-coding
teneurin tr	protein-coding
B cell CLL/l	protein-coding
ring finger	protein-coding
gem (nucle	protein-coding
SNF2 histo	protein-coding
cerebral dc	protein-coding
protein arg	protein-coding
PH domain	protein-coding
zinc finger	protein-coding
histamine r	protein-coding
solute carri	protein-coding
WD repeat	protein-coding
lysosomal-a	protein-coding
cartilage as	protein-coding
CCR4-NOT	protein-coding
REV1 homo	protein-coding
DCN1, defe	protein-coding
G protein-c	protein-coding
SWI/SNF re	protein-coding
RAS, guany	protein-coding
spectrin re	protein-coding
SRY (sex de	protein-coding
ribosomal p	protein-coding
special AT-	protein-coding
calpain 7	protein-coding
transcriptio	protein-coding
LON peptic	protein-coding
centrosom	protein-coding
chondroitir	protein-coding
caspase 3	protein-coding
6-phospho	protein-coding
early grow	protein-coding

GDNF-indu	protein-coding
hexokinase	protein-coding
B cell recep	protein-coding
histone dea	protein-coding
RAB21, me	protein-coding
tet methylc	protein-coding
SH2 domai	protein-coding
mitogen-ac	protein-coding
inositol pol	protein-coding
5-phospho	protein-coding
mitogen-ac	protein-coding
kinesin fam	protein-coding
selenocyste	protein-coding
ribosomal p	protein-coding
branched c	protein-coding
kelch repea	protein-coding
Tia1 cytoto	protein-coding
FYVE, RhoG	protein-coding
cyclic nucle	protein-coding
ubiquitin sp	protein-coding
Dmx-like 2	protein-coding
testicular c	protein-coding
family with	protein-coding
secretogra	protein-coding
sulfide quir	protein-coding
tripartite m	protein-coding
kinesin fam	protein-coding
RIKEN cDN.	protein-coding
calcium/ca	protein-coding
excision re	protein-coding
hydroxyste	protein-coding
family with	protein-coding
proteasom	protein-coding
excision re	protein-coding
transport a	protein-coding
potassium	protein-coding
mitogen-ac	protein-coding
serine/thre	protein-coding
cytohesin 1	protein-coding
RAB5B, me	protein-coding
mitochond	protein-coding
diaphanous	protein-coding
angiomotir	protein-coding
hydroxyste	protein-coding
ATP syntha	protein-coding
ets variant	protein-coding
natriuretic	protein-coding

alcohol del	protein-coding
WNT1 indu	protein-coding
bromodom	protein-coding
nei like 3 (E	protein-coding
tetratricop	protein-coding
ribosome b	protein-coding
synemin, ir	protein-coding
solute carri	protein-coding
lysophosph	protein-coding
LIM domai	protein-coding
chromodor	protein-coding
tankyrase,	protein-coding
dystrobrev	protein-coding
vacuolar pr	protein-coding
cyclin T2	protein-coding
adaptor-re	protein-coding
amylo-1,6-;	protein-coding
trichohyalii	protein-coding
DnaJ (Hsp4	protein-coding
leucine rich	protein-coding
membrane	protein-coding
sphingosin	protein-coding
DNA segme	protein-coding
deleted in	protein-coding
F-box and l	protein-coding
ubiquitin-c	protein-coding
AT-hook tra	protein-coding
low density	protein-coding
serine (or c	protein-coding
Ngfi-A bind	protein-coding
membrane	protein-coding
NADH dehy	protein-coding
sorting nex	protein-coding
RIKEN cDN.	protein-coding
ezrin	protein-coding
cDNA sequ	protein-coding
immunity-r	protein-coding
inositol pol	protein-coding
DNA segme	protein-coding
A kinase (P	protein-coding
pituitary tu	protein-coding
ribosomal	protein-coding
mitochond	protein-coding
diacylglyce	protein-coding
surfactant	protein-coding
aminoacyl	protein-coding
biorientatio	protein-coding

vacuolar pr	protein-coding
transformi	protein-coding
cardiotropl	protein-coding
coiled-coil	protein-coding
motile sper	protein-coding
interferon	protein-coding
cytoskeleto	protein-coding
mucin 13, e	protein-coding
serine palm	protein-coding
asparagine	protein-coding
RELT tumo	protein-coding
BCL6 intera	protein-coding
protein pho	protein-coding
tet methylo	protein-coding
ring finger	protein-coding
cadherin 2:	protein-coding
UDP-Gal:be	protein-coding
TBC1 doma	protein-coding
tribbles ho	protein-coding
tubulin cof	protein-coding
ubiquitin p	protein-coding
glycine clea	protein-coding
ELOVL fam	protein-coding
golgi reass	protein-coding
secernin 3	protein-coding
frizzled hor	protein-coding
leukemia ir	protein-coding
family with	protein-coding
homeobox	protein-coding
calpastatin	protein-coding
CAS1 doma	protein-coding
tubulin, alp	protein-coding
MAP kinase	protein-coding
methyl-CpG	protein-coding
N-acetyltra	protein-coding
autism sus	protein-coding
CDC16 cell	protein-coding
proteasom	protein-coding
serine/argi	protein-coding
histone clu	protein-coding
methyltran	protein-coding
G protein-c	protein-coding
UBX domai	protein-coding
tetratricop	protein-coding
H2A histon	protein-coding
RIKEN cDN.	protein-coding
zinc finger	protein-coding

seven in ab	protein-coding
cyclin G ass	protein-coding
zinc finger	protein-coding
synaptonei	protein-coding
methyltran	protein-coding
brain expre	protein-coding
glutamine a	protein-coding
lamin B rec	protein-coding
SLX4 intera	protein-coding
AE binding	protein-coding
tubulin, gai	protein-coding
family with	protein-coding
egl-9 family	protein-coding
RIKEN cDN.	protein-coding
NHP2 ribor	protein-coding
tumor necr	protein-coding
armadillo r	protein-coding
ribosomal p	protein-coding
solute carri	protein-coding
coiled-coil	protein-coding
sema doma	protein-coding
E2F transcr	protein-coding
Rho guanir	protein-coding
CD3 antige	protein-coding
syntaxin 16	protein-coding
protease, s	protein-coding
DENN/MAI	protein-coding
plasminoge	protein-coding
coenzyme (	protein-coding
tumor prot	protein-coding
calcium/ca	protein-coding
Map3k7 C-	protein-coding
tescalcin	protein-coding
diaphanous	protein-coding
integrator	protein-coding
sin3 associ	protein-coding
glutamine f	protein-coding
ankyrin rep	protein-coding
serglycin	protein-coding
inner centr	protein-coding
lymphoblas	protein-coding
NUF2, NDC	protein-coding
olfactory re	protein-coding
heparan su	protein-coding
sprouty pro	protein-coding
limbic syste	protein-coding
nuclear tra	protein-coding

family with	protein-coding
growth hor	protein-coding
nephronop	protein-coding
RAB11 fam	protein-coding
galactosylc	protein-coding
SMAD fami	protein-coding
T cell leuke	protein-coding
NOL1/NOP	protein-coding
small nucle	protein-coding
mitochond	protein-coding
olfactomec	protein-coding
ribosomal I	protein-coding
N-acetylglu	protein-coding
NIMA (neve	protein-coding
ubiquitin sp	protein-coding
ribosomal p	protein-coding
family with	protein-coding
malonyl-Co	protein-coding
ankyrin rep	protein-coding
polymerase	protein-coding
splicing reg	protein-coding
regulating s	protein-coding
pleckstrin	protein-coding
mesoderm	protein-coding
mitochond	protein-coding
ubiquitin p	protein-coding
EBNA1 binc	protein-coding
aryl hydroc	protein-coding
Ras interac	protein-coding
ankyrin rep	protein-coding
family with	protein-coding
NCK-associ	protein-coding
transmemb	protein-coding
thrombom	protein-coding
annexin A3	protein-coding
genetic sup	protein-coding
potassium	protein-coding
ubiquitin sp	protein-coding
fibroblast g	protein-coding
cyclin E2	protein-coding
hect (homo	protein-coding
neuropilin	protein-coding
ecotropic v	protein-coding
actin-like 6	protein-coding
tudor doma	protein-coding
ELOVL fam	protein-coding
DIP2 disco-	protein-coding

capping pr	protein-coding
male-speci	protein-coding
ubiquitin p	protein-coding
K(lysine) ac	protein-coding
phosphorik	protein-coding
polymerase	protein-coding
heterogene	protein-coding
transmemk	protein-coding
zinc finger,	protein-coding
recombina	protein-coding
leucine rich	protein-coding
ADP-ribosy	protein-coding
predicted g	protein-coding
gametoger	protein-coding
solute carri	protein-coding
zinc finger	protein-coding
nerve grow	protein-coding
cutC coppe	protein-coding
membrane	protein-coding
AT rich inte	protein-coding
SMC hinge	protein-coding
nuclear tra	protein-coding
KAT8 regul	protein-coding
calcium rel	protein-coding
zinc finger	protein-coding
Iroquois re	protein-coding
RAB24, me	protein-coding
nuclear fac	protein-coding
nudix (nucl	protein-coding
N-acetylne	protein-coding
tripeptidyl	protein-coding
HMG box c	protein-coding
H2.0-like h	protein-coding
KDEL (Lys-A	protein-coding
S-adenosyl	protein-coding
apoptosis e	protein-coding
protein kin	protein-coding
Rho GTPase	protein-coding
TGF-beta a	protein-coding
chemokine	protein-coding
CD274 anti	protein-coding
meiosis arr	protein-coding
prosaposin	protein-coding
transmemk	protein-coding
coiled-coil	protein-coding
chromodor	protein-coding
glycosyltra	protein-coding

YTH domai	protein-coding
SEC22 vesi	protein-coding
resistin like	protein-coding
WAS prote	protein-coding
LysM, puta	protein-coding
SEC22 vesi	protein-coding
nuclear rec	protein-coding
zinc finger	protein-coding
HERV-H LTI	protein-coding
B cell recep	protein-coding
olfactory re	protein-coding
jun proto-c	protein-coding
eosinophil-	protein-coding
ankyrin rep	protein-coding
mediator c	protein-coding
carboxypep	protein-coding
phosphodi	protein-coding
zinc finger,	protein-coding
UTP18, sm	protein-coding
interferon	protein-coding
endothelin	protein-coding
acyl-Coenz	protein-coding
suppressor	protein-coding
solute carri	protein-coding
OTU domai	protein-coding
Sec24 relat	protein-coding
nuclear rec	protein-coding
RIKEN cDN	protein-coding
signal trans	protein-coding
nicalin hon	protein-coding
gem (nucle	protein-coding
UDP-N-ace	protein-coding
RIKEN cDN	protein-coding
H3 histone	protein-coding
cell adhesi	protein-coding
potassium	protein-coding
nuclear fac	protein-coding
methyltran	protein-coding
zinc fingers	protein-coding
neuroligin	protein-coding
gap junctio	protein-coding
chemokine	protein-coding
phosphatic	protein-coding
GLI pathog	protein-coding
TBC1 doma	protein-coding
parathyroi	protein-coding
myosin IIIA	protein-coding

interferon	protein-coding
multiple EC	protein-coding
zinc finger	protein-coding
ATP-bindin	protein-coding
CART prepr	protein-coding
sine oculis-	protein-coding
canopy 2 h	protein-coding
CD2-associ	protein-coding
RIKEN cDN.	protein-coding
eukaryotic	protein-coding
UDP-N-ace	protein-coding
N-deacetyl	protein-coding
transducin	protein-coding
RIKEN cDN.	protein-coding
proteasom	protein-coding
transformi	protein-coding
LYR motif c	protein-coding
C-type lect	protein-coding
exosome c	protein-coding
Ewing tum	protein-coding
dihydrolipo	protein-coding
glutamate	protein-coding
prenyl (sola	protein-coding
raftlin fami	protein-coding
UBX domai	protein-coding
acidic (leuc	protein-coding
COX assem	protein-coding
transcriptio	protein-coding
regulation	protein-coding
hematopoi	protein-coding
RIKEN cDN.	protein-coding
neuroplast	protein-coding
NIMA (neve	protein-coding
estrogen re	protein-coding
adaptor-re	protein-coding
regulator o	protein-coding
calcium cha	protein-coding
signal-indu	protein-coding
spermidine	protein-coding
ninein	protein-coding
B cell CLL/l	protein-coding
v-maf musc	protein-coding
glycogen sy	protein-coding
LIM domai	protein-coding
filamin bin	protein-coding
spermidine	protein-coding
guanine nu	protein-coding

nuclear fac	protein-coding
forkhead b	protein-coding
major facili	protein-coding
choline kin	protein-coding
zinc finger	protein-coding
solute carri	protein-coding
transmemb	protein-coding
peroxisoma	protein-coding
ariadne ub	protein-coding
engulfmen	protein-coding
EGF-like m	protein-coding
ribosomal	protein-coding
coronin 7	protein-coding
major vault	protein-coding
solute carri	protein-coding
spinster ho	protein-coding
adiponecti	protein-coding
dihydropyr	protein-coding
tachykinin	protein-coding
small nucle	protein-coding
cytochrom	protein-coding
lipoma HM	protein-coding
RIKEN cDN	protein-coding
polyadenyl	protein-coding
protein ph	protein-coding
PET112 ho	protein-coding
CD300 anti	protein-coding
upstream b	protein-coding
glutamate-	protein-coding
LY6/PLAUR	protein-coding
PHD finger	protein-coding
N-terminal	protein-coding
dickkopf h	protein-coding
trans-actin	protein-coding
RIKEN cDN	protein-coding
activin rec	protein-coding
peroxisom	protein-coding
karyopheri	protein-coding
IQ motif co	protein-coding
midasin ho	protein-coding
RNA bindin	protein-coding
zinc finger	protein-coding
ring finger	protein-coding
growth diff	protein-coding
eukaryotic	protein-coding
NTPase, KA	protein-coding
tensin 3	protein-coding

zinc finger	protein-coding
PH domain	protein-coding
actin relate	protein-coding
Kruppel-lik	protein-coding
La ribonucl	protein-coding
tripartite m	protein-coding
unc-80 hor	protein-coding
unc-51 like	protein-coding
calcium reg	protein-coding
WW domai	protein-coding
growth arr	protein-coding
pyruvate d	protein-coding
leucine rich	protein-coding
signal-indu	protein-coding
pyrophosp	protein-coding
ATP5S-like	protein-coding
transcriptio	protein-coding
JAZF zinc fi	protein-coding
protein pho	protein-coding
adenylate c	protein-coding
ribosomal p	protein-coding
period circa	protein-coding
major urina	protein-coding
tRNA meth	protein-coding
Znrd1 antis	protein-coding
mutS homo	protein-coding
DNA (cytos	protein-coding
multiple C2	protein-coding
leukotriene	protein-coding
dachshund	protein-coding
ankyrin rep	protein-coding
ADAMTS-lil	protein-coding
ribonucleo	protein-coding
glutamate-	protein-coding
Rho GTPase	protein-coding
methionine	protein-coding
CDC42 sma	protein-coding
transmemb	protein-coding
GTPase act	protein-coding
interferon	protein-coding
UTP6, smal	protein-coding
enhancer o	protein-coding
tribbles ho	protein-coding
phosphatid	protein-coding
transmemb	protein-coding
cell cycle p	protein-coding
zinc finger	protein-coding

cDNA sequ	protein-coding
MAP-kinas	protein-coding
hect (homo	protein-coding
cortexin 3	protein-coding
deoxyhypu	protein-coding
egl-9 famil	protein-coding
CLOCK inte	protein-coding
ribophorin	protein-coding
actin filam	protein-coding
cathepsin E	protein-coding
homeobox	protein-coding
interleukin	protein-coding
frizzled ho	protein-coding
ankyrin rep	protein-coding
TSC22 dom	protein-coding
myosin XVI	protein-coding
influenza v	protein-coding
cDNA sequ	protein-coding
speckle-typ	protein-coding
IscU iron-s	protein-coding
Hedgehog-	protein-coding
apolipopro	protein-coding
oxidation r	protein-coding
insulin-like	protein-coding
predicted g	protein-coding
RNA bindin	protein-coding
ribosomal p	protein-coding
Hermansky	protein-coding
spinster ho	protein-coding
protein tyr	protein-coding
transmemb	protein-coding
ribosomal p	protein-coding
membrane	protein-coding
protein tyr	protein-coding
unc-45 hor	protein-coding
ras respons	protein-coding
major facili	protein-coding
protein tyr	protein-coding
ectonucleo	protein-coding
minichrom	protein-coding
PAX interac	protein-coding
immunity-r	protein-coding
TatD DNas	protein-coding
LSM6 hom	protein-coding
argonaute	protein-coding
activin rece	protein-coding
zinc finger,	protein-coding

ribonucleo	protein-coding
SH3-domai	protein-coding
peroxisoma	protein-coding
BMP-bindi	protein-coding
death effec	protein-coding
phosphatic	protein-coding
Rho family	protein-coding
cleavage st	protein-coding
RIKEN cDN	protein-coding
EF-hand ca	protein-coding
gasdermin	protein-coding
left-right d	protein-coding
xenotropic	protein-coding
amyloid be	protein-coding
cDNA sequ	protein-coding
hyperpolar	protein-coding
SHQ1 hom	protein-coding
beta-1,3-gl	protein-coding
kelch-like 2	protein-coding
ubiquitin s	protein-coding
low density	protein-coding
cystic fibro	protein-coding
actin, beta	protein-coding
pleckstrin	protein-coding
protein kin	protein-coding
chromodor	protein-coding
jun B proto	protein-coding
WD repeat	protein-coding
RNA bindin	protein-coding
transcriptic	protein-coding
coiled-coil	protein-coding
vacuolar pr	protein-coding
ubiquitin s	protein-coding
potassium	protein-coding
inhibitor of	protein-coding
STE20-rela	protein-coding
solute carri	protein-coding
ribonucleic	protein-coding
guanylate c	protein-coding
BCL2-assoc	protein-coding
RIKEN cDN	protein-coding
immunoglo	protein-coding
SMAD fami	protein-coding
RIKEN cDN	protein-coding
deleted in l	protein-coding
KxDL motif	protein-coding
dynein ligh	protein-coding

RIKEN cDN.	protein-coding
interleukin	protein-coding
zinc finger	protein-coding
mutS homo	protein-coding
jun proto-c	protein-coding
5'-nucleoti	protein-coding
RIKEN cDN.	protein-coding
cytochrom	protein-coding
chromodor	protein-coding
recombina	protein-coding
transmeml	protein-coding
acyl-CoA sy	protein-coding
UDP-N-ace	protein-coding
phosphodi	protein-coding
dual specif	protein-coding
RNA bindin	protein-coding
t-complex	protein-coding
mitogen-ac	protein-coding
Ras associa	protein-coding
RNA bindin	protein-coding
G-rich RNA	protein-coding
MAP/micro	protein-coding
phosphatic	protein-coding
regulator o	protein-coding
septin 14	protein-coding
trans-2,3-e	protein-coding
solute carri	protein-coding
phospholip	protein-coding
minichrom	protein-coding
small nucle	protein-coding
6-phospho	protein-coding
chemokine	protein-coding
Rho GTPas	protein-coding
ubiquitin s	protein-coding
Yip1 domai	protein-coding
Werner syr	protein-coding
sel-1 suppr	protein-coding
CCR4-NOT	protein-coding
peroxisom	protein-coding
SET domair	protein-coding
RIKEN cDN.	protein-coding
tribbles ho	protein-coding
Sfi1 homol	protein-coding
split hand/	protein-coding
chemokine	protein-coding
FAST kinas	protein-coding
CD44 antig	protein-coding

ATPase, Na	protein-coding
RIKEN cDN.	protein-coding
angiopoieti	protein-coding
OCIA doma	protein-coding
endothelin	protein-coding
ChaC, catio	protein-coding
carbohydra	protein-coding
capping pr	protein-coding
cytochrom	protein-coding
polypyrimi	protein-coding
cell divisio	protein-coding
hect domai	protein-coding
WD repeat	protein-coding
uridine mo	protein-coding
lysosomal t	protein-coding
jumonji do	protein-coding
neurobeac	protein-coding
eukaryotic	protein-coding
interleukin	protein-coding
immediate	protein-coding
fibronectin	protein-coding
armadillo r	protein-coding
homeodom	protein-coding
actin-bindi	protein-coding
DnaJ (Hsp4	protein-coding
family with	protein-coding
NADH dehy	protein-coding
zinc finger	protein-coding
KH domain	protein-coding
inositol pol	protein-coding
cyclin-depe	protein-coding
RIKEN cDN.	protein-coding
podoplanir	protein-coding
MUS81 enc	protein-coding
ferredoxin	protein-coding
zinc finger	protein-coding
GINS comp	protein-coding
runt relate	protein-coding
a disintegri	protein-coding
Rho GTPas	protein-coding
enhancer c	protein-coding
protein tyr	protein-coding
SH3 domai	protein-coding
ribosomal I	protein-coding
TLR4 intera	protein-coding
2-deoxyrib	protein-coding
potassium	protein-coding

family with	protein-coding
family with	protein-coding
F-box and \	protein-coding
transmemk	protein-coding
carbohydra	protein-coding
cell adhesi	protein-coding
potassium	protein-coding
bromo adja	protein-coding
grancalcin	protein-coding
ATPase, cla	protein-coding
myelin bas	protein-coding
NADH dehy	protein-coding
ring finger	protein-coding
protein ph	protein-coding
interferon	protein-coding
phosphatic	protein-coding
coagulatior	protein-coding
ribonuclea:	protein-coding
TRAF-inter	protein-coding
ADP-ribosy	protein-coding
osteocrin	protein-coding
platelet-ac	protein-coding
adaptor-re	protein-coding
heterogene	protein-coding
zinc finger	protein-coding
pleckstrin I	protein-coding
Bardet-Bie	protein-coding
G protein-c	protein-coding
paired imn	protein-coding
Moloney sa	protein-coding
ring finger	protein-coding
staufen (RI	protein-coding
collagen, ty	protein-coding
ribosomal		protein-coding
cell divisior	protein-coding
SWI/SNF re	protein-coding
interleukin	protein-coding
vitamin K e	protein-coding
ATP/GTP b	protein-coding
T cell-inter	protein-coding
plexin C1	protein-coding
multivesicu	protein-coding
brain-speci	protein-coding
DDB1 and (	protein-coding
additional :	protein-coding
olfactory re	protein-coding
sema doma	protein-coding

ArfGAP wit	protein-coding
thymosin, I	protein-coding
SET and MY	protein-coding
hepatocyte	protein-coding
cyclin C	protein-coding
transducin-	protein-coding
phosphofru	protein-coding
RNA bindin	protein-coding
glycine dec	protein-coding
NLR family,	protein-coding
synaptosor	protein-coding
neurexin III	protein-coding
LIM domain	protein-coding
lysine (K)-s	protein-coding
BCL6 intera	protein-coding
leukotriene	protein-coding
ARV1 homo	protein-coding
potassium	protein-coding
E2F transcr	protein-coding
palmitoyl-p	protein-coding
dynactin 1	protein-coding
paired-like	protein-coding
glutathione	protein-coding
leucyl-tRNA	protein-coding
cyclin L1	protein-coding
transforma	protein-coding
CDC14 cell	protein-coding
solute carri	protein-coding
mannoside	protein-coding
protein-L-is	protein-coding
ataxin 10	protein-coding
IQ motif co	protein-coding
chloride ch	protein-coding
WD repeat	protein-coding
zinc finger	protein-coding
ENTH doma	protein-coding
transmemb	protein-coding
excision re	protein-coding
squamous	protein-coding
serpine1 m	protein-coding
protein kin	protein-coding
nucleolar p	protein-coding
tumor necr	protein-coding
pecanex ho	protein-coding
compleme	protein-coding
taperin	protein-coding
ATP syntha	protein-coding

aryl-hydroc	protein-coding
ATPase, Ca	protein-coding
arginine/se	protein-coding
P450 (cyto	protein-coding
asparagine	protein-coding
neural prec	protein-coding
taste recep	protein-coding
RIKEN cDN	protein-coding
LIM and SH	protein-coding
predicted g	protein-coding
endoplasm	protein-coding
GUF1 GTPa	protein-coding
met proto-	protein-coding
immediate	protein-coding
suppressio	protein-coding
RIKEN cDN	protein-coding
dual specif	protein-coding
gap junctio	protein-coding
zinc finger	protein-coding
excision re	protein-coding
superoxide	protein-coding
follistatin-l	protein-coding
degenerati	protein-coding
NUAK fami	protein-coding
zinc finger	protein-coding
ORAI calciu	protein-coding
solute carri	protein-coding
Mdm2, trai	protein-coding
zinc finger,	protein-coding
DEAD (Asp-	protein-coding
forkhead b	protein-coding
zinc finger	protein-coding
interleukin	protein-coding
RIKEN cDN	protein-coding
killer cell le	protein-coding
fibronectin	protein-coding
thiosulfate	protein-coding
solute carri	protein-coding
autophagy	protein-coding
proline-rich	protein-coding
RAS-like, fa	protein-coding
C-type lect	protein-coding
predicted g	protein-coding
TBC1 doma	protein-coding
Pbx/knotte	protein-coding
phosphatic	protein-coding
calcium ho	protein-coding

granzyme I	protein-coding
DiGeorge s	protein-coding
galactosida	protein-coding
C-type lect	protein-coding
Smg-6 hom	protein-coding
cystatin 10	protein-coding
zinc finger,	protein-coding
ubiquitin C	protein-coding
discs, large	protein-coding
growth arr	protein-coding
lysine (K)-s	protein-coding
bobby sox	protein-coding
coiled-coil	protein-coding
GRAM dom	protein-coding
solute carri	protein-coding
B cell trans	protein-coding
phosphodi	protein-coding
ATP-bindin	protein-coding
GINS comp	protein-coding
general tra	protein-coding
tRNA meth	protein-coding
CLPTM1-lik	protein-coding
solute carri	protein-coding
serine inco	protein-coding
zinc finger	protein-coding
UDP-glucos	protein-coding
cytochrom	protein-coding
lysophosph	protein-coding
IQ motif ar	protein-coding
transcriptic	protein-coding
guanine nu	protein-coding
vacuolar pr	protein-coding
olfactomec	protein-coding
retinoic aci	protein-coding
Ras associa	protein-coding
protein ph	protein-coding
small integ	protein-coding
zinc finger	protein-coding
colony stin	protein-coding
protein pre	protein-coding
autophagy	protein-coding
transcriptic	protein-coding
A kinase (P	protein-coding
Rho-relate	protein-coding
peptidylprc	protein-coding
orofacial cl	protein-coding
5' nucleotic	protein-coding

protein tyr	protein-coding
LPS-induce	protein-coding
transmemb	protein-coding
phospholip	protein-coding
GTPase act	protein-coding
LIM domai	protein-coding
ribosomal l	protein-coding
nardilysin,	protein-coding
calpain 8	protein-coding
SECIS bindi	protein-coding
Rho GTPase	protein-coding
killer cell le	protein-coding
KRR1, smal	protein-coding
melanoreg	protein-coding
prothymos	protein-coding
transducer	protein-coding
ATPase, Ca	protein-coding
netrin G1	protein-coding
zinc finger	protein-coding
lysine (K)-s	protein-coding
zinc finger	protein-coding
DEAD (Asp-	protein-coding
transcriptic	protein-coding
PTK2 prote	protein-coding
NLR family,	protein-coding
sortilin 1	protein-coding
nuclear fac	protein-coding
polycomb g	protein-coding
proprotein	protein-coding
cms small r	protein-coding
SET domair	protein-coding
cyclin-depe	protein-coding
ribosomal p	protein-coding
cadherin 1:	protein-coding
B cell CLL/l	protein-coding
myocardial	protein-coding
2-phospho:	protein-coding
prostaglan	protein-coding
metastasis-	protein-coding
family with	protein-coding
cadherin 2:	protein-coding
structure s	protein-coding
cyclic nucle	protein-coding
glia matura	protein-coding
suppressor	protein-coding
ring finger	protein-coding
interleukin	protein-coding

S100 calciu	protein-coding
mannosida	protein-coding
somatostat	protein-coding
ribosomal	protein-coding
WD repeat	protein-coding
neural prec	protein-coding
phosphoin	protein-coding
adenosine	protein-coding
translocate	protein-coding
regulator o	protein-coding
proteasom	protein-coding
CD81 antig	protein-coding
RIKEN cDN.	protein-coding
phosphatic	protein-coding
kelch-like E	protein-coding
chemokine	protein-coding
phosphodi	protein-coding
phosphodi	protein-coding
fos-like ant	protein-coding
interleukin	protein-coding
zinc finger,	protein-coding
zinc finger	protein-coding
nucleopori	protein-coding
chondroitir	protein-coding
interleukin	protein-coding
poly(A) bin	protein-coding
RuvB-like p	protein-coding
mitogen-ac	protein-coding
predicted g	protein-coding
RAD51 hon	protein-coding
deformed e	protein-coding
ribosomal	protein-coding
solute carri	protein-coding
cyclin E1	protein-coding
mitochond	protein-coding
family with	protein-coding
anterior gr	protein-coding
dCMP dear	protein-coding
RCSD doma	protein-coding
RIKEN cDN.	protein-coding
small nucle	protein-coding
NHL repeat	protein-coding
BCL2-assoc	protein-coding
otogelin-lik	protein-coding
ubiquitin-c	protein-coding
CDC14 cell	protein-coding
thymocyte	protein-coding

zinc finger	protein-coding
anaphase p	protein-coding
ATPase, cla	protein-coding
PARK2 co-r	protein-coding
NADH dehy	protein-coding
ankyrin rep	protein-coding
cornichon l	protein-coding
coiled-coil	protein-coding
selenophos	protein-coding
transporter	protein-coding
E74-like fac	protein-coding
low density	protein-coding
neurogenin	protein-coding
DDB1 and C	protein-coding
solute carri	protein-coding
eukaryotic	protein-coding
cysteine-ric	protein-coding
RIKEN cDNA	protein-coding
density-reg	protein-coding
DNA segme	protein-coding
ribosomal p	protein-coding
RAN bindin	protein-coding
adrenergic	protein-coding
2-oxogluta	protein-coding
metallothio	protein-coding
sushi, von W	protein-coding
cadherin 4	protein-coding
zinc finger	protein-coding
regulator o	protein-coding
telomerase	protein-coding
mastermin	protein-coding
Rho guanin	protein-coding
leucine-rich	protein-coding
ADP-ribosy	protein-coding
OTU domai	protein-coding
zinc finger	protein-coding
ArfGAP wit	protein-coding
integrin lin	protein-coding
transmemb	protein-coding
t-complex p	protein-coding
sphingosine	protein-coding
crystallin, g	protein-coding
actin filame	protein-coding
N(alpha)-ac	protein-coding
alkylglycer	protein-coding
mediator c	protein-coding
phospholip	protein-coding

coenzyme (	protein-coding
2-oxogluta	protein-coding
solute carri	protein-coding
dystroglyca	protein-coding
transmemb	protein-coding
coagulatior	protein-coding
transaldola	protein-coding
tetratricop	protein-coding
aldehyde d	protein-coding
nucleic acic	protein-coding
ATP-bindin	protein-coding
solute carri	protein-coding
protein kin	protein-coding
centrosom	protein-coding
NOP2 nucle	protein-coding
glycosyltra	protein-coding
Ral GTPase	protein-coding
serine/argi	protein-coding
nuclear fac	protein-coding
cortactin	protein-coding
B cell leuke	protein-coding
SUN domai	protein-coding
BCDIN3 do	protein-coding
nucleopori	protein-coding
fibronectin	protein-coding
pentatricop	protein-coding
COMM dor	protein-coding
neutrophil	protein-coding
SECIS bindi	protein-coding
septin 9	protein-coding
FMS-like ty	protein-coding
CD300 anti	protein-coding
tRNA splici	protein-coding
Rho GTPase	protein-coding
dedicator c	protein-coding
predicted g	protein-coding
coxsackie v	protein-coding
zinc finger	protein-coding
4-hydroxyp	protein-coding
eukaryotic	protein-coding
phosphodi	protein-coding
potassium	protein-coding
mitochond	protein-coding
melatonin	protein-coding
Nedd4 fam	protein-coding
mitochond	protein-coding
nucleotide-	protein-coding

prostaglan	protein-coding
ankyrin rep	protein-coding
DNA segme	protein-coding
avian musc	protein-coding
tripartite m	protein-coding
mitochond	protein-coding
dual-specif	protein-coding
SR-related	protein-coding
coiled-coil	protein-coding
glutamic py	protein-coding
calumenin	protein-coding
forkhead b	protein-coding
F-box and W	protein-coding
Jun dimeriz	protein-coding
synaptotag	protein-coding
cyclin A1	protein-coding
carboxypep	protein-coding
nuclear fra	protein-coding
NLR family,	protein-coding
neurofilam	protein-coding
ATP-bindin	protein-coding
Rho guanin	protein-coding
glutamate-	protein-coding
diacylglyce	protein-coding
CDK5 regul	protein-coding
F-box and l	protein-coding
egl-9 family	protein-coding
Rho-relatec	protein-coding
splicing fac	protein-coding
solute carri	protein-coding
euchromat	protein-coding
tudor doma	protein-coding
AT hook, D	protein-coding
testis expre	protein-coding
lectin, gala	protein-coding
inositol 1,4	protein-coding
solute carri	protein-coding
UHRF1 (ICE	protein-coding
RAN, meml	protein-coding
galanin	protein-coding
solute carri	protein-coding
polymerase	protein-coding
membrane	protein-coding
influenza v	protein-coding
guanine nu	protein-coding
calsyntenir	protein-coding
K(lysine) ac	protein-coding

bone marr	protein-coding
Hedgehog-	protein-coding
ORAI calciu	protein-coding
N(alpha)-ac	protein-coding
bromodom	protein-coding
CUB and Su	protein-coding
sphingosin	protein-coding
jade family	protein-coding
nuclear tra	protein-coding
zinc finger	protein-coding
zinc finger	protein-coding
zinc finger	protein-coding
piezo-type	protein-coding
transketola	protein-coding
FK506 bind	protein-coding
CD38 antig	protein-coding
DENN/MAI	protein-coding
WD repeat	protein-coding
X-ray repai	protein-coding
zinc finger	protein-coding
gelsolin	protein-coding
E26 avian l	protein-coding
rhotekin 2	protein-coding
OTU domai	protein-coding
caspase 2	protein-coding
WD repeat	protein-coding
tribbles ho	protein-coding
alkylglycer	protein-coding
zinc finger	protein-coding
COX20 Cox	protein-coding
vacuolar pr	protein-coding
RIKEN cDN	protein-coding
cofilin 1, n	protein-coding
nuclear rec	protein-coding
interleukin	protein-coding
dedicator c	protein-coding
neuron der	protein-coding
claudin 15	protein-coding
adenosine	protein-coding
spermatog	protein-coding
phosphatas	protein-coding
sorting nex	protein-coding
DnaJ (Hsp4	protein-coding
spectrin re	protein-coding
spleen focu	protein-coding
UDP-GlcNA	protein-coding
forkhead b	protein-coding

phosphatas	protein-coding
thrombosp	protein-coding
diaphanous	protein-coding
inhibitor of	protein-coding
rhomboid 5	protein-coding
RIKEN cDNA	protein-coding
WD repeat	protein-coding
solute carri	protein-coding
protein pho	protein-coding
paired box	protein-coding
arginyltran	protein-coding
angiotensin	protein-coding
ubiquitin p	protein-coding
ankyrin rep	protein-coding
NEDD4 bin	protein-coding
zinc finger	protein-coding
pyrophospl	protein-coding
proteasom	protein-coding
migration a	protein-coding
immediate	protein-coding
CTD (carbo	protein-coding
crystallin, z	protein-coding
protein tyr	protein-coding
PRAME fan	protein-coding
coiled-coil	protein-coding
UV radiatic	protein-coding
Myb/SANT	protein-coding
SMAD fami	protein-coding
zinc finger	protein-coding
ankyrin rep	protein-coding
Rho GTPas	protein-coding
caspase 1	protein-coding
CUE domai	protein-coding
NEDD4 bin	protein-coding
hexamethy	protein-coding
thrombom	protein-coding
transmemb	protein-coding
sortilin-rela	protein-coding
BMP and a	protein-coding
leptin rece	protein-coding
endo-beta-	protein-coding
janus kinas	protein-coding
salt inducib	protein-coding
YEATS dom	protein-coding
methylster	protein-coding
glucoside x	protein-coding
G protein-c	protein-coding

glial cells m	protein-coding
tyrosine 3-	protein-coding
expressed :	protein-coding
developme	protein-coding
myosin, he	protein-coding
zinc finger	protein-coding
H3 histone,	protein-coding
angiopoieti	protein-coding
sestrin 3	protein-coding
suppressor	protein-coding
ribosomal p	protein-coding
growth arr	protein-coding
eukaryotic	protein-coding
SRY (sex de	protein-coding
UFM1-spec	protein-coding
Nipped-B h	protein-coding
akirin 2	protein-coding
exportin, tl	protein-coding
golgi appar	protein-coding
transmemb	protein-coding
thymoma v	protein-coding
mitogen-ac	protein-coding
killer cell le	protein-coding
family with	protein-coding
myotubula	protein-coding
ubiquitin sp	protein-coding
SUMO/sen	protein-coding
adenylosuc	protein-coding
RIKEN cDN.	protein-coding
GDP-mann	protein-coding
placenta ex	protein-coding
haptoglobi	protein-coding
nucleopori	protein-coding
ubiquitin-c	protein-coding
zinc finger	protein-coding
axin 1	protein-coding
DENN/MAI	protein-coding
S100 calciu	protein-coding
PTC7 prote	protein-coding
myosin, he	protein-coding
RIKEN cDN.	protein-coding
phorbol-12	protein-coding
Fc receptoi	protein-coding
abhydrolas	protein-coding
ras homolo	protein-coding
ring finger	protein-coding
insulin rece	protein-coding

proprotein	protein-coding
NCK-associ	protein-coding
aquaporin	protein-coding
C-type lect	protein-coding
TRAF-intera	protein-coding
predicted g	protein-coding
leucine zipp	protein-coding
eukaryotic	protein-coding
ribosomal r	protein-coding
THUMP do	protein-coding
transmemb	protein-coding
zinc finger	protein-coding
C-type lect	protein-coding
heat shock	protein-coding
KAT8 regul	protein-coding
TruB pseuc	protein-coding
thyrotroph	protein-coding
Ras-like wit	protein-coding
transforme	protein-coding
solute carri	protein-coding
TBC1 doma	protein-coding
acyl-Coenz	protein-coding
crumbs hoi	protein-coding
muscleblin	protein-coding
fucosyltran	protein-coding
chitinase-li	protein-coding
acyl-Coenz	protein-coding
sorcin	protein-coding
DEAD (Asp-	protein-coding
large tumo	protein-coding
dynactin 4	protein-coding
monocyte t	protein-coding
BCL2-like 1	protein-coding
dehydroge	protein-coding
family with	protein-coding
family with	protein-coding
zinc finger	protein-coding
zinc finger	protein-coding
phosphatic	protein-coding
arginyl ami	protein-coding
integrin be	protein-coding
uncoupling	protein-coding
phosphopr	protein-coding
muscleblin	protein-coding
NIMA (neve	protein-coding
RIKEN cDN	protein-coding
mediator c	protein-coding

cornichon l	protein-coding
plexin D1	protein-coding
CGG triplet	protein-coding
T cell, imm	protein-coding
Rap guanin	protein-coding
tumor necr	protein-coding
transmemb	protein-coding
PHD finger	protein-coding
ferric-chela	protein-coding
coiled-coil	protein-coding
proline-rich	protein-coding
2-phospho:	protein-coding
WAP four-c	protein-coding
myosin, he	protein-coding
SCAN doma	protein-coding
aspartyl-tR	protein-coding
SWI/SNF re	protein-coding
DCN1, defe	protein-coding
X Kell bloo	protein-coding
platelet-ac	protein-coding
zinc finger,	protein-coding
X-ray repai	protein-coding
CD302 anti	protein-coding
SWA-70 pr	protein-coding
DnaJ (Hsp4	protein-coding
UTP23, sm	protein-coding
zinc finger	protein-coding
calcium/ca	protein-coding
ELAV (emb	protein-coding
stromal int	protein-coding
G protein-c	protein-coding
nuclear rec	protein-coding
nudix (nucl	protein-coding
ATP-bindin	protein-coding
transcriptic	protein-coding
mitochond	protein-coding
solute carri	protein-coding
fucosyltran	protein-coding
ring finger	protein-coding
osteoglycir	protein-coding
purinergic	protein-coding
RIKEN cDN	protein-coding
leucyl/cysti	protein-coding
plasminoge	protein-coding
radical S-ac	protein-coding
ras homolc	protein-coding
tumor necr	protein-coding

LYR motif c	protein-coding
nibrin	protein-coding
ubiquitin p	protein-coding
NADH dehy	protein-coding
dual serine	protein-coding
origin reco	protein-coding
HemK metl	protein-coding
lin-7 homo	protein-coding
transmemb	protein-coding
iron-sulfur	protein-coding
prostaglan	protein-coding
lectin, gala	protein-coding
mitogen-ac	protein-coding
interferon	protein-coding
carboxypep	protein-coding
forty-two-t	protein-coding
tyrosyl-tRN	protein-coding
zinc finger	protein-coding
cytochrom	protein-coding
ribose 5-ph	protein-coding
TEL2, telon	protein-coding
sciellin	protein-coding
insulin-like	protein-coding
karyopheri	protein-coding
myristoylat	protein-coding
G protein-c	protein-coding
pyruvate d	protein-coding
S100 calciu	protein-coding
doublesex	protein-coding
acyl-CoA th	protein-coding
cortactin b	protein-coding
parathyroi	protein-coding
7-dehydro	protein-coding
glyceropho	protein-coding
catenin (ca	protein-coding
jade family	protein-coding
protein tyr	protein-coding
xanthine d	protein-coding
WD repeat	protein-coding
autophagy	protein-coding
mex3 hom	protein-coding
protein tyr	protein-coding
gamma-sec	protein-coding
G protein-c	protein-coding
bromodom	protein-coding
DnaJ (Hsp4	protein-coding
family with	protein-coding

cytidine mc	protein-coding
signal trans	protein-coding
non-catalyt	protein-coding
D-2-hydrox	protein-coding
coiled-coil	protein-coding
SH2 domai	protein-coding
ribonuclea:	protein-coding
protein tyr	protein-coding
transducin	protein-coding
solute carri	protein-coding
proline syn	protein-coding
non-SMC c	protein-coding
UV radiatic	protein-coding
protease, s	protein-coding
LSM6 hom	protein-coding
ring finger	protein-coding
barrier to a	protein-coding
proline deh	protein-coding
EH domain	protein-coding
ring finger	protein-coding
REV1 homc	protein-coding
AT rich inte	protein-coding
kinesin fam	protein-coding
arginine glu	protein-coding
leucine rich	protein-coding
heat shock	protein-coding
IQ motif co	protein-coding
latrophilin	protein-coding
solute carri	protein-coding
signal reco	protein-coding
tubulin, alp	protein-coding
glycine C-a	protein-coding
peroxiredo	protein-coding
peroxisom	protein-coding
jade family	protein-coding
heterogen	protein-coding
interleukin	protein-coding
F-box prot	protein-coding
OVO homo	protein-coding
transmemb	protein-coding
CCZ1 vacuc	protein-coding
sphingomy	protein-coding
calcium an	protein-coding
RIKEN cDN	protein-coding
protease, s	protein-coding
ATPase, H+	protein-coding
hematopoi	protein-coding

a disintegri	protein-coding
myozenin 1	protein-coding
signal trans	protein-coding
C-terminal	protein-coding
monocyte t	protein-coding
ring-box 1	protein-coding
nuclear rec	protein-coding
proteasom	protein-coding
MTOR asso	protein-coding
guanine mo	protein-coding
fin bud init	protein-coding
phosphatid	protein-coding
GTP cycloh	protein-coding
X Kell blood	protein-coding
ERO1-like (	protein-coding
RNA bindin	protein-coding
cytochrome	protein-coding
Abelson he	protein-coding
glutamate	protein-coding
transcriptio	protein-coding
Cbp/p300-i	protein-coding
muscle glyc	protein-coding
coiled-coil	protein-coding
COMM dom	protein-coding
chromodom	protein-coding
mitochond	protein-coding
Rho GTPase	protein-coding
serine (or c	protein-coding
casein kina	protein-coding
WAP four-c	protein-coding
nicotinami	protein-coding
THO compl	protein-coding
karyopheri	protein-coding
guanine de	protein-coding
protease, s	protein-coding
acyl-Coenz	protein-coding
AP2 associa	protein-coding
gem (nucle	protein-coding
kinectin 1	protein-coding
LEM domai	protein-coding
Kruppel-lik	protein-coding
CDC42 effe	protein-coding
expressed s	protein-coding
regulator o	protein-coding
MOK prote	protein-coding
cAMP resp	protein-coding
TBC1 doma	protein-coding

PAP associ	protein-coding
TAF5-like R	protein-coding
CCR4-NOT	protein-coding
FGFR1 onc	protein-coding
potassium	protein-coding
pyruvate d	protein-coding
ankyrin rep	protein-coding
RIKEN cDN	protein-coding
leiomodin	protein-coding
general tra	protein-coding
ubiquitin-c	protein-coding
zinc finger	protein-coding
polynucleo	protein-coding
transmemb	protein-coding
baculoviral	protein-coding
sushi doma	protein-coding
poly(rC) bi	protein-coding
CCCTC-binc	protein-coding
vomeronas	protein-coding
solute carri	protein-coding
exocyst co	protein-coding
ATP-bindin	protein-coding
protein arg	protein-coding
otolin 1 ho	protein-coding
coiled-coil	protein-coding
Y box prote	protein-coding
X-box bindi	protein-coding
lipoyl(octa	protein-coding
tetratricop	protein-coding
family with	protein-coding
selenophos	protein-coding
S-adenosyl	protein-coding
chromodor	protein-coding
bone morp	protein-coding
poly (ADP-	protein-coding
serpine1 m	protein-coding
acyl-CoA sy	protein-coding
ets variant	protein-coding
ATPase, H+	protein-coding
CDC42 effe	protein-coding
v-maf mus	protein-coding
oxysterol b	protein-coding
prothymos	protein-coding
nuclear rec	protein-coding
MOB kinas	protein-coding
purinergic	protein-coding
AF4/FMR2	protein-coding

runt relate	protein-coding
ring finger	protein-coding
myelin bas	protein-coding
solute carri	protein-coding
solute carri	protein-coding
importin 1:	protein-coding
jagunal hor	protein-coding
YY1 transcr	protein-coding
zinc finger	protein-coding
Aly/REF ex	protein-coding
SCY1-like 3	protein-coding
ubiquitin s	protein-coding
supervillin	protein-coding
UDP-GlcNA	protein-coding
transcriptic	protein-coding
N-acetylglu	protein-coding
activating t	protein-coding
phosphatic	protein-coding
prostaglan	protein-coding
kelch-like 2	protein-coding
zinc finger	protein-coding
nuclear fac	protein-coding
TNF recept	protein-coding
LPS-respon	protein-coding
nuclear poi	protein-coding
trans-actin	protein-coding
dynein, axc	protein-coding
notch 2	protein-coding
interleukin	protein-coding
transmemb	protein-coding
family with	protein-coding
RIKEN cDN	protein-coding
RAB22A, m	protein-coding
DDB1 and	protein-coding
Kruppel-lik	protein-coding
FCH domai	protein-coding
NOL1/NOP	protein-coding
cadherin, E	protein-coding
vitamin K e	protein-coding
enoyl Coen	protein-coding
elastin mic	protein-coding
RIKEN cDN	protein-coding
mitochond	protein-coding
cathepsin Z	protein-coding
secretin re	protein-coding
AT hook co	protein-coding
calcium rel	protein-coding

vaccinia rel	protein-coding
sprouty-rel	protein-coding
TRM5 tRNA	protein-coding
ribosomal p	protein-coding
nicotinami	protein-coding
casein kina	protein-coding
ubiquitin sp	protein-coding
LanC (bacte	protein-coding
family with	protein-coding
transmemb	protein-coding
calcium cha	protein-coding
F-box and l	protein-coding
Luc7 homo	protein-coding
CXADR-like	protein-coding
ADP-ribosy	protein-coding
family with	protein-coding
cell divisior	protein-coding
apolipopro	protein-coding
RIKEN cDNA	protein-coding
insulin deg	protein-coding
intraflagell	protein-coding
cyclin-depe	protein-coding
kinesin ligh	protein-coding
lysozyme 2	protein-coding
castor zinc	protein-coding
phosphatic	protein-coding
BMS1 hom	protein-coding
zinc finger	protein-coding
prothymos	protein-coding
oxysterol b	protein-coding
ArfGAP wit	protein-coding
glutamate	protein-coding
inositol 1,4	protein-coding
ataxin 2	protein-coding
transducin-	protein-coding
carbohydra	protein-coding
cyclin N-ter	protein-coding
forkhead b	protein-coding
calcium an	protein-coding
RMI1, RecQ	protein-coding
lysine (K)-s	protein-coding
Rap guanin	protein-coding
hemochror	protein-coding
atonal hom	protein-coding
ubiquitin sp	protein-coding
vasodilator	protein-coding
homeobox	protein-coding

pleckstrin h	protein-coding
early endos	protein-coding
N-myc dow	protein-coding
ribosomal p	protein-coding
LIM domain	protein-coding
FH2 domai	protein-coding
retinoblast	protein-coding
mal, T cell d	protein-coding
nuclear rec	protein-coding
phospholip	protein-coding
CD36 antig	protein-coding
N-acetylglu	protein-coding
Rho guanin	protein-coding
UDP-Gal:be	protein-coding
glucocortic	protein-coding
DnaJ (Hsp4	protein-coding
vacuolar pr	protein-coding
solute carri	protein-coding
coiled-coil	protein-coding
mastermin	protein-coding
potassium	protein-coding
signal trans	protein-coding
peroxisome	protein-coding
phosphoino	protein-coding
actin filame	protein-coding
phosphatas	protein-coding
protein kin	protein-coding
chaperonin	protein-coding
syntaxin bi	protein-coding
solute carri	protein-coding
dihydrolipo	protein-coding
zinc finger	protein-coding
scavenger r	protein-coding
suppressor	protein-coding
dihydrouric	protein-coding
integrator c	protein-coding
solute carri	protein-coding
RIKEN cDNA	protein-coding
intraflagella	protein-coding
ataxin 7-lik	protein-coding
ubiquitin-c	protein-coding
high mobili	protein-coding
SUMO1/se	protein-coding
signal-indu	protein-coding
muscleblin	protein-coding
adaptor pro	protein-coding
hydroxyste	protein-coding

KN motif ar	protein-coding
Tctex1 dom	protein-coding
K(lysine) ac	protein-coding
diazepam b	protein-coding
tetratricop	protein-coding
serine/thre	protein-coding
aldo-keto r	protein-coding
leucine rich	protein-coding
zinc finger	protein-coding
thiosulfate	protein-coding
teneurin tr	protein-coding
chondroitin	protein-coding
DEP domai	protein-coding
phospholip	protein-coding
guanine nu	protein-coding
DEAH (Asp-	protein-coding
solute carri	protein-coding
ras respons	protein-coding
forkhead b	protein-coding
zinc finger	protein-coding
ribosomal p	protein-coding
chloride ch	protein-coding
EF-hand ca	protein-coding
solute carri	protein-coding
chemokine	protein-coding
zinc finger	protein-coding
PHD finger	protein-coding
heart and r	protein-coding
syntaxin bi	protein-coding
DNA methy	protein-coding
spermatog	protein-coding
ribosomal p	protein-coding
protein pho	protein-coding
cyclin-depe	protein-coding
oxysterol b	protein-coding
paired-like	protein-coding
PHD finger	protein-coding
WD repeat	protein-coding
K(lysine) ac	protein-coding
small nucle	protein-coding
bradykinin	protein-coding
charged mu	protein-coding
topoisome	protein-coding
latrophilin	protein-coding
Kruppel-lik	protein-coding
UDP-glucur	protein-coding
leucine-rich	protein-coding

integrin-lin	protein-coding
protease, s	protein-coding
genetic sup	protein-coding
leucine zip	protein-coding
Pigy upstre	protein-coding
aldehyde d	protein-coding
histone clu	protein-coding
ATP/GTP b	protein-coding
GLTSCR1-li	protein-coding
cache dom	protein-coding
heat shock	protein-coding
family with	protein-coding
abhydrolas	protein-coding
ZXD family	protein-coding
low density	protein-coding
protein ph	protein-coding
phosphatas	protein-coding
protein ph	protein-coding
Rap guanin	protein-coding
nucleopori	protein-coding
glycogenin	protein-coding
tropomyos	protein-coding
ubiquitin C	protein-coding
basic leucir	protein-coding
mitochond	protein-coding
spleen foc	protein-coding
diacylglyce	protein-coding
trafficking	protein-coding
transformi	protein-coding
X-ray repai	protein-coding
potassium	protein-coding
myotubula	protein-coding
leucine rich	protein-coding
NLR family,	protein-coding
retinitis pig	protein-coding
PDZ and LII	protein-coding
translocase	protein-coding
ELAV (emb	protein-coding
SHQ1 hom	protein-coding
nuclear rec	protein-coding
protocadh	protein-coding
speedy hor	protein-coding
target of m	protein-coding
A kinase (P	protein-coding
solute carri	protein-coding
compleme	protein-coding
ubiquitin p	protein-coding

Smad nucle	protein-coding
homeodom	protein-coding
formin-like	protein-coding
Ras and Ra	protein-coding
tripartite m	protein-coding
CD33 antig	protein-coding
retinoic aci	protein-coding
acidic (leuc	protein-coding
TGFB-induc	protein-coding
inositol pol	protein-coding
solute carri	protein-coding
DENN/MAI	protein-coding
proline-rich	protein-coding
zinc finger,	protein-coding
ribosomal p	protein-coding
ninein	protein-coding
cyclin-depe	protein-coding
nuclear fac	protein-coding
ribonucleas	protein-coding
cell division	protein-coding
AT hook co	protein-coding
succinyl-Co	protein-coding
membrane	protein-coding
talin 1	protein-coding
tripartite m	protein-coding
C-type lect	protein-coding
calcium ho	protein-coding
RIKEN cDN.	protein-coding
glycerol ph	protein-coding
homeodom	protein-coding
ADAMTS-lil	protein-coding
mitochond	protein-coding
ring finger	protein-coding
mannoside	protein-coding
kinesin fam	protein-coding
sulfatase m	protein-coding
kelch-like 6	protein-coding
CART prepr	protein-coding
CCR4-NOT	protein-coding
glycoprotei	protein-coding
GLIS family	protein-coding
phospholip	protein-coding
translocase	protein-coding
Ewing sarc	protein-coding
transforme	protein-coding
oxidized lo	protein-coding
potassium	protein-coding

cadherin 6	protein-coding
TBC1 doma	protein-coding
RIKEN cDN.	protein-coding
NADH dehy	protein-coding
ribosomal p	protein-coding
componen	protein-coding
family with	protein-coding
torsin A int	protein-coding
signal-regu	protein-coding
acyl-CoA sy	protein-coding
cathepsin C	protein-coding
TBC1 doma	protein-coding
RNA bindin	protein-coding
ribonuclea:	protein-coding
proteasom	protein-coding
required fc	protein-coding
period circ	protein-coding
procollager	protein-coding
serine (or c	protein-coding
lysine (K)-s	protein-coding
cadherin 1!	protein-coding
TRAF famil	protein-coding
hook homc	protein-coding
septin 9	protein-coding
zinc finger	protein-coding
carbonyl re	protein-coding
thyroid hor	protein-coding
lymphoid e	protein-coding
cerebral ca	protein-coding
family with	protein-coding
DENN/MAI	protein-coding
leukocyte r	protein-coding
kinesin fan	protein-coding
jade family	protein-coding
Cbp/p300-i	protein-coding
G protein-c	protein-coding
glutamine a	protein-coding
M phase pl	protein-coding
MICAL-like	protein-coding
TBC1 doma	protein-coding
cell divisior	protein-coding
rearranged	protein-coding
centrosom	protein-coding
UDP-Gal:be	protein-coding
cyclin G ass	protein-coding
HECT doma	protein-coding
RIKEN cDN.	protein-coding

coiled-coil	protein-coding
Shc SH2-dc	protein-coding
tubulin tyr	protein-coding
E74-like fac	protein-coding
centromer	protein-coding
tRNA aspar	protein-coding
transmemb	protein-coding
striatin int	protein-coding
transient re	protein-coding
protein ph	protein-coding
PR domain	protein-coding
insulin-like	protein-coding
transient re	protein-coding
RUN and F	protein-coding
vav 2 onco	protein-coding
HOP home	protein-coding
a disintegri	protein-coding
spectrin re	protein-coding
family with	protein-coding
neutrophili	protein-coding
transcriptic	protein-coding
furin (paire	protein-coding
nicotinami	protein-coding
Ral GTPase	protein-coding
inhibitor of	protein-coding
maturin, ne	protein-coding
RAS relatec	protein-coding
ATPase typ	protein-coding
forkhead b	protein-coding
RIMS bindi	protein-coding
serine (or c	protein-coding
transformi	protein-coding
transmemb	protein-coding
Smith-Mag	protein-coding
transmemb	protein-coding
glutamyl-tF	protein-coding
interferon	protein-coding
tubulin, ga	protein-coding
LIM domai	protein-coding
dehydroge	protein-coding
potassium	protein-coding
calcyclin bi	protein-coding
integrin be	protein-coding
zinc finger,	protein-coding
G patch do	protein-coding
tetratricop	protein-coding
retinol deh	protein-coding

tumor necr	protein-coding
BCL2-like 1	protein-coding
protein kin	protein-coding
dual adapt	protein-coding
utrophin	protein-coding
prickle hon	protein-coding
DEAH (Asp-	protein-coding
apelin rece	protein-coding
survival mc	protein-coding
adducin 3 (	protein-coding
stefin A1	protein-coding
Ras homolo	protein-coding
Hus1 homc	protein-coding
zinc finger	protein-coding
myosin, ligl	protein-coding
methyltran	protein-coding
tetraspanir	protein-coding
calcineurin	protein-coding
interferon	protein-coding
RNA bindin	protein-coding
amyloid be	protein-coding
rabaptin, R	protein-coding
neuregulin	protein-coding
K(lysine) ac	protein-coding
serglycin	protein-coding
solute carri	protein-coding
interleukin	protein-coding
transmemk	protein-coding
histamine I	protein-coding
ras homolo	protein-coding
ubiquitin-c	protein-coding
protein kin	protein-coding
degenerati	protein-coding
testis expre	protein-coding
zinc finger	protein-coding
interleukin	protein-coding
TBC1 doma	protein-coding
transportin	protein-coding
glutamine-	protein-coding
syntrophin,	protein-coding
pleckstrin a	protein-coding
nucleolar p	protein-coding
protein pho	protein-coding
RIKEN cDN.	protein-coding
methylpho	protein-coding
casein kina	protein-coding
chloride int	protein-coding

solute carri	protein-coding
microtubul	protein-coding
mitochond	protein-coding
RNA bindin	protein-coding
carbohydra	protein-coding
GLI pathog	protein-coding
cryptochro	protein-coding
glycosylpho	protein-coding
glutaminas	protein-coding
outer dens	protein-coding
Meis home	protein-coding
cyclin-depe	protein-coding
glypican 1	protein-coding
zinc finger,	protein-coding
DnaJ (Hsp4	protein-coding
platelet-de	protein-coding
SPRY doma	protein-coding
ubiquitin fa	protein-coding
eukaryotic	protein-coding
cell divisior	protein-coding
endoplasm	protein-coding
Ras-relatec	protein-coding
cytohesin 1	protein-coding
NOP56 ribc	protein-coding
RIKEN cDN.	protein-coding
SH3 domai	protein-coding
NLR family,	protein-coding
methyltran	protein-coding
basic leucir	protein-coding
lysophosph	protein-coding
RIKEN cDN.	protein-coding
calpain 10	protein-coding
actin, beta	protein-coding
sperm anti	protein-coding
histocompa	protein-coding
calcium ch	protein-coding
RE1-silenci	protein-coding
AT-hook tr	protein-coding
UDP-glucos	protein-coding
homeobox	protein-coding
ribosomal	protein-coding
CLP1, cleav	protein-coding
destrin	protein-coding
androgen c	protein-coding
interleukin	protein-coding
adrenergic	protein-coding
hemochror	protein-coding

selenophos	protein-coding
polymerase	protein-coding
ubiquinol-c	protein-coding
RAB18, me	protein-coding
mitochond	protein-coding
DDHD dom	protein-coding
LIM domai	protein-coding
family with	protein-coding
Mediterrar	protein-coding
RIKEN cDN.	protein-coding
lectin, gala	protein-coding
arrestin do	protein-coding
UDP-Gal:be	protein-coding
collagen, ty	protein-coding
platelet/en	protein-coding
insulin-like	protein-coding
zinc finger,	protein-coding
aminopept	protein-coding
fibrillin 1	protein-coding
RIKEN cDN.	protein-coding
latrophilin	protein-coding
family with	protein-coding
ankyrin rep	protein-coding
high mobili	protein-coding
chaperonir	protein-coding
methyltran	protein-coding
major facili	protein-coding
low density	protein-coding
pleckstrin h	protein-coding
solute carri	protein-coding
annexin A2	protein-coding
bystin-like	protein-coding
general tra	protein-coding
inositol 1,4	protein-coding
tumor necr	protein-coding
SDE2 telom	protein-coding
Fas (TNF re	protein-coding
neutrophil	protein-coding
sortilin-rela	protein-coding
interleukin	protein-coding
serine (or c	protein-coding
ADP-ribosy	protein-coding
transmemb	protein-coding
PX domain	protein-coding
centriolin	protein-coding
proline rich	protein-coding
mitogen-ac	protein-coding

zinc finger	protein-coding
coenzyme (	protein-coding
olfactory re	protein-coding
heterogene	protein-coding
ARP3 actin-	protein-coding
NudC doma	protein-coding
G patch do	protein-coding
ribosomal p	protein-coding
ceramide s	protein-coding
vascular en	protein-coding
predicted g	protein-coding
FYVE, RhoG	protein-coding
chemokine	protein-coding
grancalcin	protein-coding
aldehyde d	protein-coding
Zinc finger	protein-coding
runt relate	protein-coding
solute carri	protein-coding
RAB2A, me	protein-coding
F-box and W	protein-coding
sulfide quir	protein-coding
cytochrome	protein-coding
prostagland	protein-coding
heat-respo	protein-coding
GTPase, IM	protein-coding
nuclear cas	protein-coding
engulfment	protein-coding
nuclear rec	protein-coding
glucuronida	protein-coding
ribonucleas	protein-coding
high-mobil	protein-coding
carcinoemb	protein-coding
F-box prote	protein-coding
sosondowa	protein-coding
tRNA-histic	protein-coding
HtrA serine	protein-coding
solute carri	protein-coding
F-box prote	protein-coding
testis expre	protein-coding
zinc finger	protein-coding
transcriptio	protein-coding
neutrophil	protein-coding
fibronectin	protein-coding
elastase, ne	protein-coding
transmemb	protein-coding
translocase	protein-coding
transducin-	protein-coding

metastasis-	protein-coding
RIKEN cDN.	protein-coding
serine/argi	protein-coding
serum/gluc	protein-coding
bromodom	protein-coding
NADH dehy	protein-coding
transmemk	protein-coding
transmemk	protein-coding
transmemk	protein-coding
GAR1 ribor	protein-coding
family with	protein-coding
tyrosine 3-	protein-coding
proline-ser	protein-coding
protein dis	protein-coding
ficolin B	protein-coding
coagulatior	protein-coding
membrane	protein-coding
family with	protein-coding
fer (fms/fp	protein-coding
Mab-21 do	protein-coding
cAMP resp	protein-coding
DDB1 and (	protein-coding
otogelin-lik	protein-coding
WD repeat	protein-coding
chromatin	protein-coding
dipeptidylp	protein-coding
growth diff	protein-coding
sphingomy	protein-coding
transmemk	protein-coding
serine/thre	protein-coding
zinc finger	protein-coding
NADH dehy	protein-coding
lymphoblas	protein-coding
transforma	protein-coding
NUF2, NDC	protein-coding
DET1 and D	protein-coding
vacuolar pr	protein-coding
calcium cha	protein-coding
phospholys	protein-coding
CASP2 and	protein-coding
carbohydra	protein-coding
adenylate l	protein-coding
oxidative s	protein-coding
DNA methy	protein-coding
LIM domai	protein-coding
autophagy	protein-coding
methionine	protein-coding

dolichol-ph	protein-coding
inhibin bet	protein-coding
ATPase fan	protein-coding
aprataxin a	protein-coding
RAP1 GTPa	protein-coding
biogenesis	protein-coding
zinc finger	protein-coding
zinc finger	protein-coding
GRAM dom	protein-coding
ribophorin	protein-coding
suppressor	protein-coding
Kruppel-lik	protein-coding
stromal int	protein-coding
eukaryotic	protein-coding
G elongatic	protein-coding
CDC14 cell	protein-coding
Meis home	protein-coding
signal-indu	protein-coding
prostaglan	protein-coding
suppressor	protein-coding
protein ph	protein-coding
thyrotroph	protein-coding
oxysterol b	protein-coding
UDP-Gal:be	protein-coding
ATP-bindin	protein-coding
placenta e	protein-coding
CAP-GLY do	protein-coding
relaxin/ins	protein-coding
coactosin-l	protein-coding
defective ir	protein-coding
PWWP dor	protein-coding
deleted in l	protein-coding
electron tr	protein-coding
fucosyltran	protein-coding
vacuolar pr	protein-coding
growth arr	protein-coding
dihydrolipc	protein-coding
F-box prote	protein-coding
transmemb	protein-coding
ATPase, an	protein-coding
senataxin	protein-coding
unc-13 hor	protein-coding
olfactomec	protein-coding
solute carri	protein-coding
sterile alph	protein-coding
FIC domain	protein-coding
RAB3D, me	protein-coding

sodium cha	protein-coding
zinc finger	protein-coding
proline-rich	protein-coding
spindle anc	protein-coding
Kruppel-lik	protein-coding
RIKEN cDN.	protein-coding
splA/ryano	protein-coding
zinc finger	protein-coding
family with	protein-coding
RAS p21 pr	protein-coding
lysophosph	protein-coding
dual specif	protein-coding
LSM14 hon	protein-coding
sperm acro	protein-coding
MAK16 hor	protein-coding
anthrax to:	protein-coding
CD101 anti	protein-coding
short chain	protein-coding
nuclear fac	protein-coding
CTS telome	protein-coding
slingshot h	protein-coding
calcium/ca	protein-coding
kelch-like 3	protein-coding
Fyn proto-c	protein-coding
myeloid ce	protein-coding
Kruppel-lik	protein-coding
olfactomec	protein-coding
ATPase, H+	protein-coding
small nucle	protein-coding
latent trans	protein-coding
ral guanine	protein-coding
vascular ce	protein-coding
sterile alph	protein-coding
lymphoblas	protein-coding
sal-like 1 (D	protein-coding
chloride int	protein-coding
secretory c	protein-coding
cyclin-depe	protein-coding
adducin 3 (	protein-coding
fibronectin	protein-coding
SUZ RNA bi	protein-coding
family with	protein-coding
protein dis	protein-coding
kelch-like 2	protein-coding
homeodom	protein-coding
predicted g	protein-coding
solute carri	protein-coding

cyclin L1	protein-coding
CASP8 and	protein-coding
translocase	protein-coding
leucine rich	protein-coding
ariadne ub	protein-coding
major facili	protein-coding
Sjogren syr	protein-coding
guanidinoa	protein-coding
family with	protein-coding
nuclear cap	protein-coding
ataxin 7	protein-coding
RIKEN cDN.	protein-coding
pleckstrin h	protein-coding
proviral int	protein-coding
zinc finger	protein-coding
phosphodi	protein-coding
neuropilin	protein-coding
alkaline cer	protein-coding
von Hippel-	protein-coding
predicted g	protein-coding
predicted g	protein-coding
multiple EC	protein-coding
interleukin	protein-coding
3'-phospho	protein-coding
MAP/micro	protein-coding
serine/thre	protein-coding
CLK4-assoc	protein-coding
transmemb	protein-coding
zinc finger,	protein-coding
anaphase p	protein-coding
solute carri	protein-coding
CD97 antig	protein-coding
schlafen 9	protein-coding
T cell activa	protein-coding
cell division	protein-coding
IscU iron-su	protein-coding
spectrin re	protein-coding
angiogenic	protein-coding
serine (or c	protein-coding
mex3 homo	protein-coding
peptidylpro	protein-coding
OTU deubi	protein-coding
rhomboid c	protein-coding
RIKEN cDN.	protein-coding
minichrom	protein-coding
RB-associat	protein-coding
mitochond	protein-coding

mannan-bi	protein-coding
mannosida	protein-coding
TBC1 doma	protein-coding
eukaryotic	protein-coding
neural prec	protein-coding
myosin VII/	protein-coding
suppressio	protein-coding
dolichol kir	protein-coding
wingless-ty	protein-coding
prenyl (sola	protein-coding
chemokine	protein-coding
arginyl ami	protein-coding
plexin D1	protein-coding
ring finger	protein-coding
transmemb	protein-coding
JAZF zinc fi	protein-coding
popeye dor	protein-coding
3-oxoacid (	protein-coding
serine/argi	protein-coding
carnitine d	protein-coding
CCR4-NOT	protein-coding
olfactory re	protein-coding
cms small r	protein-coding
karyopheri	protein-coding
methyltran	protein-coding
poly (ADP-r	protein-coding
amplified ir	protein-coding
fibronectin	protein-coding
RIKEN cDN.	protein-coding
mitogen-ac	protein-coding
oligonucle(	protein-coding
RAB12, me	protein-coding
nucleic acic	protein-coding
coiled-coil	protein-coding
strawberry	protein-coding
matrix met	protein-coding
asparagine	protein-coding
SDE2 telon	protein-coding
ski sarcoma	protein-coding
zinc finger	protein-coding
CDC42 sma	protein-coding
antagonist	protein-coding
mitochond	protein-coding
chemokine	protein-coding
zinc finger	protein-coding
zinc finger	protein-coding
tripartite m	protein-coding

ubiquitin-c	protein-coding
golgi autoa	protein-coding
family with	protein-coding
transmemb	protein-coding
zinc finger	protein-coding
furry homc	protein-coding
ubinuclein	protein-coding
cDNA sequ	protein-coding
attractin	protein-coding
bone morp	protein-coding
pumilio RN	protein-coding
solute carri	protein-coding
numb gene	protein-coding
zinc finger,	protein-coding
macrophag	protein-coding
golgi coilec	protein-coding
endothelin	protein-coding
aldehyde d	protein-coding
STAM bind	protein-coding
solute carri	protein-coding
rhomboid 5	protein-coding
glutamyl ar	protein-coding
solute carri	protein-coding
cDNA sequ	protein-coding
Kruppel-lik	protein-coding
zinc finger	protein-coding
spleen focu	protein-coding
eukaryotic	protein-coding
hect (homc	protein-coding
phosphatic	protein-coding
origin reco	protein-coding
UDP glucur	protein-coding
cAMP resp	protein-coding
protein pho	protein-coding
uridine-cyt	protein-coding
UPF1 regul	protein-coding
ADP-ribosy	protein-coding
proline rich	protein-coding
zinc finger	protein-coding
RIKEN cDN.	protein-coding
upstream b	protein-coding
endonuclea	protein-coding
myotubula	protein-coding
cytochrom	protein-coding
bone morp	protein-coding
glycoprotei	protein-coding
integral me	protein-coding

v-abl Abels	protein-coding
bleomycin	protein-coding
RNA pseud	protein-coding
dual specif	protein-coding
kelch-like 1	protein-coding
carbonic ar	protein-coding
torsin A int	protein-coding
grainyhead	protein-coding
ATPase, Ca	protein-coding
leucine rich	protein-coding
antizyme ir	protein-coding
BTG3 assoc	protein-coding
BMP2 indu	protein-coding
tumor necr	protein-coding
zinc finger,	protein-coding
protein O-f	protein-coding
protein pho	protein-coding
fer (fms/fp	protein-coding
DEAD (Asp-	protein-coding
zinc finger	protein-coding
mitochond	protein-coding
metallo-be	protein-coding
cortactin b	protein-coding
protein-L-is	protein-coding
DNA methy	protein-coding
POU doma	protein-coding
potassium	protein-coding
jun proto-c	protein-coding
RIKEN cDN.	protein-coding
predicted g	protein-coding
trafficking	protein-coding
endothelin	protein-coding
sphingomy	protein-coding
ADP-ribosy	protein-coding
regulator o	protein-coding
PTK7 prote	protein-coding
lactation el	protein-coding
transforma	protein-coding
sema doma	protein-coding
neuralized	protein-coding
cyclin-depe	protein-coding
polymeras(	protein-coding
ring finger	protein-coding
POC1 centr	protein-coding
breast carc	protein-coding
mitochond	protein-coding
serine inco	protein-coding

lysophosph	protein-coding
intraflagell	protein-coding
zinc finger	protein-coding
regulator o	protein-coding
Alport sync	protein-coding
mitochond	protein-coding
calcium cha	protein-coding
trinucleotic	protein-coding
Kruppel-lik	protein-coding
transmemb	protein-coding
zinc finger	protein-coding
ADP-deper	protein-coding
acetyl-Coe	protein-coding
ATP-bindin	protein-coding
K(lysine) ac	protein-coding
Berardinell	protein-coding
cyclin-depe	protein-coding
RIKEN cDN.	protein-coding
SEC62 hom	protein-coding
WD repeat	protein-coding
activating t	protein-coding
calcium cha	protein-coding
vinculin	protein-coding
E2F transcr	protein-coding
REX4, RNA	protein-coding
adaptor-re	protein-coding
mitogen-ac	protein-coding
glycophori	protein-coding
kelch-like 2	protein-coding
CREB/ATF I	protein-coding
zinc finger,	protein-coding
ribosomal	protein-coding
ring finger	protein-coding
FERM dom	protein-coding
XRCC6 binc	protein-coding
lysine (K)-s	protein-coding
transmemb	protein-coding
lactate deh	protein-coding
heparan-al	protein-coding
intraflagell	protein-coding
cell divisior	protein-coding
polyhomec	protein-coding
ubiquitin-li	protein-coding
GLE1 RNA	protein-coding
Kruppel-lik	protein-coding
YLP motif c	protein-coding
paxillin	protein-coding

DNA prima	protein-coding
zinc finger	protein-coding
COMM dor	protein-coding
caspase act	protein-coding
serine/argi	protein-coding
tRNA isope	protein-coding
gamma-am	protein-coding
alkaline ph	protein-coding
ATPase, Ca	protein-coding
transketola	protein-coding
bromodom	protein-coding
solute carri	protein-coding
zinc finger	protein-coding
phosphoglu	protein-coding
potassium	protein-coding
phosphatic	protein-coding
enhancer c	protein-coding
transducin	protein-coding
interferon	protein-coding
thrombosp	protein-coding
phosphofru	protein-coding
CDK5 regul	protein-coding
ATP-bindin	protein-coding
ski sarcoma	protein-coding
major urina	protein-coding
tweety hor	protein-coding
tonsoku-lik	protein-coding
breakpoint	protein-coding
OTU domai	protein-coding
sorting nex	protein-coding
ectonucleo	protein-coding
GTP bindin	protein-coding
fos-like ant	protein-coding
exosome co	protein-coding
SR-related	protein-coding
microsoma	protein-coding
ATPase, H+	protein-coding
breast canc	protein-coding
CD97 antig	protein-coding
exocyst cor	protein-coding
myosin IE	protein-coding
complemen	protein-coding
glutaredox	protein-coding
B and T lym	protein-coding
sorting nex	protein-coding
5-aminoim	protein-coding
beta-1,4-N	protein-coding

MOB family	protein-coding
peroxisoma	protein-coding
transmemb	protein-coding
WD repeat	protein-coding
myomesin	protein-coding
proteasom	protein-coding
DnaJ (Hsp4	protein-coding
astrotactin	protein-coding
family with	protein-coding
filamin C, g	protein-coding
ATPase, cla	protein-coding
serine (or c	protein-coding
exocyst cor	protein-coding
RAN, meml	protein-coding
Jun dimeriz	protein-coding
egl-9 family	protein-coding
DnaJ (Hsp4	protein-coding
plectin	protein-coding
zeta-chain	protein-coding
SMAD fami	protein-coding
formin-like	protein-coding
lymphocyte	protein-coding
mago-nash	protein-coding
complemer	protein-coding
LSM2 homo	protein-coding
transforme	protein-coding
solute carri	protein-coding
RWD doma	protein-coding
fibronectin	protein-coding
mitochond	protein-coding
von Willebı	protein-coding
Ral GEF wit	protein-coding
golgin, RAE	protein-coding
elongator a	protein-coding
eukaryotic	protein-coding
folliculin in	protein-coding
phosphogly	protein-coding
START dom	protein-coding
DAZ intera	protein-coding
calcium cha	protein-coding
RIKEN cDN.	protein-coding
transient re	protein-coding
BCL2-like 1	protein-coding
hexokinase	protein-coding
ER membra	protein-coding
phosphatic	protein-coding
transformi	protein-coding

testis expre	protein-coding
hyaluronar	protein-coding
PRP3 pre-n	protein-coding
chitinase d	protein-coding
selenoprot	protein-coding
lysyl oxidas	protein-coding
thrombosp	protein-coding
TBC1 doma	protein-coding
fidgetin-lik	protein-coding
nucleopori	protein-coding
voltage-de	protein-coding
period circa	protein-coding
Rho GTPas	protein-coding
CDC14 cell	protein-coding
COMM dor	protein-coding
mitogen-ac	protein-coding
tetratricop	protein-coding
polo-like ki	protein-coding
SDE2 telom	protein-coding
cDNA sequ	protein-coding
immediate	protein-coding
zinc finger	protein-coding
human imr	protein-coding
Y box prote	protein-coding
Rab gerany	protein-coding
family with	protein-coding
heterogene	protein-coding
UDP-GlcNA	protein-coding
Werner he	protein-coding
nitrogen pe	protein-coding
prothymos	protein-coding
ankyrin rep	protein-coding
PAS domai	protein-coding
transmeml	protein-coding
Rho GTPas	protein-coding
nudix (nucl	protein-coding
diacylglyce	protein-coding
eukaryotic	protein-coding
ancient ubi	protein-coding
tetratricop	protein-coding
zinc finger	protein-coding
anoctamin	protein-coding
cullin 7	protein-coding
fucosyltran	protein-coding
HtrA serine	protein-coding
membrane	protein-coding
mitogen-ac	protein-coding

family with	protein-coding
demethyl-(	protein-coding
cytochrom	protein-coding
phosphatic	protein-coding
gasdermin	protein-coding
predicted g	protein-coding
calcium bir	protein-coding
interferon	protein-coding
poly (ADP-r	protein-coding
zinc finger	protein-coding
CD300 anti	protein-coding
muscleblin	protein-coding
peroxisom	protein-coding
RNA bindin	protein-coding
protease (p	protein-coding
ropporin 1-	protein-coding
regulator o	protein-coding
LYR motif c	protein-coding
matrix met	protein-coding
ZPR1 zinc f	protein-coding
YOD1 OTU	protein-coding
YY1 associa	protein-coding
hect domai	protein-coding
CAP, adeny	protein-coding
Mab-21 do	protein-coding
polyamine-	protein-coding
kit oncoger	protein-coding
solute carri	protein-coding
aspartyl-tR	protein-coding
diaphanous	protein-coding
interferon	protein-coding
forkhead b	protein-coding
sperm assc	protein-coding
cDNA sequ	protein-coding
gelsolin	protein-coding
copine II	protein-coding
phosphatic	protein-coding
liver glycog	protein-coding
small integ	protein-coding
helicase (D	protein-coding
GLI pathog	protein-coding
RIKEN cDN.	protein-coding
thioredoxir	protein-coding
eukaryotic	protein-coding
ER membra	protein-coding
RIKEN cDN.	protein-coding
tripartite m	protein-coding

chimerin 2	protein-coding
SNF relatec	protein-coding
sperm spec	protein-coding
tumor necr	protein-coding
endothelia	protein-coding
peroxisoma	protein-coding
IAP promot	protein-coding
arginyl ami	protein-coding
WD repeat	protein-coding
rhotekin	protein-coding
actin relate	protein-coding
colony stim	protein-coding
Ras associa	protein-coding
phosphohis	protein-coding
thioredoxir	protein-coding
5-hydroxyt	protein-coding
disabled 2,	protein-coding
phospholip	protein-coding
BTB (POZ) c	protein-coding
integrin alp	protein-coding
transcriptic	protein-coding
phosphatic	protein-coding
THAP doma	protein-coding
nrde-2 nec	protein-coding
protein pho	protein-coding
tumor necr	protein-coding
family with	protein-coding
eukaryotic	protein-coding
sperm anti	protein-coding
alpha glucc	protein-coding
nucleolar p	protein-coding
ST6 (alpha-	protein-coding
RAB28, me	protein-coding
ankyrin rep	protein-coding
Obg-like AT	protein-coding
Bmi1 polyc	protein-coding
orofacial cl	protein-coding
tubulin folc	protein-coding
RUN and FY	protein-coding
dual specif	protein-coding
cellular rep	protein-coding
formin 2	protein-coding
zinc finger,	protein-coding
ribosomal p	protein-coding
SEH1-like (S	protein-coding
cysteine-se	protein-coding
PHD finger	protein-coding

IQ motif co	protein-coding
phosphodi	protein-coding
protein arg	protein-coding
zinc finger	protein-coding
tec protein	protein-coding
phosphatic	protein-coding
dynactin 6	protein-coding
MRS2 mag	protein-coding
mediator c	protein-coding
malonyl-Co	protein-coding
S100 calciu	protein-coding
MyoD fami	protein-coding
Rho GDP di	protein-coding
SAM doma	protein-coding
RAS-relate	protein-coding
RIKEN cDN	protein-coding
TM2 doma	protein-coding
coiled-coil	protein-coding
N-myc dow	protein-coding
regulator o	protein-coding
Moloney sa	protein-coding
influenza v	protein-coding
cyclin-depe	protein-coding
StAR-relate	protein-coding
Rho GTPas	protein-coding
Fanconi an	protein-coding
euchromat	protein-coding
synaptotag	protein-coding
guanine nu	protein-coding
CD53 antig	protein-coding
lymphocyte	protein-coding
tripartite m	protein-coding
zinc finger	protein-coding
pyroglutam	protein-coding
replication	protein-coding
guanine nu	protein-coding
alpha-kinas	protein-coding
mitogen-ac	protein-coding
ubiquitin sp	protein-coding
choline deh	protein-coding
ribosomal p	protein-coding
nucleotide	protein-coding
mast cell ex	protein-coding
ubiquitin sp	protein-coding
bromodom	protein-coding
ATP/GTP b	protein-coding
ubiquitin-c	protein-coding

cytochrom	protein-coding
small nucle	protein-coding
enhancer c	protein-coding
transmemb	protein-coding
B cell trans	protein-coding
armadillo r	protein-coding
F11 recept	protein-coding
calmodulin	protein-coding
kelch-like 1	protein-coding
jagunal hor	protein-coding
ribosomal	protein-coding
consortin,	protein-coding
RIKEN cDN	protein-coding
regulator o	protein-coding
spindle anc	protein-coding
isoprenoid	protein-coding
methionine	protein-coding
predicted g	protein-coding
THO compl	protein-coding
cyclin F	protein-coding
RIKEN cDN	protein-coding
family with	protein-coding
TAF11 RNA	protein-coding
NEDD4 bin	protein-coding
leucine rich	protein-coding
transmemb	protein-coding
ubiquitin-li	protein-coding
sorting nex	protein-coding
DCP1 deca	protein-coding
formin binc	protein-coding
heat shock	protein-coding
Sec23 inter	protein-coding
serine/argi	protein-coding
N(alpha)-ac	protein-coding
ectoderma	protein-coding
ATPase, Na	protein-coding
PQ loop re	protein-coding
1-acylglyce	protein-coding
DEAD (Asp-	protein-coding
phosphatas	protein-coding
runt relate	protein-coding
pseudouric	protein-coding
RIKEN cDN	protein-coding
septin 5	protein-coding
CDP-diacyl	protein-coding
zinc finger	protein-coding
TRAF family	protein-coding

protein kin	protein-coding
heat shock	protein-coding
kit oncoger	protein-coding
RIKEN cDN.	protein-coding
MOB kinas	protein-coding
transformi	protein-coding
lipoxygena	protein-coding
PR domain	protein-coding
cell divisior	protein-coding
ras homolc	protein-coding
small ArfG	protein-coding
immediate	protein-coding
Friend leuk	protein-coding
transporte	protein-coding
SPEN homc	protein-coding
neutral sph	protein-coding
lipase, end	protein-coding
RIKEN cDN.	protein-coding
small integ	protein-coding
DNA segme	protein-coding
HECT doma	protein-coding
proline-ricl	protein-coding
AF4/FMR2	protein-coding
small integ	protein-coding
contactin a	protein-coding
methionyl	protein-coding
tigger trans	protein-coding
cyclin J	protein-coding
E2F transcr	protein-coding
zinc finger	protein-coding
solute carri	protein-coding
salt inducik	protein-coding
transmemk	protein-coding
RAN bindin	protein-coding
dual specif	protein-coding
dual specif	protein-coding
hedgehog a	protein-coding
NLR family,	protein-coding
meiosis exp	protein-coding
forkhead b	protein-coding
EH-domain	protein-coding
nuclear mit	protein-coding
solute carri	protein-coding
UDP-N-ace	protein-coding
activating t	protein-coding
glutathione	protein-coding
methyl-CpC	protein-coding

glutamic py	protein-coding
Ras associa	protein-coding
aspartic pe	protein-coding
K(lysine) ac	protein-coding
STE20-like	protein-coding
serine palm	protein-coding
protein kin	protein-coding
CDKN2A int	protein-coding
ankyrin rep	protein-coding
zinc finger	protein-coding
aryl-hydroc	protein-coding
mitochond	protein-coding
keratinocyt	protein-coding
eukaryotic	protein-coding
progestero	protein-coding
mitochond	protein-coding
lymphocyte	protein-coding
ATPase, Na	protein-coding
O-sialoglyc	protein-coding
HIG1 doma	protein-coding
sphingomy	protein-coding
RIKEN cDN.	protein-coding
inositol 1,4	protein-coding
G protein-c	protein-coding
doublecort	protein-coding
ATP/GTP b	protein-coding
phosphatic	protein-coding
interleukin	protein-coding
kelch-like 2	protein-coding
carbohydra	protein-coding
protease, s	protein-coding
sine oculis-	protein-coding
son of seve	protein-coding
glycoprotei	protein-coding
Casitas B-li	protein-coding
guanine de	protein-coding
tetratricop	protein-coding
autophagy	protein-coding
polycomb g	protein-coding
histone clu	protein-coding
CCAAT/enh	protein-coding
tripartite m	protein-coding
testis deriv	protein-coding
dual specif	protein-coding
tumor necr	protein-coding
stromal an	protein-coding
dihydrouric	protein-coding

coiled-coil	protein-coding
AE binding	protein-coding
lactase	protein-coding
ARP3 actin	protein-coding
tetratricop	protein-coding
otolin 1 ho	protein-coding
cullin assoc	protein-coding
RasGEF dor	protein-coding
galactosida	protein-coding
diphthamir	protein-coding
autophagy	protein-coding
protein tyr	protein-coding
ring finger	protein-coding
H1 histone	protein-coding
mannose r	protein-coding
suppressor	protein-coding
son of seve	protein-coding
ankyrin rep	protein-coding
echinoderr	protein-coding
glypican 1	protein-coding
cut-like hor	protein-coding
actin-like 7	protein-coding
malectin	protein-coding
ring-box 1	protein-coding
RIKEN cDN	protein-coding
Abelson he	protein-coding
RAS-relate	protein-coding
regulator o	protein-coding
calcium cha	protein-coding
abhydrolas	protein-coding
leucine rich	protein-coding
neurofibro	protein-coding
retinoblast	protein-coding
mitochond	protein-coding
diphthamir	protein-coding
BMP2 indu	protein-coding
UDP-N-ace	protein-coding
excision re	protein-coding
mex3 hom	protein-coding
notch 2	protein-coding
ST8 alpha-I	protein-coding
kinesin fam	protein-coding
nuclear ant	protein-coding
helt bHLH t	protein-coding
zinc finger	protein-coding
muscle anc	protein-coding
serine/thre	protein-coding

nicastrin	protein-coding
G patch do	protein-coding
B9 protein	protein-coding
vomeronas	protein-coding
guanosine	protein-coding
SNW doma	protein-coding
olfactory re	protein-coding
lectin, gala	protein-coding
PDGFA ass	protein-coding
thioredoxir	protein-coding
SET domair	protein-coding
RIKEN cDN.	protein-coding
baculoviral	protein-coding
TCDD-indu	protein-coding
calcium rel	protein-coding
rho/rac gu	protein-coding
FYVE, RhoG	protein-coding
coiled-coil	protein-coding
vaccinia rel	protein-coding
poly(A) bin	protein-coding
DnaJ (Hsp4	protein-coding
limb regior	protein-coding
C1q and tu	protein-coding
torsin fami	protein-coding
selectin, lyı	protein-coding
ER membra	protein-coding
Cbp/p300-i	protein-coding
expressed :	protein-coding
neurturin	protein-coding
ATP/GTP b	protein-coding
RWD doma	protein-coding
mediator c	protein-coding
KDM1 lysin	protein-coding
FMS-like ty	protein-coding
protein ph	protein-coding
ATP-bindin	protein-coding
eukaryotic	protein-coding
protein ph	protein-coding
Rho guanir	protein-coding
guanosine	protein-coding
active BCR-	protein-coding
ATPase, H+	protein-coding
transforme	protein-coding
phosphom	protein-coding
predicted g	protein-coding
sorting nex	protein-coding
lymphocyte	protein-coding

nudix (nucl	protein-coding
excision re|	protein-coding
tribbles ho	protein-coding
histone dea	protein-coding
Aly/REF ex|	protein-coding
zinc finger	protein-coding
brain-speci	protein-coding
macrophag	protein-coding
asparagine	protein-coding
glycosyltra	protein-coding
poly(A) bin	protein-coding
versican	protein-coding
hematopoi	protein-coding
potassium	protein-coding
myosin VA	protein-coding
6-phospho	protein-coding
protein kin	protein-coding
solute carri	protein-coding
spindle ap|	protein-coding
THAP dom	protein-coding
ring finger	protein-coding
ataxin 1	protein-coding
retinol deh	protein-coding
cyclin-depe	protein-coding
adaptor pr	protein-coding
zinc finger	protein-coding
serine/thre	protein-coding
prokinetici	protein-coding
cytidine an	protein-coding
multimerin	protein-coding
palladin, c	protein-coding
nuclear fac	protein-coding
transmemb	protein-coding
F-box and \	protein-coding
versican	protein-coding
adenosine	protein-coding
2-phospho	protein-coding
coiled-coil	protein-coding
heat shock	protein-coding
DNA segme	protein-coding
cell divisior	protein-coding
SNAP-asso	protein-coding
protein kin	protein-coding
PTPRF inte	protein-coding
a disintegri	protein-coding
SWI/SNF re	protein-coding
solute carri	protein-coding

NEDD4 bin	protein-coding
cyclin-depe	protein-coding
ER membra	protein-coding
centromer	protein-coding
Rho GTPas	protein-coding
family with	protein-coding
GC-rich sec	protein-coding
predicted g	protein-coding
transmemb	protein-coding
MGAT4 far	protein-coding
ribosomal	protein-coding
coenzyme	protein-coding
a disintegri	protein-coding
T cell acute	protein-coding
succinate-C	protein-coding
TSC22 dom	protein-coding
WD repeat	protein-coding
BRF2, subu	protein-coding
carnitine d	protein-coding
serrate RN	protein-coding
RIKEN cDN	protein-coding
GTP cycloh	protein-coding
seven in ab	protein-coding
U2 small nu	protein-coding
E74-like fac	protein-coding
Friend leuk	protein-coding
ribosomal	protein-coding
HGH1 hom	protein-coding
Josephin d	protein-coding
lysine (K)-s	protein-coding
CD93 antig	protein-coding
JAZF zinc fi	protein-coding
heat shock	protein-coding
family with	protein-coding
bromodom	protein-coding
RIKEN cDN	protein-coding
mannoside	protein-coding
nidogen 2	protein-coding
ets variant	protein-coding
leucine rich	protein-coding
histocomp	protein-coding
interferon	protein-coding
CWC27 spli	protein-coding
C-type lect	protein-coding
hypoxia inc	protein-coding
myoferlin	protein-coding
mitochond	protein-coding

proteasom	protein-coding
SET binding	protein-coding
serine pept	protein-coding
RAB14, me	protein-coding
RIKEN cDN.	protein-coding
myeloblast	protein-coding
C2 calcium	protein-coding
polycystic k	protein-coding
ribosomal I	protein-coding
lamin B1	protein-coding
zinc finger	protein-coding
D-2-hydrox	protein-coding
anaphase p	protein-coding
THAP doma	protein-coding
transducin	protein-coding
Scm-like wi	protein-coding
a disintegri	protein-coding
solute carri	protein-coding
progestin a	protein-coding
3'-phospho	protein-coding
N(alpha)-ac	protein-coding
activated le	protein-coding
RIKEN cDN.	protein-coding
CD200 rece	protein-coding
inhibitor of	protein-coding
spastic par	protein-coding
COBW dom	protein-coding
N-acetylglu	protein-coding
syntaxin bi	protein-coding
spleen focu	protein-coding
retinoic aci	protein-coding
CDC42 sma	protein-coding
phosphopr	protein-coding
cyclic nucle	protein-coding
TYRO prote	protein-coding
JAZF zinc fi	protein-coding
actin relate	protein-coding
B cell leuke	protein-coding
creatine kii	protein-coding
ephrin A1	protein-coding
uridine-cyt	protein-coding
solute carri	protein-coding
phosphatic	protein-coding
scaffolding	protein-coding
RAB GTPas	protein-coding
F-box and \	protein-coding
peroxisom	protein-coding

thioredoxir	protein-coding
G0/G1 swit	protein-coding
phosphodi(	protein-coding
quiescin Q(	protein-coding
inhibitor of	protein-coding
non-SMC e	protein-coding
fibronectin	protein-coding
transforme	protein-coding
mitochond	protein-coding
mannosida	protein-coding
cyclin Y	protein-coding
cDNA sequ	protein-coding
zinc finger	protein-coding
family with	protein-coding
casein kina	protein-coding
heparan su	protein-coding
centrosom	protein-coding
histidine tr	protein-coding
FAT tumor	protein-coding
ubiquitin-c	protein-coding
Sad1 and U	protein-coding
Bcl2-like 1(	protein-coding
mediator o	protein-coding
oxysterol b	protein-coding
proteasom	protein-coding
syntrophin	protein-coding
congenital	protein-coding
ring finger	protein-coding
aquaporin	protein-coding
RIKEN cDN.	protein-coding
bora, auror	protein-coding
ataxin 10	protein-coding
GC-rich prc	protein-coding
serological	protein-coding
forkhead b	protein-coding
WD repeat	protein-coding
solute carri	protein-coding
SH3 domai	protein-coding
male-speci	protein-coding
versican	protein-coding
chemokine	protein-coding
pyruvate d	protein-coding
spermatog	protein-coding
discs, large	protein-coding
platelet-ac	protein-coding
nuclear fac	protein-coding
solute carri	protein-coding

mitogen-ac	protein-coding
chromobo›	protein-coding
jade family	protein-coding
integrin alp	protein-coding
MPN doma	protein-coding
WD repeat	protein-coding
zinc finger	protein-coding
timeless in	protein-coding
interleukin	protein-coding
trinucleotic	protein-coding
mitochond	protein-coding
melanoreg	protein-coding
expressed :	protein-coding
zinc finger	protein-coding
proline rich	protein-coding
arrestin do	protein-coding
zinc finger	protein-coding
heat shock	protein-coding
interleukin	protein-coding
Ras-relatec	protein-coding
chromodor	protein-coding
guanine nu	protein-coding
predicted g	protein-coding
Rho GTPas	protein-coding
guanine nu	protein-coding
RASD famil	protein-coding
formyl pep	protein-coding
tribbles ho	protein-coding
CDC28 pro	protein-coding
syntaxin 18	protein-coding
secretory l	protein-coding
brain abun	protein-coding
tubulin pol	protein-coding
RIKEN cDN	protein-coding
acyl-CoA th	protein-coding
lamin B rec	protein-coding
RIKEN cDN	protein-coding
disrupted i	protein-coding
family with	protein-coding
vacuolar pı	protein-coding
CDC42 effe	protein-coding
BCL2-like 1	protein-coding
ATPase fan	protein-coding
RIMS bindi	protein-coding
RIKEN cDN	protein-coding
inhibin bet	protein-coding
heterogene	protein-coding

HOP home	protein-coding
gamma-sec	protein-coding
splicing fac	protein-coding
ATP-bindin	protein-coding
xyloside xy	protein-coding
histone clu	protein-coding
glutathione	protein-coding
protein kin	protein-coding
RIKEN cDN.	protein-coding
eukaryotic	protein-coding
lysocardiol	protein-coding
DENN/MAI	protein-coding
early grow	protein-coding
heat shock	protein-coding
brain expre	protein-coding
endonucle	protein-coding
TEN1 telon	protein-coding
muscleblin	protein-coding
ARP3 actin	protein-coding
prothymos	protein-coding
utrophin	protein-coding
Fas-associa	protein-coding
RIKEN cDN.	protein-coding
NADH deh	protein-coding
ornithine d	protein-coding
SUMO/sen	protein-coding
inositol (m	protein-coding
AFG3-like	protein-coding
HGH1 hom	protein-coding
ArfGAP wit	protein-coding
diacylglyce	protein-coding
penta-EF h	protein-coding
sterol-C5-d	protein-coding
RIKEN cDN.	protein-coding
ribosomal	protein-coding
GAR1 ribor	protein-coding
ubiquitin c	protein-coding
ninjurin 2	protein-coding
histone clu	protein-coding
solute carri	protein-coding
ADP-ribosy	protein-coding
small nucle	protein-coding
acid-sensin	protein-coding
met proto-	protein-coding
chromodor	protein-coding
tet methyl	protein-coding
C-type lect	protein-coding

leucine rich	protein-coding
family with	protein-coding
phosphoty	protein-coding
glyoxalase	protein-coding
ring finger	protein-coding
E2F transcr	protein-coding
protein pho	protein-coding
integrin be	protein-coding
superoxide	protein-coding
E2F transcr	protein-coding
UDP-GlcNA	protein-coding
