# Supplementary material for: MLL1 and MLL1 fusion proteins have distinct functions in regulating leukemic transcription program
Source: Cell Discov. 2016 May 17;2:16008–. doi: 10.1038/celldisc.2016.8 (PMC4869169; doi:10.1038/celldisc.2016.8)
Supplement: Supplementary Table S2 [file celldisc20168-s8.pdf]

Supplemental Table 2 Complete lists for the joint targets of MLL1 and MLL-AF9.

MLL1 and MLL-AF9 cotargeting gene list

gene ID
Hebp2
Arrdc4
Scaf11
Lrig2
Herc3
Ttll11
Ermp1
Dusp6
Mcm3
Ggh
Mllt10
Trim45
Snx24
Peli3
Oip5
Stk24
Kcnj2
Eltd1
Utrn
Immp2l
Prkag2
Dnah17
Cerk
Cdk6
Ctdspl
Camk4
Tnpo1
D430042O09Rik
Meis1
Fam212a
Asap1
Sorl1
Zbtb16
Ptprk
Kit
Trps1
Grem2
Cinp
Tmcc1
Vgll4
Nln
C1qtnf7
Stx8
Ubr3
Xxylt1

Pdha2
Sox21
Khdrbs3
Magi1
Gbe1
Ctbp2
Baz1a
Lyst
Gm7102
Cylc2
Dnah11
Kcnip4
Setbp1
1190002N15Rik
Msl3l2
Tmem196
Ndufb3
Zbtb38
Tns3
Trim67
Pim3
Bcat1
Gm3376
Gclm
Slc45a1
Mtus2
Mboat1
Zfp326
Grik3
Sdk1
Sh2b2
Stxbp5
Dpy19l1
Snx18
Runx2
Dhrs13
Tuba8
Begain
Zfp36l2
Gm14461
Pcdh18
Mab21l3
Dlgap4
Pmp22
Cacna1b
Kctd16
Lemd3

Edem1
Grin2b
Fkbp15
Six1
Vegfa
4933430I17Rik
Osbpl1a
Tmem106a
Sort1
Gpc6
Sgk3
Papss2
Cers6
Csrnp3
Lrpprc
Elmo1
Mcm8
Zfp961
11-Mar
Slco6d1
Dpyd
Ceacam18
Anp32b
Tbc1d14
Atrnl1
Mapre3
Jak2
Ghitm
Spry2
Bmp2k
Elavl2
Camta1
Haao
Cux1
Cacna2d3
Opcml
Erg
Cdca2
Syne1
Prmt6
Ece1
Sim1
Tmem236
Sel1l
Chmp4b
Chd2
Cacnb4

Galnt7
Pak3
Slitrk1
Josd1
Olig3
Ly6c2
Dtx4
Arhgap21
Hlf
Trib2
Copb1
Robo2
Fzd1
Epm2a
9430020K01Rik
Tcf12
Pik3r1
Luzp1
Galc
Msr1
Apbb1ip
Hnrnpf
Tnfaip8
Gcnt1
St3gal1
Fryl
Kat7
Hira
Cd226
Pphln1
Lrrtm2
Zfp609
Nr4a2
Matn1
Ccdc114
Efna5
Lamtor3
1700003F12Rik
1810046K07Rik
Col25a1
Polr1c
Crim1
Adh5
Rnf144b
Rnf103
AI467606
Suz12

Stxbp3a
Il12a
Cyb561
Ubr1
Cox7a2
Plxna2
Jmy
Smarca2
Creb1
Clip1
Cgn
Nol4
Arrdc3
Tinag
Gsr
Kif21a
1700112E06Rik
Gas7
Spag17
Pde7b
Mat2b
Ftmt
Pcdh9
Susd1
Enpep
Ttc19
Grin2a
Tnfsf18
Celsr3
Epb4.1l5
Pcdh17
Trpm3
Klf6
Mapre2
Shh
Six4
Brd1
Chrna7
Galnt11
Rap2b
Pja2
Tmc3
Afap1
Fbxo21
Col9a1
Shox2
Tbxas1

Umps
Ccne2
Zfp276
Spock1
Ndst3
Sema3d
Eva1a
Rcc1
Adar
Tacc2
Tmem74
Dhrs3
Lyplal1
Coa7
Cpeb1
Ptpn12
1-Mar
Dennd2c
Nsun3
Snrpa1
Foxp4
B630005N14Rik
Slc30a8
Nfatc3
Arap1
Chd7
Rab24
Lysmd4
Dck
Mafb
Fam193a
Picalm
Mef2c
Igf1r
Gm5549
Bmper
Slmap
Socs6
Fcho2
Ston2
Stag1
Zfp40
Trub1
Fam69a
Chn2
Tiam2
Sec23a

Spns3
Cdh9
Morn2
Pard6g
Tax1bp1
Brinp1
Uso1
Tulp4
S1pr3
AI429214
6-Sep
Vps13a
Api5
B4galt6
Inhba
Clip2
Tlx3
Pbx1
Acaca
Neil3
Pawr
Nkain3
Camkmt
Cdc42
Adcyap1
Mbd2
Tex36
Reck
Jmjd1c
Dip2b
Cpsf6
Nsmce1
D2Wsu81e
Ahi1
Depdc5
Gcnt2
Vstm2a
Lmx1a
Arhgap20
Hs3st3b1
Rarb
Rad51b
Pde8b
Psat1
Spic
Grm3
Anxa3

Rsl24d1
Dfna5
Tspan11
N6amt1
Gpr125
Cep350
Cyp27a1
Zfc3h1
Il12b
Pth2r
Osbp
Foxp1
Pwwp2b
Fam107b
Apobec3
Arfgef1
Brinp3
Rnf152
Prkar2b
Rapgef5
Pex2
Hdac9
Cntnap5a
Lrtm1
Igsf10
Nr2f2
Hoxa6
Med13l
Haus2
Lnpep
Slc35b3
Pdhb
Ttc32
Papd5
Pde3b
Dclk2
Dazl
Itpr1
Dlx6
Sgcd
Atxn10
Adcy9
Hoxa10
Nat10
Slc12a7
Slc16a3
Cwc27

Tmem260
Ncs1
Tspan5
Tenm4
Rbbp8
Sstr4
Reps1
Calcrl
Anks1b
Swap70
Itgav
Murc
Flnb
Impg1
Col1a2
Mvb12b
3830403N18Rik
Bach1
Ccdc171
Irak1bp1
Fstl5
Gm15881
Spata13
Iyd
Slc25a13
Etaa1
Sfrp2
F13a1
4932414N04Rik
Cdk5rap2
Skap2
Dio2
Slc10a6
Fam46a
Samd4
Fam133b
Pax3
Ano4
Fancc
Ncor2
Rab13
2610002J02Rik
Nfat5
Ostf1
Slit3
Akap7
Gtf3c3

Cntnap2
Itih5
Ptbp2
Dennd1a
Adprhl1
Muc4
Smco4
Ndfip1
Lrrc3b
Tbc1d1
Dync2li1
Alcam
Gnb2l1
St8sia4
Ccrn4l
Prox1
Galnt18
Arhgap32
Slc2a2
Adam6b
Ski
Gnaz
Tceb3
Ppp1r9b
Ptges
Stt3b
Adpgk
Gpr75
B3galt2
Ywhag
Rgs18
Kcnn3
Zfyve21
Syt7
Synj2
Fam122a
Vwa8
C2cd4a
Raly
Abca1
Slc39a11
Plec
Myo3b
Cep95
1700016H13Rik
Mecom
Dok5

Zbtb1
Mbp
1810013L24Rik
Impdh1
Tmem126b
Gadl1
Spry4
Fut8
Atoh1
Actr3
Cngb3
Wdfy3
Cmpk2
Zfp800
Rsbn1
Sntb1
Nsun4
Zfp619
Myb
Antxr1
AW209491
Phtf1
Lypd1
Eps15
Grhl2
Rev1
Maml3
Unc5cl
Ddhd2
Htr1a
Fndc3a
BC030500
Fam222b
Rnf13
Wnt2
Ankrd28
Mapk6
Adam39
Nlgn1
Cwc15
Olfm4
Etv6
Igfbp7
Zadh2
Tsc22d1
Olfr161
Fbxl14

Nufip1
Slc23a2
Galnt13
Mef2a
Ly86
Plcb4
Grik4
Dgkg
Rab11fip2
Celf4
Ptprr
Spsb4
Cr2
Perp
Rapgef6
Klhl32
Serbp1
Ivns1abp
Rasgrp1
Heatr5a
Trib1
St18
Ctnnbip1
Fam53a
Cblb
Cse1l
Cdh17
Kctd20
Cntnap5b
Epha6
Dtx3l
Chl1
Trhde
Gm15800
Tab2
Parvg
Wdr11
Uimc1
Khdrbs2
Ugcg
Myo10
Dach1
Pspc1
Gdf7
Atp6ap1l
Vav3
Agbl1

Gtpbp4
Arhgap18
3110043O21Rik
Cdh20
Neto1
Lsm1
Sgms1
Zfp341
Car12
Atp11b
Alx1
Ubr2
Clasp2
Arhgap26
Prkcb
Pcmtd1
Nudt12
Btd
Slc8a1
Lpar1
Rbx1
Usp6nl
Phb
Kcnc2
Nbas
Ptk7
Ncam1
Hgf
Tal2
1700017N19Rik
Nabp1
Snx19
Abca13
Prkca
Efr3a
Uhrf2
Myh14
Pigp
Dnajc25
Cyp26a1
Gm13154
Dkk2
Tcf7l2
Chst9
Idh1
Ino80c
Mtfr2

Ncam2
Rreb1
Mis18a
Rasgef1b
Ch25h
Ptger2
Fam65b
Map3k14
Nrxn1
Slitrk5
Scara5
Chrm3
Ermn
Slc1a4
Ncor1
Cxcl12
Traf4
Maml2
Fry
Glrx
Ank3
Dyrk1a
Mgat4a
Gng12
Adamts5
Megf9
Nampt
Kif13b
Rbm12
Pbrm1
Glra1
Helz
Rassf9
Kcnk9
Slc25a37
Olfr845
Tbx18
4930562C15Rik
Sorbs1
Elf1
Pggt1b
Phactr2
Inpp4b
Ppargc1a
Bcl2l11
Frat2
Zfp804a

1200014J11Rik
Phtf2
Fam196b
Erc2
BC030307
Tmeff2
Wars
Anapc1
Dock9
Lims1
Kazn
Fscb
Wwox
A1bg
Ankrd54
Dnajc6
Pid1
Slc16a1
Med16
Rab21
Vcl
Runx1t1
Hist1h2bl
Exoc6
Ncald
2700049A03Rik
N4bp1
Thrb
Ubxn2b
Cers3
Ckmt2
Mut
Lmod3
Cd93
Bhlha15
Btbd3
Insm2
Fam60a
Nova1
Nav3
Mbnl1
Atad2b
Dcc
Edn1
Slc17a6
Maf
Mrpl57

Mtmr12
7-Mar
Vps53
Mpzl1
Mbd5
Gm3985
Fgd5
Lrrc8d
Pitx2
Rictor
Flrt1
Ifitm1
Agpat9
Ust
Tet2
Msl2
Il6st
Kcnh5
Adamts17
Zmiz2
Slc30a5
Dpp6
Ppapdc1a
Ube2e3
Mnt
Ptprg
Ppp1r9a
Neurod1
Cemip
Zeb2
Bptf
N4bp2l1
Mppe1
Mgat2
Gpam
Tmem132b
Gpbp1
Nrg1
Klf12
Tal1
Lmo2
Kansl1
Wasl
Ctnna2
Pisd
Cypt4
Fam105a

Cytip
Parp8
Rasd1
Esco1
Scamp1
Mdga2
Reep3
Asl
Cyp1a2
Pax9
Slc3a2
Fos
Smim14
Nrxn3
Tyrp1
Prickle2
Arhgap24
Commd8
St7
Fgf9
Purb
Tdrd3
Vps13c
Klf5
Ldlrad4
Tbcel
Atxn1
Rorb
Sorcs1
Socs5
Pla2g4a
Npsr1
Mylip
Foxa2
Flrt2
Sash1
Atp8a2
Nfia
Fbxw7
Nav2
Ikzf1
Acvr2a
Poc1a
Fam53b
Dsp
Cdkn1c
3830406C13Rik

Rsf1
Lrp8
Fam84a
Ofcc1
Igfbp3
Ninj2
Rab8b
Gm266
Ppp3ca
8030423J24Rik
Hsfy2
Btg1
Phactr1
Clnk
Nedd1
Lingo2
Galnt3
Abhd6
Patz1
Smap1
Spock3
Coq2
Ppip5k1
Ldhal6b
Xrcc6bp1
Phc3
Rassf3
Hs3st5
BC061194
Dct
U2af2
Tenm2
Themis2
Cyp26b1
Fam179b
Zcchc2
Vmn2r94
Bola3
Tnfsf8
Rgs22
Ugt8a
Kirrel3
Foxl1
Dlg2
Kcnj6
2010300C02Rik
Lrrc56

Unc5d
Mpp6
Zfy2
Kcnma1
Tcp11
Nr5a2
Ap4e1
Cenpc1
Rbfox3
Grb14
Phlpp2
Mtdh
C1galt1
Rad18
Slc37a1
Actn1
Tspan2
Hace1
Ltbp1
Grid2
Fat3
Med12l
Rims1
Tmem165
Tgm6
Sgce
Rab28
Them7
Tfap2a
Antxr2
Atg16l2
Chchd5
Ptbp3
Abcc4
Papss1
Esr1
Pdcd4
Rbms3
Egr3
Fli1
Nyap2
Top2b
Ska2
Pcdh7
Naa35
Vps4a
Mrps23

Chd9
Nbea
Mocs2
Tbc1d19
Nanos1
Nr3c1
Slc38a2
Kpna2
Hoxa7
Mark1
Bambi
Arid3b
Ttc36
Wdr27
Tmem18
Dhx30
Fbrsl1
Phykpl
Efnb2
Lphn3
Gfra2
Trim59
Eif4ebp2
Rnmt
Aebp2
Crebzf
Pkn2
Sucla2
Irf2
Crygf
Atp2b2
Hao1
Zfp217
Hnf4g
Fkbp5
Hnrnpll
Klf4
Nedd9
Fam19a4
Csmd1
Rpf2
E030018B13Rik
Ntf3
Amigo2
Med13
Ppp1r3b
Vps8

Cst3
Sema6d
Jakmip3
Usp47
Lysmd2
Larp4b
Dsel
Smim15
Ppp6c
Plekhf2
Ctnna3
Lap3
Atp10a
Pou1f1
Chordc1
Ttc29
Tas2r119
Mon2
Ero1l
Hspb1
Ets1
Lrrtm1
Ntrk3
1810011O10Rik
Rab39b
Agtpbp1
Amph
Hsd11b1
Lrmp
Mcur1
Dennd4a
Atg4c
Snx13
Erbb4
Sphkap
Dtnbp1
Syt6
Shisa2
Brd4
Sntg1
Gpr116
Tenm3
Ctnnd2
Car10
Cacnb2
Sorcs3
Lhcgr

Lonrf1
Vav1
Hmox1
Flrt3
Pitpna
Fgf12
Utp18
Mapk13
Adipor2
Atoh8
Trmt11
Adamtsl1
Hp1bp3
Khdrbs1
Gpr84
Mpp7
Plk2
Nkx6-1
Ccdc74a
Trim32
Irx1
Mapk1ip1
Rap1gap2
Cd47
Mgat5
Tbc1d2
Mctp2
4921506M07Rik
Plet1
Dppa4
Kdm4c
Kdm8
Cat
Arid2
Rbm47
Triml1
Rin3
Fam20b
Bcdin3d
Zfp710
Oprm1
Fam46c
Irf2bp2
Il15
Elovl6
Mad2l1
Zfp365

Kcnj15
Jam3
Tsc22d2
Zfp644
Adk
Rora
Angptl4
Etv1
Eya1
Zfp462
BC030336
AI314180
Epb4.1
Mgst2
Rdm1
Samsn1
Tcf4
Mkl2
Insr
Nt5c2
Tbl1xr1
Snx9
Abcg2
Hrh4
Sos2
Epha7
Tmem65
Mier3
Plac8
Rnf145
Lpp
Ubl3
Ppap2b
Ssbp2
Rtn4
Tiam1
Sipa1l1
Rnf220
Capza2
Ube2k
Hectd1
Marcks
Kctd1
Dact1
Aqp4
Wdr19
Axin2

Lhfpl2
Cetn3
Pard3
Abcd2
Diras2
Smad6
Robo1
Vmn2r102
Daam1
Macc1
Cobl
Fat4
Cd209f
Lef1
D8Ertd82e
Nos1ap
Ankrd7
Tox
Atxn7l3b
Extl3
Tmpo
Pten
Kif16b
Pparg
Itga4
1700056E22Rik
Slc9a4
Sesn1
Tle4
Gcsh
Lrp5
Adam10
Atxn7l1
Gpr176
Grxcr1
Pmaip1
Osbpl10
Gli3
Rwdd3
Met
Mgarp
Abra
Snx29
Kcnj12
Spin1
Rapgef2
Zfp407

Rps15a
Emp1
Pygo1
Aff1
Frs2
Ube3a
Rab11fip4
App
Ube2u
Dock8
1700015G11Rik
Minpp1
Plxnd1
Isl1
Basp1
Ap1s3
Akap13
Lsamp
Msantd2
Pdzrn3
Adam7
Pign
Zfp53
Rb1cc1
Crlf2
Arhgap22
Hnrnpd
4930447A16Rik
Arid1b
Eif4ebp1
Lypd6
Sema3b
Sox5
Tcerg1l
Sox6
Tsc2
Eno1b
Csnk1g1
Irs2
Stim2
Irs1
Olfm1
Ctdp1
Bard1
Dennd5a
Elf2
Mettl24

Eif2ak3
Atg7
Nt5c1b
Fam181b
Csrnp1
4931408C20Rik
Elovl4
Speer2
Epc2
Thada
Rrn3
Ube2h
Scfd2
Ddx18
Tusc1
Slc7a1
A530099J19Rik
Atg4d
Ptpn2
Adrb2
Samd12
4930523C07Rik
Cdk5r1
Etnk1
Snx10
Slc10a2
Ythdf3
Myh10
Tmem209
Gfi1
Cdk17
Hs6st3
4933402J07Rik
Stxbp6
1700025G04Rik
Lrrc4c
Tgif1
Abcg3
Osbpl8
Gm5803
Slc16a7
Fzd8
Csmd3
Lhx9
Baz2b
Vps13b
Agmo

Fggy
Vmn1r238
Abcd3
Ldb2
Socs1
Tmem261
Utp6
Srpk1
Fscn1
Sqrdl
Pbx3
Dock5
Lypd6b
Csnka2ip
Mtss1l
Aard
Irf2bpl
Wac
Hoxa9
Gcm2
Adam12
Bcl2l14
Hiat1
Fmod
Cntnap4
Nfatc1
Npy2r
Paqr4
Abca4
Nsmce2
Astn2
Mbnl2
Cdkal1
Stc2
Gstz1
Bcl11a
Snx16
Siah1a
Tle3
Plekhh2
Zfp191
Hpgd
Pgd
Gcc1
Igip
Dync1i1
Lmo4

Clvs1
Setd5
Elmsan1
Gga2
Ubash3b
Hnrnpab
Gm12695
Gramd3
Enpp6
Prss52
Fam172a
Lmbrd1
Ins1
Pgk1
Oxr1
Gda
Tlr4
Lace1
Specc1l
Zak
Stx12
Cd44
Ttll7
Rnf111
Kdm6b
Pde4a
Rabgap1l
Got2
Pdcd1lg2
Nol11
Cdca7l
Mms22l
Bves
Prr14l
1700009N14Rik
Arhgef26
Cfap20
Csgalnact1
Arid5b
Best3
Chaf1a
Kcng2
Fnbp1
Cd53
Lpin2
Efhd1
Tank

Fam174a
Spsb1
Epas1
Lrrc6
Fndc1
Trpm1
Pdia6
Clec3a
Veph1
Magi2
Tm6sf1
Adm
Itpr2
Hsd17b6
Srgap1
Gm527
Klhl25
Slc24a2
Slc2a1
Sncaip
Iqcg
Fam208a
Mast4
Usp24
Mme
Gata2
Gpr68
Cyth3
Sf3b3
Tnrc18
Cntn5
Tgfbr2
Lama2
Iqgap2
Vmn1r2
Snx11
B3gnt2
Kdm5b
Ptprd
Fsd1l
Pan3
Fgfbp3
Mkln1
1700029F12Rik
Cdyl2
4921511H03Rik
Sptssb

Aldoart2
Chst14
St6galnac3
Ctsb
Vcpkmt
Rag2
Sec23ip
Zfp536
