# Supplementary material for: MLL1 and MLL1 fusion proteins have distinct functions in regulating leukemic transcription program
Source: Cell Discov. 2016 May 17;2:16008–. doi: 10.1038/celldisc.2016.8 (PMC4869169; doi:10.1038/celldisc.2016.8)
Supplement: Supplementary Table S3 [file celldisc20168-s9.pdf]

Supplemental Table 3 Transcription Impact by MM-401, EPZ5676, MI-2 and iBET inhibitors on MLL1 and MLL1-AF9 joint targets. Selection Criteria is log2 (fold changes)>1 or <-1.

MENINi down-regulated joint targets.		MENINi up-regulated joint targets.		DOT1Li down-regulated	
gene	logfold	gene	logfold	gene	logfold
Eya1	-10.46946749	Nfatc1	1.005782	Nol4	-6.49039
Ugt8a	-9.713011705	Irf2	1.008583	Elavl2	-6.44619
Ttc29	-8.103310257	Tmc3	1.016327	Ttc29	-6.16649
Bcl11a	-7.681299656	1700003F	1.019533	Meis1	-5.66672
Lyplal1	-7.187657263	Arap1	1.020912	Clec3a	-5.07874
Elavl2	-7.148113073	4930523C	1.023291	Pcdh17	-4.62961
Eltd1	-6.41299719	Gm527	1.024879	Zbtb16	-4.61783
Isl1	-6.163345808	Wdfy3	1.039958	Gm13154	-4.43815
Meis1	-6.125483172	4931408C	1.040542	Eltd1	-4.34044
Clec3a	-5.18276713	Abcd2	1.044848	Hpgd	-4.09194
Gm13154	-5.14750847	Tmem65	1.049649	Gata2	-4.06928
Sgce	-4.685241959	Atxn1	1.053591	Bcl11a	-3.92006
Grb14	-4.662665171	Dtx3l	1.057816	Ano4	-3.84051
Pcdh17	-4.572637335	Myh10	1.058234	Isl1	-3.69403
Antxr1	-4.341266715	Hoxa6	1.064003	Bhlha15	-3.55599
Gata2	-4.121111866	Pde7b	1.066433	Fzd8	-3.29969
Igfbp7	-3.738179423	Zfp276	1.067324	Dsel	-3.22161
Amigo2	-3.661299621	Smad6	1.077566	Six4	-3.20863
Hpgd	-3.425829174	Kcnj15	1.079347	Car12	-3.16743
Car12	-3.406621009	Mgst2	1.093668	Tcerg1l	-3.16094
Dsel	-3.135948944	Tusc1	1.097795	Gpr125	-3.0991
Nrg1	-3.116977651	Mboat1	1.103553	Antxr1	-3.07727
Sdk1	-3.103343321	Snx29	1.115612	Fut8	-2.88103
Fzd8	-3.034603848	Fam222b	1.116175	C2cd4a	-2.8524
Epha7	-3.031455222	Sorl1	1.129402	Fgfbp3	-2.82017
Bcat1	-2.96029941	Tgm6	1.136668	Nrg1	-2.81807
Slc1a4	-2.943723685	Themis2	1.137191	1700016H	-2.73876
Fgfbp3	-2.928524286	St3gal1	1.156518	Irs1	-2.73088
Ano4	-2.890722269	Vcl	1.161637	Grb14	-2.65076
Ssbp2	-2.606769397	Ceacam18	1.165019	Mtss1l	-2.55932
Abcg2	-2.605154763	Gpr75	1.165276	S1pr3	-2.5006
Parp8	-2.516902407	Lhfpl2	1.168558	Igfbp7	-2.49011
Bhlha15	-2.501063466	Ggh	1.175298	Efhd1	-2.4591
Kcng2	-2.466677973	Ifitm1	1.175605	Trpm3	-2.32938
Prox1	-2.452043889	Dsp	1.185253	Il12a	-2.32689
Ptprr	-2.408601585	E030018B	1.190814	Cr2	-2.31537
Gpr125	-2.393163371	3830403N	1.191797	Parp8	-2.27062
Slc2a2	-2.338848124	Rap2b	1.197387	Slc1a4	-2.24456
Enpep	-2.327110899	Cgn	1.201733	Lysmd2	-2.23509
Col25a1	-2.276269428	Fgd5	1.209573	Slc2a2	-2.2248
Cyp27a1	-2.262297994	Slc23a2	1.215924	Elovl6	-2.22256
Mtss1l	-2.230338315	Cmpk2	1.232852	Epha7	-2.15734
Ppp1r9a	-2.22255528	Gpr68	1.238564	Cyp27a1	-2.14354
Gdf7	-2.212539403	Lrrc8d	1.251527	Enpep	-2.12197
Prkca	-2.169687376	Pparg	1.251914	Pard6g	-2.12011
Mettl24	-2.140977995	Lrrtm2	1.264236	Gpam	-2.11319
Lysmd2	-2.130891605	Map3k14	1.270141	Kcng2	-2.0627

Kazn	-2.124402298	C1galt1	1.27341	Kctd1	-2.03045
Sfrp2	-2.015447113	N4bp2l1	1.28226	Dio2	-2.00008
Kctd1	-2.013343786	Spry4	1.287738	Mettl24	-1.97397
Hist1h2bl	-2.011314467	Eva1a	1.292015	Grik4	-1.97344
Tsc22d1	-1.945965419	Rnf111	1.300376	Rab39b	-1.97292
Inpp4b	-1.884074846	Tbc1d2	1.310538	Sdk1	-1.9619
Myh14	-1.866650429	Cyth3	1.313857	Mgat5	-1.94948
Prickle2	-1.857725713	Mbp	1.313937	Cdk6	-1.91092
Nr4a2	-1.855676953	Snx10	1.315313	Pdcd4	-1.88521
Umps	-1.84229086	A530099J	1.327271	1700112E	-1.88517
Psat1	-1.840585426	Herc3	1.330935	Chl1	-1.87106
Plac8	-1.821917765	AI467606	1.336277	Tsc22d1	-1.84863
Elovl6	-1.808406016	Calcrl	1.339579	Samd4	-1.78894
Six4	-1.798485591	Ttll11	1.34688	Bcat1	-1.7709
Atp10a	-1.798177716	Reep3	1.347555	Trim45	-1.74309
Myb	-1.789942705	Mef2a	1.354107	Col25a1	-1.72097
Rwdd3	-1.769302161	Cacnb4	1.357417	Ppargc1a	-1.72032
9430020K	-1.761458019	Lims1	1.357619	Amigo2	-1.65708
Arhgap32	-1.759345501	Kdm6b	1.360346	Umps	-1.65421
Fam60a	-1.746063305	Cdk5r1	1.366522	Trib2	-1.65099
Impdh1	-1.737102692	Gcm2	1.374493	Rwdd3	-1.62926
1700112E	-1.730096339	Erg	1.393761	Adamts17	-1.62454
Trpm3	-1.714257413	Celf4	1.393942	Abcg2	-1.61765
Usp6nl	-1.706785461	Nanos1	1.394475	Bambi	-1.61735
Gpam	-1.704975989	Ube2h	1.4044	Lypd6b	-1.60849
Stc2	-1.690601227	Ubash3b	1.406012	Xrcc6bp1	-1.60822
Frat2	-1.676594483	Adcy9	1.420054	Tgif1	-1.59378
Ddx18	-1.675335308	Glrx	1.430459	Kcnh5	-1.5925
Foxp1	-1.634866238	Dach1	1.443603	Nat10	-1.58337
Cdk6	-1.634035942	Sema3b	1.45871	Myb	-1.57436
Tcf4	-1.632503579	Btg1	1.485721	Frat2	-1.57088
Flnb	-1.620591602	Gpr84	1.49498	Gcsh	-1.56338
Rcc1	-1.607792382	Arid3b	1.506646	Atp10a	-1.557
S1pr3	-1.6030447	Plet1	1.524426	St7	-1.55313
Eno1b	-1.59138902	Zfp804a	1.52946	Kazn	-1.5379
Adamts17	-1.58991956	Jak2	1.555702	Trub1	-1.53291
Trim45	-1.58574077	Fam196b	1.567047	Afap1	-1.51287
Fut8	-1.578375061	Ets1	1.568413	Prickle2	-1.50866
Nat10	-1.536039514	Abca13	1.585402	Gramd3	-1.49519
Phtf2	-1.532797602	Rdm1	1.587522	Dnajc6	-1.49141
St7	-1.52481764	Irs2	1.594018	9430020K	-1.47697
Msl3l2	-1.513919085	Peli3	1.608219	Lap3	-1.46153
Ttc36	-1.510271156	Thrb	1.615154	Wdr27	-1.45834
Sncaip	-1.506986727	Ski	1.627668	Eno1b	-1.44455
Immp2l	-1.490307514	Fry	1.634185	Rcc1	-1.41848
Trub1	-1.48431068	Dusp6	1.645764	Myh14	-1.41705
Mcm3	-1.47116514	Kif13b	1.678456	Prox1	-1.39651
Mgat5	-1.463981954	Tbxas1	1.680944	Ddx18	-1.39126
Crim1	-1.443681038	Rab11fip4	1.682413	Slc16a1	-1.37624

Efhd1	-1.437417406	2010300C	1.682993	Impdh1	-1.37504
Vcpkmt	-1.430071749	Ctdspl	1.684498	Dnajc25	-1.37364
Pard6g	-1.427608047	Zfp365	1.693936	Tcf4	-1.3697
Spns3	-1.415764429	Snx24	1.70093	Phtf2	-1.36581
Angptl4	-1.396416225	Klf6	1.717615	Bola3	-1.35998
Adamts5	-1.375389803	Ctsb	1.72363	Chd9	-1.35142
Pbx3	-1.368425565	Tulp4	1.725246	Plac8	-1.33945
Tgif1	-1.365807365	Chrm3	1.76687	Usp6nl	-1.33618
Slc7a1	-1.328932967	Matn1	1.775517	St8sia4	-1.33133
Perp	-1.315560289	Gcnt1	1.784464	Fam60a	-1.32137
Lap3	-1.311892304	Klf5	1.800294	Six1	-1.32131
Dio2	-1.290714546	Dazl	1.803892	Smco4	-1.30821
1810011O	-1.288259367	Csrnp1	1.827739	Gng12	-1.30289
Mtus2	-1.287157751	Slc8a1	1.836433	Diras2	-1.29006
Rpf2	-1.265692765	Nedd9	1.841202	Itpr1	-1.28592
C2cd4a	-1.263923518	Cyb561	1.848122	Crim1	-1.28191
Gcsh	-1.259604297	Hdac9	1.851376	D8Ertd82e	-1.27114
Dnajc6	-1.255527146	Rnf144b	1.86066	Lrpprc	-1.26453
Phb	-1.251046766	Nabp1	1.875028	B4galt6	-1.26246
B4galt6	-1.242930665	Adpgk	1.923035	Tox	-1.25829
1190002N	-1.235575871	Dock9	1.950411	Mgarp	-1.25765
Slc16a1	-1.219782202	Acvr2a	1.955574	Angptl4	-1.23862
Pdcd4	-1.216360368	Hmox1	1.966633	Mcm3	-1.22282
Dnajc25	-1.209377644	Adrb2	1.972001	Phb	-1.2197
Galnt11	-1.200757762	Cdh17	2.020277	Psat1	-1.21342
Minpp1	-1.196871498	Megf9	2.024485	Vps13c	-1.21014
Lmo2	-1.19293411	3110043O	2.040774	Inpp4b	-1.20229
Tdrd3	-1.175752085	Kirrel3	2.079347	Xxylt1	-1.20148
Epb4.1l5	-1.157615681	Hsd11b1	2.080116	Sgce	-1.20036
D8Ertd82e	-1.154013223	Tenm4	2.104887	Wars	-1.19675
Cyp26a1	-1.150797612	Tcf7l2	2.115028	Nufip1	-1.19489
Xrcc6bp1	-1.143751968	Tgfbr2	2.115292	Msl3l2	-1.18254
Bola3	-1.134905311	Fam65b	2.12031	Prkca	-1.17245
Mgarp	-1.128809819	Rassf3	2.124689	Runx2	-1.15306
Snrpa1	-1.128174829	Reck	2.127464	Pdia6	-1.13608
Acaca	-1.124968571	Sqrdl	2.135628	Arrdc3	-1.12809
Nufip1	-1.106588594	Ptges	2.137503	Osbpl1a	-1.12779
Pdia6	-1.094057192	Dlg2	2.209573	Rnf152	-1.12226
Gtpbp4	-1.093501498	Mast4	2.216103	Sfrp2	-1.12125
Serbp1	-1.092546047	Lpp	2.220999	Nr4a2	-1.10899
Sipa1l1	-1.090356751	Agpat9	2.22101	Stc2	-1.10452
Ankrd28	-1.089369416	Etv1	2.225854	Foxp1	-1.08975
Itpr1	-1.081892366	Tal2	2.251383	Papss1	-1.04644
Met	-1.060905159	Tmem106a	2.252065	Zfp609	-1.04182
Lrpprc	-1.045637316	Ptger2	2.25874	Flnb	-1.0339
Coq2	-1.045149717	Nos1ap	2.277356	Met	-1.03304
Xxylt1	-1.044404407	Cers3	2.286407	Irak1bp1	-1.03251
Chd9	-1.041978393	Luzp1	2.32117	Rrn3	-1.02464
Smco4	-1.034433574	Syt7	2.424477	Ctnna2	-1.02353

Runx2	-1.024183129	Atp8a2	2.43887	Gfra2	-1.02353
Pgk1	-1.018144905	Actn1	2.469204	Dync1i1	-1.02353
Samd4	-1.017690986	Atg16l2	2.4919	Tdrd3	-1.01921
Sgk3	-1.015343071	Dhrs3	2.530744	Coq2	-1.01292
Jmy	-1.015123809	Lyst	2.603127	Acaca	-1.00758
Cse1l	-1.012149688	Epas1	2.61665	Gtpbp4	-1.00609
Coa7	-1.011756687	Tmcc1	2.672724	Rpf2	-1.00182
Slc25a13	-1.00023967	Arhgap22	2.704005	1810011O	-1.00117
		Clvs1	2.717034	Snrpa1	-1.00081
		Syne1	2.765264		
		Fos	2.84308		
		Irs1	2.91551		
		Marcks	2.95732		
		Prss52	2.957386		
		Rap1gap2	3.035576		
		Basp1	3.056378		
		Klf4	3.081088		
		Fam46c	3.125386		
		Sgms1	3.189817		
		Maml2	3.256511		
		Hgf	3.259509		
		Arhgap24	3.276018		
		Abca1	3.356748		
		St18	3.396875		
		Mctp2	3.454766		
		Abcg3	3.462095		
		Hao1	3.52095		
		Alcam	3.731137		
		Lrrc6	3.800651		
		Dfna5	3.821065		
		Flrt3	3.854332		
		Gcnt2	3.877138		
		1-Mar	3.891465		
		Sstr4	3.909664		
		4933430I1	3.913887		
		Fzd1	3.922848		
		Olfm4	3.925131		
		Plk2	3.941655		
		Pdcd1lg2	3.973437		
		Mapre3	3.991096		
		Tmem132l	4.045656		
		Mapk13	4.04635		
		Pid1	4.089845		
		Ppap2b	4.887271		
		Cpeb1	4.945067		
		Inhba	5.047926		
		Trib1	5.060409		
		Msr1	5.447355		
		Mafb	5.458963		

Egr3	5.711404
Kcnn3	6.058659
Maf	6.116051
Erc2	6.854318
Pbx1	6.945694
Ch25h	6.971965
Slc9a4	7.060675
Emp1	7.286263

joint targets	DOT1Li up-regulated joint targets.		MLL1i down-regulated joint targets.		MLL1i up-regulated joint	
	gene	logfold	gene	logfold	gene	logfold
	Mab21l3	1.001463	Bcl11a	-9.92076	Trib1	1.085694
	A530099J:	1.003664	Eya1	-9.27677	Ankrd7	1.105795
	Calcrl	1.009999	Ttc29	-7.92246	Syt7	1.105795
	Kdm6b	1.013643	Nol4	-6.10143	Tgm6	1.105795
	Ggh	1.025585	Pcdh17	-5.96062	Kcnma1	1.111704
	Wdfy3	1.02571	Elavl2	-5.8786	Sncaip	1.115548
	Dazl	1.02603	Dsel	-4.86868	Cpeb1	1.126249
	Klhl32	1.02603	Prox1	-4.85062	Gdf7	1.1866
	Ceacam18	1.027099	Igfbp7	-4.66986	Cdh17	1.208755
	Etv1	1.02942	Eltd1	-4.34925	C2cd4a	1.227292
	Ifitm1	1.029689	Amigo2	-4.0736	Hsd11b1	1.228612
	Ly86	1.040637	Adamts17	-4.03188	Gpr84	1.238861
	Ttll7	1.047397	Grb14	-4.00531	Alcam	1.240984
	Zfp276	1.053399	Clec3a	-3.81445	Fam65b	1.30009
	Ncs1	1.059479	Sox6	-3.55534	Pid1	1.317419
	Nt5c2	1.059889	Prkca	-3.12783	Ano4	1.348374
	1700003F:	1.06924	Sgce	-2.93098	Luzp1	1.350266
	Hs3st3b1	1.073001	Abcg2	-2.89739	Ly86	1.378488
	E030018B	1.075685	Arrdc3	-2.79839	Pcdh7	1.38529
	Slc16a7	1.077184	Fzd8	-2.79675	Dazl	1.394382
	Acvr2a	1.081391	Tsc22d1	-2.5137	Klhl32	1.394382
	Snx24	1.090025	Ptprg	-2.50462	Abca1	1.410132
	Rab13	1.093554	Arhgap32	-2.41301	Flrt3	1.427723
	Ski	1.107351	Mettl24	-2.1827	Lysmd2	1.485056
	Antxr2	1.113089	Stxbp6	-2.12638	Abcg3	1.510593
	Dach1	1.116461	Hlf	-2.10059	Cers3	1.540003
	Phactr2	1.141437	Antxr1	-2.0554	Nbea	1.586463
	Herc3	1.159739	Chl1	-2.04299	Ctnna2	1.617903
	Dusp6	1.164217	Cyp27a1	-2.0396	Plk2	1.62719
	Ctsb	1.168151	1700112E	-2.02534	Fzd1	1.668676
	Cdk5r1	1.17601	Marcks	-1.98844	Atp8a2	1.950978
	Ubash3b	1.177199	Ttc36	-1.91779	Msr1	2.117295
	Slc8a1	1.190287	Rag2	-1.9018	Maf	2.358625
	Hebp2	1.191503	Mgarp	-1.89736	4933430I1	2.393042
	Nbea	1.191948	Enpep	-1.89461	Lrrc6	2.426538
	Reep3	1.252835	Col25a1	-1.89297	Ch25h	2.767978
	C1galt1	1.25449	Med12l	-1.86498	Ppargc1a	3.106327
	Mbp	1.267614	Ptprr	-1.79332	Slc9a4	3.170549
	Sox6	1.283799	Tcp11	-1.7931	Emp1	3.244175
	Snx10	1.301872	Dsp	-1.78835	Dlg2	3.39537
	Galnt3	1.301978	Rdm1	-1.7731	Hao1	3.608295
	Tiam2	1.308983	Iqgap2	-1.76584	Mafb	3.809913
	Gas7	1.322633	1700056E	-1.70569		
	Gcnt1	1.330345	1810011O	-1.70193		
	4931408C	1.341794	Sfrp2	-1.64804		
	Chrm3	1.363436	Fam46c	-1.61983		
	Kif13b	1.376333	Efnb2	-1.61403		

Pde7b	1.377264	Gpr125	-1.56542
Hdac9	1.388908	Ctnnbip1	-1.46676
Rap2b	1.38972	Olfm1	-1.44008
N4bp2l1	1.393265	Myh14	-1.43534
Luzp1	1.396773	Mtus2	-1.41717
Smad6	1.407193	Spsb1	-1.40983
Jak2	1.409306	Kazn	-1.39868
Myh10	1.41008	Adm	-1.38275
AI467606	1.413373	Eno1b	-1.38206
Adrb2	1.426767	Glra1	-1.34712
Kirrel3	1.438013	4930562C	-1.29569
Iqgap2	1.44843	Cyp26a1	-1.293
Ube2h	1.451155	Ppp1r9a	-1.2386
Gm527	1.472024	9430020K	-1.22367
Gnaz	1.490652	Wdr19	-1.22065
Rdm1	1.493549	Srgap1	-1.20504
Celf4	1.496779	Rwdd3	-1.18472
Lrrc8d	1.497593	Rbm47	-1.17548
Tulp4	1.500049	Dync2li1	-1.1716
Arid3b	1.503054	Epm2a	-1.14322
Fry	1.51155	Wdr27	-1.11857
Klf6	1.515768	Galnt11	-1.11263
Nedd9	1.516965	Mpzl1	-1.11016
Gpr68	1.529198	Galnt18	-1.10374
Bcl2l11	1.530546	Dclk2	-1.10319
Pparg	1.531655	Pard6g	-1.09968
3110043O	1.557833	Smad6	-1.08627
Adcy9	1.559878	Gcm2	-1.07008
Glrx	1.560444	Gcnt1	-1.05716
Tnfsf8	1.561433	Celf4	-1.04753
Cdh17	1.573154	Cgn	-1.04299
Abca13	1.575253	Zfp365	-1.04299
Myo3b	1.578143	1700025G	-1.03854
Hmox1	1.579971	Basp1	-1.03704
Ttll11	1.581526	Pde7b	-1.03537
Lims1	1.594342	Atp10a	-1.02399
Clvs1	1.594661	Fggy	-1.01891
Gpr75	1.611157	Prkar2b	-1.00217
Trim67	1.613209		
Tmc3	1.624302		
Tbc1d2	1.627561		
Med12l	1.647511		
Rab11fip4	1.648991		
Ankrd7	1.651319		
Ptges	1.665556		
Btg1	1.669271		
Tusc1	1.672324		
Cyb561	1.673206		
Tmem106a	1.687265		

Vcl	1.71237
Gpr84	1.739561
Fam196b	1.745308
Mast4	1.759815
Lhfpl2	1.767717
Cmpk2	1.769343
Ets1	1.776187
Csrnp1	1.776483
Tgfbr2	1.823373
Maml2	1.823462
Dock9	1.826996
Nanos1	1.833178
Nabp1	1.85078
Lmod3	1.901123
Tgm6	1.909406
Rassf3	1.91283
Nos1ap	1.919918
Megf9	1.953152
Marcks	2.004192
Fscn1	2.046465
Lpp	2.052574
Phactr1	2.056445
2010300C	2.112292
Ptger2	2.114748
Irs2	2.128325
Fam65b	2.153727
Tcf7l2	2.183762
Tenm4	2.193606
Klf5	2.201796
Atp8a2	2.210255
Dhrs3	2.240293
Epas1	2.262124
Abca1	2.268831
Sqrdl	2.271471
Rnf144b	2.275962
Actn1	2.278102
Arhgap24	2.287169
Hsd11b1	2.299253
Prss52	2.299451
Arhgap22	2.300283
Olfm1	2.312819
Cers3	2.332094
Erg	2.366312
Lrrtm2	2.411618
Atg16l2	2.427018
Cyp26a1	2.434173
Lyst	2.442742
Fzd1	2.475131
Basp1	2.489947

Rap1gap2	2.53441
Agpat9	2.553453
Tmcc1	2.557776
Glra1	2.666569
4933430I1	2.669515
Pdcd1lg2	2.687108
Klf4	2.704438
St18	2.758602
Reck	2.7651
Ctdspl	2.766534
Adpgk	2.769543
Tmem132l	2.777102
Syt7	2.917642
Cacnb2	2.962368
Fos	3.087213
Sstr4	3.097164
Sgms1	3.129292
Syne1	3.144919
Tal2	3.168632
Plk2	3.175534
Abcg3	3.194751
Mctp2	3.206169
Gcm2	3.265725
Dsp	3.279278
3830403N	3.299451
Hao1	3.316161
Hgf	3.318895
Gcnt2	3.492433
Alcam	3.584147
1-Mar	3.591914
Dfna5	3.659925
Trib1	3.825115
Fam46c	3.826628
Perp	3.997595
Ppap2b	4.041644
Cpeb1	4.060874
Mapre3	4.124883
Mafb	4.163105
Pid1	4.255439
Mapk13	4.301482
Flrt3	4.381258
Egr3	4.4541
Inhba	4.914055
Olfm4	4.955812
Dlg2	5.154774
Maf	5.382676
Erc2	5.570686
Lrrc6	5.672902
Msr1	6.015625

Pbx1	6.239948
Kcnn3	6.430341
Emp1	7.145812
Ch25h	7.264839
Slc9a4	7.324661

t targets. BRD4i down-regulated joint targets. BRD4i up-regulated MLL1 and MLL-AF9 joint targets.

gene	logfold	gene	logfold
Clec3a	-8.42737	N6amt1	1.000883
Ttc29	-7.84382	Irs1	1.018419
Elavl2	-7.37027	Maml3	1.027252
Eltd1	-6.38428	Spag17	1.02775
Hlf	-6.19637	Mbnl1	1.041482
Col25a1	-6.12461	Rnf220	1.056255
Erg	-5.72227	Flnb	1.093565
Fzd8	-5.64974	Snx24	1.11083
Sgce	-5.40183	Tgm6	1.134819
Ptprr	-5.25766	Bcl2l11	1.135765
Grb14	-4.69505	Met	1.143623
Abcg2	-4.40035	Reep3	1.171332
Spns3	-4.39164	Gnaz	1.172998
Dsel	-4.25496	Zfp365	1.172998
Cyp26a1	-4.19946	Kirrel3	1.186633
Pde7b	-4.14726	Wdfy3	1.187183
Olfm4	-4.10564	Vps13c	1.188371
Amigo2	-4.06933	Hoxa10	1.198362
Basp1	-4.02943	Plk2	1.238583
Tmem236	-3.8313	Fam65b	1.25097
Enpep	-3.82197	Ctsb	1.251775
Sfrp2	-3.80008	Map3k14	1.276882
Pcdh17	-3.67729	Tulp4	1.281274
Prox1	-3.6531	Ifitm1	1.283091
Sox6	-3.5899	N4bp2l1	1.296902
Tenm4	-3.57798	Chn2	1.346436
Lhfpl2	-3.57138	Il6st	1.349046
9430020K	-3.55495	Ski	1.378429
Mgarp	-3.52712	Nanos1	1.387769
Ceacam18	-3.51543	Arhgap24	1.388065
Ppp1r9a	-3.46049	Hmox1	1.389128
Dsp	-3.42055	St8sia4	1.431131
Kcnh5	-3.33796	Nsun4	1.442215
Trpm3	-3.26873	Ctnnbip1	1.45449
Car12	-3.23484	Gm527	1.460831
Kcnn3	-3.22053	Zfp619	1.463342
Kazn	-3.20007	3110043O	1.485105
Cyp26b1	-3.19586	Spsb1	1.544329
Adamts17	-3.14197	Fsd1l	1.554276
Ttll7	-3.12959	Cgn	1.577831
Myh14	-3.12622	Efnb2	1.582591
Dnajc6	-3.11867	Herc3	1.621244
Bhlha15	-3.046	Dusp6	1.623772
Bcat1	-2.98916	Hpgd	1.656351
Pbx1	-2.91759	Atrnl1	1.68989
Chrm3	-2.91344	Abhd6	1.73359
Inhba	-2.87363	Dennd2c	1.737681

Cdkn1c	-2.84984
Plekhh2	-2.82612
Fgfbp3	-2.82032
Camk4	-2.69782
Ptges	-2.66823
Ano4	-2.64804
Marcks	-2.64506
Gpr84	-2.62463
Erc2	-2.58372
F13a1	-2.55702
Gcm2	-2.51165
Pdcd1lg2	-2.48304
Cyp27a1	-2.46992
1810011O	-2.43398
Dio2	-2.40642
Rab13	-2.40027
Cyb561	-2.36988
Rgs18	-2.36309
Bcl2l14	-2.34163
Mettl24	-2.34064
Tacc2	-2.33403
1700112E	-2.31853
Stxbp6	-2.2705
Arrdc3	-2.23911
Igfbp7	-2.23147
Celf4	-2.22656
Sntb1	-2.21514
Perp	-2.20019
B3galt2	-2.19677
Eno1b	-2.19067
Pparg	-2.1315
Smad6	-2.13096
Iqgap2	-2.06651
Cr2	-2.06484
Olfm1	-2.03531
Med12l	-1.99616
Syne1	-1.99304
Zfp804a	-1.97945
Gcnt2	-1.96024
Plxnd1	-1.93506
Hsd11b1	-1.92271
Iqcg	-1.85523
Tiam1	-1.85027
Irak1bp1	-1.84037
Trib2	-1.81219
Slc16a3	-1.81081
Mtus2	-1.80569
A530099J	-1.78335
Stc2	-1.77247

Luzp1	1.781162
Fam105a	1.803429
Dazl	1.831111
Magi1	1.838679
Epas1	1.846091
Maf	1.896835
Ppap2b	1.927805
Pcdh7	1.954077
Tox	2.479792
Crim1	2.923024
C2cd4a	3.0906
Flrt3	3.114435
Cxcl12	3.408689
Ppargc1a	3.474915
Egr3	3.753447
Mafb	4.323838

D8Ertd82e	-1.76087
Flrt1	-1.71456
Phactr2	-1.70771
Cacna1b	-1.66861
Fam46c	-1.65739
Pmp22	-1.65575
Hgf	-1.6549
4930562C	-1.64891
Fscn1	-1.6219
Elovl6	-1.61735
Dync2li1	-1.61339
Rwdd3	-1.57089
B4galt6	-1.56111
Esr1	-1.56078
Hdac9	-1.52912
Tcp11	-1.52197
Efhd1	-1.50937
Rasd1	-1.49842
Hebp2	-1.49549
Pgk1	-1.41676
Impdh1	-1.40006
Calcrl	-1.39856
Kit	-1.39024
Mctp2	-1.3872
Angptl4	-1.37921
Camkmt	-1.37438
Matn1	-1.34064
Ctnna2	-1.34064
1700056E	-1.3363
Thrb	-1.33578
Ttc36	-1.32894
Rbm47	-1.31257
Gpr68	-1.30481
Pid1	-1.2964
Smco4	-1.28518
Plxna2	-1.27045
Rab39b	-1.24212
Dach1	-1.22495
Dfna5	-1.19954
Gm5803	-1.19895
Ets1	-1.19889
Dnah11	-1.19861
Extl3	-1.19199
Nav2	-1.19013
Ankrd28	-1.18462
Tbxas1	-1.18393
Tbc1d2	-1.15281
Gdf7	-1.14731
Rab11fip4	-1.11078

Csrnp1	-1.10031
Ldlrad4	-1.06392
1700016H	-1.05951
Asap1	-1.05765
Rab11fip2	-1.05076
Dact1	-1.04674
Immp2l	-1.03725
Susd1	-1.03469
Ero1l	-1.01516
Sema3b	-1.00232
