# Supplementary material for: MLL1 and MLL1 fusion proteins have distinct functions in regulating leukemic transcription program
Source: Cell Discov. 2016 May 17;2:16008–. doi: 10.1038/celldisc.2016.8 (PMC4869169; doi:10.1038/celldisc.2016.8)
Supplement: Supplementary Method [file celldisc20168-s10.pdf]

Supplementary Method

RNA-seq data analyses

TopHat2 (Trapnell et al., 2009) was used to map reads to mouse reference genome assembly mm9. Mapped reads from two conditions were then used as input in cuffdiff software (Trapnell et al., 2012) to calculate FPKM for each gene under each condition and call differentially expressed genes. Genes are considered significantly differentially expressed if both the following criteria are met: 1) *p* value given by cuffdiff is less than 0.05; and 2) log2 (fold changes) greater than 1 or less than -1. Scatter plot of correlation studies for MM-401, EPZ-5676, MI-02 and iBET inhibitor data set was obtained by R software (http://www.r-project.org/) using the following procedures: transcripts with enough aligned reads and corresponding to the same gene are combined. Transcripts without enough aligned reads or show opposite trends for the same gene are discarded. Genes with valid expression information were used for the analyses. Gene set enrichment analysis (GSEA) was done using downloaded GSEA software. Differential expressed genes were used as ranked gene list and GSEA was run against public database. FDR was calculated based on 1,000 permutations. R package GO stats were used for GO term association (Falcon and Gentleman, 2007). For each gene list, GO term enrichment was tested with conditional single-sided hypergeometric distribution and *p* value was calculated.

ChIP-seq data analyses

ChIP-Seq reads were aligned to mouse reference genome mm9 using bowtie allowing no more than one mismatch. Unique reads mapped to a single genomic location were kept for peak identification using MACS (Zhang et al., 2008). We used previously published MLL-AF9 ChIP-seq data (GEO/ GSM721212) for analyses (Bernt et al., 2011). Since no input was provided for the MLL-AF9 ChIP-seq data, we used MACS with significance cutoff of p <=1E-04 or p <=1E05 for peak identification. No false identification was detected using either criterion. WIG files generated by MACS were used for visualization and manual confirmation in UCSC genome

browser. Similar results were obtained by using the previously described computational background model with significance cutoff of $p<=1E5$ (Bernt et al., 2011).

To compare ChIP-seq signals at specific regions of the genome, unique mapped reads for WDR5 were annotated to putative features across the mm9 reference genome using HOMER. WDR5-enriched peaks were grouped based on whether they were located inside or outside a +/-5000 bp window around RefSeq TSSs. Tags were counted for reads aligned to WDR5 peaks using a +/-5000 bp window and a 20 bp bin. Tag counts were log2-transformed and ranked and heatmaps were plotted using R software (http://www.r-project.org/).

To compare MLL1 and MLL-AF9 peaks, the distance between MLL1 and MLL-AF9 peak centers was used. If it was less than 2kb, it is defined as an overlapping peak. Peak annotations and motif analyses were performed using HOMER (mm9, with default setting) (Heinz et al., 2010).

To identify H3K4me2 changes after MM401 treatment, we divided H3K4me2 peaks into two categories: 1) H3K4me2 peaks in mock treated samples were not identified in samples treated with MM401; 2) H3K4me2 peaks in mock treated samples were identified in samples treated with MM-401, but had decreased intensity. Overlap of H3K4me2 between Mock and MM-401 treated cells was determined by BEDTools (merge) (Quinlan and Hall, 2010) that requires >= 1bp overlap. To quantify H3K4me2 changes, overlapped H3K4me2 peaks between mock and MM-401 treated samples were merged and read counts for each peak were determined using Subread (Liao et al., 2014). Read counts were further normalized to 1M for all samples using total number of uniquely mapped reads. Change in peak intensity was defined by DMSO_peak_intensity/MM401_peak_intensity>=2 using ChIP/Input ratio after reads normalization.

SUPPLEMENTAL REFERENCE:

Bernt, K.M., Zhu, N., Sinha, A.U., Vempati, S., Faber, J., Krivtsov, A.V., Feng, Z., Punt, N., Daigle, A., Bullinger, L., *et al.* (2011). MLL-rearranged leukemia is dependent on aberrant H3K79 methylation by DOT1L. Cancer Cell *20*, 66-78.

Falcon, S., and Gentleman, R. (2007). Using GOstats to test gene lists for GO term association. Bioinformatics *23*, 257-258.

Heinz, S., Benner, C., Spann, N., Bertolino, E., Lin, Y.C., Laslo, P., Cheng, J.X., Murre, C., Singh, H., and Glass, C.K. (2010). Simple combinations of lineage-determining transcription factors prime cis-regulatory elements required for macrophage and B cell identities. Mol Cell *38*, 576-589.

Liao, Y., Smyth, G.K., and Shi, W. (2014). featureCounts: an efficient general purpose program for assigning sequence reads to genomic features. Bioinformatics *30*, 923-930.

Quinlan, A.R., and Hall, I.M. (2010). BEDTools: a flexible suite of utilities for comparing genomic features. Bioinformatics *26*, 841-842.

Trapnell, C., Pachter, L., and Salzberg, S.L. (2009). TopHat: discovering splice junctions with RNA-Seq. Bioinformatics *25*, 1105-1111.

Trapnell, C., Roberts, A., Goff, L., Pertea, G., Kim, D., Kelley, D.R., Pimentel, H., Salzberg, S.L., Rinn, J.L., and Pachter, L. (2012). Differential gene and transcript expression analysis of RNA-seq experiments with TopHat and Cufflinks. Nature protocols *7*, 562-578.

Wilkinson, A.C., Ballabio, E., Geng, H., North, P., Tapia, M., Kerry, J., Biswas, D., Roeder, R.G., Allis, C.D., Melnick, A., *et al.* (2013). RUNX1 is a key target in t(4;11) leukemias that contributes to gene activation through an AF4-MLL complex interaction. Cell Rep *3*, 116-127.

Zhang, Y., Liu, T., Meyer, C.A., Eeckhoute, J., Johnson, D.S., Bernstein, B.E., Nusbaum, C., Myers, R.M., Brown, M., Li, W., *et al.* (2008). Model-based analysis of ChIP-Seq (MACS). Genome biology *9*, R137.
